# Characteristics that modify the effect of small-quantity lipid-based nutrient supplementation on child growth: an individual participant data meta-analysis of randomized controlled trials

**DOI:** 10.1093/ajcn/nqab278

**Published:** 2021-09-29

**Authors:** Kathryn G Dewey, K Ryan Wessells, Charles D Arnold, Elizabeth L Prado, Souheila Abbeddou, Seth Adu-Afarwuah, Hasmot Ali, Benjamin F Arnold, Per Ashorn, Ulla Ashorn, Sania Ashraf, Elodie Becquey, Jaden Bendabenda, Kenneth H Brown, Parul Christian, John M Colford, Sherlie J L Dulience, Lia C H Fernald, Emanuela Galasso, Lotta Hallamaa, Sonja Y Hess, Jean H Humphrey, Lieven Huybregts, Lora L Iannotti, Kaniz Jannat, Anna Lartey, Agnes Le Port, Jef L Leroy, Stephen P Luby, Kenneth Maleta, Susana L Matias, Mduduzi N N Mbuya, Malay K Mridha, Minyanga Nkhoma, Clair Null, Rina R Paul, Harriet Okronipa, Jean-Bosco Ouédraogo, Amy J Pickering, Andrew J Prendergast, Marie Ruel, Saijuddin Shaikh, Ann M Weber, Patricia Wolff, Amanda Zongrone, Christine P Stewart

**Affiliations:** Institute for Global Nutrition and Department of Nutrition, University of California, Davis, Davis, CA, USA; Institute for Global Nutrition and Department of Nutrition, University of California, Davis, Davis, CA, USA; Institute for Global Nutrition and Department of Nutrition, University of California, Davis, Davis, CA, USA; Institute for Global Nutrition and Department of Nutrition, University of California, Davis, Davis, CA, USA; Public Health Nutrition, Department of Public Health and Primary Care, University of Ghent, Ghent, Belgium; Department of Nutrition and Food Science, University of Ghana, Legon, Accra, Ghana; The JiVitA Project of Johns Hopkins University, Bangladesh, Paschimpara, Bangladesh; Francis I Proctor Foundation, University of California, San Francisco, San Francisco, CA, USA; Center for Child Health Research, Faculty of Medicine and Health Technology, Tampere University, Tampere, Finland; Department of Paediatrics, Tampere University Hospital, Tampere, Finland; Center for Child Health Research, Faculty of Medicine and Health Technology, Tampere University, Tampere, Finland; Center for Social Norms and Behavioral Dynamics, University of Pennsylvania, Philadelphia, PA, USA; Poverty, Health, and Nutrition Division, International Food Policy Research Institute, Washington, DC, USA; Department of Nutrition and Food Safety, WHO, Geneva, Switzerland; Institute for Global Nutrition and Department of Nutrition, University of California, Davis, Davis, CA, USA; Helen Keller International, New York, NY, USA; Program in Human Nutrition, Department of International Health, Johns Hopkins Bloomberg School of Public Health, Baltimore, MD, USA; School of Public Health, University of California, Berkeley, Berkeley, CA, USA; Brown School, Washington University in St. Louis, St Louis, MO, USA; School of Public Health, University of California, Berkeley, Berkeley, CA, USA; Development Research Group, World Bank, Washington, DC, USA; Center for Child Health Research, Faculty of Medicine and Health Technology, Tampere University, Tampere, Finland; Institute for Global Nutrition and Department of Nutrition, University of California, Davis, Davis, CA, USA; Program in Human Nutrition, Department of International Health, Johns Hopkins Bloomberg School of Public Health, Baltimore, MD, USA; Zvitambo Institute for Maternal and Child Health Research, Harare, Zimbabwe; Poverty, Health, and Nutrition Division, International Food Policy Research Institute, Washington, DC, USA; Brown School, Washington University in St. Louis, St Louis, MO, USA; School of Health Sciences, Western Sydney University, Penrith, New South Wales, Australia; Department of Nutrition and Food Science, University of Ghana, Legon, Accra, Ghana; Independent consultant, Dakar, Senegal; Poverty, Health, and Nutrition Division, International Food Policy Research Institute, Washington, DC, USA; Division of Infectious Diseases and Geographic Medicine, Stanford University, Stanford, CA, USA; Department of Public Health, School of Public Health and Family Medicine, College of Medicine, University of Malawi, Blantyre, Malawi; Department of Nutritional Sciences and Toxicology, University of California, Berkeley, Berkeley, CA, USA; Zvitambo Institute for Maternal and Child Health Research, Harare, Zimbabwe; Global Alliance for Improved Nutrition, Washington, DC, USA; Center for Non-communicable Diseases and Nutrition, BRAC James P Grant School of Public Health, Dhaka, Bangladesh; Department of Public Health, School of Public Health and Family Medicine, College of Medicine, University of Malawi, Blantyre, Malawi; Mathematica, Washington, DC, USA; Center for Non-communicable Diseases and Nutrition, BRAC James P Grant School of Public Health, Dhaka, Bangladesh; Department of Population Medicine and Diagnostic Sciences, Cornell University, Ithaca, NY, USA; Health Sciences Research Institute (IRSS), Bobo-Dioulasso, Burkina Faso; School of Engineering, Tufts University, Medford, MA, USA; Zvitambo Institute for Maternal and Child Health Research, Harare, Zimbabwe; Blizard Institute, Queen Mary University of London, London, United Kingdom; Poverty, Health, and Nutrition Division, International Food Policy Research Institute, Washington, DC, USA; The JiVitA Project of Johns Hopkins University, Bangladesh, Paschimpara, Bangladesh; Division of Epidemiology, School of Community Health Sciences, University of Nevada, Reno, Reno, NV, USA; Meds & Foods for Kids, St. Louis, MO, USA; Independent consultant, Washington, DC, USA; Institute for Global Nutrition and Department of Nutrition, University of California, Davis, Davis, CA, USA

**Keywords:** stunting, wasting, child undernutrition, complementary feeding, nutrient supplements, home fortification

## Abstract

**Background:**

Meta-analyses show that small-quantity lipid-based nutrient supplements (SQ-LNSs) reduce child stunting and wasting. Identification of subgroups who benefit most from SQ-LNSs may facilitate program design.

**Objectives:**

We aimed to identify study-level and individual-level modifiers of the effect of SQ-LNSs on child growth outcomes.

**Methods:**

We conducted a 2-stage meta-analysis of individual participant data from 14 randomized controlled trials of SQ-LNSs provided to children 6–24 mo of age (*n *= 37,066). We generated study-specific and subgroup estimates of SQ-LNS compared with control and pooled the estimates using fixed-effects models. We used random-effects meta-regression to examine study-level effect modifiers. In sensitivity analyses, we examined whether results differed depending on study arm inclusion criteria and types of comparisons.

**Results:**

SQ-LNS provision decreased stunting (length-for-age *z* score < −2) by 12% (relative reduction), wasting [weight-for-length (WLZ) *z* score < −2] by 14%, low midupper arm circumference (MUAC) (<125 mm or MUAC-for-age *z* score < −2) by 18%, acute malnutrition (WLZ < −2 or MUAC < 125 mm) by 14%, underweight (weight-for-age *z* score < −2) by 13%, and small head size (head circumference-for-age *z* score < −2) by 9%. Effects of SQ-LNSs generally did not differ by study-level characteristics including region, stunting burden, malaria prevalence, sanitation, water quality, duration of supplementation, frequency of contact, or average compliance with SQ-LNS. Effects of SQ-LNSs on stunting, wasting, low MUAC, and small head size were greater among girls than among boys; effects on stunting, underweight, and low MUAC were greater among later-born (than among firstborn) children; and effects on wasting and acute malnutrition were greater among children in households with improved (as opposed to unimproved) sanitation.

**Conclusions:**

The positive impact of SQ-LNSs on growth is apparent across a variety of study-level contexts. Policy-makers and program planners should consider including SQ-LNSs in packages of interventions to prevent both stunting and wasting.

This trial was registered at www.crd.york.ac.uk/PROSPERO as CRD42019146592.

## Introduction

Undernutrition, including stunting, wasting, and micronutrient deficiencies, is prevalent among infants and young children in low- and middle-income countries and is associated with increased morbidity and mortality and delayed psychomotor and neurocognitive development (1). Among children <5 y of age globally, 21.3% (144 million) were stunted and 6.9% (47 million) were wasted in 2019 (2). The etiology of stunting and wasting is complex and multifactorial (3–6), which may explain the limited effectiveness of interventions that focus solely on improving nutrition in improving these outcomes (5, 7). Nonetheless, low-quality diets that lack adequate amounts of key nutrients during the complementary feeding period from 6 to 24 mo of age are recognized as a critical contributory factor (3). Increased dietary diversity, with foods from all of the key food groups, and selection of nutrient-rich complementary foods within each of those food groups, should be universally promoted (8, 9). However, even under the best of circumstances it is difficult to meet all nutrient needs during this age interval (10), and for low-income populations the cost of certain nutrient-rich foods is often prohibitive (11, 12). For this reason, various types of fortified products designed to fill nutrient gaps have been evaluated, including fortified blended foods and products used for home fortification such as multiple micronutrient powders (MNPs) and small-quantity (SQ) lipid-based nutrient supplements (LNSs) (13).

SQ-LNSs were developed to provide multiple micronutrients embedded in a small amount of food that also provides energy, protein, and essential fatty acids (14). This combination of macro- and micronutrients addresses multiple potential nutritional deficiencies, including gaps in the key nutrients required for growth. Because SQ-LNSs typically provide only ∼100–120 kcal/d (∼4 teaspoons) and can be mixed with other foods, they are considered a type of home fortification product (15), although they can also be consumed alone. Unlike medium-quantity (MQ) and large-quantity LNSs, which are generally aimed at treatment of moderate and severe acute malnutrition (14), SQ-LNSs were designed for the prevention of undernutrition, including stunting.

In a recent meta-analysis of LNSs given during the period of complementary feeding (16), most of the included trials (13 out of 17) provided SQ-LNSs in ≥1 arm; the other 4 trials provided MQ-LNSs only. LNSs significantly reduced the prevalence of moderate stunting (by 7%, relative reduction), severe stunting (by 15%), moderate wasting (by 17%), and moderate underweight (by 15%) compared with no intervention. Exploratory subgroup analysis suggested that MQ-LNSs did not have a greater impact than SQ-LNSs on these outcomes. The meta-analysis also suggested that LNS was more effective than fortified blended foods or MNPs at improving child anthropometric outcomes. Although the meta-analysis included some analyses disaggregated by study characteristics (such as SQ- compared with MQ-LNS, duration of supplementation, and age at follow-up), analyses stratified by individual-level characteristics were not conducted.

Differences in study design and context and the characteristics of individuals may modify the effect of SQ-LNSs on child growth and other outcomes. Identification of subgroups of infants and young children who experience greater benefits from SQ-LNSs, or are more likely to respond to the intervention, may be useful in informing the development of public health programs and policies (7). To examine effect modification, we conducted an individual participant data (IPD) meta-analysis of randomized controlled trials (RCTs) of SQ-LNSs provided to infants and young children 6–24 mo of age. For this article, our objectives were to generate pooled estimates of the effect of SQ-LNSs on each growth outcome and identify study-level and individual-level modifiers of the effect of SQ-LNSs on these outcomes. Two companion articles report results for other outcome domains: anemia and micronutrient status (17) and development (18).

## Methods

The protocol for this IPD meta-analysis was registered as PROSPERO CRD42019146592 (https://www.crd.york.ac.uk/prospero) (19). The detailed protocol was posted to Open Science Framework (https://osf.io/ymsfu) before analysis and updated after consultations with co-investigators before finalizing the analysis plan (20), and the results are reported according to Preferred Reporting Items for Systematic Reviews and Meta-Analyses (PRISMA)-IPD guidelines (21). The analyses were approved by the institutional review board of the University of California Davis (1463609-1). All individual trial protocols were approved by the relevant institutional ethics committees.

### Inclusion and exclusion criteria for this IPD meta-analysis

We included RCTs of SQ-LNSs provided to children age 6–24 mo that met the following study-level inclusion criteria: *1*) the trial was conducted in a low- or middle-income country (22); *2*) SQ-LNS (<∼125 kcal/d) was provided to the intervention group for ≥3 mo between 6 and 24 mo of age; *3*) ≥1 trial group did not receive SQ-LNS or another type of child supplementation; *4*) the trial reported ≥1 outcome of interest; and *5*) the trial used an individual or cluster randomized design in which the same participants were measured at baseline (before child supplementation) and again after completion of the intervention (longitudinal follow-up), or different participants were measured at baseline and postintervention (repeated cross-sectional data collection). Trials were excluded if *1*) only children with severe or moderate malnutrition were eligible to participate (i.e., LNS was used for treatment, not prevention, of malnutrition); *2*) the trial was conducted in a hospitalized population or among children with a pre-existing disease; or *3*) SQ-LNS provision was combined with additional supplemental food or nutrients for the child within a single arm (e.g., SQ-LNS + food rations compared with control), and there was no appropriate comparison group (e.g., food rations alone) that would allow separation of the SQ-LNS effect from effects of the other food or nutrients provided.

Trials in which there were multiple relevant SQ-LNS interventions (e.g., varying dosages or formulations of SQ-LNSs in different arms), which combined provision of child SQ-LNS with provision of maternal LNS, or which included other nonnutritional interventions [i.e., water, sanitation, and hygiene (WASH)] were eligible for inclusion. In such trials, all arms that provided child SQ-LNSs were combined into 1 group, and all non-LNS arms (i.e., no LNS for mother or child) were combined into a single comparator group for each trial (herein labeled “control”), excluding intervention arms that received non-LNS child supplementation (e.g., MNP, fortified blended food). We also conducted a sensitivity analysis restricting the comparison to specified contrasts of intervention arms within multiple-intervention trials (see below).

At the individual participant level, we included children if their age at baseline would have allowed them to receive ≥3 mo of intervention (supplementation or control group components) between 6 and 24 mo of age. We considered 3 mo to be the minimum duration for an impact on linear growth.

### Search methods and identification of studies

First, we identified studies cited in a recent systematic review and meta-analysis of child LNSs (16). We then used the same search terms used by that systematic review to search 16 international and 9 regional databases (see **Supplemental Methods**) for additional studies published from 1 July, 2018 until 1 May, 2019. One author (KRW) reviewed the titles and abstracts of all studies included in the previous systematic review, as well as the additional studies identified by our database searches, to select all potentially relevant studies for full-text review. These were screened based on the inclusion and exclusion criteria. In September 2019, KRW searched for additional publications from studies that met the inclusion and exclusion criteria to determine if results for outcomes of interest had been published subsequent to the search.

### Data collection

We invited all principal investigators of eligible trials to participate in this IPD meta-analysis. We provided a data dictionary listing definitions of variables requested for pooled analysis. Those variables were provided to the IPD analyst (CDA) in de-identified IPD sets. The IPD analyst communicated with investigators to request any missing variables or other clarifications, as needed.

### IPD integrity

We conducted a complete-case intention-to-treat analysis (23). We checked data for completeness by evaluating whether the study sample sizes in our pooled data set were the same as in study protocols and publications. We calculated length-for-age *z* score (LAZ), weight-for-length *z* score (WLZ), weight-for-age *z* score (WAZ), midupper arm circumference-for-age *z* score (MUACZ), and head circumference-for-age *z* score (HCZ) using the 2006 WHO child growth standards and checked the values for acceptable SDs and whether they were within published WHO acceptable ranges (24). Biologically implausible values were flagged, as recommended by the WHO, in the following way: LAZ <−6 or >6; WAZ <−6 or >5; WLZ <−5 or >5; HCZ <−5 or >5, and MUACZ <−5 or >5. These were inspected for errors and either winsorized (25) or removed from analysis on an outcome-by-outcome basis. Such cleaning was necessary for <0.5% of participants, with a consistently low rate of implausibility across outcomes and studies. We also checked summary statistics, such as means ± SDs, in our data set against published values for each trial.

### Assessment of risk of bias in each study and quality of evidence across studies

Two independent reviewers (KRW and CDA) assessed risk of bias in each trial against the following criteria: random sequence generation and allocation concealment (selection bias), blinding of participants and personnel (performance bias), blinding of outcome assessment (detection bias), incomplete outcome data (attrition bias), selective reporting (reporting bias), and other sources of bias (26). Any discrepancies were resolved by discussion or consultation with the core working group, as needed. The same reviewers also assessed the quality of evidence for anthropometric outcomes across all trials based on the 5 Grading of Recommendations Assessment, Development and Evaluation (GRADE) criteria: risk of bias, inconsistency of effect, imprecision, indirectness, and publication bias (27).

### Specification of outcomes and effect measures

We prespecified all anthropometric outcomes in the statistical analysis plan (20). Continuous outcomes included LAZ, WLZ, WAZ, MUACZ, and HCAZ. Binary outcomes were stunting (LAZ < −2 SD), wasting (WLZ < −2 SD), underweight (WAZ < −2 SD), small head size (HCAZ < −2 SD), low MUAC (MUACZ < −2 SD or MUAC < 125 mm), and acute malnutrition (WLZ < −2 SD or MUAC < 125 mm). For estimation of main effects, the primary outcomes were LAZ, WLZ, stunting, and wasting.

The principal measure of effect for continuous outcomes was the mean difference (MD) between intervention and comparison groups at endline, defined as the principal postintervention time point as reported for trials with infrequent child assessment or at the age closest to the end of the supplementation period for trials with monthly child assessment. The principal measure of effect for binary outcomes was the prevalence ratio (PR; relative difference in proportions between groups) at endline. We also estimated prevalence differences (PDs; in absolute percentage points) because of their importance for estimating public health impact, but considered them as secondary assessments of binary outcomes because such estimates are less consistent than PRs (26).

The treatment and comparisons of interest were provision of children with SQ-LNSs (<∼125 kcal/d, with or without co-interventions), compared with provision of no intervention or an intervention without any type of LNS or other child supplement. Other types of interventions have been delivered with or without LNS, such as WASH interventions and child morbidity monitoring and treatment. In several trials, child LNS has been delivered to children whose mothers received maternal LNS during pregnancy and lactation. Given that maternal supplementation may have an additive effect when LNS is provided to both mothers and their children, we originally planned to include trial arms that provided both maternal and child LNS in a sensitivity analysis only (i.e., the all-trials analysis). However, to maximize study inclusion and participant sample size, and allow for sufficient numbers of trials to examine effect modification for certain outcomes, we decided after initial registration of the protocol but before completing statistical analyses that if the main effects did not differ between the child-LNS-only analysis and the all-trials analysis (including maternal plus child LNS arms) by >20% for continuous outcomes or by >0.05 for PRs, the results of the all-trials analyses would be presented as the principal findings. Three additional prespecified sensitivity analyses were also conducted, as described below.

### Synthesis methods and exploration of variation in effects

We conducted 3 types of analyses to separately investigate *1*) full-sample main effects of the intervention, *2*) effect modification by study-level characteristics, and *3*) effect modification by individual-level characteristics. We used a 2-stage approach for all 3 sets of analyses. This approach is preferred when incorporating cluster-randomized trials, because it allows intracluster correlations to be study-specific (28). In the first stage, we generated intervention effect estimates within each individual study according to its study design. For longitudinal study designs we controlled for baseline anthropometric status (for each outcome) when estimating the intervention effect to gain efficiency. To deal with outcome dependence in cluster-randomized trials, we used robust SEs with randomization clusters as the independent unit. In the second stage, we pooled the first-stage estimates using inverse-variance weighted fixed effects. A fixed-effect approach generates estimates viewed as a typical intervention effect from the studies included in the analysis. This was prespecified in our statistical analysis plan because we anticipated similar intervention effects and similar individual-level effect modification patterns across studies. As a robustness check of this assumption, we also conducted sensitivity analyses in which we pooled estimates using inverse-variance weighted random effects (29, 30). If there were <3 comparisons to include in a pooled estimate then the pooled estimate was not generated (e.g., if <3 comparisons were represented within a study-level effect modification category).

1. *Full-sample main effects of the intervention:* We first estimated the intervention effect for each study. We then pooled the first-stage estimates to generate a pooled point estimate, 95% CI, and corresponding *P* value.
2. *Effect modification by study-level characteristics:* We identified potential study-level effect modifiers before receipt of data, and categorized individual studies based on the distribution of effect modifier values across all studies (**Box 1**). Study-level characteristics included variables reflecting context as well as aspects of study design. We used random-effects meta-regression to test the association between each effect modifier and the intervention. The random-effects approach is used when exploring heterogeneity across studies. In the first stage of analysis, we estimated the parameter corresponding to the intervention effect as aforementioned. In the second stage, we used a bivariate random-effects meta-regression to test the association between study intervention effect and study-level characteristics and also generated strata-level pooled estimates to aid interpretation.
3. *Effect modification by individual-level characteristics*: We identified potential individual-level characteristics based on a comprehensive review of effect modifiers considered by individual trials (either listed a priori in statistical analysis plans or as published) and selected based on biological plausibility (Box 1). Individual-level effect modifiers included maternal, child, and household characteristics. We estimated the parameter corresponding to the interaction term of the effect modifier and the intervention for each study (31), as follows. For categorical effect modifiers, we first recoded them to create binary variables if needed, and then determined the interaction between the intervention and the binary effect modifier. All continuous effect modifiers were transformed into binary variables for the analysis by modeling the relation within each study using splines and then pooling the first-stage estimates to generate a pooled, fitted line. We defined programmatically useful dichotomous cutoffs based on the pooled fitted spline results and relevant context. We then generated pooled intervention effect estimates within each category to determine how the intervention effect in 1 subgroup differed from the intervention effect in the specified reference subgroup.

Box 1: Potential effect modifiers^1^

**Table:** 

Study-level effect modifiers	Individual-level maternal, child, and household effect modifiers
• Geographic region (WHO region: African vs. South-East Asia Region)• Stunting burden among control group children at 18 mo of age (≥35% vs. <35%)^2^• Malaria prevalence (country-specific, closest in time to the study: ≥10% vs. <10%)^3^• Water quality (study-specific, <75% vs. ≥75% prevalence of improved drinking water)^4^• Sanitation (study-specific, <50% vs. ≥50% prevalence of improved sanitation)^5^• Duration of child supplementation (study target: >12 mo vs. ≤12 mo)• Child age at baseline or endline• Frequency of contact for intervention delivery or outcome assessments during the study (weekly vs. monthly)• Compliance (average percentage compliance in LNS group: mean compliance ≥80% vs. <80%)^6^	• Maternal height (<150.1 cm vs. ≥150.1 cm)^7^• Maternal BMI (in kg/m^2^) (<20 vs. ≥20)• Maternal age (<25 y vs. ≥25 y)• Maternal education (no formal or incomplete primary vs. complete primary or greater)• Maternal depressive symptoms (lower, < study 75^th^ percentile vs. higher, ≥ study 75^th^ percentile)^8^• Child sex (female vs. male)• Child birth order (firstborn vs. later-born)• Child baseline anthropometric status (lower vs. higher)^9^• Household socioeconomic status (< study median vs. ≥ study median)^10^• Food security (moderate to severe food insecurity vs. mild food insecurity to secure)^11^• Source water quality (unimproved vs. improved)^4^• Sanitation (unimproved vs. improved)^4^• Home environment (< study median vs. ≥ study median)^12^• Season at the time of growth outcome assessment (rainy vs. dry)^13^

1 Comparisons follow the format nonreference vs. reference category. HCZ, head circumference-for-age *z* score; LAZ, length-for-age *z* score; LNS, lipid-based nutrient supplement; MUACZ, midupper arm circumference-for-age *z* score; WASH, water, sanitation, and hygiene; WAZ, weight-for-age *z* score; WLZ, weight-for-length *z* score.

^2^Based on 18-mo data because baseline data were not available for all trials; the cutoff was chosen at approximately the median across trials.

^3^ *World Malaria Report 2018* (88); the cutoff was chosen based on the median across trials.

^4^Improved water source includes piped water, boreholes or tubewells, protected dug wells or springs, rainwater, and packaged or delivered water (see Supplemental Table 3) (89); based on baseline data, excluding arms that received WASH interventions; the cutoff was chosen at approximately the median across trials.

^5^Improved sanitation includes flush/pour flush to piped sewer system, septic tanks, or pit latrines; ventilated improved pit latrines, composting toilets, or pit latrines with slabs (see Supplemental Table 3) (90); based on baseline data, excluding arms that received WASH interventions; the cutoff was chosen at approximately the median across trials.

^6^Study-specific, as reported based on a study-defined indicator (see Supplemental Table 2); the cutoff was chosen based on the median across trials.

^7^Cutoff is −2 SD for height at 19 y of age: https://www.who.int/growthref/hfa_girls_5_19years_z.pdf?ua=1.

^8^Study-specific (see Supplemental Table 3); the cutoff was chosen to reflect the top quartile for risk of depression.

^9^LAZ< vs. ≥−1 when LAZ or stunting is the outcome; WLZ< vs. ≥0 when WLZ, wasting, or acute malnutrition is the outcome; MUACZ< vs. ≥0 when MUACZ or low midupper arm circumference is the outcome; WAZ< vs. ≥−1 when WAZ or underweight is the outcome; HCZ< vs. ≥−1 when HCZ or small head size is the outcome.

^10^Based on a study-defined, study-specific assets index.

^11^Study-specific (see Supplemental Table 3).

^12^As measured by the Family Care Indicators, Home Observation for the Measurement of the Environment Inventory, or other similar tools (see Supplemental Table 3).

^13^Rainy vs. dry, based on study- and child-specific average rainfall during the month of measurement and 2 mo prior (see Supplemental Methods and Supplemental Table 3).

Heterogeneity of effect estimates was assessed using *I*^2^ and Tau^2^ statistics, within strata when relevant (32). We used a *P* value <0.05 for main effects and a *P*-diff or *P*-interaction < 0.10 for effect modification by study-level or individual-level characteristics, respectively. Given that the growth outcomes are highly correlated and the effect modification analyses are inherently exploratory, we did not adjust for multiple hypothesis testing because doing so may be unnecessary and counterproductive (33).

To aid in interpretation of individual-level effect modification, we evaluated the results for binary outcomes to identify what we will call the “cutoff effect.” The distribution of the continuous outcome relative to the cutoff for the corresponding binary outcome (e.g., distribution of LAZ around the −2 SD stunting cutoff) in the 2 effect modifier subgroups can influence the PR and PD. When the mean in each of the 2 subgroups falls in a different location relative to the cutoff, the proportion of children close to the cutoff may be different between subgroups. This can lead to a greater reduction in the adverse binary outcome within one subgroup than within the other even if the shift in the mean value due to SQ-LNS is the same in both subgroups. To examine this, we simulated what would happen if we shifted the distribution of the nonreference effect modification subgroup to align with the reference subgroup (see Box 1), while maintaining the observed intervention effect MD in the continuous outcome within each subgroup. Based on ad hoc, pragmatic criteria, if the *P*-interaction shifted from <0.1 to >0.2, we concluded that the cutoff effect explained the apparent effect modification; if it shifted from <0.1 to >0.1 but <0.2, we concluded that the cutoff effect partially contributed to the apparent effect modification.

### Additional sensitivity analyses

Most trials have utilized similar SQ-LNS distribution mechanisms (e.g., weekly or monthly rations provided by study staff, community health workers, or other health extension agents), accompanied by messages to reinforce recommended infant and young child feeding (IYCF) practices. In addition, most trials have used similar formulations of SQ-LNS, specifically peanut- and milk-based products providing ∼1 RDA of most micronutrients (14). However, variations in trial design (e.g., integration of SQ-LNS supplementation with WASH interventions or enhanced morbidity monitoring and treatment; use of passive compared with active control arms) might influence the effect size estimates. In addition, some trials used several different formulations of SQ-LNSs. We therefore conducted several prespecified sensitivity analyses:

1. Separate comparisons within multicomponent intervention trials, such that the SQ-LNS with no SQ-LNS comparisons were conducted separately between pairs of arms with the same nonnutrition components (e.g., SQ-LNS + WASH compared with WASH; SQ-LNS compared with control). IYCF behavior change communication was not considered an additional component.
2. Exclusion of passive control arms, i.e., control group participants received no intervention and had no contact with project staff between baseline and endline.
3. Exclusion of intervention arms with SQ-LNS formulations that did not include both milk and peanut.

In addition, we conducted post hoc analyses to examine effects within subgroups of trials based on 2 aspects of the intervention design: *1*) whether the trial was or was not conducted within an existing program, and *2*) the extent of the social and behavior change communication (SBCC) on IYCF that was provided (minimal compared with expanded).

## Results

### Literature search and trial characteristics

**FIGURE 1:**
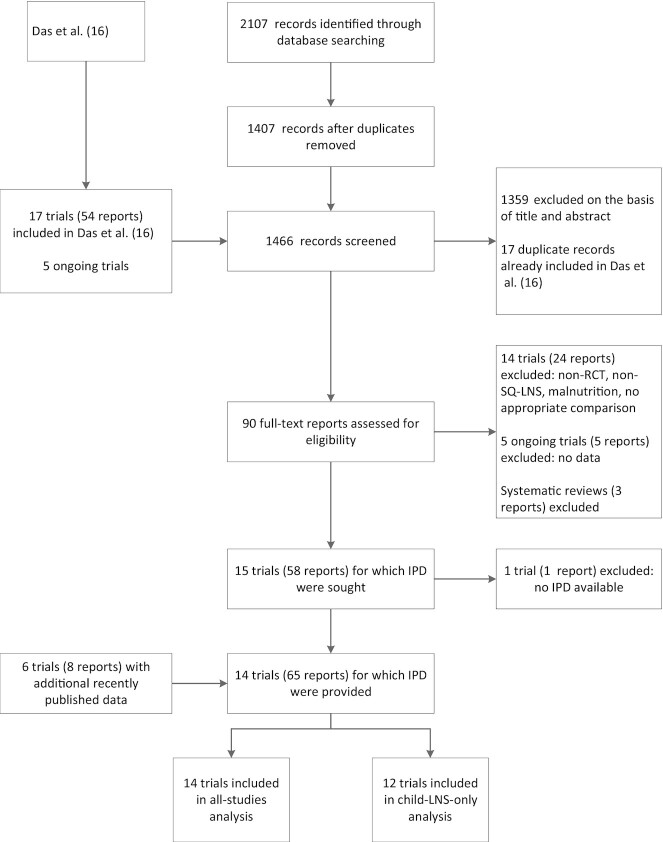
Study flow diagram. IPD, individual participant data; LNS, lipid-based nutrient supplement; SQ, small-quantity; RCT, randomized controlled trial.

We identified 15 trials that met our inclusion criteria, 14 of which provided IPD and are included in this analysis (**Figure 1**, **Table 1**) (34–48). Investigators for 1 trial were unable to participate (49). In that trial, LAZ and WAZ were reported (but not binary outcomes); therefore, we examined pooled main effects on those 2 outcomes both without and with that trial, by calculating Hedges’ *g* (50) based on endline values extracted from the published report. One trial was designed a priori to present results separately for HIV-exposed and HIV-unexposed children; therefore, we present it herein as 2 separate comparisons in all analyses (47, 48). Similarly, the 2 PROMIS trials in Burkina Faso and Mali each included an independent longitudinal cohort and repeated (at baseline and endline) cross-sectional samples, so the longitudinal and cross-sectional results are presented as separate comparisons for each trial (38, 46).

**TABLE 1:** Characteristics of trials included in the individual participant data analysis and the analytic contrasts in which they were included^1^

		Child SQ-LNS supplementation			Analysis contrasts				
Country, years of study, study name, *n*, trial design, authors	Intervention groups	Age at start, mo	Duration, mo	IYCF messages^2^	All-trials analysis	Child-LNS-only analysis	Separation of multicomponent arms	Passive control arms excluded	Nonmilk, nonpeanut LNS arms excluded
Bangladesh, 2012–2014, JiVitA-4, *n* = 4218, cluster RCT, longitudinal follow-up, Christian et al. (34)	Plumpy'Doz^3^ + IYCF counseling	6	12	Expanded	LNS	LNS	LNS	LNS	LNS
	Chickpea-based LNS + IYCF counseling	6	12	Expanded	LNS	LNS	LNS	LNS	—
	Rice-lentil LNS + IYCF counseling	6	12	Expanded	LNS	LNS	LNS	LNS	—
	WSB++ + IYCF counseling	6	12	Expanded	—	—	—	—	—
	IYCF counseling only (control)			Expanded	Control	Control	Control	Control	Control
Bangladesh, 2011–2015, RDNS, *n* = 2478, cluster RCT, longitudinal follow-up, Dewey et al. (35)	LNS-LNS: maternal SQ-LNS in pregnancy + 6 mo postpartum, child SQ-LNS 6–24 mo	6	18	Minimal	LNS	—	—	—	—
	IFA-LNS: maternal IFA in pregnancy + 3 mo postpartum, child SQ-LNS 6–24 mo	6	18	Minimal	LNS	LNS	LNS	LNS	LNS
	IFA-MNP: maternal IFA in pregnancy + 3 mo postpartum, child MNP 6–24 mo			Minimal	—	—	—	—	—
	IFA-control: maternal IFA in pregnancy + 3 mo postpartum, no child supplementation			Minimal	Control	Control	Control	Control	Control
Bangladesh, 2012–2015, WASH-Benefits, *n* = 4633, cluster RCT, cross-sectional surveys, Luby et al. (36)	Nutrition: SQ-LNS + IYCF counseling	6	18	Expanded	LNS	LNS	LNS	LNS	LNS
	Water: family received chlorine and container for drinking water and counseling on safe water storage and consumption				Control	Control	—	Control	Control
	Sanitation: family received upgraded latrine, sani-scoop, and child potty, and counseling on their use				Control	Control	—	Control	Control
	Handwashing: family received handwashing stations with soap and handwashing counseling				Control	Control	—	Control	Control
	WASH: family received all water, sanitation, and handwashing interventions				Control	Control	Control-WASH	Control	Control
	WASH + nutrition: all water, sanitation, handwashing, and nutrition interventions	6	18	Expanded	LNS	LNS	LNS-WASH	LNS	LNS
	Passive control (no intervention)				Control	Control	Control	—	Control
Burkina Faso, 2010–2012, iLiNS-ZINC, *n* = 2626, cluster RCT, longitudinal follow-up, Hess et al. (37)	LNS-Zn0: SQ-LNS containing 0 mg Zn/d and placebo tablet^4^	9	9	Minimal	LNS	LNS	LNS	—	LNS
	LNS-Zn5: SQ-LNS containing 5 mg Zn/d and placebo tablet	9	9	Minimal	LNS	LNS	LNS	—	LNS
	LNS-Zn10: SQ-LNS containing 10 mg Zn/d and placebo tablet	9	9	Minimal	LNS	LNS	LNS	—	LNS
	LNS-TabZn5: SQ-LNS containing 0 mg Zn/d and zinc tablet containing 5 mg Zn/d	9	9	Minimal	LNS	LNS	LNS	—	LNS
	Passive control (no intervention)				Control	Control	Control	—	Control
Burkina Faso, 2015–2017, PROMIS, *n* = 2651, cluster RCT, longitudinal follow-up and cross-sectional surveys,^5^ Becquey et al. (38)	SQ-LNS + IYCF counselingActive control (standard of care)	6	12	Expanded	LNSControl	LNSControl	LNSControl	LNSControl	LNSControl
Ghana, 2004–2005, *n* = 194, RCT, longitudinal follow-up, Adu-Afarwuah et al. (39)	SQ-LNS	6	6	Minimal	LNS	LNS	LNS	—	LNS
	MNP			Minimal	—	—	—	—	—
	Nutritabs (MMN)			Minimal	—	—	—	—	—
	Passive control (no intervention)				Control	Control	Control	—	Control
Ghana, 2009–2014, iLiNS-DYAD-G, *n* = 1040, RCT, longitudinal follow-up, Adu-Afarwuah et al. (40)	LNS: maternal SQ-LNS in pregnancy + 6 mo postpartum, child SQ-LNS 6–18 mo	6	12	Minimal	LNS	—	—	—	—
	MMN: maternal MMN in pregnancy + 6 mo postpartum, no child supplementation			Minimal	Control	—	—	—	—
	IFA: maternal IFA in pregnancy and placebo for 6 mo postpartum, no child supplementation			Minimal	Control	—	—	—	—
Haiti, 2011–2012, *n* = 300, RCT, longitudinal follow-up, Iannotti et al. (41)	SQ-LNS for 6 moActive control (standard of care)	6–11	6^6^	MinimalMinimal	LNSControl	LNSControl	LNSControl	LNSControl	LNSControl
Kenya, 2012–2016, WASH-Benefits, *n* = 6649, cluster RCT, cross-sectional surveys, Null et al. (42)	Nutrition: SQ-LNS + IYCF counseling	6	18	Expanded	LNS	LNS	LNS	LNS	LNS
	Water: community and family received chlorine for drinking water and family received counseling on safe water storage and consumption				Control	Control	—	Control	Control
	Sanitation: family received upgraded latrine, sani-scoop, and child potty, and counseling on their use				Control	Control	—	Control	Control
	Handwashing: family received handwashing stations with soap and handwashing counseling				Control	Control	—	Control	Control
	WASH: family received all water, sanitation, and handwashing interventions				Control	Control	Control-WASH	Control	Control
	WASH + nutrition: all water, sanitation, handwashing, and nutrition interventions	6	18	Expanded	LNS	LNS	LNS-WASH	LNS	LNS
	Passive control (no intervention)				Control	Control	Control	—	Control
	Active control (visits to measure MUAC)				Control	Control	Control	Control	Control
Madagascar, 2014–2016, MAHAY, *n* = 3390, cluster RCT, longitudinal follow-up, Galasso et al. (43)	T4: early child stimulation + IYCF counseling			Expanded	—	—	—	—	—
	T3: maternal SQ-LNS in pregnancy + 6 mo postpartum, child SQ-LNS 6–18 mo + IYCF counseling	6–11	6–12	Expanded	LNS	—	—	—	—
	T2: child SQ-LNS 6–18 mo + IYCF counseling	6–11	6–12	Expanded	LNS	LNS	LNS	LNS	LNS
	T1: IYCF counseling			Expanded	Control	Control	Control	Control	Control
	T0: control (standard of care)				Control	Control	Control	Control	Control
Malawi, 2011–2014, iLiNS-DYAD-M, *n* = 664, RCT, longitudinal follow-up, Ashorn et al. (44)	LNS: maternal SQ-LNS in pregnancy + 6 mo postpartum, child SQ-LNS 6–18 mo	6	12	Minimal	LNS	—	—	—	—
	MMN: maternal MMN in pregnancy + 6 mo postpartum, no child supplementation			Minimal	Control	—	—	—	—
	IFA: maternal IFA in pregnancy and placebo for 6 mo postpartum, no child supplementation			Minimal	Control	—	—	—	—
Malawi, 2009–2012, iLiNS-DOSE, *n* = 943, RCT, longitudinal follow-up, Maleta et al. (45)^7^	SQ-LNS containing milk (10 g/d)	6	12	Minimal	LNS	LNS	LNS	LNS	LNS
	SQ-LNS containing milk (20 g/d)	6	12	Minimal	LNS	LNS	LNS	LNS	LNS
	SQ-LNS without milk (20 g/d)	6	12	Minimal	LNS	LNS	LNS	LNS	—
	MQ-LNS containing milk (40 g/d)			Minimal	—	—	—	—	—
	MQ-LNS without milk (40 g/d)			Minimal	—	—	—	—	—
	Active control			Minimal	Control	Control	Control	Control	Control
Mali, 2015–2017, PROMIS, *n* = 2937, cluster RCT, longitudinal follow-up and cross-sectional surveys,^5^ Huybregts et al. (46)	SQ-LNS + IYCF and WASH counseling + screening for acute malnutrition	6	18	Expanded	LNS	LNS	LNS	LNS	LNS
	Active control (standard of care) + IYCF and WASH counseling + screening for acute malnutrition			Expanded	Control	Control	Control	Control	Control
Zimbabwe, 2013–2017, SHINE,^8^*n* = 4343, cluster RCT, longitudinal follow-up, Humphrey et al. (47) and Prendergast et al. (48)	IYCF: child SQ-LNS + IYCF counseling	6	12	Expanded	LNS	LNS	LNS	LNS	LNS
	WASH: family received ventilated improved pit latrine, handwashing stations, soap, chlorine, child play space, and WASH counseling				Control	Control	Control-WASH	Control	Control
	WASH and IYCF: child SQ-LNS + IYCF counseling, family received ventilated improved pit latrine, handwashing stations, soap, chlorine, child play space, and WASH counseling	6	12	Expanded	LNS	LNS	LNS-WASH	LNS	LNS
	Active control (standard of care)				Control	Control	Control	Control	Control

1 IFA, iron–folic acid; IYCF, infant and young child feeding; LNS, lipid-based nutrient supplement; MMN, multiple micronutrients; MNP, multiple micronutrient powder; MQ, medium-quantity; MUAC, midupper arm circumference; RCT, randomized controlled trial; RDNS, Rang-Din Nutrition Study; SQ, small-quantity; WASH, water, sanitation, and hygiene; WSB, wheat–soy blend.

2 Minimal IYCF messages defined as providing minimal counseling on IYCF other than reinforcing the normal IYCF messages already promoted in that setting. Expanded IYCF messages defined as providing expanded counseling on IYCF that went beyond the usual messaging.

3 All supplements were isocaloric, children age 6–12 mo received 125 kcal/d, children age 12–18 mo received 250 kcal/d.

4 All children in the 4 intervention groups received oral rehydration solution for diarrhea and treatment for malaria.

5 Cross-sectional and longitudinal cohorts within this trial are considered as separate comparisons in all analyses and the presentation of results.

6 Trial also included a 3 mo duration intervention arm which is excluded from these analyses because there is no comparable control arm available.

7 Trial is cited as Kumwenda 2014 in Das et al. (16).

8 Trial was designed a priori to present results separately for HIV-exposed and -unexposed children; thus considered as 2 comparisons in all analyses and the presentation of results.

The 14 trials in these analyses were conducted in Sub-Saharan Africa (10 trials in 7 countries), Bangladesh (3 trials), and Haiti (1 trial), and included a total of 37,066 infants and young children with anthropometric data. The majority of trials began child supplementation with SQ-LNSs at 6 mo of age and the intended duration ranged from 6 to 18 mo of supplementation; 4 trials included intervention arms that also provided SQ-LNSs to mothers during pregnancy and the first 6 mo postpartum (35, 40, 43, 44). All trials provided a peanut- and milk-based SQ-LNS in ≥1 of the arms (Table 1, **Supplemental Table 1**). Generally, this provided ∼120 kcal/d and ∼1 RDA of most micronutrients (19 micronutrients in 3 trials, 22 micronutrients in 11 trials); in 1 trial the ration was ∼120 kcal/d between 6 and 12 mo of age and ∼250 kcal/d between 12 and 24 mo of age (34). Two trials included additional arms with different formulations or doses of SQ-LNS (34, 45). Six trials were conducted within existing community-based or clinic-based programs (35, 38, 41, 43, 46–48); in the other trials, all activities were conducted by research teams. Seven trials provided minimal SBCC on IYCF other than reinforcing the normal IYCF messages already promoted in that setting (35, 37, 39–41, 44, 45), and 7 trials provided expanded SBCC on IYCF that went beyond the usual messaging, either in just the SQ-LNS intervention arms (36, 38, 42, 47, 48) or in all arms including the non-SQ-LNS control arm (34, 43, 46). Three trials included arms with WASH interventions (36, 42, 47, 48). Most trials included an active control arm (i.e., similar contact frequency as for intervention arms) but 3 included only a passive control arm (36, 37, 39).

Descriptive information on the potential study-level and individual-level effect modifiers (defined in Box 1), by trial, is presented in **Supplemental Tables 2** and **3**, respectively. At the study level, 8 of the 14 study sites had a high burden of stunting (≥35% in the control group at 18 mo). Country-level malaria prevalence ranged from <1% in Bangladesh and Haiti to 59% in Burkina Faso. Study-specific prevalence of improved water quality ranged from 27% to 100%, whereas prevalence of improved sanitation ranged from 0% to 97%. Frequency of contact during the study was weekly in 7 trials and monthly in 7 trials. Average estimated reported compliance with SQ-LNS consumption was categorized as high (≥80%) in 7 trials and ranged between 37% and 77% in the other trials. The following maternal characteristics varied widely across trials: short stature (<150.1 cm) ranged from <2% in Burkina Faso (37) to >45% in Bangladesh (35, 36); BMI < 20 kg/m^2^ ranged from 9% in Ghana (39) to 55% in Bangladesh (35); age < 25 y ranged from 24% among HIV-positive women in Zimbabwe (48) to 73% in Bangladesh (35); completion of primary education ranged from 3.8% in Burkina Faso (37) to 96.3% in Zimbabwe (47); and reported moderate to severe household food insecurity ranged from 10.3% in Kenya (42) to 73.5% in Malawi (45).

Growth outcomes in the control groups at endline showed that the burden of child malnutrition varied across studies (**Supplemental Table 4**). Prevalence of stunting ranged from 7.3% in the first Ghana trial (39) to 58.5% in Madagascar (43), whereas prevalence of wasting ranged from <2% (in the Haiti and WASH-Benefits Kenya trials) (41, 42) to 16.4% in Bangladesh (34–36). The range in prevalence for the other binary outcomes was 1.5%–18.3% for low MUAC, 3.2%–21.1% for acute malnutrition, 4.7%–39.2% for underweight, and 4.3%–42.9% for small head size. High WLZ (>1) was uncommon, with a prevalence >10% in only 5 trials.

In general, we considered the trials to have a low risk of bias (**Supplemental Table 5**, **Supplemental Figure 1**). All trials, including the program-based trials, were judged to have low risk of bias for 5 of the 7 categories in Supplemental Table 5: random sequence generation (except for 1 trial labeled “unclear”), allocation concealment, incomplete outcome, selective reporting, and “other.” For blinding of participants, all trials were judged to have high risk of bias, because blinding was not possible given the nature of the intervention. Risk of bias in outcome assessment was mixed (5 low, 9 high) because some trials did not clearly specify whether data collectors who performed the anthropometric measurements were kept unaware of group allocation.

### Main effects of SQ-LNSs on growth outcomes

Results from the child-LNS-only and all-trials analyses were similar: for nearly all outcomes, the MDs, PRs, and PDs for intervention compared with control groups were almost identical or slightly less favorable when the maternal LNS trials/arms were included (**Supplemental Figure 2**A–C). Therefore, results from the all-trials analyses, inclusive of maternal + child LNS trials/arms, are presented below, and in **Table 2**. Sample sizes for the all-trials analyses were considerably larger than for the child-LNS only analyses: for example, the total pooled sample size for stunting was ∼37,000 and ∼33,400, respectively. For LAZ, WLZ, WAZ, stunting, and wasting, all 14 trials (17 comparisons) were represented. Some trials did not measure MUAC or head circumference, so the number of trials (comparisons) was 11 (14) for MUACZ, low MUAC, and acute malnutrition and 10 (11) for HCZ and small head size.

**TABLE 2:** Main effects of small-quantity LNSs on growth outcomes^1^

	*n* Participants (comparisons)	MD/PR/PD^2^	*P* value^3^	Heterogeneity *I*^2^ (*P*-heterogeneity)^3^	Quality of the evidence (GRADE)
Continuous outcomes					
LAZ^4^	36,795 (17)	0.14 (0.11, 0.16)^5^	<0.001	0.65 (<0.001)	High
WLZ^4^	36,608 (17)	0.08 (0.06, 0.10)	<0.001	0.51 (0.008)	High
MUACZ	31,774 (14)	0.09 (0.06, 0.11)	<0.001	0.62 (0.001)	High
WAZ	36,787 (17)	0.13 (0.11, 0.15)^5^	<0.001	0.66 (<0.001)	High
HCZ	27,650 (11)	0.09 (0.06, 0.11)	<0.001	0.46 (0.045)	High
Binary outcomes					
Stunting (LAZ < −2 SD)^4^	36,795 (17)	0.88 (0.85, 0.91)	<0.001	0.49 (0.013)	High
		−5.0 (−4.1, −5.9)	<0.001	0.54 (0.005)	High
Wasting (WLZ < −2 SD)^4^	36,311 (16)	0.86 (0.80, 0.93)	<0.001	0.00 (0.872)	High
		−0.6 (−0.1, −1.0)	0.010	0.12 (0.321)	High
Low MUAC (MUACZ < −2 SD orMUAC < 125 mm)	31,774 (14)	0.82 (0.75, 0.89)	<0.001	0.16 (0.281)	High
		−0.9 (−0.5, −1.4)	<0.001	0.50 (0.018)	High
Acute malnutrition (WLZ < −2 orMUAC < 125 mm)	31,440 (14)	0.86 (0.80, 0.93)	<0.001	0.00 (0.554)	High
		−1.1 (−0.5, −1.6)	<0.001	0.21 (0.228)	High
Underweight (WAZ < −2 SD)	36,787 (17)	0.87 (0.83, 0.91)	<0.001	0.42 (0.031)	High
		−3.1 (−2.3, −3.8)	<0.001	0.60 (0.001)	High
Small head size (HCZ < −2 SD)	27,456 (10)	0.91 (0.86, 0.95)	<0.001	0.00 (0.816)	High
		−1.2 (−0.4, −1.9)	0.002	0.23 (0.230)	High

1 GRADE, Grading of Recommendations Assessment, Development and Evaluation; HCZ, head circumference-for-age *z* score; LAZ, length-for-age *z* score; LNS, lipid-based nutrient supplement; MD, mean difference; MUAC, midupper arm circumference; MUACZ, midupper arm circumference-for-age *z* score; PD, prevalence difference; PR, prevalence ratio; WAZ, weight-for-age *z* score; WLZ, weight-for-length *z* score.

2 For continuous outcomes, values are MDs: LNS – control (95% CIs). For binary outcomes, values are PRs (first row) or PDs (second row): LNS compared with control (95% CIs).

3 The *P* value column corresponds to the pooled main effect 2-sided superiority testing of the intervention effect estimate and 95% CI presented in the preceding column. *I*^2^ describes the percentage of variability in effect estimates that may be due to heterogeneity rather than chance. Roughly, 0.3–0.6 may be considered moderate heterogeneity. *P* value from chi-square test for heterogeneity. *P* < 0.05 indicates statistically significant evidence of heterogeneity of intervention effects beyond chance.

4 Primary outcomes.

5 MD values were LAZ +0.14 (95% CI: 0.11, 0.16) and WAZ +0.12 (95% CI: 0.10, 0.14) when results from the 1 trial that did not participate in the IPD analyses were included (49).

SQ-LNSs had a significant positive effect on all growth outcomes, both continuous and binary. Among the continuous outcomes, the MD between intervention groups was largest for LAZ (+0.14) and smallest for WLZ (+0.08). SQ-LNSs reduced the prevalence of adverse growth outcomes by 12% for stunting (5 percentage points), 14% for wasting and acute malnutrition (1 percentage point for each), 18% for low MUAC (1 percentage point), 13% for underweight (3 percentage points), and 9% for small head size (1 percentage point). We rated the quality of the evidence for all outcomes as high based on the GRADE criteria listed in the Methods: ≥10 RCTs were available for all outcomes, risk of bias was low, heterogeneity was generally low to moderate (Table 2), precision was rated as high because all but 2 trials had sample sizes > 600, all trials were directly aimed at evaluating SQ-LNSs, and funnel plots revealed no indication of publication bias (27).

**FIGURE 2:**
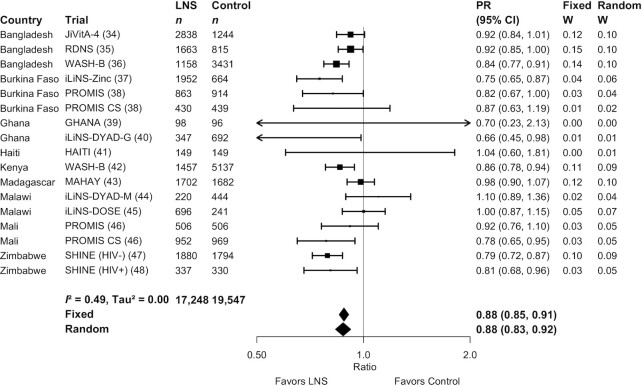
Forest plot of effect of small-quantity LNSs on stunting prevalence. Individual study estimates were generated from log-binomial regression controlling for baseline measure when available and with clustered observations using robust SEs for cluster-randomized trials. Pooled estimates were generated using inverse-variance weighting with both fixed and random effects. LNS, lipid-based nutrient supplement; PR, prevalence ratio.

**FIGURE 3:**
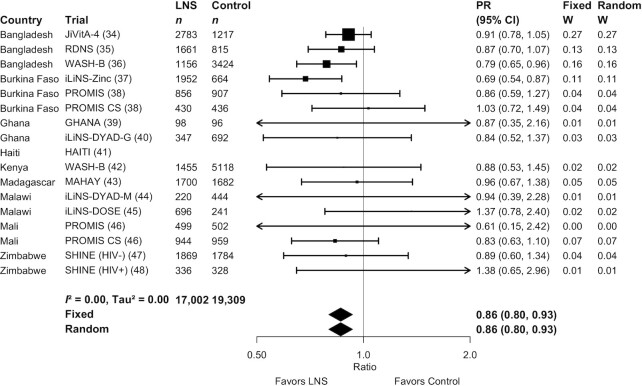
Forest plot of effect of small-quantity LNSs on wasting prevalence. Individual study estimates were generated from log-binomial regression controlling for baseline measure when available and with clustered observations using robust SEs for cluster-randomized trials. Pooled estimates were generated using inverse-variance weighting with both fixed and random effects. LNS, lipid-based nutrient supplement; PR, prevalence ratio.

**Figures 2** and **3** show forest plots for stunting and wasting PRs, respectively. **Supplemental Figure 3**A–Q shows forest plots for all other outcomes. Figure 2 shows that there was moderate heterogeneity (*I*^2^ = 0.49) in the effect on stunting prevalence across trials. For wasting, the *I*^2^ value was 0 (Figure 3), but this is attributable to relatively wide CIs for all of the point estimates rather than low variability in the PRs. For all outcomes, fixed-effects and random-effects models generated nearly identical estimates.

Results were similar in the sensitivity analyses (Supplemental Figure 2A–C) that included *1*) separation of comparisons within multicomponent intervention trials, such that the SQ-LNS with no SQ-LNS comparisons were conducted separately between pairs of arms with the same nonnutrition components; *2*) exclusion of passive control arms; or *3*) exclusion of intervention arms with SQ-LNS formulations that were not milk- and peanut-based. For example, the PRs for stunting were all 0.87–0.88 and those for wasting were 0.85–0.89.

In addition, effects of SQ-LNSs were evident in both the program-based studies and the trials in which all activities were conducted by the research teams (**Supplemental Figure 4**), and also when stratified by whether the trial simply reinforced the normal IYCF messages already promoted in that setting (35, 37, 39–41, 44, 45) or the trial provided expanded SBCC for IYCF, either in the SQ-LNS intervention arms only (36, 38, 42, 47, 48) or in both the intervention and control arms (34, 43, 46) (**Supplemental Figure 5**).

### Effect modification by study-level characteristics

**Supplemental Figure 6**A–Q presents forest plots for all outcomes stratified by study-level effect modifiers. For some outcomes, we were unable to generate pooled estimates for effect modification by certain potential study-level effect modifiers because <3 comparisons were categorized into 1 of the study-level effect modification categories (e.g., acute malnutrition and low MUAC by region). Effect modification results were consistent across all sensitivity analyses (data not shown; available upon request); the results presented below refer to the all-trials analysis.

#### LAZ and stunting

**FIGURE 4:**
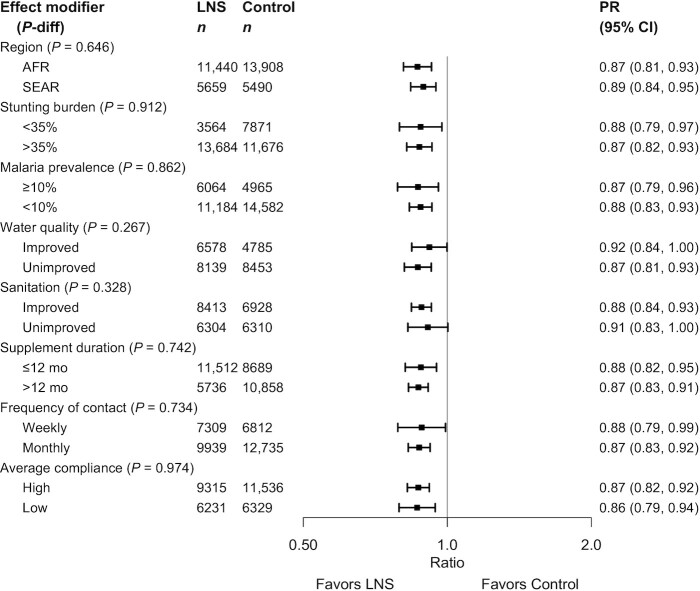
Pooled effect of small-quantity LNSs on stunting stratified by study-level characteristics. *P* value for the difference was estimated using random-effects meta-regression with the indicated effect modifier as the predictor of intervention effect size; stratified pooled estimates are presented for each stratum. AFR, African Region; LNS, lipid-based nutrient supplement; *P*-diff, *P* value for the difference in effects of small-quantity lipid-based nutrient supplements between the 2 levels of the effect modifier; PR, prevalence ratio; SEAR, South-East Asia Region.

None of the study-level characteristics significantly modified the effect of SQ-LNSs on mean LAZ (Supplemental Figure 6A) or stunting prevalence, whether expressed as a PR (Supplemental Figure 6B) or as a PD (Supplemental Figure 6C). The upper bound of the 95% CI for the PRs was ≤1 in all categories (**Figure 4**), indicating that there were significant reductions in stunting prevalence among children receiving SQ-LNSs regardless of region, stunting burden, malaria prevalence, water quality, sanitation, duration of supplementation, frequency of contact, or average reported compliance.

#### WLZ, MUACZ, wasting, low MUAC, and acute malnutrition

**FIGURE 5:**
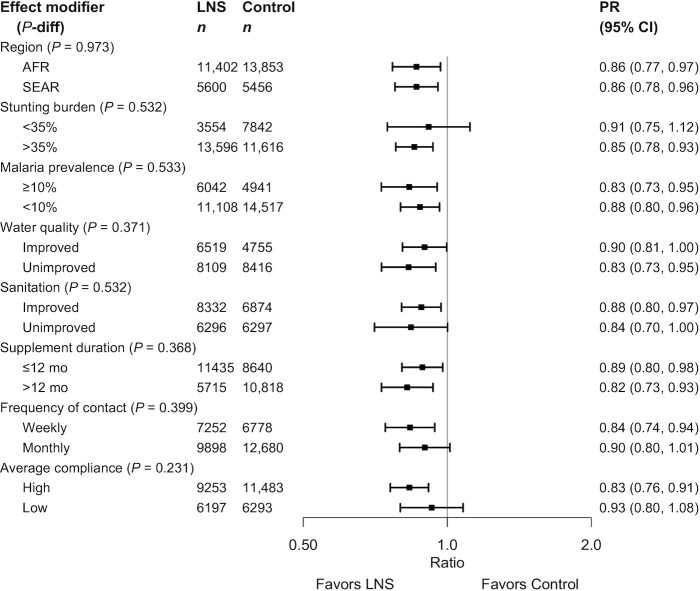
Pooled effect of small-quantity LNSs on wasting stratified by study-level effect characteristics. *P* value for the difference was estimated using random-effects meta-regression with the indicated effect modifier as the predictor of intervention effect size; stratified pooled estimates are presented for each stratum. AFR, African Region; LNS, lipid-based nutrient supplement; *P*-diff, *P* value for the difference in effects of small-quantity lipid-based nutrient supplements between the 2 levels of the effect modifier; PR, prevalence ratio; SEAR, South-East Asia Region.

None of the study-level characteristics significantly modified the effect of SQ-LNSs on mean WLZ or MUACZ, or the PRs for wasting, low MUAC, or acute malnutrition (Supplemental Figure 6D–K, **Figure 5**, **Supplemental Figure 7**). Figure 5 shows that for 5 of the 8 study-level characteristics (region, malaria prevalence, water quality, sanitation, and duration of supplementation), the upper bound of the 95% CI for wasting was ≤1 in both categories. A few of the study-level characteristics modified the effect of SQ-LNSs on the prevalence of wasting, low MUAC, or acute malnutrition when these outcomes were examined as PDs (but not PRs). The percentage point reduction in wasting associated with SQ-LNSs was greater in Bangladesh than in the other sites (Supplemental Figure 6F1) and among sites with weekly (as opposed to monthly) contact (Supplemental Figure 6F7). The percentage point reduction in low MUAC associated with SQ-LNSs was greater among sites with average reported compliance ≥80% (as opposed to <80%) (Supplemental Figure 6I8).

#### WAZ and underweight

None of the study-level characteristics significantly modified the effect of SQ-LNSs on mean WAZ or the prevalence of underweight when examined as a PR (Supplemental Figures 6L, M, 7). However, the percentage point reduction in underweight (PD) associated with SQ-LNSs was greater among sites with weekly (as opposed to monthly) contact (Supplemental Figure 6N7) and among sites with average reported compliance ≥80% (as opposed to <80%) (Supplemental Figure 6N8).

#### HCZ and small head size

Only 1 of the study-level characteristics significantly modified the effect of SQ-LNSs on HCZ: the increase in mean HCZ associated with SQ-LNSs was greater in sites with a high stunting burden (≥35%) than in sites with a stunting burden <35% (Supplemental Figure 6O2). None of the study-level characteristics significantly modified the effect of SQ-LNSs on the PR for small head size (Supplemental Figure 7), but 2 were effect modifiers for the PD: the percentage point reduction in small head size associated with SQ-LNSs was greater in sites with a malaria prevalence ≥10% than in sites with a lower malaria prevalence (Supplemental Figure 6Q3) and in sites with weekly (as opposed to monthly) contact (Supplemental Figure 6Q7).

### Effect modification by individual-level maternal, child, and household characteristics

**Supplemental Figures 8** and **9** present forest plots for all outcomes stratified by potential individual-level effect modifiers. Effect modification results were consistent across all sensitivity analyses (data not shown, available upon request), so the results presented below refer to the all-trials analysis. Results were generally similar in fixed-effects and random-effects models, although CIs were wider for the latter as expected. Results presented are for the fixed-effects models; when the effect size of effect modification differed substantially in the random-effects model, this is mentioned below.

#### LAZ and stunting

Only 1 of the individual-level characteristics modified the effect of SQ-LNSs on LAZ: the increase in mean LAZ associated with SQ-LNSs was greater among children whose mothers scored lower for depressive symptoms than among those whose mothers scored in the top quartile, although the effect was significant in both subgroups (Supplemental Figure 8A5). Several individual-level characteristics modified the effect of SQ-LNSs on stunting prevalence.

**FIGURE 6:**
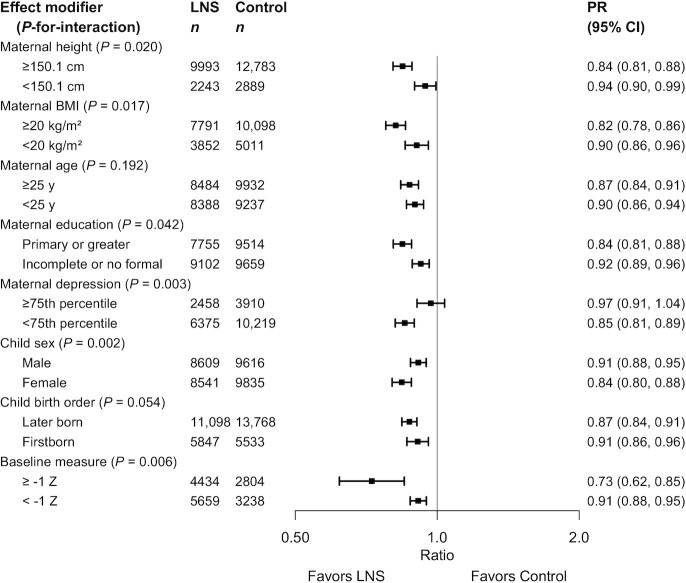
Pooled effect of small-quantity LNSs on stunting stratified by individual-level maternal and child characteristics. Individual study estimates for interaction effect were generated from log-binomial regression controlling for baseline measure when available and with clustered observations using robust SEs for cluster-randomized trials. Pooled subgroup estimates and statistical testing of the pooled interaction term were generated using inverse-variance weighting fixed effects. LNS, lipid-based nutrient supplement; *P*-interaction, *P* value for the interaction indicating the difference in effects of small-quantity lipid-based nutrient supplements between the 2 levels of the effect modifier; PR, prevalence ratio.

**FIGURE 7:**
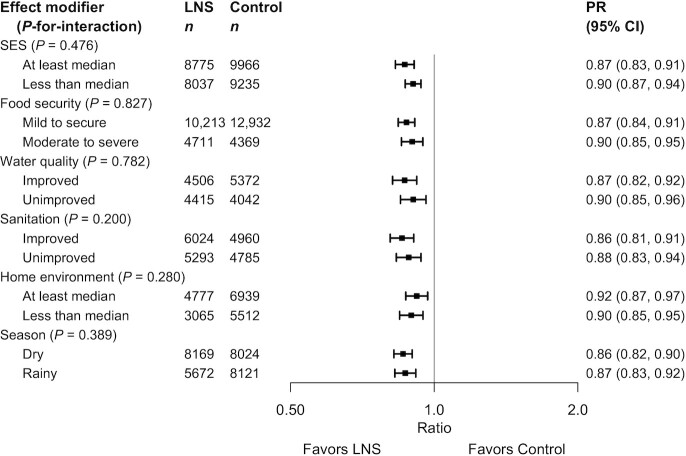
Pooled effect of small-quantity LNSs on stunting stratified by individual-level household characteristics. Individual study estimates for interaction effect were generated from log-binomial regression controlling for baseline measure when available and with clustered observations using robust SEs for cluster-randomized trials. Pooled subgroup estimates and statistical testing of the pooled interaction term were generated using inverse-variance weighting fixed effects. LNS, lipid-based nutrient supplement; *P*-interaction, *P* value for the interaction indicating the difference in effects of small-quantity lipid-based nutrient supplements between the 2 levels of the effect modifier; PR, prevalence ratio; SES, socioeconomic status.

**Figure 6** shows the PRs for stunting stratified by maternal and child characteristics; **Figure 7** shows the PRs stratified by household-level characteristics. There were significant effects in all subgroups (except among children of mothers in the top quartile for depressive symptoms). However, there was a greater effect of SQ-LNSs on stunting among children of mothers who were taller, had higher BMI, had more education, and reported fewer depressive symptoms. There was also a greater effect of SQ-LNSs on stunting among female (than among male) children and children of higher birth order (hereafter termed “later-born”) than firstborn children, although the latter difference was attenuated in the random-effects model. Baseline LAZ modified the effect of SQ-LNSs on stunting, but in opposite directions depending on whether the outcome was examined as the PR or the PD. For the former, the effect of SQ-LNSs was greater among children whose LAZ was ≥1 at baseline (27% reduction) (than among those with lower LAZ at baseline, 9% reduction), but for the latter there was a greater percentage point reduction in stunting associated with SQ-LNSs among children with lower baseline LAZ (6 percentage points) than among those with higher baseline LAZ (3 percentage points) (Supplemental Figure 8C8). None of the household-level characteristics modified the effect of SQ-LNSs on LAZ or stunting prevalence; Figure 7 shows significant reductions in stunting prevalence among children receiving SQ-LNSs regardless of socioeconomic status (SES), food security, water quality, sanitation, home environment, or season at endline.

#### WLZ, MUACZ, wasting, low MUAC, and acute malnutrition

**FIGURE 8:**
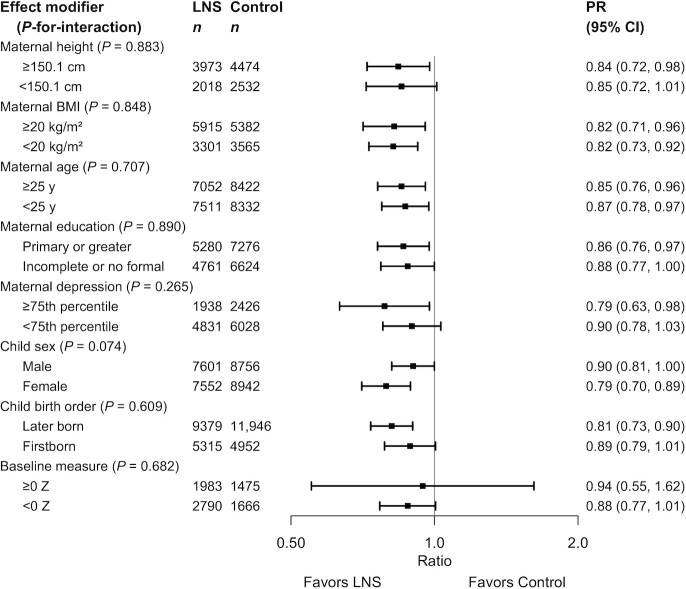
Pooled effect of small-quantity LNSs on wasting stratified by individual-level maternal and child characteristics. Individual study estimates for interaction effect were generated from log-binomial regression controlling for baseline measure when available and with clustered observations using robust SEs for cluster-randomized trials. Pooled subgroup estimates and statistical testing of the pooled interaction term were generated using inverse-variance weighting fixed effects. LNS, lipid-based nutrient supplement; *P*-interaction, *P* value for the interaction indicating the difference in effects of small-quantity lipid-based nutrient supplements between the 2 levels of the effect modifier; PR, prevalence ratio.

**FIGURE 9:**
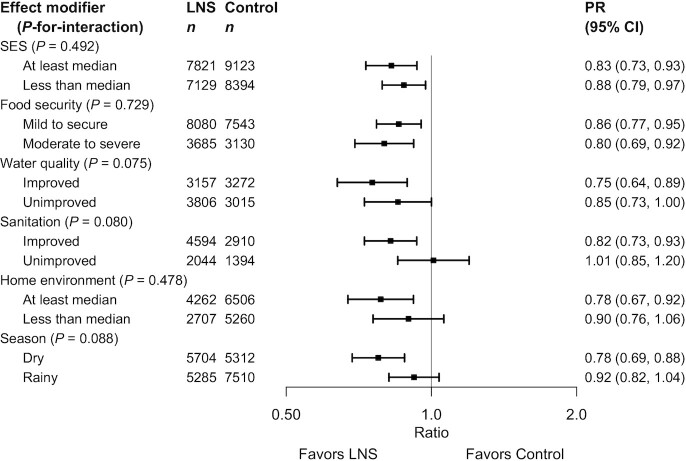
Pooled effect of small-quantity LNSs on wasting stratified by individual-level household characteristics. Individual study estimates for interaction effect were generated from log-binomial regression controlling for baseline measure when available and with clustered observations using robust SEs for cluster-randomized trials. Pooled subgroup estimates and statistical testing of the pooled interaction term were generated using inverse-variance weighting fixed effects. LNS, lipid-based nutrient supplement; *P*-interaction, *P* value for the interaction indicating the difference in effects of small-quantity lipid-based nutrient supplements between the 2 levels of the effect modifier; PR, prevalence ratio; SES, socioeconomic status.

Several individual-level characteristics modified the effects of SQ-LNSs on mean WLZ or MUACZ, or prevalence of wasting, low MUAC, or acute malnutrition. PRs for wasting, stratified by maternal and child characteristics and by household-level characteristics, are shown in **Figures 8** and **9**, respectively; all other outcomes are shown in Supplemental Figures 8 and 9. There was a greater effect of SQ-LNSs on prevalence of wasting and low MUAC among female (than among male) children. For mean WLZ and MUACZ, as well as the prevalence of wasting and acute malnutrition, the effects of SQ-LNSs were greater among children in households with improved sanitation. For wasting the effects of SQ-LNSs were also greater among children in households with improved water quality and children whose endline measurement occurred in the dry season. For mean MUACZ and the prevalence of low MUAC, the effects of SQ-LNSs were greater among later-born (than among firstborn) children; for mean WLZ and MUACZ there was a greater effect among children in households with home environment scores below the study median (as opposed to above), although these differences were attenuated in the random-effects models; and for mean MUACZ there was a greater effect among children of taller (as opposed to shorter) mothers. Lastly, there was a greater percentage point reduction in wasting and acute malnutrition associated with SQ-LNSs among children with lower baseline WLZ than among those with higher baseline WLZ, and a greater percentage point reduction in acute malnutrition among children of households with moderate to severe food insecurity (as opposed to mild food insecurity or none).

#### WAZ and underweight

There was a greater effect of SQ-LNSs on mean WAZ and prevalence of underweight among later-born (than among firstborn) children. In addition, effects of SQ-LNSs on mean WAZ were greater among children with baseline WAZ < −1 (as opposed to ≥−1), and among children in households with improved sanitation (as opposed to unimproved) or a home environment score below the median. Effects of SQ-LNSs on prevalence of underweight (PR) were greater among children whose endline measurement occurred in the dry (as opposed to the rainy) season. Lastly, there was a greater percentage point reduction in underweight associated with SQ-LNSs among children with baseline WAZ < −1 (as opposed to ≥1) and among children of households with moderate to severe food insecurity (as opposed to mild food insecurity or none) (Supplemental Figures 8, 9).

#### HCZ and small head size

None of the individual-level characteristics modified the effect of SQ-LNSs on mean HCZ. However, effects of SQ-LNSs on the prevalence of small head size were greater among female (than among male) children, those whose mothers scored in the top quartile for depressive symptoms (as opposed to lower), and those whose mothers had more education (as opposed to less). In addition, there was a greater percentage point reduction in small head size associated with SQ-LNSs among children with baseline HCZ < −1 (as opposed to ≥−1) (Supplemental Figures 8, 9).

#### Overview of individual-level effect modification

**FIGURE 10:**
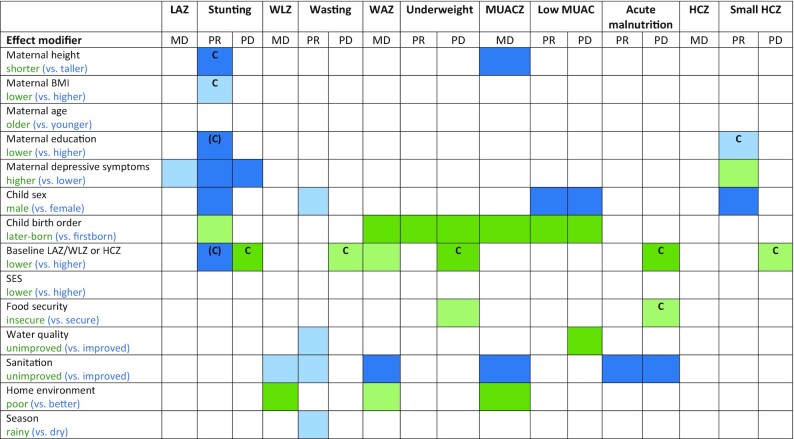
Overview of individual-level effect modification. The reference subgroup is the group expected to have the greatest potential to benefit. Green indicates a stronger effect in the reference subgroup, whereas blue indicates a stronger effect in the opposite subgroup. Box 1 provides subgroup definitions. Dark color indicates *P*-interaction < 0.05; light color indicates 0.05 < *P* < 0.1. The letter “C” indicates that the apparent effect modification is due to the cutoff effect; when “C” is in parentheses, it is partially explained by the cutoff effect. HCZ, head circumference-for-age *z* score; LAZ, length-for-age *z* score; MD, mean difference; MUAC, midupper arm circumference; MUACZ, midupper arm circumference-for-age *z* score; PD, prevalence difference; PR, prevalence ratio; SES, socioeconomic status; WAZ, weight-for-age *z* score; WLZ, weight-for-length *z* score.

**Figure 10** shows that some characteristics (e.g., child sex, birth order, household sanitation, and home environment) modified the effect of SQ-LNSs on several different growth outcomes, whereas others (e.g., maternal height, BMI, education, and age; household SES, food security, and water quality) exhibited effect modification for only 1 or 2 outcomes or none at all. Figure 10 also indicates that effect modification was more likely to be observed for binary outcomes than for continuous outcomes. When there is no significant effect modification for a continuous outcome (e.g., LAZ) but there is for the PR or PD for the corresponding binary outcome (e.g., stunting), the results could be due to the “cutoff effect,” as described in the Methods and in a companion overview article (51). Figure 10 indicates the results of the simulations to identify cutoff effects. They showed that for stunting prevalence, the cutoff effect explained the apparent effect modification by maternal stature and BMI, and some (but not all) of the apparent effect modification by maternal education. The cutoff effect also appeared to contribute, at least partially, to the apparent effect modification by baseline anthropometric status for each of the corresponding binary outcomes, and to apparent effect modification by household food security for the PD in acute malnutrition (but not underweight). For maternal depressive symptoms, however, there was significant effect modification for LAZ and both the PR and PD for stunting, so this was not due to the cutoff effect. In addition, the cutoff effect did not explain effect modification by child sex, birth order, household water quality, sanitation, home environment, or season.

## Discussion

In this IPD analysis of 14 RCTs in 9 countries, with a total sample size of >37,000 children, the relative reductions in adverse growth outcomes after provision of SQ-LNSs to infants and young children 6–24 mo of age were 12% for stunting, 14% for wasting and acute malnutrition, 18% for low MUAC, 13% for underweight, and 9% for small head size. The beneficial effects of SQ-LNSs on stunting were evident regardless of region (Africa or South Asia), stunting burden, malaria prevalence, sanitation, water quality, duration of supplementation, frequency of contact, or average reported compliance with SQ-LNS. For wasting (and other adverse growth outcomes), there was also little evidence of effect modification by study-level characteristics, although within some subgroups of trials the effect estimates did not reach statistical significance (such as sites with a lower stunting burden and trials with lower average reported compliance). Several of the individual-level characteristics appeared to modify the effects of SQ-LNSs on growth outcomes. For example, the effects of SQ-LNSs on stunting, wasting, low MUAC, and small head size were larger among girls than among boys; the effects on stunting, underweight, and low MUAC were greater among later-born children than among firstborn children; the effects on stunting were greater among children of mothers with higher (as opposed to lower) education levels as well as mothers with fewer depressive symptoms (as opposed to the top quartile); and the effects on wasting and acute malnutrition were greater among children in households with improved sanitation than among those in households with unimproved sanitation.

### Main effects

In comparison with the meta-analysis by Das et al. (16), which included 13,372 children in 9 trials for the comparison of growth outcomes between LNS (SQ- or MQ-LNS) during the period of complementary feeding and no intervention, this IPD analysis includes nearly 3 times as many participants even though we restricted the analysis to trials that provided SQ-LNSs. For all except 1 of the 14 trials in this IPD analysis, results were published in the 6-y period between 2014 and 2019. This rapid expansion in the evidence base permitted us to generate new estimates of impact on growth outcomes specific to SQ-LNSs. These new estimates for wasting and underweight were similar to those of Das et al., but the new estimate for stunting (12% reduction) was larger than previously reported (7% reduction). Moreover, we report estimates for acute malnutrition, low MUAC, and small head size, which were not included in the previous meta-analysis. Overall, the reductions in prevalence of adverse growth outcomes were modest, which is in line with previous reports for nutrition interventions (52). For example, the pooled PD for stunting was 5 percentage points. However, there was substantial heterogeneity in the effect of SQ-LNSs on stunting, with some trials indicating no impact and others reporting reductions of 10–11 percentage points (37, 48). We did not find strong evidence that this heterogeneity in impact on stunting was explained by study-level characteristics, as discussed below.

The sensitivity analyses demonstrated similar results for all outcomes regardless of inclusion/exclusion of arms with maternal plus child SQ-LNS, trials with passive control arms, or arms with nonstandard SQ-LNS formulations, as well as when analyses of multicomponent intervention trials were structured to more specifically isolate the effects of SQ-LNSs. Comparison of the all-trials analysis with the child-LNS-only analysis indicated no enhancement of effect sizes when arms or trials with maternal LNS were included. Of the 4 trials that included maternal LNSs (35, 40, 43, 44), 2 allowed for direct comparison of child-LNS only with maternal + child LNS (35, 43), both of which showed no added growth benefit of maternal LNSs. Although a meta-analysis of prenatal LNS showed that it increased mean birth weight and length and reduced the risk of small-for-gestational-age births (compared with iron and folic acid) (53), these effects may diminish over time. In Bangladesh (35), children exposed only to child LNS achieved the same mean LAZ by 24 mo as those exposed to both maternal and child LNS, despite greater LAZ at birth in the latter. In our IPD analysis, the “separation of multicomponent arms” sensitivity analysis limited comparisons to pairs of arms with the same nonnutrition components, and also excluded the maternal LNS trials/arms; results were nearly identical to those of the all-trials analysis. The consistency across sensitivity analyses indicates that the all-trials analysis, which includes a larger sample size and broader group of trials, presents a valid estimate of the causal effect.

Because the impetus for this IPD analysis was to explore effect modification, we did not report results for rare outcomes (such as severe acute malnutrition) or outcomes that were evaluated in <3 trials. An example of the latter is the incidence rate of wasting or of moderate-to-severe acute malnutrition, which warrants further exploration because incidence rate is a more complete assessment of the wasting burden than prevalence of low WLZ at a single time point. For example, cross-sectional results from the trial in Mali (46) indicated an endline prevalence of 15% for acute malnutrition in the control group, but longitudinal results indicated that more than half of control group children experienced ≥1 episode of acute malnutrition during the 18-mo follow-up period. In that trial, the SQ-LNS intervention had no effect on the prevalence of acute malnutrition in the cross-sectional sample but reduced the incidence rate of acute malnutrition in the longitudinal sample by 29%.

The objectives of this IPD analysis were focused on outcomes related to undernutrition, but there is considerable interest in whether interventions using energy-dense products such as SQ-LNSs might increase the risk of child overweight. In the trials included in this analysis, the prevalence of child overweight at endline (WLZ > 2) was 0%–4.6%, too low to calculate a pooled estimate for the PR without excluding more than half of the trials. The prevalence of WLZ > 1 was 0.6%–18%, and in only 5 of the 14 trials was the prevalence > 10% even though the prevalence in a normal healthy population should be ∼16%.

### Study-level effect modification

Study site characteristics did not appear to modify the impact of SQ-LNSs on linear growth or stunting prevalence, suggesting that those effects are not restricted to a certain region, relevant only to populations with a high burden of stunting, or limited by malaria prevalence, study-level water quality, or study-level sanitation. Study-level effect modification was also not significant for most of the other growth outcomes, but in some cases the differences in PRs were sizable (e.g., >0.05) even though the *P*-diff for interaction was not significant, presumably owing to limited statistical power for these types of analyses. For example, the relative reduction in wasting with provision of SQ-LNS was 17% in sites with unimproved water quality compared with 10% in sites with improved water quality, with similar findings for low MUAC for both water quality and sanitation. In addition, the relative reduction in wasting was 15% in sites with a higher stunting burden compared with 9% in sites with a lower stunting burden.

Aspects of study design also did not appear to modify the effects of SQ-LNSs on linear growth or stunting, even though there was wide variation in setting, duration of supplementation, frequency of contact, and average compliance with SQ-LNSs. For other growth outcomes, effect modification by study design characteristics was also generally not significant, but again there were some differences that are notable. For example, trials with average reported compliance with consumption of SQ-LNS ≥80% tended to have greater relative reductions after provision of SQ-LNSs (than trials with lower compliance) in prevalences of wasting (17% compared with 7%), low MUAC (23% compared with 14%), acute malnutrition (19% compared with 10%), and underweight (18% compared with 12%), and there was a significantly greater percentage point reduction in low MUAC and underweight in the former than in the latter. Average study compliance with SQ-LNS consumption ranged from 37% to 100%, but it should be noted that methods for assessing compliance varied widely across trials. Estimated compliance rates from self-reported and disappearance data may be overestimates when compared with observed intakes (54). In this IPD, effect modification by compliance was investigated only at the study level, because compliance at the individual level cannot be calculated for control group children who receive no supplements. Thus, further research is needed to investigate potential dose–response relations. In a recent evaluation of a program in the Democratic Republic of Congo in which SQ-LNS was distributed to infants 6–12 mo of age (55), dose-response was examined among children in the intervention zone who were 8–13 mo of age at endline, who were expected to have sufficient program exposure to expect a biological impact. Within that subsample, and adjusting for multiple potential confounding variables, children who had received ≥3 monthly distributions of SQ-LNS (*n *= 80) had significantly greater LAZ (+0.4; 95% CI: 0.02, 0.78) and a lower prevalence of stunting (−16.7 percentage points; 95% CI: −32.1, −1.2 percentage points) than those who did not receive SQ-LNSs (*n *= 89), whereas those who received 1–2 monthly distributions of SQ-LNSs (*n *= 136) did not differ from those who did not receive SQ-LNSs (LAZ: +0.08; 95% CI: −0.24, 0.41; stunting PD: −9.3; 95% CI: −22.6, 3.9).

Trials in this IPD analysis in which SQ-LNS was provided for >12 mo (as opposed to ≤12 mo) tended to demonstrate greater relative reductions in wasting (18% compared with 11%), although the *P*-diff for interaction was not significant. The meta-analysis by Das et al. (16) suggested a somewhat greater impact on stunting when the duration of LNS supplementation was >12 mo (as opposed to ≤12 mo), but we did not observe this. We also did not find significant effect modification by frequency of contact when growth results were expressed as MDs or PRs, but trials with weekly (compared with monthly) contact tended to demonstrate greater relative reductions in wasting (16% compared with 10%) and low MUAC (23% compared with 14%) after SQ-LNS provision, and the percentage point reductions in wasting, underweight, and small head size were significantly greater in the former than the latter.

We were unable to examine potential effect modification by child age at baseline because there was insufficient heterogeneity in this aspect of study design: most of the trials in this IPD analysis began supplementation at 6 mo of age. However, in the Madagascar trial, enrollment occurred across a wide age range and the investigators reported significant effects on LAZ and stunting only among children who started SQ-LNSs at 6 mo (43).

Six of the 14 trials in this IPD analysis were conducted within existing community-based or clinic-based programs (35, 38, 41, 43, 46–48), so the findings reflect impact across the spectrum from efficacy trials to effectiveness studies in a real-world context. There were no clear dividing lines between “efficacy” and “effectiveness” trials among the 14 trials because all of the program-based trials had strong, randomized study designs with active control arms, their risk of bias was generally low, and several had very high reported compliance rates. For that reason, we did not consider this dichotomy as a formal study-level effect modifier. However, there were no significant differences in effect sizes between the program-based studies and the trials in which all activities were conducted by the research teams. Similarly, we did not consider differences in SBCC for IYCF across trials as a formal study-level effect modifier. However, effects of SQ-LNSs were evident regardless of whether the trial simply reinforced the normal IYCF messages already promoted in that setting (35, 37, 39–41, 44, 45) or the trial provided expanded SBCC for IYCF, either in the SQ-LNS intervention arms only (36, 38, 42, 47, 48) or in both the intervention and control arms (34, 43, 46). In 2 of the 3 trials in the last category, which directly compared SQ-LNS + expanded SBCC for IYCF with expanded SBCC for IYCF alone (34, 46), the children provided with SQ-LNSs had improved growth compared with children in the IYCF-only arm; in the third trial (43), there was no significant overall main effect on growth even when compared with a control group without expanded SBCC for IYCF (except among children who started SQ-LNSs at 6 mo, as aforementioned).

### Individual-level effect modification

For individual-level effect modifiers, it is important to distinguish the potential to benefit from the potential to respond (9). The former is more likely when the child is more vulnerable, for example when the child is already undernourished at baseline or lives in a household with food insecurity or inadequate resources to provide adequate care. However, some children who exhibit signs of undernutrition (e.g., stunting) may actually be less likely to respond to a nutritional intervention because of other constraints on growth due to infection or inflammation, inadequate care, or other factors. Interpretation of effect modification also needs to take into account the pattern of results across continuous and binary outcomes. Significant effect modification for a continuous outcome (e.g., LAZ) is usually a clear-cut indication that the growth response to the intervention differs between subgroups. However, as explained earlier, effect modification for binary outcomes (e.g., stunting) could be due to the “cutoff effect,” depending on the distribution of the continuous outcome within each of the subgroups in relation to the cutoff. Interpretation of PRs is further complicated by the fact that the relative reduction in an adverse outcome will be greater in subgroups with a lower control group prevalence of the outcome (the denominator for the PR) than in those with a higher control group prevalence. We therefore attempt to take into account all of these considerations in the following discussion of individual-level effect modifiers.

A consistent finding was that there was a greater effect of SQ-LNSs on growth outcomes in girls than in boys. Among girls, SQ-LNSs reduced stunting by 16%, wasting by 21%, low MUAC by 27%, and small head size by 15%, whereas the corresponding reductions among boys were 9%, 10%, 7%, and 4%, respectively. The cutoff effect did not explain these differences, nor were they explained by lower control group prevalences in girls than in boys. Although the *P*-interaction for child sex and intervention group was not significant for the continuous outcomes (LAZ, WLZ, MUACZ, and HCZ), MDs were somewhat greater in girls than in boys, particularly when length, MUAC, and head circumference were examined in absolute units (cm) rather than as *z* scores. We thus conclude that there is a real difference in response to SQ-LNSs between boys and girls. Girls generally had better growth status than boys: in the control groups across the 14 trials at endline, prevalence of stunting was lower among girls in 13 trials and prevalence of wasting was lower among girls in 12, than in boys. Thus, it is unlikely that girls had greater potential to benefit. Rather, the difference probably reflects a greater potential to respond to nutritional supplementation among girls. Other studies suggest that boys are more vulnerable than girls to adverse conditions in early life, which may be driven by biological factors (56, 57) that could also constrain responses to nutrition interventions. In a previous meta-analysis, antenatal multiple micronutrient supplementation significantly reduced neonatal mortality in girls but not in boys (58), which is consistent with this hypothesis.

Among later-born children, SQ-LNSs reduced stunting by 13%, underweight by 17%, and low MUAC by 23%, whereas the corresponding reductions among firstborn children were 9%, 6%, and 5%, respectively. These results were not explained by the cutoff effect. Moreover, significant effect modification was also observed for 2 of the continuous outcomes: WAZ and MUACZ. Later-born children have ≥1 older sibling who may compete for caregiving and family resources, making them potentially more vulnerable to undernutrition and thus more likely to benefit from nutritional supplementation. Although later-born children tend to be larger at birth than firstborn children (59), prevalences of stunting and underweight at endline in the control groups for this IPD were greater among later-born children than firstborn children in 8 of the 14 trials. This suggests that there may be greater potential to benefit among later-born children in some settings.

We observed greater effects of SQ-LNSs on stunting among children of taller (as opposed to shorter) mothers (16% compared with 6% relative reduction). This could be due to greater constraints on a linear growth response among children of shorter mothers due to genetic, intrauterine, or environmental factors (60). Similarly, there were greater effects of SQ-LNSs on stunting among children of mothers with higher (as opposed to lower) BMI (18% compared with 11% relative reduction), which could be related to a higher risk of fetal growth restriction and persistent postnatal constraints on growth among children of thinner mothers (6). However, for both of these examples, there was no significant effect modification for the continuous outcome, the MD in LAZ, and the cutoff effect was the most likely explanation for the findings. The cutoff effect also appeared to explain the greater effects of SQ-LNSs on prevalence of small head size among children of women with higher levels of education (as opposed to less), but it did not fully explain effect modification by maternal education for stunting. Although the effects of SQ-LNSs on LAZ did not differ between children of women with higher and lower educational levels, the relative reduction in stunting was 16% and 8%, respectively. This may reflect a greater potential to respond to nutrient supplementation among children of mothers who are better educated; those mothers may have greater autonomy and agency, and be better able to adhere to advice regarding recommended frequency and dosage of supplementation.

Information on maternal depressive symptoms was not collected in all of the trials in this IPD analysis, but among those with such data, there was evidence of effect modification for 2 different growth outcomes in opposite directions. The effects of SQ-LNSs on LAZ and stunting (both PR and PD) were greater among children of mothers with lower (as opposed to higher) scores for depressive symptoms, whereas the effects of SQ-LNSs on the prevalence of small head size (but not mean HCZ) were greater among children of mothers in the top quartile for depressive symptoms (as opposed to those with lower scores). The former was not explained by the cutoff effect, and may reflect greater potential to respond to a nutritional supplement among children of less depressed mothers (who may have more capability to effectively use nutritional supplements in ways that improve the health of the child). Among children in the control groups in this IPD, those whose mothers had lower scores for depressive symptoms had slightly higher mean LAZ and HCZ than those whose mothers were in the top quartile for depressive symptoms (a difference of ∼ −0.10 Z), suggesting that the former subgroup was less vulnerable, which is inconsistent with a greater potential to benefit. However, in the SHINE trial in Zimbabwe (91), the effect of the intervention on LAZ was greater among children of mothers who scored ≥12 on the Edinburgh Postnatal Depression Scale [a cutoff validated against clinically diagnosed major depression in Zimbabwean women (61)], such that the difference in mean LAZ between children of depressed and nondepressed mothers observed in the control group was not evident in the intervention group. This may reflect amelioration by SQ-LNSs of the adverse influence of maternal depression on growth. A similar phenomenon might underlie the IPD analysis findings for small head size, but those results could also be spurious. Differences in the way maternal depression affects child caregiving across cultures or in the methods used to assess and define depression across studies may contribute to variation in results. Additional research to understand these relations would be useful.

We observed greater effects of SQ-LNSs on the percentage point reduction in acute malnutrition and underweight among children in households with moderate to severe food insecurity than among those in households with less food insecurity, although the results for acute malnutrition may be due to the cutoff effect (i.e., the effect of SQ-LNSs on mean WLZ was similar regardless of household food insecurity). For underweight, SQ-LNSs may have been more important for helping to fill gaps in energy and micronutrient intakes for children in food-insecure households than for children in households with greater food security. The former may have had greater potential to benefit, given that the prevalence of underweight in this IPD analysis was higher among control group children in food-insecure households (by 8 percentage points) than among those in households with greater food security. Similarly, there were greater effects of SQ-LNSs on WLZ, WAZ, and MUACZ among children in households with home environment scores below the study median (as opposed to above). Among children in the control groups in this analysis, mean WLZ, WAZ, and MUACZ were lower (by ∼0.11–0.18 Z) among those with lower (as opposed to higher) home environment scores in 7 of the 8 trials that included home environment information, suggesting greater potential to benefit.

By contrast, greater effects of SQ-LNSs on WLZ, MUACZ, wasting, and acute malnutrition were seen among children in households with improved (as opposed to unimproved) sanitation, and for wasting the effects were also greater among children in households with improved (as opposed to unimproved) water quality. These findings likely reflect a greater potential to respond. Better sanitation and water quality may reduce the constraints on growth that are linked to clinical and subclinical gastrointestinal disorders and inflammation (4, 5). However, these findings differ somewhat from what was seen in the 3 trials that included household-level WASH interventions, which demonstrated that there was no added benefit of providing WASH interventions together with SQ-LNS as compared with providing SQ-LNS alone on wasting (36, 42, 47, 48). In this IPD analysis, greater effects of SQ-LNS on wasting and underweight were also seen when outcome measurements occurred during the dry (as opposed to the rainy) season, which is consistent with the findings for household sanitation given that bacterial pathogens that cause diarrhea may be less prevalent during the dry season (62). There may also be more time for child care in farming households during the dry season. However, household sanitation and season did not significantly modify the effects of SQ-LNSs on stunting, which is consistent with the results of the 3 aforementioned trials. It is likely that large improvements in sanitation and water quality at the community level, not just at the individual household level, are needed before a positive synergy between WASH and nutrition interventions is observed with respect to reductions in stunting (63–65).

The effect of SQ-LNSs on risk of stunting (PR) was greater among children whose baseline LAZ was higher (≥−1) than among those with baseline LAZ < −1 (27% compared with 9% reduction). However, the opposite was true when examining the PD in stunting: the percentage point reduction associated with SQ-LNSs among children with higher baseline LAZ was less than the reduction among those with lower baseline LAZ (3 compared with 6 percentage points). This is because the overall prevalence of stunting at endline was lower among children with a higher baseline LAZ, and thus the PR calculation yielded a greater relative reduction in stunting than was the case for children with a lower baseline LAZ.

### Strengths and limitations

Strengths of these analyses include the large sample size, the substantial number of high-quality RCTs available, and the high participation rate among the investigators invited to contribute data. In addition, the 14 study sites were highly diverse in terms of geographic location, stunting burden, malaria prevalence, water quality, sanitation, and several aspects of study design, which provided heterogeneity for exploration of study-level potential effect modifiers. We presented results in terms of MDs for continuous outcomes, as well as both PRs and PDs for binary outcomes; triangulating the findings across these different estimates of impact aided in interpretation. For example, the absolute PDs are particularly important for understanding potential public health impact (66). In general, the results were similar regardless of whether fixed-effects or random-effects models were used. The consistency in the results of the sensitivity analyses also strengthens the conclusions.

These analyses have a few limitations. Bangladesh was the only country represented in the Southeast Asia Region, and Haiti was the only country represented in Latin America and the Caribbean. Thus, more data from countries outside of Africa are needed. Data were unavailable from some of the trials for certain outcomes (MUACZ for 3 trials and HCZ for 4 trials) and for several individual-level potential effect modifiers (particularly maternal depressive symptoms and home environment), so <14 trials were represented in those analyses. In addition, fewer trials were represented in the analyses for outcomes with relatively low prevalences (e.g., wasting and acute malnutrition) because effect estimates could not be generated, especially when the number of trials was further restricted by low proportions of children within 1 of the effect modifier subgroups in some trials. Overall, statistical power for study-level effect modification was constrained by the limited number of trials, so there may be meaningful differences in effect estimates between categories of trials even if the *P*-diff for the association between the effect modifier and effect size was not significant. On the other hand, the individual-level effect modification analyses involved multiple effect modifiers and numerous outcomes, so several of the significant *P*-interaction values are likely due to chance. As stated in the Methods, we did not adjust for multiple hypothesis testing because the outcome variables are correlated and effect modification analyses are inherently exploratory. Although we made every effort to standardize definitions and cutoffs for potential effect modifiers, there was variation in the methods used in the field to collect information on certain characteristics, such as household food insecurity and SES. Lastly, caution is needed when interpreting the effect modification results because many of the potential effect modifiers are interrelated and also may be confounded by unmeasured variables. This is particularly important for the study-level characteristics, because there is substantial overlap in terms of which trials fall into each category for certain potential effect modifiers (e.g., the categorization of water quality and sanitation as “improved” or “unimproved” was the same within a trial for all but 3 of the trials). Thus, attribution of the relative potential to benefit or respond to SQ-LNS to a particular characteristic may not be warranted.

### Programmatic implications

These results suggest that policy-makers and program planners should consider including SQ-LNSs in the mix of interventions to prevent both stunting and wasting. The overall effects on stunting (12% reduction) and wasting (14% reduction) may seem modest, but they are more substantial and more consistent than has been observed for other nutrition interventions for children <2 y of age. Educational interventions to improve complementary feeding have shown positive effects on feeding practices; however, there is insufficient evidence to demonstrate an impact of education alone on growth outcomes (67). Micronutrient supplementation, MNPs, and food fortification are effective for reducing anemia, but in a recent comparison of 5 different types of interventions for children <5 y old, growth was improved only among children provided with LNSs (52). Similarly, fortified cereals and milks for young children have shown little to no effect on growth outcomes, particularly linear growth (68–70). Provision of animal-source foods such as eggs is a promising strategy, but the evidence is too limited to draw conclusions regarding an impact on growth (71), and cost issues need to be considered. The cost of SQ-LNS is estimated at $0.07–0.14/d (not including distribution costs, which can be substantial), depending on scale and location of production (72, 73). A full discussion of cost issues is beyond the scope of this article, but information on costs and willingness-to-pay is available elsewhere (72–75).

It is reassuring that no adverse effects of SQ-LNSs on breast milk intake or infant feeding practices have been observed in any of the trials that reported on these outcomes, and in some settings there have been positive effects on feeding frequency and consumption of animal-source foods (76–80). However, SQ-LNS is not a stand-alone intervention and should always be accompanied by messaging to reinforce recommended IYCF practices in addition to appropriate use of the product. No adverse effects of SQ-LNSs on child fatness or high WLZ or BMI have been observed, either at the end of the intervention period (81) or in longer-term follow-up studies (82, 83). One of the Bangladesh trials included in this IPD analysis showed that the LNS groups had greater increases in fat-free mass than in fat mass (81), which is consistent with improved linear growth and no increase in the risk of excess adiposity.

As demonstrated by the effect modification results herein, effects of SQ-LNSs on stunting and wasting appear to be greater in girls (than in boys) and later-born (than in firstborn) children. However, significant effects were also seen in boys and in firstborn children, so we do not suggest targeting SQ-LNSs to these subgroups, which would in any case be ethically and logistically challenging. However, the greater impact of SQ-LNSs on growth of girls could be viewed as a positive finding with regard to the potential to reduce intergenerational stunting, if the increased height in early life persists later in life. Follow-up studies of 2 of the trials included in these analyses (35, 40) examined whether height differed between intervention groups at preschool age (3–6 y). In Bangladesh, the difference in height-for-age *z* score between SQ-LNS and comparison groups was significant among female preschoolers (+0.09), although not among males; in households with moderate to severe food insecurity at baseline, stunting prevalence at 3–6 y of age was lower in the SQ-LNS group, by ∼6 percentage points in the total sample and ∼9 percentage points among females (83). In Ghana, height at 4–6 y of age did not differ significantly between intervention groups in the total sample, but there was a difference of +1.1 cm (95% CI: 0.2, 2.1 cm) in the SQ-LNS group (relative to the comparison groups) among children of women who were not overweight at baseline (82). Additional follow-up of other cohorts, and at older ages, is needed to determine if there is long-term persistence of growth differences among children who received SQ-LNSs in early life.

The effect modification results regarding potential to benefit generally did not provide a strong rationale for targeting SQ-LNSs only to the neediest, because growth benefits were generally similar regardless of study-level characteristics or household-level SES. However, some of the results suggested that a greater impact of SQ-LNSs may be obtained by combining supplementation with interventions that address factors related to potential to respond. A growth response to nutritional supplementation may be constrained by infection, fetal growth restriction, or suboptimal caregiving (3). Hence, integrated programs that combine SQ-LNSs with interventions to prevent and control pre- and postnatal infection and inflammation, optimize fetal growth via improved maternal nutrition and other strategies, and support care for women and children (including maternal mental health promotion and education regarding optimal IYCF practices) should be further evaluated (13). Reducing constraints on a linear growth response may facilitate a larger reduction in stunting than observed in the pooled results of these analyses. Integrated programs that encompass an even broader set of interventions within food systems should also be a high priority. For example, the CHANGE project in Burkina Faso was designed to tackle multiple factors affecting child undernutrition by providing, in a staged process, *1*) inputs for home gardening and poultry production, with a focus on gender equity; *2*) education and training on agriculture, health, hygiene, and nutrition; *3*) WASH interventions; and *4*) SQ-LNS for children 6–24 mo of age. Earlier phases of the program (without SQ-LNS) resulted in improvements in some outcomes, such as anemia, but stunting prevalence was reduced only in the group receiving all components, including SQ-LNS (by 7.7 percentage points) (84).

To our knowledge, SQ-LNS is the only nutrition intervention for children that has been documented in meta-analyses to have positive effects not only on child growth, but also on iron deficiency and anemia (17), child development (18), and child mortality (85). SQ-LNSs can fill key nutrient gaps and reduce the cost of a nutritionally adequate diet (13), thereby potentially mitigating the adverse impact of rising food insecurity on vulnerable children (86). Although SQ-LNS is not a substitute for a diverse diet that includes healthy foods from each of the major food groups, it can play a protective role when access to certain foods (e.g., animal-source foods) is limited owing to cost or other circumstances. A critical next step that we plan to undertake is a set of formal cost-effectiveness and cost:benefit analyses that take into account the multiple outcomes that may be influenced by SQ-LNSs, similar to the recent cost:benefit analyses of MNPs (87). Also needed are additional rigorously designed evaluations of large-scale programs that include SQ-LNSs, particularly in low- and middle-income countries that are considering scaling up this intervention.

## Supplementary Material

nqab278_Supplemental_Files

## Data Availability

Data described in the article, code book, and analytic code will not be made available because they are compiled from 14 different trials, and access is under the control of the investigators of each of those trials.

## Notes

Supported by Bill & Melinda Gates Foundation grant OPP49817 (to KGD).

PC is a member of the Journal's Editorial Board.

Supplemental Methods, Supplemental Figures 1–9, and Supplemental Tables 1–5 are available from the “Supplementary data” link in the online posting of the article and from the same link in the online table of contents at https://academic.oup.com/ajcn/.

Published in a supplement to *The American Journal of Clinical Nutrition*. Publication costs for this supplement were defrayed in part by the payment of page charges. The Guest Editor for this supplement was Chris Sudfeld who reports no disclosures. The Supplement Coordinator for the supplement publication was Kathryn Dewey, University of California, Davis, Davis, CA. Kathryn Dewey has received funding from the Bill & Melinda Gates Foundation and the US Agency for International Development (via the Food and Nutrition Technical Assistance Project) for research on lipid-based nutrient supplements. The opinions expressed in this publication are those of the authors and are not attributable to the sponsors or the publisher, Editor, or Editorial Board of *The American Journal of Clinical Nutrition*.

Abbreviations used: GRADE, Grading of Recommendations Assessment, Development and Evaluation; HCZ, head circumference-for-age *z* score; IPD, individual participant data; IYCF, infant and young child feeding; LAZ, length-for-age *z* score; LNS, lipid-based nutrient supplement; MD, mean difference; MNP, multiple micronutrient powder; MQ, medium-quantity; MUAC, midupper arm circumference; MUACZ, midupper arm circumference-for-age *z* score; PD, prevalence difference; PR, prevalence ratio; RCT, randomized controlled trial; SBCC, social and behavior change communication; SES, socioeconomic status; SQ, small-quantity; WASH, water, sanitation, and hygiene; WAZ, weight-for-age *z* score; WLZ, weight-for-length *z* score.
