# Supplementary material for: Characteristics that modify the effect of small-quantity lipid-based nutrient supplementation on child growth: an individual participant data meta-analysis of randomized controlled trials
Source: Am J Clin Nutr. 2021 Sep 29;114(Suppl 1):15S–42S. doi: 10.1093/ajcn/nqab278 (PMC8560308; doi:10.1093/ajcn/nqab278)

Supplemental figure 9: Forest plots for effects of SQ-LNS on growth outcomes stratified by individual-level household effect modifiers

Contents

Supplemental figure 9A: Mean difference in LAZ 4
9A1: Stratified by Household socio-economic status . . . 4
9A2: Stratified by Household food insecurity . . . 5
9A3: Stratified by Household source water quality . . . 6
9A4: Stratified by Household sanitation . . . 7
9A5: Stratified by Home environment . . . 8
9A6: Stratified by Season at the time of assessment . . . 9

Supplemental figure 9B: Stunting prevalence ratio 10
9B1: Stratified by Household socio-economic status . . . 10
9B2: Stratified by Household food insecurity . . . 11
9B3: Stratified by Household source water quality . . . 12
9B4: Stratified by Household sanitation . . . 13
9B5: Stratified by Home environment . . . 14
9B6: Stratified by Season at the time of assessment . . . 15

Supplemental figure 9C: Stunting prevalence difference 16
9C1: Stratified by Household socio-economic status . . . 16
9C2: Stratified by Household food insecurity . . . 17
9C3: Stratified by Household source water quality . . . 18
9C4: Stratified by Household sanitation . . . 19
9C5: Stratified by Home environment . . . 20
9C6: Stratified by Season at the time of assessment . . . 21

Supplemental figure 9D: Mean difference in WLZ 22
9D1: Stratified by Household socio-economic status . . . 22
9D2: Stratified by Household food insecurity . . . 23
9D3: Stratified by Household source water quality . . . 24
9D4: Stratified by Household sanitation . . . 25
9D5: Stratified by Home environment . . . 26
9D6: Stratified by Season at the time of assessment . . . 27

Supplemental figure 9E: Wasting prevalence ratio 28
9E1: Stratified by Household socio-economic status . . . 28
9E2: Stratified by Household food insecurity . . . 29
9E3: Stratified by Household source water quality . . . 30
9E4: Stratified by Household sanitation . . . 31
9E5: Stratified by Home environment . . . 32
9E6: Stratified by Season at the time of assessment . . . 33

Supplemental figure 9F: Wasting prevalence difference 34

9F1: Stratified by Household socio-economic status . . . 34
9F2: Stratified by Household food insecurity . . . 35
9F3: Stratified by Household source water quality . . . 36
9F4: Stratified by Household sanitation . . . 37
9F5: Stratified by Home environment . . . 38
9F6: Stratified by Season at the time of assessment . . . 39

Supplemental figure 9G: Mean difference in MUACZ **40**
9G1: Stratified by Household socio-economic status . . . 40
9G2: Stratified by Household food insecurity . . . 41
9G3: Stratified by Household source water quality . . . 42
9G4: Stratified by Household sanitation . . . 43
9G5: Stratified by Home environment . . . 44
9G6: Stratified by Season at the time of assessment . . . 45

Supplemental figure 9H: Low MUAC prevalence ratio **46**
9H1: Stratified by Household socio-economic status . . . 46
9H2: Stratified by Household food insecurity . . . 47
9H3: Stratified by Household source water quality . . . 48
9H4: Stratified by Household sanitation . . . 49
9H5: Stratified by Home environment . . . 50
9H6: Stratified by Season at the time of assessment . . . 51

Supplemental figure 9I: Low MUAC prevalence difference **52**
9I1: Stratified by Household socio-economic status . . . 52
9I2: Stratified by Household food insecurity . . . 53
9I3: Stratified by Household source water quality . . . 54
9I4: Stratified by Household sanitation . . . 55
9I5: Stratified by Home environment . . . 56
9I6: Stratified by Season at the time of assessment . . . 57

Supplemental figure 9J: Acute malnutrition prevalence ratio **58**
9J1: Stratified by Household socio-economic status . . . 58
9J2: Stratified by Household food insecurity . . . 59
9J3: Stratified by Household source water quality . . . 60
9J4: Stratified by Household sanitation . . . 61
9J5: Stratified by Home environment . . . 62
9J6: Stratified by Season at the time of assessment . . . 63

Supplemental figure 9K: Acute malnutrition prevalence difference **64**
9K1: Stratified by Household socio-economic status . . . 64
9K2: Stratified by Household food insecurity . . . 65
9K3: Stratified by Household source water quality . . . 66
9K4: Stratified by Household sanitation . . . 67
9K5: Stratified by Home environment . . . 68
9K6: Stratified by Season at the time of assessment . . . 69

Supplemental figure 9L: Mean difference in WAZ **70**
9L1: Stratified by Household socio-economic status . . . 70
9L2: Stratified by Household food insecurity . . . 71
9L3: Stratified by Household source water quality . . . 72

9L4: Stratified by Household sanitation . . . 73
9L5: Stratified by Home environment . . . 74
9L6: Stratified by Season at the time of assessment . . . 75

Supplemental figure 9M: Underweight prevalence ratio **76**
9M1: Stratified by Household socio-economic status . . . 76
9M2: Stratified by Household food insecurity . . . 77
9M3: Stratified by Household source water quality . . . 78
9M4: Stratified by Household sanitation . . . 79
9M5: Stratified by Home environment . . . 80
9M6: Stratified by Season at the time of assessment . . . 81

Supplemental figure 9N: Underweight prevalence difference **82**
9N1: Stratified by Household socio-economic status . . . 82
9N2: Stratified by Household food insecurity . . . 83
9N3: Stratified by Household source water quality . . . 84
9N4: Stratified by Household sanitation . . . 85
9N5: Stratified by Home environment . . . 86
9N6: Stratified by Season at the time of assessment . . . 87

Supplemental figure 9O: Mean difference in HCZ **88**
9O1: Stratified by Household socio-economic status . . . 88
9O2: Stratified by Household food insecurity . . . 89
9O3: Stratified by Household source water quality . . . 90
9O4: Stratified by Household sanitation . . . 91
9O5: Stratified by Home environment . . . 92
9O6: Stratified by Season at the time of assessment . . . 93

Supplemental figure 9P: Small head size prevalence ratio **94**
9P1: Stratified by Household socio-economic status . . . 94
9P2: Stratified by Household food insecurity . . . 95
9P3: Stratified by Household source water quality . . . 96
9P4: Stratified by Household sanitation . . . 97
9P5: Stratified by Home environment . . . 98
9P6: Stratified by Season at the time of assessment . . . 99

Supplemental figure 9Q: Small head size prevalence difference **100**
9Q1: Stratified by Household socio-economic status . . . 100
9Q2: Stratified by Household food insecurity . . . 101
9Q3: Stratified by Household source water quality . . . 102
9Q4: Stratified by Household sanitation . . . 103
9Q5: Stratified by Home environment . . . 104
9Q6: Stratified by Season at the time of assessment . . . 105

These figures are forest plots showing the individual-level effect modification of intervention effects. Each figure has the estimates of intervention effect stratified within study by individual-level effect modifier category. For definitions of effect modifiers, see Box 1 in the main paper. Individual study estimates were generated from log-binomial regression for dichotomous outcomes and simple linear regression for continuous outcomes; controlling for baseline measure when available and with clustered observations using robust standard errors for cluster-randomized trials. Pooled interaction term and sub-group estimates were generated using inverse-variance weighting fixed and random effects. For continuous outcomes analyzed via mean differences, the effect estimate is the mean in the LNS group minus the mean in the control group. For dichotomous outcomes analyzed via prevalence ratios, the effect estimate is the prevalence in the LNS group divided by the prevalence in the control group. For dichotomous outcomes analyzed via prevalence differences, the effect estimate is the prevalence in the LNS group minus the prevalence in the control group.
The labels on the far left correspond to trial level information. In the middle left and on the right the values indicate the study level effect estimate, confidence interval, and weighting for deriving the pooled estimates is shown by subgroup. LAZ, length-for-age z-score; WLZ, weight-for-length z-score; WAZ, weight-for-age z-score; MUACZ, mid-upper arm circumference z-score; HCZ, head circumferencefor-age z-score.

Supplemental figure 9A: Mean difference in LAZ

9A1: Stratified by Household socio-economic status

P−for−interaction = 0.805
Difference in MDs = 0.01 (−0.03, 0.04)

		At least median						Less than median					
**Country**	**Trial**	**LNS N**	**Control N**	**Control Mean**	**MD (95% CI)**	**Fixed W**	**Random W**	**LNS N**	**Control N**	**Control Mean**	**MD (95% CI)**	**Fixed W**	**Random W**
Bangladesh	JiVitA−4 (34)	1429	621	−1.74	0.08 (0.02, 0.15)	0.23	0.10	1408	623	−2.07	0.09 (0.02, 0.15)	0.22	0.11
Bangladesh	RDNS (35)	834	407	−1.69	0.11 (0.00, 0.22)	0.08	0.08	829	408	−1.93	0.04 (−0.05, 0.13)	0.11	0.09
Bangladesh	WASH−B (36)	579	1716	−1.60	0.19 (0.09, 0.30)	0.08	0.08	579	1715	−2.03	0.23 (0.14, 0.32)	0.13	0.09
Burkina Faso	iLiNS−Zinc (37)	1225	299	−1.77	0.28 (0.18, 0.37)	0.10	0.09	720	364	−1.74	0.33 (0.23, 0.43)	0.10	0.09
Burkina Faso	PROMIS (38)	407	482	−1.50	0.22 (0.03, 0.41)	0.03	0.05	456	432	−1.47	0.08 (−0.06, 0.22)	0.05	0.06
Burkina Faso	PROMIS CS (38)	220	210	−1.15	−0.04 (−0.29, 0.20)	0.02	0.03	210	229	−1.29	0.12 (−0.13, 0.36)	0.02	0.03
Ghana	GHANA (39)	57	44	−0.18	0.21 (−0.21, 0.63)	0.01	0.01	41	46	−0.51	0.16 (−0.28, 0.60)	0.00	0.01
Ghana	iLiNS−DYADG (40)	157	356	−0.76	0.08 (−0.11, 0.26)	0.03	0.05	190	334	−1.03	0.33 (0.14, 0.51)	0.03	0.05
Haiti	HAITI (41)	75	60	−0.68	−0.04 (−0.23, 0.16)	0.02	0.04	61	72	−0.90	0.04 (−0.15, 0.24)	0.03	0.04
Kenya	WASH−B (42)	825	2921	−1.47	0.20 (0.12, 0.28)	0.15	0.09	630	2212	−1.70	0.09 (−0.02, 0.20)	0.08	0.08
Madagascar	MAHAY (43)	771	884	−2.22	−0.04 (−0.22, 0.13)	0.03	0.05	878	755	−2.26	0.09 (−0.06, 0.24)	0.04	0.06
Malawi	iLiNS−DYADM (44)	121	234	−1.49	−0.05 (−0.28, 0.18)	0.02	0.04	99	209	−1.77	−0.14 (−0.39, 0.12)	0.01	0.03
Malawi	iLiNS−DOSE (45)	301	104	−1.72	−0.01 (−0.16, 0.15)	0.04	0.06	294	103	−2.14	0.07 (−0.09, 0.23)	0.04	0.06
Mali	PROMIS (46)	247	251	−1.72	0.22 (0.07, 0.37)	0.04	0.06	246	246	−1.74	0.12 (−0.09, 0.34)	0.02	0.04
Mali	PROMIS CS (46)	505	463	−1.57	0.26 (0.09, 0.44)	0.03	0.05	447	506	−1.57	0.18 (−0.05, 0.41)	0.02	0.04
Zimbabwe	SHINE (HIV−) (47)	908	802	−1.52	0.15 (0.05, 0.26)	0.08	0.08	832	860	−1.70	0.18 (0.08, 0.28)	0.09	0.09
Zimbabwe	SHINE (HIV+) (48)	171	156	−1.93	0.23 (−0.02, 0.48)	0.01	0.03	158	167	−2.06	0.25 (−0.02, 0.53)	0.01	0.03
		**8832**	**10010**		**I² = 0.55, Tau² = 0.01**			**8078**	**9281**		**I² = 0.58, Tau² = 0.01**		
**Fixed**					**0.14 (0.11, 0.17)**						**0.14 (0.11, 0.17)**		
**Random**					**0.13 (0.08, 0.18)**						**0.14 (0.09, 0.19)**		

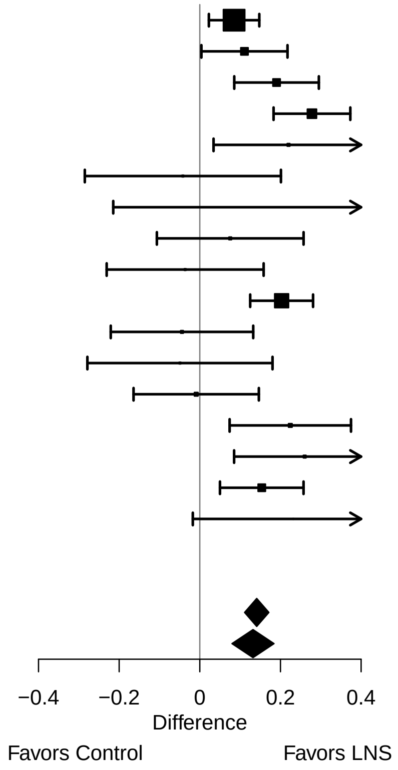

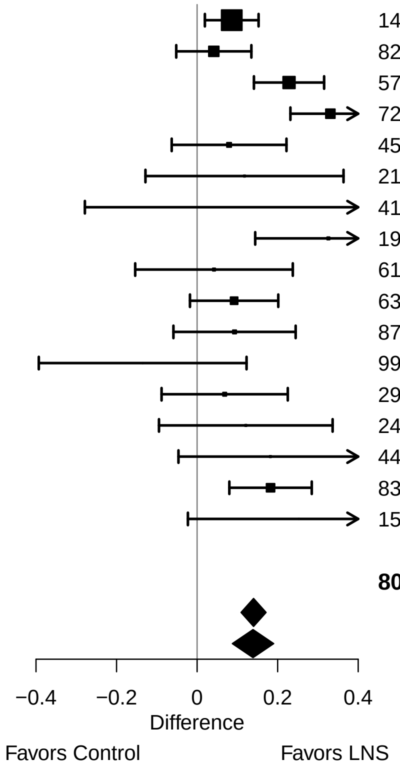

Supplemental figure 9A: Mean difference in LAZ

9A2: Stratified by Household food insecurity

P−for−interaction = 0.943
Difference in MDs = 0.00 (−0.04, 0.05)

Country	Trial	Mild to secure: LNS N	Control N	Control Mean	MD (95% CI)	Fixed W	Random W	Moderate to severe: LNS N	Control N	Control Mean	MD (95% CI)	Fixed W	Random W
Bangladesh	JiVitA−4 (34)	2012	863	−1.82	0.09 (0.03, 0.14)	0.24	0.11	825	381	−2.09	0.08 (−0.01, 0.16)	0.23	0.13
Bangladesh	RDNS (35)	1058	493	−1.71	0.03 (−0.07, 0.13)	0.08	0.09	605	322	−1.96	0.14 (0.02, 0.25)	0.13	0.11
Bangladesh	WASH−B (36)	918	2652	−1.76	0.23 (0.16, 0.31)	0.13	0.10	240	779	−2.01	0.09 (−0.05, 0.23)	0.09	0.09
Burkina Faso	iLiNS−Zinc (37)	1013	320	−1.77	0.34 (0.25, 0.44)	0.09	0.09	932	343	−1.74	0.27 (0.17, 0.37)	0.16	0.12
Burkina Faso	PROMIS (38)	502	550	−1.43	0.11 (−0.03, 0.25)	0.04	0.07	361	364	−1.56	0.21 (0.02, 0.39)	0.05	0.07
Burkina Faso	PROMIS CS (38)												
Ghana	GHANA (39)												
Ghana	iLiNS−DYADG (40)	244	470	−0.87	0.27 (0.12, 0.42)	0.03	0.07	102	219	−0.95	0.04 (−0.20, 0.28)	0.03	0.05
Haiti	HAITI (41)												
Kenya	WASH−B (42)	1299	4595	−1.55	0.15 (0.09, 0.22)	0.19	0.11	156	525	−1.75	0.17 (−0.05, 0.40)	0.03	0.05
Madagascar	MAHAY (43)	988	927	−2.25	0.02 (−0.15, 0.18)	0.03	0.06	355	410	−2.24	0.04 (−0.17, 0.25)	0.04	0.06
Malawi	iLiNS−DYADM (44)	70	122	−1.45	−0.04 (−0.37, 0.29)	0.01	0.03	148	316	−1.68	−0.12 (−0.32, 0.08)	0.04	0.06
Malawi	iLiNS−DOSE (45)	151	55	−1.73	−0.03 (−0.22, 0.17)	0.02	0.05	431	146	−2.03	0.05 (−0.08, 0.19)	0.09	0.10
Mali	PROMIS (46)	329	341	−1.69	0.13 (−0.03, 0.29)	0.03	0.06	174	163	−1.81	0.24 (0.06, 0.43)	0.05	0.07
Mali	PROMIS CS (46)												
Zimbabwe	SHINE (HIV−) (47)	1393	1305	−1.59	0.15 (0.06, 0.23)	0.11	0.10	296	325	−1.74	0.28 (0.10, 0.45)	0.06	0.07
Zimbabwe	SHINE (HIV+) (48)	236	239	−1.96	0.25 (0.05, 0.46)	0.02	0.05	86	76	−2.17	0.27 (−0.10, 0.64)	0.01	0.02
		**10213**	**12932**		**I² = 0.72, Tau² = 0.01**			**4711**	**4369**		**I² = 0.47, Tau² = 0.01**		
**Fixed**					**0.15 (0.12, 0.18)**						**0.13 (0.09, 0.18)**		
**Random**					**0.14 (0.08, 0.20)**						**0.13 (0.07, 0.19)**		

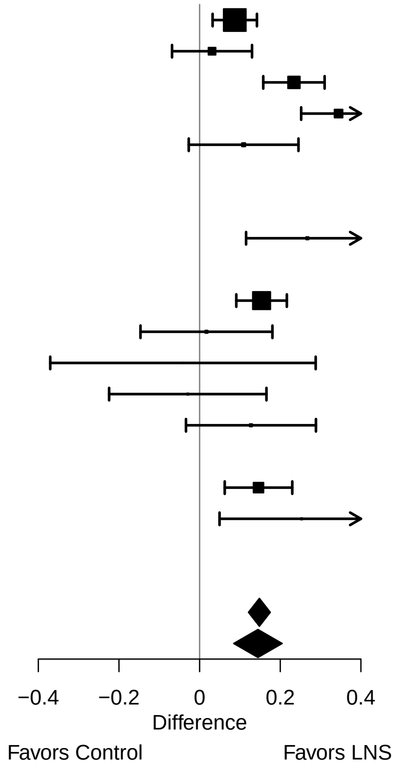

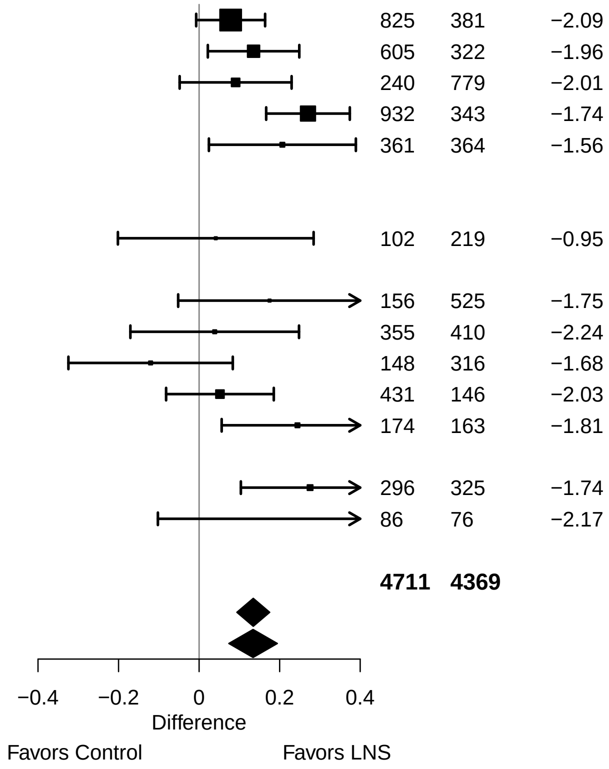

Supplemental figure 9A: Mean difference in LAZ

9A3: Stratified by Household source water quality

P−for−interaction = 0.169
Difference in MDs = 0.04 (−0.02, 0.11)

		Improved						Unimproved					
**Country**	**Trial**	**LNS N**	**Control N**	**Control Mean**	**MD (95% CI)**	**Fixed W**	**Random W**	**LNS N**	**Control N**	**Control Mean**	**MD (95% CI)**	**Fixed W**	**Random W**
Bangladesh	JiVitA−4 (34)												
Bangladesh	RDNS (35)												
Bangladesh	WASH−B (36)	496	989	−1.77	0.28 (0.18, 0.39)	0.16	0.12	70	114	−1.96	0.11 (−0.20, 0.42)	0.03	0.04
Burkina Faso	iLiNS−Zinc (37)	550	141	−1.68	0.21 (0.12, 0.30)	0.21	0.13	1394	519	−1.77	0.33 (0.24, 0.43)	0.33	0.21
Burkina Faso	PROMIS (38)	355	379	−1.49	0.12 (−0.03, 0.26)	0.08	0.09	361	355	−1.50	0.25 (0.01, 0.48)	0.05	0.07
Burkina Faso	PROMIS CS (38)	280	268	−1.25	0.09 (−0.13, 0.31)	0.04	0.06	149	171	−1.18	−0.06 (−0.32, 0.21)	0.04	0.06
Ghana	GHANA (39)												
Ghana	iLiNS−DYADG (40)												
Haiti	HAITI (41)												
Kenya	WASH−B (42)	447	1549	−1.52	0.10 (−0.03, 0.22)	0.11	0.11	248	697	−1.58	0.13 (−0.03, 0.29)	0.12	0.13
Madagascar	MAHAY (43)	419	453	−2.05	0.03 (−0.19, 0.24)	0.04	0.06	1203	1180	−2.31	0.04 (−0.11, 0.18)	0.14	0.14
Malawi	iLiNS−DYADM (44)	200	406	−1.62	−0.10 (−0.28, 0.08)	0.05	0.07	20	36	−1.63	0.08 (−0.59, 0.76)	0.01	0.01
Malawi	iLiNS−DOSE (45)	555	193	−1.89	0.03 (−0.08, 0.14)	0.13	0.11	52	17	−2.29	0.08 (−0.33, 0.48)	0.02	0.03
Mali	PROMIS (46)	249	258	−1.68	0.17 (0.00, 0.34)	0.06	0.08	247	243	−1.76	0.16 (−0.02, 0.33)	0.10	0.11
Mali	PROMIS CS (46)	549	566	−1.51	0.17 (−0.03, 0.38)	0.04	0.06	362	388	−1.66	0.27 (0.04, 0.50)	0.06	0.07
Zimbabwe	SHINE (HIV−) (47)	521	489	−1.62	0.15 (0.00, 0.30)	0.08	0.09	274	302	−1.56	0.07 (−0.11, 0.25)	0.09	0.11
Zimbabwe	SHINE (HIV+) (48)	85	87	−2.05	0.34 (−0.03, 0.71)	0.01	0.02	55	56	−2.03	0.18 (−0.24, 0.61)	0.02	0.02
		**4706**	**5778**		**I² = 0.52, Tau² = 0.01**			**4435**	**4078**		**I² = 0.45, Tau² = 0.00**		
**Fixed**					**0.14 (0.10, 0.18)**						**0.19 (0.13, 0.24)**		
**Random**					**0.13 (0.07, 0.19)**						**0.16 (0.09, 0.23)**		

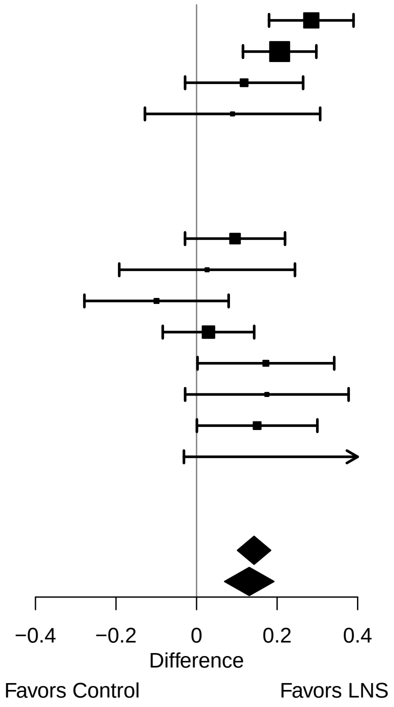

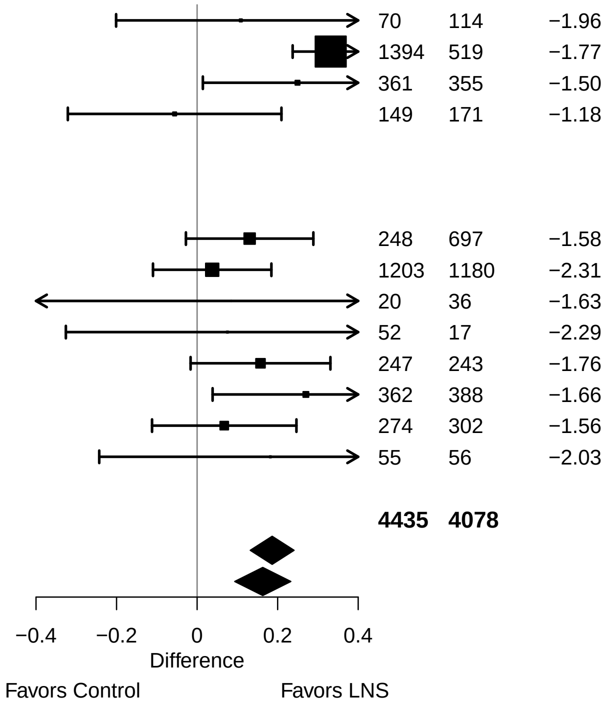

Supplemental figure 9A: Mean difference in LAZ

9A4: Stratified by Household sanitation

P−for−interaction = 0.135
Difference in MDs = −0.04 (−0.10, 0.01)

Country	Trial	Improved LNS N	Improved Control N	Improved Control Mean	Improved MD (95% CI)	Improved Fixed W	Improved Random W	Unimproved LNS N	Unimproved Control N	Unimproved Control Mean	Unimproved MD (95% CI)	Unimproved Fixed W	Unimproved Random W
Bangladesh	JiVitA−4 (34)	2189	950	−1.84	0.08 (0.03, 0.14)	0.42	0.36	648	294	−2.10	0.09 (0.00, 0.19)	0.17	0.10
Bangladesh	RDNS (35)	1179	573	−1.75	0.11 (0.02, 0.19)	0.17	0.17	482	241	−1.95	0.01 (−0.14, 0.15)	0.06	0.08
Bangladesh	WASH−B (36)	506	1001	−1.76	0.27 (0.15, 0.39)	0.09	0.10	31	54	−2.03	0.37 (0.00, 0.73)	0.01	0.03
Burkina Faso	iLiNS−Zinc (37)	43	16	−1.84	0.18 (−0.11, 0.46)	0.02	0.02	1901	644	−1.75	0.31 (0.23, 0.39)	0.21	0.11
Burkina Faso	PROMIS (38)	398	550	−1.49	0.20 (0.05, 0.35)	0.05	0.06	465	359	−1.47	0.10 (−0.04, 0.24)	0.07	0.09
Burkina Faso	PROMIS CS (38)	176	165	−1.29	0.18 (−0.05, 0.42)	0.02	0.03	252	274	−1.19	−0.06 (−0.27, 0.14)	0.03	0.07
Ghana	GHANA (39)												
Ghana	iLiNS−DYADG (40)	336	671	−0.88	0.17 (0.04, 0.30)	0.07	0.08	10	19	−1.29	0.96 (0.17, 1.75)	0.00	0.01
Haiti	HAITI (41)												
Kenya	WASH−B (42)	100	364	−1.33	0.14 (−0.09, 0.36)	0.02	0.03	595	1883	−1.58	0.10 (−0.01, 0.22)	0.11	0.09
Madagascar	MAHAY (43)												
Malawi	iLiNS−DYADM (44)	20	42	−1.35	0.20 (−0.37, 0.77)	0.00	0.00	200	400	−1.65	−0.11 (−0.29, 0.07)	0.04	0.07
Malawi	iLiNS−DOSE (45)	13	9	−1.54	0.06 (−0.55, 0.66)	0.00	0.00	593	201	−1.94	0.03 (−0.08, 0.15)	0.12	0.10
Mali	PROMIS (46)	387	366	−1.69	0.20 (0.03, 0.36)	0.05	0.05	112	135	−1.82	0.09 (−0.10, 0.28)	0.04	0.07
Mali	PROMIS CS (46)	701	696	−1.56	0.26 (0.08, 0.45)	0.04	0.04	220	248	−1.60	0.18 (−0.05, 0.41)	0.03	0.06
Zimbabwe	SHINE (HIV−) (47)	295	244	−1.51	0.14 (−0.03, 0.30)	0.05	0.05	497	546	−1.64	0.10 (−0.03, 0.22)	0.09	0.09
Zimbabwe	SHINE (HIV+) (48)	50	35	−2.11	0.46 (−0.02, 0.93)	0.01	0.01	90	107	−2.02	0.19 (−0.16, 0.53)	0.01	0.04
		**6393**	**5682**		**I² = 0.13, Tau² = 0.00**			**6096**	**5405**		**I² = 0.69, Tau² = 0.01**		
**Fixed**					**0.14 (0.10, 0.17)**						**0.12 (0.08, 0.16)**		
**Random**					**0.14 (0.11, 0.18)**						**0.11 (0.03, 0.18)**		

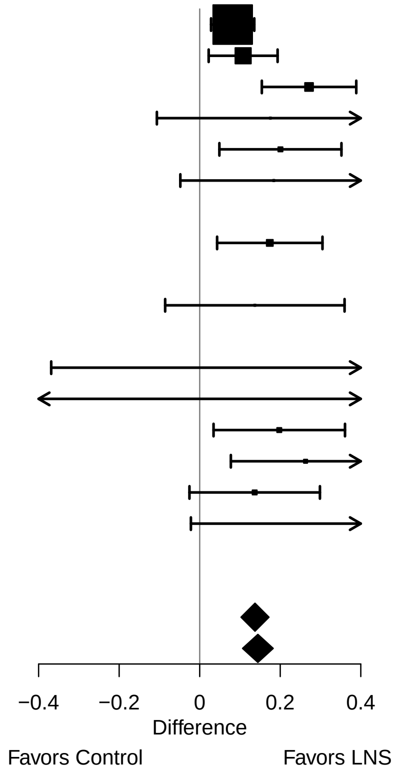

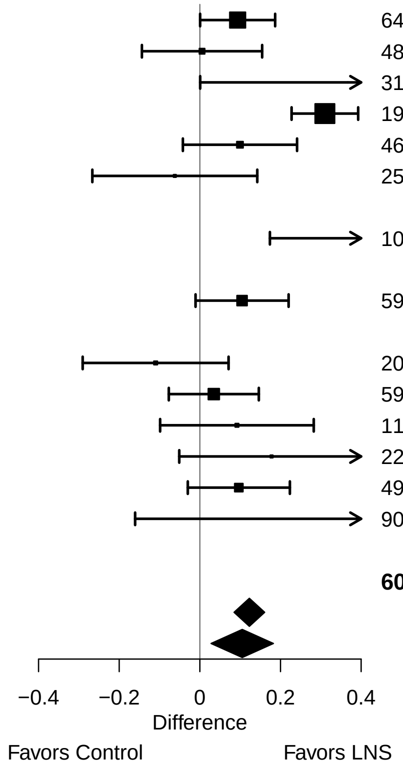

Supplemental figure 9A: Mean difference in LAZ

9A5: Stratified by Home environment

P−for−interaction = 0.394
Difference in MDs = 0.03 (−0.04, 0.09)

Country	Trial	At least median: LNS N	Control N	Control Mean	MD (95% CI)	Fixed W	Random W	Less than median: LNS N	Control N	Control Mean	MD (95% CI)	Fixed W	Random W
Bangladesh	JiVitA−4 (34)												
Bangladesh	RDNS (35)	1192	582	−1.75	0.07 (−0.03, 0.17)	0.18	0.16	468	230	−1.93	0.09 (−0.03, 0.21)	0.17	0.15
Bangladesh	WASH−B (36)	637	1653	−1.67	0.19 (0.09, 0.29)	0.18	0.16	493	1690	−1.96	0.18 (0.07, 0.29)	0.21	0.16
Burkina Faso	iLiNS−Zinc (37)	473	199	−1.62	0.24 (0.12, 0.35)	0.13	0.14	271	174	−1.87	0.25 (0.10, 0.41)	0.10	0.13
Burkina Faso	PROMIS (38)												
Burkina Faso	PROMIS CS (38)												
Ghana	GHANA (39)												
Ghana	iLiNS−DYADG (40)	213	409	−0.88	0.16 (−0.01, 0.32)	0.06	0.10	119	248	−0.93	0.27 (0.05, 0.49)	0.05	0.09
Haiti	HAITI (41)												
Kenya	WASH−B (42)	835	2764	−1.47	0.10 (0.02, 0.18)	0.27	0.18	574	2160	−1.65	0.18 (0.09, 0.28)	0.26	0.17
Madagascar	MAHAY (43)	915	907	−2.24	−0.05 (−0.22, 0.13)	0.06	0.09	787	775	−2.24	0.11 (−0.05, 0.28)	0.09	0.12
Malawi	iLiNS−DYADM (44)	137	289	−1.56	−0.06 (−0.28, 0.16)	0.04	0.07	78	149	−1.76	−0.11 (−0.40, 0.18)	0.03	0.06
Malawi	iLiNS−DOSE (45)	375	136	−2.05	0.16 (0.02, 0.30)	0.09	0.12	275	86	−1.75	−0.08 (−0.24, 0.09)	0.09	0.12
Mali	PROMIS (46)												
Mali	PROMIS CS (46)												
Zimbabwe	SHINE (HIV−) (47)												
Zimbabwe	SHINE (HIV+) (48)												
		**4777**	**6939**		**I² = 0.49, Tau² = 0.00**			**3065**	**5512**		**I² = 0.53, Tau² = 0.01**		
**Fixed**					**0.12 (0.08, 0.16)**						**0.14 (0.09, 0.19)**		
**Random**					**0.12 (0.05, 0.18)**						**0.13 (0.04, 0.21)**		

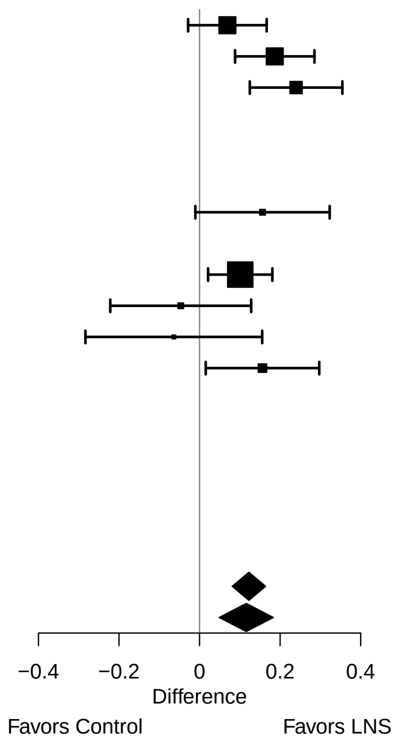

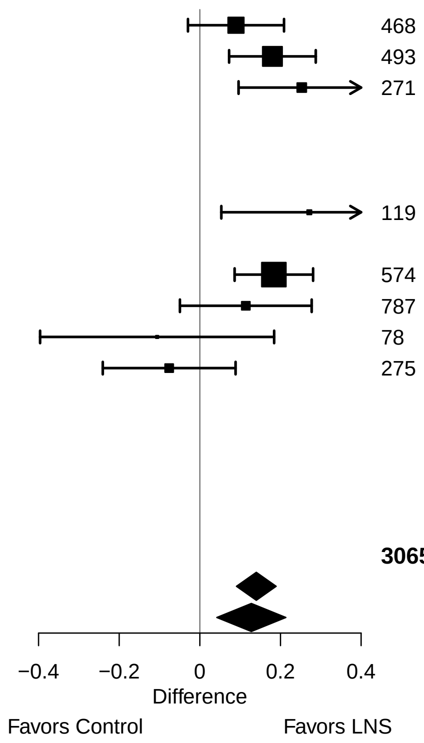

Supplemental figure 9A: Mean difference in LAZ

9A6: Stratified by Season at the time of assessment

P−for−interaction = 0.834
Difference in MDs = 0.00 (−0.04, 0.05)

Country	Trial	Dry LNS N	Dry Control N	Dry Control Mean	Dry MD (95% CI)	Dry Fixed W	Dry Random W	Rainy LNS N	Rainy Control N	Rainy Control Mean	Rainy MD (95% CI)	Rainy Fixed W	Rainy Random W
Bangladesh	JiVitA−4 (34)	1550	663	−1.90	0.08 (0.02, 0.14)	0.26	0.11	1288	581	−1.91	0.10 (0.02, 0.17)	0.22	0.15
Bangladesh	RDNS (35)	739	359	−1.82	0.05 (−0.07, 0.18)	0.06	0.08	924	456	−1.79	0.09 (−0.01, 0.20)	0.11	0.12
Bangladesh	WASH−B (36)	640	1913	−1.74	0.19 (0.09, 0.29)	0.11	0.10	518	1518	−1.91	0.23 (0.15, 0.32)	0.15	0.13
Burkina Faso	iLiNS−Zinc (37)	1394	477	−1.80	0.32 (0.22, 0.41)	0.11	0.10	558	187	−1.64	0.29 (0.18, 0.39)	0.10	0.12
Burkina Faso	PROMIS (38)	434	446	−1.41	0.09 (−0.06, 0.23)	0.05	0.08	429	468	−1.55	0.21 (0.04, 0.37)	0.04	0.08
Burkina Faso	PROMIS CS (38)	224	190	−1.30	0.16 (−0.10, 0.41)	0.02	0.04	206	249	−1.17	−0.06 (−0.33, 0.21)	0.02	0.04
Ghana	GHANA (39)	57	55	−0.43	0.48 (0.08, 0.88)	0.01	0.02	41	41	−0.38	0.00 (−0.46, 0.47)	0.01	0.01
Ghana	iLiNS−DYADG (40)	204	407	−0.88	0.14 (−0.03, 0.30)	0.04	0.07	143	285	−0.90	0.29 (0.09, 0.50)	0.03	0.06
Haiti	HAITI (41)												
Kenya	WASH−B (42)	347	1151	−1.56	0.24 (0.13, 0.35)	0.08	0.09	1110	3986	−1.57	0.13 (0.06, 0.20)	0.24	0.15
Madagascar	MAHAY (43)												
Malawi	iLiNS−DYADM (44)	156	316	−1.60	−0.06 (−0.27, 0.14)	0.02	0.06	64	128	−1.68	−0.11 (−0.44, 0.22)	0.01	0.03
Malawi	iLiNS−DOSE (45)	415	134	−1.90	−0.03 (−0.17, 0.10)	0.05	0.08	281	107	−1.99	0.20 (0.04, 0.36)	0.05	0.08
Mali	PROMIS (46)												
Mali	PROMIS CS (46)												
Zimbabwe	SHINE (HIV−) (47)	1747	1662	−1.62	0.17 (0.09, 0.25)	0.16	0.10	133	132	−1.40	−0.03 (−0.32, 0.26)	0.01	0.03
Zimbabwe	SHINE (HIV+) (48)	319	306	−2.02	0.26 (0.08, 0.44)	0.03	0.07	18	24	−1.78	0.23 (−0.35, 0.81)	0.00	0.01
		**8226**	**8079**		**I² = 0.69, Tau² = 0.01**			**5713**	**8162**		**I² = 0.48, Tau² = 0.00**		
**Fixed**					**0.15 (0.11, 0.18)**						**0.15 (0.12, 0.19)**		
**Random**					**0.15 (0.08, 0.21)**						**0.15 (0.09, 0.21)**		

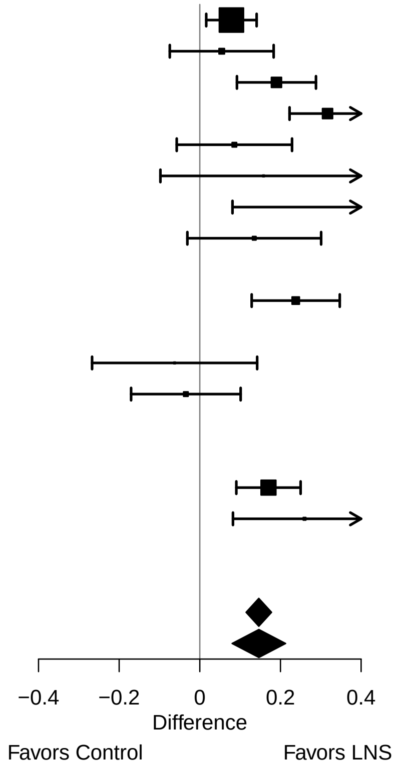

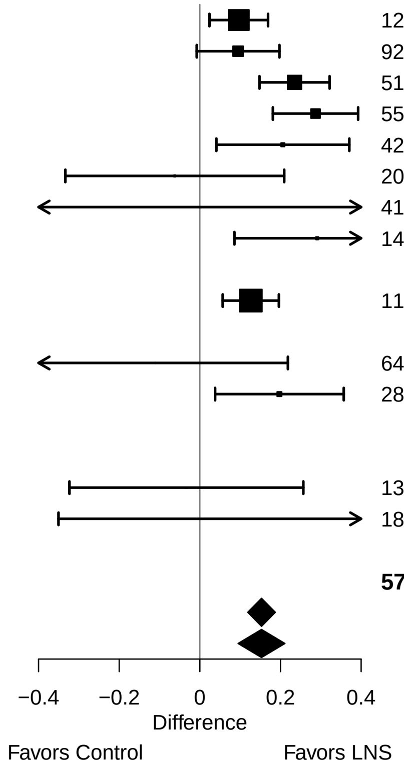

Supplemental figure 9B: Stunting prevalence ratio

9B1: Stratified by Household socio-economic status

P−for−interaction = 0.476
Ratio of PRs = 1.02 (0.96, 1.08)

		At least median						Less than median					
**Country**	**Trial**	**LNS N**	**Control N**	**Control Prevalence**	**PR (95% CI)**	**Fixed W**	**Random W**	**LNS N**	**Control N**	**Control Prevalence**	**PR (95% CI)**	**Fixed W**	**Random W**
Bangladesh	JiVitA−4 (34)	1429	621	39.0	0.87 (0.78, 0.98)	0.16	0.12	1408	623	49.4	0.94 (0.85, 1.05)	0.13	0.11
Bangladesh	RDNS (35)	834	407	35.9	0.86 (0.74, 1.01)	0.08	0.09	829	408	48.0	0.96 (0.89, 1.05)	0.21	0.13
Bangladesh	WASH−B (36)	579	1716	33.1	0.85 (0.74, 0.98)	0.10	0.09	579	1715	50.7	0.83 (0.75, 0.92)	0.15	0.12
Burkina Faso	iLiNS−Zinc (37)	1225	299	38.1	0.82 (0.71, 0.95)	0.09	0.09	720	364	40.3	0.70 (0.56, 0.87)	0.03	0.05
Burkina Faso	PROMIS (38)	407	482	32.0	0.75 (0.58, 0.98)	0.03	0.04	456	432	28.9	0.89 (0.68, 1.17)	0.02	0.04
Burkina Faso	PROMIS CS (38)	220	210	21.0	1.11 (0.76, 1.60)	0.01	0.02	210	229	27.5	0.69 (0.41, 1.16)	0.01	0.01
Ghana	GHANA (39)												
Ghana	iLiNS−DYADG (40)	157	356	10.4	0.80 (0.44, 1.46)	0.01	0.01	190	334	15.3	0.59 (0.35, 0.98)	0.01	0.01
Haiti	HAITI (41)	75	60	18.3	0.73 (0.33, 1.62)	0.00	0.01	61	72	9.7	0.99 (0.47, 2.08)	0.00	0.01
Kenya	WASH−B (42)	825	2921	28.8	0.80 (0.69, 0.93)	0.09	0.09	630	2212	36.8	0.91 (0.80, 1.03)	0.09	0.10
Madagascar	MAHAY (43)	771	884	58.5	1.02 (0.91, 1.15)	0.16	0.12	878	755	58.5	0.94 (0.86, 1.04)	0.15	0.12
Malawi	iLiNS−DYADM (44)	121	234	31.2	0.98 (0.71, 1.36)	0.02	0.03	99	209	38.3	1.24 (0.95, 1.62)	0.02	0.04
Malawi	iLiNS−DOSE (45)	301	104	37.5	1.11 (0.88, 1.40)	0.04	0.05	294	103	55.3	0.96 (0.80, 1.17)	0.04	0.06
Mali	PROMIS (46)	247	251	37.5	0.88 (0.71, 1.08)	0.05	0.06	246	246	36.2	0.93 (0.66, 1.29)	0.01	0.03
Mali	PROMIS CS (46)	505	463	33.3	0.71 (0.57, 0.90)	0.04	0.05	447	506	34.2	0.86 (0.68, 1.10)	0.02	0.04
Zimbabwe	SHINE (HIV−) (47)	908	802	33.4	0.73 (0.63, 0.85)	0.09	0.09	832	860	37.3	0.82 (0.72, 0.94)	0.08	0.09
Zimbabwe	SHINE (HIV+) (48)	171	156	45.5	0.84 (0.64, 1.10)	0.03	0.04	158	167	53.9	0.81 (0.64, 1.03)	0.03	0.05
		**8775**	**9966**		**I^2 = 0.41, Tau^2 = 0.00**			**8037**	**9235**		**I^2 = 0.38, Tau^2 = 0.01**		
**Fixed**					**0.87 (0.83, 0.91)**						**0.90 (0.87, 0.94)**		
**Random**					**0.86 (0.81, 0.92)**						**0.89 (0.84, 0.95)**		

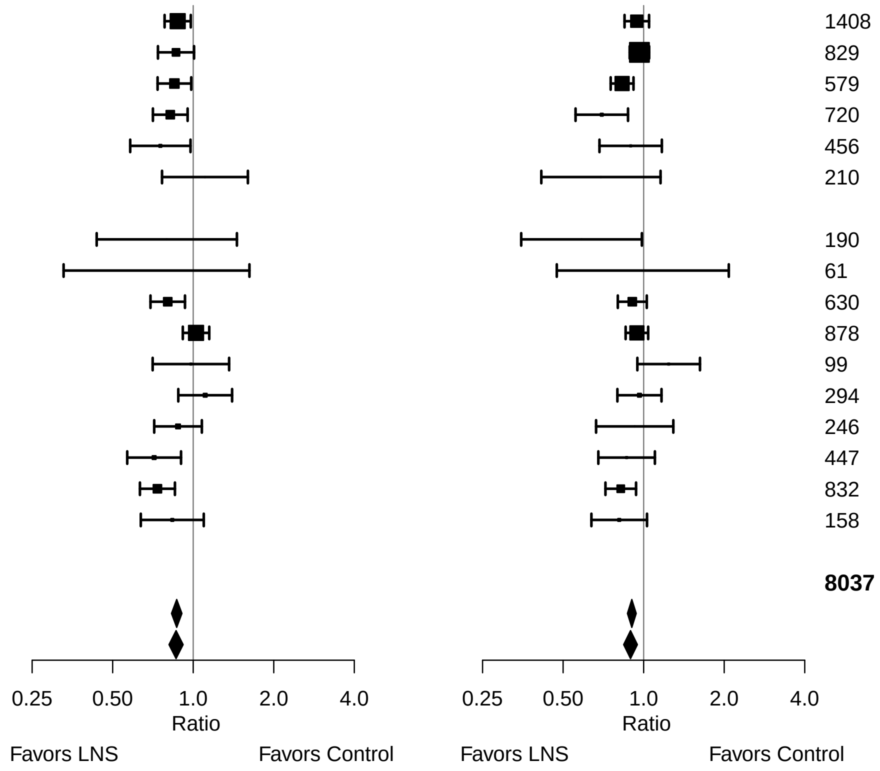

Supplemental figure 9B: Stunting prevalence ratio

9B2: Stratified by Household food insecurity

P−for−interaction = 0.827
Ratio of PRs = 1.01 (0.94, 1.08)

Mild to secure

Country	Trial	LNS N	Control N	Control Prevalence	PR (95% CI)	Fixed W	Random W
Bangladesh	JiVitA−4 (34)	2012	863	41.2	0.86 (0.78, 0.95)	0.16	0.14
Bangladesh	RDNS (35)	1058	493	36.3	0.99 (0.88, 1.12)	0.11	0.11
Bangladesh	WASH−B (36)	918	2652	39.4	0.81 (0.73, 0.90)	0.15	0.13
Burkina Faso	iLiNS−Zinc (37)	1013	320	40.5	0.76 (0.60, 0.96)	0.03	0.04
Burkina Faso	PROMIS (38)	502	550	29.6	0.88 (0.71, 1.10)	0.03	0.05
Burkina Faso	PROMIS CS (38)						
Ghana	GHANA (39)						
Ghana	iLiNS−DYADG (40)	244	470	12.6	0.62 (0.38, 1.02)	0.01	0.01
Haiti	HAITI (41)						
Kenya	WASH−B (42)	1299	4595	31.3	0.86 (0.78, 0.95)	0.16	0.14
Madagascar	MAHAY (43)	988	927	59.4	0.96 (0.86, 1.06)	0.15	0.13
Malawi	iLiNS−DYADM (44)	70	122	27.0	1.16 (0.74, 1.83)	0.01	0.01
Malawi	iLiNS−DOSE (45)	151	55	38.2	0.99 (0.73, 1.35)	0.02	0.02
Mali	PROMIS (46)	329	341	35.8	0.92 (0.73, 1.14)	0.03	0.04
Mali	PROMIS CS (46)						
Zimbabwe	SHINE (HIV−) (47)	1393	1305	34.6	0.80 (0.72, 0.90)	0.13	0.12
Zimbabwe	SHINE (HIV+) (48)	236	239	46.4	0.85 (0.68, 1.05)	0.03	0.05
		**10213**	**12932**		**I² = 0.30, Tau² = 0.00**		
**Fixed**					**0.87 (0.84, 0.91)**		
**Random**					**0.88 (0.83, 0.92)**		

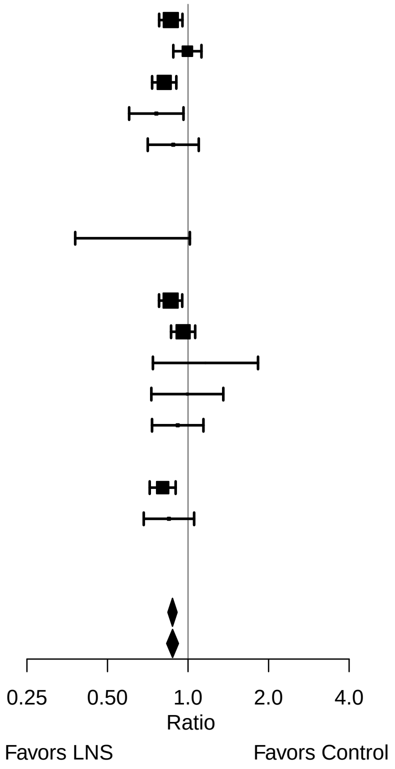

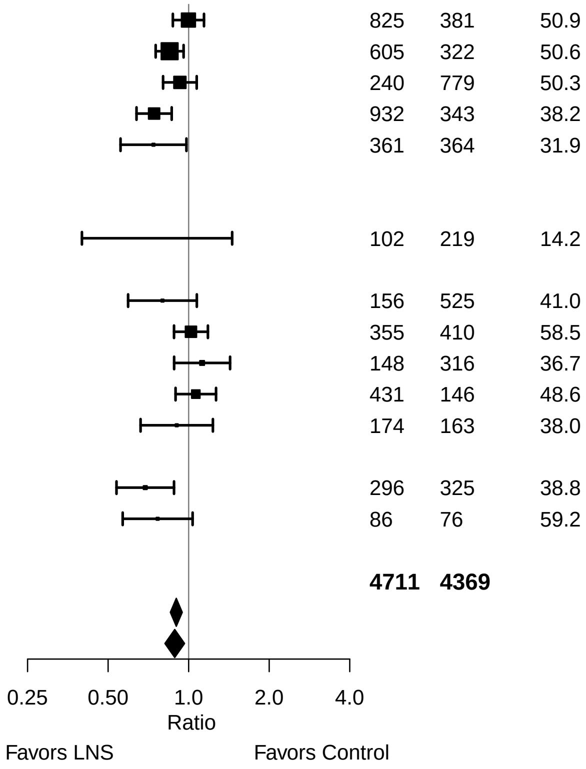

Moderate to severe

Country	Trial	LNS N	Control N	Control Prevalence	PR (95% CI)	Fixed W	Random W
Bangladesh	JiVitA−4 (34)	825	381	50.9	1.00 (0.87, 1.14)	0.15	0.11
Bangladesh	RDNS (35)	605	322	50.6	0.85 (0.75, 0.96)	0.18	0.12
Bangladesh	WASH−B (36)	240	779	50.3	0.93 (0.80, 1.07)	0.13	0.11
Burkina Faso	iLiNS−Zinc (37)	932	343	38.2	0.74 (0.64, 0.86)	0.12	0.10
Burkina Faso	PROMIS (38)	361	364	31.9	0.74 (0.56, 0.98)	0.03	0.06
Burkina Faso	PROMIS CS (38)						
Ghana	GHANA (39)						
Ghana	iLiNS−DYADG (40)	102	219	14.2	0.76 (0.40, 1.45)	0.01	0.02
Haiti	HAITI (41)						
Kenya	WASH−B (42)	156	525	41.0	0.80 (0.59, 1.07)	0.03	0.05
Madagascar	MAHAY (43)	355	410	58.5	1.02 (0.88, 1.18)	0.12	0.11
Malawi	iLiNS−DYADM (44)	148	316	36.7	1.12 (0.88, 1.43)	0.05	0.07
Malawi	iLiNS−DOSE (45)	431	146	48.6	1.06 (0.89, 1.27)	0.09	0.09
Mali	PROMIS (46)	174	163	38.0	0.90 (0.66, 1.23)	0.03	0.05
Mali	PROMIS CS (46)						
Zimbabwe	SHINE (HIV−) (47)	296	325	38.8	0.69 (0.54, 0.88)	0.04	0.07
Zimbabwe	SHINE (HIV+) (48)	86	76	59.2	0.77 (0.57, 1.03)	0.03	0.05
		**4711**	**4369**		**I² = 0.56, Tau² = 0.01**		
**Fixed**					**0.90 (0.85, 0.95)**		
**Random**					**0.89 (0.82, 0.97)**		

Supplemental figure 9B: Stunting prevalence ratio

9B3: Stratified by Household source water quality

P−for−interaction = 0.782
Ratio of PRs = 1.01 (0.93, 1.10)

		Improved						Unimproved					
**Country**	**Trial**	**LNS N**	**Control N**	**Control Prevalence**	**PR (95% CI)**	**Fixed W**	**Random W**	**LNS N**	**Control N**	**Control Prevalence**	**PR (95% CI)**	**Fixed W**	**Random W**
Bangladesh	JiVitA−4 (34)												
Bangladesh	RDNS (35)												
Bangladesh	WASH−B (36)	496	989	40.7	0.77 (0.67, 0.88)	0.18	0.15	70	114	43.0	1.00 (0.71, 1.40)	0.03	0.07
Burkina Faso	iLiNS−Zinc (37)	550	141	35.5	0.82 (0.68, 0.99)	0.10	0.10	1394	519	40.2	0.74 (0.63, 0.86)	0.15	0.14
Burkina Faso	PROMIS (38)	355	379	31.1	0.92 (0.74, 1.15)	0.07	0.08	361	355	31.5	0.62 (0.42, 0.92)	0.03	0.06
Burkina Faso	PROMIS CS (38)	280	268	25.4	0.77 (0.54, 1.11)	0.03	0.03	149	171	22.8	1.06 (0.71, 1.58)	0.02	0.05
Ghana	GHANA (39)												
Ghana	iLiNS−DYADG (40)												
Haiti	HAITI (41)												
Kenya	WASH−B (42)	447	1549	30.6	0.96 (0.80, 1.14)	0.11	0.11	248	697	33.1	0.85 (0.66, 1.09)	0.06	0.10
Madagascar	MAHAY (43)	419	453	51.4	1.02 (0.86, 1.20)	0.13	0.12	1203	1180	61.2	0.96 (0.88, 1.05)	0.47	0.18
Malawi	iLiNS−DYADM (44)												
Malawi	iLiNS−DOSE (45)	555	193	46.1	1.01 (0.86, 1.19)	0.13	0.12	52	17	47.1	1.33 (0.85, 2.07)	0.02	0.05
Mali	PROMIS (46)	249	258	36.4	0.80 (0.63, 1.01)	0.06	0.07	247	243	36.2	1.04 (0.82, 1.32)	0.07	0.10
Mali	PROMIS CS (46)	549	566	31.4	0.81 (0.64, 1.02)	0.06	0.07	362	388	37.6	0.78 (0.62, 0.98)	0.07	0.11
Zimbabwe	SHINE (HIV−) (47)	521	489	35.2	0.77 (0.65, 0.93)	0.11	0.11	274	302	32.5	0.97 (0.74, 1.26)	0.05	0.09
Zimbabwe	SHINE (HIV+) (48)	85	87	49.4	0.76 (0.55, 1.05)	0.03	0.04	55	56	57.1	0.76 (0.50, 1.16)	0.02	0.05
		**4506**	**5372**		**I² = 0.36, Tau² = 0.00**			**4415**	**4042**		**I² = 0.50, Tau² = 0.01**		
**Fixed**					**0.87 (0.82, 0.92)**						**0.90 (0.85, 0.96)**		
**Random**					**0.87 (0.81, 0.93)**						**0.89 (0.80, 0.99)**		

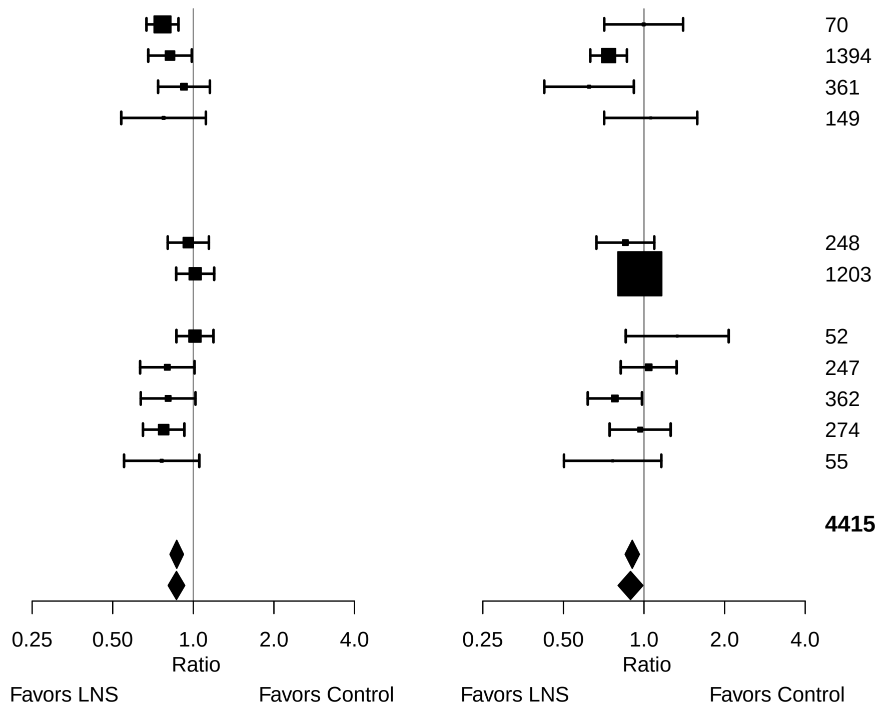

Supplemental figure 9B: Stunting prevalence ratio

9B4: Stratified by Household sanitation

P−for−interaction = 0.200
Ratio of PRs = 1.06 (0.97, 1.17)

Country	Trial	Improved LNS N	Improved Control N	Improved Control Prevalence	Improved PR (95% CI)	Improved Fixed W	Improved Random W	Unimproved LNS N	Unimproved Control N	Unimproved Control Prevalence	Unimproved PR (95% CI)	Unimproved Fixed W	Unimproved Random W
Bangladesh	JiVitA−4 (34)	2189	950	42.5	0.92 (0.82, 1.02)	0.27	0.27	648	294	49.7	0.94 (0.82, 1.07)	0.19	0.18
Bangladesh	RDNS (35)	1179	573	39.6	0.88 (0.79, 0.97)	0.30	0.30	482	241	47.7	1.01 (0.87, 1.17)	0.16	0.16
Bangladesh	WASH−B (36)	506	1001	39.7	0.78 (0.66, 0.91)	0.13	0.13	31	54	51.9	0.75 (0.45, 1.23)	0.01	0.02
Burkina Faso	iLiNS−Zinc (37)	43	16	37.5	1.37 (0.61, 3.07)	0.00	0.00	1901	644	39.2	0.75 (0.65, 0.87)	0.16	0.16
Burkina Faso	PROMIS (38)	398	550	31.3	0.79 (0.63, 0.99)	0.06	0.06	465	359	29.2	0.87 (0.69, 1.10)	0.06	0.07
Burkina Faso	PROMIS CS (38)	176	165	24.2	0.87 (0.56, 1.35)	0.02	0.02	252	274	24.5	0.88 (0.62, 1.24)	0.03	0.03
Ghana	GHANA (39)												
Ghana	iLiNS−DYADG (40)												
Haiti	HAITI (41)												
Kenya	WASH−B (42)	100	364	24.7	0.89 (0.61, 1.30)	0.02	0.02	595	1883	32.7	0.92 (0.78, 1.09)	0.13	0.13
Madagascar	MAHAY (43)												
Malawi	iLiNS−DYADM (44)												
Malawi	iLiNS−DOSE (45)												
Mali	PROMIS (46)	387	366	36.1	0.87 (0.70, 1.08)	0.07	0.07	112	135	37.8	1.03 (0.76, 1.39)	0.04	0.04
Mali	PROMIS CS (46)	701	696	32.6	0.76 (0.60, 0.96)	0.06	0.06	220	248	35.9	0.80 (0.60, 1.07)	0.04	0.04
Zimbabwe	SHINE (HIV−) (47)	295	244	32.0	0.82 (0.63, 1.05)	0.05	0.05	497	546	35.2	0.85 (0.73, 1.00)	0.14	0.14
Zimbabwe	SHINE (HIV+) (48)	50	35	54.3	0.70 (0.46, 1.08)	0.02	0.02	90	107	51.4	0.80 (0.57, 1.11)	0.03	0.03
		**6024**	**4960**		**I^2 = 0.00, Tau^2 = 0.00**			**5293**	**4785**		**I^2 = 0.09, Tau^2 = 0.00**		
**Fixed**					**0.86 (0.81, 0.91)**						**0.88 (0.83, 0.94)**		
**Random**					**0.86 (0.81, 0.91)**						**0.88 (0.83, 0.94)**		

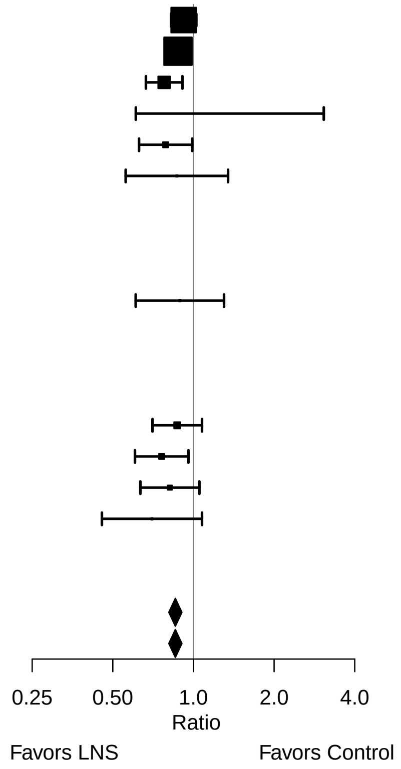

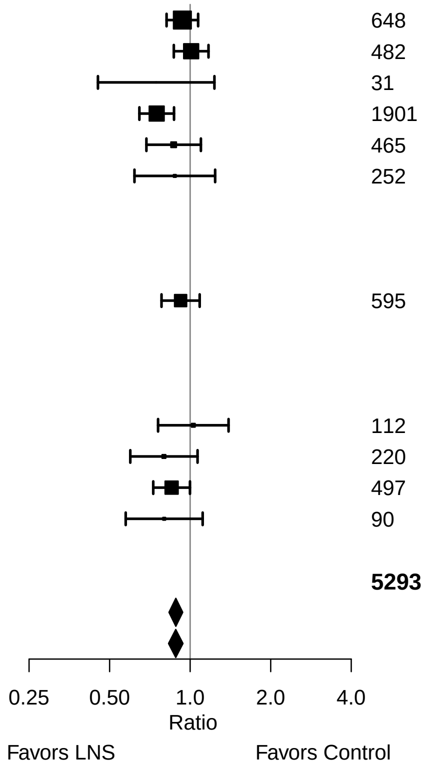

Supplemental figure 9B: Stunting prevalence ratio

9B5: Stratified by Home environment

P−for−interaction = 0.280
Ratio of PRs = 0.96 (0.89, 1.03)

		At least median						Less than median					
**Country**	**Trial**	**LNS N**	**Control N**	**Control Prevalence**	**PR (95% CI)**	**Fixed W**	**Random W**	**LNS N**	**Control N**	**Control Prevalence**	**PR (95% CI)**	**Fixed W**	**Random W**
Bangladesh	JiVitA−4 (34)												
Bangladesh	RDNS (35)	1192	582	39.2	0.94 (0.84, 1.06)	0.21	0.16	468	230	48.7	0.88 (0.78, 0.99)	0.21	0.19
Bangladesh	WASH−B (36)	637	1653	36.9	0.84 (0.74, 0.96)	0.17	0.15	493	1690	46.9	0.87 (0.77, 0.98)	0.23	0.20
Burkina Faso	iLiNS−Zinc (37)	473	199	37.7	0.66 (0.54, 0.81)	0.07	0.11	271	174	41.7	0.80 (0.60, 1.05)	0.04	0.07
Burkina Faso	PROMIS (38)												
Burkina Faso	PROMIS CS (38)												
Ghana	GHANA (39)												
Ghana	iLiNS−DYADG (40)	213	409	12.0	0.78 (0.48, 1.28)	0.01	0.04	119	248	15.3	0.55 (0.28, 1.06)	0.01	0.02
Haiti	HAITI (41)												
Kenya	WASH−B (42)	835	2764	29.6	0.89 (0.78, 1.01)	0.17	0.16	574	2160	34.3	0.86 (0.75, 1.00)	0.15	0.17
Madagascar	MAHAY (43)	915	907	57.9	1.04 (0.94, 1.16)	0.25	0.17	787	775	59.2	0.91 (0.82, 1.02)	0.27	0.21
Malawi	iLiNS−DYADM (44)	137	289	33.6	1.07 (0.81, 1.41)	0.04	0.08	78	149	36.9	1.18 (0.85, 1.64)	0.03	0.06
Malawi	iLiNS−DOSE (45)	375	136	49.3	0.95 (0.80, 1.13)	0.09	0.13	275	86	40.7	1.10 (0.86, 1.41)	0.05	0.09
Mali	PROMIS (46)												
Mali	PROMIS CS (46)												
Zimbabwe	SHINE (HIV−) (47)												
Zimbabwe	SHINE (HIV+) (48)												
		**4777**	**6939**		**I² = 0.64, Tau² = 0.01**			**3065**	**5512**		**I² = 0.22, Tau² = 0.01**		
**Fixed**					**0.92 (0.87, 0.97)**						**0.90 (0.85, 0.95)**		
**Random**					**0.90 (0.81, 1.00)**						**0.90 (0.83, 0.98)**		

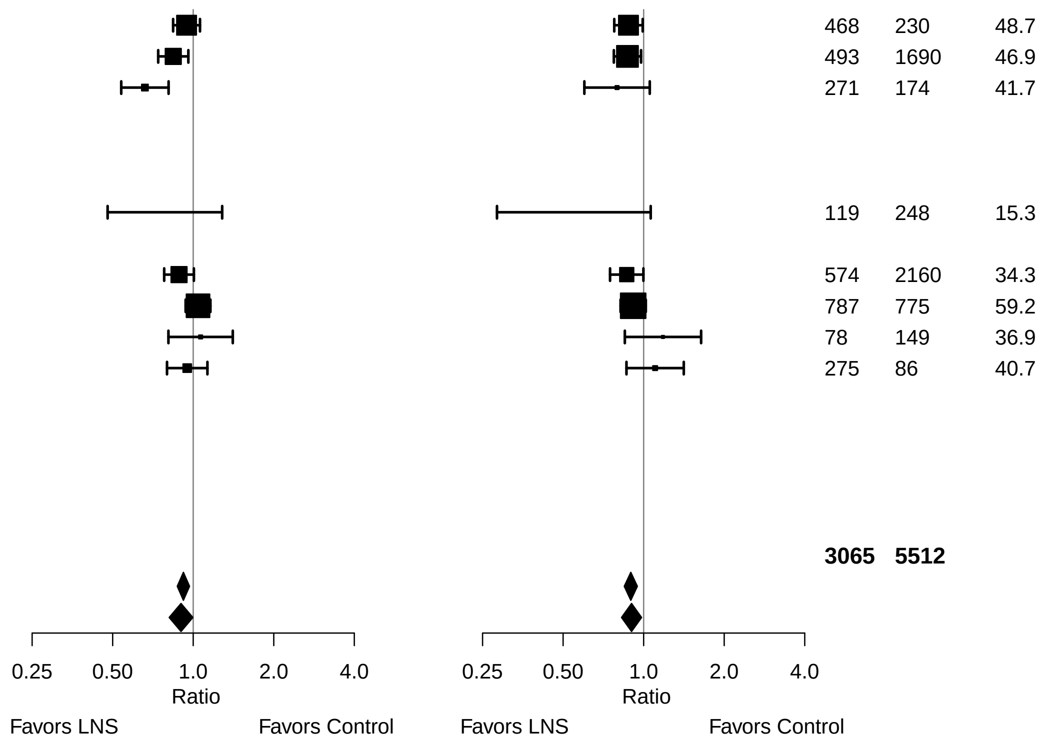

Supplemental figure 9B: Stunting prevalence ratio

9B6: Stratified by Season at the time of assessment

P−for−interaction = 0.389
Ratio of PRs = 0.97 (0.90, 1.04)

Country	Trial	LNS N	Control N	Control Prevalence	Dry PR (95% CI)	Fixed W	Random W	LNS N	Control N	Control Prevalence	Rainy PR (95% CI)	Fixed W	Random W
Bangladesh	JiVitA−4 (34)	1550	663	44.9	0.96 (0.87, 1.07)	0.17	0.13	1288	581	43.4	0.87 (0.75, 1.00)	0.13	0.13
Bangladesh	RDNS (35)	739	359	43.7	0.90 (0.78, 1.04)	0.10	0.11	924	456	40.6	0.94 (0.85, 1.04)	0.25	0.25
Bangladesh	WASH−B (36)	640	1913	39.1	0.86 (0.77, 0.96)	0.15	0.12	518	1518	45.5	0.82 (0.73, 0.91)	0.20	0.20
Burkina Faso	iLiNS−Zinc (37)	1394	477	40.3	0.76 (0.67, 0.86)	0.13	0.12	558	187	37.2	0.72 (0.54, 0.96)	0.03	0.03
Burkina Faso	PROMIS (38)	434	446	27.1	0.84 (0.65, 1.09)	0.03	0.05	429	468	33.8	0.81 (0.64, 1.01)	0.05	0.05
Burkina Faso	PROMIS CS (38)	224	190	25.3	0.80 (0.52, 1.21)	0.01	0.02	206	249	23.7	0.94 (0.58, 1.52)	0.01	0.01
Ghana	GHANA (39)												
Ghana	iLiNS−DYADG (40)	204	407	12.8	0.77 (0.47, 1.25)	0.01	0.02	143	285	13.3	0.52 (0.27, 1.02)	0.01	0.01
Haiti	HAITI (41)												
Kenya	WASH−B (42)	347	1151	30.1	0.81 (0.66, 1.00)	0.04	0.07	1110	3986	32.8	0.87 (0.79, 0.97)	0.24	0.24
Madagascar	MAHAY (43)												
Malawi	iLiNS−DYADM (44)	156	316	32.9	1.13 (0.87, 1.46)	0.03	0.05	64	128	39.1	1.04 (0.72, 1.50)	0.02	0.02
Malawi	iLiNS−DOSE (45)	415	134	44.0	1.08 (0.90, 1.28)	0.06	0.09	281	107	49.5	0.88 (0.71, 1.10)	0.05	0.05
Mali	PROMIS (46)												
Mali	PROMIS CS (46)												
Zimbabwe	SHINE (HIV−) (47)	1747	1662	35.4	0.77 (0.70, 0.86)	0.20	0.13	133	132	28.0	1.05 (0.71, 1.55)	0.02	0.02
Zimbabwe	SHINE (HIV+) (48)	319	306	50.7	0.81 (0.68, 0.97)	0.06	0.08	18	24	41.7	0.67 (0.28, 1.60)	0.00	0.00
		**8169**	**8024**		**I^2 = 0.56, Tau^2 = 0.01**			**5672**	**8121**		**I^2 = 0.00, Tau^2 = 0.00**		
**Fixed**					**0.86 (0.82, 0.90)**						**0.87 (0.83, 0.92)**		
**Random**					**0.87 (0.81, 0.93)**						**0.87 (0.83, 0.92)**		

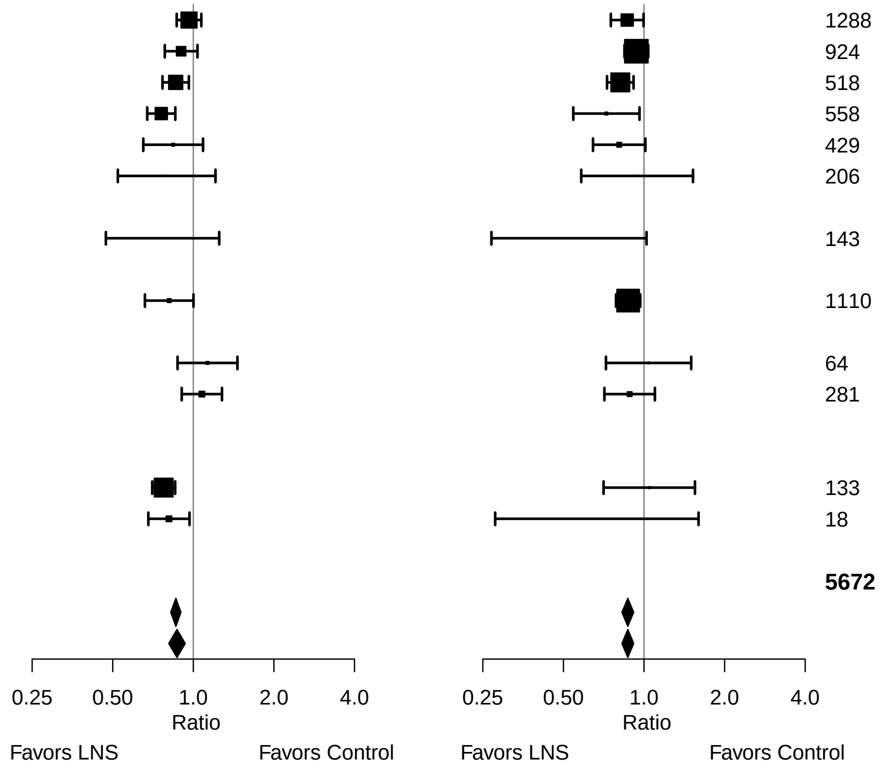

Supplemental figure 9C: Stunting prevalence difference

9C1: Stratified by Household socio-economic status

P−for−interaction = 0.724
Difference in PDs = 0.00 (−0.02, 0.02)

		At least median						Less than median					
**Country**	**Trial**	**LNS N**	**Control N**	**Control Prevalence**	**PD (95% CI)**	**Fixed W**	**Random W**	**LNS N**	**Control N**	**Control Prevalence**	**PD (95% CI)**	**Fixed W**	**Random W**
Bangladesh	JiVitA−4 (34)	1429	621	39.0	−0.04 (−0.08, 0.00)	0.13	0.10	1408	623	49.4	−0.03 (−0.07, 0.01)	0.13	0.10
Bangladesh	RDNS (35)	834	407	35.9	−0.05 (−0.10, 0.00)	0.07	0.07	829	408	48.0	−0.02 (−0.06, 0.02)	0.14	0.10
Bangladesh	WASH−B (36)	579	1716	33.1	−0.05 (−0.09, −0.01)	0.10	0.09	579	1715	50.7	−0.09 (−0.13, −0.04)	0.11	0.09
Burkina Faso	iLiNS−Zinc (37)	1225	299	38.1	−0.08 (−0.12, −0.03)	0.11	0.09	720	364	40.3	−0.14 (−0.20, −0.08)	0.06	0.07
Burkina Faso	PROMIS (38)	407	482	32.0	−0.08 (−0.15, 0.00)	0.03	0.04	456	432	28.9	−0.03 (−0.10, 0.04)	0.04	0.05
Burkina Faso	PROMIS CS (38)	220	210	21.0	0.02 (−0.06, 0.10)	0.03	0.04	210	229	27.5	−0.08 (−0.20, 0.03)	0.02	0.03
Ghana	GHANA (39)												
Ghana	iLiNS−DYADG (40)	157	356	10.4	−0.02 (−0.08, 0.03)	0.06	0.07	190	334	15.3	−0.06 (−0.12, 0.00)	0.06	0.07
Haiti	HAITI (41)	75	60	18.3	−0.08 (−0.17, 0.02)	0.02	0.03	61	72	9.7	0.01 (−0.08, 0.09)	0.03	0.04
Kenya	WASH−B (42)	825	2921	28.8	−0.06 (−0.09, −0.02)	0.15	0.11	630	2212	36.8	−0.03 (−0.08, 0.01)	0.12	0.10
Madagascar	MAHAY (43)	771	884	58.5	0.01 (−0.05, 0.08)	0.04	0.05	878	755	58.5	−0.03 (−0.09, 0.02)	0.07	0.08
Malawi	iLiNS−DYADM (44)	121	234	31.2	−0.01 (−0.11, 0.10)	0.02	0.03	99	209	38.3	0.09 (−0.03, 0.21)	0.02	0.03
Malawi	iLiNS−DOSE (45)	301	104	37.5	0.05 (−0.04, 0.13)	0.03	0.04	294	103	55.3	−0.05 (−0.14, 0.04)	0.03	0.04
Mali	PROMIS (46)	247	251	37.5	−0.06 (−0.13, 0.01)	0.04	0.05	246	246	36.2	−0.03 (−0.13, 0.07)	0.02	0.03
Mali	PROMIS CS (46)	505	463	33.3	−0.09 (−0.16, −0.03)	0.05	0.06	447	506	34.2	−0.05 (−0.12, 0.03)	0.04	0.05
Zimbabwe	SHINE (HIV−) (47)	908	802	33.4	−0.09 (−0.13, −0.05)	0.11	0.09	832	860	37.3	−0.07 (−0.11, −0.02)	0.11	0.09
Zimbabwe	SHINE (HIV+) (48)	171	156	45.5	−0.08 (−0.19, 0.04)	0.01	0.02	158	167	53.9	−0.10 (−0.22, 0.02)	0.02	0.03
		**8775**	**9966**		**I² = 0.29, Tau² = 0.00**			**8037**	**9235**		**I² = 0.39, Tau² = 0.00**		
**Fixed**					**−0.05 (−0.06, −0.04)**						**−0.05 (−0.06, −0.03)**		
**Random**					**−0.05 (−0.07, −0.03)**						**−0.05 (−0.07, −0.03)**		

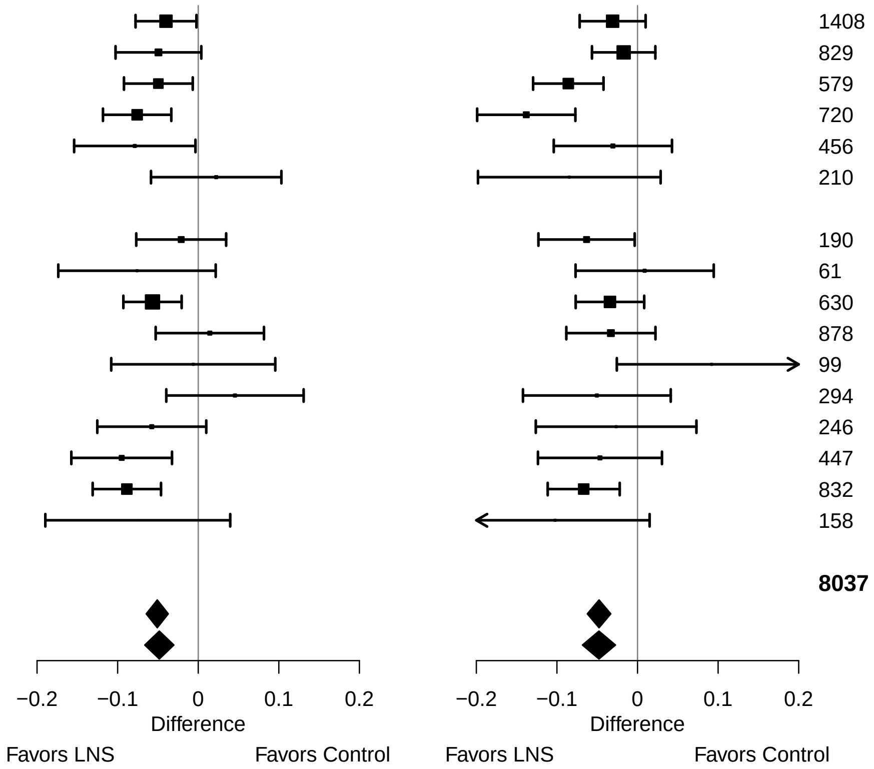

Supplemental figure 9C: Stunting prevalence difference

9C2: Stratified by Household food insecurity

P−for−interaction = 0.663
Difference in PDs = −0.01 (−0.03, 0.02)

Mild to secure

Country	Trial	LNS N	Control N	Control Prevalence	PD (95% CI)	Fixed W	Random W
Bangladesh	JiVitA−4 (34)	2012	863	41.2	−0.04 (−0.08, −0.01)	0.14	0.13
Bangladesh	RDNS (35)	1058	493	36.3	0.00 (−0.05, 0.04)	0.09	0.10
Bangladesh	WASH−B (36)	918	2652	39.4	−0.07 (−0.11, −0.04)	0.13	0.12
Burkina Faso	iLiNS−Zinc (37)	1013	320	40.5	−0.11 (−0.19, −0.04)	0.03	0.04
Burkina Faso	PROMIS (38)	502	550	29.6	−0.04 (−0.10, 0.03)	0.05	0.06
Burkina Faso	PROMIS CS (38)						
Ghana	GHANA (39)						
Ghana	iLiNS−DYADG (40)	244	470	12.6	−0.05 (−0.10, 0.00)	0.07	0.08
Haiti	HAITI (41)						
Kenya	WASH−B (42)	1299	4595	31.3	−0.04 (−0.07, −0.02)	0.22	0.17
Madagascar	MAHAY (43)	988	927	59.4	−0.02 (−0.08, 0.04)	0.05	0.06
Malawi	iLiNS−DYADM (44)	70	122	27.0	0.04 (−0.09, 0.18)	0.01	0.01
Malawi	iLiNS−DOSE (45)	151	55	38.2	0.04 (−0.07, 0.15)	0.01	0.02
Mali	PROMIS (46)	329	341	35.8	−0.04 (−0.11, 0.03)	0.04	0.05
Mali	PROMIS CS (46)						
Zimbabwe	SHINE (HIV−) (47)	1393	1305	34.6	−0.07 (−0.10, −0.03)	0.14	0.13
Zimbabwe	SHINE (HIV+) (48)	236	239	46.4	−0.07 (−0.16, 0.02)	0.02	0.03
		**10213**	**12932**		**I² = 0.23, Tau² = 0.00**		
**Fixed**					**−0.05 (−0.06, −0.03)**		
**Random**					**−0.05 (−0.06, −0.03)**		

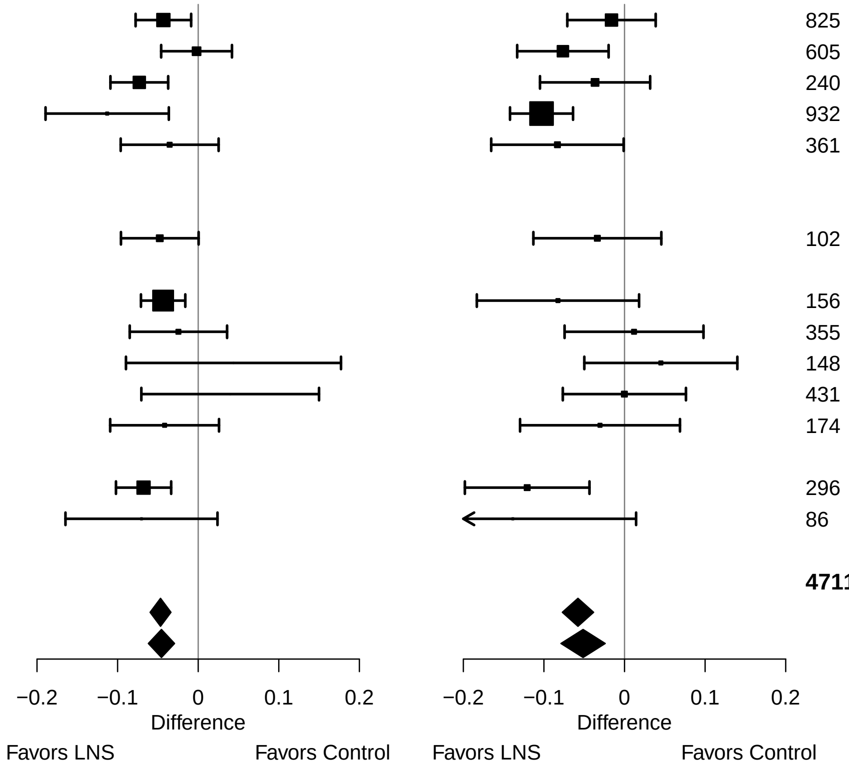

Moderate to severe

Country	Trial	LNS N	Control N	Control Prevalence	PD (95% CI)	Fixed W	Random W
Bangladesh	JiVitA−4 (34)	825	381	50.9	−0.02 (−0.07, 0.04)	0.13	0.11
Bangladesh	RDNS (35)	605	322	50.6	−0.08 (−0.13, −0.02)	0.12	0.11
Bangladesh	WASH−B (36)	240	779	50.3	−0.04 (−0.10, 0.03)	0.08	0.09
Burkina Faso	iLiNS−Zinc (37)	932	343	38.2	−0.10 (−0.14, −0.06)	0.25	0.14
Burkina Faso	PROMIS (38)	361	364	31.9	−0.08 (−0.17, 0.00)	0.06	0.07
Burkina Faso	PROMIS CS (38)						
Ghana	GHANA (39)						
Ghana	iLiNS−DYADG (40)	102	219	14.2	−0.03 (−0.11, 0.05)	0.06	0.07
Haiti	HAITI (41)						
Kenya	WASH−B (42)	156	525	41.0	−0.08 (−0.18, 0.02)	0.04	0.05
Madagascar	MAHAY (43)	355	410	58.5	0.01 (−0.07, 0.10)	0.05	0.07
Malawi	iLiNS−DYADM (44)	148	316	36.7	0.05 (−0.05, 0.14)	0.04	0.06
Malawi	iLiNS−DOSE (45)	431	146	48.6	0.00 (−0.08, 0.08)	0.06	0.08
Mali	PROMIS (46)	174	163	38.0	−0.03 (−0.13, 0.07)	0.04	0.05
Mali	PROMIS CS (46)						
Zimbabwe	SHINE (HIV−) (47)	296	325	38.8	−0.12 (−0.20, −0.04)	0.06	0.08
Zimbabwe	SHINE (HIV+) (48)	86	76	59.2	−0.14 (−0.29, 0.01)	0.02	0.03
		**4711**	**4369**		**I² = 0.46, Tau² = 0.00**		
**Fixed**					**−0.06 (−0.08, −0.04)**		
**Random**					**−0.05 (−0.08, −0.02)**		

Supplemental figure 9C: Stunting prevalence difference

9C3: Stratified by Household source water quality

P−for−interaction = 0.469
Difference in PDs = −0.01 (−0.04, 0.02)

Country	Trial	Improved LNS N	Improved Control N	Improved Control Prevalence	Improved PD (95% CI)	Improved Fixed W	Improved Random W	Unimproved LNS N	Unimproved Control N	Unimproved Control Prevalence	Unimproved PD (95% CI)	Unimproved Fixed W	Unimproved Random W
Bangladesh	JiVitA−4 (34)												
Bangladesh	RDNS (35)												
Bangladesh	WASH−B (36)	496	989	40.7	−0.09 (−0.14, −0.05)	0.17	0.16	70	114	43.0	0.00 (−0.15, 0.14)	0.03	0.05
Burkina Faso	iLiNS−Zinc (37)	550	141	35.5	−0.04 (−0.11, 0.02)	0.09	0.10	1394	519	40.2	−0.13 (−0.17, −0.08)	0.29	0.16
Burkina Faso	PROMIS (38)	355	379	31.1	−0.02 (−0.09, 0.04)	0.09	0.09	361	355	31.5	−0.12 (−0.22, −0.02)	0.05	0.08
Burkina Faso	PROMIS CS (38)	280	268	25.4	−0.06 (−0.14, 0.02)	0.06	0.06	149	171	22.8	0.01 (−0.08, 0.11)	0.06	0.09
Ghana	GHANA (39)												
Ghana	iLiNS−DYADG (40)												
Haiti	HAITI (41)												
Kenya	WASH−B (42)	447	1549	30.6	−0.01 (−0.07, 0.04)	0.14	0.13	248	697	33.1	−0.05 (−0.12, 0.02)	0.10	0.11
Madagascar	MAHAY (43)	419	453	51.4	0.01 (−0.08, 0.09)	0.05	0.06	1203	1180	61.2	−0.02 (−0.08, 0.03)	0.19	0.14
Malawi	iLiNS−DYADM (44)												
Malawi	iLiNS−DOSE (45)	555	193	46.1	−0.02 (−0.08, 0.05)	0.09	0.09	52	17	47.1	0.15 (−0.07, 0.38)	0.01	0.02
Mali	PROMIS (46)	249	258	36.4	−0.08 (−0.15, −0.01)	0.08	0.08	247	243	36.2	0.01 (−0.07, 0.09)	0.09	0.11
Mali	PROMIS CS (46)	549	566	31.4	−0.06 (−0.13, 0.00)	0.09	0.09	362	388	37.6	−0.08 (−0.16, −0.01)	0.09	0.11
Zimbabwe	SHINE (HIV−) (47)	521	489	35.2	−0.08 (−0.13, −0.02)	0.12	0.12	274	302	32.5	−0.01 (−0.09, 0.07)	0.08	0.10
Zimbabwe	SHINE (HIV+) (48)	85	87	49.4	−0.12 (−0.26, 0.02)	0.02	0.02	55	56	57.1	−0.14 (−0.34, 0.07)	0.01	0.03
		**4506**	**5372**		**I² = 0.12, Tau² = 0.00**			**4415**	**4042**		**I² = 0.59, Tau² = 0.00**		
**Fixed**					**−0.05 (−0.07, −0.03)**						**−0.06 (−0.08, −0.04)**		
**Random**					**−0.05 (−0.07, −0.03)**						**−0.05 (−0.08, −0.01)**		

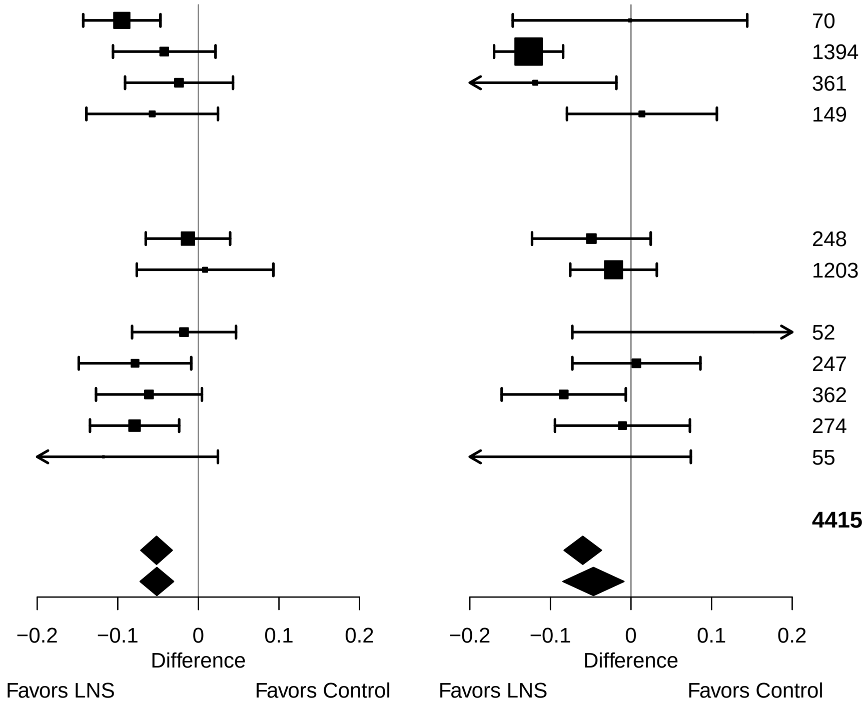

Supplemental figure 9C: Stunting prevalence difference

9C4: Stratified by Household sanitation

P−for−interaction = 0.310
Difference in PDs = 0.02 (−0.01, 0.05)

Country	Trial	Improved LNS N	Improved Control N	Improved Control Prevalence	Improved PD (95% CI)	Improved Fixed W	Improved Random W	Unimproved LNS N	Unimproved Control N	Unimproved Control Prevalence	Unimproved PD (95% CI)	Unimproved Fixed W	Unimproved Random W
Bangladesh	JiVitA−4 (34)	2189	950	42.5	−0.04 (−0.07, 0.00)	0.28	0.28	648	294	49.7	−0.03 (−0.09, 0.03)	0.13	0.13
Bangladesh	RDNS (35)	1179	573	39.6	−0.05 (−0.09, −0.01)	0.21	0.21	482	241	47.7	0.00 (−0.07, 0.08)	0.08	0.09
Bangladesh	WASH−B (36)	506	1001	39.7	−0.09 (−0.14, −0.03)	0.11	0.11	31	54	51.9	−0.13 (−0.34, 0.08)	0.01	0.01
Burkina Faso	iLiNS−Zinc (37)	43	16	37.5	−0.05 (−0.20, 0.10)	0.01	0.01	1901	644	39.2	−0.11 (−0.15, −0.06)	0.20	0.18
Burkina Faso	PROMIS (38)	398	550	31.3	−0.07 (−0.14, 0.00)	0.07	0.07	465	359	29.2	−0.04 (−0.10, 0.02)	0.10	0.11
Burkina Faso	PROMIS CS (38)	176	165	24.2	−0.03 (−0.13, 0.07)	0.03	0.03	252	274	24.5	−0.03 (−0.11, 0.05)	0.06	0.07
Ghana	GHANA (39)												
Ghana	iLiNS−DYADG (40)												
Haiti	HAITI (41)												
Kenya	WASH−B (42)	100	364	24.7	−0.03 (−0.11, 0.06)	0.05	0.05	595	1883	32.7	−0.03 (−0.08, 0.02)	0.16	0.15
Madagascar	MAHAY (43)												
Malawi	iLiNS−DYADM (44)												
Malawi	iLiNS−DOSE (45)												
Mali	PROMIS (46)	387	366	36.1	−0.06 (−0.12, 0.01)	0.07	0.07	112	135	37.8	0.01 (−0.10, 0.12)	0.03	0.04
Mali	PROMIS CS (46)	701	696	32.6	−0.08 (−0.14, −0.01)	0.08	0.08	220	248	35.9	−0.07 (−0.16, 0.02)	0.05	0.06
Zimbabwe	SHINE (HIV−) (47)	295	244	32.0	−0.06 (−0.13, 0.01)	0.06	0.06	497	546	35.2	−0.05 (−0.10, 0.00)	0.16	0.15
Zimbabwe	SHINE (HIV+) (48)	50	35	54.3	−0.16 (−0.36, 0.04)	0.01	0.01	90	107	51.4	−0.10 (−0.26, 0.05)	0.02	0.02
		**6024**	**4960**		**I² = 0.00, Tau² = 0.00**			**5293**	**4785**		**I² = 0.21, Tau² = 0.00**		
**Fixed**					**−0.05 (−0.07, −0.04)**						**−0.05 (−0.07, −0.03)**		
**Random**					**−0.05 (−0.07, −0.04)**						**−0.05 (−0.07, −0.03)**		

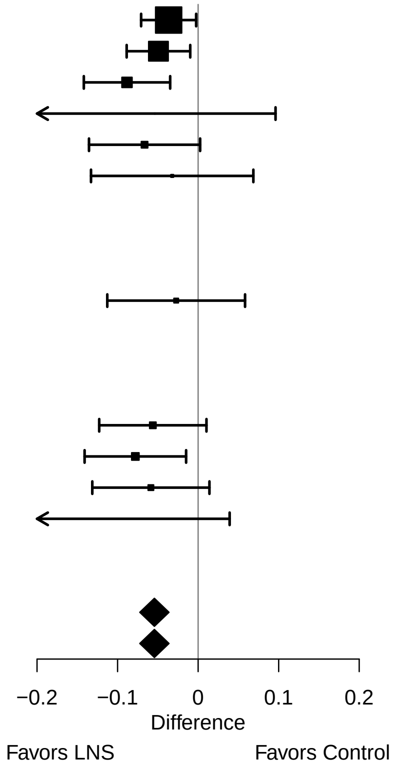

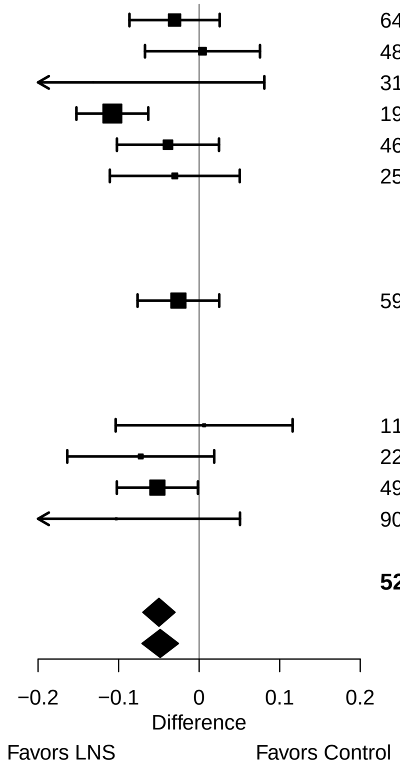

Supplemental figure 9C: Stunting prevalence difference

9C5: Stratified by Home environment

P−for−interaction = 0.228
Difference in PDs = −0.02 (−0.05, 0.01)

Country	Trial	At least median: LNS N	Control N	Control Prevalence	PD (95% CI)	Fixed W	Random W	Less than median: LNS N	Control N	Control Prevalence	PD (95% CI)	Fixed W	Random W
Bangladesh	JiVitA−4 (34)												
Bangladesh	RDNS (35)	1192	582	39.2	−0.02 (−0.07, 0.02)	0.17	0.15	468	230	48.7	−0.06 (−0.12, 0.00)	0.16	0.16
Bangladesh	WASH−B (36)	637	1653	36.9	−0.06 (−0.10, −0.02)	0.18	0.16	493	1690	46.9	−0.06 (−0.11, −0.01)	0.20	0.20
Burkina Faso	iLiNS−Zinc (37)	473	199	37.7	−0.12 (−0.18, −0.06)	0.09	0.11	271	174	41.7	−0.08 (−0.17, 0.00)	0.07	0.07
Burkina Faso	PROMIS (38)												
Burkina Faso	PROMIS CS (38)												
Ghana	GHANA (39)												
Ghana	iLiNS−DYADG (40)	213	409	12.0	−0.03 (−0.08, 0.03)	0.12	0.13	119	248	15.3	−0.07 (−0.14, 0.00)	0.09	0.09
Haiti	HAITI (41)												
Kenya	WASH−B (42)	835	2764	29.6	−0.03 (−0.07, 0.00)	0.27	0.18	574	2160	34.3	−0.05 (−0.09, 0.00)	0.26	0.26
Madagascar	MAHAY (43)	915	907	57.9	0.02 (−0.04, 0.09)	0.08	0.11	787	775	59.2	−0.05 (−0.11, 0.01)	0.13	0.13
Malawi	iLiNS−DYADM (44)	137	289	33.6	0.02 (−0.07, 0.12)	0.04	0.07	78	149	36.9	0.07 (−0.07, 0.20)	0.03	0.03
Malawi	iLiNS−DOSE (45)	375	136	49.3	−0.03 (−0.11, 0.04)	0.06	0.09	275	86	40.7	0.03 (−0.07, 0.12)	0.05	0.05
Mali	PROMIS (46)												
Mali	PROMIS CS (46)												
Zimbabwe	SHINE (HIV−) (47)												
Zimbabwe	SHINE (HIV+) (48)												
		**4777**	**6939**		**I² = 0.50, Tau² = 0.00**			**3065**	**5512**		**I² = 0.00, Tau² = 0.00**		
**Fixed**					**−0.04 (−0.05, −0.02)**						**−0.05 (−0.07, −0.03)**		
**Random**					**−0.03 (−0.06, −0.01)**						**−0.05 (−0.07, −0.03)**		

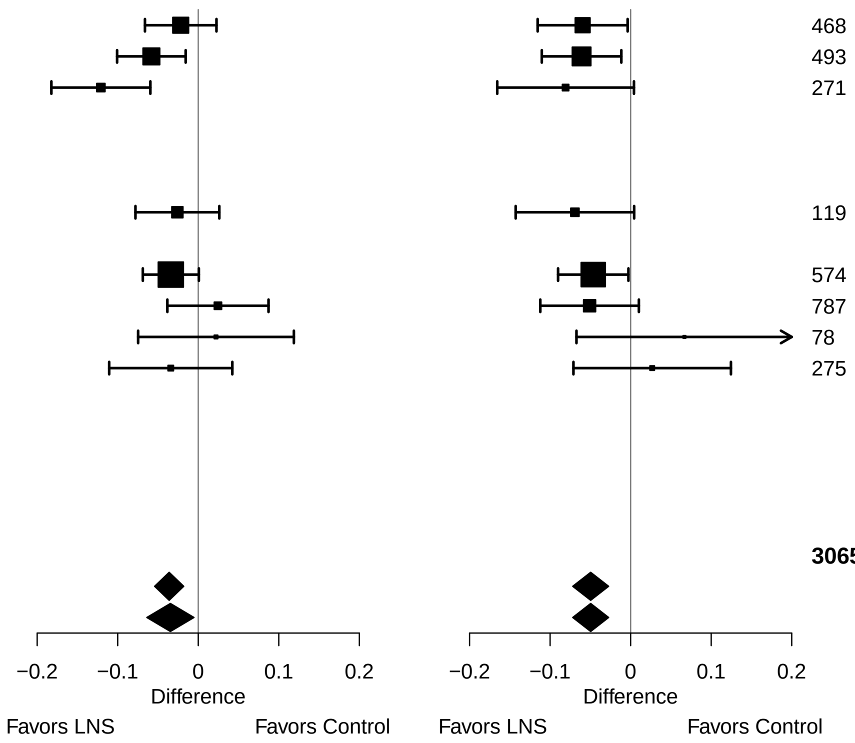

Supplemental figure 9C: Stunting prevalence difference

9C6: Stratified by Season at the time of assessment

P−for−interaction = 0.493
Difference in PDs = −0.01 (−0.03, 0.02)

Country	Trial	Dry LNS N	Dry Control N	Dry Control Prevalence	Dry PD (95% CI)	Dry Fixed W	Dry Random W	Rainy LNS N	Rainy Control N	Rainy Control Prevalence	Rainy PD (95% CI)	Rainy Fixed W	Rainy Random W
Bangladesh	JiVitA−4 (34)	1550	663	44.9	−0.03 (−0.07, 0.01)	0.15	0.12	1288	581	43.4	−0.04 (−0.09, 0.00)	0.13	0.13
Bangladesh	RDNS (35)	739	359	43.7	−0.04 (−0.10, 0.02)	0.06	0.08	924	456	40.6	−0.03 (−0.07, 0.01)	0.17	0.17
Bangladesh	WASH−B (36)	640	1913	39.1	−0.06 (−0.10, −0.01)	0.13	0.11	518	1518	45.5	−0.08 (−0.13, −0.04)	0.14	0.14
Burkina Faso	iLiNS−Zinc (37)	1394	477	40.3	−0.10 (−0.14, −0.07)	0.14	0.11	558	187	37.2	−0.11 (−0.20, −0.03)	0.04	0.04
Burkina Faso	PROMIS (38)	434	446	27.1	−0.04 (−0.11, 0.02)	0.05	0.07	429	468	33.8	−0.06 (−0.13, 0.00)	0.06	0.06
Burkina Faso	PROMIS CS (38)	224	190	25.3	−0.05 (−0.15, 0.05)	0.02	0.04	206	249	23.7	−0.01 (−0.12, 0.10)	0.02	0.02
Ghana	GHANA (39)												
Ghana	iLiNS−DYADG (40)	204	407	12.8	−0.03 (−0.08, 0.02)	0.07	0.09	143	285	13.3	−0.06 (−0.13, 0.00)	0.07	0.07
Haiti	HAITI (41)												
Kenya	WASH−B (42)	347	1151	30.1	−0.06 (−0.11, 0.00)	0.07	0.09	1110	3986	32.8	−0.04 (−0.07, −0.01)	0.29	0.29
Madagascar	MAHAY (43)												
Malawi	iLiNS−DYADM (44)	156	316	32.9	0.04 (−0.05, 0.13)	0.02	0.05	64	128	39.1	0.02 (−0.13, 0.16)	0.01	0.01
Malawi	iLiNS−DOSE (45)	415	134	44.0	0.03 (−0.04, 0.11)	0.04	0.06	281	107	49.5	−0.08 (−0.17, 0.01)	0.03	0.03
Mali	PROMIS (46)												
Mali	PROMIS CS (46)												
Zimbabwe	SHINE (HIV−) (47)	1747	1662	35.4	−0.08 (−0.11, −0.05)	0.22	0.13	133	132	28.0	0.01 (−0.10, 0.13)	0.02	0.02
Zimbabwe	SHINE (HIV+) (48)	319	306	50.7	−0.10 (−0.18, −0.01)	0.03	0.05	18	24	41.7	−0.14 (−0.42, 0.14)	0.00	0.00
		**8169**	**8024**		**I² = 0.51, Tau² = 0.00**			**5672**	**8121**		**I² = 0.00, Tau² = 0.00**		
**Fixed**					**−0.06 (−0.07, −0.04)**						**−0.05 (−0.07, −0.03)**		
**Random**					**−0.05 (−0.07, −0.03)**						**−0.05 (−0.07, −0.03)**		

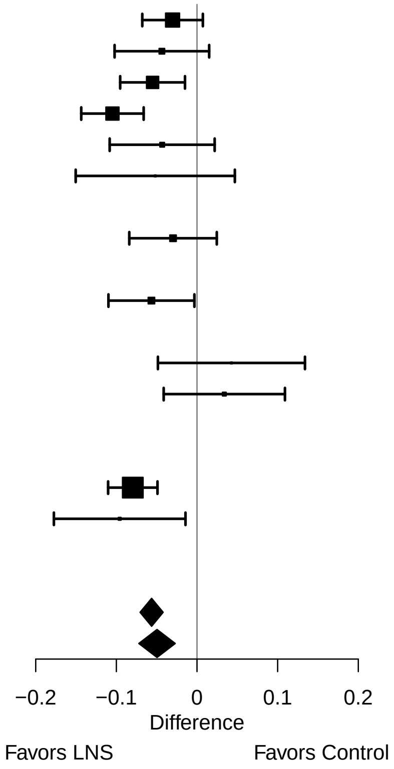

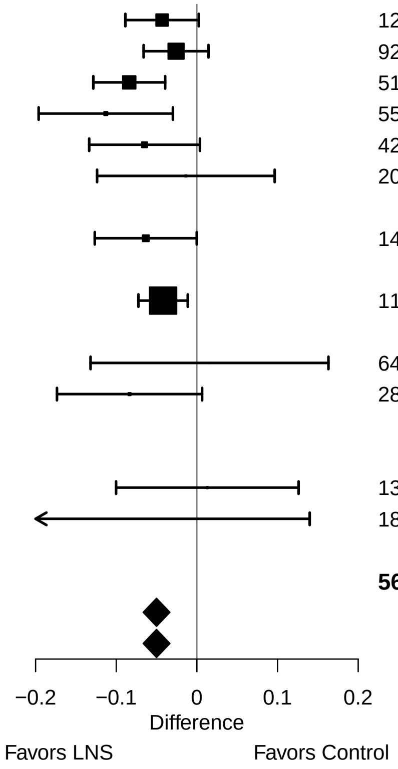

Supplemental figure 9D: Mean difference in WLZ

9D1: Stratified by Household socio-economic status

P−for−interaction = 0.814
Difference in MDs = 0.00 (−0.03, 0.04)

		At least median							Less than median					
**Country**	**Trial**	**LNS N**	**Control N**	**Control Mean**	**MD (95% CI)**	**Fixed W**	**Random W**	**LNS N**	**Control N**	**Control Mean**	**MD (95% CI)**	**Fixed W**	**Random W**
Bangladesh	JiVitA−4 (34)	1401	607	−1.10	0.10 (0.04, 0.16)	0.22	0.15	1381	610	−1.24	0.06 (−0.01, 0.12)	0.20	0.13
Bangladesh	RDNS (35)	834	407	−1.01	0.04 (−0.07, 0.15)	0.07	0.08	827	408	−1.21	0.10 (0.01, 0.19)	0.10	0.10
Bangladesh	WASH−B (36)	579	1714	−0.72	0.12 (0.02, 0.21)	0.09	0.09	577	1710	−1.02	0.13 (0.04, 0.22)	0.10	0.10
Burkina Faso	iLiNS−Zinc (37)	1225	299	−0.90	0.19 (0.09, 0.29)	0.08	0.09	720	364	−0.97	0.16 (0.09, 0.23)	0.15	0.12
Burkina Faso	PROMIS (38)	405	479	−0.66	0.05 (−0.07, 0.17)	0.06	0.06	451	428	−0.77	0.16 (−0.04, 0.35)	0.02	0.03
Burkina Faso	PROMIS CS (38)	220	208	−0.91	−0.06 (−0.25, 0.13)	0.02	0.03	210	228	−0.86	−0.16 (−0.34, 0.02)	0.03	0.04
Ghana	GHANA (39)	57	44	−0.41	0.06 (−0.46, 0.57)	0.00	0.00	41	46	−0.84	0.30 (−0.15, 0.74)	0.00	0.01
Ghana	iLiNS−DYADG (40)	157	356	−0.53	0.10 (−0.09, 0.29)	0.02	0.03	190	334	−0.65	0.10 (−0.08, 0.28)	0.03	0.04
Haiti	HAITI (41)	74	60	0.23	−0.04 (−0.28, 0.20)	0.01	0.02	61	72	−0.22	0.15 (−0.07, 0.37)	0.02	0.03
Kenya	WASH−B (42)	825	2914	0.13	0.07 (−0.01, 0.15)	0.13	0.11	628	2200	0.05	0.06 (−0.03, 0.15)	0.11	0.10
Madagascar	MAHAY (43)	771	884	−0.31	0.01 (−0.12, 0.13)	0.05	0.06	876	755	−0.56	0.04 (−0.10, 0.19)	0.04	0.05
Malawi	iLiNS−DYADM (44)	121	234	−0.12	−0.04 (−0.24, 0.17)	0.02	0.03	99	209	−0.14	−0.08 (−0.33, 0.18)	0.01	0.02
Malawi	iLiNS−DOSE (45)	301	104	−0.13	−0.04 (−0.21, 0.13)	0.03	0.04	294	103	−0.38	0.11 (−0.10, 0.32)	0.02	0.03
Mali	PROMIS (46)	246	250	−0.32	0.13 (−0.01, 0.27)	0.04	0.05	245	246	−0.32	0.06 (−0.09, 0.22)	0.03	0.05
Mali	PROMIS CS (46)	501	458	−0.77	0.17 (0.04, 0.30)	0.05	0.06	443	501	−0.77	0.15 (0.01, 0.29)	0.04	0.05
Zimbabwe	SHINE (HIV−) (47)	903	797	0.08	0.04 (−0.07, 0.14)	0.08	0.08	826	856	−0.06	0.15 (0.06, 0.25)	0.09	0.09
Zimbabwe	SHINE (HIV+) (48)	171	156	0.06	−0.16 (−0.42, 0.09)	0.01	0.02	157	166	−0.03	−0.11 (−0.38, 0.15)	0.01	0.02
		**8791**	**9971**		**I² = 0.23, Tau² = 0.00**			**8026**	**9236**		**I² = 0.27, Tau² = 0.00**		
**Fixed**					**0.08 (0.05, 0.11)**						**0.10 (0.07, 0.12)**		
**Random**					**0.07 (0.03, 0.11)**						**0.09 (0.05, 0.13)**		

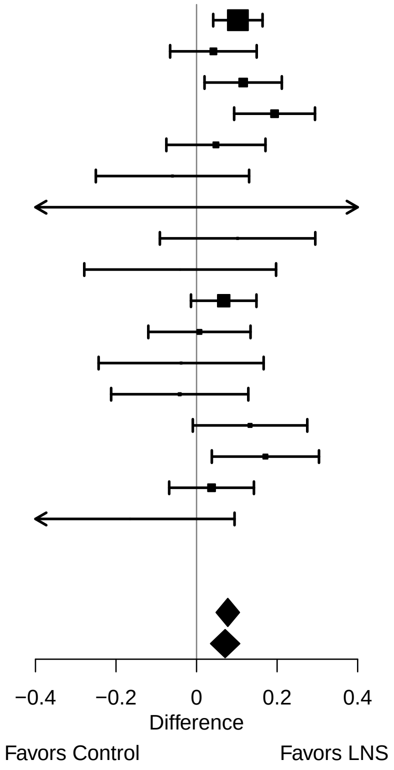

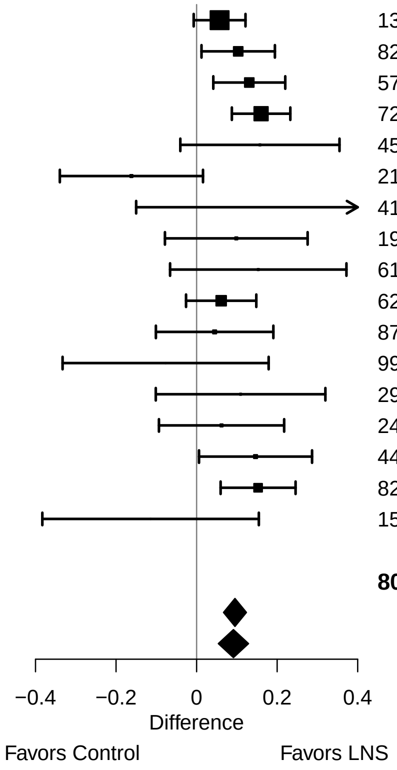

Supplemental figure 9D: Mean difference in WLZ

9D2: Stratified by Household food insecurity

P−for−interaction = 0.813
Difference in MDs = 0.01 (−0.04, 0.05)

Country	Trial	Mild to secure: LNS N	Control N	Control Mean	MD (95% CI)	Fixed W	Random W	Moderate to severe: LNS N	Control N	Control Mean	MD (95% CI)	Fixed W	Random W
Bangladesh	JiVitA−4 (34)	1977	841	−1.14	0.09 (0.03, 0.14)	0.23	0.18	805	376	−1.22	0.06 (−0.02, 0.14)	0.25	0.19
Bangladesh	RDNS (35)	1057	493	−1.02	0.01 (−0.09, 0.11)	0.08	0.09	604	322	−1.24	0.16 (0.04, 0.28)	0.11	0.11
Bangladesh	WASH−B (36)	917	2647	−0.84	0.13 (0.06, 0.21)	0.13	0.13	239	777	−1.01	0.07 (−0.06, 0.19)	0.10	0.10
Burkina Faso	iLiNS−Zinc (37)	1013	320	−0.87	0.16 (0.07, 0.25)	0.09	0.10	932	343	−1.00	0.21 (0.12, 0.30)	0.19	0.17
Burkina Faso	PROMIS (38)	501	548	−0.72	0.14 (0.00, 0.29)	0.04	0.05	355	359	−0.70	0.04 (−0.17, 0.24)	0.03	0.04
Burkina Faso	PROMIS CS (38)												
Ghana	GHANA (39)												
Ghana	iLiNS−DYADG (40)	244	470	−0.57	0.09 (−0.07, 0.25)	0.03	0.04	102	219	−0.61	0.09 (−0.13, 0.31)	0.03	0.04
Haiti	HAITI (41)												
Kenya	WASH−B (42)	1297	4577	0.09	0.07 (0.01, 0.14)	0.18	0.15	156	524	0.10	−0.02 (−0.21, 0.17)	0.04	0.05
Madagascar	MAHAY (43)	987	927	−0.41	0.03 (−0.10, 0.15)	0.04	0.06	354	410	−0.48	0.02 (−0.16, 0.19)	0.05	0.06
Malawi	iLiNS−DYADM (44)	70	122	0.02	−0.16 (−0.45, 0.13)	0.01	0.01	148	316	−0.19	−0.01 (−0.21, 0.18)	0.04	0.05
Malawi	iLiNS−DOSE (45)	151	55	−0.26	0.05 (−0.21, 0.31)	0.01	0.02	431	146	−0.28	0.06 (−0.10, 0.22)	0.06	0.07
Mali	PROMIS (46)	326	338	−0.33	0.12 (−0.01, 0.25)	0.04	0.05	170	163	−0.34	0.08 (−0.13, 0.29)	0.03	0.04
Mali	PROMIS CS (46)												
Zimbabwe	SHINE (HIV−) (47)	1384	1297	0.03	0.08 (0.00, 0.17)	0.10	0.11	294	324	−0.07	0.17 (0.01, 0.33)	0.06	0.07
Zimbabwe	SHINE (HIV+) (48)	236	238	−0.02	−0.12 (−0.33, 0.09)	0.02	0.02	85	76	0.09	−0.18 (−0.54, 0.17)	0.01	0.02
		**10160**	**12873**		**I² = 0.18, Tau² = 0.00**			**4675**	**4355**		**I² = 0.23, Tau² = 0.00**		
**Fixed**					**0.09 (0.06, 0.11)**						**0.10 (0.06, 0.13)**		
**Random**					**0.08 (0.05, 0.12)**						**0.09 (0.05, 0.14)**		

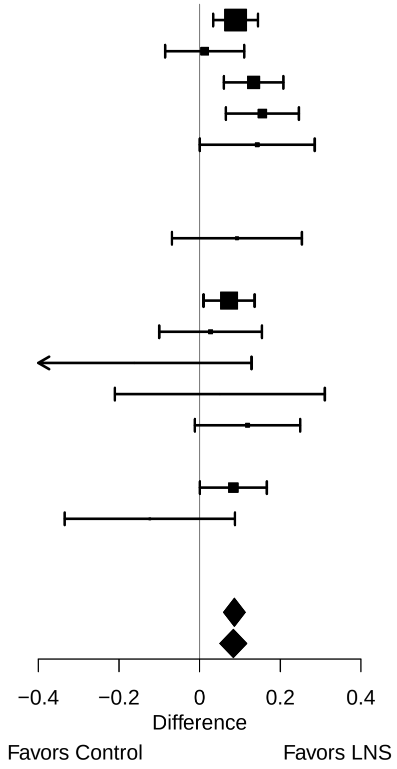

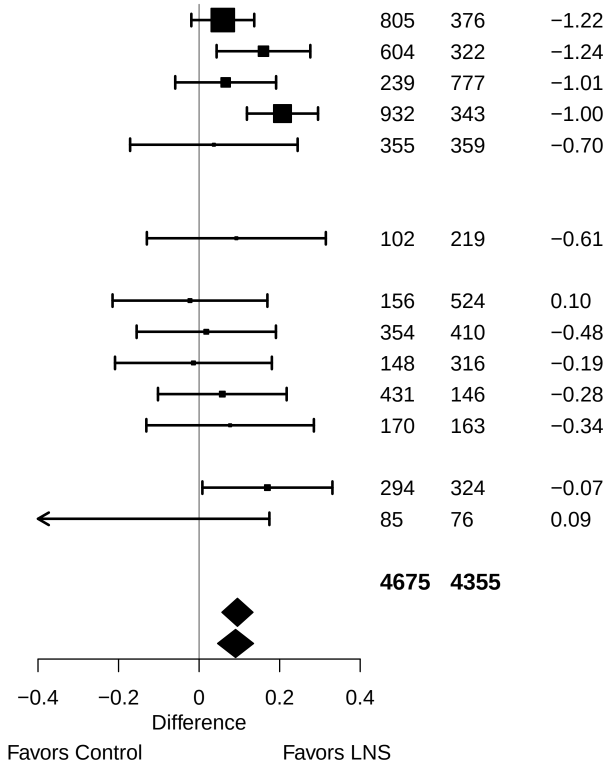

Supplemental figure 9D: Mean difference in WLZ

9D3: Stratified by Household source water quality

P−for−interaction = 0.860
Difference in MDs = 0.01 (−0.05, 0.07)

		Improved						Unimproved					
**Country**	**Trial**	**LNS N**	**Control N**	**Control Mean**	**MD (95% CI)**	**Fixed W**	**Random W**	**LNS N**	**Control N**	**Control Mean**	**MD (95% CI)**	**Fixed W**	**Random W**
Bangladesh	JiVitA−4 (34)												
Bangladesh	RDNS (35)												
Bangladesh	WASH−B (36)	495	986	−0.87	0.19 (0.07, 0.31)	0.11	0.10	70	114	−0.90	−0.06 (−0.36, 0.25)	0.02	0.04
Burkina Faso	iLiNS−Zinc (37)	550	141	−0.89	0.16 (0.05, 0.26)	0.15	0.12	1394	519	−0.96	0.19 (0.12, 0.27)	0.36	0.21
Burkina Faso	PROMIS (38)	352	377	−0.71	0.03 (−0.13, 0.19)	0.06	0.07	357	350	−0.70	0.16 (−0.02, 0.35)	0.06	0.08
Burkina Faso	PROMIS CS (38)	280	267	−0.91	−0.09 (−0.30, 0.11)	0.04	0.05	149	169	−0.83	−0.13 (−0.31, 0.04)	0.07	0.09
Ghana	GHANA (39)												
Ghana	iLiNS−DYADG (40)												
Haiti	HAITI (41)												
Kenya	WASH−B (42)	447	1542	0.12	0.04 (−0.06, 0.14)	0.15	0.12	247	695	0.06	0.03 (−0.11, 0.17)	0.11	0.12
Madagascar	MAHAY (43)	418	453	−0.36	0.00 (−0.17, 0.18)	0.05	0.06	1202	1180	−0.45	0.01 (−0.11, 0.12)	0.15	0.15
Malawi	iLiNS−DYADM (44)	200	406	−0.12	−0.06 (−0.23, 0.10)	0.06	0.07	20	36	−0.28	0.07 (−0.61, 0.74)	0.00	0.01
Malawi	iLiNS−DOSE (45)	555	193	−0.24	0.04 (−0.10, 0.18)	0.08	0.09	52	17	−0.42	−0.03 (−0.45, 0.39)	0.01	0.02
Mali	PROMIS (46)	247	257	−0.26	0.13 (−0.01, 0.27)	0.08	0.09	242	240	−0.41	0.08 (−0.11, 0.26)	0.06	0.08
Mali	PROMIS CS (46)	544	562	−0.74	0.15 (0.04, 0.25)	0.14	0.12	361	382	−0.79	0.17 (0.00, 0.34)	0.07	0.09
Zimbabwe	SHINE (HIV−) (47)	518	486	0.01	0.08 (−0.06, 0.22)	0.08	0.09	273	301	0.10	0.00 (−0.17, 0.17)	0.07	0.09
Zimbabwe	SHINE (HIV+) (48)	85	87	−0.01	−0.13 (−0.45, 0.18)	0.02	0.02	55	56	−0.02	−0.11 (−0.55, 0.34)	0.01	0.02
		**4691**	**5757**		**I² = 0.35, Tau² = 0.00**			**4422**	**4059**		**I² = 0.47, Tau² = 0.00**		
**Fixed**					**0.08 (0.04, 0.12)**						**0.09 (0.04, 0.13)**		
**Random**					**0.07 (0.02, 0.12)**						**0.06 (0.00, 0.13)**		

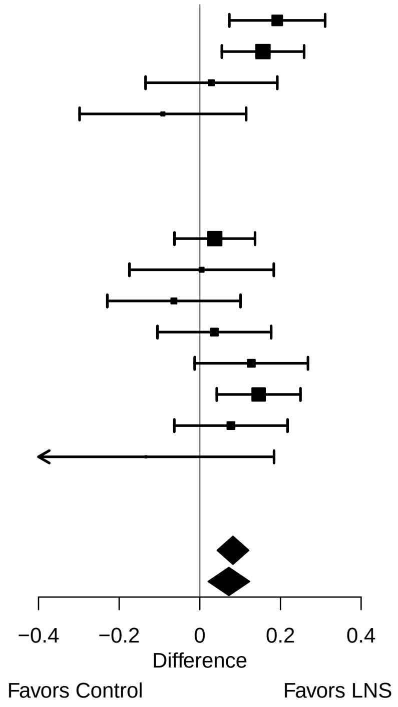

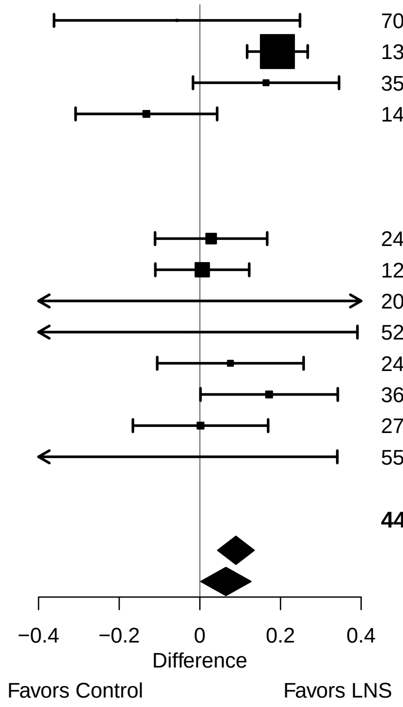

Supplemental figure 9D: Mean difference in WLZ

9D4: Stratified by Household sanitation

P−for−interaction = 0.062
Difference in MDs = −0.05 (−0.11, 0.00)

		Improved						Unimproved					
**Country**	**Trial**	**LNS N**	**Control N**	**Control Mean**	**MD (95% CI)**	**Fixed W**	**Random W**	**LNS N**	**Control N**	**Control Mean**	**MD (95% CI)**	**Fixed W**	**Random W**
Bangladesh	JiVitA−4 (34)	2151	932	−1.15	0.10 (0.05, 0.15)	0.41	0.24	631	285	−1.23	0.01 (−0.08, 0.09)	0.15	0.13
Bangladesh	RDNS (35)	1179	573	−1.10	0.09 (0.00, 0.18)	0.13	0.14	480	241	−1.15	0.03 (−0.11, 0.16)	0.07	0.08
Bangladesh	WASH−B (36)	506	998	−0.85	0.15 (0.03, 0.27)	0.08	0.10	30	54	−0.98	0.31 (−0.08, 0.71)	0.01	0.02
Burkina Faso	iLiNS−Zinc (37)	43	16	−1.00	0.25 (−0.06, 0.56)	0.01	0.02	1901	644	−0.94	0.18 (0.11, 0.26)	0.24	0.15
Burkina Faso	PROMIS (38)	392	548	−0.74	0.12 (−0.01, 0.24)	0.07	0.09	464	355	−0.67	0.07 (−0.11, 0.24)	0.04	0.06
Burkina Faso	PROMIS CS (38)	176	165	−0.90	−0.09 (−0.32, 0.13)	0.02	0.03	252	271	−0.87	−0.12 (−0.28, 0.04)	0.05	0.07
Ghana	GHANA (39)												
Ghana	iLiNS−DYADG (40)	336	671	−0.58	0.09 (−0.05, 0.22)	0.06	0.08	10	19	−0.72	0.33 (−0.57, 1.23)	0.00	0.00
Haiti	HAITI (41)												
Kenya	WASH−B (42)	100	364	0.18	0.12 (−0.05, 0.30)	0.04	0.05	594	1874	0.08	0.02 (−0.07, 0.11)	0.17	0.13
Madagascar	MAHAY (43)												
Malawi	iLiNS−DYADM (44)	20	42	−0.16	0.22 (−0.20, 0.65)	0.01	0.01	200	400	−0.13	−0.08 (−0.25, 0.09)	0.04	0.06
Malawi	iLiNS−DOSE (45)	13	9	−0.42	−0.45 (−1.07, 0.16)	0.00	0.00	593	201	−0.24	0.04 (−0.09, 0.18)	0.07	0.08
Mali	PROMIS (46)	380	363	−0.32	0.08 (−0.06, 0.21)	0.06	0.08	112	134	−0.38	0.18 (−0.02, 0.38)	0.03	0.05
Mali	PROMIS CS (46)	696	692	−0.78	0.18 (0.07, 0.30)	0.08	0.10	217	242	−0.73	0.11 (−0.05, 0.26)	0.05	0.07
Zimbabwe	SHINE (HIV−) (47)	293	243	0.05	0.10 (−0.09, 0.29)	0.03	0.04	495	543	0.04	0.02 (−0.11, 0.15)	0.07	0.08
Zimbabwe	SHINE (HIV+) (48)	50	35	0.10	−0.43 (−0.95, 0.09)	0.00	0.01	90	107	−0.05	0.02 (−0.28, 0.32)	0.01	0.02
		**6335**	**5651**		**I² = 0.08, Tau² = 0.00**			**6069**	**5370**		**I² = 0.50, Tau² = 0.00**		
**Fixed**					**0.11 (0.07, 0.14)**						**0.06 (0.03, 0.10)**		
**Random**					**0.10 (0.06, 0.15)**						**0.05 (0.00, 0.10)**		

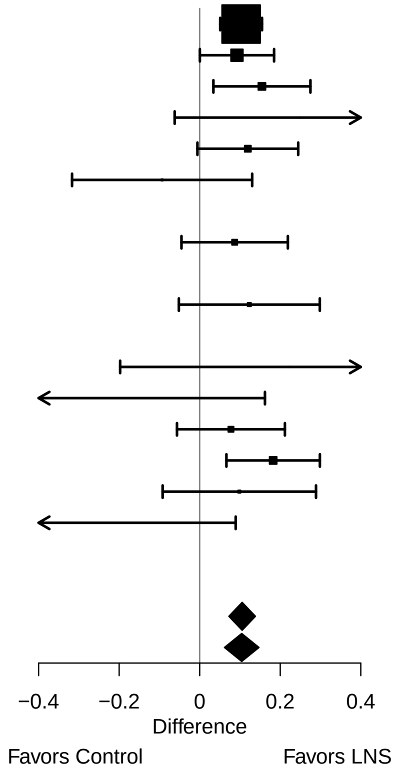

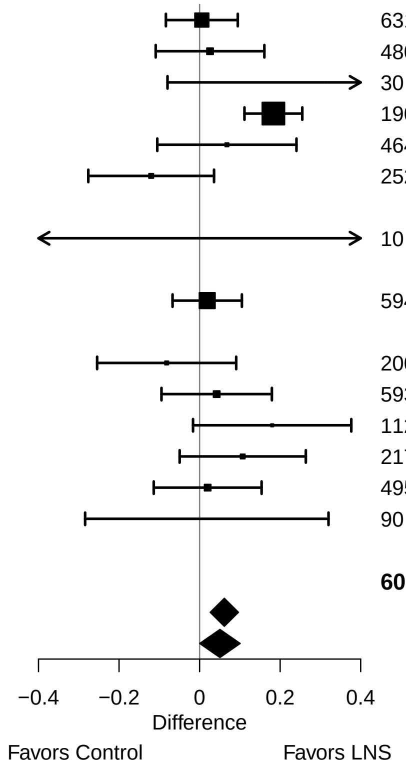

Supplemental figure 9D: Mean difference in WLZ

9D5: Stratified by Home environment

P−for−interaction = 0.043
Difference in MDs = 0.06 (0.00, 0.11)

		At least median							Less than median						
**Country**	**Trial**	**LNS N**	**Control N**	**Control Mean**	**MD (95% CI)**	**Fixed W**	**Random W**		**LNS N**	**Control N**	**Control Mean**	**MD (95% CI)**	**Fixed W**	**Random W**
Bangladesh	JiVitA−4 (34)													
Bangladesh	RDNS (35)	1192	582	−1.08	0.07 (−0.02, 0.17)	0.15	0.15		466	230	−1.17	0.07 (−0.10, 0.23)	0.09	0.11
Bangladesh	WASH−B (36)	636	1652	−0.79	0.13 (0.03, 0.23)	0.15	0.15		492	1684	−0.95	0.07 (−0.03, 0.18)	0.23	0.22
Burkina Faso	iLiNS−Zinc (37)	473	199	−0.91	0.20 (0.09, 0.30)	0.13	0.14		271	174	−1.08	0.20 (0.03, 0.36)	0.09	0.10
Burkina Faso	PROMIS (38)													
Burkina Faso	PROMIS CS (38)													
Ghana	GHANA (39)													
Ghana	iLiNS−DYADG (40)	213	409	−0.56	0.06 (−0.10, 0.23)	0.05	0.08		119	248	−0.67	0.23 (0.00, 0.45)	0.05	0.06
Haiti	HAITI (41)													
Kenya	WASH−B (42)	834	2757	0.13	−0.02 (−0.09, 0.05)	0.30	0.19		573	2149	0.07	0.14 (0.06, 0.22)	0.35	0.28
Madagascar	MAHAY (43)	914	907	−0.36	0.00 (−0.11, 0.11)	0.12	0.14		786	775	−0.49	0.00 (−0.15, 0.14)	0.11	0.13
Malawi	iLiNS−DYADM (44)	137	289	−0.08	−0.07 (−0.27, 0.13)	0.03	0.06		78	149	−0.25	−0.03 (−0.30, 0.25)	0.03	0.04
Malawi	iLiNS−DOSE (45)	375	136	−0.27	0.10 (−0.06, 0.25)	0.06	0.09		275	86	−0.23	−0.06 (−0.29, 0.16)	0.05	0.06
Mali	PROMIS (46)													
Mali	PROMIS CS (46)													
Zimbabwe	SHINE (HIV−) (47)													
Zimbabwe	SHINE (HIV+) (48)													
		**4774**	**6931**		**I² = 0.59, Tau² = 0.00**				**3060**	**5495**		**I² = 0.18, Tau² = 0.00**		
**Fixed**					**0.06 (0.02, 0.09)**							**0.10 (0.05, 0.15)**		
**Random**					**0.06 (0.01, 0.12)**							**0.09 (0.03, 0.15)**		

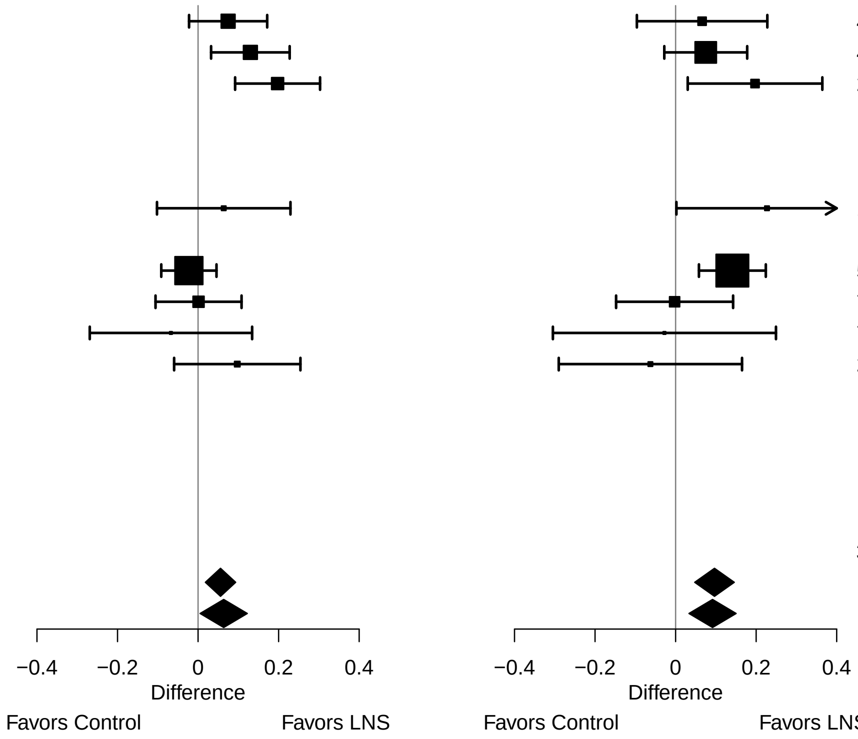

Supplemental figure 9D: Mean difference in WLZ

9D6: Stratified by Season at the time of assessment

P−for−interaction = 0.197
Difference in MDs = −0.03 (−0.08, 0.02)

Country	Trial	Dry LNS N	Dry Control N	Dry Control Mean	Dry MD (95% CI)	Dry Fixed W	Dry Random W	Rainy LNS N	Rainy Control N	Rainy Control Mean	Rainy MD (95% CI)	Rainy Fixed W	Rainy Random W
Bangladesh	JiVitA−4 (34)	1520	649	−1.09	0.10 (0.03, 0.16)	0.27	0.11	1263	568	−1.26	0.06 (−0.01, 0.13)	0.25	0.17
Bangladesh	RDNS (35)	738	359	−1.05	0.11 (−0.02, 0.23)	0.06	0.09	923	456	−1.16	0.04 (−0.05, 0.14)	0.12	0.13
Bangladesh	WASH−B (36)	639	1912	−0.75	0.13 (0.03, 0.23)	0.10	0.09	517	1512	−1.03	0.12 (0.03, 0.21)	0.14	0.14
Burkina Faso	iLiNS−Zinc (37)	1394	477	−0.97	0.24 (0.14, 0.34)	0.11	0.10	558	187	−0.88	0.05 (−0.07, 0.17)	0.08	0.10
Burkina Faso	PROMIS (38)	428	445	−0.64	0.06 (−0.11, 0.23)	0.04	0.07	428	462	−0.78	0.13 (−0.02, 0.28)	0.05	0.07
Burkina Faso	PROMIS CS (38)	224	190	−0.80	−0.14 (−0.36, 0.08)	0.02	0.06	206	246	−0.95	−0.11 (−0.29, 0.07)	0.03	0.06
Ghana	GHANA (39)	57	55	−0.84	0.52 (0.12, 0.92)	0.01	0.03	41	41	−0.37	−0.22 (−0.78, 0.35)	0.00	0.01
Ghana	iLiNS−DYADG (40)	204	407	−0.62	−0.03 (−0.20, 0.13)	0.04	0.07	143	285	−0.54	0.27 (0.06, 0.49)	0.02	0.04
Haiti	HAITI (41)												
Kenya	WASH−B (42)	346	1146	0.03	0.07 (−0.06, 0.19)	0.07	0.09	1109	3972	0.11	0.07 (−0.01, 0.14)	0.23	0.16
Madagascar	MAHAY (43)												
Malawi	iLiNS−DYADM (44)	156	316	−0.07	−0.03 (−0.22, 0.16)	0.03	0.06	64	128	−0.29	−0.12 (−0.42, 0.18)	0.01	0.02
Malawi	iLiNS−DOSE (45)	415	134	−0.17	0.00 (−0.17, 0.17)	0.03	0.07	281	107	−0.30	0.01 (−0.17, 0.20)	0.03	0.06
Mali	PROMIS (46)												
Mali	PROMIS CS (46)												
Zimbabwe	SHINE (HIV−) (47)	1736	1655	0.00	0.10 (0.02, 0.17)	0.19	0.10	133	129	0.24	−0.19 (−0.47, 0.10)	0.01	0.03
Zimbabwe	SHINE (HIV+) (48)	318	304	0.03	−0.16 (−0.34, 0.03)	0.03	0.07	18	24	−0.23	0.02 (−0.59, 0.63)	0.00	0.01
		**8175**	**8049**		**I^2 = 0.60, Tau^2 = 0.01**			**5684**	**8117**		**I^2 = 0.22, Tau^2 = 0.00**		
**Fixed**					**0.09 (0.06, 0.12)**						**0.06 (0.03, 0.09)**		
**Random**					**0.07 (−0.01, 0.14)**						**0.05 (0.01, 0.10)**		

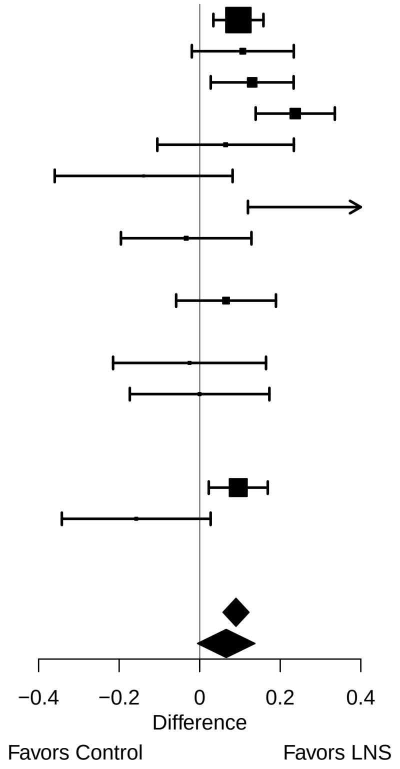

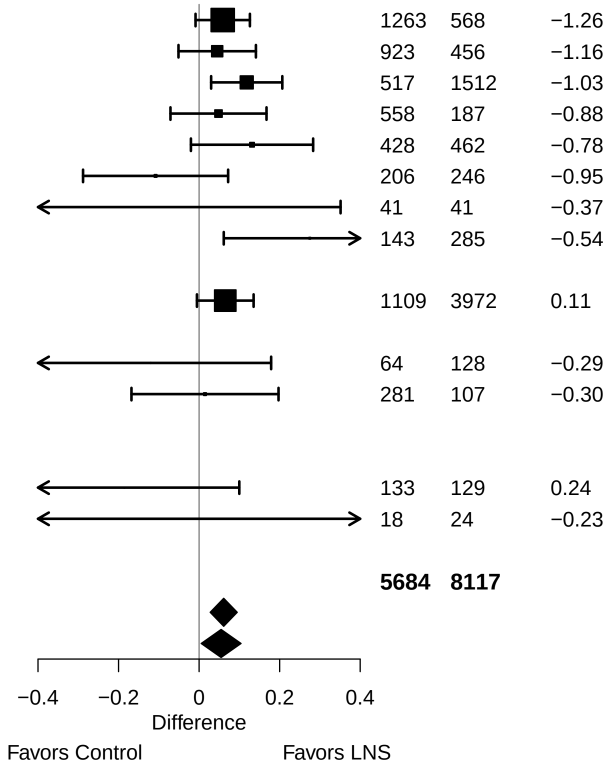

Supplemental figure 9E: Wasting prevalence ratio

9E1: Stratified by Household socio-economic status

P−for−interaction = 0.492
Ratio of PRs = 1.06 (0.90, 1.24)

		At least median						Less than median					
**Country**	**Trial**	**LNS N**	**Control N**	**Control Prevalence**	**PR (95% CI)**	**Fixed W**	**Random W**	**LNS N**	**Control N**	**Control Prevalence**	**PR (95% CI)**	**Fixed W**	**Random W**
Bangladesh	JiVitA−4 (34)	1401	607	13.6	0.84 (0.67, 1.06)	0.29	0.29	1381	610	19.2	0.95 (0.78, 1.15)	0.28	0.28
Bangladesh	RDNS (35)	834	407	13.3	0.88 (0.63, 1.22)	0.13	0.13	827	408	16.9	0.86 (0.65, 1.13)	0.14	0.14
Bangladesh	WASH−B (36)	579	1714	8.7	0.75 (0.53, 1.08)	0.12	0.12	577	1710	13.7	0.81 (0.63, 1.04)	0.17	0.17
Burkina Faso	iLiNS−Zinc (37)	1225	299	12.4	0.58 (0.40, 0.84)	0.11	0.11	720	364	14.2	0.86 (0.62, 1.17)	0.11	0.11
Burkina Faso	PROMIS (38)	405	479	7.7	0.86 (0.47, 1.58)	0.04	0.04	451	428	10.3	0.84 (0.51, 1.38)	0.04	0.04
Burkina Faso	PROMIS CS (38)	220	208	13.0	0.77 (0.45, 1.31)	0.05	0.05	210	228	11.8	1.33 (0.83, 2.11)	0.05	0.05
Ghana	GHANA (39)												
Ghana	iLiNS−DYADG (40)	157	356	6.7	0.94 (0.46, 1.93)	0.03	0.03	190	334	8.4	0.75 (0.39, 1.45)	0.03	0.03
Haiti	HAITI (41)												
Kenya	WASH−B (42)	825	2914	1.2	1.01 (0.52, 1.96)	0.03	0.03	628	2200	1.9	0.77 (0.40, 1.49)	0.02	0.02
Madagascar	MAHAY (43)	771	884	4.1	1.05 (0.60, 1.84)	0.05	0.05	876	755	7.9	0.85 (0.54, 1.34)	0.05	0.05
Malawi	iLiNS−DYADM (44)												
Malawi	iLiNS−DOSE (45)												
Mali	PROMIS (46)												
Mali	PROMIS CS (46)	501	458	10.5	0.84 (0.56, 1.25)	0.09	0.09	443	501	10.2	0.82 (0.57, 1.18)	0.08	0.08
Zimbabwe	SHINE (HIV−) (47)	903	797	2.4	1.07 (0.62, 1.85)	0.05	0.05	826	856	2.9	0.66 (0.34, 1.30)	0.02	0.02
Zimbabwe	SHINE (HIV+) (48)												
		**7821**	**9123**		**I² = 0.00, Tau² = 0.00**			**7129**	**8394**		**I² = 0.00, Tau² = 0.00**		
**Fixed**					**0.83 (0.73, 0.93)**						**0.88 (0.79, 0.97)**		
**Random**					**0.83 (0.73, 0.93)**						**0.88 (0.79, 0.97)**		

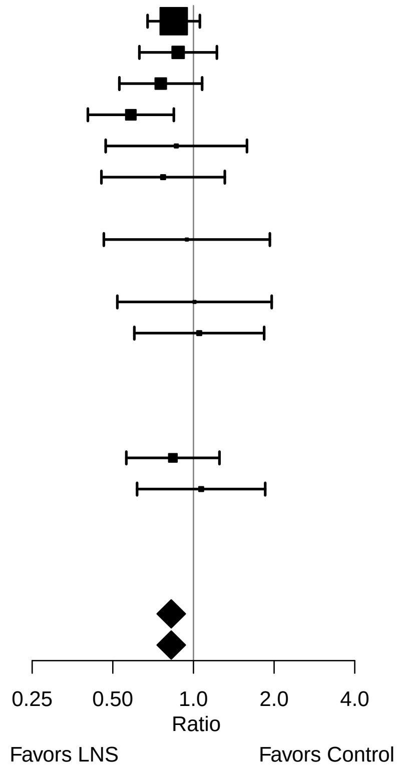

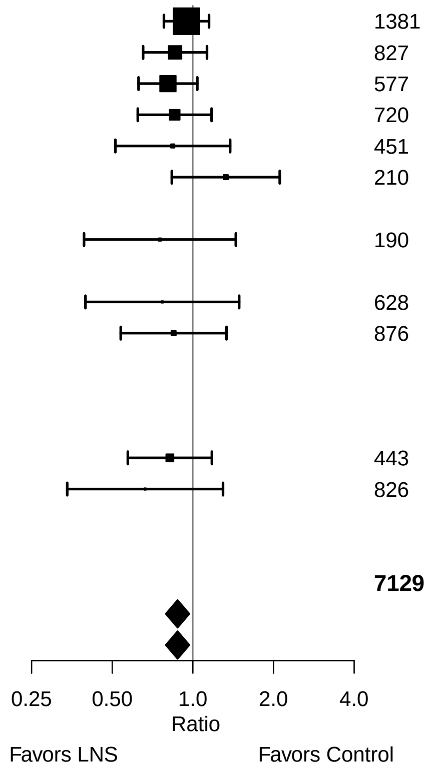

Supplemental figure 9E: Wasting prevalence ratio

9E2: Stratified by Household food insecurity

P−for−interaction = 0.729
Ratio of PRs = 0.97 (0.81, 1.16)

		Mild to secure						Moderate to severe					
**Country**	**Trial**	**LNS N**	**Control N**	**Control Prevalence**	**PR (95% CI)**	**Fixed W**	**Random W**	**LNS N**	**Control N**	**Control Prevalence**	**PR (95% CI)**	**Fixed W**	**Random W**
Bangladesh	JiVitA−4 (34)	1977	841	16.2	0.89 (0.74, 1.07)	0.34	0.34	805	376	16.8	0.96 (0.73, 1.27)	0.26	0.24
Bangladesh	RDNS (35)	1057	493	13.8	0.90 (0.68, 1.18)	0.15	0.15	604	322	17.1	0.83 (0.62, 1.13)	0.21	0.21
Bangladesh	WASH−B (36)	917	2647	10.2	0.79 (0.63, 0.98)	0.23	0.23	239	777	14.5	0.81 (0.54, 1.19)	0.13	0.14
Burkina Faso	iLiNS−Zinc (37)	1013	320	10.9	0.75 (0.50, 1.13)	0.07	0.07	932	343	15.7	0.63 (0.48, 0.82)	0.27	0.25
Burkina Faso	PROMIS (38)	501	548	8.4	0.97 (0.64, 1.48)	0.06	0.06	355	359	9.7	0.72 (0.39, 1.32)	0.05	0.07
Burkina Faso	PROMIS CS (38)												
Ghana	GHANA (39)												
Ghana	iLiNS−DYADG (40)	244	470	8.1	0.81 (0.46, 1.42)	0.04	0.04	102	219	6.4	0.92 (0.36, 2.33)	0.02	0.03
Haiti	HAITI (41)												
Kenya	WASH−B (42)												
Madagascar	MAHAY (43)	987	927	6.8	0.78 (0.51, 1.17)	0.07	0.07	354	410	4.6	1.40 (0.66, 2.96)	0.04	0.04
Malawi	iLiNS−DYADM (44)												
Malawi	iLiNS−DOSE (45)												
Mali	PROMIS (46)												
Mali	PROMIS CS (46)												
Zimbabwe	SHINE (HIV−) (47)	1384	1297	2.3	1.03 (0.62, 1.71)	0.04	0.04	294	324	4.0	0.42 (0.15, 1.19)	0.02	0.02
Zimbabwe	SHINE (HIV+) (48)												
		**8080**	**7543**		**I² = 0.00, Tau² = 0.00**			**3685**	**3130**		**I² = 0.19, Tau² = 0.01**		
**Fixed**					**0.86 (0.77, 0.95)**						**0.80 (0.69, 0.92)**		
**Random**					**0.86 (0.77, 0.95)**						**0.80 (0.68, 0.94)**		

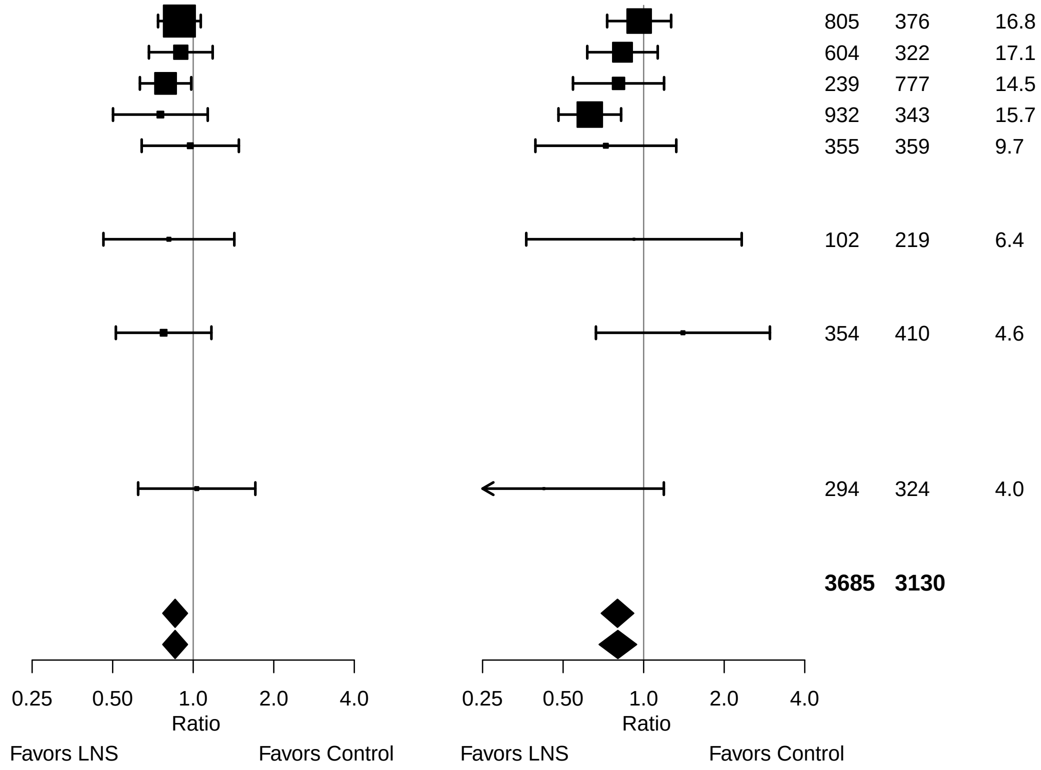

Supplemental figure 9E: Wasting prevalence ratio

9E3: Stratified by Household source water quality

P−for−interaction = 0.075
Ratio of PRs = 1.27 (0.98, 1.65)

Country	Trial	LNS N	Control N	Control Prevalence	Improved PR (95% CI)	Fixed W	Random W	LNS N	Control N	Control Prevalence	Unimproved PR (95% CI)	Fixed W	Random W
Bangladesh	JiVitA−4 (34)												
Bangladesh	RDNS (35)												
Bangladesh	WASH−B (36)	495	986	10.6	0.76 (0.55, 1.04)	0.27	0.27	70	114	10.5	1.36 (0.58, 3.19)	0.03	0.09
Burkina Faso	iLiNS−Zinc (37)	550	141	12.8	0.52 (0.31, 0.89)	0.10	0.10	1394	519	13.7	0.72 (0.58, 0.90)	0.53	0.24
Burkina Faso	PROMIS (38)	352	377	9.3	0.73 (0.48, 1.12)	0.15	0.15	357	350	9.1	0.83 (0.44, 1.54)	0.07	0.13
Burkina Faso	PROMIS CS (38)	280	267	16.1	0.69 (0.43, 1.09)	0.13	0.13	149	169	6.5	2.47 (1.25, 4.88)	0.06	0.11
Ghana	GHANA (39)												
Ghana	iLiNS−DYADG (40)												
Haiti	HAITI (41)												
Kenya	WASH−B (42)												
Madagascar	MAHAY (43)	418	453	5.5	1.13 (0.60, 2.11)	0.07	0.07	1202	1180	6.0	0.90 (0.60, 1.35)	0.16	0.18
Malawi	iLiNS−DYADM (44)												
Malawi	iLiNS−DOSE (45)												
Mali	PROMIS (46)												
Mali	PROMIS CS (46)	544	562	9.8	0.77 (0.54, 1.10)	0.22	0.22	361	382	10.7	0.93 (0.60, 1.44)	0.13	0.17
Zimbabwe	SHINE (HIV−) (47)	518	486	2.9	1.01 (0.50, 2.00)	0.06	0.06	273	301	3.0	0.74 (0.29, 1.87)	0.03	0.08
Zimbabwe	SHINE (HIV+) (48)												
		**3157**	**3272**		**I² = 0.00, Tau² = 0.00**			**3806**	**3015**		**I² = 0.54, Tau² = 0.09**		
**Fixed**					**0.75 (0.64, 0.89)**						**0.85 (0.73, 1.00)**		
**Random**					**0.75 (0.64, 0.89)**						**0.97 (0.72, 1.31)**		

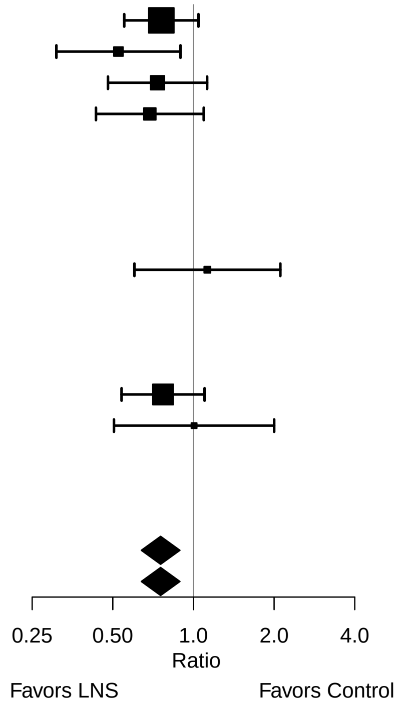

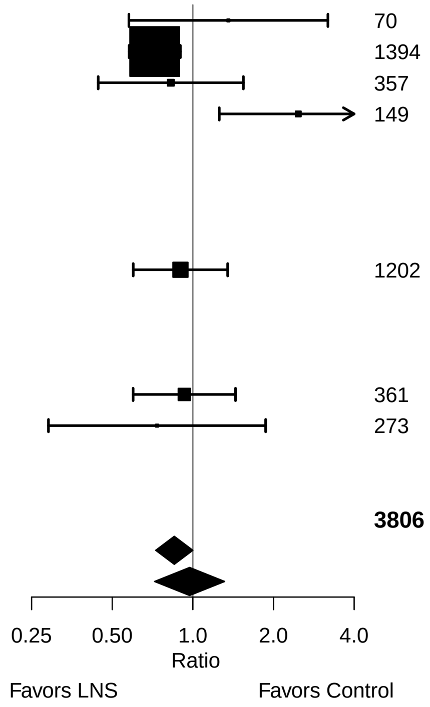

Supplemental figure 9E: Wasting prevalence ratio

9E4: Stratified by Household sanitation

P−for−interaction = 0.080
Ratio of PRs = 1.20 (0.98, 1.48)

Country	Trial	Improved LNS N	Improved Control N	Improved Control Prevalence	Improved PR (95% CI)	Improved Fixed W	Improved Random W	Unimproved LNS N	Unimproved Control N	Unimproved Control Prevalence	Unimproved PR (95% CI)	Unimproved Fixed W	Unimproved Random W
Bangladesh	JiVitA−4 (34)	2151	932	16.1	0.87 (0.72, 1.04)	0.48	0.48	631	285	17.4	1.06 (0.80, 1.39)	0.39	0.27
Bangladesh	RDNS (35)	1179	573	15.2	0.74 (0.58, 0.96)	0.25	0.25	480	241	14.9	1.17 (0.84, 1.63)	0.27	0.24
Bangladesh	WASH−B (36)												
Burkina Faso	iLiNS−Zinc (37)												
Burkina Faso	PROMIS (38)	392	548	9.1	0.87 (0.52, 1.44)	0.06	0.06	464	355	8.7	0.86 (0.55, 1.36)	0.14	0.18
Burkina Faso	PROMIS CS (38)	176	165	13.3	0.77 (0.43, 1.37)	0.05	0.05	252	271	11.8	1.24 (0.76, 2.04)	0.12	0.16
Ghana	GHANA (39)												
Ghana	iLiNS−DYADG (40)												
Haiti	HAITI (41)												
Kenya	WASH−B (42)												
Madagascar	MAHAY (43)												
Malawi	iLiNS−DYADM (44)												
Malawi	iLiNS−DOSE (45)												
Mali	PROMIS (46)												
Mali	PROMIS CS (46)	696	692	11.0	0.82 (0.61, 1.12)	0.17	0.17	217	242	8.7	0.53 (0.30, 0.93)	0.09	0.14
Zimbabwe	SHINE (HIV−) (47)												
Zimbabwe	SHINE (HIV+) (48)												
		**4594**	**2910**		**I² = 0.00, Tau² = 0.00**			**2044**	**1394**		**I² = 0.43, Tau² = 0.05**		
**Fixed**					**0.82 (0.73, 0.93)**						**1.01 (0.85, 1.20)**		
**Random**					**0.82 (0.73, 0.93)**						**0.97 (0.74, 1.27)**		

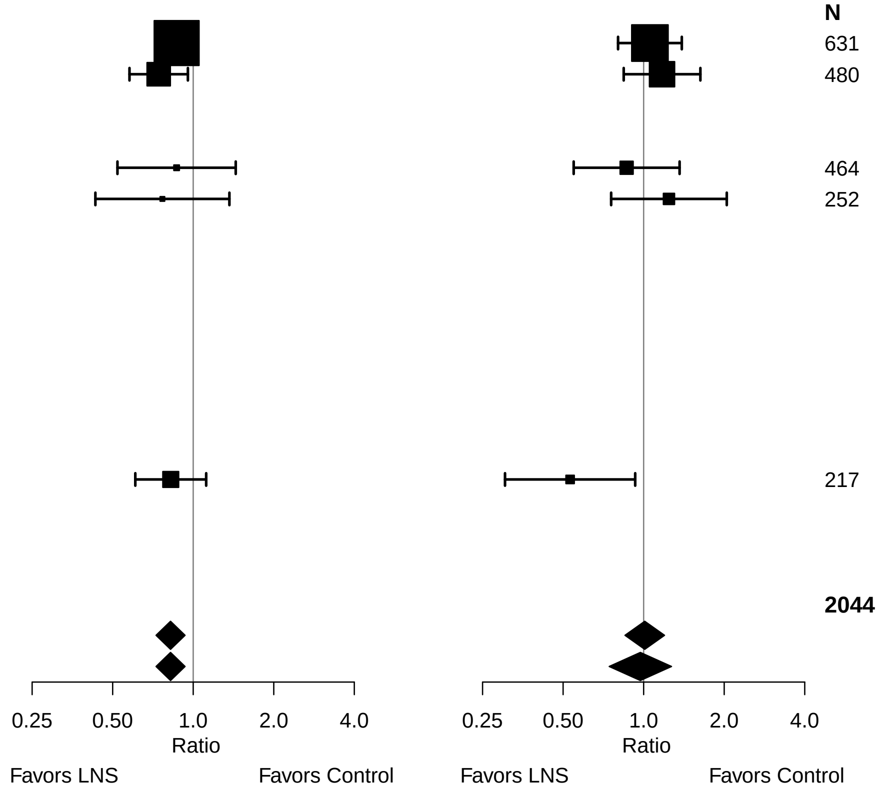

Supplemental figure 9E: Wasting prevalence ratio

9E5: Stratified by Home environment

P−for−interaction = 0.478
Ratio of PRs = 1.09 (0.86, 1.37)

Country	Trial	At least median: LNS N	Control N	Control Prevalence	PR (95% CI)	Fixed W	Random W	Less than median: LNS N	Control N	Control Prevalence	PR (95% CI)	Fixed W	Random W
Bangladesh	JiVitA−4 (34)												
Bangladesh	RDNS (35)	1192	582	14.6	0.80 (0.61, 1.05)	0.33	0.26	466	230	15.7	1.06 (0.77, 1.46)	0.28	0.28
Bangladesh	WASH−B (36)	636	1652	10.4	0.68 (0.50, 0.92)	0.27	0.24	492	1684	11.9	0.94 (0.71, 1.25)	0.35	0.35
Burkina Faso	iLiNS−Zinc (37)	473	199	11.6	0.61 (0.42, 0.87)	0.18	0.19	271	174	17.7	0.72 (0.45, 1.16)	0.12	0.12
Burkina Faso	PROMIS (38)												
Burkina Faso	PROMIS CS (38)												
Ghana	GHANA (39)												
Ghana	iLiNS−DYADG (40)	213	409	6.4	1.03 (0.55, 1.94)	0.06	0.09	119	248	9.7	0.43 (0.17, 1.11)	0.03	0.03
Haiti	HAITI (41)												
Kenya	WASH−B (42)	834	2757	1.1	1.21 (0.61, 2.41)	0.05	0.08	573	2149	1.5	0.80 (0.36, 1.74)	0.05	0.05
Madagascar	MAHAY (43)	914	907	3.7	1.14 (0.71, 1.83)	0.11	0.14	786	775	8.3	0.86 (0.57, 1.30)	0.17	0.17
Malawi	iLiNS−DYADM (44)												
Malawi	iLiNS−DOSE (45)												
Mali	PROMIS (46)												
Mali	PROMIS CS (46)												
Zimbabwe	SHINE (HIV−) (47)												
Zimbabwe	SHINE (HIV+) (48)												
		**4262**	**6506**		**I² = 0.33, Tau² = 0.02**			**2707**	**5260**		**I² = 0.00, Tau² = 0.00**		
**Fixed**					**0.78 (0.67, 0.92)**						**0.90 (0.76, 1.06)**		
**Random**					**0.81 (0.66, 1.00)**						**0.90 (0.76, 1.06)**		

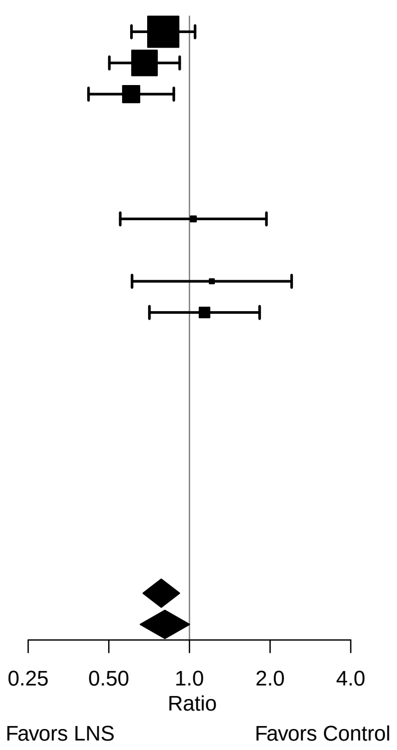

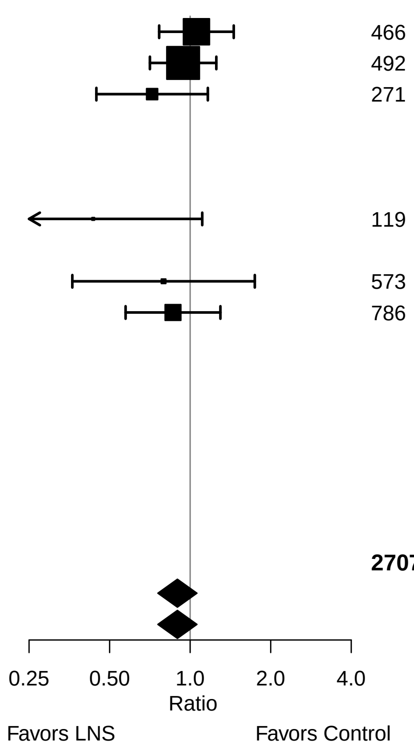

Supplemental figure 9E: Wasting prevalence ratio

9E6: Stratified by Season at the time of assessment

P−for−interaction = 0.088
Ratio of PRs = 1.16 (0.98, 1.39)

Country	Trial	Dry LNS N	Dry Control N	Dry Control Prevalence	Dry PR (95% CI)	Dry Fixed W	Dry Random W	Rainy LNS N	Rainy Control N	Rainy Control Prevalence	Rainy PR (95% CI)	Rainy Fixed W	Rainy Random W
Bangladesh	JiVitA−4 (34)	1520	649	13.7	0.87 (0.69, 1.09)	0.29	0.28	1263	568	19.4	0.95 (0.77, 1.17)	0.33	0.33
Bangladesh	RDNS (35)	738	359	13.6	0.81 (0.59, 1.12)	0.15	0.15	923	456	16.2	0.90 (0.68, 1.19)	0.19	0.19
Bangladesh	WASH−B (36)	639	1912	8.9	0.77 (0.57, 1.05)	0.17	0.17	517	1512	14.2	0.79 (0.61, 1.02)	0.22	0.22
Burkina Faso	iLiNS−Zinc (37)	1394	477	13.8	0.60 (0.47, 0.78)	0.24	0.24	558	187	12.8	0.95 (0.62, 1.44)	0.08	0.08
Burkina Faso	PROMIS (38)	428	445	8.5	0.85 (0.51, 1.41)	0.06	0.06	428	462	9.3	0.88 (0.54, 1.42)	0.06	0.06
Burkina Faso	PROMIS CS (38)	224	190	13.2	0.81 (0.45, 1.46)	0.05	0.05	206	246	11.8	1.28 (0.80, 2.04)	0.06	0.06
Ghana	GHANA (39)												
Ghana	iLiNS−DYADG (40)												
Haiti	HAITI (41)												
Kenya	WASH−B (42)	346	1146	1.9	0.75 (0.30, 1.88)	0.02	0.02	1109	3972	1.4	0.93 (0.51, 1.68)	0.04	0.04
Madagascar	MAHAY (43)												
Malawi	iLiNS−DYADM (44)												
Malawi	iLiNS−DOSE (45)	415	134	4.5	1.52 (0.68, 3.41)	0.02	0.03	281	107	6.5	1.24 (0.56, 2.74)	0.02	0.02
Mali	PROMIS (46)												
Mali	PROMIS CS (46)												
Zimbabwe	SHINE (HIV−) (47)												
Zimbabwe	SHINE (HIV+) (48)												
		**5704**	**5312**		**I^2 = 0.08, Tau^2 = 0.00**			**5285**	**7510**		**I^2 = 0.00, Tau^2 = 0.00**		
**Fixed**					**0.78 (0.69, 0.88)**						**0.92 (0.82, 1.04)**		
**Random**					**0.78 (0.68, 0.89)**						**0.92 (0.82, 1.04)**		

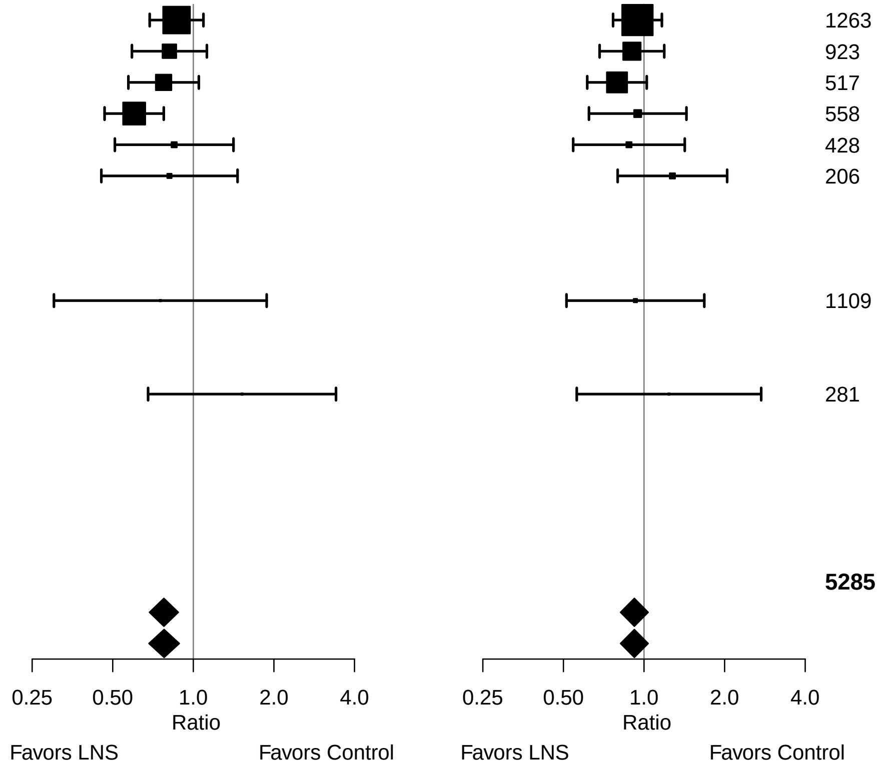

Supplemental figure 9F: Wasting prevalence difference

9F1: Stratified by Household socio-economic status

P−for−interaction = 0.223
Difference in PDs = −0.01 (−0.02, 0.00)

Country	Trial	At least median: LNS N	Control N	Control Prevalence	PD (95% CI)	Fixed W	Random W	Less than median: LNS N	Control N	Control Prevalence	PD (95% CI)	Fixed W	Random W
Bangladesh	JiVitA−4 (34)	1401	607	13.6	−0.01 (−0.04, 0.02)	0.04	0.04	1381	610	19.2	−0.02 (−0.05, 0.02)	0.04	0.04
Bangladesh	RDNS (35)	834	407	13.3	−0.02 (−0.06, 0.03)	0.02	0.02	827	408	16.9	−0.02 (−0.07, 0.02)	0.02	0.02
Bangladesh	WASH−B (36)	579	1714	8.7	−0.02 (−0.05, 0.00)	0.05	0.05	577	1710	13.7	−0.03 (−0.06, 0.00)	0.05	0.05
Burkina Faso	iLiNS−Zinc (37)	1225	299	12.4	−0.05 (−0.08, −0.01)	0.03	0.03	720	364	14.2	−0.03 (−0.07, 0.01)	0.03	0.03
Burkina Faso	PROMIS (38)	405	479	7.7	−0.01 (−0.05, 0.03)	0.02	0.02	451	428	10.3	−0.02 (−0.06, 0.03)	0.02	0.02
Burkina Faso	PROMIS CS (38)	220	208	13.0	−0.03 (−0.09, 0.03)	0.01	0.01	210	228	11.8	0.04 (−0.02, 0.10)	0.01	0.01
Ghana	GHANA (39)												
Ghana	iLiNS−DYADG (40)	157	356	6.7	0.00 (−0.05, 0.04)	0.02	0.02	190	334	8.4	−0.02 (−0.07, 0.03)	0.02	0.02
Haiti	HAITI (41)												
Kenya	WASH−B (42)	825	2914	1.2	0.00 (−0.01, 0.01)	0.54	0.54	628	2200	1.9	0.00 (−0.01, 0.01)	0.51	0.51
Madagascar	MAHAY (43)	771	884	4.1	0.00 (−0.02, 0.03)	0.06	0.06	876	755	7.9	−0.01 (−0.05, 0.02)	0.04	0.04
Malawi	iLiNS−DYADM (44)												
Malawi	iLiNS−DOSE (45)												
Mali	PROMIS (46)												
Mali	PROMIS CS (46)	501	458	10.5	−0.02 (−0.05, 0.02)	0.02	0.02	443	501	10.2	−0.02 (−0.05, 0.01)	0.04	0.04
Zimbabwe	SHINE (HIV−) (47)	903	797	2.4	0.00 (−0.01, 0.02)	0.19	0.19	826	856	2.9	−0.01 (−0.03, 0.01)	0.19	0.19
Zimbabwe	SHINE (HIV+) (48)												
		**7821**	**9123**		**I² = 0.14, Tau² = 0.00**			**7129**	**8394**		**I² = 0.00, Tau² = 0.00**		
**Fixed**					**0.00 (−0.01, 0.00)**						**−0.01 (−0.02, 0.00)**		
**Random**					**0.00 (−0.01, 0.00)**						**−0.01 (−0.02, 0.00)**		

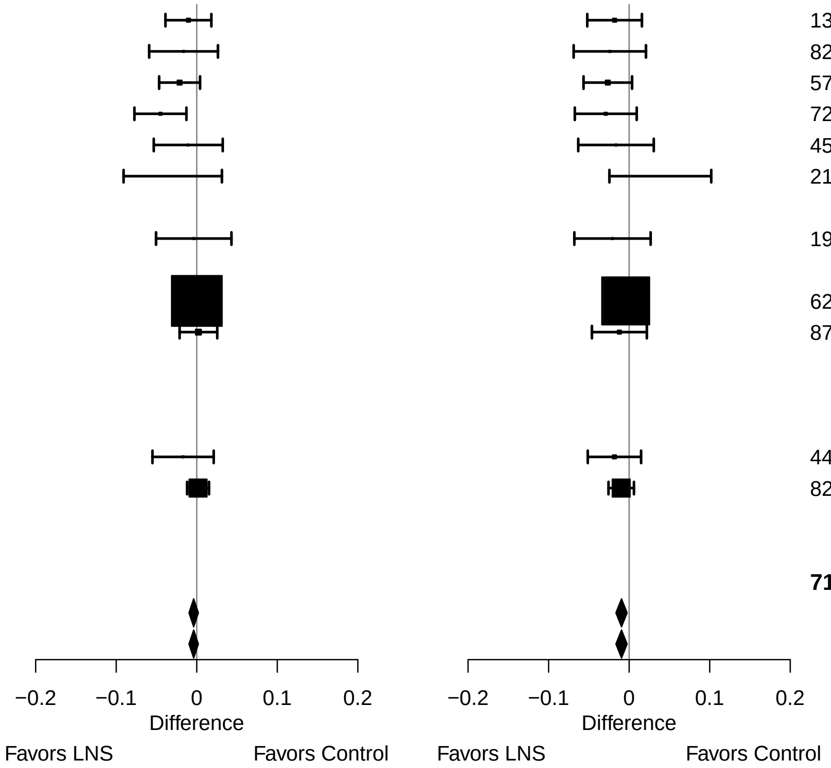

Supplemental figure 9F: Wasting prevalence difference

9F2: Stratified by Household food insecurity

P−for−interaction = 0.433
Difference in PDs = −0.01 (−0.02, 0.01)

Country	Trial	Mild to secure: LNS N	Mild to secure: Control N	Mild to secure: Control Prevalence	Mild to secure: PD (95% CI)	Mild to secure: Fixed W	Mild to secure: Random W	Moderate to severe: LNS N	Moderate to severe: Control N	Moderate to severe: Control Prevalence	Moderate to severe: PD (95% CI)	Moderate to severe: Fixed W	Moderate to severe: Random W
Bangladesh	JiVitA−4 (34)	1977	841	16.2	−0.02 (−0.05, 0.01)	0.09	0.09	805	376	16.8	0.00 (−0.04, 0.04)	0.09	0.11
Bangladesh	RDNS (35)	1057	493	13.8	−0.01 (−0.05, 0.02)	0.05	0.05	604	322	17.1	−0.03 (−0.08, 0.02)	0.08	0.10
Bangladesh	WASH−B (36)	917	2647	10.2	−0.02 (−0.04, 0.00)	0.17	0.17	239	777	14.5	−0.03 (−0.08, 0.02)	0.07	0.10
Burkina Faso	iLiNS−Zinc (37)	1013	320	10.9	−0.02 (−0.07, 0.02)	0.03	0.03	932	343	15.7	−0.06 (−0.08, −0.03)	0.27	0.21
Burkina Faso	PROMIS (38)	501	548	8.4	0.00 (−0.04, 0.03)	0.05	0.05	355	359	9.7	−0.03 (−0.08, 0.02)	0.07	0.09
Burkina Faso	PROMIS CS (38)												
Ghana	GHANA (39)												
Ghana	iLiNS−DYADG (40)	244	470	8.1	−0.02 (−0.06, 0.03)	0.04	0.04	102	219	6.4	−0.01 (−0.06, 0.05)	0.05	0.08
Haiti	HAITI (41)												
Kenya	WASH−B (42)												
Madagascar	MAHAY (43)	987	927	6.8	−0.02 (−0.04, 0.01)	0.10	0.10	354	410	4.6	0.02 (−0.02, 0.06)	0.12	0.13
Malawi	iLiNS−DYADM (44)												
Malawi	iLiNS−DOSE (45)												
Mali	PROMIS (46)												
Mali	PROMIS CS (46)												
Zimbabwe	SHINE (HIV−) (47)	1384	1297	2.3	0.00 (−0.01, 0.01)	0.46	0.46	294	324	4.0	−0.02 (−0.05, 0.00)	0.24	0.19
Zimbabwe	SHINE (HIV+) (48)												
		**8080**	**7543**		**I² = 0.00, Tau² = 0.00**			**3685**	**3130**		**I² = 0.44, Tau² = 0.00**		
**Fixed**					**−0.01 (−0.02, 0.00)**						**−0.03 (−0.04, −0.01)**		
**Random**					**−0.01 (−0.02, 0.00)**						**−0.02 (−0.04, 0.00)**		

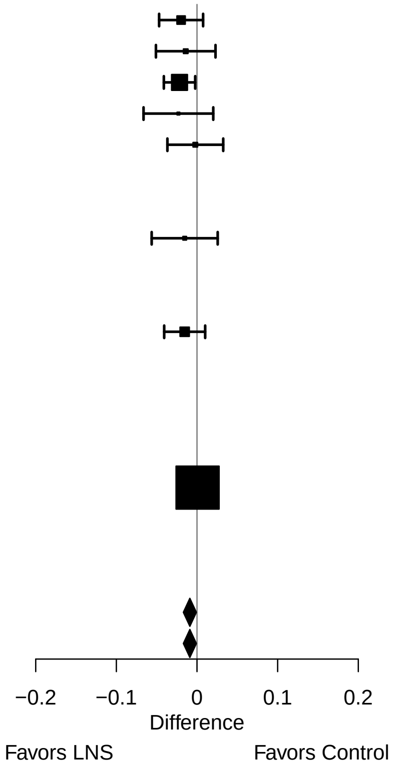

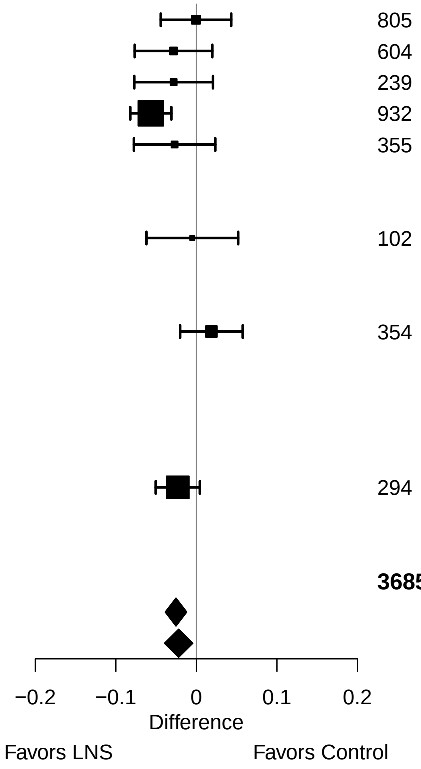

Supplemental figure 9F: Wasting prevalence difference

9F3: Stratified by Household source water quality

P−for−interaction = 0.612
Difference in PDs = 0.00 (−0.01, 0.02)

Country	Trial	Improved LNS N	Improved Control N	Improved Control Prevalence	Improved PD (95% CI)	Improved Fixed W	Improved Random W	Unimproved LNS N	Unimproved Control N	Unimproved Control Prevalence	Unimproved PD (95% CI)	Unimproved Fixed W	Unimproved Random W
Bangladesh	JiVitA−4 (34)												
Bangladesh	RDNS (35)												
Bangladesh	WASH−B (36)	495	986	10.6	−0.03 (−0.05, 0.00)	0.17	0.17	70	114	10.5	0.04 (−0.08, 0.15)	0.01	0.05
Burkina Faso	iLiNS−Zinc (37)	550	141	12.8	−0.04 (−0.09, 0.01)	0.05	0.05	1394	519	13.7	−0.04 (−0.07, −0.02)	0.24	0.19
Burkina Faso	PROMIS (38)	352	377	9.3	−0.02 (−0.06, 0.01)	0.14	0.14	357	350	9.1	−0.02 (−0.07, 0.04)	0.06	0.13
Burkina Faso	PROMIS CS (38)	280	267	16.1	−0.05 (−0.12, 0.02)	0.03	0.03	149	169	6.5	0.10 (0.03, 0.16)	0.03	0.10
Ghana	GHANA (39)												
Ghana	iLiNS−DYADG (40)												
Haiti	HAITI (41)												
Kenya	WASH−B (42)												
Madagascar	MAHAY (43)	418	453	5.5	0.01 (−0.03, 0.04)	0.11	0.11	1202	1180	6.0	−0.01 (−0.03, 0.02)	0.30	0.19
Malawi	iLiNS−DYADM (44)												
Malawi	iLiNS−DOSE (45)												
Mali	PROMIS (46)												
Mali	PROMIS CS (46)	544	562	9.8	−0.02 (−0.05, 0.01)	0.15	0.15	361	382	10.7	−0.01 (−0.05, 0.04)	0.08	0.14
Zimbabwe	SHINE (HIV−) (47)	518	486	2.9	0.00 (−0.02, 0.02)	0.36	0.36	273	301	3.0	−0.01 (−0.03, 0.02)	0.29	0.19
Zimbabwe	SHINE (HIV+) (48)												
		**3157**	**3272**		**I² = 0.10, Tau² = 0.00**			**3806**	**3015**		**I² = 0.62, Tau² = 0.00**		
**Fixed**					**−0.01 (−0.03, 0.00)**						**−0.01 (−0.02, 0.00)**		
**Random**					**−0.01 (−0.03, 0.00)**						**0.00 (−0.03, 0.03)**		

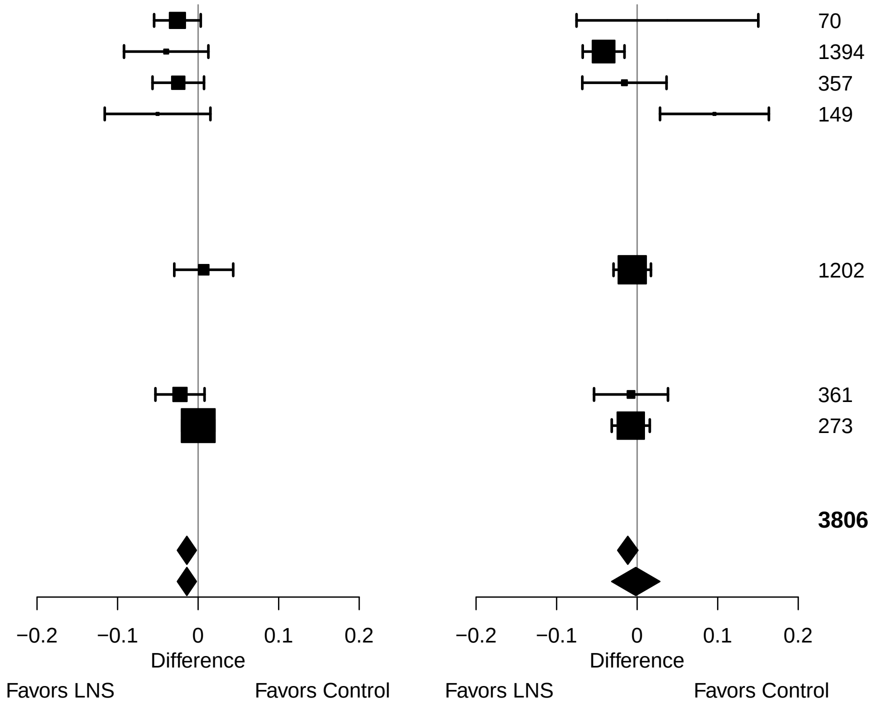

Supplemental figure 9F: Wasting prevalence difference

9F4: Stratified by Household sanitation

P−for−interaction = 0.134
Difference in PDs = 0.02 (−0.01, 0.05)

Country	Trial	Improved LNS N	Improved Control N	Improved Control Prevalence	Improved PD (95% CI)	Improved Fixed W	Improved Random W	Unimproved LNS N	Unimproved Control N	Unimproved Control Prevalence	Unimproved PD (95% CI)	Unimproved Fixed W	Unimproved Random W
Bangladesh	JiVitA−4 (34)	2151	932	16.1	−0.02 (−0.05, 0.00)	0.37	0.37	631	285	17.4	0.02 (−0.03, 0.06)	0.18	0.19
Bangladesh	RDNS (35)	1179	573	15.2	−0.04 (−0.07, 0.00)	0.19	0.19	480	241	14.9	0.03 (−0.03, 0.08)	0.15	0.17
Bangladesh	WASH−B (36)												
Burkina Faso	iLiNS−Zinc (37)												
Burkina Faso	PROMIS (38)	392	548	9.1	−0.01 (−0.05, 0.03)	0.14	0.14	464	355	8.7	−0.01 (−0.05, 0.03)	0.28	0.25
Burkina Faso	PROMIS CS (38)	176	165	13.3	−0.03 (−0.10, 0.04)	0.05	0.05	252	271	11.8	0.03 (−0.04, 0.09)	0.10	0.13
Ghana	GHANA (39)												
Ghana	iLiNS−DYADG (40)												
Haiti	HAITI (41)												
Kenya	WASH−B (42)												
Madagascar	MAHAY (43)												
Malawi	iLiNS−DYADM (44)												
Malawi	iLiNS−DOSE (45)												
Mali	PROMIS (46)												
Mali	PROMIS CS (46)	696	692	11.0	−0.02 (−0.05, 0.01)	0.26	0.26	217	242	8.7	−0.04 (−0.08, 0.00)	0.29	0.26
Zimbabwe	SHINE (HIV−) (47)												
Zimbabwe	SHINE (HIV+) (48)												
		**4594**	**2910**		**I^2 = 0.00, Tau^2 = 0.00**			**2044**	**1394**		**I^2 = 0.42, Tau^2 = 0.00**		
**Fixed**					**−0.02 (−0.04, −0.01)**						**−0.01 (−0.03, 0.01)**		
**Random**					**−0.02 (−0.04, −0.01)**						**0.00 (−0.03, 0.02)**		

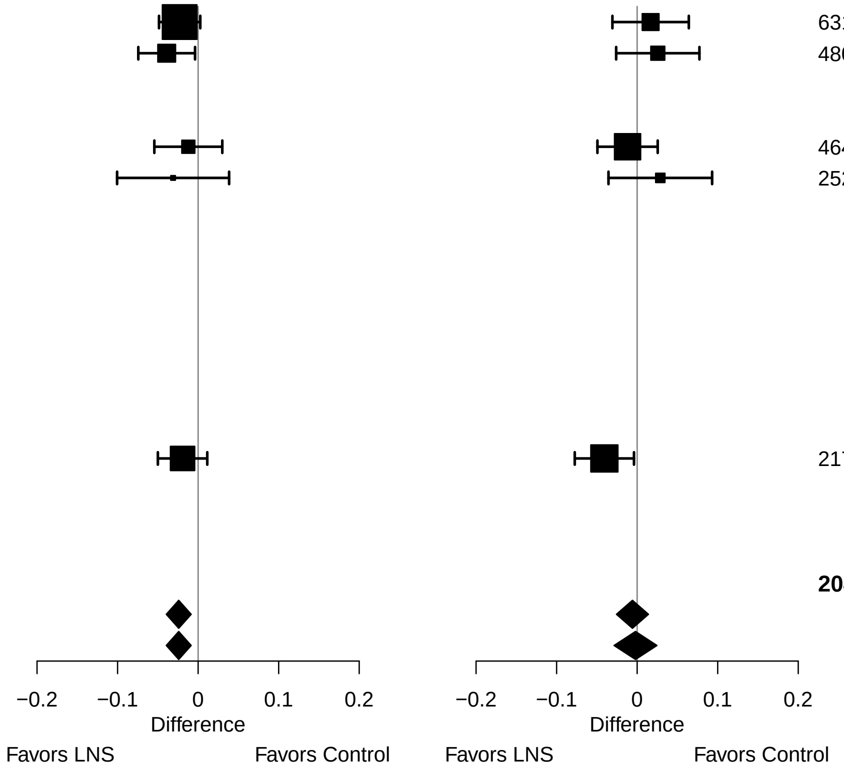

Supplemental figure 9F: Wasting prevalence difference

9F5: Stratified by Home environment

P−for−interaction = 0.413
Difference in PDs = 0.00 (−0.02, 0.01)

		At least median						Less than median					
Country	Trial	LNS N	Control N	Control Prevalence	PD (95% CI)	Fixed W	Random W	LNS N	Control N	Control Prevalence	PD (95% CI)	Fixed W	Random W
Bangladesh	JiVitA−4 (34)												
Bangladesh	RDNS (35)	1192	582	14.6	−0.03 (−0.07, 0.01)	0.04	0.12	466	230	15.7	0.01 (−0.04, 0.06)	0.03	0.03
Bangladesh	WASH−B (36)	636	1652	10.4	−0.03 (−0.06, −0.01)	0.09	0.18	492	1684	11.9	−0.01 (−0.04, 0.03)	0.08	0.08
Burkina Faso	iLiNS−Zinc (37)	473	199	11.6	−0.04 (−0.08, 0.00)	0.03	0.11	271	174	17.7	−0.04 (−0.11, 0.02)	0.02	0.02
Burkina Faso	PROMIS (38)												
Burkina Faso	PROMIS CS (38)												
Ghana	GHANA (39)												
Ghana	iLiNS−DYADG (40)	213	409	6.4	0.00 (−0.04, 0.04)	0.03	0.10	119	248	9.7	−0.05 (−0.11, 0.00)	0.02	0.02
Haiti	HAITI (41)												
Kenya	WASH−B (42)	834	2757	1.1	0.00 (−0.01, 0.01)	0.66	0.28	573	2149	1.5	0.00 (−0.01, 0.01)	0.77	0.77
Madagascar	MAHAY (43)	914	907	3.7	0.01 (−0.01, 0.02)	0.14	0.21	786	775	8.3	−0.01 (−0.04, 0.02)	0.08	0.08
Malawi	iLiNS−DYADM (44)												
Malawi	iLiNS−DOSE (45)												
Mali	PROMIS (46)												
Mali	PROMIS CS (46)												
Zimbabwe	SHINE (HIV−) (47)												
Zimbabwe	SHINE (HIV+) (48)												
		**4262**	**6506**		**I² = 0.60, Tau² = 0.00**			**2707**	**5260**		**I² = 0.00, Tau² = 0.00**		
**Fixed**					**0.00 (−0.01, 0.00)**						**−0.01 (−0.01, 0.00)**		
**Random**					**−0.01 (−0.03, 0.00)**						**−0.01 (−0.01, 0.00)**		

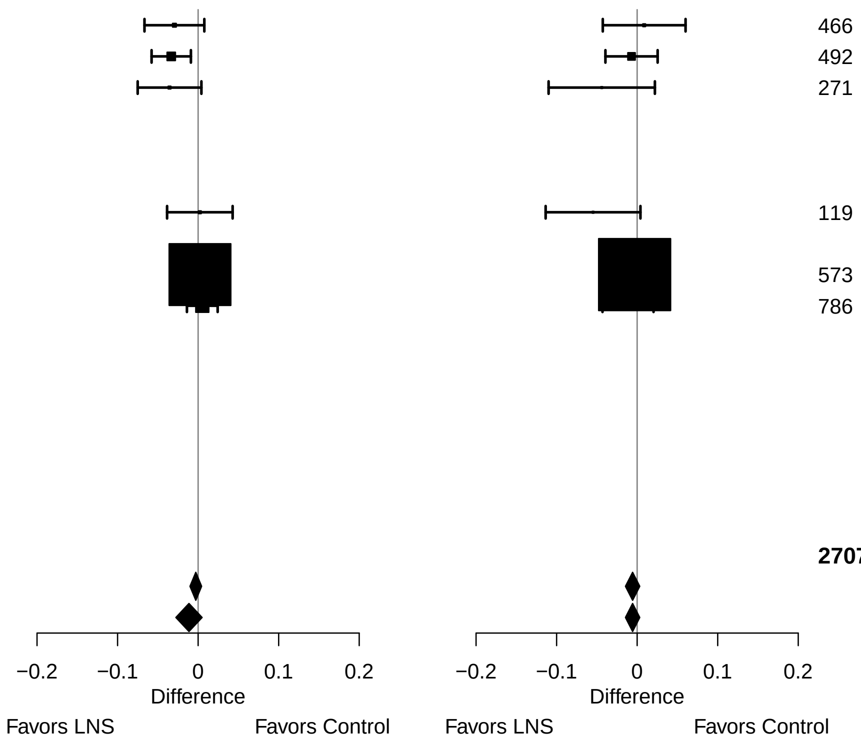

Supplemental figure 9F: Wasting prevalence difference

9F6: Stratified by Season at the time of assessment

P−for−interaction = 0.536
Difference in PDs = 0.00 (−0.01, 0.02)

Country	Trial	Dry LNS N	Dry Control N	Dry Control Prevalence	Dry PD (95% CI)	Dry Fixed W	Dry Random W	Rainy LNS N	Rainy Control N	Rainy Control Prevalence	Rainy PD (95% CI)	Rainy Fixed W	Rainy Random W
Bangladesh	JiVitA−4 (34)	1520	649	13.7	−0.02 (−0.04, 0.01)	0.10	0.13	1263	568	19.4	−0.01 (−0.05, 0.03)	0.03	0.03
Bangladesh	RDNS (35)	738	359	13.6	−0.03 (−0.07, 0.02)	0.05	0.07	923	456	16.2	−0.02 (−0.06, 0.03)	0.02	0.02
Bangladesh	WASH−B (36)	639	1912	8.9	−0.02 (−0.04, 0.00)	0.16	0.18	517	1512	14.2	−0.03 (−0.06, 0.00)	0.05	0.05
Burkina Faso	iLiNS−Zinc (37)	1394	477	13.8	−0.05 (−0.07, −0.03)	0.18	0.18	558	187	12.8	−0.03 (−0.08, 0.03)	0.02	0.02
Burkina Faso	PROMIS (38)	428	445	8.5	−0.01 (−0.05, 0.03)	0.05	0.08	428	462	9.3	−0.01 (−0.05, 0.03)	0.03	0.03
Burkina Faso	PROMIS CS (38)	224	190	13.2	−0.02 (−0.10, 0.05)	0.02	0.03	206	246	11.8	0.03 (−0.03, 0.10)	0.01	0.01
Ghana	GHANA (39)												
Ghana	iLiNS−DYADG (40)												
Haiti	HAITI (41)												
Kenya	WASH−B (42)	346	1146	1.9	0.00 (−0.02, 0.01)	0.40	0.27	1109	3972	1.4	0.00 (−0.01, 0.01)	0.83	0.83
Madagascar	MAHAY (43)												
Malawi	iLiNS−DYADM (44)												
Malawi	iLiNS−DOSE (45)	415	134	4.5	0.01 (−0.03, 0.06)	0.04	0.06	281	107	6.5	0.00 (−0.05, 0.06)	0.01	0.01
Mali	PROMIS (46)												
Mali	PROMIS CS (46)												
Zimbabwe	SHINE (HIV−) (47)												
Zimbabwe	SHINE (HIV+) (48)												
		**5704**	**5312**		**I² = 0.42, Tau² = 0.00**			**5285**	**7510**		**I² = 0.00, Tau² = 0.00**		
**Fixed**					**−0.02 (−0.03, −0.01)**						**0.00 (−0.01, 0.00)**		
**Random**					**−0.02 (−0.03, −0.01)**						**0.00 (−0.01, 0.00)**		

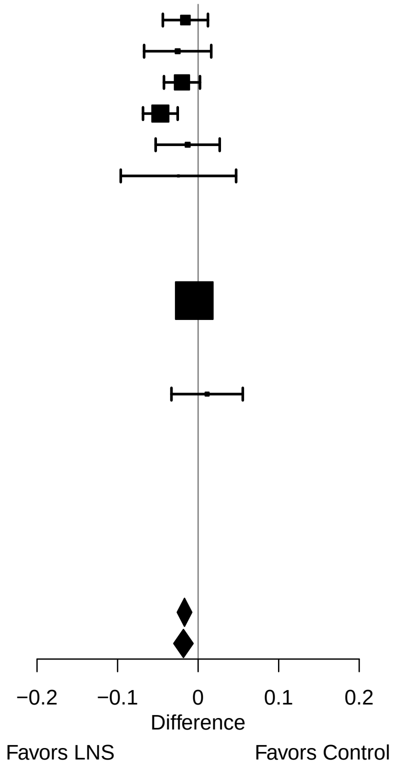

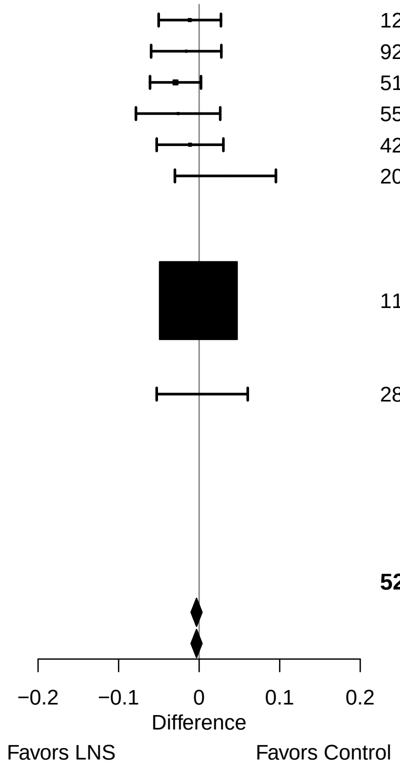

Supplemental figure 9G: Mean difference in MUACZ

9G1: Stratified by Household socio-economic status

P−for−interaction = 0.768
Difference in MDs = 0.01 (−0.03, 0.04)

		At least median						Less than median					
**Country**	**Trial**	**LNS N**	**Control N**	**Control Mean**	**MD (95% CI)**	**Fixed W**	**Random W**	**LNS N**	**Control N**	**Control Mean**	**MD (95% CI)**	**Fixed W**	**Random W**
Bangladesh	JiVitA−4 (34)	1463	641	−0.95	0.09 (0.03, 0.15)	0.29	0.12	1433	630	−1.18	0.07 (0.01, 0.13)	0.24	0.13
Bangladesh	RDNS (35)	834	407	−0.60	0.05 (−0.06, 0.15)	0.09	0.09	829	408	−0.80	0.03 (−0.07, 0.12)	0.11	0.10
Bangladesh	WASH−B (36)												
Burkina Faso	iLiNS−Zinc (37)	1232	300	−1.09	0.30 (0.18, 0.42)	0.06	0.08	720	365	−1.16	0.29 (0.20, 0.38)	0.11	0.10
Burkina Faso	PROMIS (38)	403	479	−0.71	0.08 (−0.09, 0.25)	0.03	0.05	451	433	−0.76	0.10 (−0.02, 0.22)	0.07	0.08
Burkina Faso	PROMIS CS (38)	219	207	−0.82	−0.06 (−0.26, 0.13)	0.02	0.05	205	229	−0.81	−0.06 (−0.27, 0.15)	0.02	0.04
Ghana	GHANA (39)												
Ghana	iLiNS−DYADG (40)	157	356	−0.40	0.07 (−0.10, 0.25)	0.03	0.05	190	335	−0.54	0.11 (−0.05, 0.27)	0.04	0.06
Haiti	HAITI (41)												
Kenya	WASH−B (42)	827	2949	−0.39	0.14 (0.06, 0.22)	0.15	0.11	632	2232	−0.55	0.09 (−0.01, 0.19)	0.10	0.10
Madagascar	MAHAY (43)	761	865	−0.61	−0.05 (−0.22, 0.12)	0.03	0.06	866	750	−0.87	0.02 (−0.12, 0.17)	0.05	0.07
Malawi	iLiNS−DYADM (44)	121	234	0.14	−0.06 (−0.26, 0.15)	0.02	0.04	99	209	0.00	−0.02 (−0.27, 0.23)	0.02	0.03
Malawi	iLiNS−DOSE (45)	303	104	0.18	0.05 (−0.11, 0.21)	0.03	0.06	292	102	−0.06	0.00 (−0.17, 0.18)	0.03	0.05
Mali	PROMIS (46)	248	250	−0.78	0.17 (0.02, 0.31)	0.04	0.07	246	246	−0.76	0.19 (0.01, 0.37)	0.03	0.05
Mali	PROMIS CS (46)	505	463	−0.86	0.16 (0.01, 0.31)	0.04	0.07	449	507	−0.80	0.10 (−0.04, 0.24)	0.05	0.06
Zimbabwe	SHINE (HIV−) (47)	905	797	0.11	0.00 (−0.09, 0.08)	0.13	0.10	827	852	−0.11	0.15 (0.06, 0.24)	0.12	0.10
Zimbabwe	SHINE (HIV+) (48)	171	156	−0.14	0.02 (−0.17, 0.21)	0.02	0.05	158	166	−0.23	0.04 (−0.17, 0.25)	0.02	0.04
		**8149**	**8208**		**I² = 0.54, Tau² = 0.00**			**7397**	**7464**		**I² = 0.52, Tau² = 0.00**		
**Fixed**					**0.08 (0.05, 0.11)**						**0.10 (0.07, 0.13)**		
**Random**					**0.08 (0.03, 0.13)**						**0.10 (0.05, 0.14)**		

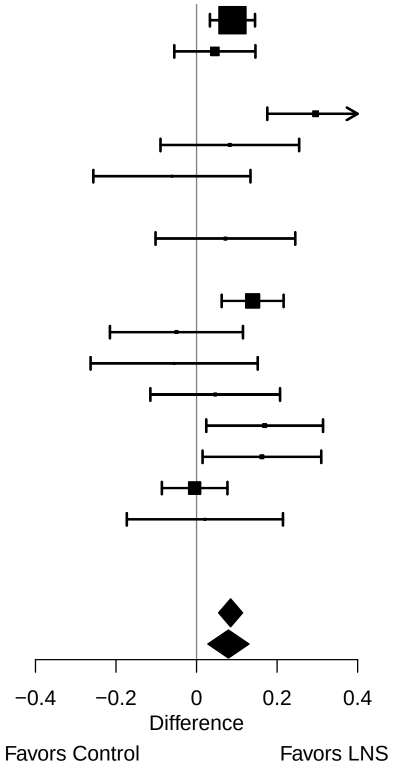

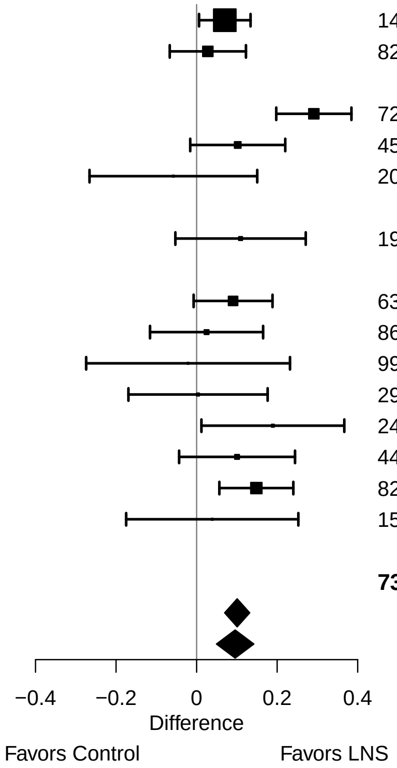

Supplemental figure 9G: Mean difference in MUACZ

9G2: Stratified by Household food insecurity

P−for−interaction = 0.595
Difference in MDs = −0.01 (−0.06, 0.03)

		Mild to secure						Moderate to severe					
**Country**	**Trial**	**LNS N**	**Control N**	**Control Mean**	**MD (95% CI)**	**Fixed W**	**Random W**	**LNS N**	**Control N**	**Control Mean**	**MD (95% CI)**	**Fixed W**	**Random W**
Bangladesh	JiVitA−4 (34)	2054	882	−1.03	0.11 (0.06, 0.16)	0.29	0.14	842	389	−1.14	0.01 (−0.07, 0.09)	0.26	0.13
Bangladesh	RDNS (35)	1058	493	−0.62	0.00 (−0.09, 0.09)	0.09	0.11	605	322	−0.83	0.09 (−0.02, 0.20)	0.12	0.11
Bangladesh	WASH−B (36)												
Burkina Faso	iLiNS−Zinc (37)	1017	321	−1.08	0.29 (0.17, 0.41)	0.06	0.09	935	344	−1.17	0.30 (0.19, 0.40)	0.14	0.12
Burkina Faso	PROMIS (38)	496	547	−0.70	0.12 (−0.04, 0.28)	0.03	0.06	358	365	−0.78	0.05 (−0.09, 0.19)	0.08	0.09
Burkina Faso	PROMIS CS (38)												
Ghana	GHANA (39)												
Ghana	iLiNS−DYADG (40)	244	471	−0.45	0.09 (−0.05, 0.24)	0.04	0.07	102	219	−0.50	0.07 (−0.13, 0.28)	0.04	0.06
Haiti	HAITI (41)												
Kenya	WASH−B (42)	1303	4640	−0.44	0.12 (0.05, 0.18)	0.19	0.13	156	528	−0.54	0.12 (−0.08, 0.32)	0.04	0.06
Madagascar	MAHAY (43)	968	907	−0.67	−0.06 (−0.21, 0.10)	0.03	0.07	353	405	−0.81	−0.02 (−0.17, 0.14)	0.06	0.08
Malawi	iLiNS−DYADM (44)	70	122	0.28	−0.07 (−0.35, 0.20)	0.01	0.03	148	316	0.00	−0.04 (−0.24, 0.15)	0.04	0.06
Malawi	iLiNS−DOSE (45)	151	55	0.11	0.15 (−0.07, 0.38)	0.02	0.04	431	145	0.00	0.00 (−0.14, 0.14)	0.08	0.09
Mali	PROMIS (46)	330	341	−0.75	0.15 (0.00, 0.31)	0.03	0.07	174	163	−0.85	0.25 (0.05, 0.45)	0.04	0.06
Mali	PROMIS CS (46)												
Zimbabwe	SHINE (HIV−) (47)	1386	1295	0.04	0.06 (−0.01, 0.12)	0.18	0.13	295	322	−0.16	0.15 (0.01, 0.29)	0.08	0.09
Zimbabwe	SHINE (HIV+) (48)	236	238	−0.20	0.06 (−0.11, 0.22)	0.03	0.06	86	76	−0.15	0.01 (−0.24, 0.26)	0.02	0.04
		**9313**	**10312**		**I² = 0.52, Tau² = 0.00**			**4485**	**3594**		**I² = 0.61, Tau² = 0.01**		
**Fixed**					**0.09 (0.07, 0.12)**						**0.09 (0.05, 0.13)**		
**Random**					**0.09 (0.04, 0.14)**						**0.09 (0.03, 0.15)**		

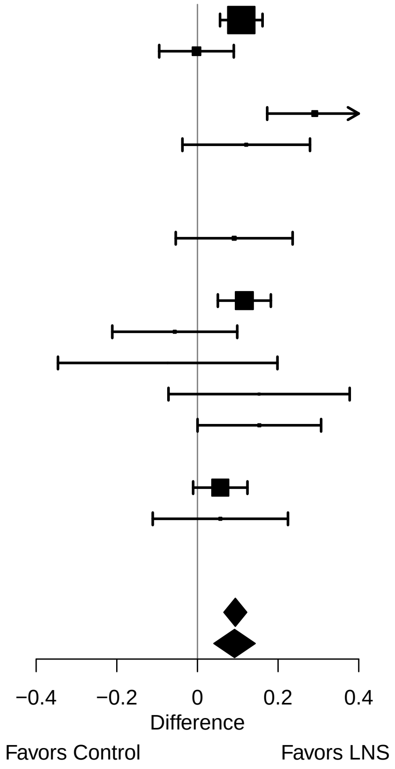

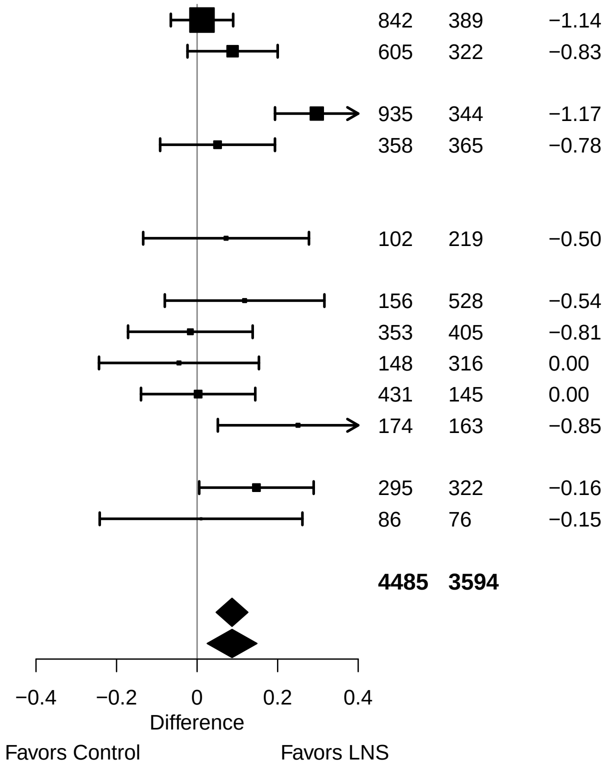

Supplemental figure 9G: Mean difference in MUACZ

9G3: Stratified by Household source water quality

P−for−interaction = 0.924
Difference in MDs = 0.00 (−0.06, 0.06)

Country	Trial	Improved LNS N	Improved Control N	Improved Control Mean	Improved MD (95% CI)	Improved Fixed W	Improved Random W	Unimproved LNS N	Unimproved Control N	Unimproved Control Mean	Unimproved MD (95% CI)	Unimproved Fixed W	Unimproved Random W
Bangladesh	JiVitA−4 (34)												
Bangladesh	RDNS (35)												
Bangladesh	WASH−B (36)												
Burkina Faso	iLiNS−Zinc (37)	555	141	−1.08	0.27 (0.15, 0.38)	0.14	0.12	1396	521	−1.15	0.31 (0.21, 0.40)	0.28	0.17
Burkina Faso	PROMIS (38)	352	376	−0.77	0.10 (−0.06, 0.25)	0.08	0.09	358	355	−0.72	0.12 (−0.07, 0.31)	0.07	0.09
Burkina Faso	PROMIS CS (38)	277	265	−0.81	−0.06 (−0.26, 0.13)	0.05	0.07	147	171	−0.82	−0.05 (−0.32, 0.21)	0.04	0.06
Ghana	GHANA (39)												
Ghana	iLiNS−DYADG (40)												
Haiti	HAITI (41)												
Kenya	WASH−B (42)	449	1562	−0.43	0.08 (−0.02, 0.19)	0.17	0.13	248	701	−0.49	0.13 (−0.02, 0.27)	0.12	0.12
Madagascar	MAHAY (43)	415	452	−0.65	−0.08 (−0.34, 0.18)	0.03	0.04	1185	1157	−0.76	−0.01 (−0.13, 0.12)	0.17	0.14
Malawi	iLiNS−DYADM (44)	200	406	0.09	−0.04 (−0.21, 0.13)	0.06	0.08	20	36	−0.07	0.06 (−0.49, 0.60)	0.01	0.02
Malawi	iLiNS−DOSE (45)	556	192	0.08	0.03 (−0.09, 0.15)	0.12	0.11	51	17	−0.27	0.05 (−0.37, 0.47)	0.01	0.03
Mali	PROMIS (46)	249	258	−0.73	0.21 (0.04, 0.38)	0.06	0.08	248	242	−0.84	0.16 (−0.02, 0.34)	0.08	0.10
Mali	PROMIS CS (46)	549	567	−0.82	0.13 (0.00, 0.26)	0.11	0.11	364	388	−0.84	0.13 (−0.03, 0.29)	0.10	0.11
Zimbabwe	SHINE (HIV−) (47)	519	482	0.00	0.09 (−0.02, 0.20)	0.16	0.13	273	300	0.07	−0.06 (−0.23, 0.10)	0.10	0.11
Zimbabwe	SHINE (HIV+) (48)	85	87	−0.23	0.12 (−0.14, 0.39)	0.03	0.04	55	56	−0.17	−0.11 (−0.49, 0.27)	0.02	0.03
		**4206**	**4788**		**I² = 0.46, Tau² = 0.00**			**4345**	**3944**		**I² = 0.63, Tau² = 0.01**		
**Fixed**					**0.10 (0.06, 0.14)**						**0.13 (0.08, 0.18)**		
**Random**					**0.09 (0.03, 0.15)**						**0.10 (0.02, 0.17)**		

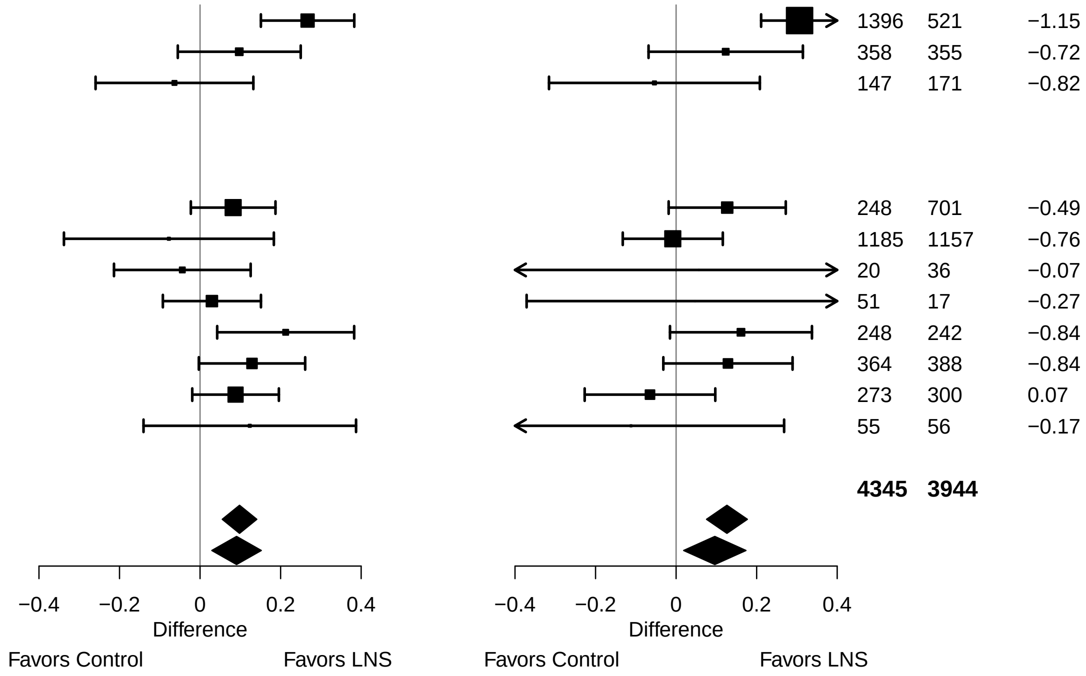

Supplemental figure 9G: Mean difference in MUACZ

9G4: Stratified by Household sanitation

P−for−interaction = 0.026
Difference in MDs = −0.06 (−0.12, −0.01)

		Improved						Unimproved					
**Country**	**Trial**	**LNS N**	**Control N**	**Control Mean**	**MD (95% CI)**	**Fixed W**	**Random W**	**LNS N**	**Control N**	**Control Mean**	**MD (95% CI)**	**Fixed W**	**Random W**
Bangladesh	JiVitA−4 (34)	2241	974	−1.01	0.09 (0.04, 0.14)	0.46	0.46	655	297	−1.23	0.05 (−0.03, 0.13)	0.19	0.11
Bangladesh	RDNS (35)	1179	573	−0.69	0.08 (0.00, 0.16)	0.16	0.16	482	241	−0.75	−0.06 (−0.20, 0.07)	0.07	0.09
Bangladesh	WASH−B (36)												
Burkina Faso	iLiNS−Zinc (37)	43	16	−1.33	0.33 (−0.14, 0.81)	0.01	0.01	1908	646	−1.13	0.30 (0.21, 0.39)	0.17	0.11
Burkina Faso	PROMIS (38)	395	548	−0.73	0.15 (0.00, 0.29)	0.05	0.05	459	359	−0.74	0.04 (−0.10, 0.18)	0.07	0.08
Burkina Faso	PROMIS CS (38)	175	163	−0.86	−0.03 (−0.24, 0.18)	0.03	0.03	248	273	−0.79	−0.08 (−0.26, 0.11)	0.04	0.07
Ghana	GHANA (39)												
Ghana	iLiNS−DYADG (40)	336	672	−0.46	0.07 (−0.05, 0.19)	0.08	0.08	10	19	−0.64	0.53 (−0.32, 1.37)	0.00	0.01
Haiti	HAITI (41)												
Kenya	WASH−B (42)	101	366	−0.34	0.15 (−0.04, 0.35)	0.03	0.03	596	1898	−0.47	0.09 (−0.01, 0.19)	0.14	0.11
Madagascar	MAHAY (43)												
Malawi	iLiNS−DYADM (44)	20	42	0.04	0.49 (0.01, 0.96)	0.01	0.01	200	400	0.08	−0.09 (−0.26, 0.08)	0.05	0.07
Malawi	iLiNS−DOSE (45)	15	9	0.12	0.27 (−0.28, 0.83)	0.00	0.00	591	200	0.05	0.02 (−0.10, 0.14)	0.09	0.10
Mali	PROMIS (46)	388	366	−0.77	0.18 (0.03, 0.34)	0.05	0.05	112	134	−0.81	0.18 (−0.02, 0.38)	0.03	0.06
Mali	PROMIS CS (46)	703	696	−0.81	0.15 (0.02, 0.28)	0.07	0.07	220	249	−0.87	0.12 (−0.07, 0.30)	0.04	0.06
Zimbabwe	SHINE (HIV−) (47)	293	241	0.11	0.02 (−0.13, 0.18)	0.05	0.05	496	540	−0.01	0.03 (−0.09, 0.16)	0.09	0.09
Zimbabwe	SHINE (HIV+) (48)	50	35	−0.13	−0.02 (−0.43, 0.40)	0.01	0.01	90	107	−0.23	0.04 (−0.23, 0.31)	0.02	0.04
		**5939**	**4701**		**I² = 0.00, Tau² = 0.00**			**6067**	**5363**		**I² = 0.68, Tau² = 0.01**		
**Fixed**					**0.10 (0.06, 0.13)**						**0.08 (0.04, 0.12)**		
**Random**					**0.10 (0.06, 0.13)**						**0.06 (0.00, 0.13)**		

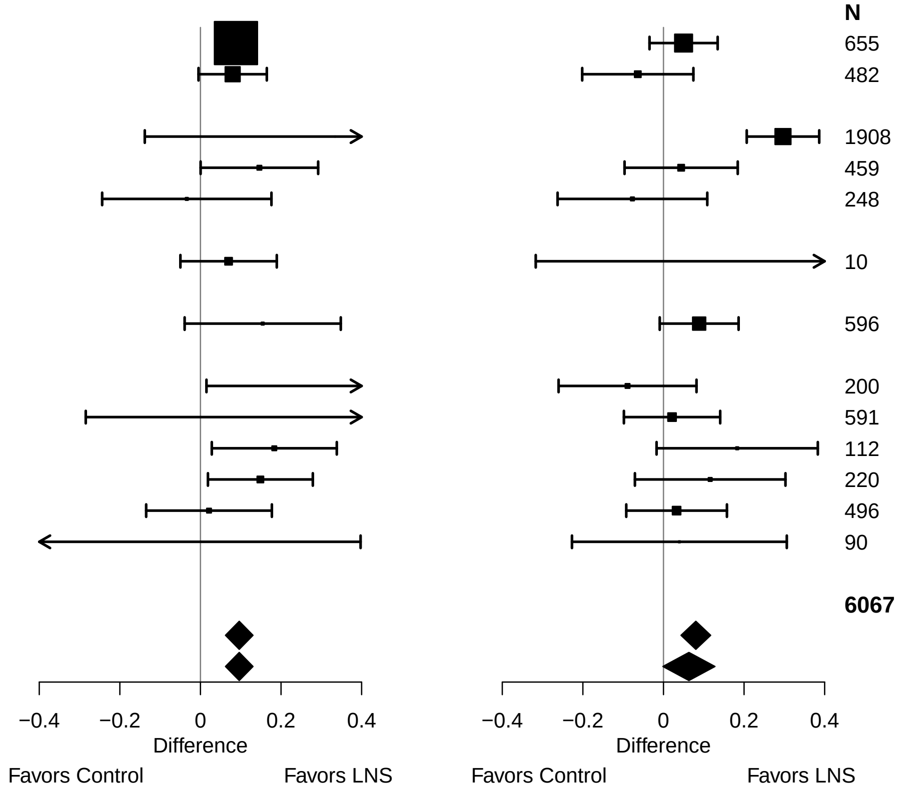

Supplemental figure 9G: Mean difference in MUACZ

9G5: Stratified by Home environment

P−for−interaction = 0.048
Difference in MDs = 0.07 (0.00, 0.13)

Country	Trial	At least median: LNS N	Control N	Control Mean	MD (95% CI)	Fixed W	Random W	Less than median: LNS N	Control N	Control Mean	MD (95% CI)	Fixed W	Random W
Bangladesh	JiVitA−4 (34)												
Bangladesh	RDNS (35)	1192	582	−0.68	0.06 (−0.03, 0.16)	0.22	0.16	468	230	−0.75	−0.03 (−0.19, 0.13)	0.13	0.15
Bangladesh	WASH−B (36)												
Burkina Faso	iLiNS−Zinc (37)	473	199	−1.08	0.32 (0.20, 0.44)	0.13	0.15	272	175	−1.22	0.32 (0.12, 0.52)	0.08	0.13
Burkina Faso	PROMIS (38)												
Burkina Faso	PROMIS CS (38)												
Ghana	GHANA (39)												
Ghana	iLiNS−DYADG (40)	213	410	−0.45	0.04 (−0.11, 0.20)	0.08	0.13	119	248	−0.52	0.19 (−0.01, 0.38)	0.08	0.13
Haiti	HAITI (41)												
Kenya	WASH−B (42)	840	2782	−0.39	0.03 (−0.05, 0.11)	0.33	0.17	573	2179	−0.50	0.19 (0.10, 0.28)	0.43	0.21
Madagascar	MAHAY (43)	905	889	−0.68	−0.04 (−0.19, 0.10)	0.09	0.14	775	769	−0.79	−0.02 (−0.16, 0.13)	0.15	0.17
Malawi	iLiNS−DYADM (44)	137	289	0.15	−0.09 (−0.28, 0.10)	0.05	0.11	78	149	−0.07	0.05 (−0.25, 0.35)	0.04	0.08
Malawi	iLiNS−DOSE (45)	378	136	−0.02	0.13 (−0.02, 0.27)	0.09	0.14	275	86	0.07	−0.02 (−0.21, 0.16)	0.09	0.13
Mali	PROMIS (46)												
Mali	PROMIS CS (46)												
Zimbabwe	SHINE (HIV−) (47)												
Zimbabwe	SHINE (HIV+) (48)												
		**4138**	**5287**		**I² = 0.75, Tau² = 0.01**			**2560**	**3836**		**I² = 0.63, Tau² = 0.01**		
**Fixed**					**0.07 (0.03, 0.12)**						**0.12 (0.06, 0.17)**		
**Random**					**0.07 (−0.03, 0.17)**						**0.10 (0.00, 0.20)**		

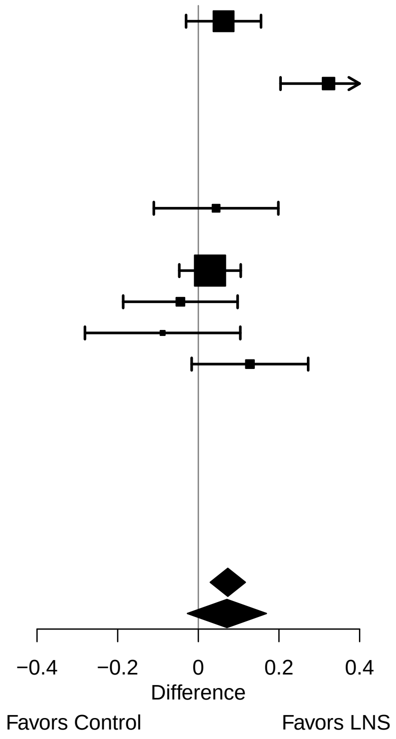

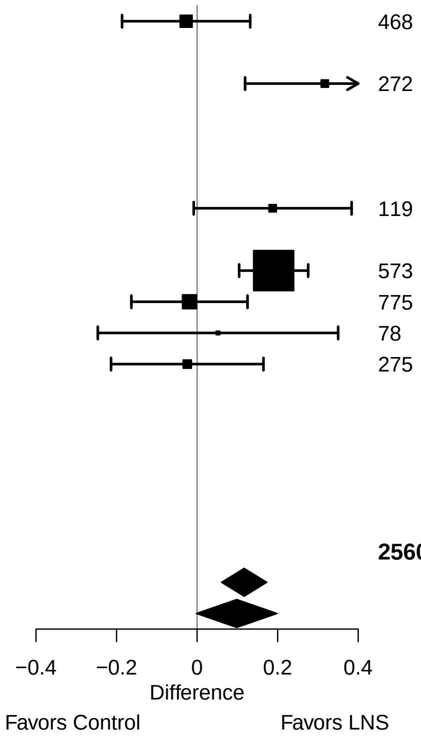

Supplemental figure 9G: Mean difference in MUACZ

9G6: Stratified by Season at the time of assessment

P−for−interaction = 0.977
Difference in MDs = 0.00 (−0.05, 0.05)

		Dry						Rainy					
**Country**	**Trial**	**LNS N**	**Control N**	**Control Mean**	**MD (95% CI)**	**Fixed W**	**Random W**	**LNS N**	**Control N**	**Control Mean**	**MD (95% CI)**	**Fixed W**	**Random W**
Bangladesh	JiVitA−4 (34)	1570	673	−1.08	0.08 (0.02, 0.14)	0.28	0.13	1327	598	−1.05	0.08 (0.01, 0.14)	0.28	0.18
Bangladesh	RDNS (35)	739	359	−0.59	0.03 (−0.09, 0.16)	0.07	0.10	924	456	−0.79	0.04 (−0.04, 0.12)	0.19	0.16
Bangladesh	WASH−B (36)												
Burkina Faso	iLiNS−Zinc (37)	1399	477	−1.17	0.31 (0.21, 0.42)	0.09	0.11	560	189	−1.05	0.25 (0.14, 0.36)	0.09	0.12
Burkina Faso	PROMIS (38)	425	444	−0.74	0.04 (−0.10, 0.18)	0.05	0.09	429	468	−0.72	0.14 (−0.01, 0.29)	0.05	0.08
Burkina Faso	PROMIS CS (38)	222	190	−0.82	−0.09 (−0.39, 0.20)	0.01	0.04	202	246	−0.81	−0.02 (−0.19, 0.15)	0.04	0.07
Ghana	GHANA (39)												
Ghana	iLiNS−DYADG (40)	204	408	−0.52	−0.02 (−0.17, 0.13)	0.05	0.08	143	285	−0.41	0.24 (0.05, 0.43)	0.03	0.06
Haiti	HAITI (41)												
Kenya	WASH−B (42)	349	1163	−0.50	0.18 (0.05, 0.31)	0.06	0.09	1112	4022	−0.44	0.10 (0.02, 0.17)	0.22	0.17
Madagascar	MAHAY (43)												
Malawi	iLiNS−DYADM (44)	156	316	0.15	−0.06 (−0.24, 0.13)	0.03	0.07	64	128	−0.11	0.01 (−0.30, 0.33)	0.01	0.03
Malawi	iLiNS−DOSE (45)	421	134	0.03	0.03 (−0.12, 0.18)	0.05	0.08	275	106	0.00	0.11 (−0.06, 0.27)	0.04	0.07
Mali	PROMIS (46)												
Mali	PROMIS CS (46)												
Zimbabwe	SHINE (HIV−) (47)	1740	1652	−0.01	0.07 (0.01, 0.13)	0.26	0.13	132	128	0.24	−0.08 (−0.30, 0.14)	0.02	0.05
Zimbabwe	SHINE (HIV+) (48)	319	305	−0.20	0.04 (−0.10, 0.18)	0.05	0.09	18	23	−0.03	0.06 (−0.45, 0.57)	0.00	0.01
		**7544**	**6121**		**I² = 0.64, Tau² = 0.01**			**5186**	**6649**		**I² = 0.39, Tau² = 0.00**		
**Fixed**					**0.08 (0.05, 0.12)**						**0.09 (0.06, 0.13)**		
**Random**					**0.07 (0.01, 0.14)**						**0.10 (0.04, 0.15)**		

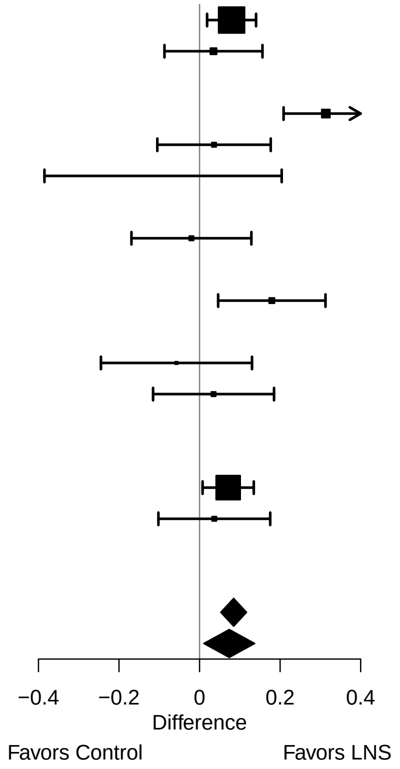

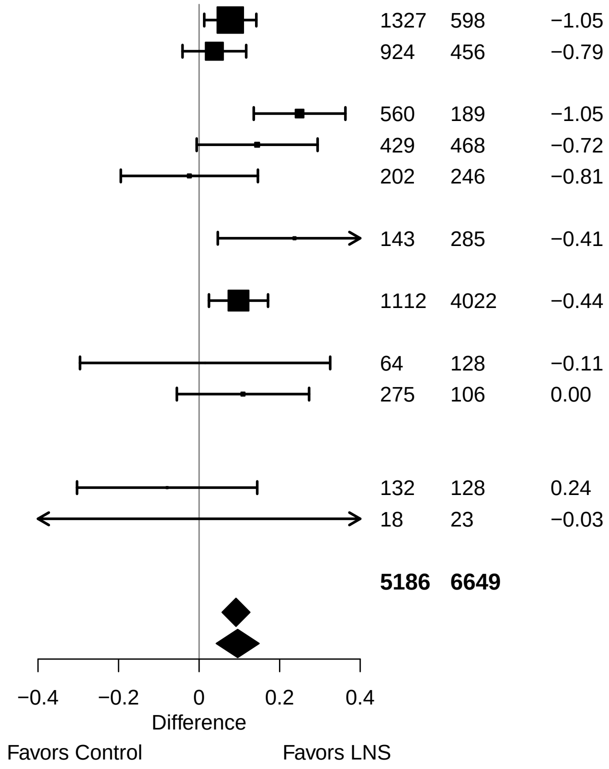

Supplemental figure 9H: Low MUAC prevalence ratio

9H1: Stratified by Household socio-economic status

P−for−interaction = 0.129
Ratio of PRs = 1.14 (0.96, 1.35)

		At least median						Less than median					
**Country**	**Trial**	**LNS N**	**Control N**	**Control Prevalence**	**PR (95% CI)**	**Fixed W**	**Random W**	**LNS N**	**Control N**	**Control Prevalence**	**PR (95% CI)**	**Fixed W**	**Random W**
Bangladesh	JiVitA−4 (34)	1463	641	11.6	0.76 (0.59, 0.96)	0.29	0.29	1433	630	18.8	0.87 (0.69, 1.10)	0.25	0.17
Bangladesh	RDNS (35)	834	407	4.4	0.84 (0.50, 1.40)	0.06	0.06	829	408	5.9	1.15 (0.70, 1.89)	0.06	0.08
Bangladesh	WASH−B (36)												
Burkina Faso	iLiNS−Zinc (37)	1232	300	16.0	0.60 (0.44, 0.80)	0.19	0.19	720	365	20.0	0.68 (0.53, 0.87)	0.23	0.17
Burkina Faso	PROMIS (38)	403	479	9.4	0.79 (0.45, 1.39)	0.05	0.05	451	433	6.5	1.17 (0.70, 1.95)	0.05	0.08
Burkina Faso	PROMIS CS (38)	219	207	14.5	0.82 (0.50, 1.33)	0.07	0.07	205	229	15.3	1.02 (0.69, 1.51)	0.09	0.11
Ghana	GHANA (39)												
Ghana	iLiNS−DYADG (40)												
Haiti	HAITI (41)												
Kenya	WASH−B (42)	827	2949	3.7	0.72 (0.46, 1.12)	0.08	0.08	632	2232	7.8	0.71 (0.48, 1.04)	0.09	0.11
Madagascar	MAHAY (43)	761	865	8.1	0.97 (0.66, 1.44)	0.11	0.11	866	750	11.2	0.91 (0.66, 1.25)	0.13	0.13
Malawi	iLiNS−DYADM (44)												
Malawi	iLiNS−DOSE (45)												
Mali	PROMIS (46)	248	250	4.8	0.90 (0.39, 2.10)	0.02	0.02	246	246	6.9	0.33 (0.12, 0.88)	0.01	0.03
Mali	PROMIS CS (46)	505	463	12.7	0.73 (0.50, 1.07)	0.12	0.12	449	507	9.5	1.13 (0.76, 1.67)	0.09	0.11
Zimbabwe	SHINE (HIV−) (47)												
Zimbabwe	SHINE (HIV+) (48)												
		**6492**	**6561**		**I² = 0.00, Tau² = 0.00**			**5831**	**5800**		**I² = 0.41, Tau² = 0.03**		
**Fixed**					**0.75 (0.66, 0.85)**						**0.86 (0.76, 0.96)**		
**Random**					**0.75 (0.66, 0.85)**						**0.88 (0.73, 1.05)**		

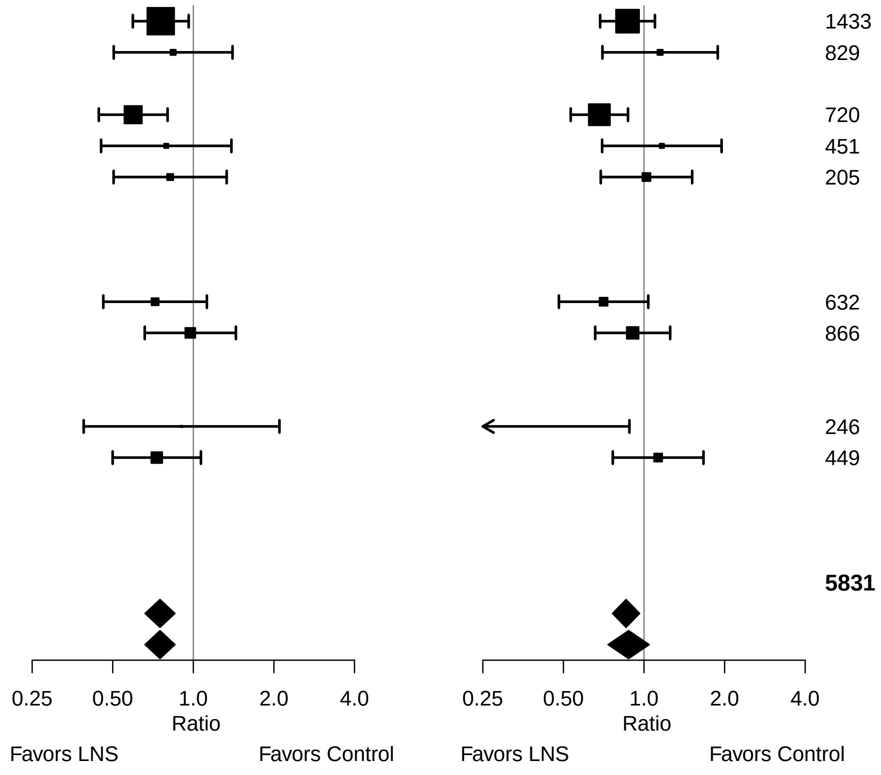

Supplemental figure 9H: Low MUAC prevalence ratio

9H2: Stratified by Household food insecurity

P−for−interaction = 0.182
Ratio of PRs = 1.15 (0.93, 1.42)

		Mild to secure						Moderate to severe					
**Country**	**Trial**	**LNS N**	**Control N**	**Control Prevalence**	**PR (95% CI)**	**Fixed W**	**Random W**	**LNS N**	**Control N**	**Control Prevalence**	**PR (95% CI)**	**Fixed W**	**Random W**
Bangladesh	JiVitA−4 (34)	2054	882	14.6	0.69 (0.57, 0.84)	0.39	0.25	842	389	16.3	1.09 (0.80, 1.51)	0.29	0.27
Bangladesh	RDNS (35)	1058	493	4.9	1.01 (0.59, 1.74)	0.05	0.08	605	322	5.6	1.03 (0.57, 1.88)	0.08	0.09
Bangladesh	WASH−B (36)												
Burkina Faso	iLiNS−Zinc (37)	1017	321	16.2	0.62 (0.47, 0.81)	0.18	0.19	935	344	20.1	0.65 (0.48, 0.88)	0.31	0.29
Burkina Faso	PROMIS (38)	496	547	7.3	1.10 (0.73, 1.66)	0.08	0.12	358	365	9.0	0.74 (0.41, 1.34)	0.08	0.09
Burkina Faso	PROMIS CS (38)												
Ghana	GHANA (39)												
Ghana	iLiNS−DYADG (40)												
Haiti	HAITI (41)												
Kenya	WASH−B (42)	1303	4640	5.4	0.67 (0.50, 0.91)	0.15	0.17	156	528	6.2	1.03 (0.52, 2.03)	0.06	0.07
Madagascar	MAHAY (43)	968	907	9.5	0.96 (0.69, 1.33)	0.13	0.16	353	405	9.1	1.05 (0.67, 1.65)	0.14	0.15
Malawi	iLiNS−DYADM (44)												
Malawi	iLiNS−DOSE (45)												
Mali	PROMIS (46)	330	341	5.0	0.57 (0.24, 1.31)	0.02	0.04	174	163	7.4	0.63 (0.24, 1.66)	0.03	0.04
Mali	PROMIS CS (46)												
Zimbabwe	SHINE (HIV−) (47)												
Zimbabwe	SHINE (HIV+) (48)												
		**7226**	**8131**		**I² = 0.41, Tau² = 0.02**			**3423**	**2516**		**I² = 0.20, Tau² = 0.01**		
**Fixed**					**0.74 (0.66, 0.83)**						**0.87 (0.74, 1.04)**		
**Random**					**0.77 (0.65, 0.91)**						**0.88 (0.73, 1.06)**		

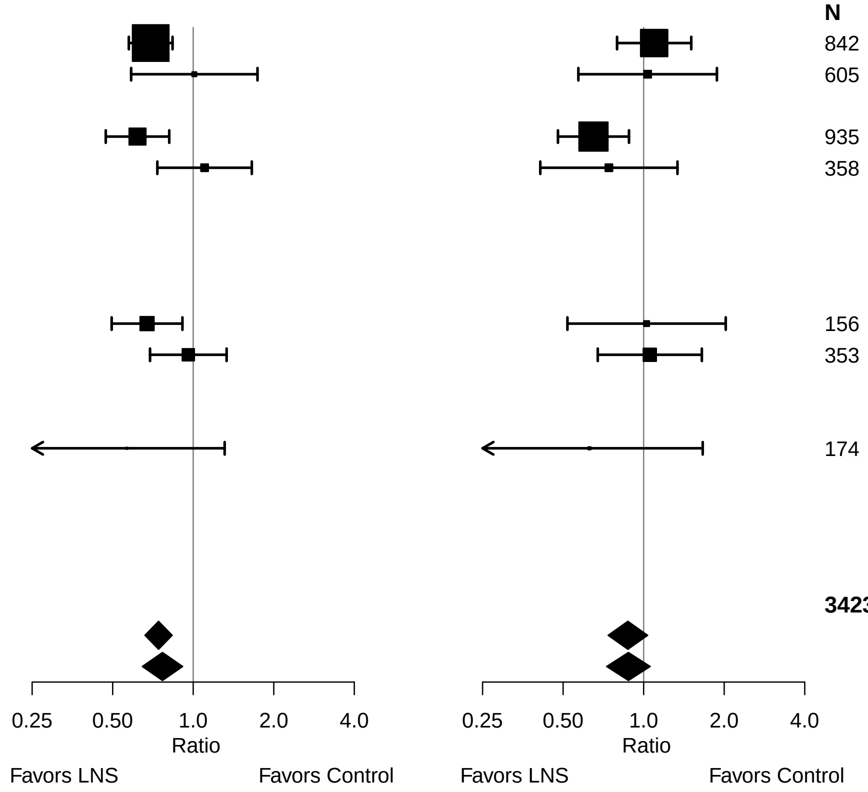

Supplemental figure 9H: Low MUAC prevalence ratio

9H3: Stratified by Household source water quality

P−for−interaction = 0.130
Ratio of PRs = 0.85 (0.69, 1.05)

		Improved						Unimproved					
**Country**	**Trial**	**LNS N**	**Control N**	**Control Prevalence**	**PR (95% CI)**	**Fixed W**	**Random W**	**LNS N**	**Control N**	**Control Prevalence**	**PR (95% CI)**	**Fixed W**	**Random W**
Bangladesh	JiVitA−4 (34)												
Bangladesh	RDNS (35)												
Bangladesh	WASH−B (36)												
Burkina Faso	iLiNS−Zinc (37)	555	141	14.2	0.66 (0.47, 0.91)	0.24	0.21	1396	521	19.4	0.63 (0.50, 0.79)	0.43	0.41
Burkina Faso	PROMIS (38)	352	376	7.7	1.14 (0.69, 1.88)	0.10	0.12	358	355	9.3	0.72 (0.43, 1.21)	0.08	0.09
Burkina Faso	PROMIS CS (38)	277	265	17.0	0.79 (0.52, 1.18)	0.16	0.16	147	171	11.7	1.22 (0.63, 2.37)	0.05	0.05
Ghana	GHANA (39)												
Ghana	iLiNS−DYADG (40)												
Haiti	HAITI (41)												
Kenya	WASH−B (42)	449	1562	5.3	0.75 (0.44, 1.29)	0.09	0.11	248	701	5.8	0.55 (0.24, 1.25)	0.03	0.04
Madagascar	MAHAY (43)	415	452	7.7	1.34 (0.86, 2.08)	0.13	0.15	1185	1157	10.1	0.83 (0.62, 1.12)	0.25	0.25
Malawi	iLiNS−DYADM (44)												
Malawi	iLiNS−DOSE (45)												
Mali	PROMIS (46)	249	258	5.0	0.58 (0.22, 1.56)	0.03	0.04	248	242	6.6	0.54 (0.25, 1.20)	0.03	0.04
Mali	PROMIS CS (46)	549	567	10.4	0.96 (0.70, 1.33)	0.25	0.22	364	388	11.6	0.88 (0.57, 1.34)	0.12	0.13
Zimbabwe	SHINE (HIV−) (47)												
Zimbabwe	SHINE (HIV+) (48)												
		**2846**	**3621**		**I² = 0.34, Tau² = 0.02**			**3946**	**3535**		**I² = 0.06, Tau² = 0.00**		
**Fixed**					**0.88 (0.74, 1.03)**						**0.73 (0.63, 0.84)**		
**Random**					**0.88 (0.72, 1.08)**						**0.73 (0.63, 0.86)**		

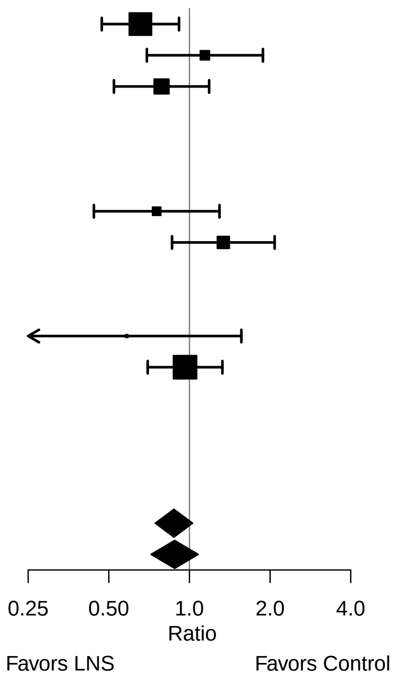

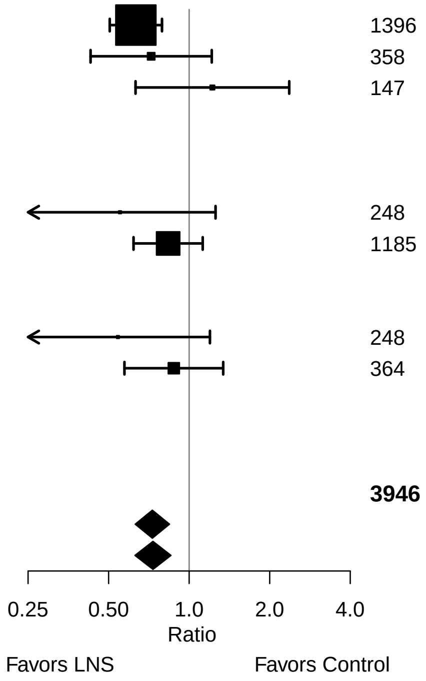

Supplemental figure 9H: Low MUAC prevalence ratio

9H4: Stratified by Household sanitation

P−for−interaction = 0.239
Ratio of PRs = 1.15 (0.91, 1.46)

		Improved						Unimproved					
Country	Trial	LNS N	Control N	Control Prevalence	PR (95% CI)	Fixed W	Random W	LNS N	Control N	Control Prevalence	PR (95% CI)	Fixed W	Random W
Bangladesh	JiVitA−4 (34)	2241	974	13.6	0.83 (0.66, 1.05)	0.41	0.41	655	297	20.1	0.91 (0.67, 1.25)	0.37	0.28
Bangladesh	RDNS (35)	1179	573	5.8	0.72 (0.50, 1.05)	0.16	0.16	482	241	3.7	2.11 (0.90, 4.94)	0.05	0.07
Bangladesh	WASH−B (36)												
Burkina Faso	iLiNS−Zinc (37)												
Burkina Faso	PROMIS (38)	395	548	8.8	0.87 (0.54, 1.40)	0.10	0.10	459	359	7.0	1.06 (0.65, 1.74)	0.14	0.16
Burkina Faso	PROMIS CS (38)	175	163	16.0	0.90 (0.57, 1.41)	0.11	0.11	248	273	14.3	0.93 (0.63, 1.38)	0.23	0.22
Ghana	GHANA (39)												
Ghana	iLiNS−DYADG (40)												
Haiti	HAITI (41)												
Kenya	WASH−B (42)	101	366	3.6	1.39 (0.54, 3.57)	0.03	0.03	596	1898	5.8	0.60 (0.34, 1.06)	0.11	0.13
Madagascar	MAHAY (43)												
Malawi	iLiNS−DYADM (44)												
Malawi	iLiNS−DOSE (45)												
Mali	PROMIS (46)												
Mali	PROMIS CS (46)	703	696	10.8	0.86 (0.61, 1.21)	0.19	0.19	220	249	11.2	0.93 (0.52, 1.65)	0.11	0.13
Zimbabwe	SHINE (HIV−) (47)												
Zimbabwe	SHINE (HIV+) (48)												
		**4794**	**3320**		**I² = 0.00, Tau² = 0.00**			**2660**	**3317**		**I² = 0.18, Tau² = 0.03**		
**Fixed**					**0.84 (0.72, 0.97)**						**0.94 (0.78, 1.13)**		
**Random**					**0.84 (0.72, 0.97)**						**0.95 (0.74, 1.20)**		

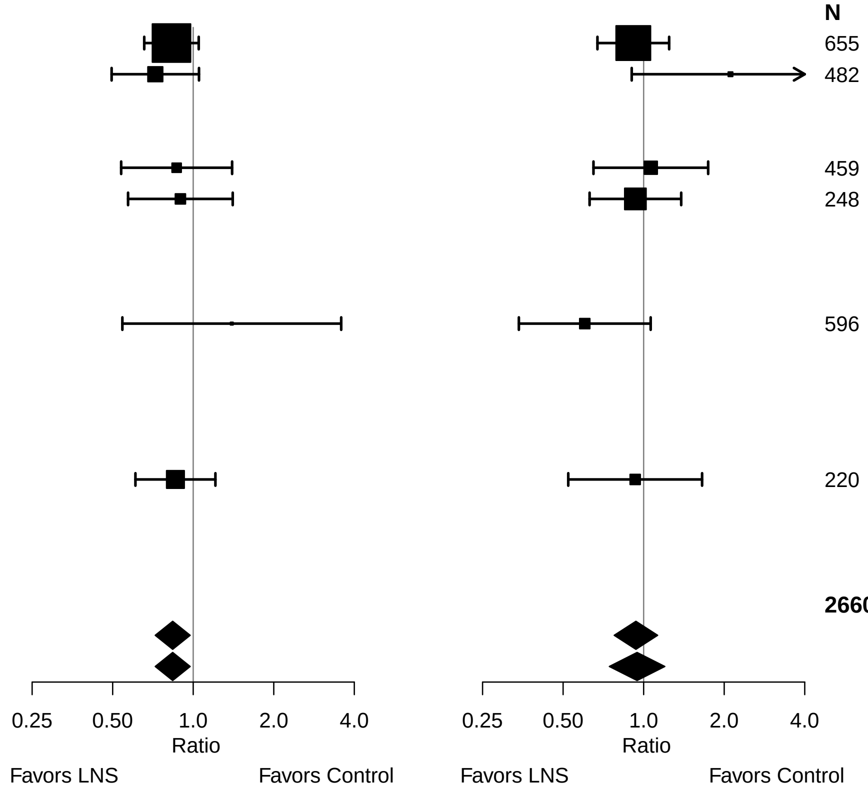

Supplemental figure 9H: Low MUAC prevalence ratio

9H5: Stratified by Home environment

P−for−interaction = 0.543
Ratio of PRs = 1.10 (0.81, 1.48)

Country	Trial	LNS N	Control N	Control Prevalence	At least median PR (95% CI)	Fixed W	Random W	LNS N	Control N	Control Prevalence	Less than median PR (95% CI)	Fixed W	Random W
Bangladesh	JiVitA−4 (34)												
Bangladesh	RDNS (35)	1192	582	4.1	1.06 (0.66, 1.70)	0.16	0.21	468	230	7.8	0.96 (0.49, 1.87)	0.11	0.13
Bangladesh	WASH−B (36)												
Burkina Faso	iLiNS−Zinc (37)	473	199	19.1	0.51 (0.37, 0.72)	0.31	0.27	272	175	16.6	0.77 (0.42, 1.41)	0.14	0.16
Burkina Faso	PROMIS (38)												
Burkina Faso	PROMIS CS (38)												
Ghana	GHANA (39)												
Ghana	iLiNS−DYADG (40)												
Haiti	HAITI (41)												
Kenya	WASH−B (42)	840	2782	4.3	0.89 (0.61, 1.31)	0.24	0.25	573	2179	6.0	0.64 (0.41, 0.99)	0.25	0.27
Madagascar	MAHAY (43)	905	889	8.8	0.86 (0.61, 1.21)	0.30	0.27	775	769	10.7	1.09 (0.80, 1.49)	0.50	0.44
Malawi	iLiNS−DYADM (44)												
Malawi	iLiNS−DOSE (45)												
Mali	PROMIS (46)												
Mali	PROMIS CS (46)												
Zimbabwe	SHINE (HIV−) (47)												
Zimbabwe	SHINE (HIV+) (48)												
		**3410**	**4452**		**I² = 0.64, Tau² = 0.06**			**2088**	**3353**		**I² = 0.25, Tau² = 0.01**		
**Fixed**					**0.76 (0.63, 0.92)**						**0.89 (0.72, 1.12)**		
**Random**					**0.79 (0.58, 1.07)**						**0.88 (0.68, 1.13)**		

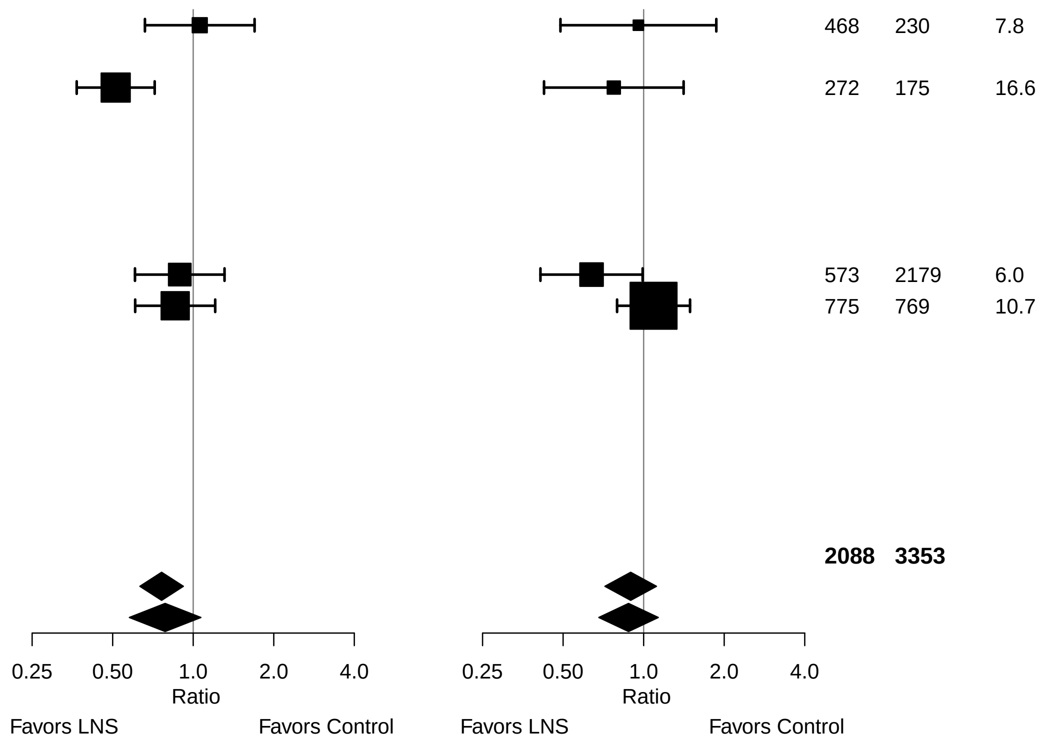

Supplemental figure 9H: Low MUAC prevalence ratio

9H6: Stratified by Season at the time of assessment

P−for−interaction = 0.301
Ratio of PRs = 0.89 (0.72, 1.11)

Country	Trial	Dry LNS N	Dry Control N	Dry Control Prevalence	Dry PR (95% CI)	Dry Fixed W	Dry Random W	Rainy LNS N	Rainy Control N	Rainy Control Prevalence	Rainy PR (95% CI)	Rainy Fixed W	Rainy Random W
Bangladesh	JiVitA−4 (34)	1570	673	14.8	0.91 (0.69, 1.20)	0.30	0.25	1327	598	15.7	0.81 (0.63, 1.04)	0.30	0.30
Bangladesh	RDNS (35)	739	359	2.5	1.30 (0.63, 2.67)	0.04	0.07	924	456	7.2	0.94 (0.63, 1.41)	0.12	0.12
Bangladesh	WASH−B (36)												
Burkina Faso	iLiNS−Zinc (37)	1399	477	19.3	0.61 (0.47, 0.79)	0.35	0.27	560	189	15.9	0.71 (0.48, 1.05)	0.13	0.13
Burkina Faso	PROMIS (38)	425	444	9.2	1.02 (0.67, 1.55)	0.13	0.16	429	468	6.8	0.82 (0.45, 1.48)	0.06	0.06
Burkina Faso	PROMIS CS (38)	222	190	14.2	1.08 (0.63, 1.85)	0.08	0.11	202	246	15.4	0.77 (0.57, 1.03)	0.23	0.23
Ghana	GHANA (39)												
Ghana	iLiNS−DYADG (40)												
Haiti	HAITI (41)												
Kenya	WASH−B (42)	349	1163	5.7	0.76 (0.47, 1.23)	0.10	0.13	1112	4022	5.4	0.69 (0.49, 0.98)	0.17	0.17
Madagascar	MAHAY (43)												
Malawi	iLiNS−DYADM (44)												
Malawi	iLiNS−DOSE (45)												
Mali	PROMIS (46)												
Mali	PROMIS CS (46)												
Zimbabwe	SHINE (HIV−) (47)												
Zimbabwe	SHINE (HIV+) (48)												
		**4704**	**3306**		**I² = 0.45, Tau² = 0.02**			**4554**	**5979**		**I² = 0.00, Tau² = 0.00**		
**Fixed**					**0.81 (0.70, 0.95)**						**0.78 (0.68, 0.90)**		
**Random**					**0.85 (0.69, 1.04)**						**0.78 (0.68, 0.90)**		

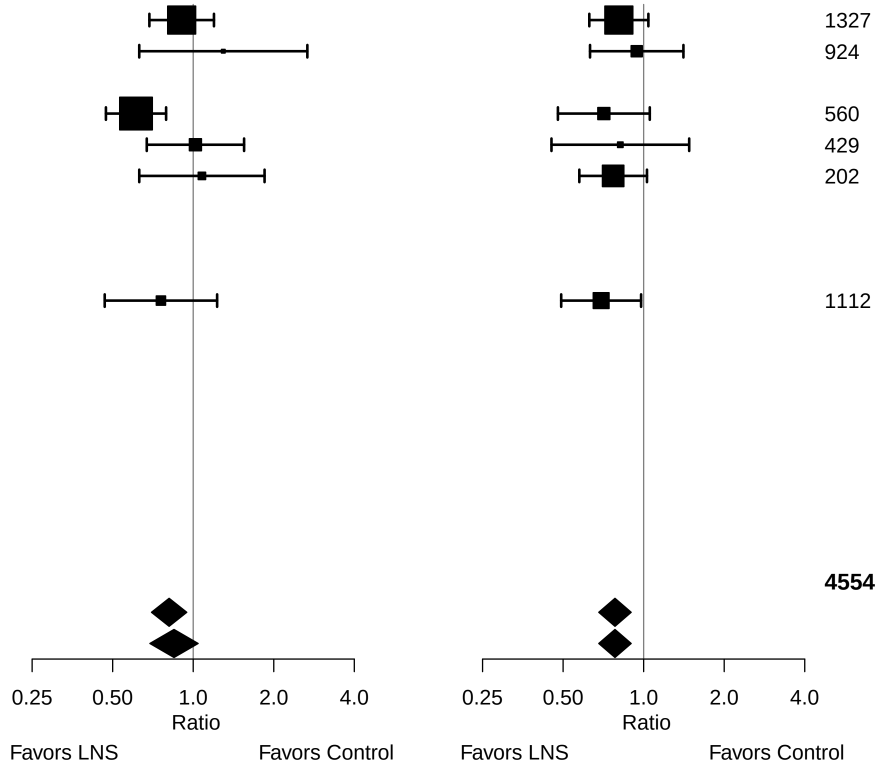

Supplemental figure 9I: Low MUAC prevalence difference

9I1: Stratified by Household socio-economic status

P−for−interaction = 0.735
Difference in PDs = 0.00 (−0.02, 0.01)

		At least median						Less than median					
**Country**	**Trial**	**LNS N**	**Control N**	**Control Prevalence**	**PD (95% CI)**	**Fixed W**	**Random W**	**LNS N**	**Control N**	**Control Prevalence**	**PD (95% CI)**	**Fixed W**	**Random W**
Bangladesh	JiVitA−4 (34)	1463	641	11.6	−0.01 (−0.04, 0.01)	0.11	0.11	1433	630	18.8	−0.03 (−0.06, 0.00)	0.12	0.12
Bangladesh	RDNS (35)	834	407	4.4	−0.01 (−0.03, 0.01)	0.16	0.16	829	408	5.9	0.01 (−0.02, 0.04)	0.14	0.13
Bangladesh	WASH−B (36)												
Burkina Faso	iLiNS−Zinc (37)	1232	300	16.0	−0.06 (−0.10, −0.02)	0.05	0.05	720	365	20.0	−0.07 (−0.11, −0.03)	0.08	0.10
Burkina Faso	PROMIS (38)	403	479	9.4	−0.02 (−0.07, 0.03)	0.03	0.03	451	433	6.5	0.01 (−0.02, 0.05)	0.11	0.11
Burkina Faso	PROMIS CS (38)	219	207	14.5	−0.03 (−0.09, 0.04)	0.02	0.02	205	229	15.3	0.00 (−0.06, 0.06)	0.04	0.06
Ghana	GHANA (39)												
Ghana	iLiNS−DYADG (40)												
Haiti	HAITI (41)												
Kenya	WASH−B (42)	827	2949	3.7	−0.01 (−0.02, 0.00)	0.46	0.46	632	2232	7.8	−0.02 (−0.05, 0.00)	0.24	0.15
Madagascar	MAHAY (43)	761	865	8.1	0.00 (−0.03, 0.03)	0.07	0.07	866	750	11.2	−0.01 (−0.05, 0.02)	0.11	0.12
Malawi	iLiNS−DYADM (44)												
Malawi	iLiNS−DOSE (45)												
Mali	PROMIS (46)	248	250	4.8	−0.01 (−0.05, 0.03)	0.04	0.04	246	246	6.9	−0.04 (−0.08, 0.00)	0.08	0.10
Mali	PROMIS CS (46)	505	463	12.7	−0.03 (−0.08, 0.01)	0.04	0.04	449	507	9.5	0.01 (−0.03, 0.05)	0.09	0.10
Zimbabwe	SHINE (HIV−) (47)												
Zimbabwe	SHINE (HIV+) (48)												
		**6492**	**6561**		**I² = 0.03, Tau² = 0.00**			**5831**	**5800**		**I² = 0.55, Tau² = 0.00**		
**Fixed**					**−0.01 (−0.02, −0.01)**						**−0.02 (−0.03, 0.00)**		
**Random**					**−0.01 (−0.02, −0.01)**						**−0.02 (−0.03, 0.00)**		

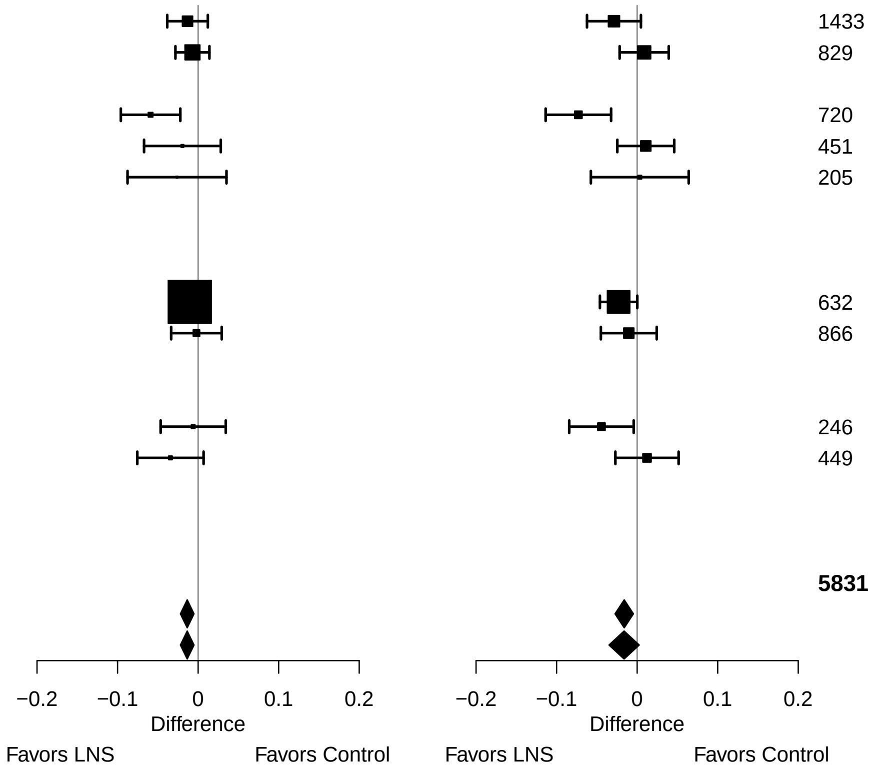

Supplemental figure 9I: Low MUAC prevalence difference

9I2: Stratified by Household food insecurity

P−for−interaction = 0.630
Difference in PDs = 0.00 (−0.01, 0.02)

Country	Trial	LNS N	Control N	Control Prevalence	Mild to secure PD (95% CI)	Fixed W	Random W	LNS N	Control N	Control Prevalence	Moderate to severe PD (95% CI)	Fixed W	Random W
Bangladesh	JiVitA−4 (34)	2054	882	14.6	−0.03 (−0.06, −0.01)	0.12	0.16	842	389	16.3	0.01 (−0.03, 0.05)	0.15	0.15
Bangladesh	RDNS (35)	1058	493	4.9	0.00 (−0.03, 0.03)	0.10	0.15	605	322	5.6	0.00 (−0.03, 0.04)	0.22	0.18
Bangladesh	WASH−B (36)												
Burkina Faso	iLiNS−Zinc (37)	1017	321	16.2	−0.06 (−0.10, −0.02)	0.04	0.08	935	344	20.1	−0.07 (−0.12, −0.03)	0.12	0.13
Burkina Faso	PROMIS (38)	496	547	7.3	0.01 (−0.02, 0.04)	0.07	0.12	358	365	9.0	−0.02 (−0.07, 0.02)	0.12	0.13
Burkina Faso	PROMIS CS (38)												
Ghana	GHANA (39)												
Ghana	iLiNS−DYADG (40)												
Haiti	HAITI (41)												
Kenya	WASH−B (42)	1303	4640	5.4	−0.02 (−0.03, −0.01)	0.51	0.24	156	528	6.2	0.00 (−0.04, 0.05)	0.14	0.14
Madagascar	MAHAY (43)	968	907	9.5	0.00 (−0.03, 0.03)	0.08	0.13	353	405	9.1	0.00 (−0.04, 0.05)	0.14	0.15
Malawi	iLiNS−DYADM (44)												
Malawi	iLiNS−DOSE (45)												
Mali	PROMIS (46)	330	341	5.0	−0.02 (−0.05, 0.01)	0.08	0.13	174	163	7.4	−0.03 (−0.08, 0.02)	0.11	0.12
Mali	PROMIS CS (46)												
Zimbabwe	SHINE (HIV−) (47)												
Zimbabwe	SHINE (HIV+) (48)												
		**7226**	**8131**		**I² = 0.43, Tau² = 0.00**			**3423**	**2516**		**I² = 0.40, Tau² = 0.00**		
**Fixed**					**−0.02 (−0.03, −0.01)**						**−0.01 (−0.03, 0.00)**		
**Random**					**−0.02 (−0.03, 0.00)**						**−0.01 (−0.04, 0.01)**		

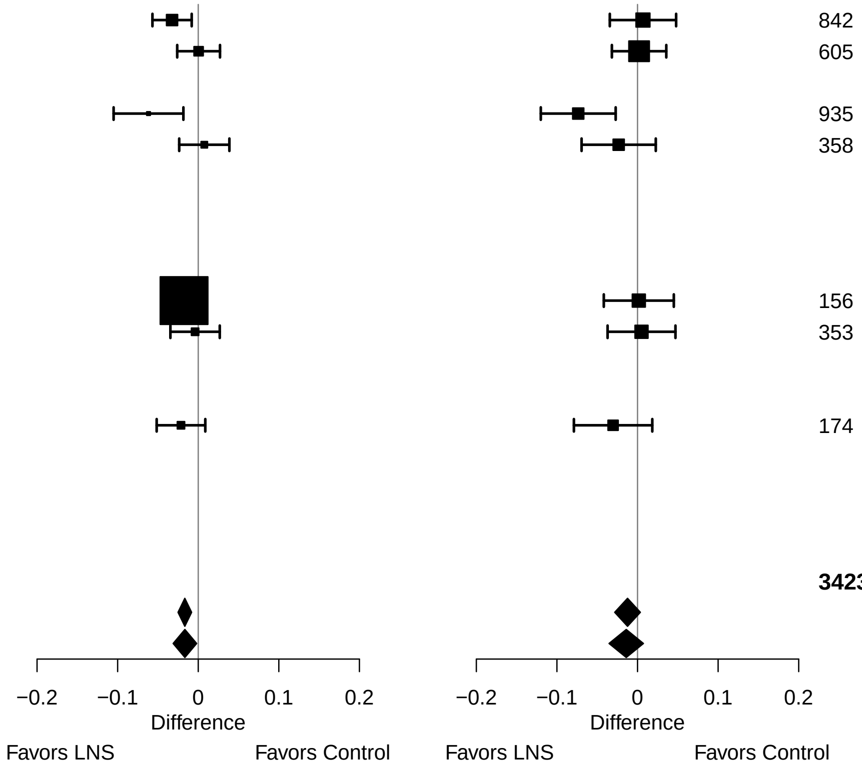

Supplemental figure 9I: Low MUAC prevalence difference

9I3: Stratified by Household source water quality

P−for−interaction = 0.039
Difference in PDs = −0.02 (−0.04, 0.00)

Country	Trial	LNS N	Control N	Control Prevalence	Improved PD (95% CI)	Fixed W	Random W	LNS N	Control N	Control Prevalence	Unimproved PD (95% CI)	Fixed W	Random W
Bangladesh	JiVitA−4 (34)												
Bangladesh	RDNS (35)												
Bangladesh	WASH−B (36)												
Burkina Faso	iLiNS−Zinc (37)	555	141	14.2	−0.03 (−0.07, 0.00)	0.12	0.13	1396	521	19.4	−0.08 (−0.11, −0.05)	0.18	0.17
Burkina Faso	PROMIS (38)	352	376	7.7	0.01 (−0.03, 0.05)	0.10	0.12	358	355	9.3	−0.03 (−0.07, 0.01)	0.11	0.13
Burkina Faso	PROMIS CS (38)	277	265	17.0	−0.04 (−0.10, 0.02)	0.05	0.06	147	171	11.7	0.03 (−0.06, 0.11)	0.03	0.05
Ghana	GHANA (39)												
Ghana	iLiNS−DYADG (40)												
Haiti	HAITI (41)												
Kenya	WASH−B (42)	449	1562	5.3	−0.01 (−0.04, 0.01)	0.33	0.26	248	701	5.8	−0.03 (−0.06, 0.01)	0.19	0.17
Madagascar	MAHAY (43)	415	452	7.7	0.03 (−0.01, 0.06)	0.12	0.13	1185	1157	10.1	−0.02 (−0.04, 0.01)	0.24	0.19
Malawi	iLiNS−DYADM (44)												
Malawi	iLiNS−DOSE (45)												
Mali	PROMIS (46)	249	258	5.0	−0.02 (−0.06, 0.02)	0.11	0.13	248	242	6.6	−0.03 (−0.07, 0.00)	0.16	0.16
Mali	PROMIS CS (46)	549	567	10.4	0.00 (−0.04, 0.03)	0.16	0.17	364	388	11.6	−0.01 (−0.06, 0.03)	0.09	0.12
Zimbabwe	SHINE (HIV−) (47)												
Zimbabwe	SHINE (HIV+) (48)												
		**2846**	**3621**		**I² = 0.18, Tau² = 0.00**			**3946**	**3535**		**I² = 0.50, Tau² = 0.00**		
**Fixed**					**−0.01 (−0.02, 0.00)**						**−0.03 (−0.05, −0.02)**		
**Random**					**−0.01 (−0.02, 0.01)**						**−0.03 (−0.05, −0.01)**		

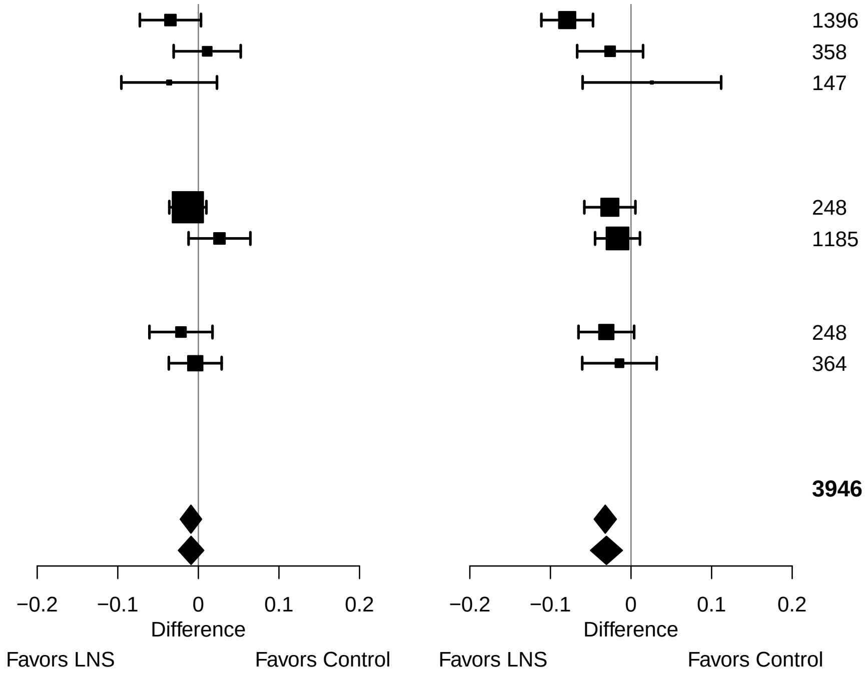

Supplemental figure 9I: Low MUAC prevalence difference

9I4: Stratified by Household sanitation

P−for−interaction = 0.165
Difference in PDs = 0.02 (−0.01, 0.04)

Country	Trial	Improved LNS N	Improved Control N	Improved Control Prevalence	Improved PD (95% CI)	Improved Fixed W	Improved Random W	Unimproved LNS N	Unimproved Control N	Unimproved Control Prevalence	Unimproved PD (95% CI)	Unimproved Fixed W	Unimproved Random W
Bangladesh	JiVitA−4 (34)	2241	974	13.6	−0.02 (−0.05, 0.00)	0.27	0.27	655	297	20.1	−0.01 (−0.06, 0.04)	0.10	0.12
Bangladesh	RDNS (35)	1179	573	5.8	−0.02 (−0.03, 0.00)	0.41	0.41	482	241	3.7	0.04 (0.00, 0.08)	0.15	0.17
Bangladesh	WASH−B (36)												
Burkina Faso	iLiNS−Zinc (37)												
Burkina Faso	PROMIS (38)	395	548	8.8	−0.01 (−0.05, 0.03)	0.10	0.10	459	359	7.0	0.00 (−0.03, 0.04)	0.18	0.20
Burkina Faso	PROMIS CS (38)	175	163	16.0	−0.02 (−0.08, 0.05)	0.03	0.03	248	273	14.3	−0.01 (−0.06, 0.04)	0.08	0.10
Ghana	GHANA (39)												
Ghana	iLiNS−DYADG (40)												
Haiti	HAITI (41)												
Kenya	WASH−B (42)	101	366	3.6	0.01 (−0.03, 0.06)	0.08	0.08	596	1898	5.8	−0.02 (−0.05, 0.00)	0.44	0.32
Madagascar	MAHAY (43)												
Malawi	iLiNS−DYADM (44)												
Malawi	iLiNS−DOSE (45)												
Mali	PROMIS (46)												
Mali	PROMIS CS (46)	703	696	10.8	−0.02 (−0.05, 0.02)	0.12	0.12	220	249	11.2	−0.01 (−0.07, 0.06)	0.06	0.08
Zimbabwe	SHINE (HIV−) (47)												
Zimbabwe	SHINE (HIV+) (48)												
		**4794**	**3320**		**I² = 0.00, Tau² = 0.00**			**2660**	**3317**		**I² = 0.42, Tau² = 0.00**		
**Fixed**					**−0.02 (−0.03, 0.00)**						**−0.01 (−0.02, 0.01)**		
**Random**					**−0.02 (−0.03, 0.00)**						**0.00 (−0.02, 0.02)**		

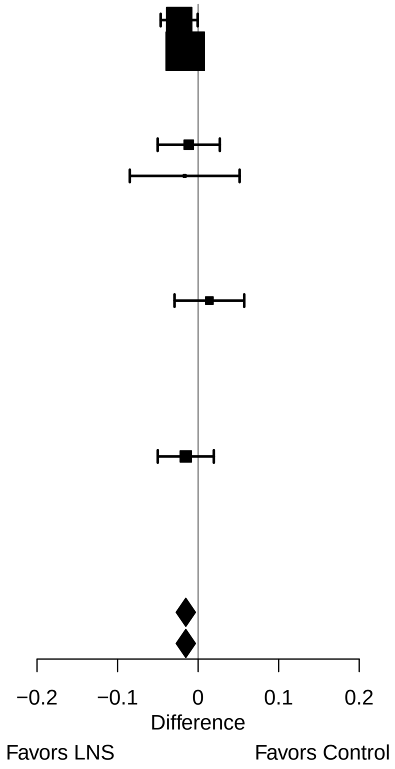

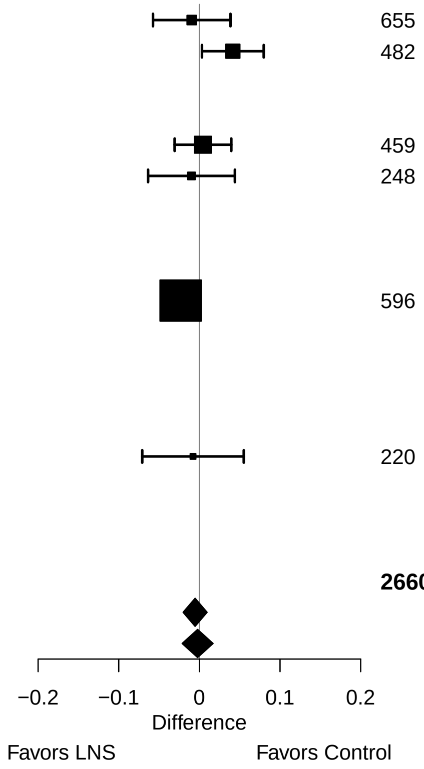

Supplemental figure 9I: Low MUAC prevalence difference

9I5: Stratified by Home environment

P−for−interaction = 0.688
Difference in PDs = 0.00 (−0.02, 0.01)

Country	Trial	At least median: LNS N	Control N	Control Prevalence	PD (95% CI)	Fixed W	Random W	Less than median: LNS N	Control N	Control Prevalence	PD (95% CI)	Fixed W	Random W
Bangladesh	JiVitA−4 (34)												
Bangladesh	RDNS (35)	1192	582	4.1	0.00 (−0.02, 0.02)	0.29	0.26	468	230	7.8	0.00 (−0.06, 0.05)	0.08	0.08
Bangladesh	WASH−B (36)												
Burkina Faso	iLiNS−Zinc (37)	473	199	19.1	−0.09 (−0.13, −0.05)	0.07	0.22	272	175	16.6	−0.04 (−0.13, 0.05)	0.03	0.03
Burkina Faso	PROMIS (38)												
Burkina Faso	PROMIS CS (38)												
Ghana	GHANA (39)												
Ghana	iLiNS−DYADG (40)												
Haiti	HAITI (41)												
Kenya	WASH−B (42)	840	2782	4.3	0.00 (−0.02, 0.01)	0.51	0.27	573	2179	6.0	−0.02 (−0.04, 0.00)	0.70	0.70
Madagascar	MAHAY (43)	905	889	8.8	−0.01 (−0.04, 0.02)	0.14	0.25	775	769	10.7	0.01 (−0.03, 0.04)	0.19	0.19
Malawi	iLiNS−DYADM (44)												
Malawi	iLiNS−DOSE (45)												
Mali	PROMIS (46)												
Mali	PROMIS CS (46)												
Zimbabwe	SHINE (HIV−) (47)												
Zimbabwe	SHINE (HIV+) (48)												
		**3410**	**4452**		**I² = 0.82, Tau² = 0.00**			**2088**	**3353**		**I² = 0.00, Tau² = 0.00**		
**Fixed**					**−0.01 (−0.02, 0.00)**						**−0.01 (−0.03, 0.00)**		
**Random**					**−0.02 (−0.06, 0.02)**						**−0.01 (−0.03, 0.00)**		

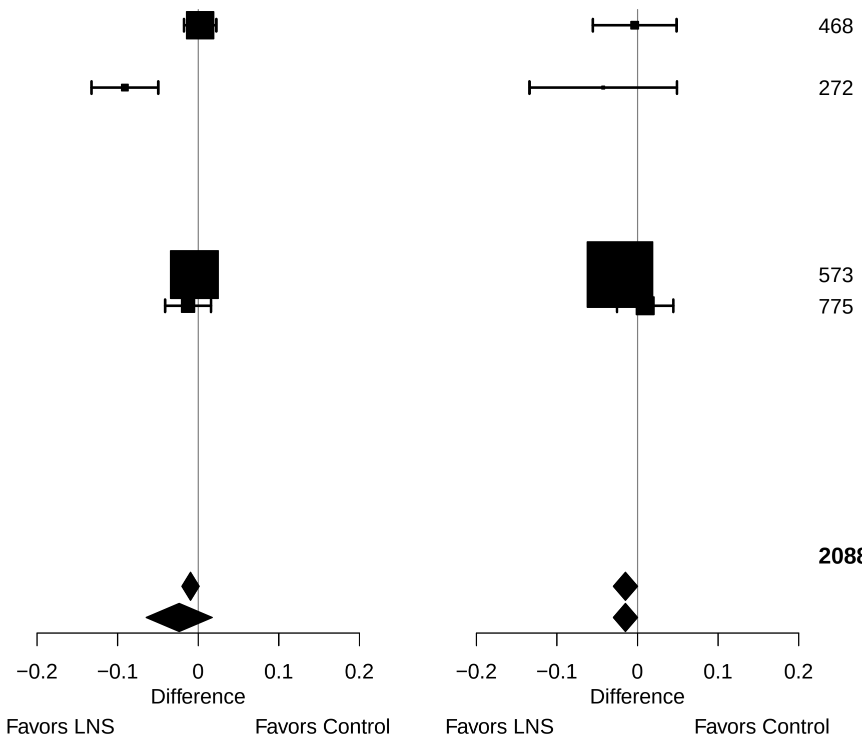

Supplemental figure 9I: Low MUAC prevalence difference

9I6: Stratified by Season at the time of assessment

P−for−interaction = 0.322
Difference in PDs = −0.01 (−0.03, 0.01)

Country	Trial	Dry LNS N	Dry Control N	Dry Control Prevalence	Dry PD (95% CI)	Dry Fixed W	Dry Random W	Rainy LNS N	Rainy Control N	Rainy Control Prevalence	Rainy PD (95% CI)	Rainy Fixed W	Rainy Random W
Bangladesh	JiVitA−4 (34)	1570	673	14.8	−0.01 (−0.04, 0.01)	0.17	0.19	1327	598	15.7	−0.03 (−0.06, 0.00)	0.11	0.11
Bangladesh	RDNS (35)	739	359	2.5	0.01 (−0.01, 0.03)	0.35	0.22	924	456	7.2	0.00 (−0.03, 0.02)	0.13	0.13
Bangladesh	WASH−B (36)												
Burkina Faso	iLiNS−Zinc (37)	1399	477	19.3	−0.07 (−0.11, −0.04)	0.11	0.16	560	189	15.9	−0.06 (−0.11, −0.01)	0.04	0.04
Burkina Faso	PROMIS (38)	425	444	9.2	0.00 (−0.04, 0.04)	0.09	0.15	429	468	6.8	−0.01 (−0.05, 0.02)	0.08	0.08
Burkina Faso	PROMIS CS (38)	222	190	14.2	0.01 (−0.07, 0.09)	0.02	0.07	202	246	15.4	−0.04 (−0.08, 0.01)	0.06	0.06
Ghana	GHANA (39)												
Ghana	iLiNS−DYADG (40)												
Haiti	HAITI (41)												
Kenya	WASH−B (42)	349	1163	5.7	−0.01 (−0.04, 0.01)	0.27	0.21	1112	4022	5.4	−0.02 (−0.03, 0.00)	0.58	0.58
Madagascar	MAHAY (43)												
Malawi	iLiNS−DYADM (44)												
Malawi	iLiNS−DOSE (45)												
Mali	PROMIS (46)												
Mali	PROMIS CS (46)												
Zimbabwe	SHINE (HIV−) (47)												
Zimbabwe	SHINE (HIV+) (48)												
		**4704**	**3306**		**I² = 0.67, Tau² = 0.00**			**4554**	**5979**		**I² = 0.00, Tau² = 0.00**		
**Fixed**					**−0.01 (−0.02, 0.00)**						**−0.02 (−0.03, −0.01)**		
**Random**					**−0.01 (−0.04, 0.01)**						**−0.02 (−0.03, −0.01)**		

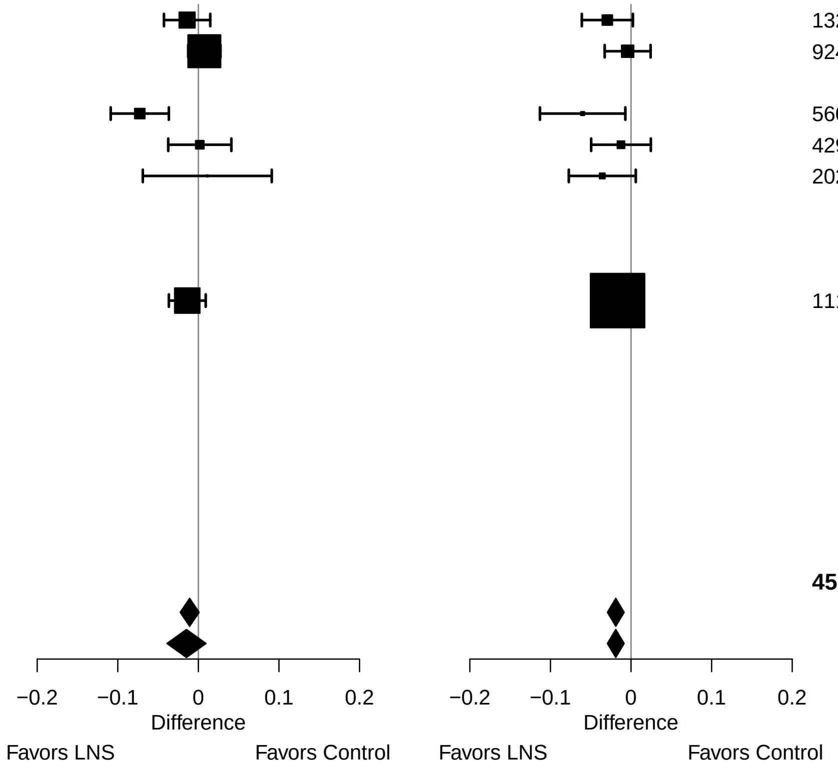

Supplemental figure 9J: Acute malnutrition prevalence ratio

9J1: Stratified by Household socio-economic status

P−for−interaction = 0.312
Ratio of PRs = 1.08 (0.93, 1.25)

		At least median						Less than median					
**Country**	**Trial**	**LNS N**	**Control N**	**Control Prevalence**	**PR (95% CI)**	**Fixed W**	**Random W**	**LNS N**	**Control N**	**Control Prevalence**	**PR (95% CI)**	**Fixed W**	**Random W**
Bangladesh	JiVitA−4 (34)	1401	606	17.7	0.82 (0.68, 0.98)	0.34	0.34	1381	610	24.4	0.94 (0.79, 1.12)	0.34	0.34
Bangladesh	RDNS (35)	834	407	13.5	0.86 (0.62, 1.19)	0.11	0.11	827	408	17.2	0.86 (0.66, 1.12)	0.14	0.14
Bangladesh	WASH−B (36)												
Burkina Faso	iLiNS−Zinc (37)	1224	299	18.4	0.58 (0.42, 0.79)	0.12	0.12	720	364	19.7	0.81 (0.60, 1.09)	0.11	0.11
Burkina Faso	PROMIS (38)	401	473	11.6	0.81 (0.53, 1.26)	0.06	0.06	445	428	11.7	0.85 (0.53, 1.34)	0.05	0.05
Burkina Faso	PROMIS CS (38)	219	206	19.4	0.80 (0.53, 1.20)	0.07	0.07	205	228	19.3	1.11 (0.80, 1.55)	0.09	0.09
Ghana	GHANA (39)												
Ghana	iLiNS−DYADG (40)	157	356	7.6	1.01 (0.52, 1.94)	0.03	0.03	190	334	8.7	0.73 (0.38, 1.39)	0.02	0.02
Haiti	HAITI (41)												
Kenya	WASH−B (42)	824	2914	2.3	0.75 (0.44, 1.27)	0.04	0.04	628	2199	4.4	0.69 (0.44, 1.07)	0.05	0.05
Madagascar	MAHAY (43)	760	865	7.5	1.09 (0.74, 1.60)	0.08	0.08	862	747	11.5	0.88 (0.60, 1.28)	0.07	0.07
Malawi	iLiNS−DYADM (44)												
Malawi	iLiNS−DOSE (45)												
Mali	PROMIS (46)												
Mali	PROMIS CS (46)	501	458	13.5	0.81 (0.57, 1.16)	0.09	0.09	443	501	12.6	0.97 (0.70, 1.34)	0.09	0.09
Zimbabwe	SHINE (HIV−) (47)	901	795	2.9	0.96 (0.57, 1.62)	0.04	0.04	826	851	4.1	0.62 (0.36, 1.07)	0.03	0.03
Zimbabwe	SHINE (HIV+) (48)	171	156	3.8	1.52 (0.53, 4.33)	0.01	0.01	157	165	5.5	0.93 (0.36, 2.42)	0.01	0.01
		**7393**	**7535**		**I² = 0.00, Tau² = 0.00**			**6684**	**6835**		**I² = 0.00, Tau² = 0.00**		
**Fixed**					**0.82 (0.73, 0.91)**						**0.89 (0.81, 0.98)**		
**Random**					**0.82 (0.73, 0.91)**						**0.89 (0.81, 0.98)**		

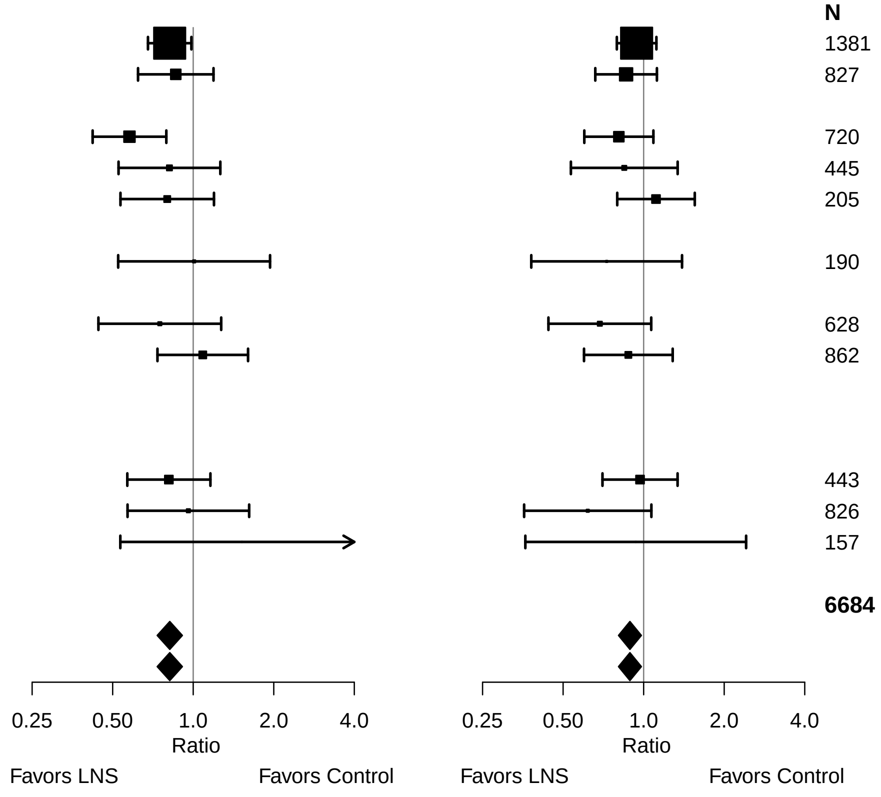

Supplemental figure 9J: Acute malnutrition prevalence ratio

9J2: Stratified by Household food insecurity

P−for−interaction = 0.565
Ratio of PRs = 0.95 (0.80, 1.13)

Country	Trial	LNS N	Control N	Control Prevalence	Mild to secure PR (95% CI)	Fixed W	Random W	LNS N	Control N	Control Prevalence	Moderate to severe PR (95% CI)	Fixed W	Random W
Bangladesh	JiVitA−4 (34)	1977	840	20.8	0.87 (0.74, 1.01)	0.46	0.46	805	376	21.5	0.95 (0.76, 1.20)	0.27	0.23
Bangladesh	RDNS (35)	1057	493	13.8	0.91 (0.69, 1.19)	0.15	0.15	604	322	17.7	0.81 (0.61, 1.08)	0.18	0.20
Bangladesh	WASH−B (36)												
Burkina Faso	iLiNS−Zinc (37)	1013	320	16.5	0.70 (0.46, 1.04)	0.07	0.07	931	343	21.6	0.66 (0.54, 0.80)	0.40	0.25
Burkina Faso	PROMIS (38)	494	543	10.9	0.99 (0.73, 1.33)	0.13	0.13	352	358	12.8	0.64 (0.36, 1.14)	0.04	0.10
Burkina Faso	PROMIS CS (38)												
Ghana	GHANA (39)												
Ghana	iLiNS−DYADG (40)	244	470	8.5	0.87 (0.51, 1.48)	0.04	0.04	102	219	7.3	0.81 (0.32, 2.00)	0.02	0.05
Haiti	HAITI (41)												
Kenya	WASH−B (42)												
Madagascar	MAHAY (43)	964	905	10.1	0.94 (0.67, 1.32)	0.10	0.10	352	405	8.6	1.12 (0.71, 1.76)	0.07	0.13
Malawi	iLiNS−DYADM (44)												
Malawi	iLiNS−DOSE (45)												
Mali	PROMIS (46)												
Mali	PROMIS CS (46)												
Zimbabwe	SHINE (HIV−) (47)	1382	1292	2.9	0.99 (0.63, 1.54)	0.06	0.06	294	322	6.2	0.33 (0.13, 0.81)	0.02	0.05
Zimbabwe	SHINE (HIV+) (48)												
		**7131**	**4863**		**I² = 0.00, Tau² = 0.00**			**3440**	**2345**		**I² = 0.52, Tau² = 0.04**		
**Fixed**					**0.89 (0.80, 0.99)**						**0.78 (0.69, 0.88)**		
**Random**					**0.89 (0.80, 0.99)**						**0.78 (0.63, 0.97)**		

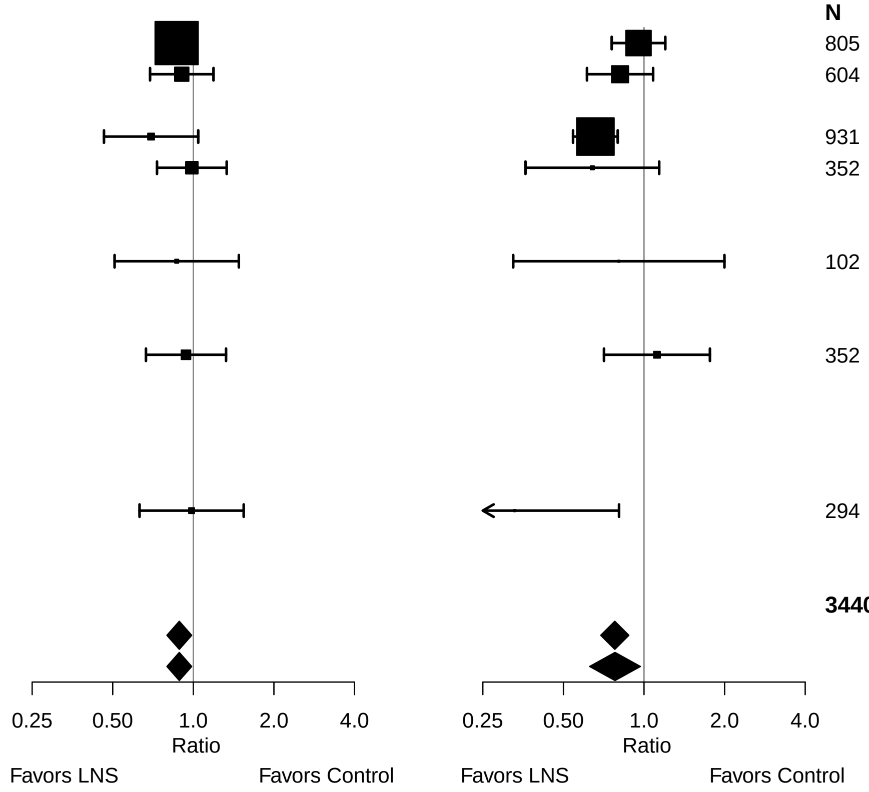

Supplemental figure 9J: Acute malnutrition prevalence ratio

9J3: Stratified by Household source water quality

P−for−interaction = 0.789
Ratio of PRs = 1.03 (0.82, 1.30)

Country	Trial	Improved LNS N	Improved Control N	Improved Control Prevalence	Improved PR (95% CI)	Improved Fixed W	Improved Random W	Unimproved LNS N	Unimproved Control N	Unimproved Control Prevalence	Unimproved PR (95% CI)	Unimproved Fixed W	Unimproved Random W
Bangladesh	JiVitA−4 (34)												
Bangladesh	RDNS (35)												
Bangladesh	WASH−B (36)												
Burkina Faso	iLiNS−Zinc (37)	550	141	17.7	0.53 (0.34, 0.82)	0.13	0.14	1393	519	19.6	0.71 (0.56, 0.90)	0.38	0.34
Burkina Faso	PROMIS (38)	349	372	11.3	0.89 (0.65, 1.22)	0.26	0.24	353	349	12.9	0.68 (0.42, 1.11)	0.09	0.10
Burkina Faso	PROMIS CS (38)	277	265	21.1	0.82 (0.55, 1.22)	0.16	0.17	147	169	16.6	1.23 (0.77, 1.98)	0.10	0.11
Ghana	GHANA (39)												
Ghana	iLiNS−DYADG (40)												
Haiti	HAITI (41)												
Kenya	WASH−B (42)												
Madagascar	MAHAY (43)	414	451	8.0	1.18 (0.69, 2.01)	0.09	0.11	1181	1155	9.9	0.91 (0.67, 1.24)	0.22	0.23
Malawi	iLiNS−DYADM (44)												
Malawi	iLiNS−DOSE (45)												
Mali	PROMIS (46)												
Mali	PROMIS CS (46)	544	562	12.5	0.86 (0.64, 1.15)	0.30	0.27	361	382	13.6	0.94 (0.66, 1.33)	0.18	0.19
Zimbabwe	SHINE (HIV−) (47)	517	482	3.7	0.83 (0.42, 1.63)	0.06	0.07	273	300	3.7	0.70 (0.30, 1.64)	0.03	0.04
Zimbabwe	SHINE (HIV+) (48)												
		**2651**	**2273**		**I² = 0.16, Tau² = 0.01**			**3708**	**2874**		**I² = 0.14, Tau² = 0.01**		
**Fixed**					**0.83 (0.70, 0.97)**						**0.83 (0.72, 0.96)**		
**Random**					**0.82 (0.69, 0.99)**						**0.84 (0.71, 0.98)**		

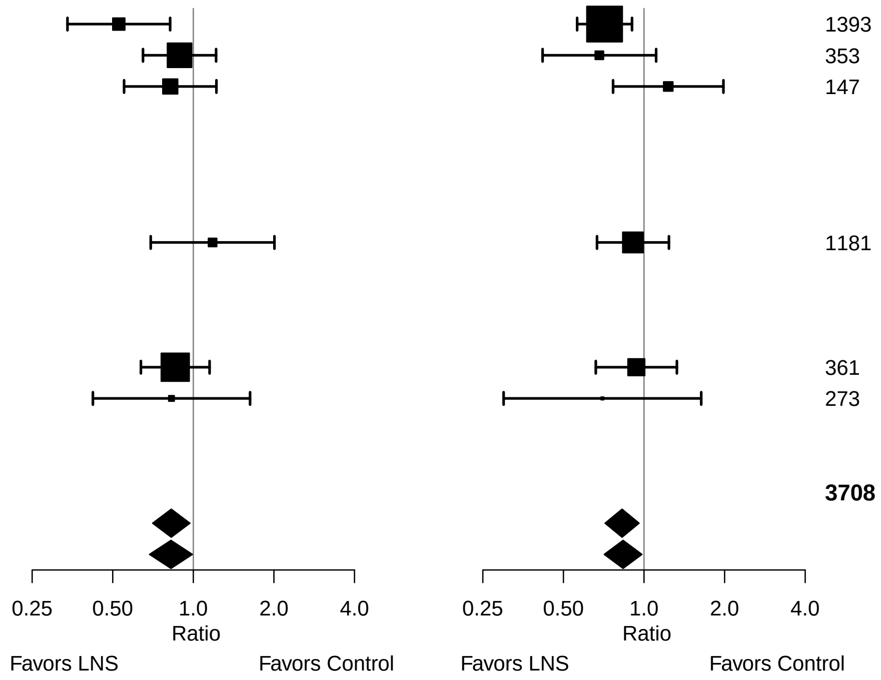

Supplemental figure 9J: Acute malnutrition prevalence ratio

9J4: Stratified by Household sanitation

P−for−interaction = 0.013
Ratio of PRs = 1.25 (1.05, 1.49)

Country	Trial	Improved LNS N	Improved Control N	Improved Control Prevalence	Improved PR (95% CI)	Improved Fixed W	Improved Random W	Unimproved LNS N	Unimproved Control N	Unimproved Control Prevalence	Unimproved PR (95% CI)	Unimproved Fixed W	Unimproved Random W
Bangladesh	JiVitA−4 (34)	2151	931	20.1	0.86 (0.74, 1.01)	0.50	0.50	631	285	24.0	0.99 (0.79, 1.24)	0.37	0.37
Bangladesh	RDNS (35)	1179	573	15.5	0.73 (0.57, 0.92)	0.21	0.21	480	241	14.9	1.20 (0.87, 1.66)	0.18	0.18
Bangladesh	WASH−B (36)												
Burkina Faso	iLiNS−Zinc (37)												
Burkina Faso	PROMIS (38)	389	544	12.5	0.78 (0.53, 1.16)	0.08	0.08	457	353	10.5	0.92 (0.59, 1.42)	0.10	0.10
Burkina Faso	PROMIS CS (38)	175	163	19.6	0.87 (0.58, 1.32)	0.07	0.07	248	271	19.2	1.01 (0.74, 1.37)	0.21	0.21
Ghana	GHANA (39)												
Ghana	iLiNS−DYADG (40)												
Haiti	HAITI (41)												
Kenya	WASH−B (42)												
Madagascar	MAHAY (43)												
Malawi	iLiNS−DYADM (44)												
Malawi	iLiNS−DOSE (45)												
Mali	PROMIS (46)												
Mali	PROMIS CS (46)	696	692	13.6	0.85 (0.63, 1.14)	0.13	0.13	217	242	10.7	0.86 (0.55, 1.33)	0.10	0.10
Zimbabwe	SHINE (HIV−) (47)	292	242	4.1	0.41 (0.16, 1.09)	0.01	0.01	495	539	3.5	1.03 (0.52, 2.05)	0.04	0.04
Zimbabwe	SHINE (HIV+) (48)												
		**4882**	**3145**		**I^2 = 0.00, Tau^2 = 0.00**			**2528**	**1931**		**I^2 = 0.00, Tau^2 = 0.00**		
**Fixed**					**0.82 (0.73, 0.91)**						**1.01 (0.88, 1.16)**		
**Random**					**0.82 (0.73, 0.91)**						**1.01 (0.88, 1.16)**		

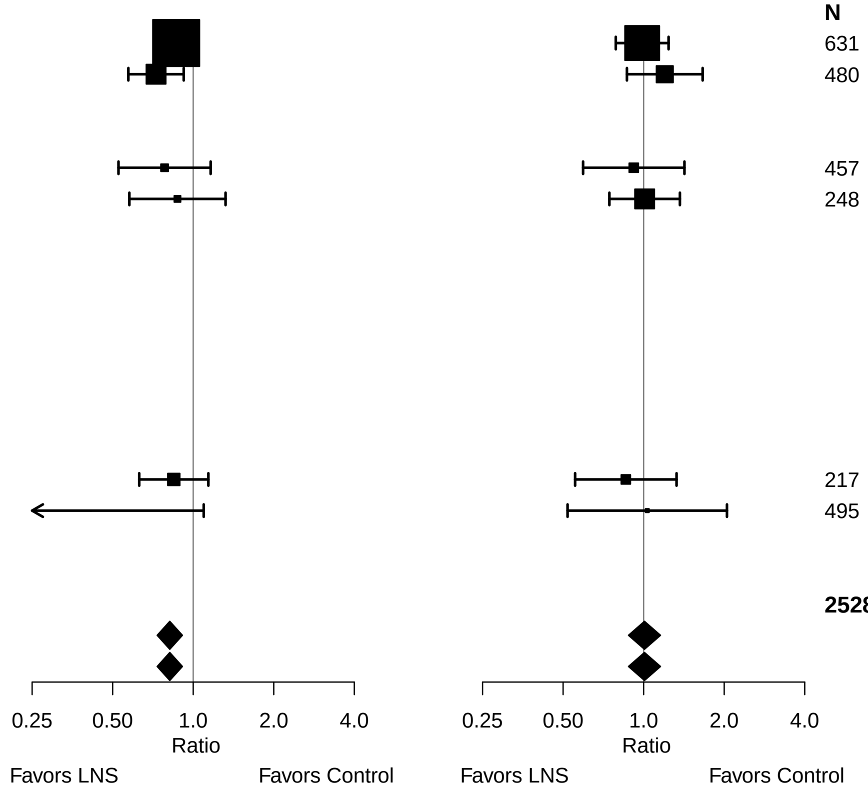

Supplemental figure 9J: Acute malnutrition prevalence ratio

9J5: Stratified by Home environment

P−for−interaction = 0.310
Ratio of PRs = 1.14 (0.89, 1.45)

Country	Trial	At least median: LNS N	Control N	Control Prevalence	PR (95% CI)	Fixed W	Random W	Less than median: LNS N	Control N	Control Prevalence	PR (95% CI)	Fixed W	Random W
Bangladesh	JiVitA−4 (34)												
Bangladesh	RDNS (35)	1192	582	14.9	0.78 (0.60, 1.01)	0.36	0.26	466	230	15.7	1.08 (0.79, 1.49)	0.35	0.30
Bangladesh	WASH−B (36)												
Burkina Faso	iLiNS−Zinc (37)	473	199	18.6	0.50 (0.38, 0.66)	0.30	0.25	271	174	21.7	0.79 (0.50, 1.25)	0.17	0.20
Burkina Faso	PROMIS (38)												
Burkina Faso	PROMIS CS (38)												
Ghana	GHANA (39)												
Ghana	iLiNS−DYADG (40)	213	409	7.1	1.06 (0.59, 1.91)	0.07	0.13	119	248	9.7	0.43 (0.17, 1.11)	0.04	0.07
Haiti	HAITI (41)												
Kenya	WASH−B (42)	834	2756	2.4	0.90 (0.56, 1.45)	0.10	0.16	572	2149	3.7	0.61 (0.34, 1.11)	0.10	0.14
Madagascar	MAHAY (43)	902	889	7.3	0.97 (0.66, 1.43)	0.16	0.20	773	766	11.9	0.99 (0.72, 1.37)	0.34	0.29
Malawi	iLiNS−DYADM (44)												
Malawi	iLiNS−DOSE (45)												
Mali	PROMIS (46)												
Mali	PROMIS CS (46)												
Zimbabwe	SHINE (HIV−) (47)												
Zimbabwe	SHINE (HIV+) (48)												
		**3614**	**4835**		**I² = 0.66, Tau² = 0.05**			**2201**	**3567**		**I² = 0.32, Tau² = 0.03**		
**Fixed**					**0.73 (0.63, 0.85)**						**0.91 (0.75, 1.10)**		
**Random**					**0.78 (0.59, 1.02)**						**0.86 (0.66, 1.12)**		

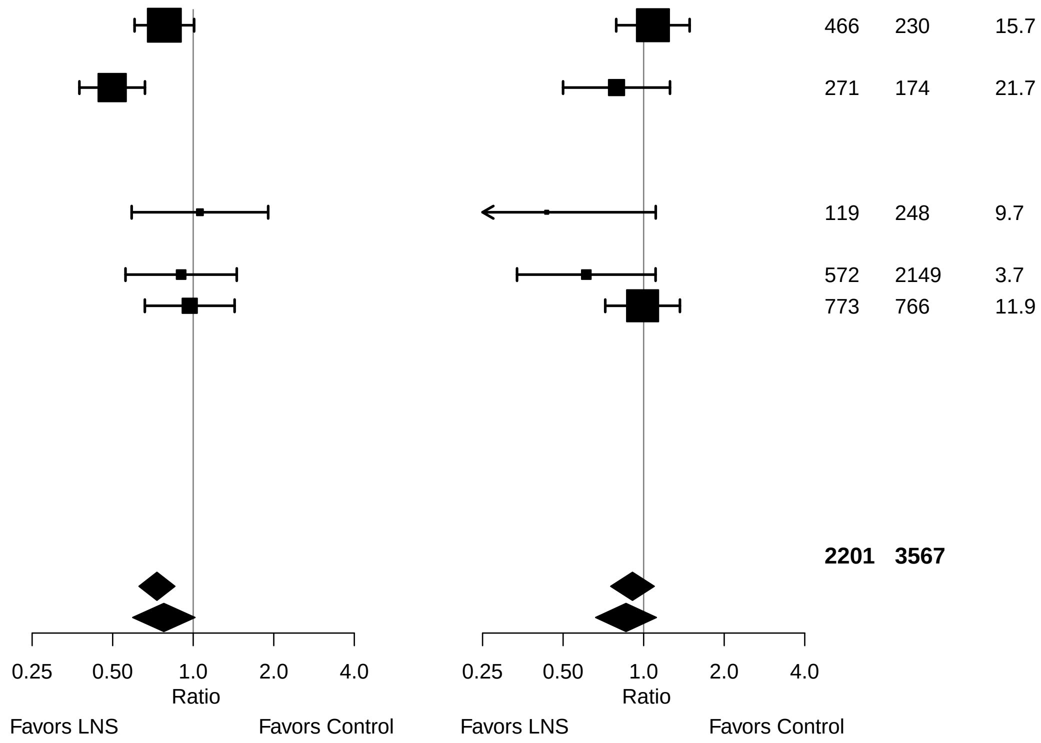

Supplemental figure 9J: Acute malnutrition prevalence ratio

9J6: Stratified by Season at the time of assessment

P−for−interaction = 0.172
Ratio of PRs = 1.12 (0.95, 1.32)

Country	Trial	Dry LNS N	Dry Control N	Dry Control Prevalence	Dry PR (95% CI)	Dry Fixed W	Dry Random W	Rainy LNS N	Rainy Control N	Rainy Control Prevalence	Rainy PR (95% CI)	Rainy Fixed W	Rainy Random W
Bangladesh	JiVitA−4 (34)	1520	648	18.7	0.86 (0.71, 1.05)	0.39	0.26	1263	568	23.7	0.92 (0.77, 1.10)	0.40	0.40
Bangladesh	RDNS (35)	738	359	13.6	0.81 (0.59, 1.12)	0.14	0.17	923	456	16.7	0.89 (0.69, 1.15)	0.20	0.20
Bangladesh	WASH−B (36)												
Burkina Faso	iLiNS−Zinc (37)	1393	477	20.1	0.62 (0.48, 0.78)	0.25	0.22	558	187	17.0	0.87 (0.62, 1.22)	0.11	0.11
Burkina Faso	PROMIS (38)	418	441	12.0	0.82 (0.53, 1.25)	0.08	0.12	428	460	11.3	0.85 (0.56, 1.28)	0.08	0.08
Burkina Faso	PROMIS CS (38)	222	190	18.4	1.00 (0.64, 1.57)	0.07	0.11	202	244	20.1	0.91 (0.65, 1.27)	0.12	0.12
Ghana	GHANA (39)												
Ghana	iLiNS−DYADG (40)												
Haiti	HAITI (41)												
Kenya	WASH−B (42)	346	1146	3.8	0.62 (0.33, 1.16)	0.04	0.07	1108	3971	3.0	0.74 (0.48, 1.13)	0.07	0.07
Madagascar	MAHAY (43)												
Malawi	iLiNS−DYADM (44)												
Malawi	iLiNS−DOSE (45)	415	134	4.5	1.64 (0.74, 3.64)	0.02	0.04	275	106	7.5	1.18 (0.56, 2.48)	0.02	0.02
Mali	PROMIS (46)												
Mali	PROMIS CS (46)												
Zimbabwe	SHINE (HIV−) (47)												
Zimbabwe	SHINE (HIV+) (48)												
		**5052**	**3395**		**I² = 0.39, Tau² = 0.02**			**4757**	**5992**		**I² = 0.00, Tau² = 0.00**		
**Fixed**					**0.79 (0.70, 0.89)**						**0.89 (0.80, 1.00)**		
**Random**					**0.81 (0.67, 0.96)**						**0.89 (0.80, 1.00)**		

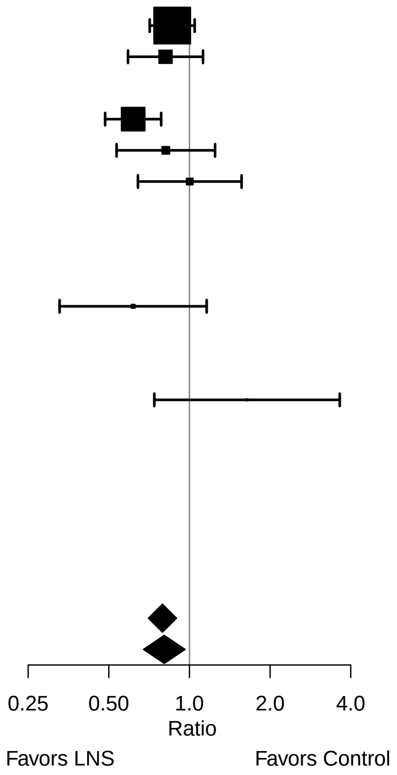

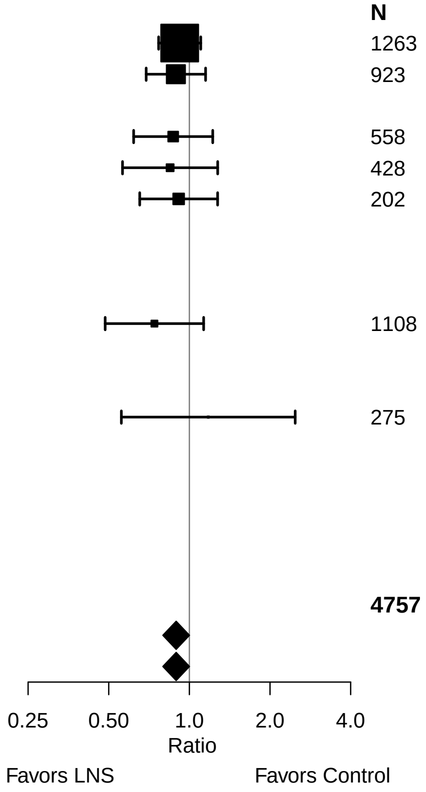

Supplemental figure 9K: Acute malnutrition prevalence difference

9K1: Stratified by Household socio-economic status

P−for−interaction = 0.197
Difference in PDs = −0.01 (−0.02, 0.00)

		At least median						Less than median					
**Country**	**Trial**	**LNS N**	**Control N**	**Control Prevalence**	**PD (95% CI)**	**Fixed W**	**Random W**	**LNS N**	**Control N**	**Control Prevalence**	**PD (95% CI)**	**Fixed W**	**Random W**
Bangladesh	JiVitA−4 (34)	1401	606	17.7	−0.02 (−0.05, 0.01)	0.06	0.10	1381	610	24.4	−0.02 (−0.06, 0.01)	0.06	0.06
Bangladesh	RDNS (35)	834	407	13.5	−0.02 (−0.06, 0.02)	0.03	0.06	827	408	17.2	−0.02 (−0.07, 0.02)	0.04	0.04
Bangladesh	WASH−B (36)												
Burkina Faso	iLiNS−Zinc (37)	1224	299	18.4	−0.07 (−0.12, −0.02)	0.02	0.05	720	364	19.7	−0.05 (−0.10, 0.00)	0.03	0.03
Burkina Faso	PROMIS (38)	401	473	11.6	−0.02 (−0.07, 0.02)	0.02	0.05	445	428	11.7	−0.02 (−0.07, 0.03)	0.03	0.03
Burkina Faso	PROMIS CS (38)	219	206	19.4	−0.04 (−0.11, 0.03)	0.01	0.03	205	228	19.3	0.02 (−0.05, 0.09)	0.02	0.02
Ghana	GHANA (39)												
Ghana	iLiNS−DYADG (40)	157	356	7.6	0.00 (−0.05, 0.05)	0.02	0.04	190	334	8.7	−0.02 (−0.07, 0.02)	0.04	0.04
Haiti	HAITI (41)												
Kenya	WASH−B (42)	824	2914	2.3	−0.01 (−0.02, 0.00)	0.52	0.27	628	2199	4.4	−0.01 (−0.03, 0.00)	0.39	0.39
Madagascar	MAHAY (43)	760	865	7.5	0.01 (−0.02, 0.04)	0.05	0.10	862	747	11.5	−0.01 (−0.06, 0.03)	0.05	0.05
Malawi	iLiNS−DYADM (44)												
Malawi	iLiNS−DOSE (45)												
Mali	PROMIS (46)												
Mali	PROMIS CS (46)	501	458	13.5	−0.03 (−0.07, 0.02)	0.03	0.06	443	501	12.6	0.00 (−0.04, 0.04)	0.05	0.05
Zimbabwe	SHINE (HIV−) (47)	901	795	2.9	0.00 (−0.02, 0.01)	0.22	0.21	826	851	4.1	−0.02 (−0.03, 0.00)	0.26	0.26
Zimbabwe	SHINE (HIV+) (48)	171	156	3.8	0.02 (−0.03, 0.07)	0.02	0.04	157	165	5.5	0.00 (−0.05, 0.05)	0.03	0.03
		**7393**	**7535**		**I² = 0.20, Tau² = 0.00**			**6684**	**6835**		**I² = 0.00, Tau² = 0.00**		
**Fixed**					**−0.01 (−0.01, 0.00)**						**−0.02 (−0.02, −0.01)**		
**Random**					**−0.01 (−0.02, 0.00)**						**−0.02 (−0.02, −0.01)**		

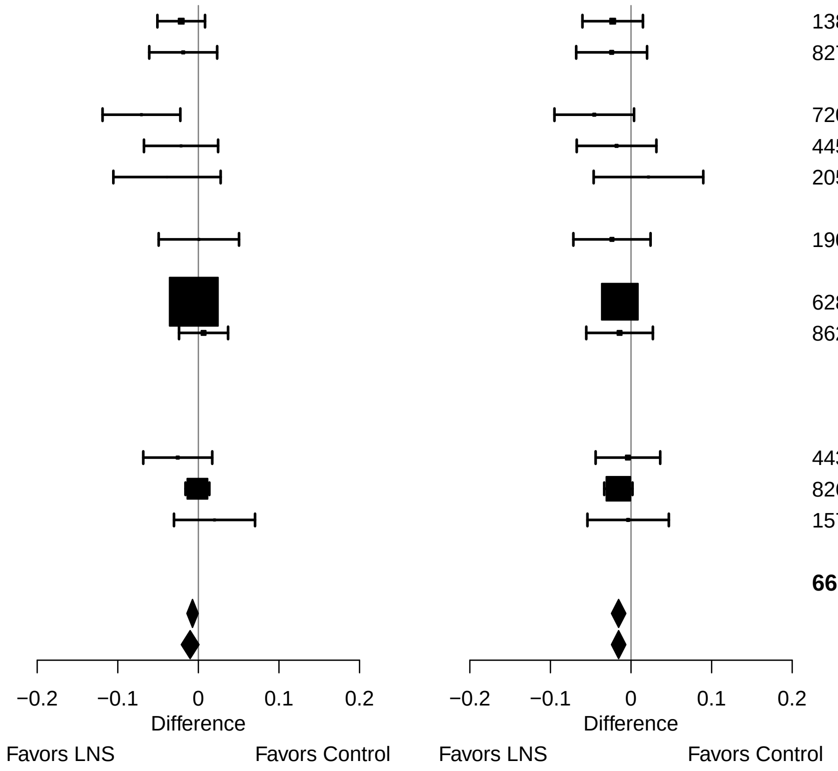

Supplemental figure 9K: Acute malnutrition prevalence difference

9K2: Stratified by Household food insecurity

P−for−interaction = 0.074
Difference in PDs = −0.02 (−0.04, 0.00)

Country	Trial	LNS N	Control N	Control Prevalence	Mild to secure PD (95% CI)	Fixed W	Random W	LNS N	Control N	Control Prevalence	Moderate to severe PD (95% CI)	Fixed W	Random W
Bangladesh	JiVitA−4 (34)	1977	840	20.8	−0.03 (−0.06, 0.00)	0.11	0.11	805	376	21.5	0.00 (−0.05, 0.04)	0.10	0.13
Bangladesh	RDNS (35)	1057	493	13.8	−0.01 (−0.05, 0.02)	0.07	0.07	604	322	17.7	−0.03 (−0.08, 0.01)	0.10	0.13
Bangladesh	WASH−B (36)												
Burkina Faso	iLiNS−Zinc (37)	1013	320	16.5	−0.05 (−0.11, 0.01)	0.02	0.02	931	343	21.6	−0.07 (−0.10, −0.05)	0.35	0.21
Burkina Faso	PROMIS (38)	494	543	10.9	0.00 (−0.03, 0.03)	0.09	0.09	352	358	12.8	−0.05 (−0.11, 0.01)	0.06	0.10
Burkina Faso	PROMIS CS (38)												
Ghana	GHANA (39)												
Ghana	iLiNS−DYADG (40)	244	470	8.5	−0.01 (−0.05, 0.03)	0.05	0.05	102	219	7.3	−0.01 (−0.07, 0.05)	0.06	0.10
Haiti	HAITI (41)												
Kenya	WASH−B (42)												
Madagascar	MAHAY (43)	964	905	10.1	−0.01 (−0.04, 0.03)	0.08	0.08	352	405	8.6	0.01 (−0.03, 0.05)	0.12	0.15
Malawi	iLiNS−DYADM (44)												
Malawi	iLiNS−DOSE (45)												
Mali	PROMIS (46)												
Mali	PROMIS CS (46)												
Zimbabwe	SHINE (HIV−) (47)	1382	1292	2.9	0.00 (−0.01, 0.01)	0.58	0.58	294	322	6.2	−0.04 (−0.07, −0.01)	0.21	0.18
Zimbabwe	SHINE (HIV+) (48)												
		**7131**	**4863**		**I² = 0.00, Tau² = 0.00**			**3440**	**2345**		**I² = 0.60, Tau² = 0.00**		
**Fixed**					**−0.01 (−0.02, 0.00)**						**−0.04 (−0.05, −0.03)**		
**Random**					**−0.01 (−0.02, 0.00)**						**−0.03 (−0.05, −0.01)**		

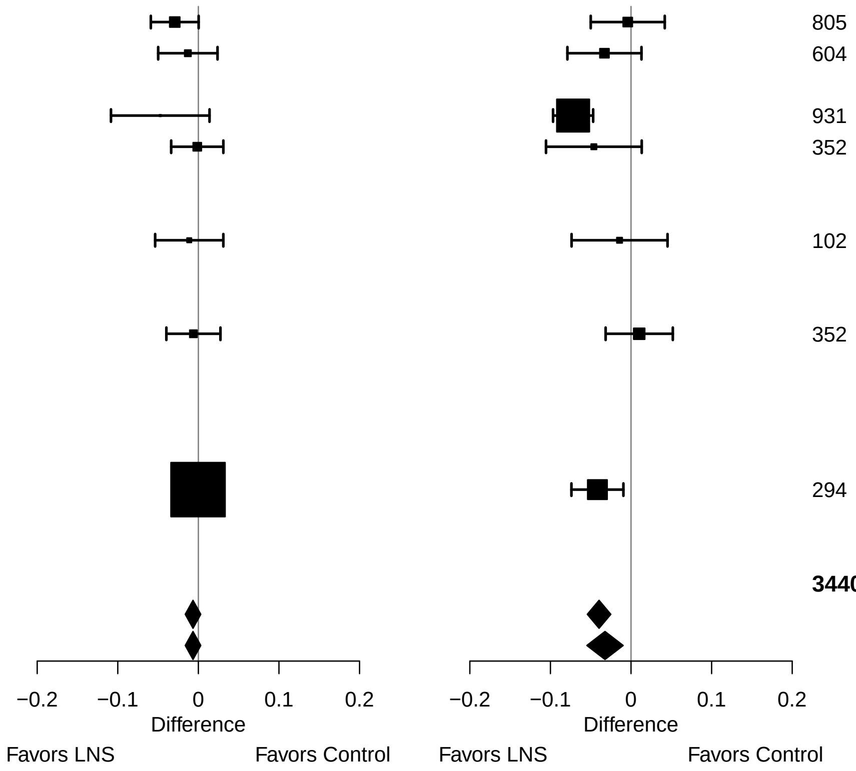

Supplemental figure 9K: Acute malnutrition prevalence difference

9K3: Stratified by Household source water quality

P−for−interaction = 0.574
Difference in PDs = −0.01 (−0.03, 0.02)

Country	Trial	LNS N	Control N	Control Prevalence	Improved PD (95% CI)	Fixed W	Random W	LNS N	Control N	Control Prevalence	Unimproved PD (95% CI)	Fixed W	Random W
Bangladesh	JiVitA−4 (34)												
Bangladesh	RDNS (35)												
Bangladesh	WASH−B (36)												
Burkina Faso	iLiNS−Zinc (37)	550	141	17.7	−0.06 (−0.12, −0.01)	0.07	0.07	1393	519	19.6	−0.06 (−0.10, −0.02)	0.16	0.18
Burkina Faso	PROMIS (38)	349	372	11.3	−0.01 (−0.05, 0.02)	0.19	0.19	353	349	12.9	−0.04 (−0.09, 0.01)	0.08	0.12
Burkina Faso	PROMIS CS (38)	277	265	21.1	−0.04 (−0.11, 0.04)	0.04	0.04	147	169	16.6	0.04 (−0.05, 0.12)	0.03	0.05
Ghana	GHANA (39)												
Ghana	iLiNS−DYADG (40)												
Haiti	HAITI (41)												
Kenya	WASH−B (42)												
Madagascar	MAHAY (43)	414	451	8.0	0.01 (−0.03, 0.06)	0.10	0.10	1181	1155	9.9	−0.01 (−0.04, 0.02)	0.28	0.24
Malawi	iLiNS−DYADM (44)												
Malawi	iLiNS−DOSE (45)												
Mali	PROMIS (46)												
Mali	PROMIS CS (46)	544	562	12.5	−0.02 (−0.05, 0.02)	0.19	0.19	361	382	13.6	−0.01 (−0.06, 0.04)	0.11	0.14
Zimbabwe	SHINE (HIV−) (47)	517	482	3.7	−0.01 (−0.03, 0.02)	0.41	0.41	273	300	3.7	−0.01 (−0.04, 0.02)	0.34	0.26
Zimbabwe	SHINE (HIV+) (48)												
		**2651**	**2273**		**I² = 0.05, Tau² = 0.00**			**3708**	**2874**		**I² = 0.38, Tau² = 0.00**		
**Fixed**					**−0.01 (−0.03, 0.00)**						**−0.02 (−0.03, 0.00)**		
**Random**					**−0.01 (−0.03, 0.00)**						**−0.02 (−0.04, 0.00)**		

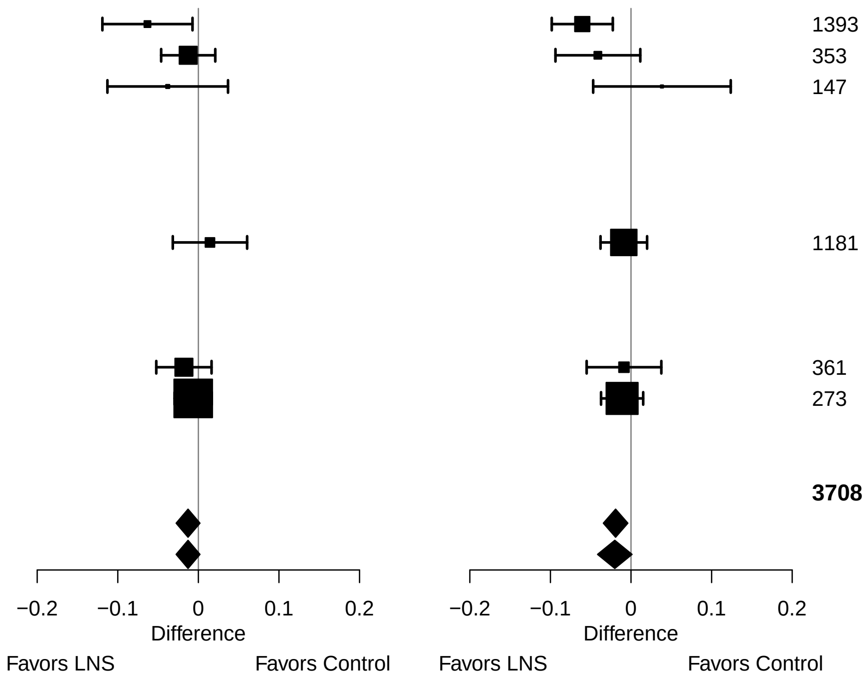

Supplemental figure 9K: Acute malnutrition prevalence difference

9K4: Stratified by Household sanitation

P−for−interaction = 0.016
Difference in PDs = 0.03 (0.01, 0.05)

Country	Trial	LNS N	Control N	Control Prevalence	Improved PD (95% CI)	Fixed W	Random W	LNS N	Control N	Control Prevalence	Unimproved PD (95% CI)	Fixed W	Random W
Bangladesh	JiVitA−4 (34)	2151	931	20.1	−0.03 (−0.06, 0.00)	0.27	0.27	631	285	24.0	0.00 (−0.05, 0.06)	0.10	0.10
Bangladesh	RDNS (35)	1179	573	15.5	−0.04 (−0.08, −0.01)	0.18	0.18	480	241	14.9	0.03 (−0.02, 0.08)	0.10	0.10
Bangladesh	WASH−B (36)												
Burkina Faso	iLiNS−Zinc (37)												
Burkina Faso	PROMIS (38)	389	544	12.5	−0.03 (−0.07, 0.02)	0.11	0.11	457	353	10.5	−0.01 (−0.05, 0.04)	0.14	0.14
Burkina Faso	PROMIS CS (38)	175	163	19.6	−0.02 (−0.10, 0.05)	0.03	0.03	248	271	19.2	0.00 (−0.06, 0.06)	0.08	0.08
Ghana	GHANA (39)												
Ghana	iLiNS−DYADG (40)												
Haiti	HAITI (41)												
Kenya	WASH−B (42)												
Madagascar	MAHAY (43)												
Malawi	iLiNS−DYADM (44)												
Malawi	iLiNS−DOSE (45)												
Mali	PROMIS (46)												
Mali	PROMIS CS (46)	696	692	13.6	−0.02 (−0.06, 0.02)	0.14	0.14	217	242	10.7	−0.02 (−0.06, 0.03)	0.13	0.13
Zimbabwe	SHINE (HIV−) (47)	292	242	4.1	−0.02 (−0.05, 0.00)	0.27	0.27	495	539	3.5	0.00 (−0.02, 0.03)	0.45	0.45
Zimbabwe	SHINE (HIV+) (48)												
		**4882**	**3145**		**I² = 0.00, Tau² = 0.00**			**2528**	**1931**		**I² = 0.00, Tau² = 0.00**		
**Fixed**					**−0.03 (−0.04, −0.01)**						**0.00 (−0.02, 0.02)**		
**Random**					**−0.03 (−0.04, −0.01)**						**0.00 (−0.02, 0.02)**		

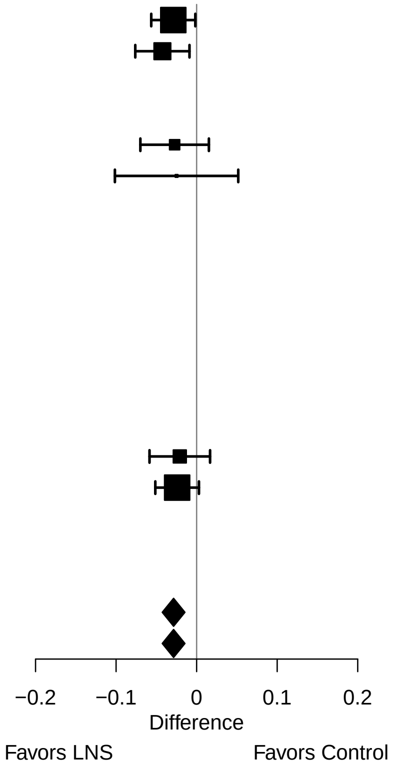

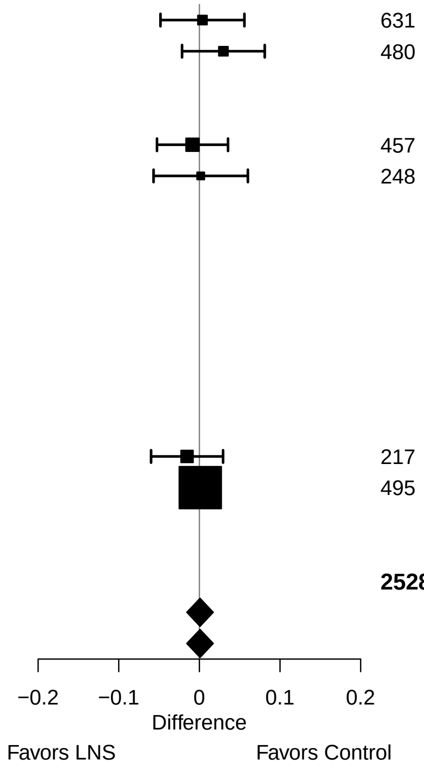

Supplemental figure 9K: Acute malnutrition prevalence difference

9K5: Stratified by Home environment

P−for−interaction = 0.318
Difference in PDs = −0.01 (−0.02, 0.01)

Country	Trial	LNS N	Control N	Control Prevalence	At least median PD (95% CI)	Fixed W	Random W	LNS N	Control N	Control Prevalence	Less than median PD (95% CI)	Fixed W	Random W
Bangladesh	JiVitA−4 (34)												
Bangladesh	RDNS (35)	1192	582	14.9	−0.03 (−0.07, 0.00)	0.06	0.19	466	230	15.7	0.01 (−0.04, 0.06)	0.07	0.07
Bangladesh	WASH−B (36)												
Burkina Faso	iLiNS−Zinc (37)	473	199	18.6	−0.08 (−0.12, −0.05)	0.07	0.19	271	174	21.7	−0.04 (−0.13, 0.05)	0.02	0.02
Burkina Faso	PROMIS (38)												
Burkina Faso	PROMIS CS (38)												
Ghana	GHANA (39)												
Ghana	iLiNS−DYADG (40)	213	409	7.1	0.00 (−0.04, 0.05)	0.04	0.17	119	248	9.7	−0.05 (−0.11, 0.00)	0.05	0.05
Haiti	HAITI (41)												
Kenya	WASH−B (42)	834	2756	2.4	0.00 (−0.01, 0.01)	0.72	0.24	572	2149	3.7	−0.01 (−0.03, 0.00)	0.75	0.75
Madagascar	MAHAY (43)	902	889	7.3	0.00 (−0.03, 0.03)	0.10	0.21	773	766	11.9	0.00 (−0.04, 0.04)	0.12	0.12
Malawi	iLiNS−DYADM (44)												
Malawi	iLiNS−DOSE (45)												
Mali	PROMIS (46)												
Mali	PROMIS CS (46)												
Zimbabwe	SHINE (HIV−) (47)												
Zimbabwe	SHINE (HIV+) (48)												
		**3614**	**4835**		**I² = 0.83, Tau² = 0.00**			**2201**	**3567**		**I² = 0.00, Tau² = 0.00**		
**Fixed**					**−0.01 (−0.02, 0.00)**						**−0.01 (−0.03, 0.00)**		
**Random**					**−0.02 (−0.05, 0.01)**						**−0.01 (−0.03, 0.00)**		

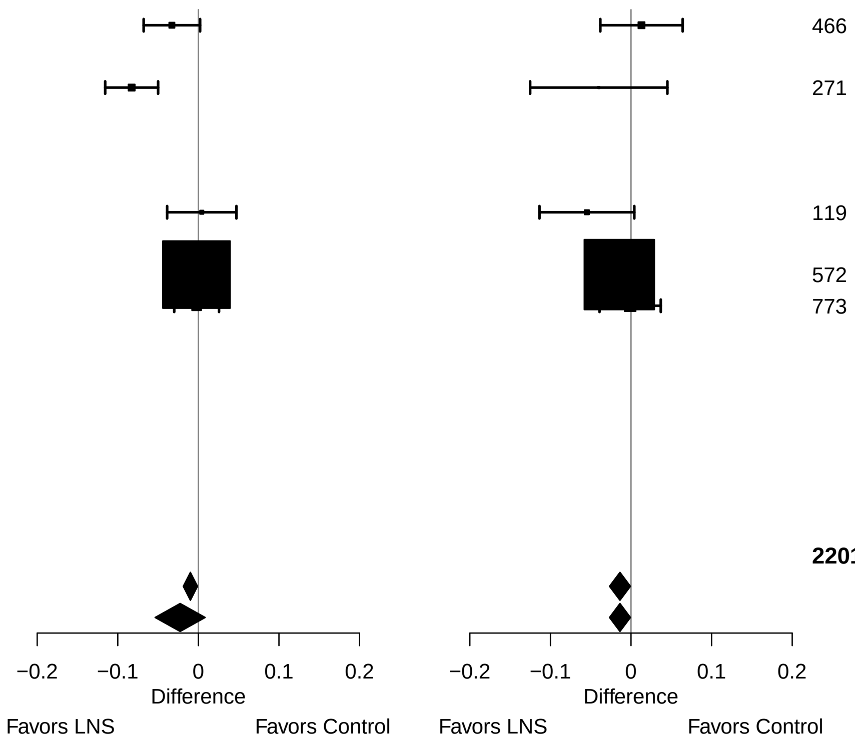

Supplemental figure 9K: Acute malnutrition prevalence difference

9K6: Stratified by Season at the time of assessment

P−for−interaction = 0.534
Difference in PDs = 0.01 (−0.01, 0.02)

Country	Trial	Dry LNS N	Dry Control N	Dry Control Prevalence	Dry PD (95% CI)	Dry Fixed W	Dry Random W	Rainy LNS N	Rainy Control N	Rainy Control Prevalence	Rainy PD (95% CI)	Rainy Fixed W	Rainy Random W
Bangladesh	JiVitA−4 (34)	1520	648	18.7	−0.02 (−0.05, 0.01)	0.14	0.17	1263	568	23.7	−0.02 (−0.06, 0.02)	0.05	0.05
Bangladesh	RDNS (35)	738	359	13.6	−0.03 (−0.07, 0.02)	0.08	0.13	923	456	16.7	−0.02 (−0.06, 0.02)	0.05	0.05
Bangladesh	WASH−B (36)												
Burkina Faso	iLiNS−Zinc (37)	1393	477	20.1	−0.07 (−0.10, −0.03)	0.12	0.16	558	187	17.0	−0.04 (−0.10, 0.01)	0.03	0.03
Burkina Faso	PROMIS (38)	418	441	12.0	−0.02 (−0.07, 0.02)	0.07	0.12	428	460	11.3	−0.02 (−0.06, 0.03)	0.04	0.04
Burkina Faso	PROMIS CS (38)	222	190	18.4	0.00 (−0.08, 0.08)	0.02	0.05	202	244	20.1	−0.02 (−0.08, 0.05)	0.02	0.02
Ghana	GHANA (39)												
Ghana	iLiNS−DYADG (40)												
Haiti	HAITI (41)												
Kenya	WASH−B (42)	346	1146	3.8	−0.01 (−0.03, 0.00)	0.50	0.26	1108	3971	3.0	−0.01 (−0.02, 0.00)	0.79	0.79
Madagascar	MAHAY (43)												
Malawi	iLiNS−DYADM (44)												
Malawi	iLiNS−DOSE (45)	415	134	4.5	0.02 (−0.03, 0.06)	0.07	0.11	275	106	7.5	0.00 (−0.06, 0.06)	0.02	0.02
Mali	PROMIS (46)												
Mali	PROMIS CS (46)												
Zimbabwe	SHINE (HIV−) (47)												
Zimbabwe	SHINE (HIV+) (48)												
		**5052**	**3395**		**I² = 0.44, Tau² = 0.00**			**4757**	**5992**		**I² = 0.00, Tau² = 0.00**		
**Fixed**					**−0.02 (−0.03, −0.01)**						**−0.01 (−0.02, 0.00)**		
**Random**					**−0.02 (−0.04, 0.00)**						**−0.01 (−0.02, 0.00)**		

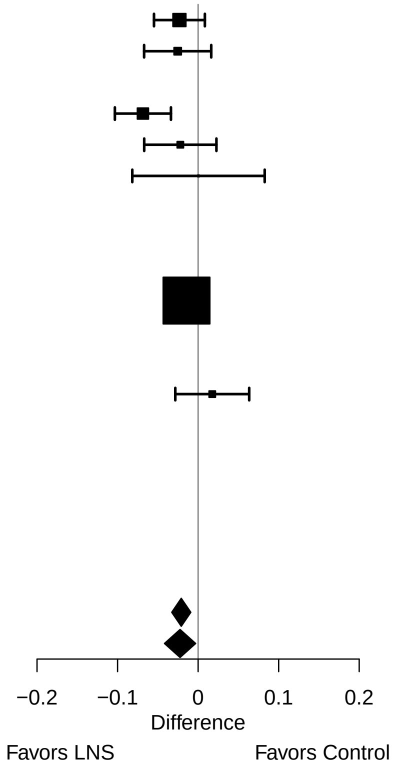

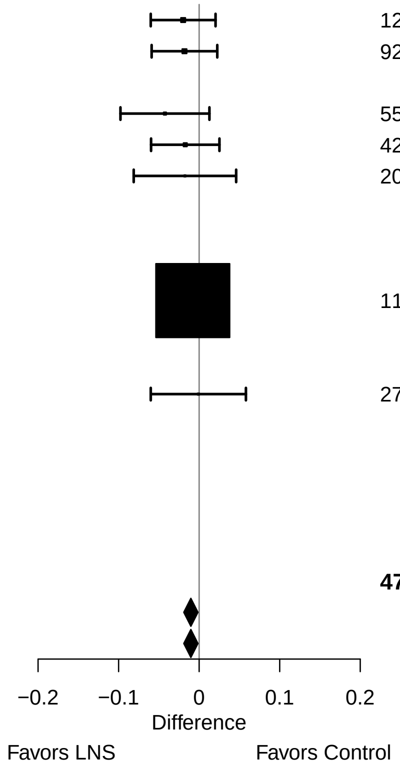

Supplemental figure 9L: Mean difference in WAZ

9L1: Stratified by Household socio-economic status

P−for−interaction = **0.954**
Difference in MDs = **0.00 (−0.03, 0.04)**

		At least median						Less than median					
**Country**	**Trial**	**LNS N**	**Control N**	**Control Mean**	**MD (95% CI)**	**Fixed W**	**Random W**	**LNS N**	**Control N**	**Control Mean**	**MD (95% CI)**	**Fixed W**	**Random W**
Bangladesh	JiVitA−4 (34)	1421	619	−1.64	0.12 (0.07, 0.17)	0.32	0.11	1392	613	−1.91	0.09 (0.04, 0.14)	0.29	0.13
Bangladesh	RDNS (35)	834	407	−1.61	0.09 (−0.02, 0.20)	0.06	0.07	829	408	−1.88	0.10 (0.01, 0.19)	0.09	0.09
Bangladesh	WASH−B (36)	584	1741	−1.33	0.18 (0.07, 0.28)	0.07	0.08	579	1720	−1.78	0.22 (0.14, 0.30)	0.10	0.09
Burkina Faso	iLiNS−Zinc (37)	1233	300	−1.51	0.28 (0.19, 0.37)	0.09	0.08	720	365	−1.54	0.27 (0.20, 0.34)	0.15	0.11
Burkina Faso	PROMIS (38)	405	482	−1.19	0.14 (−0.02, 0.29)	0.03	0.05	452	429	−1.27	0.16 (0.00, 0.33)	0.02	0.04
Burkina Faso	PROMIS CS (38)	220	208	−1.25	−0.05 (−0.26, 0.17)	0.02	0.04	210	228	−1.28	−0.05 (−0.23, 0.14)	0.02	0.04
Ghana	GHANA (39)	57	44	−0.40	0.14 (−0.35, 0.62)	0.00	0.01	41	46	−0.87	0.29 (−0.16, 0.74)	0.00	0.01
Ghana	iLiNS−DYADG (40)	157	356	−0.75	0.11 (−0.09, 0.30)	0.02	0.04	190	335	−0.96	0.22 (0.04, 0.41)	0.02	0.04
Haiti	HAITI (41)	74	61	−0.21	−0.03 (−0.21, 0.15)	0.02	0.04	61	72	−0.60	0.12 (−0.06, 0.30)	0.02	0.04
Kenya	WASH−B (42)	828	2941	−0.67	0.16 (0.09, 0.24)	0.13	0.09	631	2220	−0.85	0.10 (−0.01, 0.20)	0.07	0.08
Madagascar	MAHAY (43)	772	884	−1.35	−0.02 (−0.16, 0.13)	0.04	0.06	877	758	−1.56	0.08 (−0.07, 0.23)	0.03	0.05
Malawi	iLiNS−DYADM (44)	121	234	−0.80	−0.05 (−0.27, 0.16)	0.02	0.04	99	209	−0.96	−0.10 (−0.36, 0.16)	0.01	0.02
Malawi	iLiNS−DOSE (45)	307	104	−0.91	0.00 (−0.15, 0.15)	0.03	0.06	294	103	−1.30	0.11 (−0.06, 0.27)	0.03	0.05
Mali	PROMIS (46)	247	250	−1.13	0.23 (0.09, 0.36)	0.04	0.06	245	246	−1.14	0.10 (−0.05, 0.26)	0.03	0.05
Mali	PROMIS CS (46)	501	458	−1.33	0.25 (0.11, 0.40)	0.04	0.06	445	502	−1.34	0.20 (0.04, 0.37)	0.03	0.04
Zimbabwe	SHINE (HIV−) (47)	903	797	−0.66	0.10 (0.00, 0.20)	0.07	0.08	826	858	−0.87	0.21 (0.11, 0.30)	0.09	0.09
Zimbabwe	SHINE (HIV+) (48)	171	156	−0.87	−0.02 (−0.27, 0.22)	0.01	0.03	157	166	−1.01	0.04 (−0.21, 0.29)	0.01	0.02
		**8835**	**10042**		**I² = 0.53, Tau² = 0.01**			**8048**	**9278**		**I² = 0.55, Tau² = 0.00**		
**Fixed**					**0.13 (0.10, 0.16)**						**0.14 (0.12, 0.17)**		
**Random**					**0.11 (0.07, 0.16)**						**0.14 (0.10, 0.18)**		

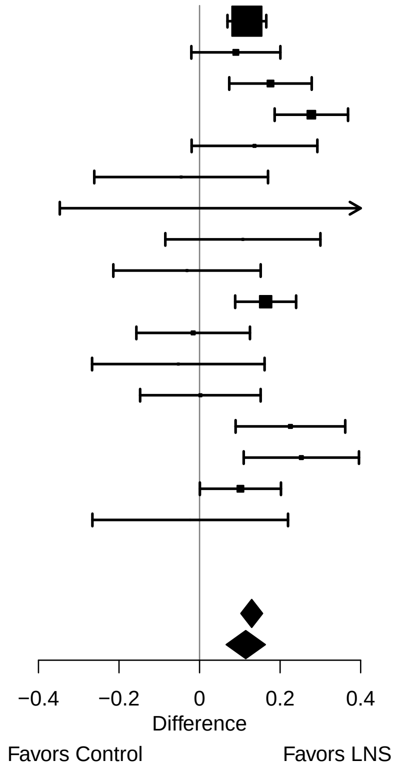

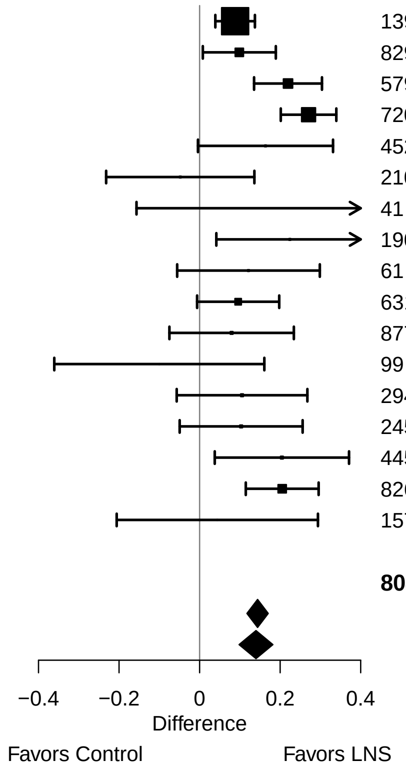

Supplemental figure 9L: Mean difference in WAZ

9L2: Stratified by Household food insecurity

P−for−interaction = 0.751
Difference in MDs = −0.01 (−0.05, 0.04)

Country	Trial	Mild to secure: LNS N	Control N	Control Mean	MD (95% CI)	Fixed W	Random W	Moderate to severe: LNS N	Control N	Control Mean	MD (95% CI)	Fixed W	Random W
Bangladesh	JiVitA−4 (34)	2000	851	−1.71	0.12 (0.08, 0.16)	0.36	0.13	813	381	−1.90	0.07 (0.01, 0.14)	0.28	0.16
Bangladesh	RDNS (35)	1058	493	−1.63	0.02 (−0.07, 0.12)	0.06	0.09	605	322	−1.93	0.19 (0.07, 0.31)	0.09	0.10
Bangladesh	WASH−B (36)	923	2681	−1.49	0.22 (0.14, 0.29)	0.10	0.11	240	780	−1.76	0.10 (−0.02, 0.22)	0.08	0.10
Burkina Faso	iLiNS−Zinc (37)	1017	321	−1.48	0.28 (0.19, 0.37)	0.08	0.10	936	344	−1.57	0.27 (0.19, 0.35)	0.21	0.15
Burkina Faso	PROMIS (38)	502	550	−1.21	0.16 (0.02, 0.31)	0.03	0.06	355	361	−1.25	0.12 (−0.05, 0.29)	0.04	0.06
Burkina Faso	PROMIS CS (38)												
Ghana	GHANA (39)												
Ghana	iLiNS−DYADG (40)	244	471	−0.83	0.19 (0.03, 0.35)	0.02	0.06	102	219	−0.89	0.08 (−0.15, 0.31)	0.02	0.04
Haiti	HAITI (41)												
Kenya	WASH−B (42)	1303	4621	−0.74	0.14 (0.07, 0.20)	0.15	0.11	156	527	−0.84	0.09 (−0.12, 0.30)	0.03	0.05
Madagascar	MAHAY (43)	989	929	−1.43	0.03 (−0.10, 0.16)	0.04	0.07	354	410	−1.49	0.04 (−0.15, 0.23)	0.04	0.06
Malawi	iLiNS−DYADM (44)	70	122	−0.68	−0.13 (−0.42, 0.16)	0.01	0.02	148	316	−0.94	−0.06 (−0.26, 0.15)	0.03	0.05
Malawi	iLiNS−DOSE (45)	154	55	−1.02	0.05 (−0.16, 0.25)	0.01	0.04	434	146	−1.17	0.07 (−0.06, 0.21)	0.07	0.09
Mali	PROMIS (46)	327	338	−1.13	0.16 (0.02, 0.30)	0.03	0.07	170	163	−1.19	0.18 (0.00, 0.36)	0.04	0.06
Mali	PROMIS CS (46)												
Zimbabwe	SHINE (HIV−) (47)	1384	1299	−0.74	0.14 (0.06, 0.21)	0.10	0.10	294	324	−0.89	0.26 (0.10, 0.42)	0.05	0.07
Zimbabwe	SHINE (HIV+) (48)	236	238	−0.94	0.02 (−0.19, 0.23)	0.01	0.04	85	76	−0.99	0.01 (−0.30, 0.32)	0.01	0.02
		**10207**	**12969**		**I² = 0.58, Tau² = 0.00**			**4692**	**4369**		**I² = 0.52, Tau² = 0.00**		
**Fixed**					**0.14 (0.11, 0.16)**						**0.14 (0.10, 0.17)**		
**Random**					**0.13 (0.08, 0.18)**						**0.13 (0.08, 0.18)**		

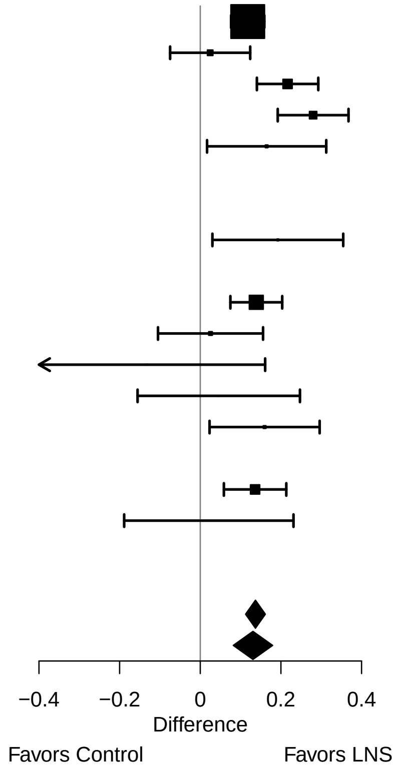

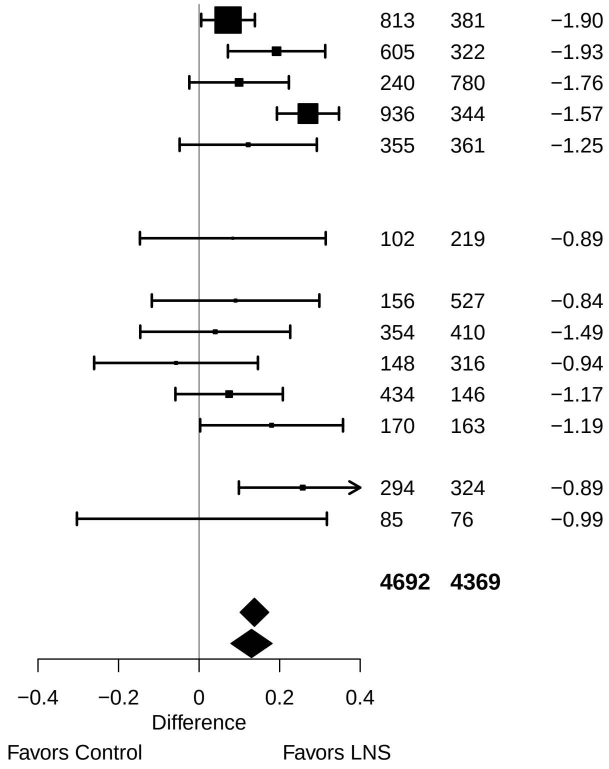

Supplemental figure 9L: Mean difference in WAZ

9L3: Stratified by Household source water quality

P−for−interaction = 0.359
Difference in MDs = 0.03 (−0.03, 0.08)

Country	Trial	Improved LNS N	Improved Control N	Improved Control Mean	Improved MD (95% CI)	Improved Fixed W	Improved Random W	Unimproved LNS N	Unimproved Control N	Unimproved Control Mean	Unimproved MD (95% CI)	Unimproved Fixed W	Unimproved Random W
Bangladesh	JiVitA−4 (34)												
Bangladesh	RDNS (35)												
Bangladesh	WASH−B (36)	501	1002	−1.53	0.29 (0.17, 0.41)	0.10	0.10	70	116	−1.67	0.03 (−0.28, 0.34)	0.02	0.05
Burkina Faso	iLiNS−Zinc (37)	555	141	−1.45	0.21 (0.12, 0.29)	0.21	0.13	1397	521	−1.55	0.30 (0.23, 0.37)	0.39	0.17
Burkina Faso	PROMIS (38)	352	379	−1.21	0.07 (−0.08, 0.22)	0.06	0.08	358	351	−1.23	0.25 (0.06, 0.45)	0.05	0.09
Burkina Faso	PROMIS CS (38)	280	267	−1.30	−0.01 (−0.21, 0.20)	0.03	0.05	149	169	−1.21	−0.11 (−0.31, 0.08)	0.05	0.09
Ghana	GHANA (39)												
Ghana	iLiNS−DYADG (40)												
Haiti	HAITI (41)												
Kenya	WASH−B (42)	449	1555	−0.71	0.09 (−0.02, 0.19)	0.13	0.11	248	699	−0.78	0.09 (−0.07, 0.25)	0.08	0.10
Madagascar	MAHAY (43)	418	454	−1.30	0.02 (−0.18, 0.22)	0.04	0.06	1204	1182	−1.50	0.02 (−0.09, 0.14)	0.16	0.14
Malawi	iLiNS−DYADM (44)	200	406	−0.86	−0.09 (−0.26, 0.09)	0.05	0.07	20	36	−1.00	0.12 (−0.49, 0.73)	0.01	0.01
Malawi	iLiNS−DOSE (45)	561	193	−1.08	0.06 (−0.05, 0.18)	0.11	0.10	52	17	−1.41	0.02 (−0.37, 0.41)	0.01	0.03
Mali	PROMIS (46)	247	257	−1.07	0.18 (0.05, 0.32)	0.08	0.09	243	240	−1.22	0.14 (−0.03, 0.30)	0.07	0.10
Mali	PROMIS CS (46)	544	563	−1.30	0.20 (0.07, 0.33)	0.09	0.09	363	382	−1.40	0.25 (0.05, 0.45)	0.05	0.08
Zimbabwe	SHINE (HIV−) (47)	518	486	−0.77	0.14 (0.01, 0.27)	0.09	0.09	273	301	−0.67	0.03 (−0.11, 0.17)	0.10	0.12
Zimbabwe	SHINE (HIV+) (48)	85	87	−1.00	0.07 (−0.23, 0.38)	0.02	0.03	55	56	−1.00	0.02 (−0.37, 0.40)	0.01	0.03
		**4710**	**5790**		**I² = 0.52, Tau² = 0.01**			**4432**	**4070**		**I² = 0.69, Tau² = 0.01**		
**Fixed**					**0.14 (0.10, 0.17)**						**0.16 (0.12, 0.20)**		
**Random**					**0.12 (0.06, 0.18)**						**0.12 (0.04, 0.19)**		

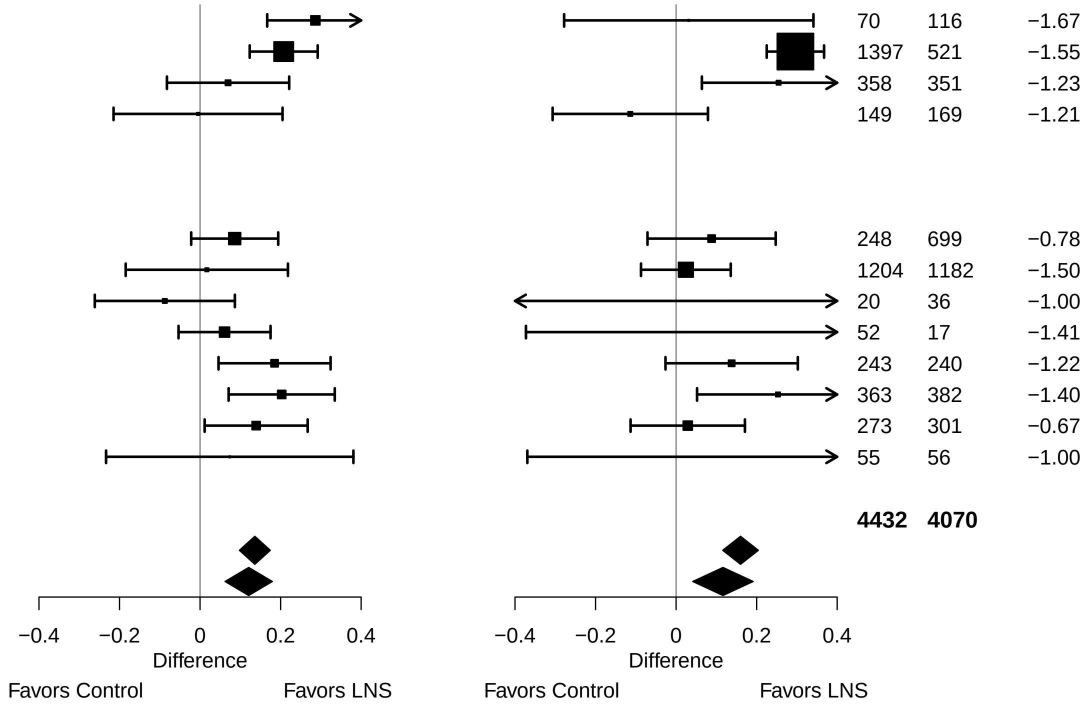

Supplemental figure 9L: Mean difference in WAZ

9L4: Stratified by Household sanitation

P−for−interaction = 0.035
Difference in MDs = −0.06 (−0.11, 0.00)

Country	Trial	Improved LNS N	Improved Control N	Improved Control Mean	Improved MD (95% CI)	Improved Fixed W	Improved Random W	Unimproved LNS N	Unimproved Control N	Unimproved Control Mean	Unimproved MD (95% CI)	Unimproved Fixed W	Unimproved Random W
Bangladesh	JiVitA−4 (34)	2179	944	−1.72	0.12 (0.08, 0.16)	0.54	0.54	634	288	−1.92	0.06 (−0.01, 0.13)	0.23	0.11
Bangladesh	RDNS (35)	1179	573	−1.71	0.13 (0.03, 0.22)	0.10	0.10	482	241	−1.85	0.02 (−0.13, 0.17)	0.05	0.08
Bangladesh	WASH−B (36)	512	1015	−1.51	0.25 (0.13, 0.38)	0.05	0.05	30	55	−1.73	0.42 (0.07, 0.77)	0.01	0.03
Burkina Faso	iLiNS−Zinc (37)	43	16	−1.62	0.23 (−0.14, 0.60)	0.01	0.01	1909	646	−1.53	0.28 (0.21, 0.35)	0.25	0.11
Burkina Faso	PROMIS (38)	392	550	−1.24	0.18 (0.06, 0.30)	0.06	0.06	465	357	−1.19	0.11 (−0.06, 0.27)	0.04	0.07
Burkina Faso	PROMIS CS (38)	176	165	−1.31	0.03 (−0.18, 0.25)	0.02	0.02	252	271	−1.24	−0.10 (−0.26, 0.07)	0.04	0.07
Ghana	GHANA (39)												
Ghana	iLiNS−DYADG (40)	336	672	−0.84	0.14 (0.01, 0.28)	0.05	0.05	10	19	−1.13	0.66 (−0.24, 1.56)	0.00	0.01
Haiti	HAITI (41)												
Kenya	WASH−B (42)	101	366	−0.55	0.16 (−0.02, 0.34)	0.03	0.03	596	1889	−0.76	0.07 (−0.03, 0.18)	0.11	0.10
Madagascar	MAHAY (43)												
Malawi	iLiNS−DYADM (44)	20	42	−0.76	0.27 (−0.21, 0.75)	0.00	0.00	200	400	−0.89	−0.10 (−0.28, 0.07)	0.03	0.07
Malawi	iLiNS−DOSE (45)	15	9	−1.02	−0.20 (−0.76, 0.36)	0.00	0.00	597	201	−1.11	0.06 (−0.05, 0.17)	0.09	0.09
Mali	PROMIS (46)	381	363	−1.12	0.17 (0.03, 0.30)	0.05	0.05	112	134	−1.23	0.16 (−0.01, 0.33)	0.04	0.07
Mali	PROMIS CS (46)	698	692	−1.34	0.26 (0.12, 0.40)	0.04	0.04	217	243	−1.34	0.19 (0.01, 0.37)	0.03	0.07
Zimbabwe	SHINE (HIV−) (47)	293	243	−0.68	0.14 (−0.03, 0.32)	0.03	0.03	495	543	−0.76	0.07 (−0.05, 0.19)	0.08	0.09
Zimbabwe	SHINE (HIV+) (48)	50	35	−0.95	−0.08 (−0.50, 0.34)	0.00	0.00	90	107	−1.01	0.11 (−0.18, 0.40)	0.01	0.04
		**6375**	**5685**		**I² = 0.00, Tau² = 0.00**			**6089**	**5394**		**I² = 0.72, Tau² = 0.01**		
**Fixed**					**0.14 (0.11, 0.17)**						**0.12 (0.08, 0.15)**		
**Random**					**0.14 (0.11, 0.17)**						**0.09 (0.03, 0.16)**		

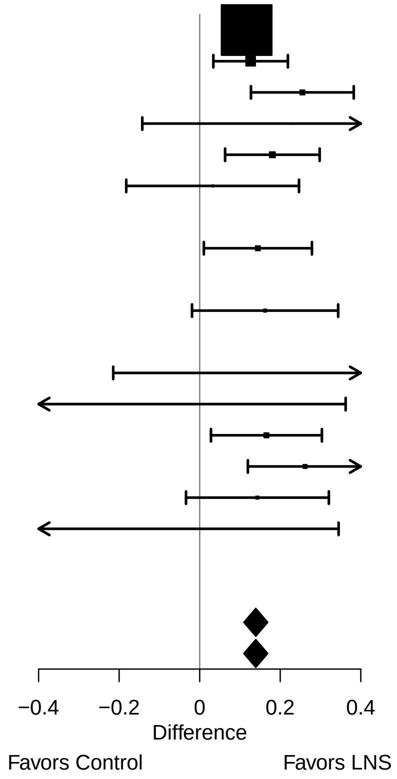

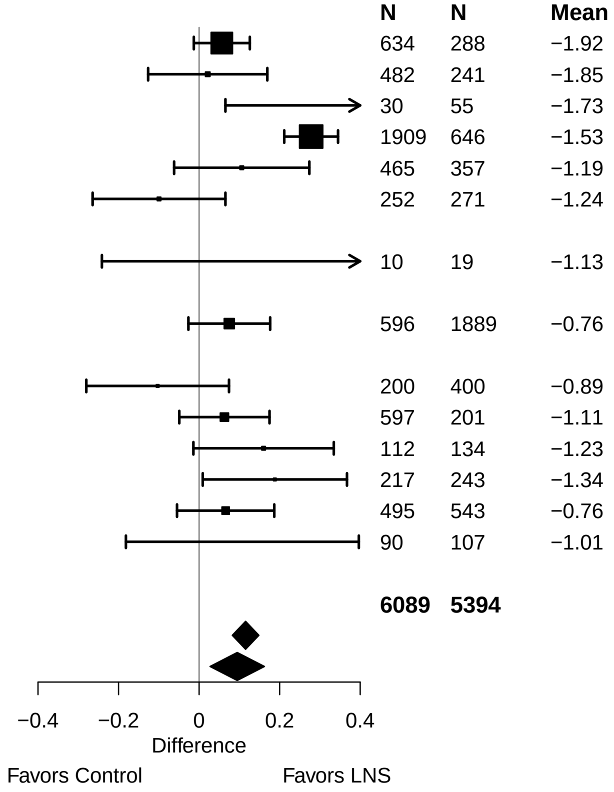

Supplemental figure 9L: Mean difference in WAZ

9L5: Stratified by Home environment

P−for−interaction = 0.076
Difference in MDs = 0.06 (−0.01, 0.12)

Country	Trial	At least median: LNS N	Control N	Control Mean	MD (95% CI)	Fixed W	Random W	Less than median: LNS N	Control N	Control Mean	MD (95% CI)	Fixed W	Random W
Bangladesh	JiVitA−4 (34)												
Bangladesh	RDNS (35)	1192	582	−1.70	0.09 (−0.01, 0.20)	0.13	0.14	468	230	−1.86	0.09 (−0.06, 0.25)	0.11	0.13
Bangladesh	WASH−B (36)	641	1668	−1.41	0.19 (0.09, 0.28)	0.17	0.15	494	1702	−1.69	0.15 (0.04, 0.26)	0.22	0.17
Burkina Faso	iLiNS−Zinc (37)	473	199	−1.44	0.26 (0.15, 0.36)	0.14	0.14	272	175	−1.68	0.24 (0.05, 0.42)	0.08	0.11
Burkina Faso	PROMIS (38)												
Burkina Faso	PROMIS CS (38)												
Ghana	GHANA (39)												
Ghana	iLiNS−DYADG (40)	213	410	−0.83	0.12 (−0.05, 0.29)	0.05	0.10	119	248	−0.93	0.29 (0.06, 0.52)	0.05	0.08
Haiti	HAITI (41)												
Kenya	WASH−B (42)	840	2776	−0.67	0.04 (−0.03, 0.11)	0.30	0.16	573	2167	−0.80	0.20 (0.11, 0.29)	0.32	0.20
Madagascar	MAHAY (43)	915	907	−1.40	−0.03 (−0.15, 0.10)	0.09	0.12	787	778	−1.49	0.06 (−0.09, 0.21)	0.12	0.14
Malawi	iLiNS−DYADM (44)	137	289	−0.80	−0.08 (−0.28, 0.13)	0.04	0.08	78	149	−1.03	−0.05 (−0.35, 0.24)	0.03	0.06
Malawi	iLiNS−DOSE (45)	379	136	−1.18	0.17 (0.03, 0.30)	0.08	0.12	277	86	−1.00	−0.07 (−0.26, 0.11)	0.08	0.11
Mali	PROMIS (46)												
Mali	PROMIS CS (46)												
Zimbabwe	SHINE (HIV−) (47)												
Zimbabwe	SHINE (HIV+) (48)												
		**4790**	**6967**		**I² = 0.69, Tau² = 0.01**			**3068**	**5535**		**I² = 0.46, Tau² = 0.01**		
**Fixed**					**0.11 (0.07, 0.15)**						**0.14 (0.09, 0.19)**		
**Random**					**0.10 (0.03, 0.18)**						**0.12 (0.04, 0.20)**		

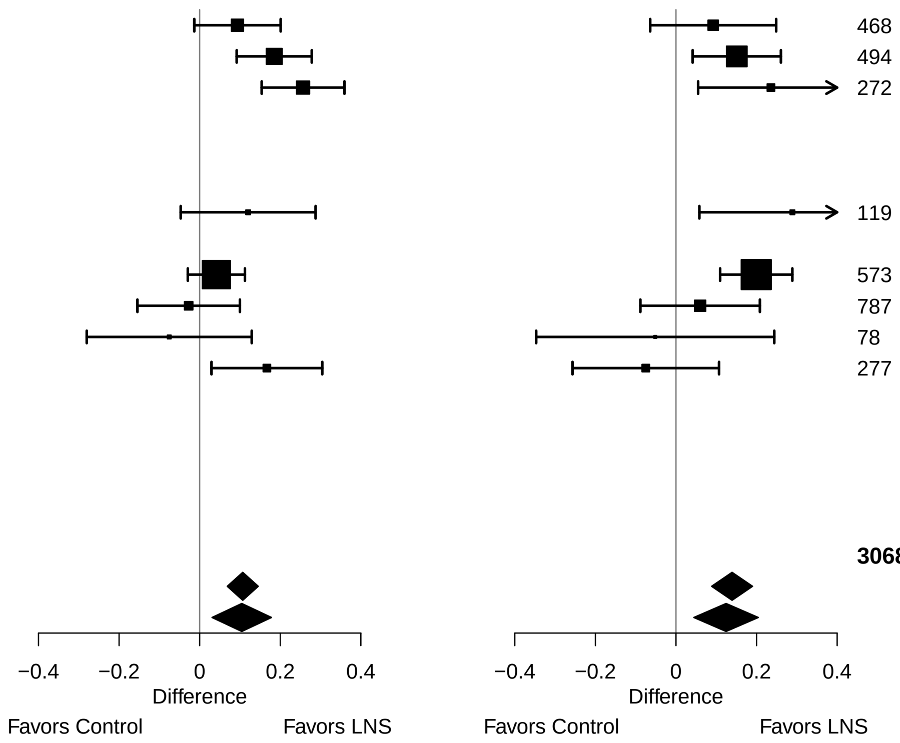

Supplemental figure 9L: Mean difference in WAZ

9L6: Stratified by Season at the time of assessment

P−for−interaction = 0.212
Difference in MDs = −0.03 (−0.07, 0.02)

Country	Trial	Dry LNS N	Dry Control N	Dry Control Mean	Dry MD (95% CI)	Dry Fixed W	Dry Random W	Rainy LNS N	Rainy Control N	Rainy Control Mean	Rainy MD (95% CI)	Rainy Fixed W	Rainy Random W
Bangladesh	JiVitA−4 (34)	1533	656	−1.71	0.11 (0.06, 0.16)	0.33	0.11	1281	576	−1.84	0.10 (0.04, 0.15)	0.34	0.14
Bangladesh	RDNS (35)	739	359	−1.71	0.11 (−0.04, 0.25)	0.04	0.08	924	456	−1.78	0.09 (−0.01, 0.18)	0.10	0.12
Bangladesh	WASH−B (36)	645	1938	−1.42	0.19 (0.09, 0.30)	0.08	0.09	518	1523	−1.72	0.21 (0.13, 0.28)	0.15	0.13
Burkina Faso	iLiNS−Zinc (37)	1400	477	−1.57	0.32 (0.24, 0.40)	0.12	0.10	560	189	−1.43	0.17 (0.07, 0.28)	0.09	0.11
Burkina Faso	PROMIS (38)	429	447	−1.14	0.10 (−0.04, 0.23)	0.05	0.08	428	464	−1.30	0.19 (0.03, 0.36)	0.03	0.08
Burkina Faso	PROMIS CS (38)	224	190	−1.23	−0.02 (−0.27, 0.24)	0.01	0.05	206	246	−1.29	−0.09 (−0.29, 0.11)	0.02	0.06
Ghana	GHANA (39)	57	55	−0.84	0.61 (0.20, 1.01)	0.01	0.02	41	41	−0.46	−0.16 (−0.70, 0.39)	0.00	0.01
Ghana	iLiNS−DYADG (40)	204	408	−0.87	0.04 (−0.13, 0.20)	0.03	0.07	143	285	−0.83	0.34 (0.12, 0.56)	0.02	0.06
Haiti	HAITI (41)												
Kenya	WASH−B (42)	348	1157	−0.80	0.19 (0.08, 0.30)	0.07	0.09	1113	4008	−0.74	0.12 (0.04, 0.19)	0.17	0.13
Madagascar	MAHAY (43)												
Malawi	iLiNS−DYADM (44)	156	316	−0.82	−0.04 (−0.23, 0.16)	0.02	0.06	64	128	−1.02	−0.14 (−0.45, 0.18)	0.01	0.03
Malawi	iLiNS−DOSE (45)	421	134	−1.02	0.00 (−0.14, 0.15)	0.04	0.08	281	107	−1.17	0.11 (−0.04, 0.26)	0.04	0.08
Mali	PROMIS (46)												
Mali	PROMIS CS (46)												
Zimbabwe	SHINE (HIV−) (47)	1736	1655	−0.77	0.15 (0.09, 0.22)	0.17	0.10	133	131	−0.51	−0.14 (−0.43, 0.15)	0.01	0.04
Zimbabwe	SHINE (HIV+) (48)	318	304	−0.94	0.00 (−0.17, 0.18)	0.03	0.07	18	24	−1.01	0.13 (−0.42, 0.68)	0.00	0.01
		**8210**	**8096**		**I² = 0.70, Tau² = 0.01**			**5710**	**8178**		**I² = 0.47, Tau² = 0.01**		
**Fixed**					**0.14 (0.11, 0.17)**						**0.12 (0.09, 0.15)**		
**Random**					**0.13 (0.05, 0.20)**						**0.11 (0.05, 0.17)**		

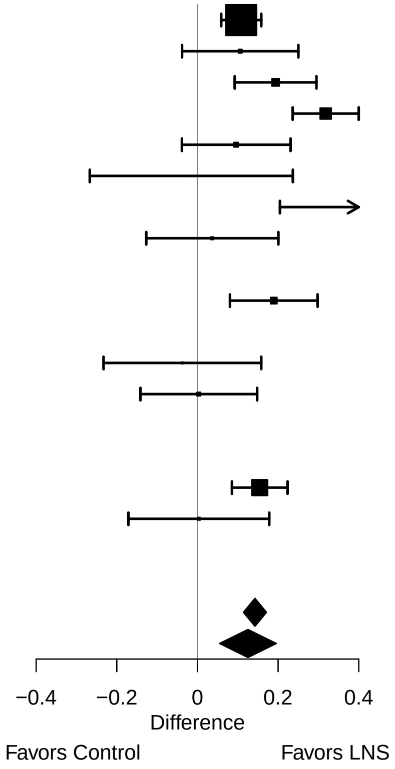

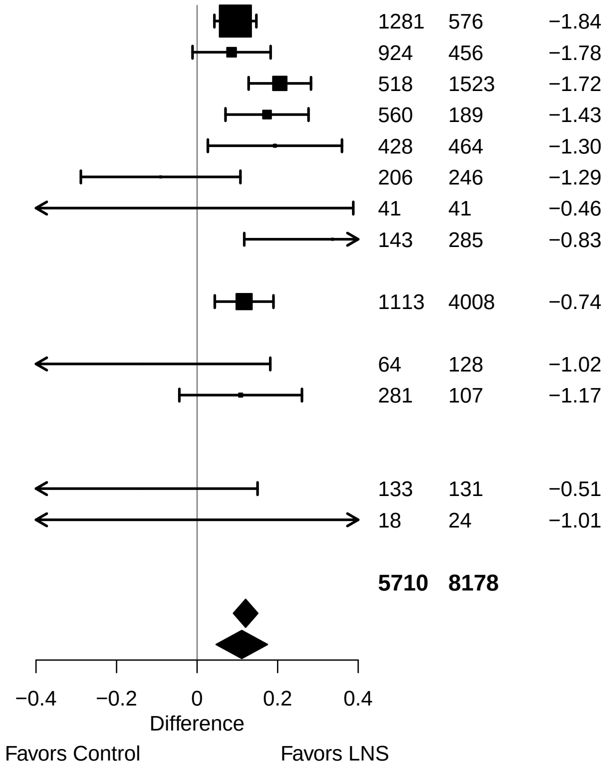

Supplemental figure 9M: Underweight prevalence ratio

9M1: Stratified by Household socio-economic status

P−for−interaction = 0.877
Ratio of PRs = 1.01 (0.93, 1.09)

		**At least median**						**Less than median**					
**Country**	**Trial**	**LNS N**	**Control N**	**Control Prevalence**	**PR (95% CI)**	**Fixed W**	**Random W**	**LNS N**	**Control N**	**Control Prevalence**	**PR (95% CI)**	**Fixed W**	**Random W**
Bangladesh	JiVitA−4 (34)	1421	619	35.4	0.81 (0.71, 0.93)	0.24	0.16	1392	613	42.9	0.97 (0.86, 1.08)	0.22	0.16
Bangladesh	RDNS (35)	834	407	32.2	0.91 (0.77, 1.06)	0.16	0.13	829	408	43.4	0.93 (0.83, 1.05)	0.23	0.17
Bangladesh	WASH−B (36)	584	1741	23.9	0.85 (0.70, 1.03)	0.11	0.11	579	1720	41.0	0.75 (0.65, 0.86)	0.15	0.13
Burkina Faso	iLiNS−Zinc (37)	1233	300	28.0	0.72 (0.57, 0.90)	0.08	0.09	720	365	33.2	0.69 (0.57, 0.83)	0.08	0.09
Burkina Faso	PROMIS (38)	405	482	19.9	0.81 (0.59, 1.11)	0.04	0.05	452	429	19.3	0.91 (0.67, 1.24)	0.03	0.04
Burkina Faso	PROMIS CS (38)	220	208	21.6	1.07 (0.79, 1.45)	0.04	0.06	210	228	26.8	0.93 (0.68, 1.26)	0.03	0.04
Ghana	GHANA (39)												
Ghana	iLiNS−DYADG (40)	157	356	11.0	0.81 (0.46, 1.46)	0.01	0.02	190	335	14.9	0.81 (0.51, 1.29)	0.01	0.02
Haiti	HAITI (41)												
Kenya	WASH−B (42)	828	2941	8.0	0.68 (0.50, 0.91)	0.05	0.06	631	2220	12.7	0.83 (0.63, 1.08)	0.04	0.05
Madagascar	MAHAY (43)	772	884	23.2	1.07 (0.87, 1.30)	0.10	0.10	877	758	29.6	0.97 (0.79, 1.20)	0.07	0.08
Malawi	iLiNS−DYADM (44)	121	234	9.0	1.38 (0.74, 2.58)	0.01	0.02	99	209	14.4	0.99 (0.55, 1.77)	0.01	0.01
Malawi	iLiNS−DOSE (45)	307	104	10.6	1.28 (0.73, 2.25)	0.01	0.02	294	103	30.1	0.91 (0.67, 1.24)	0.03	0.05
Mali	PROMIS (46)	247	250	15.5	0.75 (0.53, 1.06)	0.03	0.05	245	246	17.1	0.88 (0.52, 1.49)	0.01	0.02
Mali	PROMIS CS (46)	501	458	21.4	0.90 (0.69, 1.16)	0.06	0.07	445	502	23.5	0.88 (0.67, 1.16)	0.04	0.05
Zimbabwe	SHINE (HIV−) (47)	903	797	9.0	0.76 (0.55, 1.05)	0.04	0.05	826	858	12.5	0.62 (0.46, 0.84)	0.03	0.05
Zimbabwe	SHINE (HIV+) (48)	171	156	14.7	1.03 (0.61, 1.74)	0.01	0.02	157	166	19.9	0.96 (0.59, 1.56)	0.01	0.02
		**8704**	**9937**		**I² = 0.25, Tau² = 0.01**			**7946**	**9160**		**I² = 0.35, Tau² = 0.00**		
**Fixed**					**0.86 (0.81, 0.92)**						**0.87 (0.82, 0.92)**		
**Random**					**0.87 (0.80, 0.94)**						**0.86 (0.80, 0.92)**		

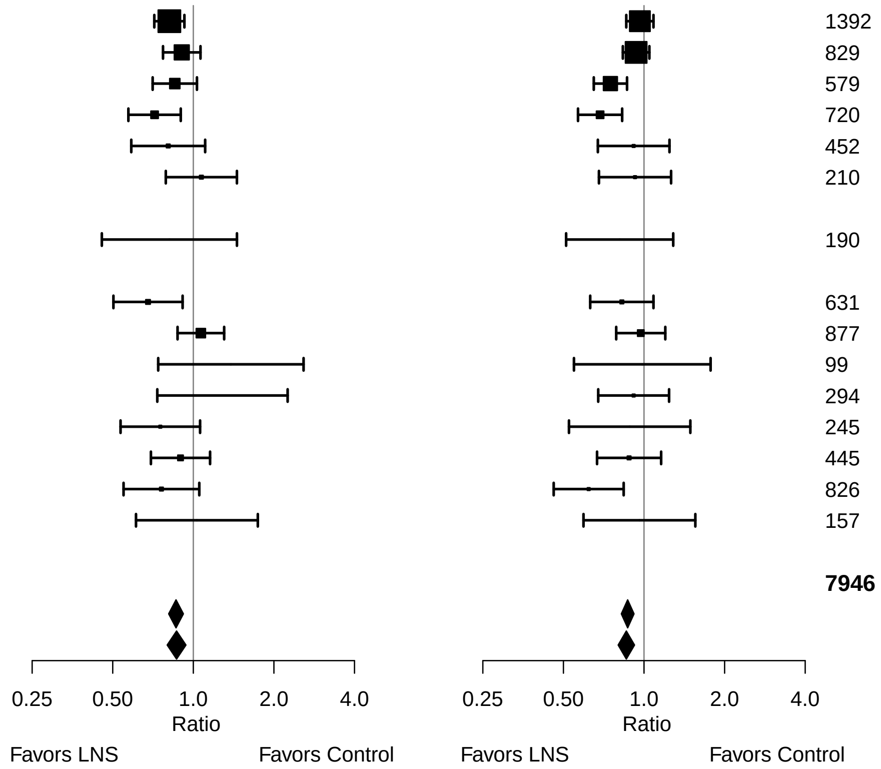

Supplemental figure 9M: Underweight prevalence ratio

9M2: Stratified by Household food insecurity

P−for−interaction = 0.661
Ratio of PRs = 0.98 (0.89, 1.08)

Country	Trial	LNS N	Control N	Control Prevalence	Mild to secure PR (95% CI)	Fixed W	Random W	LNS N	Control N	Control Prevalence	Moderate to severe PR (95% CI)	Fixed W	Random W
Bangladesh	JiVitA−4 (34)	2000	851	37.0	0.86 (0.77, 0.95)	0.27	0.16	813	381	43.9	0.97 (0.83, 1.13)	0.24	0.16
Bangladesh	RDNS (35)	1058	493	32.9	1.00 (0.86, 1.15)	0.14	0.13	605	322	45.3	0.85 (0.73, 0.99)	0.22	0.16
Bangladesh	WASH−B (36)	923	2681	30.8	0.76 (0.67, 0.87)	0.18	0.14	240	780	38.1	0.89 (0.72, 1.09)	0.13	0.13
Burkina Faso	iLiNS−Zinc (37)	1017	321	27.4	0.71 (0.60, 0.85)	0.10	0.11	936	344	34.0	0.67 (0.55, 0.81)	0.15	0.14
Burkina Faso	PROMIS (38)	502	550	19.3	0.95 (0.74, 1.22)	0.05	0.07	355	361	20.2	0.74 (0.51, 1.07)	0.04	0.06
Burkina Faso	PROMIS CS (38)												
Ghana	GHANA (39)												
Ghana	iLiNS−DYADG (40)	244	471	12.7	0.77 (0.49, 1.21)	0.02	0.03	102	219	13.7	0.93 (0.51, 1.71)	0.02	0.03
Haiti	HAITI (41)												
Kenya	WASH−B (42)	1303	4621	9.8	0.77 (0.62, 0.95)	0.07	0.09	156	527	12.1	0.69 (0.34, 1.37)	0.01	0.02
Madagascar	MAHAY (43)	989	929	25.6	1.03 (0.86, 1.24)	0.09	0.10	354	410	27.3	1.03 (0.75, 1.43)	0.05	0.08
Malawi	iLiNS−DYADM (44)	70	122	8.2	1.57 (0.67, 3.67)	0.00	0.01	148	316	12.7	1.07 (0.65, 1.76)	0.02	0.04
Malawi	iLiNS−DOSE (45)	154	55	14.5	1.00 (0.55, 1.85)	0.01	0.02	434	146	21.9	1.03 (0.75, 1.42)	0.05	0.08
Mali	PROMIS (46)	327	338	15.2	0.88 (0.59, 1.32)	0.02	0.03	170	163	19.0	0.80 (0.50, 1.29)	0.02	0.04
Mali	PROMIS CS (46)												
Zimbabwe	SHINE (HIV−) (47)	1384	1299	9.8	0.75 (0.57, 0.97)	0.04	0.07	294	324	15.4	0.48 (0.31, 0.77)	0.03	0.04
Zimbabwe	SHINE (HIV+) (48)	236	238	17.6	1.03 (0.71, 1.50)	0.02	0.04	85	76	17.1	0.89 (0.45, 1.76)	0.01	0.02
		**10207**	**12969**		**I² = 0.45, Tau² = 0.01**			**4692**	**4369**		**I² = 0.38, Tau² = 0.01**		
**Fixed**					**0.85 (0.81, 0.90)**						**0.85 (0.79, 0.92)**		
**Random**					**0.86 (0.79, 0.93)**						**0.85 (0.76, 0.94)**		

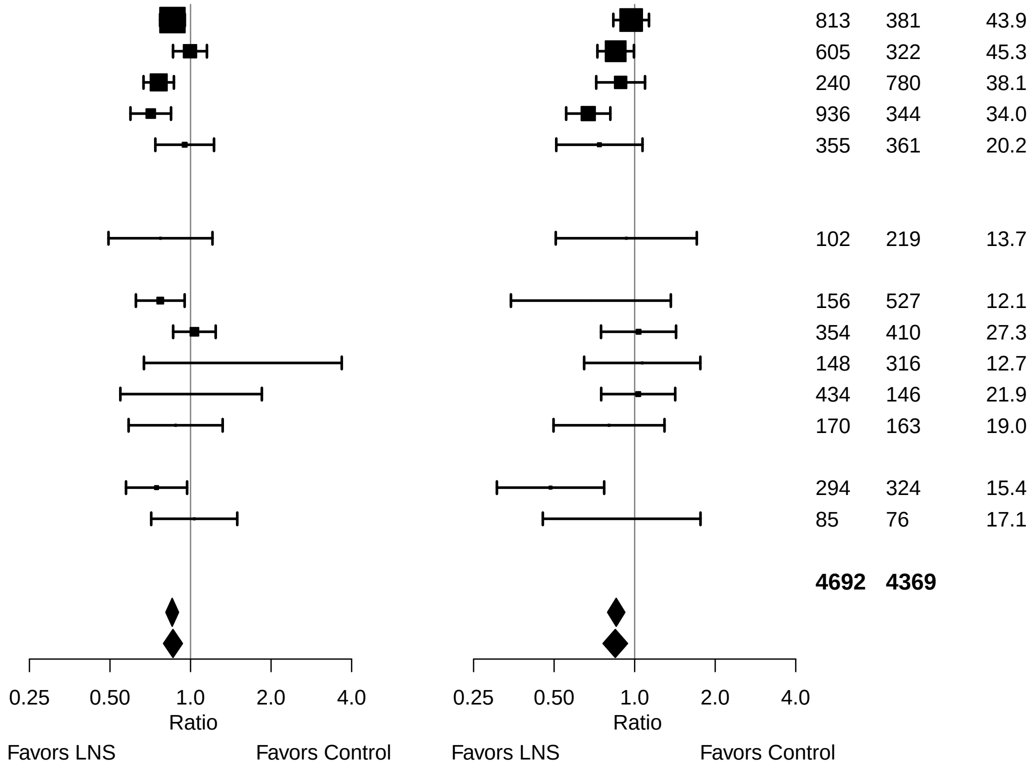

Supplemental figure 9M: Underweight prevalence ratio

9M3: Stratified by Household source water quality

P−for−interaction = 0.762
Ratio of PRs = 0.98 (0.86, 1.12)

Country	Trial	Improved LNS N	Improved Control N	Improved Control Prevalence	Improved PR (95% CI)	Improved Fixed W	Improved Random W	Unimproved LNS N	Unimproved Control N	Unimproved Control Prevalence	Unimproved PR (95% CI)	Unimproved Fixed W	Unimproved Random W
Bangladesh	JiVitA−4 (34)												
Bangladesh	RDNS (35)												
Bangladesh	WASH−B (36)	501	1002	30.7	0.81 (0.68, 0.97)	0.28	0.19	70	116	29.3	1.12 (0.72, 1.74)	0.04	0.08
Burkina Faso	iLiNS−Zinc (37)	555	141	27.7	0.66 (0.52, 0.85)	0.14	0.13	1397	521	31.9	0.69 (0.59, 0.82)	0.30	0.17
Burkina Faso	PROMIS (38)	352	379	17.9	1.01 (0.75, 1.36)	0.10	0.11	358	351	23.4	0.57 (0.42, 0.79)	0.08	0.11
Burkina Faso	PROMIS CS (38)	280	267	27.3	0.82 (0.61, 1.11)	0.10	0.11	149	169	19.5	1.37 (0.95, 1.99)	0.06	0.10
Ghana	GHANA (39)												
Ghana	iLiNS−DYADG (40)												
Haiti	HAITI (41)												
Kenya	WASH−B (42)	449	1555	8.8	0.94 (0.65, 1.34)	0.07	0.08	248	699	10.7	0.83 (0.50, 1.36)	0.03	0.07
Madagascar	MAHAY (43)	418	454	20.5	1.26 (0.95, 1.67)	0.11	0.12	1204	1182	28.1	0.96 (0.82, 1.13)	0.31	0.17
Malawi	iLiNS−DYADM (44)												
Malawi	iLiNS−DOSE (45)												
Mali	PROMIS (46)	247	257	14.3	0.80 (0.47, 1.36)	0.03	0.05	243	240	18.5	0.88 (0.57, 1.36)	0.04	0.08
Mali	PROMIS CS (46)	544	563	21.5	0.88 (0.66, 1.17)	0.11	0.12	363	382	23.6	0.91 (0.67, 1.24)	0.09	0.12
Zimbabwe	SHINE (HIV−) (47)	518	486	9.7	0.76 (0.50, 1.14)	0.05	0.07	273	301	8.6	1.02 (0.62, 1.68)	0.03	0.07
Zimbabwe	SHINE (HIV+) (48)	85	87	13.8	1.28 (0.64, 2.54)	0.02	0.03	55	56	21.4	0.85 (0.42, 1.73)	0.02	0.04
		**3949**	**5191**		**I² = 0.39, Tau² = 0.01**			**4360**	**4017**		**I² = 0.61, Tau² = 0.03**		
**Fixed**					**0.87 (0.79, 0.95)**						**0.85 (0.78, 0.93)**		
**Random**					**0.88 (0.78, 1.00)**						**0.88 (0.75, 1.03)**		

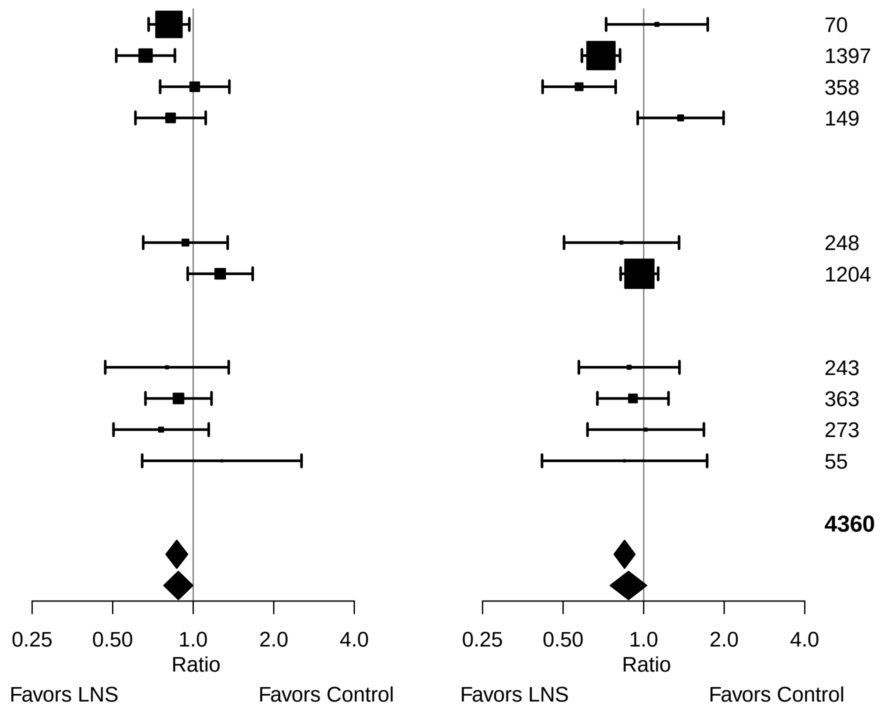

Supplemental figure 9M: Underweight prevalence ratio

9M4: Stratified by Household sanitation

P−for−interaction = 0.840
Ratio of PRs = 1.01 (0.89, 1.15)

		Improved						Unimproved					
Country	Trial	LNS N	Control N	Control Prevalence	PR (95% CI)	Fixed W	Random W	LNS N	Control N	Control Prevalence	PR (95% CI)	Fixed W	Random W
Bangladesh	JiVitA−4 (34)	2179	944	37.7	0.91 (0.81, 1.02)	0.37	0.37	634	288	43.9	0.94 (0.80, 1.09)	0.37	0.37
Bangladesh	RDNS (35)	1179	573	34.9	0.93 (0.83, 1.04)	0.41	0.41	482	241	44.8	0.90 (0.74, 1.10)	0.22	0.22
Bangladesh	WASH−B (36)												
Burkina Faso	iLiNS−Zinc (37)												
Burkina Faso	PROMIS (38)	392	550	20.0	0.85 (0.65, 1.13)	0.07	0.07	465	357	19.3	0.87 (0.64, 1.17)	0.10	0.10
Burkina Faso	PROMIS CS (38)	176	165	23.0	1.04 (0.69, 1.55)	0.03	0.03	252	271	25.1	0.96 (0.73, 1.28)	0.11	0.11
Ghana	GHANA (39)												
Ghana	iLiNS−DYADG (40)												
Haiti	HAITI (41)												
Kenya	WASH−B (42)												
Madagascar	MAHAY (43)												
Malawi	iLiNS−DYADM (44)												
Malawi	iLiNS−DOSE (45)												
Mali	PROMIS (46)	381	363	16.1	0.86 (0.60, 1.23)	0.04	0.04	112	134	17.0	0.90 (0.55, 1.47)	0.04	0.04
Mali	PROMIS CS (46)	698	692	22.5	0.87 (0.67, 1.14)	0.07	0.07	217	243	21.4	0.88 (0.66, 1.18)	0.10	0.10
Zimbabwe	SHINE (HIV−) (47)	293	243	7.8	0.70 (0.35, 1.38)	0.01	0.01	495	543	9.9	0.93 (0.64, 1.36)	0.06	0.06
Zimbabwe	SHINE (HIV+) (48)												
		**5298**	**3530**		**I² = 0.00, Tau² = 0.00**			**2657**	**2077**		**I² = 0.00, Tau² = 0.00**		
**Fixed**					**0.91 (0.85, 0.98)**						**0.92 (0.84, 1.01)**		
**Random**					**0.91 (0.85, 0.98)**						**0.92 (0.84, 1.01)**		

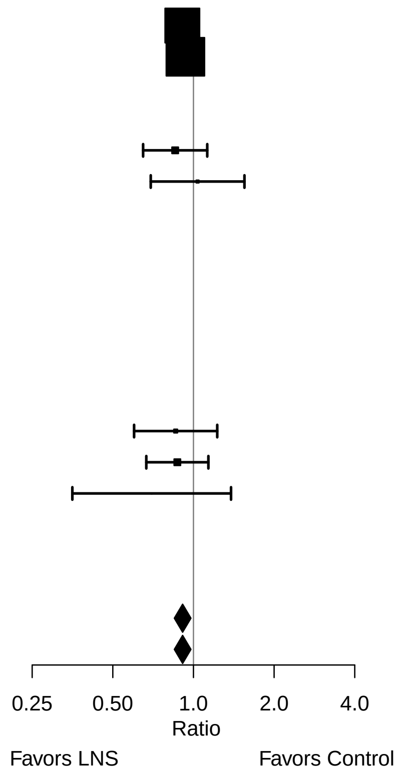

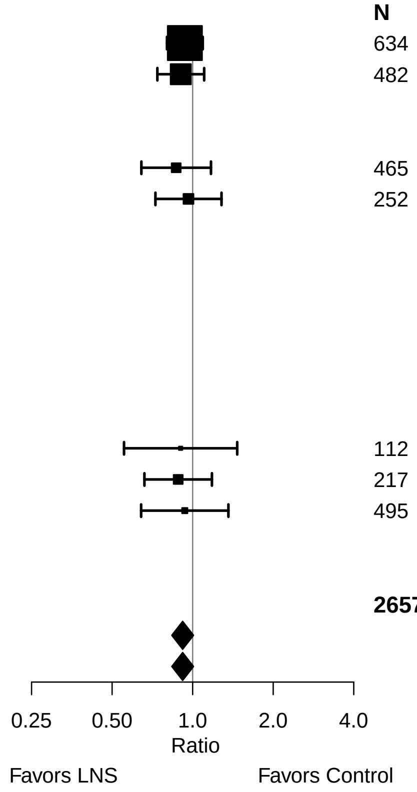

Supplemental figure 9M: Underweight prevalence ratio

9M5: Stratified by Home environment

P−for−interaction = **0.416**
Ratio of PRs = **1.05 (0.94, 1.17)**

		At least median						Less than median					
**Country**	**Trial**	**LNS N**	**Control N**	**Control Prevalence**	**PR (95% CI)**	**Fixed W**	**Random W**	**LNS N**	**Control N**	**Control Prevalence**	**PR (95% CI)**	**Fixed W**	**Random W**
Bangladesh	JiVitA−4 (34)												
Bangladesh	RDNS (35)	1192	582	36.1	0.89 (0.78, 1.01)	0.35	0.25	468	230	41.7	1.00 (0.85, 1.18)	0.28	0.22
Bangladesh	WASH−B (36)	641	1668	28.1	0.80 (0.68, 0.94)	0.23	0.21	494	1702	36.5	0.82 (0.69, 0.97)	0.25	0.21
Burkina Faso	iLiNS−Zinc (37)	473	199	27.6	0.64 (0.49, 0.84)	0.08	0.11	272	175	31.4	0.86 (0.67, 1.09)	0.13	0.15
Burkina Faso	PROMIS (38)												
Burkina Faso	PROMIS CS (38)												
Ghana	GHANA (39)												
Ghana	iLiNS−DYADG (40)	213	410	12.0	0.98 (0.62, 1.54)	0.03	0.05	119	248	15.3	0.55 (0.28, 1.06)	0.02	0.03
Haiti	HAITI (41)												
Kenya	WASH−B (42)	840	2776	8.0	0.88 (0.67, 1.16)	0.08	0.11	573	2167	10.8	0.79 (0.57, 1.09)	0.07	0.10
Madagascar	MAHAY (43)	915	907	24.8	1.07 (0.89, 1.29)	0.17	0.18	787	778	27.4	1.00 (0.82, 1.23)	0.19	0.18
Malawi	iLiNS−DYADM (44)	137	289	10.4	0.91 (0.49, 1.70)	0.01	0.03	78	149	14.1	1.46 (0.81, 2.63)	0.02	0.04
Malawi	iLiNS−DOSE (45)	379	136	22.8	0.90 (0.64, 1.25)	0.05	0.08	277	86	18.6	1.04 (0.67, 1.60)	0.04	0.06
Mali	PROMIS (46)												
Mali	PROMIS CS (46)												
Zimbabwe	SHINE (HIV−) (47)												
Zimbabwe	SHINE (HIV+) (48)												
		**4790**	**6967**		**I² = 0.38, Tau² = 0.01**			**3068**	**5535**		**I² = 0.26, Tau² = 0.01**		
**Fixed**					**0.88 (0.81, 0.95)**						**0.92 (0.84, 1.00)**		
**Random**					**0.87 (0.79, 0.97)**						**0.91 (0.81, 1.03)**		

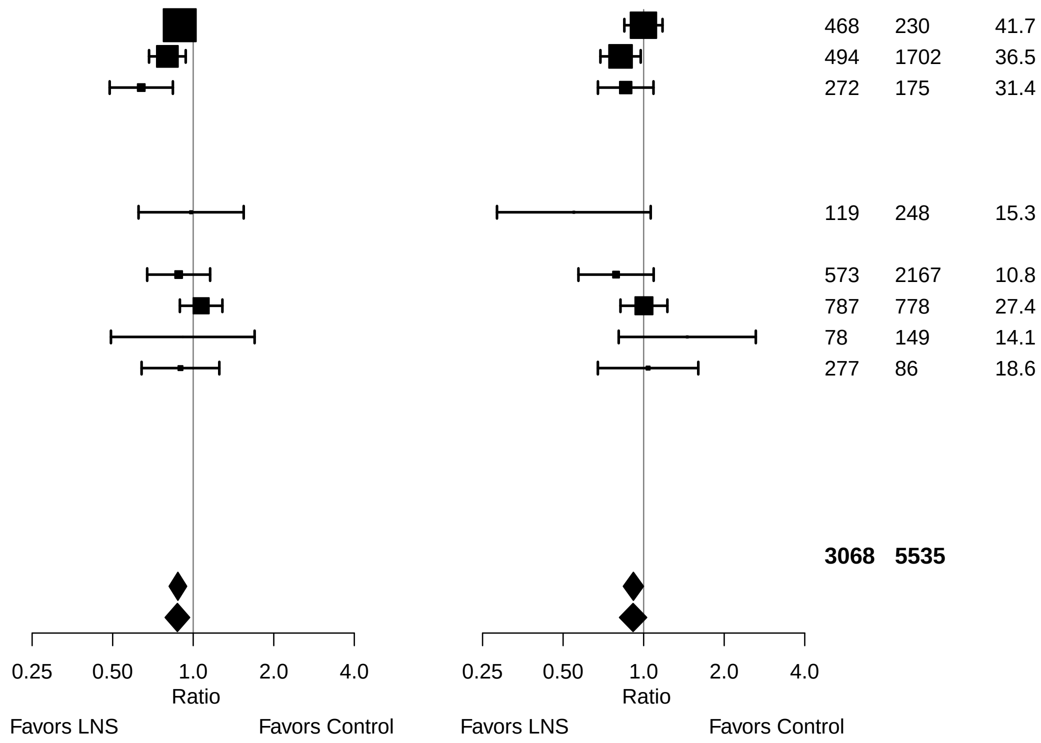

Supplemental figure 9M: Underweight prevalence ratio

9M6: Stratified by Season at the time of assessment

P−for−interaction = 0.102
Ratio of PRs = 1.08 (0.98, 1.19)

		Dry						Rainy					
**Country**	**Trial**	**LNS N**	**Control N**	**Control Prevalence**	**PR (95% CI)**	**Fixed W**	**Random W**	**LNS N**	**Control N**	**Control Prevalence**	**PR (95% CI)**	**Fixed W**	**Random W**
Bangladesh	JiVitA−4 (34)	1533	656	37.5	0.92 (0.80, 1.05)	0.26	0.17	1281	576	41.1	0.91 (0.80, 1.04)	0.24	0.21
Bangladesh	RDNS (35)	739	359	37.0	0.91 (0.75, 1.10)	0.13	0.13	924	456	38.4	0.93 (0.81, 1.07)	0.24	0.21
Bangladesh	WASH−B (36)	645	1938	28.3	0.76 (0.64, 0.91)	0.14	0.13	518	1523	37.7	0.81 (0.70, 0.93)	0.21	0.20
Burkina Faso	iLiNS−Zinc (37)	1400	477	31.7	0.66 (0.55, 0.78)	0.16	0.14	560	189	29.1	0.81 (0.65, 1.01)	0.09	0.10
Burkina Faso	PROMIS (38)	429	447	18.3	0.76 (0.59, 0.98)	0.07	0.09	428	464	20.9	0.95 (0.71, 1.27)	0.05	0.06
Burkina Faso	PROMIS CS (38)	224	190	23.2	0.91 (0.62, 1.32)	0.03	0.05	206	246	25.2	1.08 (0.74, 1.58)	0.03	0.04
Ghana	GHANA (39)												
Ghana	iLiNS−DYADG (40)	204	408	11.5	1.06 (0.67, 1.68)	0.02	0.04	143	285	15.1	0.56 (0.30, 1.02)	0.01	0.02
Haiti	HAITI (41)												
Kenya	WASH−B (42)	348	1157	10.1	0.65 (0.40, 1.06)	0.02	0.04	1113	4008	10.0	0.79 (0.63, 0.99)	0.09	0.10
Madagascar	MAHAY (43)												
Malawi	iLiNS−DYADM (44)	156	316	11.1	0.93 (0.53, 1.62)	0.02	0.03	64	128	13.3	1.53 (0.79, 2.95)	0.01	0.01
Malawi	iLiNS−DOSE (45)	421	134	20.1	1.06 (0.74, 1.51)	0.04	0.06	281	107	19.6	0.95 (0.65, 1.39)	0.03	0.04
Mali	PROMIS (46)												
Mali	PROMIS CS (46)												
Zimbabwe	SHINE (HIV−) (47)	1736	1655	10.8	0.67 (0.54, 0.83)	0.10	0.11	133	131	8.4	1.34 (0.60, 3.03)	0.01	0.01
Zimbabwe	SHINE (HIV+) (48)												
		**7835**	**7737**		**I² = 0.47, Tau² = 0.01**			**5651**	**8113**		**I² = 0.10, Tau² = 0.00**		
**Fixed**					**0.81 (0.76, 0.87)**						**0.88 (0.83, 0.94)**		
**Random**					**0.81 (0.73, 0.89)**						**0.88 (0.82, 0.95)**		

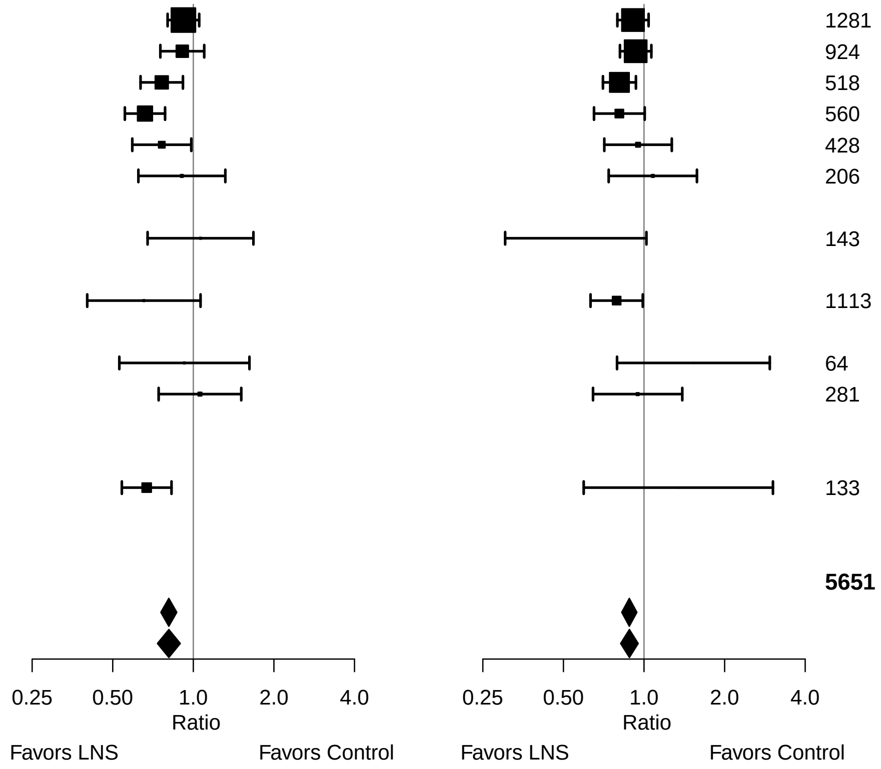

Supplemental figure 9N: Underweight prevalence difference

9N1: Stratified by Household socio-economic status

P−for−interaction = 0.123
Difference in PDs = −0.01 (−0.03, 0.00)

		At least median						Less than median					
**Country**	**Trial**	**LNS N**	**Control N**	**Control Prevalence**	**PD (95% CI)**	**Fixed W**	**Random W**	**LNS N**	**Control N**	**Control Prevalence**	**PD (95% CI)**	**Fixed W**	**Random W**
Bangladesh	JiVitA−4 (34)	1421	619	35.4	−0.05 (−0.09, −0.02)	0.08	0.10	1392	613	42.9	−0.03 (−0.07, 0.01)	0.12	0.10
Bangladesh	RDNS (35)	834	407	32.2	−0.03 (−0.08, 0.02)	0.04	0.06	829	408	43.4	−0.03 (−0.08, 0.02)	0.07	0.08
Bangladesh	WASH−B (36)	584	1741	23.9	−0.04 (−0.08, 0.01)	0.06	0.08	579	1720	41.0	−0.10 (−0.15, −0.06)	0.07	0.08
Burkina Faso	iLiNS−Zinc (37)	1233	300	28.0	−0.08 (−0.12, −0.03)	0.05	0.07	720	365	33.2	−0.12 (−0.15, −0.08)	0.11	0.10
Burkina Faso	PROMIS (38)	405	482	19.9	−0.04 (−0.10, 0.02)	0.03	0.04	452	429	19.3	−0.02 (−0.07, 0.04)	0.05	0.06
Burkina Faso	PROMIS CS (38)	220	208	21.6	0.02 (−0.05, 0.08)	0.02	0.03	210	228	26.8	−0.02 (−0.10, 0.06)	0.02	0.04
Ghana	GHANA (39)												
Ghana	iLiNS−DYADG (40)	157	356	11.0	−0.02 (−0.08, 0.04)	0.03	0.05	190	335	14.9	−0.03 (−0.09, 0.03)	0.04	0.06
Haiti	HAITI (41)												
Kenya	WASH−B (42)	828	2941	8.0	−0.03 (−0.04, −0.01)	0.35	0.18	631	2220	12.7	−0.02 (−0.05, 0.01)	0.17	0.11
Madagascar	MAHAY (43)	772	884	23.2	0.02 (−0.03, 0.06)	0.04	0.06	877	758	29.6	−0.01 (−0.07, 0.05)	0.04	0.06
Malawi	iLiNS−DYADM (44)	121	234	9.0	0.03 (−0.03, 0.10)	0.02	0.04	99	209	14.4	0.00 (−0.09, 0.08)	0.02	0.04
Malawi	iLiNS−DOSE (45)	307	104	10.6	0.02 (−0.05, 0.09)	0.02	0.03	294	103	30.1	−0.06 (−0.14, 0.02)	0.02	0.04
Mali	PROMIS (46)	247	250	15.5	−0.05 (−0.10, 0.00)	0.04	0.06	245	246	17.1	−0.03 (−0.10, 0.03)	0.04	0.05
Mali	PROMIS CS (46)	501	458	21.4	−0.02 (−0.07, 0.03)	0.04	0.05	445	502	23.5	−0.03 (−0.09, 0.03)	0.04	0.06
Zimbabwe	SHINE (HIV−) (47)	903	797	9.0	−0.02 (−0.05, 0.00)	0.16	0.13	826	858	12.5	−0.05 (−0.08, −0.02)	0.17	0.11
Zimbabwe	SHINE (HIV+) (48)	171	156	14.7	0.00 (−0.07, 0.08)	0.02	0.03	157	166	19.9	−0.01 (−0.10, 0.09)	0.02	0.03
		**8704**	**9937**		**I² = 0.24, Tau² = 0.00**			**7946**	**9160**		**I² = 0.49, Tau² = 0.00**		
**Fixed**					**−0.03 (−0.04, −0.02)**						**−0.04 (−0.06, −0.03)**		
**Random**					**−0.03 (−0.04, −0.01)**						**−0.04 (−0.06, −0.02)**		

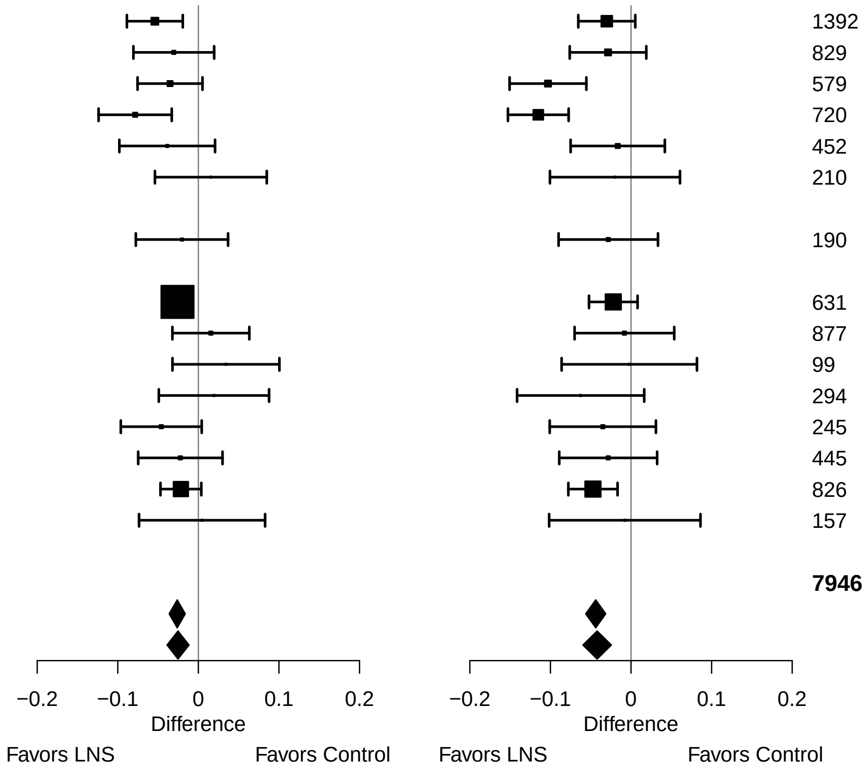

Supplemental figure 9N: Underweight prevalence difference

9N2: Stratified by Household food insecurity

P−for−interaction = 0.068
Difference in PDs = −0.02 (−0.04, 0.00)

		Mild to secure						Moderate to severe					
**Country**	**Trial**	**LNS N**	**Control N**	**Control Prevalence**	**PD (95% CI)**	**Fixed W**	**Random W**	**LNS N**	**Control N**	**Control Prevalence**	**PD (95% CI)**	**Fixed W**	**Random W**
Bangladesh	JiVitA−4 (34)	2000	851	37.0	−0.05 (−0.08, −0.02)	0.10	0.11	813	381	43.9	−0.03 (−0.07, 0.02)	0.13	0.11
Bangladesh	RDNS (35)	1058	493	32.9	0.00 (−0.05, 0.05)	0.04	0.07	605	322	45.3	−0.07 (−0.13, 0.00)	0.07	0.07
Bangladesh	WASH−B (36)	923	2681	30.8	−0.07 (−0.11, −0.04)	0.09	0.10	240	780	38.1	−0.04 (−0.12, 0.03)	0.06	0.06
Burkina Faso	iLiNS−Zinc (37)	1017	321	27.4	−0.08 (−0.13, −0.03)	0.04	0.07	936	344	34.0	−0.12 (−0.16, −0.08)	0.18	0.13
Burkina Faso	PROMIS (38)	502	550	19.3	−0.01 (−0.06, 0.04)	0.04	0.07	355	361	20.2	−0.05 (−0.12, 0.01)	0.07	0.08
Burkina Faso	PROMIS CS (38)												
Ghana	GHANA (39)												
Ghana	iLiNS−DYADG (40)	244	471	12.7	−0.03 (−0.08, 0.02)	0.04	0.07	102	219	13.7	−0.01 (−0.09, 0.07)	0.05	0.06
Haiti	HAITI (41)												
Kenya	WASH−B (42)	1303	4621	9.8	−0.02 (−0.04, −0.01)	0.34	0.14	156	527	12.1	−0.04 (−0.10, 0.02)	0.08	0.09
Madagascar	MAHAY (43)	989	929	25.6	0.01 (−0.04, 0.06)	0.04	0.07	354	410	27.3	0.01 (−0.08, 0.10)	0.04	0.05
Malawi	iLiNS−DYADM (44)	70	122	8.2	0.05 (−0.04, 0.13)	0.01	0.03	148	316	12.7	0.01 (−0.06, 0.07)	0.07	0.07
Malawi	iLiNS−DOSE (45)	154	55	14.5	0.00 (−0.09, 0.10)	0.01	0.02	434	146	21.9	−0.03 (−0.09, 0.04)	0.07	0.08
Mali	PROMIS (46)	327	338	15.2	−0.03 (−0.08, 0.01)	0.04	0.07	170	163	19.0	−0.05 (−0.12, 0.03)	0.06	0.06
Mali	PROMIS CS (46)												
Zimbabwe	SHINE (HIV−) (47)	1384	1299	9.8	−0.02 (−0.05, 0.00)	0.19	0.13	294	324	15.4	−0.08 (−0.13, −0.03)	0.11	0.10
Zimbabwe	SHINE (HIV+) (48)	236	238	17.6	0.01 (−0.06, 0.07)	0.02	0.05	85	76	17.1	−0.02 (−0.13, 0.09)	0.02	0.03
		**10207**	**12969**		**I² = 0.45, Tau² = 0.00**			**4692**	**4369**		**I² = 0.41, Tau² = 0.00**		
**Fixed**					**−0.03 (−0.04, −0.02)**						**−0.05 (−0.07, −0.03)**		
**Random**					**−0.03 (−0.04, −0.01)**						**−0.05 (−0.07, −0.03)**		

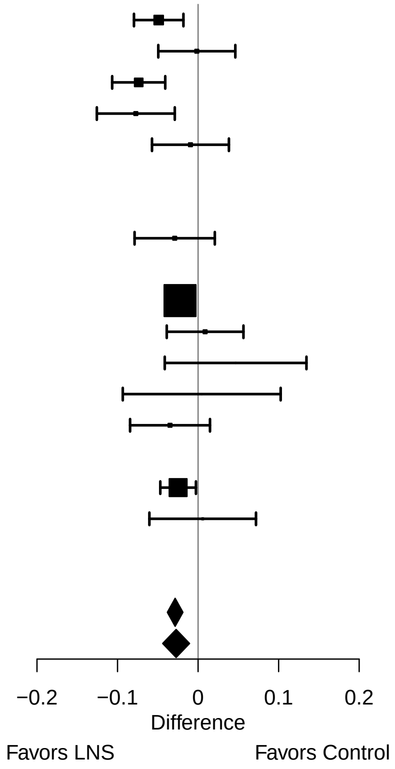

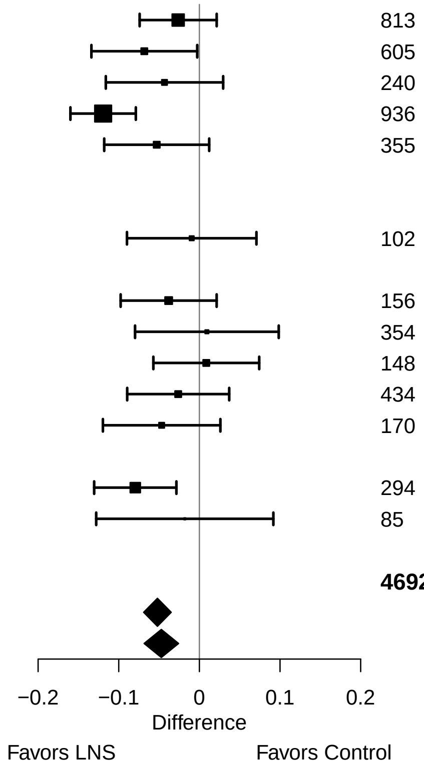

Supplemental figure 9N: Underweight prevalence difference

9N3: Stratified by Household source water quality

P−for−interaction = 0.232
Difference in PDs = −0.01 (−0.04, 0.01)

Country	Trial	LNS N	Control N	Control Prevalence	Improved PD (95% CI)	Fixed W	Random W	LNS N	Control N	Control Prevalence	Unimproved PD (95% CI)	Fixed W	Random W
Bangladesh	JiVitA−4 (34)												
Bangladesh	RDNS (35)												
Bangladesh	WASH−B (36)	501	1002	30.7	−0.06 (−0.10, −0.01)	0.11	0.12	70	116	29.3	0.04 (−0.10, 0.17)	0.02	0.04
Burkina Faso	iLiNS−Zinc (37)	555	141	27.7	−0.06 (−0.12, −0.01)	0.09	0.10	1397	521	31.9	−0.11 (−0.15, −0.08)	0.25	0.14
Burkina Faso	PROMIS (38)	352	379	17.9	0.00 (−0.05, 0.06)	0.08	0.10	358	351	23.4	−0.10 (−0.16, −0.04)	0.08	0.11
Burkina Faso	PROMIS CS (38)	280	267	27.3	−0.05 (−0.12, 0.03)	0.04	0.06	149	169	19.5	0.07 (−0.01, 0.16)	0.04	0.08
Ghana	GHANA (39)												
Ghana	iLiNS−DYADG (40)												
Haiti	HAITI (41)												
Kenya	WASH−B (42)	449	1555	8.8	−0.01 (−0.04, 0.02)	0.26	0.17	248	699	10.7	−0.02 (−0.07, 0.03)	0.14	0.13
Madagascar	MAHAY (43)	418	454	20.5	0.05 (−0.01, 0.12)	0.06	0.08	1204	1182	28.1	−0.01 (−0.05, 0.03)	0.16	0.13
Malawi	iLiNS−DYADM (44)												
Malawi	iLiNS−DOSE (45)												
Mali	PROMIS (46)	247	257	14.3	−0.04 (−0.10, 0.02)	0.07	0.09	243	240	18.5	−0.04 (−0.10, 0.02)	0.08	0.10
Mali	PROMIS CS (46)	544	563	21.5	−0.03 (−0.08, 0.03)	0.07	0.09	363	382	23.6	−0.02 (−0.09, 0.05)	0.06	0.10
Zimbabwe	SHINE (HIV−) (47)	518	486	9.7	−0.02 (−0.06, 0.01)	0.20	0.15	273	301	8.6	0.00 (−0.04, 0.05)	0.16	0.13
Zimbabwe	SHINE (HIV+) (48)	85	87	13.8	0.04 (−0.07, 0.15)	0.02	0.04	55	56	21.4	−0.03 (−0.17, 0.11)	0.02	0.04
		**3949**	**5191**		**I² = 0.36, Tau² = 0.00**			**4360**	**4017**		**I² = 0.73, Tau² = 0.00**		
**Fixed**					**−0.02 (−0.04, 0.00)**						**−0.04 (−0.06, −0.02)**		
**Random**					**−0.02 (−0.04, 0.00)**						**−0.03 (−0.06, 0.00)**		

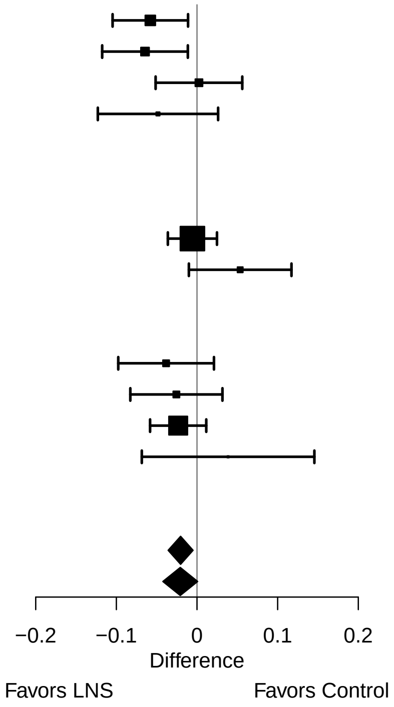

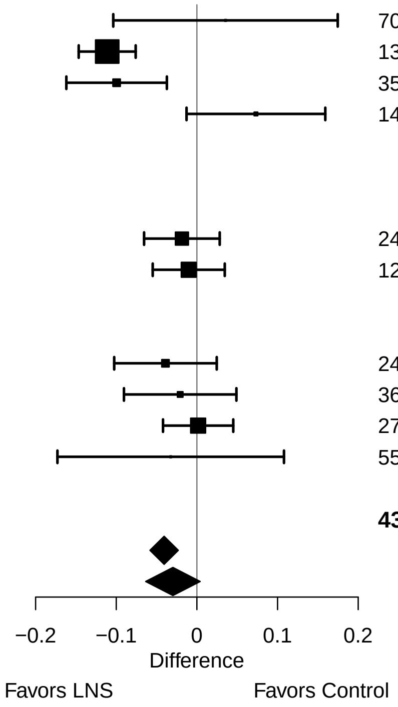

Supplemental figure 9N: Underweight prevalence difference

9N4: Stratified by Household sanitation

P−for−interaction = 0.679
Difference in PDs = 0.01 (−0.02, 0.03)

Country	Trial	Improved LNS N	Improved Control N	Improved Control Prevalence	Improved PD (95% CI)	Improved Fixed W	Improved Random W	Unimproved LNS N	Unimproved Control N	Unimproved Control Prevalence	Unimproved PD (95% CI)	Unimproved Fixed W	Unimproved Random W
Bangladesh	JiVitA−4 (34)	2179	944	37.7	−0.05 (−0.07, −0.02)	0.36	0.36	634	288	43.9	−0.03 (−0.08, 0.02)	0.17	0.17
Bangladesh	RDNS (35)	1179	573	34.9	−0.02 (−0.06, 0.01)	0.19	0.19	482	241	44.8	−0.04 (−0.13, 0.04)	0.06	0.06
Bangladesh	WASH−B (36)												
Burkina Faso	iLiNS−Zinc (37)												
Burkina Faso	PROMIS (38)	392	550	20.0	−0.03 (−0.08, 0.02)	0.10	0.10	465	357	19.3	−0.03 (−0.08, 0.03)	0.14	0.14
Burkina Faso	PROMIS CS (38)	176	165	23.0	0.01 (−0.09, 0.10)	0.03	0.03	252	271	25.1	−0.01 (−0.08, 0.06)	0.09	0.09
Ghana	GHANA (39)												
Ghana	iLiNS−DYADG (40)												
Haiti	HAITI (41)												
Kenya	WASH−B (42)												
Madagascar	MAHAY (43)												
Malawi	iLiNS−DYADM (44)												
Malawi	iLiNS−DOSE (45)												
Mali	PROMIS (46)	381	363	16.1	−0.04 (−0.09, 0.01)	0.11	0.11	112	134	17.0	−0.03 (−0.10, 0.04)	0.09	0.09
Mali	PROMIS CS (46)	698	692	22.5	−0.03 (−0.09, 0.03)	0.08	0.08	217	243	21.4	−0.03 (−0.08, 0.03)	0.13	0.13
Zimbabwe	SHINE (HIV−) (47)	293	243	7.8	−0.02 (−0.07, 0.02)	0.13	0.13	495	543	9.9	−0.01 (−0.04, 0.03)	0.33	0.33
Zimbabwe	SHINE (HIV+) (48)												
		**5298**	**3530**		**I² = 0.00, Tau² = 0.00**			**2657**	**2077**		**I² = 0.00, Tau² = 0.00**		
**Fixed**					**−0.03 (−0.05, −0.02)**						**−0.02 (−0.04, 0.00)**		
**Random**					**−0.03 (−0.05, −0.02)**						**−0.02 (−0.04, 0.00)**		

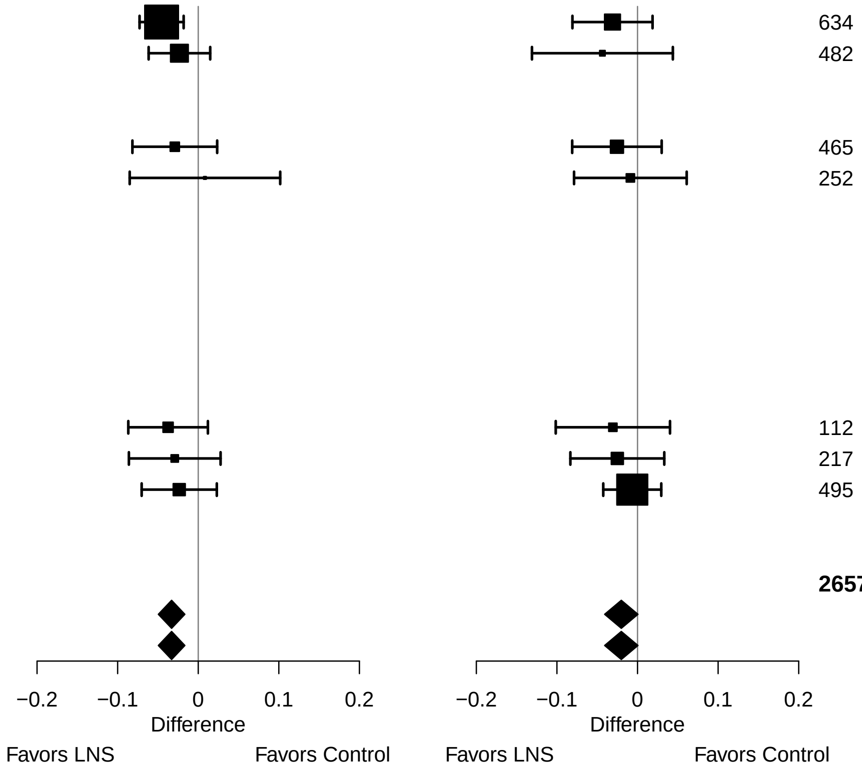

Supplemental figure 9N: Underweight prevalence difference

9N5: Stratified by Home environment

P−for−interaction = 0.750
Difference in PDs = 0.00 (−0.03, 0.02)

Country	Trial	At least median: LNS N	Control N	Control Prevalence	PD (95% CI)	Fixed W	Random W	Less than median: LNS N	Control N	Control Prevalence	PD (95% CI)	Fixed W	Random W
Bangladesh	JiVitA−4 (34)												
Bangladesh	RDNS (35)	1192	582	36.1	−0.04 (−0.08, 0.01)	0.09	0.13	468	230	41.7	0.00 (−0.07, 0.07)	0.07	0.09
Bangladesh	WASH−B (36)	641	1668	28.1	−0.06 (−0.09, −0.02)	0.13	0.14	494	1702	36.5	−0.07 (−0.12, −0.01)	0.12	0.13
Burkina Faso	iLiNS−Zinc (37)	473	199	27.6	−0.09 (−0.14, −0.05)	0.08	0.12	272	175	31.4	−0.03 (−0.09, 0.02)	0.12	0.14
Burkina Faso	PROMIS (38)												
Burkina Faso	PROMIS CS (38)												
Ghana	GHANA (39)												
Ghana	iLiNS−DYADG (40)	213	410	12.0	0.00 (−0.06, 0.05)	0.06	0.11	119	248	15.3	−0.07 (−0.14, 0.00)	0.06	0.08
Haiti	HAITI (41)												
Kenya	WASH−B (42)	840	2776	8.0	−0.01 (−0.03, 0.01)	0.47	0.19	573	2167	10.8	−0.02 (−0.05, 0.01)	0.42	0.30
Madagascar	MAHAY (43)	915	907	24.8	0.02 (−0.03, 0.06)	0.08	0.12	787	778	27.4	0.00 (−0.05, 0.06)	0.11	0.13
Malawi	iLiNS−DYADM (44)	137	289	10.4	−0.01 (−0.07, 0.05)	0.05	0.09	78	149	14.1	0.06 (−0.04, 0.17)	0.03	0.05
Malawi	iLiNS−DOSE (45)	379	136	22.8	−0.06 (−0.12, 0.01)	0.04	0.09	277	86	18.6	0.00 (−0.08, 0.08)	0.05	0.07
Mali	PROMIS (46)												
Mali	PROMIS CS (46)												
Zimbabwe	SHINE (HIV−) (47)												
Zimbabwe	SHINE (HIV+) (48)												
		**4790**	**6967**		**I² = 0.63, Tau² = 0.00**			**3068**	**5535**		**I² = 0.15, Tau² = 0.00**		
**Fixed**					**−0.02 (−0.04, −0.01)**						**−0.02 (−0.04, −0.01)**		
**Random**					**−0.03 (−0.06, −0.01)**						**−0.02 (−0.05, 0.00)**		

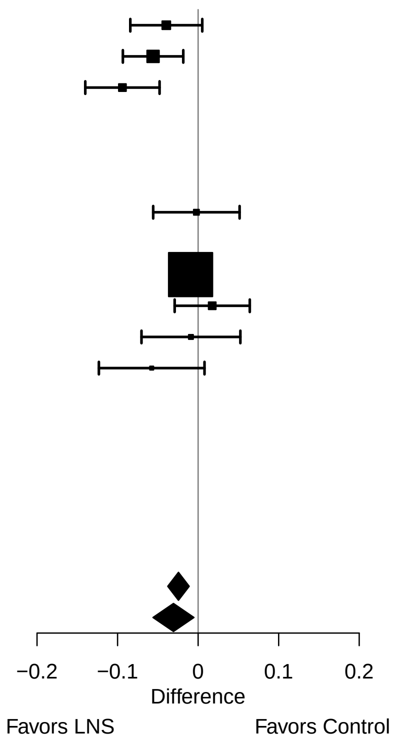

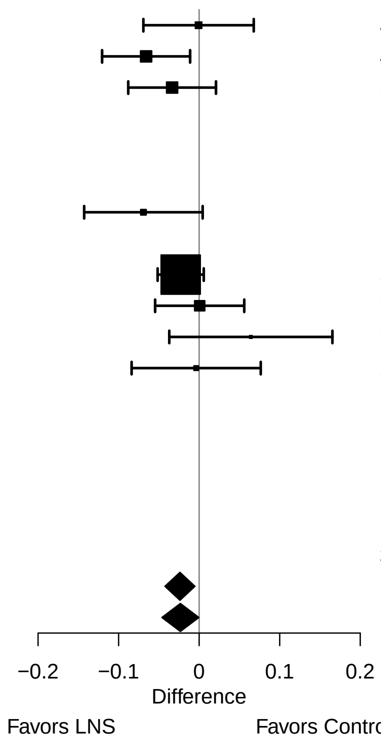

Supplemental figure 9N: Underweight prevalence difference

9N6: Stratified by Season at the time of assessment

P−for−interaction = 0.254
Difference in PDs = 0.01 (−0.01, 0.03)

		Dry						Rainy					
**Country**	**Trial**	**LNS N**	**Control N**	**Control Prevalence**	**PD (95% CI)**	**Fixed W**	**Random W**	**LNS N**	**Control N**	**Control Prevalence**	**PD (95% CI)**	**Fixed W**	**Random W**
Bangladesh	JiVitA−4 (34)	1533	656	37.5	−0.04 (−0.08, −0.01)	0.12	0.12	1281	576	41.1	−0.04 (−0.08, 0.00)	0.12	0.13
Bangladesh	RDNS (35)	739	359	37.0	−0.03 (−0.10, 0.03)	0.03	0.05	924	456	38.4	−0.03 (−0.08, 0.02)	0.07	0.10
Bangladesh	WASH−B (36)	645	1938	28.3	−0.07 (−0.11, −0.03)	0.08	0.10	518	1523	37.7	−0.07 (−0.12, −0.03)	0.08	0.11
Burkina Faso	iLiNS−Zinc (37)	1400	477	31.7	−0.10 (−0.14, −0.06)	0.08	0.10	560	189	29.1	−0.09 (−0.15, −0.03)	0.05	0.09
Burkina Faso	PROMIS (38)	429	447	18.3	−0.04 (−0.08, 0.00)	0.08	0.10	428	464	20.9	−0.01 (−0.07, 0.05)	0.05	0.09
Burkina Faso	PROMIS CS (38)	224	190	23.2	−0.02 (−0.10, 0.06)	0.02	0.04	206	246	25.2	0.02 (−0.08, 0.12)	0.02	0.04
Ghana	GHANA (39)												
Ghana	iLiNS−DYADG (40)	204	408	11.5	0.01 (−0.05, 0.06)	0.05	0.07	143	285	15.1	−0.07 (−0.13, 0.00)	0.04	0.08
Haiti	HAITI (41)												
Kenya	WASH−B (42)	348	1157	10.1	−0.04 (−0.07, 0.00)	0.12	0.12	1113	4008	10.0	−0.02 (−0.04, 0.00)	0.50	0.18
Madagascar	MAHAY (43)												
Malawi	iLiNS−DYADM (44)	156	316	11.1	−0.01 (−0.07, 0.05)	0.04	0.06	64	128	13.3	0.07 (−0.04, 0.18)	0.01	0.04
Malawi	iLiNS−DOSE (45)	421	134	20.1	−0.01 (−0.08, 0.05)	0.03	0.06	281	107	19.6	−0.03 (−0.10, 0.05)	0.03	0.07
Mali	PROMIS (46)												
Mali	PROMIS CS (46)												
Zimbabwe	SHINE (HIV−) (47)	1736	1655	10.8	−0.04 (−0.05, −0.02)	0.36	0.17	133	131	8.4	0.03 (−0.05, 0.11)	0.03	0.06
Zimbabwe	SHINE (HIV+) (48)												
		**7835**	**7737**		**I² = 0.39, Tau² = 0.00**			**5651**	**8113**		**I² = 0.39, Tau² = 0.00**		
**Fixed**					**−0.04 (−0.05, −0.03)**						**−0.03 (−0.04, −0.02)**		
**Random**					**−0.04 (−0.06, −0.02)**						**−0.03 (−0.05, −0.01)**		

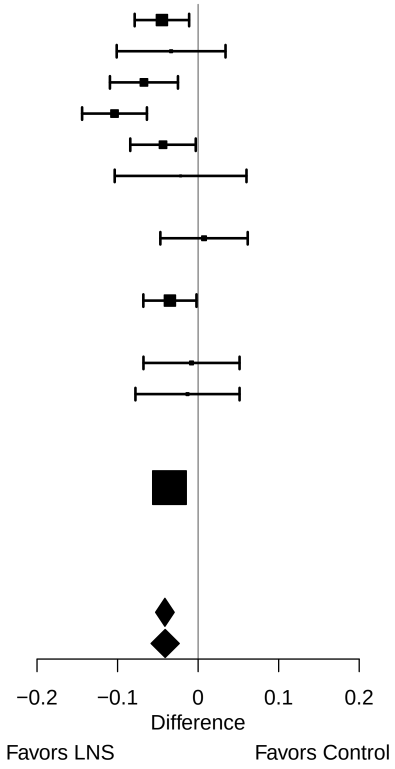

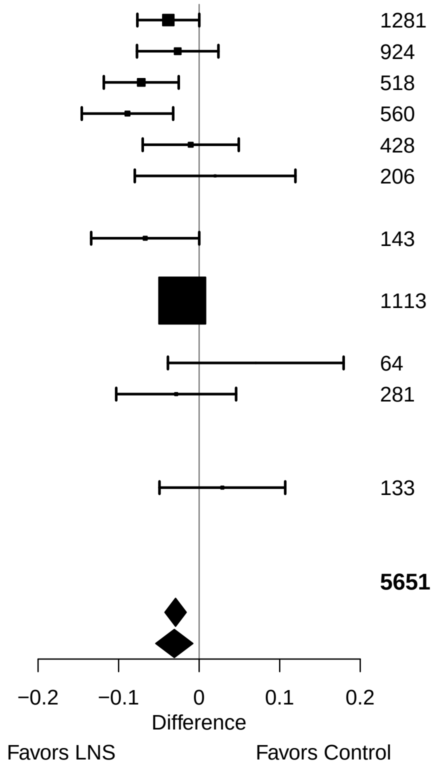

Supplemental figure 9O: Mean difference in HCZ

9O1: Stratified by Household socio-economic status

P−for−interaction = 0.842
Difference in MDs = 0.00 (−0.04, 0.04)

		At least median						Less than median					
**Country**	**Trial**	**LNS N**	**Control N**	**Control Mean**	**MD (95% CI)**	**Fixed W**	**Random W**	**LNS N**	**Control N**	**Control Mean**	**MD (95% CI)**	**Fixed W**	**Random W**
Bangladesh	JiVitA−4 (34)	1456	640	−1.36	0.07 (0.00, 0.13)	0.22	0.17	1428	627	−1.54	0.05 (0.00, 0.10)	0.32	0.26
Bangladesh	RDNS (35)	834	407	−1.78	0.06 (−0.04, 0.17)	0.09	0.11	829	408	−1.95	0.14 (0.05, 0.22)	0.12	0.13
Bangladesh	WASH−B (36)	583	1740	−1.50	0.15 (0.05, 0.24)	0.11	0.12	580	1723	−1.73	0.10 (0.02, 0.18)	0.13	0.14
Burkina Faso	iLiNS−Zinc (37)	1226	299	−1.81	0.15 (0.09, 0.22)	0.23	0.17	713	364	−1.86	0.18 (0.09, 0.27)	0.12	0.13
Burkina Faso	PROMIS (38)												
Burkina Faso	PROMIS CS (38)												
Ghana	GHANA (39)	57	44	−0.35	0.22 (−0.18, 0.61)	0.01	0.01	41	46	−0.79	0.00 (−0.30, 0.31)	0.01	0.01
Ghana	iLiNS−DYADG (40)	157	355	−1.05	0.03 (−0.13, 0.20)	0.04	0.06	190	335	−1.23	0.11 (−0.05, 0.27)	0.04	0.04
Haiti	HAITI (41)												
Kenya	WASH−B (42)	827	2946	−0.20	0.04 (−0.04, 0.12)	0.16	0.15	632	2226	−0.39	0.05 (−0.04, 0.14)	0.12	0.13
Madagascar	MAHAY (43)												
Malawi	iLiNS−DYADM (44)	121	234	−0.70	−0.16 (−0.37, 0.04)	0.02	0.04	99	209	−0.85	−0.10 (−0.35, 0.14)	0.02	0.02
Malawi	iLiNS−DOSE (45)	302	102	−1.09	0.11 (−0.08, 0.30)	0.03	0.04	290	102	−1.19	0.07 (−0.11, 0.26)	0.03	0.03
Mali	PROMIS (46)												
Mali	PROMIS CS (46)												
Zimbabwe	SHINE (HIV−) (47)	905	799	−0.22	0.15 (0.05, 0.25)	0.09	0.11	826	853	−0.35	0.04 (−0.06, 0.14)	0.09	0.10
Zimbabwe	SHINE (HIV+) (48)	171	156	−0.57	0.12 (−0.14, 0.38)	0.01	0.03	158	167	−0.53	0.08 (−0.16, 0.33)	0.02	0.02
		**6639**	**7722**		**I² = 0.36, Tau² = 0.00**			**5786**	**7060**		**I² = 0.15, Tau² = 0.00**		
**Fixed**					**0.10 (0.06, 0.13)**						**0.08 (0.05, 0.11)**		
**Random**					**0.09 (0.05, 0.13)**						**0.08 (0.05, 0.12)**		

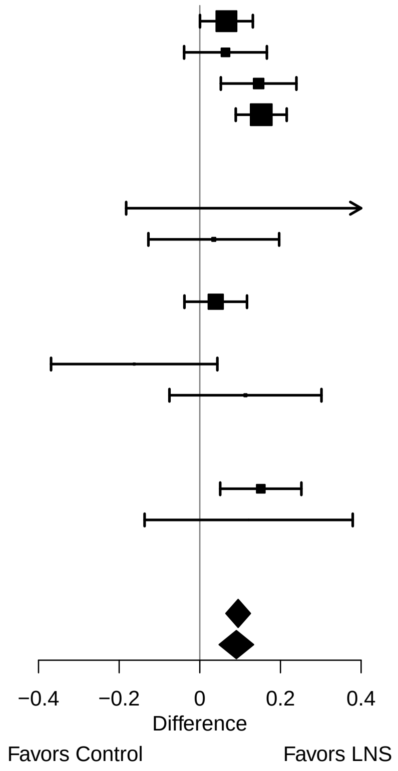

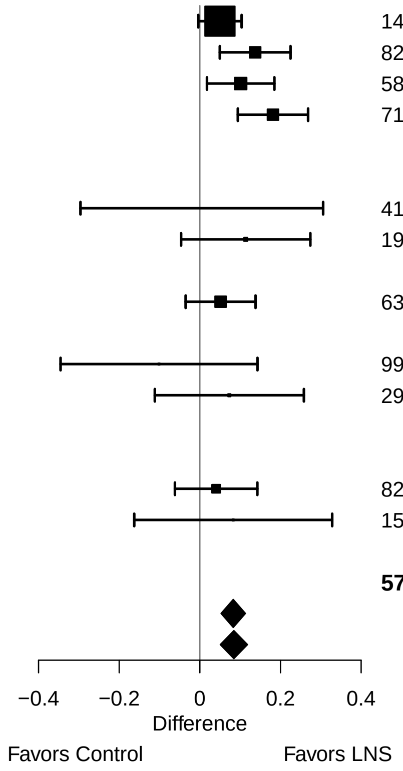

Supplemental figure 9O: Mean difference in HCZ

9O2: Stratified by Household food insecurity

P−for−interaction = 0.603
Difference in MDs = −0.01 (−0.06, 0.03)

		Mild to secure						Moderate to severe					
**Country**	**Trial**	**LNS N**	**Control N**	**Control Mean**	**MD (95% CI)**	**Fixed W**	**Random W**	**LNS N**	**Control N**	**Control Mean**	**MD (95% CI)**	**Fixed W**	**Random W**
Bangladesh	JiVitA−4 (34)	2045	882	−1.41	0.07 (0.02, 0.13)	0.23	0.22	839	385	−1.53	0.02 (−0.04, 0.09)	0.34	0.18
Bangladesh	RDNS (35)	1058	493	−1.80	0.07 (−0.02, 0.16)	0.09	0.09	605	322	−1.96	0.14 (0.03, 0.24)	0.14	0.14
Bangladesh	WASH−B (36)	922	2682	−1.58	0.12 (0.04, 0.19)	0.14	0.14	241	781	−1.74	0.13 (0.00, 0.27)	0.09	0.11
Burkina Faso	iLiNS−Zinc (37)	1008	320	−1.78	0.15 (0.09, 0.22)	0.19	0.19	931	343	−1.89	0.17 (0.08, 0.27)	0.19	0.16
Burkina Faso	PROMIS (38)												
Burkina Faso	PROMIS CS (38)												
Ghana	GHANA (39)												
Ghana	iLiNS−DYADG (40)	244	470	−1.12	0.08 (−0.06, 0.21)	0.04	0.04	102	219	−1.19	0.04 (−0.17, 0.25)	0.03	0.06
Haiti	HAITI (41)												
Kenya	WASH−B (42)	1303	4632	−0.27	0.04 (−0.02, 0.11)	0.16	0.16	156	527	−0.33	0.04 (−0.15, 0.23)	0.04	0.07
Madagascar	MAHAY (43)												
Malawi	iLiNS−DYADM (44)	70	122	−0.77	−0.06 (−0.34, 0.21)	0.01	0.01	148	316	−0.77	−0.16 (−0.36, 0.03)	0.04	0.07
Malawi	iLiNS−DOSE (45)	149	55	−1.06	0.04 (−0.21, 0.29)	0.01	0.01	431	143	−1.18	0.12 (−0.04, 0.28)	0.06	0.09
Mali	PROMIS (46)												
Mali	PROMIS CS (46)												
Zimbabwe	SHINE (HIV−) (47)	1385	1297	−0.28	0.13 (0.05, 0.22)	0.11	0.11	295	323	−0.30	−0.05 (−0.22, 0.13)	0.05	0.08
Zimbabwe	SHINE (HIV+) (48)	236	239	−0.52	0.05 (−0.17, 0.28)	0.01	0.02	86	76	−0.60	0.20 (−0.13, 0.53)	0.01	0.03
		**8420**	**11192**		**I² = 0.05, Tau² = 0.00**			**3834**	**3435**		**I² = 0.48, Tau² = 0.00**		
**Fixed**					**0.09 (0.07, 0.12)**						**0.08 (0.04, 0.12)**		
**Random**					**0.09 (0.07, 0.12)**						**0.07 (0.01, 0.13)**		

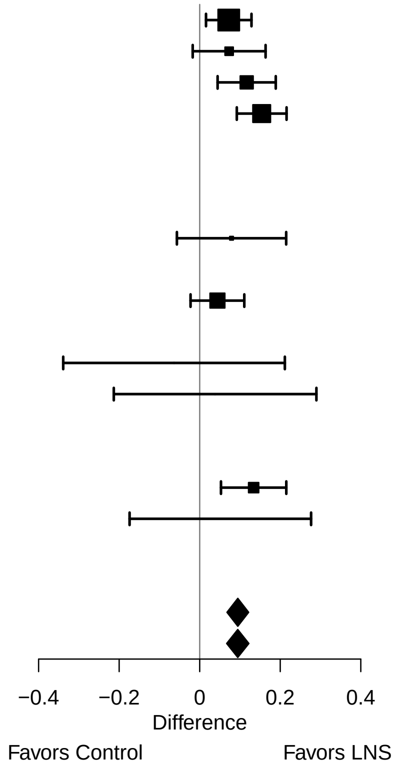

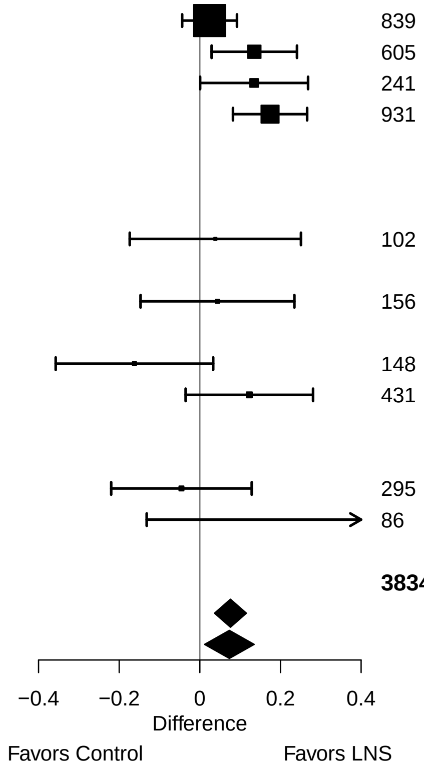

Supplemental figure 9O: Mean difference in HCZ

9O3: Stratified by Household source water quality

P−for−interaction = 0.341
Difference in MDs = 0.04 (−0.04, 0.12)

Country	Trial	Improved LNS N	Improved Control N	Improved Control Mean	Improved MD (95% CI)	Improved Fixed W	Improved Random W	Unimproved LNS N	Unimproved Control N	Unimproved Control Mean	Unimproved MD (95% CI)	Unimproved Fixed W	Unimproved Random W
Bangladesh	JiVitA−4 (34)												
Bangladesh	RDNS (35)												
Bangladesh	WASH−B (36)	500	1004	−1.62	0.17 (0.05, 0.28)	0.18	0.17	70	116	−1.58	0.10 (−0.19, 0.38)	0.04	0.06
Burkina Faso	iLiNS−Zinc (37)	552	141	−1.77	0.11 (0.01, 0.20)	0.28	0.20	1386	519	−1.85	0.18 (0.11, 0.25)	0.69	0.54
Burkina Faso	PROMIS (38)												
Burkina Faso	PROMIS CS (38)												
Ghana	GHANA (39)												
Ghana	iLiNS−DYADG (40)												
Haiti	HAITI (41)												
Kenya	WASH−B (42)	448	1560	−0.26	0.02 (−0.09, 0.14)	0.18	0.17	248	699	−0.31	0.07 (−0.10, 0.23)	0.12	0.17
Madagascar	MAHAY (43)												
Malawi	iLiNS−DYADM (44)	200	406	−0.75	−0.15 (−0.32, 0.01)	0.09	0.12	20	36	−1.01	0.11 (−0.48, 0.70)	0.01	0.02
Malawi	iLiNS−DOSE (45)	553	192	−1.14	0.08 (−0.06, 0.22)	0.12	0.14	51	15	−1.26	0.39 (−0.04, 0.81)	0.02	0.03
Mali	PROMIS (46)												
Mali	PROMIS CS (46)												
Zimbabwe	SHINE (HIV−) (47)	518	485	−0.28	0.09 (−0.04, 0.23)	0.13	0.15	272	299	−0.22	−0.05 (−0.24, 0.14)	0.10	0.14
Zimbabwe	SHINE (HIV+) (48)	85	87	−0.55	0.00 (−0.37, 0.36)	0.02	0.04	55	56	−0.58	0.13 (−0.23, 0.48)	0.03	0.04
		**2856**	**3875**		**I² = 0.47, Tau² = 0.00**			**2102**	**1740**		**I² = 0.20, Tau² = 0.00**		
**Fixed**					**0.07 (0.02, 0.12)**						**0.14 (0.09, 0.20)**		
**Random**					**0.06 (−0.01, 0.13)**						**0.13 (0.05, 0.20)**		

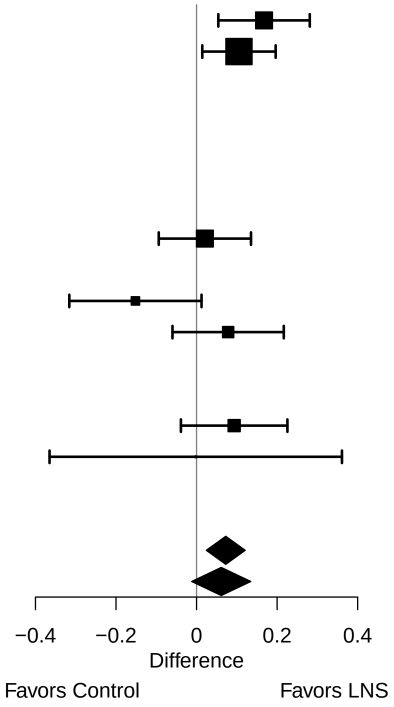

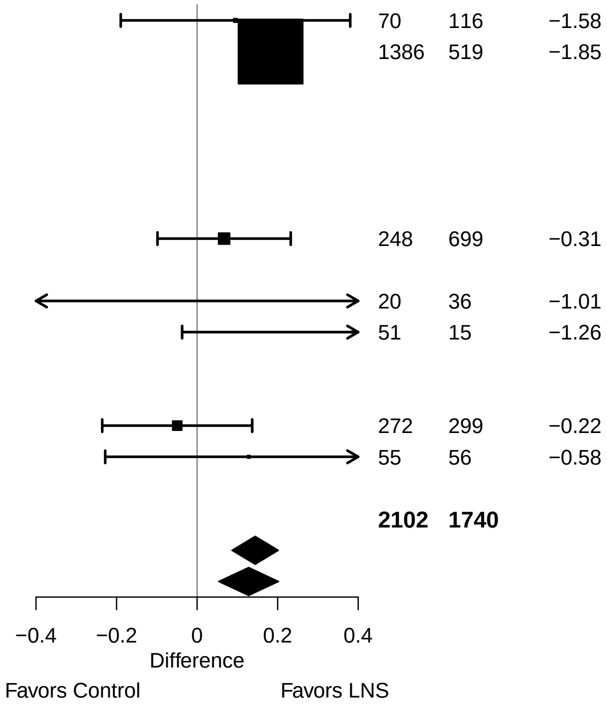

Supplemental figure 9O: Mean difference in HCZ

9O4: Stratified by Household sanitation

P−for−interaction = 0.339
Difference in MDs = −0.03 (−0.09, 0.03)

Country	Trial	Improved LNS N	Improved Control N	Improved Control Mean	Improved MD (95% CI)	Improved Fixed W	Improved Random W	Unimproved LNS N	Unimproved Control N	Unimproved Control Mean	Unimproved MD (95% CI)	Unimproved Fixed W	Unimproved Random W
Bangladesh	JiVitA−4 (34)	2231	972	−1.41	0.07 (0.01, 0.12)	0.48	0.48	653	295	−1.57	0.02 (−0.05, 0.10)	0.22	0.19
Bangladesh	RDNS (35)	1179	573	−1.86	0.12 (0.04, 0.21)	0.20	0.20	482	241	−1.88	0.05 (−0.05, 0.15)	0.12	0.14
Bangladesh	WASH−B (36)	510	1017	−1.61	0.16 (0.04, 0.27)	0.11	0.11	31	55	−1.56	0.04 (−0.32, 0.40)	0.01	0.02
Burkina Faso	iLiNS−Zinc (37)	43	16	−1.95	0.18 (−0.11, 0.47)	0.02	0.02	1895	644	−1.83	0.16 (0.10, 0.22)	0.33	0.22
Burkina Faso	PROMIS (38)												
Burkina Faso	PROMIS CS (38)												
Ghana	GHANA (39)												
Ghana	iLiNS−DYADG (40)	336	671	−1.14	0.07 (−0.05, 0.19)	0.11	0.11	10	19	−1.29	0.00 (−0.66, 0.66)	0.00	0.01
Haiti	HAITI (41)												
Kenya	WASH−B (42)	100	366	−0.11	−0.09 (−0.29, 0.11)	0.04	0.04	596	1894	−0.31	0.06 (−0.04, 0.15)	0.13	0.15
Madagascar	MAHAY (43)												
Malawi	iLiNS−DYADM (44)	20	42	−0.61	−0.16 (−0.58, 0.26)	0.01	0.01	200	400	−0.79	−0.13 (−0.30, 0.04)	0.04	0.06
Malawi	iLiNS−DOSE (45)	15	9	−1.02	0.23 (−0.62, 1.08)	0.00	0.00	588	198	−1.15	0.11 (−0.02, 0.24)	0.07	0.09
Mali	PROMIS (46)												
Mali	PROMIS CS (46)												
Zimbabwe	SHINE (HIV−) (47)	293	243	−0.21	0.05 (−0.15, 0.24)	0.04	0.04	494	540	−0.28	0.04 (−0.09, 0.17)	0.07	0.10
Zimbabwe	SHINE (HIV+) (48)	50	35	−0.45	−0.15 (−0.63, 0.34)	0.01	0.01	90	107	−0.59	0.12 (−0.17, 0.42)	0.01	0.02
		**4777**	**3944**		**I^2 = 0.00, Tau^2 = 0.00**			**5039**	**4393**		**I^2 = 0.48, Tau^2 = 0.00**		
**Fixed**					**0.08 (0.04, 0.12)**						**0.08 (0.04, 0.11)**		
**Random**					**0.08 (0.04, 0.12)**						**0.07 (0.02, 0.11)**		

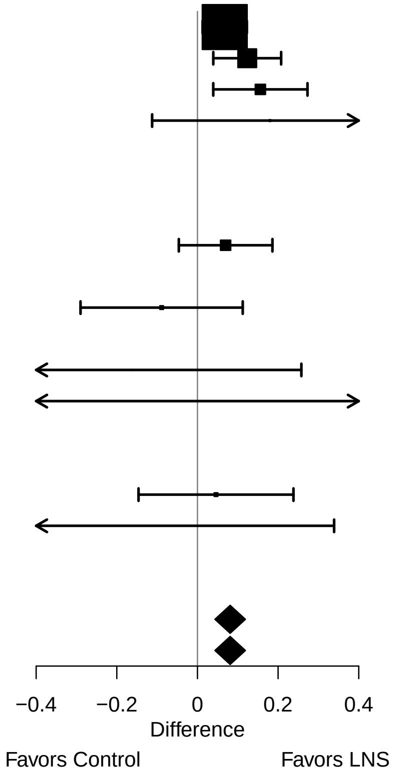

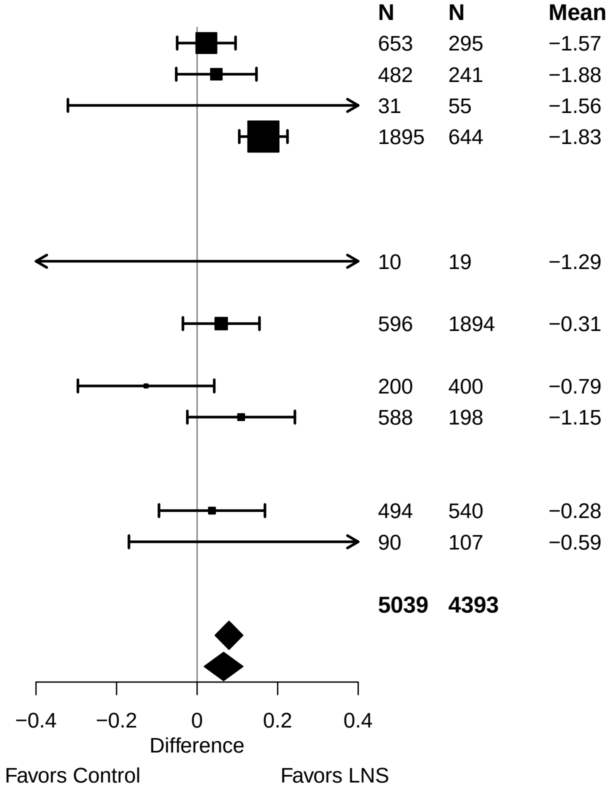

Supplemental figure 9O: Mean difference in HCZ

9O5: Stratified by Home environment

P−for−interaction = 0.416
Difference in MDs = 0.03 (−0.04, 0.09)

Country	Trial	At least median: LNS N	Control N	Control Mean	MD (95% CI)	Fixed W	Random W	Less than median: LNS N	Control N	Control Mean	MD (95% CI)	Fixed W	Random W
Bangladesh	JiVitA−4 (34)												
Bangladesh	RDNS (35)	1192	582	−1.85	0.10 (0.01, 0.20)	0.18	0.17	468	230	−1.90	0.09 (0.00, 0.19)	0.23	0.23
Bangladesh	WASH−B (36)	641	1665	−1.55	0.13 (0.04, 0.22)	0.21	0.17	494	1707	−1.68	0.07 (−0.02, 0.17)	0.22	0.22
Burkina Faso	iLiNS−Zinc (37)	471	199	−1.77	0.14 (0.04, 0.23)	0.17	0.16	270	174	−1.95	0.16 (0.06, 0.27)	0.19	0.19
Burkina Faso	PROMIS (38)												
Burkina Faso	PROMIS CS (38)												
Ghana	GHANA (39)												
Ghana	iLiNS−DYADG (40)	213	410	−1.10	0.03 (−0.12, 0.17)	0.08	0.12	119	247	−1.18	0.08 (−0.12, 0.28)	0.05	0.05
Haiti	HAITI (41)												
Kenya	WASH−B (42)	839	2778	−0.20	−0.02 (−0.10, 0.06)	0.27	0.18	574	2174	−0.35	0.09 (0.00, 0.19)	0.23	0.23
Madagascar	MAHAY (43)												
Malawi	iLiNS−DYADM (44)	137	289	−0.73	−0.16 (−0.35, 0.03)	0.04	0.09	78	149	−0.88	−0.07 (−0.36, 0.22)	0.02	0.02
Malawi	iLiNS−DOSE (45)	377	135	−1.11	0.12 (−0.04, 0.28)	0.06	0.11	272	85	−1.10	0.07 (−0.14, 0.28)	0.05	0.05
Mali	PROMIS (46)												
Mali	PROMIS CS (46)												
Zimbabwe	SHINE (HIV−) (47)												
Zimbabwe	SHINE (HIV+) (48)												
		**3870**	**6058**		**I² = 0.62, Tau² = 0.01**			**2275**	**4766**		**I² = 0.00, Tau² = 0.00**		
**Fixed**					**0.06 (0.03, 0.10)**						**0.10 (0.05, 0.14)**		
**Random**					**0.06 (−0.01, 0.13)**						**0.10 (0.05, 0.14)**		

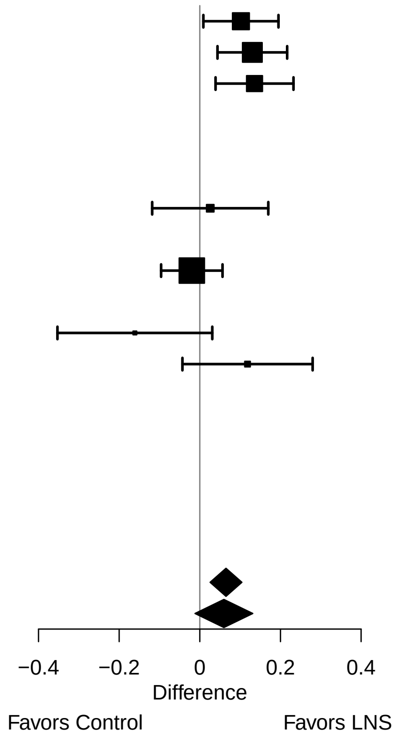

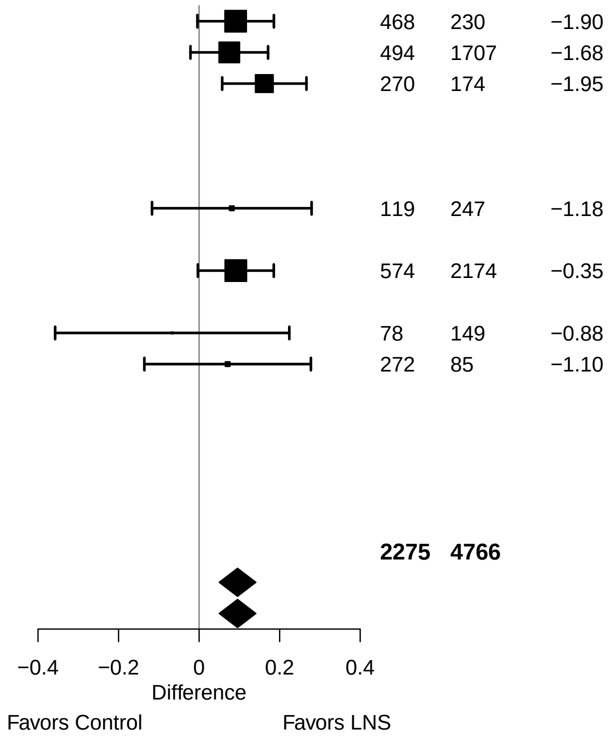

Supplemental figure 9O: Mean difference in HCZ

9O6: Stratified by Season at the time of assessment

P−for−interaction = 0.477
Difference in MDs = −0.02 (−0.06, 0.03)

Country	Trial	Dry LNS N	Dry Control N	Dry Control Mean	Dry MD (95% CI)	Dry Fixed W	Dry Random W	Rainy LNS N	Rainy Control N	Rainy Control Mean	Rainy MD (95% CI)	Rainy Fixed W	Rainy Random W
Bangladesh	JiVitA−4 (34)	1566	674	−1.44	0.06 (0.00, 0.12)	0.26	0.20	1319	593	−1.45	0.06 (−0.01, 0.12)	0.27	0.17
Bangladesh	RDNS (35)	739	359	−1.81	0.09 (−0.01, 0.19)	0.08	0.10	924	456	−1.91	0.11 (0.02, 0.20)	0.13	0.14
Bangladesh	WASH−B (36)	644	1936	−1.56	0.08 (−0.01, 0.17)	0.10	0.11	519	1527	−1.68	0.18 (0.09, 0.26)	0.17	0.15
Burkina Faso	iLiNS−Zinc (37)	1387	477	−1.86	0.17 (0.11, 0.24)	0.21	0.18	559	187	−1.80	0.14 (0.02, 0.25)	0.09	0.12
Burkina Faso	PROMIS (38)												
Burkina Faso	PROMIS CS (38)												
Ghana	GHANA (39)	57	55	−0.72	0.43 (0.06, 0.79)	0.01	0.01	41	41	−0.44	−0.11 (−0.47, 0.24)	0.01	0.02
Ghana	iLiNS−DYADG (40)	204	408	−1.20	0.12 (−0.03, 0.27)	0.04	0.05	143	284	−1.05	−0.01 (−0.18, 0.17)	0.04	0.07
Haiti	HAITI (41)												
Kenya	WASH−B (42)	349	1161	−0.34	0.10 (−0.02, 0.21)	0.06	0.08	1112	4015	−0.26	0.03 (−0.04, 0.10)	0.23	0.17
Madagascar	MAHAY (43)												
Malawi	iLiNS−DYADM (44)	156	316	−0.75	−0.05 (−0.24, 0.13)	0.02	0.03	64	128	−0.84	−0.30 (−0.59, −0.01)	0.01	0.03
Malawi	iLiNS−DOSE (45)	418	133	−1.18	0.16 (−0.01, 0.32)	0.03	0.04	274	105	−1.06	0.04 (−0.15, 0.23)	0.03	0.07
Mali	PROMIS (46)												
Mali	PROMIS CS (46)												
Zimbabwe	SHINE (HIV−) (47)	1738	1652	−0.30	0.09 (0.02, 0.16)	0.16	0.15	133	131	−0.03	0.05 (−0.19, 0.29)	0.02	0.05
Zimbabwe	SHINE (HIV+) (48)	319	305	−0.58	0.12 (−0.06, 0.30)	0.03	0.04	18	24	−0.28	−0.11 (−0.77, 0.56)	0.00	0.01
		**7577**	**7476**		**I² = 0.27, Tau² = 0.00**			**5106**	**7491**		**I² = 0.44, Tau² = 0.00**		
**Fixed**					**0.10 (0.07, 0.13)**						**0.08 (0.04, 0.11)**		
**Random**					**0.10 (0.07, 0.14)**						**0.06 (0.01, 0.12)**		

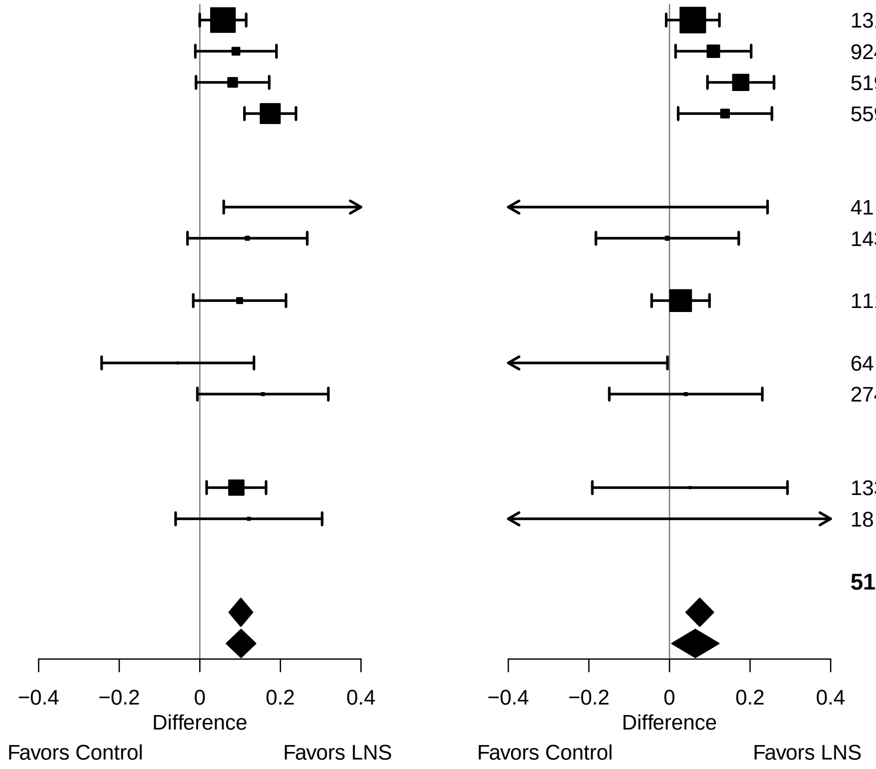

Supplemental figure 9P: Small head size prevalence ratio

9P1: Stratified by Household socio-economic status

P−for−interaction = 0.462
Ratio of PRs = 1.04 (0.94, 1.14)

		At least median						Less than median					
**Country**	**Trial**	**LNS N**	**Control N**	**Control Prevalence**	**PR (95% CI)**	**Fixed W**	**Random W**	**LNS N**	**Control N**	**Control Prevalence**	**PR (95% CI)**	**Fixed W**	**Random W**
Bangladesh	JiVitA−4 (34)	1456	640	20.2	1.00 (0.82, 1.23)	0.13	0.13	1428	627	28.1	1.05 (0.90, 1.23)	0.17	0.17
Bangladesh	RDNS (35)	834	407	39.1	0.89 (0.76, 1.04)	0.23	0.23	829	408	46.8	0.90 (0.80, 1.01)	0.32	0.30
Bangladesh	WASH−B (36)	583	1740	29.1	0.79 (0.67, 0.93)	0.22	0.22	580	1723	36.5	1.00 (0.88, 1.13)	0.27	0.25
Burkina Faso	iLiNS−Zinc (37)	1226	299	39.3	0.94 (0.81, 1.08)	0.28	0.28	713	364	42.2	0.82 (0.68, 0.99)	0.12	0.13
Burkina Faso	PROMIS (38)												
Burkina Faso	PROMIS CS (38)												
Ghana	GHANA (39)												
Ghana	iLiNS−DYADG (40)	157	355	14.1	0.72 (0.43, 1.23)	0.02	0.02	190	335	18.2	0.95 (0.65, 1.40)	0.03	0.03
Haiti	HAITI (41)												
Kenya	WASH−B (42)	827	2946	3.5	0.90 (0.57, 1.41)	0.03	0.03	632	2226	5.4	0.93 (0.61, 1.43)	0.02	0.03
Madagascar	MAHAY (43)												
Malawi	iLiNS−DYADM (44)	121	234	6.8	1.69 (0.85, 3.35)	0.01	0.01	99	209	14.8	0.48 (0.22, 1.04)	0.01	0.01
Malawi	iLiNS−DOSE (45)	302	102	19.4	0.82 (0.53, 1.26)	0.03	0.03	290	102	23.5	0.81 (0.55, 1.20)	0.03	0.03
Mali	PROMIS (46)												
Mali	PROMIS CS (46)												
Zimbabwe	SHINE (HIV−) (47)	905	799	4.6	0.81 (0.53, 1.23)	0.03	0.03	826	853	6.9	0.84 (0.59, 1.19)	0.03	0.04
Zimbabwe	SHINE (HIV+) (48)	171	156	10.9	0.80 (0.41, 1.58)	0.01	0.01	158	167	8.4	1.28 (0.62, 2.64)	0.01	0.01
		**6582**	**7678**		**I² = 0.00, Tau² = 0.00**			**5745**	**7014**		**I² = 0.09, Tau² = 0.00**		
**Fixed**					**0.89 (0.83, 0.96)**						**0.93 (0.88, 1.00)**		
**Random**					**0.89 (0.83, 0.96)**						**0.93 (0.87, 1.00)**		

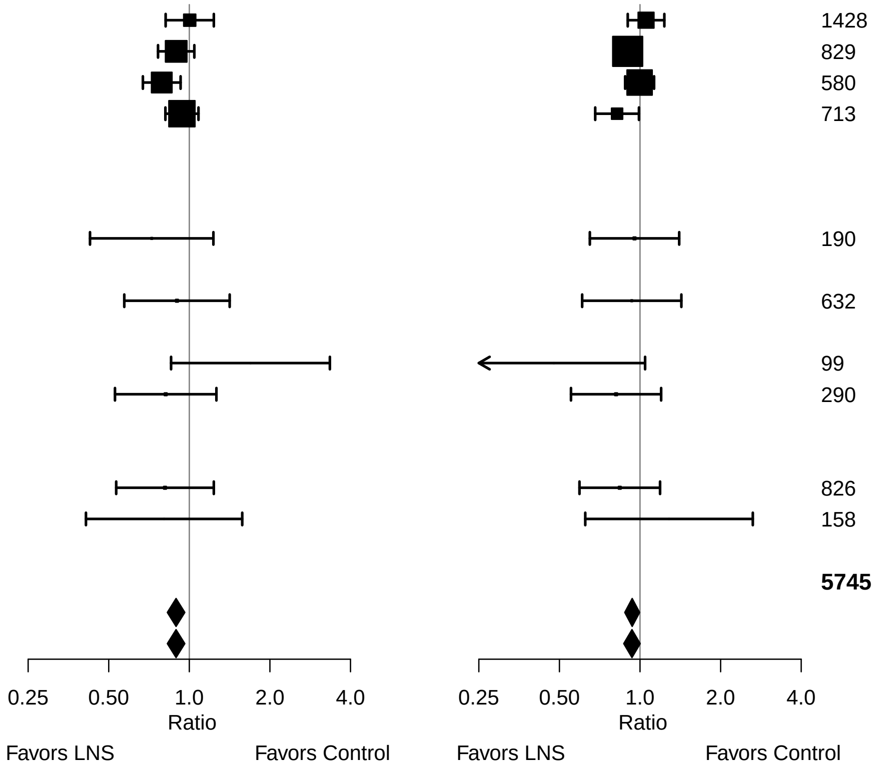

Supplemental figure 9P: Small head size prevalence ratio

9P2: Stratified by Household food insecurity

P−for−interaction = 0.542
Ratio of PRs = 1.03 (0.94, 1.13)

		Mild to secure						Moderate to severe					
**Country**	**Trial**	**LNS N**	**Control N**	**Control Prevalence**	**PR (95% CI)**	**Fixed W**	**Random W**	**LNS N**	**Control N**	**Control Prevalence**	**PR (95% CI)**	**Fixed W**	**Random W**
Bangladesh	JiVitA−4 (34)	2045	882	23.6	0.88 (0.75, 1.03)	0.13	0.13	839	385	25.2	1.33 (1.07, 1.64)	0.15	0.16
Bangladesh	RDNS (35)	1058	493	40.8	0.91 (0.82, 1.01)	0.30	0.30	605	322	46.3	0.88 (0.76, 1.02)	0.33	0.21
Bangladesh	WASH−B (36)	922	2682	31.5	0.90 (0.80, 1.01)	0.24	0.24	241	781	37.4	0.94 (0.77, 1.15)	0.16	0.17
Burkina Faso	iLiNS−Zinc (37)	1008	320	38.3	0.92 (0.82, 1.03)	0.24	0.24	931	343	43.3	0.85 (0.71, 1.02)	0.20	0.18
Burkina Faso	PROMIS (38)												
Burkina Faso	PROMIS CS (38)												
Ghana	GHANA (39)												
Ghana	iLiNS−DYADG (40)	244	470	14.7	0.81 (0.54, 1.21)	0.02	0.02	102	219	19.2	1.02 (0.63, 1.65)	0.03	0.06
Haiti	HAITI (41)												
Kenya	WASH−B (42)	1303	4632	4.1	0.96 (0.70, 1.33)	0.03	0.03	156	527	6.6	0.68 (0.29, 1.56)	0.01	0.02
Madagascar	MAHAY (43)												
Malawi	iLiNS−DYADM (44)	70	122	9.0	0.79 (0.29, 2.19)	0.00	0.00	148	316	11.4	0.95 (0.54, 1.65)	0.02	0.04
Malawi	iLiNS−DOSE (45)	149	55	16.4	1.11 (0.57, 2.16)	0.01	0.01	431	143	22.9	0.74 (0.54, 1.03)	0.06	0.10
Mali	PROMIS (46)												
Mali	PROMIS CS (46)												
Zimbabwe	SHINE (HIV−) (47)	1385	1297	5.7	0.71 (0.51, 0.98)	0.03	0.03	295	323	6.2	1.37 (0.80, 2.35)	0.02	0.04
Zimbabwe	SHINE (HIV+) (48)	236	239	9.6	1.10 (0.63, 1.91)	0.01	0.01	86	76	10.5	0.77 (0.31, 1.93)	0.01	0.02
		**8420**	**11192**		**I² = 0.00, Tau² = 0.00**			**3834**	**3435**		**I² = 0.46, Tau² = 0.01**		
**Fixed**					**0.90 (0.85, 0.95)**						**0.94 (0.87, 1.02)**		
**Random**					**0.90 (0.85, 0.95)**						**0.95 (0.84, 1.08)**		

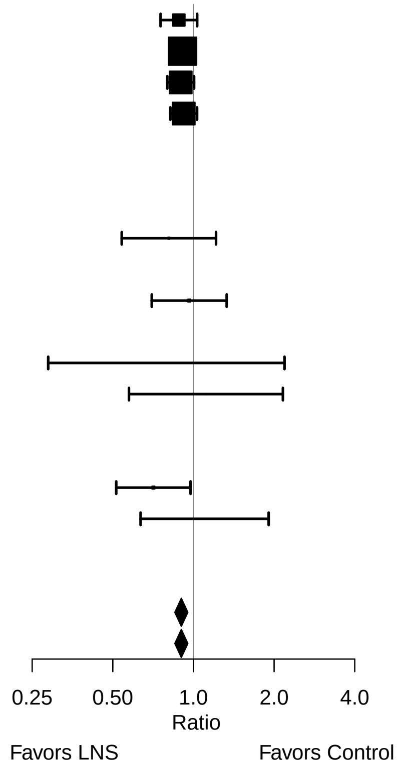

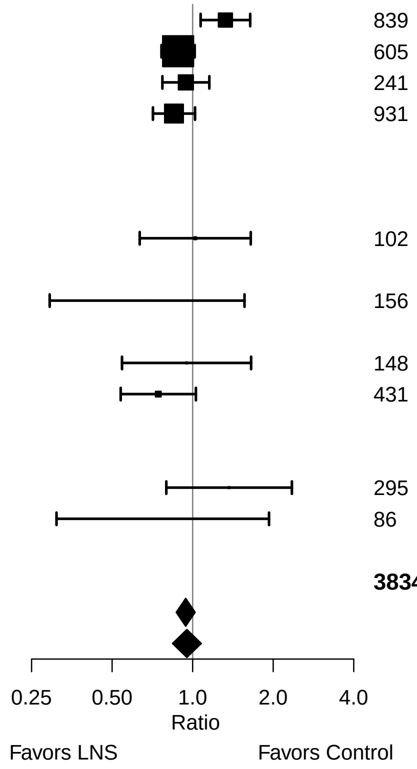

Supplemental figure 9P: Small head size prevalence ratio

9P3: Stratified by Household source water quality

P−for−interaction = 0.505
Ratio of PRs = 0.93 (0.74, 1.16)

Country	Trial	Improved LNS N	Improved Control N	Improved Control Prevalence	Improved PR (95% CI)	Improved Fixed W	Improved Random W	Unimproved LNS N	Unimproved Control N	Unimproved Control Prevalence	Unimproved PR (95% CI)	Unimproved Fixed W	Unimproved Random W
Bangladesh	JiVitA−4 (34)												
Bangladesh	RDNS (35)												
Bangladesh	WASH−B (36)	500	1004	34.3	0.84 (0.70, 1.01)	0.50	0.50	70	116	28.4	1.05 (0.69, 1.61)	0.08	0.08
Burkina Faso	iLiNS−Zinc (37)	552	141	36.9	1.05 (0.78, 1.42)	0.19	0.19	1386	519	41.8	0.85 (0.75, 0.96)	0.86	0.86
Burkina Faso	PROMIS (38)												
Burkina Faso	PROMIS CS (38)												
Ghana	GHANA (39)												
Ghana	iLiNS−DYADG (40)												
Haiti	HAITI (41)												
Kenya	WASH−B (42)	448	1560	4.2	0.69 (0.36, 1.30)	0.04	0.04	248	699	5.2	0.78 (0.40, 1.55)	0.03	0.03
Madagascar	MAHAY (43)												
Malawi	iLiNS−DYADM (44)												
Malawi	iLiNS−DOSE (45)	553	192	20.8	0.84 (0.62, 1.14)	0.19	0.19	51	15	31.2	0.48 (0.20, 1.13)	0.02	0.02
Mali	PROMIS (46)												
Mali	PROMIS CS (46)												
Zimbabwe	SHINE (HIV−) (47)	518	485	5.8	0.90 (0.58, 1.42)	0.08	0.08	272	299	4.0	1.28 (0.59, 2.77)	0.02	0.02
Zimbabwe	SHINE (HIV+) (48)												
		**2571**	**3382**		**I² = 0.00, Tau² = 0.00**			**2027**	**1648**		**I² = 0.00, Tau² = 0.00**		
**Fixed**					**0.88 (0.77, 1.00)**						**0.86 (0.77, 0.96)**		
**Random**					**0.88 (0.77, 1.00)**						**0.86 (0.77, 0.96)**		

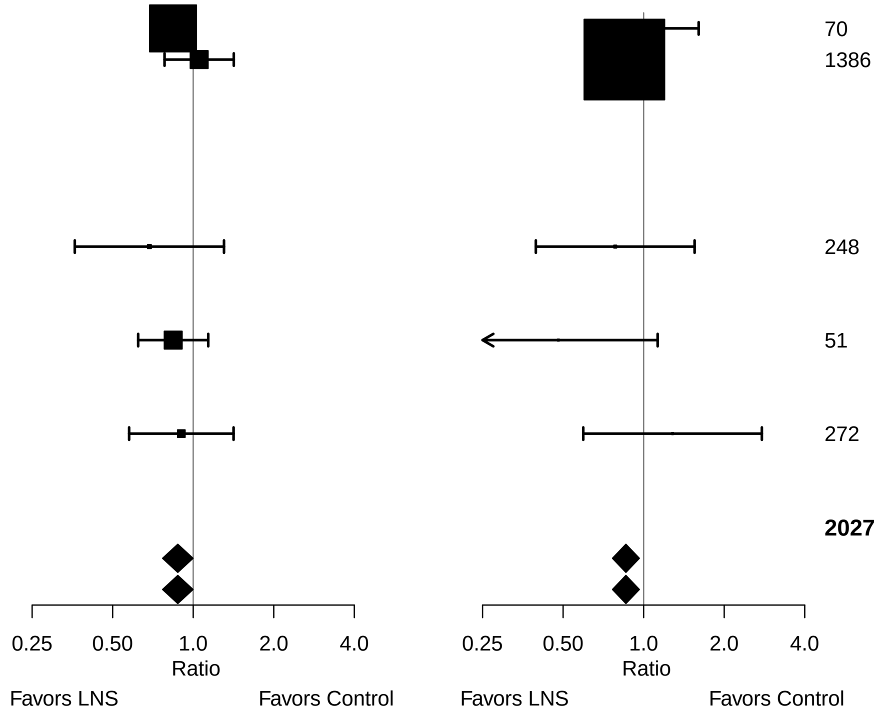

Supplemental figure 9P: Small head size prevalence ratio

9P4: Stratified by Household sanitation

P−for−interaction = 0.109
Ratio of PRs = 1.13 (0.97, 1.31)

Country	Trial	LNS N	Control N	Control Prevalence	Improved PR (95% CI)	Fixed W	Random W	LNS N	Control N	Control Prevalence	Unimproved PR (95% CI)	Fixed W	Random W
Bangladesh	JiVitA−4 (34)	2231	972	22.9	1.01 (0.86, 1.19)	0.23	0.28	653	295	27.9	1.09 (0.88, 1.35)	0.18	0.18
Bangladesh	RDNS (35)	1179	573	42.8	0.86 (0.77, 0.95)	0.56	0.45	482	241	43.6	0.98 (0.84, 1.15)	0.32	0.32
Bangladesh	WASH−B (36)	510	1017	33.3	0.87 (0.72, 1.05)	0.18	0.24	31	55	27.3	1.06 (0.54, 2.10)	0.02	0.02
Burkina Faso	iLiNS−Zinc (37)	43	16	50.0	0.66 (0.34, 1.28)	0.01	0.03	1895	644	40.6	0.89 (0.78, 1.02)	0.45	0.45
Burkina Faso	PROMIS (38)												
Burkina Faso	PROMIS CS (38)												
Ghana	GHANA (39)												
Ghana	iLiNS−DYADG (40)												
Haiti	HAITI (41)												
Kenya	WASH−B (42)												
Madagascar	MAHAY (43)												
Malawi	iLiNS−DYADM (44)												
Malawi	iLiNS−DOSE (45)												
Mali	PROMIS (46)												
Mali	PROMIS CS (46)												
Zimbabwe	SHINE (HIV−) (47)	293	243	3.3	1.56 (0.65, 3.69)	0.01	0.02	494	540	5.9	0.85 (0.53, 1.37)	0.04	0.04
Zimbabwe	SHINE (HIV+) (48)												
		**4256**	**2821**		**I² = 0.23, Tau² = 0.00**			**3555**	**1775**		**I² = 0.00, Tau² = 0.00**		
**Fixed**					**0.90 (0.83, 0.97)**						**0.96 (0.87, 1.05)**		
**Random**					**0.90 (0.81, 1.01)**						**0.96 (0.87, 1.05)**		

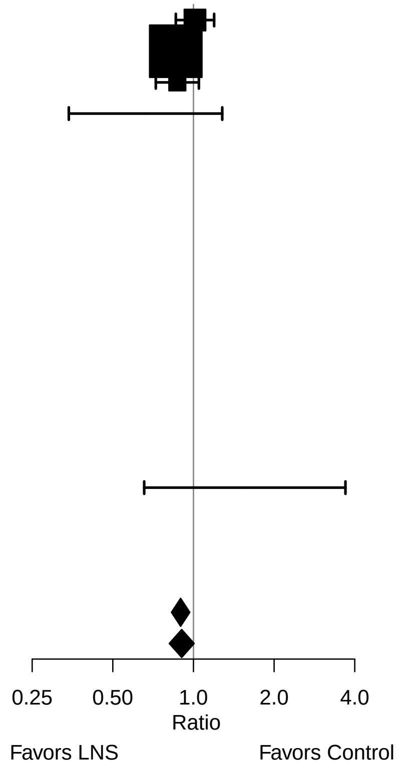

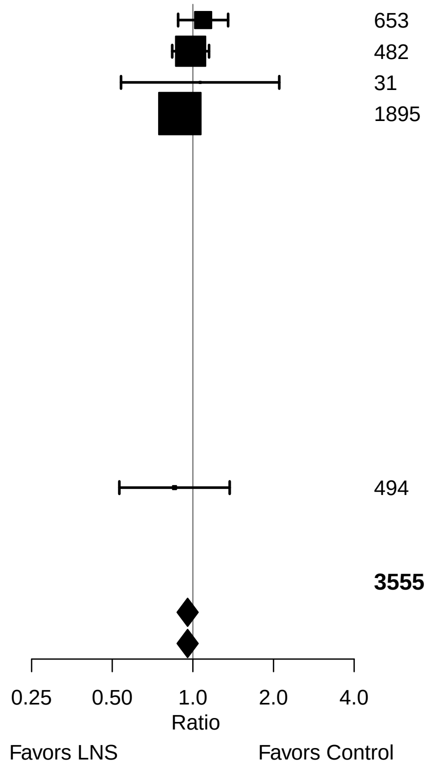

Supplemental figure 9P: Small head size prevalence ratio

9P5: Stratified by Home environment

P−for−interaction = 0.826
Ratio of PRs = 0.99 (0.89, 1.10)

		At least median						Less than median					
**Country**	**Trial**	**LNS N**	**Control N**	**Control Prevalence**	**PR (95% CI)**	**Fixed W**	**Random W**	**LNS N**	**Control N**	**Control Prevalence**	**PR (95% CI)**	**Fixed W**	**Random W**
Bangladesh	JiVitA−4 (34)												
Bangladesh	RDNS (35)	1192	582	41.4	0.91 (0.82, 1.01)	0.53	0.53	468	230	46.5	0.87 (0.77, 0.98)	0.36	0.36
Bangladesh	WASH−B (36)	641	1665	30.9	0.86 (0.73, 1.01)	0.24	0.24	494	1707	34.9	0.97 (0.85, 1.11)	0.33	0.33
Burkina Faso	iLiNS−Zinc (37)	471	199	36.2	0.93 (0.73, 1.18)	0.10	0.10	270	174	44.0	0.90 (0.76, 1.05)	0.22	0.22
Burkina Faso	PROMIS (38)												
Burkina Faso	PROMIS CS (38)												
Ghana	GHANA (39)												
Ghana	iLiNS−DYADG (40)	213	410	14.1	0.93 (0.61, 1.41)	0.03	0.03	119	247	18.2	0.92 (0.57, 1.49)	0.02	0.02
Haiti	HAITI (41)												
Kenya	WASH−B (42)	839	2778	3.1	1.22 (0.82, 1.81)	0.04	0.04	574	2174	5.1	0.78 (0.49, 1.25)	0.03	0.03
Madagascar	MAHAY (43)												
Malawi	iLiNS−DYADM (44)	137	289	9.0	0.89 (0.45, 1.75)	0.01	0.01	78	149	14.8	0.87 (0.43, 1.74)	0.01	0.01
Malawi	iLiNS−DOSE (45)	377	135	19.3	0.80 (0.54, 1.17)	0.04	0.04	272	85	23.3	0.75 (0.49, 1.13)	0.03	0.03
Mali	PROMIS (46)												
Mali	PROMIS CS (46)												
Zimbabwe	SHINE (HIV−) (47)												
Zimbabwe	SHINE (HIV+) (48)												
		**3870**	**6058**		**I² = 0.00, Tau² = 0.00**			**2275**	**4766**		**I² = 0.00, Tau² = 0.00**		
**Fixed**					**0.90 (0.84, 0.98)**						**0.90 (0.84, 0.97)**		
**Random**					**0.90 (0.84, 0.98)**						**0.90 (0.84, 0.97)**		

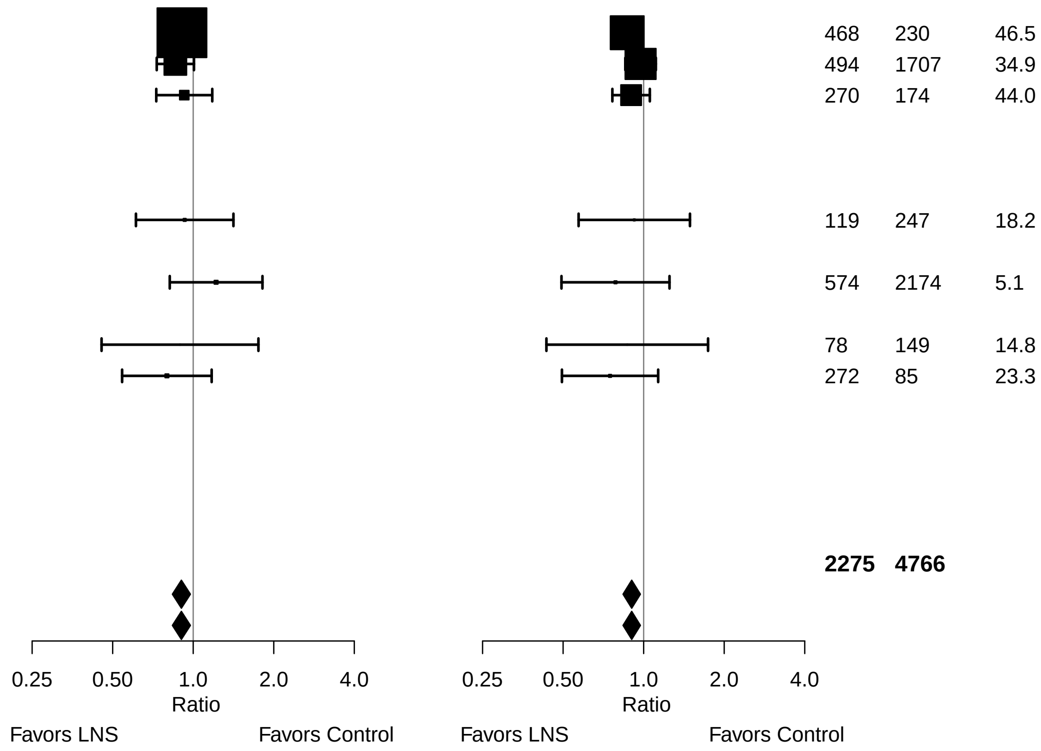

Supplemental figure 9P: Small head size prevalence ratio

9P6: Stratified by Season at the time of assessment

P−for−interaction = 0.434

Ratio of PRs = 1.04 (0.94, 1.15)

Country	Trial	LNS N	Control N	Control Prevalence	Dry PR (95% CI)	Fixed W	Random W	LNS N	Control N	Control Prevalence	Rainy PR (95% CI)	Fixed W	Random W
Bangladesh	JiVitA−4 (34)	1566	674	24.6	1.00 (0.85, 1.17)	0.16	0.16	1319	593	23.5	1.08 (0.88, 1.32)	0.13	0.13
Bangladesh	RDNS (35)	739	359	39.6	0.90 (0.80, 1.02)	0.27	0.27	924	456	45.6	0.89 (0.79, 1.00)	0.42	0.42
Bangladesh	WASH−B (36)	644	1936	31.8	0.92 (0.80, 1.06)	0.23	0.23	519	1527	34.1	0.88 (0.76, 1.02)	0.25	0.25
Burkina Faso	iLiNS−Zinc (37)	1387	477	40.9	0.87 (0.77, 0.99)	0.26	0.26	559	187	41.3	0.93 (0.72, 1.19)	0.09	0.09
Burkina Faso	PROMIS (38)												
Burkina Faso	PROMIS CS (38)												
Ghana	GHANA (39)												
Ghana	iLiNS−DYADG (40)	204	408	18.4	0.72 (0.48, 1.08)	0.03	0.03	143	284	12.7	1.21 (0.74, 1.98)	0.02	0.02
Haiti	HAITI (41)												
Kenya	WASH−B (42)	349	1161	4.9	0.99 (0.55, 1.80)	0.01	0.01	1112	4015	4.2	0.89 (0.62, 1.26)	0.04	0.04
Madagascar	MAHAY (43)												
Malawi	iLiNS−DYADM (44)	156	316	10.8	0.66 (0.34, 1.26)	0.01	0.01	64	128	10.9	1.43 (0.67, 3.04)	0.01	0.01
Malawi	iLiNS−DOSE (45)	418	133	25.4	0.67 (0.47, 0.94)	0.04	0.04	274	105	17.1	0.98 (0.64, 1.51)	0.03	0.03
Mali	PROMIS (46)												
Mali	PROMIS CS (46)												
Zimbabwe	SHINE (HIV−) (47)												
Zimbabwe	SHINE (HIV+) (48)												
		**5463**	**5464**		**I² = 0.00, Tau² = 0.00**			**4914**	**7295**		**I² = 0.00, Tau² = 0.00**		
**Fixed**					**0.90 (0.84, 0.96)**						**0.93 (0.86, 1.00)**		
**Random**					**0.90 (0.84, 0.96)**						**0.93 (0.86, 1.00)**		

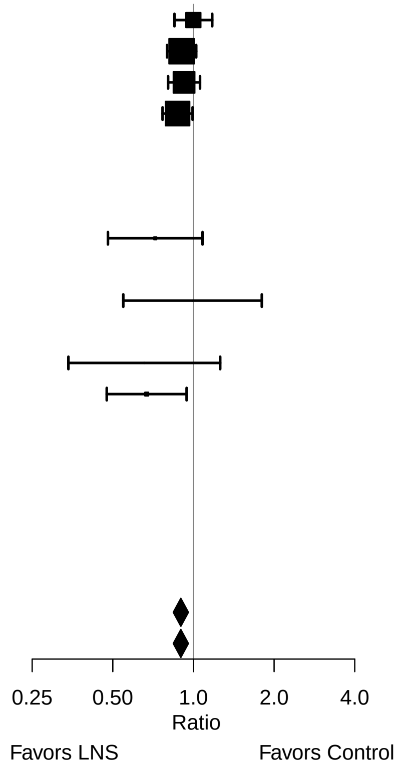

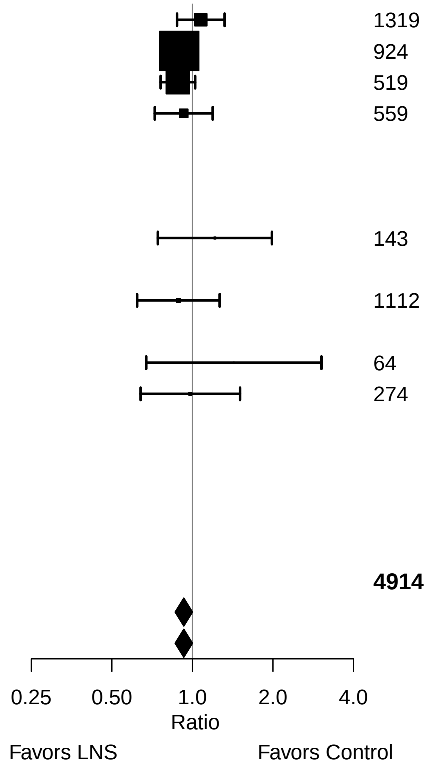

Supplemental figure 9Q: Small head size prevalence difference

9Q1: Stratified by Household socio-economic status

P−for−interaction = 0.965
Difference in PDs = 0.00 (−0.01, 0.02)

Country	Trial	At least median: LNS N	Control N	Control Prevalence	PD (95% CI)	Fixed W	Random W	Less than median: LNS N	Control N	Control Prevalence	PD (95% CI)	Fixed W	Random W
Bangladesh	JiVitA−4 (34)	1456	640	20.2	−0.01 (−0.04, 0.03)	0.07	0.11	1428	627	28.1	0.00 (−0.03, 0.04)	0.11	0.13
Bangladesh	RDNS (35)	834	407	39.1	−0.04 (−0.10, 0.02)	0.03	0.05	829	408	46.8	−0.05 (−0.10, 0.00)	0.06	0.07
Bangladesh	WASH−B (36)	583	1740	29.1	−0.06 (−0.10, −0.02)	0.06	0.10	580	1723	36.5	0.00 (−0.05, 0.04)	0.07	0.09
Burkina Faso	iLiNS−Zinc (37)	1226	299	39.3	−0.03 (−0.06, 0.01)	0.07	0.11	713	364	42.2	−0.07 (−0.14, 0.01)	0.03	0.03
Burkina Faso	PROMIS (38)												
Burkina Faso	PROMIS CS (38)												
Ghana	GHANA (39)												
Ghana	iLiNS−DYADG (40)	157	355	14.1	−0.04 (−0.10, 0.02)	0.02	0.05	190	335	18.2	−0.01 (−0.08, 0.06)	0.03	0.04
Haiti	HAITI (41)												
Kenya	WASH−B (42)	827	2946	3.5	0.00 (−0.02, 0.01)	0.42	0.24	632	2226	5.4	0.00 (−0.03, 0.02)	0.31	0.27
Madagascar	MAHAY (43)												
Malawi	iLiNS−DYADM (44)	121	234	6.8	0.05 (−0.01, 0.11)	0.02	0.05	99	209	14.8	−0.08 (−0.16, 0.00)	0.02	0.03
Malawi	iLiNS−DOSE (45)	302	102	19.4	−0.02 (−0.10, 0.06)	0.01	0.03	290	102	23.5	−0.05 (−0.13, 0.03)	0.02	0.03
Mali	PROMIS (46)												
Mali	PROMIS CS (46)												
Zimbabwe	SHINE (HIV−) (47)	905	799	4.6	−0.01 (−0.03, 0.01)	0.28	0.22	826	853	6.9	−0.01 (−0.03, 0.01)	0.31	0.27
Zimbabwe	SHINE (HIV+) (48)	171	156	10.9	−0.02 (−0.09, 0.04)	0.02	0.05	158	167	8.4	0.02 (−0.04, 0.09)	0.03	0.04
		**6582**	**7678**		**I² = 0.34, Tau² = 0.00**			**5745**	**7014**		**I² = 0.09, Tau² = 0.00**		
**Fixed**					**−0.01 (−0.02, 0.00)**						**−0.01 (−0.02, 0.00)**		
**Random**					**−0.02 (−0.03, 0.00)**						**−0.01 (−0.03, 0.00)**		

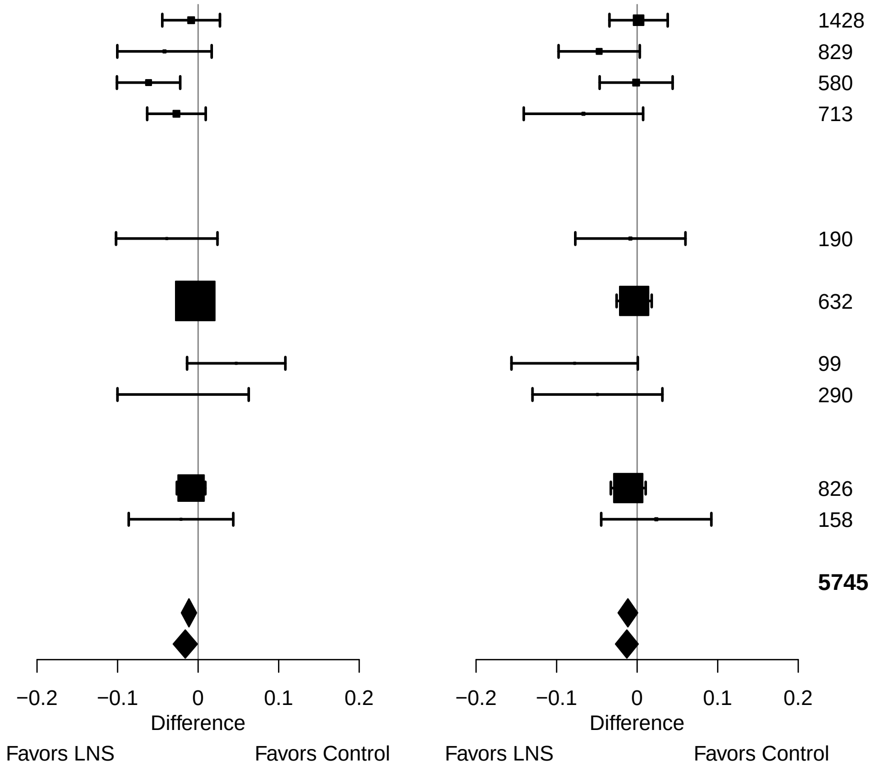

Supplemental figure 9Q: Small head size prevalence difference

9Q2: Stratified by Household food insecurity

P−for−interaction = 0.396
Difference in PDs = 0.01 (−0.01, 0.03)

		Mild to secure						Moderate to severe					
Country	Trial	LNS N	Control N	Control Prevalence	PD (95% CI)	Fixed W	Random W	LNS N	Control N	Control Prevalence	PD (95% CI)	Fixed W	Random W
Bangladesh	JiVitA−4 (34)	2045	882	23.6	−0.02 (−0.06, 0.01)	0.06	0.06	839	385	25.2	0.04 (0.00, 0.09)	0.17	0.15
Bangladesh	RDNS (35)	1058	493	40.8	−0.04 (−0.08, 0.00)	0.04	0.04	605	322	46.3	−0.05 (−0.12, 0.01)	0.08	0.09
Bangladesh	WASH−B (36)	922	2682	31.5	−0.03 (−0.07, 0.00)	0.06	0.06	241	781	37.4	−0.02 (−0.09, 0.05)	0.06	0.08
Burkina Faso	iLiNS−Zinc (37)	1008	320	38.3	−0.03 (−0.06, 0.01)	0.05	0.05	931	343	43.3	−0.06 (−0.13, 0.01)	0.06	0.08
Burkina Faso	PROMIS (38)												
Burkina Faso	PROMIS CS (38)												
Ghana	GHANA (39)												
Ghana	iLiNS−DYADG (40)	244	470	14.7	−0.03 (−0.08, 0.03)	0.02	0.02	102	219	19.2	0.00 (−0.09, 0.10)	0.04	0.05
Haiti	HAITI (41)												
Kenya	WASH−B (42)	1303	4632	4.1	0.00 (−0.01, 0.01)	0.43	0.43	156	527	6.6	−0.02 (−0.06, 0.02)	0.19	0.16
Madagascar	MAHAY (43)												
Malawi	iLiNS−DYADM (44)	70	122	9.0	−0.02 (−0.10, 0.06)	0.01	0.01	148	316	11.4	−0.01 (−0.07, 0.06)	0.08	0.10
Malawi	iLiNS−DOSE (45)	149	55	16.4	0.03 (−0.08, 0.14)	0.01	0.01	431	143	22.9	−0.05 (−0.12, 0.01)	0.07	0.08
Mali	PROMIS (46)												
Mali	PROMIS CS (46)												
Zimbabwe	SHINE (HIV−) (47)	1385	1297	5.7	−0.02 (−0.03, 0.00)	0.29	0.29	295	323	6.2	0.02 (−0.02, 0.06)	0.20	0.16
Zimbabwe	SHINE (HIV+) (48)	236	239	9.6	0.01 (−0.05, 0.07)	0.02	0.02	86	76	10.5	−0.02 (−0.11, 0.06)	0.04	0.06
		**8420**	**11192**		**I² = 0.00, Tau² = 0.00**			**3834**	**3435**		**I² = 0.37, Tau² = 0.00**		
**Fixed**					**−0.01 (−0.02, 0.00)**						**−0.01 (−0.02, 0.01)**		
**Random**					**−0.01 (−0.02, 0.00)**						**−0.01 (−0.03, 0.01)**		

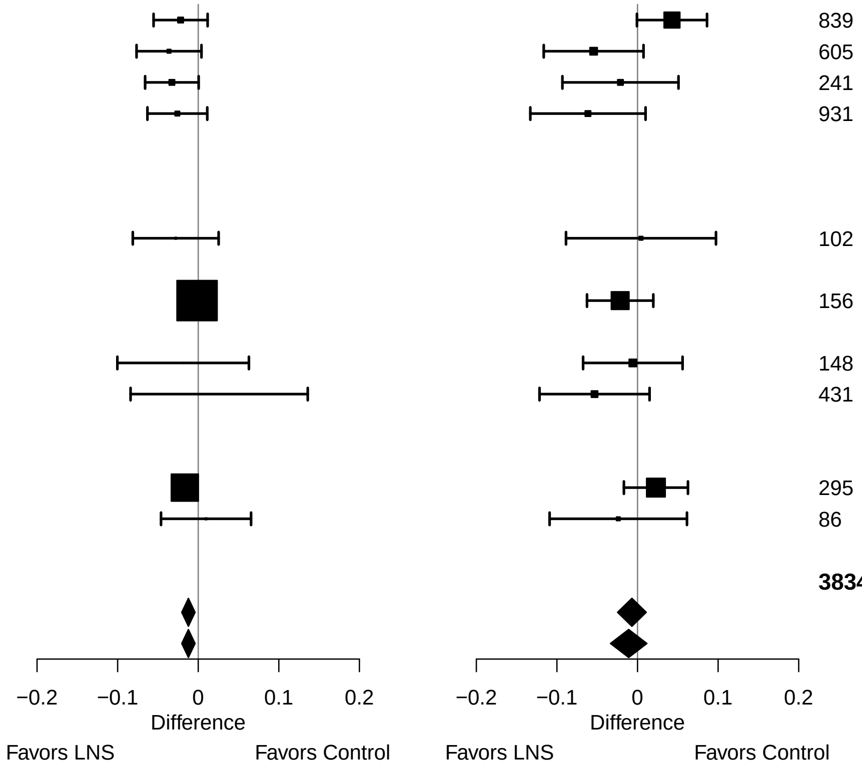

Supplemental figure 9Q: Small head size prevalence difference

9Q3: Stratified by Household source water quality

P−for−interaction = 0.976
Difference in PDs = 0.00 (−0.02, 0.03)

Country	Trial	LNS N	Control N	Control Prevalence	Improved PD (95% CI)	Fixed W	Random W	LNS N	Control N	Control Prevalence	Unimproved PD (95% CI)	Fixed W	Random W
Bangladesh	JiVitA−4 (34)												
Bangladesh	RDNS (35)												
Bangladesh	WASH−B (36)	500	1004	34.3	−0.05 (−0.11, 0.00)	0.07	0.07	70	116	28.4	0.02 (−0.11, 0.14)	0.03	0.09
Burkina Faso	iLiNS−Zinc (37)	552	141	36.9	0.00 (−0.09, 0.09)	0.03	0.03	1386	519	41.8	−0.06 (−0.11, 0.00)	0.14	0.24
Burkina Faso	PROMIS (38)												
Burkina Faso	PROMIS CS (38)												
Ghana	GHANA (39)												
Ghana	iLiNS−DYADG (40)												
Haiti	HAITI (41)												
Kenya	WASH−B (42)	448	1560	4.2	−0.01 (−0.03, 0.01)	0.52	0.52	248	699	5.2	−0.01 (−0.04, 0.02)	0.50	0.33
Madagascar	MAHAY (43)												
Malawi	iLiNS−DYADM (44)												
Malawi	iLiNS−DOSE (45)	553	192	20.8	−0.02 (−0.08, 0.04)	0.06	0.06	51	15	31.2	−0.19 (−0.39, 0.01)	0.01	0.04
Mali	PROMIS (46)												
Mali	PROMIS CS (46)												
Zimbabwe	SHINE (HIV−) (47)	518	485	5.8	−0.01 (−0.03, 0.02)	0.33	0.33	272	299	4.0	0.01 (−0.02, 0.05)	0.33	0.30
Zimbabwe	SHINE (HIV+) (48)												
		**2571**	**3382**		**I^2 = 0.00, Tau^2 = 0.00**			**2027**	**1648**		**I^2 = 0.45, Tau^2 = 0.00**		
**Fixed**					**−0.01 (−0.03, 0.00)**						**−0.01 (−0.03, 0.01)**		
**Random**					**−0.01 (−0.03, 0.00)**						**−0.02 (−0.06, 0.02)**		

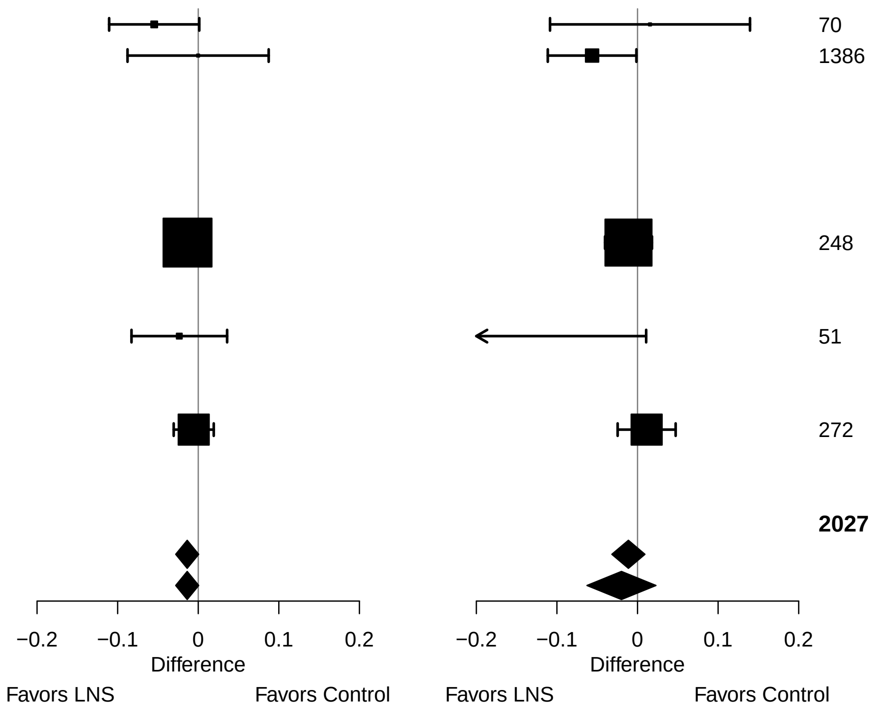

Supplemental figure 9Q: Small head size prevalence difference

9Q4: Stratified by Household sanitation

P−for−interaction = 0.794
Difference in PDs = 0.00 (−0.03, 0.04)

		Improved						Unimproved					
Country	Trial	LNS N	Control N	Control Prevalence	PD (95% CI)	Fixed W	Random W	LNS N	Control N	Control Prevalence	PD (95% CI)	Fixed W	Random W
Bangladesh	JiVitA−4 (34)	2231	972	22.9	−0.01 (−0.04, 0.02)	0.37	0.29	653	295	27.9	0.02 (−0.03, 0.07)	0.16	0.16
Bangladesh	RDNS (35)	1179	573	42.8	−0.06 (−0.10, −0.02)	0.20	0.23	482	241	43.6	−0.01 (−0.08, 0.06)	0.08	0.08
Bangladesh	WASH−B (36)	510	1017	33.3	−0.04 (−0.10, 0.01)	0.11	0.18	31	55	27.3	0.02 (−0.18, 0.21)	0.01	0.01
Burkina Faso	iLiNS−Zinc (37)	43	16	50.0	−0.11 (−0.33, 0.11)	0.01	0.02	1895	644	40.6	−0.04 (−0.09, 0.01)	0.14	0.14
Burkina Faso	PROMIS (38)												
Burkina Faso	PROMIS CS (38)												
Ghana	GHANA (39)												
Ghana	iLiNS−DYADG (40)												
Haiti	HAITI (41)												
Kenya	WASH−B (42)												
Madagascar	MAHAY (43)												
Malawi	iLiNS−DYADM (44)												
Malawi	iLiNS−DOSE (45)												
Mali	PROMIS (46)												
Mali	PROMIS CS (46)												
Zimbabwe	SHINE (HIV−) (47)	293	243	3.3	0.02 (−0.02, 0.05)	0.31	0.28	494	540	5.9	−0.01 (−0.03, 0.02)	0.60	0.60
Zimbabwe	SHINE (HIV+) (48)												
		**4256**	**2821**		**I² = 0.60, Tau² = 0.00**			**3555**	**1775**		**I² = 0.00, Tau² = 0.00**		
**Fixed**					**−0.02 (−0.03, 0.00)**						**−0.01 (−0.03, 0.01)**		
**Random**					**−0.02 (−0.05, 0.01)**						**−0.01 (−0.03, 0.01)**		

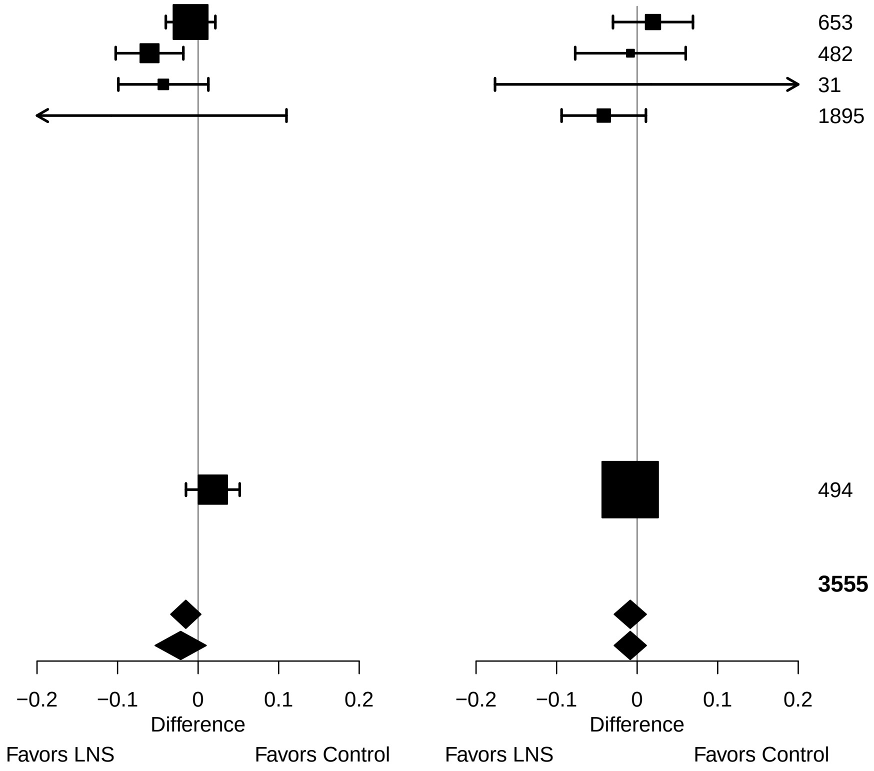

Supplemental figure 9Q: Small head size prevalence difference

9Q5: Stratified by Home environment

P−for−interaction = 0.291
Difference in PDs = −0.01 (−0.03, 0.01)

Country	Trial	LNS N	Control N	Control Prevalence	At least median PD (95% CI)	Fixed W	Random W	LNS N	Control N	Control Prevalence	Less than median PD (95% CI)	Fixed W	Random W
Bangladesh	JiVitA−4 (34)												
Bangladesh	RDNS (35)	1192	582	41.4	−0.04 (−0.08, 0.00)	0.08	0.12	468	230	46.5	−0.06 (−0.11, −0.01)	0.09	0.09
Bangladesh	WASH−B (36)	641	1665	30.9	−0.04 (−0.09, 0.00)	0.08	0.12	494	1707	34.9	−0.01 (−0.05, 0.04)	0.12	0.12
Burkina Faso	iLiNS−Zinc (37)	471	199	36.2	−0.03 (−0.10, 0.05)	0.03	0.04	270	174	44.0	−0.01 (−0.08, 0.06)	0.05	0.05
Burkina Faso	PROMIS (38)												
Burkina Faso	PROMIS CS (38)												
Ghana	GHANA (39)												
Ghana	iLiNS−DYADG (40)	213	410	14.1	−0.01 (−0.07, 0.05)	0.05	0.07	119	247	18.2	−0.01 (−0.10, 0.07)	0.03	0.03
Haiti	HAITI (41)												
Kenya	WASH−B (42)	839	2778	3.1	0.01 (−0.01, 0.02)	0.69	0.52	574	2174	5.1	−0.01 (−0.03, 0.01)	0.65	0.65
Madagascar	MAHAY (43)												
Malawi	iLiNS−DYADM (44)	137	289	9.0	−0.01 (−0.07, 0.05)	0.05	0.07	78	149	14.8	−0.02 (−0.12, 0.08)	0.03	0.03
Malawi	iLiNS−DOSE (45)	377	135	19.3	−0.03 (−0.10, 0.04)	0.03	0.05	272	85	23.3	−0.06 (−0.15, 0.03)	0.03	0.03
Mali	PROMIS (46)												
Mali	PROMIS CS (46)												
Zimbabwe	SHINE (HIV−) (47)												
Zimbabwe	SHINE (HIV+) (48)												
		**3870**	**6058**		**I² = 0.33, Tau² = 0.00**			**2275**	**4766**		**I² = 0.00, Tau² = 0.00**		
**Fixed**					**0.00 (−0.02, 0.01)**						**−0.02 (−0.03, 0.00)**		
**Random**					**−0.01 (−0.03, 0.01)**						**−0.02 (−0.03, 0.00)**		

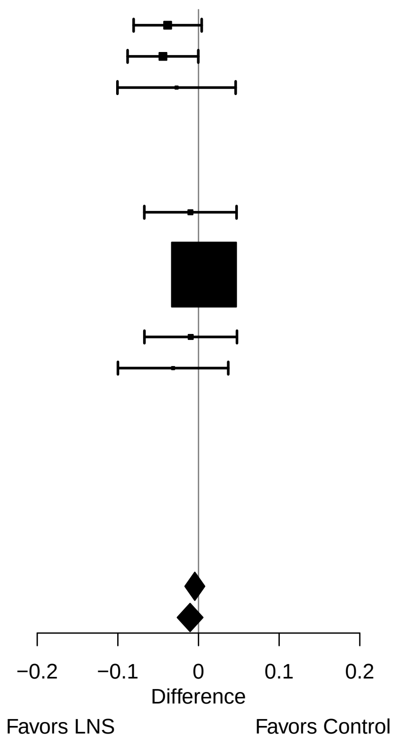

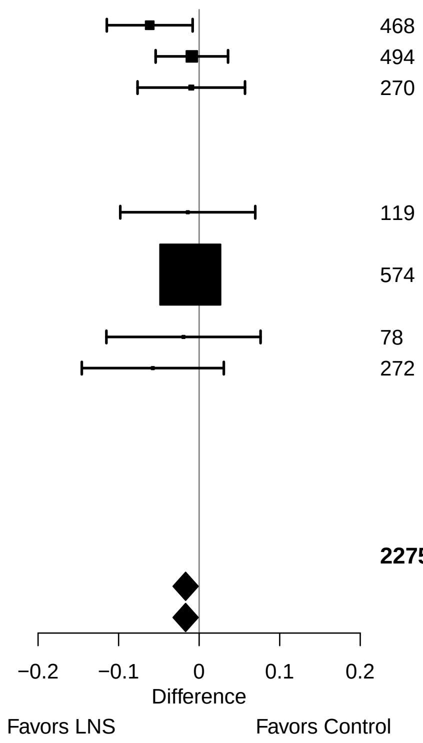

Supplemental figure 9Q: Small head size prevalence difference

9Q6: Stratified by Season at the time of assessment

P−for−interaction = 0.258
Difference in PDs = 0.01 (−0.01, 0.03)

Country	Trial	Dry: LNS N	Dry: Control N	Dry: Control Prevalence	Dry: PD (95% CI)	Dry: Fixed W	Dry: Random W	Rainy: LNS N	Rainy: Control N	Rainy: Control Prevalence	Rainy: PD (95% CI)	Rainy: Fixed W	Rainy: Random W
Bangladesh	JiVitA−4 (34)	1566	674	24.6	−0.01 (−0.05, 0.02)	0.22	0.20	1319	593	23.5	0.01 (−0.03, 0.05)	0.08	0.14
Bangladesh	RDNS (35)	739	359	39.6	−0.04 (−0.09, 0.01)	0.10	0.11	924	456	45.6	−0.05 (−0.10, 0.00)	0.05	0.09
Bangladesh	WASH−B (36)	644	1936	31.8	−0.02 (−0.07, 0.02)	0.14	0.14	519	1527	34.1	−0.04 (−0.09, 0.01)	0.06	0.11
Burkina Faso	iLiNS−Zinc (37)	1387	477	40.9	−0.05 (−0.10, 0.00)	0.09	0.10	559	187	41.3	−0.02 (−0.11, 0.06)	0.02	0.04
Burkina Faso	PROMIS (38)												
Burkina Faso	PROMIS CS (38)												
Ghana	GHANA (39)												
Ghana	iLiNS−DYADG (40)	204	408	18.4	−0.05 (−0.11, 0.01)	0.06	0.07	143	284	12.7	0.03 (−0.04, 0.10)	0.03	0.05
Haiti	HAITI (41)												
Kenya	WASH−B (42)	349	1161	4.9	0.00 (−0.03, 0.03)	0.27	0.24	1112	4015	4.2	0.00 (−0.02, 0.01)	0.73	0.50
Madagascar	MAHAY (43)												
Malawi	iLiNS−DYADM (44)	156	316	10.8	−0.04 (−0.09, 0.02)	0.07	0.08	64	128	10.9	0.05 (−0.05, 0.15)	0.01	0.03
Malawi	iLiNS−DOSE (45)	418	133	25.4	−0.09 (−0.16, −0.02)	0.05	0.06	274	105	17.1	0.01 (−0.07, 0.09)	0.02	0.04
Mali	PROMIS (46)												
Mali	PROMIS CS (46)												
Zimbabwe	SHINE (HIV−) (47)												
Zimbabwe	SHINE (HIV+) (48)												
		**5463**	**5464**		**I² = 0.15, Tau² = 0.00**			**4914**	**7295**		**I² = 0.10, Tau² = 0.00**		
**Fixed**					**−0.02 (−0.04, −0.01)**						**−0.01 (−0.02, 0.00)**		
**Random**					**−0.03 (−0.04, −0.01)**						**−0.01 (−0.02, 0.01)**		

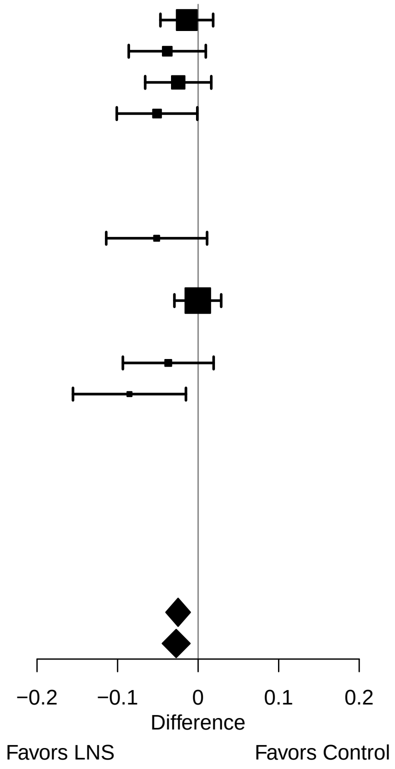

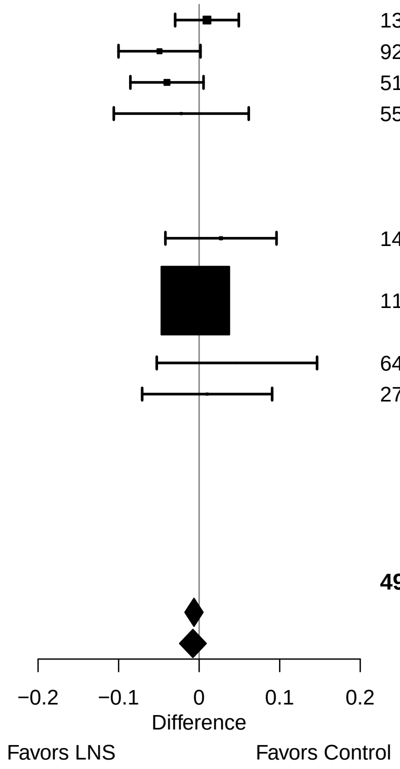

Supplement: nqab278_Supplemental_Files [file nqab278_supplemental_files.zip › 13_SQ-LNS_IPD_growth_Supplemental_Figure_9.pdf]
