# Supplementary material for: Characteristics that modify the effect of small-quantity lipid-based nutrient supplementation on child growth: an individual participant data meta-analysis of randomized controlled trials
Source: Am J Clin Nutr. 2021 Sep 29;114(Suppl 1):15S–42S. doi: 10.1093/ajcn/nqab278 (PMC8560308; doi:10.1093/ajcn/nqab278)
Supplement: nqab278_Supplemental_Files [file nqab278_supplemental_files.zip › 4_SQ-LNS_IPD_growth_Supplemental_Figures_Table_of_Contents.docx]

**Online Supplemental Material**

Characteristics that modify the effect of small-quantity lipid-based nutrient supplementation on child growth: an individual participant data meta-analysis of randomized controlled trials

Dewey *et al.* (2021)

**Table of Contents: Supplemental Figures**

Supplemental Figure 1: Summary risk of bias as a percentage of all included studies for the effects of SQ-LNS on growth outcomes

Supplemental Figure 2: Sensitivity analyses of main effects of SQ-LNS on growth outcomes

Supplemental Figure 3: Forest plots for all main effects of SQ-LNS on growth outcomes

Supplemental Figure 4: Forest plots for effects of SQ-LNS on growth outcomes stratified by study implementation within an existing program vs. not program-based

Supplemental Figure 5: Forest plots for effects of SQ-LNS on growth outcomes stratified by extent of social and behavioral change communication (SBCC) for infant and young child feeding (IYCF) provided by the study

Supplemental Figure 6: Forest plots for effects of SQ-LNS on growth outcomes stratified by study-level effect modifiers

Supplemental Figure 7: Pooled effect of SQ-LNS on prevalence ratios for low MUAC, acute malnutrition, underweight and small head size, stratified by study-level and individual-level effect characteristics

Supplemental Figure 8: Forest plots for effects of SQ-LNS on growth outcomes stratified by individual-level maternal and child effect modifiers

Supplemental Figure 9: Forest plots for effects of SQ-LNS on growth outcomes stratified by individual-level household effect modifiers
