# Supplementary material for: Characteristics that modify the effect of small-quantity lipid-based nutrient supplementation on child growth: an individual participant data meta-analysis of randomized controlled trials
Source: Am J Clin Nutr. 2021 Sep 29;114(Suppl 1):15S–42S. doi: 10.1093/ajcn/nqab278 (PMC8560308; doi:10.1093/ajcn/nqab278)

Supplemental Figure 1: Summary risk of bias as a percentage of all included studies for the effects of SQ-LNS on growth outcomes

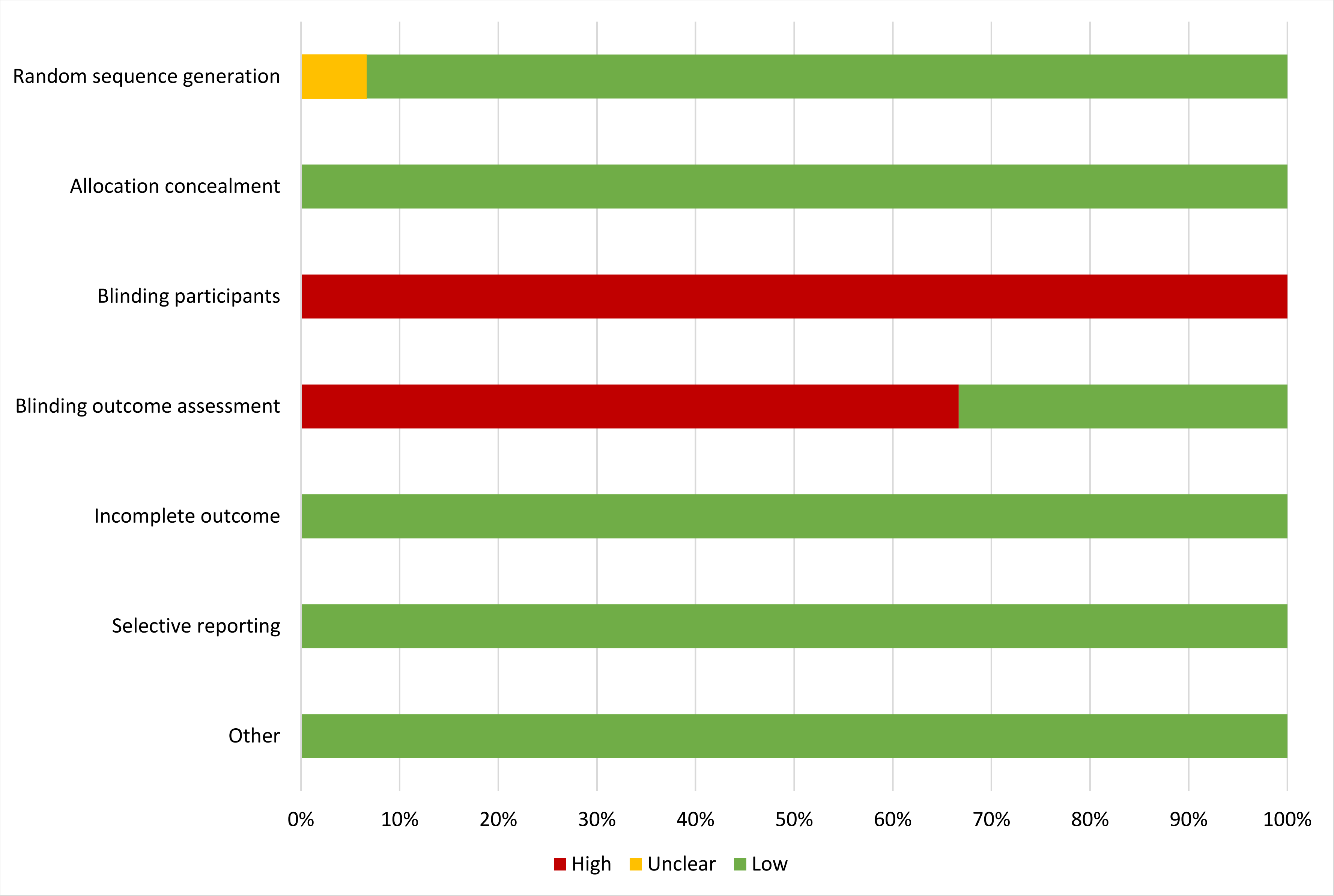

Supplement: nqab278_Supplemental_Files [file nqab278_supplemental_files.zip › 5_SQ-LNS_IPD_growth_Supplemental_Figure_1.pdf]
