# Supplementary material for: Characteristics that modify the effect of small-quantity lipid-based nutrient supplementation on child growth: an individual participant data meta-analysis of randomized controlled trials
Source: Am J Clin Nutr. 2021 Sep 29;114(Suppl 1):15S–42S. doi: 10.1093/ajcn/nqab278 (PMC8560308; doi:10.1093/ajcn/nqab278)

Supplemental figure 3: Forest plots for all main effects of SQ-LNS on growth outcomes

Contents

Supplemental figure 3A: Mean difference in LAZ 2

Supplemental figure 3B: Stunting prevalence ratio 3

Supplemental figure 3C: Stunting prevalence difference 4

Supplemental figure 3D: Mean difference in WLZ 5

Supplemental figure 3E: Wasting prevalence ratio 6

Supplemental figure 3F: Wasting prevalence difference 7

Supplemental figure 3G: Mean difference in MUACZ 8

Supplemental figure 3H: Low MUAC prevalence ratio 9

Supplemental figure 3I: Low MUAC prevalence difference 10

Supplemental figure 3J: Acute malnutrition prevalence ratio 11

Supplemental figure 3K: Acute malnutrition prevalence difference 12

Supplemental figure 3L: Mean difference in WAZ 13

Supplemental figure 3M: Underweight prevalence ratio 14

Supplemental figure 3N: Underweight prevalence difference 15

Supplemental figure 3O: Mean difference in HCZ 16

Supplemental figure 3P: Small head size prevalence ratio 17

Supplemental figure 3Q: Small head size prevalence difference 18

These figures are forest plots showing the study-level estimates of intervention effect with the pooled estimate in the bottom summary rows. Individual study estimates were generated from log-binomial regression for dichotomous outcomes and simple linear regression for continuous outcomes; controlling for baseline measure when available and with clustered observations using robust standard errors for cluster-randomized trials. Pooled estimates were generated using inverse-variance weighting in both fixed and random effects models. For continuous outcomes the intervention effect is measured by the difference in mean of the LNS group minus control. For dichotomous outcomes analyzed via prevalence ratios the effect estimate is the prevalence in the LNS group divided by the prevalence in the control group. For dichotomous outcomes analyzed via prevalence differences the effect estimate is the prevalence in the LNS group minus the prevalence in the control group. The labels on the left y-axis correspond to trial level information. The values on the right indicate the study level effect estimate, confidence interval, and weighting for deriving the pooled estimate. LAZ, length-for-age z-score; WLZ, weight-for-length z-score; WAZ, weight-for-age z-score; MUACZ, mid-upper arm circumference z-score; HCZ, head circumference-for-age z-score.

Supplemental figure 3A: Mean difference in LAZ

Country	Trial	LNS N	Control N	MD (95% CI)	Fixed W	Random W
Bangladesh	JiVitA−4 (34)	2838	1244	0.09 (0.03, 0.14)	0.21	0.09
Bangladesh	RDNS (35)	1663	815	0.08 (−0.01, 0.16)	0.08	0.08
Bangladesh	WASH−B (36)	1158	3431	0.21 (0.14, 0.28)	0.12	0.08
Burkina Faso	iLiNS−Zinc (37)	1952	664	0.31 (0.22, 0.39)	0.08	0.07
Burkina Faso	PROMIS (38)	863	914	0.15 (0.02, 0.27)	0.03	0.06
Burkina Faso	PROMIS CS (38)	430	439	0.04 (−0.14, 0.22)	0.02	0.04
Ghana	GHANA (39)	98	96	0.28 (−0.02, 0.58)	0.01	0.02
Ghana	iLiNS−DYAD−G (40)	347	692	0.20 (0.07, 0.33)	0.03	0.06
Haiti	HAITI (41)	149	149	−0.01 (−0.14, 0.13)	0.03	0.05
Kenya	WASH−B (42)	1457	5137	0.15 (0.09, 0.21)	0.15	0.08
Madagascar	MAHAY (43)	1702	1682	0.03 (−0.11, 0.17)	0.03	0.05
Malawi	iLiNS−DYAD−M (44)	220	444	−0.08 (−0.25, 0.10)	0.02	0.04
Malawi	iLiNS−DOSE (45)	696	241	0.06 (−0.04, 0.17)	0.05	0.07
Mali	PROMIS (46)	506	506	0.16 (0.01, 0.31)	0.02	0.05
Mali	PROMIS CS (46)	952	969	0.22 (0.05, 0.39)	0.02	0.04
Zimbabwe	SHINE (HIV−) (47)	1880	1794	0.16 (0.08, 0.23)	0.09	0.08
Zimbabwe	SHINE (HIV+) (48)	337	330	0.25 (0.08, 0.43)	0.02	0.04
	**I² = 0.65, Tau² = 0.01**	**17248**	**19547**			
	**Fixed**			**0.14 (0.11, 0.16)**		
	**Random**			**0.13 (0.09, 0.18)**		

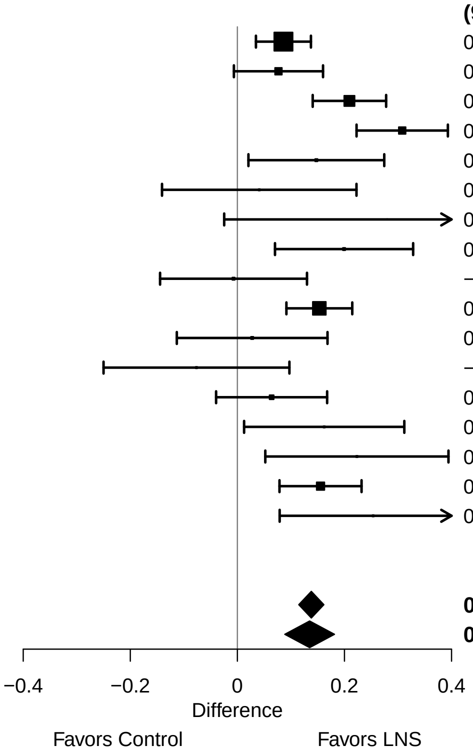

Supplemental figure 3B: Stunting prevalence ratio

Country	Trial	LNS N	Control N	PR (95% CI)	Fixed W	Random W
Bangladesh	JiVitA−4 (34)	2838	1244	0.92 (0.84, 1.01)	0.12	0.10
Bangladesh	RDNS (35)	1663	815	0.92 (0.85, 1.00)	0.15	0.10
Bangladesh	WASH−B (36)	1158	3431	0.84 (0.77, 0.91)	0.14	0.10
Burkina Faso	iLiNS−Zinc (37)	1952	664	0.75 (0.65, 0.87)	0.04	0.06
Burkina Faso	PROMIS (38)	863	914	0.82 (0.67, 1.00)	0.03	0.04
Burkina Faso	PROMIS CS (38)	430	439	0.87 (0.63, 1.19)	0.01	0.02
Ghana	GHANA (39)	98	96	0.70 (0.23, 2.13)	0.00	0.00
Ghana	iLiNS−DYAD−G (40)	347	692	0.66 (0.45, 0.98)	0.01	0.01
Haiti	HAITI (41)	149	149	1.04 (0.60, 1.81)	0.00	0.01
Kenya	WASH−B (42)	1457	5137	0.86 (0.78, 0.94)	0.11	0.09
Madagascar	MAHAY (43)	1702	1682	0.98 (0.90, 1.07)	0.12	0.10
Malawi	iLiNS−DYAD−M (44)	220	444	1.10 (0.89, 1.36)	0.02	0.04
Malawi	iLiNS−DOSE (45)	696	241	1.00 (0.87, 1.15)	0.05	0.07
Mali	PROMIS (46)	506	506	0.92 (0.76, 1.10)	0.03	0.05
Mali	PROMIS CS (46)	952	969	0.78 (0.65, 0.95)	0.03	0.05
Zimbabwe	SHINE (HIV−) (47)	1880	1794	0.79 (0.72, 0.87)	0.10	0.09
Zimbabwe	SHINE (HIV+) (48)	337	330	0.81 (0.68, 0.96)	0.03	0.05
	**I^2 = 0.49, Tau^2 = 0.00**	**17248**	**19547**			
	**Fixed**			**0.88 (0.85, 0.91)**		
	**Random**			**0.88 (0.83, 0.92)**		

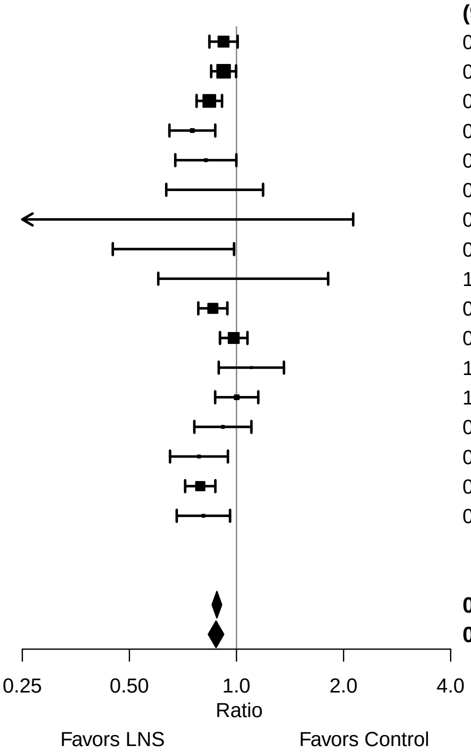

Supplemental figure 3C: Stunting prevalence difference

Country	Trial	LNS N	Control N	PD (95% CI)	Fixed W	Random W
Bangladesh	JiVitA−4 (34)	2838	1244	−0.04 (−0.06, −0.01)	0.13	0.09
Bangladesh	RDNS (35)	1663	815	−0.03 (−0.07, 0.01)	0.05	0.06
Bangladesh	WASH−B (36)	1158	3431	−0.07 (−0.10, −0.04)	0.09	0.08
Burkina Faso	iLiNS−Zinc (37)	1952	664	−0.11 (−0.14, −0.08)	0.09	0.08
Burkina Faso	PROMIS (38)	863	914	−0.05 (−0.10, −0.01)	0.05	0.06
Burkina Faso	PROMIS CS (38)	430	439	−0.03 (−0.09, 0.02)	0.03	0.04
Ghana	GHANA (39)	98	96	−0.02 (−0.09, 0.05)	0.02	0.03
Ghana	iLiNS−DYAD−G (40)	347	692	−0.04 (−0.08, 0.00)	0.06	0.07
Haiti	HAITI (41)	149	149	−0.02 (−0.08, 0.04)	0.02	0.04
Kenya	WASH−B (42)	1457	5137	−0.05 (−0.07, −0.02)	0.13	0.09
Madagascar	MAHAY (43)	1702	1682	−0.01 (−0.04, 0.02)	0.08	0.07
Malawi	iLiNS−DYAD−M (44)	220	444	0.03 (−0.04, 0.11)	0.01	0.03
Malawi	iLiNS−DOSE (45)	696	241	−0.02 (−0.07, 0.04)	0.03	0.04
Mali	PROMIS (46)	506	506	−0.04 (−0.09, 0.01)	0.04	0.05
Mali	PROMIS CS (46)	952	969	−0.07 (−0.11, −0.03)	0.05	0.06
Zimbabwe	SHINE (HIV−) (47)	1880	1794	−0.07 (−0.10, −0.04)	0.10	0.08
Zimbabwe	SHINE (HIV+) (48)	337	330	−0.10 (−0.17, −0.02)	0.02	0.03
	**I^2 = 0.54, Tau^2 = 0.00**	**17248**	**19547**			
	**Fixed**			**−0.05 (−0.06, −0.04)**		
	**Random**			**−0.05 (−0.06, −0.03)**		

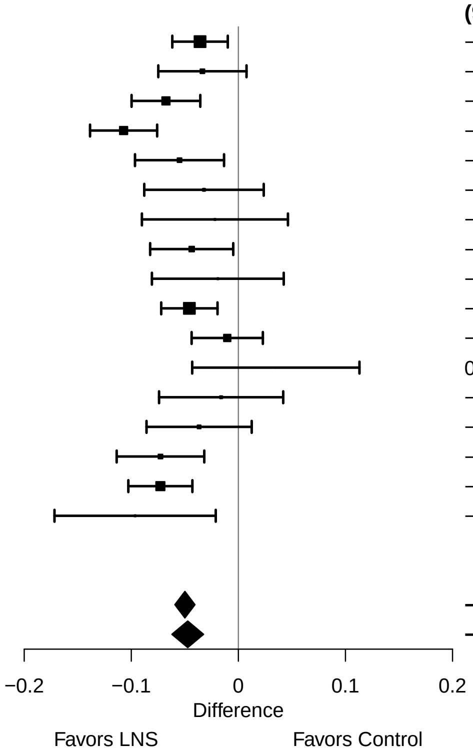

Supplemental figure 3D: Mean difference in WLZ

Country	Trial	LNS N	Control N	MD (95% CI)	Fixed W	Random W
Bangladesh	JiVitA−4 (34)	2783	1217	0.08 (0.03, 0.13)	0.22	0.10
Bangladesh	RDNS (35)	1661	815	0.07 (0.00, 0.15)	0.08	0.08
Bangladesh	WASH−B (36)	1156	3424	0.12 (0.05, 0.19)	0.10	0.08
Burkina Faso	iLiNS−Zinc (37)	1952	664	0.18 (0.11, 0.26)	0.09	0.08
Burkina Faso	PROMIS (38)	856	907	0.10 (−0.03, 0.22)	0.03	0.05
Burkina Faso	PROMIS CS (38)	430	436	−0.11 (−0.24, 0.02)	0.03	0.05
Ghana	GHANA (39)	98	96	0.21 (−0.13, 0.54)	0.00	0.01
Ghana	iLiNS−DYAD−G (40)	347	692	0.09 (−0.04, 0.22)	0.03	0.05
Haiti	HAITI (41)	148	149	0.09 (−0.07, 0.24)	0.02	0.04
Kenya	WASH−B (42)	1455	5118	0.06 (0.00, 0.13)	0.13	0.09
Madagascar	MAHAY (43)	1700	1682	0.00 (−0.11, 0.11)	0.04	0.06
Malawi	iLiNS−DYAD−M (44)	220	444	−0.05 (−0.21, 0.11)	0.02	0.04
Malawi	iLiNS−DOSE (45)	696	241	0.01 (−0.12, 0.14)	0.03	0.05
Mali	PROMIS (46)	499	502	0.10 (−0.02, 0.23)	0.03	0.05
Mali	PROMIS CS (46)	944	959	0.16 (0.06, 0.26)	0.05	0.06
Zimbabwe	SHINE (HIV−) (47)	1869	1784	0.08 (0.00, 0.15)	0.09	0.08
Zimbabwe	SHINE (HIV+) (48)	336	328	−0.14 (−0.32, 0.04)	0.01	0.03
	**I^2 = 0.51, Tau^2 = 0.00**	**17150**	**19458**			
	**Fixed**			**0.08 (0.06, 0.10)**		
	**Random**			**0.07 (0.03, 0.11)**		

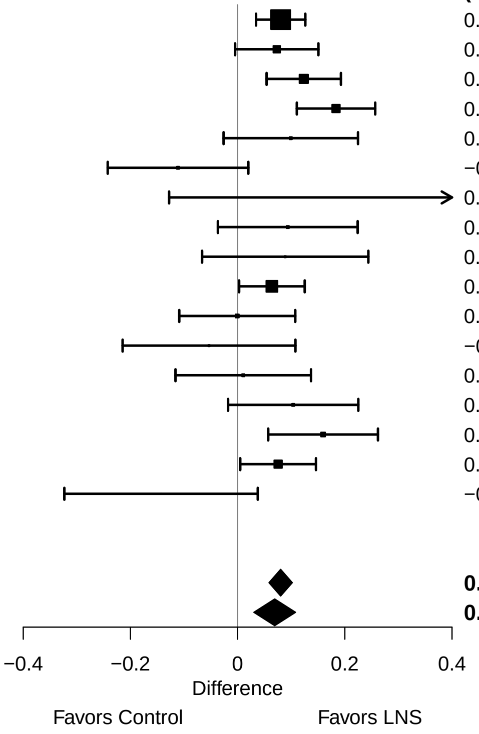

Supplemental figure 3E: Wasting prevalence ratio

Country	Trial	LNS N	Control N	PR (95% CI)	Fixed W	Random W
Bangladesh	JiVitA−4 (34)	2783	1217	0.91 (0.78, 1.05)	0.27	0.27
Bangladesh	RDNS (35)	1661	815	0.87 (0.70, 1.07)	0.13	0.13
Bangladesh	WASH−B (36)	1156	3424	0.79 (0.65, 0.96)	0.16	0.16
Burkina Faso	iLiNS−Zinc (37)	1952	664	0.69 (0.54, 0.87)	0.11	0.11
Burkina Faso	PROMIS (38)	856	907	0.86 (0.59, 1.27)	0.04	0.04
Burkina Faso	PROMIS CS (38)	430	436	1.03 (0.72, 1.49)	0.04	0.04
Ghana	GHANA (39)	98	96	0.87 (0.35, 2.16)	0.01	0.01
Ghana	iLiNS−DYAD−G (40)	347	692	0.84 (0.52, 1.37)	0.03	0.03
Haiti	HAITI (41)					
Kenya	WASH−B (42)	1455	5118	0.88 (0.53, 1.45)	0.02	0.02
Madagascar	MAHAY (43)	1700	1682	0.96 (0.67, 1.38)	0.05	0.05
Malawi	iLiNS−DYAD−M (44)	220	444	0.94 (0.39, 2.28)	0.01	0.01
Malawi	iLiNS−DOSE (45)	696	241	1.37 (0.78, 2.40)	0.02	0.02
Mali	PROMIS (46)	499	502	0.61 (0.15, 2.42)	0.00	0.00
Mali	PROMIS CS (46)	944	959	0.83 (0.63, 1.10)	0.07	0.07
Zimbabwe	SHINE (HIV−) (47)	1869	1784	0.89 (0.60, 1.34)	0.04	0.04
Zimbabwe	SHINE (HIV+) (48)	336	328	1.38 (0.65, 2.96)	0.01	0.01
	**I² = 0.00, Tau² = 0.00**	**17002**	**19309**			
	**Fixed**			**0.86 (0.80, 0.93)**		
	**Random**			**0.86 (0.80, 0.93)**		

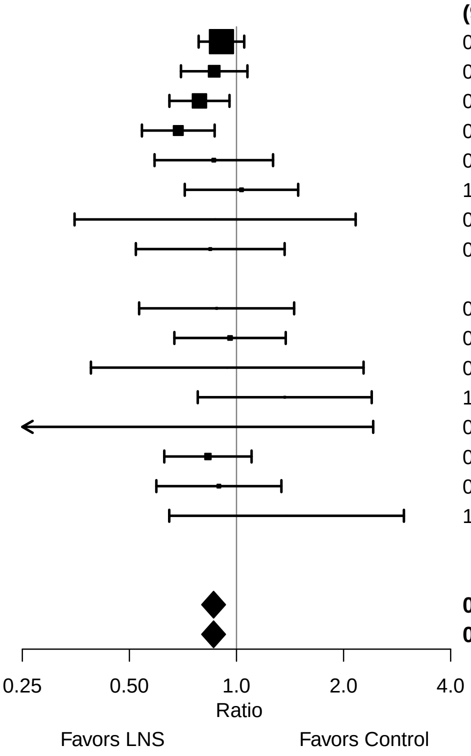

Supplemental figure 3F: Wasting prevalence difference

Country	Trial	LNS N	Control N	PD (95% CI)	Fixed W	Random W
Bangladesh	JiVitA−4 (34)	2783	1217	−0.01 (−0.04, 0.01)	0.04	0.04
Bangladesh	RDNS (35)	1661	815	−0.02 (−0.05, 0.01)	0.02	0.02
Bangladesh	WASH−B (36)	1156	3424	−0.02 (−0.04, 0.00)	0.05	0.05
Burkina Faso	iLiNS−Zinc (37)	1952	664	−0.04 (−0.07, −0.01)	0.03	0.03
Burkina Faso	PROMIS (38)	856	907	−0.01 (−0.04, 0.01)	0.03	0.03
Burkina Faso	PROMIS CS (38)	430	436	0.00 (−0.04, 0.05)	0.01	0.01
Ghana	GHANA (39)	98	96	−0.01 (−0.09, 0.07)	0.00	0.00
Ghana	iLiNS−DYAD−G (40)	347	692	−0.01 (−0.04, 0.02)	0.02	0.02
Haiti	HAITI (41)					
Kenya	WASH−B (42)	1455	5118	0.00 (−0.01, 0.00)	0.39	0.39
Madagascar	MAHAY (43)	1700	1682	0.00 (−0.02, 0.01)	0.07	0.07
Malawi	iLiNS−DYAD−M (44)	220	444	0.00 (−0.03, 0.03)	0.02	0.02
Malawi	iLiNS−DOSE (45)	696	241	0.01 (−0.03, 0.04)	0.02	0.02
Mali	PROMIS (46)	499	502	−0.01 (−0.02, 0.01)	0.09	0.09
Mali	PROMIS CS (46)	944	959	−0.02 (−0.04, 0.01)	0.03	0.03
Zimbabwe	SHINE (HIV−) (47)	1869	1784	0.00 (−0.01, 0.01)	0.18	0.18
Zimbabwe	SHINE (HIV+) (48)	336	328	0.01 (−0.02, 0.05)	0.02	0.02
	**I^2 = 0.12, Tau^2 = 0.00**	**17002**	**19309**			
	**Fixed**			**−0.01 (−0.01, 0.00)**		
	**Random**			**−0.01 (−0.01, 0.00)**		

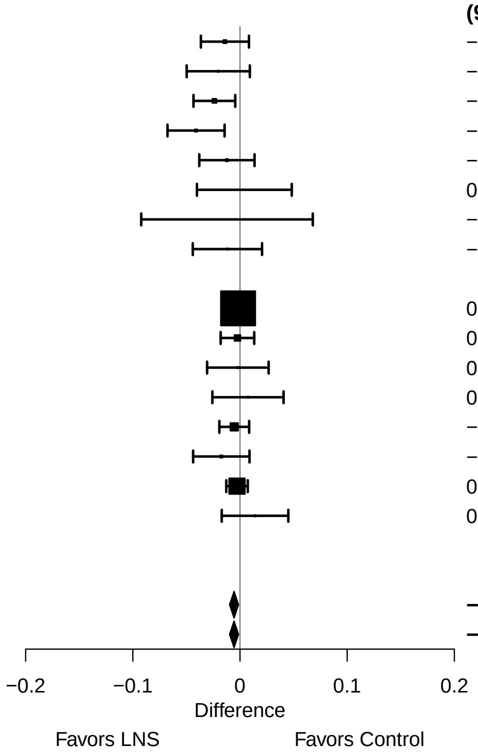

Supplemental figure 3G: Mean difference in MUACZ

Country	Trial	LNS N	Control N	MD (95% CI)	Fixed W	Random W
Bangladesh	JiVitA−4 (34)	2897	1271	0.08 (0.03, 0.13)	0.25	0.10
Bangladesh	RDNS (35)	1663	815	0.04 (−0.04, 0.12)	0.09	0.09
Bangladesh	WASH−B (36)					
Burkina Faso	iLiNS−Zinc (37)	1959	666	0.30 (0.20, 0.39)	0.06	0.08
Burkina Faso	PROMIS (38)	854	912	0.09 (−0.03, 0.21)	0.04	0.06
Burkina Faso	PROMIS CS (38)	424	436	−0.06 (−0.22, 0.10)	0.02	0.05
Ghana	GHANA (39)					
Ghana	iLiNS−DYAD−G (40)	347	693	0.09 (−0.03, 0.20)	0.04	0.07
Haiti	HAITI (41)					
Kenya	WASH−B (42)	1461	5185	0.12 (0.05, 0.18)	0.13	0.09
Madagascar	MAHAY (43)	1680	1658	−0.03 (−0.16, 0.09)	0.04	0.06
Malawi	iLiNS−DYAD−M (44)	220	444	−0.04 (−0.20, 0.12)	0.02	0.05
Malawi	iLiNS−DOSE (45)	696	240	0.06 (−0.05, 0.18)	0.05	0.07
Mali	PROMIS (46)	507	505	0.19 (0.05, 0.32)	0.03	0.06
Mali	PROMIS CS (46)	954	970	0.13 (0.01, 0.25)	0.04	0.07
Zimbabwe	SHINE (HIV−) (47)	1872	1780	0.06 (0.00, 0.12)	0.15	0.10
Zimbabwe	SHINE (HIV+) (48)	337	328	0.04 (−0.10, 0.17)	0.03	0.06
	**$I^2 = 0.62$, $Tau^2 = 0.00$**	**15871**	**15903**			
	**Fixed**			**0.09 (0.06, 0.11)**		
	**Random**			**0.08 (0.04, 0.13)**		

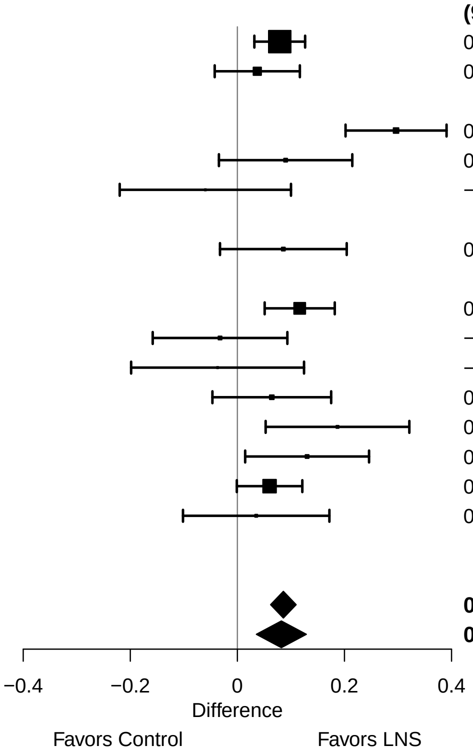

Supplemental figure 3H: Low MUAC prevalence ratio

Country	Trial	LNS N	Control N	PR (95% CI)	Fixed W	Random W
Bangladesh	JiVitA−4 (34)	2897	1271	0.85 (0.70, 1.03)	0.22	0.19
Bangladesh	RDNS (35)	1663	815	1.02 (0.69, 1.49)	0.06	0.06
Bangladesh	WASH−B (36)					
Burkina Faso	iLiNS−Zinc (37)	1959	666	0.64 (0.51, 0.79)	0.18	0.16
Burkina Faso	PROMIS (38)	854	912	0.94 (0.63, 1.40)	0.05	0.06
Burkina Faso	PROMIS CS (38)	424	436	0.92 (0.66, 1.28)	0.08	0.08
Ghana	GHANA (39)					
Ghana	iLiNS−DYAD−G (40)	347	693	0.77 (0.37, 1.57)	0.02	0.02
Haiti	HAITI (41)					
Kenya	WASH−B (42)	1461	5185	0.71 (0.54, 0.94)	0.11	0.11
Madagascar	MAHAY (43)	1680	1658	0.97 (0.76, 1.26)	0.13	0.13
Malawi	iLiNS−DYAD−M (44)	220	444	0.94 (0.39, 2.28)	0.01	0.01
Malawi	iLiNS−DOSE (45)	696	240	1.35 (0.54, 3.36)	0.01	0.01
Mali	PROMIS (46)	507	505	0.58 (0.31, 1.07)	0.02	0.03
Mali	PROMIS CS (46)	954	970	0.90 (0.68, 1.19)	0.10	0.11
Zimbabwe	SHINE (HIV−) (47)	1872	1780	0.51 (0.27, 0.97)	0.02	0.02
Zimbabwe	SHINE (HIV+) (48)	337	328	0.97 (0.29, 3.28)	0.01	0.01
	**I² = 0.16, Tau² = 0.00**	**15871**	**15903**			
	**Fixed**			**0.82 (0.75, 0.89)**		
	**Random**			**0.82 (0.74, 0.91)**		

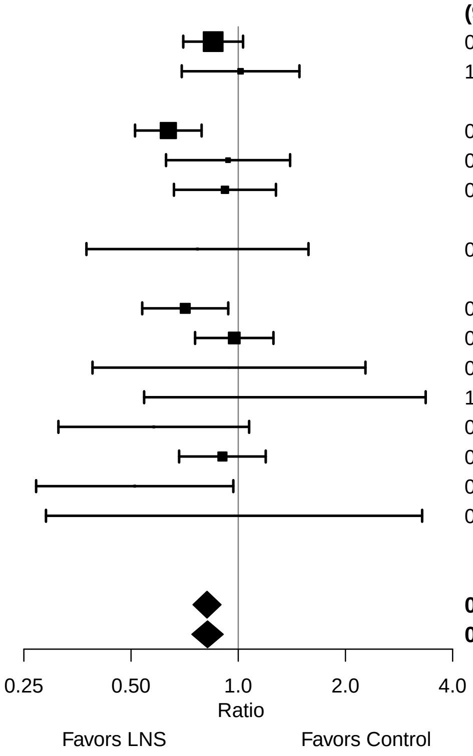

Supplemental figure 3I: Low MUAC prevalence difference

Country	Trial	LNS N	Control N	PD (95% CI)	Fixed W	Random W
Bangladesh	JiVitA−4 (34)	2897	1271	−0.02 (−0.04, 0.00)	0.05	0.08
Bangladesh	RDNS (35)	1663	815	0.00 (−0.02, 0.02)	0.06	0.08
Bangladesh	WASH−B (36)					
Burkina Faso	iLiNS−Zinc (37)	1959	666	−0.07 (−0.10, −0.04)	0.02	0.05
Burkina Faso	PROMIS (38)	854	912	−0.01 (−0.03, 0.02)	0.03	0.06
Burkina Faso	PROMIS CS (38)	424	436	−0.01 (−0.06, 0.03)	0.01	0.03
Ghana	GHANA (39)					
Ghana	iLiNS−DYAD−G (40)	347	693	−0.01 (−0.03, 0.01)	0.04	0.07
Haiti	HAITI (41)					
Kenya	WASH−B (42)	1461	5185	−0.02 (−0.03, 0.00)	0.14	0.10
Madagascar	MAHAY (43)	1680	1658	0.00 (−0.02, 0.02)	0.05	0.08
Malawi	iLiNS−DYAD−M (44)	220	444	0.00 (−0.03, 0.03)	0.02	0.06
Malawi	iLiNS−DOSE (45)	696	240	0.01 (−0.01, 0.03)	0.04	0.07
Mali	PROMIS (46)	507	505	−0.02 (−0.05, 0.00)	0.03	0.06
Mali	PROMIS CS (46)	954	970	−0.01 (−0.04, 0.02)	0.03	0.06
Zimbabwe	SHINE (HIV−) (47)	1872	1780	−0.01 (−0.01, 0.00)	0.42	0.11
Zimbabwe	SHINE (HIV+) (48)	337	328	0.00 (−0.02, 0.02)	0.06	0.08
	**I² = 0.50, Tau² = 0.00**	**15871**	**15903**			
	**Fixed**			**−0.01 (−0.01, 0.00)**		
	**Random**			**−0.01 (−0.02, 0.00)**		

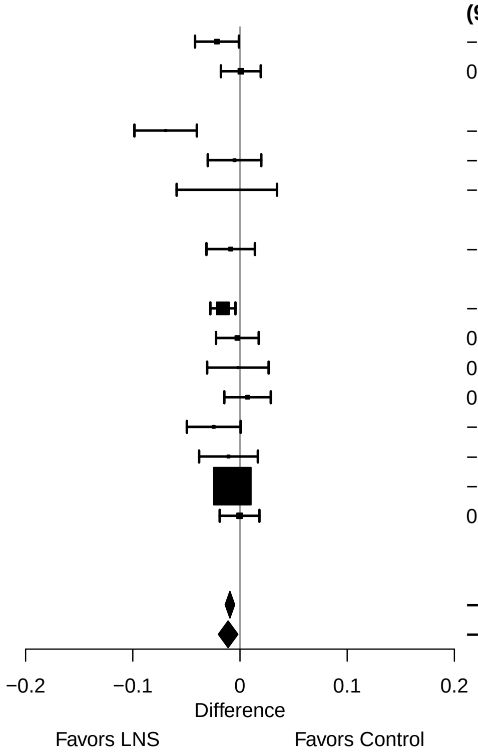

Supplemental figure 3J: Acute malnutrition prevalence ratio

Country	Trial	LNS N	Control N	PR (95% CI)	Fixed W	Random W
Bangladesh	JiVitA−4 (34)	2783	1216	0.89 (0.78, 1.01)	0.33	0.33
Bangladesh	RDNS (35)	1661	815	0.86 (0.70, 1.05)	0.13	0.13
Bangladesh	WASH−B (36)					
Burkina Faso	iLiNS−Zinc (37)	1951	664	0.67 (0.54, 0.85)	0.10	0.10
Burkina Faso	PROMIS (38)	846	901	0.83 (0.60, 1.14)	0.05	0.05
Burkina Faso	PROMIS CS (38)	424	434	0.95 (0.73, 1.23)	0.08	0.08
Ghana	GHANA (39)					
Ghana	iLiNS−DYAD−G (40)	347	692	0.85 (0.54, 1.35)	0.02	0.02
Haiti	HAITI (41)					
Kenya	WASH−B (42)	1454	5117	0.71 (0.49, 1.02)	0.04	0.04
Madagascar	MAHAY (43)	1675	1655	0.98 (0.75, 1.29)	0.07	0.07
Malawi	iLiNS−DYAD−M (44)	220	444	0.81 (0.36, 1.80)	0.01	0.01
Malawi	iLiNS−DOSE (45)	690	240	1.42 (0.83, 2.43)	0.02	0.02
Mali	PROMIS (46)	499	502	0.59 (0.23, 1.47)	0.01	0.01
Mali	PROMIS CS (46)	944	959	0.89 (0.70, 1.13)	0.09	0.09
Zimbabwe	SHINE (HIV−) (47)	1867	1777	0.79 (0.55, 1.14)	0.04	0.04
Zimbabwe	SHINE (HIV+) (48)	336	327	1.23 (0.61, 2.48)	0.01	0.01
	**I^2 = 0.00, Tau^2 = 0.00**	**15697**	**15743**			
	**Fixed**			**0.86 (0.80, 0.93)**		
	**Random**			**0.86 (0.80, 0.93)**		

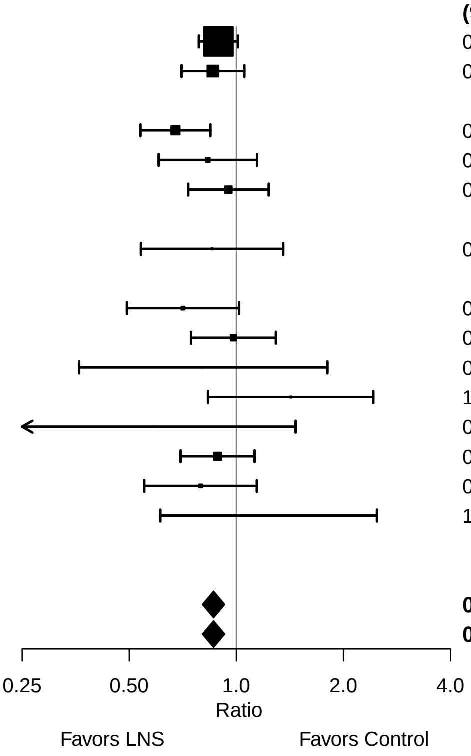

Supplemental figure 3K: Acute malnutrition prevalence difference

Country	Trial	LNS N	Control N	PD (95% CI)	Fixed W	Random W
Bangladesh	JiVitA−4 (34)	2783	1216	−0.02 (−0.05, 0.00)	0.05	0.07
Bangladesh	RDNS (35)	1661	815	−0.02 (−0.05, 0.01)	0.03	0.05
Bangladesh	WASH−B (36)					
Burkina Faso	iLiNS−Zinc (37)	1951	664	−0.06 (−0.09, −0.03)	0.03	0.05
Burkina Faso	PROMIS (38)	846	901	−0.02 (−0.05, 0.01)	0.03	0.06
Burkina Faso	PROMIS CS (38)	424	434	−0.01 (−0.06, 0.04)	0.01	0.02
Ghana	GHANA (39)					
Ghana	iLiNS−DYAD−G (40)	347	692	−0.01 (−0.05, 0.02)	0.02	0.04
Haiti	HAITI (41)					
Kenya	WASH−B (42)	1454	5117	−0.01 (−0.02, 0.00)	0.34	0.17
Madagascar	MAHAY (43)	1675	1655	0.00 (−0.02, 0.02)	0.07	0.09
Malawi	iLiNS−DYAD−M (44)	220	444	−0.01 (−0.04, 0.02)	0.03	0.05
Malawi	iLiNS−DOSE (45)	690	240	0.01 (−0.03, 0.04)	0.02	0.04
Mali	PROMIS (46)	499	502	−0.01 (−0.03, 0.01)	0.08	0.10
Mali	PROMIS CS (46)	944	959	−0.01 (−0.04, 0.01)	0.03	0.05
Zimbabwe	SHINE (HIV−) (47)	1867	1777	−0.01 (−0.02, 0.00)	0.23	0.16
Zimbabwe	SHINE (HIV+) (48)	336	327	0.01 (−0.02, 0.04)	0.02	0.04
	**I² = 0.21, Tau² = 0.00**	**15697**	**15743**			
	**Fixed**			**−0.01 (−0.02, −0.01)**		
	**Random**			**−0.01 (−0.02, 0.00)**		

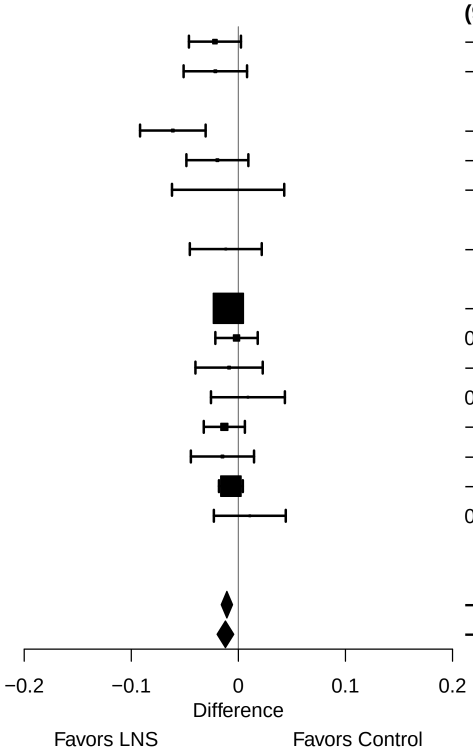

Supplemental figure 3L: Mean difference in WAZ

Country	Trial	LNS N	Control N	MD (95% CI)	Fixed W	Random W
Bangladesh	JiVitA−4 (34)	2814	1232	0.10 (0.07, 0.14)	0.33	0.09
Bangladesh	RDNS (35)	1663	815	0.09 (0.01, 0.18)	0.05	0.07
Bangladesh	WASH−B (36)	1163	3461	0.20 (0.13, 0.26)	0.09	0.08
Burkina Faso	iLiNS−Zinc (37)	1960	666	0.28 (0.21, 0.35)	0.08	0.08
Burkina Faso	PROMIS (38)	857	911	0.15 (0.03, 0.27)	0.03	0.06
Burkina Faso	PROMIS CS (38)	430	436	−0.05 (−0.19, 0.09)	0.02	0.05
Ghana	GHANA (39)	98	96	0.28 (−0.05, 0.61)	0.00	0.01
Ghana	iLiNS−DYAD−G (40)	347	693	0.16 (0.03, 0.29)	0.02	0.05
Haiti	HAITI (41)	148	150	0.06 (−0.06, 0.19)	0.03	0.05
Kenya	WASH−B (42)	1461	5165	0.13 (0.07, 0.20)	0.10	0.08
Madagascar	MAHAY (43)	1702	1685	0.01 (−0.10, 0.13)	0.03	0.06
Malawi	iLiNS−DYAD−M (44)	220	444	−0.07 (−0.23, 0.10)	0.01	0.04
Malawi	iLiNS−DOSE (45)	702	241	0.05 (−0.05, 0.16)	0.04	0.06
Mali	PROMIS (46)	500	502	0.16 (0.05, 0.28)	0.03	0.06
Mali	PROMIS CS (46)	946	960	0.23 (0.11, 0.36)	0.03	0.05
Zimbabwe	SHINE (HIV−) (47)	1869	1786	0.13 (0.07, 0.20)	0.09	0.08
Zimbabwe	SHINE (HIV+) (48)	336	328	0.01 (−0.16, 0.18)	0.01	0.04
	**I^2 = 0.66, Tau^2 = 0.01**	**17216**	**19571**			
	**Fixed**			**0.13 (0.11, 0.15)**		
	**Random**			**0.12 (0.07, 0.16)**		

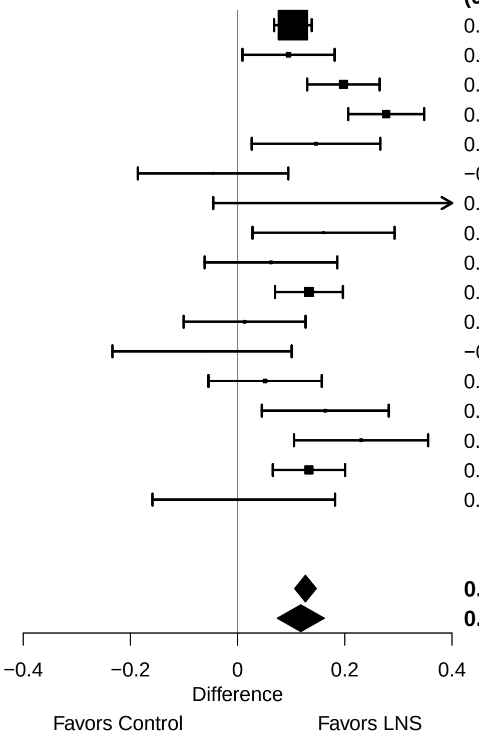

Supplemental figure 3M: Underweight prevalence ratio

Country	Trial	LNS N	Control N	PR (95% CI)	Fixed W	Random W
Bangladesh	JiVitA−4 (34)	2814	1232	0.91 (0.83, 1.01)	0.21	0.13
Bangladesh	RDNS (35)	1663	815	0.92 (0.83, 1.02)	0.18	0.12
Bangladesh	WASH−B (36)	1163	3461	0.79 (0.70, 0.89)	0.14	0.11
Burkina Faso	iLiNS−Zinc (37)	1960	666	0.70 (0.59, 0.82)	0.08	0.09
Burkina Faso	PROMIS (38)	857	911	0.86 (0.71, 1.05)	0.05	0.07
Burkina Faso	PROMIS CS (38)	430	436	0.99 (0.77, 1.26)	0.03	0.05
Ghana	GHANA (39)	98	96	0.71 (0.30, 1.69)	0.00	0.01
Ghana	iLiNS−DYAD−G (40)	347	693	0.82 (0.57, 1.18)	0.02	0.03
Haiti	HAITI (41)	148	150	0.71 (0.28, 1.78)	0.00	0.00
Kenya	WASH−B (42)	1461	5165	0.76 (0.62, 0.93)	0.05	0.06
Madagascar	MAHAY (43)	1702	1685	1.04 (0.89, 1.21)	0.08	0.09
Malawi	iLiNS−DYAD−M (44)	220	444	1.13 (0.74, 1.72)	0.01	0.02
Malawi	iLiNS−DOSE (45)	702	241	1.01 (0.78, 1.31)	0.03	0.05
Mali	PROMIS (46)	500	502	0.85 (0.62, 1.18)	0.02	0.03
Mali	PROMIS CS (46)	946	960	0.88 (0.71, 1.10)	0.04	0.06
Zimbabwe	SHINE (HIV−) (47)	1869	1786	0.71 (0.57, 0.87)	0.04	0.06
Zimbabwe	SHINE (HIV+) (48)	336	328	1.01 (0.73, 1.40)	0.02	0.03
	**I^2 = 0.42, Tau^2 = 0.01**	**17216**	**19571**			
	**Fixed**			**0.87 (0.83, 0.91)**		
	**Random**			**0.87 (0.81, 0.92)**		

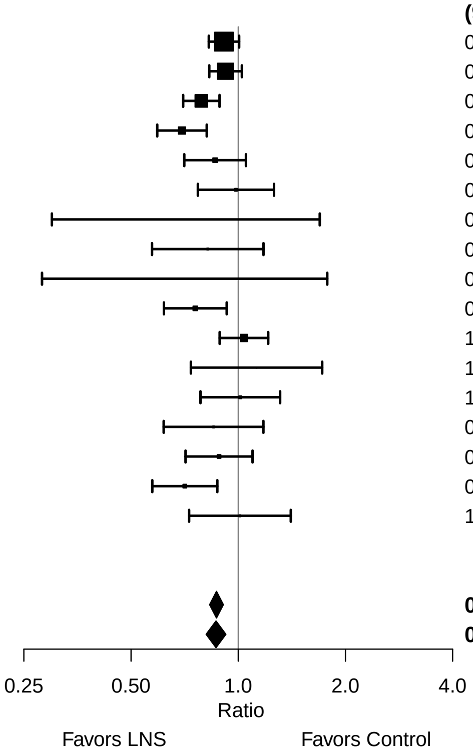

Supplemental figure 3N: Underweight prevalence difference

Country	Trial	LNS N	Control N	PD (95% CI)	Fixed W	Random W
Bangladesh	JiVitA−4 (34)	2814	1232	−0.04 (−0.07, −0.02)	0.09	0.08
Bangladesh	RDNS (35)	1663	815	−0.03 (−0.07, 0.01)	0.04	0.06
Bangladesh	WASH−B (36)	1163	3461	−0.07 (−0.10, −0.04)	0.07	0.07
Burkina Faso	iLiNS−Zinc (37)	1960	666	−0.10 (−0.13, −0.07)	0.06	0.07
Burkina Faso	PROMIS (38)	857	911	−0.03 (−0.06, 0.01)	0.05	0.06
Burkina Faso	PROMIS CS (38)	430	436	0.00 (−0.06, 0.05)	0.02	0.04
Ghana	GHANA (39)	98	96	−0.03 (−0.12, 0.05)	0.01	0.02
Ghana	iLiNS−DYAD−G (40)	347	693	−0.02 (−0.06, 0.02)	0.03	0.05
Haiti	HAITI (41)	148	150	0.00 (−0.05, 0.04)	0.03	0.05
Kenya	WASH−B (42)	1461	5165	−0.02 (−0.04, −0.01)	0.23	0.09
Madagascar	MAHAY (43)	1702	1685	0.01 (−0.02, 0.04)	0.07	0.07
Malawi	iLiNS−DYAD−M (44)	220	444	0.01 (−0.04, 0.07)	0.02	0.04
Malawi	iLiNS−DOSE (45)	702	241	−0.02 (−0.07, 0.03)	0.02	0.05
Mali	PROMIS (46)	500	502	−0.04 (−0.08, 0.00)	0.04	0.06
Mali	PROMIS CS (46)	946	960	−0.03 (−0.06, 0.01)	0.04	0.06
Zimbabwe	SHINE (HIV−) (47)	1869	1786	−0.03 (−0.05, −0.01)	0.17	0.09
Zimbabwe	SHINE (HIV+) (48)	336	328	0.00 (−0.06, 0.06)	0.02	0.04
	**I^2 = 0.60, Tau^2 = 0.00**	**17216**	**19571**			
	**Fixed**			**−0.03 (−0.04, −0.02)**		
	**Random**			**−0.03 (−0.04, −0.02)**		

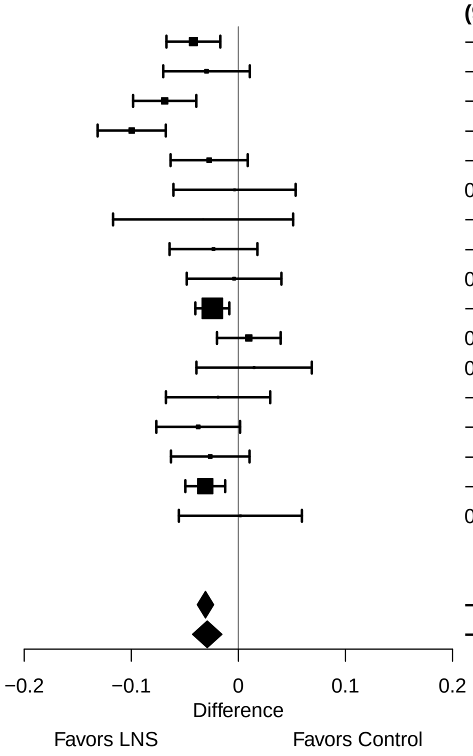

Supplemental figure 3O: Mean difference in HCZ

Country	Trial	LNS N	Control N	MD (95% CI)	Fixed W	Random W
Bangladesh	JiVitA−4 (34)	2885	1267	0.06 (0.01, 0.11)	0.23	0.15
Bangladesh	RDNS (35)	1663	815	0.10 (0.03, 0.17)	0.11	0.12
Bangladesh	WASH−B (36)	1163	3463	0.12 (0.06, 0.19)	0.13	0.13
Burkina Faso	iLiNS−Zinc (37)	1946	664	0.16 (0.10, 0.23)	0.14	0.13
Burkina Faso	PROMIS (38)					
Burkina Faso	PROMIS CS (38)					
Ghana	GHANA (39)	98	96	0.20 (−0.06, 0.46)	0.01	0.02
Ghana	iLiNS−DYAD−G (40)	347	692	0.07 (−0.05, 0.18)	0.04	0.07
Haiti	HAITI (41)					
Kenya	WASH−B (42)	1461	5176	0.04 (−0.02, 0.10)	0.15	0.13
Madagascar	MAHAY (43)					
Malawi	iLiNS−DYAD−M (44)	220	444	−0.13 (−0.28, 0.03)	0.02	0.04
Malawi	iLiNS−DOSE (45)	692	238	0.11 (−0.02, 0.23)	0.04	0.06
Mali	PROMIS (46)					
Mali	PROMIS CS (46)					
Zimbabwe	SHINE (HIV−) (47)	1871	1783	0.09 (0.02, 0.16)	0.11	0.12
Zimbabwe	SHINE (HIV+) (48)	337	329	0.10 (−0.07, 0.28)	0.02	0.04
	**I² = 0.46, Tau² = 0.00**	**12683**	**14967**			
	**Fixed**			**0.09 (0.06, 0.11)**		
	**Random**			**0.09 (0.05, 0.12)**		

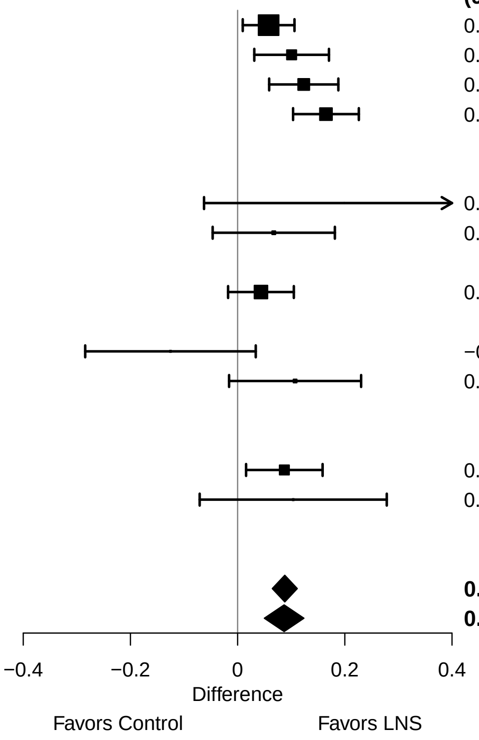

Supplemental figure 3P: Small head size prevalence ratio

Country	Trial	LNS N	Control N	PR (95% CI)	Fixed W	Random W
Bangladesh	JiVitA−4 (34)	2885	1267	1.03 (0.90, 1.18)	0.13	0.13
Bangladesh	RDNS (35)	1663	815	0.90 (0.82, 0.98)	0.33	0.33
Bangladesh	WASH−B (36)	1163	3463	0.90 (0.82, 1.00)	0.24	0.24
Burkina Faso	iLiNS−Zinc (37)	1946	664	0.89 (0.78, 1.01)	0.14	0.14
Burkina Faso	PROMIS (38)					
Burkina Faso	PROMIS CS (38)					
Ghana	GHANA (39)					
Ghana	iLiNS−DYAD−G (40)	347	692	0.88 (0.65, 1.20)	0.03	0.03
Haiti	HAITI (41)					
Kenya	WASH−B (42)	1461	5176	0.92 (0.67, 1.25)	0.03	0.03
Madagascar	MAHAY (43)					
Malawi	iLiNS−DYAD−M (44)	220	444	0.88 (0.54, 1.44)	0.01	0.01
Malawi	iLiNS−DOSE (45)	692	238	0.79 (0.60, 1.03)	0.04	0.04
Mali	PROMIS (46)					
Mali	PROMIS CS (46)					
Zimbabwe	SHINE (HIV−) (47)	1871	1783	0.81 (0.63, 1.05)	0.04	0.04
Zimbabwe	SHINE (HIV+) (48)	337	329	0.98 (0.61, 1.57)	0.01	0.01
	**I^2 = 0.00, Tau^2 = 0.00**	**12585**	**14871**			
	**Fixed**			**0.91 (0.86, 0.95)**		
	**Random**			**0.91 (0.86, 0.95)**		

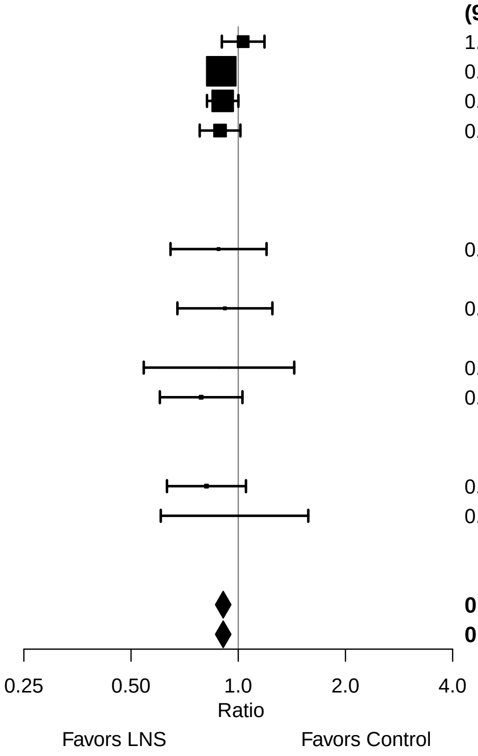

Supplemental figure 3Q: Small head size prevalence difference

Country	Trial	LNS N	Control N	PD (95% CI)	Fixed W	Random W
Bangladesh	JiVitA−4 (34)	2885	1267	0.00 (−0.03, 0.02)	0.10	0.14
Bangladesh	RDNS (35)	1663	815	−0.04 (−0.09, 0.00)	0.03	0.06
Bangladesh	WASH−B (36)	1163	3463	−0.03 (−0.06, 0.00)	0.06	0.09
Burkina Faso	iLiNS−Zinc (37)	1946	664	−0.04 (−0.08, −0.01)	0.05	0.09
Burkina Faso	PROMIS (38)					
Burkina Faso	PROMIS CS (38)					
Ghana	GHANA (39)					
Ghana	iLiNS−DYAD−G (40)	347	692	−0.02 (−0.06, 0.03)	0.03	0.05
Haiti	HAITI (41)					
Kenya	WASH−B (42)	1461	5176	0.00 (−0.02, 0.01)	0.41	0.24
Madagascar	MAHAY (43)					
Malawi	iLiNS−DYAD−M (44)	220	444	−0.01 (−0.06, 0.04)	0.02	0.05
Malawi	iLiNS−DOSE (45)	692	238	−0.04 (−0.10, 0.01)	0.02	0.04
Mali	PROMIS (46)					
Mali	PROMIS CS (46)					
Zimbabwe	SHINE (HIV−) (47)	1871	1783	−0.01 (−0.03, 0.00)	0.26	0.21
Zimbabwe	SHINE (HIV+) (48)	337	329	0.00 (−0.05, 0.04)	0.03	0.05
	**I^2 = 0.23, Tau^2 = 0.00**	**12585**	**14871**			
	**Fixed**			**−0.01 (−0.02, 0.00)**		
	**Random**			**−0.02 (−0.03, 0.00)**		

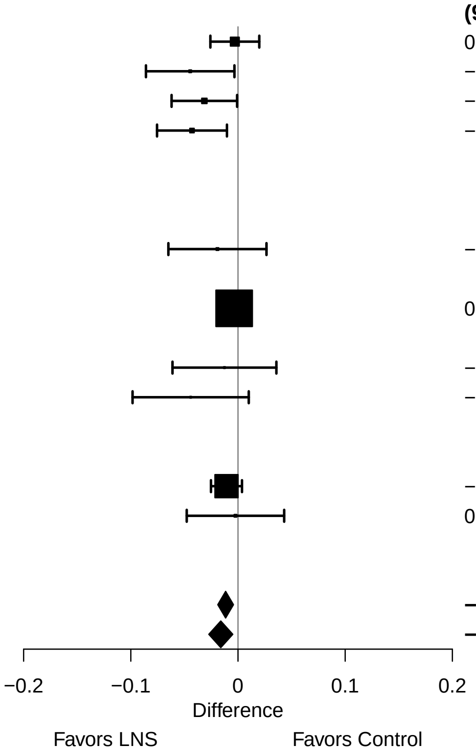

Supplement: nqab278_Supplemental_Files [file nqab278_supplemental_files.zip › 7_SQ-LNS_IPD_growth_Supplemental_Figure_3.pdf]
