# Supplementary material for: Characteristics that modify the effect of small-quantity lipid-based nutrient supplementation on child growth: an individual participant data meta-analysis of randomized controlled trials
Source: Am J Clin Nutr. 2021 Sep 29;114(Suppl 1):15S–42S. doi: 10.1093/ajcn/nqab278 (PMC8560308; doi:10.1093/ajcn/nqab278)

Supplemental figure 4: Forest plots for effects of SQ-LNS on growth outcomes stratified by study implementation within an existing program vs not program-based

Contents

Supplemental figure 4A: Stunting prevalence ratio stratified by Study implementation **2**

Supplemental figure 4B: Wasting prevalence ratio stratified by Study implementation **3**

These figures are forest plots showing intervention effects stratified by study implementation. Each figure shows the study-level estimates along with the corresponding pooled estimate grouped by category. For dichotomous outcomes analyzed via prevalence ratios, the effect estimate is the prevalence in the LNS group divided by the prevalence in the control group. The labels on the left y-axis correspond to trial level information. The values on the right indicate the study level effect estimate, confidence interval, and weighting for deriving the pooled estimates.

Supplemental figure 4A: Stunting prevalence ratio stratified by Study implementation

Country	Trial	N	N	PR (95% CI)	W
**Study implementation − Program−based**					
Bangladesh	RDNS (35)	1663	815	0.92 (0.85, 1.00)	0.20
Burkina Faso	PROMIS (38)	863	914	0.82 (0.67, 1.00)	0.09
Burkina Faso	PROMIS CS (38)	430	439	0.87 (0.63, 1.19)	0.04
Haiti	HAITI (41)	149	149	1.04 (0.60, 1.81)	0.02
Madagascar	MAHAY (43)	1702	1682	0.98 (0.90, 1.07)	0.19
Mali	PROMIS (46)	506	506	0.92 (0.76, 1.10)	0.09
Mali	PROMIS CS (46)	952	969	0.78 (0.65, 0.95)	0.09
Zimbabwe	SHINE (HIV−) (47)	1880	1794	0.79 (0.72, 0.87)	0.18
Zimbabwe	SHINE (HIV+) (48)	337	330	0.81 (0.68, 0.96)	0.10
**I² = 0.47, Tau² = 0.00**		**8482**	**7598**	**0.88 (0.82, 0.93)**	
**Study implementation − Not program−based**					
Bangladesh	JiVitA−4 (34)	2838	1244	0.92 (0.84, 1.01)	0.20
Bangladesh	WASH−B (36)	1158	3431	0.84 (0.77, 0.91)	0.21
Burkina Faso	iLiNS−Zinc (37)	1952	664	0.75 (0.65, 0.87)	0.13
Ghana	GHANA (39)	98	96	0.70 (0.23, 2.13)	0.00
Ghana	iLiNS−DYAD−G (40)	347	692	0.66 (0.45, 0.98)	0.03
Kenya	WASH−B (42)	1457	5137	0.86 (0.78, 0.94)	0.20
Malawi	iLiNS−DYAD−M (44)	220	444	1.10 (0.89, 1.36)	0.08
Malawi	iLiNS−DOSE (45)	696	241	1.00 (0.87, 1.15)	0.14
**I² = 0.58, Tau² = 0.01**		**8766**	**11949**	**0.88 (0.80, 0.97)**	

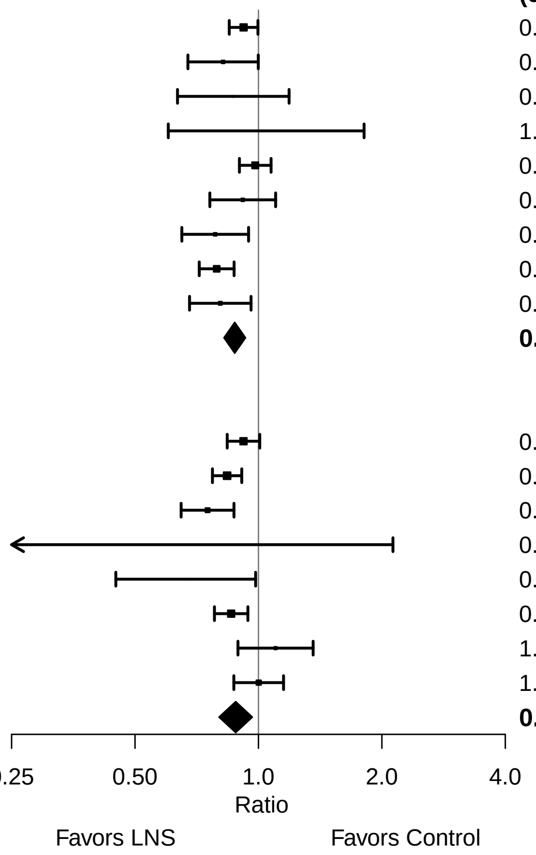

Supplemental figure 4B: Wasting prevalence ratio stratified by Study implementation

Country	Trial	N	N	PR (95% CI)	W
**Study implementation − Program−based**					
Bangladesh	RDNS (35)	1661	815	0.87 (0.70, 1.07)	0.33
Burkina Faso	PROMIS (38)	856	907	0.86 (0.59, 1.27)	0.11
Burkina Faso	PROMIS CS (38)	430	436	1.03 (0.72, 1.49)	0.11
Haiti	HAITI (41)	148	149	0.94 (0.13, 6.52)	0.00
Madagascar	MAHAY (43)	1700	1682	0.96 (0.67, 1.38)	0.12
Mali	PROMIS (46)	499	502	0.61 (0.15, 2.42)	0.01
Mali	PROMIS CS (46)	944	959	0.83 (0.63, 1.10)	0.19
Zimbabwe	SHINE (HIV−) (47)	1869	1784	0.89 (0.60, 1.34)	0.09
Zimbabwe	SHINE (HIV+) (48)	336	328	1.38 (0.65, 2.96)	0.03
**I^2 = 0.00, Tau^2 = 0.00**		**8443**	**7562**	**0.90 (0.79, 1.02)**	
**Study implementation − Not program−based**					
Bangladesh	JiVitA−4 (34)	2783	1217	0.91 (0.78, 1.05)	0.44
Bangladesh	WASH−B (36)	1156	3424	0.79 (0.65, 0.96)	0.25
Burkina Faso	iLiNS−Zinc (37)	1952	664	0.69 (0.54, 0.87)	0.17
Ghana	GHANA (39)	98	96	0.87 (0.35, 2.16)	0.01
Ghana	iLiNS−DYAD−G (40)	347	692	0.84 (0.52, 1.37)	0.04
Kenya	WASH−B (42)	1455	5118	0.88 (0.53, 1.45)	0.04
Malawi	iLiNS−DYAD−M (44)	220	444	0.94 (0.39, 2.28)	0.01
Malawi	iLiNS−DOSE (45)	696	241	1.37 (0.78, 2.40)	0.03
**I^2 = 0.04, Tau^2 = 0.00**		**8707**	**11896**	**0.84 (0.76, 0.93)**	

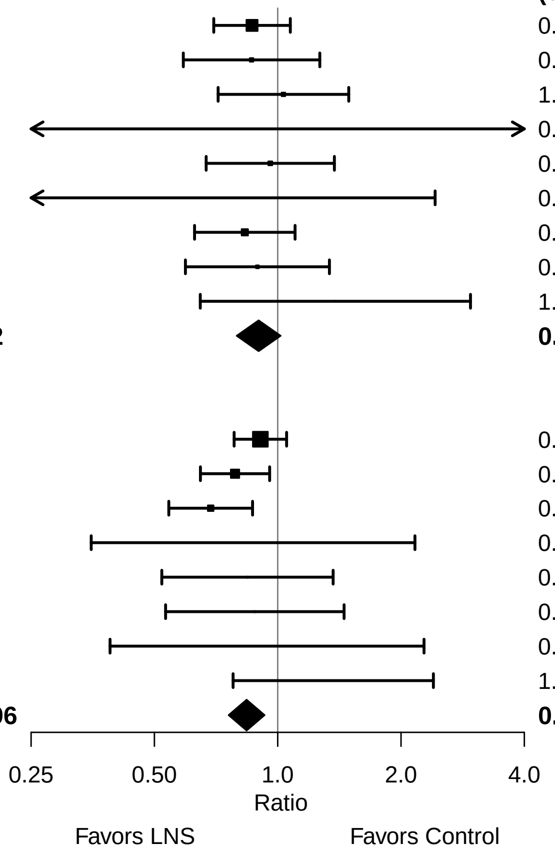

Supplement: nqab278_Supplemental_Files [file nqab278_supplemental_files.zip › 8_SQ-LNS_IPD_growth_Supplemental_Figure_4.pdf]
