# Supplementary material for: Characteristics that modify the effect of small-quantity lipid-based nutrient supplementation on child growth: an individual participant data meta-analysis of randomized controlled trials
Source: Am J Clin Nutr. 2021 Sep 29;114(Suppl 1):15S–42S. doi: 10.1093/ajcn/nqab278 (PMC8560308; doi:10.1093/ajcn/nqab278)

Supplemental figure 5: Forest plots for effects of SQ-LNS on growth outcomes stratified by extent of social and behavioral change communication (SBCC) for infant and young child feeding (IYCF) provided in the study

Contents

Supplemental figure 5A: Stunting prevalence ratio stratified by Study SBCC for IYCF 2

Supplemental figure 5B: Wasting prevalence ratio stratified by Study SBCC for IYCF 3

These figures are forest plots showing intervention effects by study SBCC for IYCF. Each figure shows the study-level estimates along with the corresponding pooled estimate grouped by category. For dichotomous outcomes analyzed via prevalence ratios, the effect estimate is the prevalence in the LNS group divided by the prevalence in the control group. The labels on the left y-axis correspond to trial level information. The values on the right indicate the study level effect estimate, confidence interval, and weighting for deriving the pooled estimates.

Supplemental figure 5A: Stunting prevalence ratio stratified by Study SBCC for IYCF

Country	Trial	N	N	PR (95% CI)	W
**Study SBCC for IYCF − Minimal**					
Bangladesh	RDNS (35)	1663	815	0.92 (0.85, 1.00)	0.35
Burkina Faso	iLiNS−Zinc (37)	1952	664	0.75 (0.65, 0.87)	0.21
Ghana	GHANA (39)	98	96	0.70 (0.23, 2.13)	0.01
Ghana	iLiNS−DYAD−G (40)	347	692	0.66 (0.45, 0.98)	0.05
Haiti	HAITI (41)	149	149	1.04 (0.60, 1.81)	0.03
Malawi	iLiNS−DYAD−M (44)	220	444	1.10 (0.89, 1.36)	0.13
Malawi	iLiNS−DOSE (45)	696	241	1.00 (0.87, 1.15)	0.23
**I² = 0.58, Tau² = 0.01**		**5125**	**3101**	**0.90 (0.80, 1.03)**	
**Study SBCC for IYCF − Expanded in LNS arm(s) only**					
Burkina Faso	PROMIS (38)	863	914	0.82 (0.67, 1.00)	0.11
Burkina Faso	PROMIS CS (38)	430	439	0.87 (0.63, 1.19)	0.05
Kenya	WASH−B (42)	1457	5137	0.86 (0.78, 0.94)	0.23
Zimbabwe	SHINE (HIV−) (47)	1880	1794	0.79 (0.72, 0.87)	0.23
Zimbabwe	SHINE (HIV+) (48)	337	330	0.81 (0.68, 0.96)	0.13
**I² = 0.00, Tau² = 0.00**		**6125**	**12045**	**0.83 (0.79, 0.87)**	
**Study SBCC for IYCF − Expanded in LNS & control arms**					
Bangladesh	JiVitA−4 (34)	2838	1244	0.92 (0.84, 1.01)	0.36
Madagascar	MAHAY (43)	1702	866	0.98 (0.87, 1.10)	0.29
Mali	PROMIS (46)	506	506	0.92 (0.76, 1.10)	0.18
Mali	PROMIS CS (46)	952	969	0.78 (0.65, 0.95)	0.17
**I² = 0.19, Tau² = 0.00**		**5998**	**3585**	**0.91 (0.85, 0.99)**	

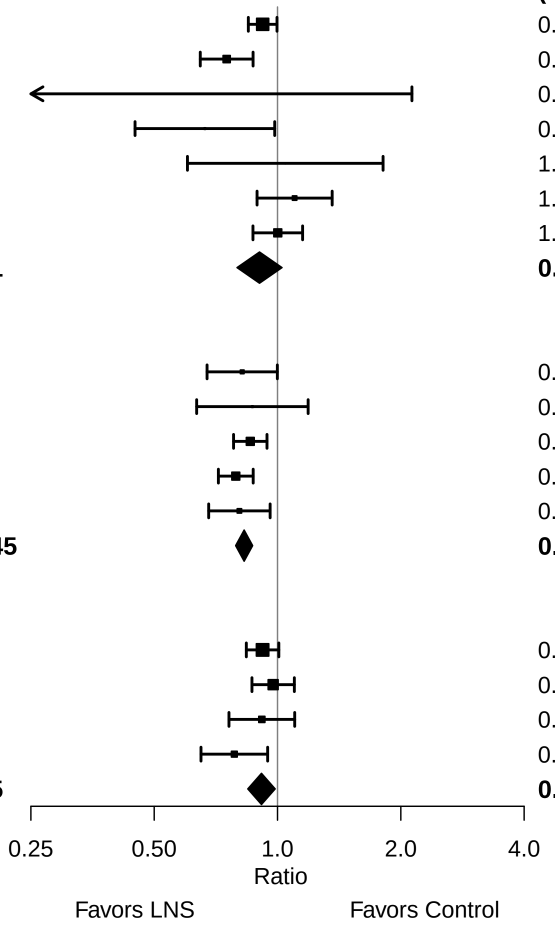

Supplemental figure 5B: Wasting prevalence ratio stratified by Study SBCC for IYCF

Country	Trial	N	N	PR (95% CI)	W
**Study SBCC for IYCF − Minimal**					
Bangladesh	RDNS (35)	1661	815	0.87 (0.70, 1.07)	0.43
Burkina Faso	iLiNS−Zinc (37)	1952	664	0.69 (0.54, 0.87)	0.36
Ghana	GHANA (39)	98	96	0.87 (0.35, 2.16)	0.02
Ghana	iLiNS−DYAD−G (40)	347	692	0.84 (0.52, 1.37)	0.09
Haiti	HAITI (41)	148	149	0.94 (0.13, 6.52)	0.01
Malawi	iLiNS−DYAD−M (44)	220	444	0.94 (0.39, 2.28)	0.03
Malawi	iLiNS−DOSE (45)	696	241	1.37 (0.78, 2.40)	0.06
**I² = 0.00, Tau² = 0.00**		**5122**	**3101**	**0.82 (0.71, 0.94)**	
**Study SBCC for IYCF − Expanded in LNS arm(s) only**					
Burkina Faso	PROMIS (38)	856	907	0.86 (0.59, 1.27)	0.13
Burkina Faso	PROMIS CS (38)	430	436	1.03 (0.72, 1.49)	0.14
Kenya	WASH−B (42)	1455	5118	0.88 (0.53, 1.45)	0.08
Zimbabwe	SHINE (HIV−) (47)	1869	1784	0.89 (0.60, 1.34)	0.12
Zimbabwe	SHINE (HIV+) (48)	336	328	1.38 (0.65, 2.96)	0.03
**I² = 0.00, Tau² = 0.00**		**6102**	**11997**	**0.86 (0.75, 0.99)**	
**Study SBCC for IYCF − Expanded in LNS & control arms**					
Bangladesh	JiVitA−4 (34)	2783	1217	0.91 (0.78, 1.05)	0.72
Madagascar	MAHAY (43)	1700	866	0.93 (0.59, 1.46)	0.08
Mali	PROMIS (46)	499	502	0.61 (0.15, 2.42)	0.01
Mali	PROMIS CS (46)	944	959	0.83 (0.63, 1.10)	0.20
**I² = 0.00, Tau² = 0.00**		**5926**	**3544**	**0.89 (0.79, 1.01)**	

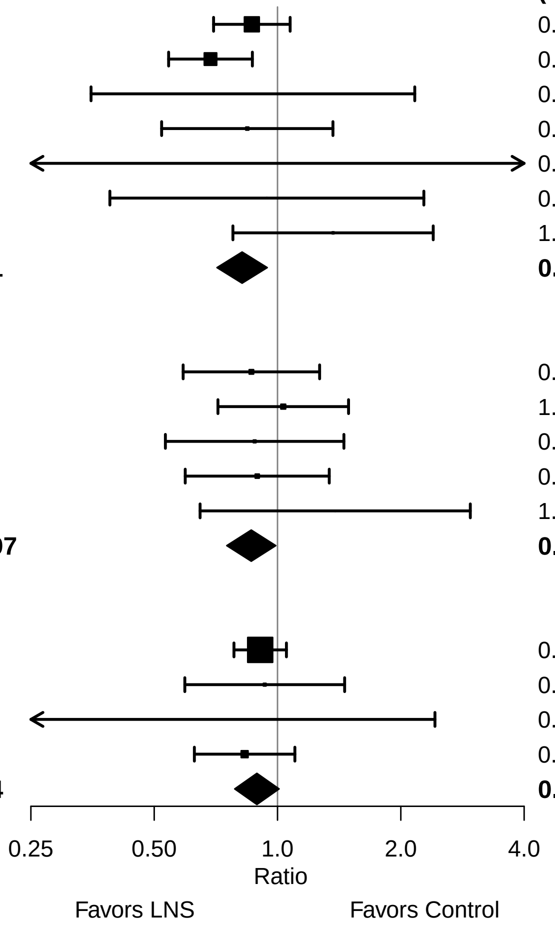

Supplement: nqab278_Supplemental_Files [file nqab278_supplemental_files.zip › 9_SQ-LNS_IPD_growth_Supplemental_Figure_5.pdf]
