# Supplementary material for: Characteristics that modify the effect of small-quantity lipid-based nutrient supplementation on child growth: an individual participant data meta-analysis of randomized controlled trials
Source: Am J Clin Nutr. 2021 Sep 29;114(Suppl 1):15S–42S. doi: 10.1093/ajcn/nqab278 (PMC8560308; doi:10.1093/ajcn/nqab278)

Supplemental figure 6: Forest plots for effects of SQ-LNS on growth outcomes stratified by study-level effect modifiers

Contents

Supplemental figure 6A: Mean difference in LAZ **6**
6A1: Stratified by Geographic region . . . 6
6A2: Stratified by Stunting burden . . . 7
6A3: Stratified by Malaria prevalence . . . 8
6A4: Stratified by Source water quality . . . 9
6A5: Stratified by Sanitation . . . 10
6A6: Stratified by Supplement duration . . . 11
6A7: Stratified by Frequency of contact . . . 12
6A8: Stratified by Average SQ-LNS compliance . . . 13

Supplemental figure 6B: Stunting prevalence ratio **14**
6B1: Stratified by Geographic region . . . 14
6B2: Stratified by Stunting burden . . . 15
6B3: Stratified by Malaria prevalence . . . 16
6B4: Stratified by Source water quality . . . 17
6B5: Stratified by Sanitation . . . 18
6B6: Stratified by Supplement duration . . . 19
6B7: Stratified by Frequency of contact . . . 20
6B8: Stratified by Average SQ-LNS compliance . . . 21

Supplemental figure 6C: Stunting prevalence difference **22**
6C1: Stratified by Geographic region . . . 22
6C2: Stratified by Stunting burden . . . 23
6C3: Stratified by Malaria prevalence . . . 24
6C4: Stratified by Source water quality . . . 25
6C5: Stratified by Sanitation . . . 26
6C6: Stratified by Supplement duration . . . 27
6C7: Stratified by Frequency of contact . . . 28
6C8: Stratified by Average SQ-LNS compliance . . . 29

Supplemental figure 6D: Mean difference in WLZ **30**
6D1: Stratified by Geographic region . . . 30
6D2: Stratified by Stunting burden . . . 31
6D3: Stratified by Malaria prevalence . . . 32
6D4: Stratified by Source water quality . . . 33
6D5: Stratified by Sanitation . . . 34
6D6: Stratified by Supplement duration . . . 35
6D7: Stratified by Frequency of contact . . . 36
6D8: Stratified by Average SQ-LNS compliance . . . 37

Supplemental figure 6E: Wasting prevalence ratio **38**
6E1: Stratified by Geographic region . . . 38
6E2: Stratified by Stunting burden . . . 39
6E3: Stratified by Malaria prevalence . . . 40
6E4: Stratified by Source water quality . . . 41
6E5: Stratified by Sanitation . . . 42
6E6: Stratified by Supplement duration . . . 43
6E7: Stratified by Frequency of contact . . . 44
6E8: Stratified by Average SQ-LNS compliance . . . 45

Supplemental figure 6F: Wasting prevalence difference **46**
6F1: Stratified by Geographic region . . . 46
6F2: Stratified by Stunting burden . . . 47
6F3: Stratified by Malaria prevalence . . . 48
6F4: Stratified by Source water quality . . . 49
6F5: Stratified by Sanitation . . . 50
6F6: Stratified by Supplement duration . . . 51
6F7: Stratified by Frequency of contact . . . 52
6F8: Stratified by Average SQ-LNS compliance . . . 53

Supplemental figure 6G: Mean difference in MUACZ **54**
6G1: Stratified by Geographic region (insufficient comparisons) . . . 54
6G2: Stratified by Stunting burden . . . 55
6G3: Stratified by Malaria prevalence . . . 56
6G4: Stratified by Source water quality . . . 57
6G5: Stratified by Sanitation . . . 58
6G6: Stratified by Supplement duration . . . 59
6G7: Stratified by Frequency of contact . . . 60
6G8: Stratified by Average SQ-LNS compliance . . . 61

Supplemental figure 6H: Low MUAC prevalence ratio **62**
6H1: Stratified by Geographic region (insufficient comparisons) . . . 62

6H2: Stratified by Stunting burden . . . 63
6H3: Stratified by Malaria prevalence . . . 64
6H4: Stratified by Source water quality . . . 65
6H5: Stratified by Sanitation . . . 66
6H6: Stratified by Supplement duration . . . 67
6H7: Stratified by Frequency of contact . . . 68
6H8: Stratified by Average SQ-LNS compliance . . . 69

Supplemental figure 6I: Low MUAC prevalence difference **70**
6I1: Stratified by Geographic region (insufficient comparisons) . . . 70
6I2: Stratified by Stunting burden . . . 71
6I3: Stratified by Malaria prevalence . . . 72
6I4: Stratified by Source water quality . . . 73
6I5: Stratified by Sanitation . . . 74
6I6: Stratified by Supplement duration . . . 75
6I7: Stratified by Frequency of contact . . . 76
6I8: Stratified by Average SQ-LNS compliance . . . 77

Supplemental figure 6J: Acute malnutrition prevalence ratio **78**
6J1: Stratified by Geographic region (insufficient comparisons) . . . 78
6J2: Stratified by Stunting burden . . . 79
6J3: Stratified by Malaria prevalence . . . 80
6J4: Stratified by Source water quality . . . 81
6J5: Stratified by Sanitation . . . 82
6J6: Stratified by Supplement duration . . . 83
6J7: Stratified by Frequency of contact . . . 84
6J8: Stratified by Average SQ-LNS compliance . . . 85

Supplemental figure 6K: Acute malnutrition prevalence difference **86**
6K1: Stratified by Geographic region (insufficient comparisons) . . . 86
6K2: Stratified by Stunting burden . . . 87
6K3: Stratified by Malaria prevalence . . . 88
6K4: Stratified by Source water quality . . . 89
6K5: Stratified by Sanitation . . . 90
6K6: Stratified by Supplement duration . . . 91
6K7: Stratified by Frequency of contact . . . 92
6K8: Stratified by Average SQ-LNS compliance . . . 93

Supplemental figure 6L: Mean difference in WAZ **94**
6L1: Stratified by Geographic region . . . 94
6L2: Stratified by Stunting burden . . . 95
6L3: Stratified by Malaria prevalence . . . 96

6L4: Stratified by Source water quality . . . 97
6L5: Stratified by Sanitation . . . 98
6L6: Stratified by Supplement duration . . . 99
6L7: Stratified by Frequency of contact . . . 100
6L8: Stratified by Average SQ-LNS compliance . . . 101

Supplemental figure 6M: Underweight prevalence ratio 102
6M1: Stratified by Geographic region . . . 102
6M2: Stratified by Stunting burden . . . 103
6M3: Stratified by Malaria prevalence . . . 104
6M4: Stratified by Source water quality . . . 105
6M5: Stratified by Sanitation . . . 106
6M6: Stratified by Supplement duration . . . 107
6M7: Stratified by Frequency of contact . . . 108
6M8: Stratified by Average SQ-LNS compliance . . . 109

Supplemental figure 6N: Underweight prevalence difference 110
6N1: Stratified by Geographic region . . . 110
6N2: Stratified by Stunting burden . . . 111
6N3: Stratified by Malaria prevalence . . . 112
6N4: Stratified by Source water quality . . . 113
6N5: Stratified by Sanitation . . . 114
6N6: Stratified by Supplement duration . . . 115
6N7: Stratified by Frequency of contact . . . 116
6N8: Stratified by Average SQ-LNS compliance . . . 117

Supplemental figure 6O: Mean difference in HCZ 118
6O1: Stratified by Geographic region . . . 118
6O2: Stratified by Stunting burden . . . 119
6O3: Stratified by Malaria prevalence . . . 120
6O4: Stratified by Source water quality . . . 121
6O5: Stratified by Sanitation . . . 122
6O6: Stratified by Supplement duration . . . 123
6O7: Stratified by Frequency of contact . . . 124
6O8: Stratified by Average SQ-LNS compliance . . . 125

Supplemental figure 6P: Small head size prevalence ratio 126
6P1: Stratified by Geographic region . . . 126
6P2: Stratified by Stunting burden . . . 127
6P3: Stratified by Malaria prevalence . . . 128
6P4: Stratified by Source water quality . . . 129
6P5: Stratified by Sanitation . . . 130

6P6: Stratified by Supplement duration . . . 131
6P7: Stratified by Frequency of contact . . . 132
6P8: Stratified by Average SQ-LNS compliance . . . 133

Supplemental figure 6Q: Small head size prevalence difference **134**
6Q1: Stratified by Geographic region . . . 134
6Q2: Stratified by Stunting burden . . . 135
6Q3: Stratified by Malaria prevalence . . . 136
6Q4: Stratified by Source water quality . . . 137
6Q5: Stratified by Sanitation . . . 138
6Q6: Stratified by Supplement duration . . . 139
6Q7: Stratified by Frequency of contact . . . 140
6Q8: Stratified by Average SQ-LNS compliance . . . 141

These figures are forest plots showing the study-level effect modification of intervention effects. Each figure shows the study-level estimates along with the corresponding pooled estimate grouped by study-level effect modifier category. For definitions of effect modifiers, see Box 1 in the main paper.

Individual study estimates were generated from log-binomial regression for dichotomous outcomes and simple linear regression for continuous outcomes; controlling for baseline measure when available and with clustered observations using robust standard errors for cluster-randomized trials. Pooled sub-group estimates and statistical testing of the pooled interaction term were generated using inverse-variance weighting fixed effects. P-value for the difference was estimated using random effects meta-regression with the indicated effect modifier as the predictor of intervention effect size; stratified pooled estimates are presented for each strata. For dichotomous outcomes analyzed via prevalence ratios, the effect estimate is the prevalence in the LNS group divided by the prevalence in the control group. For dichotomous outcomes analyzed via prevalence differences, the effect estimate is the prevalence in the LNS group minus the prevalence in the control group.

The labels on the left y-axis correspond to trial level information. The values on the right indicate the study level effect estimate, confidence interval, and weighting for deriving the pooled estimates. LAZ, length-for-age z-score; WLZ, weight-for-length z-score; WAZ, weight-for-age z-score; MUACZ, mid-upper arm circumference z-score; HCZ, head circumference-for-age z-score.

Supplemental figure 6A: Mean difference in LAZ

6A1: Stratified by Geographic region

Geographic region
(p−diff = 0.636)

Country	Trial	N	N	MD (95% CI)	W
**Geographic region − SEAR**					
Bangladesh	JiVitA−4 (34)	2838	1244	0.09 (0.03, 0.14)	0.36
Bangladesh	RDNS (35)	1663	815	0.08 (−0.01, 0.16)	0.30
Bangladesh	WASH−B (36)	1158	3431	0.21 (0.14, 0.28)	0.33
**I² = 0.78, Tau² = 0.00**		**5659**	**5490**	**0.12 (0.04, 0.21)**	
**Geographic region − AFR**					
Burkina Faso	iLiNS−Zinc (37)	1952	664	0.31 (0.22, 0.39)	0.11
Burkina Faso	PROMIS (38)	863	914	0.15 (0.02, 0.27)	0.08
Burkina Faso	PROMIS CS (38)	430	439	0.04 (−0.14, 0.22)	0.05
Ghana	GHANA (39)	98	96	0.28 (−0.02, 0.58)	0.03
Ghana	iLiNS−DYAD−G (40)	347	692	0.20 (0.07, 0.33)	0.08
Kenya	WASH−B (42)	1457	5137	0.15 (0.09, 0.21)	0.12
Madagascar	MAHAY (43)	1702	1682	0.03 (−0.11, 0.17)	0.07
Malawi	iLiNS−DYAD−M (44)	220	444	−0.08 (−0.25, 0.10)	0.06
Malawi	iLiNS−DOSE (45)	696	241	0.06 (−0.04, 0.17)	0.10
Mali	PROMIS (46)	506	506	0.16 (0.01, 0.31)	0.07
Mali	PROMIS CS (46)	952	969	0.22 (0.05, 0.39)	0.06
Zimbabwe	SHINE (HIV−) (47)	1880	1794	0.16 (0.08, 0.23)	0.11
Zimbabwe	SHINE (HIV+) (48)	337	330	0.25 (0.08, 0.43)	0.06
**I² = 0.60, Tau² = 0.01**		**11440**	**13908**	**0.15 (0.09, 0.21)**	

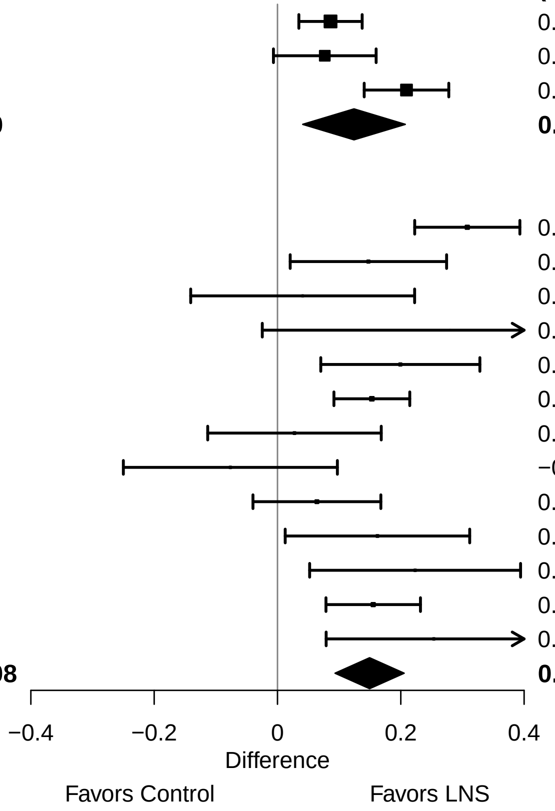

Supplemental figure 6A: Mean difference in LAZ

6A2: Stratified by Stunting burden

Stunting burden
(p−diff = 0.309)

Country	Trial	N	N	MD (95% CI)	W
**Stunting burden − Less than 35%**					
Burkina Faso	PROMIS (38)	863	914	0.15 (0.02, 0.27)	0.16
Burkina Faso	PROMIS CS (38)	430	439	0.04 (−0.14, 0.22)	0.11
Ghana	GHANA (39)	98	96	0.28 (−0.02, 0.58)	0.05
Ghana	iLiNS−DYAD−G (40)	347	692	0.20 (0.07, 0.33)	0.16
Haiti	HAITI (41)	149	149	−0.01 (−0.14, 0.13)	0.15
Kenya	WASH−B (42)	1457	5137	0.15 (0.09, 0.21)	0.24
Malawi	iLiNS−DYAD−M (44)	220	444	−0.08 (−0.25, 0.10)	0.12
**I² = 0.53, Tau² = 0.01**		**3564**	**7871**	**0.10 (0.02, 0.18)**	
**Stunting burden − More than 35%**					
Bangladesh	JiVitA−4 (34)	2838	1244	0.09 (0.03, 0.14)	0.14
Bangladesh	RDNS (35)	1663	815	0.08 (−0.01, 0.16)	0.12
Bangladesh	WASH−B (36)	1158	3431	0.21 (0.14, 0.28)	0.13
Burkina Faso	iLiNS−Zinc (37)	1952	664	0.31 (0.22, 0.39)	0.11
Madagascar	MAHAY (43)	1702	1682	0.03 (−0.11, 0.17)	0.08
Malawi	iLiNS−DOSE (45)	696	241	0.06 (−0.04, 0.17)	0.10
Mali	PROMIS (46)	506	506	0.16 (0.01, 0.31)	0.08
Mali	PROMIS CS (46)	952	969	0.22 (0.05, 0.39)	0.07
Zimbabwe	SHINE (HIV−) (47)	1880	1794	0.16 (0.08, 0.23)	0.12
Zimbabwe	SHINE (HIV+) (48)	337	330	0.25 (0.08, 0.43)	0.06
**I² = 0.72, Tau² = 0.01**		**13684**	**11676**	**0.15 (0.10, 0.21)**	

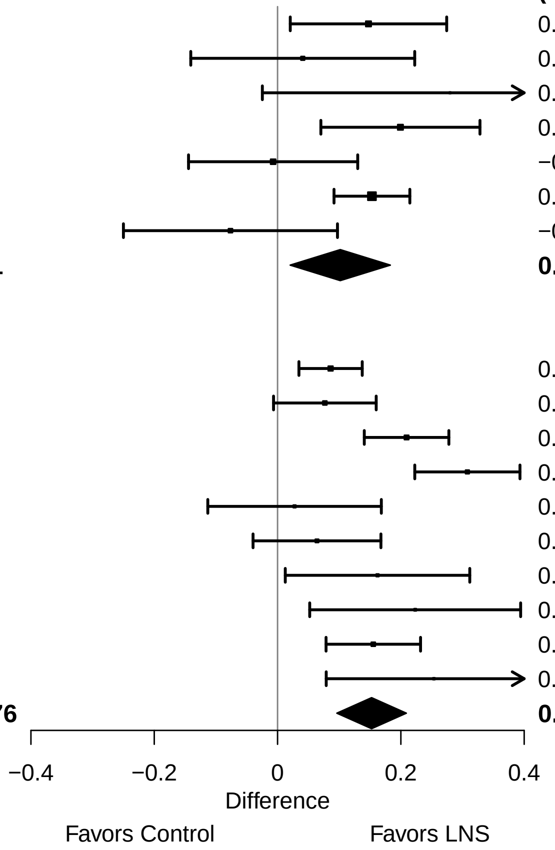

Supplemental figure 6A: Mean difference in LAZ

6A3: Stratified by Malaria prevalence

Malaria prevalence
(p−diff = 0.508)

Country	Trial	N	N	MD (95% CI)	W
**Malaria prevalence − Less than 10%**					
Bangladesh	JiVitA−4 (34)	2838	1244	0.09 (0.03, 0.14)	0.16
Bangladesh	RDNS (35)	1663	815	0.08 (−0.01, 0.16)	0.14
Bangladesh	WASH−B (36)	1158	3431	0.21 (0.14, 0.28)	0.15
Haiti	HAITI (41)	149	149	−0.01 (−0.14, 0.13)	0.10
Kenya	WASH−B (42)	1457	5137	0.15 (0.09, 0.21)	0.15
Madagascar	MAHAY (43)	1702	1682	0.03 (−0.11, 0.17)	0.09
Zimbabwe	SHINE (HIV−) (47)	1880	1794	0.16 (0.08, 0.23)	0.14
Zimbabwe	SHINE (HIV+) (48)	337	330	0.25 (0.08, 0.43)	0.07
**I^2 = 0.62, Tau^2 = 0.00**		**11184**	**14582**	**0.12 (0.07, 0.18)**	
**Malaria prevalence − At least 10%**					
Burkina Faso	iLiNS−Zinc (37)	1952	664	0.31 (0.22, 0.39)	0.17
Burkina Faso	PROMIS (38)	863	914	0.15 (0.02, 0.27)	0.13
Burkina Faso	PROMIS CS (38)	430	439	0.04 (−0.14, 0.22)	0.09
Ghana	GHANA (39)	98	96	0.28 (−0.02, 0.58)	0.04
Ghana	iLiNS−DYAD−G (40)	347	692	0.20 (0.07, 0.33)	0.13
Malawi	iLiNS−DYAD−M (44)	220	444	−0.08 (−0.25, 0.10)	0.09
Malawi	iLiNS−DOSE (45)	696	241	0.06 (−0.04, 0.17)	0.15
Mali	PROMIS (46)	506	506	0.16 (0.01, 0.31)	0.11
Mali	PROMIS CS (46)	952	969	0.22 (0.05, 0.39)	0.10
**I^2 = 0.68, Tau^2 = 0.01**		**6064**	**4965**	**0.15 (0.07, 0.23)**	

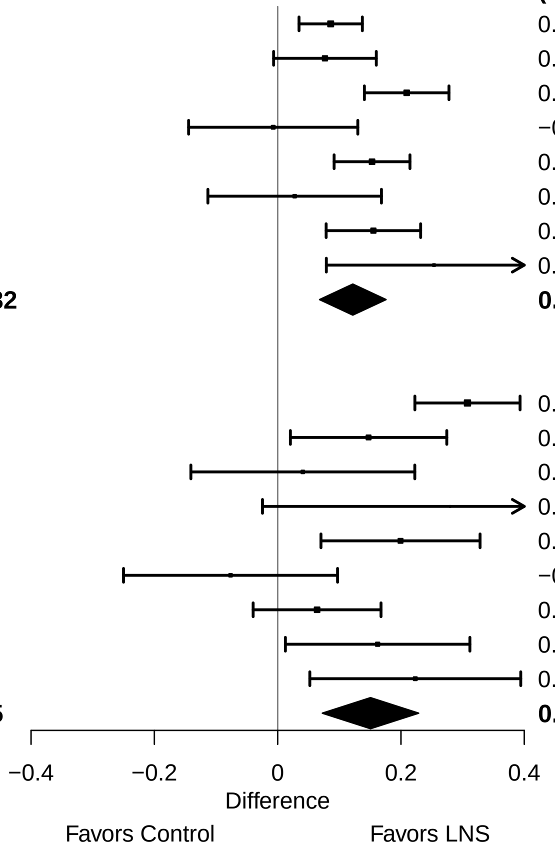

Supplemental figure 6A: Mean difference in LAZ

6A4: Stratified by Source water quality

Source water quality
(p−diff = 0.313)

Country	Trial	N	N	MD (95% CI)	W
**Source water quality − Improved**					
Bangladesh	JiVitA−4 (34)	2838	1244	0.09 (0.03, 0.14)	0.19
Bangladesh	RDNS (35)	1663	815	0.08 (−0.01, 0.16)	0.16
Bangladesh	WASH−B (36)	567	1104	0.26 (0.16, 0.36)	0.14
Ghana	GHANA (39)	98	96	0.28 (−0.02, 0.58)	0.04
Ghana	iLiNS−DYAD−G (40)	347	692	0.20 (0.07, 0.33)	0.12
Haiti	HAITI (41)	149	149	−0.01 (−0.14, 0.13)	0.11
Malawi	iLiNS−DYAD−M (44)	220	444	−0.08 (−0.25, 0.10)	0.09
Malawi	iLiNS−DOSE (45)	696	241	0.06 (−0.04, 0.17)	0.14
**I² = 0.65, Tau² = 0.01**		**6578**	**4785**	**0.10 (0.02, 0.18)**	
**Source water quality − Unimproved**					
Burkina Faso	iLiNS−Zinc (37)	1952	664	0.31 (0.22, 0.39)	0.16
Burkina Faso	PROMIS (38)	863	914	0.15 (0.02, 0.27)	0.12
Burkina Faso	PROMIS CS (38)	430	439	0.04 (−0.14, 0.22)	0.09
Kenya	WASH−B (42)	695	2253	0.11 (0.00, 0.21)	0.14
Madagascar	MAHAY (43)	1702	1682	0.03 (−0.11, 0.17)	0.11
Mali	PROMIS (46)	506	506	0.16 (0.01, 0.31)	0.11
Mali	PROMIS CS (46)	952	969	0.22 (0.05, 0.39)	0.09
Zimbabwe	SHINE (HIV−) (47)	892	880	0.11 (0.00, 0.21)	0.14
Zimbabwe	SHINE (HIV+) (48)	147	146	0.27 (−0.01, 0.55)	0.05
**I² = 0.59, Tau² = 0.00**		**8139**	**8453**	**0.15 (0.09, 0.22)**	

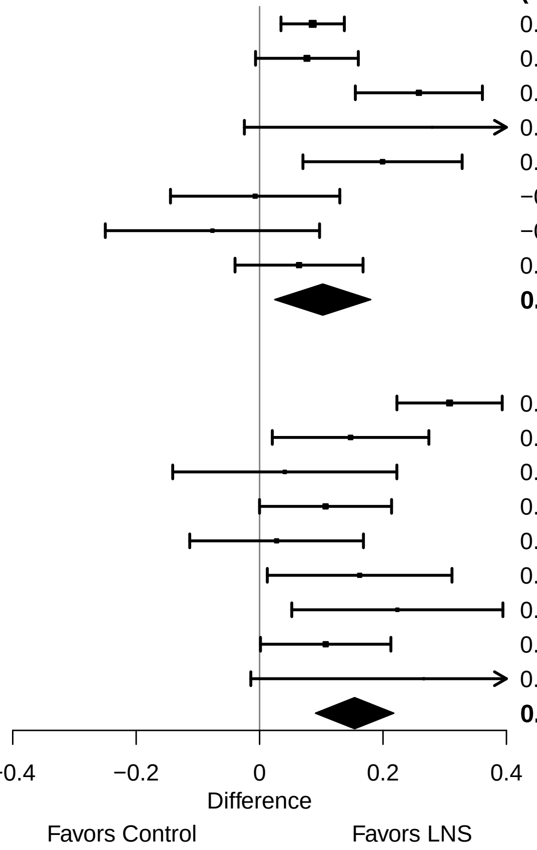

Supplemental figure 6A: Mean difference in LAZ

6A5: Stratified by Sanitation

Sanitation
(p−diff = 0.694)

Country	Trial	N	N	MD (95% CI)	W
**Sanitation − Improved**					
Bangladesh	JiVitA−4 (34)	2838	1244	0.09 (0.03, 0.14)	0.16
Bangladesh	RDNS (35)	1663	815	0.08 (−0.01, 0.16)	0.14
Bangladesh	WASH−B (36)	567	1104	0.26 (0.16, 0.36)	0.12
Burkina Faso	PROMIS (38)	863	914	0.15 (0.02, 0.27)	0.10
Burkina Faso	PROMIS CS (38)	430	439	0.04 (−0.14, 0.22)	0.07
Ghana	GHANA (39)	98	96	0.28 (−0.02, 0.58)	0.04
Ghana	iLiNS−DYAD−G (40)	347	692	0.20 (0.07, 0.33)	0.10
Haiti	HAITI (41)	149	149	−0.01 (−0.14, 0.13)	0.10
Mali	PROMIS (46)	506	506	0.16 (0.01, 0.31)	0.09
Mali	PROMIS CS (46)	952	969	0.22 (0.05, 0.39)	0.08
**I^2 = 0.50, Tau^2 = 0.00**		**8413**	**6928**	**0.13 (0.08, 0.19)**	
**Sanitation − Unimproved**					
Burkina Faso	iLiNS−Zinc (37)	1952	664	0.31 (0.22, 0.39)	0.19
Kenya	WASH−B (42)	695	2253	0.11 (0.00, 0.21)	0.17
Madagascar	MAHAY (43)	1702	1682	0.03 (−0.11, 0.17)	0.14
Malawi	iLiNS−DYAD−M (44)	220	444	−0.08 (−0.25, 0.10)	0.11
Malawi	iLiNS−DOSE (45)	696	241	0.06 (−0.04, 0.17)	0.17
Zimbabwe	SHINE (HIV−) (47)	892	880	0.11 (0.00, 0.21)	0.17
Zimbabwe	SHINE (HIV+) (48)	147	146	0.27 (−0.01, 0.55)	0.06
**I^2 = 0.78, Tau^2 = 0.01**		**6304**	**6310**	**0.11 (0.02, 0.21)**	

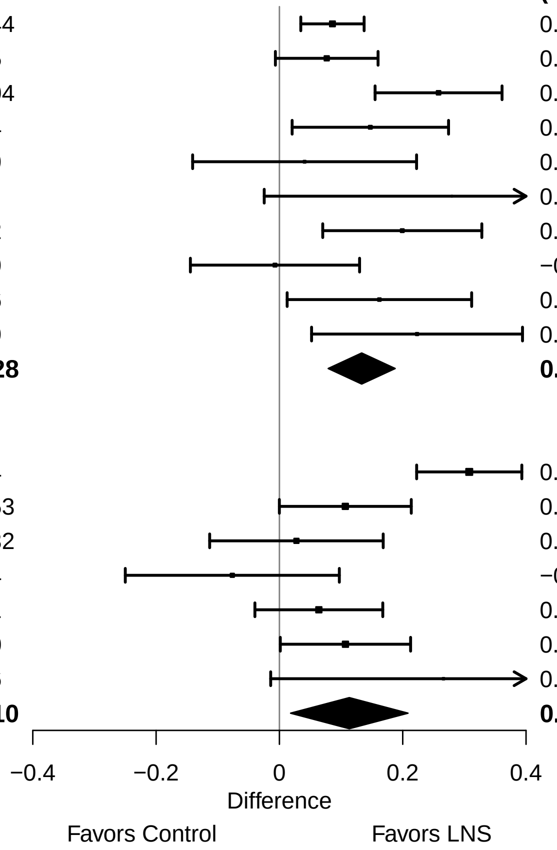

Supplemental figure 6A: Mean difference in LAZ

6A6: Stratified by Supplement duration

Supplement duration
(p−diff = 0.461)

Country	Trial	N	N	MD (95% CI)	W
**Supplement duration − 12m or less**					
Bangladesh	JiVitA−4 (34)	2838	1244	0.09 (0.03, 0.14)	0.13
Burkina Faso	iLiNS−Zinc (37)	1952	664	0.31 (0.22, 0.39)	0.11
Burkina Faso	PROMIS (38)	863	914	0.15 (0.02, 0.27)	0.09
Burkina Faso	PROMIS CS (38)	430	439	0.04 (−0.14, 0.22)	0.06
Ghana	GHANA (39)	98	96	0.28 (−0.02, 0.58)	0.03
Ghana	iLiNS−DYAD−G (40)	347	692	0.20 (0.07, 0.33)	0.08
Haiti	HAITI (41)	149	149	−0.01 (−0.14, 0.13)	0.08
Madagascar	MAHAY (43)	1702	1682	0.03 (−0.11, 0.17)	0.08
Malawi	iLiNS−DYAD−M (44)	220	444	−0.08 (−0.25, 0.10)	0.06
Malawi	iLiNS−DOSE (45)	696	241	0.06 (−0.04, 0.17)	0.10
Zimbabwe	SHINE (HIV−) (47)	1880	1794	0.16 (0.08, 0.23)	0.12
Zimbabwe	SHINE (HIV+) (48)	337	330	0.25 (0.08, 0.43)	0.06
**I² = 0.71, Tau² = 0.01**		**11512**	**8689**	**0.12 (0.06, 0.19)**	
**Supplement duration − > 12m**					
Bangladesh	RDNS (35)	1663	815	0.08 (−0.01, 0.16)	0.23
Bangladesh	WASH−B (36)	1158	3431	0.21 (0.14, 0.28)	0.25
Kenya	WASH−B (42)	1457	5137	0.15 (0.09, 0.21)	0.25
Mali	PROMIS (46)	506	506	0.16 (0.01, 0.31)	0.15
Mali	PROMIS CS (46)	952	969	0.22 (0.05, 0.39)	0.13
**I² = 0.37, Tau² = 0.00**		**5736**	**10858**	**0.16 (0.11, 0.21)**	

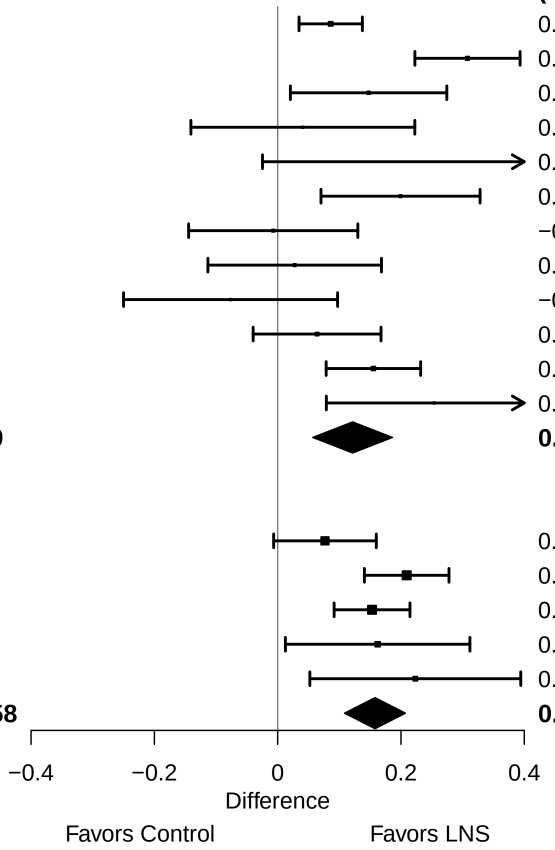

Supplemental figure 6A: Mean difference in LAZ

6A7: Stratified by Frequency of contact

Frequency of contact
(p−diff = 0.527)

Country	Trial	N	N	MD (95% CI)	W
**Frequency of contact − Monthly**					
Bangladesh	RDNS (35)	1663	815	0.08 (−0.01, 0.16)	0.13
Burkina Faso	PROMIS (38)	863	914	0.15 (0.02, 0.27)	0.10
Burkina Faso	PROMIS CS (38)	430	439	0.04 (−0.14, 0.22)	0.07
Haiti	HAITI (41)	149	149	−0.01 (−0.14, 0.13)	0.09
Kenya	WASH−B (42)	1457	5137	0.15 (0.09, 0.21)	0.15
Madagascar	MAHAY (43)	1702	1682	0.03 (−0.11, 0.17)	0.09
Mali	PROMIS (46)	506	506	0.16 (0.01, 0.31)	0.09
Mali	PROMIS CS (46)	952	969	0.22 (0.05, 0.39)	0.07
Zimbabwe	SHINE (HIV−) (47)	1880	1794	0.16 (0.08, 0.23)	0.14
Zimbabwe	SHINE (HIV+) (48)	337	330	0.25 (0.08, 0.43)	0.07
**I² = 0.29, Tau² = 0.00**		**9939**	**12735**	**0.12 (0.08, 0.17)**	
**Frequency of contact − Weekly**					
Bangladesh	JiVitA−4 (34)	2838	1244	0.09 (0.03, 0.14)	0.21
Bangladesh	WASH−B (36)	1158	3431	0.21 (0.14, 0.28)	0.19
Burkina Faso	iLiNS−Zinc (37)	1952	664	0.31 (0.22, 0.39)	0.17
Ghana	GHANA (39)	98	96	0.28 (−0.02, 0.58)	0.04
Ghana	iLiNS−DYAD−G (40)	347	692	0.20 (0.07, 0.33)	0.13
Malawi	iLiNS−DYAD−M (44)	220	444	−0.08 (−0.25, 0.10)	0.10
Malawi	iLiNS−DOSE (45)	696	241	0.06 (−0.04, 0.17)	0.16
**I² = 0.81, Tau² = 0.01**		**7309**	**6812**	**0.15 (0.06, 0.25)**	

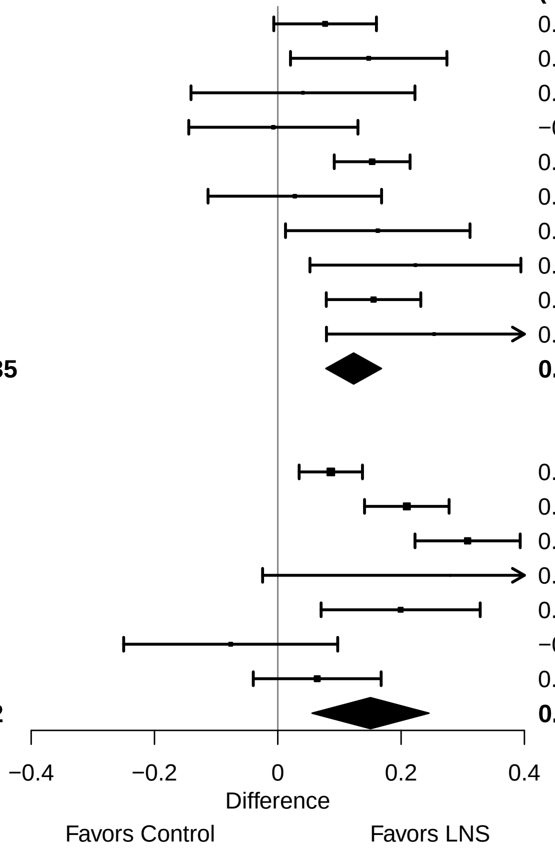

Supplemental figure 6A: Mean difference in LAZ

6A8: Stratified by Average SQ-LNS compliance

Average SQ−LNS compliance
(p−diff = 0.724)

Country	Trial	N	N	MD (95% CI)	W
**Average SQ−LNS compliance − Low**					
Burkina Faso	PROMIS (38)	863	914	0.15 (0.02, 0.27)	0.12
Burkina Faso	PROMIS CS (38)	430	439	0.04 (−0.14, 0.22)	0.08
Ghana	iLiNS−DYAD−G (40)	347	692	0.20 (0.07, 0.33)	0.12
Malawi	iLiNS−DYAD−M (44)	220	444	−0.08 (−0.25, 0.10)	0.09
Malawi	iLiNS−DOSE (45)	696	241	0.06 (−0.04, 0.17)	0.14
Mali	PROMIS (46)	506	506	0.16 (0.01, 0.31)	0.10
Mali	PROMIS CS (46)	952	969	0.22 (0.05, 0.39)	0.09
Zimbabwe	SHINE (HIV−) (47)	1880	1794	0.16 (0.08, 0.23)	0.17
Zimbabwe	SHINE (HIV+) (48)	337	330	0.25 (0.08, 0.43)	0.09
**I² = 0.37, Tau² = 0.00**		**6231**	**6329**	**0.13 (0.07, 0.19)**	
**Average SQ−LNS compliance − High**					
Bangladesh	JiVitA−4 (34)	2838	1244	0.09 (0.03, 0.14)	0.19
Bangladesh	RDNS (35)	1663	815	0.08 (−0.01, 0.16)	0.16
Bangladesh	WASH−B (36)	1158	3431	0.21 (0.14, 0.28)	0.17
Burkina Faso	iLiNS−Zinc (37)	1952	664	0.31 (0.22, 0.39)	0.16
Ghana	GHANA (39)	98	96	0.28 (−0.02, 0.58)	0.04
Haiti	HAITI (41)	149	149	−0.01 (−0.14, 0.13)	0.11
Kenya	WASH−B (42)	1457	5137	0.15 (0.09, 0.21)	0.18
**I² = 0.80, Tau² = 0.01**		**9315**	**11536**	**0.15 (0.07, 0.23)**	

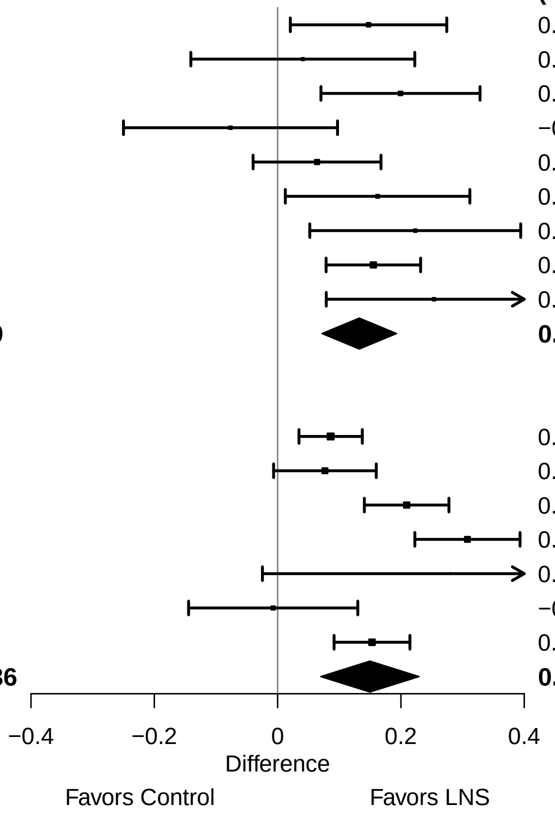

Supplemental figure 6B: Stunting prevalence ratio

6B1: Stratified by Geographic region

Geographic region
(p−diff = 0.646)

Country	Trial	N	N	PR (95% CI)	W
**Geographic region − SEAR**					
Bangladesh	JiVitA−4 (34)	2838	1244	0.92 (0.84, 1.01)	0.32
Bangladesh	RDNS (35)	1663	815	0.92 (0.85, 1.00)	0.34
Bangladesh	WASH−B (36)	1158	3431	0.84 (0.77, 0.91)	0.34
**I² = 0.36, Tau² = 0.00**		**5659**	**5490**	**0.89 (0.84, 0.95)**	
**Geographic region − AFR**					
Burkina Faso	iLiNS−Zinc (37)	1952	664	0.75 (0.65, 0.87)	0.09
Burkina Faso	PROMIS (38)	863	914	0.82 (0.67, 1.00)	0.07
Burkina Faso	PROMIS CS (38)	430	439	0.87 (0.63, 1.19)	0.03
Ghana	GHANA (39)	98	96	0.70 (0.23, 2.13)	0.00
Ghana	iLiNS−DYAD−G (40)	347	692	0.66 (0.45, 0.98)	0.02
Kenya	WASH−B (42)	1457	5137	0.86 (0.78, 0.94)	0.14
Madagascar	MAHAY (43)	1702	1682	0.98 (0.90, 1.07)	0.14
Malawi	iLiNS−DYAD−M (44)	220	444	1.10 (0.89, 1.36)	0.06
Malawi	iLiNS−DOSE (45)	696	241	1.00 (0.87, 1.15)	0.10
Mali	PROMIS (46)	506	506	0.92 (0.76, 1.10)	0.07
Mali	PROMIS CS (46)	952	969	0.78 (0.65, 0.95)	0.07
Zimbabwe	SHINE (HIV−) (47)	1880	1794	0.79 (0.72, 0.87)	0.13
Zimbabwe	SHINE (HIV+) (48)	337	330	0.81 (0.68, 0.96)	0.08
**I² = 0.57, Tau² = 0.01**		**11440**	**13908**	**0.87 (0.81, 0.93)**	

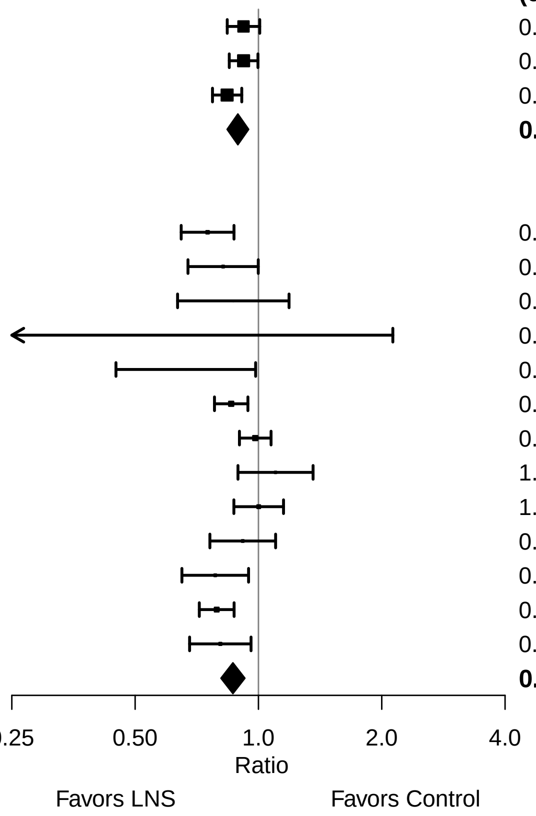

Supplemental figure 6B: Stunting prevalence ratio

6B2: Stratified by Stunting burden

Stunting burden
(p−diff = 0.912)

Country	Trial	N	N	PR (95% CI)	W
**Stunting burden − Less than 35%**					
Burkina Faso	PROMIS (38)	863	914	0.82 (0.67, 1.00)	0.20
Burkina Faso	PROMIS CS (38)	430	439	0.87 (0.63, 1.19)	0.10
Ghana	GHANA (39)	98	96	0.70 (0.23, 2.13)	0.01
Ghana	iLiNS−DYAD−G (40)	347	692	0.66 (0.45, 0.98)	0.06
Haiti	HAITI (41)	149	149	1.04 (0.60, 1.81)	0.03
Kenya	WASH−B (42)	1457	5137	0.86 (0.78, 0.94)	0.42
Malawi	iLiNS−DYAD−M (44)	220	444	1.10 (0.89, 1.36)	0.18
**I² = 0.21, Tau² = 0.00**		**3564**	**7871**	**0.88 (0.79, 0.97)**	
**Stunting burden − More than 35%**					
Bangladesh	JiVitA−4 (34)	2838	1244	0.92 (0.84, 1.01)	0.12
Bangladesh	RDNS (35)	1663	815	0.92 (0.85, 1.00)	0.13
Bangladesh	WASH−B (36)	1158	3431	0.84 (0.77, 0.91)	0.13
Burkina Faso	iLiNS−Zinc (37)	1952	664	0.75 (0.65, 0.87)	0.08
Madagascar	MAHAY (43)	1702	1682	0.98 (0.90, 1.07)	0.13
Malawi	iLiNS−DOSE (45)	696	241	1.00 (0.87, 1.15)	0.09
Mali	PROMIS (46)	506	506	0.92 (0.76, 1.10)	0.06
Mali	PROMIS CS (46)	952	969	0.78 (0.65, 0.95)	0.06
Zimbabwe	SHINE (HIV−) (47)	1880	1794	0.79 (0.72, 0.87)	0.12
Zimbabwe	SHINE (HIV+) (48)	337	330	0.81 (0.68, 0.96)	0.07
**I² = 0.63, Tau² = 0.01**		**13684**	**11676**	**0.87 (0.82, 0.93)**	

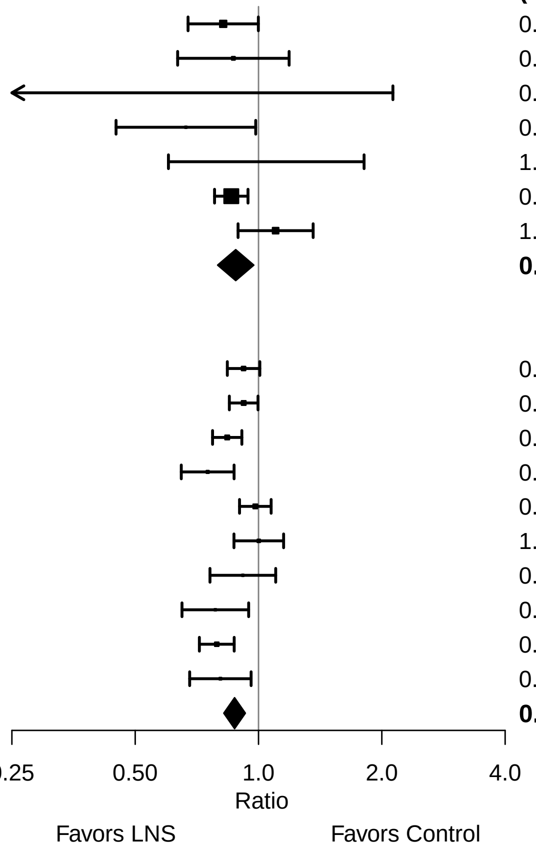

Supplemental figure 6B: Stunting prevalence ratio

6B3: Stratified by Malaria prevalence

Malaria prevalence
(p−diff = 0.862)

Country	Trial	N	N	PR (95% CI)	W
**Malaria prevalence − Less than 10%**					
Bangladesh	JiVitA−4 (34)	2838	1244	0.92 (0.84, 1.01)	0.15
Bangladesh	RDNS (35)	1663	815	0.92 (0.85, 1.00)	0.16
Bangladesh	WASH−B (36)	1158	3431	0.84 (0.77, 0.91)	0.16
Haiti	HAITI (41)	149	149	1.04 (0.60, 1.81)	0.01
Kenya	WASH−B (42)	1457	5137	0.86 (0.78, 0.94)	0.15
Madagascar	MAHAY (43)	1702	1682	0.98 (0.90, 1.07)	0.15
Zimbabwe	SHINE (HIV−) (47)	1880	1794	0.79 (0.72, 0.87)	0.14
Zimbabwe	SHINE (HIV+) (48)	337	330	0.81 (0.68, 0.96)	0.08
**I² = 0.55, Tau² = 0.00**		**11184**	**14582**	**0.88 (0.83, 0.93)**	
**Malaria prevalence − At least 10%**					
Burkina Faso	iLiNS−Zinc (37)	1952	664	0.75 (0.65, 0.87)	0.18
Burkina Faso	PROMIS (38)	863	914	0.82 (0.67, 1.00)	0.13
Burkina Faso	PROMIS CS (38)	430	439	0.87 (0.63, 1.19)	0.06
Ghana	GHANA (39)	98	96	0.70 (0.23, 2.13)	0.01
Ghana	iLiNS−DYAD−G (40)	347	692	0.66 (0.45, 0.98)	0.04
Malawi	iLiNS−DYAD−M (44)	220	444	1.10 (0.89, 1.36)	0.12
Malawi	iLiNS−DOSE (45)	696	241	1.00 (0.87, 1.15)	0.19
Mali	PROMIS (46)	506	506	0.92 (0.76, 1.10)	0.14
Mali	PROMIS CS (46)	952	969	0.78 (0.65, 0.95)	0.14
**I² = 0.51, Tau² = 0.01**		**6064**	**4965**	**0.87 (0.79, 0.96)**	

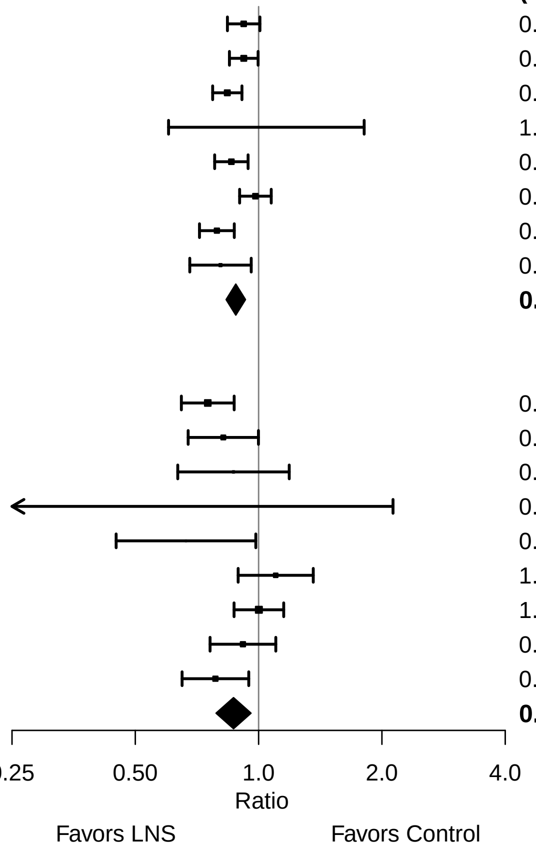

Supplemental figure 6B: Stunting prevalence ratio

6B4: Stratified by Source water quality

Source water quality
(p−diff = 0.267)

**Source water quality − Improved**				**PR**	
**Country**	**Trial**	**N**	**N**	**(95% CI)**	**W**
Bangladesh	JiVitA−4 (34)	2838	1244	0.92 (0.84, 1.01)	0.24
Bangladesh	RDNS (35)	1663	815	0.92 (0.85, 1.00)	0.27
Bangladesh	WASH−B (36)	567	1104	0.80 (0.70, 0.92)	0.17
Ghana	GHANA (39)	98	96	0.70 (0.23, 2.13)	0.00
Ghana	iLiNS−DYAD−G (40)	347	692	0.66 (0.45, 0.98)	0.03
Haiti	HAITI (41)	149	149	1.04 (0.60, 1.81)	0.02
Malawi	iLiNS−DYAD−M (44)	220	444	1.10 (0.89, 1.36)	0.10
Malawi	iLiNS−DOSE (45)	696	241	1.00 (0.87, 1.15)	0.17
**I² = 0.38, Tau² = 0.01**		**6578**	**4785**	**0.92 (0.84, 1.00)**	
**Source water quality − Unimproved**					
Burkina Faso	iLiNS−Zinc (37)	1952	664	0.75 (0.65, 0.87)	0.13
Burkina Faso	PROMIS (38)	863	914	0.82 (0.67, 1.00)	0.09
Burkina Faso	PROMIS CS (38)	430	439	0.87 (0.63, 1.19)	0.04
Kenya	WASH−B (42)	695	2253	0.92 (0.79, 1.08)	0.12
Madagascar	MAHAY (43)	1702	1682	0.98 (0.90, 1.07)	0.21
Mali	PROMIS (46)	506	506	0.92 (0.76, 1.10)	0.10
Mali	PROMIS CS (46)	952	969	0.78 (0.65, 0.95)	0.10
Zimbabwe	SHINE (HIV−) (47)	892	880	0.85 (0.74, 0.97)	0.15
Zimbabwe	SHINE (HIV+) (48)	147	146	0.79 (0.61, 1.04)	0.06
**I² = 0.41, Tau² = 0.00**		**8139**	**8453**	**0.87 (0.81, 0.93)**	

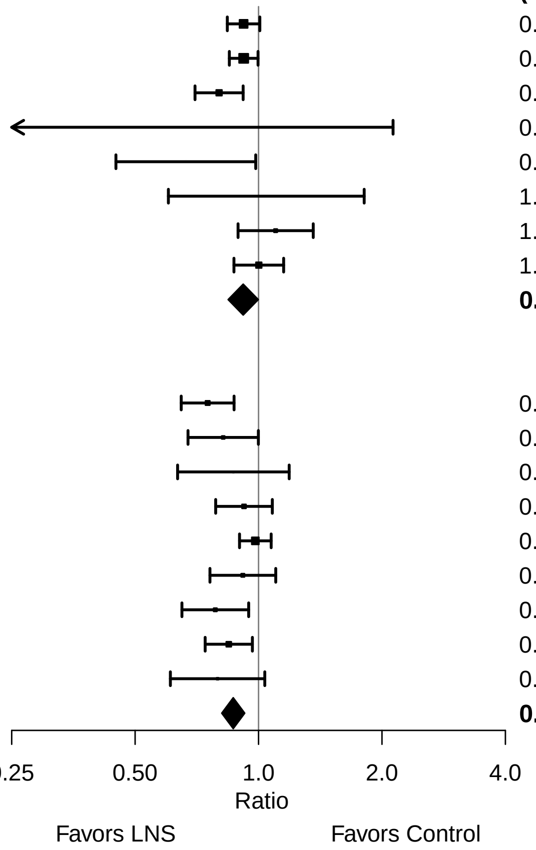

Supplemental figure 6B: Stunting prevalence ratio

6B5: Stratified by Sanitation

Sanitation
(p−diff = 0.328)

Country	Trial	N	N	PR (95% CI)	W
**Sanitation − Improved**					
Bangladesh	JiVitA−4 (34)	2838	1244	0.92 (0.84, 1.01)	0.22
Bangladesh	RDNS (35)	1663	815	0.92 (0.85, 1.00)	0.24
Bangladesh	WASH−B (36)	567	1104	0.80 (0.70, 0.92)	0.15
Burkina Faso	PROMIS (38)	863	914	0.82 (0.67, 1.00)	0.09
Burkina Faso	PROMIS CS (38)	430	439	0.87 (0.63, 1.19)	0.04
Ghana	GHANA (39)	98	96	0.70 (0.23, 2.13)	0.00
Ghana	iLiNS−DYAD−G (40)	347	692	0.66 (0.45, 0.98)	0.03
Haiti	HAITI (41)	149	149	1.04 (0.60, 1.81)	0.02
Mali	PROMIS (46)	506	506	0.92 (0.76, 1.10)	0.10
Mali	PROMIS CS (46)	952	969	0.78 (0.65, 0.95)	0.10
**I^2 = 0.00, Tau^2 = 0.00**		**8413**	**6928**	**0.88 (0.84, 0.93)**	
**Sanitation − Unimproved**					
Burkina Faso	iLiNS−Zinc (37)	1952	664	0.75 (0.65, 0.87)	0.15
Kenya	WASH−B (42)	695	2253	0.92 (0.79, 1.08)	0.13
Madagascar	MAHAY (43)	1702	1682	0.98 (0.90, 1.07)	0.24
Malawi	iLiNS−DYAD−M (44)	220	444	1.10 (0.89, 1.36)	0.09
Malawi	iLiNS−DOSE (45)	696	241	1.00 (0.87, 1.15)	0.16
Zimbabwe	SHINE (HIV−) (47)	892	880	0.85 (0.74, 0.97)	0.17
Zimbabwe	SHINE (HIV+) (48)	147	146	0.79 (0.61, 1.04)	0.06
**I^2 = 0.63, Tau^2 = 0.01**		**6304**	**6310**	**0.91 (0.83, 1.00)**	

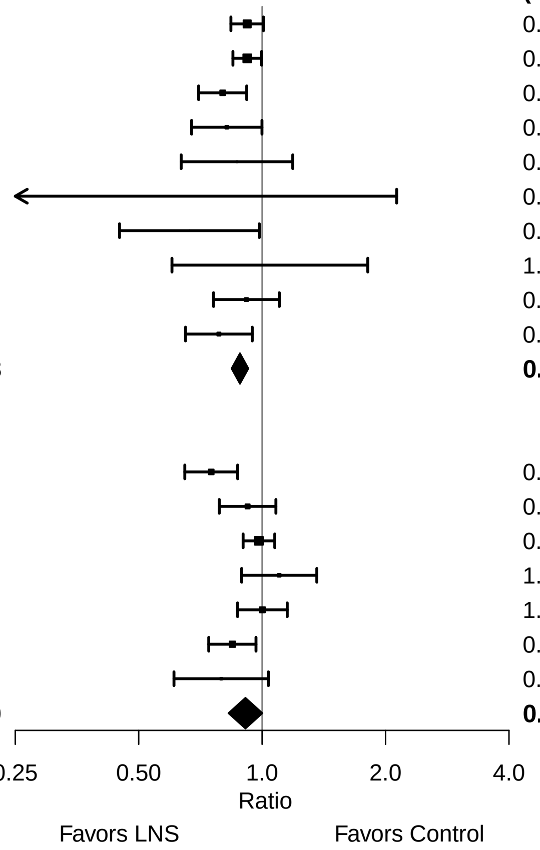

Supplemental figure 6B: Stunting prevalence ratio

6B6: Stratified by Supplement duration

Supplement duration
(p−diff = 0.742)

Country	Trial	N	N	PR (95% CI)	W
**Supplement duration − 12m or less**					
Bangladesh	JiVitA−4 (34)	2838	1244	0.92 (0.84, 1.01)	0.16
Burkina Faso	iLiNS−Zinc (37)	1952	664	0.75 (0.65, 0.87)	0.11
Burkina Faso	PROMIS (38)	863	914	0.82 (0.67, 1.00)	0.07
Burkina Faso	PROMIS CS (38)	430	439	0.87 (0.63, 1.19)	0.04
Ghana	GHANA (39)	98	96	0.70 (0.23, 2.13)	0.00
Ghana	iLiNS−DYAD−G (40)	347	692	0.66 (0.45, 0.98)	0.02
Haiti	HAITI (41)	149	149	1.04 (0.60, 1.81)	0.01
Madagascar	MAHAY (43)	1702	1682	0.98 (0.90, 1.07)	0.16
Malawi	iLiNS−DYAD−M (44)	220	444	1.10 (0.89, 1.36)	0.07
Malawi	iLiNS−DOSE (45)	696	241	1.00 (0.87, 1.15)	0.11
Zimbabwe	SHINE (HIV−) (47)	1880	1794	0.79 (0.72, 0.87)	0.15
Zimbabwe	SHINE (HIV+) (48)	337	330	0.81 (0.68, 0.96)	0.09
**I² = 0.59, Tau² = 0.01**		**11512**	**8689**	**0.88 (0.82, 0.95)**	
**Supplement duration − > 12m**					
Bangladesh	RDNS (35)	1663	815	0.92 (0.85, 1.00)	0.26
Bangladesh	WASH−B (36)	1158	3431	0.84 (0.77, 0.91)	0.26
Kenya	WASH−B (42)	1457	5137	0.86 (0.78, 0.94)	0.24
Mali	PROMIS (46)	506	506	0.92 (0.76, 1.10)	0.12
Mali	PROMIS CS (46)	952	969	0.78 (0.65, 0.95)	0.12
**I² = 0.04, Tau² = 0.00**		**5736**	**10858**	**0.87 (0.83, 0.91)**	

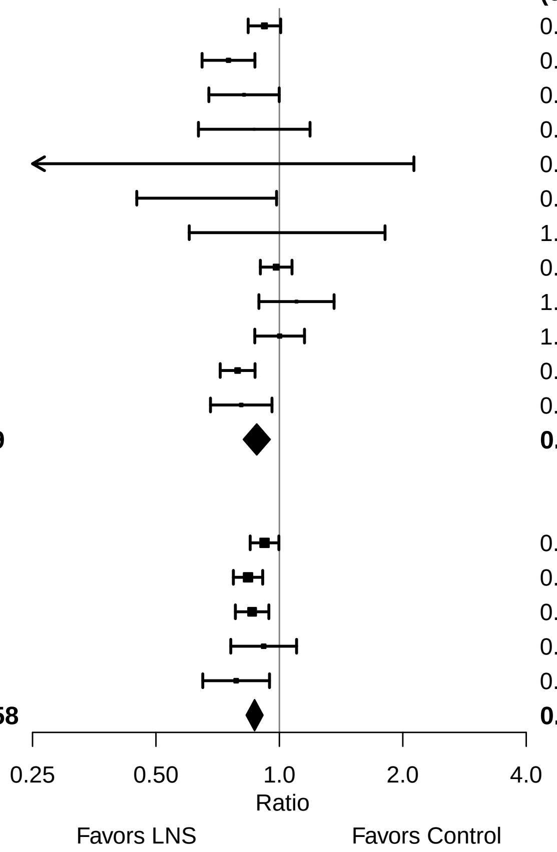

Supplemental figure 6B: Stunting prevalence ratio

6B7: Stratified by Frequency of contact

Frequency of contact
(p−diff = 0.734)

Country	Trial	N	N	PR (95% CI)	W
**Frequency of contact − Monthly**					
Bangladesh	RDNS (35)	1663	815	0.92 (0.85, 1.00)	0.17
Burkina Faso	PROMIS (38)	863	914	0.82 (0.67, 1.00)	0.07
Burkina Faso	PROMIS CS (38)	430	439	0.87 (0.63, 1.19)	0.04
Haiti	HAITI (41)	149	149	1.04 (0.60, 1.81)	0.01
Kenya	WASH−B (42)	1457	5137	0.86 (0.78, 0.94)	0.15
Madagascar	MAHAY (43)	1702	1682	0.98 (0.90, 1.07)	0.16
Mali	PROMIS (46)	506	506	0.92 (0.76, 1.10)	0.08
Mali	PROMIS CS (46)	952	969	0.78 (0.65, 0.95)	0.08
Zimbabwe	SHINE (HIV−) (47)	1880	1794	0.79 (0.72, 0.87)	0.15
Zimbabwe	SHINE (HIV+) (48)	337	330	0.81 (0.68, 0.96)	0.09
**I^2 = 0.41, Tau^2 = 0.00**		**9939**	**12735**	**0.87 (0.83, 0.92)**	
**Frequency of contact − Weekly**					
Bangladesh	JiVitA−4 (34)	2838	1244	0.92 (0.84, 1.01)	0.25
Bangladesh	WASH−B (36)	1158	3431	0.84 (0.77, 0.91)	0.27
Burkina Faso	iLiNS−Zinc (37)	1952	664	0.75 (0.65, 0.87)	0.16
Ghana	GHANA (39)	98	96	0.70 (0.23, 2.13)	0.01
Ghana	iLiNS−DYAD−G (40)	347	692	0.66 (0.45, 0.98)	0.04
Malawi	iLiNS−DYAD−M (44)	220	444	1.10 (0.89, 1.36)	0.10
Malawi	iLiNS−DOSE (45)	696	241	1.00 (0.87, 1.15)	0.17
**I^2 = 0.63, Tau^2 = 0.01**		**7309**	**6812**	**0.88 (0.79, 0.99)**	

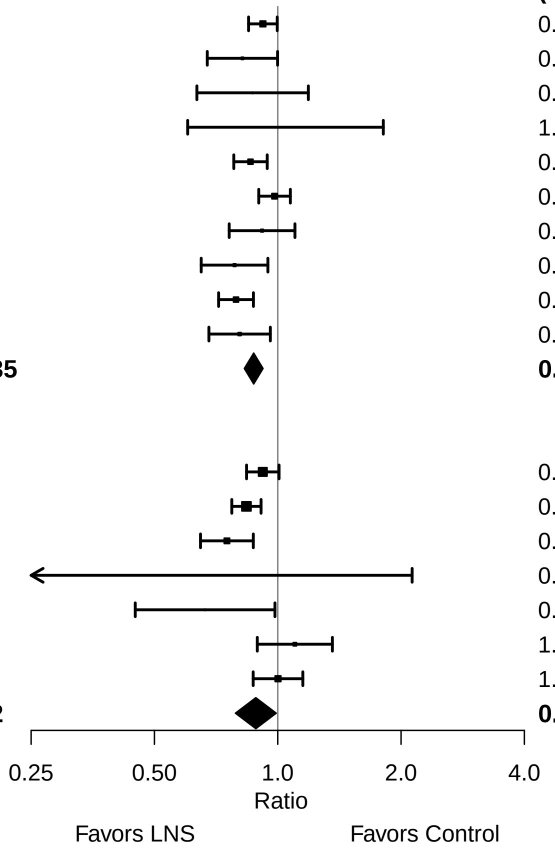

Supplemental figure 6B: Stunting prevalence ratio

6B8: Stratified by Average SQ-LNS compliance

Average SQ−LNS compliance
(p−diff = 0.974)

Country	Trial	N	N	PR (95% CI)	W
**Average SQ−LNS compliance − Low**					
Burkina Faso	PROMIS (38)	863	914	0.82 (0.67, 1.00)	0.10
Burkina Faso	PROMIS CS (38)	430	439	0.87 (0.63, 1.19)	0.05
Ghana	iLiNS−DYAD−G (40)	347	692	0.66 (0.45, 0.98)	0.03
Malawi	iLiNS−DYAD−M (44)	220	444	1.10 (0.89, 1.36)	0.09
Malawi	iLiNS−DOSE (45)	696	241	1.00 (0.87, 1.15)	0.16
Mali	PROMIS (46)	506	506	0.92 (0.76, 1.10)	0.11
Mali	PROMIS CS (46)	952	969	0.78 (0.65, 0.95)	0.11
Zimbabwe	SHINE (HIV−) (47)	1880	1794	0.79 (0.72, 0.87)	0.23
Zimbabwe	SHINE (HIV+) (48)	337	330	0.81 (0.68, 0.96)	0.12
**I^2 = 0.51, Tau^2 = 0.01**		**6231**	**6329**	**0.86 (0.79, 0.94)**	
**Average SQ−LNS compliance − High**					
Bangladesh	JiVitA−4 (34)	2838	1244	0.92 (0.84, 1.01)	0.21
Bangladesh	RDNS (35)	1663	815	0.92 (0.85, 1.00)	0.22
Bangladesh	WASH−B (36)	1158	3431	0.84 (0.77, 0.91)	0.22
Burkina Faso	iLiNS−Zinc (37)	1952	664	0.75 (0.65, 0.87)	0.13
Ghana	GHANA (39)	98	96	0.70 (0.23, 2.13)	0.00
Haiti	HAITI (41)	149	149	1.04 (0.60, 1.81)	0.01
Kenya	WASH−B (42)	1457	5137	0.86 (0.78, 0.94)	0.20
**I^2 = 0.29, Tau^2 = 0.00**		**9315**	**11536**	**0.87 (0.82, 0.92)**	

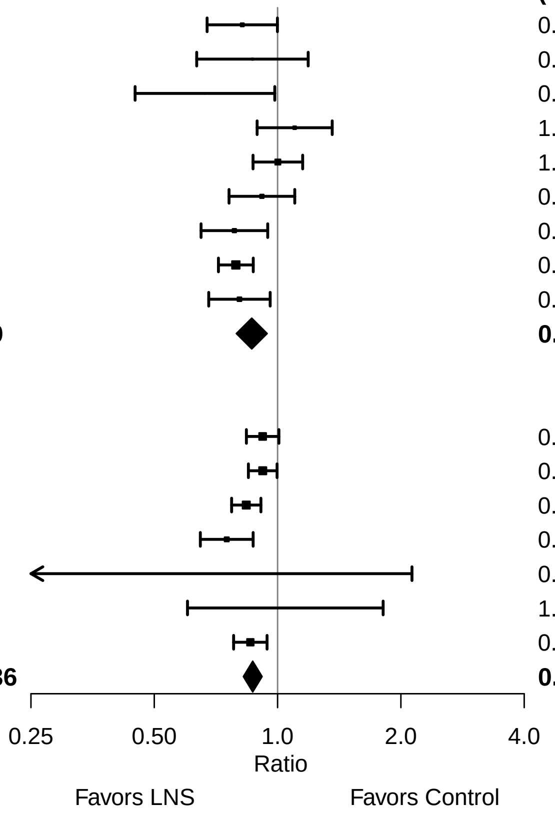

Supplemental figure 6C: Stunting prevalence difference

6C1: Stratified by Geographic region

Geographic region
(p−diff = 0.874)

Country	Trial	N	N	PD (95% CI)	W
**Geographic region − SEAR**					
Bangladesh	JiVitA−4 (34)	2838	1244	−0.04 (−0.06, −0.01)	0.38
Bangladesh	RDNS (35)	1663	815	−0.03 (−0.07, 0.01)	0.28
Bangladesh	WASH−B (36)	1158	3431	−0.07 (−0.10, −0.04)	0.34
**I² = 0.26, Tau² = 0.00**		**5659**	**5490**	**−0.05 (−0.07, −0.03)**	
**Geographic region − AFR**					
Burkina Faso	iLiNS−Zinc (37)	1952	664	−0.11 (−0.14, −0.08)	0.10
Burkina Faso	PROMIS (38)	863	914	−0.05 (−0.10, −0.01)	0.08
Burkina Faso	PROMIS CS (38)	430	439	−0.03 (−0.09, 0.02)	0.06
Ghana	GHANA (39)	98	96	−0.02 (−0.09, 0.05)	0.05
Ghana	iLiNS−DYAD−G (40)	347	692	−0.04 (−0.08, 0.00)	0.09
Kenya	WASH−B (42)	1457	5137	−0.05 (−0.07, −0.02)	0.12
Madagascar	MAHAY (43)	1702	1682	−0.01 (−0.04, 0.02)	0.10
Malawi	iLiNS−DYAD−M (44)	220	444	0.03 (−0.04, 0.11)	0.04
Malawi	iLiNS−DOSE (45)	696	241	−0.02 (−0.07, 0.04)	0.06
Mali	PROMIS (46)	506	506	−0.04 (−0.09, 0.01)	0.07
Mali	PROMIS CS (46)	952	969	−0.07 (−0.11, −0.03)	0.08
Zimbabwe	SHINE (HIV−) (47)	1880	1794	−0.07 (−0.10, −0.04)	0.11
Zimbabwe	SHINE (HIV+) (48)	337	330	−0.10 (−0.17, −0.02)	0.04
**I² = 0.60, Tau² = 0.00**		**11440**	**13908**	**−0.05 (−0.07, −0.03)**	

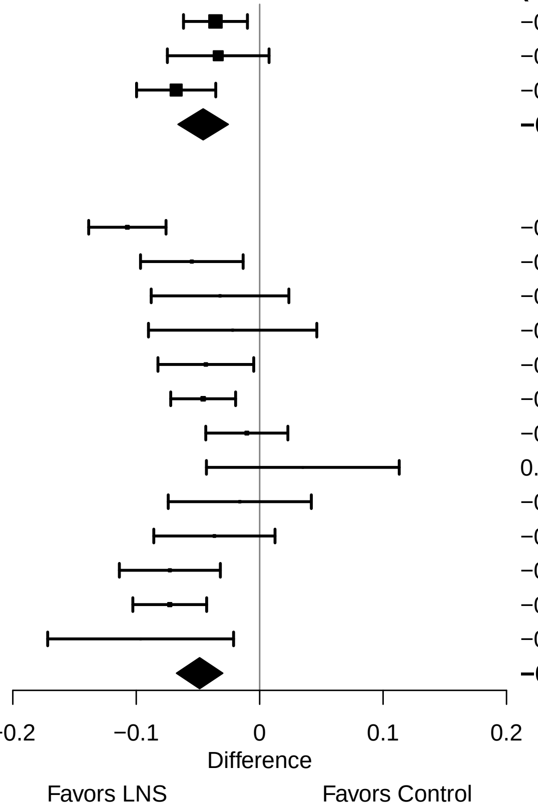

Supplemental figure 6C: Stunting prevalence difference

6C2: Stratified by Stunting burden

Stunting burden
(p−diff = 0.181)

Stunting burden − Less than 35%

Country	Trial	N	N	PD (95% CI)	W
Burkina Faso	PROMIS (38)	863	914	−0.05 (−0.10, −0.01)	0.17
Burkina Faso	PROMIS CS (38)	430	439	−0.03 (−0.09, 0.02)	0.12
Ghana	GHANA (39)	98	96	−0.02 (−0.09, 0.05)	0.09
Ghana	iLiNS−DYAD−G (40)	347	692	−0.04 (−0.08, 0.00)	0.18
Haiti	HAITI (41)	149	149	−0.02 (−0.08, 0.04)	0.11
Kenya	WASH−B (42)	1457	5137	−0.05 (−0.07, −0.02)	0.24
Malawi	iLiNS−DYAD−M (44)	220	444	0.03 (−0.04, 0.11)	0.08
**I² = 0.00, Tau² = 0.00**		**3564**	**7871**	**−0.04 (−0.06, −0.02)**	

Stunting burden − More than 35%

Country	Trial	N	N	PD (95% CI)	W
Bangladesh	JiVitA−4 (34)	2838	1244	−0.04 (−0.06, −0.01)	0.14
Bangladesh	RDNS (35)	1663	815	−0.03 (−0.07, 0.01)	0.10
Bangladesh	WASH−B (36)	1158	3431	−0.07 (−0.10, −0.04)	0.12
Burkina Faso	iLiNS−Zinc (37)	1952	664	−0.11 (−0.14, −0.08)	0.12
Madagascar	MAHAY (43)	1702	1682	−0.01 (−0.04, 0.02)	0.12
Malawi	iLiNS−DOSE (45)	696	241	−0.02 (−0.07, 0.04)	0.07
Mali	PROMIS (46)	506	506	−0.04 (−0.09, 0.01)	0.08
Mali	PROMIS CS (46)	952	969	−0.07 (−0.11, −0.03)	0.10
Zimbabwe	SHINE (HIV−) (47)	1880	1794	−0.07 (−0.10, −0.04)	0.12
Zimbabwe	SHINE (HIV+) (48)	337	330	−0.10 (−0.17, −0.02)	0.05
**I² = 0.66, Tau² = 0.00**		**13684**	**11676**	**−0.05 (−0.07, −0.03)**	

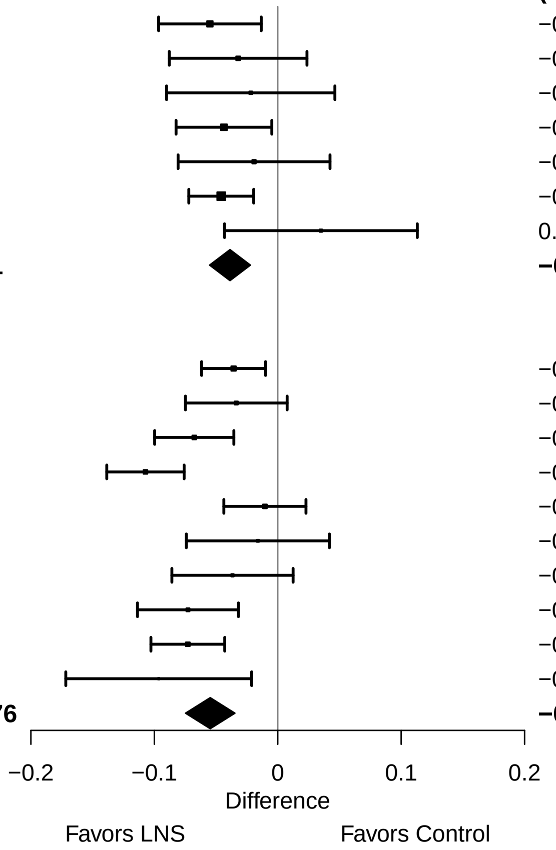

Supplemental figure 6C: Stunting prevalence difference

6C3: Stratified by Malaria prevalence

Malaria prevalence
(p−diff = 0.848)

Malaria prevalence − Less than 10%

Country	Trial	N	N	PD (95% CI)	W
Bangladesh	JiVitA−4 (34)	2838	1244	−0.04 (−0.06, −0.01)	0.16
Bangladesh	RDNS (35)	1663	815	−0.03 (−0.07, 0.01)	0.12
Bangladesh	WASH−B (36)	1158	3431	−0.07 (−0.10, −0.04)	0.14
Haiti	HAITI (41)	149	149	−0.02 (−0.08, 0.04)	0.07
Kenya	WASH−B (42)	1457	5137	−0.05 (−0.07, −0.02)	0.16
Madagascar	MAHAY (43)	1702	1682	−0.01 (−0.04, 0.02)	0.14
Zimbabwe	SHINE (HIV−) (47)	1880	1794	−0.07 (−0.10, −0.04)	0.15
Zimbabwe	SHINE (HIV+) (48)	337	330	−0.10 (−0.17, −0.02)	0.05
**I² = 0.45, Tau² = 0.00**		**11184**	**14582**	**−0.05 (−0.06, −0.03)**	

Malaria prevalence − At least 10%

Country	Trial	N	N	PD (95% CI)	W
Burkina Faso	iLiNS−Zinc (37)	1952	664	−0.11 (−0.14, −0.08)	0.17
Burkina Faso	PROMIS (38)	863	914	−0.05 (−0.10, −0.01)	0.13
Burkina Faso	PROMIS CS (38)	430	439	−0.03 (−0.09, 0.02)	0.10
Ghana	GHANA (39)	98	96	−0.02 (−0.09, 0.05)	0.07
Ghana	iLiNS−DYAD−G (40)	347	692	−0.04 (−0.08, 0.00)	0.14
Malawi	iLiNS−DYAD−M (44)	220	444	0.03 (−0.04, 0.11)	0.06
Malawi	iLiNS−DOSE (45)	696	241	−0.02 (−0.07, 0.04)	0.09
Mali	PROMIS (46)	506	506	−0.04 (−0.09, 0.01)	0.11
Mali	PROMIS CS (46)	952	969	−0.07 (−0.11, −0.03)	0.13
**I² = 0.61, Tau² = 0.00**		**6064**	**4965**	**−0.05 (−0.07, −0.02)**	

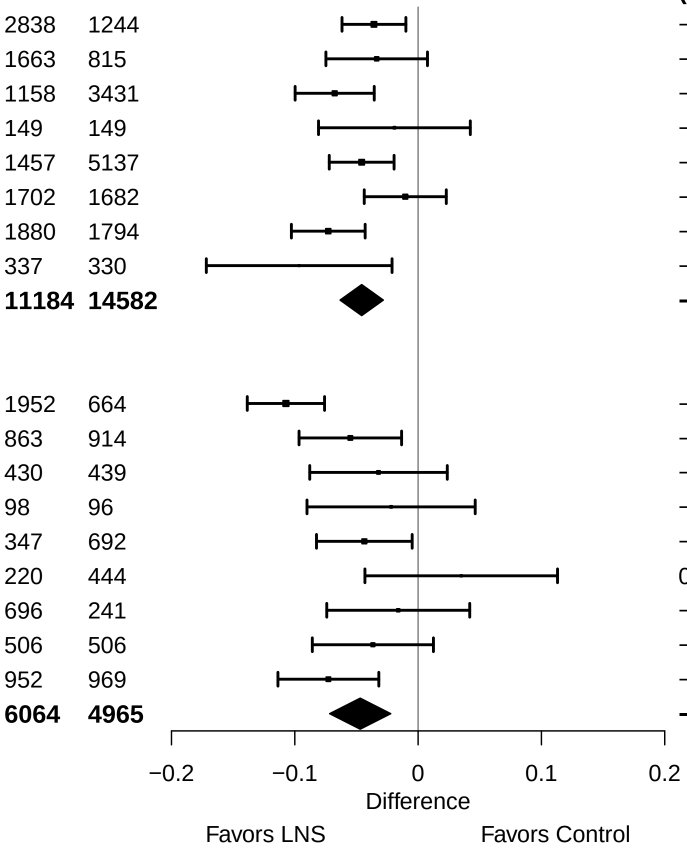

Supplemental figure 6C: Stunting prevalence difference

6C4: Stratified by Source water quality

Source water quality
(p−diff = 0.234)

Source water quality − Improved

Country	Trial	N	N	PD (95% CI)	W
Bangladesh	JiVitA−4 (34)	2838	1244	−0.04 (−0.06, −0.01)	0.22
Bangladesh	RDNS (35)	1663	815	−0.03 (−0.07, 0.01)	0.15
Bangladesh	WASH−B (36)	567	1104	−0.08 (−0.13, −0.03)	0.13
Ghana	GHANA (39)	98	96	−0.02 (−0.09, 0.05)	0.08
Ghana	iLiNS−DYAD−G (40)	347	692	−0.04 (−0.08, 0.00)	0.16
Haiti	HAITI (41)	149	149	−0.02 (−0.08, 0.04)	0.09
Malawi	iLiNS−DYAD−M (44)	220	444	0.03 (−0.04, 0.11)	0.07
Malawi	iLiNS−DOSE (45)	696	241	−0.02 (−0.07, 0.04)	0.10
**I² = 0.08, Tau² = 0.00**		**6578**	**4785**	**−0.03 (−0.05, −0.02)**	

Source water quality − Unimproved

Country	Trial	N	N	PD (95% CI)	W
Burkina Faso	iLiNS−Zinc (37)	1952	664	−0.11 (−0.14, −0.08)	0.15
Burkina Faso	PROMIS (38)	863	914	−0.05 (−0.10, −0.01)	0.12
Burkina Faso	PROMIS CS (38)	430	439	−0.03 (−0.09, 0.02)	0.09
Kenya	WASH−B (42)	695	2253	−0.02 (−0.06, 0.01)	0.13
Madagascar	MAHAY (43)	1702	1682	−0.01 (−0.04, 0.02)	0.15
Mali	PROMIS (46)	506	506	−0.04 (−0.09, 0.01)	0.10
Mali	PROMIS CS (46)	952	969	−0.07 (−0.11, −0.03)	0.12
Zimbabwe	SHINE (HIV−) (47)	892	880	−0.05 (−0.09, −0.01)	0.12
Zimbabwe	SHINE (HIV+) (48)	147	146	−0.11 (−0.22, 0.01)	0.03
**I² = 0.65, Tau² = 0.00**		**8139**	**8453**	**−0.05 (−0.07, −0.03)**	

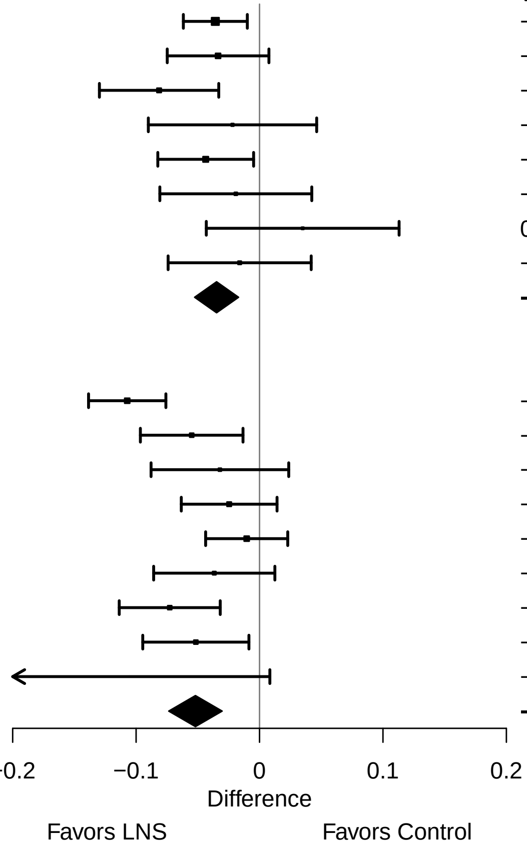

Supplemental figure 6C: Stunting prevalence difference

6C5: Stratified by Sanitation

Sanitation
(p−diff = 0.839)

Sanitation − Improved

**Country**	**Trial**	**N**	**N**	**PD (95% CI)**	**W**
Bangladesh	JiVitA−4 (34)	2838	1244	−0.04 (−0.06, −0.01)	0.16
Bangladesh	RDNS (35)	1663	815	−0.03 (−0.07, 0.01)	0.11
Bangladesh	WASH−B (36)	567	1104	−0.08 (−0.13, −0.03)	0.09
Burkina Faso	PROMIS (38)	863	914	−0.05 (−0.10, −0.01)	0.11
Burkina Faso	PROMIS CS (38)	430	439	−0.03 (−0.09, 0.02)	0.08
Ghana	GHANA (39)	98	96	−0.02 (−0.09, 0.05)	0.06
Ghana	iLiNS−DYAD−G (40)	347	692	−0.04 (−0.08, 0.00)	0.12
Haiti	HAITI (41)	149	149	−0.02 (−0.08, 0.04)	0.07
Mali	PROMIS (46)	506	506	−0.04 (−0.09, 0.01)	0.09
Mali	PROMIS CS (46)	952	969	−0.07 (−0.11, −0.03)	0.11
**I^2 = 0.00, Tau^2 = 0.00**		**8413**	**6928**	**−0.04 (−0.06, −0.03)**	

Sanitation − Unimproved

Burkina Faso	iLiNS−Zinc (37)	1952	664	−0.11 (−0.14, −0.08)	0.22
Kenya	WASH−B (42)	695	2253	−0.02 (−0.06, 0.01)	0.18
Madagascar	MAHAY (43)	1702	1682	−0.01 (−0.04, 0.02)	0.21
Malawi	iLiNS−DYAD−M (44)	220	444	0.03 (−0.04, 0.11)	0.08
Malawi	iLiNS−DOSE (45)	696	241	−0.02 (−0.07, 0.04)	0.12
Zimbabwe	SHINE (HIV−) (47)	892	880	−0.05 (−0.09, −0.01)	0.16
Zimbabwe	SHINE (HIV+) (48)	147	146	−0.11 (−0.22, 0.01)	0.04
**I^2 = 0.77, Tau^2 = 0.00**		**6304**	**6310**	**−0.04 (−0.08, 0.00)**	

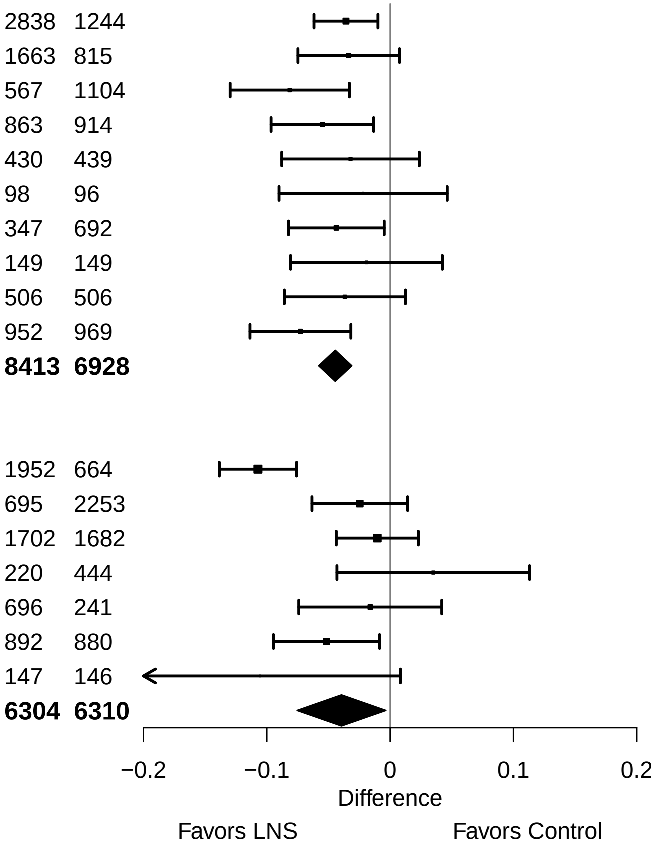

Supplemental figure 6C: Stunting prevalence difference

6C6: Stratified by Supplement duration

Supplement duration
(p−diff = 0.654)

Country	Trial	N	N	PD (95% CI)	W
**Supplement duration − 12m or less**					
Bangladesh	JiVitA−4 (34)	2838	1244	−0.04 (−0.06, −0.01)	0.13
Burkina Faso	iLiNS−Zinc (37)	1952	664	−0.11 (−0.14, −0.08)	0.12
Burkina Faso	PROMIS (38)	863	914	−0.05 (−0.10, −0.01)	0.09
Burkina Faso	PROMIS CS (38)	430	439	−0.03 (−0.09, 0.02)	0.07
Ghana	GHANA (39)	98	96	−0.02 (−0.09, 0.05)	0.05
Ghana	iLiNS−DYAD−G (40)	347	692	−0.04 (−0.08, 0.00)	0.10
Haiti	HAITI (41)	149	149	−0.02 (−0.08, 0.04)	0.06
Madagascar	MAHAY (43)	1702	1682	−0.01 (−0.04, 0.02)	0.11
Malawi	iLiNS−DYAD−M (44)	220	444	0.03 (−0.04, 0.11)	0.04
Malawi	iLiNS−DOSE (45)	696	241	−0.02 (−0.07, 0.04)	0.06
Zimbabwe	SHINE (HIV−) (47)	1880	1794	−0.07 (−0.10, −0.04)	0.12
Zimbabwe	SHINE (HIV+) (48)	337	330	−0.10 (−0.17, −0.02)	0.04
**I² = 0.65, Tau² = 0.00**		**11512**	**8689**	**−0.04 (−0.06, −0.02)**	
**Supplement duration − > 12m**					
Bangladesh	RDNS (35)	1663	815	−0.03 (−0.07, 0.01)	0.18
Bangladesh	WASH−B (36)	1158	3431	−0.07 (−0.10, −0.04)	0.23
Kenya	WASH−B (42)	1457	5137	−0.05 (−0.07, −0.02)	0.25
Mali	PROMIS (46)	506	506	−0.04 (−0.09, 0.01)	0.15
Mali	PROMIS CS (46)	952	969	−0.07 (−0.11, −0.03)	0.18
**I² = 0.00, Tau² = 0.00**		**5736**	**10858**	**−0.05 (−0.07, −0.04)**	

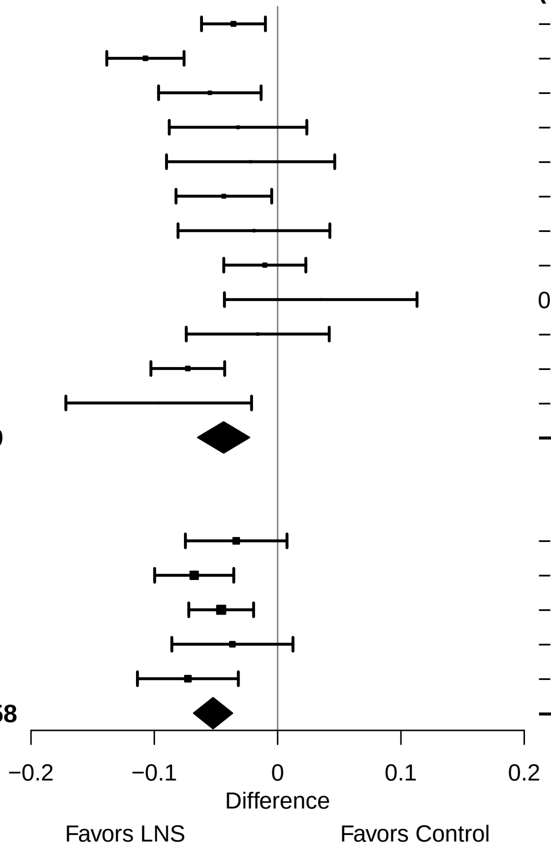

Supplemental figure 6C: Stunting prevalence difference

6C7: Stratified by Frequency of contact

Frequency of contact
(p−diff = 0.910)

Country	Trial	N	N	PD (95% CI)	W
**Frequency of contact − Monthly**					
Bangladesh	RDNS (35)	1663	815	−0.03 (−0.07, 0.01)	0.10
Burkina Faso	PROMIS (38)	863	914	−0.05 (−0.10, −0.01)	0.10
Burkina Faso	PROMIS CS (38)	430	439	−0.03 (−0.09, 0.02)	0.08
Haiti	HAITI (41)	149	149	−0.02 (−0.08, 0.04)	0.07
Kenya	WASH−B (42)	1457	5137	−0.05 (−0.07, −0.02)	0.15
Madagascar	MAHAY (43)	1702	1682	−0.01 (−0.04, 0.02)	0.13
Mali	PROMIS (46)	506	506	−0.04 (−0.09, 0.01)	0.09
Mali	PROMIS CS (46)	952	969	−0.07 (−0.11, −0.03)	0.11
Zimbabwe	SHINE (HIV−) (47)	1880	1794	−0.07 (−0.10, −0.04)	0.14
Zimbabwe	SHINE (HIV+) (48)	337	330	−0.10 (−0.17, −0.02)	0.05
**I² = 0.28, Tau² = 0.00**		**9939**	**12735**	**−0.05 (−0.06, −0.03)**	
**Frequency of contact − Weekly**					
Bangladesh	JiVitA−4 (34)	2838	1244	−0.04 (−0.06, −0.01)	0.21
Bangladesh	WASH−B (36)	1158	3431	−0.07 (−0.10, −0.04)	0.19
Burkina Faso	iLiNS−Zinc (37)	1952	664	−0.11 (−0.14, −0.08)	0.19
Ghana	GHANA (39)	98	96	−0.02 (−0.09, 0.05)	0.08
Ghana	iLiNS−DYAD−G (40)	347	692	−0.04 (−0.08, 0.00)	0.16
Malawi	iLiNS−DYAD−M (44)	220	444	0.03 (−0.04, 0.11)	0.07
Malawi	iLiNS−DOSE (45)	696	241	−0.02 (−0.07, 0.04)	0.10
**I² = 0.72, Tau² = 0.00**		**7309**	**6812**	**−0.04 (−0.08, −0.01)**	

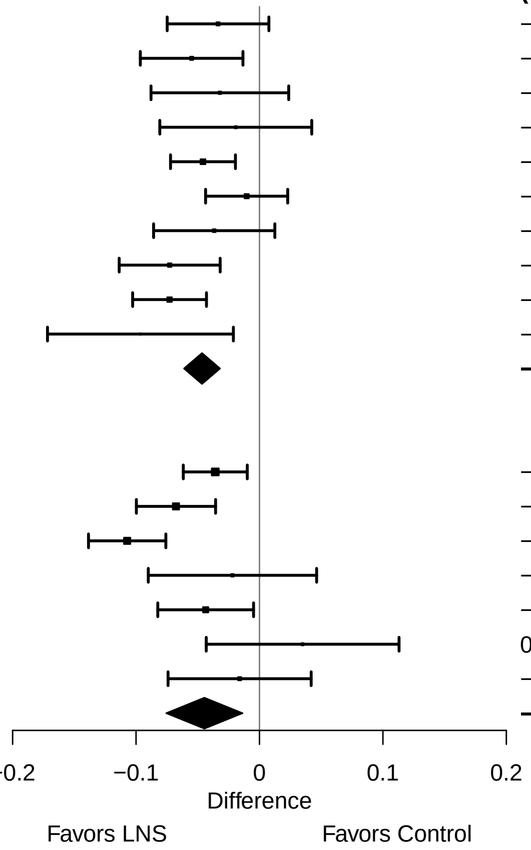

Supplemental figure 6C: Stunting prevalence difference

6C8: Stratified by Average SQ-LNS compliance

Average SQ−LNS compliance
(p−diff = 0.803)

Average SQ−LNS compliance − Low

Country	Trial	N	N	PD (95% CI)	W
Burkina Faso	PROMIS (38)	863	914	−0.05 (−0.10, −0.01)	0.13
Burkina Faso	PROMIS CS (38)	430	439	−0.03 (−0.09, 0.02)	0.09
Ghana	iLiNS−DYAD−G (40)	347	692	−0.04 (−0.08, 0.00)	0.14
Malawi	iLiNS−DYAD−M (44)	220	444	0.03 (−0.04, 0.11)	0.06
Malawi	iLiNS−DOSE (45)	696	241	−0.02 (−0.07, 0.04)	0.09
Mali	PROMIS (46)	506	506	−0.04 (−0.09, 0.01)	0.11
Mali	PROMIS CS (46)	952	969	−0.07 (−0.11, −0.03)	0.14
Zimbabwe	SHINE (HIV−) (47)	1880	1794	−0.07 (−0.10, −0.04)	0.18
Zimbabwe	SHINE (HIV+) (48)	337	330	−0.10 (−0.17, −0.02)	0.06
**I² = 0.30, Tau² = 0.00**		**6231**	**6329**	**−0.05 (−0.07, −0.03)**	

Average SQ−LNS compliance − High

Country	Trial	N	N	PD (95% CI)	W
Bangladesh	JiVitA−4 (34)	2838	1244	−0.04 (−0.06, −0.01)	0.19
Bangladesh	RDNS (35)	1663	815	−0.03 (−0.07, 0.01)	0.13
Bangladesh	WASH−B (36)	1158	3431	−0.07 (−0.10, −0.04)	0.17
Burkina Faso	iLiNS−Zinc (37)	1952	664	−0.11 (−0.14, −0.08)	0.17
Ghana	GHANA (39)	98	96	−0.02 (−0.09, 0.05)	0.07
Haiti	HAITI (41)	149	149	−0.02 (−0.08, 0.04)	0.08
Kenya	WASH−B (42)	1457	5137	−0.05 (−0.07, −0.02)	0.19
**I² = 0.65, Tau² = 0.00**		**9315**	**11536**	**−0.05 (−0.08, −0.03)**	

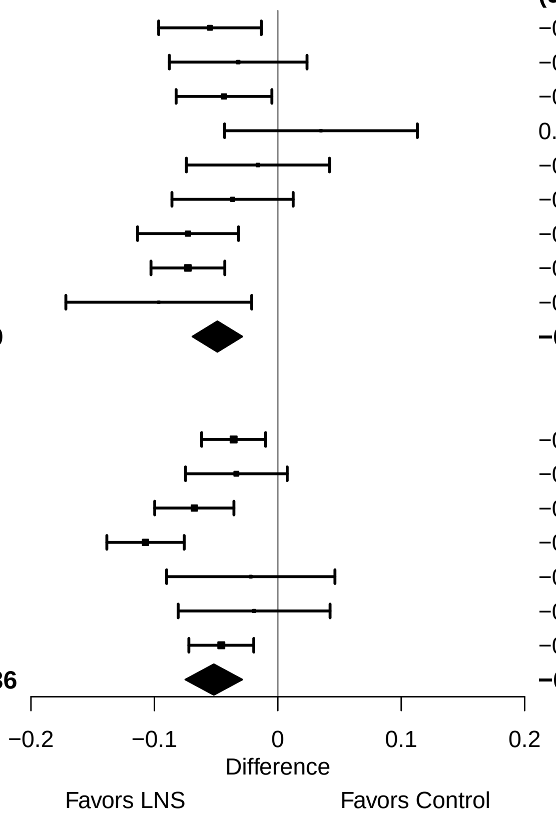

Supplemental figure 6D: Mean difference in WLZ

6D1: Stratified by Geographic region

Geographic region
(p−diff = 0.482)

Country	Trial	N	N	MD (95% CI)	W
**Geographic region − SEAR**					
Bangladesh	JiVitA−4 (34)	2783	1217	0.08 (0.03, 0.13)	0.37
Bangladesh	RDNS (35)	1661	815	0.07 (0.00, 0.15)	0.30
Bangladesh	WASH−B (36)	1156	3424	0.12 (0.05, 0.19)	0.32
**I² = 0.00, Tau² = 0.00**		**5600**	**5456**	**0.09 (0.06, 0.12)**	
**Geographic region − AFR**					
Burkina Faso	iLiNS−Zinc (37)	1952	664	0.18 (0.11, 0.26)	0.11
Burkina Faso	PROMIS (38)	856	907	0.10 (−0.03, 0.22)	0.07
Burkina Faso	PROMIS CS (38)	430	436	−0.11 (−0.24, 0.02)	0.07
Ghana	GHANA (39)	98	96	0.21 (−0.13, 0.54)	0.02
Ghana	iLiNS−DYAD−G (40)	347	692	0.09 (−0.04, 0.22)	0.07
Kenya	WASH−B (42)	1455	5118	0.06 (0.00, 0.13)	0.12
Madagascar	MAHAY (43)	1700	1682	0.00 (−0.11, 0.11)	0.08
Malawi	iLiNS−DYAD−M (44)	220	444	−0.05 (−0.21, 0.11)	0.06
Malawi	iLiNS−DOSE (45)	696	241	0.01 (−0.12, 0.14)	0.07
Mali	PROMIS (46)	499	502	0.10 (−0.02, 0.23)	0.08
Mali	PROMIS CS (46)	944	959	0.16 (0.06, 0.26)	0.09
Zimbabwe	SHINE (HIV−) (47)	1869	1784	0.08 (0.00, 0.15)	0.11
Zimbabwe	SHINE (HIV+) (48)	336	328	−0.14 (−0.32, 0.04)	0.05
**I² = 0.61, Tau² = 0.01**		**11402**	**13853**	**0.06 (0.00, 0.11)**	

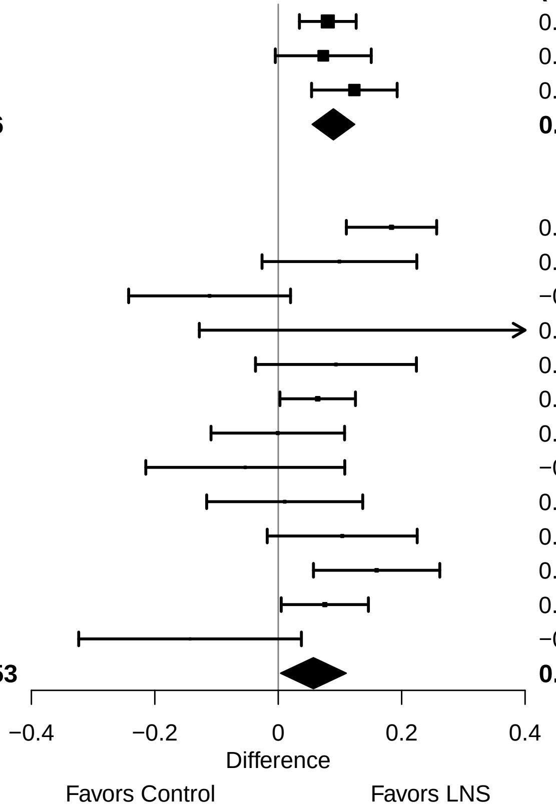

Supplemental figure 6D: Mean difference in WLZ

6D2: Stratified by Stunting burden

Stunting burden
(p−diff = 0.264)

Country	Trial	N	N	MD (95% CI)	W
**Stunting burden − Less than 35%**					
Burkina Faso	PROMIS (38)	856	907	0.10 (−0.03, 0.22)	0.16
Burkina Faso	PROMIS CS (38)	430	436	−0.11 (−0.24, 0.02)	0.15
Ghana	GHANA (39)	98	96	0.21 (−0.13, 0.54)	0.04
Ghana	iLiNS−DYAD−G (40)	347	692	0.09 (−0.04, 0.22)	0.15
Haiti	HAITI (41)	148	149	0.09 (−0.07, 0.24)	0.12
Kenya	WASH−B (42)	1455	5118	0.06 (0.00, 0.13)	0.27
Malawi	iLiNS−DYAD−M (44)	220	444	−0.05 (−0.21, 0.11)	0.12
**I² = 0.38, Tau² = 0.00**		**3554**	**7842**	**0.04 (−0.02, 0.11)**	
**Stunting burden − More than 35%**					
Bangladesh	JiVitA−4 (34)	2783	1217	0.08 (0.03, 0.13)	0.14
Bangladesh	RDNS (35)	1661	815	0.07 (0.00, 0.15)	0.11
Bangladesh	WASH−B (36)	1156	3424	0.12 (0.05, 0.19)	0.12
Burkina Faso	iLiNS−Zinc (37)	1952	664	0.18 (0.11, 0.26)	0.12
Madagascar	MAHAY (43)	1700	1682	0.00 (−0.11, 0.11)	0.09
Malawi	iLiNS−DOSE (45)	696	241	0.01 (−0.12, 0.14)	0.08
Mali	PROMIS (46)	499	502	0.10 (−0.02, 0.23)	0.08
Mali	PROMIS CS (46)	944	959	0.16 (0.06, 0.26)	0.09
Zimbabwe	SHINE (HIV−) (47)	1869	1784	0.08 (0.00, 0.15)	0.12
Zimbabwe	SHINE (HIV+) (48)	336	328	−0.14 (−0.32, 0.04)	0.05
**I² = 0.55, Tau² = 0.00**		**13596**	**11616**	**0.08 (0.03, 0.13)**	

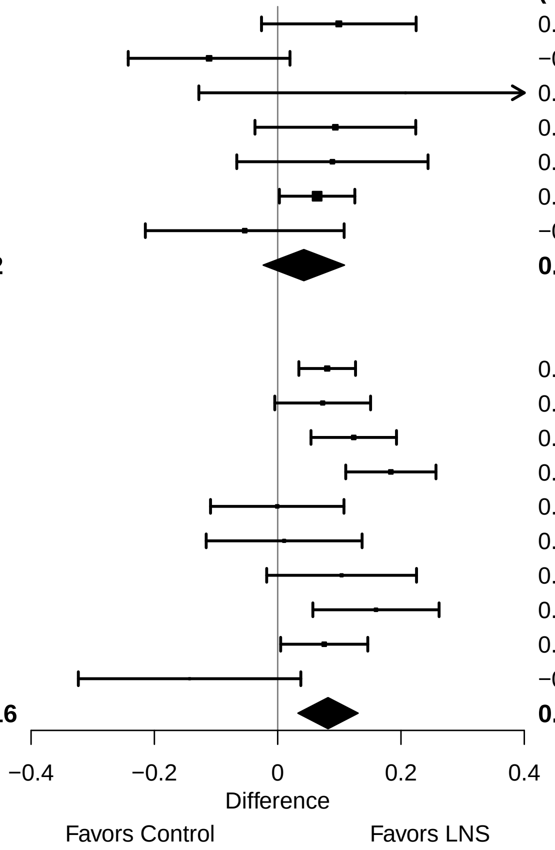

Supplemental figure 6D: Mean difference in WLZ

6D3: Stratified by Malaria prevalence

Malaria prevalence
(p−diff = 0.629)

Country	Trial	N	N	MD (95% CI)	W
**Malaria prevalence − Less than 10%**					
Bangladesh	JiVitA−4 (34)	2783	1217	0.08 (0.03, 0.13)	0.17
Bangladesh	RDNS (35)	1661	815	0.07 (0.00, 0.15)	0.14
Bangladesh	WASH−B (36)	1156	3424	0.12 (0.05, 0.19)	0.15
Haiti	HAITI (41)	148	149	0.09 (−0.07, 0.24)	0.07
Kenya	WASH−B (42)	1455	5118	0.06 (0.00, 0.13)	0.16
Madagascar	MAHAY (43)	1700	1682	0.00 (−0.11, 0.11)	0.11
Zimbabwe	SHINE (HIV−) (47)	1869	1784	0.08 (0.00, 0.15)	0.15
Zimbabwe	SHINE (HIV+) (48)	336	328	−0.14 (−0.32, 0.04)	0.06
**I² = 0.27, Tau² = 0.00**		**11108**	**14517**	**0.07 (0.03, 0.11)**	
**Malaria prevalence − At least 10%**					
Burkina Faso	iLiNS−Zinc (37)	1952	664	0.18 (0.11, 0.26)	0.18
Burkina Faso	PROMIS (38)	856	907	0.10 (−0.03, 0.22)	0.12
Burkina Faso	PROMIS CS (38)	430	436	−0.11 (−0.24, 0.02)	0.11
Ghana	GHANA (39)	98	96	0.21 (−0.13, 0.54)	0.03
Ghana	iLiNS−DYAD−G (40)	347	692	0.09 (−0.04, 0.22)	0.11
Malawi	iLiNS−DYAD−M (44)	220	444	−0.05 (−0.21, 0.11)	0.09
Malawi	iLiNS−DOSE (45)	696	241	0.01 (−0.12, 0.14)	0.11
Mali	PROMIS (46)	499	502	0.10 (−0.02, 0.23)	0.12
Mali	PROMIS CS (46)	944	959	0.16 (0.06, 0.26)	0.14
**I² = 0.64, Tau² = 0.01**		**6042**	**4941**	**0.08 (0.01, 0.14)**	

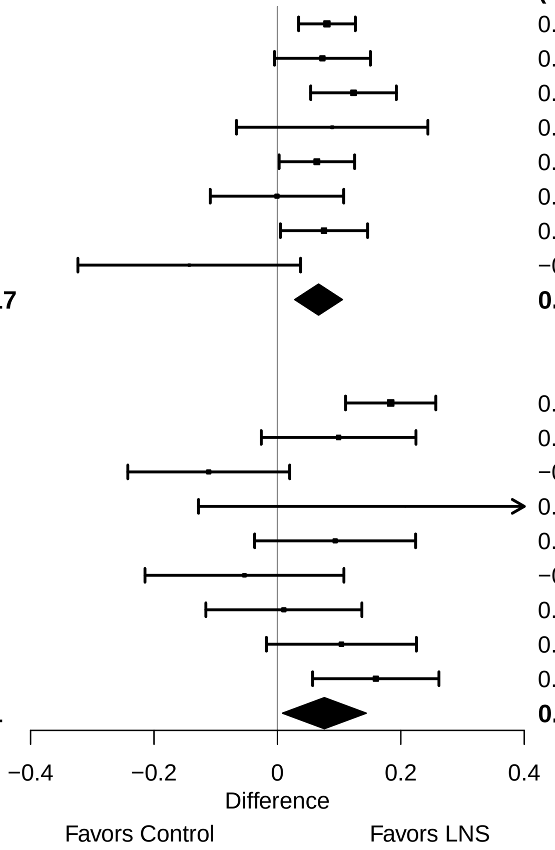

Supplemental figure 6D: Mean difference in WLZ

6D4: Stratified by Source water quality

Source water quality
(p−diff = 0.707)

Country	Trial	N	N	MD (95% CI)	W
**Source water quality − Improved**					
Bangladesh	JiVitA−4 (34)	2783	1217	0.08 (0.03, 0.13)	0.23
Bangladesh	RDNS (35)	1661	815	0.07 (0.00, 0.15)	0.18
Bangladesh	WASH−B (36)	566	1101	0.16 (0.05, 0.27)	0.14
Ghana	GHANA (39)	98	96	0.21 (−0.13, 0.54)	0.03
Ghana	iLiNS−DYAD−G (40)	347	692	0.09 (−0.04, 0.22)	0.12
Haiti	HAITI (41)	148	149	0.09 (−0.07, 0.24)	0.09
Malawi	iLiNS−DYAD−M (44)	220	444	−0.05 (−0.21, 0.11)	0.09
Malawi	iLiNS−DOSE (45)	696	241	0.01 (−0.12, 0.14)	0.12
**I² = 0.00, Tau² = 0.00**		**6519**	**4755**	**0.08 (0.05, 0.11)**	
**Source water quality − Unimproved**					
Burkina Faso	iLiNS−Zinc (37)	1952	664	0.18 (0.11, 0.26)	0.16
Burkina Faso	PROMIS (38)	856	907	0.10 (−0.03, 0.22)	0.10
Burkina Faso	PROMIS CS (38)	430	436	−0.11 (−0.24, 0.02)	0.10
Kenya	WASH−B (42)	694	2244	0.03 (−0.04, 0.11)	0.15
Madagascar	MAHAY (43)	1700	1682	0.00 (−0.11, 0.11)	0.12
Mali	PROMIS (46)	499	502	0.10 (−0.02, 0.23)	0.10
Mali	PROMIS CS (46)	944	959	0.16 (0.06, 0.26)	0.12
Zimbabwe	SHINE (HIV−) (47)	887	876	0.02 (−0.09, 0.13)	0.12
Zimbabwe	SHINE (HIV+) (48)	147	146	−0.15 (−0.40, 0.10)	0.04
**I² = 0.69, Tau² = 0.01**		**8109**	**8416**	**0.05 (−0.01, 0.12)**	

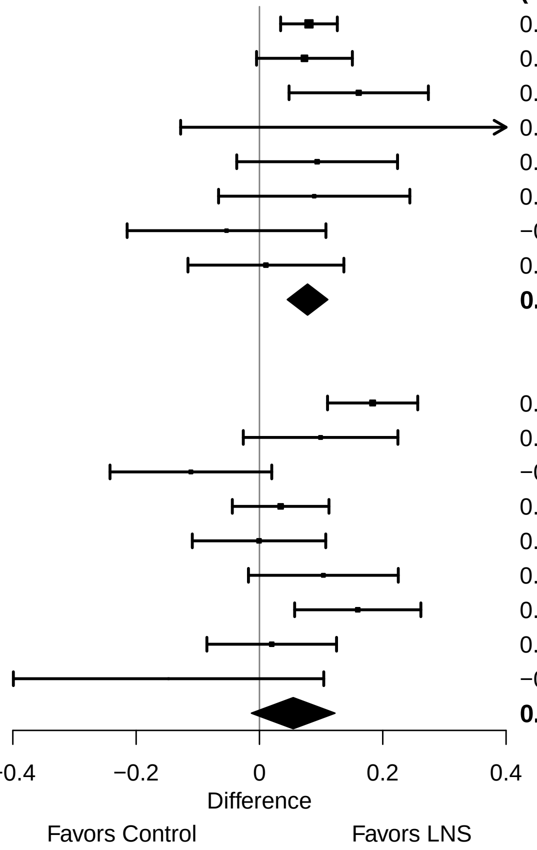

Supplemental figure 6D: Mean difference in WLZ

6D5: Stratified by Sanitation

Sanitation
(p−diff = 0.211)

Country	Trial	N	N	MD (95% CI)	W
**Sanitation − Improved**					
Bangladesh	JiVitA−4 (34)	2783	1217	0.08 (0.03, 0.13)	0.18
Bangladesh	RDNS (35)	1661	815	0.07 (0.00, 0.15)	0.14
Bangladesh	WASH−B (36)	566	1101	0.16 (0.05, 0.27)	0.10
Burkina Faso	PROMIS (38)	856	907	0.10 (−0.03, 0.22)	0.09
Burkina Faso	PROMIS CS (38)	430	436	−0.11 (−0.24, 0.02)	0.09
Ghana	GHANA (39)	98	96	0.21 (−0.13, 0.54)	0.02
Ghana	iLiNS−DYAD−G (40)	347	692	0.09 (−0.04, 0.22)	0.09
Haiti	HAITI (41)	148	149	0.09 (−0.07, 0.24)	0.07
Mali	PROMIS (46)	499	502	0.10 (−0.02, 0.23)	0.10
Mali	PROMIS CS (46)	944	959	0.16 (0.06, 0.26)	0.11
**I² = 0.32, Tau² = 0.00**		**8332**	**6874**	**0.09 (0.04, 0.13)**	
**Sanitation − Unimproved**					
Burkina Faso	iLiNS−Zinc (37)	1952	664	0.18 (0.11, 0.26)	0.21
Kenya	WASH−B (42)	694	2244	0.03 (−0.04, 0.11)	0.20
Madagascar	MAHAY (43)	1700	1682	0.00 (−0.11, 0.11)	0.15
Malawi	iLiNS−DYAD−M (44)	220	444	−0.05 (−0.21, 0.11)	0.10
Malawi	iLiNS−DOSE (45)	696	241	0.01 (−0.12, 0.14)	0.13
Zimbabwe	SHINE (HIV−) (47)	887	876	0.02 (−0.09, 0.13)	0.16
Zimbabwe	SHINE (HIV+) (48)	147	146	−0.15 (−0.40, 0.10)	0.05
**I² = 0.67, Tau² = 0.00**		**6296**	**6297**	**0.03 (−0.04, 0.10)**	

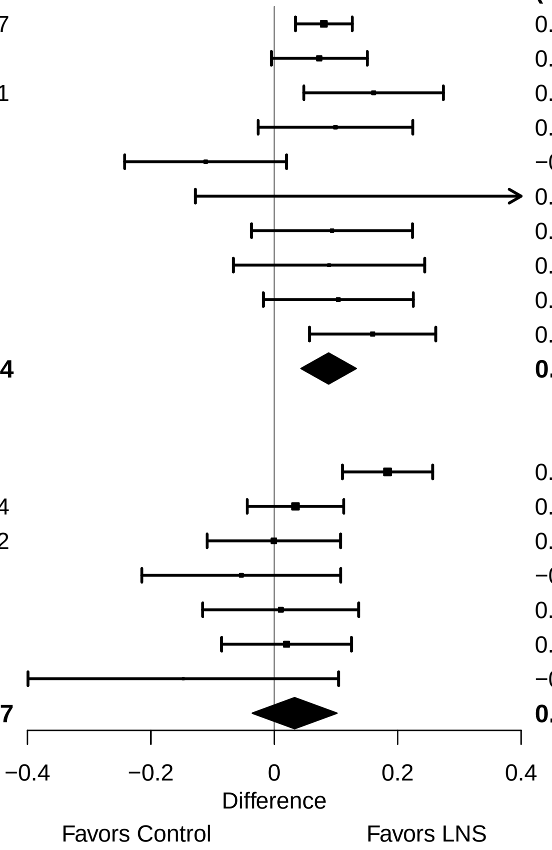

Supplemental figure 6D: Mean difference in WLZ

6D6: Stratified by Supplement duration

Supplement duration
(p−diff = 0.207)

Supplement duration − 12m or less

Country	Trial	N	N	MD (95% CI)	W
Bangladesh	JiVitA−4 (34)	2783	1217	0.08 (0.03, 0.13)	0.15
Burkina Faso	iLiNS−Zinc (37)	1952	664	0.18 (0.11, 0.26)	0.12
Burkina Faso	PROMIS (38)	856	907	0.10 (−0.03, 0.22)	0.08
Burkina Faso	PROMIS CS (38)	430	436	−0.11 (−0.24, 0.02)	0.08
Ghana	GHANA (39)	98	96	0.21 (−0.13, 0.54)	0.02
Ghana	iLiNS−DYAD−G (40)	347	692	0.09 (−0.04, 0.22)	0.08
Haiti	HAITI (41)	148	149	0.09 (−0.07, 0.24)	0.06
Madagascar	MAHAY (43)	1700	1682	0.00 (−0.11, 0.11)	0.09
Malawi	iLiNS−DYAD−M (44)	220	444	−0.05 (−0.21, 0.11)	0.06
Malawi	iLiNS−DOSE (45)	696	241	0.01 (−0.12, 0.14)	0.08
Zimbabwe	SHINE (HIV−) (47)	1869	1784	0.08 (0.00, 0.15)	0.13
Zimbabwe	SHINE (HIV+) (48)	336	328	−0.14 (−0.32, 0.04)	0.05
**I^2 = 0.61, Tau^2 = 0.01**		**11435**	**8640**	**0.05 (−0.01, 0.10)**	

Supplement duration − > 12m

Country	Trial	N	N	MD (95% CI)	W
Bangladesh	RDNS (35)	1661	815	0.07 (0.00, 0.15)	0.21
Bangladesh	WASH−B (36)	1156	3424	0.12 (0.05, 0.19)	0.23
Kenya	WASH−B (42)	1455	5118	0.06 (0.00, 0.13)	0.24
Mali	PROMIS (46)	499	502	0.10 (−0.02, 0.23)	0.15
Mali	PROMIS CS (46)	944	959	0.16 (0.06, 0.26)	0.17
**I^2 = 0.00, Tau^2 = 0.00**		**5715**	**10818**	**0.10 (0.06, 0.13)**	

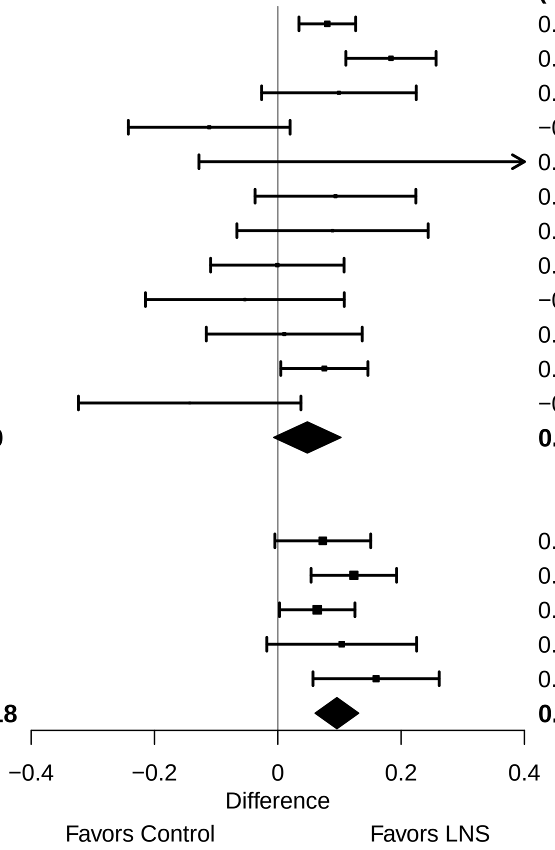

Supplemental figure 6D: Mean difference in WLZ

6D7: Stratified by Frequency of contact

Frequency of contact
(p−diff = 0.251)

Country	Trial	N	N	MD (95% CI)	W
**Frequency of contact − Monthly**					
Bangladesh	RDNS (35)	1661	815	0.07 (0.00, 0.15)	0.13
Burkina Faso	PROMIS (38)	856	907	0.10 (−0.03, 0.22)	0.09
Burkina Faso	PROMIS CS (38)	430	436	−0.11 (−0.24, 0.02)	0.08
Haiti	HAITI (41)	148	149	0.09 (−0.07, 0.24)	0.07
Kenya	WASH−B (42)	1455	5118	0.06 (0.00, 0.13)	0.15
Madagascar	MAHAY (43)	1700	1682	0.00 (−0.11, 0.11)	0.10
Mali	PROMIS (46)	499	502	0.10 (−0.02, 0.23)	0.09
Mali	PROMIS CS (46)	944	959	0.16 (0.06, 0.26)	0.11
Zimbabwe	SHINE (HIV−) (47)	1869	1784	0.08 (0.00, 0.15)	0.14
Zimbabwe	SHINE (HIV+) (48)	336	328	−0.14 (−0.32, 0.04)	0.06
**I² = 0.49, Tau² = 0.00**		**9898**	**12680**	**0.05 (0.00, 0.11)**	
**Frequency of contact − Weekly**					
Bangladesh	JiVitA−4 (34)	2783	1217	0.08 (0.03, 0.13)	0.23
Bangladesh	WASH−B (36)	1156	3424	0.12 (0.05, 0.19)	0.20
Burkina Faso	iLiNS−Zinc (37)	1952	664	0.18 (0.11, 0.26)	0.20
Ghana	GHANA (39)	98	96	0.21 (−0.13, 0.54)	0.03
Ghana	iLiNS−DYAD−G (40)	347	692	0.09 (−0.04, 0.22)	0.12
Malawi	iLiNS−DYAD−M (44)	220	444	−0.05 (−0.21, 0.11)	0.09
Malawi	iLiNS−DOSE (45)	696	241	0.01 (−0.12, 0.14)	0.13
**I² = 0.50, Tau² = 0.00**		**7252**	**6778**	**0.09 (0.04, 0.15)**	

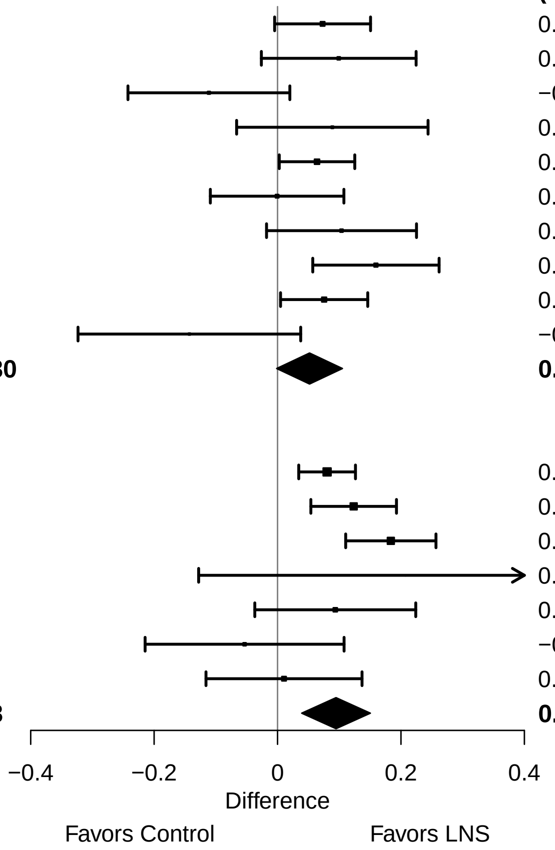

Supplemental figure 6D: Mean difference in WLZ

6D8: Stratified by Average SQ-LNS compliance

Average SQ−LNS compliance
(p−diff = 0.101)

Average SQ−LNS compliance − Low

**Country**	**Trial**	**N**	**N**	**MD (95% CI)**	**W**
Burkina Faso	PROMIS (38)	856	907	0.10 (−0.03, 0.22)	0.11
Burkina Faso	PROMIS CS (38)	430	436	−0.11 (−0.24, 0.02)	0.10
Ghana	iLiNS−DYAD−G (40)	347	692	0.09 (−0.04, 0.22)	0.11
Malawi	iLiNS−DYAD−M (44)	220	444	−0.05 (−0.21, 0.11)	0.08
Malawi	iLiNS−DOSE (45)	696	241	0.01 (−0.12, 0.14)	0.11
Mali	PROMIS (46)	499	502	0.10 (−0.02, 0.23)	0.11
Mali	PROMIS CS (46)	944	959	0.16 (0.06, 0.26)	0.13
Zimbabwe	SHINE (HIV−) (47)	1869	1784	0.08 (0.00, 0.15)	0.17
Zimbabwe	SHINE (HIV+) (48)	336	328	−0.14 (−0.32, 0.04)	0.07
**I² = 0.57, Tau² = 0.01**		**6197**	**6293**	**0.04 (−0.03, 0.10)**	

Average SQ−LNS compliance − High

**Country**	**Trial**	**N**	**N**	**MD (95% CI)**	**W**
Bangladesh	JiVitA−4 (34)	2783	1217	0.08 (0.03, 0.13)	0.20
Bangladesh	RDNS (35)	1661	815	0.07 (0.00, 0.15)	0.16
Bangladesh	WASH−B (36)	1156	3424	0.12 (0.05, 0.19)	0.17
Burkina Faso	iLiNS−Zinc (37)	1952	664	0.18 (0.11, 0.26)	0.17
Ghana	GHANA (39)	98	96	0.21 (−0.13, 0.54)	0.03
Haiti	HAITI (41)	148	149	0.09 (−0.07, 0.24)	0.09
Kenya	WASH−B (42)	1455	5118	0.06 (0.00, 0.13)	0.18
**I² = 0.28, Tau² = 0.00**		**9253**	**11483**	**0.10 (0.07, 0.13)**	

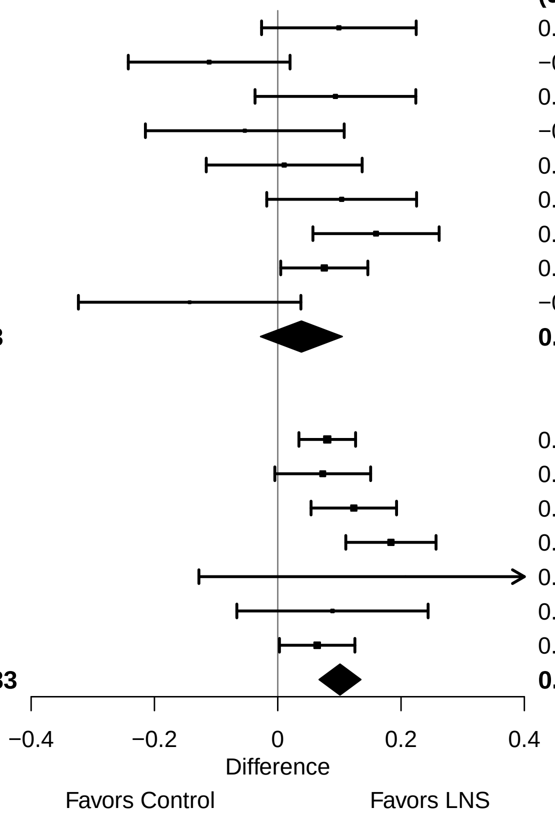

Supplemental figure 6E: Wasting prevalence ratio

6E1: Stratified by Geographic region

Geographic region
(p−diff = 0.973)

Country	Trial	N	N	PR (95% CI)	W
**Geographic region − SEAR**					
Bangladesh	JiVitA−4 (34)	2783	1217	0.91 (0.78, 1.05)	0.49
Bangladesh	RDNS (35)	1661	815	0.87 (0.70, 1.07)	0.23
Bangladesh	WASH−B (36)	1156	3424	0.79 (0.65, 0.96)	0.28
**I² = 0.00, Tau² = 0.00**		**5600**	**5456**	**0.86 (0.78, 0.96)**	
**Geographic region − AFR**					
Burkina Faso	iLiNS−Zinc (37)	1952	664	0.69 (0.54, 0.87)	0.24
Burkina Faso	PROMIS (38)	856	907	0.86 (0.59, 1.27)	0.09
Burkina Faso	PROMIS CS (38)	430	436	1.03 (0.72, 1.49)	0.10
Ghana	GHANA (39)	98	96	0.87 (0.35, 2.16)	0.02
Ghana	iLiNS−DYAD−G (40)	347	692	0.84 (0.52, 1.37)	0.06
Kenya	WASH−B (42)	1455	5118	0.88 (0.53, 1.45)	0.05
Madagascar	MAHAY (43)	1700	1682	0.96 (0.67, 1.38)	0.10
Malawi	iLiNS−DYAD−M (44)	220	444	0.94 (0.39, 2.28)	0.02
Malawi	iLiNS−DOSE (45)	696	241	1.37 (0.78, 2.40)	0.04
Mali	PROMIS (46)	499	502	0.61 (0.15, 2.42)	0.01
Mali	PROMIS CS (46)	944	959	0.83 (0.63, 1.10)	0.17
Zimbabwe	SHINE (HIV−) (47)	1869	1784	0.89 (0.60, 1.34)	0.08
Zimbabwe	SHINE (HIV+) (48)	336	328	1.38 (0.65, 2.96)	0.02
**I² = 0.00, Tau² = 0.00**		**11402**	**13853**	**0.86 (0.77, 0.97)**	

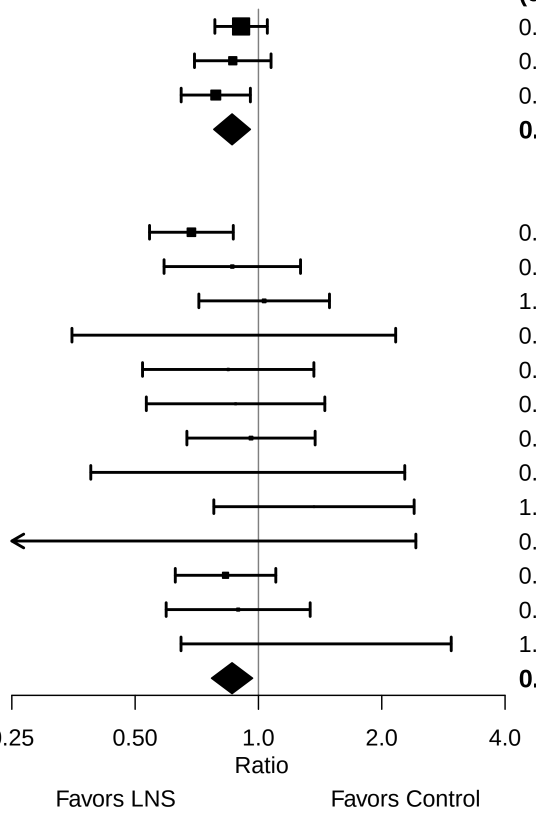

Supplemental figure 6E: Wasting prevalence ratio

6E2: Stratified by Stunting burden

Stunting burden
(p−diff = 0.532)

Country	Trial	N	N	PR (95% CI)	W
**Stunting burden − Less than 35%**					
Burkina Faso	PROMIS (38)	856	907	0.86 (0.59, 1.27)	0.27
Burkina Faso	PROMIS CS (38)	430	436	1.03 (0.72, 1.49)	0.29
Ghana	GHANA (39)	98	96	0.87 (0.35, 2.16)	0.05
Ghana	iLiNS−DYAD−G (40)	347	692	0.84 (0.52, 1.37)	0.17
Haiti	HAITI (41)	148	149	0.94 (0.13, 6.52)	0.01
Kenya	WASH−B (42)	1455	5118	0.88 (0.53, 1.45)	0.16
Malawi	iLiNS−DYAD−M (44)	220	444	0.94 (0.39, 2.28)	0.05
**I² = 0.00, Tau² = 0.00**		**3554**	**7842**	**0.91 (0.75, 1.12)**	
**Stunting burden − More than 35%**					
Bangladesh	JiVitA−4 (34)	2783	1217	0.91 (0.78, 1.05)	0.32
Bangladesh	RDNS (35)	1661	815	0.87 (0.70, 1.07)	0.15
Bangladesh	WASH−B (36)	1156	3424	0.79 (0.65, 0.96)	0.18
Burkina Faso	iLiNS−Zinc (37)	1952	664	0.69 (0.54, 0.87)	0.13
Madagascar	MAHAY (43)	1700	1682	0.96 (0.67, 1.38)	0.05
Malawi	iLiNS−DOSE (45)	696	241	1.37 (0.78, 2.40)	0.02
Mali	PROMIS (46)	499	502	0.61 (0.15, 2.42)	0.00
Mali	PROMIS CS (46)	944	959	0.83 (0.63, 1.10)	0.09
Zimbabwe	SHINE (HIV−) (47)	1869	1784	0.89 (0.60, 1.34)	0.04
Zimbabwe	SHINE (HIV+) (48)	336	328	1.38 (0.65, 2.96)	0.01
**I² = 0.06, Tau² = 0.00**		**13596**	**11616**	**0.85 (0.78, 0.93)**	

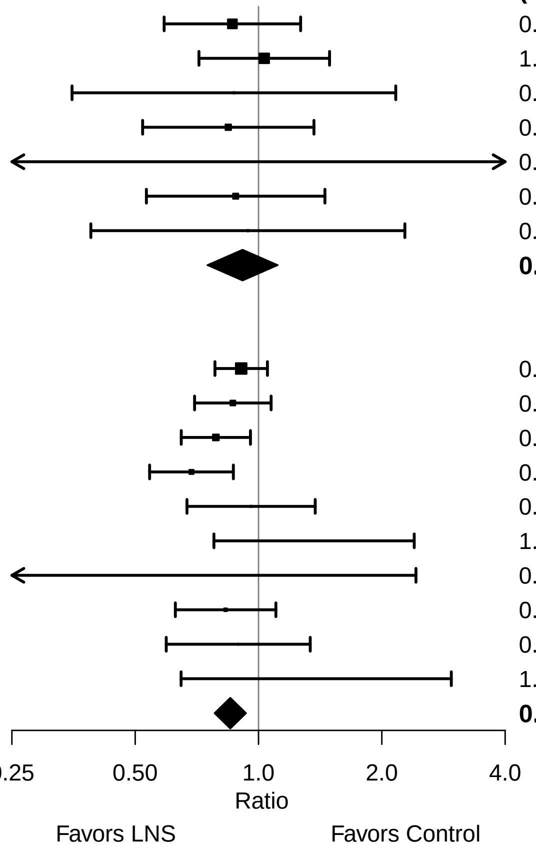

Supplemental figure 6E: Wasting prevalence ratio

6E3: Stratified by Malaria prevalence

Malaria prevalence
(p−diff = 0.533)

Country	Trial	N	N	PR (95% CI)	W
**Malaria prevalence − Less than 10%**					
Bangladesh	JiVitA−4 (34)	2783	1217	0.91 (0.78, 1.05)	0.40
Bangladesh	RDNS (35)	1661	815	0.87 (0.70, 1.07)	0.19
Bangladesh	WASH−B (36)	1156	3424	0.79 (0.65, 0.96)	0.23
Haiti	HAITI (41)	148	149	0.94 (0.13, 6.52)	0.00
Kenya	WASH−B (42)	1455	5118	0.88 (0.53, 1.45)	0.03
Madagascar	MAHAY (43)	1700	1682	0.96 (0.67, 1.38)	0.07
Zimbabwe	SHINE (HIV−) (47)	1869	1784	0.89 (0.60, 1.34)	0.05
Zimbabwe	SHINE (HIV+) (48)	336	328	1.38 (0.65, 2.96)	0.02
**I² = 0.00, Tau² = 0.00**		**11108**	**14517**	**0.88 (0.80, 0.96)**	
**Malaria prevalence − At least 10%**					
Burkina Faso	iLiNS−Zinc (37)	1952	664	0.69 (0.54, 0.87)	0.33
Burkina Faso	PROMIS (38)	856	907	0.86 (0.59, 1.27)	0.12
Burkina Faso	PROMIS CS (38)	430	436	1.03 (0.72, 1.49)	0.13
Ghana	GHANA (39)	98	96	0.87 (0.35, 2.16)	0.02
Ghana	iLiNS−DYAD−G (40)	347	692	0.84 (0.52, 1.37)	0.08
Malawi	iLiNS−DYAD−M (44)	220	444	0.94 (0.39, 2.28)	0.02
Malawi	iLiNS−DOSE (45)	696	241	1.37 (0.78, 2.40)	0.06
Mali	PROMIS (46)	499	502	0.61 (0.15, 2.42)	0.01
Mali	PROMIS CS (46)	944	959	0.83 (0.63, 1.10)	0.23
**I² = 0.00, Tau² = 0.00**		**6042**	**4941**	**0.83 (0.73, 0.95)**	

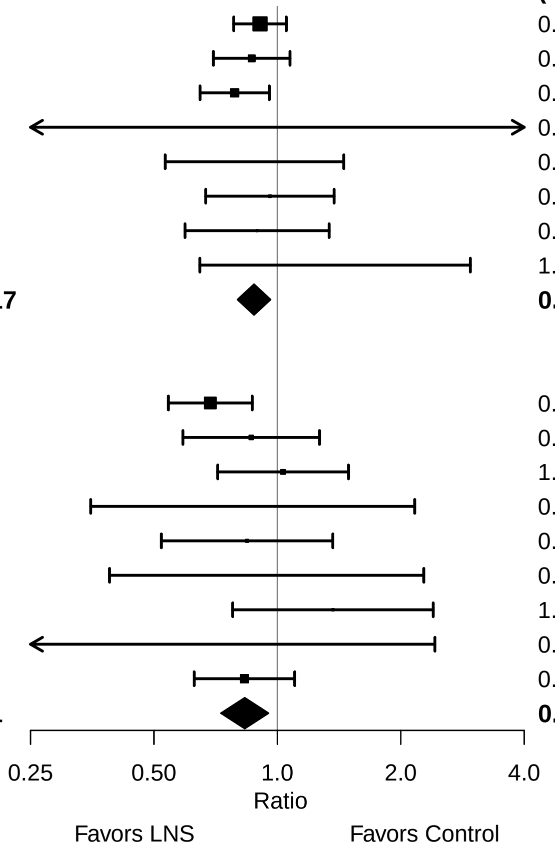

Supplemental figure 6E: Wasting prevalence ratio

6E4: Stratified by Source water quality

Source water quality
(p−diff = 0.371)

Country	Trial	N	N	PR (95% CI)	W
**Source water quality − Improved**					
Bangladesh	JiVitA−4 (34)	2783	1217	0.91 (0.78, 1.05)	0.52
Bangladesh	RDNS (35)	1661	815	0.87 (0.70, 1.07)	0.24
Bangladesh	WASH−B (36)	566	1101	0.82 (0.61, 1.12)	0.12
Ghana	GHANA (39)	98	96	0.87 (0.35, 2.16)	0.01
Ghana	iLiNS−DYAD−G (40)	347	692	0.84 (0.52, 1.37)	0.05
Haiti	HAITI (41)	148	149	0.94 (0.13, 6.52)	0.00
Malawi	iLiNS−DYAD−M (44)	220	444	0.94 (0.39, 2.28)	0.01
Malawi	iLiNS−DOSE (45)	696	241	1.37 (0.78, 2.40)	0.04
**I² = 0.00, Tau² = 0.00**		**6519**	**4755**	**0.90 (0.81, 1.00)**	
**Source water quality − Unimproved**					
Burkina Faso	iLiNS−Zinc (37)	1952	664	0.69 (0.54, 0.87)	0.31
Burkina Faso	PROMIS (38)	856	907	0.86 (0.59, 1.27)	0.12
Burkina Faso	PROMIS CS (38)	430	436	1.03 (0.72, 1.49)	0.13
Kenya	WASH−B (42)	694	2244	0.83 (0.37, 1.90)	0.03
Madagascar	MAHAY (43)	1700	1682	0.96 (0.67, 1.38)	0.13
Mali	PROMIS (46)	499	502	0.61 (0.15, 2.42)	0.01
Mali	PROMIS CS (46)	944	959	0.83 (0.63, 1.10)	0.21
Zimbabwe	SHINE (HIV−) (47)	887	876	0.95 (0.56, 1.61)	0.06
Zimbabwe	SHINE (HIV+) (48)	147	146	0.99 (0.28, 3.54)	0.01
**I² = 0.00, Tau² = 0.00**		**8109**	**8416**	**0.83 (0.73, 0.95)**	

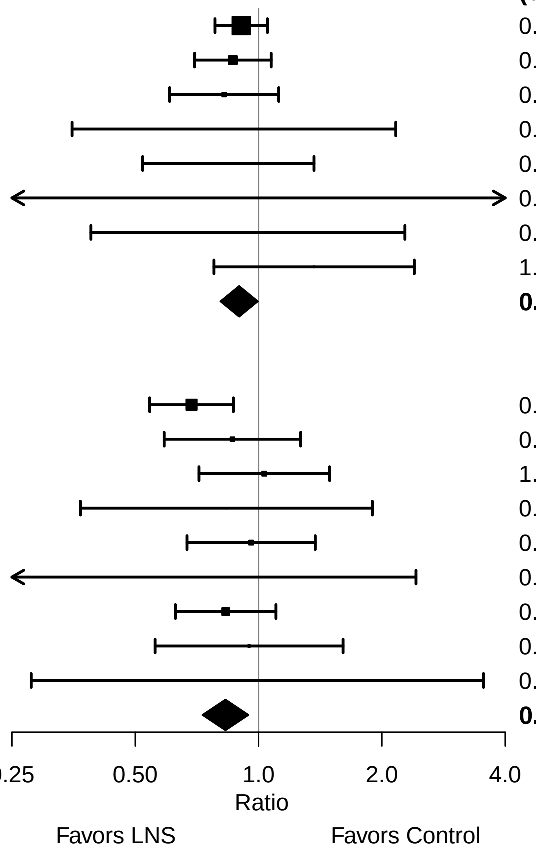

Supplemental figure 6E: Wasting prevalence ratio

6E5: Stratified by Sanitation

Sanitation
(p−diff = 0.532)

**Sanitation − Improved**					
**Country**	**Trial**	**N**	**N**	**PR (95% CI)**	**W**
Bangladesh	JiVitA−4 (34)	2783	1217	0.91 (0.78, 1.05)	0.41
Bangladesh	RDNS (35)	1661	815	0.87 (0.70, 1.07)	0.19
Bangladesh	WASH−B (36)	566	1101	0.82 (0.61, 1.12)	0.10
Burkina Faso	PROMIS (38)	856	907	0.86 (0.59, 1.27)	0.06
Burkina Faso	PROMIS CS (38)	430	436	1.03 (0.72, 1.49)	0.07
Ghana	GHANA (39)	98	96	0.87 (0.35, 2.16)	0.01
Ghana	iLiNS−DYAD−G (40)	347	692	0.84 (0.52, 1.37)	0.04
Haiti	HAITI (41)	148	149	0.94 (0.13, 6.52)	0.00
Mali	PROMIS (46)	499	502	0.61 (0.15, 2.42)	0.00
Mali	PROMIS CS (46)	944	959	0.83 (0.63, 1.10)	0.11
**I² = 0.00, Tau² = 0.00**		**8332**	**6874**	**0.88 (0.80, 0.97)**	
**Sanitation − Unimproved**					
Burkina Faso	iLiNS−Zinc (37)	1952	664	0.69 (0.54, 0.87)	0.50
Kenya	WASH−B (42)	694	2244	0.83 (0.37, 1.90)	0.04
Madagascar	MAHAY (43)	1700	1682	0.96 (0.67, 1.38)	0.21
Malawi	iLiNS−DYAD−M (44)	220	444	0.94 (0.39, 2.28)	0.04
Malawi	iLiNS−DOSE (45)	696	241	1.37 (0.78, 2.40)	0.09
Zimbabwe	SHINE (HIV−) (47)	887	876	0.95 (0.56, 1.61)	0.10
Zimbabwe	SHINE (HIV+) (48)	147	146	0.99 (0.28, 3.54)	0.02
**I² = 0.08, Tau² = 0.00**		**6296**	**6297**	**0.84 (0.70, 1.00)**	

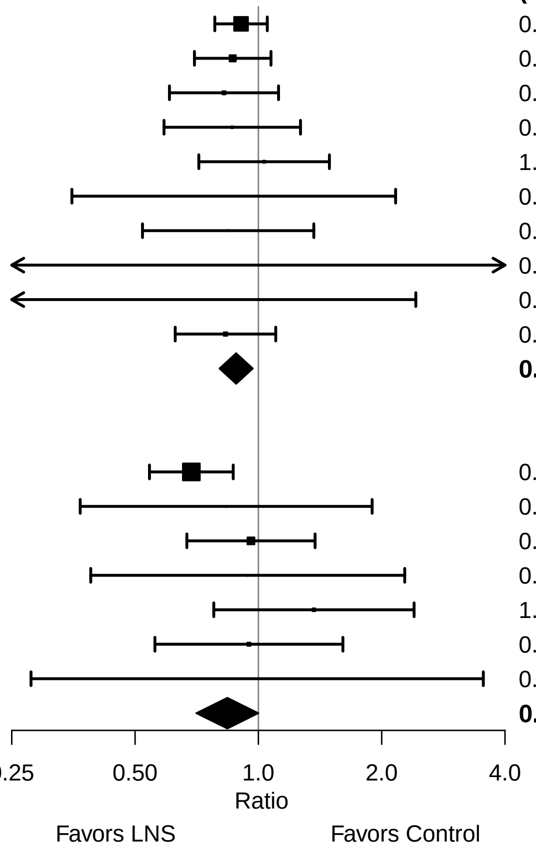

Supplemental figure 6E: Wasting prevalence ratio

6E6: Stratified by Supplement duration

Supplement duration
(p−diff = 0.368)

Country	Trial	N	N	PR (95% CI)	W
**Supplement duration − 12m or less**					
Bangladesh	JiVitA−4 (34)	2783	1217	0.91 (0.78, 1.05)	0.44
Burkina Faso	iLiNS−Zinc (37)	1952	664	0.69 (0.54, 0.87)	0.17
Burkina Faso	PROMIS (38)	856	907	0.86 (0.59, 1.27)	0.07
Burkina Faso	PROMIS CS (38)	430	436	1.03 (0.72, 1.49)	0.07
Ghana	GHANA (39)	98	96	0.87 (0.35, 2.16)	0.01
Ghana	iLiNS−DYAD−G (40)	347	692	0.84 (0.52, 1.37)	0.04
Haiti	HAITI (41)	148	149	0.94 (0.13, 6.52)	0.00
Madagascar	MAHAY (43)	1700	1682	0.96 (0.67, 1.38)	0.07
Malawi	iLiNS−DYAD−M (44)	220	444	0.94 (0.39, 2.28)	0.01
Malawi	iLiNS−DOSE (45)	696	241	1.37 (0.78, 2.40)	0.03
Zimbabwe	SHINE (HIV−) (47)	1869	1784	0.89 (0.60, 1.34)	0.06
Zimbabwe	SHINE (HIV+) (48)	336	328	1.38 (0.65, 2.96)	0.02
**I² = 0.00, Tau² = 0.00**		**11435**	**8640**	**0.89 (0.80, 0.98)**	
**Supplement duration − > 12m**					
Bangladesh	RDNS (35)	1661	815	0.87 (0.70, 1.07)	0.33
Bangladesh	WASH−B (36)	1156	3424	0.79 (0.65, 0.96)	0.41
Kenya	WASH−B (42)	1455	5118	0.88 (0.53, 1.45)	0.06
Mali	PROMIS (46)	499	502	0.61 (0.15, 2.42)	0.01
Mali	PROMIS CS (46)	944	959	0.83 (0.63, 1.10)	0.19
**I² = 0.00, Tau² = 0.00**		**5715**	**10818**	**0.82 (0.73, 0.93)**	

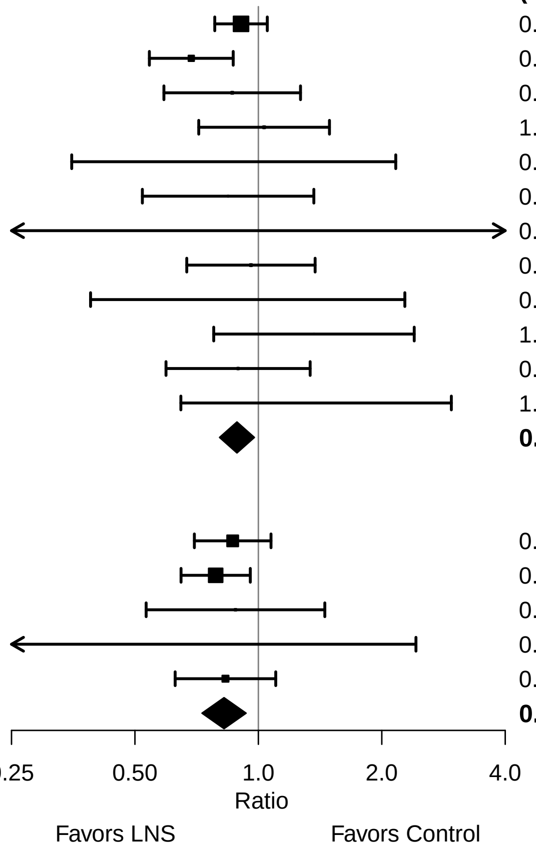

Supplemental figure 6E: Wasting prevalence ratio

6E7: Stratified by Frequency of contact

Frequency of contact
(p−diff = 0.399)

Country	Trial	N	N	PR (95% CI)	W
**Frequency of contact − Monthly**					
Bangladesh	RDNS (35)	1661	815	0.87 (0.70, 1.07)	0.31
Burkina Faso	PROMIS (38)	856	907	0.86 (0.59, 1.27)	0.10
Burkina Faso	PROMIS CS (38)	430	436	1.03 (0.72, 1.49)	0.11
Haiti	HAITI (41)	148	149	0.94 (0.13, 6.52)	0.00
Kenya	WASH−B (42)	1455	5118	0.88 (0.53, 1.45)	0.06
Madagascar	MAHAY (43)	1700	1682	0.96 (0.67, 1.38)	0.11
Mali	PROMIS (46)	499	502	0.61 (0.15, 2.42)	0.01
Mali	PROMIS CS (46)	944	959	0.83 (0.63, 1.10)	0.18
Zimbabwe	SHINE (HIV−) (47)	1869	1784	0.89 (0.60, 1.34)	0.09
Zimbabwe	SHINE (HIV+) (48)	336	328	1.38 (0.65, 2.96)	0.03
**I² = 0.00, Tau² = 0.00**		**9898**	**12680**	**0.90 (0.80, 1.01)**	
**Frequency of contact − Weekly**					
Bangladesh	JiVitA−4 (34)	2783	1217	0.91 (0.78, 1.05)	0.46
Bangladesh	WASH−B (36)	1156	3424	0.79 (0.65, 0.96)	0.26
Burkina Faso	iLiNS−Zinc (37)	1952	664	0.69 (0.54, 0.87)	0.18
Ghana	GHANA (39)	98	96	0.87 (0.35, 2.16)	0.01
Ghana	iLiNS−DYAD−G (40)	347	692	0.84 (0.52, 1.37)	0.04
Malawi	iLiNS−DYAD−M (44)	220	444	0.94 (0.39, 2.28)	0.01
Malawi	iLiNS−DOSE (45)	696	241	1.37 (0.78, 2.40)	0.03
**I² = 0.17, Tau² = 0.00**		**7252**	**6778**	**0.84 (0.74, 0.94)**	

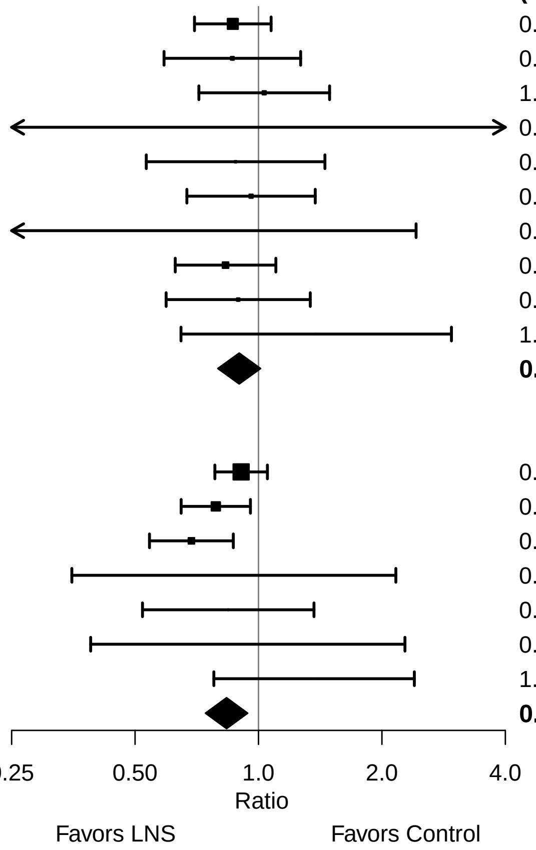

Supplemental figure 6E: Wasting prevalence ratio

6E8: Stratified by Average SQ-LNS compliance

Average SQ−LNS compliance
(p−diff = 0.231)

**Average SQ−LNS compliance − Low**				**PR**	
**Country**	**Trial**	**N**	**N**	**(95% CI)**	**W**
Burkina Faso	PROMIS (38)	856	907	0.86 (0.59, 1.27)	0.15
Burkina Faso	PROMIS CS (38)	430	436	1.03 (0.72, 1.49)	0.17
Ghana	iLiNS−DYAD−G (40)	347	692	0.84 (0.52, 1.37)	0.10
Malawi	iLiNS−DYAD−M (44)	220	444	0.94 (0.39, 2.28)	0.03
Malawi	iLiNS−DOSE (45)	696	241	1.37 (0.78, 2.40)	0.07
Mali	PROMIS (46)	499	502	0.61 (0.15, 2.42)	0.01
Mali	PROMIS CS (46)	944	959	0.83 (0.63, 1.10)	0.29
Zimbabwe	SHINE (HIV−) (47)	1869	1784	0.89 (0.60, 1.34)	0.14
Zimbabwe	SHINE (HIV+) (48)	336	328	1.38 (0.65, 2.96)	0.04
**I² = 0.00, Tau² = 0.00**		**6197**	**6293**	**0.93 (0.80, 1.08)**	
**Average SQ−LNS compliance − High**					
Bangladesh	JiVitA−4 (34)	2783	1217	0.91 (0.78, 1.05)	0.39
Bangladesh	RDNS (35)	1661	815	0.87 (0.70, 1.07)	0.18
Bangladesh	WASH−B (36)	1156	3424	0.79 (0.65, 0.96)	0.22
Burkina Faso	iLiNS−Zinc (37)	1952	664	0.69 (0.54, 0.87)	0.15
Ghana	GHANA (39)	98	96	0.87 (0.35, 2.16)	0.01
Haiti	HAITI (41)	148	149	0.94 (0.13, 6.52)	0.00
Kenya	WASH−B (42)	1455	5118	0.88 (0.53, 1.45)	0.03
**I² = 0.00, Tau² = 0.00**		**9253**	**11483**	**0.83 (0.76, 0.91)**	

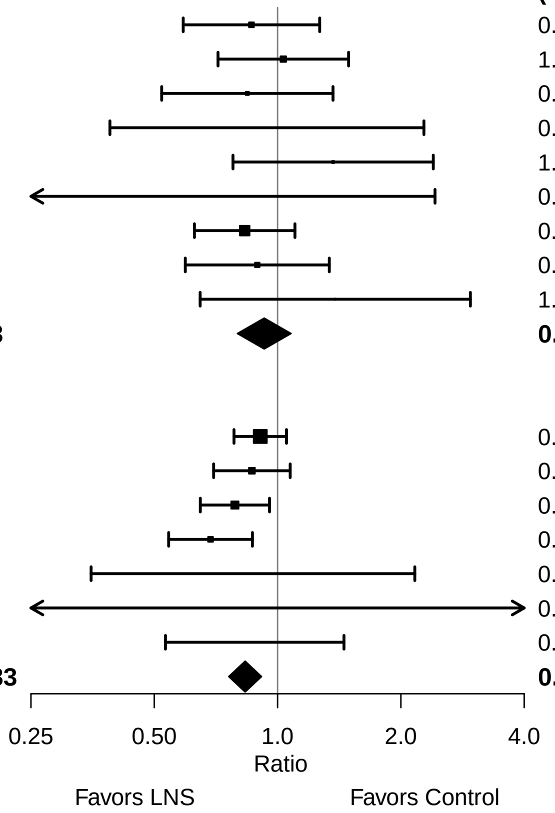

Supplemental figure 6F: Wasting prevalence difference

6F1: Stratified by Geographic region

Geographic region
(p−diff = 0.025)

Country	Trial	N	N	PD (95% CI)	W
**Geographic region − SEAR**					
Bangladesh	JiVitA−4 (34)	2783	1217	−0.01 (−0.04, 0.01)	0.34
Bangladesh	RDNS (35)	1661	815	−0.02 (−0.05, 0.01)	0.20
Bangladesh	WASH−B (36)	1156	3424	−0.02 (−0.04, 0.00)	0.46
**I² = 0.00, Tau² = 0.00**		**5600**	**5456**	**−0.02 (−0.03, −0.01)**	
**Geographic region − AFR**					
Burkina Faso	iLiNS−Zinc (37)	1952	664	−0.04 (−0.07, −0.01)	0.03
Burkina Faso	PROMIS (38)	856	907	−0.01 (−0.04, 0.01)	0.03
Burkina Faso	PROMIS CS (38)	430	436	0.00 (−0.04, 0.05)	0.01
Ghana	GHANA (39)	98	96	−0.01 (−0.09, 0.07)	0.00
Ghana	iLiNS−DYAD−G (40)	347	692	−0.01 (−0.04, 0.02)	0.02
Kenya	WASH−B (42)	1455	5118	0.00 (−0.01, 0.00)	0.44
Madagascar	MAHAY (43)	1700	1682	0.00 (−0.02, 0.01)	0.08
Malawi	iLiNS−DYAD−M (44)	220	444	0.00 (−0.03, 0.03)	0.02
Malawi	iLiNS−DOSE (45)	696	241	0.01 (−0.03, 0.04)	0.02
Mali	PROMIS (46)	499	502	−0.01 (−0.02, 0.01)	0.10
Mali	PROMIS CS (46)	944	959	−0.02 (−0.04, 0.01)	0.03
Zimbabwe	SHINE (HIV−) (47)	1869	1784	0.00 (−0.01, 0.01)	0.20
Zimbabwe	SHINE (HIV+) (48)	336	328	0.01 (−0.02, 0.05)	0.02
**I² = 0.00, Tau² = 0.00**		**11402**	**13853**	**0.00 (−0.01, 0.00)**	

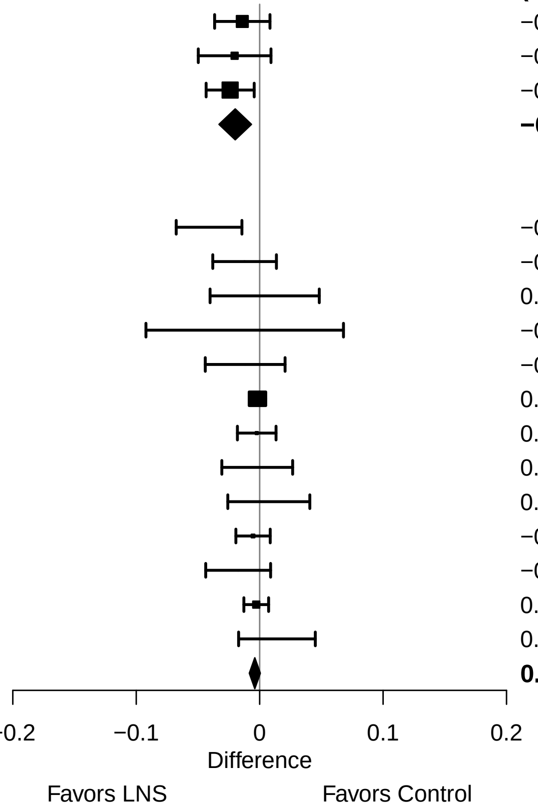

Supplemental figure 6F: Wasting prevalence difference

6F2: Stratified by Stunting burden

Stunting burden
(p−diff = 0.203)

Country	Trial	N	N	PD (95% CI)	W
**Stunting burden − Less than 35%**					
Burkina Faso	PROMIS (38)	856	907	−0.01 (−0.04, 0.01)	0.05
Burkina Faso	PROMIS CS (38)	430	436	0.00 (−0.04, 0.05)	0.02
Ghana	GHANA (39)	98	96	−0.01 (−0.09, 0.07)	0.01
Ghana	iLiNS−DYAD−G (40)	347	692	−0.01 (−0.04, 0.02)	0.03
Haiti	HAITI (41)	148	149	0.00 (−0.03, 0.03)	0.05
Kenya	WASH−B (42)	1455	5118	0.00 (−0.01, 0.00)	0.79
Malawi	iLiNS−DYAD−M (44)	220	444	0.00 (−0.03, 0.03)	0.04
**I² = 0.00, Tau² = 0.00**		**3554**	**7842**	**0.00 (−0.01, 0.00)**	
**Stunting burden − More than 35%**					
Bangladesh	JiVitA−4 (34)	2783	1217	−0.01 (−0.04, 0.01)	0.07
Bangladesh	RDNS (35)	1661	815	−0.02 (−0.05, 0.01)	0.04
Bangladesh	WASH−B (36)	1156	3424	−0.02 (−0.04, 0.00)	0.09
Burkina Faso	iLiNS−Zinc (37)	1952	664	−0.04 (−0.07, −0.01)	0.05
Madagascar	MAHAY (43)	1700	1682	0.00 (−0.02, 0.01)	0.14
Malawi	iLiNS−DOSE (45)	696	241	0.01 (−0.03, 0.04)	0.03
Mali	PROMIS (46)	499	502	−0.01 (−0.02, 0.01)	0.17
Mali	PROMIS CS (46)	944	959	−0.02 (−0.04, 0.01)	0.05
Zimbabwe	SHINE (HIV−) (47)	1869	1784	0.00 (−0.01, 0.01)	0.33
Zimbabwe	SHINE (HIV+) (48)	336	328	0.01 (−0.02, 0.05)	0.03
**I² = 0.37, Tau² = 0.00**		**13596**	**11616**	**−0.01 (−0.02, 0.00)**	

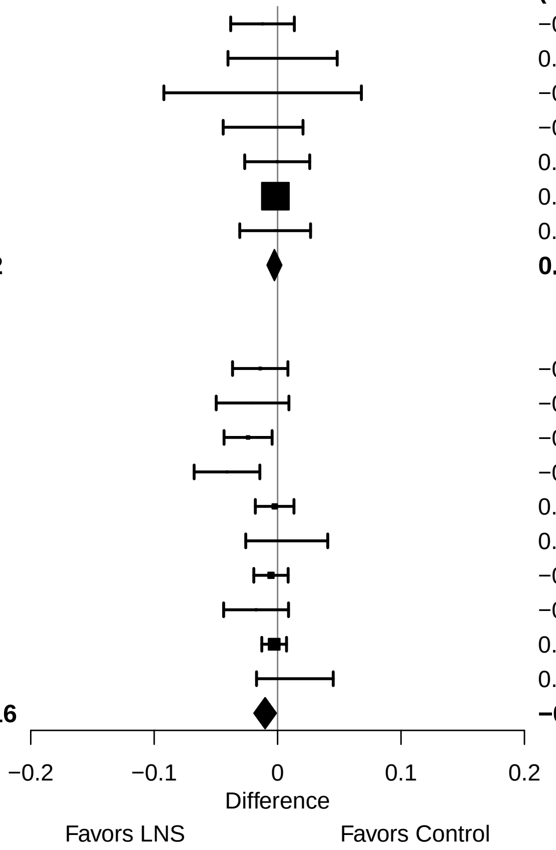

Supplemental figure 6F: Wasting prevalence difference

6F3: Stratified by Malaria prevalence

Malaria prevalence
(p−diff = 0.216)

Malaria prevalence − Less than 10%

Country	Trial	N	N	PD (95% CI)	W
Bangladesh	JiVitA−4 (34)	2783	1217	−0.01 (−0.04, 0.01)	0.04
Bangladesh	RDNS (35)	1661	815	−0.02 (−0.05, 0.01)	0.03
Bangladesh	WASH−B (36)	1156	3424	−0.02 (−0.04, 0.00)	0.06
Haiti	HAITI (41)	148	149	0.00 (−0.03, 0.03)	0.03
Kenya	WASH−B (42)	1455	5118	0.00 (−0.01, 0.00)	0.50
Madagascar	MAHAY (43)	1700	1682	0.00 (−0.02, 0.01)	0.09
Zimbabwe	SHINE (HIV−) (47)	1869	1784	0.00 (−0.01, 0.01)	0.22
Zimbabwe	SHINE (HIV+) (48)	336	328	0.01 (−0.02, 0.05)	0.02
**I^2 = 0.11, Tau^2 = 0.00**		**11108**	**14517**	**0.00 (−0.01, 0.00)**	

Malaria prevalence − At least 10%

Country	Trial	N	N	PD (95% CI)	W
Burkina Faso	iLiNS−Zinc (37)	1952	664	−0.04 (−0.07, −0.01)	0.11
Burkina Faso	PROMIS (38)	856	907	−0.01 (−0.04, 0.01)	0.11
Burkina Faso	PROMIS CS (38)	430	436	0.00 (−0.04, 0.05)	0.04
Ghana	GHANA (39)	98	96	−0.01 (−0.09, 0.07)	0.01
Ghana	iLiNS−DYAD−G (40)	347	692	−0.01 (−0.04, 0.02)	0.07
Malawi	iLiNS−DYAD−M (44)	220	444	0.00 (−0.03, 0.03)	0.09
Malawi	iLiNS−DOSE (45)	696	241	0.01 (−0.03, 0.04)	0.07
Mali	PROMIS (46)	499	502	−0.01 (−0.02, 0.01)	0.39
Mali	PROMIS CS (46)	944	959	−0.02 (−0.04, 0.01)	0.11
**I^2 = 0.00, Tau^2 = 0.00**		**6042**	**4941**	**−0.01 (−0.02, 0.00)**	

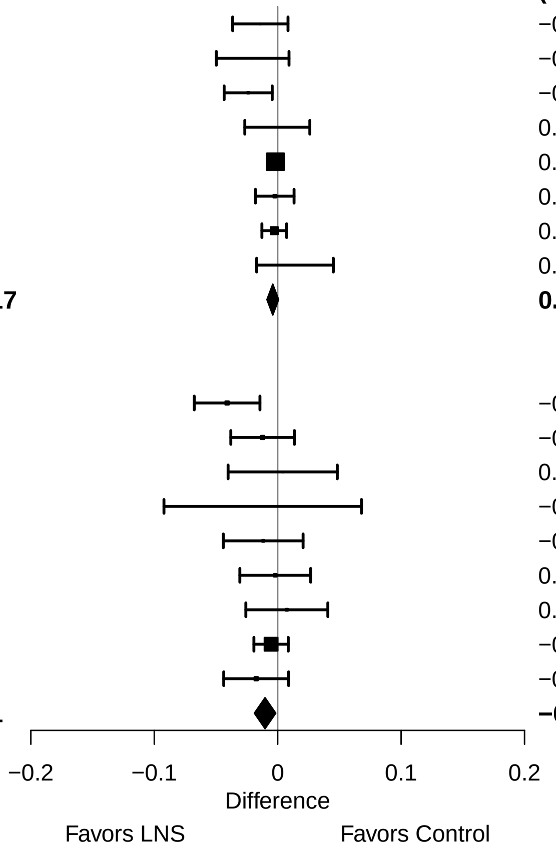

Supplemental figure 6F: Wasting prevalence difference

6F4: Stratified by Source water quality

Source water quality
(p−diff = 0.560)

Source water quality − Improved

Country	Trial	N	N	PD (95% CI)	W
Bangladesh	JiVitA−4 (34)	2783	1217	−0.01 (−0.04, 0.01)	0.22
Bangladesh	RDNS (35)	1661	815	−0.02 (−0.05, 0.01)	0.13
Bangladesh	WASH−B (36)	566	1101	−0.02 (−0.05, 0.01)	0.13
Ghana	GHANA (39)	98	96	−0.01 (−0.09, 0.07)	0.02
Ghana	iLiNS−DYAD−G (40)	347	692	−0.01 (−0.04, 0.02)	0.11
Haiti	HAITI (41)	148	149	0.00 (−0.03, 0.03)	0.16
Malawi	iLiNS−DYAD−M (44)	220	444	0.00 (−0.03, 0.03)	0.14
Malawi	iLiNS−DOSE (45)	696	241	0.01 (−0.03, 0.04)	0.10
**I² = 0.00, Tau² = 0.00**		**6519**	**4755**	**−0.01 (−0.02, 0.00)**	
**Source water quality − Unimproved**					
Burkina Faso	iLiNS−Zinc (37)	1952	664	−0.04 (−0.07, −0.01)	0.05
Burkina Faso	PROMIS (38)	856	907	−0.01 (−0.04, 0.01)	0.05
Burkina Faso	PROMIS CS (38)	430	436	0.00 (−0.04, 0.05)	0.02
Kenya	WASH−B (42)	694	2244	0.00 (−0.01, 0.01)	0.38
Madagascar	MAHAY (43)	1700	1682	0.00 (−0.02, 0.01)	0.13
Mali	PROMIS (46)	499	502	−0.01 (−0.02, 0.01)	0.17
Mali	PROMIS CS (46)	944	959	−0.02 (−0.04, 0.01)	0.05
Zimbabwe	SHINE (HIV−) (47)	887	876	0.00 (−0.02, 0.01)	0.14
Zimbabwe	SHINE (HIV+) (48)	147	146	0.00 (−0.05, 0.05)	0.02
**I² = 0.11, Tau² = 0.00**		**8109**	**8416**	**−0.01 (−0.01, 0.00)**	

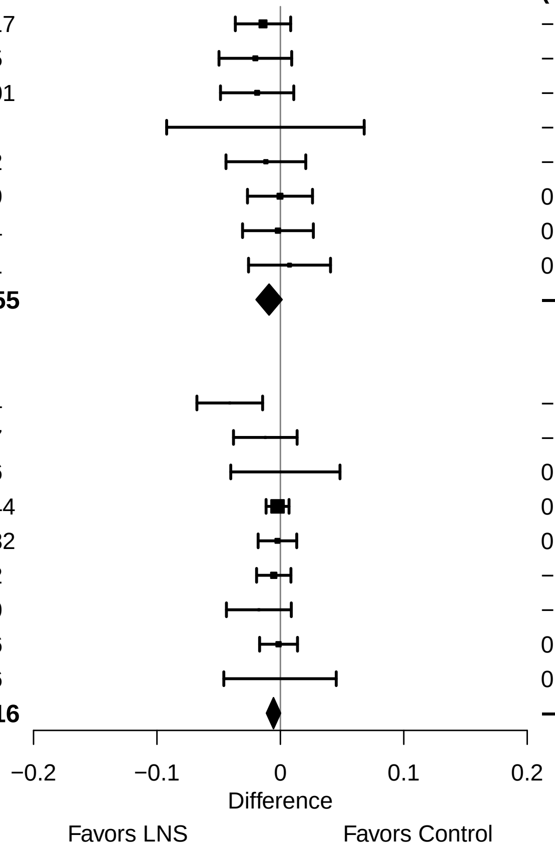

Supplemental figure 6F: Wasting prevalence difference

6F5: Stratified by Sanitation

Sanitation
(p−diff = 0.251)

Country	Trial	N	N	PD (95% CI)	W
**Sanitation − Improved**					
Bangladesh	JiVitA−4 (34)	2783	1217	−0.01 (−0.04, 0.01)	0.13
Bangladesh	RDNS (35)	1661	815	−0.02 (−0.05, 0.01)	0.07
Bangladesh	WASH−B (36)	566	1101	−0.02 (−0.05, 0.01)	0.07
Burkina Faso	PROMIS (38)	856	907	−0.01 (−0.04, 0.01)	0.10
Burkina Faso	PROMIS CS (38)	430	436	0.00 (−0.04, 0.05)	0.03
Ghana	GHANA (39)	98	96	−0.01 (−0.09, 0.07)	0.01
Ghana	iLiNS−DYAD−G (40)	347	692	−0.01 (−0.04, 0.02)	0.06
Haiti	HAITI (41)	148	149	0.00 (−0.03, 0.03)	0.09
Mali	PROMIS (46)	499	502	−0.01 (−0.02, 0.01)	0.33
Mali	PROMIS CS (46)	944	959	−0.02 (−0.04, 0.01)	0.09
**I² = 0.00, Tau² = 0.00**		**8332**	**6874**	**−0.01 (−0.02, 0.00)**	
**Sanitation − Unimproved**					
Burkina Faso	iLiNS−Zinc (37)	1952	664	−0.04 (−0.07, −0.01)	0.06
Kenya	WASH−B (42)	694	2244	0.00 (−0.01, 0.01)	0.48
Madagascar	MAHAY (43)	1700	1682	0.00 (−0.02, 0.01)	0.17
Malawi	iLiNS−DYAD−M (44)	220	444	0.00 (−0.03, 0.03)	0.05
Malawi	iLiNS−DOSE (45)	696	241	0.01 (−0.03, 0.04)	0.04
Zimbabwe	SHINE (HIV−) (47)	887	876	0.00 (−0.02, 0.01)	0.18
Zimbabwe	SHINE (HIV+) (48)	147	146	0.00 (−0.05, 0.05)	0.02
**I² = 0.27, Tau² = 0.00**		**6296**	**6297**	**−0.01 (−0.02, 0.01)**	

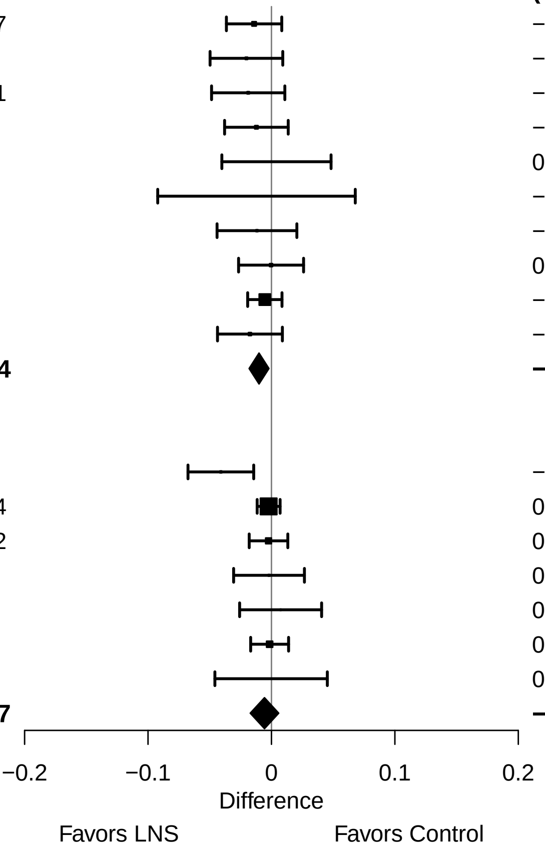

Supplemental figure 6F: Wasting prevalence difference

6F6: Stratified by Supplement duration

Supplement duration
(p−diff = 0.696)

**Supplement duration − 12m or less**				**PD**	
**Country**	**Trial**	**N**	**N**	**(95% CI)**	**W**
Bangladesh	JiVitA−4 (34)	2783	1217	−0.01 (−0.04, 0.01)	0.08
Burkina Faso	iLiNS−Zinc (37)	1952	664	−0.04 (−0.07, −0.01)	0.06
Burkina Faso	PROMIS (38)	856	907	−0.01 (−0.04, 0.01)	0.06
Burkina Faso	PROMIS CS (38)	430	436	0.00 (−0.04, 0.05)	0.02
Ghana	GHANA (39)	98	96	−0.01 (−0.09, 0.07)	0.01
Ghana	iLiNS−DYAD−G (40)	347	692	−0.01 (−0.04, 0.02)	0.04
Haiti	HAITI (41)	148	149	0.00 (−0.03, 0.03)	0.06
Madagascar	MAHAY (43)	1700	1682	0.00 (−0.02, 0.01)	0.16
Malawi	iLiNS−DYAD−M (44)	220	444	0.00 (−0.03, 0.03)	0.05
Malawi	iLiNS−DOSE (45)	696	241	0.01 (−0.03, 0.04)	0.04
Zimbabwe	SHINE (HIV−) (47)	1869	1784	0.00 (−0.01, 0.01)	0.40
Zimbabwe	SHINE (HIV+) (48)	336	328	0.01 (−0.02, 0.05)	0.04
**I^2 = 0.00, Tau^2 = 0.00**		**11435**	**8640**	**−0.01 (−0.01, 0.00)**	
**Supplement duration − > 12m**					
Bangladesh	RDNS (35)	1661	815	−0.02 (−0.05, 0.01)	0.04
Bangladesh	WASH−B (36)	1156	3424	−0.02 (−0.04, 0.00)	0.08
Kenya	WASH−B (42)	1455	5118	0.00 (−0.01, 0.00)	0.68
Mali	PROMIS (46)	499	502	−0.01 (−0.02, 0.01)	0.16
Mali	PROMIS CS (46)	944	959	−0.02 (−0.04, 0.01)	0.04
**I^2 = 0.37, Tau^2 = 0.00**		**5715**	**10818**	**−0.01 (−0.02, 0.00)**	

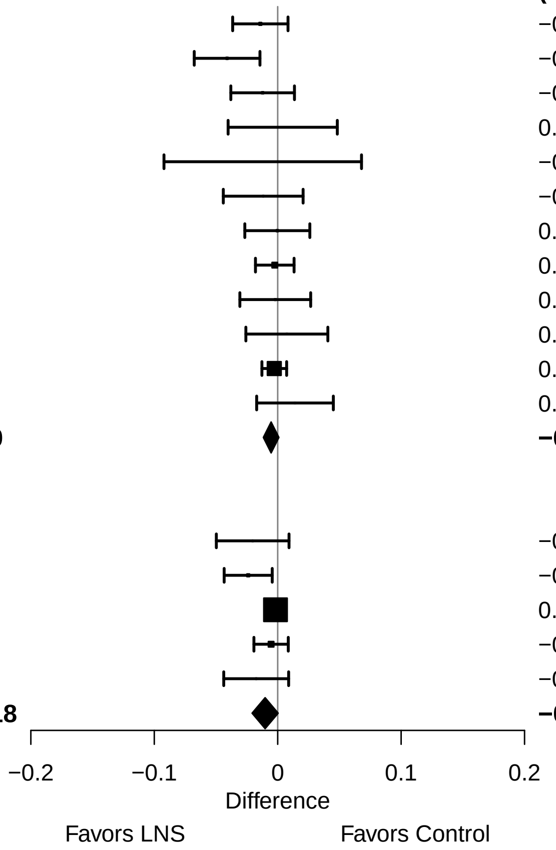

Supplemental figure 6F: Wasting prevalence difference

6F7: Stratified by Frequency of contact

Frequency of contact
(p−diff = 0.017)

Country	Trial	N	N	PD (95% CI)	W
**Frequency of contact − Monthly**					
Bangladesh	RDNS (35)	1661	815	−0.02 (−0.05, 0.01)	0.02
Burkina Faso	PROMIS (38)	856	907	−0.01 (−0.04, 0.01)	0.03
Burkina Faso	PROMIS CS (38)	430	436	0.00 (−0.04, 0.05)	0.01
Haiti	HAITI (41)	148	149	0.00 (−0.03, 0.03)	0.03
Kenya	WASH−B (42)	1455	5118	0.00 (−0.01, 0.00)	0.46
Madagascar	MAHAY (43)	1700	1682	0.00 (−0.02, 0.01)	0.08
Mali	PROMIS (46)	499	502	−0.01 (−0.02, 0.01)	0.11
Mali	PROMIS CS (46)	944	959	−0.02 (−0.04, 0.01)	0.03
Zimbabwe	SHINE (HIV−) (47)	1869	1784	0.00 (−0.01, 0.01)	0.21
Zimbabwe	SHINE (HIV+) (48)	336	328	0.01 (−0.02, 0.05)	0.02
**I^2 = 0.00, Tau^2 = 0.00**		**9898**	**12680**	**0.00 (−0.01, 0.00)**	
**Frequency of contact − Weekly**					
Bangladesh	JiVitA−4 (34)	2783	1217	−0.01 (−0.04, 0.01)	0.21
Bangladesh	WASH−B (36)	1156	3424	−0.02 (−0.04, 0.00)	0.28
Burkina Faso	iLiNS−Zinc (37)	1952	664	−0.04 (−0.07, −0.01)	0.15
Ghana	GHANA (39)	98	96	−0.01 (−0.09, 0.07)	0.02
Ghana	iLiNS−DYAD−G (40)	347	692	−0.01 (−0.04, 0.02)	0.10
Malawi	iLiNS−DYAD−M (44)	220	444	0.00 (−0.03, 0.03)	0.13
Malawi	iLiNS−DOSE (45)	696	241	0.01 (−0.03, 0.04)	0.10
**I^2 = 0.13, Tau^2 = 0.00**		**7252**	**6778**	**−0.02 (−0.03, −0.01)**	

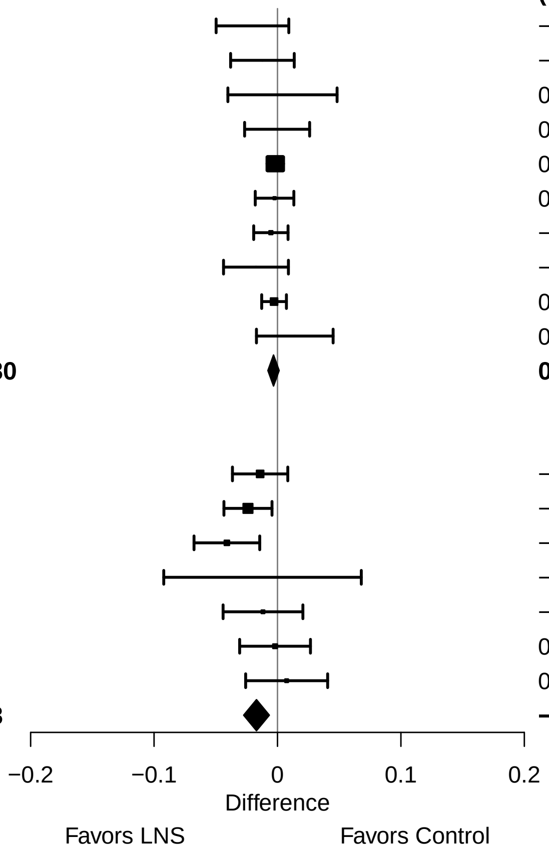

Supplemental figure 6F: Wasting prevalence difference

6F8: Stratified by Average SQ-LNS compliance

Average SQ−LNS compliance
(p−diff = 0.204)

**Average SQ−LNS compliance − Low**				**PD**	
**Country**	**Trial**	**N**	**N**	**(95% CI)**	**W**
Burkina Faso	PROMIS (38)	856	907	−0.01 (−0.04, 0.01)	0.07
Burkina Faso	PROMIS CS (38)	430	436	0.00 (−0.04, 0.05)	0.02
Ghana	iLiNS−DYAD−G (40)	347	692	−0.01 (−0.04, 0.02)	0.04
Malawi	iLiNS−DYAD−M (44)	220	444	0.00 (−0.03, 0.03)	0.05
Malawi	iLiNS−DOSE (45)	696	241	0.01 (−0.03, 0.04)	0.04
Mali	PROMIS (46)	499	502	−0.01 (−0.02, 0.01)	0.23
Mali	PROMIS CS (46)	944	959	−0.02 (−0.04, 0.01)	0.06
Zimbabwe	SHINE (HIV−) (47)	1869	1784	0.00 (−0.01, 0.01)	0.44
Zimbabwe	SHINE (HIV+) (48)	336	328	0.01 (−0.02, 0.05)	0.05
**I² = 0.00, Tau² = 0.00**		**6197**	**6293**	**0.00 (−0.01, 0.00)**	
**Average SQ−LNS compliance − High**					
Bangladesh	JiVitA−4 (34)	2783	1217	−0.01 (−0.04, 0.01)	0.06
Bangladesh	RDNS (35)	1661	815	−0.02 (−0.05, 0.01)	0.04
Bangladesh	WASH−B (36)	1156	3424	−0.02 (−0.04, 0.00)	0.09
Burkina Faso	iLiNS−Zinc (37)	1952	664	−0.04 (−0.07, −0.01)	0.05
Ghana	GHANA (39)	98	96	−0.01 (−0.09, 0.07)	0.01
Haiti	HAITI (41)	148	149	0.00 (−0.03, 0.03)	0.05
Kenya	WASH−B (42)	1455	5118	0.00 (−0.01, 0.00)	0.72
**I² = 0.54, Tau² = 0.00**		**9253**	**11483**	**−0.01 (−0.03, 0.00)**	

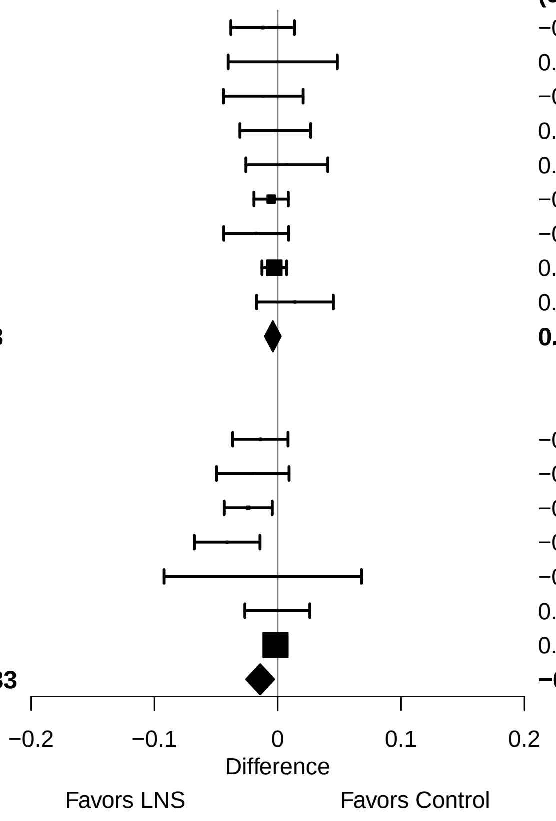

Supplemental figure 6G: Mean difference in MUACZ

6G1: Stratified by Geographic region (insufficient comparisons)

Supplemental figure 6G: Mean difference in MUACZ

6G2: Stratified by Stunting burden

Stunting burden
(p−diff = 0.433)

Country	Trial	N	N	MD (95% CI)	W
**Stunting burden − Less than 35%**					
Burkina Faso	PROMIS (38)	854	912	0.09 (−0.03, 0.21)	0.20
Burkina Faso	PROMIS CS (38)	424	436	−0.06 (−0.22, 0.10)	0.15
Ghana	GHANA (39)				
Ghana	iLiNS−DYAD−G (40)	347	693	0.09 (−0.03, 0.20)	0.21
Haiti	HAITI (41)				
Kenya	WASH−B (42)	1461	5185	0.12 (0.05, 0.18)	0.29
Malawi	iLiNS−DYAD−M (44)	220	444	−0.04 (−0.20, 0.12)	0.15
**I² = 0.35, Tau² = 0.00**		**3306**	**7670**	**0.06 (0.00, 0.13)**	
**Stunting burden − More than 35%**					
Bangladesh	JiVitA−4 (34)	2897	1271	0.08 (0.03, 0.13)	0.15
Bangladesh	RDNS (35)	1663	815	0.04 (−0.04, 0.12)	0.13
Bangladesh	WASH−B (36)				
Burkina Faso	iLiNS−Zinc (37)	1959	666	0.30 (0.20, 0.39)	0.12
Madagascar	MAHAY (43)	1680	1658	−0.03 (−0.16, 0.09)	0.09
Malawi	iLiNS−DOSE (45)	696	240	0.06 (−0.05, 0.18)	0.10
Mali	PROMIS (46)	507	505	0.19 (0.05, 0.32)	0.09
Mali	PROMIS CS (46)	954	970	0.13 (0.01, 0.25)	0.10
Zimbabwe	SHINE (HIV−) (47)	1872	1780	0.06 (0.00, 0.12)	0.14
Zimbabwe	SHINE (HIV+) (48)	337	328	0.04 (−0.10, 0.17)	0.09
**I² = 0.72, Tau² = 0.01**		**12565**	**8233**	**0.10 (0.03, 0.16)**	

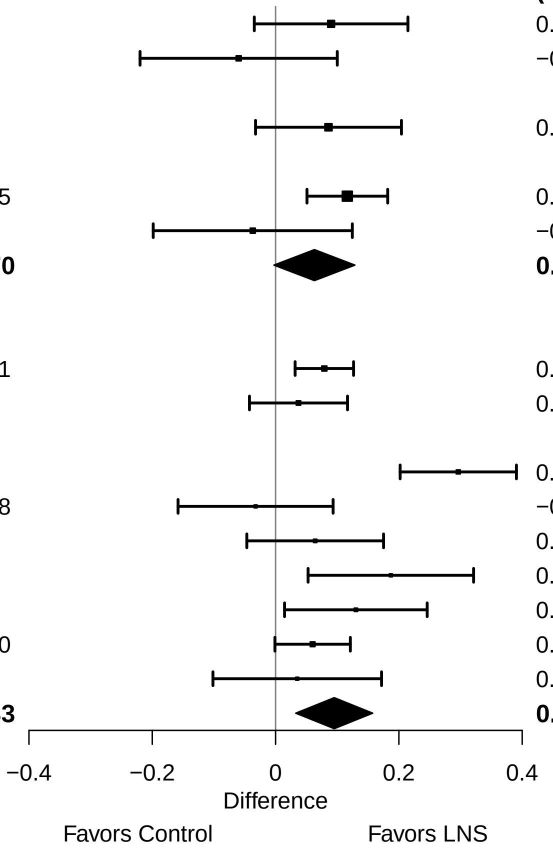

Supplemental figure 6G: Mean difference in MUACZ

6G3: Stratified by Malaria prevalence

Malaria prevalence
(p−diff = 0.248)

Country	Trial	N	N	MD (95% CI)	W
**Malaria prevalence − Less than 10%**					
Bangladesh	JiVitA−4 (34)	2897	1271	0.08 (0.03, 0.13)	0.21
Bangladesh	RDNS (35)	1663	815	0.04 (−0.04, 0.12)	0.17
Bangladesh	WASH−B (36)				
Haiti	HAITI (41)				
Kenya	WASH−B (42)	1461	5185	0.12 (0.05, 0.18)	0.19
Madagascar	MAHAY (43)	1680	1658	−0.03 (−0.16, 0.09)	0.13
Zimbabwe	SHINE (HIV−) (47)	1872	1780	0.06 (0.00, 0.12)	0.19
Zimbabwe	SHINE (HIV+) (48)	337	328	0.04 (−0.10, 0.17)	0.12
**I² = 0.11, Tau² = 0.00**		**9910**	**11037**	**0.07 (0.03, 0.10)**	
**Malaria prevalence − At least 10%**					
Burkina Faso	iLiNS−Zinc (37)	1959	666	0.30 (0.20, 0.39)	0.16
Burkina Faso	PROMIS (38)	854	912	0.09 (−0.03, 0.21)	0.13
Burkina Faso	PROMIS CS (38)	424	436	−0.06 (−0.22, 0.10)	0.10
Ghana	GHANA (39)				
Ghana	iLiNS−DYAD−G (40)	347	693	0.09 (−0.03, 0.20)	0.13
Malawi	iLiNS−DYAD−M (44)	220	444	−0.04 (−0.20, 0.12)	0.10
Malawi	iLiNS−DOSE (45)	696	240	0.06 (−0.05, 0.18)	0.14
Mali	PROMIS (46)	507	505	0.19 (0.05, 0.32)	0.12
Mali	PROMIS CS (46)	954	970	0.13 (0.01, 0.25)	0.13
**I² = 0.71, Tau² = 0.01**		**5961**	**4866**	**0.10 (0.02, 0.18)**	

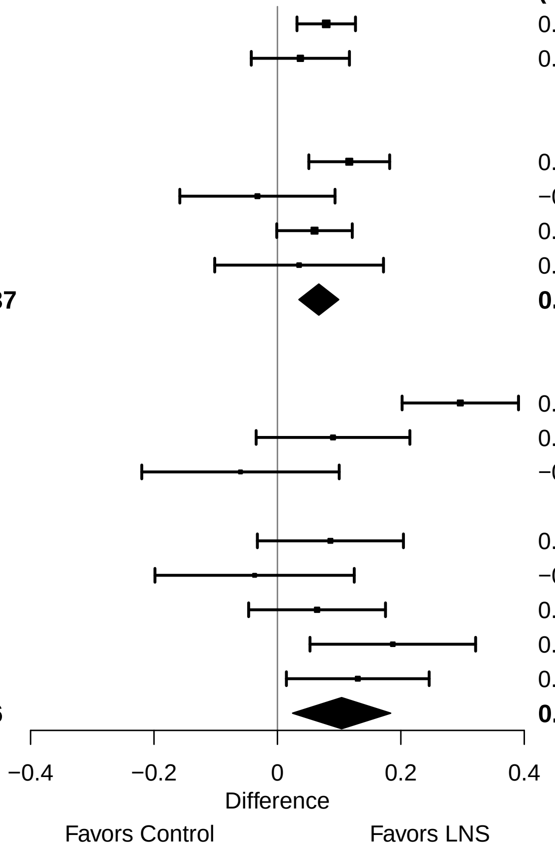

Supplemental figure 6G: Mean difference in MUACZ

6G4: Stratified by Source water quality

Source water quality
(p−diff = 0.438)

Country	Trial	N	N	MD (95% CI)	W
**Source water quality − Improved**					
Bangladesh	JiVitA−4 (34)	2897	1271	0.08 (0.03, 0.13)	0.27
Bangladesh	RDNS (35)	1663	815	0.04 (−0.04, 0.12)	0.23
Bangladesh	WASH−B (36)				
Ghana	GHANA (39)				
Ghana	iLiNS−DYAD−G (40)	347	693	0.09 (−0.03, 0.20)	0.18
Haiti	HAITI (41)				
Malawi	iLiNS−DYAD−M (44)	220	444	−0.04 (−0.20, 0.12)	0.13
Malawi	iLiNS−DOSE (45)	696	240	0.06 (−0.05, 0.18)	0.19
**I² = 0.00, Tau² = 0.00**		**5823**	**3463**	**0.06 (0.03, 0.10)**	
**Source water quality − Unimproved**					
Burkina Faso	iLiNS−Zinc (37)	1959	666	0.30 (0.20, 0.39)	0.14
Burkina Faso	PROMIS (38)	854	912	0.09 (−0.03, 0.21)	0.11
Burkina Faso	PROMIS CS (38)	424	436	−0.06 (−0.22, 0.10)	0.09
Kenya	WASH−B (42)	697	2270	0.10 (0.01, 0.19)	0.14
Madagascar	MAHAY (43)	1680	1658	−0.03 (−0.16, 0.09)	0.11
Mali	PROMIS (46)	507	505	0.19 (0.05, 0.32)	0.10
Mali	PROMIS CS (46)	954	970	0.13 (0.01, 0.25)	0.12
Zimbabwe	SHINE (HIV−) (47)	889	871	0.02 (−0.07, 0.11)	0.14
Zimbabwe	SHINE (HIV+) (48)	147	146	0.00 (−0.23, 0.24)	0.05
**I² = 0.74, Tau² = 0.01**		**8111**	**8434**	**0.09 (0.02, 0.17)**	

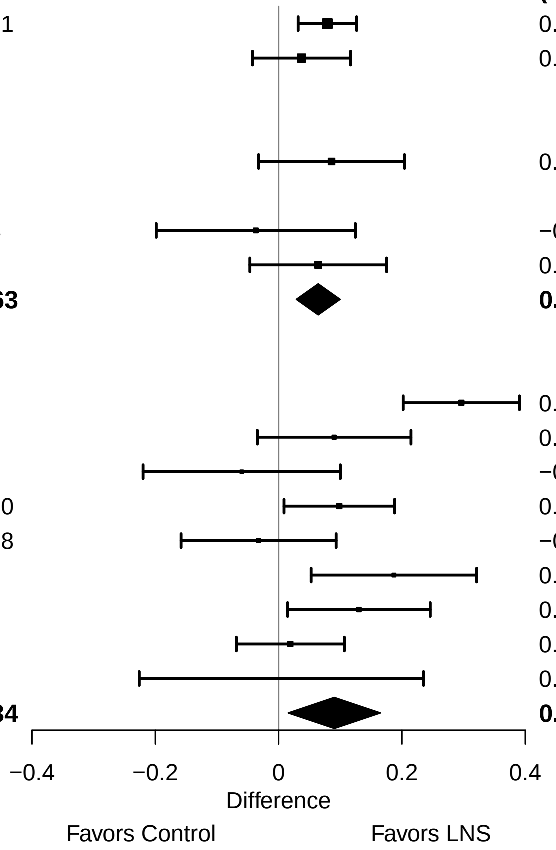

Supplemental figure 6G: Mean difference in MUACZ

6G5: Stratified by Sanitation

Sanitation
(p−diff = 0.887)

Country	Trial	N	N	MD (95% CI)	W
**Sanitation − Improved**					
Bangladesh	JiVitA−4 (34)	2897	1271	0.08 (0.03, 0.13)	0.21
Bangladesh	RDNS (35)	1663	815	0.04 (−0.04, 0.12)	0.17
Bangladesh	WASH−B (36)				
Burkina Faso	PROMIS (38)	854	912	0.09 (−0.03, 0.21)	0.13
Burkina Faso	PROMIS CS (38)	424	436	−0.06 (−0.22, 0.10)	0.10
Ghana	GHANA (39)				
Ghana	iLiNS−DYAD−G (40)	347	693	0.09 (−0.03, 0.20)	0.13
Haiti	HAITI (41)				
Mali	PROMIS (46)	507	505	0.19 (0.05, 0.32)	0.12
Mali	PROMIS CS (46)	954	970	0.13 (0.01, 0.25)	0.14
**I^2 = 0.17, Tau^2 = 0.00**		**7646**	**5602**	**0.08 (0.04, 0.12)**	
**Sanitation − Unimproved**					
Burkina Faso	iLiNS−Zinc (37)	1959	666	0.30 (0.20, 0.39)	0.17
Kenya	WASH−B (42)	697	2270	0.10 (0.01, 0.19)	0.18
Madagascar	MAHAY (43)	1680	1658	−0.03 (−0.16, 0.09)	0.14
Malawi	iLiNS−DYAD−M (44)	220	444	−0.04 (−0.20, 0.12)	0.11
Malawi	iLiNS−DOSE (45)	696	240	0.06 (−0.05, 0.18)	0.15
Zimbabwe	SHINE (HIV−) (47)	889	871	0.02 (−0.07, 0.11)	0.18
Zimbabwe	SHINE (HIV+) (48)	147	146	0.00 (−0.23, 0.24)	0.07
**I^2 = 0.78, Tau^2 = 0.01**		**6288**	**6295**	**0.07 (−0.02, 0.16)**	

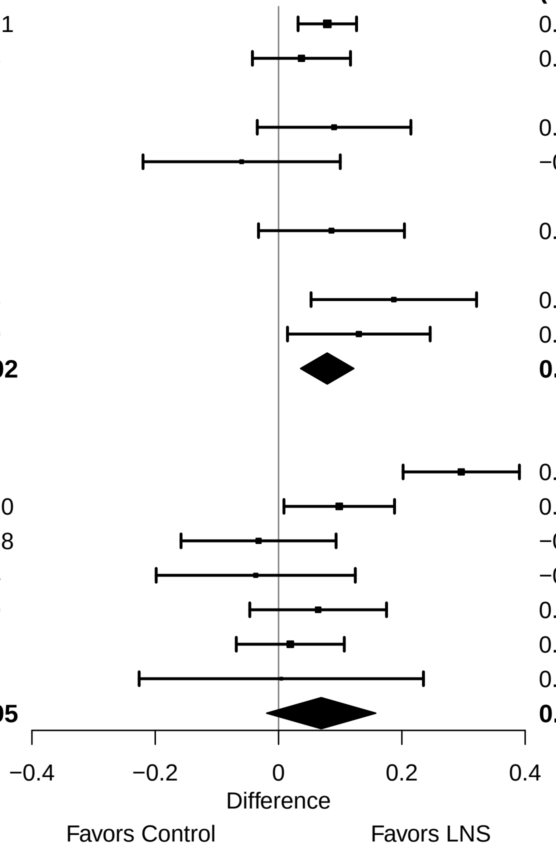

Supplemental figure 6G: Mean difference in MUACZ

6G6: Stratified by Supplement duration

Supplement duration
(p−diff = 0.427)

Country	Trial	N	N	MD (95% CI)	W
**Supplement duration − 12m or less**					
Bangladesh	JiVitA−4 (34)	2897	1271	0.08 (0.03, 0.13)	0.15
Burkina Faso	iLiNS−Zinc (37)	1959	666	0.30 (0.20, 0.39)	0.11
Burkina Faso	PROMIS (38)	854	912	0.09 (−0.03, 0.21)	0.09
Burkina Faso	PROMIS CS (38)	424	436	−0.06 (−0.22, 0.10)	0.07
Ghana	GHANA (39)				
Ghana	iLiNS−DYAD−G (40)	347	693	0.09 (−0.03, 0.20)	0.10
Haiti	HAITI (41)				
Madagascar	MAHAY (43)	1680	1658	−0.03 (−0.16, 0.09)	0.09
Malawi	iLiNS−DYAD−M (44)	220	444	−0.04 (−0.20, 0.12)	0.07
Malawi	iLiNS−DOSE (45)	696	240	0.06 (−0.05, 0.18)	0.10
Zimbabwe	SHINE (HIV−) (47)	1872	1780	0.06 (0.00, 0.12)	0.14
Zimbabwe	SHINE (HIV+) (48)	337	328	0.04 (−0.10, 0.17)	0.08
**I^2 = 0.69, Tau^2 = 0.01**		**11286**	**8428**	**0.07 (0.01, 0.13)**	
**Supplement duration − > 12m**					
Bangladesh	RDNS (35)	1663	815	0.04 (−0.04, 0.12)	0.28
Bangladesh	WASH−B (36)				
Kenya	WASH−B (42)	1461	5185	0.12 (0.05, 0.18)	0.31
Mali	PROMIS (46)	507	505	0.19 (0.05, 0.32)	0.19
Mali	PROMIS CS (46)	954	970	0.13 (0.01, 0.25)	0.22
**I^2 = 0.33, Tau^2 = 0.00**		**4585**	**7475**	**0.11 (0.05, 0.16)**	

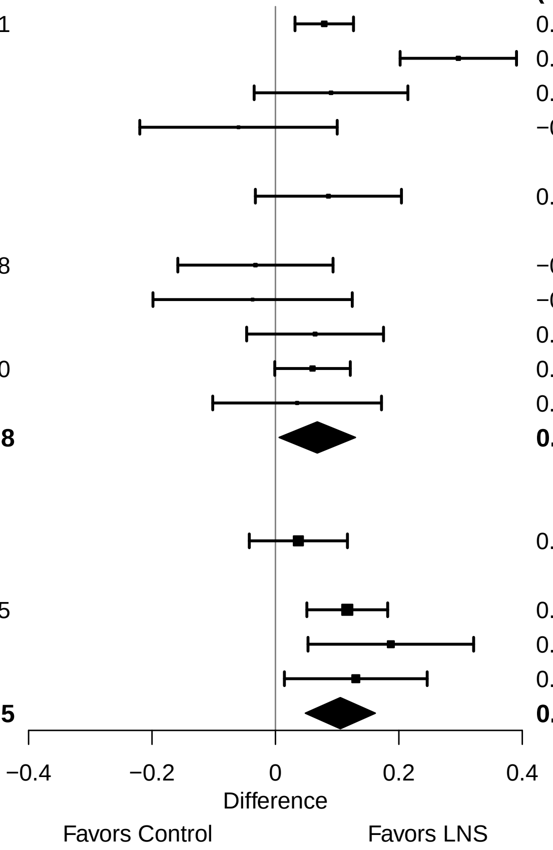

Supplemental figure 6G: Mean difference in MUACZ

6G7: Stratified by Frequency of contact

Frequency of contact
(p−diff = 0.388)

Frequency of contact − Monthly

**Country**	**Trial**	**N**	**N**	**MD (95% CI)**	**W**
Bangladesh	RDNS (35)	1663	815	0.04 (−0.04, 0.12)	0.14
Burkina Faso	PROMIS (38)	854	912	0.09 (−0.03, 0.21)	0.10
Burkina Faso	PROMIS CS (38)	424	436	−0.06 (−0.22, 0.10)	0.08
Haiti	HAITI (41)				
Kenya	WASH−B (42)	1461	5185	0.12 (0.05, 0.18)	0.15
Madagascar	MAHAY (43)	1680	1658	−0.03 (−0.16, 0.09)	0.10
Mali	PROMIS (46)	507	505	0.19 (0.05, 0.32)	0.09
Mali	PROMIS CS (46)	954	970	0.13 (0.01, 0.25)	0.11
Zimbabwe	SHINE (HIV−) (47)	1872	1780	0.06 (0.00, 0.12)	0.15
Zimbabwe	SHINE (HIV+) (48)	337	328	0.04 (−0.10, 0.17)	0.09
**I² = 0.34, Tau² = 0.00**		**9752**	**12589**	**0.07 (0.03, 0.11)**	
**Frequency of contact − Weekly**					
Bangladesh	JiVitA−4 (34)	2897	1271	0.08 (0.03, 0.13)	0.28
Bangladesh	WASH−B (36)				
Burkina Faso	iLiNS−Zinc (37)	1959	666	0.30 (0.20, 0.39)	0.21
Ghana	GHANA (39)				
Ghana	iLiNS−DYAD−G (40)	347	693	0.09 (−0.03, 0.20)	0.18
Malawi	iLiNS−DYAD−M (44)	220	444	−0.04 (−0.20, 0.12)	0.13
Malawi	iLiNS−DOSE (45)	696	240	0.06 (−0.05, 0.18)	0.19
**I² = 0.81, Tau² = 0.01**		**6119**	**3314**	**0.10 (0.00, 0.21)**	

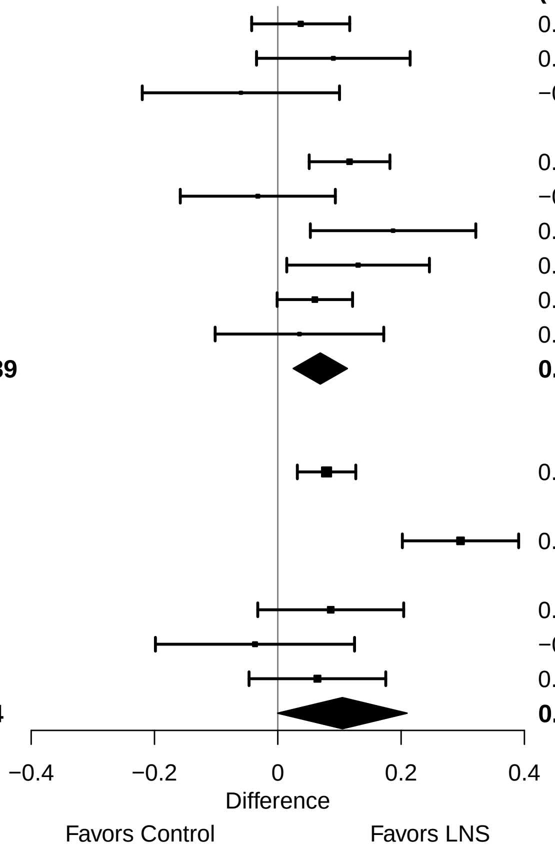

Supplemental figure 6G: Mean difference in MUACZ

6G8: Stratified by Average SQ-LNS compliance

Average SQ−LNS compliance
(p−diff = 0.228)

Average SQ−LNS compliance − Low

Country	Trial	N	N	MD (95% CI)	W
Burkina Faso	PROMIS (38)	854	912	0.09 (−0.03, 0.21)	0.11
Burkina Faso	PROMIS CS (38)	424	436	−0.06 (−0.22, 0.10)	0.08
Ghana	iLiNS−DYAD−G (40)	347	693	0.09 (−0.03, 0.20)	0.11
Malawi	iLiNS−DYAD−M (44)	220	444	−0.04 (−0.20, 0.12)	0.08
Malawi	iLiNS−DOSE (45)	696	240	0.06 (−0.05, 0.18)	0.12
Mali	PROMIS (46)	507	505	0.19 (0.05, 0.32)	0.10
Mali	PROMIS CS (46)	954	970	0.13 (0.01, 0.25)	0.12
Zimbabwe	SHINE (HIV−) (47)	1872	1780	0.06 (0.00, 0.12)	0.17
Zimbabwe	SHINE (HIV+) (48)	337	328	0.04 (−0.10, 0.17)	0.10
**I² = 0.08, Tau² = 0.00**		**6211**	**6308**	**0.07 (0.03, 0.11)**	

Average SQ−LNS compliance − High

Country	Trial	N	N	MD (95% CI)	W
Bangladesh	JiVitA−4 (34)	2897	1271	0.08 (0.03, 0.13)	0.29
Bangladesh	RDNS (35)	1663	815	0.04 (−0.04, 0.12)	0.24
Bangladesh	WASH−B (36)				
Burkina Faso	iLiNS−Zinc (37)	1959	666	0.30 (0.20, 0.39)	0.21
Ghana	GHANA (39)				
Haiti	HAITI (41)				
Kenya	WASH−B (42)	1461	5185	0.12 (0.05, 0.18)	0.26
**I² = 0.85, Tau² = 0.01**		**7980**	**7937**	**0.13 (0.02, 0.24)**	

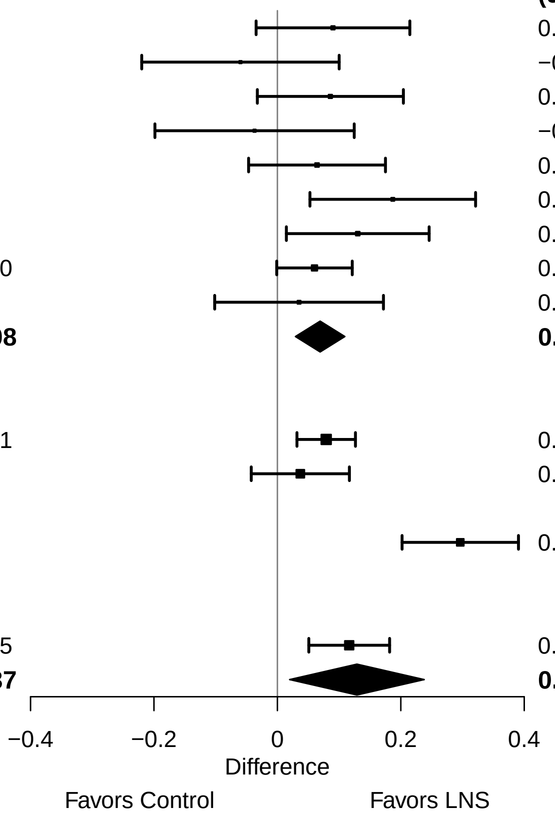

Supplemental figure 6H: Low MUAC prevalence ratio

6H1: Stratified by Geographic region (insufficient comparisons)

Supplemental figure 6H: Low MUAC prevalence ratio

6H2: Stratified by Stunting burden

Stunting burden
(p−diff = 0.925)

Country	Trial	N	N	PR (95% CI)	W
**Stunting burden − Less than 35%**					
Burkina Faso	PROMIS (38)	854	912	0.94 (0.63, 1.40)	0.21
Burkina Faso	PROMIS CS (38)	424	436	0.92 (0.66, 1.28)	0.29
Ghana	GHANA (39)				
Ghana	iLiNS−DYAD−G (40)	347	693	0.77 (0.37, 1.57)	0.07
Haiti	HAITI (41)				
Kenya	WASH−B (42)	1461	5185	0.71 (0.54, 0.94)	0.39
Malawi	iLiNS−DYAD−M (44)	220	444	0.94 (0.39, 2.28)	0.05
**I² = 0.00, Tau² = 0.00**		**3306**	**7670**	**0.82 (0.69, 0.98)**	
**Stunting burden − More than 35%**					
Bangladesh	JiVitA−4 (34)	2897	1271	0.85 (0.70, 1.03)	0.26
Bangladesh	RDNS (35)	1663	815	1.02 (0.69, 1.49)	0.09
Bangladesh	WASH−B (36)				
Burkina Faso	iLiNS−Zinc (37)	1959	666	0.64 (0.51, 0.79)	0.23
Madagascar	MAHAY (43)	1680	1658	0.97 (0.76, 1.26)	0.18
Malawi	iLiNS−DOSE (45)	696	240	1.35 (0.54, 3.36)	0.02
Mali	PROMIS (46)	507	505	0.58 (0.31, 1.07)	0.04
Mali	PROMIS CS (46)	954	970	0.90 (0.68, 1.19)	0.15
Zimbabwe	SHINE (HIV−) (47)	1872	1780	0.51 (0.27, 0.97)	0.03
Zimbabwe	SHINE (HIV+) (48)	337	328	0.97 (0.29, 3.28)	0.01
**I² = 0.41, Tau² = 0.02**		**12565**	**8233**	**0.82 (0.70, 0.96)**	

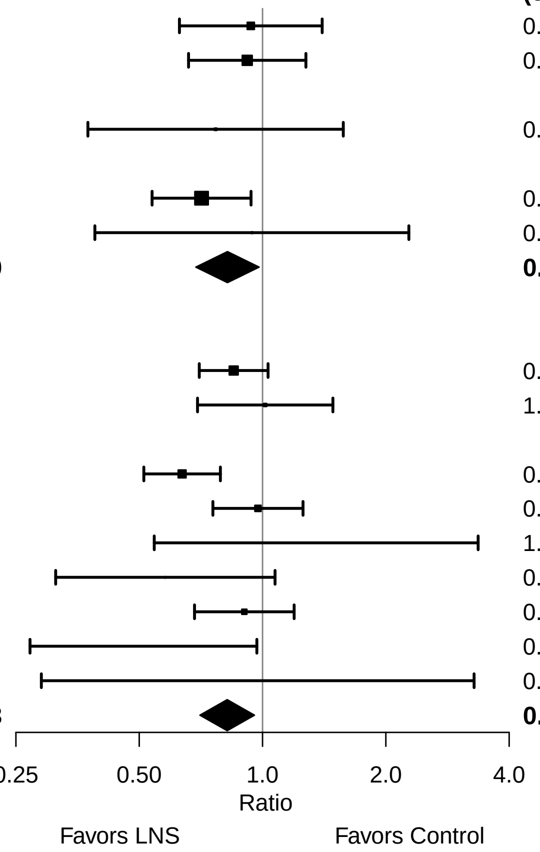

Supplemental figure 6H: Low MUAC prevalence ratio

6H3: Stratified by Malaria prevalence

Malaria prevalence
(p−diff = 0.684)

Country	Trial	N	N	PR (95% CI)	W
**Malaria prevalence − Less than 10%**					
Bangladesh	JiVitA−4 (34)	2897	1271	0.85 (0.70, 1.03)	0.36
Bangladesh	RDNS (35)	1663	815	1.02 (0.69, 1.49)	0.12
Bangladesh	WASH−B (36)				
Haiti	HAITI (41)				
Kenya	WASH−B (42)	1461	5185	0.71 (0.54, 0.94)	0.21
Madagascar	MAHAY (43)	1680	1658	0.97 (0.76, 1.26)	0.24
Zimbabwe	SHINE (HIV−) (47)	1872	1780	0.51 (0.27, 0.97)	0.05
Zimbabwe	SHINE (HIV+) (48)	337	328	0.97 (0.29, 3.28)	0.01
**I² = 0.17, Tau² = 0.01**		**9910**	**11037**	**0.84 (0.73, 0.98)**	
**Malaria prevalence − At least 10%**					
Burkina Faso	iLiNS−Zinc (37)	1959	666	0.64 (0.51, 0.79)	0.34
Burkina Faso	PROMIS (38)	854	912	0.94 (0.63, 1.40)	0.12
Burkina Faso	PROMIS CS (38)	424	436	0.92 (0.66, 1.28)	0.17
Ghana	GHANA (39)				
Ghana	iLiNS−DYAD−G (40)	347	693	0.77 (0.37, 1.57)	0.04
Malawi	iLiNS−DYAD−M (44)	220	444	0.94 (0.39, 2.28)	0.03
Malawi	iLiNS−DOSE (45)	696	240	1.35 (0.54, 3.36)	0.03
Mali	PROMIS (46)	507	505	0.58 (0.31, 1.07)	0.05
Mali	PROMIS CS (46)	954	970	0.90 (0.68, 1.19)	0.22
**I² = 0.19, Tau² = 0.01**		**5961**	**4866**	**0.79 (0.68, 0.92)**	

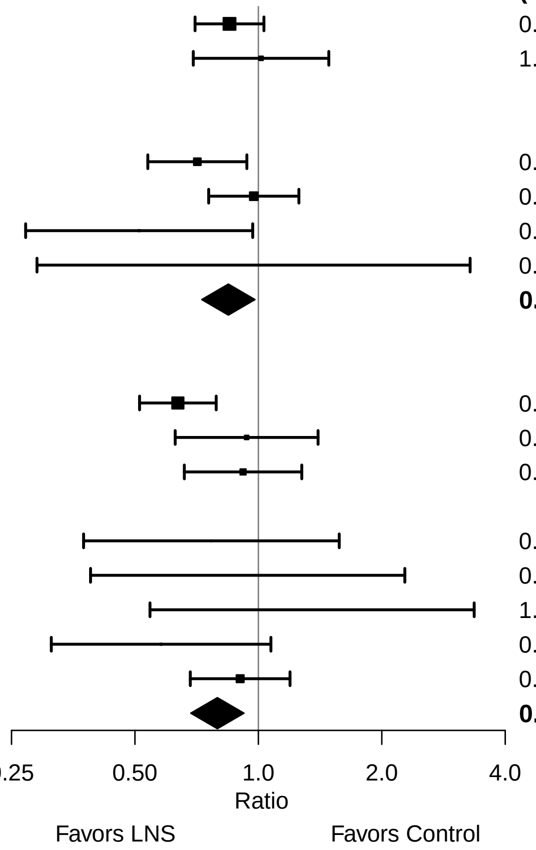

Supplemental figure 6H: Low MUAC prevalence ratio

6H4: Stratified by Source water quality

Source water quality
(p−diff = 0.367)

Country	Trial	N	N	PR (95% CI)	W
**Source water quality − Improved**					
Bangladesh	JiVitA−4 (34)	2897	1271	0.85 (0.70, 1.03)	0.69
Bangladesh	RDNS (35)	1663	815	1.02 (0.69, 1.49)	0.19
Bangladesh	WASH−B (36)				
Ghana	GHANA (39)				
Ghana	iLiNS−DYAD−G (40)	347	693	0.77 (0.37, 1.57)	0.05
Haiti	HAITI (41)				
Malawi	iLiNS−DYAD−M (44)	220	444	0.94 (0.39, 2.28)	0.03
Malawi	iLiNS−DOSE (45)	696	240	1.35 (0.54, 3.36)	0.03
**I² = 0.00, Tau² = 0.00**		**5823**	**3463**	**0.89 (0.76, 1.05)**	
**Source water quality − Unimproved**					
Burkina Faso	iLiNS−Zinc (37)	1959	666	0.64 (0.51, 0.79)	0.29
Burkina Faso	PROMIS (38)	854	912	0.94 (0.63, 1.40)	0.09
Burkina Faso	PROMIS CS (38)	424	436	0.92 (0.66, 1.28)	0.13
Kenya	WASH−B (42)	697	2270	0.67 (0.41, 1.10)	0.06
Madagascar	MAHAY (43)	1680	1658	0.97 (0.76, 1.26)	0.21
Mali	PROMIS (46)	507	505	0.58 (0.31, 1.07)	0.04
Mali	PROMIS CS (46)	954	970	0.90 (0.68, 1.19)	0.17
Zimbabwe	SHINE (HIV−) (47)	889	871	0.65 (0.25, 1.68)	0.02
Zimbabwe	SHINE (HIV+) (48)	147	146	0.50 (0.05, 5.43)	0.00
**I² = 0.24, Tau² = 0.01**		**8111**	**8434**	**0.80 (0.70, 0.92)**	

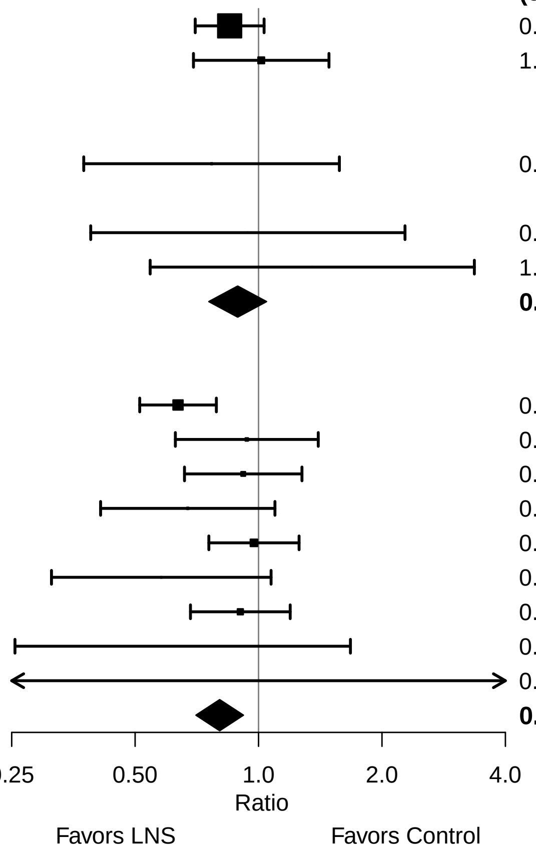

Supplemental figure 6H: Low MUAC prevalence ratio

6H5: Stratified by Sanitation

Sanitation
(p−diff = 0.291)

Country	Trial	N	N	PR (95% CI)	W
**Sanitation − Improved**					
Bangladesh	JiVitA−4 (34)	2897	1271	0.85 (0.70, 1.03)	0.40
Bangladesh	RDNS (35)	1663	815	1.02 (0.69, 1.49)	0.11
Bangladesh	WASH−B (36)				
Burkina Faso	PROMIS (38)	854	912	0.94 (0.63, 1.40)	0.10
Burkina Faso	PROMIS CS (38)	424	436	0.92 (0.66, 1.28)	0.14
Ghana	GHANA (39)				
Ghana	iLiNS−DYAD−G (40)	347	693	0.77 (0.37, 1.57)	0.03
Haiti	HAITI (41)				
Mali	PROMIS (46)	507	505	0.58 (0.31, 1.07)	0.04
Mali	PROMIS CS (46)	954	970	0.90 (0.68, 1.19)	0.19
**I² = 0.00, Tau² = 0.00**		**7646**	**5602**	**0.88 (0.78, 0.99)**	
**Sanitation − Unimproved**					
Burkina Faso	iLiNS−Zinc (37)	1959	666	0.64 (0.51, 0.79)	0.47
Kenya	WASH−B (42)	697	2270	0.67 (0.41, 1.10)	0.09
Madagascar	MAHAY (43)	1680	1658	0.97 (0.76, 1.26)	0.35
Malawi	iLiNS−DYAD−M (44)	220	444	0.94 (0.39, 2.28)	0.03
Malawi	iLiNS−DOSE (45)	696	240	1.35 (0.54, 3.36)	0.03
Zimbabwe	SHINE (HIV−) (47)	889	871	0.65 (0.25, 1.68)	0.03
Zimbabwe	SHINE (HIV+) (48)	147	146	0.50 (0.05, 5.43)	0.00
**I² = 0.30, Tau² = 0.01**		**6288**	**6295**	**0.77 (0.65, 0.93)**	

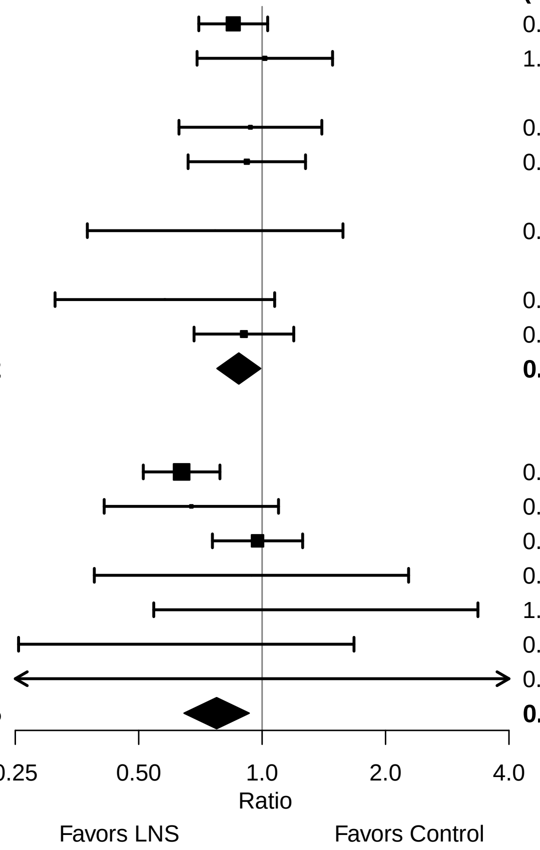

Supplemental figure 6H: Low MUAC prevalence ratio

6H6: Stratified by Supplement duration

Supplement duration
(p−diff = 0.928)

Country	Trial	N	N	PR (95% CI)	W
**Supplement duration − 12m or less**					
Bangladesh	JiVitA−4 (34)	2897	1271	0.85 (0.70, 1.03)	0.27
Burkina Faso	iLiNS−Zinc (37)	1959	666	0.64 (0.51, 0.79)	0.23
Burkina Faso	PROMIS (38)	854	912	0.94 (0.63, 1.40)	0.08
Burkina Faso	PROMIS CS (38)	424	436	0.92 (0.66, 1.28)	0.12
Ghana	GHANA (39)				
Ghana	iLiNS−DYAD−G (40)	347	693	0.77 (0.37, 1.57)	0.03
Haiti	HAITI (41)				
Madagascar	MAHAY (43)	1680	1658	0.97 (0.76, 1.26)	0.18
Malawi	iLiNS−DYAD−M (44)	220	444	0.94 (0.39, 2.28)	0.02
Malawi	iLiNS−DOSE (45)	696	240	1.35 (0.54, 3.36)	0.02
Zimbabwe	SHINE (HIV−) (47)	1872	1780	0.51 (0.27, 0.97)	0.03
Zimbabwe	SHINE (HIV+) (48)	337	328	0.97 (0.29, 3.28)	0.01
**I^2 = 0.22, Tau^2 = 0.01**		**11286**	**8428**	**0.82 (0.72, 0.93)**	
**Supplement duration − > 12m**					
Bangladesh	RDNS (35)	1663	815	1.02 (0.69, 1.49)	0.21
Bangladesh	WASH−B (36)				
Kenya	WASH−B (42)	1461	5185	0.71 (0.54, 0.94)	0.36
Mali	PROMIS (46)	507	505	0.58 (0.31, 1.07)	0.08
Mali	PROMIS CS (46)	954	970	0.90 (0.68, 1.19)	0.35
**I^2 = 0.23, Tau^2 = 0.01**		**4585**	**7475**	**0.82 (0.67, 1.00)**	

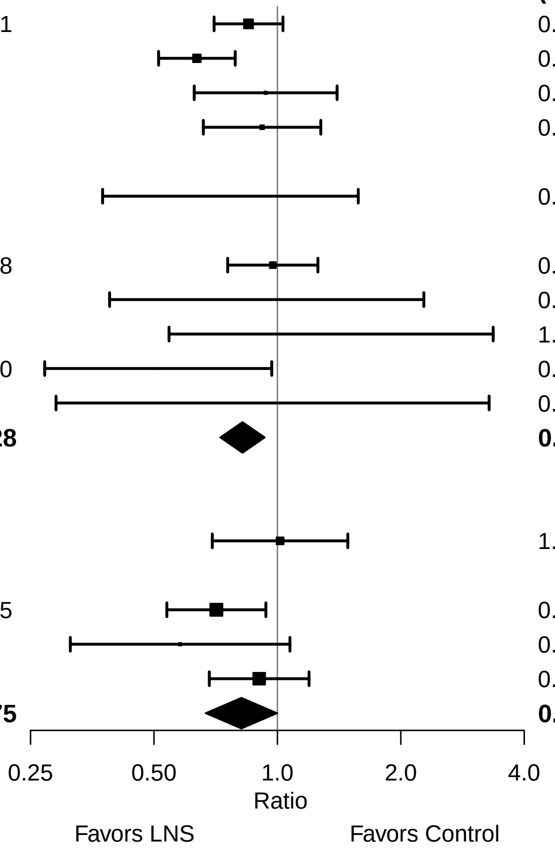

Supplemental figure 6H: Low MUAC prevalence ratio

6H7: Stratified by Frequency of contact

Frequency of contact
(p−diff = 0.354)

Country	Trial	N	N	PR (95% CI)	W
**Frequency of contact − Monthly**					
Bangladesh	RDNS (35)	1663	815	1.02 (0.69, 1.49)	0.11
Burkina Faso	PROMIS (38)	854	912	0.94 (0.63, 1.40)	0.10
Burkina Faso	PROMIS CS (38)	424	436	0.92 (0.66, 1.28)	0.14
Haiti	HAITI (41)				
Kenya	WASH−B (42)	1461	5185	0.71 (0.54, 0.94)	0.18
Madagascar	MAHAY (43)	1680	1658	0.97 (0.76, 1.26)	0.21
Mali	PROMIS (46)	507	505	0.58 (0.31, 1.07)	0.04
Mali	PROMIS CS (46)	954	970	0.90 (0.68, 1.19)	0.18
Zimbabwe	SHINE (HIV−) (47)	1872	1780	0.51 (0.27, 0.97)	0.04
Zimbabwe	SHINE (HIV+) (48)	337	328	0.97 (0.29, 3.28)	0.01
**I² = 0.01, Tau² = 0.00**		**9752**	**12589**	**0.86 (0.76, 0.97)**	
**Frequency of contact − Weekly**					
Bangladesh	JiVitA−4 (34)	2897	1271	0.85 (0.70, 1.03)	0.48
Bangladesh	WASH−B (36)				
Burkina Faso	iLiNS−Zinc (37)	1959	666	0.64 (0.51, 0.79)	0.41
Ghana	GHANA (39)				
Ghana	iLiNS−DYAD−G (40)	347	693	0.77 (0.37, 1.57)	0.05
Malawi	iLiNS−DYAD−M (44)	220	444	0.94 (0.39, 2.28)	0.03
Malawi	iLiNS−DOSE (45)	696	240	1.35 (0.54, 3.36)	0.03
**I² = 0.30, Tau² = 0.01**		**6119**	**3314**	**0.77 (0.64, 0.91)**	

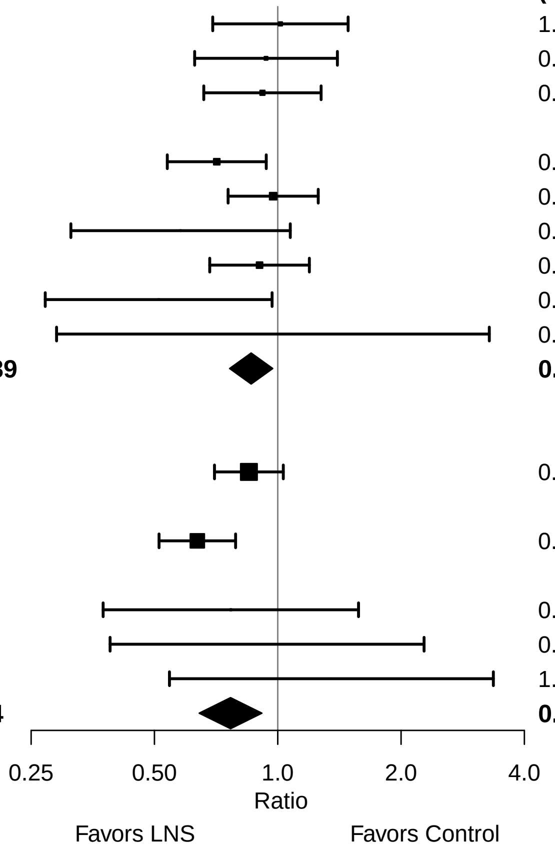

Supplemental figure 6H: Low MUAC prevalence ratio

6H8: Stratified by Average SQ-LNS compliance

Average SQ−LNS compliance
(p−diff = 0.377)

Country	Trial	N	N	PR (95% CI)	W
**Average SQ−LNS compliance − Low**					
Burkina Faso	PROMIS (38)	854	912	0.94 (0.63, 1.40)	0.16
Burkina Faso	PROMIS CS (38)	424	436	0.92 (0.66, 1.28)	0.24
Ghana	iLiNS−DYAD−G (40)	347	693	0.77 (0.37, 1.57)	0.05
Malawi	iLiNS−DYAD−M (44)	220	444	0.94 (0.39, 2.28)	0.04
Malawi	iLiNS−DOSE (45)	696	240	1.35 (0.54, 3.36)	0.03
Mali	PROMIS (46)	507	505	0.58 (0.31, 1.07)	0.07
Mali	PROMIS CS (46)	954	970	0.90 (0.68, 1.19)	0.32
Zimbabwe	SHINE (HIV−) (47)	1872	1780	0.51 (0.27, 0.97)	0.07
Zimbabwe	SHINE (HIV+) (48)	337	328	0.97 (0.29, 3.28)	0.02
**I² = 0.00, Tau² = 0.00**		**6211**	**6308**	**0.86 (0.73, 1.01)**	
**Average SQ−LNS compliance − High**					
Bangladesh	JiVitA−4 (34)	2897	1271	0.85 (0.70, 1.03)	0.37
Bangladesh	RDNS (35)	1663	815	1.02 (0.69, 1.49)	0.11
Bangladesh	WASH−B (36)				
Burkina Faso	iLiNS−Zinc (37)	1959	666	0.64 (0.51, 0.79)	0.31
Ghana	GHANA (39)				
Haiti	HAITI (41)				
Kenya	WASH−B (42)	1461	5185	0.71 (0.54, 0.94)	0.20
**I² = 0.53, Tau² = 0.02**		**7980**	**7937**	**0.77 (0.64, 0.93)**	

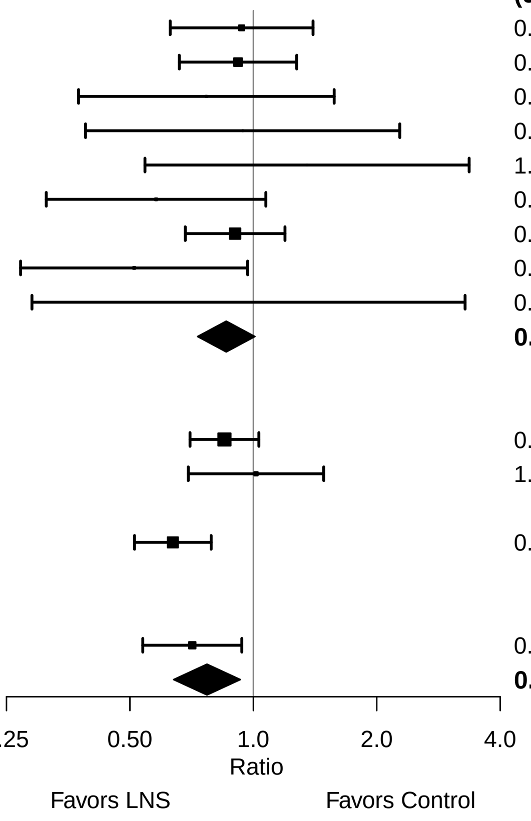

Supplemental figure 6I: Low MUAC prevalence difference

6I1: Stratified by Geographic region (insufficient comparisons)

Supplemental figure 6I: Low MUAC prevalence difference

6I2: Stratified by Stunting burden

Stunting burden
(p−diff = 0.829)

Stunting burden − Less than 35%

**Country**	**Trial**	**N**	**N**	**PD (95% CI)**	**W**
Burkina Faso	PROMIS (38)	854	912	−0.01 (−0.03, 0.02)	0.20
Burkina Faso	PROMIS CS (38)	424	436	−0.01 (−0.06, 0.03)	0.09
Ghana	GHANA (39)				
Ghana	iLiNS−DYAD−G (40)	347	693	−0.01 (−0.03, 0.01)	0.22
Haiti	HAITI (41)				
Kenya	WASH−B (42)	1461	5185	−0.02 (−0.03, 0.00)	0.32
Malawi	iLiNS−DYAD−M (44)	220	444	0.00 (−0.03, 0.03)	0.17
**I² = 0.00, Tau² = 0.00**		**3306**	**7670**	**−0.01 (−0.02, 0.00)**	

Stunting burden − More than 35%

**Country**	**Trial**	**N**	**N**	**PD (95% CI)**	**W**
Bangladesh	JiVitA−4 (34)	2897	1271	−0.02 (−0.04, 0.00)	0.11
Bangladesh	RDNS (35)	1663	815	0.00 (−0.02, 0.02)	0.12
Bangladesh	WASH−B (36)				
Burkina Faso	iLiNS−Zinc (37)	1959	666	−0.07 (−0.10, −0.04)	0.08
Madagascar	MAHAY (43)	1680	1658	0.00 (−0.02, 0.02)	0.11
Malawi	iLiNS−DOSE (45)	696	240	0.01 (−0.01, 0.03)	0.11
Mali	PROMIS (46)	507	505	−0.02 (−0.05, 0.00)	0.09
Mali	PROMIS CS (46)	954	970	−0.01 (−0.04, 0.02)	0.09
Zimbabwe	SHINE (HIV−) (47)	1872	1780	−0.01 (−0.01, 0.00)	0.17
Zimbabwe	SHINE (HIV+) (48)	337	328	0.00 (−0.02, 0.02)	0.12
**I² = 0.67, Tau² = 0.00**		**12565**	**8233**	**−0.01 (−0.03, 0.00)**	

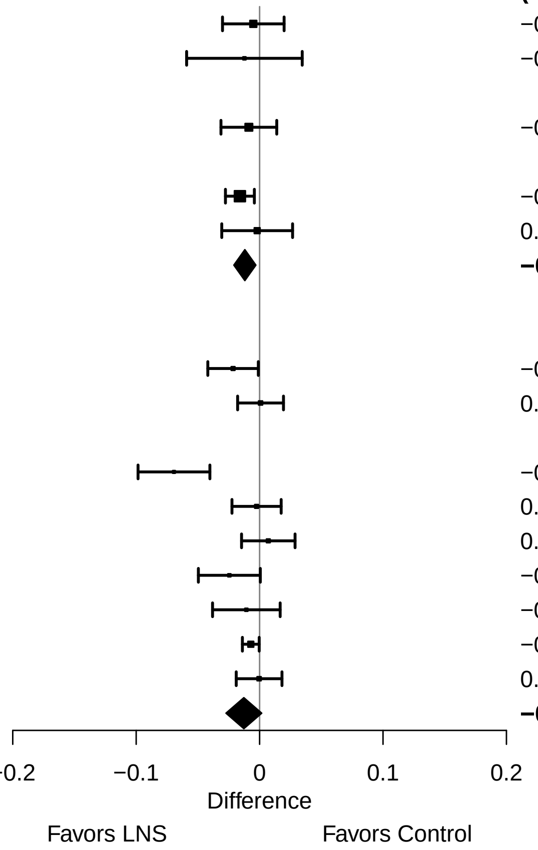

Supplemental figure 6I: Low MUAC prevalence difference

6I3: Stratified by Malaria prevalence

Malaria prevalence
(p−diff = 0.447)

Country	Trial	N	N	PD (95% CI)	W
**Malaria prevalence − Less than 10%**					
Bangladesh	JiVitA−4 (34)	2897	1271	−0.02 (−0.04, 0.00)	0.14
Bangladesh	RDNS (35)	1663	815	0.00 (−0.02, 0.02)	0.15
Bangladesh	WASH−B (36)				
Haiti	HAITI (41)				
Kenya	WASH−B (42)	1461	5185	−0.02 (−0.03, 0.00)	0.19
Madagascar	MAHAY (43)	1680	1658	0.00 (−0.02, 0.02)	0.15
Zimbabwe	SHINE (HIV−) (47)	1872	1780	−0.01 (−0.01, 0.00)	0.22
Zimbabwe	SHINE (HIV+) (48)	337	328	0.00 (−0.02, 0.02)	0.15
**I² = 0.06, Tau² = 0.00**		**9910**	**11037**	**−0.01 (−0.01, 0.00)**	
**Malaria prevalence − At least 10%**					
Burkina Faso	iLiNS−Zinc (37)	1959	666	−0.07 (−0.10, −0.04)	0.12
Burkina Faso	PROMIS (38)	854	912	−0.01 (−0.03, 0.02)	0.14
Burkina Faso	PROMIS CS (38)	424	436	−0.01 (−0.06, 0.03)	0.06
Ghana	GHANA (39)				
Ghana	iLiNS−DYAD−G (40)	347	693	−0.01 (−0.03, 0.01)	0.15
Malawi	iLiNS−DYAD−M (44)	220	444	0.00 (−0.03, 0.03)	0.12
Malawi	iLiNS−DOSE (45)	696	240	0.01 (−0.01, 0.03)	0.16
Mali	PROMIS (46)	507	505	−0.02 (−0.05, 0.00)	0.14
Mali	PROMIS CS (46)	954	970	−0.01 (−0.04, 0.02)	0.12
**I² = 0.64, Tau² = 0.00**		**5961**	**4866**	**−0.02 (−0.03, 0.00)**	

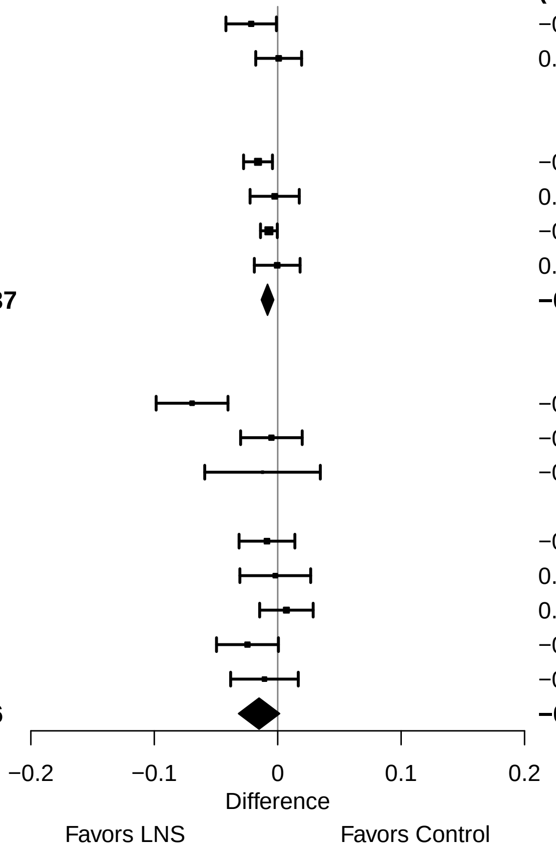

Supplemental figure 6I: Low MUAC prevalence difference

6I4: Stratified by Source water quality

Source water quality
(p−diff = 0.262)

Country	Trial	N	N	PD (95% CI)	W
**Source water quality − Improved**					
Bangladesh	JiVitA−4 (34)	2897	1271	−0.02 (−0.04, 0.00)	0.22
Bangladesh	RDNS (35)	1663	815	0.00 (−0.02, 0.02)	0.23
Bangladesh	WASH−B (36)				
Ghana	GHANA (39)				
Ghana	iLiNS−DYAD−G (40)	347	693	−0.01 (−0.03, 0.01)	0.20
Haiti	HAITI (41)				
Malawi	iLiNS−DYAD−M (44)	220	444	0.00 (−0.03, 0.03)	0.15
Malawi	iLiNS−DOSE (45)	696	240	0.01 (−0.01, 0.03)	0.20
**I² = 0.05, Tau² = 0.00**		**5823**	**3463**	**−0.01 (−0.01, 0.00)**	
**Source water quality − Unimproved**					
Burkina Faso	iLiNS−Zinc (37)	1959	666	−0.07 (−0.10, −0.04)	0.08
Burkina Faso	PROMIS (38)	854	912	−0.01 (−0.03, 0.02)	0.10
Burkina Faso	PROMIS CS (38)	424	436	−0.01 (−0.06, 0.03)	0.04
Kenya	WASH−B (42)	697	2270	−0.02 (−0.04, 0.00)	0.15
Madagascar	MAHAY (43)	1680	1658	0.00 (−0.02, 0.02)	0.13
Mali	PROMIS (46)	507	505	−0.02 (−0.05, 0.00)	0.10
Mali	PROMIS CS (46)	954	970	−0.01 (−0.04, 0.02)	0.09
Zimbabwe	SHINE (HIV−) (47)	889	871	0.00 (−0.01, 0.01)	0.19
Zimbabwe	SHINE (HIV+) (48)	147	146	−0.01 (−0.03, 0.02)	0.11
**I² = 0.60, Tau² = 0.00**		**8111**	**8434**	**−0.02 (−0.03, 0.00)**	

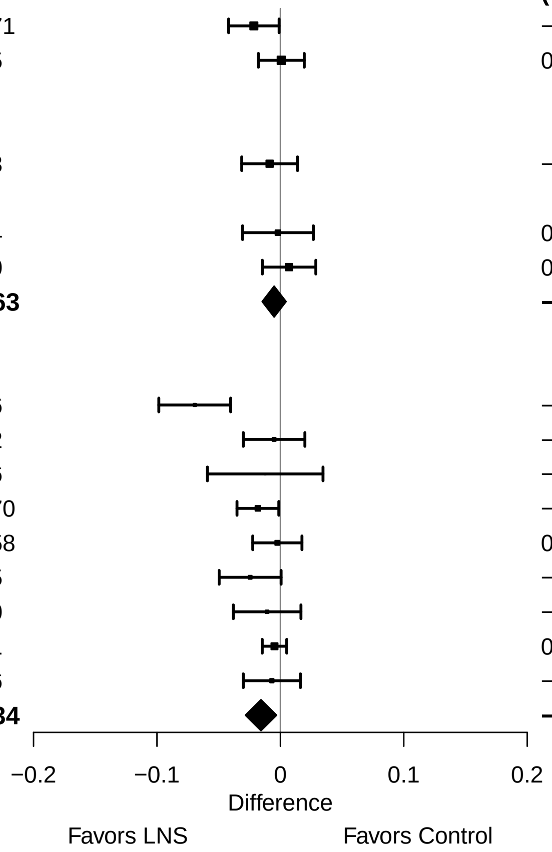

Supplemental figure 6I: Low MUAC prevalence difference

6I5: Stratified by Sanitation

Sanitation
(p−diff = 0.982)

Country	Trial	N	N	PD (95% CI)	W
**Sanitation − Improved**					
Bangladesh	JiVitA−4 (34)	2897	1271	−0.02 (−0.04, 0.00)	0.18
Bangladesh	RDNS (35)	1663	815	0.00 (−0.02, 0.02)	0.19
Bangladesh	WASH−B (36)				
Burkina Faso	PROMIS (38)	854	912	−0.01 (−0.03, 0.02)	0.14
Burkina Faso	PROMIS CS (38)	424	436	−0.01 (−0.06, 0.03)	0.06
Ghana	GHANA (39)				
Ghana	iLiNS−DYAD−G (40)	347	693	−0.01 (−0.03, 0.01)	0.16
Haiti	HAITI (41)				
Mali	PROMIS (46)	507	505	−0.02 (−0.05, 0.00)	0.14
Mali	PROMIS CS (46)	954	970	−0.01 (−0.04, 0.02)	0.13
**I² = 0.00, Tau² = 0.00**		**7646**	**5602**	**−0.01 (−0.02, 0.00)**	
**Sanitation − Unimproved**					
Burkina Faso	iLiNS−Zinc (37)	1959	666	−0.07 (−0.10, −0.04)	0.10
Kenya	WASH−B (42)	697	2270	−0.02 (−0.04, 0.00)	0.17
Madagascar	MAHAY (43)	1680	1658	0.00 (−0.02, 0.02)	0.15
Malawi	iLiNS−DYAD−M (44)	220	444	0.00 (−0.03, 0.03)	0.10
Malawi	iLiNS−DOSE (45)	696	240	0.01 (−0.01, 0.03)	0.14
Zimbabwe	SHINE (HIV−) (47)	889	871	0.00 (−0.01, 0.01)	0.22
Zimbabwe	SHINE (HIV+) (48)	147	146	−0.01 (−0.03, 0.02)	0.13
**I² = 0.72, Tau² = 0.00**		**6288**	**6295**	**−0.01 (−0.03, 0.01)**	

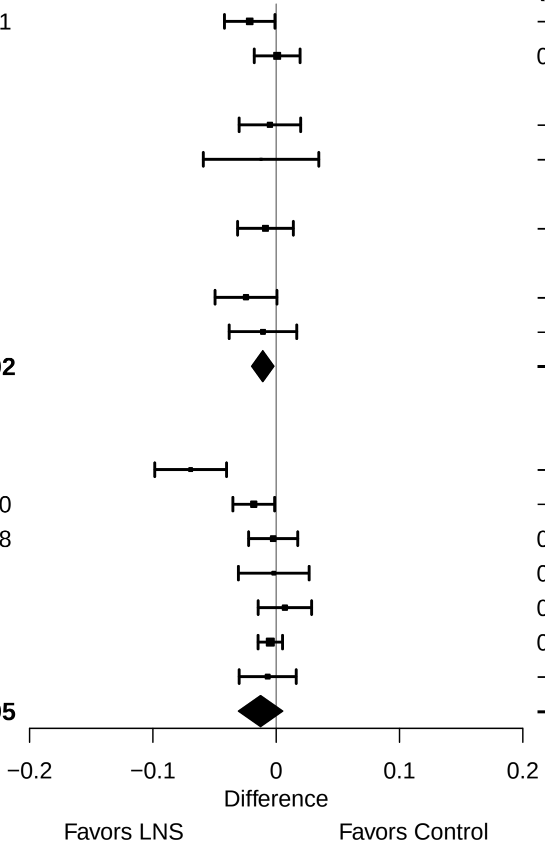

Supplemental figure 6I: Low MUAC prevalence difference

6I6: Stratified by Supplement duration

Supplement duration
(p−diff = 0.842)

Country	Trial	N	N	PD (95% CI)	W
**Supplement duration − 12m or less**					
Bangladesh	JiVitA−4 (34)	2897	1271	−0.02 (−0.04, 0.00)	0.11
Burkina Faso	iLiNS−Zinc (37)	1959	666	−0.07 (−0.10, −0.04)	0.08
Burkina Faso	PROMIS (38)	854	912	−0.01 (−0.03, 0.02)	0.09
Burkina Faso	PROMIS CS (38)	424	436	−0.01 (−0.06, 0.03)	0.04
Ghana	GHANA (39)				
Ghana	iLiNS−DYAD−G (40)	347	693	−0.01 (−0.03, 0.01)	0.10
Haiti	HAITI (41)				
Madagascar	MAHAY (43)	1680	1658	0.00 (−0.02, 0.02)	0.11
Malawi	iLiNS−DYAD−M (44)	220	444	0.00 (−0.03, 0.03)	0.08
Malawi	iLiNS−DOSE (45)	696	240	0.01 (−0.01, 0.03)	0.10
Zimbabwe	SHINE (HIV−) (47)	1872	1780	−0.01 (−0.01, 0.00)	0.17
Zimbabwe	SHINE (HIV+) (48)	337	328	0.00 (−0.02, 0.02)	0.12
**I² = 0.59, Tau² = 0.00**		**11286**	**8428**	**−0.01 (−0.02, 0.00)**	
**Supplement duration − > 12m**					
Bangladesh	RDNS (35)	1663	815	0.00 (−0.02, 0.02)	0.27
Bangladesh	WASH−B (36)				
Kenya	WASH−B (42)	1461	5185	−0.02 (−0.03, 0.00)	0.33
Mali	PROMIS (46)	507	505	−0.02 (−0.05, 0.00)	0.21
Mali	PROMIS CS (46)	954	970	−0.01 (−0.04, 0.02)	0.19
**I² = 0.06, Tau² = 0.00**		**4585**	**7475**	**−0.01 (−0.02, 0.00)**	

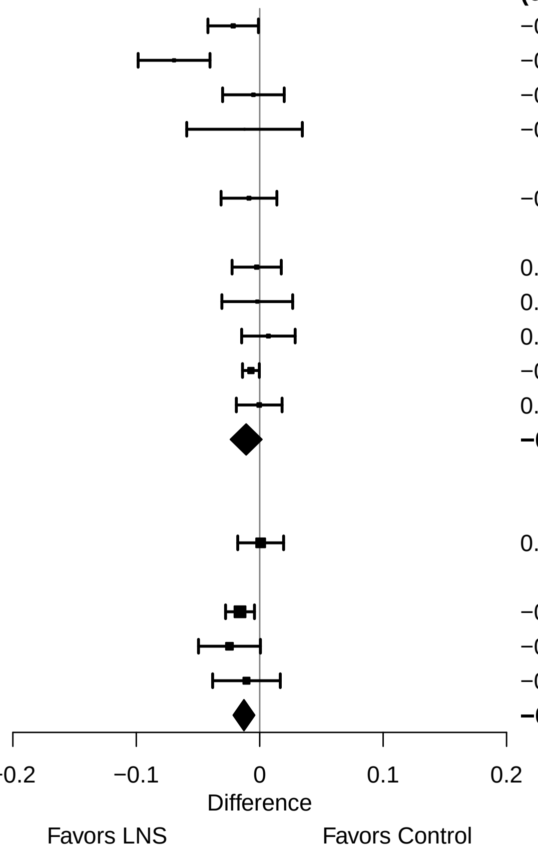

Supplemental figure 6I: Low MUAC prevalence difference

6I7: Stratified by Frequency of contact

Frequency of contact
(p−diff = 0.331)

Country	Trial	N	N	PD (95% CI)	W
**Frequency of contact − Monthly**					
Bangladesh	RDNS (35)	1663	815	0.00 (−0.02, 0.02)	0.12
Burkina Faso	PROMIS (38)	854	912	−0.01 (−0.03, 0.02)	0.10
Burkina Faso	PROMIS CS (38)	424	436	−0.01 (−0.06, 0.03)	0.04
Haiti	HAITI (41)				
Kenya	WASH−B (42)	1461	5185	−0.02 (−0.03, 0.00)	0.15
Madagascar	MAHAY (43)	1680	1658	0.00 (−0.02, 0.02)	0.12
Mali	PROMIS (46)	507	505	−0.02 (−0.05, 0.00)	0.09
Mali	PROMIS CS (46)	954	970	−0.01 (−0.04, 0.02)	0.09
Zimbabwe	SHINE (HIV−) (47)	1872	1780	−0.01 (−0.01, 0.00)	0.17
Zimbabwe	SHINE (HIV+) (48)	337	328	0.00 (−0.02, 0.02)	0.12
**I² = 0.00, Tau² = 0.00**		**9752**	**12589**	**−0.01 (−0.01, 0.00)**	
**Frequency of contact − Weekly**					
Bangladesh	JiVitA−4 (34)	2897	1271	−0.02 (−0.04, 0.00)	0.23
Bangladesh	WASH−B (36)				
Burkina Faso	iLiNS−Zinc (37)	1959	666	−0.07 (−0.10, −0.04)	0.17
Ghana	GHANA (39)				
Ghana	iLiNS−DYAD−G (40)	347	693	−0.01 (−0.03, 0.01)	0.21
Malawi	iLiNS−DYAD−M (44)	220	444	0.00 (−0.03, 0.03)	0.17
Malawi	iLiNS−DOSE (45)	696	240	0.01 (−0.01, 0.03)	0.22
**I² = 0.79, Tau² = 0.00**		**6119**	**3314**	**−0.02 (−0.04, 0.01)**	

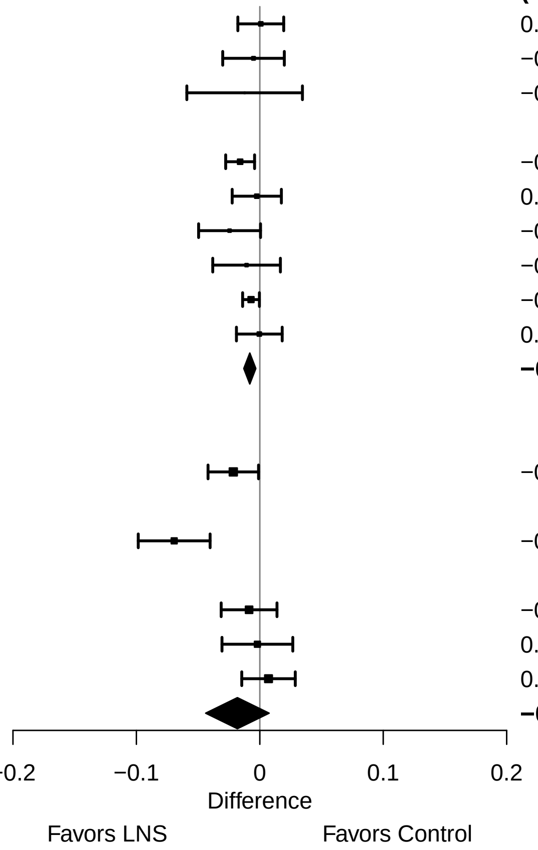

Supplemental figure 6I: Low MUAC prevalence difference

6I8: Stratified by Average SQ-LNS compliance

Average SQ−LNS compliance
(p−diff = 0.094)

Average SQ−LNS compliance − Low

Country	Trial	N	N	PD (95% CI)	W
Burkina Faso	PROMIS (38)	854	912	−0.01 (−0.03, 0.02)	0.11
Burkina Faso	PROMIS CS (38)	424	436	−0.01 (−0.06, 0.03)	0.05
Ghana	iLiNS−DYAD−G (40)	347	693	−0.01 (−0.03, 0.01)	0.12
Malawi	iLiNS−DYAD−M (44)	220	444	0.00 (−0.03, 0.03)	0.09
Malawi	iLiNS−DOSE (45)	696	240	0.01 (−0.01, 0.03)	0.12
Mali	PROMIS (46)	507	505	−0.02 (−0.05, 0.00)	0.11
Mali	PROMIS CS (46)	954	970	−0.01 (−0.04, 0.02)	0.10
Zimbabwe	SHINE (HIV−) (47)	1872	1780	−0.01 (−0.01, 0.00)	0.18
Zimbabwe	SHINE (HIV+) (48)	337	328	0.00 (−0.02, 0.02)	0.13
**I² = 0.00, Tau² = 0.00**		**6211**	**6308**	**−0.01 (−0.01, 0.00)**	
**Average SQ−LNS compliance − High**					
Bangladesh	JiVitA−4 (34)	2897	1271	−0.02 (−0.04, 0.00)	0.24
Bangladesh	RDNS (35)	1663	815	0.00 (−0.02, 0.02)	0.26
Bangladesh	WASH−B (36)				
Burkina Faso	iLiNS−Zinc (37)	1959	666	−0.07 (−0.10, −0.04)	0.18
Ghana	GHANA (39)				
Haiti	HAITI (41)				
Kenya	WASH−B (42)	1461	5185	−0.02 (−0.03, 0.00)	0.32
**I² = 0.81, Tau² = 0.00**		**7980**	**7937**	**−0.02 (−0.05, 0.00)**	

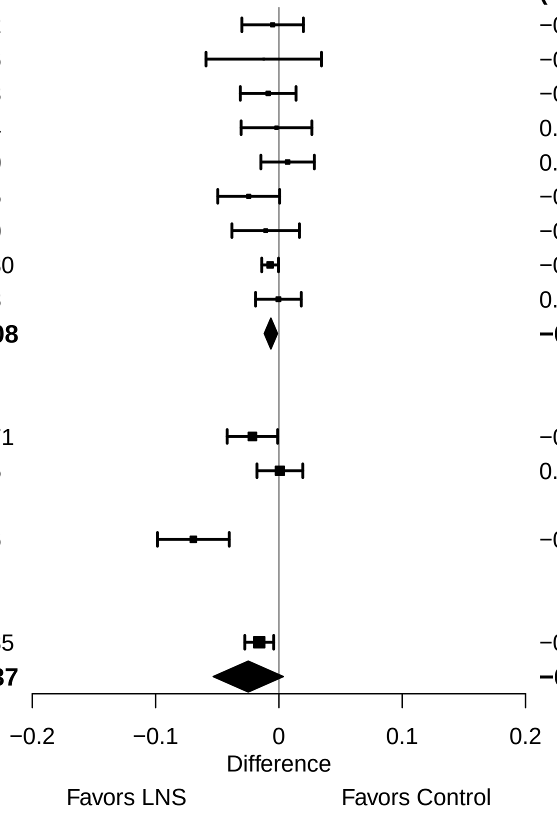

Supplemental figure 6J: Acute malnutrition prevalence ratio

6J1: Stratified by Geographic region (insufficient comparisons)

Supplemental figure 6J: Acute malnutrition prevalence ratio

6J2: Stratified by Stunting burden

Stunting burden
(p−diff = 0.835)

Country	Trial	N	N	PR (95% CI)	W
**Stunting burden − Less than 35%**					
Burkina Faso	PROMIS (38)	846	901	0.83 (0.60, 1.14)	0.26
Burkina Faso	PROMIS CS (38)	424	434	0.95 (0.73, 1.23)	0.38
Ghana	GHANA (39)				
Ghana	iLiNS−DYAD−G (40)	347	692	0.85 (0.54, 1.35)	0.12
Haiti	HAITI (41)				
Kenya	WASH−B (42)	1454	5117	0.71 (0.49, 1.02)	0.20
Malawi	iLiNS−DYAD−M (44)	220	444	0.81 (0.36, 1.80)	0.04
**I² = 0.00, Tau² = 0.00**		**3291**	**7588**	**0.85 (0.72, 1.00)**	
**Stunting burden − More than 35%**					
Bangladesh	JiVitA−4 (34)	2783	1216	0.89 (0.78, 1.01)	0.41
Bangladesh	RDNS (35)	1661	815	0.86 (0.70, 1.05)	0.16
Bangladesh	WASH−B (36)				
Burkina Faso	iLiNS−Zinc (37)	1951	664	0.67 (0.54, 0.85)	0.13
Madagascar	MAHAY (43)	1675	1655	0.98 (0.75, 1.29)	0.09
Malawi	iLiNS−DOSE (45)	690	240	1.42 (0.83, 2.43)	0.02
Mali	PROMIS (46)	499	502	0.59 (0.23, 1.47)	0.01
Mali	PROMIS CS (46)	944	959	0.89 (0.70, 1.13)	0.12
Zimbabwe	SHINE (HIV−) (47)	1867	1777	0.79 (0.55, 1.14)	0.05
Zimbabwe	SHINE (HIV+) (48)	336	327	1.23 (0.61, 2.48)	0.01
**I² = 0.27, Tau² = 0.01**		**12406**	**8155**	**0.87 (0.77, 0.98)**	

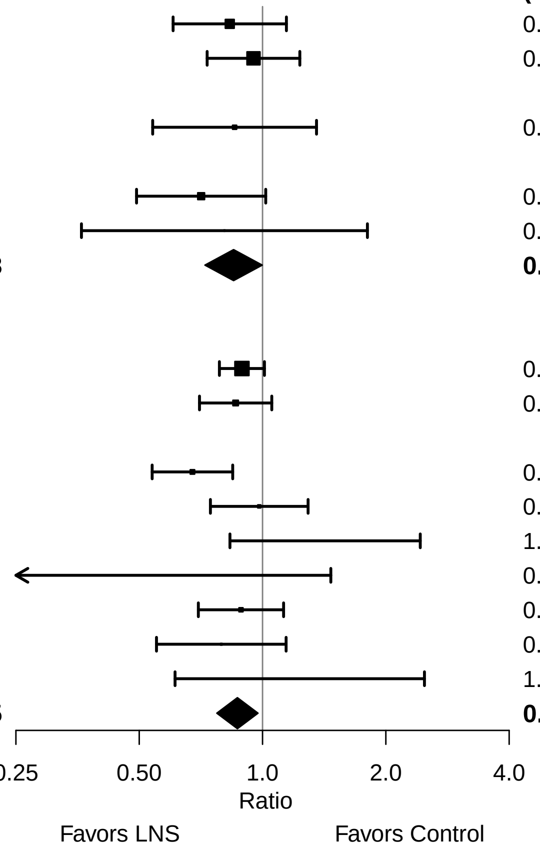

Supplemental figure 6J: Acute malnutrition prevalence ratio

6J3: Stratified by Malaria prevalence

Malaria prevalence
(p−diff = 0.526)

**Country**	**Trial**	**N**	**N**	**PR (95% CI)**	**W**
**Malaria prevalence − Less than 10%**					
Bangladesh	JiVitA−4 (34)	2783	1216	0.89 (0.78, 1.01)	0.53
Bangladesh	RDNS (35)	1661	815	0.86 (0.70, 1.05)	0.21
Bangladesh	WASH−B (36)				
Haiti	HAITI (41)				
Kenya	WASH−B (42)	1454	5117	0.71 (0.49, 1.02)	0.06
Madagascar	MAHAY (43)	1675	1655	0.98 (0.75, 1.29)	0.11
Zimbabwe	SHINE (HIV−) (47)	1867	1777	0.79 (0.55, 1.14)	0.06
Zimbabwe	SHINE (HIV+) (48)	336	327	1.23 (0.61, 2.48)	0.02
**I² = 0.00, Tau² = 0.00**		**9776**	**10907**	**0.88 (0.80, 0.96)**	
**Malaria prevalence − At least 10%**					
Burkina Faso	iLiNS−Zinc (37)	1951	664	0.67 (0.54, 0.85)	0.27
Burkina Faso	PROMIS (38)	846	901	0.83 (0.60, 1.14)	0.14
Burkina Faso	PROMIS CS (38)	424	434	0.95 (0.73, 1.23)	0.20
Ghana	GHANA (39)				
Ghana	iLiNS−DYAD−G (40)	347	692	0.85 (0.54, 1.35)	0.07
Malawi	iLiNS−DYAD−M (44)	220	444	0.81 (0.36, 1.80)	0.02
Malawi	iLiNS−DOSE (45)	690	240	1.42 (0.83, 2.43)	0.05
Mali	PROMIS (46)	499	502	0.59 (0.23, 1.47)	0.02
Mali	PROMIS CS (46)	944	959	0.89 (0.70, 1.13)	0.24
**I² = 0.22, Tau² = 0.01**		**5921**	**4836**	**0.85 (0.74, 0.97)**	

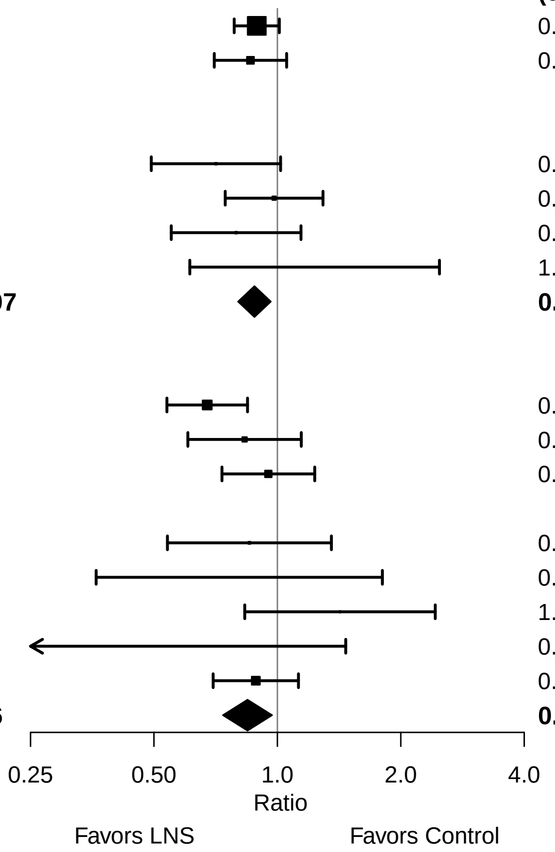

Supplemental figure 6J: Acute malnutrition prevalence ratio

6J4: Stratified by Source water quality

Source water quality
(p−diff = 0.378)

Country	Trial	N	N	PR (95% CI)	W
**Source water quality − Improved**					
Bangladesh	JiVitA−4 (34)	2783	1216	0.89 (0.78, 1.01)	0.65
Bangladesh	RDNS (35)	1661	815	0.86 (0.70, 1.05)	0.25
Bangladesh	WASH−B (36)				
Ghana	GHANA (39)				
Ghana	iLiNS−DYAD−G (40)	347	692	0.85 (0.54, 1.35)	0.05
Haiti	HAITI (41)				
Malawi	iLiNS−DYAD−M (44)	220	444	0.81 (0.36, 1.80)	0.02
Malawi	iLiNS−DOSE (45)	690	240	1.42 (0.83, 2.43)	0.04
**I² = 0.00, Tau² = 0.00**		**5701**	**3407**	**0.89 (0.81, 0.99)**	
**Source water quality − Unimproved**					
Burkina Faso	iLiNS−Zinc (37)	1951	664	0.67 (0.54, 0.85)	0.23
Burkina Faso	PROMIS (38)	846	901	0.83 (0.60, 1.14)	0.12
Burkina Faso	PROMIS CS (38)	424	434	0.95 (0.73, 1.23)	0.18
Kenya	WASH−B (42)	694	2244	0.76 (0.43, 1.34)	0.04
Madagascar	MAHAY (43)	1675	1655	0.98 (0.75, 1.29)	0.16
Mali	PROMIS (46)	499	502	0.59 (0.23, 1.47)	0.01
Mali	PROMIS CS (46)	944	959	0.89 (0.70, 1.13)	0.21
Zimbabwe	SHINE (HIV−) (47)	886	871	0.82 (0.49, 1.37)	0.05
Zimbabwe	SHINE (HIV+) (48)	147	146	0.87 (0.27, 2.79)	0.01
**I² = 0.00, Tau² = 0.00**		**8066**	**8376**	**0.84 (0.75, 0.93)**	

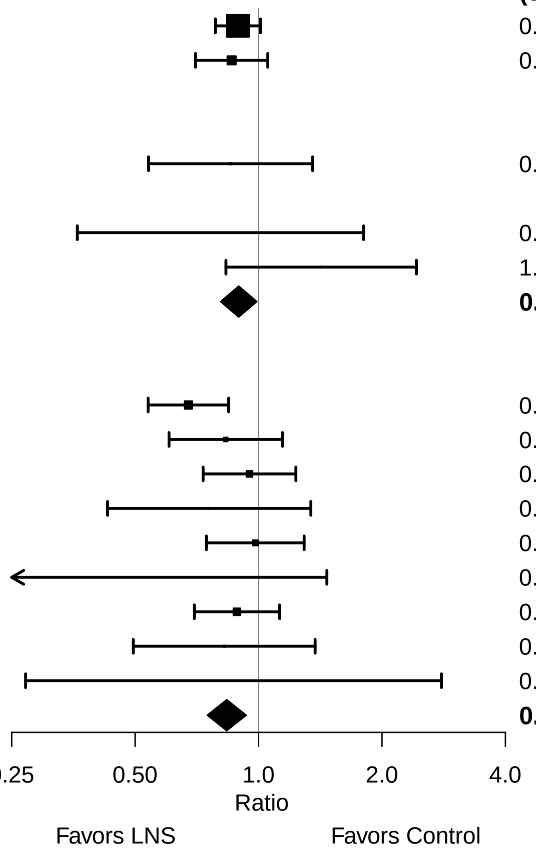

Supplemental figure 6J: Acute malnutrition prevalence ratio

6J5: Stratified by Sanitation

Sanitation
(p−diff = 0.450)

Country	Trial	N	N	PR (95% CI)	W
**Sanitation − Improved**					
Bangladesh	JiVitA−4 (34)	2783	1216	0.89 (0.78, 1.01)	0.46
Bangladesh	RDNS (35)	1661	815	0.86 (0.70, 1.05)	0.18
Bangladesh	WASH−B (36)				
Burkina Faso	PROMIS (38)	846	901	0.83 (0.60, 1.14)	0.07
Burkina Faso	PROMIS CS (38)	424	434	0.95 (0.73, 1.23)	0.11
Ghana	GHANA (39)				
Ghana	iLiNS−DYAD−G (40)	347	692	0.85 (0.54, 1.35)	0.04
Haiti	HAITI (41)				
Mali	PROMIS (46)	499	502	0.59 (0.23, 1.47)	0.01
Mali	PROMIS CS (46)	944	959	0.89 (0.70, 1.13)	0.13
**I² = 0.00, Tau² = 0.00**		**7504**	**5519**	**0.88 (0.81, 0.96)**	
**Sanitation − Unimproved**					
Burkina Faso	iLiNS−Zinc (37)	1951	664	0.67 (0.54, 0.85)	0.43
Kenya	WASH−B (42)	694	2244	0.76 (0.43, 1.34)	0.07
Madagascar	MAHAY (43)	1675	1655	0.98 (0.75, 1.29)	0.29
Malawi	iLiNS−DYAD−M (44)	220	444	0.81 (0.36, 1.80)	0.03
Malawi	iLiNS−DOSE (45)	690	240	1.42 (0.83, 2.43)	0.08
Zimbabwe	SHINE (HIV−) (47)	886	871	0.82 (0.49, 1.37)	0.08
Zimbabwe	SHINE (HIV+) (48)	147	146	0.87 (0.27, 2.79)	0.02
**I² = 0.31, Tau² = 0.01**		**6263**	**6264**	**0.85 (0.70, 1.02)**	

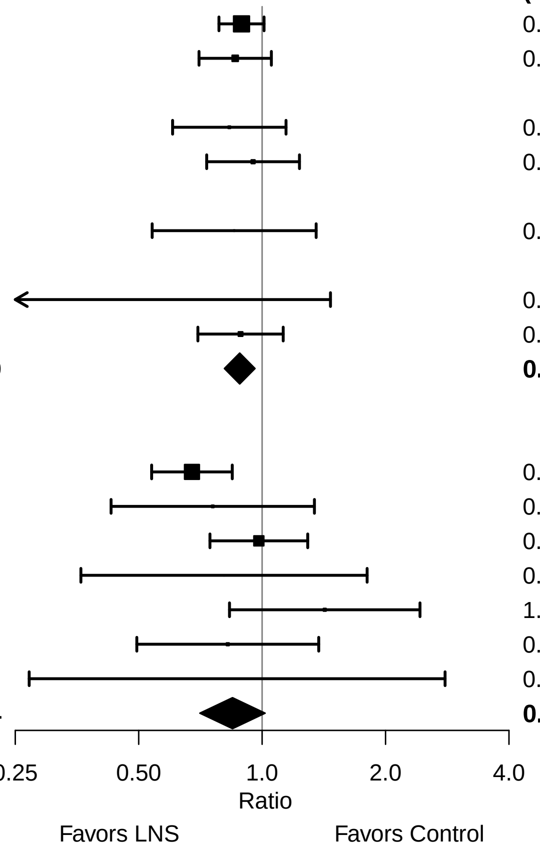

Supplemental figure 6J: Acute malnutrition prevalence ratio

6J6: Stratified by Supplement duration

Supplement duration
(p−diff = 0.607)

Country	Trial	N	N	PR (95% CI)	W
**Supplement duration − 12m or less**					
Bangladesh	JiVitA−4 (34)	2783	1216	0.89 (0.78, 1.01)	0.45
Burkina Faso	iLiNS−Zinc (37)	1951	664	0.67 (0.54, 0.85)	0.14
Burkina Faso	PROMIS (38)	846	901	0.83 (0.60, 1.14)	0.07
Burkina Faso	PROMIS CS (38)	424	434	0.95 (0.73, 1.23)	0.11
Ghana	GHANA (39)				
Ghana	iLiNS−DYAD−G (40)	347	692	0.85 (0.54, 1.35)	0.03
Haiti	HAITI (41)				
Madagascar	MAHAY (43)	1675	1655	0.98 (0.75, 1.29)	0.10
Malawi	iLiNS−DYAD−M (44)	220	444	0.81 (0.36, 1.80)	0.01
Malawi	iLiNS−DOSE (45)	690	240	1.42 (0.83, 2.43)	0.03
Zimbabwe	SHINE (HIV−) (47)	1867	1777	0.79 (0.55, 1.14)	0.05
Zimbabwe	SHINE (HIV+) (48)	336	327	1.23 (0.61, 2.48)	0.01
**I² = 0.16, Tau² = 0.00**		**11139**	**8350**	**0.87 (0.79, 0.97)**	
**Supplement duration − > 12m**					
Bangladesh	RDNS (35)	1661	815	0.86 (0.70, 1.05)	0.48
Bangladesh	WASH−B (36)				
Kenya	WASH−B (42)	1454	5117	0.71 (0.49, 1.02)	0.15
Mali	PROMIS (46)	499	502	0.59 (0.23, 1.47)	0.02
Mali	PROMIS CS (46)	944	959	0.89 (0.70, 1.13)	0.35
**I² = 0.00, Tau² = 0.00**		**4558**	**7393**	**0.84 (0.73, 0.96)**	

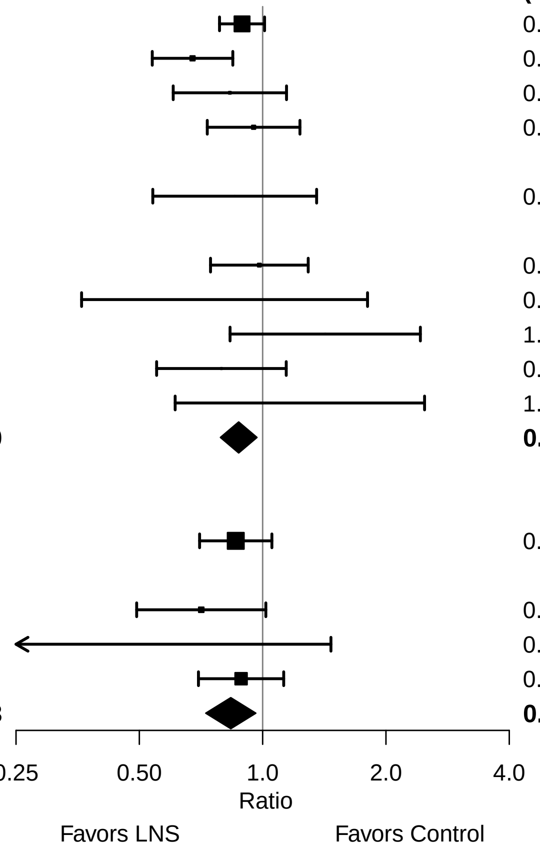

Supplemental figure 6J: Acute malnutrition prevalence ratio

6J7: Stratified by Frequency of contact

Frequency of contact
(p−diff = 0.706)

Country	Trial	N	N	PR (95% CI)	W
**Frequency of contact − Monthly**					
Bangladesh	RDNS (35)	1661	815	0.86 (0.70, 1.05)	0.25
Burkina Faso	PROMIS (38)	846	901	0.83 (0.60, 1.14)	0.10
Burkina Faso	PROMIS CS (38)	424	434	0.95 (0.73, 1.23)	0.15
Haiti	HAITI (41)				
Kenya	WASH−B (42)	1454	5117	0.71 (0.49, 1.02)	0.08
Madagascar	MAHAY (43)	1675	1655	0.98 (0.75, 1.29)	0.14
Mali	PROMIS (46)	499	502	0.59 (0.23, 1.47)	0.01
Mali	PROMIS CS (46)	944	959	0.89 (0.70, 1.13)	0.18
Zimbabwe	SHINE (HIV−) (47)	1867	1777	0.79 (0.55, 1.14)	0.08
Zimbabwe	SHINE (HIV+) (48)	336	327	1.23 (0.61, 2.48)	0.02
**I² = 0.00, Tau² = 0.00**		**9706**	**12487**	**0.87 (0.79, 0.97)**	
**Frequency of contact − Weekly**					
Bangladesh	JiVitA−4 (34)	2783	1216	0.89 (0.78, 1.01)	0.68
Bangladesh	WASH−B (36)				
Burkina Faso	iLiNS−Zinc (37)	1951	664	0.67 (0.54, 0.85)	0.21
Ghana	GHANA (39)				
Ghana	iLiNS−DYAD−G (40)	347	692	0.85 (0.54, 1.35)	0.05
Malawi	iLiNS−DYAD−M (44)	220	444	0.81 (0.36, 1.80)	0.02
Malawi	iLiNS−DOSE (45)	690	240	1.42 (0.83, 2.43)	0.04
**I² = 0.51, Tau² = 0.03**		**5991**	**3256**	**0.86 (0.69, 1.06)**	

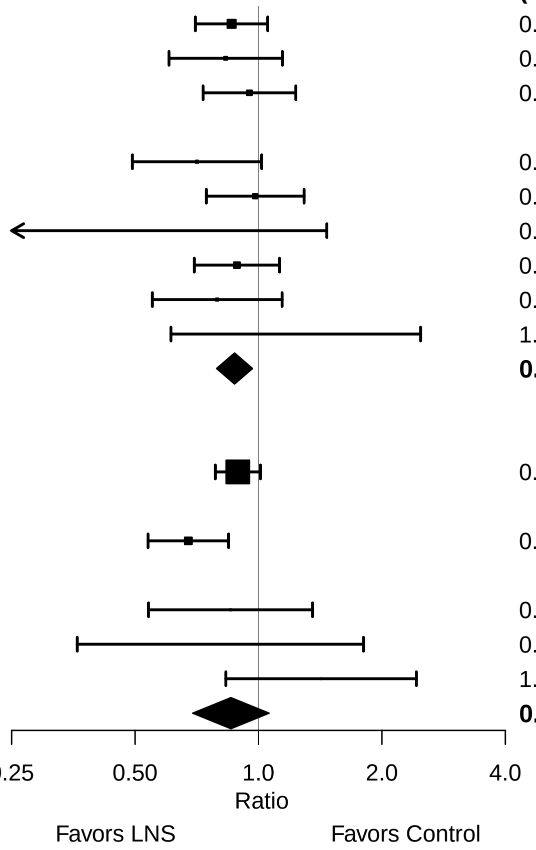

Supplemental figure 6J: Acute malnutrition prevalence ratio

6J8: Stratified by Average SQ-LNS compliance

Average SQ−LNS compliance
(p−diff = 0.269)

Country	Trial	N	N	PR (95% CI)	W
**Average SQ−LNS compliance − Low**					
Burkina Faso	PROMIS (38)	846	901	0.83 (0.60, 1.14)	0.16
Burkina Faso	PROMIS CS (38)	424	434	0.95 (0.73, 1.23)	0.24
Ghana	iLiNS−DYAD−G (40)	347	692	0.85 (0.54, 1.35)	0.08
Malawi	iLiNS−DYAD−M (44)	220	444	0.81 (0.36, 1.80)	0.02
Malawi	iLiNS−DOSE (45)	690	240	1.42 (0.83, 2.43)	0.06
Mali	PROMIS (46)	499	502	0.59 (0.23, 1.47)	0.02
Mali	PROMIS CS (46)	944	959	0.89 (0.70, 1.13)	0.28
Zimbabwe	SHINE (HIV−) (47)	1867	1777	0.79 (0.55, 1.14)	0.12
Zimbabwe	SHINE (HIV+) (48)	336	327	1.23 (0.61, 2.48)	0.03
**I² = 0.00, Tau² = 0.00**		**6173**	**6276**	**0.90 (0.79, 1.02)**	
**Average SQ−LNS compliance − High**					
Bangladesh	JiVitA−4 (34)	2783	1216	0.89 (0.78, 1.01)	0.55
Bangladesh	RDNS (35)	1661	815	0.86 (0.70, 1.05)	0.21
Bangladesh	WASH−B (36)				
Burkina Faso	iLiNS−Zinc (37)	1951	664	0.67 (0.54, 0.85)	0.17
Ghana	GHANA (39)				
Haiti	HAITI (41)				
Kenya	WASH−B (42)	1454	5117	0.71 (0.49, 1.02)	0.07
**I² = 0.43, Tau² = 0.01**		**7849**	**7812**	**0.81 (0.71, 0.92)**	

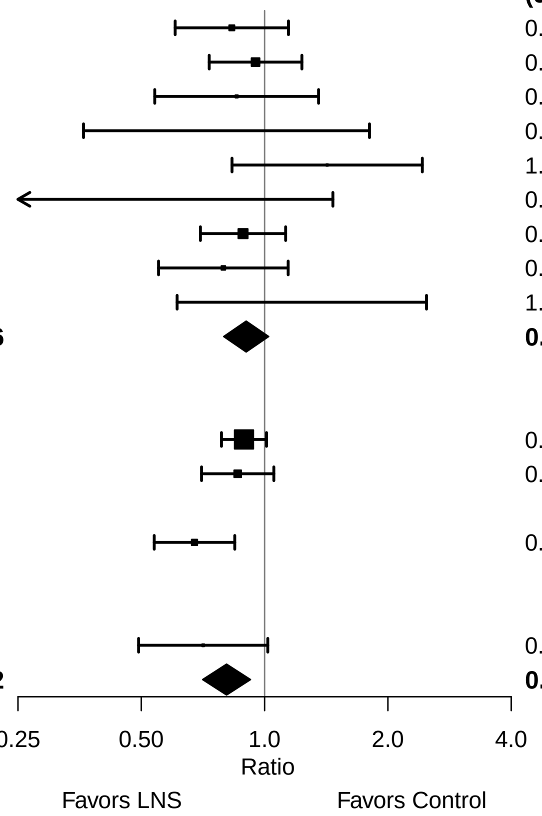

Supplemental figure 6K: Acute malnutrition prevalence difference

6K1: Stratified by Geographic region (insufficient comparisons)

Supplemental figure 6K: Acute malnutrition prevalence difference

6K2: Stratified by Stunting burden

Stunting burden
(p−diff = 0.872)

**Stunting burden − Less than 35%**				
**Country**	**Trial**	**N**	**N**	**PD (95% CI)**	**W**
Burkina Faso	PROMIS (38)	846	901	−0.02 (−0.05, 0.01)	0.16
Burkina Faso	PROMIS CS (38)	424	434	−0.01 (−0.06, 0.04)	0.06
Ghana	GHANA (39)				
Ghana	iLiNS−DYAD−G (40)	347	692	−0.01 (−0.05, 0.02)	0.13
Haiti	HAITI (41)				
Kenya	WASH−B (42)	1454	5117	−0.01 (−0.02, 0.00)	0.51
Malawi	iLiNS−DYAD−M (44)	220	444	−0.01 (−0.04, 0.02)	0.14
**I² = 0.00, Tau² = 0.00**		**3291**	**7588**	**−0.01 (−0.02, 0.00)**	
**Stunting burden − More than 35%**					
Bangladesh	JiVitA−4 (34)	2783	1216	−0.02 (−0.05, 0.00)	0.11
Bangladesh	RDNS (35)	1661	815	−0.02 (−0.05, 0.01)	0.08
Bangladesh	WASH−B (36)				
Burkina Faso	iLiNS−Zinc (37)	1951	664	−0.06 (−0.09, −0.03)	0.08
Madagascar	MAHAY (43)	1675	1655	0.00 (−0.02, 0.02)	0.14
Malawi	iLiNS−DOSE (45)	690	240	0.01 (−0.03, 0.04)	0.06
Mali	PROMIS (46)	499	502	−0.01 (−0.03, 0.01)	0.15
Mali	PROMIS CS (46)	944	959	−0.01 (−0.04, 0.01)	0.08
Zimbabwe	SHINE (HIV−) (47)	1867	1777	−0.01 (−0.02, 0.00)	0.24
Zimbabwe	SHINE (HIV+) (48)	336	327	0.01 (−0.02, 0.04)	0.07
**I² = 0.50, Tau² = 0.00**		**12406**	**8155**	**−0.01 (−0.03, 0.00)**	

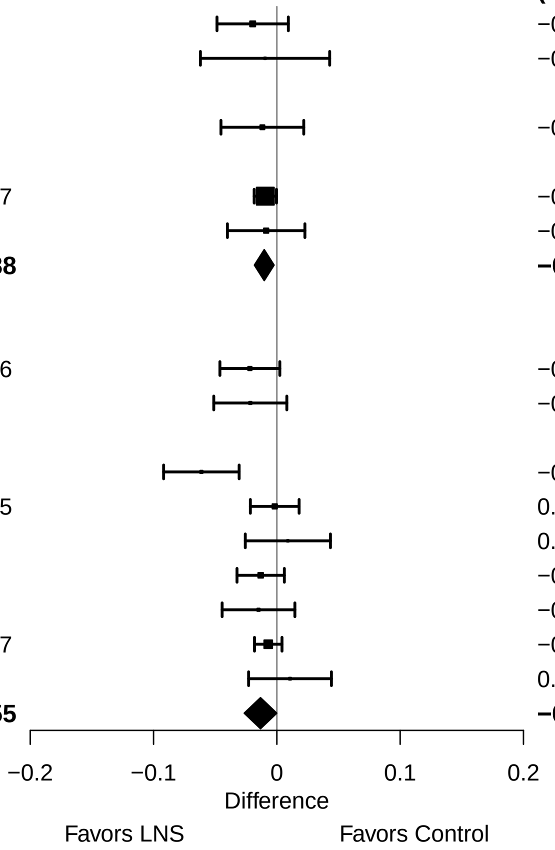

Supplemental figure 6K: Acute malnutrition prevalence difference

6K3: Stratified by Malaria prevalence

Malaria prevalence
(p−diff = 0.181)

Country	Trial	N	N	PD (95% CI)	W
**Malaria prevalence − Less than 10%**					
Bangladesh	JiVitA−4 (34)	2783	1216	−0.02 (−0.05, 0.00)	0.12
Bangladesh	RDNS (35)	1661	815	−0.02 (−0.05, 0.01)	0.09
Bangladesh	WASH−B (36)				
Haiti	HAITI (41)				
Kenya	WASH−B (42)	1454	5117	−0.01 (−0.02, 0.00)	0.30
Madagascar	MAHAY (43)	1675	1655	0.00 (−0.02, 0.02)	0.16
Zimbabwe	SHINE (HIV−) (47)	1867	1777	−0.01 (−0.02, 0.00)	0.26
Zimbabwe	SHINE (HIV+) (48)	336	327	0.01 (−0.02, 0.04)	0.07
**I² = 0.00, Tau² = 0.00**		**9776**	**10907**	**−0.01 (−0.01, 0.00)**	
**Malaria prevalence − At least 10%**					
Burkina Faso	iLiNS−Zinc (37)	1951	664	−0.06 (−0.09, −0.03)	0.12
Burkina Faso	PROMIS (38)	846	901	−0.02 (−0.05, 0.01)	0.13
Burkina Faso	PROMIS CS (38)	424	434	−0.01 (−0.06, 0.04)	0.05
Ghana	GHANA (39)				
Ghana	iLiNS−DYAD−G (40)	347	692	−0.01 (−0.05, 0.02)	0.11
Malawi	iLiNS−DYAD−M (44)	220	444	−0.01 (−0.04, 0.02)	0.12
Malawi	iLiNS−DOSE (45)	690	240	0.01 (−0.03, 0.04)	0.10
Mali	PROMIS (46)	499	502	−0.01 (−0.03, 0.01)	0.23
Mali	PROMIS CS (46)	944	959	−0.01 (−0.04, 0.01)	0.13
**I² = 0.35, Tau² = 0.00**		**5921**	**4836**	**−0.02 (−0.03, 0.00)**	

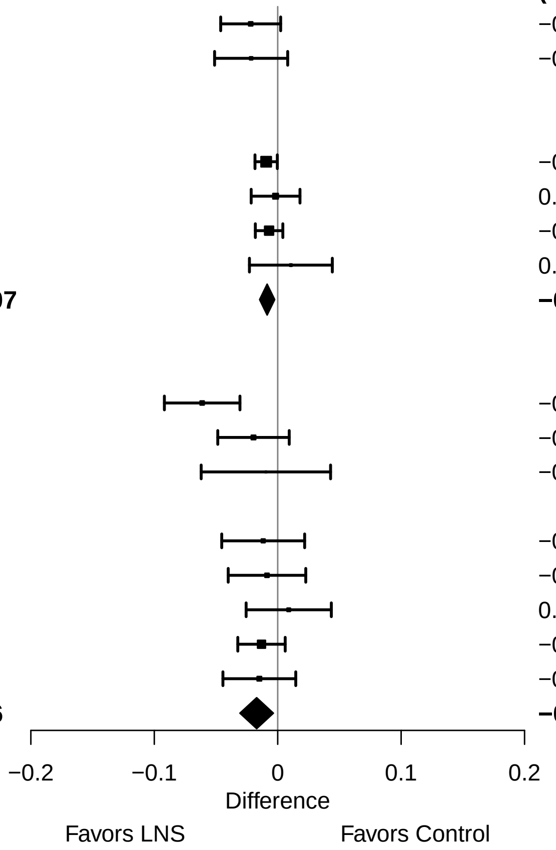

Supplemental figure 6K: Acute malnutrition prevalence difference

6K4: Stratified by Source water quality

Source water quality
(p−diff = 0.852)

Country	Trial	N	N	PD (95% CI)	W
**Source water quality − Improved**					
Bangladesh	JiVitA−4 (34)	2783	1216	−0.02 (−0.05, 0.00)	0.30
Bangladesh	RDNS (35)	1661	815	−0.02 (−0.05, 0.01)	0.20
Bangladesh	WASH−B (36)				
Ghana	GHANA (39)				
Ghana	iLiNS−DYAD−G (40)	347	692	−0.01 (−0.05, 0.02)	0.16
Haiti	HAITI (41)				
Malawi	iLiNS−DYAD−M (44)	220	444	−0.01 (−0.04, 0.02)	0.18
Malawi	iLiNS−DOSE (45)	690	240	0.01 (−0.03, 0.04)	0.15
**I² = 0.00, Tau² = 0.00**		**5701**	**3407**	**−0.01 (−0.03, 0.00)**	
**Source water quality − Unimproved**					
Burkina Faso	iLiNS−Zinc (37)	1951	664	−0.06 (−0.09, −0.03)	0.06
Burkina Faso	PROMIS (38)	846	901	−0.02 (−0.05, 0.01)	0.06
Burkina Faso	PROMIS CS (38)	424	434	−0.01 (−0.06, 0.04)	0.02
Kenya	WASH−B (42)	694	2244	−0.01 (−0.02, 0.01)	0.32
Madagascar	MAHAY (43)	1675	1655	0.00 (−0.02, 0.02)	0.13
Mali	PROMIS (46)	499	502	−0.01 (−0.03, 0.01)	0.14
Mali	PROMIS CS (46)	944	959	−0.01 (−0.04, 0.01)	0.06
Zimbabwe	SHINE (HIV−) (47)	886	871	−0.01 (−0.02, 0.01)	0.19
Zimbabwe	SHINE (HIV+) (48)	147	146	−0.01 (−0.06, 0.04)	0.02
**I² = 0.35, Tau² = 0.00**		**8066**	**8376**	**−0.01 (−0.02, 0.00)**	

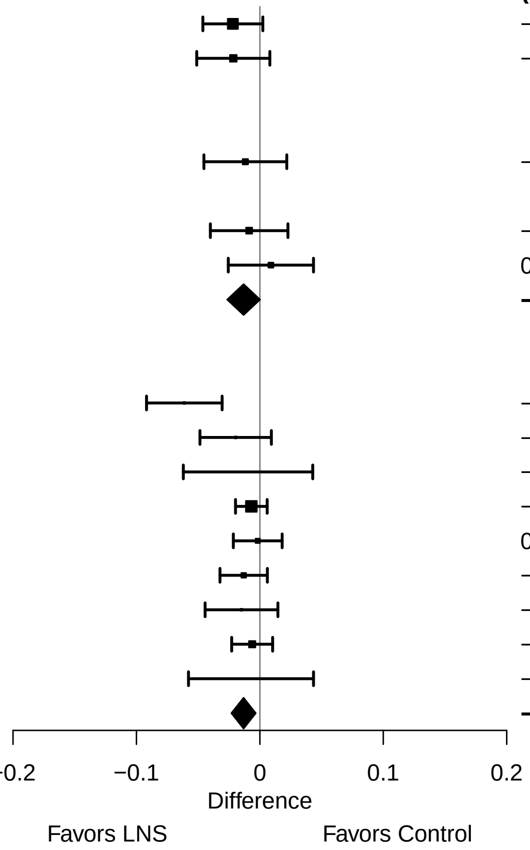

Supplemental figure 6K: Acute malnutrition prevalence difference

6K5: Stratified by Sanitation

Sanitation
(p−diff = 0.263)

**Country**	**Trial**	**N**	**N**	**PD (95% CI)**	**W**
**Sanitation − Improved**					
Bangladesh	JiVitA−4 (34)	2783	1216	−0.02 (−0.05, 0.00)	0.19
Bangladesh	RDNS (35)	1661	815	−0.02 (−0.05, 0.01)	0.12
Bangladesh	WASH−B (36)				
Burkina Faso	PROMIS (38)	846	901	−0.02 (−0.05, 0.01)	0.13
Burkina Faso	PROMIS CS (38)	424	434	−0.01 (−0.06, 0.04)	0.04
Ghana	GHANA (39)				
Ghana	iLiNS−DYAD−G (40)	347	692	−0.01 (−0.05, 0.02)	0.10
Haiti	HAITI (41)				
Mali	PROMIS (46)	499	502	−0.01 (−0.03, 0.01)	0.30
Mali	PROMIS CS (46)	944	959	−0.01 (−0.04, 0.01)	0.13
**I² = 0.00, Tau² = 0.00**		**7504**	**5519**	**−0.02 (−0.03, −0.01)**	
**Sanitation − Unimproved**					
Burkina Faso	iLiNS−Zinc (37)	1951	664	−0.06 (−0.09, −0.03)	0.07
Kenya	WASH−B (42)	694	2244	−0.01 (−0.02, 0.01)	0.39
Madagascar	MAHAY (43)	1675	1655	0.00 (−0.02, 0.02)	0.16
Malawi	iLiNS−DYAD−M (44)	220	444	−0.01 (−0.04, 0.02)	0.07
Malawi	iLiNS−DOSE (45)	690	240	0.01 (−0.03, 0.04)	0.05
Zimbabwe	SHINE (HIV−) (47)	886	871	−0.01 (−0.02, 0.01)	0.23
Zimbabwe	SHINE (HIV+) (48)	147	146	−0.01 (−0.06, 0.04)	0.03
**I² = 0.54, Tau² = 0.00**		**6263**	**6264**	**−0.01 (−0.03, 0.00)**	

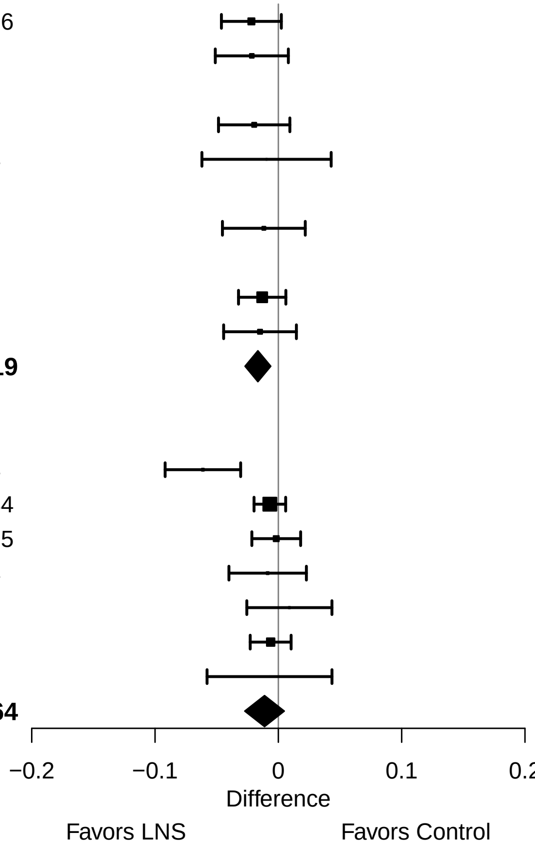

Supplemental figure 6K: Acute malnutrition prevalence difference

6K6: Stratified by Supplement duration

Supplement duration
(p−diff = 0.887)

Country	Trial	N	N	PD (95% CI)	W
**Supplement duration − 12m or less**					
Bangladesh	JiVitA−4 (34)	2783	1216	−0.02 (−0.05, 0.00)	0.11
Burkina Faso	iLiNS−Zinc (37)	1951	664	−0.06 (−0.09, −0.03)	0.08
Burkina Faso	PROMIS (38)	846	901	−0.02 (−0.05, 0.01)	0.09
Burkina Faso	PROMIS CS (38)	424	434	−0.01 (−0.06, 0.04)	0.03
Ghana	GHANA (39)				
Ghana	iLiNS−DYAD−G (40)	347	692	−0.01 (−0.05, 0.02)	0.07
Haiti	HAITI (41)				
Madagascar	MAHAY (43)	1675	1655	0.00 (−0.02, 0.02)	0.15
Malawi	iLiNS−DYAD−M (44)	220	444	−0.01 (−0.04, 0.02)	0.08
Malawi	iLiNS−DOSE (45)	690	240	0.01 (−0.03, 0.04)	0.07
Zimbabwe	SHINE (HIV−) (47)	1867	1777	−0.01 (−0.02, 0.00)	0.25
Zimbabwe	SHINE (HIV+) (48)	336	327	0.01 (−0.02, 0.04)	0.07
**I² = 0.43, Tau² = 0.00**		**11139**	**8350**	**−0.01 (−0.02, 0.00)**	
**Supplement duration − > 12m**					
Bangladesh	RDNS (35)	1661	815	−0.02 (−0.05, 0.01)	0.14
Bangladesh	WASH−B (36)				
Kenya	WASH−B (42)	1454	5117	−0.01 (−0.02, 0.00)	0.46
Mali	PROMIS (46)	499	502	−0.01 (−0.03, 0.01)	0.25
Mali	PROMIS CS (46)	944	959	−0.01 (−0.04, 0.01)	0.14
**I² = 0.00, Tau² = 0.00**		**4558**	**7393**	**−0.01 (−0.02, 0.00)**	

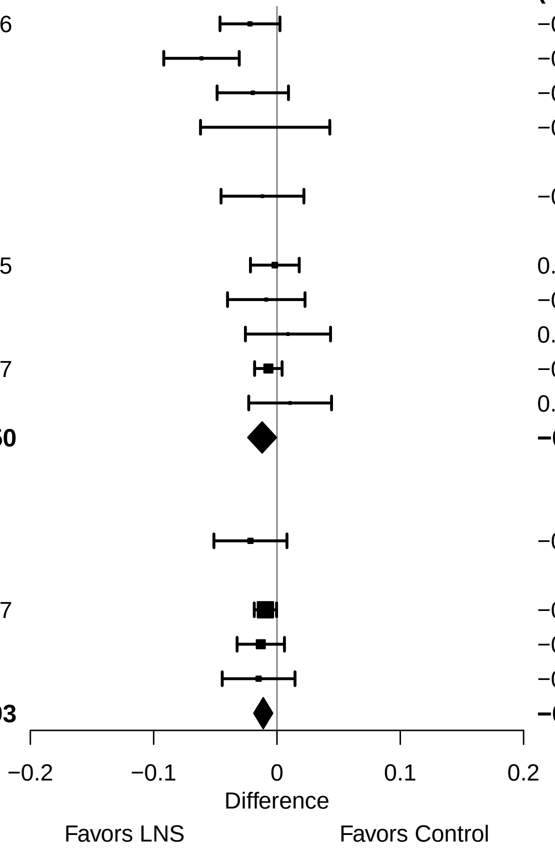

Supplemental figure 6K: Acute malnutrition prevalence difference

6K7: Stratified by Frequency of contact

Frequency of contact
(p−diff = 0.113)

Country	Trial	N	N	PD (95% CI)	W
**Frequency of contact − Monthly**					
Bangladesh	RDNS (35)	1661	815	−0.02 (−0.05, 0.01)	0.07
Burkina Faso	PROMIS (38)	846	901	−0.02 (−0.05, 0.01)	0.07
Burkina Faso	PROMIS CS (38)	424	434	−0.01 (−0.06, 0.04)	0.03
Haiti	HAITI (41)				
Kenya	WASH−B (42)	1454	5117	−0.01 (−0.02, 0.00)	0.23
Madagascar	MAHAY (43)	1675	1655	0.00 (−0.02, 0.02)	0.12
Mali	PROMIS (46)	499	502	−0.01 (−0.03, 0.01)	0.13
Mali	PROMIS CS (46)	944	959	−0.01 (−0.04, 0.01)	0.07
Zimbabwe	SHINE (HIV−) (47)	1867	1777	−0.01 (−0.02, 0.00)	0.21
Zimbabwe	SHINE (HIV+) (48)	336	327	0.01 (−0.02, 0.04)	0.06
**I² = 0.00, Tau² = 0.00**		**9706**	**12487**	**−0.01 (−0.01, 0.00)**	
**Frequency of contact − Weekly**					
Bangladesh	JiVitA−4 (34)	2783	1216	−0.02 (−0.05, 0.00)	0.28
Bangladesh	WASH−B (36)				
Burkina Faso	iLiNS−Zinc (37)	1951	664	−0.06 (−0.09, −0.03)	0.20
Ghana	GHANA (39)				
Ghana	iLiNS−DYAD−G (40)	347	692	−0.01 (−0.05, 0.02)	0.17
Malawi	iLiNS−DYAD−M (44)	220	444	−0.01 (−0.04, 0.02)	0.19
Malawi	iLiNS−DOSE (45)	690	240	0.01 (−0.03, 0.04)	0.16
**I² = 0.62, Tau² = 0.00**		**5991**	**3256**	**−0.02 (−0.04, 0.00)**	

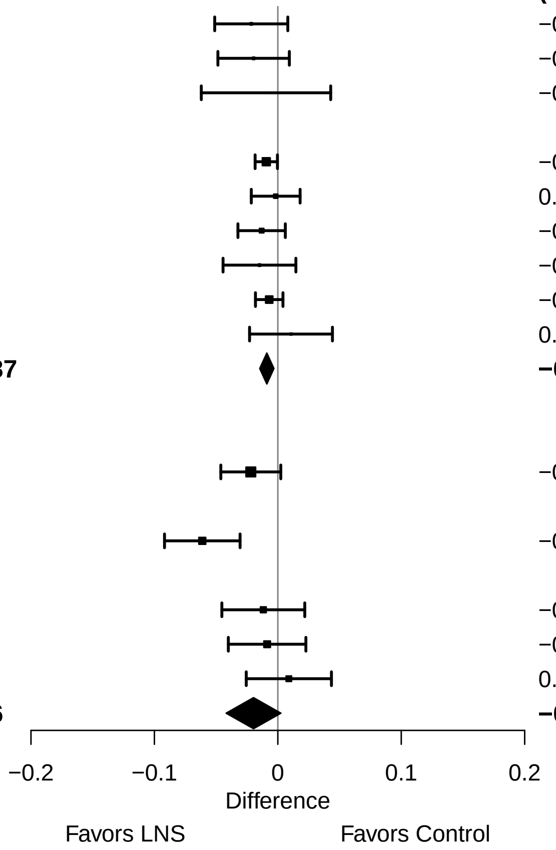

Supplemental figure 6K: Acute malnutrition prevalence difference

6K8: Stratified by Average SQ-LNS compliance

Average SQ−LNS compliance
(p−diff = 0.129)

Country	Trial	N	N	PD (95% CI)	W
**Average SQ−LNS compliance − Low**					
Burkina Faso	PROMIS (38)	846	901	−0.02 (−0.05, 0.01)	0.10
Burkina Faso	PROMIS CS (38)	424	434	−0.01 (−0.06, 0.04)	0.04
Ghana	iLiNS−DYAD−G (40)	347	692	−0.01 (−0.05, 0.02)	0.08
Malawi	iLiNS−DYAD−M (44)	220	444	−0.01 (−0.04, 0.02)	0.09
Malawi	iLiNS−DOSE (45)	690	240	0.01 (−0.03, 0.04)	0.08
Mali	PROMIS (46)	499	502	−0.01 (−0.03, 0.01)	0.17
Mali	PROMIS CS (46)	944	959	−0.01 (−0.04, 0.01)	0.10
Zimbabwe	SHINE (HIV−) (47)	1867	1777	−0.01 (−0.02, 0.00)	0.25
Zimbabwe	SHINE (HIV+) (48)	336	327	0.01 (−0.02, 0.04)	0.08
**I² = 0.00, Tau² = 0.00**		**6173**	**6276**	**−0.01 (−0.02, 0.00)**	
**Average SQ−LNS compliance − High**					
Bangladesh	JiVitA−4 (34)	2783	1216	−0.02 (−0.05, 0.00)	0.22
Bangladesh	RDNS (35)	1661	815	−0.02 (−0.05, 0.01)	0.17
Bangladesh	WASH−B (36)				
Burkina Faso	iLiNS−Zinc (37)	1951	664	−0.06 (−0.09, −0.03)	0.16
Ghana	GHANA (39)				
Haiti	HAITI (41)				
Kenya	WASH−B (42)	1454	5117	−0.01 (−0.02, 0.00)	0.45
**I² = 0.72, Tau² = 0.00**		**7849**	**7812**	**−0.03 (−0.05, 0.00)**	

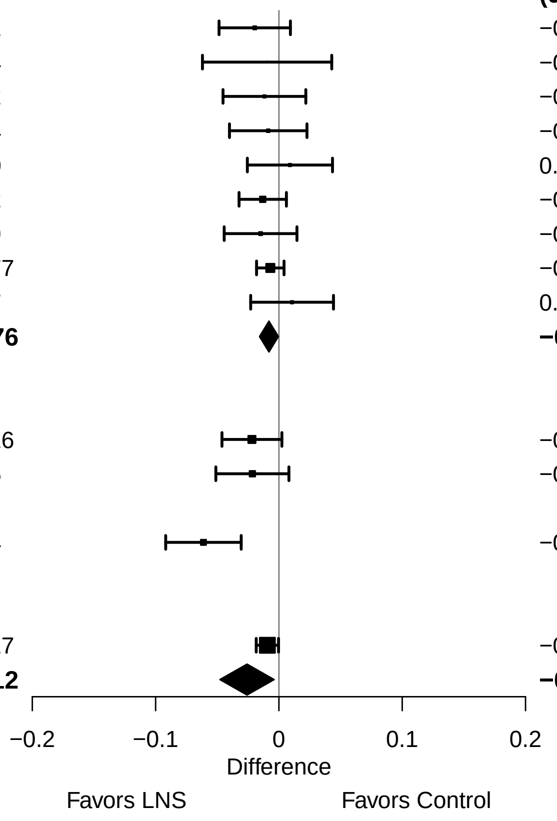

Supplemental figure 6L: Mean difference in WAZ

6L1: Stratified by Geographic region

Geographic region
(p−diff = 0.791)

Country	Trial	N	N	MD (95% CI)	W
**Geographic region − SEAR**					
Bangladesh	JiVitA−4 (34)	2814	1232	0.10 (0.07, 0.14)	0.38
Bangladesh	RDNS (35)	1663	815	0.09 (0.01, 0.18)	0.30
Bangladesh	WASH−B (36)	1163	3461	0.20 (0.13, 0.26)	0.33
**I² = 0.68, Tau² = 0.00**		**5640**	**5508**	**0.13 (0.07, 0.19)**	
**Geographic region − AFR**					
Burkina Faso	iLiNS−Zinc (37)	1960	666	0.28 (0.21, 0.35)	0.11
Burkina Faso	PROMIS (38)	857	911	0.15 (0.03, 0.27)	0.08
Burkina Faso	PROMIS CS (38)	430	436	−0.05 (−0.19, 0.09)	0.07
Ghana	GHANA (39)	98	96	0.28 (−0.05, 0.61)	0.02
Ghana	iLiNS−DYAD−G (40)	347	693	0.16 (0.03, 0.29)	0.07
Kenya	WASH−B (42)	1461	5165	0.13 (0.07, 0.20)	0.11
Madagascar	MAHAY (43)	1702	1685	0.01 (−0.10, 0.13)	0.08
Malawi	iLiNS−DYAD−M (44)	220	444	−0.07 (−0.23, 0.10)	0.06
Malawi	iLiNS−DOSE (45)	702	241	0.05 (−0.05, 0.16)	0.09
Mali	PROMIS (46)	500	502	0.16 (0.05, 0.28)	0.08
Mali	PROMIS CS (46)	946	960	0.23 (0.11, 0.36)	0.08
Zimbabwe	SHINE (HIV−) (47)	1869	1786	0.13 (0.07, 0.20)	0.11
Zimbabwe	SHINE (HIV+) (48)	336	328	0.01 (−0.16, 0.18)	0.06
**I² = 0.70, Tau² = 0.01**		**11428**	**13913**	**0.12 (0.06, 0.17)**	

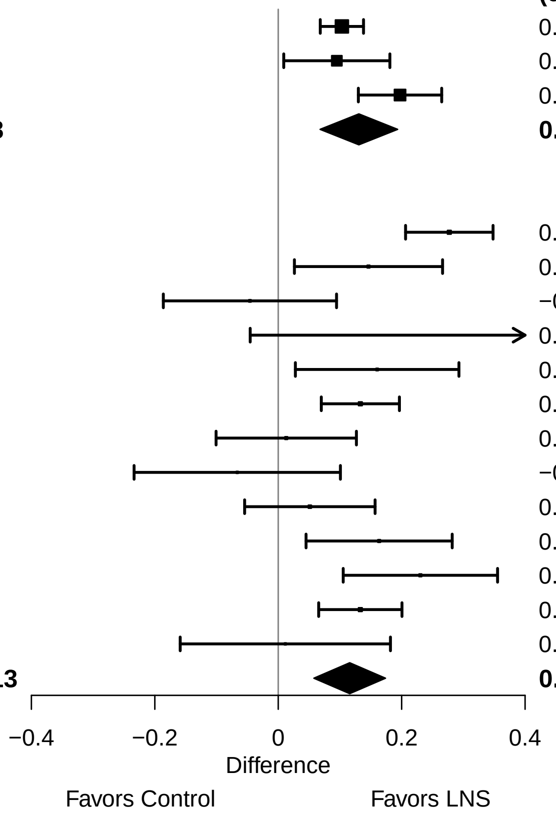

Supplemental figure 6L: Mean difference in WAZ

6L2: Stratified by Stunting burden

Stunting burden
(p−diff = 0.292)

Country	Trial	N	N	MD (95% CI)	W
**Stunting burden − Less than 35%**					
Burkina Faso	PROMIS (38)	857	911	0.15 (0.03, 0.27)	0.16
Burkina Faso	PROMIS CS (38)	430	436	−0.05 (−0.19, 0.09)	0.14
Ghana	GHANA (39)	98	96	0.28 (−0.05, 0.61)	0.04
Ghana	iLiNS−DYAD−G (40)	347	693	0.16 (0.03, 0.29)	0.15
Haiti	HAITI (41)	148	150	0.06 (−0.06, 0.19)	0.16
Kenya	WASH−B (42)	1461	5165	0.13 (0.07, 0.20)	0.23
Malawi	iLiNS−DYAD−M (44)	220	444	−0.07 (−0.23, 0.10)	0.12
**I² = 0.50, Tau² = 0.01**		**3561**	**7895**	**0.09 (0.01, 0.16)**	
**Stunting burden − More than 35%**					
Bangladesh	JiVitA−4 (34)	2814	1232	0.10 (0.07, 0.14)	0.14
Bangladesh	RDNS (35)	1663	815	0.09 (0.01, 0.18)	0.11
Bangladesh	WASH−B (36)	1163	3461	0.20 (0.13, 0.26)	0.12
Burkina Faso	iLiNS−Zinc (37)	1960	666	0.28 (0.21, 0.35)	0.12
Madagascar	MAHAY (43)	1702	1685	0.01 (−0.10, 0.13)	0.09
Malawi	iLiNS−DOSE (45)	702	241	0.05 (−0.05, 0.16)	0.09
Mali	PROMIS (46)	500	502	0.16 (0.05, 0.28)	0.09
Mali	PROMIS CS (46)	946	960	0.23 (0.11, 0.36)	0.08
Zimbabwe	SHINE (HIV−) (47)	1869	1786	0.13 (0.07, 0.20)	0.12
Zimbabwe	SHINE (HIV+) (48)	336	328	0.01 (−0.16, 0.18)	0.06
**I² = 0.74, Tau² = 0.01**		**13655**	**11676**	**0.13 (0.08, 0.19)**	

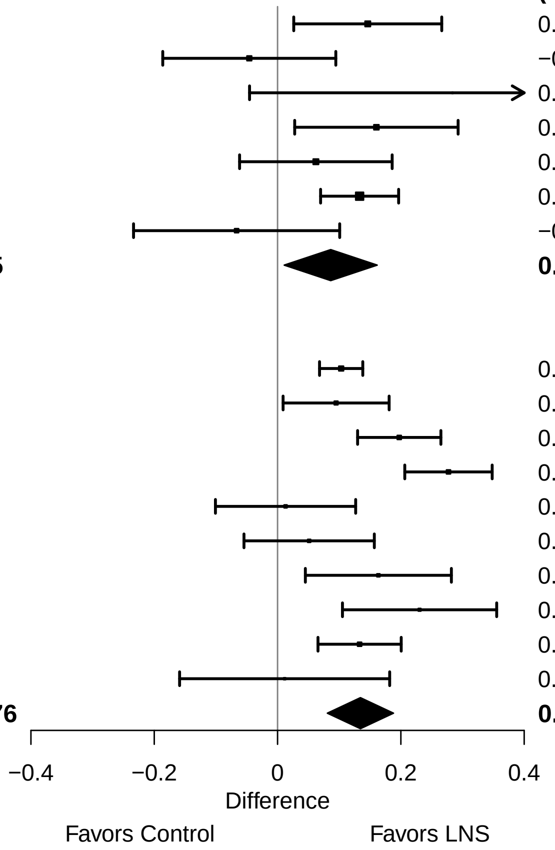

Supplemental figure 6L: Mean difference in WAZ

6L3: Stratified by Malaria prevalence

Malaria prevalence
(p−diff = 0.490)

Country	Trial	N	N	MD (95% CI)	W
**Malaria prevalence − Less than 10%**					
Bangladesh	JiVitA−4 (34)	2814	1232	0.10 (0.07, 0.14)	0.16
Bangladesh	RDNS (35)	1663	815	0.09 (0.01, 0.18)	0.13
Bangladesh	WASH−B (36)	1163	3461	0.20 (0.13, 0.26)	0.14
Haiti	HAITI (41)	148	150	0.06 (−0.06, 0.19)	0.10
Kenya	WASH−B (42)	1461	5165	0.13 (0.07, 0.20)	0.15
Madagascar	MAHAY (43)	1702	1685	0.01 (−0.10, 0.13)	0.11
Zimbabwe	SHINE (HIV−) (47)	1869	1786	0.13 (0.07, 0.20)	0.14
Zimbabwe	SHINE (HIV+) (48)	336	328	0.01 (−0.16, 0.18)	0.07
**I² = 0.42, Tau² = 0.00**		**11156**	**14622**	**0.11 (0.07, 0.15)**	
**Malaria prevalence − At least 10%**					
Burkina Faso	iLiNS−Zinc (37)	1960	666	0.28 (0.21, 0.35)	0.17
Burkina Faso	PROMIS (38)	857	911	0.15 (0.03, 0.27)	0.12
Burkina Faso	PROMIS CS (38)	430	436	−0.05 (−0.19, 0.09)	0.11
Ghana	GHANA (39)	98	96	0.28 (−0.05, 0.61)	0.03
Ghana	iLiNS−DYAD−G (40)	347	693	0.16 (0.03, 0.29)	0.11
Malawi	iLiNS−DYAD−M (44)	220	444	−0.07 (−0.23, 0.10)	0.09
Malawi	iLiNS−DOSE (45)	702	241	0.05 (−0.05, 0.16)	0.13
Mali	PROMIS (46)	500	502	0.16 (0.05, 0.28)	0.12
Mali	PROMIS CS (46)	946	960	0.23 (0.11, 0.36)	0.12
**I² = 0.75, Tau² = 0.01**		**6060**	**4949**	**0.13 (0.05, 0.21)**	

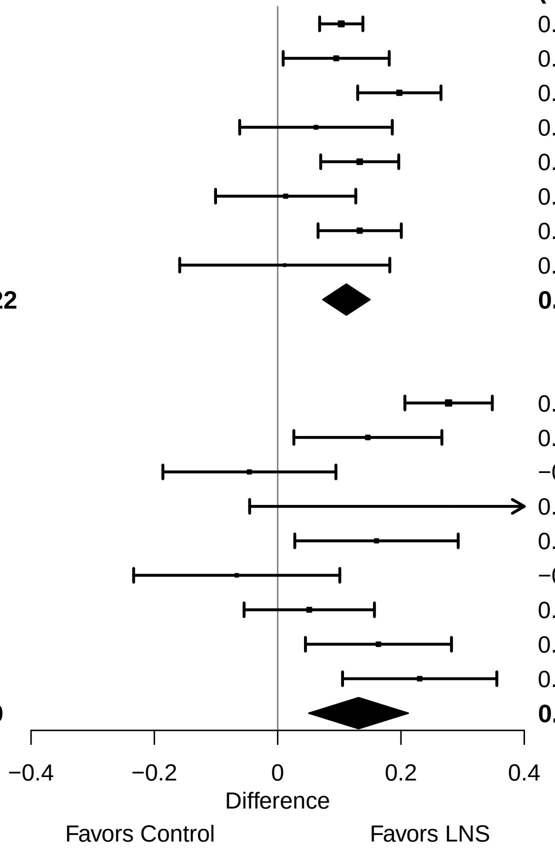

Supplemental figure 6L: Mean difference in WAZ

6L4: Stratified by Source water quality

Source water quality
(p−diff = 0.814)

**Source water quality − Improved**				**MD**	
**Country**	**Trial**	**N**	**N**	**(95% CI)**	**W**
Bangladesh	JiVitA−4 (34)	2814	1232	0.10 (0.07, 0.14)	0.20
Bangladesh	RDNS (35)	1663	815	0.09 (0.01, 0.18)	0.16
Bangladesh	WASH−B (36)	572	1119	0.25 (0.14, 0.37)	0.13
Ghana	GHANA (39)	98	96	0.28 (−0.05, 0.61)	0.04
Ghana	iLiNS−DYAD−G (40)	347	693	0.16 (0.03, 0.29)	0.12
Haiti	HAITI (41)	148	150	0.06 (−0.06, 0.19)	0.12
Malawi	iLiNS−DYAD−M (44)	220	444	−0.07 (−0.23, 0.10)	0.09
Malawi	iLiNS−DOSE (45)	702	241	0.05 (−0.05, 0.16)	0.14
**I² = 0.49, Tau² = 0.00**		**6564**	**4790**	**0.11 (0.04, 0.17)**	
**Source water quality − Unimproved**					
Burkina Faso	iLiNS−Zinc (37)	1960	666	0.28 (0.21, 0.35)	0.15
Burkina Faso	PROMIS (38)	857	911	0.15 (0.03, 0.27)	0.11
Burkina Faso	PROMIS CS (38)	430	436	−0.05 (−0.19, 0.09)	0.10
Kenya	WASH−B (42)	697	2261	0.09 (−0.01, 0.18)	0.13
Madagascar	MAHAY (43)	1702	1685	0.01 (−0.10, 0.13)	0.11
Mali	PROMIS (46)	500	502	0.16 (0.05, 0.28)	0.11
Mali	PROMIS CS (46)	946	960	0.23 (0.11, 0.36)	0.11
Zimbabwe	SHINE (HIV−) (47)	887	876	0.07 (−0.02, 0.16)	0.13
Zimbabwe	SHINE (HIV+) (48)	147	146	0.03 (−0.21, 0.26)	0.05
**I² = 0.75, Tau² = 0.01**		**8126**	**8443**	**0.12 (0.05, 0.19)**	

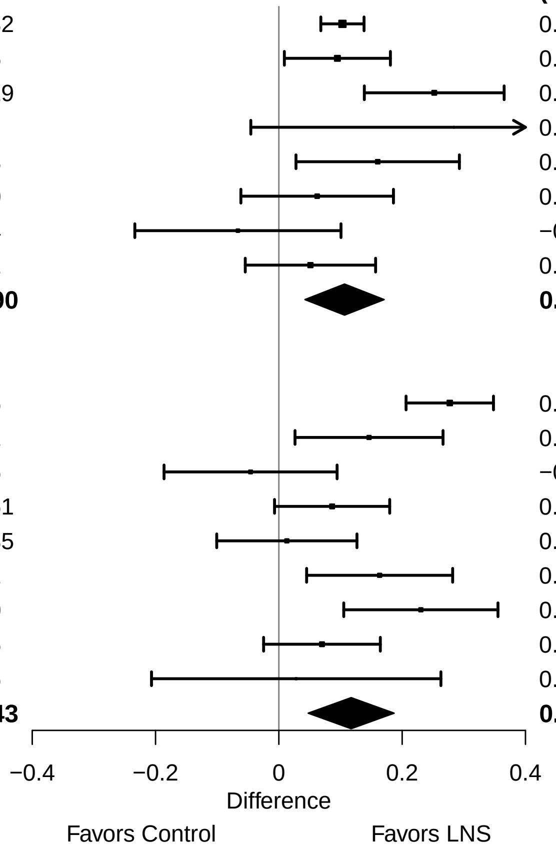

Supplemental figure 6L: Mean difference in WAZ

6L5: Stratified by Sanitation

Sanitation
(p−diff = 0.301)

**Sanitation − Improved**					
**Country**	**Trial**	**N**	**N**	**MD (95% CI)**	**W**
Bangladesh	JiVitA−4 (34)	2814	1232	0.10 (0.07, 0.14)	0.16
Bangladesh	RDNS (35)	1663	815	0.09 (0.01, 0.18)	0.13
Bangladesh	WASH−B (36)	572	1119	0.25 (0.14, 0.37)	0.11
Burkina Faso	PROMIS (38)	857	911	0.15 (0.03, 0.27)	0.10
Burkina Faso	PROMIS CS (38)	430	436	−0.05 (−0.19, 0.09)	0.09
Ghana	GHANA (39)	98	96	0.28 (−0.05, 0.61)	0.03
Ghana	iLiNS−DYAD−G (40)	347	693	0.16 (0.03, 0.29)	0.09
Haiti	HAITI (41)	148	150	0.06 (−0.06, 0.19)	0.10
Mali	PROMIS (46)	500	502	0.16 (0.05, 0.28)	0.10
Mali	PROMIS CS (46)	946	960	0.23 (0.11, 0.36)	0.10
**I² = 0.49, Tau² = 0.00**		**8375**	**6914**	**0.13 (0.08, 0.19)**	
**Sanitation − Unimproved**					
Burkina Faso	iLiNS−Zinc (37)	1960	666	0.28 (0.21, 0.35)	0.19
Kenya	WASH−B (42)	697	2261	0.09 (−0.01, 0.18)	0.17
Madagascar	MAHAY (43)	1702	1685	0.01 (−0.10, 0.13)	0.15
Malawi	iLiNS−DYAD−M (44)	220	444	−0.07 (−0.23, 0.10)	0.10
Malawi	iLiNS−DOSE (45)	702	241	0.05 (−0.05, 0.16)	0.16
Zimbabwe	SHINE (HIV−) (47)	887	876	0.07 (−0.02, 0.16)	0.17
Zimbabwe	SHINE (HIV+) (48)	147	146	0.03 (−0.21, 0.26)	0.07
**I² = 0.81, Tau² = 0.01**		**6315**	**6319**	**0.08 (0.00, 0.16)**	

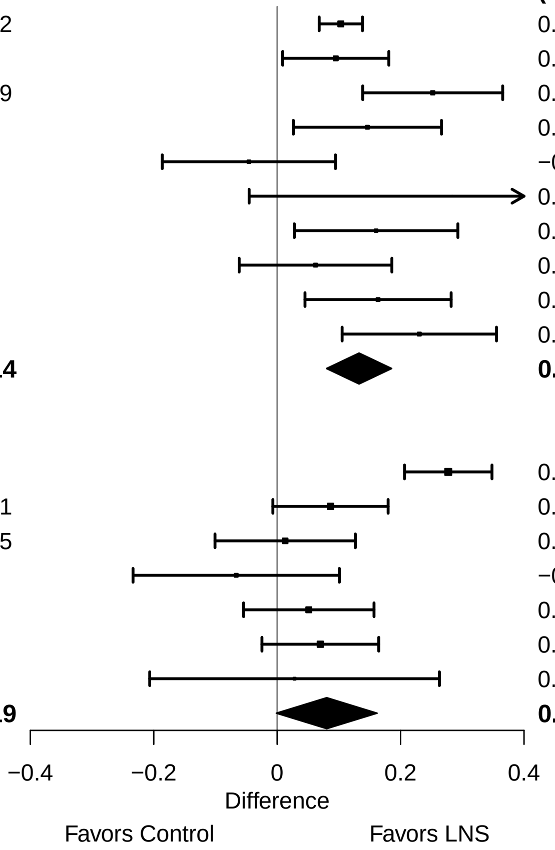

Supplemental figure 6L: Mean difference in WAZ

6L6: Stratified by Supplement duration

Supplement duration
(p−diff = 0.165)

Supplement duration − 12m or less

**Country**	**Trial**	**N**	**N**	**MD (95% CI)**	**W**
Bangladesh	JiVitA−4 (34)	2814	1232	0.10 (0.07, 0.14)	0.13
Burkina Faso	iLiNS−Zinc (37)	1960	666	0.28 (0.21, 0.35)	0.11
Burkina Faso	PROMIS (38)	857	911	0.15 (0.03, 0.27)	0.08
Burkina Faso	PROMIS CS (38)	430	436	−0.05 (−0.19, 0.09)	0.07
Ghana	GHANA (39)	98	96	0.28 (−0.05, 0.61)	0.02
Ghana	iLiNS−DYAD−G (40)	347	693	0.16 (0.03, 0.29)	0.08
Haiti	HAITI (41)	148	150	0.06 (−0.06, 0.19)	0.08
Madagascar	MAHAY (43)	1702	1685	0.01 (−0.10, 0.13)	0.09
Malawi	iLiNS−DYAD−M (44)	220	444	−0.07 (−0.23, 0.10)	0.06
Malawi	iLiNS−DOSE (45)	702	241	0.05 (−0.05, 0.16)	0.09
Zimbabwe	SHINE (HIV−) (47)	1869	1786	0.13 (0.07, 0.20)	0.12
Zimbabwe	SHINE (HIV+) (48)	336	328	0.01 (−0.16, 0.18)	0.06
**I^2 = 0.72, Tau^2 = 0.01**		**11483**	**8668**	**0.09 (0.04, 0.15)**	

Supplement duration − > 12m

**Country**	**Trial**	**N**	**N**	**MD (95% CI)**	**W**
Bangladesh	RDNS (35)	1663	815	0.09 (0.01, 0.18)	0.21
Bangladesh	WASH−B (36)	1163	3461	0.20 (0.13, 0.26)	0.23
Kenya	WASH−B (42)	1461	5165	0.13 (0.07, 0.20)	0.24
Mali	PROMIS (46)	500	502	0.16 (0.05, 0.28)	0.17
Mali	PROMIS CS (46)	946	960	0.23 (0.11, 0.36)	0.16
**I^2 = 0.24, Tau^2 = 0.00**		**5733**	**10903**	**0.16 (0.11, 0.20)**	

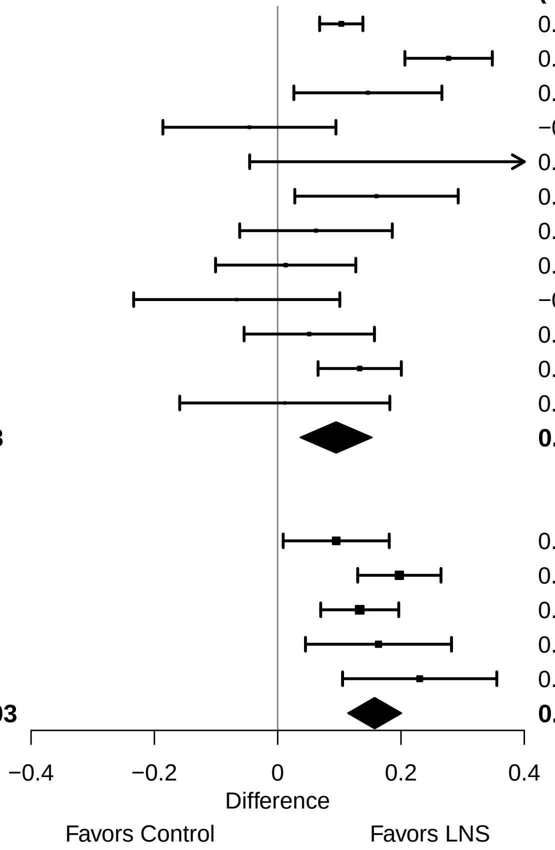

Supplemental figure 6L: Mean difference in WAZ

6L7: Stratified by Frequency of contact

Frequency of contact
(p−diff = 0.339)

Frequency of contact − Monthly

Country	Trial	N	N	MD (95% CI)	W
Bangladesh	RDNS (35)	1663	815	0.09 (0.01, 0.18)	0.12
Burkina Faso	PROMIS (38)	857	911	0.15 (0.03, 0.27)	0.09
Burkina Faso	PROMIS CS (38)	430	436	−0.05 (−0.19, 0.09)	0.08
Haiti	HAITI (41)	148	150	0.06 (−0.06, 0.19)	0.09
Kenya	WASH−B (42)	1461	5165	0.13 (0.07, 0.20)	0.13
Madagascar	MAHAY (43)	1702	1685	0.01 (−0.10, 0.13)	0.10
Mali	PROMIS (46)	500	502	0.16 (0.05, 0.28)	0.09
Mali	PROMIS CS (46)	946	960	0.23 (0.11, 0.36)	0.09
Zimbabwe	SHINE (HIV−) (47)	1869	1786	0.13 (0.07, 0.20)	0.13
Zimbabwe	SHINE (HIV+) (48)	336	328	0.01 (−0.16, 0.18)	0.07
**I² = 0.41, Tau² = 0.00**		**9912**	**12738**	**0.10 (0.06, 0.15)**	

Frequency of contact − Weekly

Country	Trial	N	N	MD (95% CI)	W
Bangladesh	JiVitA−4 (34)	2814	1232	0.10 (0.07, 0.14)	0.22
Bangladesh	WASH−B (36)	1163	3461	0.20 (0.13, 0.26)	0.19
Burkina Faso	iLiNS−Zinc (37)	1960	666	0.28 (0.21, 0.35)	0.19
Ghana	GHANA (39)	98	96	0.28 (−0.05, 0.61)	0.04
Ghana	iLiNS−DYAD−G (40)	347	693	0.16 (0.03, 0.29)	0.12
Malawi	iLiNS−DYAD−M (44)	220	444	−0.07 (−0.23, 0.10)	0.10
Malawi	iLiNS−DOSE (45)	702	241	0.05 (−0.05, 0.16)	0.15
**I² = 0.81, Tau² = 0.01**		**7304**	**6833**	**0.14 (0.06, 0.23)**	

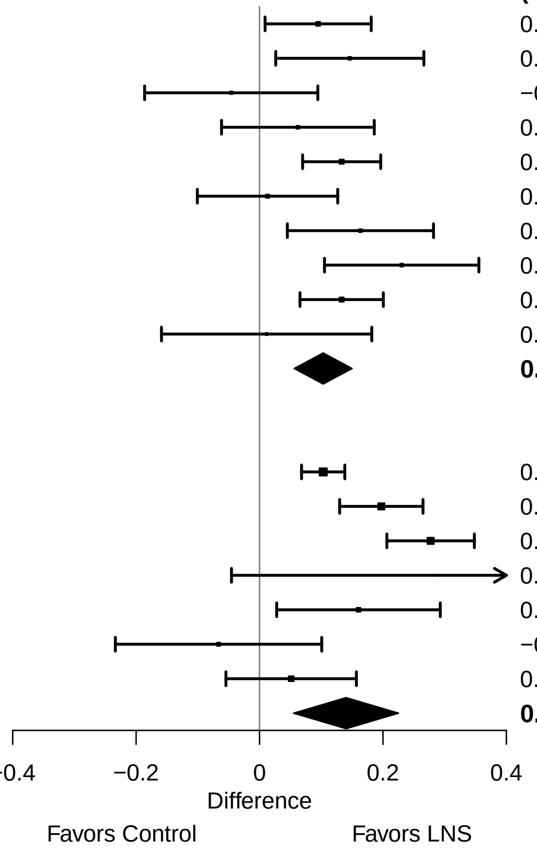

Supplemental figure 6L: Mean difference in WAZ

6L8: Stratified by Average SQ-LNS compliance

Average SQ−LNS compliance
(p−diff = 0.210)

Country	Trial	N	N	MD (95% CI)	W
**Average SQ−LNS compliance − Low**					
Burkina Faso	PROMIS (38)	857	911	0.15 (0.03, 0.27)	0.12
Burkina Faso	PROMIS CS (38)	430	436	−0.05 (−0.19, 0.09)	0.10
Ghana	iLiNS−DYAD−G (40)	347	693	0.16 (0.03, 0.29)	0.11
Malawi	iLiNS−DYAD−M (44)	220	444	−0.07 (−0.23, 0.10)	0.08
Malawi	iLiNS−DOSE (45)	702	241	0.05 (−0.05, 0.16)	0.13
Mali	PROMIS (46)	500	502	0.16 (0.05, 0.28)	0.12
Mali	PROMIS CS (46)	946	960	0.23 (0.11, 0.36)	0.11
Zimbabwe	SHINE (HIV−) (47)	1869	1786	0.13 (0.07, 0.20)	0.16
Zimbabwe	SHINE (HIV+) (48)	336	328	0.01 (−0.16, 0.18)	0.08
**I² = 0.53, Tau² = 0.01**		**6207**	**6301**	**0.10 (0.03, 0.16)**	
**Average SQ−LNS compliance − High**					
Bangladesh	JiVitA−4 (34)	2814	1232	0.10 (0.07, 0.14)	0.20
Bangladesh	RDNS (35)	1663	815	0.09 (0.01, 0.18)	0.15
Bangladesh	WASH−B (36)	1163	3461	0.20 (0.13, 0.26)	0.17
Burkina Faso	iLiNS−Zinc (37)	1960	666	0.28 (0.21, 0.35)	0.17
Ghana	GHANA (39)	98	96	0.28 (−0.05, 0.61)	0.03
Haiti	HAITI (41)	148	150	0.06 (−0.06, 0.19)	0.12
Kenya	WASH−B (42)	1461	5165	0.13 (0.07, 0.20)	0.17
**I² = 0.76, Tau² = 0.00**		**9307**	**11585**	**0.15 (0.09, 0.21)**	

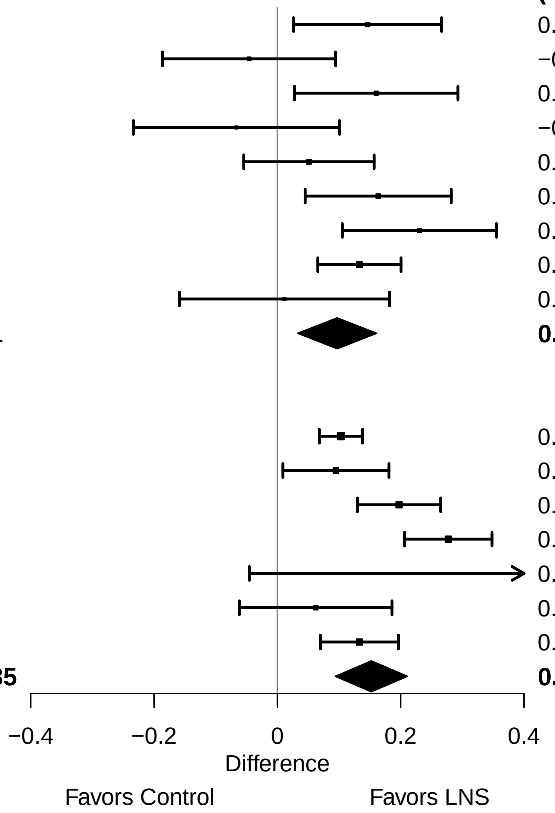

Supplemental figure 6M: Underweight prevalence ratio

6M1: Stratified by Geographic region

Geographic region
(p−diff = 0.909)

Country	Trial	N	N	PR (95% CI)	W
**Geographic region − SEAR**					
Bangladesh	JiVitA−4 (34)	2814	1232	0.91 (0.83, 1.01)	0.35
Bangladesh	RDNS (35)	1663	815	0.92 (0.83, 1.02)	0.34
Bangladesh	WASH−B (36)	1163	3461	0.79 (0.70, 0.89)	0.31
**I² = 0.57, Tau² = 0.00**		**5640**	**5508**	**0.87 (0.79, 0.96)**	
**Geographic region − AFR**					
Burkina Faso	iLiNS−Zinc (37)	1960	666	0.70 (0.59, 0.82)	0.13
Burkina Faso	PROMIS (38)	857	911	0.86 (0.71, 1.05)	0.10
Burkina Faso	PROMIS CS (38)	430	436	0.99 (0.77, 1.26)	0.08
Ghana	GHANA (39)	98	96	0.71 (0.30, 1.69)	0.01
Ghana	iLiNS−DYAD−G (40)	347	693	0.82 (0.57, 1.18)	0.04
Kenya	WASH−B (42)	1461	5165	0.76 (0.62, 0.93)	0.10
Madagascar	MAHAY (43)	1702	1685	1.04 (0.89, 1.21)	0.13
Malawi	iLiNS−DYAD−M (44)	220	444	1.13 (0.74, 1.72)	0.03
Malawi	iLiNS−DOSE (45)	702	241	1.01 (0.78, 1.31)	0.07
Mali	PROMIS (46)	500	502	0.85 (0.62, 1.18)	0.05
Mali	PROMIS CS (46)	946	960	0.88 (0.71, 1.10)	0.09
Zimbabwe	SHINE (HIV−) (47)	1869	1786	0.71 (0.57, 0.87)	0.10
Zimbabwe	SHINE (HIV+) (48)	336	328	1.01 (0.73, 1.40)	0.05
**I² = 0.47, Tau² = 0.01**		**11428**	**13913**	**0.86 (0.79, 0.94)**	

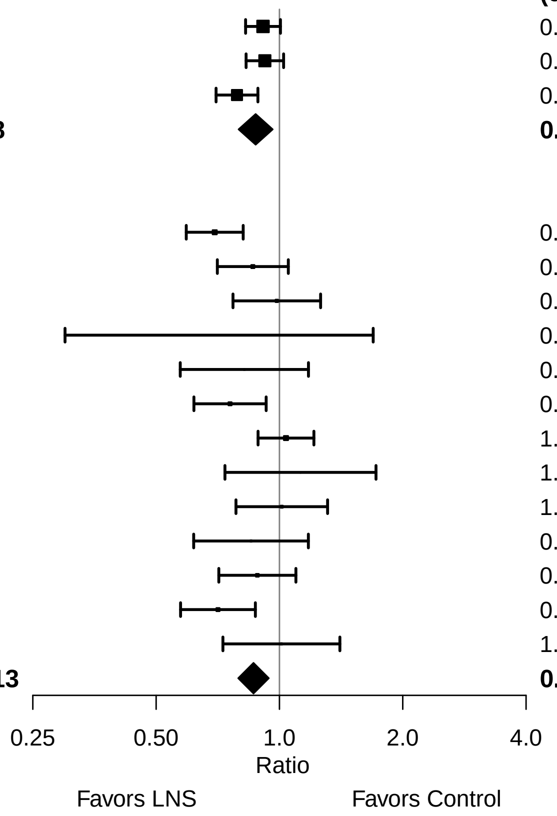

Supplemental figure 6M: Underweight prevalence ratio

6M2: Stratified by Stunting burden

Stunting burden
(p−diff = 0.979)

Country	Trial	N	N	PR (95% CI)	W
**Stunting burden − Less than 35%**					
Burkina Faso	PROMIS (38)	857	911	0.86 (0.71, 1.05)	0.28
Burkina Faso	PROMIS CS (38)	430	436	0.99 (0.77, 1.26)	0.21
Ghana	GHANA (39)	98	96	0.71 (0.30, 1.69)	0.02
Ghana	iLiNS−DYAD−G (40)	347	693	0.82 (0.57, 1.18)	0.11
Haiti	HAITI (41)	148	150	0.71 (0.28, 1.78)	0.02
Kenya	WASH−B (42)	1461	5165	0.76 (0.62, 0.93)	0.27
Malawi	iLiNS−DYAD−M (44)	220	444	1.13 (0.74, 1.72)	0.09
**I² = 0.00, Tau² = 0.00**		**3561**	**7895**	**0.86 (0.77, 0.96)**	
**Stunting burden − More than 35%**					
Bangladesh	JiVitA−4 (34)	2814	1232	0.91 (0.83, 1.01)	0.17
Bangladesh	RDNS (35)	1663	815	0.92 (0.83, 1.02)	0.16
Bangladesh	WASH−B (36)	1163	3461	0.79 (0.70, 0.89)	0.15
Burkina Faso	iLiNS−Zinc (37)	1960	666	0.70 (0.59, 0.82)	0.11
Madagascar	MAHAY (43)	1702	1685	1.04 (0.89, 1.21)	0.11
Malawi	iLiNS−DOSE (45)	702	241	1.01 (0.78, 1.31)	0.06
Mali	PROMIS (46)	500	502	0.85 (0.62, 1.18)	0.04
Mali	PROMIS CS (46)	946	960	0.88 (0.71, 1.10)	0.08
Zimbabwe	SHINE (HIV−) (47)	1869	1786	0.71 (0.57, 0.87)	0.08
Zimbabwe	SHINE (HIV+) (48)	336	328	1.01 (0.73, 1.40)	0.04
**I² = 0.61, Tau² = 0.01**		**13655**	**11676**	**0.87 (0.79, 0.95)**	

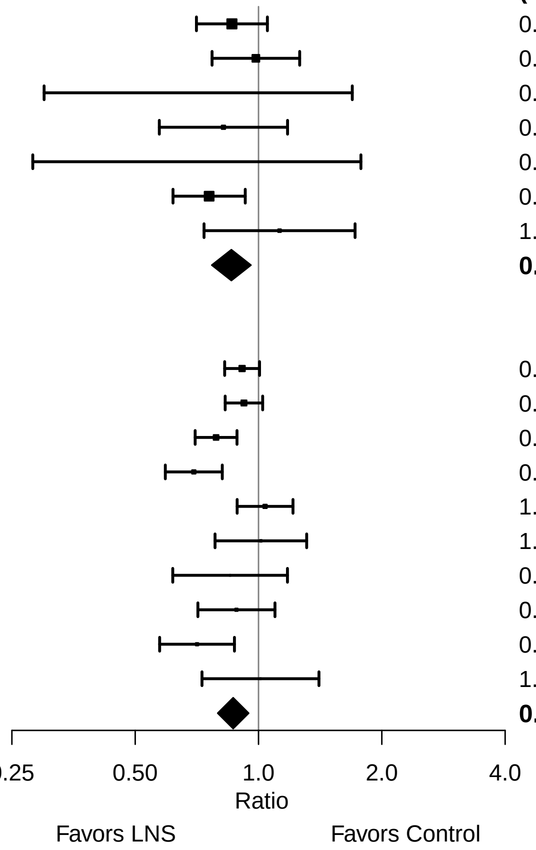

Supplemental figure 6M: Underweight prevalence ratio

6M3: Stratified by Malaria prevalence

Malaria prevalence
(p−diff = 0.998)

Country	Trial	N	N	PR (95% CI)	W
**Malaria prevalence − Less than 10%**					
Bangladesh	JiVitA−4 (34)	2814	1232	0.91 (0.83, 1.01)	0.21
Bangladesh	RDNS (35)	1663	815	0.92 (0.83, 1.02)	0.20
Bangladesh	WASH−B (36)	1163	3461	0.79 (0.70, 0.89)	0.18
Haiti	HAITI (41)	148	150	0.71 (0.28, 1.78)	0.01
Kenya	WASH−B (42)	1461	5165	0.76 (0.62, 0.93)	0.11
Madagascar	MAHAY (43)	1702	1685	1.04 (0.89, 1.21)	0.14
Zimbabwe	SHINE (HIV−) (47)	1869	1786	0.71 (0.57, 0.87)	0.10
Zimbabwe	SHINE (HIV+) (48)	336	328	1.01 (0.73, 1.40)	0.05
**I² = 0.56, Tau² = 0.01**		**11156**	**14622**	**0.87 (0.79, 0.95)**	
**Malaria prevalence − At least 10%**					
Burkina Faso	iLiNS−Zinc (37)	1960	666	0.70 (0.59, 0.82)	0.22
Burkina Faso	PROMIS (38)	857	911	0.86 (0.71, 1.05)	0.17
Burkina Faso	PROMIS CS (38)	430	436	0.99 (0.77, 1.26)	0.13
Ghana	GHANA (39)	98	96	0.71 (0.30, 1.69)	0.01
Ghana	iLiNS−DYAD−G (40)	347	693	0.82 (0.57, 1.18)	0.07
Malawi	iLiNS−DYAD−M (44)	220	444	1.13 (0.74, 1.72)	0.05
Malawi	iLiNS−DOSE (45)	702	241	1.01 (0.78, 1.31)	0.12
Mali	PROMIS (46)	500	502	0.85 (0.62, 1.18)	0.08
Mali	PROMIS CS (46)	946	960	0.88 (0.71, 1.10)	0.15
**I² = 0.29, Tau² = 0.00**		**6060**	**4949**	**0.86 (0.78, 0.95)**	

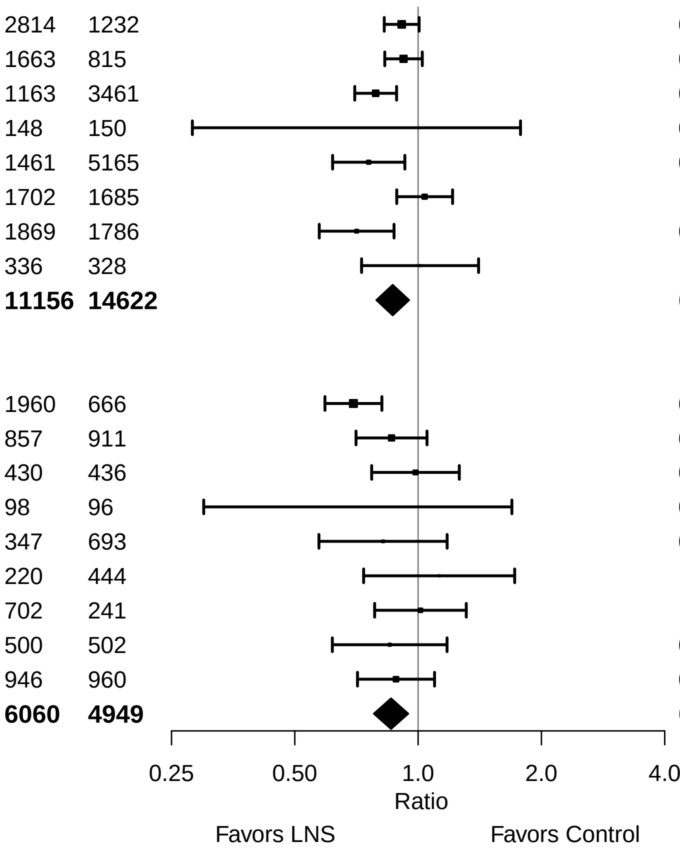

Supplemental figure 6M: Underweight prevalence ratio

6M4: Stratified by Source water quality

Source water quality
(p−diff = 0.601)

**Source water quality − Improved**				**PR**	
**Country**	**Trial**	**N**	**N**	**(95% CI)**	**W**
Bangladesh	JiVitA−4 (34)	2814	1232	0.91 (0.83, 1.01)	0.36
Bangladesh	RDNS (35)	1663	815	0.92 (0.83, 1.02)	0.33
Bangladesh	WASH−B (36)	572	1119	0.85 (0.73, 1.00)	0.17
Ghana	GHANA (39)	98	96	0.71 (0.30, 1.69)	0.01
Ghana	iLiNS−DYAD−G (40)	347	693	0.82 (0.57, 1.18)	0.04
Haiti	HAITI (41)	148	150	0.71 (0.28, 1.78)	0.01
Malawi	iLiNS−DYAD−M (44)	220	444	1.13 (0.74, 1.72)	0.03
Malawi	iLiNS−DOSE (45)	702	241	1.01 (0.78, 1.31)	0.07
**I² = 0.00, Tau² = 0.00**		**6564**	**4790**	**0.91 (0.86, 0.97)**	
**Source water quality − Unimproved**					
Burkina Faso	iLiNS−Zinc (37)	1960	666	0.70 (0.59, 0.82)	0.21
Burkina Faso	PROMIS (38)	857	911	0.86 (0.71, 1.05)	0.14
Burkina Faso	PROMIS CS (38)	430	436	0.99 (0.77, 1.26)	0.10
Kenya	WASH−B (42)	697	2261	0.89 (0.65, 1.23)	0.06
Madagascar	MAHAY (43)	1702	1685	1.04 (0.89, 1.21)	0.22
Mali	PROMIS (46)	500	502	0.85 (0.62, 1.18)	0.06
Mali	PROMIS CS (46)	946	960	0.88 (0.71, 1.10)	0.12
Zimbabwe	SHINE (HIV−) (47)	887	876	0.86 (0.64, 1.14)	0.07
Zimbabwe	SHINE (HIV+) (48)	147	146	1.03 (0.64, 1.69)	0.03
**I² = 0.43, Tau² = 0.01**		**8126**	**8443**	**0.88 (0.80, 0.96)**	

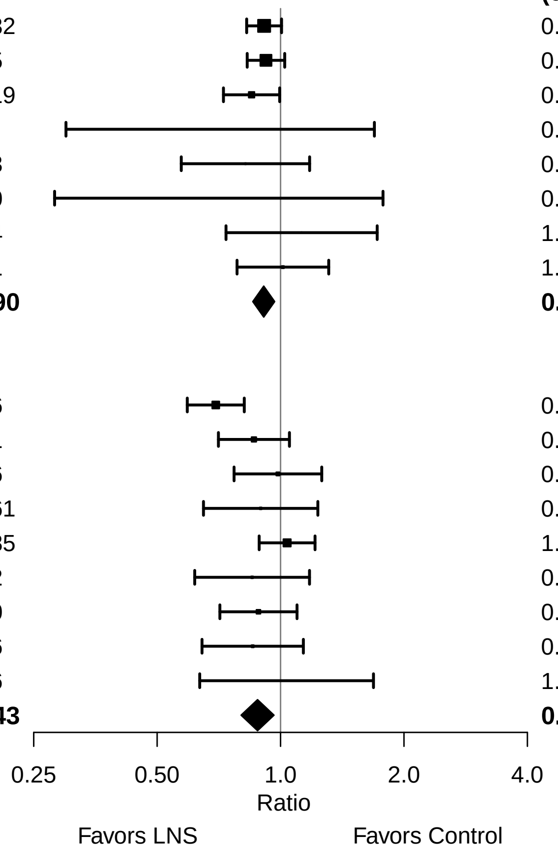

Supplemental figure 6M: Underweight prevalence ratio

6M5: Stratified by Sanitation

Sanitation
(p−diff = 0.873)

Country	Trial	N	N	PR (95% CI)	W
**Sanitation − Improved**					
Bangladesh	JiVitA−4 (34)	2814	1232	0.91 (0.83, 1.01)	0.30
Bangladesh	RDNS (35)	1663	815	0.92 (0.83, 1.02)	0.27
Bangladesh	WASH−B (36)	572	1119	0.85 (0.73, 1.00)	0.14
Burkina Faso	PROMIS (38)	857	911	0.86 (0.71, 1.05)	0.09
Burkina Faso	PROMIS CS (38)	430	436	0.99 (0.77, 1.26)	0.06
Ghana	GHANA (39)	98	96	0.71 (0.30, 1.69)	0.01
Ghana	iLiNS−DYAD−G (40)	347	693	0.82 (0.57, 1.18)	0.03
Haiti	HAITI (41)	148	150	0.71 (0.28, 1.78)	0.00
Mali	PROMIS (46)	500	502	0.85 (0.62, 1.18)	0.04
Mali	PROMIS CS (46)	946	960	0.88 (0.71, 1.10)	0.08
**I^2 = 0.00, Tau^2 = 0.00**		**8375**	**6914**	**0.90 (0.85, 0.95)**	
**Sanitation − Unimproved**					
Burkina Faso	iLiNS−Zinc (37)	1960	666	0.70 (0.59, 0.82)	0.30
Kenya	WASH−B (42)	697	2261	0.89 (0.65, 1.23)	0.08
Madagascar	MAHAY (43)	1702	1685	1.04 (0.89, 1.21)	0.31
Malawi	iLiNS−DYAD−M (44)	220	444	1.13 (0.74, 1.72)	0.05
Malawi	iLiNS−DOSE (45)	702	241	1.01 (0.78, 1.31)	0.12
Zimbabwe	SHINE (HIV−) (47)	887	876	0.86 (0.64, 1.14)	0.10
Zimbabwe	SHINE (HIV+) (48)	147	146	1.03 (0.64, 1.69)	0.04
**I^2 = 0.61, Tau^2 = 0.01**		**6315**	**6319**	**0.91 (0.80, 1.04)**	

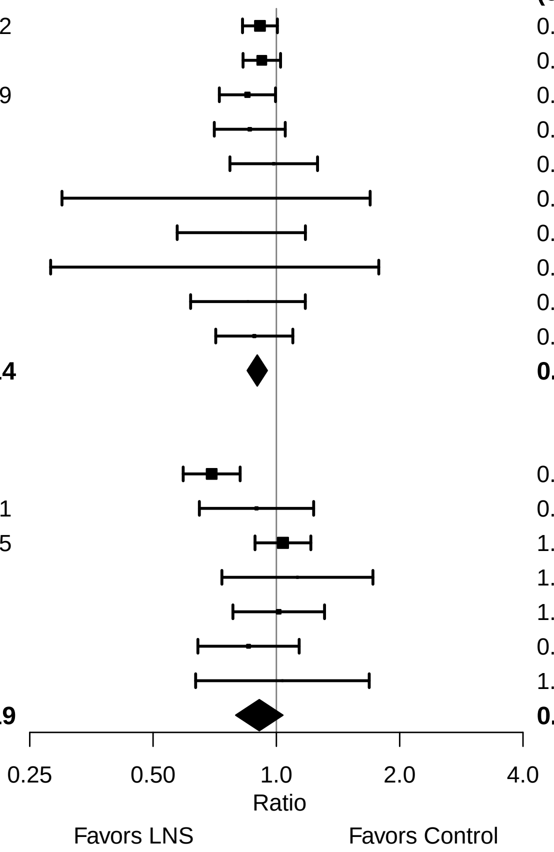

Supplemental figure 6M: Underweight prevalence ratio

6M6: Stratified by Supplement duration

Supplement duration
(p−diff = 0.519)

Country	Trial	N	N	PR (95% CI)	W
**Supplement duration − 12m or less**					
Bangladesh	JiVitA−4 (34)	2814	1232	0.91 (0.83, 1.01)	0.21
Burkina Faso	iLiNS−Zinc (37)	1960	666	0.70 (0.59, 0.82)	0.14
Burkina Faso	PROMIS (38)	857	911	0.86 (0.71, 1.05)	0.11
Burkina Faso	PROMIS CS (38)	430	436	0.99 (0.77, 1.26)	0.08
Ghana	GHANA (39)	98	96	0.71 (0.30, 1.69)	0.01
Ghana	iLiNS−DYAD−G (40)	347	693	0.82 (0.57, 1.18)	0.04
Haiti	HAITI (41)	148	150	0.71 (0.28, 1.78)	0.01
Madagascar	MAHAY (43)	1702	1685	1.04 (0.89, 1.21)	0.14
Malawi	iLiNS−DYAD−M (44)	220	444	1.13 (0.74, 1.72)	0.03
Malawi	iLiNS−DOSE (45)	702	241	1.01 (0.78, 1.31)	0.08
Zimbabwe	SHINE (HIV−) (47)	1869	1786	0.71 (0.57, 0.87)	0.10
Zimbabwe	SHINE (HIV+) (48)	336	328	1.01 (0.73, 1.40)	0.05
**I² = 0.49, Tau² = 0.01**		**11483**	**8668**	**0.88 (0.81, 0.97)**	
**Supplement duration − > 12m**					
Bangladesh	RDNS (35)	1663	815	0.92 (0.83, 1.02)	0.31
Bangladesh	WASH−B (36)	1163	3461	0.79 (0.70, 0.89)	0.29
Kenya	WASH−B (42)	1461	5165	0.76 (0.62, 0.93)	0.16
Mali	PROMIS (46)	500	502	0.85 (0.62, 1.18)	0.08
Mali	PROMIS CS (46)	946	960	0.88 (0.71, 1.10)	0.15
**I² = 0.23, Tau² = 0.00**		**5733**	**10903**	**0.85 (0.78, 0.91)**	

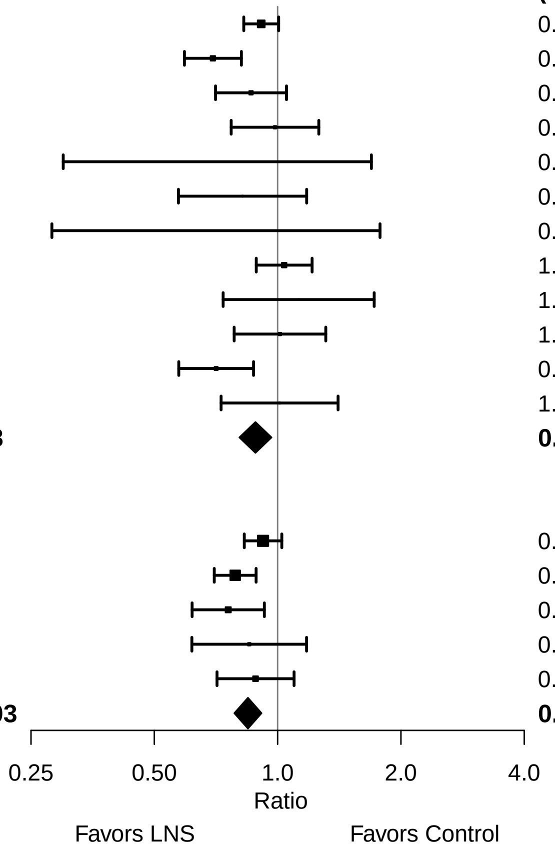

Supplemental figure 6M: Underweight prevalence ratio

6M7: Stratified by Frequency of contact

Frequency of contact
(p−diff = 0.516)

Country	Trial	N	N	PR (95% CI)	W
**Frequency of contact − Monthly**					
Bangladesh	RDNS (35)	1663	815	0.92 (0.83, 1.02)	0.21
Burkina Faso	PROMIS (38)	857	911	0.86 (0.71, 1.05)	0.11
Burkina Faso	PROMIS CS (38)	430	436	0.99 (0.77, 1.26)	0.09
Haiti	HAITI (41)	148	150	0.71 (0.28, 1.78)	0.01
Kenya	WASH−B (42)	1461	5165	0.76 (0.62, 0.93)	0.11
Madagascar	MAHAY (43)	1702	1685	1.04 (0.89, 1.21)	0.15
Mali	PROMIS (46)	500	502	0.85 (0.62, 1.18)	0.06
Mali	PROMIS CS (46)	946	960	0.88 (0.71, 1.10)	0.10
Zimbabwe	SHINE (HIV−) (47)	1869	1786	0.71 (0.57, 0.87)	0.11
Zimbabwe	SHINE (HIV+) (48)	336	328	1.01 (0.73, 1.40)	0.05
**I^2 = 0.29, Tau^2 = 0.00**		**9912**	**12738**	**0.89 (0.82, 0.96)**	
**Frequency of contact − Weekly**					
Bangladesh	JiVitA−4 (34)	2814	1232	0.91 (0.83, 1.01)	0.30
Bangladesh	WASH−B (36)	1163	3461	0.79 (0.70, 0.89)	0.27
Burkina Faso	iLiNS−Zinc (37)	1960	666	0.70 (0.59, 0.82)	0.20
Ghana	GHANA (39)	98	96	0.71 (0.30, 1.69)	0.01
Ghana	iLiNS−DYAD−G (40)	347	693	0.82 (0.57, 1.18)	0.06
Malawi	iLiNS−DYAD−M (44)	220	444	1.13 (0.74, 1.72)	0.05
Malawi	iLiNS−DOSE (45)	702	241	1.01 (0.78, 1.31)	0.11
**I^2 = 0.55, Tau^2 = 0.01**		**7304**	**6833**	**0.84 (0.75, 0.94)**	

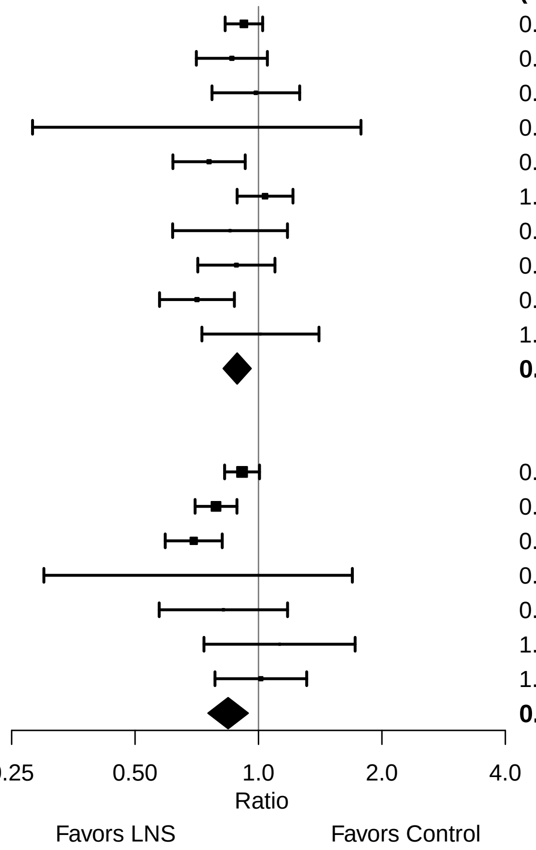

Supplemental figure 6M: Underweight prevalence ratio

6M8: Stratified by Average SQ-LNS compliance

Average SQ−LNS compliance
(p−diff = 0.272)

Average SQ−LNS compliance − Low

**Country**	**Trial**	**N**	**N**	**PR (95% CI)**	**W**
Burkina Faso	PROMIS (38)	857	911	0.86 (0.71, 1.05)	0.17
Burkina Faso	PROMIS CS (38)	430	436	0.99 (0.77, 1.26)	0.13
Ghana	iLiNS−DYAD−G (40)	347	693	0.82 (0.57, 1.18)	0.07
Malawi	iLiNS−DYAD−M (44)	220	444	1.13 (0.74, 1.72)	0.05
Malawi	iLiNS−DOSE (45)	702	241	1.01 (0.78, 1.31)	0.12
Mali	PROMIS (46)	500	502	0.85 (0.62, 1.18)	0.08
Mali	PROMIS CS (46)	946	960	0.88 (0.71, 1.10)	0.15
Zimbabwe	SHINE (HIV−) (47)	1869	1786	0.71 (0.57, 0.87)	0.16
Zimbabwe	SHINE (HIV+) (48)	336	328	1.01 (0.73, 1.40)	0.08
**I^2 = 0.03, Tau^2 = 0.00**		**6207**	**6301**	**0.88 (0.81, 0.97)**	

Average SQ−LNS compliance − High

Bangladesh	JiVitA−4 (34)	2814	1232	0.91 (0.83, 1.01)	0.26
Bangladesh	RDNS (35)	1663	815	0.92 (0.83, 1.02)	0.24
Bangladesh	WASH−B (36)	1163	3461	0.79 (0.70, 0.89)	0.22
Burkina Faso	iLiNS−Zinc (37)	1960	666	0.70 (0.59, 0.82)	0.15
Ghana	GHANA (39)	98	96	0.71 (0.30, 1.69)	0.01
Haiti	HAITI (41)	148	150	0.71 (0.28, 1.78)	0.01
Kenya	WASH−B (42)	1461	5165	0.76 (0.62, 0.93)	0.11
**I^2 = 0.55, Tau^2 = 0.01**		**9307**	**11585**	**0.82 (0.76, 0.90)**	

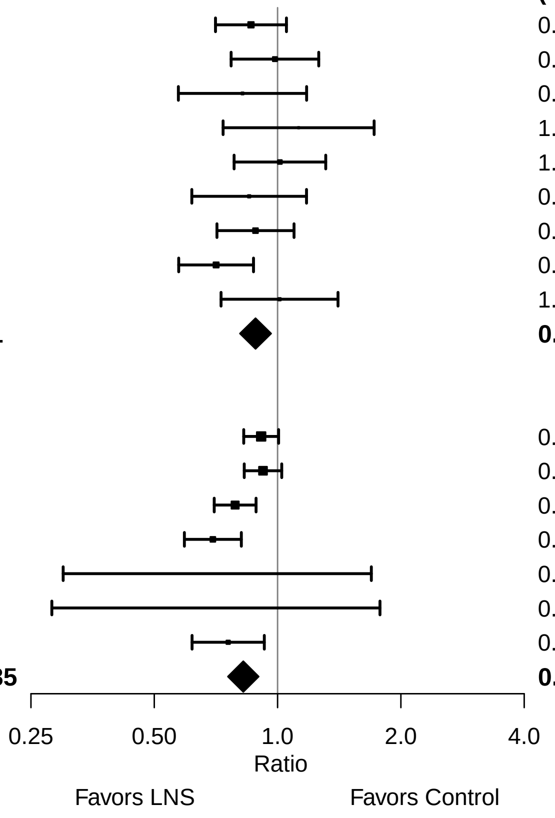

Supplemental figure 6N: Underweight prevalence difference

6N1: Stratified by Geographic region

Geographic region
(p−diff = 0.198)

Country	Trial	N	N	PD (95% CI)	W
**Geographic region − SEAR**					
Bangladesh	JiVitA−4 (34)	2814	1232	−0.04 (−0.07, −0.02)	0.38
Bangladesh	RDNS (35)	1663	815	−0.03 (−0.07, 0.01)	0.27
Bangladesh	WASH−B (36)	1163	3461	−0.07 (−0.10, −0.04)	0.35
**I² = 0.31, Tau² = 0.00**		**5640**	**5508**	**−0.05 (−0.07, −0.03)**	
**Geographic region − AFR**					
Burkina Faso	iLiNS−Zinc (37)	1960	666	−0.10 (−0.13, −0.07)	0.09
Burkina Faso	PROMIS (38)	857	911	−0.03 (−0.06, 0.01)	0.08
Burkina Faso	PROMIS CS (38)	430	436	0.00 (−0.06, 0.05)	0.05
Ghana	GHANA (39)	98	96	−0.03 (−0.12, 0.05)	0.03
Ghana	iLiNS−DYAD−G (40)	347	693	−0.02 (−0.06, 0.02)	0.07
Kenya	WASH−B (42)	1461	5165	−0.02 (−0.04, −0.01)	0.13
Madagascar	MAHAY (43)	1702	1685	0.01 (−0.02, 0.04)	0.10
Malawi	iLiNS−DYAD−M (44)	220	444	0.01 (−0.04, 0.07)	0.05
Malawi	iLiNS−DOSE (45)	702	241	−0.02 (−0.07, 0.03)	0.06
Mali	PROMIS (46)	500	502	−0.04 (−0.08, 0.00)	0.08
Mali	PROMIS CS (46)	946	960	−0.03 (−0.06, 0.01)	0.08
Zimbabwe	SHINE (HIV−) (47)	1869	1786	−0.03 (−0.05, −0.01)	0.12
Zimbabwe	SHINE (HIV+) (48)	336	328	0.00 (−0.06, 0.06)	0.05
**I² = 0.61, Tau² = 0.00**		**11428**	**13913**	**−0.03 (−0.04, −0.01)**	

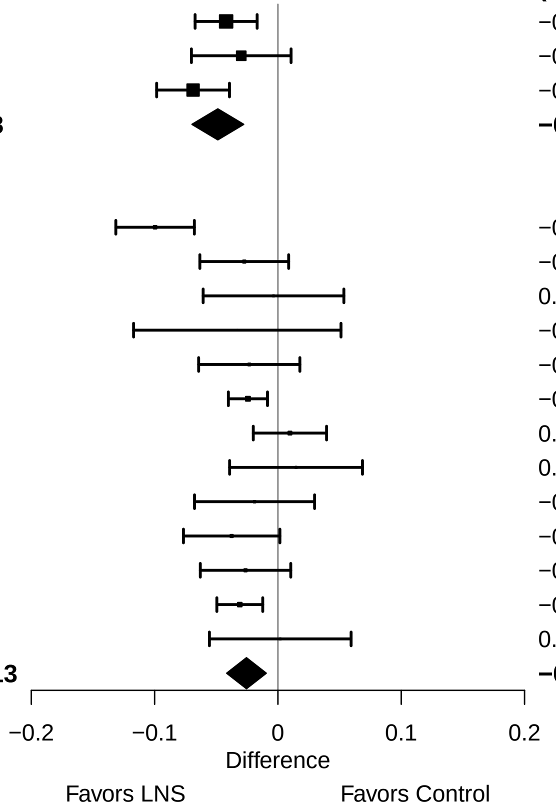

Supplemental figure 6N: Underweight prevalence difference

6N2: Stratified by Stunting burden

Stunting burden
(p−diff = 0.149)

Country	Trial	N	N	PD (95% CI)	W
**Stunting burden − Less than 35%**					
Burkina Faso	PROMIS (38)	857	911	−0.03 (−0.06, 0.01)	0.17
Burkina Faso	PROMIS CS (38)	430	436	0.00 (−0.06, 0.05)	0.10
Ghana	GHANA (39)	98	96	−0.03 (−0.12, 0.05)	0.06
Ghana	iLiNS−DYAD−G (40)	347	693	−0.02 (−0.06, 0.02)	0.15
Haiti	HAITI (41)	148	150	0.00 (−0.05, 0.04)	0.14
Kenya	WASH−B (42)	1461	5165	−0.02 (−0.04, −0.01)	0.26
Malawi	iLiNS−DYAD−M (44)	220	444	0.01 (−0.04, 0.07)	0.11
**I² = 0.00, Tau² = 0.00**		**3561**	**7895**	**−0.02 (−0.03, −0.01)**	
**Stunting burden − More than 35%**					
Bangladesh	JiVitA−4 (34)	2814	1232	−0.04 (−0.07, −0.02)	0.12
Bangladesh	RDNS (35)	1663	815	−0.03 (−0.07, 0.01)	0.09
Bangladesh	WASH−B (36)	1163	3461	−0.07 (−0.10, −0.04)	0.11
Burkina Faso	iLiNS−Zinc (37)	1960	666	−0.10 (−0.13, −0.07)	0.11
Madagascar	MAHAY (43)	1702	1685	0.01 (−0.02, 0.04)	0.11
Malawi	iLiNS−DOSE (45)	702	241	−0.02 (−0.07, 0.03)	0.07
Mali	PROMIS (46)	500	502	−0.04 (−0.08, 0.00)	0.09
Mali	PROMIS CS (46)	946	960	−0.03 (−0.06, 0.01)	0.10
Zimbabwe	SHINE (HIV−) (47)	1869	1786	−0.03 (−0.05, −0.01)	0.14
Zimbabwe	SHINE (HIV+) (48)	336	328	0.00 (−0.06, 0.06)	0.06
**I² = 0.72, Tau² = 0.00**		**13655**	**11676**	**−0.04 (−0.06, −0.02)**	

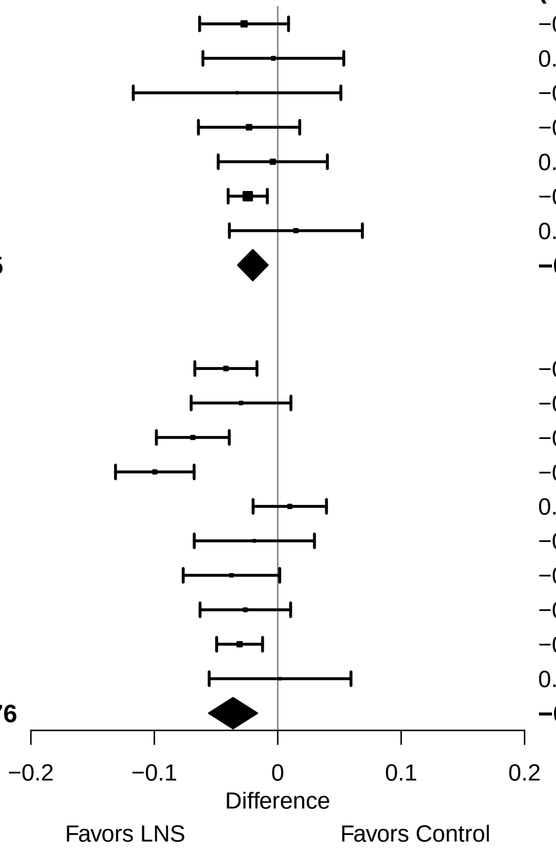

Supplemental figure 6N: Underweight prevalence difference

6N3: Stratified by Malaria prevalence

Malaria prevalence
(p−diff = 0.671)

Country	Trial	N	N	PD (95% CI)	W
**Malaria prevalence − Less than 10%**					
Bangladesh	JiVitA−4 (34)	2814	1232	−0.04 (−0.07, −0.02)	0.14
Bangladesh	RDNS (35)	1663	815	−0.03 (−0.07, 0.01)	0.10
Bangladesh	WASH−B (36)	1163	3461	−0.07 (−0.10, −0.04)	0.13
Haiti	HAITI (41)	148	150	0.00 (−0.05, 0.04)	0.09
Kenya	WASH−B (42)	1461	5165	−0.02 (−0.04, −0.01)	0.17
Madagascar	MAHAY (43)	1702	1685	0.01 (−0.02, 0.04)	0.13
Zimbabwe	SHINE (HIV−) (47)	1869	1786	−0.03 (−0.05, −0.01)	0.16
Zimbabwe	SHINE (HIV+) (48)	336	328	0.00 (−0.06, 0.06)	0.07
**I² = 0.59, Tau² = 0.00**		**11156**	**14622**	**−0.03 (−0.04, −0.01)**	
**Malaria prevalence − At least 10%**					
Burkina Faso	iLiNS−Zinc (37)	1960	666	−0.10 (−0.13, −0.07)	0.15
Burkina Faso	PROMIS (38)	857	911	−0.03 (−0.06, 0.01)	0.14
Burkina Faso	PROMIS CS (38)	430	436	0.00 (−0.06, 0.05)	0.08
Ghana	GHANA (39)	98	96	−0.03 (−0.12, 0.05)	0.05
Ghana	iLiNS−DYAD−G (40)	347	693	−0.02 (−0.06, 0.02)	0.12
Malawi	iLiNS−DYAD−M (44)	220	444	0.01 (−0.04, 0.07)	0.09
Malawi	iLiNS−DOSE (45)	702	241	−0.02 (−0.07, 0.03)	0.10
Mali	PROMIS (46)	500	502	−0.04 (−0.08, 0.00)	0.13
Mali	PROMIS CS (46)	946	960	−0.03 (−0.06, 0.01)	0.14
**I² = 0.62, Tau² = 0.00**		**6060**	**4949**	**−0.03 (−0.05, −0.01)**	

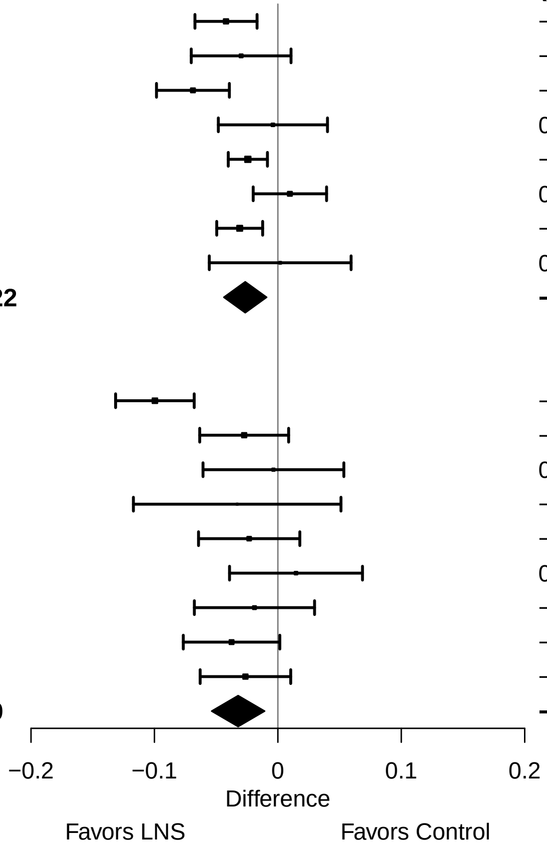

Supplemental figure 6N: Underweight prevalence difference

6N4: Stratified by Source water quality

Source water quality
(p−diff = 0.966)

Country	Trial	N	N	PD (95% CI)	W
**Source water quality − Improved**					
Bangladesh	JiVitA−4 (34)	2814	1232	−0.04 (−0.07, −0.02)	0.22
Bangladesh	RDNS (35)	1663	815	−0.03 (−0.07, 0.01)	0.14
Bangladesh	WASH−B (36)	572	1119	−0.05 (−0.09, 0.00)	0.12
Ghana	GHANA (39)	98	96	−0.03 (−0.12, 0.05)	0.05
Ghana	iLiNS−DYAD−G (40)	347	693	−0.02 (−0.06, 0.02)	0.14
Haiti	HAITI (41)	148	150	0.00 (−0.05, 0.04)	0.13
Malawi	iLiNS−DYAD−M (44)	220	444	0.01 (−0.04, 0.07)	0.10
Malawi	iLiNS−DOSE (45)	702	241	−0.02 (−0.07, 0.03)	0.11
**I² = 0.00, Tau² = 0.00**		**6564**	**4790**	**−0.03 (−0.04, −0.01)**	
**Source water quality − Unimproved**					
Burkina Faso	iLiNS−Zinc (37)	1960	666	−0.10 (−0.13, −0.07)	0.13
Burkina Faso	PROMIS (38)	857	911	−0.03 (−0.06, 0.01)	0.11
Burkina Faso	PROMIS CS (38)	430	436	0.00 (−0.06, 0.05)	0.06
Kenya	WASH−B (42)	697	2261	−0.01 (−0.03, 0.01)	0.16
Madagascar	MAHAY (43)	1702	1685	0.01 (−0.02, 0.04)	0.14
Mali	PROMIS (46)	500	502	−0.04 (−0.08, 0.00)	0.10
Mali	PROMIS CS (46)	946	960	−0.03 (−0.06, 0.01)	0.11
Zimbabwe	SHINE (HIV−) (47)	887	876	−0.01 (−0.04, 0.01)	0.15
Zimbabwe	SHINE (HIV+) (48)	147	146	0.01 (−0.08, 0.09)	0.03
**I² = 0.73, Tau² = 0.00**		**8126**	**8443**	**−0.02 (−0.05, 0.00)**	

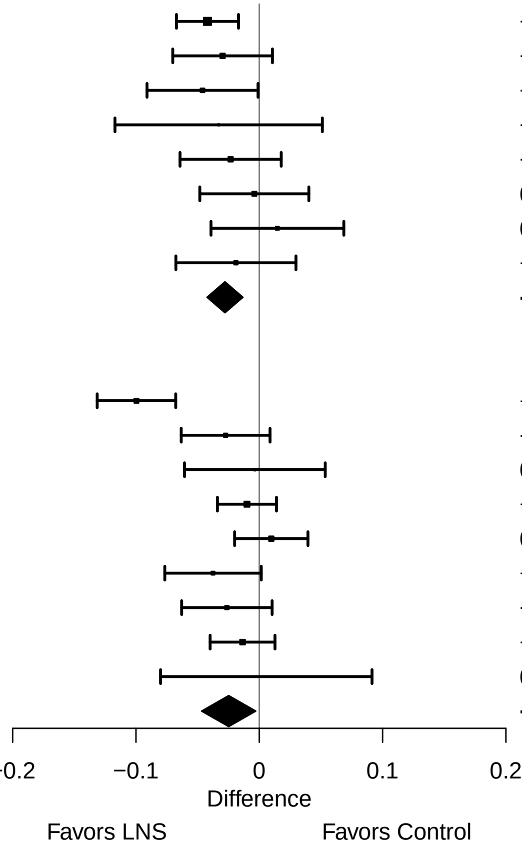

Supplemental figure 6N: Underweight prevalence difference

6N5: Stratified by Sanitation

Sanitation
(p−diff = 0.573)

Country	Trial	N	N	PD (95% CI)	W
**Sanitation − Improved**					
Bangladesh	JiVitA−4 (34)	2814	1232	−0.04 (−0.07, −0.02)	0.16
Bangladesh	RDNS (35)	1663	815	−0.03 (−0.07, 0.01)	0.10
Bangladesh	WASH−B (36)	572	1119	−0.05 (−0.09, 0.00)	0.09
Burkina Faso	PROMIS (38)	857	911	−0.03 (−0.06, 0.01)	0.12
Burkina Faso	PROMIS CS (38)	430	436	0.00 (−0.06, 0.05)	0.07
Ghana	GHANA (39)	98	96	−0.03 (−0.12, 0.05)	0.04
Ghana	iLiNS−DYAD−G (40)	347	693	−0.02 (−0.06, 0.02)	0.10
Haiti	HAITI (41)	148	150	0.00 (−0.05, 0.04)	0.09
Mali	PROMIS (46)	500	502	−0.04 (−0.08, 0.00)	0.11
Mali	PROMIS CS (46)	946	960	−0.03 (−0.06, 0.01)	0.12
**I² = 0.00, Tau² = 0.00**		**8375**	**6914**	**−0.03 (−0.04, −0.02)**	
**Sanitation − Unimproved**					
Burkina Faso	iLiNS−Zinc (37)	1960	666	−0.10 (−0.13, −0.07)	0.17
Kenya	WASH−B (42)	697	2261	−0.01 (−0.03, 0.01)	0.21
Madagascar	MAHAY (43)	1702	1685	0.01 (−0.02, 0.04)	0.18
Malawi	iLiNS−DYAD−M (44)	220	444	0.01 (−0.04, 0.07)	0.09
Malawi	iLiNS−DOSE (45)	702	241	−0.02 (−0.07, 0.03)	0.11
Zimbabwe	SHINE (HIV−) (47)	887	876	−0.01 (−0.04, 0.01)	0.20
Zimbabwe	SHINE (HIV+) (48)	147	146	0.01 (−0.08, 0.09)	0.04
**I² = 0.80, Tau² = 0.00**		**6315**	**6319**	**−0.02 (−0.05, 0.01)**	

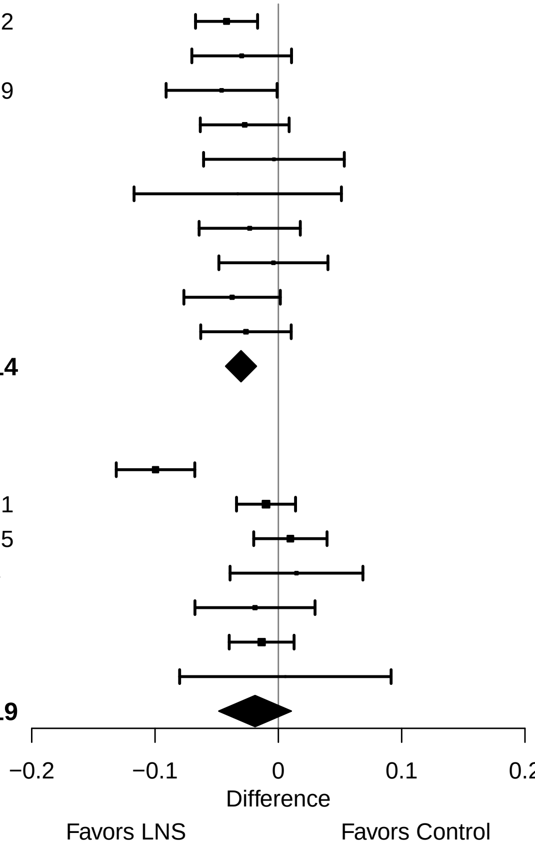

Supplemental figure 6N: Underweight prevalence difference

6N6: Stratified by Supplement duration

Supplement duration
(p−diff = 0.415)

Supplement duration − 12m or less

**Country**	**Trial**	**N**	**N**	**PD (95% CI)**	**W**
Bangladesh	JiVitA−4 (34)	2814	1232	−0.04 (−0.07, −0.02)	0.12
Burkina Faso	iLiNS−Zinc (37)	1960	666	−0.10 (−0.13, −0.07)	0.10
Burkina Faso	PROMIS (38)	857	911	−0.03 (−0.06, 0.01)	0.09
Burkina Faso	PROMIS CS (38)	430	436	0.00 (−0.06, 0.05)	0.06
Ghana	GHANA (39)	98	96	−0.03 (−0.12, 0.05)	0.03
Ghana	iLiNS−DYAD−G (40)	347	693	−0.02 (−0.06, 0.02)	0.08
Haiti	HAITI (41)	148	150	0.00 (−0.05, 0.04)	0.08
Madagascar	MAHAY (43)	1702	1685	0.01 (−0.02, 0.04)	0.11
Malawi	iLiNS−DYAD−M (44)	220	444	0.01 (−0.04, 0.07)	0.06
Malawi	iLiNS−DOSE (45)	702	241	−0.02 (−0.07, 0.03)	0.07
Zimbabwe	SHINE (HIV−) (47)	1869	1786	−0.03 (−0.05, −0.01)	0.14
Zimbabwe	SHINE (HIV+) (48)	336	328	0.00 (−0.06, 0.06)	0.06
**I² = 0.66, Tau² = 0.00**		**11483**	**8668**	**−0.02 (−0.04, −0.01)**	

Supplement duration − > 12m

**Country**	**Trial**	**N**	**N**	**PD (95% CI)**	**W**
Bangladesh	RDNS (35)	1663	815	−0.03 (−0.07, 0.01)	0.16
Bangladesh	WASH−B (36)	1163	3461	−0.07 (−0.10, −0.04)	0.21
Kenya	WASH−B (42)	1461	5165	−0.02 (−0.04, −0.01)	0.28
Mali	PROMIS (46)	500	502	−0.04 (−0.08, 0.00)	0.17
Mali	PROMIS CS (46)	946	960	−0.03 (−0.06, 0.01)	0.18
**I² = 0.43, Tau² = 0.00**		**5733**	**10903**	**−0.04 (−0.05, −0.02)**	

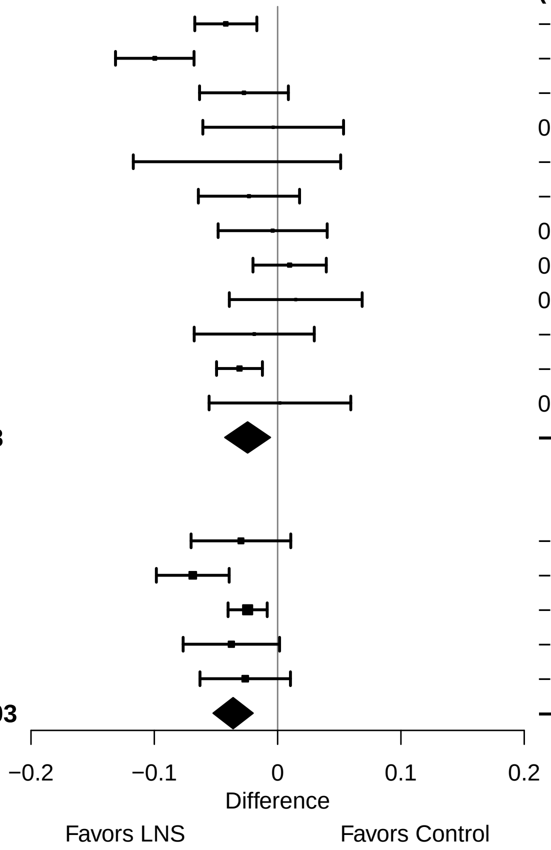

Supplemental figure 6N: Underweight prevalence difference

6N7: Stratified by Frequency of contact

Frequency of contact
(p−diff = 0.032)

Country	Trial	N	N	PD (95% CI)	W
**Frequency of contact − Monthly**					
Bangladesh	RDNS (35)	1663	815	−0.03 (−0.07, 0.01)	0.09
Burkina Faso	PROMIS (38)	857	911	−0.03 (−0.06, 0.01)	0.10
Burkina Faso	PROMIS CS (38)	430	436	0.00 (−0.06, 0.05)	0.06
Haiti	HAITI (41)	148	150	0.00 (−0.05, 0.04)	0.08
Kenya	WASH−B (42)	1461	5165	−0.02 (−0.04, −0.01)	0.15
Madagascar	MAHAY (43)	1702	1685	0.01 (−0.02, 0.04)	0.12
Mali	PROMIS (46)	500	502	−0.04 (−0.08, 0.00)	0.09
Mali	PROMIS CS (46)	946	960	−0.03 (−0.06, 0.01)	0.10
Zimbabwe	SHINE (HIV−) (47)	1869	1786	−0.03 (−0.05, −0.01)	0.15
Zimbabwe	SHINE (HIV+) (48)	336	328	0.00 (−0.06, 0.06)	0.06
**I² = 0.00, Tau² = 0.00**		**9912**	**12738**	**−0.02 (−0.03, −0.01)**	
**Frequency of contact − Weekly**					
Bangladesh	JiVitA−4 (34)	2814	1232	−0.04 (−0.07, −0.02)	0.21
Bangladesh	WASH−B (36)	1163	3461	−0.07 (−0.10, −0.04)	0.19
Burkina Faso	iLiNS−Zinc (37)	1960	666	−0.10 (−0.13, −0.07)	0.18
Ghana	GHANA (39)	98	96	−0.03 (−0.12, 0.05)	0.06
Ghana	iLiNS−DYAD−G (40)	347	693	−0.02 (−0.06, 0.02)	0.14
Malawi	iLiNS−DYAD−M (44)	220	444	0.01 (−0.04, 0.07)	0.11
Malawi	iLiNS−DOSE (45)	702	241	−0.02 (−0.07, 0.03)	0.12
**I² = 0.70, Tau² = 0.00**		**7304**	**6833**	**−0.04 (−0.07, −0.02)**	

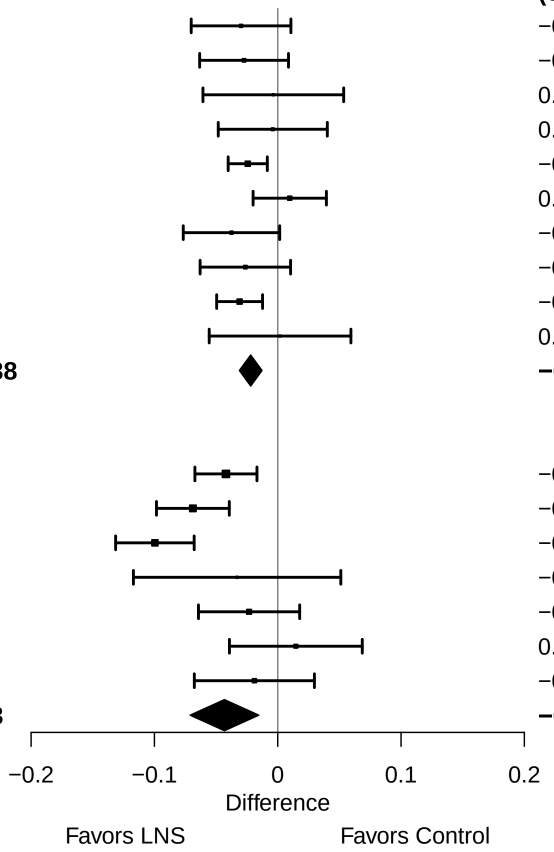

Supplemental figure 6N: Underweight prevalence difference

6N8: Stratified by Average SQ-LNS compliance

Average SQ−LNS compliance
(p−diff = 0.062)

Average SQ−LNS compliance − Low

**Country**	**Trial**	**N**	**N**	**PD (95% CI)**	**W**
Burkina Faso	PROMIS (38)	857	911	−0.03 (−0.06, 0.01)	0.13
Burkina Faso	PROMIS CS (38)	430	436	0.00 (−0.06, 0.05)	0.07
Ghana	iLiNS−DYAD−G (40)	347	693	−0.02 (−0.06, 0.02)	0.11
Malawi	iLiNS−DYAD−M (44)	220	444	0.01 (−0.04, 0.07)	0.08
Malawi	iLiNS−DOSE (45)	702	241	−0.02 (−0.07, 0.03)	0.09
Mali	PROMIS (46)	500	502	−0.04 (−0.08, 0.00)	0.12
Mali	PROMIS CS (46)	946	960	−0.03 (−0.06, 0.01)	0.13
Zimbabwe	SHINE (HIV−) (47)	1869	1786	−0.03 (−0.05, −0.01)	0.20
Zimbabwe	SHINE (HIV+) (48)	336	328	0.00 (−0.06, 0.06)	0.07
**I² = 0.00, Tau² = 0.00**		**6207**	**6301**	**−0.02 (−0.04, −0.01)**	
**Average SQ−LNS compliance − High**					
Bangladesh	JiVitA−4 (34)	2814	1232	−0.04 (−0.07, −0.02)	0.18
Bangladesh	RDNS (35)	1663	815	−0.03 (−0.07, 0.01)	0.12
Bangladesh	WASH−B (36)	1163	3461	−0.07 (−0.10, −0.04)	0.16
Burkina Faso	iLiNS−Zinc (37)	1960	666	−0.10 (−0.13, −0.07)	0.15
Ghana	GHANA (39)	98	96	−0.03 (−0.12, 0.05)	0.04
Haiti	HAITI (41)	148	150	0.00 (−0.05, 0.04)	0.11
Kenya	WASH−B (42)	1461	5165	−0.02 (−0.04, −0.01)	0.23
**I² = 0.75, Tau² = 0.00**		**9307**	**11585**	**−0.04 (−0.07, −0.02)**	

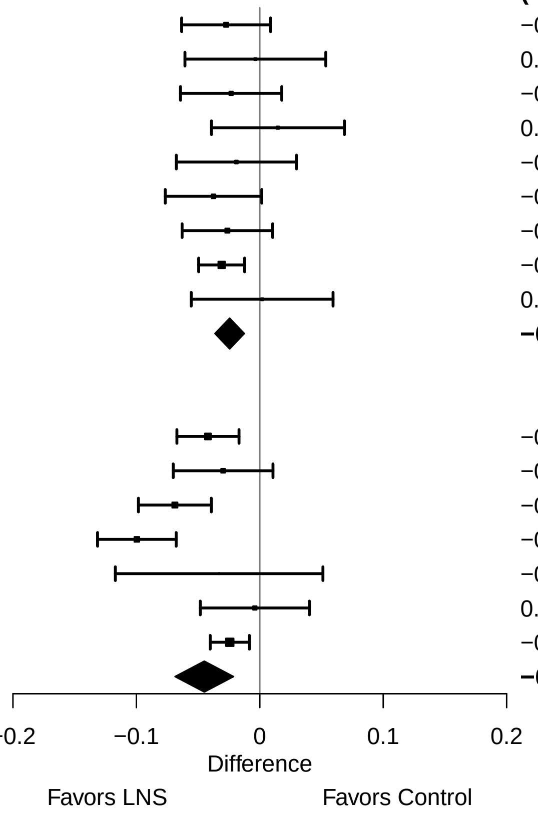

Supplemental figure 6O: Mean difference in HCZ

6O1: Stratified by Geographic region

Geographic region
(p−diff = 0.840)

Country	Trial	N	N	MD (95% CI)	W
**Geographic region − SEAR**					
Bangladesh	JiVitA−4 (34)	2885	1267	0.06 (0.01, 0.11)	0.38
Bangladesh	RDNS (35)	1663	815	0.10 (0.03, 0.17)	0.30
Bangladesh	WASH−B (36)	1163	3463	0.12 (0.06, 0.19)	0.32
**I² = 0.28, Tau² = 0.00**		**5711**	**5545**	**0.09 (0.05, 0.13)**	
**Geographic region − AFR**					
Burkina Faso	iLiNS−Zinc (37)	1946	664	0.16 (0.10, 0.23)	0.22
Burkina Faso	PROMIS (38)				
Burkina Faso	PROMIS CS (38)				
Ghana	GHANA (39)	98	96	0.20 (−0.06, 0.46)	0.03
Ghana	iLiNS−DYAD−G (40)	347	692	0.07 (−0.05, 0.18)	0.11
Kenya	WASH−B (42)	1461	5176	0.04 (−0.02, 0.10)	0.22
Madagascar	MAHAY (43)				
Malawi	iLiNS−DYAD−M (44)	220	444	−0.13 (−0.28, 0.03)	0.07
Malawi	iLiNS−DOSE (45)	692	238	0.11 (−0.02, 0.23)	0.10
Mali	PROMIS (46)				
Mali	PROMIS CS (46)				
Zimbabwe	SHINE (HIV−) (47)	1871	1783	0.09 (0.02, 0.16)	0.19
Zimbabwe	SHINE (HIV+) (48)	337	329	0.10 (−0.07, 0.28)	0.06
**I² = 0.56, Tau² = 0.00**		**6972**	**9422**	**0.08 (0.02, 0.14)**	

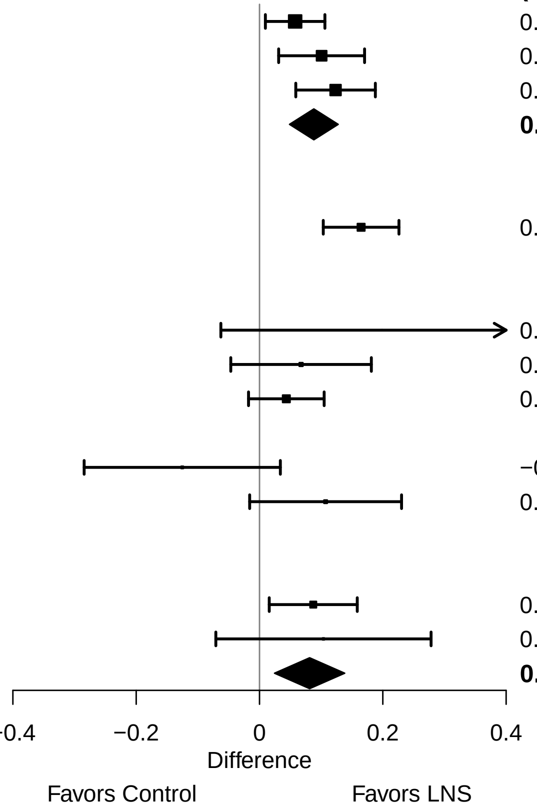

Supplemental figure 6O: Mean difference in HCZ

6O2: Stratified by Stunting burden

Stunting burden
(p−diff = 0.053)

Country	Trial	N	N	MD (95% CI)	W
**Stunting burden − Less than 35%**					
Burkina Faso	PROMIS (38)				
Burkina Faso	PROMIS CS (38)				
Ghana	GHANA (39)	98	96	0.20 (−0.06, 0.46)	0.07
Ghana	iLiNS−DYAD−G (40)	347	692	0.07 (−0.05, 0.18)	0.27
Haiti	HAITI (41)				
Kenya	WASH−B (42)	1461	5176	0.04 (−0.02, 0.10)	0.50
Malawi	iLiNS−DYAD−M (44)	220	444	−0.13 (−0.28, 0.03)	0.16
**I² = 0.48, Tau² = 0.01**		**2126**	**6408**	**0.03 (−0.08, 0.14)**	
**Stunting burden − More than 35%**					
Bangladesh	JiVitA−4 (34)	2885	1267	0.06 (0.01, 0.11)	0.20
Bangladesh	RDNS (35)	1663	815	0.10 (0.03, 0.17)	0.16
Bangladesh	WASH−B (36)	1163	3463	0.12 (0.06, 0.19)	0.17
Burkina Faso	iLiNS−Zinc (37)	1946	664	0.16 (0.10, 0.23)	0.18
Madagascar	MAHAY (43)				
Malawi	iLiNS−DOSE (45)	692	238	0.11 (−0.02, 0.23)	0.08
Mali	PROMIS (46)				
Mali	PROMIS CS (46)				
Zimbabwe	SHINE (HIV−) (47)	1871	1783	0.09 (0.02, 0.16)	0.16
Zimbabwe	SHINE (HIV+) (48)	337	329	0.10 (−0.07, 0.28)	0.05
**I² = 0.23, Tau² = 0.00**		**10557**	**8559**	**0.10 (0.07, 0.13)**	

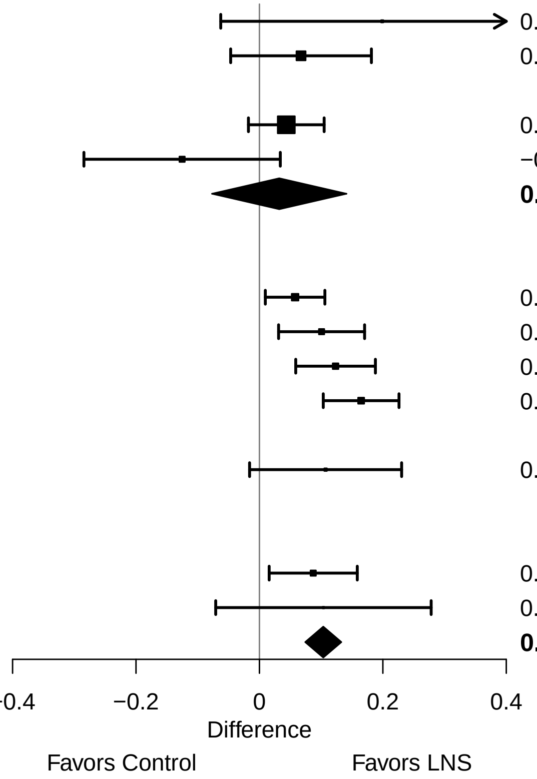

Supplemental figure 6O: Mean difference in HCZ

6O3: Stratified by Malaria prevalence

Malaria prevalence
(p−diff = 0.621)

Country	Trial	N	N	MD (95% CI)	W
**Malaria prevalence − Less than 10%**					
Bangladesh	JiVitA−4 (34)	2885	1267	0.06 (0.01, 0.11)	0.22
Bangladesh	RDNS (35)	1663	815	0.10 (0.03, 0.17)	0.17
Bangladesh	WASH−B (36)	1163	3463	0.12 (0.06, 0.19)	0.19
Haiti	HAITI (41)				
Kenya	WASH−B (42)	1461	5176	0.04 (−0.02, 0.10)	0.19
Madagascar	MAHAY (43)				
Zimbabwe	SHINE (HIV−) (47)	1871	1783	0.09 (0.02, 0.16)	0.17
Zimbabwe	SHINE (HIV+) (48)	337	329	0.10 (−0.07, 0.28)	0.05
**I² = 0.00, Tau² = 0.00**		**9380**	**12833**	**0.08 (0.05, 0.11)**	
**Malaria prevalence − At least 10%**					
Burkina Faso	iLiNS−Zinc (37)	1946	664	0.16 (0.10, 0.23)	0.40
Burkina Faso	PROMIS (38)				
Burkina Faso	PROMIS CS (38)				
Ghana	GHANA (39)	98	96	0.20 (−0.06, 0.46)	0.06
Ghana	iLiNS−DYAD−G (40)	347	692	0.07 (−0.05, 0.18)	0.21
Malawi	iLiNS−DYAD−M (44)	220	444	−0.13 (−0.28, 0.03)	0.13
Malawi	iLiNS−DOSE (45)	692	238	0.11 (−0.02, 0.23)	0.19
Mali	PROMIS (46)				
Mali	PROMIS CS (46)				
**I² = 0.68, Tau² = 0.01**		**3303**	**2134**	**0.08 (−0.02, 0.19)**	

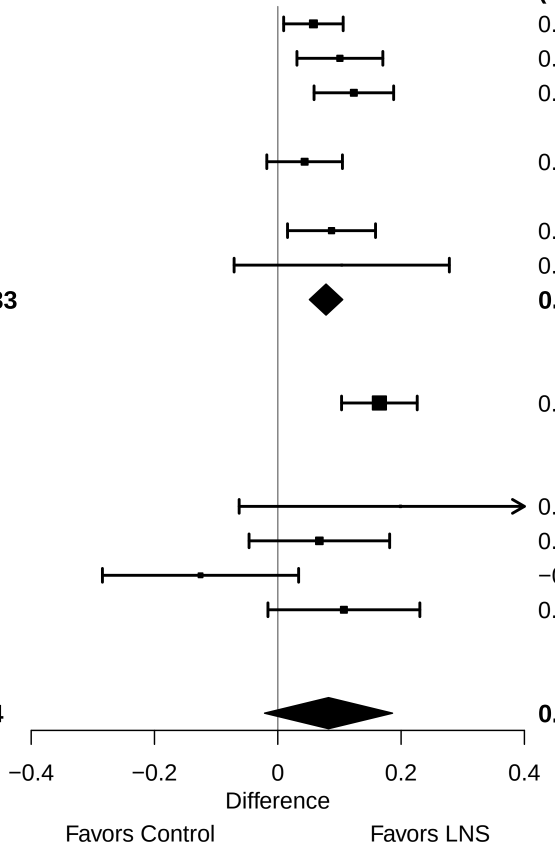

Supplemental figure 6O: Mean difference in HCZ

6O4: Stratified by Source water quality

Source water quality
(p−diff = 0.875)

Country	Trial	N	N	MD (95% CI)	W
**Source water quality − Improved**					
Bangladesh	JiVitA−4 (34)	2885	1267	0.06 (0.01, 0.11)	0.26
Bangladesh	RDNS (35)	1663	815	0.10 (0.03, 0.17)	0.21
Bangladesh	WASH−B (36)	571	1121	0.16 (0.05, 0.27)	0.14
Ghana	GHANA (39)	98	96	0.20 (−0.06, 0.46)	0.04
Ghana	iLiNS−DYAD−G (40)	347	692	0.07 (−0.05, 0.18)	0.13
Haiti	HAITI (41)				
Malawi	iLiNS−DYAD−M (44)	220	444	−0.13 (−0.28, 0.03)	0.09
Malawi	iLiNS−DOSE (45)	692	238	0.11 (−0.02, 0.23)	0.12
**I² = 0.43, Tau² = 0.00**		**6476**	**4673**	**0.08 (0.02, 0.14)**	
**Source water quality − Unimproved**					
Burkina Faso	iLiNS−Zinc (37)	1946	664	0.16 (0.10, 0.23)	0.39
Burkina Faso	PROMIS (38)				
Burkina Faso	PROMIS CS (38)				
Kenya	WASH−B (42)	696	2266	0.04 (−0.05, 0.13)	0.29
Madagascar	MAHAY (43)				
Mali	PROMIS (46)				
Mali	PROMIS CS (46)				
Zimbabwe	SHINE (HIV−) (47)	887	873	0.04 (−0.07, 0.14)	0.24
Zimbabwe	SHINE (HIV+) (48)	147	146	0.04 (−0.21, 0.28)	0.07
**I² = 0.62, Tau² = 0.00**		**3676**	**3949**	**0.09 (0.01, 0.16)**	

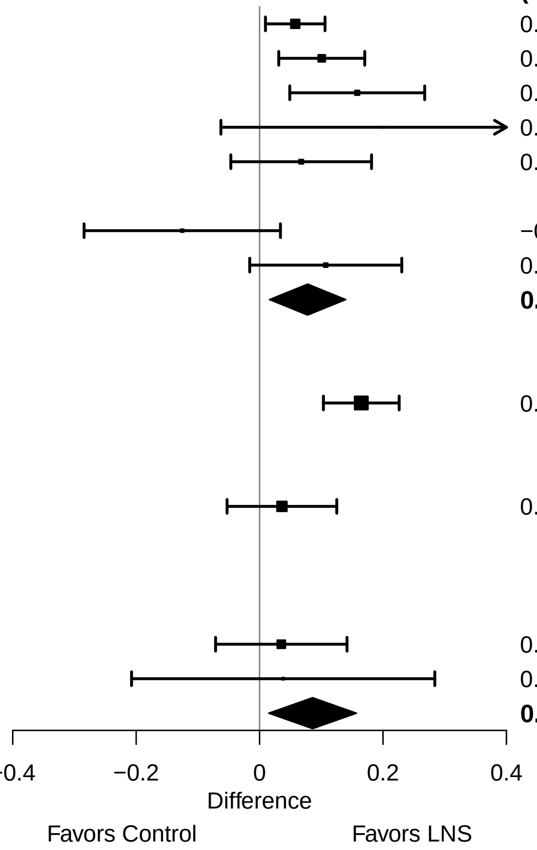

Supplemental figure 6O: Mean difference in HCZ

6O5: Stratified by Sanitation

Sanitation
(p−diff = 0.484)

Country	Trial	N	N	MD (95% CI)	W
**Sanitation − Improved**					
Bangladesh	JiVitA−4 (34)	2885	1267	0.06 (0.01, 0.11)	0.33
Bangladesh	RDNS (35)	1663	815	0.10 (0.03, 0.17)	0.27
Bangladesh	WASH−B (36)	571	1121	0.16 (0.05, 0.27)	0.18
Burkina Faso	PROMIS (38)				
Burkina Faso	PROMIS CS (38)				
Ghana	GHANA (39)	98	96	0.20 (−0.06, 0.46)	0.05
Ghana	iLiNS−DYAD−G (40)	347	692	0.07 (−0.05, 0.18)	0.17
Haiti	HAITI (41)				
Mali	PROMIS (46)				
Mali	PROMIS CS (46)				
**I² = 0.00, Tau² = 0.00**		**5564**	**3991**	**0.08 (0.05, 0.12)**	
**Sanitation − Unimproved**					
Burkina Faso	iLiNS−Zinc (37)	1946	664	0.16 (0.10, 0.23)	0.29
Kenya	WASH−B (42)	696	2266	0.04 (−0.05, 0.13)	0.22
Madagascar	MAHAY (43)				
Malawi	iLiNS−DYAD−M (44)	220	444	−0.13 (−0.28, 0.03)	0.11
Malawi	iLiNS−DOSE (45)	692	238	0.11 (−0.02, 0.23)	0.15
Zimbabwe	SHINE (HIV−) (47)	887	873	0.04 (−0.07, 0.14)	0.18
Zimbabwe	SHINE (HIV+) (48)	147	146	0.04 (−0.21, 0.28)	0.05
**I² = 0.68, Tau² = 0.01**		**4588**	**4631**	**0.06 (−0.02, 0.14)**	

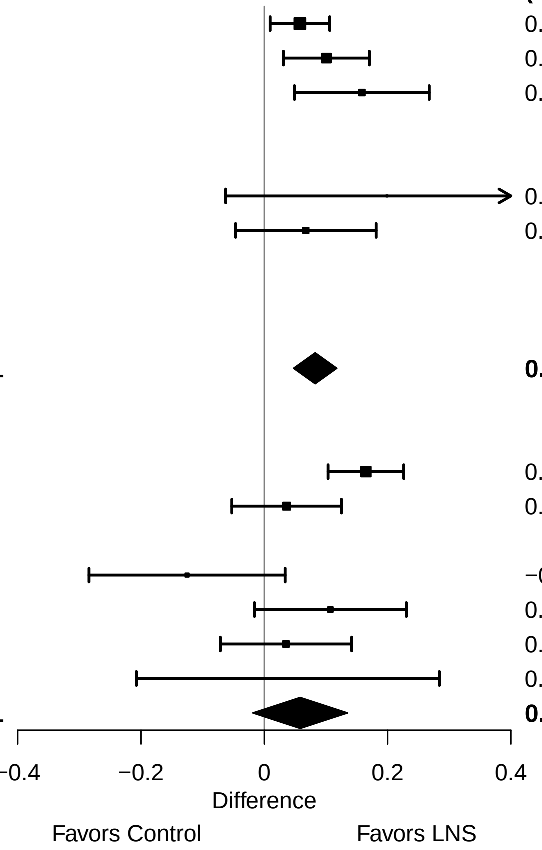

Supplemental figure 6O: Mean difference in HCZ

6O6: Stratified by Supplement duration

Supplement duration
(p−diff = 0.949)

Country	Trial	N	N	MD (95% CI)	W
**Supplement duration − 12m or less**					
Bangladesh	JiVitA−4 (34)	2885	1267	0.06 (0.01, 0.11)	0.24
Burkina Faso	iLiNS−Zinc (37)	1946	664	0.16 (0.10, 0.23)	0.21
Burkina Faso	PROMIS (38)				
Burkina Faso	PROMIS CS (38)				
Ghana	GHANA (39)	98	96	0.20 (−0.06, 0.46)	0.03
Ghana	iLiNS−DYAD−G (40)	347	692	0.07 (−0.05, 0.18)	0.11
Haiti	HAITI (41)				
Madagascar	MAHAY (43)				
Malawi	iLiNS−DYAD−M (44)	220	444	−0.13 (−0.28, 0.03)	0.07
Malawi	iLiNS−DOSE (45)	692	238	0.11 (−0.02, 0.23)	0.10
Zimbabwe	SHINE (HIV−) (47)	1871	1783	0.09 (0.02, 0.16)	0.18
Zimbabwe	SHINE (HIV+) (48)	337	329	0.10 (−0.07, 0.28)	0.06
**I² = 0.54, Tau² = 0.00**		**8396**	**5513**	**0.08 (0.03, 0.14)**	
**Supplement duration − > 12m**					
Bangladesh	RDNS (35)	1663	815	0.10 (0.03, 0.17)	0.32
Bangladesh	WASH−B (36)	1163	3463	0.12 (0.06, 0.19)	0.34
Kenya	WASH−B (42)	1461	5176	0.04 (−0.02, 0.10)	0.35
Mali	PROMIS (46)				
Mali	PROMIS CS (46)				
**I² = 0.39, Tau² = 0.00**		**4287**	**9454**	**0.09 (0.04, 0.14)**	

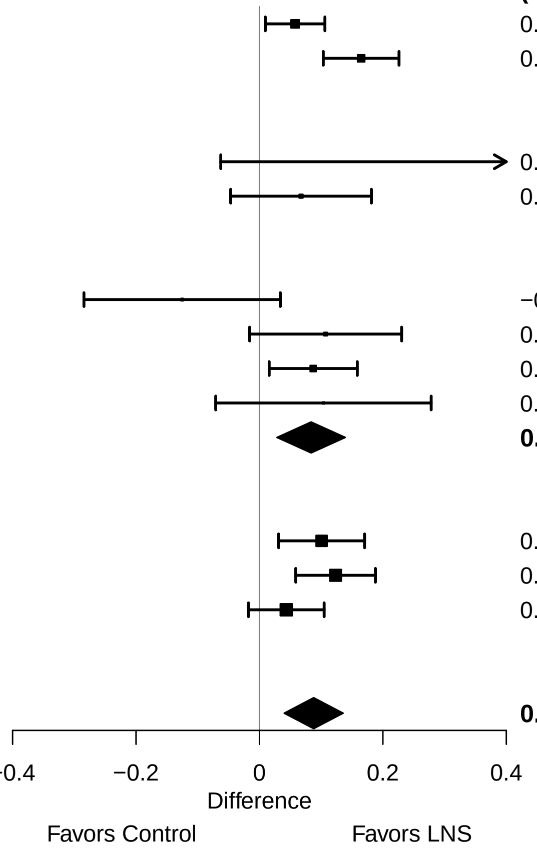

Supplemental figure 6O: Mean difference in HCZ

6O7: Stratified by Frequency of contact

Frequency of contact
(p−diff = 0.687)

Country	Trial	N	N	MD (95% CI)	W
**Frequency of contact − Monthly**					
Bangladesh	RDNS (35)	1663	815	0.10 (0.03, 0.17)	0.29
Burkina Faso	PROMIS (38)				
Burkina Faso	PROMIS CS (38)				
Haiti	HAITI (41)				
Kenya	WASH−B (42)	1461	5176	0.04 (−0.02, 0.10)	0.33
Madagascar	MAHAY (43)				
Mali	PROMIS (46)				
Mali	PROMIS CS (46)				
Zimbabwe	SHINE (HIV−) (47)	1871	1783	0.09 (0.02, 0.16)	0.29
Zimbabwe	SHINE (HIV+) (48)	337	329	0.10 (−0.07, 0.28)	0.09
**I^2 = 0.00, Tau^2 = 0.00**		**5332**	**8103**	**0.08 (0.04, 0.11)**	
**Frequency of contact − Weekly**					
Bangladesh	JiVitA−4 (34)	2885	1267	0.06 (0.01, 0.11)	0.25
Bangladesh	WASH−B (36)	1163	3463	0.12 (0.06, 0.19)	0.21
Burkina Faso	iLiNS−Zinc (37)	1946	664	0.16 (0.10, 0.23)	0.22
Ghana	GHANA (39)	98	96	0.20 (−0.06, 0.46)	0.03
Ghana	iLiNS−DYAD−G (40)	347	692	0.07 (−0.05, 0.18)	0.12
Malawi	iLiNS−DYAD−M (44)	220	444	−0.13 (−0.28, 0.03)	0.07
Malawi	iLiNS−DOSE (45)	692	238	0.11 (−0.02, 0.23)	0.10
**I^2 = 0.63, Tau^2 = 0.00**		**7351**	**6864**	**0.09 (0.02, 0.15)**	

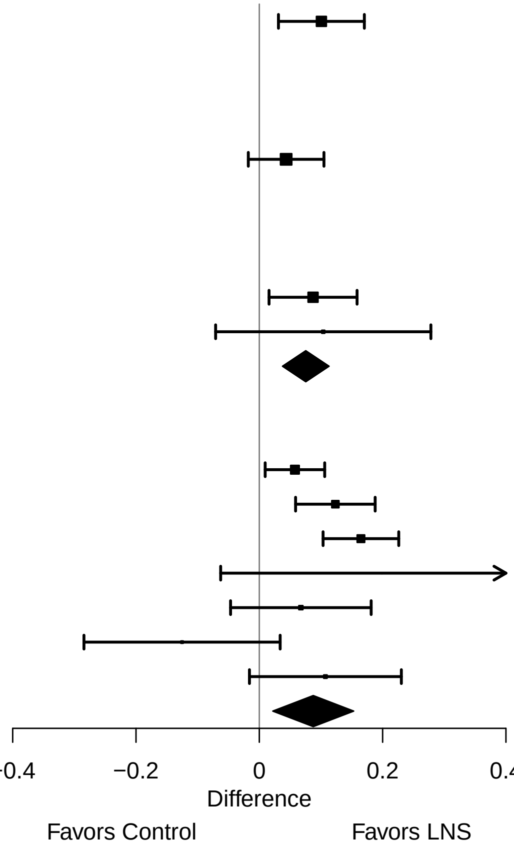

Supplemental figure 6O: Mean difference in HCZ

6O8: Stratified by Average SQ-LNS compliance

Average SQ−LNS compliance
(p−diff = 0.336)

Country	Trial	N	N	MD (95% CI)	W
**Average SQ−LNS compliance − Low**					
Burkina Faso	PROMIS (38)				
Burkina Faso	PROMIS CS (38)				
Ghana	iLiNS−DYAD−G (40)	347	692	0.07 (−0.05, 0.18)	0.21
Malawi	iLiNS−DYAD−M (44)	220	444	−0.13 (−0.28, 0.03)	0.13
Malawi	iLiNS−DOSE (45)	692	238	0.11 (−0.02, 0.23)	0.19
Mali	PROMIS (46)				
Mali	PROMIS CS (46)				
Zimbabwe	SHINE (HIV−) (47)	1871	1783	0.09 (0.02, 0.16)	0.35
Zimbabwe	SHINE (HIV+) (48)	337	329	0.10 (−0.07, 0.28)	0.11
**I² = 0.38, Tau² = 0.00**		**3467**	**3486**	**0.06 (−0.02, 0.13)**	
**Average SQ−LNS compliance − High**					
Bangladesh	JiVitA−4 (34)	2885	1267	0.06 (0.01, 0.11)	0.22
Bangladesh	RDNS (35)	1663	815	0.10 (0.03, 0.17)	0.18
Bangladesh	WASH−B (36)	1163	3463	0.12 (0.06, 0.19)	0.19
Burkina Faso	iLiNS−Zinc (37)	1946	664	0.16 (0.10, 0.23)	0.19
Ghana	GHANA (39)	98	96	0.20 (−0.06, 0.46)	0.03
Haiti	HAITI (41)				
Kenya	WASH−B (42)	1461	5176	0.04 (−0.02, 0.10)	0.19
**I² = 0.56, Tau² = 0.00**		**9216**	**11481**	**0.10 (0.06, 0.14)**	

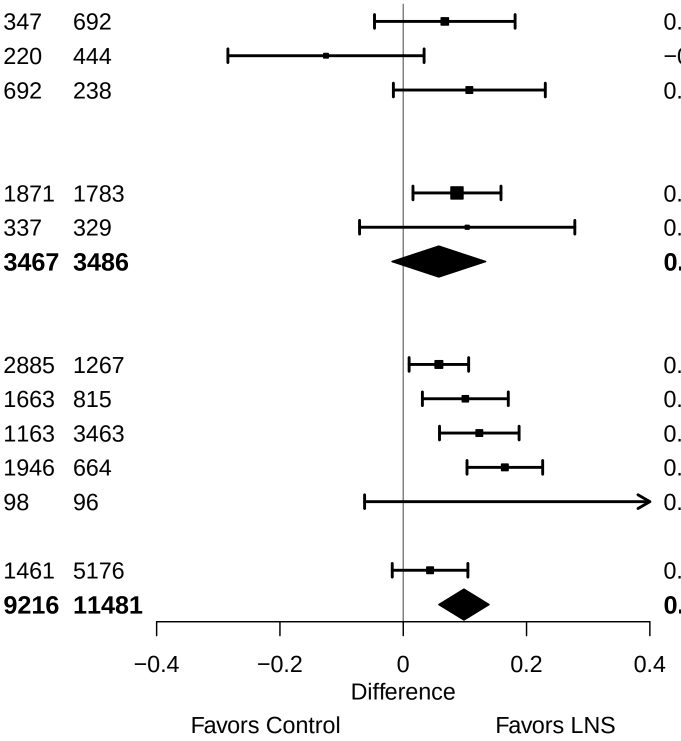

Supplemental figure 6P: Small head size prevalence ratio

6P1: Stratified by Geographic region

Geographic region
(p−diff = 0.271)

**Country**	**Trial**	**N**	**N**	**PR (95% CI)**	**W**
**Geographic region − SEAR**					
Bangladesh	JiVitA−4 (34)	2885	1267	1.03 (0.90, 1.18)	0.19
Bangladesh	RDNS (35)	1663	815	0.90 (0.82, 0.98)	0.47
Bangladesh	WASH−B (36)	1163	3463	0.90 (0.82, 1.00)	0.34
**I² = 0.36, Tau² = 0.00**		**5711**	**5545**	**0.93 (0.86, 1.01)**	
**Geographic region − AFR**					
Burkina Faso	iLiNS−Zinc (37)	1946	664	0.89 (0.78, 1.01)	0.49
Burkina Faso	PROMIS (38)				
Burkina Faso	PROMIS CS (38)				
Ghana	GHANA (39)	98	96	0.33 (0.07, 1.58)	0.00
Ghana	iLiNS−DYAD−G (40)	347	692	0.88 (0.65, 1.20)	0.09
Kenya	WASH−B (42)	1461	5176	0.92 (0.67, 1.25)	0.09
Madagascar	MAHAY (43)				
Malawi	iLiNS−DYAD−M (44)	220	444	0.88 (0.54, 1.44)	0.04
Malawi	iLiNS−DOSE (45)	692	238	0.79 (0.60, 1.03)	0.12
Mali	PROMIS (46)				
Mali	PROMIS CS (46)				
Zimbabwe	SHINE (HIV−) (47)	1871	1783	0.81 (0.63, 1.05)	0.13
Zimbabwe	SHINE (HIV+) (48)	337	329	0.98 (0.61, 1.57)	0.04
**I² = 0.00, Tau² = 0.00**		**6972**	**9422**	**0.87 (0.79, 0.95)**	

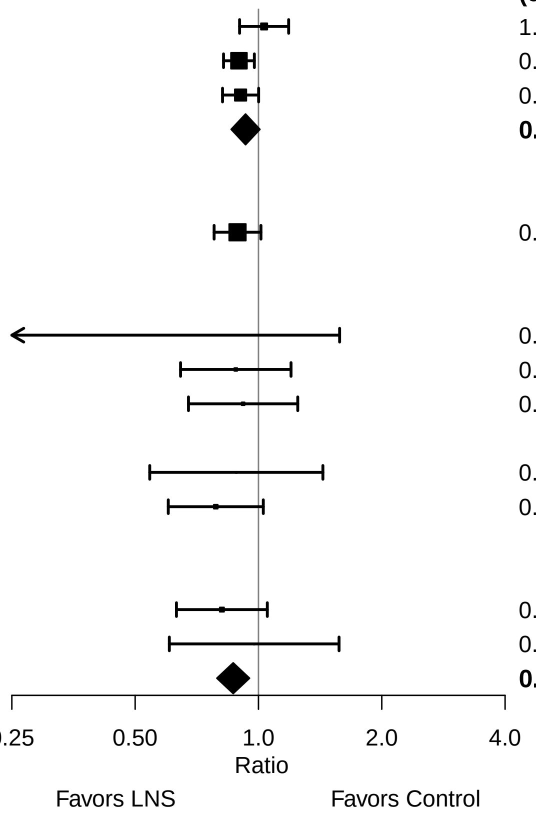

Supplemental figure 6P: Small head size prevalence ratio

6P2: Stratified by Stunting burden

Stunting burden
(p−diff = 0.780)

Country	Trial	N	N	PR (95% CI)	W
**Stunting burden − Less than 35%**					
Burkina Faso	PROMIS (38)				
Burkina Faso	PROMIS CS (38)				
Ghana	GHANA (39)	98	96	0.33 (0.07, 1.58)	0.02
Ghana	iLiNS−DYAD−G (40)	347	692	0.88 (0.65, 1.20)	0.41
Haiti	HAITI (41)				
Kenya	WASH−B (42)	1461	5176	0.92 (0.67, 1.25)	0.41
Malawi	iLiNS−DYAD−M (44)	220	444	0.88 (0.54, 1.44)	0.16
**I² = 0.00, Tau² = 0.00**		**2126**	**6408**	**0.88 (0.72, 1.07)**	
**Stunting burden − More than 35%**					
Bangladesh	JiVitA−4 (34)	2885	1267	1.03 (0.90, 1.18)	0.14
Bangladesh	RDNS (35)	1663	815	0.90 (0.82, 0.98)	0.36
Bangladesh	WASH−B (36)	1163	3463	0.90 (0.82, 1.00)	0.26
Burkina Faso	iLiNS−Zinc (37)	1946	664	0.89 (0.78, 1.01)	0.15
Madagascar	MAHAY (43)				
Malawi	iLiNS−DOSE (45)	692	238	0.79 (0.60, 1.03)	0.04
Mali	PROMIS (46)				
Mali	PROMIS CS (46)				
Zimbabwe	SHINE (HIV−) (47)	1871	1783	0.81 (0.63, 1.05)	0.04
Zimbabwe	SHINE (HIV+) (48)	337	329	0.98 (0.61, 1.57)	0.01
**I² = 0.00, Tau² = 0.00**		**10557**	**8559**	**0.91 (0.86, 0.96)**	

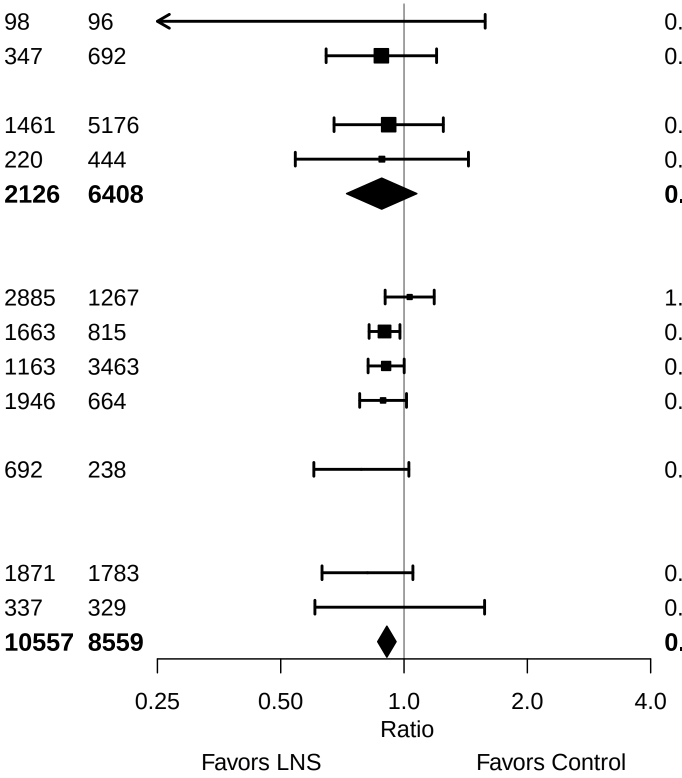

Supplemental figure 6P: Small head size prevalence ratio

6P3: Stratified by Malaria prevalence

Malaria prevalence
(p−diff = 0.351)

Country	Trial	N	N	PR (95% CI)	W
**Malaria prevalence − Less than 10%**					
Bangladesh	JiVitA−4 (34)	2885	1267	1.03 (0.90, 1.18)	0.17
Bangladesh	RDNS (35)	1663	815	0.90 (0.82, 0.98)	0.43
Bangladesh	WASH−B (36)	1163	3463	0.90 (0.82, 1.00)	0.31
Haiti	HAITI (41)				
Kenya	WASH−B (42)	1461	5176	0.92 (0.67, 1.25)	0.03
Madagascar	MAHAY (43)				
Zimbabwe	SHINE (HIV−) (47)	1871	1783	0.81 (0.63, 1.05)	0.05
Zimbabwe	SHINE (HIV+) (48)	337	329	0.98 (0.61, 1.57)	0.01
**I² = 0.00, Tau² = 0.00**		**9380**	**12833**	**0.92 (0.87, 0.97)**	
**Malaria prevalence − At least 10%**					
Burkina Faso	iLiNS−Zinc (37)	1946	664	0.89 (0.78, 1.01)	0.67
Burkina Faso	PROMIS (38)				
Burkina Faso	PROMIS CS (38)				
Ghana	GHANA (39)	98	96	0.33 (0.07, 1.58)	0.00
Ghana	iLiNS−DYAD−G (40)	347	692	0.88 (0.65, 1.20)	0.12
Malawi	iLiNS−DYAD−M (44)	220	444	0.88 (0.54, 1.44)	0.05
Malawi	iLiNS−DOSE (45)	692	238	0.79 (0.60, 1.03)	0.16
Mali	PROMIS (46)				
Mali	PROMIS CS (46)				
**I² = 0.00, Tau² = 0.00**		**3303**	**2134**	**0.87 (0.78, 0.96)**	

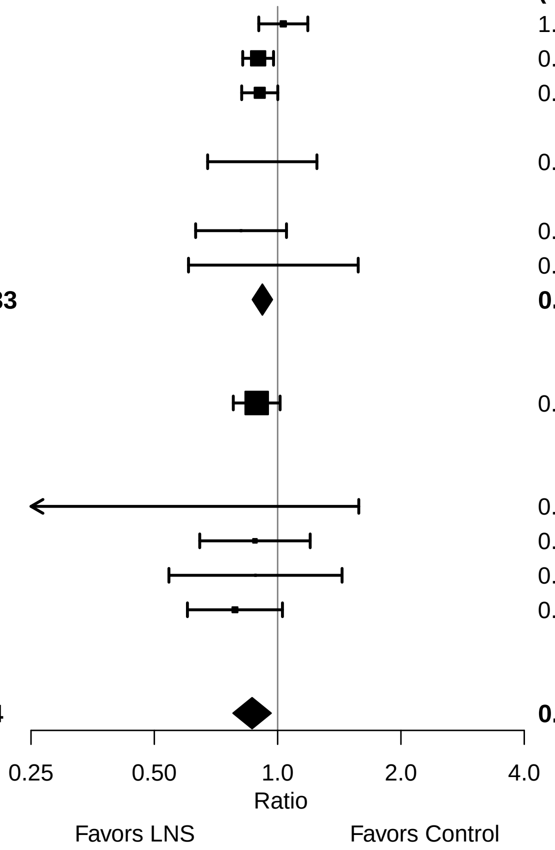

Supplemental figure 6P: Small head size prevalence ratio

6P4: Stratified by Source water quality

Source water quality
(p−diff = 0.958)

Source water quality − Improved

Country	Trial	N	N	PR (95% CI)	W
Bangladesh	JiVitA−4 (34)	2885	1267	1.03 (0.90, 1.18)	0.21
Bangladesh	RDNS (35)	1663	815	0.90 (0.82, 0.98)	0.54
Bangladesh	WASH−B (36)	571	1121	0.86 (0.73, 1.03)	0.13
Ghana	GHANA (39)	98	96	0.33 (0.07, 1.58)	0.00
Ghana	iLiNS−DYAD−G (40)	347	692	0.88 (0.65, 1.20)	0.04
Haiti	HAITI (41)				
Malawi	iLiNS−DYAD−M (44)	220	444	0.88 (0.54, 1.44)	0.02
Malawi	iLiNS−DOSE (45)	692	238	0.79 (0.60, 1.03)	0.06
**I² = 0.08, Tau² = 0.00**		**6476**	**4673**	**0.91 (0.85, 0.98)**	

Source water quality − Unimproved

Country	Trial	N	N	PR (95% CI)	W
Burkina Faso	iLiNS−Zinc (37)	1946	664	0.89 (0.78, 1.01)	0.79
Burkina Faso	PROMIS (38)				
Burkina Faso	PROMIS CS (38)				
Kenya	WASH−B (42)	696	2266	0.73 (0.45, 1.17)	0.06
Madagascar	MAHAY (43)				
Mali	PROMIS (46)				
Mali	PROMIS CS (46)				
Zimbabwe	SHINE (HIV−) (47)	887	873	1.01 (0.73, 1.39)	0.13
Zimbabwe	SHINE (HIV+) (48)	147	146	1.44 (0.68, 3.06)	0.02
**I² = 0.00, Tau² = 0.00**		**3676**	**3949**	**0.90 (0.80, 1.01)**	

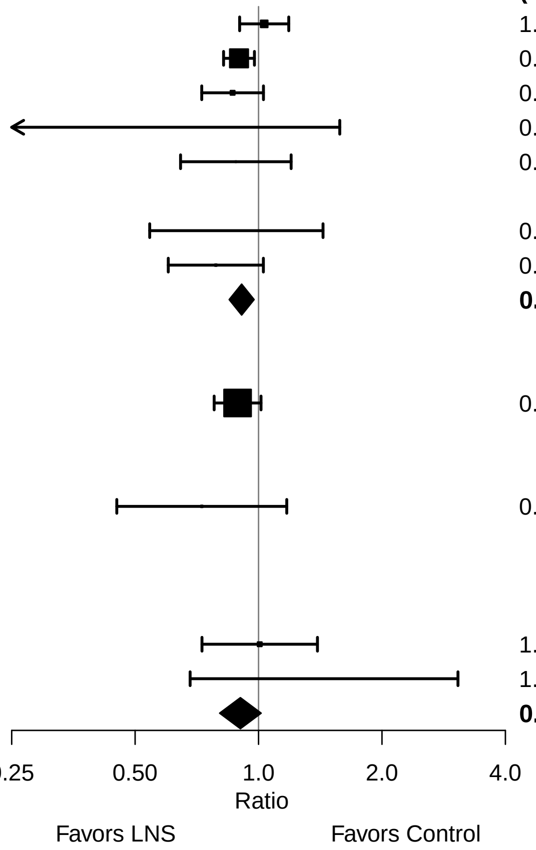

Supplemental figure 6P: Small head size prevalence ratio

6P5: Stratified by Sanitation

Sanitation
(p−diff = 0.549)

Country	Trial	N	N	PR (95% CI)	W
**Sanitation − Improved**					
Bangladesh	JiVitA−4 (34)	2885	1267	1.03 (0.90, 1.18)	0.23
Bangladesh	RDNS (35)	1663	815	0.90 (0.82, 0.98)	0.58
Bangladesh	WASH−B (36)	571	1121	0.86 (0.73, 1.03)	0.14
Burkina Faso	PROMIS (38)				
Burkina Faso	PROMIS CS (38)				
Ghana	GHANA (39)	98	96	0.33 (0.07, 1.58)	0.00
Ghana	iLiNS−DYAD−G (40)	347	692	0.88 (0.65, 1.20)	0.05
Haiti	HAITI (41)				
Mali	PROMIS (46)				
Mali	PROMIS CS (46)				
**I² = 0.24, Tau² = 0.00**		**5564**	**3991**	**0.92 (0.84, 1.01)**	
**Sanitation − Unimproved**					
Burkina Faso	iLiNS−Zinc (37)	1946	664	0.89 (0.78, 1.01)	0.63
Kenya	WASH−B (42)	696	2266	0.73 (0.45, 1.17)	0.05
Madagascar	MAHAY (43)				
Malawi	iLiNS−DYAD−M (44)	220	444	0.88 (0.54, 1.44)	0.05
Malawi	iLiNS−DOSE (45)	692	238	0.79 (0.60, 1.03)	0.15
Zimbabwe	SHINE (HIV−) (47)	887	873	1.01 (0.73, 1.39)	0.10
Zimbabwe	SHINE (HIV+) (48)	147	146	1.44 (0.68, 3.06)	0.02
**I² = 0.00, Tau² = 0.00**		**4588**	**4631**	**0.88 (0.80, 0.98)**	

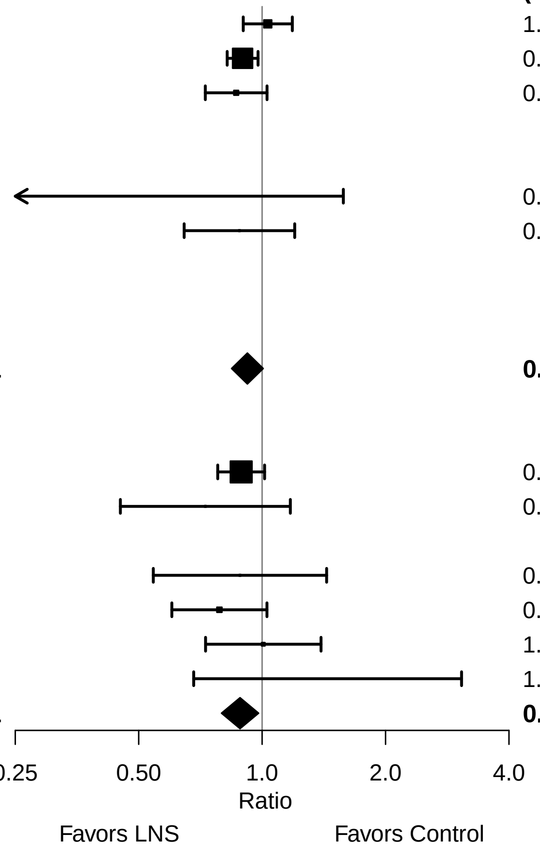

Supplemental figure 6P: Small head size prevalence ratio

6P6: Stratified by Supplement duration

Supplement duration
(p−diff = 0.749)

Country	Trial	N	N	PR (95% CI)	W
**Supplement duration − 12m or less**					
Bangladesh	JiVitA−4 (34)	2885	1267	1.03 (0.90, 1.18)	0.33
Burkina Faso	iLiNS−Zinc (37)	1946	664	0.89 (0.78, 1.01)	0.36
Burkina Faso	PROMIS (38)				
Burkina Faso	PROMIS CS (38)				
Ghana	GHANA (39)	98	96	0.33 (0.07, 1.58)	0.00
Ghana	iLiNS−DYAD−G (40)	347	692	0.88 (0.65, 1.20)	0.07
Haiti	HAITI (41)				
Madagascar	MAHAY (43)				
Malawi	iLiNS−DYAD−M (44)	220	444	0.88 (0.54, 1.44)	0.03
Malawi	iLiNS−DOSE (45)	692	238	0.79 (0.60, 1.03)	0.09
Zimbabwe	SHINE (HIV−) (47)	1871	1783	0.81 (0.63, 1.05)	0.10
Zimbabwe	SHINE (HIV+) (48)	337	329	0.98 (0.61, 1.57)	0.03
**I² = 0.00, Tau² = 0.00**		**8396**	**5513**	**0.92 (0.85, 0.99)**	
**Supplement duration − > 12m**					
Bangladesh	RDNS (35)	1663	815	0.90 (0.82, 0.98)	0.55
Bangladesh	WASH−B (36)	1163	3463	0.90 (0.82, 1.00)	0.40
Kenya	WASH−B (42)	1461	5176	0.92 (0.67, 1.25)	0.04
Mali	PROMIS (46)				
Mali	PROMIS CS (46)				
**I² = 0.00, Tau² = 0.00**		**4287**	**9454**	**0.90 (0.84, 0.96)**	

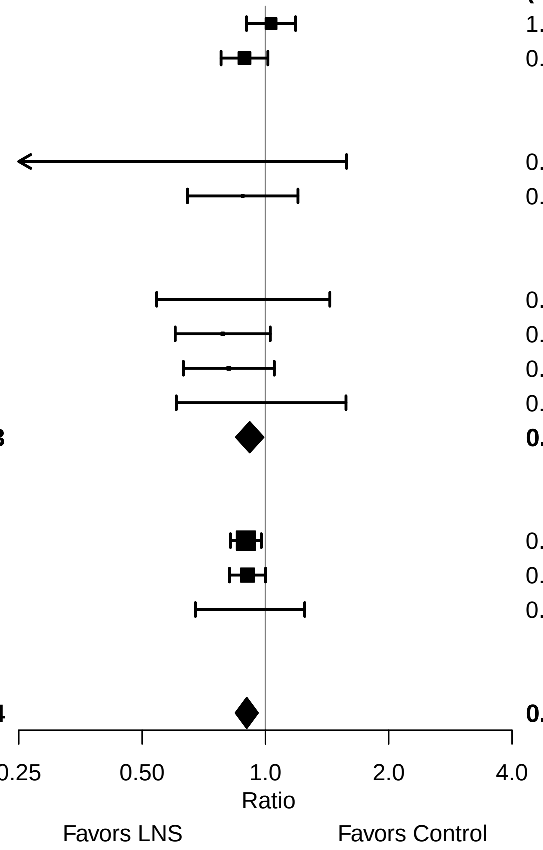

Supplemental figure 6P: Small head size prevalence ratio

6P7: Stratified by Frequency of contact

Frequency of contact
(p−diff = 0.591)

Frequency of contact − Monthly

**Country**	**Trial**	**N**	**N**	**PR (95% CI)**	**W**
Bangladesh	RDNS (35)	1663	815	0.90 (0.82, 0.98)	0.81
Burkina Faso	PROMIS (38)				
Burkina Faso	PROMIS CS (38)				
Haiti	HAITI (41)				
Kenya	WASH−B (42)	1461	5176	0.92 (0.67, 1.25)	0.06
Madagascar	MAHAY (43)				
Mali	PROMIS (46)				
Mali	PROMIS CS (46)				
Zimbabwe	SHINE (HIV−) (47)	1871	1783	0.81 (0.63, 1.05)	0.09
Zimbabwe	SHINE (HIV+) (48)	337	329	0.98 (0.61, 1.57)	0.03
**I^2 = 0.00, Tau^2 = 0.00**		**5332**	**8103**	**0.89 (0.82, 0.96)**	

Frequency of contact − Weekly

**Country**	**Trial**	**N**	**N**	**PR (95% CI)**	**W**
Bangladesh	JiVitA−4 (34)	2885	1267	1.03 (0.90, 1.18)	0.22
Bangladesh	WASH−B (36)	1163	3463	0.90 (0.82, 1.00)	0.41
Burkina Faso	iLiNS−Zinc (37)	1946	664	0.89 (0.78, 1.01)	0.24
Ghana	GHANA (39)	98	96	0.33 (0.07, 1.58)	0.00
Ghana	iLiNS−DYAD−G (40)	347	692	0.88 (0.65, 1.20)	0.04
Malawi	iLiNS−DYAD−M (44)	220	444	0.88 (0.54, 1.44)	0.02
Malawi	iLiNS−DOSE (45)	692	238	0.79 (0.60, 1.03)	0.06
**I^2 = 0.02, Tau^2 = 0.00**		**7351**	**6864**	**0.92 (0.86, 0.98)**	

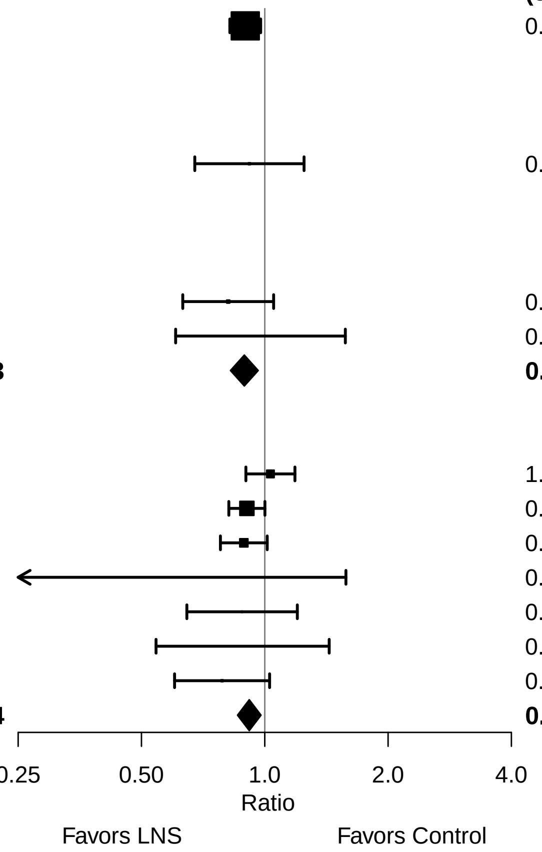

Supplemental figure 6P: Small head size prevalence ratio

6P8: Stratified by Average SQ-LNS compliance

Average SQ−LNS compliance
(p−diff = 0.264)

Country	Trial	N	N	PR (95% CI)	W
**Average SQ−LNS compliance − Low**					
Burkina Faso	PROMIS (38)				
Burkina Faso	PROMIS CS (38)				
Ghana	iLiNS−DYAD−G (40)	347	692	0.88 (0.65, 1.20)	0.21
Malawi	iLiNS−DYAD−M (44)	220	444	0.88 (0.54, 1.44)	0.09
Malawi	iLiNS−DOSE (45)	692	238	0.79 (0.60, 1.03)	0.29
Mali	PROMIS (46)				
Mali	PROMIS CS (46)				
Zimbabwe	SHINE (HIV−) (47)	1871	1783	0.81 (0.63, 1.05)	0.32
Zimbabwe	SHINE (HIV+) (48)	337	329	0.98 (0.61, 1.57)	0.09
**I² = 0.00, Tau² = 0.00**		**3467**	**3486**	**0.84 (0.73, 0.97)**	
**Average SQ−LNS compliance − High**					
Bangladesh	JiVitA−4 (34)	2885	1267	1.03 (0.90, 1.18)	0.15
Bangladesh	RDNS (35)	1663	815	0.90 (0.82, 0.98)	0.38
Bangladesh	WASH−B (36)	1163	3463	0.90 (0.82, 1.00)	0.28
Burkina Faso	iLiNS−Zinc (37)	1946	664	0.89 (0.78, 1.01)	0.16
Ghana	GHANA (39)	98	96	0.33 (0.07, 1.58)	0.00
Haiti	HAITI (41)				
Kenya	WASH−B (42)	1461	5176	0.92 (0.67, 1.25)	0.03
**I² = 0.01, Tau² = 0.00**		**9216**	**11481**	**0.92 (0.87, 0.97)**	

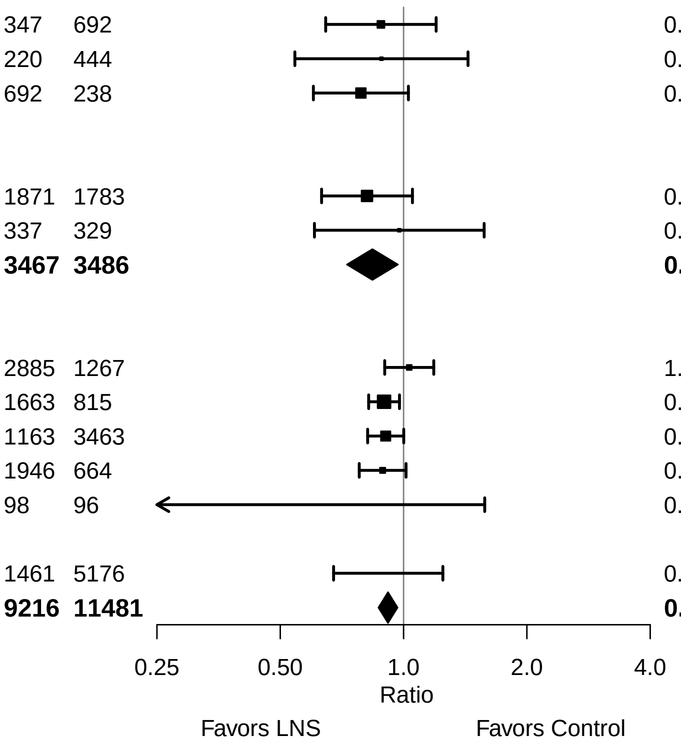

Supplemental figure 6Q: Small head size prevalence difference

6Q1: Stratified by Geographic region

Geographic region
(p−diff = 0.664)

Country	Trial	N	N	PD (95% CI)	W
**Geographic region − SEAR**					
Bangladesh	JiVitA−4 (34)	2885	1267	0.00 (−0.03, 0.02)	0.48
Bangladesh	RDNS (35)	1663	815	−0.04 (−0.09, 0.00)	0.20
Bangladesh	WASH−B (36)	1163	3463	−0.03 (−0.06, 0.00)	0.32
**I² = 0.50, Tau² = 0.00**		**5711**	**5545**	**−0.02 (−0.05, 0.00)**	
**Geographic region − AFR**					
Burkina Faso	iLiNS−Zinc (37)	1946	664	−0.04 (−0.08, −0.01)	0.11
Burkina Faso	PROMIS (38)				
Burkina Faso	PROMIS CS (38)				
Ghana	GHANA (39)	98	96	−0.04 (−0.10, 0.01)	0.04
Ghana	iLiNS−DYAD−G (40)	347	692	−0.02 (−0.06, 0.03)	0.06
Kenya	WASH−B (42)	1461	5176	0.00 (−0.02, 0.01)	0.34
Madagascar	MAHAY (43)				
Malawi	iLiNS−DYAD−M (44)	220	444	−0.01 (−0.06, 0.04)	0.06
Malawi	iLiNS−DOSE (45)	692	238	−0.04 (−0.10, 0.01)	0.05
Mali	PROMIS (46)				
Mali	PROMIS CS (46)				
Zimbabwe	SHINE (HIV−) (47)	1871	1783	−0.01 (−0.03, 0.00)	0.29
Zimbabwe	SHINE (HIV+) (48)	337	329	0.00 (−0.05, 0.04)	0.06
**I² = 0.14, Tau² = 0.00**		**6972**	**9422**	**−0.01 (−0.03, 0.00)**	

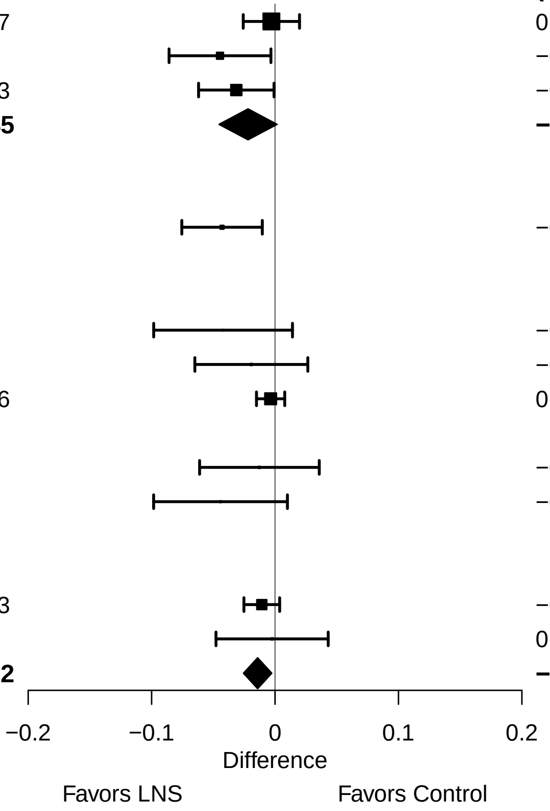

Supplemental figure 6Q: Small head size prevalence difference

6Q2: Stratified by Stunting burden

Stunting burden
(p−diff = 0.345)

Country	Trial	N	N	PD (95% CI)	W
**Stunting burden − Less than 35%**					
Burkina Faso	PROMIS (38)				
Burkina Faso	PROMIS CS (38)				
Ghana	GHANA (39)	98	96	−0.04 (−0.10, 0.01)	0.09
Ghana	iLiNS−DYAD−G (40)	347	692	−0.02 (−0.06, 0.03)	0.13
Haiti	HAITI (41)				
Kenya	WASH−B (42)	1461	5176	0.00 (−0.02, 0.01)	0.67
Malawi	iLiNS−DYAD−M (44)	220	444	−0.01 (−0.06, 0.04)	0.11
**I² = 0.00, Tau² = 0.00**		**2126**	**6408**	**−0.01 (−0.02, 0.00)**	
**Stunting burden − More than 35%**					
Bangladesh	JiVitA−4 (34)	2885	1267	0.00 (−0.03, 0.02)	0.20
Bangladesh	RDNS (35)	1663	815	−0.04 (−0.09, 0.00)	0.08
Bangladesh	WASH−B (36)	1163	3463	−0.03 (−0.06, 0.00)	0.14
Burkina Faso	iLiNS−Zinc (37)	1946	664	−0.04 (−0.08, −0.01)	0.12
Madagascar	MAHAY (43)				
Malawi	iLiNS−DOSE (45)	692	238	−0.04 (−0.10, 0.01)	0.05
Mali	PROMIS (46)				
Mali	PROMIS CS (46)				
Zimbabwe	SHINE (HIV−) (47)	1871	1783	−0.01 (−0.03, 0.00)	0.33
Zimbabwe	SHINE (HIV+) (48)	337	329	0.00 (−0.05, 0.04)	0.07
**I² = 0.30, Tau² = 0.00**		**10557**	**8559**	**−0.02 (−0.03, −0.01)**	

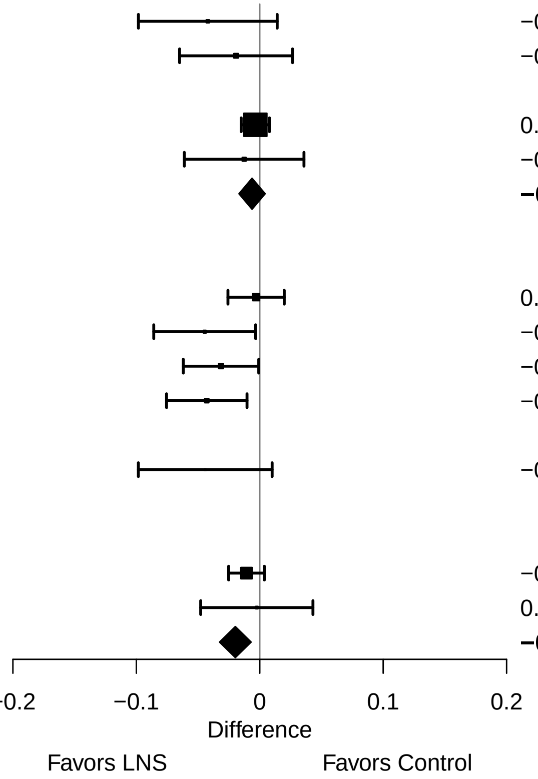

Supplemental figure 6Q: Small head size prevalence difference

6Q3: Stratified by Malaria prevalence

Malaria prevalence
(p−diff = 0.025)

Country	Trial	N	N	PD (95% CI)	W
**Malaria prevalence − Less than 10%**					
Bangladesh	JiVitA−4 (34)	2885	1267	0.00 (−0.03, 0.02)	0.17
Bangladesh	RDNS (35)	1663	815	−0.04 (−0.09, 0.00)	0.07
Bangladesh	WASH−B (36)	1163	3463	−0.03 (−0.06, 0.00)	0.11
Haiti	HAITI (41)				
Kenya	WASH−B (42)	1461	5176	0.00 (−0.02, 0.01)	0.32
Madagascar	MAHAY (43)				
Zimbabwe	SHINE (HIV−) (47)	1871	1783	−0.01 (−0.03, 0.00)	0.27
Zimbabwe	SHINE (HIV+) (48)	337	329	0.00 (−0.05, 0.04)	0.06
**I² = 0.19, Tau² = 0.00**		**9380**	**12833**	**−0.01 (−0.02, 0.00)**	
**Malaria prevalence − At least 10%**					
Burkina Faso	iLiNS−Zinc (37)	1946	664	−0.04 (−0.08, −0.01)	0.34
Burkina Faso	PROMIS (38)				
Burkina Faso	PROMIS CS (38)				
Ghana	GHANA (39)	98	96	−0.04 (−0.10, 0.01)	0.14
Ghana	iLiNS−DYAD−G (40)	347	692	−0.02 (−0.06, 0.03)	0.20
Malawi	iLiNS−DYAD−M (44)	220	444	−0.01 (−0.06, 0.04)	0.18
Malawi	iLiNS−DOSE (45)	692	238	−0.04 (−0.10, 0.01)	0.15
Mali	PROMIS (46)				
Mali	PROMIS CS (46)				
**I² = 0.00, Tau² = 0.00**		**3303**	**2134**	**−0.03 (−0.05, −0.01)**	

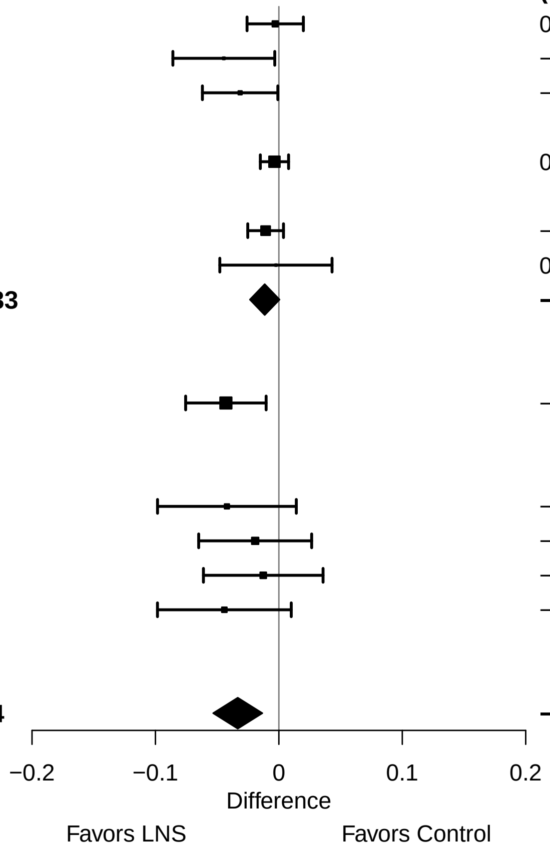

Supplemental figure 6Q: Small head size prevalence difference

6Q4: Stratified by Source water quality

Source water quality
(p−diff = 0.301)

Country	Trial	N	N	PD (95% CI)	W
**Source water quality − Improved**					
Bangladesh	JiVitA−4 (34)	2885	1267	0.00 (−0.03, 0.02)	0.30
Bangladesh	RDNS (35)	1663	815	−0.04 (−0.09, 0.00)	0.15
Bangladesh	WASH−B (36)	571	1121	−0.05 (−0.09, 0.00)	0.12
Ghana	GHANA (39)	98	96	−0.04 (−0.10, 0.01)	0.09
Ghana	iLiNS−DYAD−G (40)	347	692	−0.02 (−0.06, 0.03)	0.13
Haiti	HAITI (41)				
Malawi	iLiNS−DYAD−M (44)	220	444	−0.01 (−0.06, 0.04)	0.11
Malawi	iLiNS−DOSE (45)	692	238	−0.04 (−0.10, 0.01)	0.10
**I² = 0.02, Tau² = 0.00**		**6476**	**4673**	**−0.02 (−0.04, −0.01)**	
**Source water quality − Unimproved**					
Burkina Faso	iLiNS−Zinc (37)	1946	664	−0.04 (−0.08, −0.01)	0.20
Burkina Faso	PROMIS (38)				
Burkina Faso	PROMIS CS (38)				
Kenya	WASH−B (42)	696	2266	−0.01 (−0.03, 0.00)	0.40
Madagascar	MAHAY (43)				
Mali	PROMIS (46)				
Mali	PROMIS CS (46)				
Zimbabwe	SHINE (HIV−) (47)	887	873	0.00 (−0.02, 0.02)	0.33
Zimbabwe	SHINE (HIV+) (48)	147	146	0.03 (−0.03, 0.10)	0.07
**I² = 0.55, Tau² = 0.00**		**3676**	**3949**	**−0.01 (−0.04, 0.01)**	

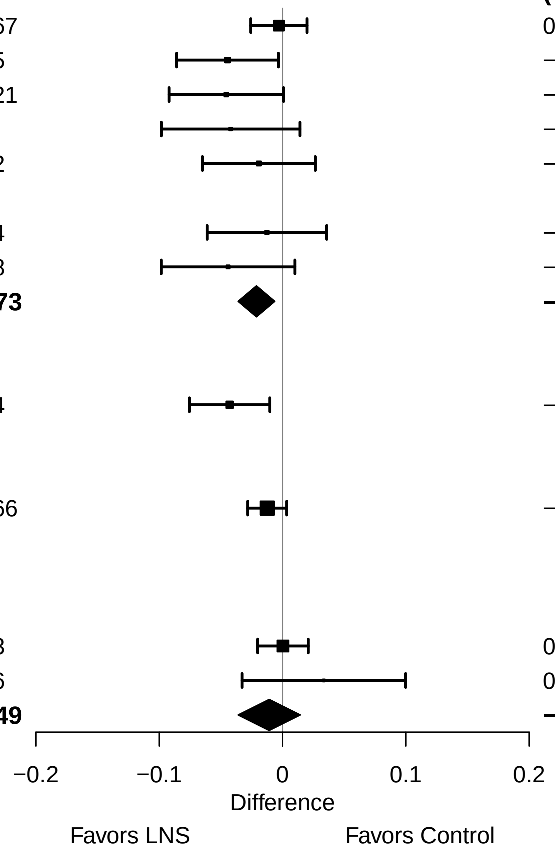

Supplemental figure 6Q: Small head size prevalence difference

6Q5: Stratified by Sanitation

Sanitation
(p−diff = 0.442)

Country	Trial	N	N	PD (95% CI)	W
**Sanitation − Improved**					
Bangladesh	JiVitA−4 (34)	2885	1267	0.00 (−0.03, 0.02)	0.38
Bangladesh	RDNS (35)	1663	815	−0.04 (−0.09, 0.00)	0.19
Bangladesh	WASH−B (36)	571	1121	−0.05 (−0.09, 0.00)	0.16
Burkina Faso	PROMIS (38)				
Burkina Faso	PROMIS CS (38)				
Ghana	GHANA (39)	98	96	−0.04 (−0.10, 0.01)	0.11
Ghana	iLiNS−DYAD−G (40)	347	692	−0.02 (−0.06, 0.03)	0.16
Haiti	HAITI (41)				
Mali	PROMIS (46)				
Mali	PROMIS CS (46)				
**I² = 0.24, Tau² = 0.00**		**5564**	**3991**	**−0.02 (−0.04, 0.00)**	
**Sanitation − Unimproved**					
Burkina Faso	iLiNS−Zinc (37)	1946	664	−0.04 (−0.08, −0.01)	0.17
Kenya	WASH−B (42)	696	2266	−0.01 (−0.03, 0.00)	0.33
Madagascar	MAHAY (43)				
Malawi	iLiNS−DYAD−M (44)	220	444	−0.01 (−0.06, 0.04)	0.09
Malawi	iLiNS−DOSE (45)	692	238	−0.04 (−0.10, 0.01)	0.08
Zimbabwe	SHINE (HIV−) (47)	887	873	0.00 (−0.02, 0.02)	0.27
Zimbabwe	SHINE (HIV+) (48)	147	146	0.03 (−0.03, 0.10)	0.06
**I² = 0.38, Tau² = 0.00**		**4588**	**4631**	**−0.01 (−0.03, 0.00)**	

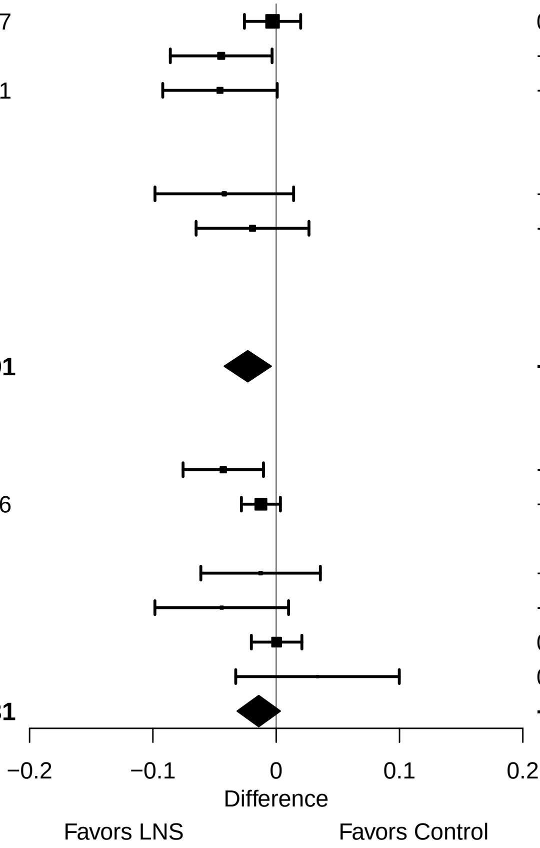

Supplemental figure 6Q: Small head size prevalence difference

6Q6: Stratified by Supplement duration

Supplement duration
(p−diff = 0.995)

**Supplement duration − 12m or less**				**PD**	
**Country**	**Trial**	**N**	**N**	**(95% CI)**	**W**
Bangladesh	JiVitA−4 (34)	2885	1267	0.00 (−0.03, 0.02)	0.21
Burkina Faso	iLiNS−Zinc (37)	1946	664	−0.04 (−0.08, −0.01)	0.13
Burkina Faso	PROMIS (38)				
Burkina Faso	PROMIS CS (38)				
Ghana	GHANA (39)	98	96	−0.04 (−0.10, 0.01)	0.05
Ghana	iLiNS−DYAD−G (40)	347	692	−0.02 (−0.06, 0.03)	0.07
Haiti	HAITI (41)				
Madagascar	MAHAY (43)				
Malawi	iLiNS−DYAD−M (44)	220	444	−0.01 (−0.06, 0.04)	0.07
Malawi	iLiNS−DOSE (45)	692	238	−0.04 (−0.10, 0.01)	0.05
Zimbabwe	SHINE (HIV−) (47)	1871	1783	−0.01 (−0.03, 0.00)	0.34
Zimbabwe	SHINE (HIV+) (48)	337	329	0.00 (−0.05, 0.04)	0.07
**I² = 0.00, Tau² = 0.00**		**8396**	**5513**	**−0.01 (−0.02, 0.00)**	
**Supplement duration − > 12m**					
Bangladesh	RDNS (35)	1663	815	−0.04 (−0.09, 0.00)	0.14
Bangladesh	WASH−B (36)	1163	3463	−0.03 (−0.06, 0.00)	0.23
Kenya	WASH−B (42)	1461	5176	0.00 (−0.02, 0.01)	0.63
Mali	PROMIS (46)				
Mali	PROMIS CS (46)				
**I² = 0.65, Tau² = 0.00**		**4287**	**9454**	**−0.02 (−0.04, 0.00)**	

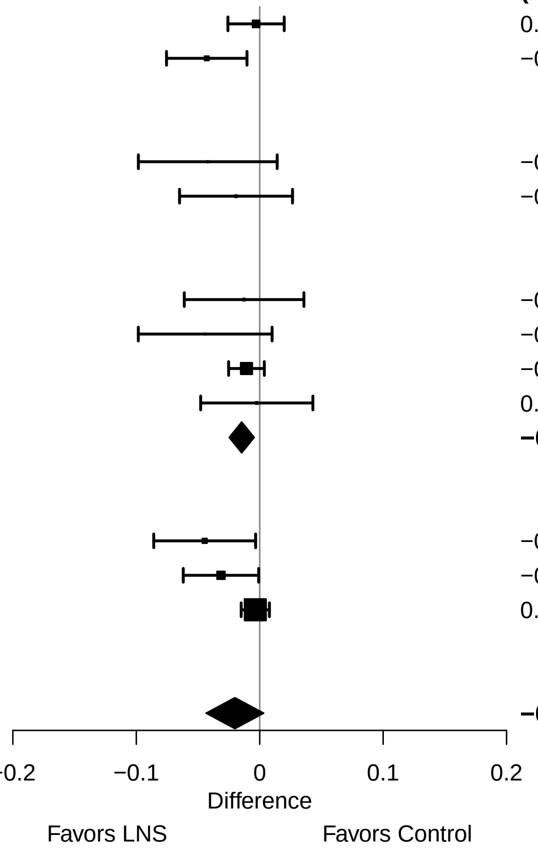

Supplemental figure 6Q: Small head size prevalence difference

6Q7: Stratified by Frequency of contact

Frequency of contact
(p−diff = 0.091)

Country	Trial	N	N	PD (95% CI)	W
**Frequency of contact − Monthly**					
Bangladesh	RDNS (35)	1663	815	−0.04 (−0.09, 0.00)	0.10
Burkina Faso	PROMIS (38)				
Burkina Faso	PROMIS CS (38)				
Haiti	HAITI (41)				
Kenya	WASH−B (42)	1461	5176	0.00 (−0.02, 0.01)	0.44
Madagascar	MAHAY (43)				
Mali	PROMIS (46)				
Mali	PROMIS CS (46)				
Zimbabwe	SHINE (HIV−) (47)	1871	1783	−0.01 (−0.03, 0.00)	0.38
Zimbabwe	SHINE (HIV+) (48)	337	329	0.00 (−0.05, 0.04)	0.08
**I² = 0.21, Tau² = 0.00**		**5332**	**8103**	**−0.01 (−0.02, 0.00)**	
**Frequency of contact − Weekly**					
Bangladesh	JiVitA−4 (34)	2885	1267	0.00 (−0.03, 0.02)	0.29
Bangladesh	WASH−B (36)	1163	3463	−0.03 (−0.06, 0.00)	0.19
Burkina Faso	iLiNS−Zinc (37)	1946	664	−0.04 (−0.08, −0.01)	0.18
Ghana	GHANA (39)	98	96	−0.04 (−0.10, 0.01)	0.07
Ghana	iLiNS−DYAD−G (40)	347	692	−0.02 (−0.06, 0.03)	0.10
Malawi	iLiNS−DYAD−M (44)	220	444	−0.01 (−0.06, 0.04)	0.09
Malawi	iLiNS−DOSE (45)	692	238	−0.04 (−0.10, 0.01)	0.07
**I² = 0.00, Tau² = 0.00**		**7351**	**6864**	**−0.02 (−0.04, −0.01)**	

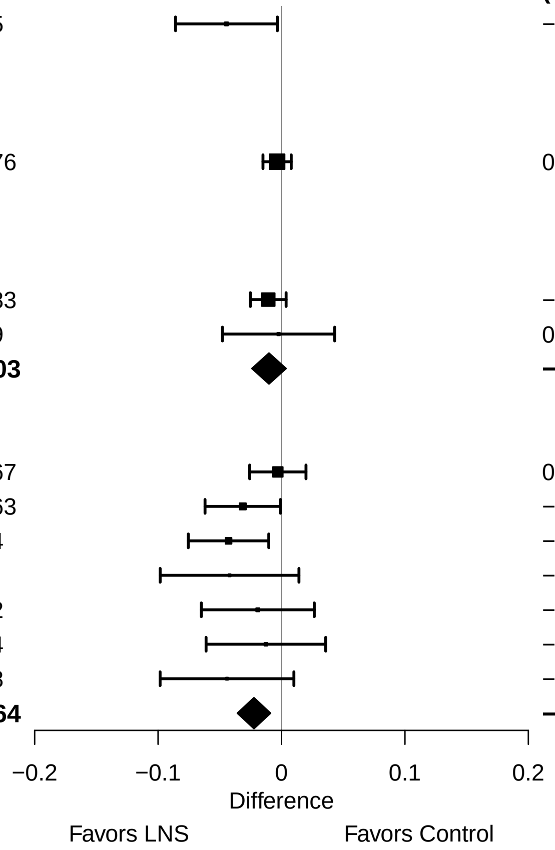

Supplemental figure 6Q: Small head size prevalence difference

6Q8: Stratified by Average SQ-LNS compliance

Average SQ−LNS compliance
(p−diff = 0.682)

**Average SQ−LNS compliance − Low**				**PD**	
**Country**	**Trial**	**N**	**N**	**(95% CI)**	**W**
Burkina Faso	PROMIS (38)				
Burkina Faso	PROMIS CS (38)				
Ghana	iLiNS−DYAD−G (40)	347	692	−0.02 (−0.06, 0.03)	0.12
Malawi	iLiNS−DYAD−M (44)	220	444	−0.01 (−0.06, 0.04)	0.11
Malawi	iLiNS−DOSE (45)	692	238	−0.04 (−0.10, 0.01)	0.09
Mali	PROMIS (46)				
Mali	PROMIS CS (46)				
Zimbabwe	SHINE (HIV−) (47)	1871	1783	−0.01 (−0.03, 0.00)	0.56
Zimbabwe	SHINE (HIV+) (48)	337	329	0.00 (−0.05, 0.04)	0.12
**I² = 0.00, Tau² = 0.00**		**3467**	**3486**	**−0.01 (−0.03, 0.00)**	
**Average SQ−LNS compliance − High**					
Bangladesh	JiVitA−4 (34)	2885	1267	0.00 (−0.03, 0.02)	0.21
Bangladesh	RDNS (35)	1663	815	−0.04 (−0.09, 0.00)	0.09
Bangladesh	WASH−B (36)	1163	3463	−0.03 (−0.06, 0.00)	0.14
Burkina Faso	iLiNS−Zinc (37)	1946	664	−0.04 (−0.08, −0.01)	0.13
Ghana	GHANA (39)	98	96	−0.04 (−0.10, 0.01)	0.05
Haiti	HAITI (41)				
Kenya	WASH−B (42)	1461	5176	0.00 (−0.02, 0.01)	0.39
**I² = 0.55, Tau² = 0.00**		**9216**	**11481**	**−0.02 (−0.04, 0.00)**	

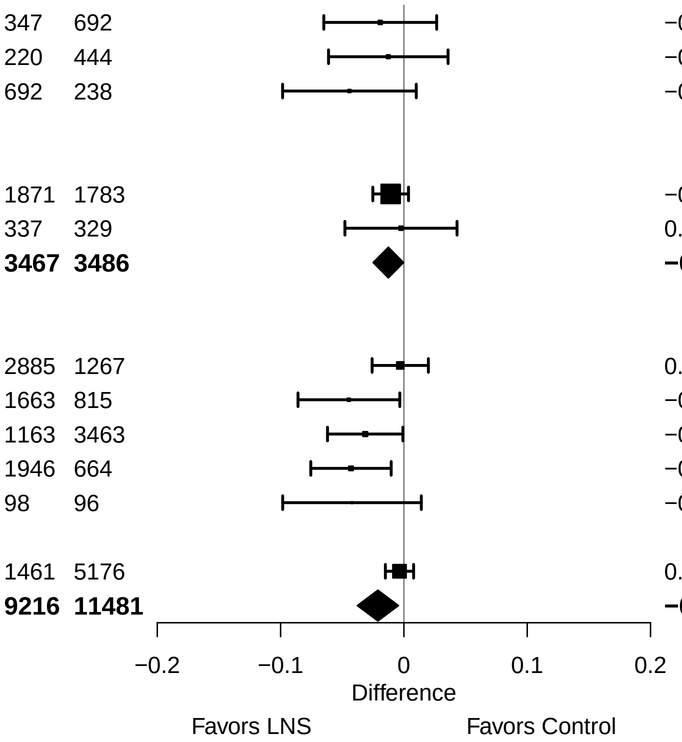

Supplement: nqab278_Supplemental_Files [file nqab278_supplemental_files.zip › 10_SQ-LNS_IPD_growth_Supplemental_Figure_6.pdf]
