# Supplementary material for: Characteristics that modify the effect of small-quantity lipid-based nutrient supplementation on child growth: an individual participant data meta-analysis of randomized controlled trials
Source: Am J Clin Nutr. 2021 Sep 29;114(Suppl 1):15S–42S. doi: 10.1093/ajcn/nqab278 (PMC8560308; doi:10.1093/ajcn/nqab278)

Supplemental figure 7: Pooled effect of SQ-LNS on prevalence ratios for low MUAC, acute malnutrition, underweight, and small head size stratified by study-level and individual-level characteristics

Contents

Supplemental figure 7A: Low MUAC prevalence ratio **2**
7A1: Stratified by study-level characteristics 2
7A2: Stratified by individual-level maternal and child characteristics 3
7A3: Stratified by individual-level household characteristics 4

Supplemental figure 7B: Acute malnutrition prevalence ratio **5**
7B1: Stratified by study-level characteristics 5
7B2: Stratified by individual-level maternal and child characteristics 6
7B3: Stratified by individual-level household characteristics 7

Supplemental figure 7C: Underweight prevalence ratio **8**
7C1: Stratified by study-level characteristics 8
7C2: Stratified by individual-level maternal and child characteristics 9
7C3: Stratified by individual-level household characteristics 10

Supplemental figure 7D: Small head size prevalence ratio **11**
7D1: Stratified by study-level characteristics 11
7D2: Stratified by individual-level maternal and child characteristics 12
7D3: Stratified by individual-level household characteristics 13

These figures show pooled effects of SQ-LNS within study-level and individual-level characteristic subgroups along with the p-for-interaction or p-diff. For definitions of effect modifiers, see Box 1 in the main paper. Individual study estimates were generated from log-binomial regression for dichotomous outcomes and simple linear regression for continuous outcomes; controlling for baseline measure when available and with clustered observations using robust standard errors for cluster-randomized trials. Pooled sub-group estimates and statistical testing of the pooled interaction term were generated using inverse-variance weighting. P-value for the difference was estimated using random effects meta-regression with the indicated effect modifier as the predictor of intervention effect size; stratified pooled estimates are presented for each strata. For dichotomous outcomes analyzed via prevalence ratios, the effect estimate is the prevalence in the LNS group divided by the prevalence in the control group. For dichotomous outcomes analyzed via prevalence differences, the effect estimate is the prevalence in the LNS group minus the prevalence in the control group.
The labels on the left y-axis correspond to the characteristic subgroups and their sample sizes. The values on the right indicate the pooled prevalence ratio and confidence interval within that subgroup.
LAZ, length-for-age z-score; WLZ, weight-for-length z-score; WAZ, weight-for-age z-score; MUACZ, mid-upper arm circumference z-score; HCZ, head circumference-for-age z-score.

Supplemental figure 7A: Low MUAC prevalence ratio

7A1: Stratified by study-level characteristics

Effect modifier (p−diff)	LNS N	Control N	PR (95% CI)
Stunting burden (p=0.925)			
Less than 35%	3306	7670	0.82 (0.69, 0.98)
More than 35%	12565	8233	0.82 (0.70, 0.96)
Malaria prevalence (p=0.684)			
At least 10%	5961	4866	0.79 (0.68, 0.92)
Less than 10%	9910	11037	0.84 (0.73, 0.98)
Source water quality (p=0.367)			
Improved	5823	3463	0.89 (0.76, 1.05)
Unimproved	8111	8434	0.80 (0.70, 0.92)
Sanitation (p=0.291)			
Improved	7646	5602	0.88 (0.78, 0.99)
Unimproved	6288	6295	0.77 (0.65, 0.93)
Supplement duration (p=0.928)			
12 mo or less	11286	8428	0.82 (0.72, 0.93)
> 12 mo	4585	7475	0.82 (0.67, 1.00)
Frequency of contact (p=0.354)			
Weekly	6119	3314	0.77 (0.64, 0.91)
Monthly	9752	12589	0.86 (0.76, 0.97)
Average SQ−LNS compliance (p=0.377)			
High	7980	7937	0.77 (0.64, 0.93)
Low	6211	6308	0.86 (0.73, 1.01)

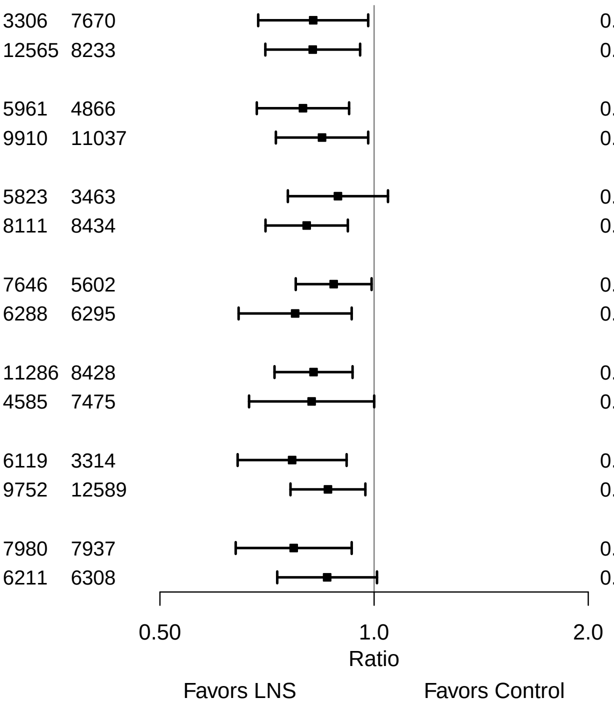

Supplemental figure 7A: Low MUAC prevalence ratio

7A2: Stratified by individual-level maternal and child characteristics

Effect modifier (p−for−interaction)	LNS N	Control N	PR (95% CI)
Maternal BMI (p=0.337)			
At least 20 kg/m²	5015	6556	0.76 (0.65, 0.89)
Less than 20 kg/m²	2643	2629	0.84 (0.71, 0.99)
Maternal age (p=0.741)			
At least 25 y	6964	7323	0.80 (0.70, 0.91)
Less than 25 y	7068	6639	0.84 (0.74, 0.95)
Maternal education (p=0.240)			
Primary or greater	4299	4381	0.78 (0.66, 0.92)
Incomplete or no formal	4773	5946	0.92 (0.81, 1.04)
Maternal depressive symptoms (p=0.798)			
At least 75th percentile	1875	2484	0.85 (0.64, 1.13)
Less than 75th percentile	4430	6410	0.85 (0.71, 1.02)
Child sex (p=0.002)			
Male	7368	7357	0.93 (0.82, 1.05)
Female	7250	7534	0.73 (0.65, 0.82)
Child birth order (p=0.002)			
Later born	7377	8512	0.77 (0.69, 0.86)
Firstborn	4490	3376	0.95 (0.81, 1.11)

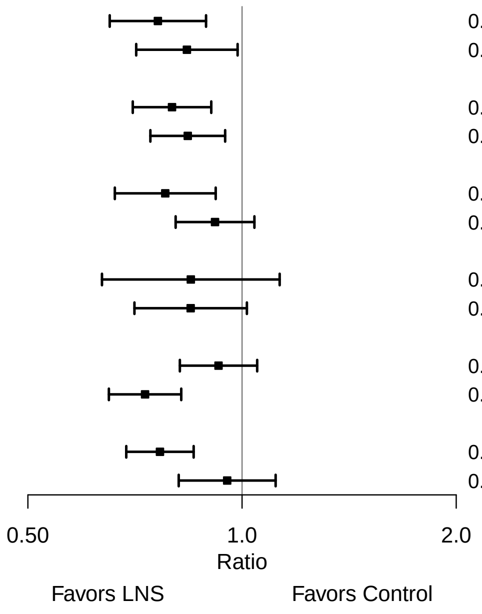

Supplemental figure 7A: Low MUAC prevalence ratio

7A3: Stratified by individual-level household characteristics

Effect modifier (p−for−interaction)	LNS N	Control N	PR (95% CI)
Socio−economic status (p=0.129)			
At least median	6492	6561	0.75 (0.66, 0.85)
Less than median	5831	5800	0.86 (0.76, 0.96)
Food security (p=0.182)			
Mild to secure	7226	8131	0.74 (0.66, 0.83)
Moderate to severe	3423	2516	0.87 (0.74, 1.04)
Source Water quality (p=0.130)			
Improved	2846	3621	0.88 (0.74, 1.03)
Unimproved	3946	3535	0.73 (0.63, 0.84)
Sanitation (p=0.239)			
Improved	4794	3320	0.84 (0.72, 0.97)
Unimproved	2660	3317	0.94 (0.78, 1.13)
Home environment (p=0.543)			
At least median	3410	4452	0.76 (0.63, 0.92)
Less than median	2088	3353	0.89 (0.72, 1.12)
Season at time of assessment (p=0.301)			
Dry	4704	3306	0.81 (0.70, 0.95)
Rainy	4554	5979	0.78 (0.68, 0.90)

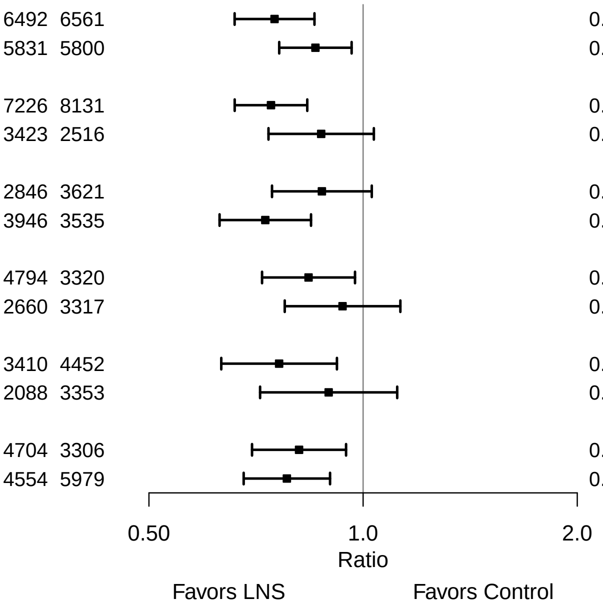

Supplemental figure 7B: Acute malnutrition prevalence ratio

7B1: Stratified by study-level characteristics

**Effect modifier (p−diff)**	**LNS N**	**Control N**	**PR (95% CI)**
Stunting burden (p=0.835)			
Less than 35%	3291	7588	0.85 (0.72, 1.00)
More than 35%	12406	8155	0.87 (0.77, 0.98)
Malaria prevalence (p=0.526)			
At least 10%	5921	4836	0.85 (0.74, 0.97)
Less than 10%	9776	10907	0.88 (0.80, 0.96)
Source water quality (p=0.378)			
Improved	5701	3407	0.89 (0.81, 0.99)
Unimproved	8066	8376	0.84 (0.75, 0.93)
Sanitation (p=0.450)			
Improved	7504	5519	0.88 (0.81, 0.96)
Unimproved	6263	6264	0.85 (0.70, 1.02)
Supplement duration (p=0.607)			
12 mo or less	11139	8350	0.87 (0.79, 0.97)
> 12 mo	4558	7393	0.84 (0.73, 0.96)
Frequency of contact (p=0.706)			
Weekly	5991	3256	0.86 (0.69, 1.06)
Monthly	9706	12487	0.87 (0.79, 0.97)
Average SQ−LNS compliance (p=0.269)			
High	7849	7812	0.81 (0.71, 0.92)
Low	6173	6276	0.90 (0.79, 1.02)

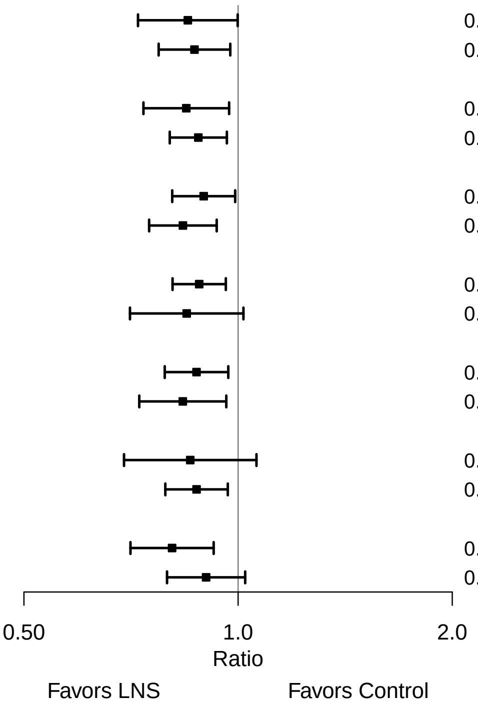

Supplemental figure 7B: Acute malnutrition prevalence ratio

7B2: Stratified by individual-level maternal and child characteristics

Effect modifier (p−for−interaction)	LNS N	Control N	PR (95% CI)
Maternal height (p=0.435)			
At least 150.1 cm	3335	2574	0.93 (0.80, 1.09)
Less than 150.1 cm	1471	983	0.88 (0.70, 1.09)
Maternal BMI (p=0.490)			
At least 20 kg/m²	5380	3795	0.82 (0.70, 0.95)
Less than 20 kg/m²	2655	1715	0.84 (0.74, 0.94)
Maternal age (p=0.429)			
At least 25 y	6543	6888	0.83 (0.74, 0.92)
Less than 25 y	6824	6414	0.89 (0.80, 0.99)
Maternal education (p=0.659)			
Primary or greater	4492	4857	0.87 (0.77, 0.98)
Incomplete or no formal	4789	6023	0.92 (0.82, 1.02)
Maternal depressive symptoms (p=0.532)			
At least 75th percentile	2035	2770	0.81 (0.65, 1.00)
Less than 75th percentile	4903	7032	0.88 (0.76, 1.03)
Child sex (p=0.307)			
Male	7013	7016	0.88 (0.79, 0.97)
Female	6939	7214	0.84 (0.75, 0.92)
Child birth order (p=0.124)			
Later born	8620	9626	0.80 (0.73, 0.88)
Firstborn	4873	3805	0.93 (0.83, 1.04)

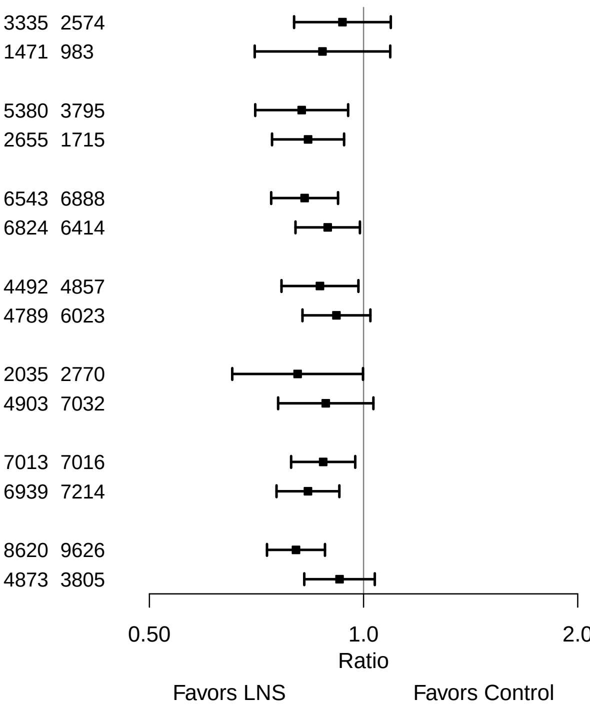

Supplemental figure 7B: Acute malnutrition prevalence ratio

7B3: Stratified by individual-level household characteristics

**Effect modifier (p−for−interaction)**	**LNS N**	**Control N**	**PR (95% CI)**
Socio−economic status (p=0.312)			
At least median	7393	7535	0.82 (0.73, 0.91)
Less than median	6684	6835	0.89 (0.81, 0.98)
Food security (p=0.565)			
Mild to secure	7131	4863	0.89 (0.80, 0.99)
Moderate to severe	3440	2345	0.78 (0.69, 0.88)
Source Water quality (p=0.789)			
Improved	2651	2273	0.83 (0.70, 0.97)
Unimproved	3708	2874	0.83 (0.72, 0.96)
Sanitation (p=0.013)			
Improved	4882	3145	0.82 (0.73, 0.91)
Unimproved	2528	1931	1.01 (0.88, 1.16)
Home environment (p=0.310)			
At least median	3614	4835	0.73 (0.63, 0.85)
Less than median	2201	3567	0.91 (0.75, 1.10)
Season at time of assessment (p=0.172)			
Dry	5052	3395	0.79 (0.70, 0.89)
Rainy	4757	5992	0.89 (0.80, 1.00)

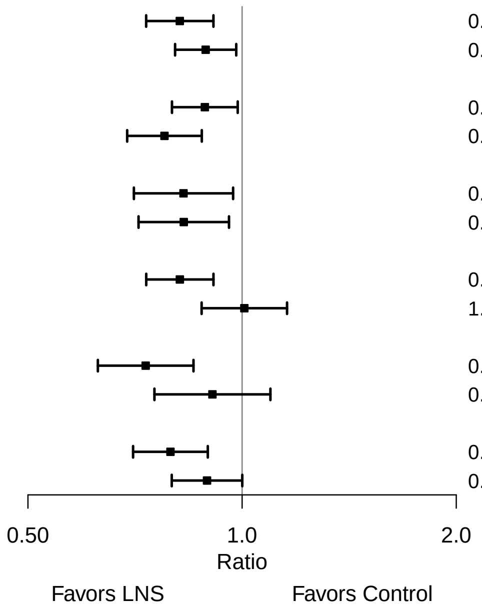

Supplemental figure 7C: Underweight prevalence ratio

7C1: Stratified by study-level characteristics

**Effect modifier (p−diff)**	**LNS N**	**Control N**	**PR (95% CI)**
Geographic region (p=0.909)			
AFR	11428	13913	0.86 (0.79, 0.94)
SEAR	5640	5508	0.87 (0.79, 0.96)
Stunting burden (p=0.979)			
Less than 35%	3561	7895	0.86 (0.77, 0.96)
More than 35%	13655	11676	0.87 (0.79, 0.95)
Malaria prevalence (p=0.998)			
At least 10%	6060	4949	0.86 (0.78, 0.95)
Less than 10%	11156	14622	0.87 (0.79, 0.95)
Source water quality (p=0.601)			
Improved	6564	4790	0.91 (0.86, 0.97)
Unimproved	8126	8443	0.88 (0.80, 0.96)
Sanitation (p=0.873)			
Improved	8375	6914	0.90 (0.85, 0.95)
Unimproved	6315	6319	0.91 (0.80, 1.04)
Supplement duration (p=0.519)			
12 mo or less	11483	8668	0.88 (0.81, 0.97)
> 12 mo	5733	10903	0.85 (0.78, 0.91)
Frequency of contact (p=0.516)			
Weekly	7304	6833	0.84 (0.75, 0.94)
Monthly	9912	12738	0.89 (0.82, 0.96)
Average SQ−LNS compliance (p=0.272)			
High	9307	11585	0.82 (0.76, 0.90)
Low	6207	6301	0.88 (0.81, 0.97)

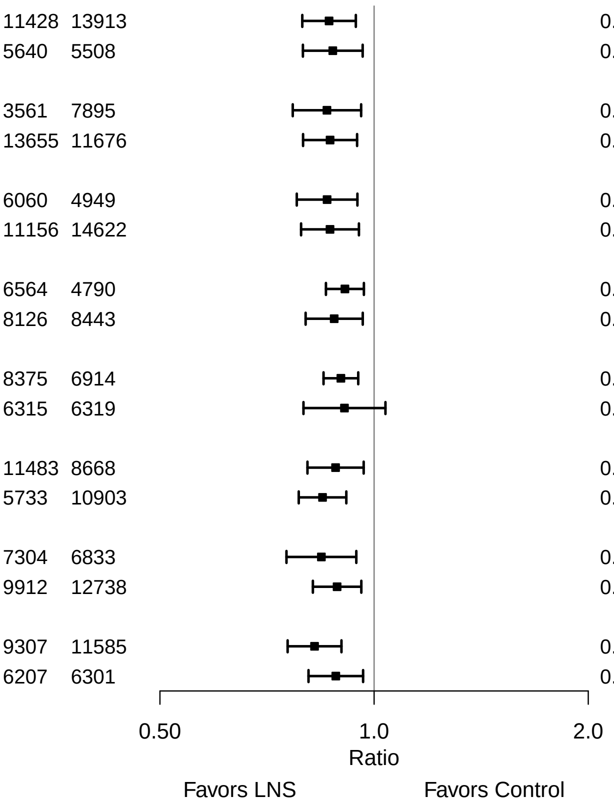

Supplemental figure 7C: Underweight prevalence ratio

7C2: Stratified by individual-level maternal and child characteristics

**Effect modifier (p−for−interaction)**	**LNS N**	**Control N**	**PR (95% CI)**
Maternal height (p=0.546)			
At least 150.1 cm	9992	12808	0.82 (0.77, 0.88)
Less than 150.1 cm	2242	2905	0.89 (0.82, 0.96)
Maternal BMI (p=0.545)			
At least 20 kg/m²	7782	10117	0.83 (0.76, 0.90)
Less than 20 kg/m²	3854	5024	0.85 (0.80, 0.91)
Maternal age (p=0.245)			
At least 25 y	8357	9838	0.85 (0.80, 0.91)
Less than 25 y	8335	9208	0.89 (0.85, 0.94)
Maternal education (p=0.399)			
Primary or greater	7121	9070	0.85 (0.79, 0.90)
Incomplete or no formal	6029	7977	0.91 (0.85, 0.97)
Maternal depressive symptoms (p=0.490)			
At least 75th percentile	2454	3925	0.90 (0.81, 1.01)
Less than 75th percentile	6364	10243	0.87 (0.81, 0.93)
Child sex (p=0.617)			
Male	8523	9577	0.87 (0.82, 0.92)
Female	8447	9748	0.86 (0.81, 0.92)
Child birth order (p=0.001)			
Later born	10986	13686	0.83 (0.79, 0.88)
Firstborn	5779	5491	0.94 (0.88, 1.00)
Child baseline anthropometric status (p=0.493)			
WAZ at least −1 Z	4114	2898	0.83 (0.65, 1.05)
WAZ less than −1 Z	3585	2144	0.92 (0.87, 0.96)

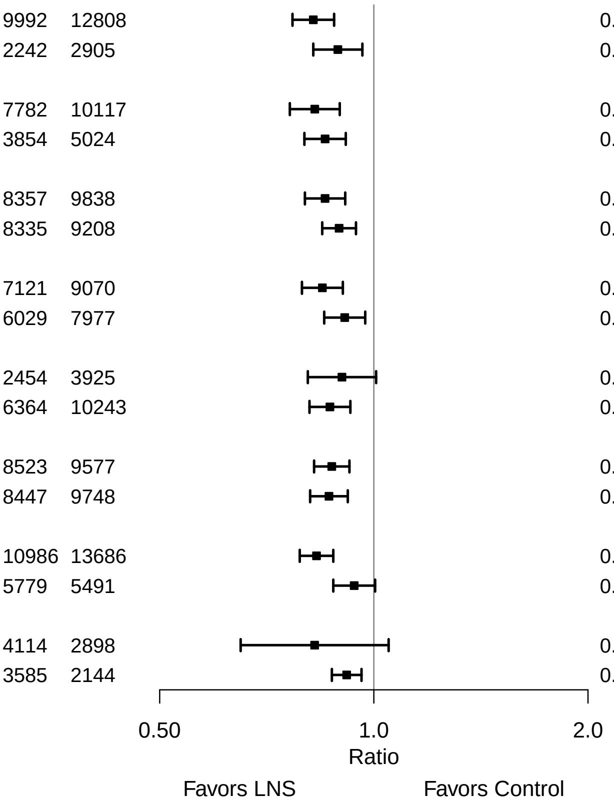

Supplemental figure 7C: Underweight prevalence ratio

7C3: Stratified by individual-level household characteristics

**Effect modifier (p−for−interaction)**	**LNS N**	**Control N**	**PR (95% CI)**
Socio−economic status (p=0.877)			
At least median	8704	9937	0.86 (0.81, 0.92)
Less than median	7946	9160	0.87 (0.82, 0.92)
Food security (p=0.661)			
Mild to secure	10207	12969	0.85 (0.81, 0.90)
Moderate to severe	4692	4369	0.85 (0.79, 0.92)
Source Water quality (p=0.762)			
Improved	3949	5191	0.87 (0.79, 0.95)
Unimproved	4360	4017	0.85 (0.78, 0.93)
Sanitation (p=0.840)			
Improved	5298	3530	0.91 (0.85, 0.98)
Unimproved	2657	2077	0.92 (0.84, 1.01)
Home environment (p=0.416)			
At least median	4790	6967	0.88 (0.81, 0.95)
Less than median	3068	5535	0.92 (0.84, 1.00)
Season at time of assessment (p=0.102)			
Dry	7835	7737	0.81 (0.76, 0.87)
Rainy	5651	8113	0.88 (0.83, 0.94)

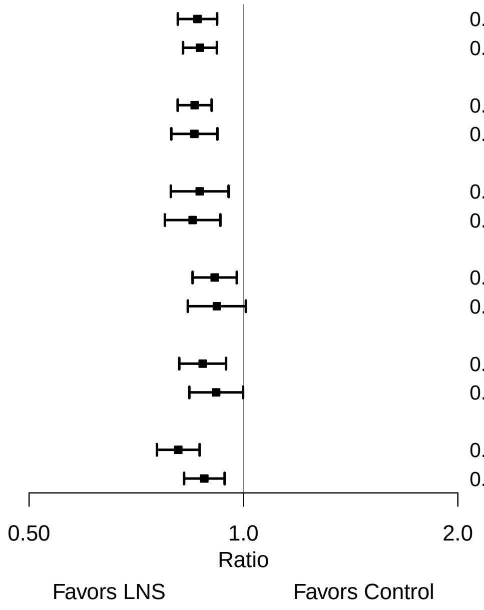

Supplemental figure 7D: Small head size prevalence ratio

7D1: Stratified by study-level characteristics

**Effect modifier (p−diff)**	**LNS N**	**Control N**	**PR (95% CI)**
Geographic region (p=0.271)			
AFR	6972	9422	0.87 (0.79, 0.95)
SEAR	5711	5545	0.93 (0.86, 1.01)
Stunting burden (p=0.780)			
Less than 35%	2126	6408	0.88 (0.72, 1.07)
More than 35%	10557	8559	0.91 (0.86, 0.96)
Malaria prevalence (p=0.351)			
At least 10%	3303	2134	0.87 (0.78, 0.96)
Less than 10%	9380	12833	0.92 (0.87, 0.97)
Source water quality (p=0.958)			
Improved	6476	4673	0.91 (0.85, 0.98)
Unimproved	3676	3949	0.90 (0.80, 1.01)
Sanitation (p=0.549)			
Improved	5564	3991	0.92 (0.84, 1.01)
Unimproved	4588	4631	0.88 (0.80, 0.98)
Supplement duration (p=0.749)			
12 mo or less	8396	5513	0.92 (0.85, 0.99)
> 12 mo	4287	9454	0.90 (0.84, 0.96)
Frequency of contact (p=0.591)			
Weekly	7351	6864	0.92 (0.86, 0.98)
Monthly	5332	8103	0.89 (0.82, 0.96)
Average SQ−LNS compliance (p=0.264)			
High	9216	11481	0.92 (0.87, 0.97)
Low	3467	3486	0.84 (0.73, 0.97)

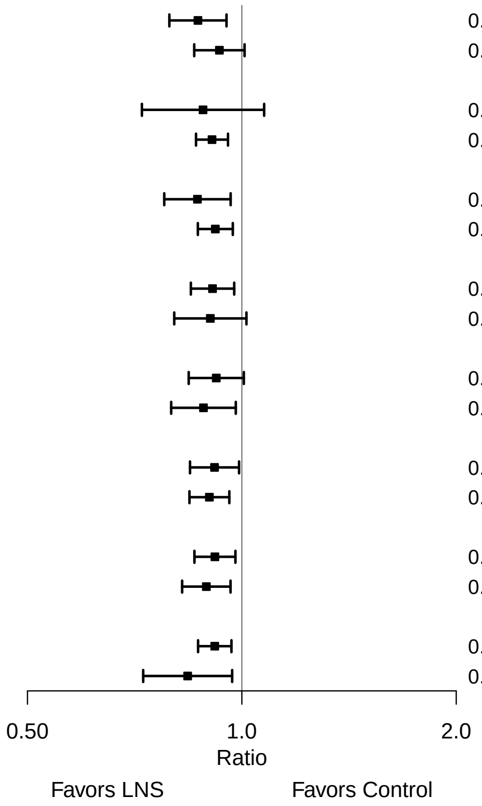

Supplemental figure 7D: Small head size prevalence ratio

7D2: Stratified by individual-level maternal and child characteristics

Effect modifier (p−for−interaction)	LNS N	Control N	PR (95% CI)
Maternal height (p=0.420)			
At least 150.1 cm	7041	9582	0.89 (0.83, 0.96)
Less than 150.1 cm	1556	2230	0.87 (0.80, 0.94)
Maternal BMI (p=0.607)			
At least 20 kg/m²	5865	8445	0.89 (0.83, 0.96)
Less than 20 kg/m²	3066	4285	0.87 (0.81, 0.94)
Maternal age (p=0.907)			
At least 25 y	5963	7331	0.91 (0.84, 0.99)
Less than 25 y	6345	7261	0.92 (0.86, 0.98)
Maternal education (p=0.091)			
Primary or greater	5132	6916	0.88 (0.82, 0.95)
Incomplete or no formal	4996	5382	0.94 (0.87, 1.02)
Maternal depressive symptoms (p=0.095)			
At least 75th percentile	1827	3333	0.84 (0.75, 0.95)
Less than 75th percentile	4816	8588	0.94 (0.87, 1.01)
Child sex (p=0.020)			
Male	6321	7278	0.96 (0.90, 1.03)
Female	6264	7593	0.85 (0.79, 0.92)
Child birth order (p=0.696)			
Later born	7141	9861	0.89 (0.83, 0.95)
Firstborn	4925	4553	0.93 (0.86, 1.01)
Child baseline anthropometric status (p=0.152)			
HCZ at least −1 Z	4324	2696	0.73 (0.55, 0.97)
HCZ less than −1 Z	4720	2315	0.92 (0.87, 0.97)

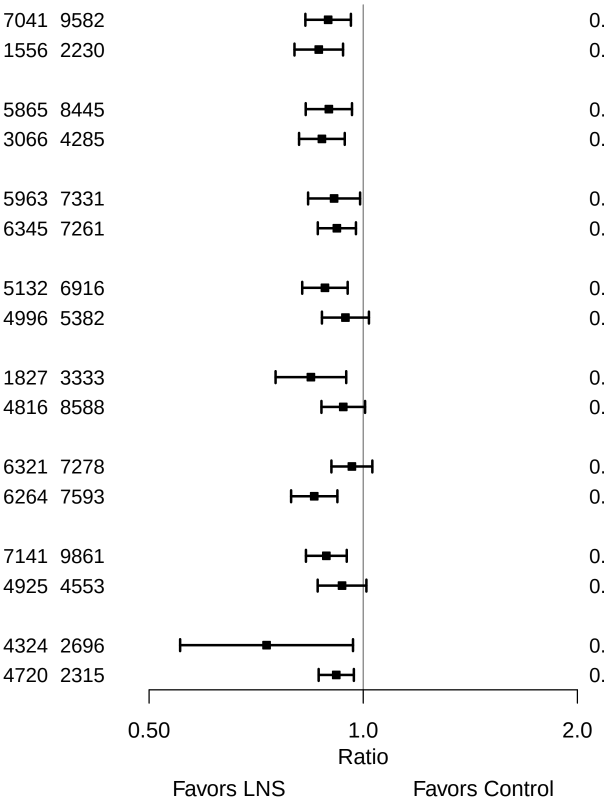

Supplemental figure 7D: Small head size prevalence ratio

7D3: Stratified by individual-level household characteristics

Effect modifier (p−for−interaction)	LNS N	Control N	PR (95% CI)
Socio−economic status (p=0.462)			
At least median	6582	7678	0.89 (0.83, 0.96)
Less than median	5745	7014	0.93 (0.88, 1.00)
Food security (p=0.542)			
Mild to secure	8420	11192	0.90 (0.85, 0.95)
Moderate to severe	3834	3435	0.94 (0.87, 1.02)
Source Water quality (p=0.505)			
Improved	2571	3382	0.88 (0.77, 1.00)
Unimproved	2027	1648	0.86 (0.77, 0.96)
Sanitation (p=0.109)			
Improved	4256	2821	0.90 (0.83, 0.97)
Unimproved	3555	1775	0.96 (0.87, 1.05)
Home environment (p=0.826)			
At least median	3870	6058	0.90 (0.84, 0.98)
Less than median	2275	4766	0.90 (0.84, 0.97)
Season at time of assessment (p=0.434)			
Dry	5463	5464	0.90 (0.84, 0.96)
Rainy	4914	7295	0.93 (0.86, 1.00)

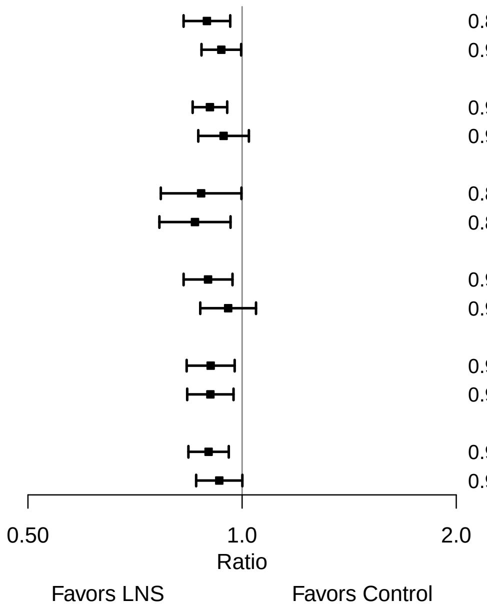

Supplement: nqab278_Supplemental_Files [file nqab278_supplemental_files.zip › 11_SQ-LNS_IPD_growth_Supplemental_Figure_7.pdf]
