# Supplementary material for: Characteristics that modify the effect of small-quantity lipid-based nutrient supplementation on child growth: an individual participant data meta-analysis of randomized controlled trials
Source: Am J Clin Nutr. 2021 Sep 29;114(Suppl 1):15S–42S. doi: 10.1093/ajcn/nqab278 (PMC8560308; doi:10.1093/ajcn/nqab278)

Supplemental figure 8: Forest plots for effects of SQ-LNS on growth outcomes stratified by individual-level maternal and child effect modifiers

Contents

Supplemental figure 8A: Mean difference in LAZ **5**
8A1: Stratified by Maternal height . . . 5
8A2: Stratified by Maternal BMI . . . 6
8A3: Stratified by Maternal age . . . 7
8A4: Stratified by Maternal education . . . 8
8A5: Stratified by Maternal depressive symptoms . . . 9
8A6: Stratified by Child sex . . . 10
8A7: Stratified by Child birth order . . . 11
8A8: Stratified by Child baseline anthropometric status . . . 12

Supplemental figure 8B: Stunting prevalence ratio **13**
8B1: Stratified by Maternal height . . . 13
8B2: Stratified by Maternal BMI . . . 14
8B3: Stratified by Maternal age . . . 15
8B4: Stratified by Maternal education . . . 16
8B5: Stratified by Maternal depressive symptoms . . . 17
8B6: Stratified by Child sex . . . 18
8B7: Stratified by Child birth order . . . 19
8B8: Stratified by Child baseline anthropometric status . . . 20

Supplemental figure 8C: Stunting prevalence difference **21**
8C1: Stratified by Maternal height . . . 21
8C2: Stratified by Maternal BMI . . . 22
8C3: Stratified by Maternal age . . . 23
8C4: Stratified by Maternal education . . . 24
8C5: Stratified by Maternal depressive symptoms . . . 25
8C6: Stratified by Child sex . . . 26
8C7: Stratified by Child birth order . . . 27
8C8: Stratified by Child baseline anthropometric status . . . 28

Supplemental figure 8D: Mean difference in WLZ **29**
8D1: Stratified by Maternal height . . . 29
8D2: Stratified by Maternal BMI . . . 30
8D3: Stratified by Maternal age . . . 31
8D4: Stratified by Maternal education . . . 32
8D5: Stratified by Maternal depressive symptoms . . . 33
8D6: Stratified by Child sex . . . 34
8D7: Stratified by Child birth order . . . 35
8D8: Stratified by Child baseline anthropometric status . . . 36

Supplemental figure 8E: Wasting prevalence ratio **37**

8E1: Stratified by Maternal height . . . 37
8E2: Stratified by Maternal BMI . . . 38
8E3: Stratified by Maternal age . . . 39
8E4: Stratified by Maternal education . . . 40
8E5: Stratified by Maternal depressive symptoms . . . 41
8E6: Stratified by Child sex . . . 42
8E7: Stratified by Child birth order . . . 43
8E8: Stratified by Child baseline anthropometric status . . . 44

Supplemental figure 8F: Wasting prevalence difference **45**
8F1: Stratified by Maternal height . . . 45
8F2: Stratified by Maternal BMI . . . 46
8F3: Stratified by Maternal age . . . 47
8F4: Stratified by Maternal education . . . 48
8F5: Stratified by Maternal depressive symptoms . . . 49
8F6: Stratified by Child sex . . . 50
8F7: Stratified by Child birth order . . . 51
8F8: Stratified by Child baseline anthropometric status . . . 52

Supplemental figure 8G: Mean difference in MUACZ **53**
8G1: Stratified by Maternal height . . . 53
8G2: Stratified by Maternal BMI . . . 54
8G3: Stratified by Maternal age . . . 55
8G4: Stratified by Maternal education . . . 56
8G5: Stratified by Maternal depressive symptoms . . . 57
8G6: Stratified by Child sex . . . 58
8G7: Stratified by Child birth order . . . 59
8G8: Stratified by Child baseline anthropometric status . . . 60

Supplemental figure 8H: Low MUAC prevalence ratio **61**
8H1: Stratified by Maternal height (insufficient comparisons) . . . 61
8H2: Stratified by Maternal BMI . . . 62
8H3: Stratified by Maternal age . . . 63
8H4: Stratified by Maternal education . . . 64
8H5: Stratified by Maternal depressive symptoms . . . 65
8H6: Stratified by Child sex . . . 66
8H7: Stratified by Child birth order . . . 67
8H8: Stratified by Child baseline anthropometric status (insufficient comparisons) . . . 67

Supplemental figure 8I: Low MUAC prevalence difference **68**
8I1: Stratified by Maternal height (insufficient comparisons) . . . 68
8I2: Stratified by Maternal BMI . . . 69
8I3: Stratified by Maternal age . . . 70
8I4: Stratified by Maternal education . . . 71
8I5: Stratified by Maternal depressive symptoms . . . 72
8I6: Stratified by Child sex . . . 73
8I7: Stratified by Child birth order . . . 74
8I8: Stratified by Child baseline anthropometric status (insufficient comparisons) . . . 74

Supplemental figure 8J: Acute malnutrition prevalence ratio **75**
8J1: Stratified by Maternal height . . . 75

8J2: Stratified by Maternal BMI . . . 76
8J3: Stratified by Maternal age . . . 77
8J4: Stratified by Maternal education . . . 78
8J5: Stratified by Maternal depressive symptoms . . . 79
8J6: Stratified by Child sex . . . 80
8J7: Stratified by Child birth order . . . 81
8J8: Stratified by Child baseline anthropometric status . . . 82

Supplemental figure 8K: Acute malnutrition prevalence difference 83
8K1: Stratified by Maternal height . . . 83
8K2: Stratified by Maternal BMI . . . 84
8K3: Stratified by Maternal age . . . 85
8K4: Stratified by Maternal education . . . 86
8K5: Stratified by Maternal depressive symptoms . . . 87
8K6: Stratified by Child sex . . . 88
8K7: Stratified by Child birth order . . . 89
8K8: Stratified by Child baseline anthropometric status . . . 90

Supplemental figure 8L: Mean difference in WAZ 91
8L1: Stratified by Maternal height . . . 91
8L2: Stratified by Maternal BMI . . . 92
8L3: Stratified by Maternal age . . . 93
8L4: Stratified by Maternal education . . . 94
8L5: Stratified by Maternal depressive symptoms . . . 95
8L6: Stratified by Child sex . . . 96
8L7: Stratified by Child birth order . . . 97
8L8: Stratified by Child baseline anthropometric status . . . 98

Supplemental figure 8M: Underweight prevalence ratio 99
8M1: Stratified by Maternal height . . . 99
8M2: Stratified by Maternal BMI . . . 100
8M3: Stratified by Maternal age . . . 101
8M4: Stratified by Maternal education . . . 102
8M5: Stratified by Maternal depressive symptoms . . . 103
8M6: Stratified by Child sex . . . 104
8M7: Stratified by Child birth order . . . 105
8M8: Stratified by Child baseline anthropometric status . . . 106

Supplemental figure 8N: Underweight prevalence difference 107
8N1: Stratified by Maternal height . . . 107
8N2: Stratified by Maternal BMI . . . 108
8N3: Stratified by Maternal age . . . 109
8N4: Stratified by Maternal education . . . 110
8N5: Stratified by Maternal depressive symptoms . . . 111
8N6: Stratified by Child sex . . . 112
8N7: Stratified by Child birth order . . . 113
8N8: Stratified by Child baseline anthropometric status . . . 114

Supplemental figure 8O: Mean difference in HCZ 115
8O1: Stratified by Maternal height . . . 115
8O2: Stratified by Maternal BMI . . . 116

8O3: Stratified by Maternal age . . . 117
8O4: Stratified by Maternal education . . . 118
8O5: Stratified by Maternal depressive symptoms . . . 119
8O6: Stratified by Child sex . . . 120
8O7: Stratified by Child birth order . . . 121
8O8: Stratified by Child baseline anthropometric status . . . 122

Supplemental figure 8P: Small head size prevalence ratio **123**
8P1: Stratified by Maternal height . . . 123
8P2: Stratified by Maternal BMI . . . 124
8P3: Stratified by Maternal age . . . 125
8P4: Stratified by Maternal education . . . 126
8P5: Stratified by Maternal depressive symptoms . . . 127
8P6: Stratified by Child sex . . . 128
8P7: Stratified by Child birth order . . . 129
8P8: Stratified by Child baseline anthropometric status . . . 130

Supplemental figure 8Q: Small head size prevalence difference **131**
8Q1: Stratified by Maternal height . . . 131
8Q2: Stratified by Maternal BMI . . . 132
8Q3: Stratified by Maternal age . . . 133
8Q4: Stratified by Maternal education . . . 134
8Q5: Stratified by Maternal depressive symptoms . . . 135
8Q6: Stratified by Child sex . . . 136
8Q7: Stratified by Child birth order . . . 137
8Q8: Stratified by Child baseline anthropometric status . . . 138

These figures are forest plots showing the individual-level effect modification of intervention effects. Each figure has the estimates of intervention effect stratified within study by individual-level effect modifier category. For definitions of effect modifiers, see Box 1 in the main paper. Individual study estimates were generated from log-binomial regression for dichotomous outcomes and simple linear regression for continuous outcomes; controlling for baseline measure when available and with clustered observations using robust standard errors for cluster-randomized trials. Pooled interaction term and sub-group estimates were generated using inverse-variance weighting fixed and random effects. For continuous outcomes analyzed via mean differences, the effect estimate is the mean in the LNS group minus the mean in the control group. For dichotomous outcomes analyzed via prevalence ratios, the effect estimate is the prevalence in the LNS group divided by the prevalence in the control group. For dichotomous outcomes analyzed via prevalence differences, the effect estimate is the prevalence in the LNS group minus the prevalence in the control group.
The labels on the far left correspond to trial level information. In the middle left and on the right the values indicate the study level effect estimate, confidence interval, and weighting for deriving the pooled estimates is shown by subgroup. LAZ, length-for-age z-score; WLZ, weight-for-length z-score; WAZ, weight-for-age z-score; MUACZ, mid-upper arm circumference z-score; HCZ, head circumference-for-age z-score.

Supplemental figure 8A: Mean difference in LAZ

8A1: Stratified by Maternal height

P−for−interaction = 0.215
Difference in MDs = −0.04 (−0.11, 0.02)

Country	Trial	At least 150.1 cm: LNS N	Control N	Control Mean	MD (95% CI)	Fixed W	Random W	Less than 150.1 cm: LNS N	Control N	Control Mean	MD (95% CI)	Fixed W	Random W
Bangladesh	JiVitA−4 (34)												
Bangladesh	RDNS (35)	868	432	−1.55	0.12 (0.01, 0.22)	0.08	0.08	735	358	−2.12	0.06 (−0.05, 0.16)	0.26	0.26
Bangladesh	WASH−B (36)	616	1883	−1.56	0.24 (0.15, 0.33)	0.11	0.09	542	1545	−2.13	0.19 (0.11, 0.28)	0.44	0.40
Burkina Faso	iLiNS−Zinc (37)	1916	649	−1.74	0.31 (0.22, 0.39)	0.12	0.09	34	15	−2.62	0.35 (0.04, 0.66)	0.03	0.04
Burkina Faso	PROMIS (38)	835	884	−1.46	0.15 (0.02, 0.29)	0.05	0.07	25	25	−2.21	0.02 (−0.76, 0.80)	0.01	0.01
Burkina Faso	PROMIS CS (38)	419	426	−1.19	0.03 (−0.15, 0.22)	0.03	0.05	11	9	−2.53	0.26 (−0.71, 1.23)	0.00	0.00
Ghana	GHANA (39)												
Ghana	iLiNS−DYADG (40)	326	645	−0.86	0.20 (0.07, 0.33)	0.05	0.07	16	36	−1.51	0.10 (−0.46, 0.66)	0.01	0.01
Haiti	HAITI (41)												
Kenya	WASH−B (42)	1313	4686	−1.53	0.15 (0.08, 0.22)	0.19	0.10	57	190	−2.16	0.02 (−0.29, 0.33)	0.03	0.04
Madagascar	MAHAY (43)	1004	1037	−2.11	0.09 (−0.06, 0.24)	0.04	0.06	598	565	−2.54	0.00 (−0.20, 0.19)	0.08	0.09
Malawi	iLiNS−DYADM (44)	192	381	−1.53	−0.09 (−0.27, 0.09)	0.03	0.05	28	60	−2.21	−0.11 (−0.59, 0.37)	0.01	0.02
Malawi	iLiNS−DOSE (45)	580	198	−1.80	0.03 (−0.09, 0.15)	0.07	0.08	115	42	−2.55	0.22 (0.01, 0.43)	0.07	0.08
Mali	PROMIS (46)	481	496	−1.73	0.19 (0.04, 0.34)	0.04	0.06	19	9	−1.81	−0.31 (−0.86, 0.24)	0.01	0.01
Mali	PROMIS CS (46)	914	936	−1.55	0.24 (0.06, 0.41)	0.03	0.05	33	27	−2.39	0.12 (−0.36, 0.61)	0.01	0.01
Zimbabwe	SHINE (HIV−) (47)	1755	1697	−1.58	0.16 (0.09, 0.24)	0.15	0.10	76	62	−2.42	0.23 (−0.10, 0.56)	0.03	0.03
Zimbabwe	SHINE (HIV+) (48)	314	312	−1.96	0.23 (0.05, 0.41)	0.03	0.05	18	12	−2.81	0.63 (−0.30, 1.55)	0.00	0.00
		**11533**	**14662**		**I^2 = 0.58, Tau^2 = 0.01**			**2307**	**2955**		**I^2 = 0.06, Tau^2 = 0.00**		
**Fixed**					**0.17 (0.14, 0.20)**						**0.13 (0.08, 0.19)**		
**Random**					**0.16 (0.10, 0.21)**						**0.13 (0.07, 0.19)**		

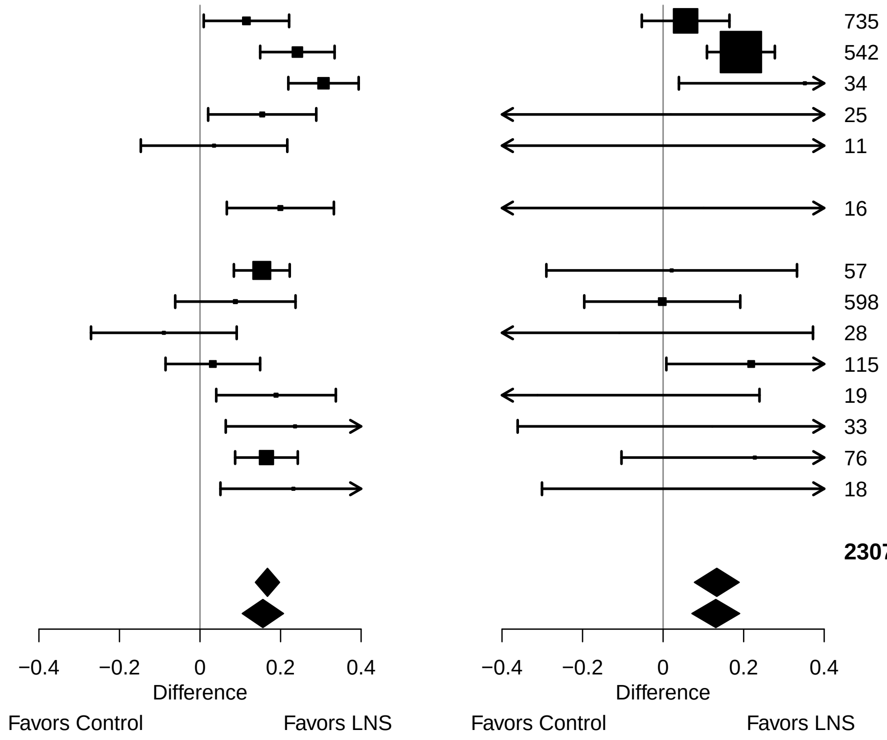

Supplemental figure 8A: Mean difference in LAZ

8A2: Stratified by Maternal BMI

P−for−interaction = 0.676
Difference in MDs = −0.01 (−0.06, 0.04)

Country	Trial	At least 20 kg/m² LNS N	Control N	Control Mean	MD (95% CI)	Fixed W	Random W	Less than 20 kg/m² LNS N	Control N	Control Mean	MD (95% CI)	Fixed W	Random W
Bangladesh	JiVitA−4 (34)												
Bangladesh	RDNS (35)	730	335	−1.69	0.10 (−0.04, 0.23)	0.07	0.08	873	455	−1.90	0.06 (−0.03, 0.16)	0.18	0.11
Bangladesh	WASH−B (36)	519	1576	−1.69	0.21 (0.09, 0.33)	0.08	0.09	639	1852	−1.92	0.21 (0.13, 0.29)	0.25	0.12
Burkina Faso	iLiNS−Zinc (37)	1214	390	−1.65	0.30 (0.21, 0.40)	0.13	0.11	736	274	−1.91	0.32 (0.20, 0.43)	0.14	0.11
Burkina Faso	PROMIS (38)	647	675	−1.47	0.22 (0.10, 0.34)	0.08	0.09	213	233	−1.52	−0.07 (−0.30, 0.16)	0.03	0.06
Burkina Faso	PROMIS CS (38)	246	269	−1.09	0.04 (−0.15, 0.23)	0.03	0.05	184	166	−1.42	0.06 (−0.19, 0.32)	0.03	0.06
Ghana	GHANA (39)												
Ghana	iLiNS−DYADG (40)	305	562	−0.87	0.17 (0.03, 0.31)	0.06	0.07	37	119	−1.01	0.31 (−0.05, 0.68)	0.01	0.04
Haiti	HAITI (41)												
Kenya	WASH−B (42)	1098	3803	−1.52	0.11 (0.03, 0.18)	0.20	0.13	272	1073	−1.68	0.29 (0.16, 0.42)	0.10	0.10
Madagascar	MAHAY (43)												
Malawi	iLiNS−DYADM (44)	131	263	−1.61	−0.01 (−0.23, 0.22)	0.02	0.04	88	178	−1.63	−0.20 (−0.47, 0.08)	0.02	0.05
Malawi	iLiNS−DOSE (45)	513	174	−1.88	0.06 (−0.06, 0.18)	0.08	0.09	180	65	−2.09	0.08 (−0.12, 0.28)	0.04	0.07
Mali	PROMIS (46)	380	370	−1.68	0.20 (0.02, 0.38)	0.04	0.05	120	135	−1.85	0.09 (−0.06, 0.23)	0.08	0.09
Mali	PROMIS CS (46)	702	684	−1.50	0.20 (0.01, 0.38)	0.03	0.05	245	279	−1.75	0.28 (0.09, 0.47)	0.05	0.08
Zimbabwe	SHINE (HIV−) (47)	1364	1305	−1.62	0.20 (0.11, 0.28)	0.16	0.12	240	249	−1.67	0.03 (−0.16, 0.22)	0.05	0.08
Zimbabwe	SHINE (HIV+) (48)	247	254	−1.96	0.27 (0.06, 0.47)	0.03	0.04	62	52	−2.17	0.21 (−0.19, 0.61)	0.01	0.03
		**8096**	**10660**		**I² = 0.44, Tau² = 0.00**			**3889**	**5130**		**I² = 0.64, Tau² = 0.01**		
**Fixed**					**0.17 (0.14, 0.20)**						**0.16 (0.12, 0.21)**		
**Random**					**0.17 (0.12, 0.21)**						**0.14 (0.06, 0.22)**		

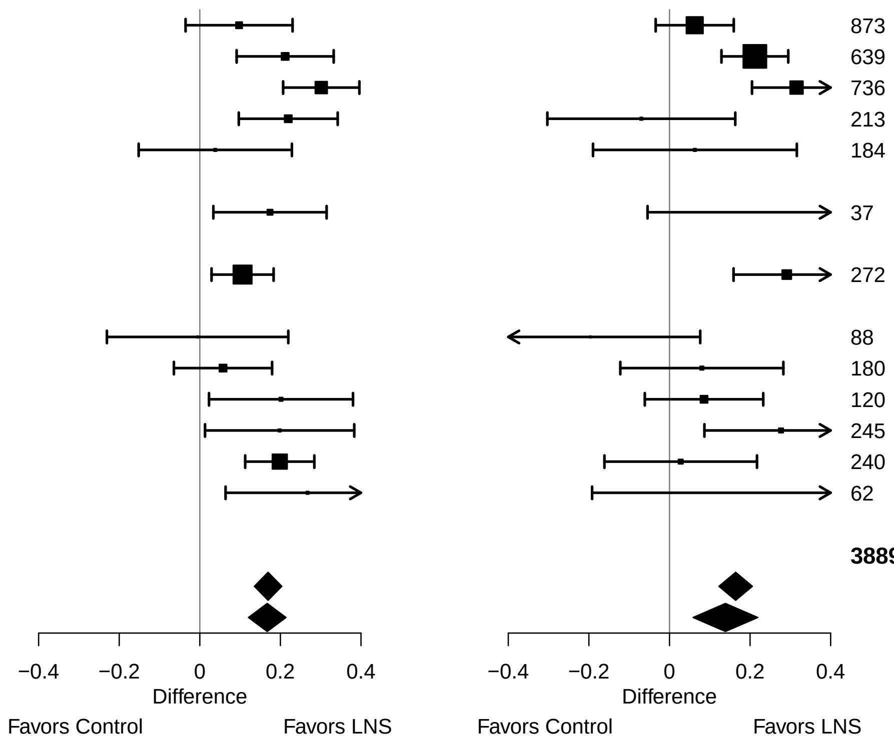

Supplemental figure 8A: Mean difference in LAZ

8A3: Stratified by Maternal age

P−for−interaction = 0.818
Difference in MDs = 0.00 (−0.05, 0.04)

At least 25 y

Country	Trial	LNS N	Control N	Control Mean	MD (95% CI)	Fixed W	Random W
Bangladesh	JiVitA−4 (34)	1218	546	−1.94	0.10 (0.03, 0.16)	0.21	0.12
Bangladesh	RDNS (35)	453	221	−1.80	0.19 (0.03, 0.35)	0.04	0.05
Bangladesh	WASH−B (36)	492	1512	−1.83	0.17 (0.09, 0.26)	0.12	0.10
Burkina Faso	iLiNS−Zinc (37)	1131	399	−1.72	0.33 (0.22, 0.45)	0.07	0.08
Burkina Faso	PROMIS (38)	485	526	−1.47	0.15 (−0.01, 0.32)	0.04	0.05
Burkina Faso	PROMIS CS (38)	202	192	−1.17	0.05 (−0.17, 0.27)	0.02	0.03
Ghana	GHANA (39)	69	63	−0.30	0.28 (−0.08, 0.65)	0.01	0.01
Ghana	iLiNS−DYADG (40)	220	416	−0.84	0.11 (−0.05, 0.27)	0.04	0.05
Haiti	HAITI (41)	101	103	−0.67	0.04 (−0.13, 0.21)	0.03	0.05
Kenya	WASH−B (42)	844	2811	−1.50	0.13 (0.05, 0.21)	0.16	0.11
Madagascar	MAHAY (43)	896	949	−2.26	0.03 (−0.13, 0.18)	0.04	0.05
Malawi	iLiNS−DYADM (44)	106	226	−1.65	−0.04 (−0.30, 0.21)	0.02	0.03
Malawi	iLiNS−DOSE (45)	368	134	−1.88	0.00 (−0.14, 0.14)	0.05	0.06
Mali	PROMIS (46)	346	359	−1.72	0.17 (0.02, 0.33)	0.04	0.05
Mali	PROMIS CS (46)	503	507	−1.47	0.16 (−0.04, 0.36)	0.02	0.04
Zimbabwe	SHINE (HIV−) (47)	875	800	−1.55	0.20 (0.08, 0.31)	0.07	0.08
Zimbabwe	SHINE (HIV+) (48)	244	231	−1.97	0.24 (0.04, 0.45)	0.02	0.04
		**8553**	**9995**		**I² = 0.38, Tau² = 0.00**		
**Fixed**					**0.14 (0.11, 0.17)**		
**Random**					**0.14 (0.10, 0.18)**		

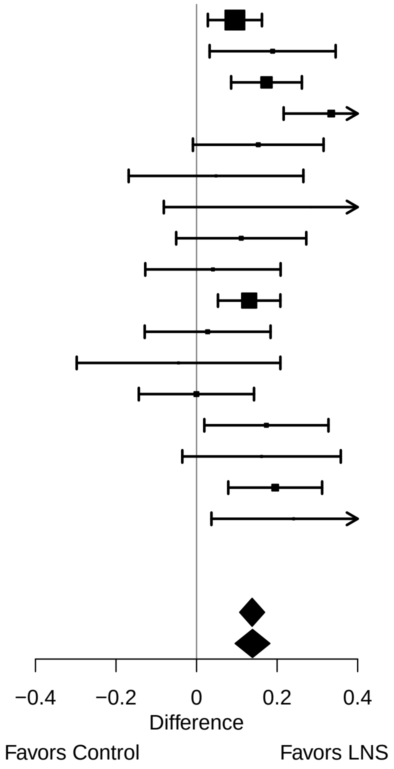

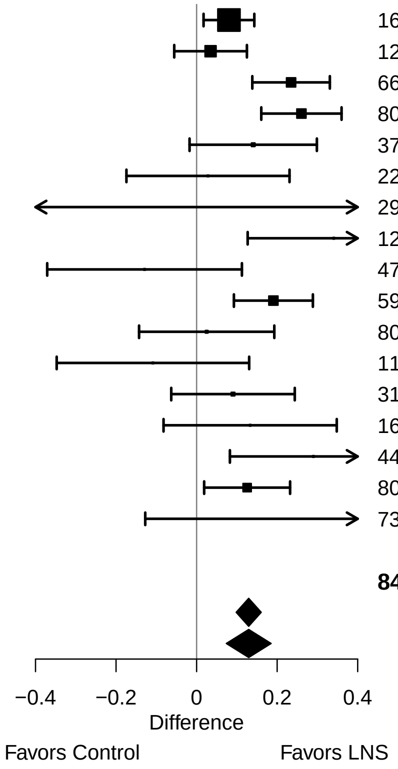

Less than 25 y

Country	Trial	LNS N	Control N	Control Mean	MD (95% CI)	Fixed W	Random W
Bangladesh	JiVitA−4 (34)	1608	693	−1.88	0.08 (0.02, 0.14)	0.24	0.10
Bangladesh	RDNS (35)	1210	594	−1.81	0.03 (−0.06, 0.12)	0.12	0.09
Bangladesh	WASH−B (36)	661	1903	−1.80	0.23 (0.14, 0.33)	0.10	0.09
Burkina Faso	iLiNS−Zinc (37)	808	262	−1.79	0.26 (0.16, 0.36)	0.10	0.09
Burkina Faso	PROMIS (38)	378	388	−1.49	0.14 (−0.02, 0.30)	0.04	0.06
Burkina Faso	PROMIS CS (38)	228	247	−1.27	0.03 (−0.17, 0.23)	0.02	0.05
Ghana	GHANA (39)	29	25	−0.58	0.16 (−0.42, 0.75)	0.00	0.01
Ghana	iLiNS−DYADG (40)	127	276	−0.97	0.34 (0.13, 0.55)	0.02	0.04
Haiti	HAITI (41)	47	45	−0.88	−0.13 (−0.37, 0.11)	0.02	0.04
Kenya	WASH−B (42)	598	2294	−1.65	0.19 (0.09, 0.29)	0.10	0.09
Madagascar	MAHAY (43)	805	733	−2.22	0.03 (−0.14, 0.19)	0.03	0.06
Malawi	iLiNS−DYADM (44)	114	218	−1.60	−0.11 (−0.35, 0.13)	0.02	0.04
Malawi	iLiNS−DOSE (45)	314	100	−1.99	0.09 (−0.06, 0.24)	0.04	0.06
Mali	PROMIS (46)	160	147	−1.75	0.13 (−0.08, 0.35)	0.02	0.04
Mali	PROMIS CS (46)	449	462	−1.68	0.29 (0.08, 0.50)	0.02	0.05
Zimbabwe	SHINE (HIV−) (47)	808	797	−1.66	0.13 (0.02, 0.23)	0.09	0.08
Zimbabwe	SHINE (HIV+) (48)	73	78	−2.07	0.24 (−0.13, 0.60)	0.01	0.02
		**8417**	**9262**		**I² = 0.56, Tau² = 0.01**		
**Fixed**					**0.13 (0.10, 0.16)**		
**Random**					**0.13 (0.07, 0.19)**		

Supplemental figure 8A: Mean difference in LAZ

8A4: Stratified by Maternal education

P−for−interaction = 0.699
Difference in MDs = 0.01 (−0.04, 0.06)

Primary or greater

Country	Trial	LNS N	Control N	Control Mean	MD (95% CI)	Fixed W	Random W
Bangladesh	JiVitA−4 (34)	1830	756	−1.78	0.08 (0.02, 0.14)	0.27	0.15
Bangladesh	RDNS (35)	1245	589	−1.77	0.10 (0.01, 0.19)	0.11	0.11
Bangladesh	WASH−B (36)	813	2452	−1.72	0.24 (0.16, 0.32)	0.14	0.12
Burkina Faso	iLiNS−Zinc (37)	82	16	−1.47	0.41 (0.11, 0.71)	0.01	0.02
Burkina Faso	PROMIS (38)	70	54	−1.39	−0.08 (−0.38, 0.23)	0.01	0.02
Burkina Faso	PROMIS CS (38)	34	39	−1.26	0.00 (−0.49, 0.49)	0.00	0.01
Ghana	GHANA (39)	91	75	−0.36	0.28 (−0.05, 0.62)	0.01	0.02
Ghana	iLiNS−DYADG (40)	267	548	−0.89	0.18 (0.03, 0.32)	0.04	0.07
Haiti	HAITI (41)	130	126	−0.71	−0.04 (−0.19, 0.11)	0.04	0.07
Kenya	WASH−B (42)	700	2434	−1.39	0.09 (0.00, 0.18)	0.11	0.11
Madagascar	MAHAY (43)	351	431	−2.17	0.03 (−0.17, 0.23)	0.02	0.04
Malawi	iLiNS−DYADM (44)	35	69	−1.37	−0.10 (−0.51, 0.32)	0.01	0.01
Malawi	iLiNS−DOSE (45)	162	56	−1.73	0.12 (−0.08, 0.32)	0.02	0.04
Mali	PROMIS (46)	41	37	−1.25	0.10 (−0.29, 0.49)	0.01	0.01
Mali	PROMIS CS (46)	107	96	−1.42	0.29 (−0.04, 0.63)	0.01	0.02
Zimbabwe	SHINE (HIV−) (47)	1717	1644	−1.59	0.15 (0.07, 0.23)	0.14	0.12
Zimbabwe	SHINE (HIV+) (48)	301	293	−1.96	0.21 (0.03, 0.40)	0.03	0.05
		**7976**	**9715**		**I² = 0.40, Tau² = 0.00**		
**Fixed**					**0.12 (0.09, 0.15)**		
**Random**					**0.12 (0.08, 0.17)**		

Incomplete or no formal

Country	Trial	LNS N	Control N	Control Mean	MD (95% CI)	Fixed W	Random W
Bangladesh	JiVitA−4 (34)	1006	485	−2.10	0.09 (0.01, 0.16)	0.20	0.11
Bangladesh	RDNS (35)	418	226	−1.89	−0.01 (−0.14, 0.13)	0.06	0.07
Bangladesh	WASH−B (36)	345	979	−2.06	0.16 (0.02, 0.29)	0.06	0.08
Burkina Faso	iLiNS−Zinc (37)	1857	645	−1.76	0.30 (0.22, 0.39)	0.15	0.10
Burkina Faso	PROMIS (38)	793	860	−1.49	0.17 (0.04, 0.30)	0.06	0.08
Burkina Faso	PROMIS CS (38)	393	399	−1.22	0.04 (−0.15, 0.22)	0.03	0.05
Ghana	GHANA (39)	7	15	−0.47	−0.39 (−1.25, 0.46)	0.00	0.00
Ghana	iLiNS−DYADG (40)	80	144	−0.91	0.28 (0.00, 0.55)	0.01	0.03
Haiti	HAITI (41)	19	23	−0.88	0.13 (−0.26, 0.53)	0.01	0.02
Kenya	WASH−B (42)	755	2699	−1.73	0.21 (0.13, 0.29)	0.17	0.10
Madagascar	MAHAY (43)	1351	1251	−2.26	0.03 (−0.12, 0.18)	0.05	0.07
Malawi	iLiNS−DYADM (44)	184	372	−1.68	−0.07 (−0.26, 0.12)	0.03	0.05
Malawi	iLiNS−DOSE (45)	522	178	−1.99	0.02 (−0.10, 0.15)	0.07	0.08
Mali	PROMIS (46)	464	469	−1.76	0.17 (0.03, 0.31)	0.05	0.07
Mali	PROMIS CS (46)	844	873	−1.58	0.21 (0.04, 0.39)	0.04	0.06
Zimbabwe	SHINE (HIV−) (47)	69	61	−1.69	0.09 (−0.32, 0.50)	0.01	0.02
Zimbabwe	SHINE (HIV+) (48)	21	18	−2.40	0.54 (−0.17, 1.25)	0.00	0.01
		**9128**	**9697**		**I² = 0.60, Tau² = 0.01**		
**Fixed**					**0.14 (0.11, 0.18)**		
**Random**					**0.13 (0.07, 0.18)**		

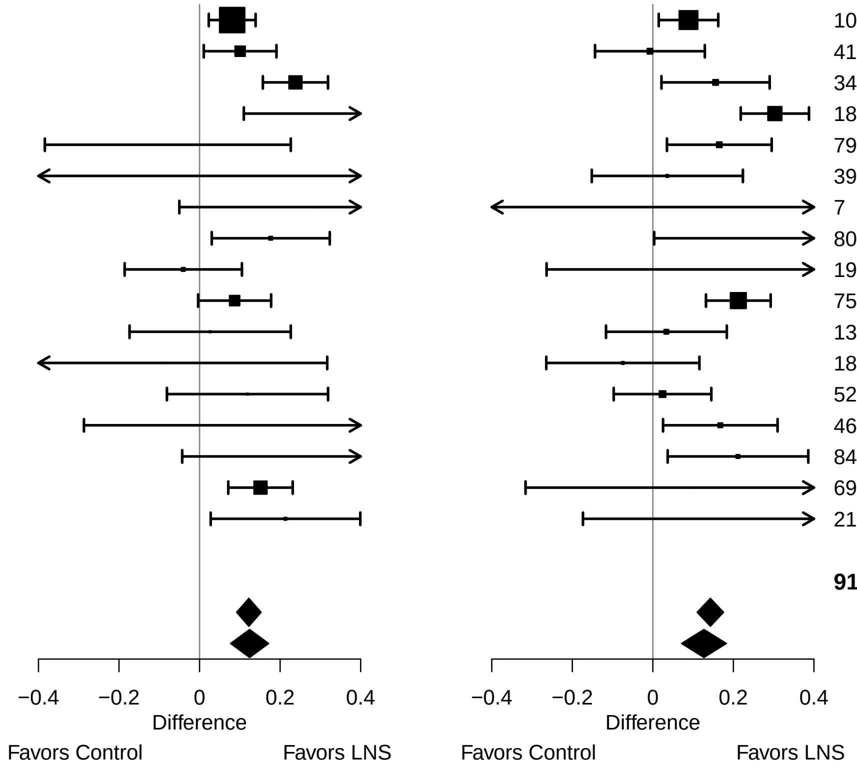

Supplemental figure 8A: Mean difference in LAZ

8A5: Stratified by Maternal depressive symptoms

P−for−interaction = 0.060
Difference in MDs = 0.06 (0.00, 0.13)

		Less than 75th percentile						At least 75th percentile					
**Country**	**Trial**	**LNS N**	**Control N**	**Control Mean**	**MD (95% CI)**	**Fixed W**	**Random W**	**LNS N**	**Control N**	**Control Mean**	**MD (95% CI)**	**Fixed W**	**Random W**
Bangladesh	JiVitA−4 (34)												
Bangladesh	RDNS (35)	1081	472	−1.80	0.12 (0.02, 0.23)	0.13	0.13	520	288	−1.81	0.01 (−0.14, 0.15)	0.14	0.13
Bangladesh	WASH−B (36)	897	2462	−1.79	0.23 (0.14, 0.31)	0.21	0.16	237	878	−1.88	0.15 (0.02, 0.28)	0.18	0.14
Burkina Faso	iLiNS−Zinc (37)												
Burkina Faso	PROMIS (38)	508	573	−1.53	0.24 (0.08, 0.40)	0.05	0.07	223	215	−1.40	−0.06 (−0.24, 0.12)	0.10	0.11
Burkina Faso	PROMIS CS (38)												
Ghana	GHANA (39)												
Ghana	iLiNS−DYADG (40)	252	459	−0.90	0.23 (0.08, 0.38)	0.06	0.08	83	209	−0.84	0.12 (−0.13, 0.37)	0.05	0.07
Haiti	HAITI (41)												
Kenya	WASH−B (42)	996	3496	−1.53	0.14 (0.07, 0.22)	0.25	0.18	339	1235	−1.63	0.12 (−0.02, 0.25)	0.17	0.14
Madagascar	MAHAY (43)	673	721	−2.28	0.04 (−0.15, 0.22)	0.04	0.06	284	252	−2.27	−0.14 (−0.36, 0.08)	0.06	0.09
Malawi	iLiNS−DYADM (44)	157	303	−1.55	−0.07 (−0.28, 0.14)	0.03	0.05	47	113	−1.77	−0.15 (−0.49, 0.20)	0.03	0.05
Malawi	iLiNS−DOSE (45)												
Mali	PROMIS (46)	379	373	−1.68	0.17 (0.01, 0.32)	0.06	0.08	123	131	−1.86	0.17 (−0.04, 0.37)	0.07	0.09
Mali	PROMIS CS (46)												
Zimbabwe	SHINE (HIV−) (47)	1198	1145	−1.56	0.12 (0.02, 0.22)	0.14	0.14	513	489	−1.73	0.27 (0.14, 0.40)	0.18	0.14
Zimbabwe	SHINE (HIV+) (48)	234	215	−1.96	0.25 (0.05, 0.46)	0.03	0.05	89	100	−2.13	0.21 (−0.14, 0.56)	0.02	0.05
		**6375**	**10219**		**I² = 0.30, Tau² = 0.00**			**2458**	**3910**		**I² = 0.53, Tau² = 0.01**		
**Fixed**					**0.16 (0.12, 0.20)**						**0.10 (0.05, 0.16)**		
**Random**					**0.15 (0.10, 0.21)**						**0.08 (0.00, 0.17)**		

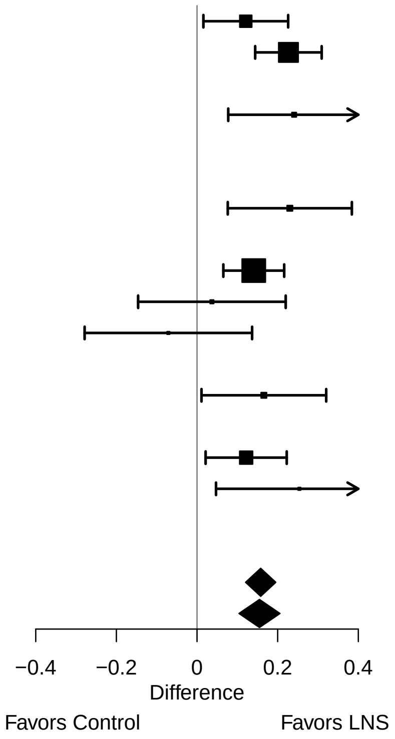

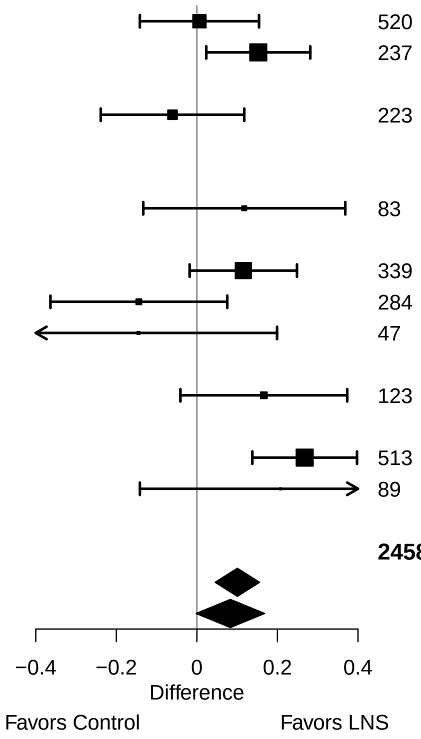

Supplemental figure 8A: Mean difference in LAZ

8A6: Stratified by Child sex

P−for−interaction = 0.170
Difference in MDs = 0.03 (−0.01, 0.07)

Country	Trial	Male LNS N	Male Control N	Male Control Mean	Male MD (95% CI)	Male Fixed W	Male Random W	Female LNS N	Female Control N	Female Control Mean	Female MD (95% CI)	Female Fixed W	Female Random W
Bangladesh	JiVitA−4 (34)	1448	600	−1.91	0.07 (0.00, 0.13)	0.24	0.11	1390	644	−1.90	0.11 (0.04, 0.17)	0.21	0.10
Bangladesh	RDNS (35)	833	411	−1.82	0.00 (−0.12, 0.13)	0.06	0.07	830	404	−1.80	0.15 (0.04, 0.26)	0.08	0.08
Bangladesh	WASH−B (36)	565	1714	−1.83	0.21 (0.12, 0.30)	0.13	0.10	593	1717	−1.80	0.21 (0.11, 0.31)	0.09	0.08
Burkina Faso	iLiNS−Zinc (37)	990	336	−1.89	0.31 (0.19, 0.42)	0.07	0.08	962	328	−1.63	0.31 (0.21, 0.40)	0.10	0.09
Burkina Faso	PROMIS (38)	438	483	−1.60	0.11 (−0.05, 0.28)	0.04	0.05	425	431	−1.35	0.17 (0.02, 0.32)	0.04	0.06
Burkina Faso	PROMIS CS (38)	234	222	−1.37	0.05 (−0.18, 0.27)	0.02	0.04	196	217	−1.07	0.06 (−0.13, 0.24)	0.03	0.04
Ghana	GHANA (39)	60	41	−0.44	0.27 (−0.15, 0.69)	0.01	0.01	38	55	−0.39	0.32 (−0.14, 0.78)	0.00	0.01
Ghana	iLiNS−DYADG (40)	175	323	−0.92	0.19 (−0.02, 0.39)	0.02	0.04	172	369	−0.87	0.22 (0.05, 0.38)	0.03	0.05
Haiti	HAITI (41)	70	59	−0.87	0.06 (−0.16, 0.27)	0.02	0.04	79	90	−0.66	−0.02 (−0.19, 0.16)	0.03	0.05
Kenya	WASH−B (42)	734	2440	−1.70	0.14 (0.05, 0.23)	0.12	0.10	723	2697	−1.45	0.18 (0.09, 0.27)	0.12	0.09
Madagascar	MAHAY (43)	826	832	−2.40	0.01 (−0.14, 0.17)	0.04	0.06	876	850	−2.08	0.03 (−0.13, 0.20)	0.04	0.05
Malawi	iLiNS−DYADM (44)	107	207	−1.73	−0.07 (−0.33, 0.19)	0.01	0.03	113	237	−1.54	−0.08 (−0.31, 0.16)	0.02	0.03
Malawi	iLiNS−DOSE (45)	352	124	−2.14	0.01 (−0.14, 0.16)	0.04	0.06	344	117	−1.73	0.11 (−0.03, 0.26)	0.05	0.06
Mali	PROMIS (46)	266	263	−1.80	0.07 (−0.09, 0.24)	0.04	0.05	240	243	−1.64	0.26 (0.09, 0.44)	0.03	0.05
Mali	PROMIS CS (46)	477	525	−1.63	0.21 (0.00, 0.42)	0.02	0.04	475	444	−1.49	0.23 (0.04, 0.42)	0.02	0.04
Zimbabwe	SHINE (HIV−) (47)	932	908	−1.80	0.24 (0.14, 0.34)	0.10	0.09	948	886	−1.40	0.06 (−0.04, 0.17)	0.08	0.08
Zimbabwe	SHINE (HIV+) (48)	162	169	−2.14	0.19 (−0.07, 0.44)	0.02	0.03	175	161	−1.86	0.30 (0.10, 0.50)	0.02	0.04
		**8669**	**9657**		**I² = 0.53, Tau² = 0.00**			**8579**	**9890**		**I² = 0.51, Tau² = 0.00**		
**Fixed**					**0.13 (0.10, 0.16)**						**0.15 (0.12, 0.18)**		
**Random**					**0.13 (0.08, 0.17)**						**0.15 (0.10, 0.20)**		

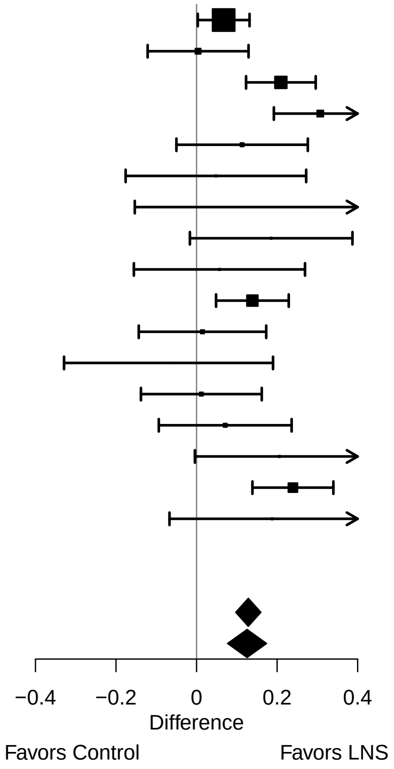

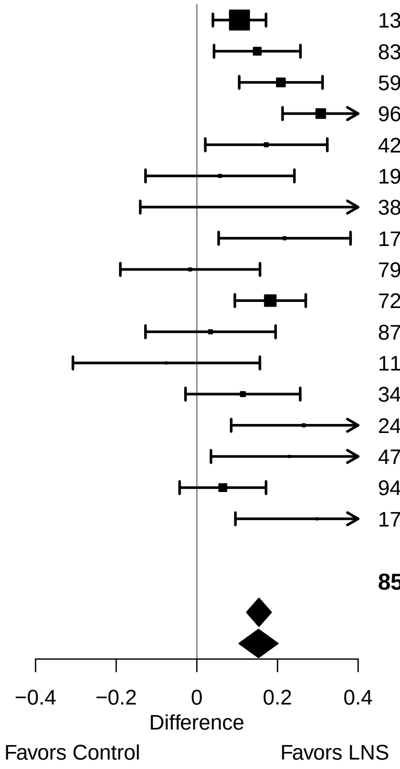

Supplemental figure 8A: Mean difference in LAZ

8A7: Stratified by Child birth order

P−for−interaction = 0.134
Difference in MDs = −0.03 (−0.08, 0.01)

Later born

Country	Trial	LNS N	Control N	Control Mean	MD (95% CI)	Fixed W	Random W
Bangladesh	JiVitA−4 (34)	611	255	−2.03	0.14 (0.04, 0.24)	0.08	0.08
Bangladesh	RDNS (35)	974	508	−1.83	0.11 (0.01, 0.22)	0.07	0.07
Bangladesh	WASH−B (36)	736	2290	−1.85	0.20 (0.12, 0.28)	0.14	0.09
Burkina Faso	iLiNS−Zinc (37)	1520	519	−1.72	0.30 (0.22, 0.39)	0.11	0.08
Burkina Faso	PROMIS (38)	715	754	−1.46	0.16 (0.02, 0.31)	0.04	0.05
Burkina Faso	PROMIS CS (38)	366	359	−1.20	0.03 (−0.14, 0.20)	0.03	0.05
Ghana	GHANA (39)	60	54	−0.39	0.40 (0.01, 0.80)	0.01	0.01
Ghana	iLiNS−DYADG (40)	237	458	−0.90	0.12 (−0.03, 0.28)	0.03	0.05
Haiti	HAITI (41)	88	99	−0.83	0.02 (−0.15, 0.19)	0.03	0.05
Kenya	WASH−B (42)	1134	4045	−1.57	0.14 (0.07, 0.20)	0.19	0.10
Madagascar	MAHAY (43)	1272	1217	−2.28	0.09 (−0.06, 0.24)	0.04	0.05
Malawi	iLiNS−DYADM (44)	173	353	−1.64	−0.09 (−0.28, 0.11)	0.02	0.04
Malawi	iLiNS−DOSE (45)	458	165	−1.92	0.00 (−0.12, 0.13)	0.05	0.06
Mali	PROMIS (46)	442	445	−1.74	0.21 (0.06, 0.37)	0.03	0.05
Mali	PROMIS CS (46)	790	831	−1.54	0.23 (0.06, 0.40)	0.03	0.04
Zimbabwe	SHINE (HIV−) (47)	1302	1223	−1.59	0.18 (0.09, 0.28)	0.09	0.08
Zimbabwe	SHINE (HIV+) (48)	280	247	−1.89	0.15 (−0.04, 0.33)	0.02	0.04
		**11158**	**13822**		**I² = 0.53, Tau² = 0.00**		
**Fixed**					**0.15 (0.13, 0.18)**		
**Random**					**0.14 (0.10, 0.19)**		

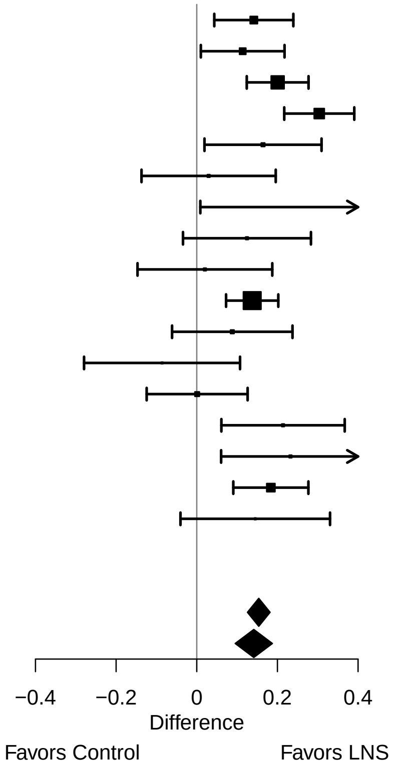

Firstborn

Country	Trial	LNS N	Control N	Control Mean	MD (95% CI)	Fixed W	Random W
Bangladesh	JiVitA−4 (34)	2223	982	−1.87	0.07 (0.02, 0.13)	0.44	0.10
Bangladesh	RDNS (35)	688	307	−1.78	0.02 (−0.13, 0.17)	0.06	0.08
Bangladesh	WASH−B (36)	422	1141	−1.73	0.21 (0.09, 0.34)	0.09	0.08
Burkina Faso	iLiNS−Zinc (37)	432	145	−1.88	0.32 (0.20, 0.44)	0.09	0.08
Burkina Faso	PROMIS (38)	148	158	−1.59	0.06 (−0.18, 0.30)	0.02	0.06
Burkina Faso	PROMIS CS (38)	63	80	−1.33	0.07 (−0.41, 0.55)	0.01	0.02
Ghana	GHANA (39)	38	38	−0.36	−0.01 (−0.49, 0.47)	0.01	0.02
Ghana	iLiNS−DYADG (40)	110	234	−0.87	0.36 (0.14, 0.58)	0.03	0.06
Haiti	HAITI (41)	61	50	−0.56	−0.10 (−0.33, 0.13)	0.02	0.06
Kenya	WASH−B (42)	321	1087	−1.57	0.22 (0.06, 0.37)	0.05	0.08
Madagascar	MAHAY (43)	430	465	−2.14	−0.14 (−0.34, 0.06)	0.03	0.06
Malawi	iLiNS−DYADM (44)	47	89	−1.57	−0.05 (−0.44, 0.34)	0.01	0.03
Malawi	iLiNS−DOSE (45)	149	45	−1.95	0.12 (−0.10, 0.34)	0.03	0.06
Mali	PROMIS (46)	63	59	−1.61	−0.20 (−0.47, 0.07)	0.02	0.05
Mali	PROMIS CS (46)	145	126	−1.74	0.27 (−0.02, 0.57)	0.01	0.04
Zimbabwe	SHINE (HIV−) (47)	491	487	−1.64	0.10 (−0.02, 0.23)	0.08	0.08
Zimbabwe	SHINE (HIV+) (48)	54	78	−2.29	0.56 (0.12, 0.99)	0.01	0.03
		**5885**	**5571**		**I² = 0.65, Tau² = 0.02**		
**Fixed**					**0.11 (0.08, 0.15)**		
**Random**					**0.11 (0.03, 0.20)**		

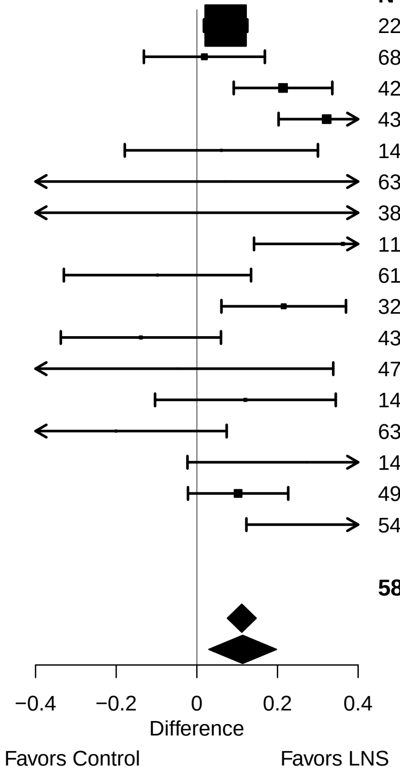

Supplemental figure 8A: Mean difference in LAZ

8A8: Stratified by Child baseline anthropometric status

P−for−interaction = 0.932
Difference in MDs = 0.00 (−0.06, 0.05)

		LAZ at least −1 Z						LAZ less than −1 Z					
**Country**	**Trial**	**LNS N**	**Control N**	**Control Mean**	**MD (95% CI)**	**Fixed W**	**Random W**	**LNS N**	**Control N**	**Control Mean**	**MD (95% CI)**	**Fixed W**	**Random W**
Bangladesh	JiVitA−4 (34)	1055	454	−1.11	0.10 (0.01, 0.19)	0.21	0.20	1783	790	−2.36	0.07 (−0.01, 0.15)	0.26	0.16
Bangladesh	RDNS (35)	630	306	−1.08	0.09 (−0.01, 0.19)	0.18	0.18	971	453	−2.30	0.10 (0.00, 0.20)	0.18	0.14
Bangladesh	WASH−B (36)												
Burkina Faso	iLiNS−Zinc (37)	839	291	−0.85	0.21 (0.04, 0.38)	0.06	0.06	1113	373	−2.47	0.38 (0.23, 0.52)	0.08	0.10
Burkina Faso	PROMIS (38)	444	484	−1.04	0.16 (0.04, 0.28)	0.12	0.12	257	274	−2.17	0.07 (−0.10, 0.23)	0.06	0.08
Burkina Faso	PROMIS CS (38)												
Ghana	GHANA (39)												
Ghana	iLiNS−DYADG (40)	203	387	−0.37	0.16 (0.03, 0.30)	0.10	0.10	120	254	−1.68	0.10 (−0.05, 0.26)	0.07	0.09
Haiti	HAITI (41)	101	109	−0.30	0.13 (−0.12, 0.37)	0.03	0.03	48	40	−1.95	0.12 (−0.23, 0.46)	0.01	0.03
Kenya	WASH−B (42)												
Madagascar	MAHAY (43)												
Malawi	iLiNS−DYADM (44)	82	179	−0.91	−0.06 (−0.29, 0.17)	0.03	0.03	122	244	−2.16	−0.04 (−0.24, 0.15)	0.04	0.07
Malawi	iLiNS−DOSE (45)	260	79	−0.94	−0.06 (−0.27, 0.15)	0.04	0.04	436	162	−2.42	0.12 (−0.04, 0.27)	0.07	0.09
Mali	PROMIS (46)	296	266	−1.18	0.21 (0.00, 0.42)	0.04	0.04	210	240	−2.33	0.15 (−0.05, 0.35)	0.04	0.07
Mali	PROMIS CS (46)												
Zimbabwe	SHINE (HIV−) (47)	725	638	−1.01	0.09 (−0.01, 0.19)	0.17	0.17	602	571	−2.11	0.08 (−0.03, 0.19)	0.14	0.13
Zimbabwe	SHINE (HIV+) (48)	103	107	−1.28	0.29 (0.03, 0.56)	0.02	0.03	165	131	−2.40	0.22 (0.00, 0.45)	0.03	0.05
		**4738**	**3300**		**I² = 0.03, Tau² = 0.00**			**5827**	**3532**		**I² = 0.43, Tau² = 0.00**		
**Fixed**					**0.11 (0.07, 0.15)**						**0.11 (0.07, 0.15)**		
**Random**					**0.11 (0.07, 0.16)**						**0.12 (0.06, 0.18)**		

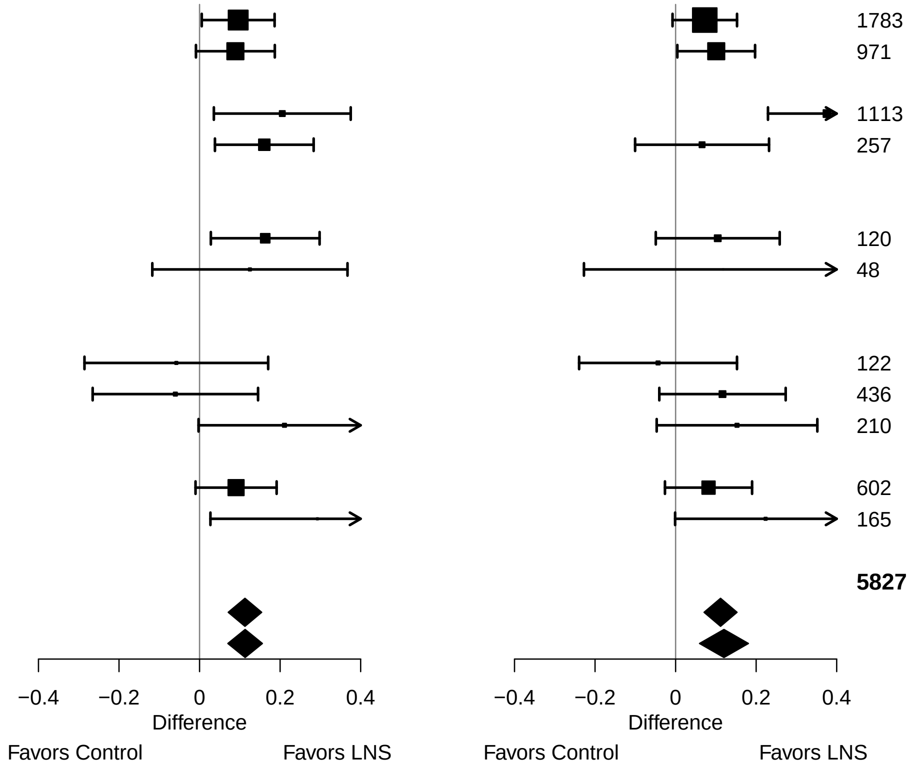

Supplemental figure 8B: Stunting prevalence ratio

8B1: Stratified by Maternal height

P−for−interaction = 0.020
Ratio of PRs = 1.09 (1.01, 1.18)

		At least 150.1 cm						Less than 150.1 cm					
**Country**	**Trial**	**LNS N**	**Control N**	**Control Prevalence**	**PR (95% CI)**	**Fixed W**	**Random W**	**LNS N**	**Control N**	**Control Prevalence**	**PR (95% CI)**	**Fixed W**	**Random W**
Bangladesh	JiVitA−4 (34)												
Bangladesh	RDNS (35)	868	432	30.6	0.86 (0.70, 1.06)	0.05	0.08	735	358	56.1	0.94 (0.84, 1.05)	0.23	0.23
Bangladesh	WASH−B (36)	616	1883	32.4	0.76 (0.66, 0.88)	0.10	0.11	542	1545	53.5	0.88 (0.80, 0.97)	0.29	0.29
Burkina Faso	iLiNS−Zinc (37)	1916	649	38.8	0.75 (0.64, 0.87)	0.09	0.10	34	15	66.7	0.99 (0.65, 1.50)	0.02	0.02
Burkina Faso	PROMIS (38)	835	884	29.6	0.80 (0.64, 1.00)	0.04	0.07	25	25	68.0	0.88 (0.47, 1.65)	0.01	0.01
Burkina Faso	PROMIS CS (38)												
Ghana	GHANA (39)												
Ghana	iLiNS−DYADG (40)												
Haiti	HAITI (41)												
Kenya	WASH−B (42)	1313	4686	30.7	0.87 (0.78, 0.97)	0.18	0.13	57	190	58.4	0.84 (0.61, 1.15)	0.03	0.03
Madagascar	MAHAY (43)	1004	1037	53.9	0.94 (0.84, 1.05)	0.17	0.13	598	565	68.8	1.01 (0.91, 1.11)	0.29	0.29
Malawi	iLiNS−DYADM (44)	192	381	29.9	1.13 (0.88, 1.45)	0.03	0.06	28	60	61.7	1.10 (0.80, 1.52)	0.03	0.03
Malawi	iLiNS−DOSE (45)	580	198	41.9	0.98 (0.83, 1.16)	0.08	0.10	115	42	66.7	1.03 (0.82, 1.30)	0.05	0.05
Mali	PROMIS (46)												
Mali	PROMIS CS (46)	914	936	32.9	0.76 (0.63, 0.93)	0.06	0.08	33	27	66.7	0.91 (0.67, 1.23)	0.03	0.03
Zimbabwe	SHINE (HIV−) (47)	1755	1697	34.0	0.77 (0.69, 0.85)	0.19	0.13	76	62	61.3	0.90 (0.70, 1.16)	0.04	0.04
Zimbabwe	SHINE (HIV+) (48)												
		**9993**	**12783**		**I² = 0.57, Tau² = 0.01**			**2243**	**2889**		**I² = 0.00, Tau² = 0.00**		
**Fixed**					**0.84 (0.81, 0.88)**						**0.94 (0.90, 0.99)**		
**Random**					**0.85 (0.78, 0.92)**						**0.94 (0.90, 0.99)**		

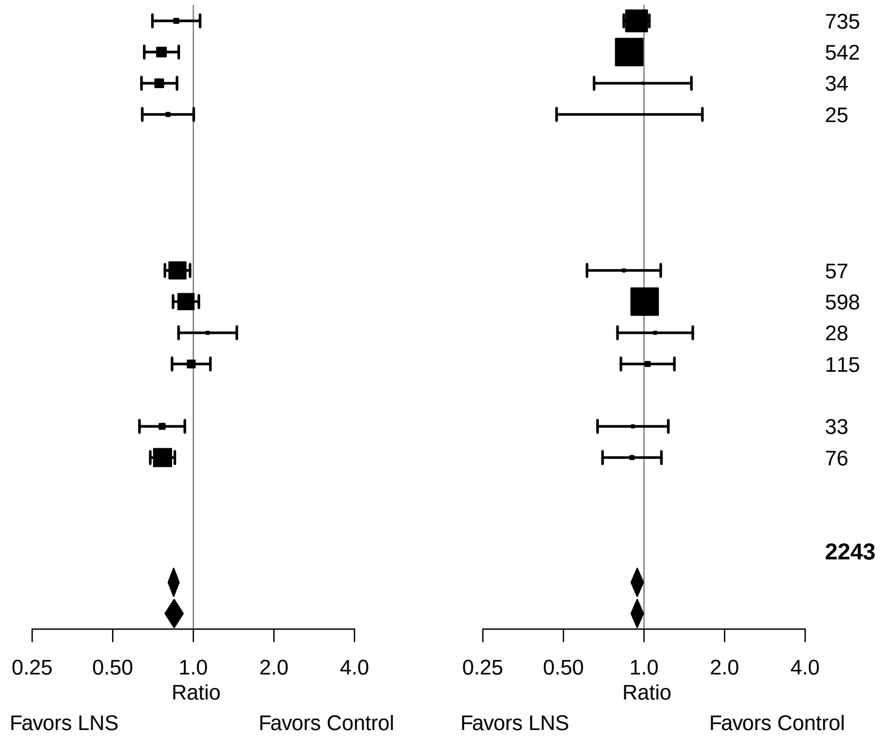

Supplemental figure 8B: Stunting prevalence ratio

8B2: Stratified by Maternal BMI

P−for−interaction = 0.017
Ratio of PRs = 1.10 (1.02, 1.19)

		At least 20 kg/m²						Less than 20 kg/m²					
**Country**	**Trial**	**LNS N**	**Control N**	**Control Prevalence**	**PR (95% CI)**	**Fixed W**	**Random W**	**LNS N**	**Control N**	**Control Prevalence**	**PR (95% CI)**	**Fixed W**	**Random W**
Bangladesh	JiVitA−4 (34)												
Bangladesh	RDNS (35)	730	335	38.8	0.86 (0.73, 1.01)	0.09	0.10	873	455	44.6	0.96 (0.86, 1.06)	0.30	0.22
Bangladesh	WASH−B (36)	519	1576	37.3	0.84 (0.72, 0.98)	0.10	0.10	639	1852	45.8	0.84 (0.75, 0.93)	0.26	0.20
Burkina Faso	iLiNS−Zinc (37)	1214	390	37.1	0.67 (0.58, 0.77)	0.11	0.10	736	274	42.7	0.87 (0.69, 1.10)	0.06	0.08
Burkina Faso	PROMIS (38)	647	675	29.9	0.71 (0.56, 0.89)	0.04	0.07	213	233	32.6	1.11 (0.82, 1.50)	0.04	0.05
Burkina Faso	PROMIS CS (38)	246	269	21.6	0.89 (0.61, 1.29)	0.02	0.03	184	166	28.3	0.84 (0.59, 1.21)	0.02	0.03
Ghana	GHANA (39)												
Ghana	iLiNS−DYADG (40)												
Haiti	HAITI (41)												
Kenya	WASH−B (42)	1098	3803	30.6	0.90 (0.81, 1.01)	0.18	0.12	272	1073	36.1	0.78 (0.63, 0.98)	0.07	0.08
Madagascar	MAHAY (43)												
Malawi	iLiNS−DYADM (44)	131	263	33.8	1.11 (0.84, 1.46)	0.03	0.05	88	178	34.8	1.11 (0.80, 1.54)	0.03	0.04
Malawi	iLiNS−DOSE (45)	513	174	44.8	0.98 (0.83, 1.16)	0.08	0.09	180	65	50.8	1.04 (0.80, 1.34)	0.05	0.06
Mali	PROMIS (46)	380	370	34.9	0.88 (0.69, 1.11)	0.04	0.06	120	135	40.7	1.01 (0.78, 1.31)	0.05	0.06
Mali	PROMIS CS (46)	702	684	31.1	0.83 (0.66, 1.03)	0.05	0.07	245	279	40.5	0.70 (0.54, 0.90)	0.05	0.06
Zimbabwe	SHINE (HIV−) (47)	1364	1305	35.9	0.74 (0.66, 0.82)	0.20	0.12	240	249	34.1	0.98 (0.77, 1.24)	0.06	0.07
Zimbabwe	SHINE (HIV+) (48)	247	254	48.8	0.75 (0.62, 0.92)	0.06	0.08	62	52	53.8	0.93 (0.65, 1.33)	0.02	0.04
		**7791**	**10098**		**I² = 0.58, Tau² = 0.01**			**3852**	**5011**		**I² = 0.22, Tau² = 0.00**		
**Fixed**					**0.82 (0.78, 0.86)**						**0.90 (0.86, 0.96)**		
**Random**					**0.83 (0.76, 0.89)**						**0.91 (0.85, 0.97)**		

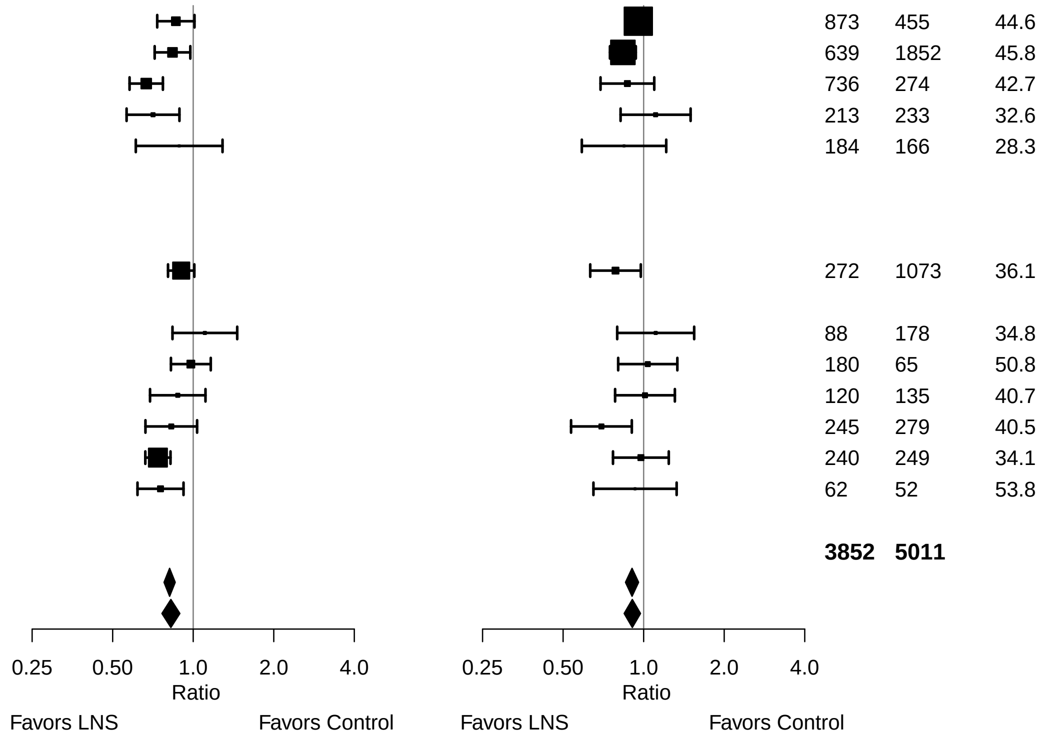

Supplemental figure 8B: Stunting prevalence ratio

8B3: Stratified by Maternal age

P−for−interaction = 0.192
Ratio of PRs = 1.04 (0.98, 1.10)

		At least 25 y						Less than 25 y					
**Country**	**Trial**	**LNS N**	**Control N**	**Control Prevalence**	**PR (95% CI)**	**Fixed W**	**Random W**	**LNS N**	**Control N**	**Control Prevalence**	**PR (95% CI)**	**Fixed W**	**Random W**
Bangladesh	JiVitA−4 (34)	1218	546	46.3	0.91 (0.80, 1.03)	0.11	0.10	1608	693	42.6	0.93 (0.83, 1.04)	0.14	0.10
Bangladesh	RDNS (35)	453	221	40.7	0.80 (0.65, 0.97)	0.05	0.06	1210	594	42.4	0.96 (0.88, 1.06)	0.20	0.11
Bangladesh	WASH−B (36)	492	1512	42.7	0.85 (0.76, 0.95)	0.15	0.11	661	1903	41.4	0.82 (0.73, 0.93)	0.12	0.10
Burkina Faso	iLiNS−Zinc (37)	1131	399	38.0	0.66 (0.55, 0.80)	0.06	0.07	808	262	41.6	0.86 (0.73, 1.03)	0.06	0.08
Burkina Faso	PROMIS (38)	485	526	30.8	0.77 (0.60, 0.99)	0.03	0.04	378	388	30.2	0.89 (0.70, 1.13)	0.03	0.05
Burkina Faso	PROMIS CS (38)	202	192	22.4	0.86 (0.62, 1.20)	0.02	0.03	228	247	25.9	0.88 (0.61, 1.26)	0.01	0.03
Ghana	GHANA (39)												
Ghana	iLiNS−DYADG (40)	220	416	11.1	0.95 (0.59, 1.52)	0.01	0.01	127	276	15.9	0.35 (0.16, 0.75)	0.00	0.01
Haiti	HAITI (41)	101	103	9.7	1.02 (0.46, 2.23)	0.00	0.01	47	45	22.2	1.15 (0.59, 2.23)	0.00	0.01
Kenya	WASH−B (42)	844	2811	30.1	0.89 (0.79, 1.02)	0.11	0.10	598	2294	34.8	0.81 (0.71, 0.92)	0.10	0.09
Madagascar	MAHAY (43)	896	949	59.0	0.97 (0.88, 1.07)	0.19	0.12	805	733	57.8	0.99 (0.89, 1.12)	0.13	0.10
Malawi	iLiNS−DYADM (44)	106	226	38.9	1.04 (0.79, 1.38)	0.02	0.04	114	218	30.3	1.19 (0.87, 1.63)	0.02	0.04
Malawi	iLiNS−DOSE (45)	368	134	41.8	1.09 (0.90, 1.33)	0.05	0.06	314	100	51.0	0.95 (0.77, 1.16)	0.04	0.06
Mali	PROMIS (46)	346	359	36.8	0.85 (0.70, 1.03)	0.05	0.06	160	147	35.4	1.08 (0.75, 1.54)	0.01	0.03
Mali	PROMIS CS (46)	503	507	31.0	0.82 (0.63, 1.06)	0.03	0.04	449	462	36.8	0.75 (0.60, 0.94)	0.03	0.06
Zimbabwe	SHINE (HIV−) (47)	875	800	32.0	0.79 (0.68, 0.93)	0.07	0.08	808	797	38.5	0.79 (0.68, 0.91)	0.09	0.09
Zimbabwe	SHINE (HIV+) (48)	244	231	50.2	0.78 (0.64, 0.95)	0.05	0.06	73	78	50.0	0.93 (0.68, 1.28)	0.02	0.04
		**8484**	**9932**		**I² = 0.40, Tau² = 0.01**			**8388**	**9237**		**I² = 0.44, Tau² = 0.01**		
**Fixed**					**0.87 (0.84, 0.91)**						**0.90 (0.86, 0.94)**		
**Random**					**0.86 (0.81, 0.92)**						**0.89 (0.83, 0.95)**		

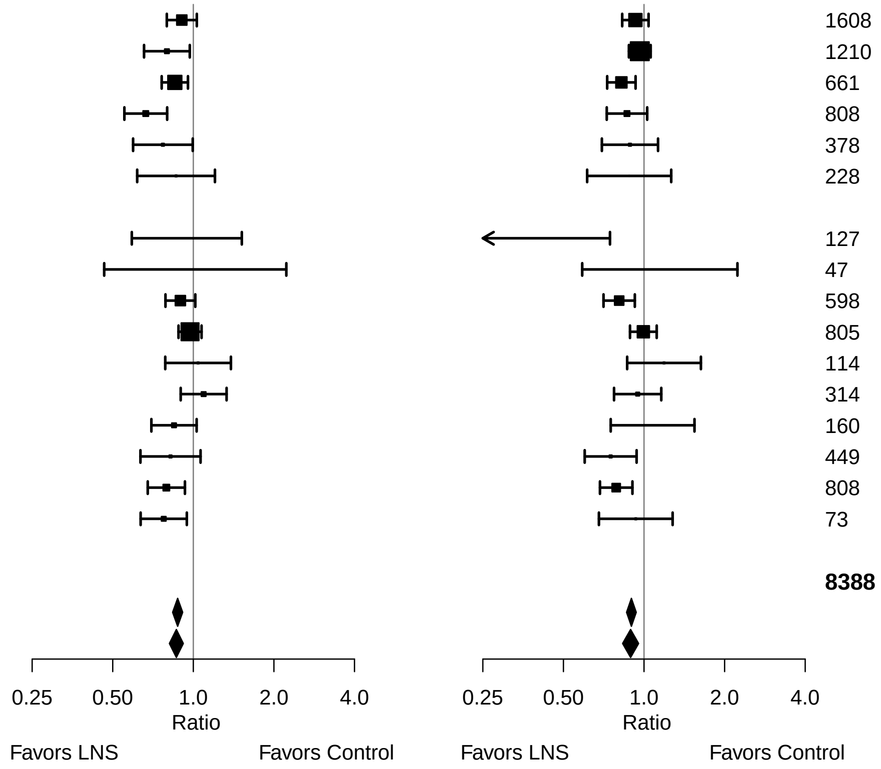

Supplemental figure 8B: Stunting prevalence ratio

8B4: Stratified by Maternal education

P−for−interaction = 0.042
Ratio of PRs = 1.07 (1.00, 1.15)

		Primary or greater						Incomplete or no formal					
**Country**	**Trial**	**LNS N**	**Control N**	**Control Prevalence**	**PR (95% CI)**	**Fixed W**	**Random W**	**LNS N**	**Control N**	**Control Prevalence**	**PR (95% CI)**	**Fixed W**	**Random W**
Bangladesh	JiVitA−4 (34)	1830	756	40.1	0.87 (0.78, 0.97)	0.16	0.16	1006	485	50.8	0.97 (0.86, 1.09)	0.12	0.10
Bangladesh	RDNS (35)	1245	589	40.2	0.88 (0.79, 0.99)	0.16	0.15	418	226	46.5	1.03 (0.90, 1.18)	0.09	0.09
Bangladesh	WASH−B (36)	813	2452	38.4	0.78 (0.71, 0.87)	0.19	0.17	345	979	50.7	0.93 (0.81, 1.06)	0.09	0.09
Burkina Faso	iLiNS−Zinc (37)	82	16	31.2	0.46 (0.22, 0.97)	0.00	0.00	1857	645	39.5	0.77 (0.66, 0.89)	0.08	0.08
Burkina Faso	PROMIS (38)	70	54	27.8	1.13 (0.62, 2.05)	0.01	0.01	793	860	30.7	0.80 (0.65, 0.97)	0.04	0.06
Burkina Faso	PROMIS CS (38)	34	39	25.6	0.69 (0.35, 1.37)	0.00	0.01	393	399	24.3	0.89 (0.65, 1.23)	0.02	0.03
Ghana	GHANA (39)												
Ghana	iLiNS−DYADG (40)	267	548	13.7	0.57 (0.36, 0.91)	0.01	0.01	80	144	10.4	1.08 (0.50, 2.36)	0.00	0.01
Haiti	HAITI (41)												
Kenya	WASH−B (42)	700	2434	26.0	0.91 (0.78, 1.06)	0.09	0.10	755	2699	37.8	0.82 (0.74, 0.92)	0.14	0.11
Madagascar	MAHAY (43)	351	431	56.8	0.97 (0.84, 1.12)	0.10	0.10	1351	1251	59.1	0.98 (0.90, 1.08)	0.19	0.12
Malawi	iLiNS−DYADM (44)	35	69	29.0	0.99 (0.52, 1.87)	0.00	0.01	184	372	35.8	1.12 (0.90, 1.41)	0.03	0.06
Malawi	iLiNS−DOSE (45)	162	56	44.6	0.86 (0.58, 1.26)	0.01	0.02	522	178	46.1	1.07 (0.92, 1.25)	0.07	0.08
Mali	PROMIS (46)	41	37	24.3	1.08 (0.54, 2.17)	0.00	0.01	464	469	37.3	0.91 (0.77, 1.09)	0.06	0.07
Mali	PROMIS CS (46)	107	96	32.3	0.69 (0.43, 1.12)	0.01	0.01	844	873	33.9	0.80 (0.66, 0.96)	0.05	0.07
Zimbabwe	SHINE (HIV−) (47)	1717	1644	34.8	0.79 (0.71, 0.87)	0.20	0.18	69	61	36.1	0.88 (0.56, 1.40)	0.01	0.02
Zimbabwe	SHINE (HIV+) (48)	301	293	48.5	0.84 (0.70, 1.01)	0.06	0.07	21	18	66.7	0.57 (0.31, 1.05)	0.00	0.01
		**7755**	**9514**		**I² = 0.16, Tau² = 0.00**			**9102**	**9659**		**I² = 0.52, Tau² = 0.01**		
**Fixed**					**0.84 (0.81, 0.88)**						**0.92 (0.89, 0.96)**		
**Random**					**0.85 (0.80, 0.89)**						**0.92 (0.86, 0.98)**		

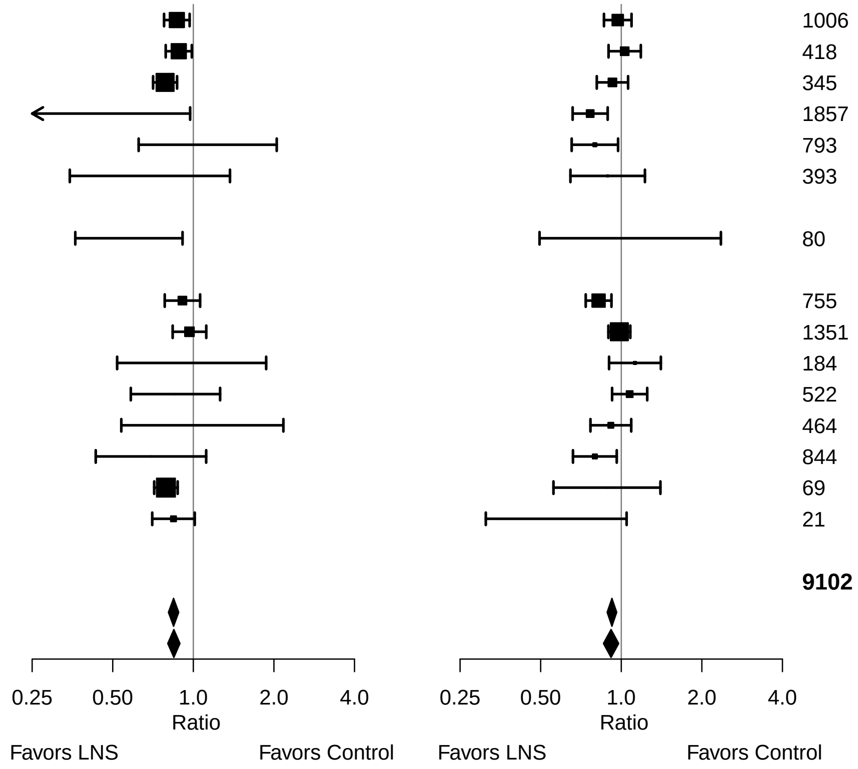

Supplemental figure 8B: Stunting prevalence ratio

8B5: Stratified by Maternal depressive symptoms

P−for−interaction = 0.003
Ratio of PRs = 0.89 (0.82, 0.96)

		Less than 75th percentile						At least 75th percentile					
**Country**	**Trial**	**LNS N**	**Control N**	**Control Prevalence**	**PR (95% CI)**	**Fixed W**	**Random W**	**LNS N**	**Control N**	**Control Prevalence**	**PR (95% CI)**	**Fixed W**	**Random W**
Bangladesh	JiVitA−4 (34)												
Bangladesh	RDNS (35)	1081	472	42.4	0.87 (0.79, 0.97)	0.21	0.17	520	288	41.0	1.00 (0.81, 1.23)	0.11	0.12
Bangladesh	WASH−B (36)	897	2462	41.3	0.80 (0.72, 0.89)	0.21	0.17	237	878	43.8	0.94 (0.79, 1.12)	0.15	0.13
Burkina Faso	iLiNS−Zinc (37)												
Burkina Faso	PROMIS (38)	508	573	30.7	0.77 (0.58, 1.02)	0.03	0.04	223	215	28.8	1.03 (0.77, 1.37)	0.06	0.09
Burkina Faso	PROMIS CS (38)												
Ghana	GHANA (39)												
Ghana	iLiNS−DYADG (40)	252	459	13.3	0.51 (0.30, 0.85)	0.01	0.01	83	209	10.5	1.37 (0.71, 2.65)	0.01	0.03
Haiti	HAITI (41)												
Kenya	WASH−B (42)	996	3496	31.1	0.87 (0.76, 0.98)	0.14	0.14	339	1235	34.3	0.94 (0.79, 1.12)	0.15	0.13
Madagascar	MAHAY (43)	673	721	60.5	0.94 (0.83, 1.05)	0.16	0.15	284	252	60.3	1.08 (0.94, 1.23)	0.25	0.15
Malawi	iLiNS−DYADM (44)	157	303	31.7	1.03 (0.78, 1.36)	0.03	0.04	47	113	39.8	1.44 (1.03, 2.02)	0.04	0.07
Malawi	iLiNS−DOSE (45)												
Mali	PROMIS (46)	379	373	35.1	0.92 (0.74, 1.13)	0.05	0.07	123	131	40.5	0.89 (0.67, 1.18)	0.06	0.09
Mali	PROMIS CS (46)												
Zimbabwe	SHINE (HIV−) (47)	1198	1145	33.8	0.82 (0.72, 0.94)	0.13	0.13	513	489	38.2	0.71 (0.59, 0.86)	0.13	0.12
Zimbabwe	SHINE (HIV+) (48)	234	215	49.3	0.75 (0.60, 0.94)	0.04	0.06	89	100	53.0	0.98 (0.73, 1.31)	0.05	0.08
		**6375**	**10219**		**I² = 0.26, Tau² = 0.00**			**2458**	**3910**		**I² = 0.56, Tau² = 0.02**		
**Fixed**					**0.85 (0.81, 0.89)**						**0.97 (0.91, 1.04)**		
**Random**					**0.85 (0.80, 0.91)**						**0.98 (0.87, 1.09)**		

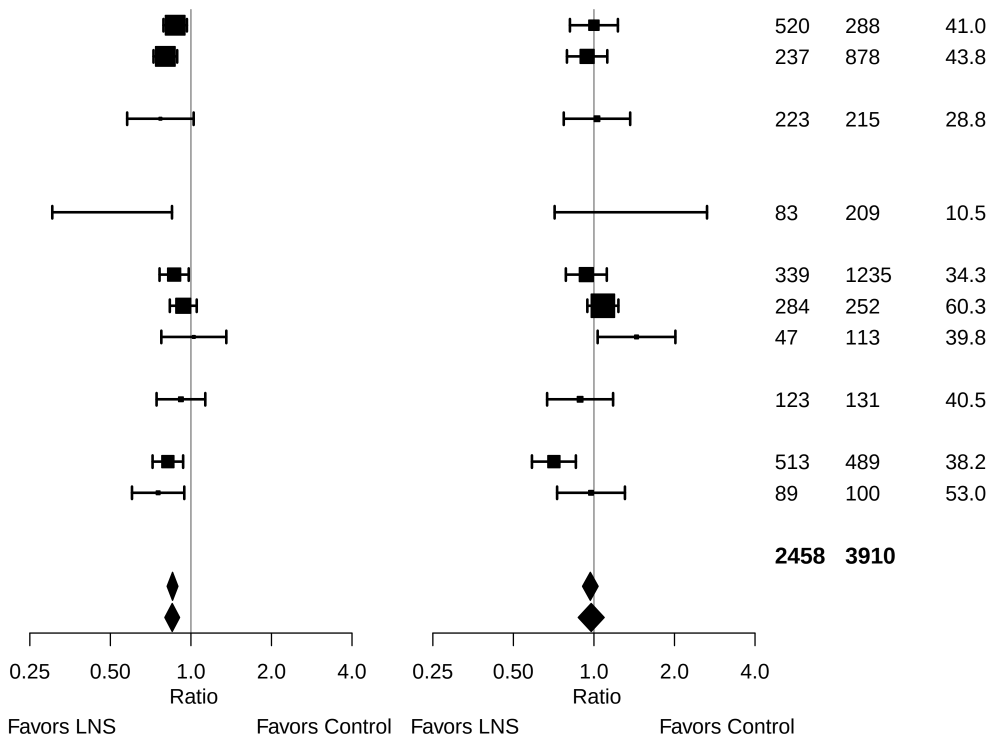

Supplemental figure 8B: Stunting prevalence ratio

8B6: Stratified by Child sex

P−for−interaction = 0.002
Ratio of PRs = 0.92 (0.87, 0.97)

Male

Country	Trial	LNS N	Control N	Control Prevalence	PR (95% CI)	Fixed W	Random W
Bangladesh	JiVitA−4 (34)	1448	600	44.5	0.94 (0.84, 1.06)	0.11	0.10
Bangladesh	RDNS (35)	833	411	42.6	1.00 (0.87, 1.15)	0.07	0.08
Bangladesh	WASH−B (36)	565	1714	43.1	0.83 (0.75, 0.93)	0.12	0.10
Burkina Faso	iLiNS−Zinc (37)	990	336	43.2	0.81 (0.69, 0.94)	0.06	0.07
Burkina Faso	PROMIS (38)	438	483	36.2	0.79 (0.62, 1.01)	0.03	0.04
Burkina Faso	PROMIS CS (38)	234	222	29.3	0.95 (0.68, 1.32)	0.01	0.02
Ghana	GHANA (39)						
Ghana	iLiNS−DYADG (40)	175	323	16.7	0.75 (0.47, 1.19)	0.01	0.01
Haiti	HAITI (41)	70	59	18.6	0.78 (0.40, 1.52)	0.00	0.01
Kenya	WASH−B (42)	734	2440	36.8	0.93 (0.83, 1.04)	0.11	0.10
Madagascar	MAHAY (43)	826	832	63.3	1.02 (0.94, 1.12)	0.19	0.12
Malawi	iLiNS−DYADM (44)	107	207	38.6	1.11 (0.84, 1.47)	0.02	0.03
Malawi	iLiNS−DOSE (45)	352	124	54.8	1.00 (0.85, 1.18)	0.06	0.07
Mali	PROMIS (46)	266	263	38.8	1.01 (0.82, 1.25)	0.03	0.05
Mali	PROMIS CS (46)	477	525	36.6	0.85 (0.69, 1.05)	0.03	0.05
Zimbabwe	SHINE (HIV−) (47)	932	908	41.9	0.77 (0.68, 0.86)	0.11	0.10
Zimbabwe	SHINE (HIV+) (48)	162	169	53.8	0.86 (0.70, 1.05)	0.04	0.05
		**8609**	**9616**		**I² = 0.49, Tau² = 0.00**		
**Fixed**					**0.91 (0.88, 0.95)**		
**Random**					**0.91 (0.86, 0.96)**		

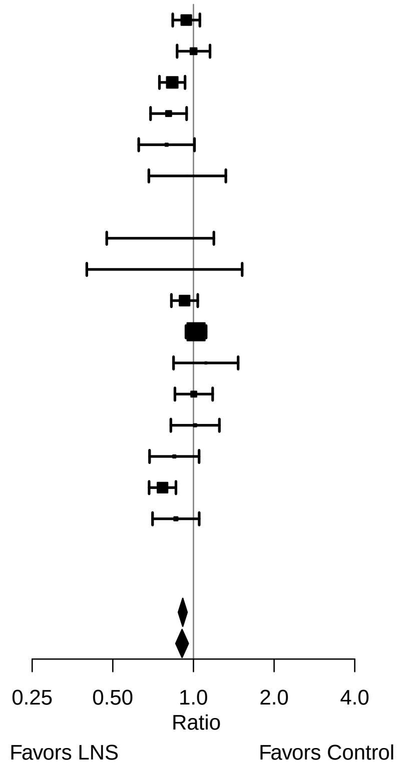

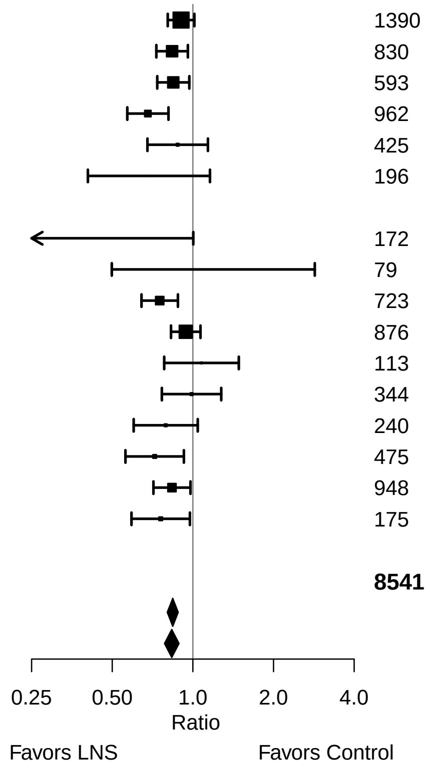

Female

Country	Trial	LNS N	Control N	Control Prevalence	PR (95% CI)	Fixed W	Random W
Bangladesh	JiVitA−4 (34)	1390	644	43.9	0.90 (0.81, 1.01)	0.17	0.13
Bangladesh	RDNS (35)	830	404	41.3	0.84 (0.73, 0.96)	0.12	0.11
Bangladesh	WASH−B (36)	593	1717	40.7	0.85 (0.74, 0.97)	0.12	0.11
Burkina Faso	iLiNS−Zinc (37)	962	328	35.6	0.68 (0.57, 0.81)	0.07	0.08
Burkina Faso	PROMIS (38)	425	431	24.1	0.88 (0.68, 1.14)	0.03	0.05
Burkina Faso	PROMIS CS (38)	196	217	19.4	0.69 (0.41, 1.16)	0.01	0.01
Ghana	GHANA (39)						
Ghana	iLiNS−DYADG (40)	172	369	9.8	0.48 (0.23, 1.00)	0.00	0.01
Haiti	HAITI (41)	79	90	10.0	1.19 (0.50, 2.85)	0.00	0.01
Kenya	WASH−B (42)	723	2697	28.1	0.75 (0.64, 0.88)	0.09	0.09
Madagascar	MAHAY (43)	876	850	53.8	0.94 (0.83, 1.07)	0.14	0.11
Malawi	iLiNS−DYADM (44)	113	237	31.2	1.08 (0.78, 1.48)	0.02	0.03
Malawi	iLiNS−DOSE (45)	344	117	37.6	0.99 (0.77, 1.28)	0.03	0.05
Mali	PROMIS (46)	240	243	33.7	0.79 (0.60, 1.04)	0.03	0.04
Mali	PROMIS CS (46)	475	444	30.4	0.72 (0.56, 0.93)	0.04	0.05
Zimbabwe	SHINE (HIV−) (47)	948	886	27.7	0.84 (0.71, 0.98)	0.09	0.09
Zimbabwe	SHINE (HIV+) (48)	175	161	46.0	0.76 (0.59, 0.97)	0.04	0.05
		**8541**	**9835**		**I² = 0.31, Tau² = 0.00**		
**Fixed**					**0.84 (0.80, 0.88)**		
**Random**					**0.83 (0.78, 0.89)**		

Supplemental figure 8B: Stunting prevalence ratio

8B7: Stratified by Child birth order

P−for−interaction = 0.054
Ratio of PRs = 1.06 (1.00, 1.13)

Country	Trial	Later born LNS N	Later born Control N	Later born Control Prevalence	Later born PR (95% CI)	Later born Fixed W	Later born Random W	Firstborn LNS N	Firstborn Control N	Firstborn Control Prevalence	Firstborn PR (95% CI)	Firstborn Fixed W	Firstborn Random W
Bangladesh	JiVitA−4 (34)	611	255	49.0	0.84 (0.73, 0.98)	0.06	0.07	2223	982	42.8	0.94 (0.85, 1.04)	0.27	0.12
Bangladesh	RDNS (35)	974	508	42.7	0.89 (0.79, 0.99)	0.11	0.09	688	307	40.7	0.97 (0.83, 1.14)	0.11	0.10
Bangladesh	WASH−B (36)	736	2290	43.4	0.84 (0.76, 0.92)	0.16	0.11	422	1141	38.8	0.85 (0.72, 0.99)	0.11	0.10
Burkina Faso	iLiNS−Zinc (37)	1520	519	38.8	0.71 (0.60, 0.84)	0.05	0.06	432	145	41.4	0.88 (0.73, 1.06)	0.08	0.09
Burkina Faso	PROMIS (38)	715	754	30.1	0.81 (0.65, 1.01)	0.03	0.04	148	158	32.3	0.88 (0.62, 1.26)	0.02	0.05
Burkina Faso	PROMIS CS (38)	366	359	23.7	0.89 (0.65, 1.22)	0.01	0.03	63	80	27.5	0.81 (0.44, 1.48)	0.01	0.02
Ghana	GHANA (39)												
Ghana	iLiNS−DYADG (40)	237	458	13.3	0.79 (0.51, 1.23)	0.01	0.01	110	234	12.4	0.37 (0.15, 0.92)	0.00	0.01
Haiti	HAITI (41)	88	99	15.2	0.86 (0.46, 1.61)	0.00	0.01	61	50	10.0	1.71 (0.65, 4.48)	0.00	0.01
Kenya	WASH−B (42)	1134	4045	32.1	0.88 (0.80, 0.97)	0.14	0.10	321	1087	32.7	0.76 (0.62, 0.94)	0.06	0.08
Madagascar	MAHAY (43)	1272	1217	59.7	0.94 (0.86, 1.04)	0.16	0.11	430	465	55.3	1.09 (0.96, 1.25)	0.15	0.11
Malawi	iLiNS−DYADM (44)	173	353	35.7	1.12 (0.89, 1.41)	0.03	0.04	47	89	30.3	1.05 (0.62, 1.77)	0.01	0.03
Malawi	iLiNS−DOSE (45)	458	165	44.2	1.12 (0.95, 1.32)	0.05	0.06	149	45	53.3	0.84 (0.60, 1.16)	0.03	0.05
Mali	PROMIS (46)	442	445	37.1	0.86 (0.72, 1.03)	0.04	0.06	63	59	30.5	1.43 (0.86, 2.36)	0.01	0.03
Mali	PROMIS CS (46)	790	831	32.1	0.78 (0.65, 0.95)	0.04	0.05	145	126	42.9	0.72 (0.53, 0.98)	0.03	0.06
Zimbabwe	SHINE (HIV−) (47)	1302	1223	33.5	0.81 (0.71, 0.91)	0.09	0.09	491	487	37.8	0.78 (0.65, 0.93)	0.08	0.09
Zimbabwe	SHINE (HIV+) (48)	280	247	46.6	0.87 (0.71, 1.05)	0.04	0.05	54	78	59.0	0.66 (0.45, 0.96)	0.02	0.04
		**11098**	**13768**		**I^2 = 0.43, Tau^2 = 0.01**			**5847**	**5533**		**I^2 = 0.48, Tau^2 = 0.02**		
**Fixed**					**0.87 (0.84, 0.91)**						**0.91 (0.86, 0.96)**		
**Random**					**0.87 (0.82, 0.92)**						**0.88 (0.80, 0.97)**		

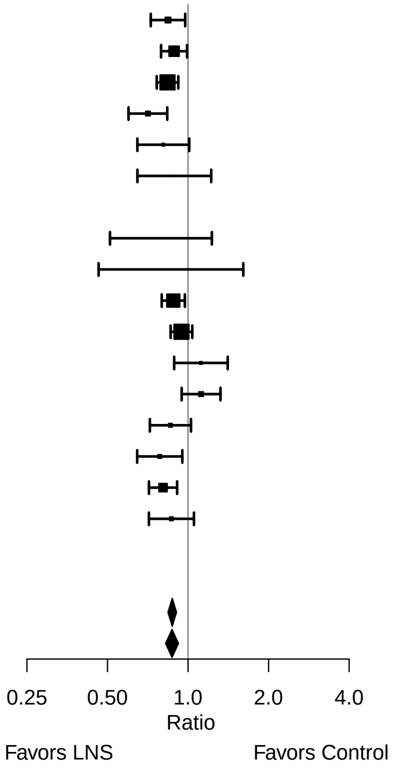

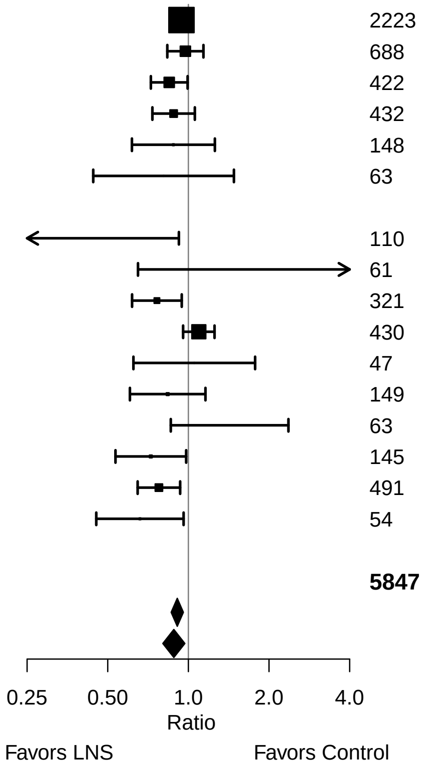

Supplemental figure 8B: Stunting prevalence ratio

8B8: Stratified by Child baseline anthropometric status

P−for−interaction = 0.006
Ratio of PRs = 1.25 (1.07, 1.46)

Country	Trial	LAZ at least −1 Z: LNS N	Control N	Control Prevalence	PR (95% CI)	Fixed W	Random W	LAZ less than −1 Z: LNS N	Control N	Control Prevalence	PR (95% CI)	Fixed W	Random W
Bangladesh	JiVitA−4 (34)	1055	454	8.8	0.79 (0.52, 1.18)	0.15	0.15	1783	790	64.9	0.94 (0.87, 1.01)	0.30	0.19
Bangladesh	RDNS (35)	630	306	9.5	0.72 (0.47, 1.10)	0.14	0.14	971	453	63.8	0.92 (0.85, 1.00)	0.23	0.18
Bangladesh	WASH−B (36)												
Burkina Faso	iLiNS−Zinc (37)	839	291	6.2	0.40 (0.19, 0.86)	0.04	0.04	1113	373	65.4	0.76 (0.65, 0.88)	0.07	0.10
Burkina Faso	PROMIS (38)	444	484	12.4	0.69 (0.48, 0.99)	0.19	0.19	257	274	58.4	0.93 (0.77, 1.12)	0.04	0.07
Burkina Faso	PROMIS CS (38)												
Ghana	GHANA (39)												
Ghana	iLiNS−DYADG (40)												
Haiti	HAITI (41)												
Kenya	WASH−B (42)												
Madagascar	MAHAY (43)												
Malawi	iLiNS−DYADM (44)	82	179	8.4	1.31 (0.60, 2.87)	0.04	0.04	122	244	53.7	1.10 (0.91, 1.33)	0.04	0.07
Malawi	iLiNS−DOSE (45)	260	79	8.9	1.13 (0.51, 2.50)	0.04	0.04	436	162	64.8	0.96 (0.84, 1.10)	0.09	0.11
Mali	PROMIS (46)	296	266	13.2	0.87 (0.51, 1.50)	0.09	0.09	210	240	62.1	0.88 (0.74, 1.05)	0.05	0.08
Mali	PROMIS CS (46)												
Zimbabwe	SHINE (HIV−) (47)	725	638	12.9	0.71 (0.51, 0.98)	0.24	0.24	602	571	54.6	0.87 (0.78, 0.97)	0.14	0.14
Zimbabwe	SHINE (HIV+) (48)	103	107	25.2	0.50 (0.27, 0.91)	0.07	0.07	165	131	65.6	0.88 (0.73, 1.05)	0.05	0.07
		**4434**	**2804**		**I² = 0.00, Tau² = 0.00**			**5659**	**3238**		**I² = 0.34, Tau² = 0.00**		
**Fixed**					**0.73 (0.62, 0.85)**						**0.91 (0.88, 0.95)**		
**Random**					**0.73 (0.62, 0.85)**						**0.91 (0.86, 0.96)**		

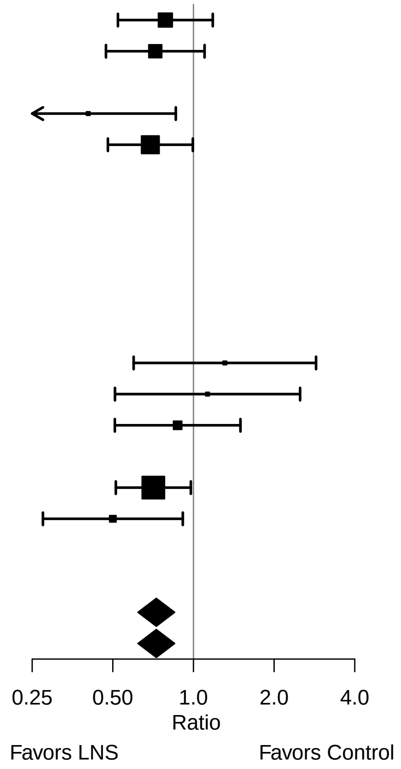

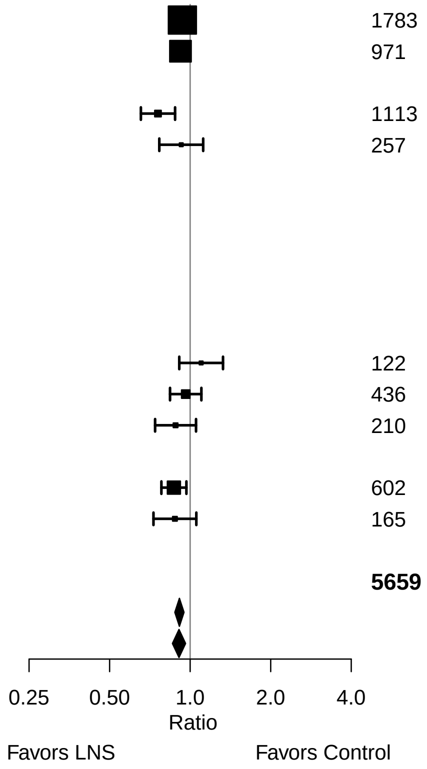

Supplemental figure 8C: Stunting prevalence difference

8C1: Stratified by Maternal height

P−for−interaction = 0.339
Difference in PDs = 0.02 (−0.02, 0.05)

Country	Trial	At least 150.1 cm: LNS N	Control N	Control Prevalence	PD (95% CI)	Fixed W	Random W	Less than 150.1 cm: LNS N	Control N	Control Prevalence	PD (95% CI)	Fixed W	Random W
Bangladesh	JiVitA−4 (34)												
Bangladesh	RDNS (35)	868	432	30.6	−0.04 (−0.10, 0.02)	0.06	0.08	735	358	56.1	−0.03 (−0.09, 0.03)	0.24	0.24
Bangladesh	WASH−B (36)	616	1883	32.4	−0.08 (−0.12, −0.04)	0.13	0.12	542	1545	53.5	−0.06 (−0.11, −0.02)	0.39	0.39
Burkina Faso	iLiNS−Zinc (37)	1916	649	38.8	−0.11 (−0.15, −0.06)	0.10	0.11	34	15	66.7	−0.02 (−0.28, 0.24)	0.01	0.01
Burkina Faso	PROMIS (38)	835	884	29.6	−0.06 (−0.12, 0.00)	0.06	0.08	25	25	68.0	−0.08 (−0.48, 0.32)	0.01	0.01
Burkina Faso	PROMIS CS (38)												
Ghana	GHANA (39)												
Ghana	iLiNS−DYADG (40)												
Haiti	HAITI (41)												
Kenya	WASH−B (42)	1313	4686	30.7	−0.04 (−0.07, −0.01)	0.23	0.15	57	190	58.4	−0.09 (−0.25, 0.07)	0.03	0.03
Madagascar	MAHAY (43)	1004	1037	53.9	−0.03 (−0.09, 0.02)	0.06	0.09	598	565	68.8	0.00 (−0.06, 0.07)	0.19	0.19
Malawi	iLiNS−DYADM (44)	192	381	29.9	0.04 (−0.04, 0.12)	0.03	0.06	28	60	61.7	0.06 (−0.16, 0.28)	0.02	0.02
Malawi	iLiNS−DOSE (45)	580	198	41.9	−0.02 (−0.08, 0.04)	0.05	0.08	115	42	66.7	0.01 (−0.12, 0.14)	0.05	0.05
Mali	PROMIS (46)												
Mali	PROMIS CS (46)	914	936	32.9	−0.08 (−0.13, −0.02)	0.07	0.09	33	27	66.7	−0.06 (−0.26, 0.13)	0.02	0.02
Zimbabwe	SHINE (HIV−) (47)	1755	1697	34.0	−0.08 (−0.11, −0.05)	0.21	0.14	76	62	61.3	−0.06 (−0.21, 0.09)	0.04	0.04
Zimbabwe	SHINE (HIV+) (48)												
		**9993**	**12783**		**I² = 0.48, Tau² = 0.00**			**2243**	**2889**		**I² = 0.00, Tau² = 0.00**		
**Fixed**					**−0.06 (−0.07, −0.05)**						**−0.04 (−0.07, −0.01)**		
**Random**					**−0.06 (−0.08, −0.03)**						**−0.04 (−0.07, −0.01)**		

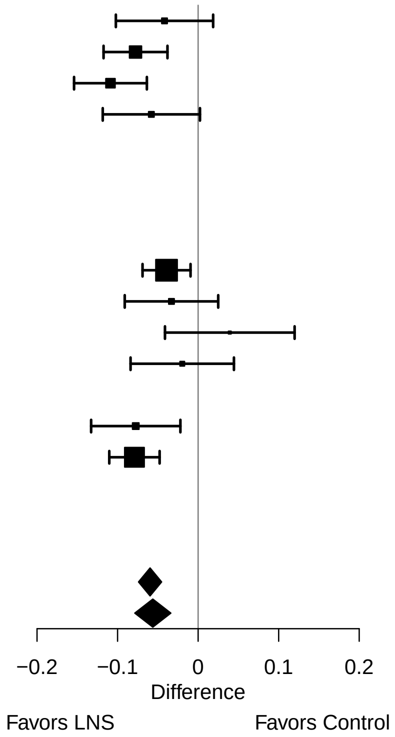

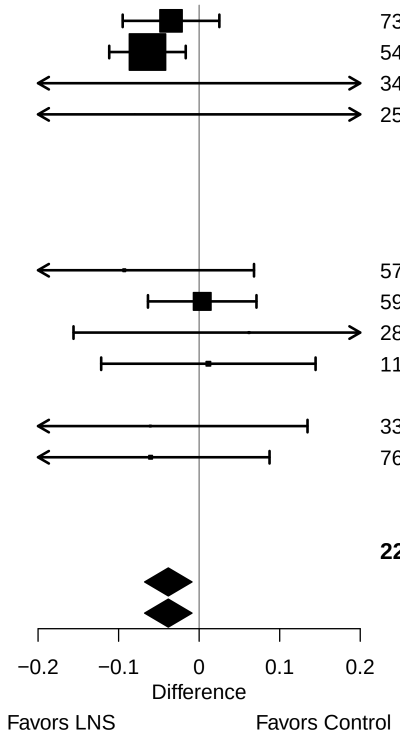

Supplemental figure 8C: Stunting prevalence difference

8C2: Stratified by Maternal BMI

P−for−interaction = 0.306
Difference in PDs = 0.01 (−0.01, 0.04)

		At least 20 kg/m²						Less than 20 kg/m²					
**Country**	**Trial**	**LNS N**	**Control N**	**Control Prevalence**	**PD (95% CI)**	**Fixed W**	**Random W**	**LNS N**	**Control N**	**Control Prevalence**	**PD (95% CI)**	**Fixed W**	**Random W**
Bangladesh	JiVitA−4 (34)												
Bangladesh	RDNS (35)	730	335	38.8	−0.05 (−0.11, 0.01)	0.06	0.08	873	455	44.6	−0.02 (−0.07, 0.02)	0.22	0.17
Bangladesh	WASH−B (36)	519	1576	37.3	−0.06 (−0.11, −0.01)	0.08	0.09	639	1852	45.8	−0.08 (−0.12, −0.03)	0.22	0.17
Burkina Faso	iLiNS−Zinc (37)	1214	390	37.1	−0.12 (−0.16, −0.08)	0.15	0.12	736	274	42.7	−0.08 (−0.15, −0.02)	0.10	0.11
Burkina Faso	PROMIS (38)	647	675	29.9	−0.09 (−0.15, −0.03)	0.06	0.08	213	233	32.6	0.04 (−0.07, 0.14)	0.04	0.05
Burkina Faso	PROMIS CS (38)	246	269	21.6	−0.02 (−0.10, 0.05)	0.04	0.06	184	166	28.3	−0.04 (−0.14, 0.05)	0.05	0.06
Ghana	GHANA (39)												
Ghana	iLiNS−DYADG (40)												
Haiti	HAITI (41)												
Kenya	WASH−B (42)	1098	3803	30.6	−0.03 (−0.06, 0.00)	0.21	0.13	272	1073	36.1	−0.08 (−0.14, −0.01)	0.11	0.11
Madagascar	MAHAY (43)												
Malawi	iLiNS−DYADM (44)	131	263	33.8	0.04 (−0.06, 0.14)	0.02	0.04	88	178	34.8	0.04 (−0.08, 0.16)	0.03	0.04
Malawi	iLiNS−DOSE (45)	513	174	44.8	−0.02 (−0.08, 0.05)	0.05	0.07	180	65	50.8	−0.02 (−0.14, 0.09)	0.03	0.04
Mali	PROMIS (46)	380	370	34.9	−0.05 (−0.12, 0.01)	0.04	0.07	120	135	40.7	0.01 (−0.08, 0.10)	0.05	0.07
Mali	PROMIS CS (46)	702	684	31.1	−0.05 (−0.12, 0.01)	0.05	0.08	245	279	40.5	−0.12 (−0.21, −0.04)	0.06	0.08
Zimbabwe	SHINE (HIV−) (47)	1364	1305	35.9	−0.09 (−0.13, −0.06)	0.20	0.13	240	249	34.1	−0.01 (−0.09, 0.07)	0.07	0.08
Zimbabwe	SHINE (HIV+) (48)	247	254	48.8	−0.12 (−0.21, −0.03)	0.03	0.05	62	52	53.8	−0.04 (−0.22, 0.15)	0.01	0.02
		**7791**	**10098**		**I² = 0.57, Tau² = 0.00**			**3852**	**5011**		**I² = 0.26, Tau² = 0.00**		
**Fixed**					**−0.07 (−0.08, −0.05)**						**−0.05 (−0.07, −0.03)**		
**Random**					**−0.06 (−0.09, −0.04)**						**−0.04 (−0.07, −0.02)**		

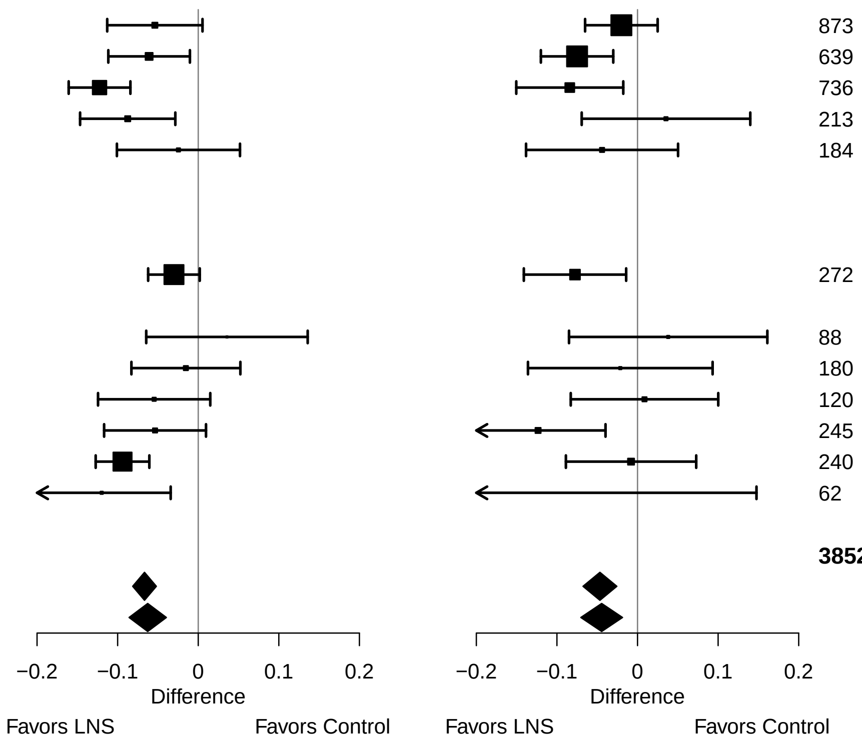

Supplemental figure 8C: Stunting prevalence difference

8C3: Stratified by Maternal age

P−for−interaction = 0.541
Difference in PDs = −0.01 (−0.03, 0.01)

Country	Trial	At least 25 y: LNS N	Control N	Control Prevalence	PD (95% CI)	Fixed W	Random W	Less than 25 y: LNS N	Control N	Control Prevalence	PD (95% CI)	Fixed W	Random W
Bangladesh	JiVitA−4 (34)	1218	546	46.3	−0.04 (−0.09, 0.00)	0.10	0.09	1608	693	42.6	−0.03 (−0.07, 0.01)	0.14	0.11
Bangladesh	RDNS (35)	453	221	40.7	−0.08 (−0.16, −0.01)	0.04	0.05	1210	594	42.4	−0.02 (−0.05, 0.02)	0.14	0.11
Bangladesh	WASH−B (36)	492	1512	42.7	−0.06 (−0.10, −0.02)	0.11	0.09	661	1903	41.4	−0.07 (−0.12, −0.03)	0.11	0.10
Burkina Faso	iLiNS−Zinc (37)	1131	399	38.0	−0.11 (−0.16, −0.06)	0.08	0.08	808	262	41.6	−0.10 (−0.15, −0.05)	0.09	0.09
Burkina Faso	PROMIS (38)	485	526	30.8	−0.07 (−0.14, 0.00)	0.04	0.05	378	388	30.2	−0.03 (−0.10, 0.04)	0.04	0.05
Burkina Faso	PROMIS CS (38)	202	192	22.4	−0.03 (−0.10, 0.04)	0.04	0.05	228	247	25.9	−0.03 (−0.12, 0.06)	0.03	0.04
Ghana	GHANA (39)												
Ghana	iLiNS−DYADG (40)	220	416	11.1	−0.01 (−0.06, 0.04)	0.08	0.08	127	276	15.9	−0.10 (−0.17, −0.04)	0.04	0.06
Haiti	HAITI (41)	101	103	9.7	−0.01 (−0.08, 0.06)	0.04	0.05	47	45	22.2	−0.02 (−0.14, 0.09)	0.02	0.03
Kenya	WASH−B (42)	844	2811	30.1	−0.03 (−0.07, 0.00)	0.16	0.11	598	2294	34.8	−0.07 (−0.11, −0.03)	0.14	0.11
Madagascar	MAHAY (43)	896	949	59.0	−0.02 (−0.07, 0.04)	0.06	0.06	805	733	57.8	0.00 (−0.07, 0.06)	0.05	0.06
Malawi	iLiNS−DYADM (44)	106	226	38.9	0.02 (−0.10, 0.13)	0.02	0.02	114	218	30.3	0.06 (−0.05, 0.16)	0.02	0.03
Malawi	iLiNS−DOSE (45)	368	134	41.8	0.03 (−0.05, 0.11)	0.03	0.04	314	100	51.0	−0.05 (−0.14, 0.04)	0.03	0.04
Mali	PROMIS (46)	346	359	36.8	−0.05 (−0.11, 0.00)	0.06	0.06	160	147	35.4	0.00 (−0.11, 0.12)	0.02	0.03
Mali	PROMIS CS (46)	503	507	31.0	−0.06 (−0.13, 0.02)	0.04	0.05	449	462	36.8	−0.09 (−0.16, −0.02)	0.05	0.06
Zimbabwe	SHINE (HIV−) (47)	875	800	32.0	−0.07 (−0.11, −0.02)	0.10	0.09	808	797	38.5	−0.08 (−0.13, −0.04)	0.10	0.09
Zimbabwe	SHINE (HIV+) (48)	244	231	50.2	−0.11 (−0.20, −0.02)	0.03	0.03	73	78	50.0	−0.03 (−0.19, 0.12)	0.01	0.01
		**8484**	**9932**		**I^2 = 0.30, Tau^2 = 0.00**			**8388**	**9237**		**I^2 = 0.35, Tau^2 = 0.00**		
**Fixed**					**−0.05 (−0.06, −0.03)**						**−0.05 (−0.07, −0.04)**		
**Random**					**−0.05 (−0.06, −0.03)**						**−0.05 (−0.07, −0.03)**		

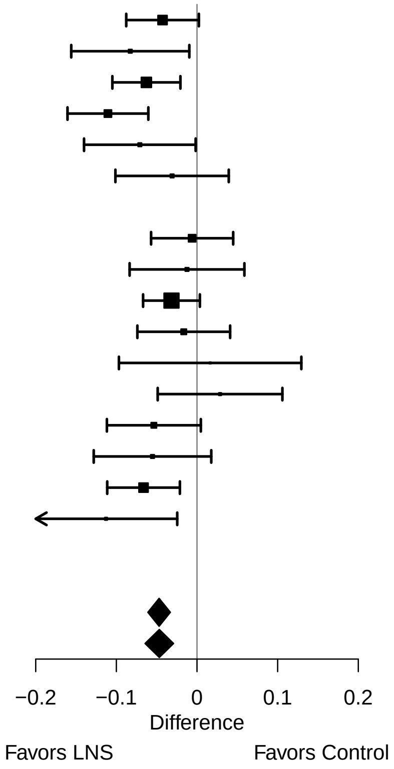

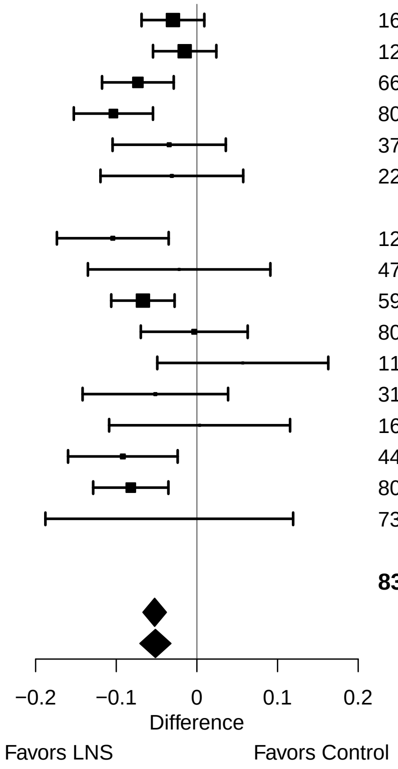

Supplemental figure 8C: Stunting prevalence difference

8C4: Stratified by Maternal education

P−for−interaction = 0.233
Difference in PDs = 0.02 (−0.01, 0.04)

Country	Trial	LNS N	Control N	Control Prevalence	Primary or greater PD (95% CI)	Fixed W	Random W	LNS N	Control N	Control Prevalence	Incomplete or no formal PD (95% CI)	Fixed W	Random W
Bangladesh	JiVitA−4 (34)	1830	756	40.1	−0.04 (−0.08, −0.01)	0.16	0.16	1006	485	50.8	−0.02 (−0.07, 0.03)	0.11	0.09
Bangladesh	RDNS (35)	1245	589	40.2	−0.05 (−0.09, 0.00)	0.10	0.10	418	226	46.5	0.01 (−0.05, 0.08)	0.06	0.07
Bangladesh	WASH−B (36)	813	2452	38.4	−0.08 (−0.12, −0.05)	0.18	0.18	345	979	50.7	−0.04 (−0.10, 0.03)	0.06	0.07
Burkina Faso	iLiNS−Zinc (37)	82	16	31.2	−0.15 (−0.39, 0.09)	0.00	0.00	1857	645	39.5	−0.11 (−0.15, −0.06)	0.12	0.10
Burkina Faso	PROMIS (38)	70	54	27.8	0.04 (−0.14, 0.21)	0.01	0.01	793	860	30.7	−0.06 (−0.12, −0.01)	0.08	0.08
Burkina Faso	PROMIS CS (38)	34	39	25.6	−0.08 (−0.23, 0.07)	0.01	0.01	393	399	24.3	−0.03 (−0.10, 0.05)	0.04	0.06
Ghana	GHANA (39)												
Ghana	iLiNS−DYADG (40)	267	548	13.7	−0.06 (−0.11, −0.01)	0.09	0.09	80	144	10.4	0.01 (−0.08, 0.09)	0.03	0.05
Haiti	HAITI (41)												
Kenya	WASH−B (42)	700	2434	26.0	−0.02 (−0.06, 0.01)	0.15	0.15	755	2699	37.8	−0.07 (−0.10, −0.03)	0.19	0.11
Madagascar	MAHAY (43)	351	431	56.8	−0.02 (−0.10, 0.06)	0.03	0.03	1351	1251	59.1	−0.01 (−0.06, 0.05)	0.08	0.08
Malawi	iLiNS−DYADM (44)	35	69	29.0	0.00 (−0.19, 0.18)	0.01	0.01	184	372	35.8	0.04 (−0.04, 0.13)	0.03	0.05
Malawi	iLiNS−DOSE (45)	162	56	44.6	−0.10 (−0.21, 0.01)	0.02	0.02	522	178	46.1	0.02 (−0.05, 0.09)	0.05	0.07
Mali	PROMIS (46)	41	37	24.3	−0.07 (−0.23, 0.09)	0.01	0.01	464	469	37.3	−0.03 (−0.09, 0.03)	0.07	0.08
Mali	PROMIS CS (46)	107	96	32.3	−0.10 (−0.23, 0.04)	0.01	0.01	844	873	33.9	−0.07 (−0.13, −0.01)	0.07	0.08
Zimbabwe	SHINE (HIV−) (47)	1717	1644	34.8	−0.07 (−0.10, −0.04)	0.21	0.21	69	61	36.1	−0.04 (−0.20, 0.11)	0.01	0.02
Zimbabwe	SHINE (HIV+) (48)	301	293	48.5	−0.08 (−0.16, 0.01)	0.03	0.03	21	18	66.7	−0.29 (−0.58, 0.01)	0.00	0.01
		**7755**	**9514**		**I² = 0.00, Tau² = 0.00**			**9102**	**9659**		**I² = 0.49, Tau² = 0.00**		
**Fixed**					**−0.06 (−0.07, −0.04)**						**−0.04 (−0.06, −0.03)**		
**Random**					**−0.06 (−0.07, −0.04)**						**−0.03 (−0.06, −0.01)**		

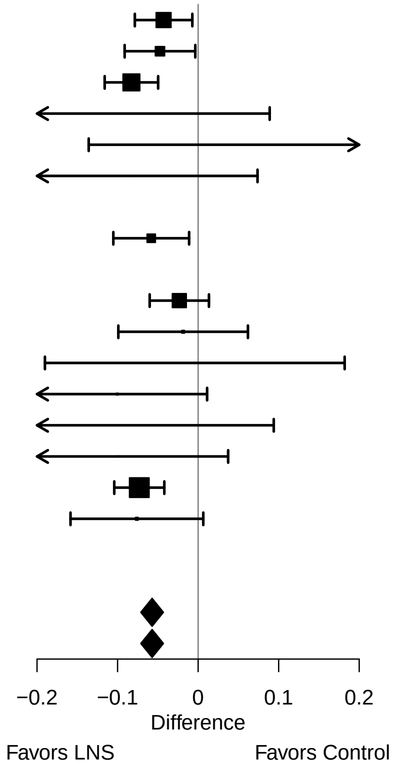

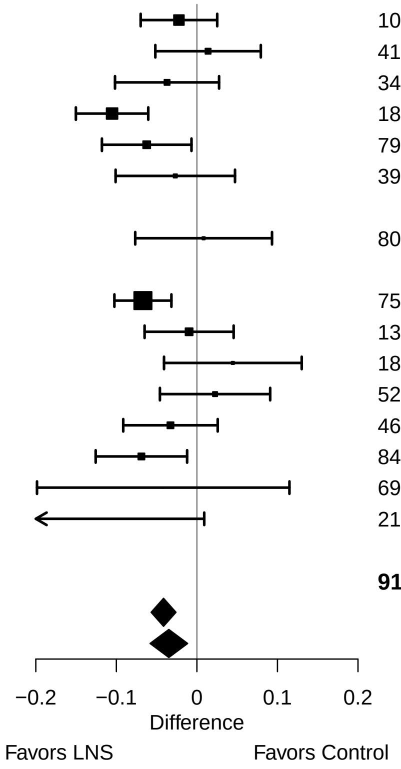

Supplemental figure 8C: Stunting prevalence difference

8C5: Stratified by Maternal depressive symptoms

-for−interaction = 0.010
fference in PDs = −0.04 (−0.07, −0.01)

		Less than 75th percentile						At least 75th percentile					
**ountry**	**Trial**	**LNS N**	**Control N**	**Control Prevalence**	**PD (95% CI)**	**Fixed W**	**Random W**	**LNS N**	**Control N**	**Control Prevalence**	**PD (95% CI)**	**Fixed W**	**Random W**
ıngladesh	JiVitA−4 (34)												
ıngladesh	RDNS (35)	1081	472	42.4	−0.05 (−0.09, −0.01)	0.15	0.15	520	288	41.0	0.00 (−0.08, 0.08)	0.09	0.10
ıngladesh	WASH−B (36)	897	2462	41.3	−0.08 (−0.12, −0.05)	0.19	0.19	237	878	43.8	−0.02 (−0.10, 0.05)	0.12	0.12
ırkina Faso	iLiNS−Zinc (37)												
ırkina Faso	PROMIS (38)	508	573	30.7	−0.07 (−0.15, 0.01)	0.04	0.04	223	215	28.8	0.01 (−0.08, 0.09)	0.09	0.11
ırkina Faso	PROMIS CS (38)												
ıana	GHANA (39)												
ıana	iLiNS−DYADG (40)	252	459	13.3	−0.07 (−0.11, −0.02)	0.11	0.11	83	209	10.5	0.04 (−0.04, 0.12)	0.10	0.11
ıiti	HAITI (41)												
·nya	WASH−B (42)	996	3496	31.1	−0.04 (−0.08, −0.01)	0.20	0.20	339	1235	34.3	−0.02 (−0.08, 0.04)	0.20	0.14
ıadagascar	MAHAY (43)	673	721	60.5	−0.04 (−0.11, 0.03)	0.05	0.05	284	252	60.3	0.05 (−0.04, 0.13)	0.09	0.11
ılawi	iLiNS−DYADM (44)	157	303	31.7	0.01 (−0.08, 0.10)	0.03	0.03	47	113	39.8	0.18 (0.01, 0.34)	0.02	0.04
ılawi	iLiNS−DOSE (45)												
ıli	PROMIS (46)	379	373	35.1	−0.04 (−0.10, 0.03)	0.06	0.06	123	131	40.5	−0.04 (−0.15, 0.06)	0.06	0.09
ıli	PROMIS CS (46)												
ımbabwe	SHINE (HIV−) (47)	1198	1145	33.8	−0.06 (−0.10, −0.02)	0.15	0.15	513	489	38.2	−0.11 (−0.17, −0.05)	0.20	0.14
ımbabwe	SHINE (HIV+) (48)	234	215	49.3	−0.12 (−0.22, −0.02)	0.03	0.03	89	100	53.0	−0.01 (−0.17, 0.14)	0.03	0.05
		**6375**	**10219**		**I² = 0.00, Tau² = 0.00**			**2458**	**3910**		**I² = 0.56, Tau² = 0.00**		
**xed**					**−0.06 (−0.07, −0.04)**						**−0.02 (−0.04, 0.01)**		
**ındom**					**−0.06 (−0.07, −0.04)**						**−0.01 (−0.05, 0.03)**		

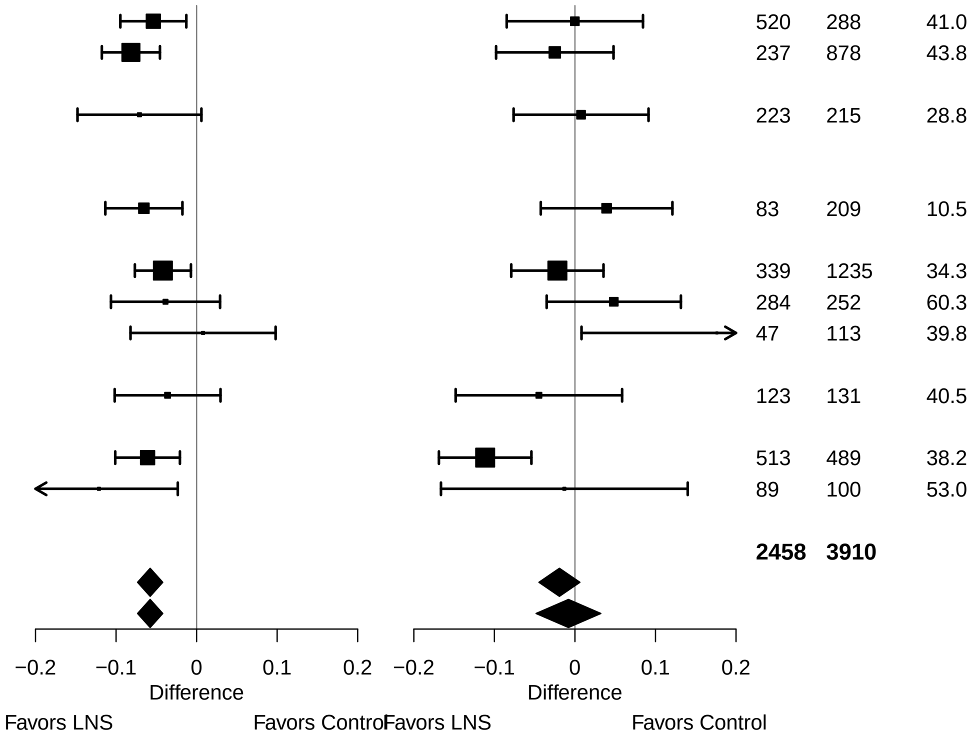

Supplemental figure 8C: Stunting prevalence difference

8C6: Stratified by Child sex

P−for−interaction = 0.187
Difference in PDs = −0.01 (−0.03, 0.01)

Country	Trial	Male LNS N	Male Control N	Male Control Prevalence	Male PD (95% CI)	Male Fixed W	Male Random W	Female LNS N	Female Control N	Female Control Prevalence	Female PD (95% CI)	Female Fixed W	Female Random W
Bangladesh	JiVitA−4 (34)	1448	600	44.5	−0.03 (−0.07, 0.01)	0.13	0.10	1390	644	43.9	−0.04 (−0.08, 0.00)	0.13	0.12
Bangladesh	RDNS (35)	833	411	42.6	0.00 (−0.06, 0.06)	0.06	0.07	830	404	41.3	−0.07 (−0.12, −0.02)	0.07	0.07
Bangladesh	WASH−B (36)	565	1714	43.1	−0.07 (−0.11, −0.03)	0.13	0.10	593	1717	40.7	−0.06 (−0.11, −0.01)	0.08	0.08
Burkina Faso	iLiNS−Zinc (37)	990	336	43.2	−0.10 (−0.15, −0.04)	0.07	0.08	962	328	35.6	−0.12 (−0.17, −0.07)	0.07	0.07
Burkina Faso	PROMIS (38)	438	483	36.2	−0.07 (−0.15, 0.01)	0.03	0.04	425	431	24.1	−0.03 (−0.09, 0.03)	0.06	0.06
Burkina Faso	PROMIS CS (38)	234	222	29.3	−0.02 (−0.11, 0.08)	0.02	0.03	196	217	19.4	−0.06 (−0.14, 0.02)	0.03	0.03
Ghana	GHANA (39)												
Ghana	iLiNS−DYADG (40)	175	323	16.7	−0.04 (−0.11, 0.02)	0.05	0.06	172	369	9.8	−0.05 (−0.10, 0.00)	0.08	0.08
Haiti	HAITI (41)	70	59	18.6	−0.05 (−0.15, 0.06)	0.02	0.03	79	90	10.0	−0.01 (−0.08, 0.06)	0.04	0.04
Kenya	WASH−B (42)	734	2440	36.8	−0.03 (−0.07, 0.01)	0.14	0.11	723	2697	28.1	−0.07 (−0.11, −0.03)	0.15	0.14
Madagascar	MAHAY (43)	826	832	63.3	0.01 (−0.04, 0.07)	0.07	0.07	876	850	53.8	−0.03 (−0.10, 0.03)	0.04	0.05
Malawi	iLiNS−DYADM (44)	107	207	38.6	0.04 (−0.07, 0.16)	0.02	0.02	113	237	31.2	0.02 (−0.08, 0.13)	0.02	0.02
Malawi	iLiNS−DOSE (45)	352	124	54.8	−0.02 (−0.10, 0.06)	0.03	0.04	344	117	37.6	−0.01 (−0.09, 0.07)	0.03	0.03
Mali	PROMIS (46)	266	263	38.8	−0.02 (−0.09, 0.06)	0.04	0.05	240	243	33.7	−0.06 (−0.14, 0.02)	0.03	0.03
Mali	PROMIS CS (46)	477	525	36.6	−0.06 (−0.13, 0.01)	0.04	0.05	475	444	30.4	−0.09 (−0.15, −0.02)	0.04	0.04
Zimbabwe	SHINE (HIV−) (47)	932	908	41.9	−0.10 (−0.14, −0.06)	0.12	0.10	948	886	27.7	−0.05 (−0.09, −0.01)	0.12	0.12
Zimbabwe	SHINE (HIV+) (48)	162	169	53.8	−0.08 (−0.18, 0.02)	0.02	0.03	175	161	46.0	−0.11 (−0.21, −0.01)	0.02	0.02
		**8609**	**9616**		**I² = 0.37, Tau² = 0.00**			**8541**	**9835**		**I² = 0.04, Tau² = 0.00**		
**Fixed**					**−0.05 (−0.06, −0.03)**						**−0.06 (−0.07, −0.04)**		
**Random**					**−0.04 (−0.06, −0.02)**						**−0.06 (−0.07, −0.04)**		

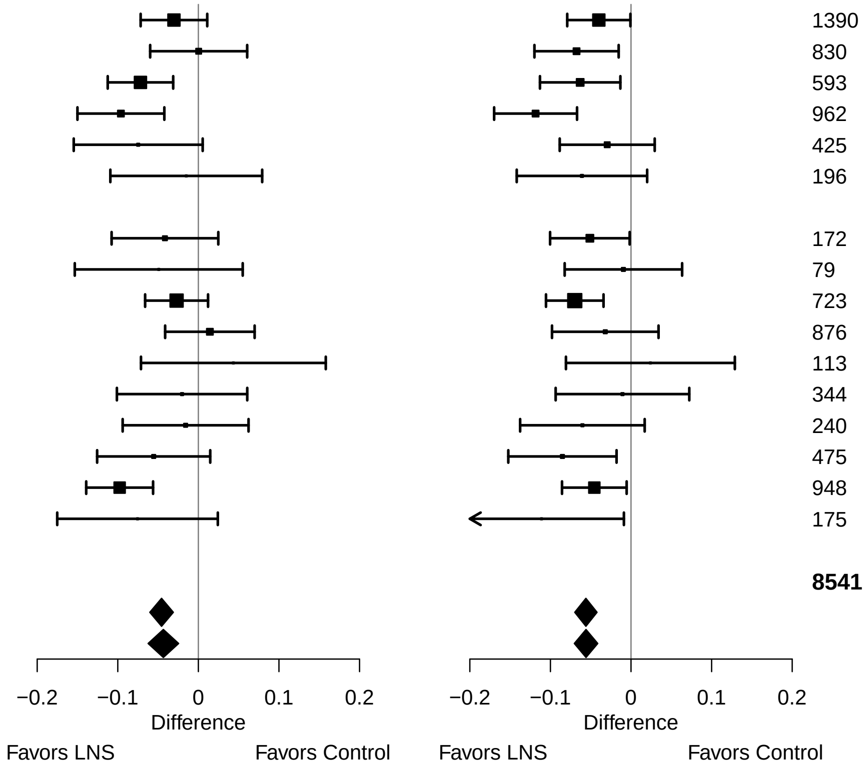

Supplemental figure 8C: Stunting prevalence difference

8C7: Stratified by Child birth order

P−for−interaction = 0.858
Difference in PDs = 0.00 (−0.02, 0.02)

Country	Trial	LNS N	Control N	Control Prevalence	Later born PD (95% CI)	Fixed W	Random W	LNS N	Control N	Control Prevalence	Firstborn PD (95% CI)	Fixed W	Random W
Bangladesh	JiVitA−4 (34)	611	255	49.0	−0.05 (−0.12, 0.01)	0.04	0.05	2223	982	42.8	−0.03 (−0.06, 0.00)	0.28	0.12
Bangladesh	RDNS (35)	974	508	42.7	−0.05 (−0.09, 0.00)	0.08	0.08	688	307	40.7	−0.01 (−0.07, 0.05)	0.07	0.08
Bangladesh	WASH−B (36)	736	2290	43.4	−0.07 (−0.11, −0.04)	0.13	0.10	422	1141	38.8	−0.06 (−0.11, 0.00)	0.10	0.09
Burkina Faso	iLiNS−Zinc (37)	1520	519	38.8	−0.11 (−0.16, −0.06)	0.07	0.07	432	145	41.4	−0.10 (−0.15, −0.04)	0.10	0.09
Burkina Faso	PROMIS (38)	715	754	30.1	−0.06 (−0.12, 0.00)	0.04	0.05	148	158	32.3	−0.04 (−0.15, 0.07)	0.03	0.05
Burkina Faso	PROMIS CS (38)	366	359	23.7	−0.03 (−0.10, 0.04)	0.03	0.04	63	80	27.5	−0.05 (−0.20, 0.10)	0.01	0.03
Ghana	GHANA (39)												
Ghana	iLiNS−DYADG (40)	237	458	13.3	−0.03 (−0.08, 0.02)	0.06	0.07	110	234	12.4	−0.08 (−0.15, −0.01)	0.07	0.08
Haiti	HAITI (41)	88	99	15.2	−0.03 (−0.11, 0.06)	0.02	0.03	61	50	10.0	0.00 (−0.09, 0.09)	0.04	0.06
Kenya	WASH−B (42)	1134	4045	32.1	−0.04 (−0.07, −0.01)	0.19	0.12	321	1087	32.7	−0.08 (−0.13, −0.02)	0.10	0.09
Madagascar	MAHAY (43)	1272	1217	59.7	−0.03 (−0.09, 0.02)	0.05	0.06	430	465	55.3	0.05 (−0.03, 0.13)	0.05	0.07
Malawi	iLiNS−DYADM (44)	173	353	35.7	0.04 (−0.05, 0.13)	0.02	0.03	47	89	30.3	0.02 (−0.15, 0.18)	0.01	0.02
Malawi	iLiNS−DOSE (45)	458	165	44.2	0.03 (−0.04, 0.10)	0.03	0.04	149	45	53.3	−0.11 (−0.24, 0.02)	0.02	0.04
Mali	PROMIS (46)	442	445	37.1	−0.06 (−0.11, 0.00)	0.05	0.06	63	59	30.5	0.11 (−0.05, 0.26)	0.01	0.03
Mali	PROMIS CS (46)	790	831	32.1	−0.07 (−0.12, −0.01)	0.05	0.06	145	126	42.9	−0.12 (−0.23, −0.01)	0.02	0.05
Zimbabwe	SHINE (HIV−) (47)	1302	1223	33.5	−0.06 (−0.10, −0.03)	0.12	0.10	491	487	37.8	−0.08 (−0.14, −0.03)	0.09	0.09
Zimbabwe	SHINE (HIV+) (48)	280	247	46.6	−0.06 (−0.15, 0.02)	0.02	0.03	54	78	59.0	−0.20 (−0.37, −0.04)	0.01	0.02
		**11098**	**13768**		**I^2 = 0.26, Tau^2 = 0.00**			**5847**	**5533**		**I^2 = 0.44, Tau^2 = 0.00**		
**Fixed**					**−0.05 (−0.06, −0.04)**						**−0.05 (−0.07, −0.03)**		
**Random**					**−0.05 (−0.06, −0.03)**						**−0.05 (−0.08, −0.02)**		

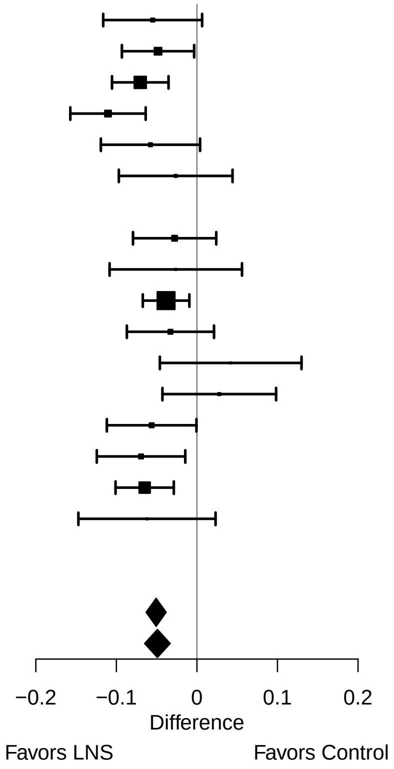

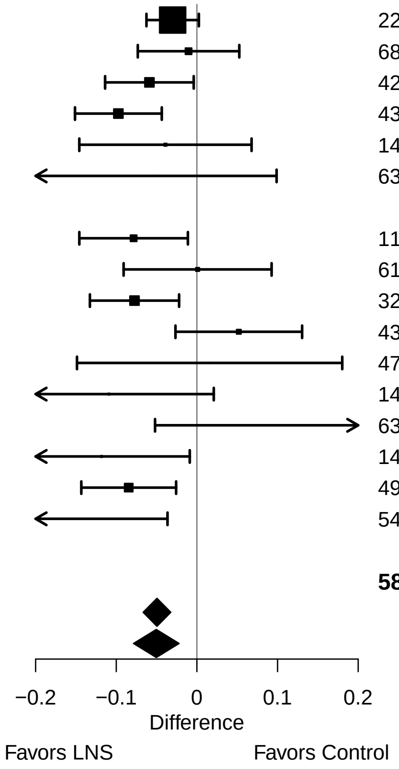

Supplemental figure 8C: Stunting prevalence difference

8C8: Stratified by Child baseline anthropometric status

P−for−interaction = 0.004
Difference in PDs = −0.04 (−0.06, −0.01)

		LAZ at least −1 Z						LAZ less than −1 Z					
**Country**	**Trial**	**LNS N**	**Control N**	**Control Prevalence**	**PD (95% CI)**	**Fixed W**	**Random W**	**LNS N**	**Control N**	**Control Prevalence**	**PD (95% CI)**	**Fixed W**	**Random W**
Bangladesh	JiVitA−4 (34)	1055	454	8.8	−0.02 (−0.05, 0.01)	0.19	0.19	1783	790	64.9	−0.04 (−0.09, 0.00)	0.26	0.20
Bangladesh	RDNS (35)	630	306	9.5	−0.03 (−0.06, 0.01)	0.16	0.16	971	453	63.8	−0.05 (−0.10, 0.00)	0.21	0.18
Bangladesh	WASH−B (36)												
Burkina Faso	iLiNS−Zinc (37)	839	291	6.2	−0.04 (−0.07, 0.00)	0.18	0.18	1113	373	65.4	−0.16 (−0.24, −0.07)	0.08	0.10
Burkina Faso	PROMIS (38)	444	484	12.4	−0.04 (−0.08, 0.00)	0.15	0.15	257	274	58.4	−0.04 (−0.15, 0.06)	0.05	0.07
Burkina Faso	PROMIS CS (38)												
Ghana	GHANA (39)												
Ghana	iLiNS−DYADG (40)												
Haiti	HAITI (41)												
Kenya	WASH−B (42)												
Madagascar	MAHAY (43)												
Malawi	iLiNS−DYADM (44)	82	179	8.4	0.03 (−0.05, 0.10)	0.04	0.04	122	244	53.7	0.05 (−0.05, 0.16)	0.05	0.07
Malawi	iLiNS−DOSE (45)	260	79	8.9	0.01 (−0.06, 0.09)	0.04	0.04	436	162	64.8	−0.02 (−0.11, 0.06)	0.07	0.09
Mali	PROMIS (46)	296	266	13.2	−0.02 (−0.08, 0.05)	0.05	0.05	210	240	62.1	−0.07 (−0.18, 0.03)	0.05	0.07
Mali	PROMIS CS (46)												
Zimbabwe	SHINE (HIV−) (47)	725	638	12.9	−0.04 (−0.07, 0.00)	0.17	0.17	602	571	54.6	−0.07 (−0.13, −0.02)	0.18	0.17
Zimbabwe	SHINE (HIV+) (48)	103	107	25.2	−0.13 (−0.23, −0.02)	0.02	0.02	165	131	65.6	−0.08 (−0.19, 0.03)	0.04	0.06
		**4434**	**2804**		**I² = 0.00, Tau² = 0.00**			**5659**	**3238**		**I² = 0.27, Tau² = 0.00**		
**Fixed**					**−0.03 (−0.04, −0.01)**						**−0.06 (−0.08, −0.03)**		
**Random**					**−0.03 (−0.04, −0.01)**						**−0.06 (−0.09, −0.02)**		

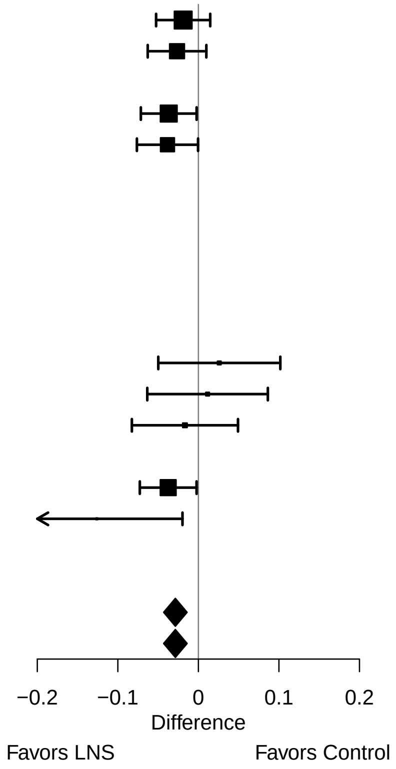

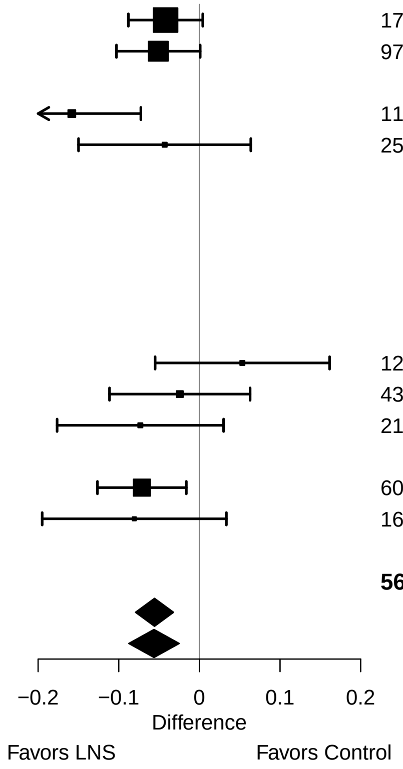

Supplemental figure 8D: Mean difference in WLZ

8D1: Stratified by Maternal height

P−for−interaction = 0.630
Difference in MDs = −0.02 (−0.09, 0.05)

		At least 150.1 cm						Less than 150.1 cm					
Country	Trial	LNS N	Control N	Control Mean	MD (95% CI)	Fixed W	Random W	LNS N	Control N	Control Mean	MD (95% CI)	Fixed W	Random W
Bangladesh	JiVitA−4 (34)												
Bangladesh	RDNS (35)	868	432	−1.07	0.09 (−0.01, 0.18)	0.08	0.08	733	358	−1.17	0.09 (−0.04, 0.21)	0.27	0.27
Bangladesh	WASH−B (36)	616	1880	−0.80	0.13 (0.04, 0.22)	0.09	0.08	540	1541	−0.96	0.13 (0.02, 0.23)	0.36	0.36
Burkina Faso	iLiNS−Zinc (37)	1916	649	−0.95	0.19 (0.12, 0.26)	0.14	0.09	34	15	−0.73	−0.32 (−0.73, 0.10)	0.02	0.02
Burkina Faso	PROMIS (38)	828	877	−0.70	0.10 (−0.02, 0.23)	0.05	0.07	25	25	−1.13	0.09 (−0.43, 0.61)	0.02	0.02
Burkina Faso	PROMIS CS (38)	419	423	−0.88	−0.11 (−0.24, 0.03)	0.04	0.06	11	9	−1.14	−0.12 (−1.06, 0.82)	0.00	0.00
Ghana	GHANA (39)												
Ghana	iLiNS−DYADG (40)	326	645	−0.57	0.09 (−0.04, 0.23)	0.04	0.06	16	36	−0.73	0.06 (−0.50, 0.63)	0.01	0.01
Haiti	HAITI (41)												
Kenya	WASH−B (42)	1311	4668	0.10	0.06 (−0.01, 0.12)	0.17	0.09	57	190	−0.06	0.10 (−0.22, 0.41)	0.04	0.04
Madagascar	MAHAY (43)	1002	1037	−0.37	−0.04 (−0.15, 0.07)	0.06	0.07	598	565	−0.52	0.05 (−0.11, 0.21)	0.15	0.15
Malawi	iLiNS−DYADM (44)	192	381	−0.12	−0.03 (−0.20, 0.14)	0.02	0.05	28	60	−0.20	−0.27 (−0.73, 0.19)	0.02	0.02
Malawi	iLiNS−DOSE (45)	580	198	−0.19	−0.01 (−0.15, 0.12)	0.04	0.06	115	42	−0.40	0.14 (−0.22, 0.50)	0.03	0.03
Mali	PROMIS (46)	474	492	−0.33	0.11 (−0.01, 0.24)	0.05	0.07	19	9	−0.51	−0.13 (−0.72, 0.46)	0.01	0.01
Mali	PROMIS CS (46)	907	927	−0.77	0.18 (0.08, 0.29)	0.07	0.08	32	26	−0.78	−0.28 (−0.78, 0.23)	0.02	0.02
Zimbabwe	SHINE (HIV−) (47)	1745	1688	0.01	0.09 (0.02, 0.17)	0.14	0.09	75	62	0.21	−0.26 (−0.60, 0.08)	0.03	0.03
Zimbabwe	SHINE (HIV+) (48)	313	311	0.03	−0.15 (−0.34, 0.04)	0.02	0.04	18	12	−0.31	0.05 (−0.69, 0.78)	0.01	0.01
		**11497**	**14608**		**I² = 0.64, Tau² = 0.01**			**2301**	**2950**		**I² = 0.00, Tau² = 0.00**		
**Fixed**					**0.08 (0.06, 0.11)**						**0.06 (0.00, 0.12)**		
**Random**					**0.06 (0.01, 0.11)**						**0.06 (0.00, 0.12)**		

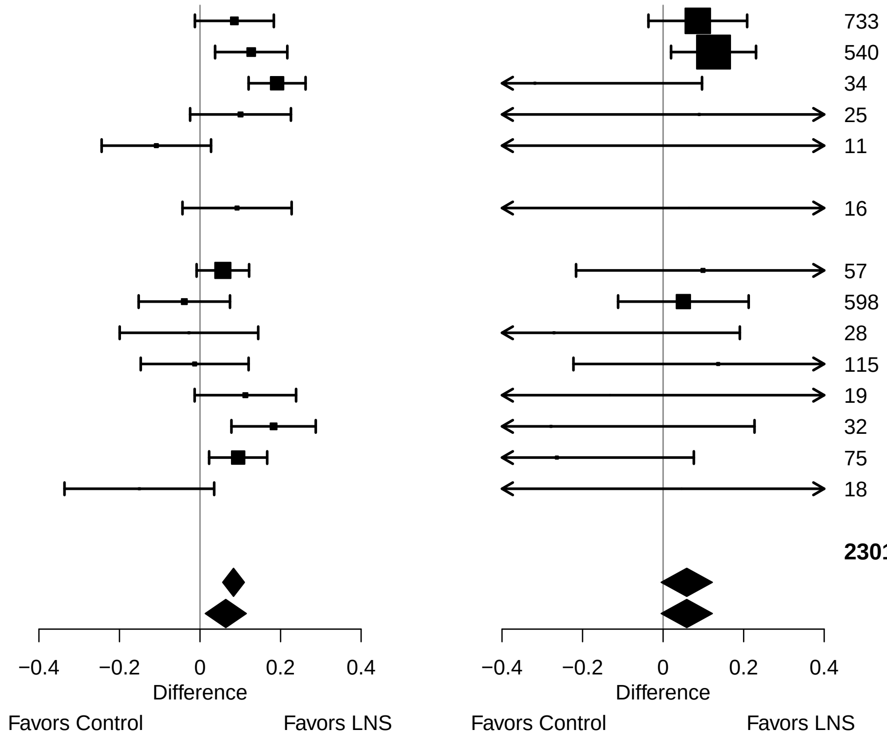

Supplemental figure 8D: Mean difference in WLZ

8D2: Stratified by Maternal BMI

P−for−interaction = 0.849
Difference in MDs = 0.00 (−0.05, 0.04)

		At least 20 kg/m²						Less than 20 kg/m²					
**Country**	**Trial**	**LNS N**	**Control N**	**Control Mean**	**MD (95% CI)**	**Fixed W**	**Random W**	**LNS N**	**Control N**	**Control Mean**	**MD (95% CI)**	**Fixed W**	**Random W**
Bangladesh	JiVitA−4 (34)												
Bangladesh	RDNS (35)	730	335	−0.90	0.04 (−0.07, 0.14)	0.08	0.09	871	455	−1.28	0.10 (0.00, 0.21)	0.14	0.14
Bangladesh	WASH−B (36)	517	1573	−0.67	0.15 (0.05, 0.26)	0.09	0.09	639	1848	−1.05	0.11 (0.02, 0.19)	0.22	0.22
Burkina Faso	iLiNS−Zinc (37)	1214	390	−0.82	0.20 (0.12, 0.29)	0.12	0.10	736	274	−1.11	0.14 (0.06, 0.22)	0.26	0.26
Burkina Faso	PROMIS (38)	644	669	−0.62	0.11 (−0.02, 0.25)	0.05	0.07	209	232	−0.96	0.04 (−0.13, 0.20)	0.05	0.05
Burkina Faso	PROMIS CS (38)	246	267	−0.73	−0.17 (−0.31, −0.03)	0.05	0.07	184	165	−1.13	0.01 (−0.17, 0.19)	0.05	0.05
Ghana	GHANA (39)												
Ghana	iLiNS−DYADG (40)	305	562	−0.54	0.07 (−0.07, 0.21)	0.05	0.07	37	119	−0.79	0.13 (−0.23, 0.48)	0.01	0.01
Haiti	HAITI (41)												
Kenya	WASH−B (42)	1096	3788	0.17	0.03 (−0.03, 0.10)	0.21	0.11	272	1070	−0.19	0.13 (−0.02, 0.27)	0.07	0.07
Madagascar	MAHAY (43)												
Malawi	iLiNS−DYADM (44)	131	263	0.03	−0.10 (−0.31, 0.11)	0.02	0.04	88	178	−0.37	0.01 (−0.22, 0.25)	0.03	0.03
Malawi	iLiNS−DOSE (45)	513	174	−0.09	0.01 (−0.14, 0.16)	0.04	0.07	180	65	−0.59	0.01 (−0.23, 0.25)	0.03	0.03
Mali	PROMIS (46)	376	368	−0.23	0.08 (−0.05, 0.21)	0.05	0.07	117	133	−0.61	0.15 (−0.05, 0.35)	0.04	0.04
Mali	PROMIS CS (46)	696	675	−0.70	0.18 (0.07, 0.30)	0.07	0.08	243	278	−0.96	0.09 (−0.10, 0.28)	0.04	0.04
Zimbabwe	SHINE (HIV−) (47)	1355	1299	0.06	0.09 (0.02, 0.17)	0.15	0.10	239	248	−0.29	0.15 (−0.03, 0.33)	0.05	0.05
Zimbabwe	SHINE (HIV+) (48)	246	253	0.08	−0.06 (−0.26, 0.15)	0.02	0.05	62	52	−0.39	−0.22 (−0.55, 0.12)	0.01	0.01
		**8069**	**10616**		**I² = 0.64, Tau² = 0.01**			**3877**	**5117**		**I² = 0.00, Tau² = 0.00**		
**Fixed**					**0.08 (0.05, 0.11)**						**0.10 (0.06, 0.14)**		
**Random**					**0.06 (0.01, 0.12)**						**0.10 (0.06, 0.14)**		

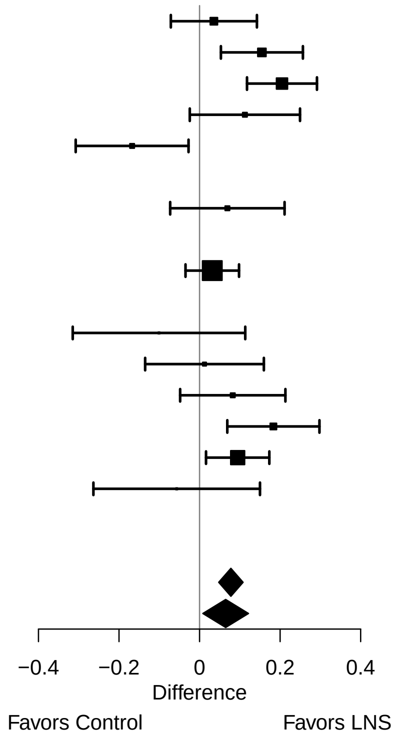

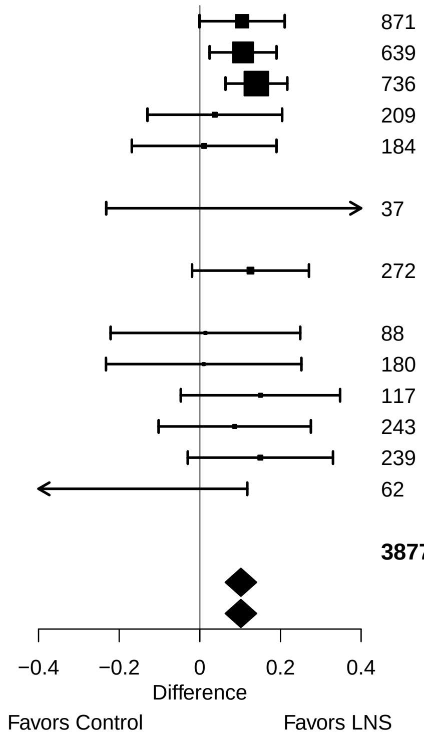

Supplemental figure 8D: Mean difference in WLZ

8D3: Stratified by Maternal age

P−for−interaction = 0.354
Difference in MDs = −0.02 (−0.06, 0.02)

		At least 25 y						Less than 25 y					
Country	Trial	LNS N	Control N	Control Mean	MD (95% CI)	Fixed W	Random W	LNS N	Control N	Control Mean	MD (95% CI)	Fixed W	Random W
Bangladesh	JiVitA−4 (34)	1191	535	−1.18	0.09 (0.02, 0.15)	0.19	0.15	1580	677	−1.16	0.07 (0.01, 0.14)	0.22	0.11
Bangladesh	RDNS (35)	453	221	−1.11	0.01 (−0.13, 0.16)	0.04	0.05	1208	594	−1.11	0.10 (0.01, 0.18)	0.12	0.09
Bangladesh	WASH−B (36)	491	1508	−0.92	0.05 (−0.05, 0.16)	0.07	0.08	660	1900	−0.84	0.17 (0.07, 0.26)	0.11	0.09
Burkina Faso	iLiNS−Zinc (37)	1131	399	−0.94	0.17 (0.10, 0.25)	0.15	0.13	808	262	−0.94	0.19 (0.06, 0.32)	0.05	0.07
Burkina Faso	PROMIS (38)	480	522	−0.74	0.13 (−0.01, 0.28)	0.04	0.05	376	385	−0.68	0.05 (−0.12, 0.23)	0.03	0.05
Burkina Faso	PROMIS CS (38)	202	190	−0.93	−0.09 (−0.28, 0.11)	0.02	0.03	228	246	−0.85	−0.13 (−0.30, 0.04)	0.03	0.05
Ghana	GHANA (39)	69	63	−0.72	0.37 (−0.04, 0.79)	0.00	0.01	29	25	−0.40	−0.24 (−0.86, 0.37)	0.00	0.01
Ghana	iLiNS−DYADG (40)	220	416	−0.54	0.02 (−0.15, 0.19)	0.03	0.04	127	276	−0.66	0.21 (0.01, 0.41)	0.02	0.04
Haiti	HAITI (41)	100	103	−0.03	0.07 (−0.12, 0.25)	0.03	0.03	47	45	0.04	0.12 (−0.16, 0.41)	0.01	0.03
Kenya	WASH−B (42)	842	2801	0.09	0.09 (0.01, 0.17)	0.14	0.12	598	2285	0.09	0.03 (−0.05, 0.11)	0.14	0.10
Madagascar	MAHAY (43)	894	949	−0.48	0.05 (−0.07, 0.17)	0.06	0.07	805	733	−0.34	−0.07 (−0.19, 0.05)	0.06	0.07
Malawi	iLiNS−DYADM (44)	106	226	−0.11	−0.04 (−0.27, 0.19)	0.02	0.02	114	218	−0.17	−0.07 (−0.29, 0.16)	0.02	0.04
Malawi	iLiNS−DOSE (45)	368	134	−0.28	0.01 (−0.15, 0.18)	0.03	0.04	314	100	−0.17	0.02 (−0.18, 0.22)	0.02	0.04
Mali	PROMIS (46)	341	355	−0.29	0.08 (−0.07, 0.23)	0.04	0.04	158	147	−0.43	0.18 (−0.02, 0.37)	0.02	0.04
Mali	PROMIS CS (46)	498	501	−0.78	0.15 (0.02, 0.29)	0.05	0.05	446	458	−0.75	0.17 (0.04, 0.29)	0.06	0.07
Zimbabwe	SHINE (HIV−) (47)	870	796	−0.01	0.12 (0.02, 0.23)	0.08	0.08	802	792	0.05	0.04 (−0.07, 0.15)	0.08	0.08
Zimbabwe	SHINE (HIV+) (48)	243	231	−0.04	−0.11 (−0.33, 0.10)	0.02	0.02	73	77	0.20	−0.25 (−0.63, 0.14)	0.01	0.02
		**8499**	**9950**		**I² = 0.17, Tau² = 0.00**			**8373**	**9220**		**I² = 0.47, Tau² = 0.01**		
**Fixed**					**0.09 (0.06, 0.12)**						**0.07 (0.04, 0.10)**		
**Random**					**0.08 (0.05, 0.12)**						**0.07 (0.02, 0.12)**		

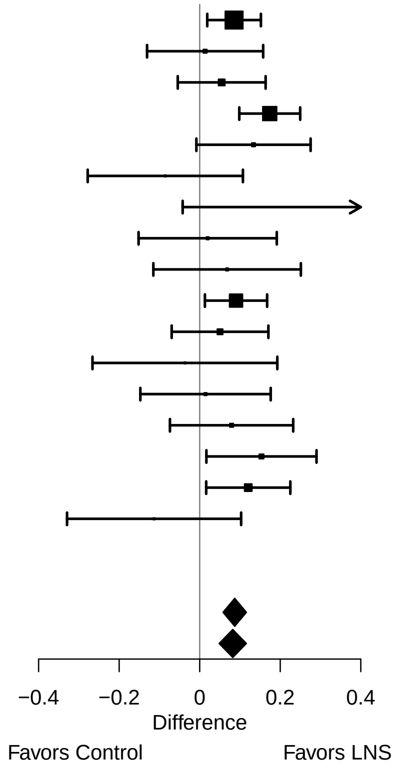

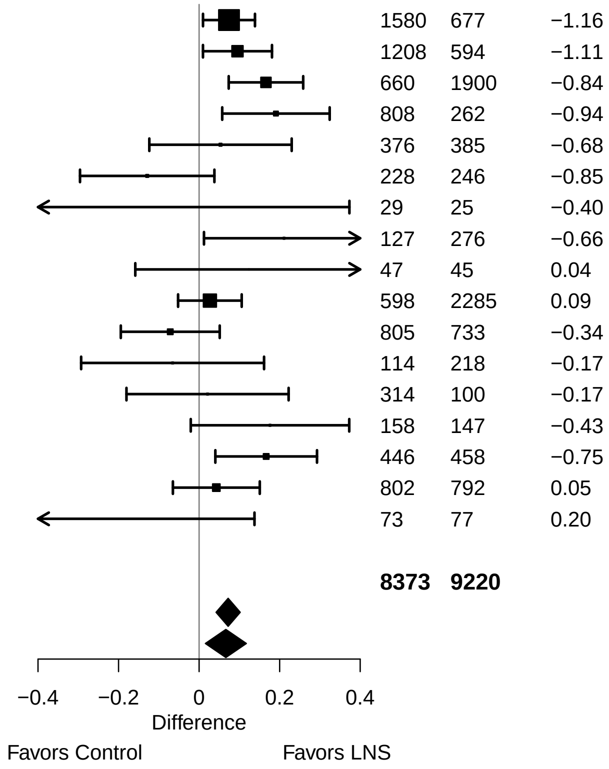

Supplemental figure 8D: Mean difference in WLZ

8D4: Stratified by Maternal education

P−for−interaction = 0.629
Difference in MDs = 0.01 (−0.04, 0.06)

		Primary or greater							Incomplete or no formal					
**Country**	**Trial**	**LNS N**	**Control N**	**Control Mean**	**MD (95% CI)**	**Fixed W**	**Random W**	**LNS N**	**Control N**	**Control Mean**	**MD (95% CI)**	**Fixed W**	**Random W**
Bangladesh	JiVitA−4 (34)	1797	739	−1.12	0.08 (0.02, 0.14)	0.25	0.24	984	475	−1.24	0.08 (0.01, 0.15)	0.16	0.12
Bangladesh	RDNS (35)	1245	589	−1.10	0.10 (0.00, 0.19)	0.11	0.11	416	226	−1.13	0.00 (−0.16, 0.17)	0.03	0.05
Bangladesh	WASH−B (36)	813	2447	−0.80	0.13 (0.05, 0.21)	0.15	0.15	343	977	−1.06	0.12 (0.00, 0.23)	0.06	0.08
Burkina Faso	iLiNS−Zinc (37)	82	16	−0.64	−0.01 (−0.41, 0.39)	0.01	0.01	1857	645	−0.95	0.19 (0.12, 0.26)	0.20	0.12
Burkina Faso	PROMIS (38)	70	54	−0.77	0.08 (−0.19, 0.34)	0.01	0.01	786	853	−0.71	0.10 (−0.03, 0.23)	0.05	0.07
Burkina Faso	PROMIS CS (38)	34	39	−0.82	−0.16 (−0.64, 0.32)	0.00	0.00	393	396	−0.89	−0.10 (−0.23, 0.02)	0.06	0.07
Ghana	GHANA (39)	91	75	−0.68	0.25 (−0.12, 0.62)	0.01	0.01	7	15	−0.32	−0.06 (−1.07, 0.95)	0.00	0.00
Ghana	iLiNS−DYADG (40)	267	548	−0.56	0.06 (−0.09, 0.21)	0.04	0.04	80	144	−0.69	0.20 (−0.06, 0.46)	0.01	0.02
Haiti	HAITI (41)	129	126	0.09	0.12 (−0.04, 0.28)	0.04	0.04	19	23	−0.44	−0.19 (−0.71, 0.32)	0.00	0.01
Kenya	WASH−B (42)	700	2426	0.13	0.04 (−0.05, 0.12)	0.12	0.12	753	2688	0.06	0.09 (0.01, 0.16)	0.15	0.11
Madagascar	MAHAY (43)	351	431	−0.20	−0.01 (−0.17, 0.14)	0.04	0.04	1349	1251	−0.50	0.02 (−0.09, 0.14)	0.07	0.08
Malawi	iLiNS−DYADM (44)	35	69	−0.02	−0.20 (−0.62, 0.22)	0.01	0.01	184	372	−0.16	−0.03 (−0.21, 0.14)	0.03	0.04
Malawi	iLiNS−DOSE (45)	162	56	0.01	0.02 (−0.25, 0.28)	0.01	0.01	522	178	−0.31	0.02 (−0.13, 0.16)	0.04	0.06
Mali	PROMIS (46)	39	37	−0.38	−0.08 (−0.38, 0.21)	0.01	0.01	459	465	−0.33	0.12 (−0.02, 0.25)	0.05	0.07
Mali	PROMIS CS (46)	107	96	−0.69	0.31 (−0.02, 0.64)	0.01	0.01	836	863	−0.78	0.14 (0.03, 0.24)	0.08	0.09
Zimbabwe	SHINE (HIV−) (47)	1707	1634	0.03	0.06 (−0.02, 0.13)	0.16	0.16	68	61	0.01	0.15 (−0.23, 0.54)	0.01	0.01
Zimbabwe	SHINE (HIV+) (48)	300	292	0.03	−0.14 (−0.33, 0.05)	0.02	0.03	21	18	0.00	−0.34 (−0.93, 0.25)	0.00	0.01
		**7929**	**9674**		**I² = 0.03, Tau² = 0.00**			**9077**	**9650**		**I² = 0.44, Tau² = 0.00**		
**Fixed**					**0.07 (0.04, 0.10)**						**0.09 (0.06, 0.12)**		
**Random**					**0.07 (0.04, 0.10)**						**0.08 (0.03, 0.12)**		

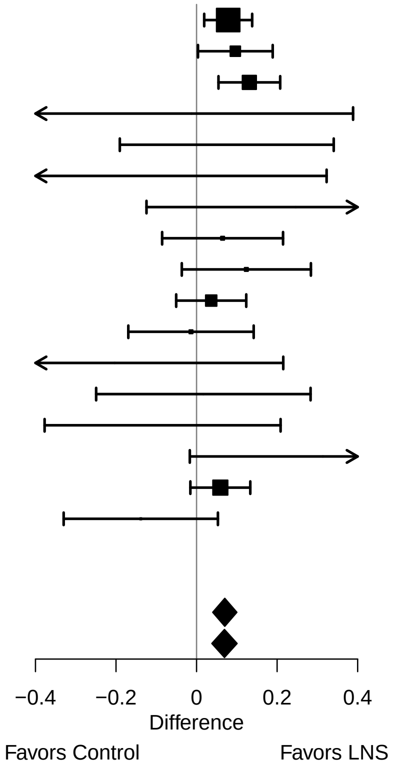

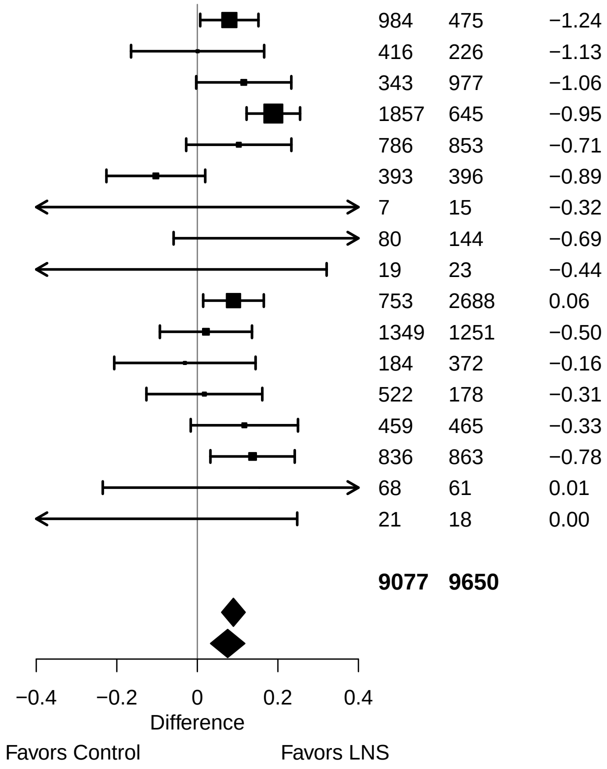

Supplemental figure 8D: Mean difference in WLZ

8D5: Stratified by Maternal depressive symptoms

P−for−interaction = 0.863
Difference in MDs = −0.01 (−0.07, 0.05)

		Less than 75th percentile						At least 75th percentile					
**Country**	**Trial**	**LNS N**	**Control N**	**Control Mean**	**MD (95% CI)**	**Fixed W**	**Random W**	**LNS N**	**Control N**	**Control Mean**	**MD (95% CI)**	**Fixed W**	**Random W**
Bangladesh	JiVitA−4 (34)												
Bangladesh	RDNS (35)	1081	472	−1.08	0.04 (−0.06, 0.14)	0.12	0.12	519	288	−1.14	0.09 (−0.04, 0.23)	0.14	0.14
Bangladesh	WASH−B (36)	895	2457	−0.86	0.13 (0.05, 0.21)	0.19	0.17	237	876	−0.90	0.10 (−0.04, 0.23)	0.14	0.14
Burkina Faso	iLiNS−Zinc (37)												
Burkina Faso	PROMIS (38)	505	569	−0.75	0.15 (0.00, 0.30)	0.05	0.06	219	212	−0.74	0.06 (−0.09, 0.21)	0.12	0.12
Burkina Faso	PROMIS CS (38)												
Ghana	GHANA (39)												
Ghana	iLiNS−DYADG (40)	252	459	−0.55	0.08 (−0.07, 0.24)	0.05	0.06	83	209	−0.67	0.15 (−0.12, 0.41)	0.04	0.04
Haiti	HAITI (41)												
Kenya	WASH−B (42)	994	3480	0.10	0.07 (−0.01, 0.14)	0.23	0.19	339	1233	0.09	0.04 (−0.07, 0.15)	0.21	0.21
Madagascar	MAHAY (43)	672	721	−0.45	0.08 (−0.06, 0.21)	0.07	0.08	283	252	−0.38	−0.08 (−0.26, 0.10)	0.08	0.08
Malawi	iLiNS−DYADM (44)	157	303	−0.09	−0.08 (−0.27, 0.11)	0.03	0.04	47	113	−0.26	0.12 (−0.22, 0.47)	0.02	0.02
Malawi	iLiNS−DOSE (45)												
Mali	PROMIS (46)	374	372	−0.31	0.06 (−0.06, 0.18)	0.08	0.09	121	129	−0.42	0.24 (0.02, 0.45)	0.06	0.06
Mali	PROMIS CS (46)												
Zimbabwe	SHINE (HIV−) (47)	1192	1136	0.02	0.09 (0.00, 0.17)	0.16	0.16	509	489	−0.02	0.13 (0.00, 0.26)	0.16	0.16
Zimbabwe	SHINE (HIV+) (48)	234	214	0.04	−0.18 (−0.41, 0.05)	0.02	0.03	88	100	−0.07	−0.08 (−0.38, 0.23)	0.03	0.03
		**6356**	**10183**		**I² = 0.13, Tau² = 0.00**			**2445**	**3901**		**I² = 0.00, Tau² = 0.00**		
**Fixed**					**0.07 (0.04, 0.11)**						**0.08 (0.02, 0.13)**		
**Random**					**0.07 (0.03, 0.11)**						**0.08 (0.02, 0.13)**		

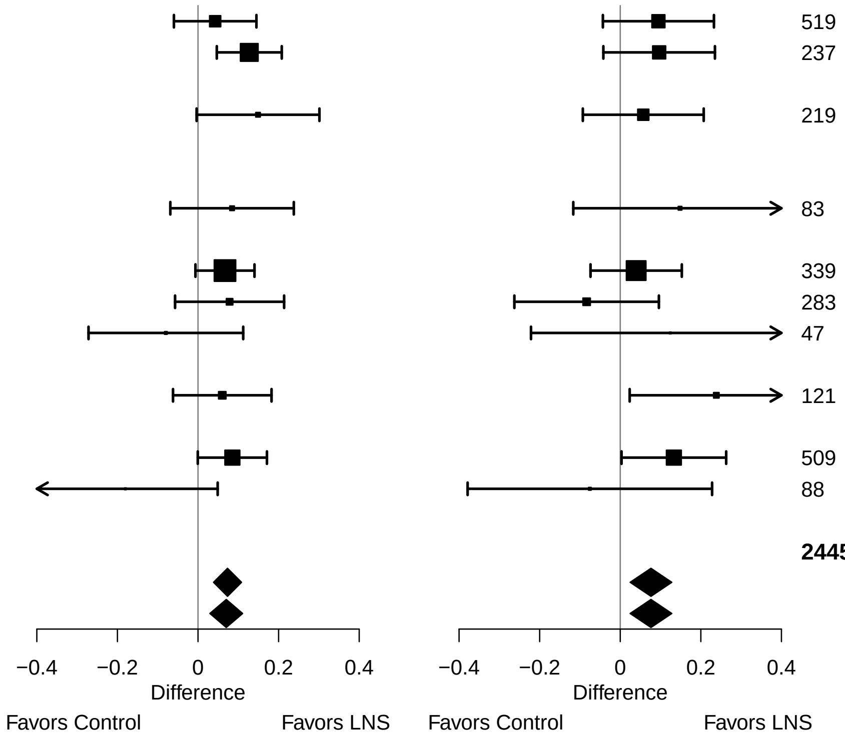

Supplemental figure 8D: Mean difference in WLZ

8D6: Stratified by Child sex

P−for−interaction = 0.647
Difference in MDs = 0.01 (−0.03, 0.05)

Country	Trial	Male LNS N	Male Control N	Male Control Mean	Male MD (95% CI)	Male Fixed W	Male Random W	Female LNS N	Female Control N	Female Control Mean	Female MD (95% CI)	Female Fixed W	Female Random W
Bangladesh	JiVitA−4 (34)	1420	586	−1.16	0.07 (0.00, 0.14)	0.21	0.12	1363	631	−1.18	0.10 (0.04, 0.16)	0.20	0.13
Bangladesh	RDNS (35)	832	411	−1.08	0.00 (−0.12, 0.12)	0.07	0.07	829	404	−1.14	0.14 (0.05, 0.24)	0.08	0.08
Bangladesh	WASH−B (36)	564	1711	−0.88	0.13 (0.03, 0.23)	0.10	0.09	592	1713	−0.86	0.11 (0.02, 0.20)	0.10	0.09
Burkina Faso	iLiNS−Zinc (37)	990	336	−1.04	0.21 (0.10, 0.32)	0.08	0.08	962	328	−0.85	0.15 (0.07, 0.24)	0.10	0.09
Burkina Faso	PROMIS (38)	434	480	−0.82	0.17 (0.01, 0.32)	0.04	0.06	422	427	−0.59	0.02 (−0.14, 0.18)	0.03	0.04
Burkina Faso	PROMIS CS (38)	234	221	−0.89	−0.17 (−0.36, 0.02)	0.03	0.04	196	215	−0.87	−0.04 (−0.19, 0.12)	0.03	0.04
Ghana	GHANA (39)	60	41	−0.69	0.13 (−0.31, 0.56)	0.01	0.01	38	55	−0.61	0.37 (−0.17, 0.90)	0.00	0.00
Ghana	iLiNS−DYADG (40)	175	323	−0.69	0.09 (−0.10, 0.28)	0.03	0.04	172	369	−0.50	0.11 (−0.07, 0.28)	0.03	0.04
Haiti	HAITI (41)	70	59	0.11	−0.06 (−0.29, 0.18)	0.02	0.03	78	90	−0.06	0.19 (−0.01, 0.40)	0.02	0.03
Kenya	WASH−B (42)	732	2431	0.07	0.05 (−0.03, 0.14)	0.14	0.10	723	2687	0.11	0.08 (0.00, 0.15)	0.14	0.11
Madagascar	MAHAY (43)	824	832	−0.53	0.01 (−0.13, 0.14)	0.05	0.07	876	850	−0.31	−0.01 (−0.13, 0.10)	0.06	0.07
Malawi	iLiNS−DYADM (44)	107	207	−0.15	−0.09 (−0.33, 0.15)	0.02	0.03	113	237	−0.12	−0.02 (−0.24, 0.20)	0.02	0.03
Malawi	iLiNS−DOSE (45)	352	124	−0.37	−0.01 (−0.19, 0.17)	0.03	0.04	344	117	−0.08	0.02 (−0.15, 0.20)	0.03	0.04
Mali	PROMIS (46)	264	262	−0.36	0.13 (−0.02, 0.28)	0.04	0.06	235	240	−0.30	0.08 (−0.07, 0.23)	0.03	0.05
Mali	PROMIS CS (46)	472	520	−0.81	0.14 (−0.02, 0.30)	0.04	0.05	472	439	−0.71	0.17 (0.04, 0.30)	0.04	0.06
Zimbabwe	SHINE (HIV−) (47)	924	905	−0.04	0.09 (−0.01, 0.19)	0.09	0.09	945	879	0.08	0.06 (−0.04, 0.16)	0.08	0.08
Zimbabwe	SHINE (HIV+) (48)	161	167	−0.12	−0.08 (−0.32, 0.16)	0.02	0.03	175	161	0.14	−0.22 (−0.46, 0.03)	0.01	0.02
		**8615**	**9616**		**I² = 0.35, Tau² = 0.00**			**8535**	**9842**		**I² = 0.25, Tau² = 0.00**		
**Fixed**					**0.07 (0.04, 0.10)**						**0.09 (0.06, 0.12)**		
**Random**					**0.07 (0.02, 0.11)**						**0.08 (0.04, 0.12)**		

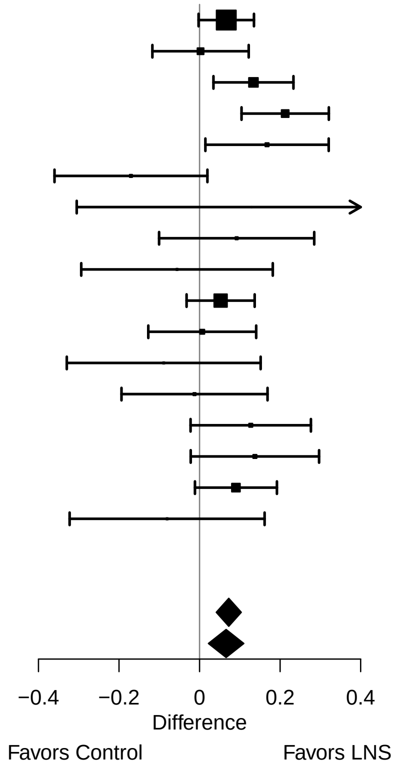

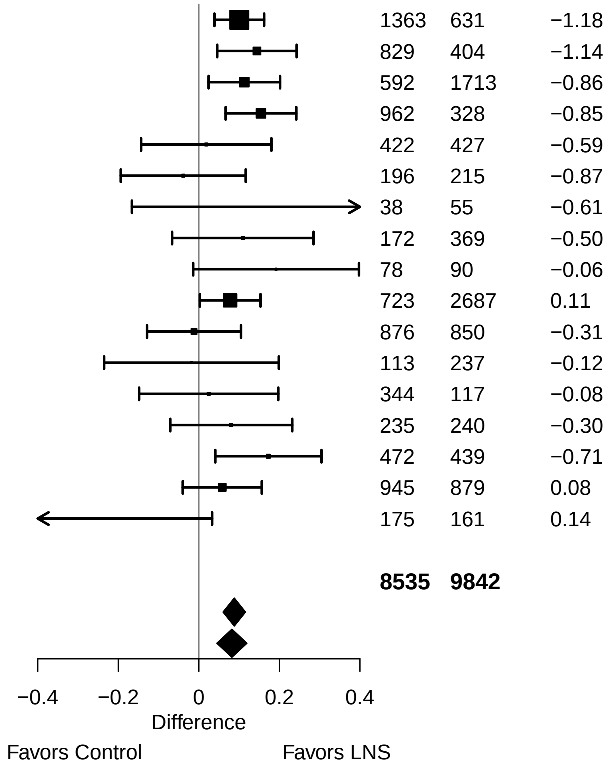

Supplemental figure 8D: Mean difference in WLZ

8D7: Stratified by Child birth order

P−for−interaction = 0.250
Difference in MDs = −0.03 (−0.07, 0.02)

Country	Trial	Later born LNS N	Later born Control N	Later born Control Mean	Later born MD (95% CI)	Later born Fixed W	Later born Random W	Firstborn LNS N	Firstborn Control N	Firstborn Control Mean	Firstborn MD (95% CI)	Firstborn Fixed W	Firstborn Random W
Bangladesh	JiVitA−4 (34)	598	247	−1.24	0.11 (0.02, 0.21)	0.08	0.07	2181	964	−1.15	0.07 (0.02, 0.13)	0.41	0.20
Bangladesh	RDNS (35)	972	508	−1.13	0.08 (−0.01, 0.17)	0.09	0.08	688	307	−1.08	0.06 (−0.04, 0.17)	0.09	0.11
Bangladesh	WASH−B (36)	735	2285	−0.92	0.11 (0.03, 0.20)	0.11	0.08	421	1139	−0.78	0.13 (0.01, 0.24)	0.08	0.10
Burkina Faso	iLiNS−Zinc (37)	1520	519	−0.94	0.19 (0.12, 0.26)	0.14	0.08	432	145	−0.95	0.16 (0.01, 0.32)	0.05	0.07
Burkina Faso	PROMIS (38)	708	747	−0.73	0.12 (0.00, 0.25)	0.05	0.06	148	158	−0.65	−0.02 (−0.24, 0.21)	0.02	0.04
Burkina Faso	PROMIS CS (38)	366	357	−0.87	−0.13 (−0.28, 0.02)	0.03	0.05	63	79	−0.92	−0.03 (−0.27, 0.21)	0.02	0.03
Ghana	GHANA (39)	60	54	−0.64	0.27 (−0.21, 0.75)	0.00	0.01	38	38	−0.59	0.06 (−0.41, 0.53)	0.00	0.01
Ghana	iLiNS−DYADG (40)	237	458	−0.56	0.02 (−0.14, 0.18)	0.03	0.05	110	234	−0.65	0.24 (0.02, 0.47)	0.02	0.04
Haiti	HAITI (41)	88	99	−0.05	0.16 (−0.04, 0.36)	0.02	0.04	60	50	0.13	−0.03 (−0.28, 0.22)	0.02	0.03
Kenya	WASH−B (42)	1132	4027	0.08	0.07 (−0.01, 0.14)	0.12	0.08	321	1086	0.12	0.06 (−0.04, 0.15)	0.12	0.12
Madagascar	MAHAY (43)	1270	1217	−0.46	0.02 (−0.10, 0.14)	0.05	0.06	430	465	−0.32	−0.05 (−0.21, 0.11)	0.04	0.06
Malawi	iLiNS−DYADM (44)	173	353	−0.12	−0.05 (−0.23, 0.12)	0.02	0.04	47	89	−0.20	−0.06 (−0.45, 0.33)	0.01	0.01
Malawi	iLiNS−DOSE (45)	458	165	−0.29	0.06 (−0.09, 0.21)	0.03	0.05	149	45	−0.11	−0.06 (−0.37, 0.25)	0.01	0.02
Mali	PROMIS (46)	436	441	−0.32	0.10 (−0.03, 0.24)	0.04	0.06	62	59	−0.43	0.07 (−0.17, 0.31)	0.02	0.03
Mali	PROMIS CS (46)	783	821	−0.76	0.14 (0.03, 0.24)	0.07	0.07	144	126	−0.81	0.32 (0.12, 0.52)	0.03	0.04
Zimbabwe	SHINE (HIV−) (47)	1295	1218	−0.02	0.15 (0.07, 0.23)	0.12	0.08	487	483	0.10	−0.09 (−0.23, 0.06)	0.05	0.08
Zimbabwe	SHINE (HIV+) (48)	279	245	0.07	−0.22 (−0.43, −0.02)	0.02	0.03	54	78	−0.17	0.16 (−0.19, 0.51)	0.01	0.02
		**11110**	**13761**		**I^2 = 0.54, Tau^2 = 0.01**			**5835**	**5545**		**I^2 = 0.21, Tau^2 = 0.00**		
**Fixed**					**0.10 (0.07, 0.12)**						**0.07 (0.03, 0.10)**		
**Random**					**0.08 (0.03, 0.12)**						**0.07 (0.02, 0.11)**		

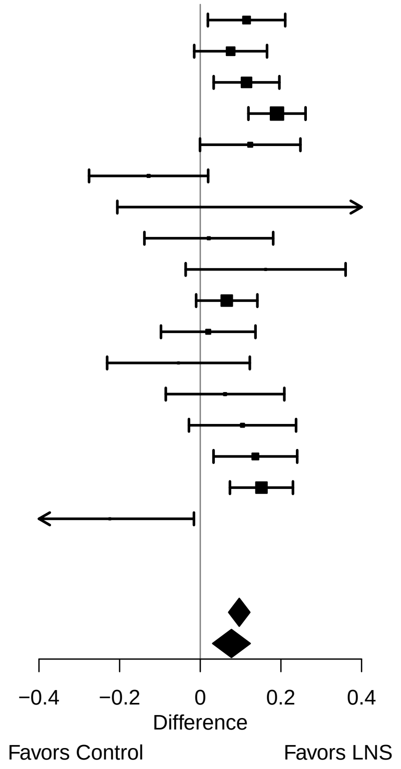

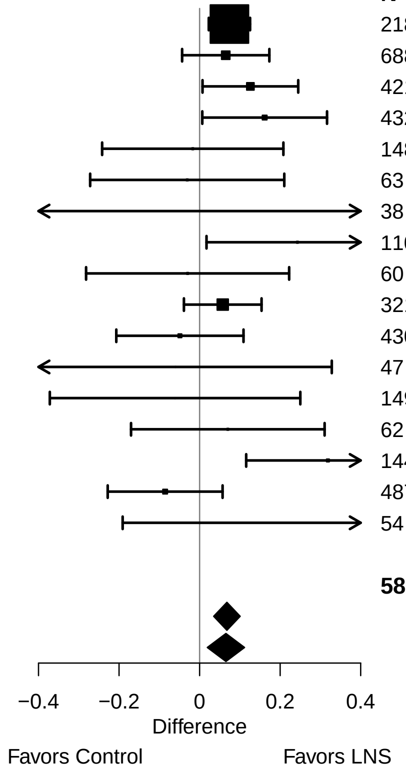

Supplemental figure 8D: Mean difference in WLZ

8D8: Stratified by Child baseline anthropometric status

P−for−interaction = 0.325
Difference in MDs = 0.03 (−0.03, 0.09)

Country	Trial	WLZ at least 0 Z: LNS N	Control N	Control Mean	MD (95% CI)	Fixed W	Random W	WLZ less than 0 Z: LNS N	Control N	Control Mean	MD (95% CI)	Fixed W	Random W
Bangladesh	JiVitA−4 (34)	961	423	−0.51	0.10 (0.01, 0.19)	0.26	0.26	1822	794	−1.51	0.08 (0.02, 0.15)	0.31	0.14
Bangladesh	RDNS (35)	592	270	−0.45	−0.01 (−0.13, 0.11)	0.14	0.14	1008	489	−1.46	0.08 (−0.01, 0.18)	0.15	0.13
Bangladesh	WASH−B (36)												
Burkina Faso	iLiNS−Zinc (37)	349	103	0.17	0.07 (−0.17, 0.30)	0.04	0.04	1603	561	−1.15	0.22 (0.14, 0.30)	0.20	0.13
Burkina Faso	PROMIS (38)	279	350	−0.22	0.12 (−0.05, 0.30)	0.07	0.07	413	393	−1.15	0.15 (0.00, 0.30)	0.06	0.09
Burkina Faso	PROMIS CS (38)												
Ghana	GHANA (39)												
Ghana	iLiNS−DYADG (40)	154	310	0.02	0.11 (−0.06, 0.27)	0.07	0.07	169	328	−1.14	0.10 (−0.05, 0.25)	0.05	0.09
Haiti	HAITI (41)	76	76	0.66	−0.10 (−0.37, 0.16)	0.03	0.03	72	73	−0.67	0.24 (0.00, 0.48)	0.02	0.05
Kenya	WASH−B (42)												
Madagascar	MAHAY (43)												
Malawi	iLiNS−DYADM (44)	131	274	0.21	0.01 (−0.18, 0.20)	0.06	0.06	73	148	−0.80	−0.12 (−0.35, 0.11)	0.02	0.06
Malawi	iLiNS−DOSE (45)	398	141	0.23	−0.07 (−0.26, 0.12)	0.06	0.06	298	100	−0.88	−0.02 (−0.24, 0.20)	0.03	0.06
Mali	PROMIS (46)	166	152	0.21	0.03 (−0.15, 0.20)	0.06	0.06	333	350	−0.56	0.13 (−0.02, 0.29)	0.05	0.09
Mali	PROMIS CS (46)												
Zimbabwe	SHINE (HIV−) (47)	743	702	0.35	0.09 (−0.02, 0.19)	0.19	0.19	555	479	−0.49	0.12 (0.00, 0.24)	0.08	0.11
Zimbabwe	SHINE (HIV+) (48)	129	135	0.26	0.10 (−0.17, 0.38)	0.03	0.03	132	99	−0.34	−0.21 (−0.46, 0.04)	0.02	0.05
		**3978**	**2936**		**I^2 = 0.00, Tau^2 = 0.00**			**6478**	**3814**		**I^2 = 0.54, Tau^2 = 0.01**		
**Fixed**					**0.06 (0.01, 0.10)**						**0.11 (0.08, 0.15)**		
**Random**					**0.06 (0.01, 0.10)**						**0.09 (0.03, 0.16)**		

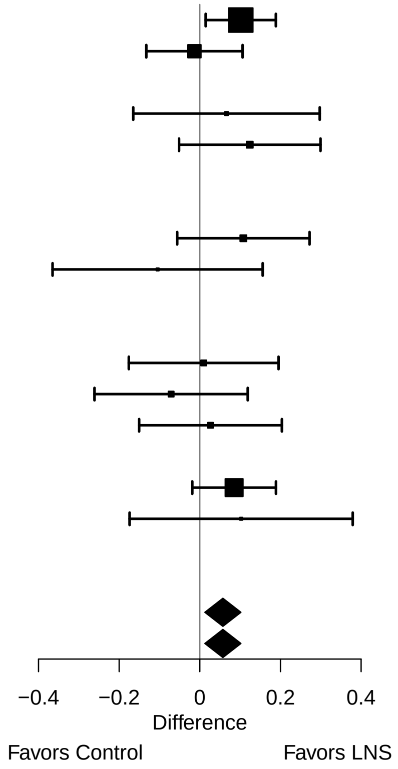

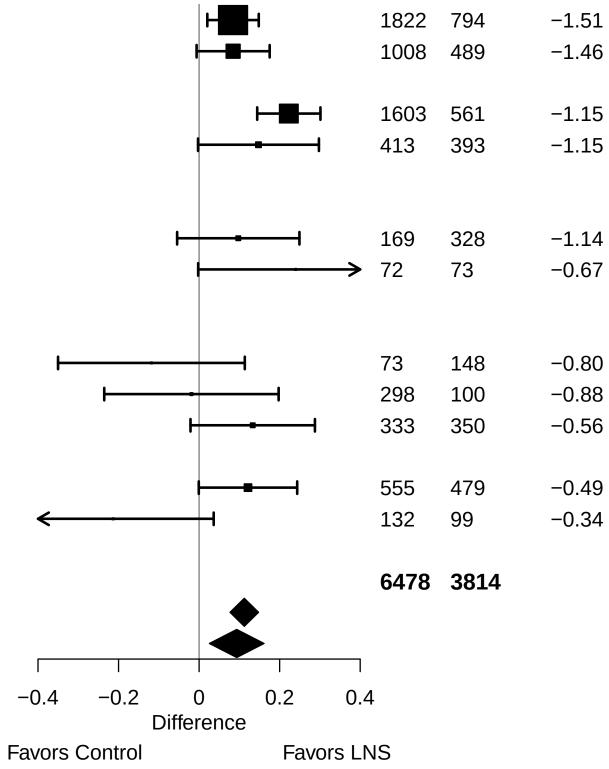

Supplemental figure 8E: Wasting prevalence ratio

8E1: Stratified by Maternal height

P−for−interaction = 0.883
Ratio of PRs = 1.02 (0.81, 1.28)

		At least 150.1 cm						Less than 150.1 cm					
**Country**	**Trial**	**LNS N**	**Control N**	**Control Prevalence**	**PR (95% CI)**	**Fixed W**	**Random W**	**LNS N**	**Control N**	**Control Prevalence**	**PR (95% CI)**	**Fixed W**	**Random W**
Bangladesh	JiVitA−4 (34)												
Bangladesh	RDNS (35)	868	432	13.9	0.87 (0.67, 1.14)	0.32	0.25	733	358	16.8	0.83 (0.60, 1.15)	0.27	0.27
Bangladesh	WASH−B (36)	616	1880	10.2	0.65 (0.49, 0.87)	0.26	0.24	540	1541	12.5	0.91 (0.71, 1.15)	0.50	0.50
Burkina Faso	iLiNS−Zinc (37)												
Burkina Faso	PROMIS (38)												
Burkina Faso	PROMIS CS (38)												
Ghana	GHANA (39)												
Ghana	iLiNS−DYADG (40)												
Haiti	HAITI (41)												
Kenya	WASH−B (42)												
Madagascar	MAHAY (43)	1002	1037	4.6	1.19 (0.74, 1.89)	0.11	0.17	598	565	8.1	0.72 (0.46, 1.11)	0.15	0.15
Malawi	iLiNS−DYADM (44)												
Malawi	iLiNS−DOSE (45)	580	198	4.0	1.65 (0.81, 3.36)	0.05	0.10	115	42	11.9	0.78 (0.30, 2.04)	0.03	0.03
Mali	PROMIS (46)												
Mali	PROMIS CS (46)	907	927	10.0	0.80 (0.60, 1.08)	0.27	0.24	32	26	23.1	0.95 (0.44, 2.03)	0.05	0.05
Zimbabwe	SHINE (HIV−) (47)												
Zimbabwe	SHINE (HIV+) (48)												
		**3973**	**4474**		**I² = 0.54, Tau² = 0.06**			**2018**	**2532**		**I² = 0.00, Tau² = 0.00**		
**Fixed**					**0.84 (0.72, 0.98)**						**0.85 (0.72, 1.01)**		
**Random**					**0.90 (0.68, 1.18)**						**0.85 (0.72, 1.01)**		

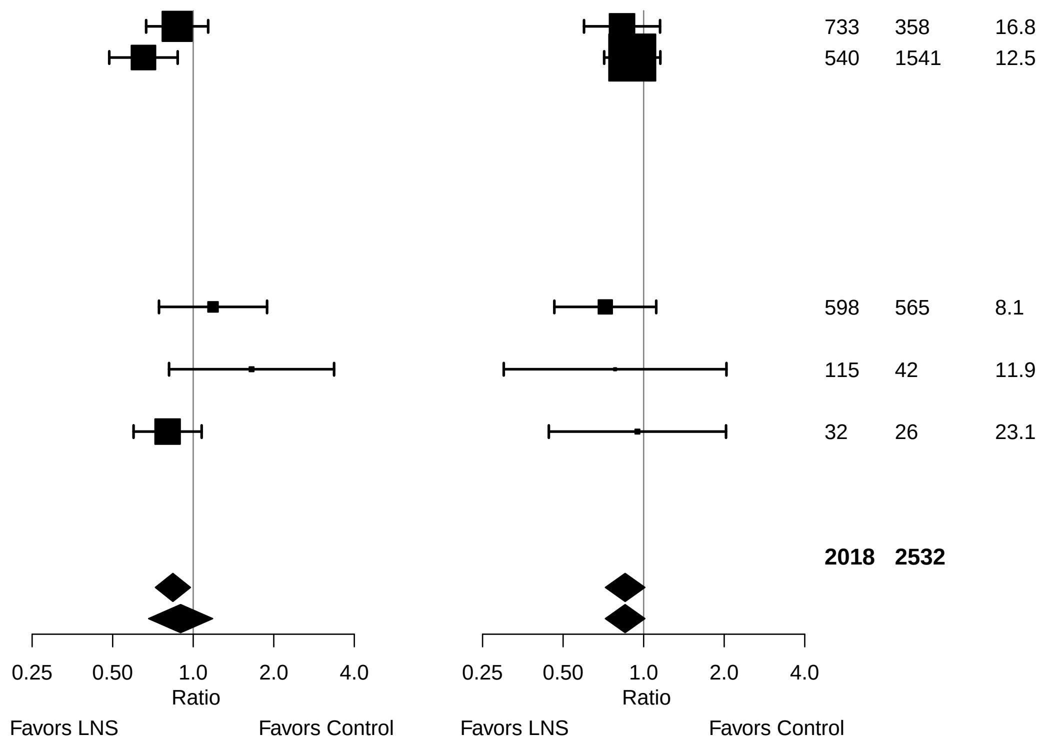

Supplemental figure 8E: Wasting prevalence ratio

8E2: Stratified by Maternal BMI

P−for−interaction = 0.848
Ratio of PRs = 1.02 (0.84, 1.23)

		At least 20 kg/m²						Less than 20 kg/m²					
**Country**	**Trial**	**LNS N**	**Control N**	**Control Prevalence**	**PR (95% CI)**	**Fixed W**	**Random W**	**LNS N**	**Control N**	**Control Prevalence**	**PR (95% CI)**	**Fixed W**	**Random W**
Bangladesh	JiVitA−4 (34)												
Bangladesh	RDNS (35)	730	335	9.6	0.99 (0.68, 1.44)	0.16	0.16	871	455	19.3	0.82 (0.65, 1.04)	0.25	0.25
Bangladesh	WASH−B (36)	517	1573	7.1	0.79 (0.55, 1.14)	0.17	0.17	639	1848	14.8	0.77 (0.62, 0.96)	0.30	0.30
Burkina Faso	iLiNS−Zinc (37)	1214	390	10.5	0.60 (0.41, 0.88)	0.16	0.16	736	274	17.9	0.78 (0.60, 1.00)	0.22	0.22
Burkina Faso	PROMIS (38)	644	669	7.3	0.89 (0.56, 1.43)	0.10	0.10	209	232	13.8	0.80 (0.47, 1.35)	0.05	0.05
Burkina Faso	PROMIS CS (38)	246	267	8.2	1.18 (0.70, 2.00)	0.08	0.08	184	165	19.4	0.87 (0.57, 1.33)	0.08	0.08
Ghana	GHANA (39)												
Ghana	iLiNS−DYADG (40)												
Haiti	HAITI (41)												
Kenya	WASH−B (42)												
Madagascar	MAHAY (43)												
Malawi	iLiNS−DYADM (44)												
Malawi	iLiNS−DOSE (45)	513	174	4.6	1.07 (0.51, 2.25)	0.04	0.04	180	65	7.7	1.49 (0.60, 3.66)	0.02	0.02
Mali	PROMIS (46)												
Mali	PROMIS CS (46)	696	675	9.8	0.71 (0.50, 0.99)	0.20	0.20	243	278	11.9	1.11 (0.71, 1.73)	0.07	0.07
Zimbabwe	SHINE (HIV−) (47)	1355	1299	2.4	0.90 (0.54, 1.49)	0.09	0.09	239	248	5.2	0.64 (0.28, 1.44)	0.02	0.02
Zimbabwe	SHINE (HIV+) (48)												
		**5915**	**5382**		**I² = 0.00, Tau² = 0.00**			**3301**	**3565**		**I² = 0.00, Tau² = 0.00**		
**Fixed**					**0.82 (0.71, 0.96)**						**0.82 (0.73, 0.92)**		
**Random**					**0.82 (0.71, 0.96)**						**0.82 (0.73, 0.92)**		

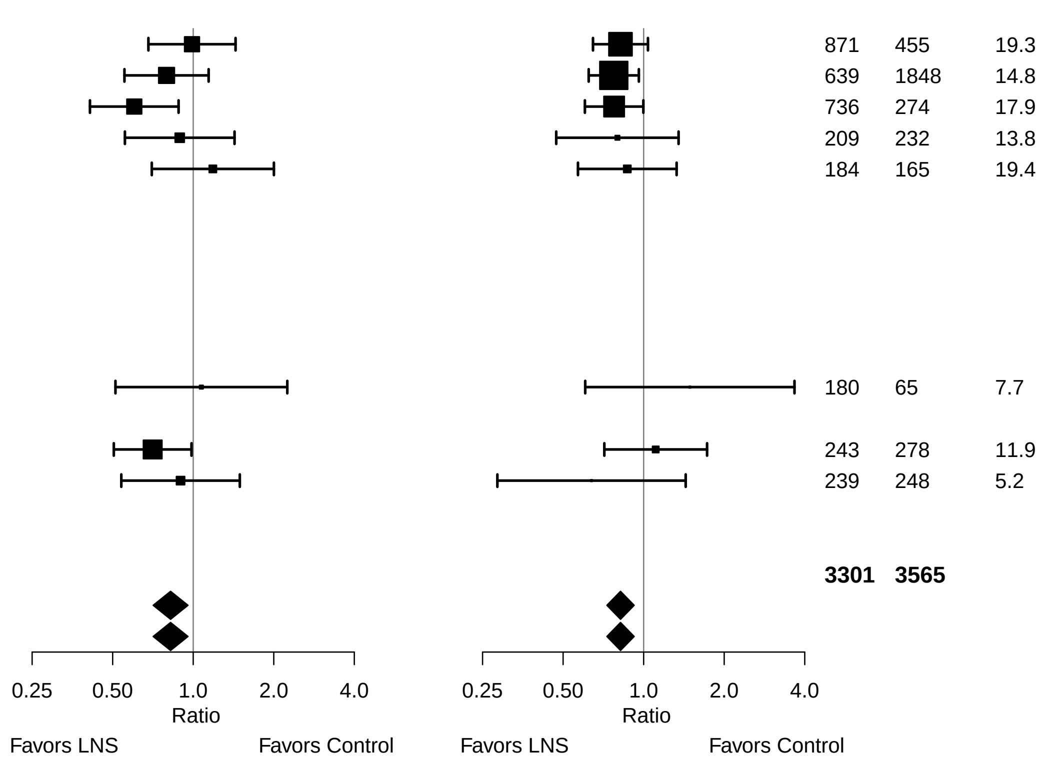

Supplemental figure 8E: Wasting prevalence ratio

8E3: Stratified by Maternal age

P−for−interaction = 0.707
Ratio of PRs = 1.03 (0.88, 1.22)

Country	Trial	At least 25 y: LNS N	At least 25 y: Control N	At least 25 y: Control Prevalence	At least 25 y: PR (95% CI)	At least 25 y: Fixed W	At least 25 y: Random W	Less than 25 y: LNS N	Less than 25 y: Control N	Less than 25 y: Control Prevalence	Less than 25 y: PR (95% CI)	Less than 25 y: Fixed W	Less than 25 y: Random W
Bangladesh	JiVitA−4 (34)	1191	535	17.3	0.88 (0.70, 1.11)	0.26	0.25	1580	677	15.5	0.94 (0.76, 1.15)	0.30	0.30
Bangladesh	RDNS (35)	453	221	14.0	1.16 (0.82, 1.66)	0.11	0.11	1208	594	15.5	0.76 (0.58, 1.01)	0.16	0.16
Bangladesh	WASH−B (36)	491	1508	11.6	0.95 (0.69, 1.29)	0.14	0.14	660	1900	10.9	0.65 (0.48, 0.88)	0.14	0.14
Burkina Faso	iLiNS−Zinc (37)	1131	399	14.0	0.62 (0.46, 0.84)	0.14	0.14	808	262	12.6	0.80 (0.56, 1.14)	0.10	0.10
Burkina Faso	PROMIS (38)	480	522	8.6	0.92 (0.63, 1.34)	0.09	0.09	376	385	9.4	0.80 (0.42, 1.51)	0.03	0.03
Burkina Faso	PROMIS CS (38)	202	190	13.2	0.94 (0.52, 1.69)	0.04	0.04	228	246	11.8	1.12 (0.75, 1.67)	0.08	0.08
Ghana	GHANA (39)												
Ghana	iLiNS−DYADG (40)												
Haiti	HAITI (41)												
Kenya	WASH−B (42)	842	2801	1.7	0.62 (0.31, 1.24)	0.03	0.03	598	2285	1.2	1.23 (0.57, 2.63)	0.02	0.02
Madagascar	MAHAY (43)	894	949	6.8	0.85 (0.58, 1.23)	0.10	0.10	805	733	4.5	1.19 (0.71, 1.98)	0.05	0.05
Malawi	iLiNS−DYADM (44)												
Malawi	iLiNS−DOSE (45)												
Mali	PROMIS (46)												
Mali	PROMIS CS (46)	498	501	11.0	0.79 (0.51, 1.22)	0.07	0.07	446	458	9.6	0.89 (0.60, 1.31)	0.08	0.08
Zimbabwe	SHINE (HIV−) (47)	870	796	3.1	0.66 (0.33, 1.31)	0.03	0.03	802	792	2.1	0.99 (0.53, 1.86)	0.03	0.03
Zimbabwe	SHINE (HIV+) (48)												
		**7052**	**8422**		**I² = 0.05, Tau² = 0.00**			**7511**	**8332**		**I² = 0.00, Tau² = 0.00**		
**Fixed**					**0.85 (0.76, 0.96)**						**0.87 (0.78, 0.97)**		
**Random**					**0.85 (0.76, 0.96)**						**0.87 (0.78, 0.97)**		

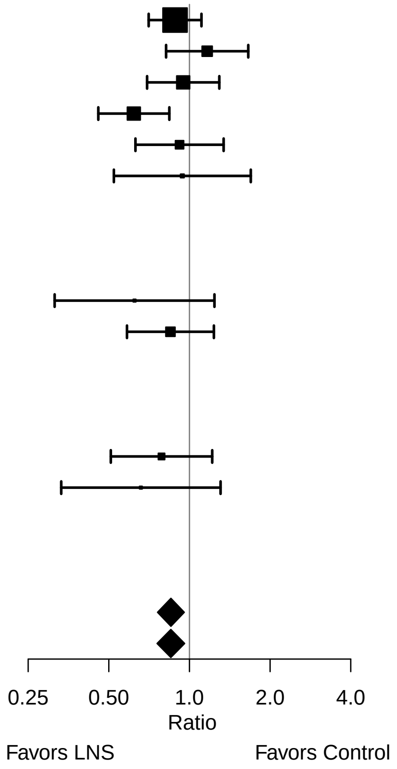

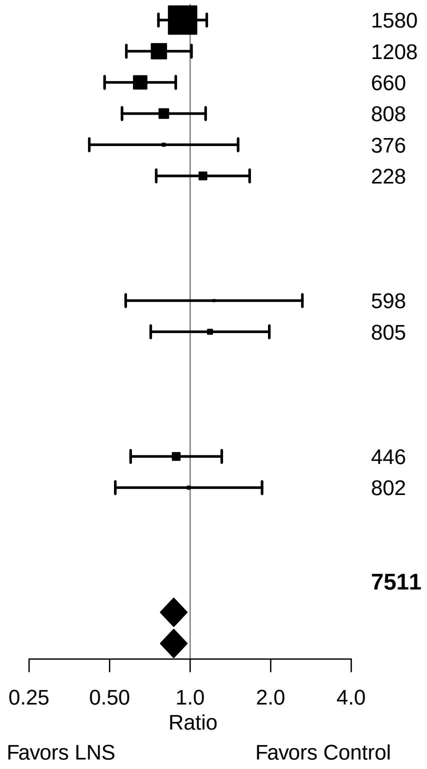

Supplemental figure 8E: Wasting prevalence ratio

8E4: Stratified by Maternal education

P−for−interaction = 0.890

Ratio of PRs = 1.01 (0.83, 1.23)

Country	Trial	Primary or greater: LNS N	Control N	Control Prevalence	PR (95% CI)	Fixed W	Random W	Incomplete or no formal: LNS N	Control N	Control Prevalence	PR (95% CI)	Fixed W	Random W
Bangladesh	JiVitA−4 (34)	1797	739	15.2	0.94 (0.77, 1.16)	0.38	0.38	984	475	18.3	0.88 (0.70, 1.09)	0.34	0.34
Bangladesh	RDNS (35)	1245	589	14.9	0.81 (0.63, 1.05)	0.23	0.23	416	226	15.5	1.02 (0.74, 1.42)	0.15	0.15
Bangladesh	WASH−B (36)	813	2447	9.9	0.77 (0.61, 0.97)	0.28	0.28	343	977	14.4	0.81 (0.58, 1.13)	0.15	0.15
Burkina Faso	iLiNS−Zinc (37)												
Burkina Faso	PROMIS (38)												
Burkina Faso	PROMIS CS (38)												
Ghana	GHANA (39)												
Ghana	iLiNS−DYADG (40)	267	548	6.6	0.97 (0.55, 1.69)	0.05	0.05	80	144	11.1	0.56 (0.21, 1.48)	0.02	0.02
Haiti	HAITI (41)												
Kenya	WASH−B (42)	700	2426	1.2	1.11 (0.51, 2.44)	0.03	0.03	753	2688	1.8	0.74 (0.35, 1.56)	0.03	0.03
Madagascar	MAHAY (43)	351	431	3.2	1.05 (0.49, 2.24)	0.03	0.03	1349	1251	6.7	0.92 (0.62, 1.36)	0.11	0.11
Malawi	iLiNS−DYADM (44)												
Malawi	iLiNS−DOSE (45)												
Mali	PROMIS (46)												
Mali	PROMIS CS (46)	107	96	9.4	0.50 (0.17, 1.49)	0.01	0.01	836	863	10.4	0.87 (0.65, 1.16)	0.20	0.20
Zimbabwe	SHINE (HIV−) (47)												
Zimbabwe	SHINE (HIV+) (48)												
		**5280**	**7276**		**I² = 0.00, Tau² = 0.00**			**4761**	**6624**		**I² = 0.00, Tau² = 0.00**		
**Fixed**					**0.86 (0.76, 0.97)**						**0.88 (0.77, 1.00)**		
**Random**					**0.86 (0.76, 0.97)**						**0.88 (0.77, 1.00)**		

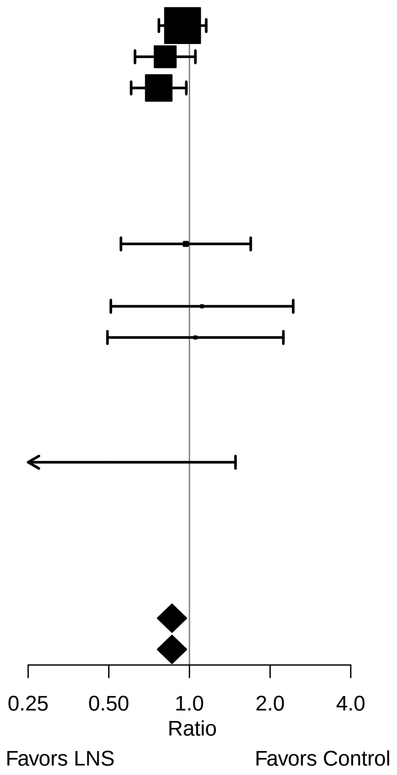

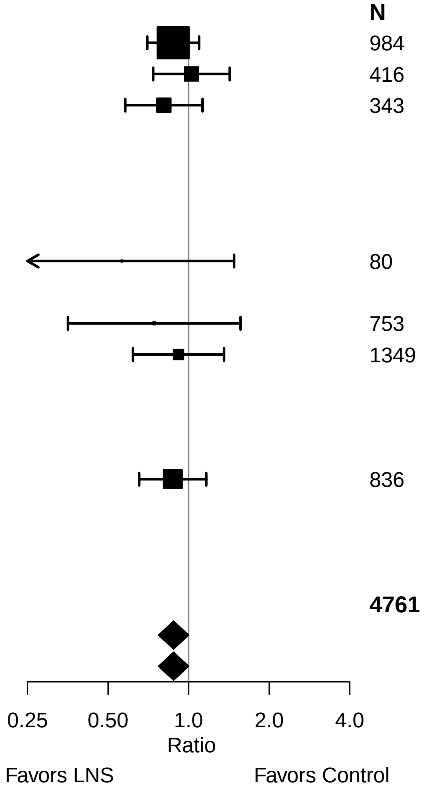

Supplemental figure 8E: Wasting prevalence ratio

8E5: Stratified by Maternal depressive symptoms

P−for−interaction = 0.265
Ratio of PRs = 1.15 (0.90, 1.48)

		Less than 75th percentile						At least 75th percentile					
**Country**	**Trial**	**LNS N**	**Control N**	**Control Prevalence**	**PR (95% CI)**	**Fixed W**	**Random W**	**LNS N**	**Control N**	**Control Prevalence**	**PR (95% CI)**	**Fixed W**	**Random W**
Bangladesh	JiVitA−4 (34)												
Bangladesh	RDNS (35)	1081	472	13.6	1.00 (0.76, 1.32)	0.27	0.27	519	288	16.7	0.72 (0.48, 1.07)	0.30	0.30
Bangladesh	WASH−B (36)	895	2457	10.9	0.81 (0.65, 1.01)	0.41	0.41	237	876	12.0	0.74 (0.49, 1.10)	0.29	0.29
Burkina Faso	iLiNS−Zinc (37)												
Burkina Faso	PROMIS (38)	505	569	8.3	0.86 (0.56, 1.34)	0.10	0.10	219	212	12.7	0.72 (0.41, 1.26)	0.15	0.15
Burkina Faso	PROMIS CS (38)												
Ghana	GHANA (39)												
Ghana	iLiNS−DYADG (40)	252	459	6.8	0.88 (0.49, 1.60)	0.05	0.05	83	209	8.1	1.04 (0.45, 2.41)	0.07	0.07
Haiti	HAITI (41)												
Kenya	WASH−B (42)												
Madagascar	MAHAY (43)	672	721	5.8	0.95 (0.58, 1.54)	0.08	0.08	283	252	4.0	1.16 (0.54, 2.50)	0.08	0.08
Malawi	iLiNS−DYADM (44)												
Malawi	iLiNS−DOSE (45)												
Mali	PROMIS (46)												
Mali	PROMIS CS (46)												
Zimbabwe	SHINE (HIV−) (47)	1192	1136	2.7	0.83 (0.49, 1.41)	0.07	0.07	509	489	2.7	0.81 (0.39, 1.69)	0.09	0.09
Zimbabwe	SHINE (HIV+) (48)	234	214	2.3	2.01 (0.74, 5.44)	0.02	0.02	88	100	6.0	0.95 (0.28, 3.16)	0.03	0.03
		**4831**	**6028**		**I² = 0.00, Tau² = 0.00**			**1938**	**2426**		**I² = 0.00, Tau² = 0.00**		
**Fixed**					**0.90 (0.78, 1.03)**						**0.79 (0.63, 0.98)**		
**Random**					**0.90 (0.78, 1.03)**						**0.79 (0.63, 0.98)**		

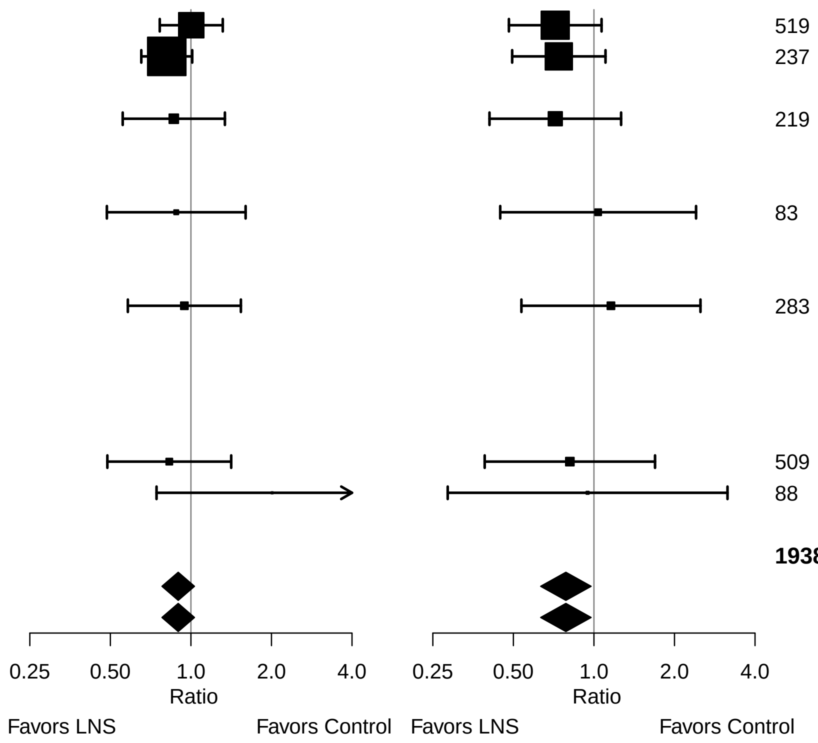

Supplemental figure 8E: Wasting prevalence ratio

8E6: Stratified by Child sex

P−for−interaction = 0.074
Ratio of PRs = 0.87 (0.75, 1.01)

		Male						Female					
**Country**	**Trial**	**LNS N**	**Control N**	**Control Prevalence**	**PR (95% CI)**	**Fixed W**	**Random W**	**LNS N**	**Control N**	**Control Prevalence**	**PR (95% CI)**	**Fixed W**	**Random W**
Bangladesh	JiVitA−4 (34)	1420	586	15.6	1.04 (0.85, 1.28)	0.24	0.24	1363	631	17.1	0.77 (0.63, 0.95)	0.33	0.28
Bangladesh	RDNS (35)	832	411	15.6	0.90 (0.68, 1.18)	0.14	0.14	829	404	14.6	0.83 (0.61, 1.15)	0.13	0.14
Bangladesh	WASH−B (36)	564	1711	12.2	0.90 (0.71, 1.16)	0.17	0.17	592	1713	10.3	0.66 (0.48, 0.90)	0.14	0.14
Burkina Faso	iLiNS−Zinc (37)	990	336	14.9	0.75 (0.57, 0.98)	0.15	0.15	962	328	12.2	0.62 (0.44, 0.87)	0.12	0.13
Burkina Faso	PROMIS (38)	434	480	12.5	0.72 (0.43, 1.20)	0.04	0.04	422	427	4.9	1.30 (0.76, 2.23)	0.05	0.05
Burkina Faso	PROMIS CS (38)	234	221	13.6	1.16 (0.70, 1.94)	0.04	0.04	196	215	11.2	0.82 (0.44, 1.52)	0.04	0.04
Ghana	GHANA (39)												
Ghana	iLiNS−DYADG (40)	175	323	9.9	0.63 (0.33, 1.23)	0.02	0.02	172	369	5.4	1.18 (0.58, 2.41)	0.03	0.03
Haiti	HAITI (41)												
Kenya	WASH−B (42)	732	2431	2.0	0.97 (0.50, 1.89)	0.02	0.02	723	2687	1.0	0.66 (0.25, 1.75)	0.01	0.02
Madagascar	MAHAY (43)	824	832	8.1	0.90 (0.59, 1.39)	0.06	0.06	876	850	3.6	1.10 (0.67, 1.80)	0.06	0.06
Malawi	iLiNS−DYADM (44)												
Malawi	iLiNS−DOSE (45)												
Mali	PROMIS (46)												
Mali	PROMIS CS (46)	472	520	11.5	0.94 (0.63, 1.40)	0.07	0.07	472	439	8.9	0.72 (0.45, 1.14)	0.06	0.07
Zimbabwe	SHINE (HIV−) (47)	924	905	3.3	0.78 (0.47, 1.32)	0.04	0.04	945	879	1.8	1.10 (0.57, 2.16)	0.03	0.04
Zimbabwe	SHINE (HIV+) (48)												
		**7601**	**8756**		**I² = 0.00, Tau² = 0.00**			**7552**	**8942**		**I² = 0.08, Tau² = 0.00**		
**Fixed**					**0.90 (0.81, 1.00)**						**0.79 (0.70, 0.89)**		
**Random**					**0.90 (0.81, 1.00)**						**0.80 (0.70, 0.91)**		

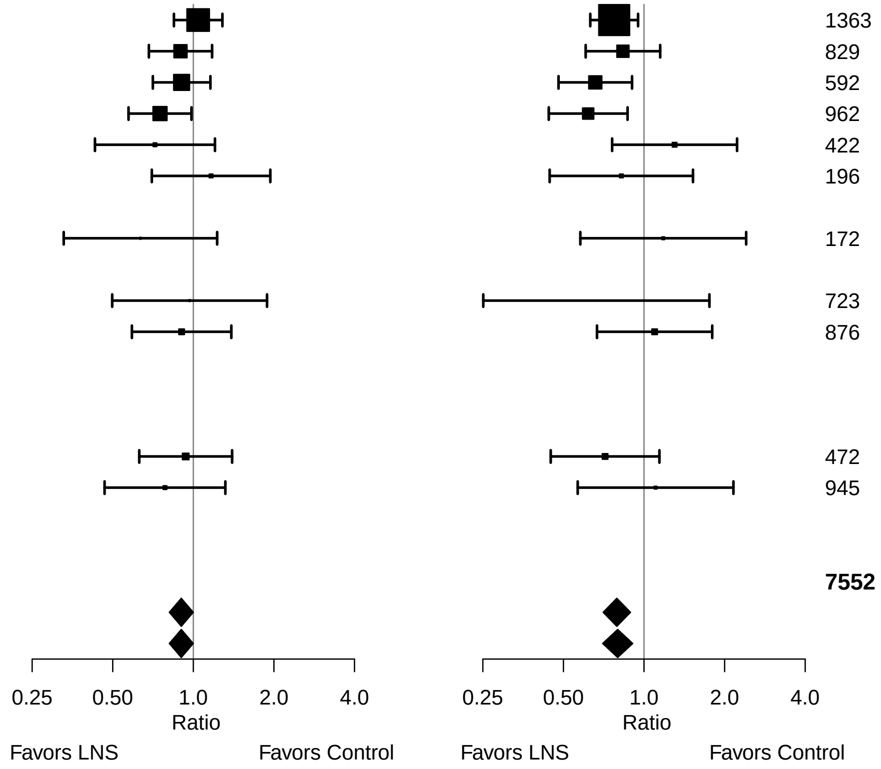

Supplemental figure 8E: Wasting prevalence ratio

8E7: Stratified by Child birth order

P−for−interaction = 0.609
Ratio of PRs = 1.05 (0.88, 1.25)

Country	Trial	Later born LNS N	Later born Control N	Later born Control Prevalence	Later born PR (95% CI)	Later born Fixed W	Later born Random W	Firstborn LNS N	Firstborn Control N	Firstborn Control Prevalence	Firstborn PR (95% CI)	Firstborn Fixed W	Firstborn Random W
Bangladesh	JiVitA−4 (34)	598	247	17.1	0.87 (0.64, 1.19)	0.11	0.11	2181	964	16.2	0.93 (0.78, 1.10)	0.50	0.50
Bangladesh	RDNS (35)	972	508	15.7	0.86 (0.66, 1.10)	0.16	0.16	688	307	14.0	0.89 (0.63, 1.27)	0.12	0.12
Bangladesh	WASH−B (36)	735	2285	11.3	0.78 (0.59, 1.02)	0.14	0.14	421	1139	11.0	0.80 (0.58, 1.11)	0.14	0.14
Burkina Faso	iLiNS−Zinc (37)	1520	519	13.7	0.64 (0.51, 0.80)	0.21	0.21	432	145	13.1	0.84 (0.50, 1.42)	0.06	0.06
Burkina Faso	PROMIS (38)	708	747	9.4	0.87 (0.61, 1.26)	0.08	0.08	148	158	7.0	0.78 (0.34, 1.75)	0.02	0.02
Burkina Faso	PROMIS CS (38)	366	357	11.5	1.14 (0.74, 1.76)	0.05	0.05	63	79	16.5	0.68 (0.33, 1.39)	0.03	0.03
Ghana	GHANA (39)												
Ghana	iLiNS−DYADG (40)												
Haiti	HAITI (41)												
Kenya	WASH−B (42)	1132	4027	1.5	0.83 (0.45, 1.52)	0.03	0.03	321	1086	1.5	1.06 (0.41, 2.75)	0.02	0.02
Madagascar	MAHAY (43)	1270	1217	6.4	0.91 (0.62, 1.34)	0.07	0.07	430	465	4.3	1.14 (0.67, 1.94)	0.05	0.05
Malawi	iLiNS−DYADM (44)												
Malawi	iLiNS−DOSE (45)												
Mali	PROMIS (46)												
Mali	PROMIS CS (46)	783	821	10.5	0.85 (0.64, 1.14)	0.12	0.12	144	126	9.5	0.51 (0.22, 1.19)	0.02	0.02
Zimbabwe	SHINE (HIV−) (47)	1295	1218	2.5	0.82 (0.49, 1.38)	0.04	0.04	487	483	2.9	0.99 (0.50, 1.98)	0.03	0.03
Zimbabwe	SHINE (HIV+) (48)												
		**9379**	**11946**		**I² = 0.00, Tau² = 0.00**			**5315**	**4952**		**I² = 0.00, Tau² = 0.00**		
**Fixed**					**0.81 (0.73, 0.90)**						**0.89 (0.79, 1.01)**		
**Random**					**0.81 (0.73, 0.90)**						**0.89 (0.79, 1.01)**		

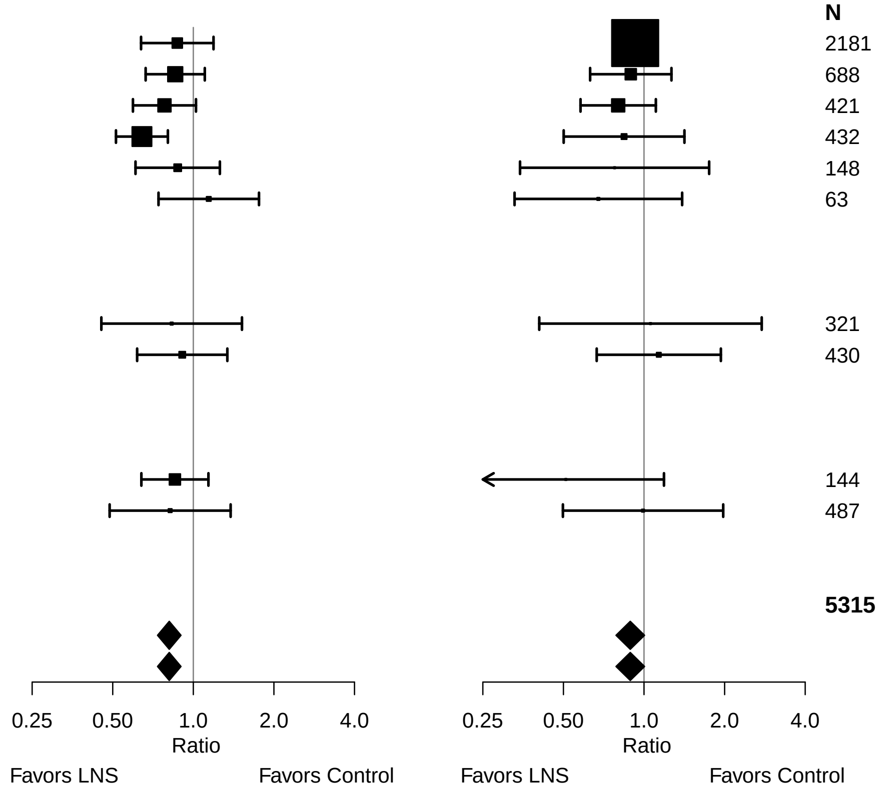

Supplemental figure 8E: Wasting prevalence ratio

8E8: Stratified by Child baseline anthropometric status

P−for−interaction = 0.682
Ratio of PRs = 0.89 (0.50, 1.58)

					WLZ at least 0 Z						WLZ less than 0 Z		
**Country**	**Trial**	**LNS N**	**Control N**	**Control Prevalence**	**PR (95% CI)**	**Fixed W**	**Random W**	**LNS N**	**Control N**	**Control Prevalence**	**PR (95% CI)**	**Fixed W**	**Random W**
Bangladesh	JiVitA−4 (34)	961	423	2.4	0.88 (0.41, 1.89)	0.49	0.49	1822	794	23.9	0.90 (0.78, 1.04)	0.84	0.84
Bangladesh	RDNS (35)												
Bangladesh	WASH−B (36)												
Burkina Faso	iLiNS−Zinc (37)												
Burkina Faso	PROMIS (38)	279	350	2.0	0.90 (0.25, 3.19)	0.18	0.18	413	393	14.2	0.80 (0.54, 1.18)	0.12	0.12
Burkina Faso	PROMIS CS (38)												
Ghana	GHANA (39)												
Ghana	iLiNS−DYADG (40)												
Haiti	HAITI (41)												
Kenya	WASH−B (42)												
Madagascar	MAHAY (43)												
Malawi	iLiNS−DYADM (44)												
Malawi	iLiNS−DOSE (45)												
Mali	PROMIS (46)												
Mali	PROMIS CS (46)												
Zimbabwe	SHINE (HIV−) (47)	743	702	1.0	1.08 (0.42, 2.76)	0.33	0.33	555	479	4.8	0.68 (0.34, 1.33)	0.04	0.04
Zimbabwe	SHINE (HIV+) (48)												
		**1983**	**1475**		**I² = 0.00, Tau² = 0.00**			**2790**	**1666**		**I² = 0.00, Tau² = 0.00**		
**Fixed**					**0.94 (0.55, 1.62)**						**0.88 (0.77, 1.01)**		
**Random**					**0.94 (0.55, 1.62)**						**0.88 (0.77, 1.01)**		

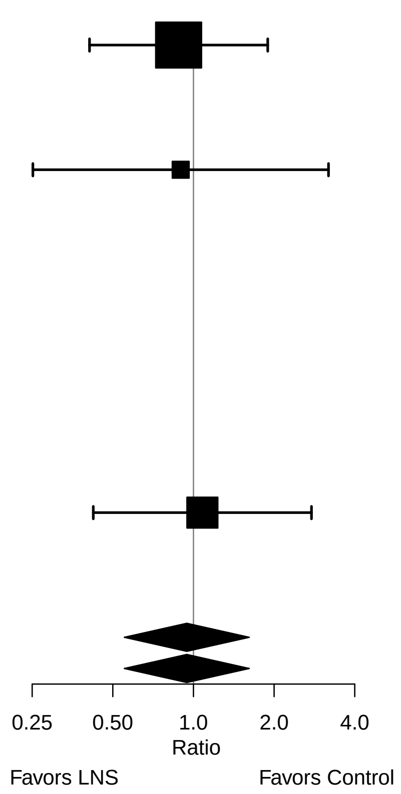

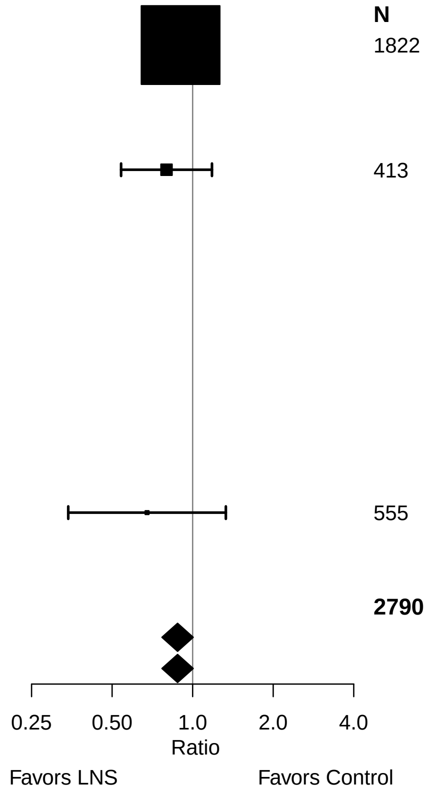

Supplemental figure 8F: Wasting prevalence difference

8F1: Stratified by Maternal height

P−for−interaction = 0.438
Difference in PDs = −0.01 (−0.03, 0.01)

Country	Trial	LNS N	Control N	Control Prevalence	At least 150.1 cm PD (95% CI)	Fixed W	Random W	LNS N	Control N	Control Prevalence	Less than 150.1 cm PD (95% CI)	Fixed W	Random W
Bangladesh	JiVitA−4 (34)												
Bangladesh	RDNS (35)	868	432	13.9	−0.02 (−0.05, 0.02)	0.12	0.16	733	358	16.8	−0.03 (−0.08, 0.02)	0.13	0.13
Bangladesh	WASH−B (36)	616	1880	10.2	−0.04 (−0.06, −0.01)	0.30	0.25	540	1541	12.5	−0.01 (−0.04, 0.02)	0.46	0.46
Burkina Faso	iLiNS−Zinc (37)												
Burkina Faso	PROMIS (38)												
Burkina Faso	PROMIS CS (38)												
Ghana	GHANA (39)												
Ghana	iLiNS−DYADG (40)												
Haiti	HAITI (41)												
Kenya	WASH−B (42)												
Madagascar	MAHAY (43)	1002	1037	4.6	0.01 (−0.01, 0.03)	0.27	0.23	598	565	8.1	−0.02 (−0.05, 0.01)	0.37	0.37
Malawi	iLiNS−DYADM (44)												
Malawi	iLiNS−DOSE (45)	580	198	4.0	0.01 (−0.02, 0.05)	0.11	0.15	115	42	11.9	−0.03 (−0.14, 0.07)	0.03	0.03
Mali	PROMIS (46)												
Mali	PROMIS CS (46)	907	927	10.0	−0.02 (−0.05, 0.01)	0.20	0.21	32	26	23.1	−0.01 (−0.18, 0.16)	0.01	0.01
Zimbabwe	SHINE (HIV−) (47)												
Zimbabwe	SHINE (HIV+) (48)												
		**3973**	**4474**		**I² = 0.60, Tau² = 0.00**			**2018**	**2532**		**I² = 0.00, Tau² = 0.00**		
**Fixed**					**−0.01 (−0.03, 0.00)**						**−0.02 (−0.04, 0.00)**		
**Random**					**−0.01 (−0.03, 0.01)**						**−0.02 (−0.04, 0.00)**		

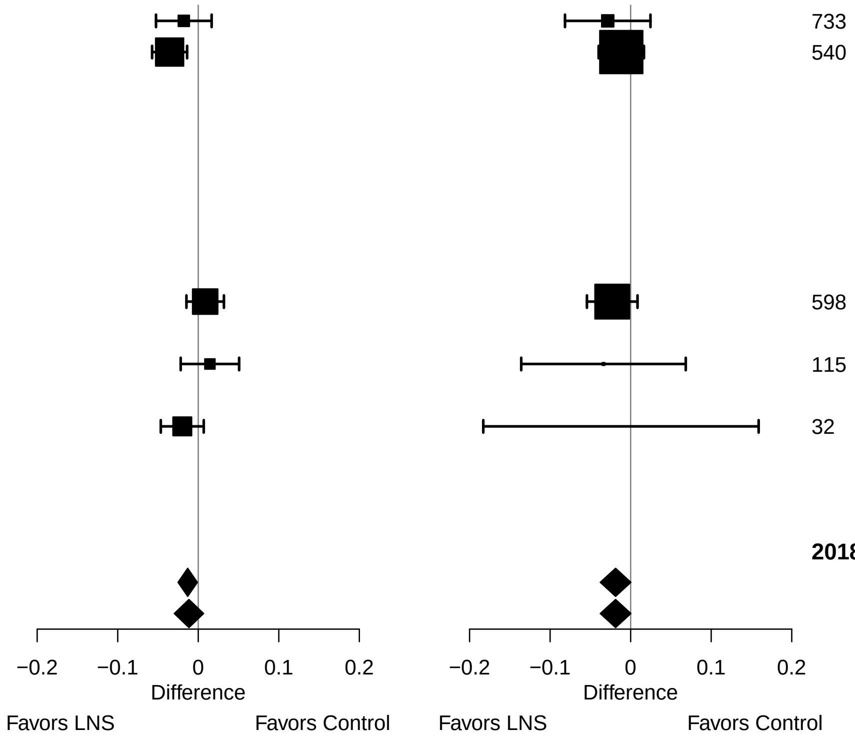

Supplemental figure 8F: Wasting prevalence difference

8F2: Stratified by Maternal BMI

P−for−interaction = 0.228
Difference in PDs = −0.01 (−0.03, 0.01)

		At least 20 kg/m²						Less than 20 kg/m²					
**Country**	**Trial**	**LNS N**	**Control N**	**Control Prevalence**	**PD (95% CI)**	**Fixed W**	**Random W**	**LNS N**	**Control N**	**Control Prevalence**	**PD (95% CI)**	**Fixed W**	**Random W**
Bangladesh	JiVitA−4 (34)												
Bangladesh	RDNS (35)	730	335	9.6	0.00 (−0.04, 0.03)	0.05	0.09	871	455	19.3	−0.03 (−0.08, 0.01)	0.12	0.12
Bangladesh	WASH−B (36)	517	1573	7.1	−0.01 (−0.04, 0.01)	0.13	0.16	639	1848	14.8	−0.03 (−0.06, −0.01)	0.33	0.33
Burkina Faso	iLiNS−Zinc (37)	1214	390	10.5	−0.04 (−0.06, −0.01)	0.11	0.14	736	274	17.9	−0.04 (−0.09, 0.00)	0.14	0.14
Burkina Faso	PROMIS (38)	644	669	7.3	−0.01 (−0.04, 0.02)	0.06	0.10	209	232	13.8	−0.03 (−0.09, 0.04)	0.05	0.05
Burkina Faso	PROMIS CS (38)	246	267	8.2	0.02 (−0.03, 0.06)	0.03	0.06	184	165	19.4	−0.03 (−0.10, 0.05)	0.04	0.04
Ghana	GHANA (39)												
Ghana	iLiNS−DYADG (40)												
Haiti	HAITI (41)												
Kenya	WASH−B (42)												
Madagascar	MAHAY (43)												
Malawi	iLiNS−DYADM (44)												
Malawi	iLiNS−DOSE (45)	513	174	4.6	0.00 (−0.04, 0.03)	0.05	0.08	180	65	7.7	0.02 (−0.06, 0.11)	0.03	0.03
Mali	PROMIS (46)												
Mali	PROMIS CS (46)	696	675	9.8	−0.03 (−0.06, 0.00)	0.08	0.11	243	278	11.9	0.01 (−0.04, 0.07)	0.08	0.08
Zimbabwe	SHINE (HIV−) (47)	1355	1299	2.4	0.00 (−0.01, 0.01)	0.49	0.26	239	248	5.2	−0.02 (−0.05, 0.01)	0.21	0.21
Zimbabwe	SHINE (HIV+) (48)												
		**5915**	**5382**		**I² = 0.32, Tau² = 0.00**			**3301**	**3565**		**I² = 0.00, Tau² = 0.00**		
**Fixed**					**−0.01 (−0.02, 0.00)**						**−0.03 (−0.04, −0.01)**		
**Random**					**−0.01 (−0.02, 0.00)**						**−0.03 (−0.04, −0.01)**		

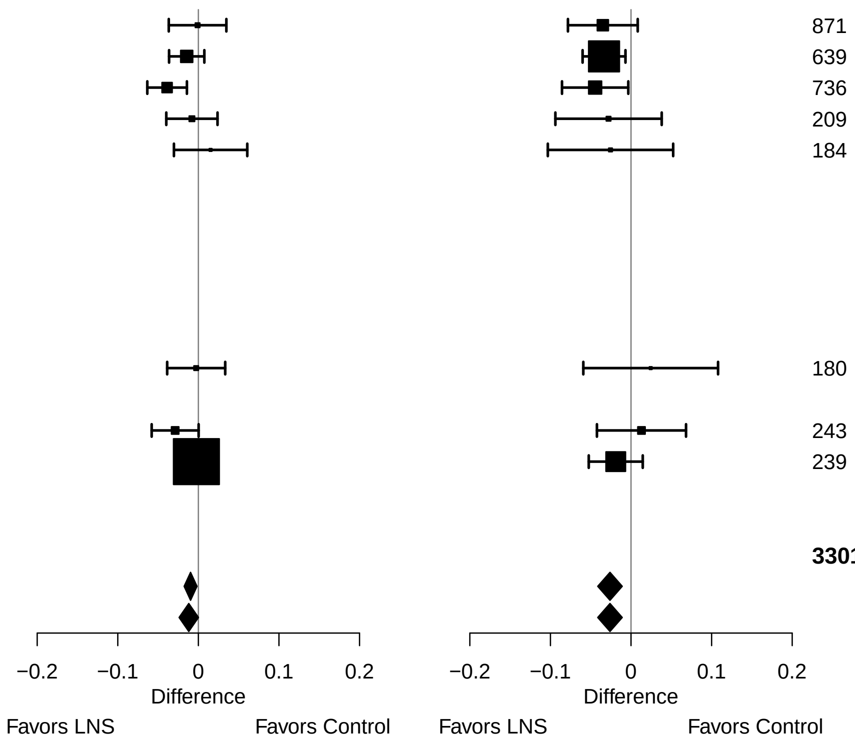

Supplemental figure 8F: Wasting prevalence difference

8F3: Stratified by Maternal age

P−for−interaction = 0.171
Difference in PDs = 0.01 (0.00, 0.02)

Country	Trial	LNS N	Control N	Control Prevalence	At least 25 y PD (95% CI)	Fixed W	Random W	LNS N	Control N	Control Prevalence	Less than 25 y PD (95% CI)	Fixed W	Random W
Bangladesh	JiVitA−4 (34)	1191	535	17.3	−0.02 (−0.05, 0.02)	0.03	0.03	1580	677	15.5	−0.01 (−0.04, 0.02)	0.05	0.09
Bangladesh	RDNS (35)	453	221	14.0	0.02 (−0.03, 0.08)	0.01	0.01	1208	594	15.5	−0.04 (−0.08, 0.00)	0.03	0.05
Bangladesh	WASH−B (36)	491	1508	11.6	−0.01 (−0.04, 0.03)	0.03	0.03	660	1900	10.9	−0.04 (−0.06, −0.01)	0.08	0.11
Burkina Faso	iLiNS−Zinc (37)	1131	399	14.0	−0.05 (−0.09, −0.01)	0.03	0.03	808	262	12.6	−0.03 (−0.07, 0.02)	0.03	0.05
Burkina Faso	PROMIS (38)	480	522	8.6	−0.01 (−0.04, 0.02)	0.04	0.04	376	385	9.4	−0.02 (−0.07, 0.03)	0.02	0.03
Burkina Faso	PROMIS CS (38)	202	190	13.2	−0.01 (−0.08, 0.07)	0.01	0.01	228	246	11.8	0.01 (−0.04, 0.06)	0.02	0.04
Ghana	GHANA (39)												
Ghana	iLiNS−DYADG (40)												
Haiti	HAITI (41)												
Kenya	WASH−B (42)	842	2801	1.7	−0.01 (−0.01, 0.00)	0.62	0.62	598	2285	1.2	0.00 (−0.01, 0.01)	0.40	0.24
Madagascar	MAHAY (43)	894	949	6.8	−0.01 (−0.03, 0.01)	0.07	0.07	805	733	4.5	0.01 (−0.02, 0.03)	0.08	0.11
Malawi	iLiNS−DYADM (44)												
Malawi	iLiNS−DOSE (45)												
Mali	PROMIS (46)												
Mali	PROMIS CS (46)	498	501	11.0	−0.02 (−0.07, 0.02)	0.02	0.02	446	458	9.6	−0.01 (−0.05, 0.02)	0.04	0.07
Zimbabwe	SHINE (HIV−) (47)	870	796	3.1	−0.01 (−0.03, 0.01)	0.14	0.14	802	792	2.1	0.00 (−0.01, 0.01)	0.26	0.21
Zimbabwe	SHINE (HIV+) (48)												
		**7052**	**8422**		**I² = 0.00, Tau² = 0.00**			**7511**	**8332**		**I² = 0.39, Tau² = 0.00**		
**Fixed**					**−0.01 (−0.02, 0.00)**						**0.00 (−0.01, 0.00)**		
**Random**					**−0.01 (−0.02, 0.00)**						**−0.01 (−0.02, 0.00)**		

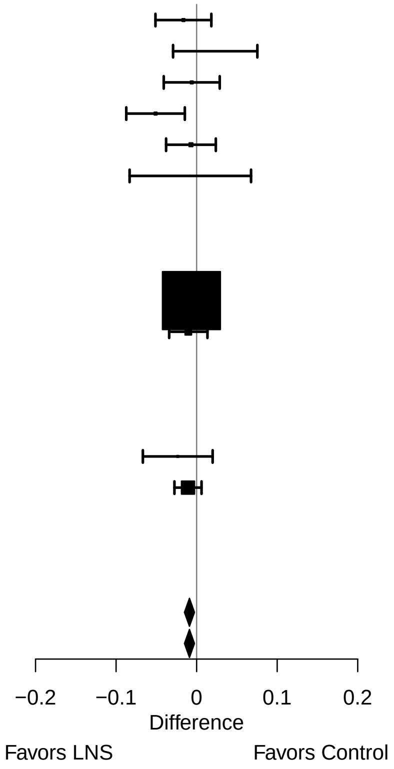

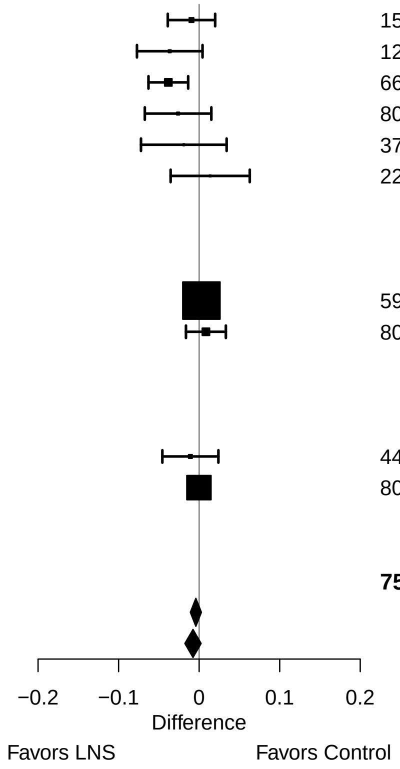

Supplemental figure 8F: Wasting prevalence difference

8F4: Stratified by Maternal education

P−for−interaction = 0.380
Difference in PDs = −0.01 (−0.02, 0.01)

		Primary or greater						Incomplete or no formal					
Country	Trial	LNS N	Control N	Control Prevalence	PD (95% CI)	Fixed W	Random W	LNS N	Control N	Control Prevalence	PD (95% CI)	Fixed W	Random W
Bangladesh	JiVitA−4 (34)	1797	739	15.2	−0.01 (−0.04, 0.02)	0.08	0.13	984	475	18.3	−0.02 (−0.06, 0.02)	0.05	0.05
Bangladesh	RDNS (35)	1245	589	14.9	−0.03 (−0.06, 0.01)	0.04	0.09	416	226	15.5	0.00 (−0.05, 0.06)	0.03	0.03
Bangladesh	WASH−B (36)	813	2447	9.9	−0.02 (−0.04, 0.00)	0.15	0.20	343	977	14.4	−0.03 (−0.07, 0.01)	0.04	0.04
Burkina Faso	iLiNS−Zinc (37)												
Burkina Faso	PROMIS (38)												
Burkina Faso	PROMIS CS (38)												
Ghana	GHANA (39)												
Ghana	iLiNS−DYADG (40)	267	548	6.6	0.00 (−0.04, 0.03)	0.04	0.09	80	144	11.1	−0.05 (−0.13, 0.03)	0.01	0.01
Haiti	HAITI (41)												
Kenya	WASH−B (42)	700	2426	1.2	0.00 (−0.01, 0.01)	0.59	0.32	753	2688	1.8	0.00 (−0.01, 0.01)	0.68	0.68
Madagascar	MAHAY (43)	351	431	3.2	0.00 (−0.02, 0.03)	0.09	0.15	1349	1251	6.7	−0.01 (−0.03, 0.02)	0.11	0.11
Malawi	iLiNS−DYADM (44)												
Malawi	iLiNS−DOSE (45)												
Mali	PROMIS (46)												
Mali	PROMIS CS (46)	107	96	9.4	−0.05 (−0.12, 0.02)	0.01	0.03	836	863	10.4	−0.01 (−0.04, 0.01)	0.09	0.09
Zimbabwe	SHINE (HIV−) (47)												
Zimbabwe	SHINE (HIV+) (48)												
		**5280**	**7276**		**I² = 0.27, Tau² = 0.00**			**4761**	**6624**		**I² = 0.00, Tau² = 0.00**		
**Fixed**					**−0.01 (−0.01, 0.00)**						**−0.01 (−0.02, 0.00)**		
**Random**					**−0.01 (−0.02, 0.00)**						**−0.01 (−0.02, 0.00)**		

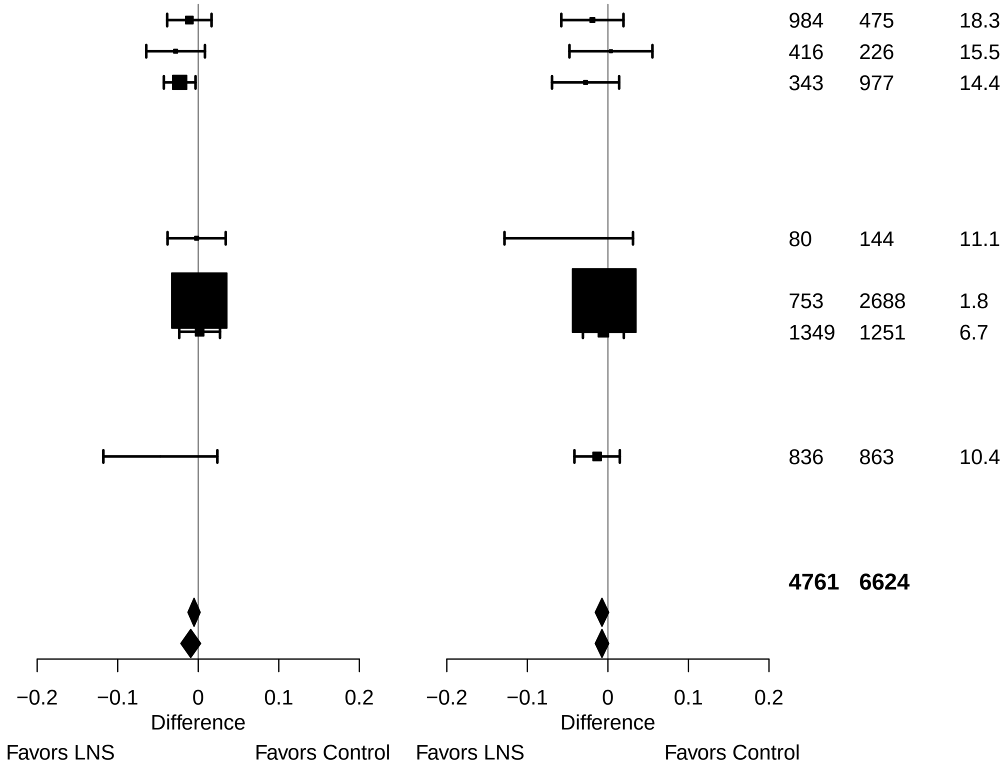

Supplemental figure 8F: Wasting prevalence difference

8F5: Stratified by Maternal depressive symptoms

P−for−interaction = 0.511
Difference in PDs = 0.01 (−0.01, 0.02)

		Less than 75th percentile						At least 75th percentile					
**Country**	**Trial**	**LNS N**	**Control N**	**Control Prevalence**	**PD (95% CI)**	**Fixed W**	**Random W**	**LNS N**	**Control N**	**Control Prevalence**	**PD (95% CI)**	**Fixed W**	**Random W**
Bangladesh	JiVitA−4 (34)												
Bangladesh	RDNS (35)	1081	472	13.6	0.00 (−0.04, 0.04)	0.06	0.06	519	288	16.7	−0.05 (−0.11, 0.01)	0.05	0.05
Bangladesh	WASH−B (36)	895	2457	10.9	−0.02 (−0.04, 0.00)	0.18	0.18	237	876	12.0	−0.03 (−0.07, 0.01)	0.12	0.12
Burkina Faso	iLiNS−Zinc (37)												
Burkina Faso	PROMIS (38)	505	569	8.3	−0.01 (−0.05, 0.02)	0.07	0.07	219	212	12.7	−0.04 (−0.10, 0.03)	0.04	0.04
Burkina Faso	PROMIS CS (38)												
Ghana	GHANA (39)												
Ghana	iLiNS−DYADG (40)	252	459	6.8	−0.01 (−0.05, 0.03)	0.05	0.05	83	209	8.1	0.00 (−0.07, 0.07)	0.04	0.04
Haiti	HAITI (41)												
Kenya	WASH−B (42)												
Madagascar	MAHAY (43)	672	721	5.8	0.00 (−0.03, 0.02)	0.10	0.10	283	252	4.0	0.01 (−0.03, 0.04)	0.16	0.16
Malawi	iLiNS−DYADM (44)												
Malawi	iLiNS−DOSE (45)												
Mali	PROMIS (46)												
Mali	PROMIS CS (46)												
Zimbabwe	SHINE (HIV−) (47)	1192	1136	2.7	0.00 (−0.02, 0.01)	0.45	0.45	509	489	2.7	0.00 (−0.02, 0.01)	0.56	0.56
Zimbabwe	SHINE (HIV+) (48)	234	214	2.3	0.02 (−0.01, 0.05)	0.08	0.08	88	100	6.0	0.00 (−0.07, 0.07)	0.03	0.03
		**4831**	**6028**		**I² = 0.00, Tau² = 0.00**			**1938**	**2426**		**I² = 0.00, Tau² = 0.00**		
**Fixed**					**−0.01 (−0.01, 0.00)**						**−0.01 (−0.02, 0.00)**		
**Random**					**−0.01 (−0.01, 0.00)**						**−0.01 (−0.02, 0.00)**		

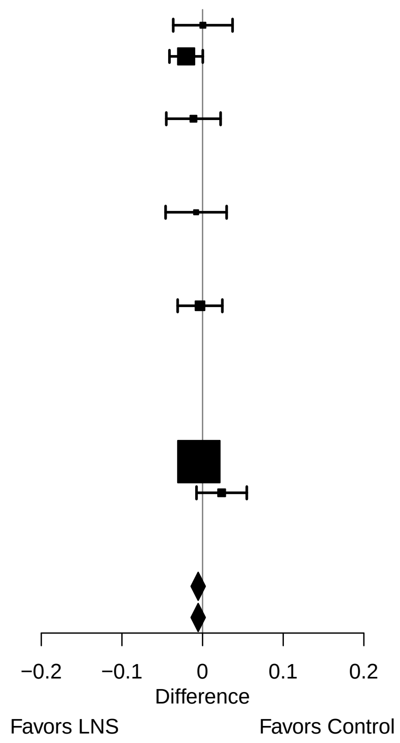

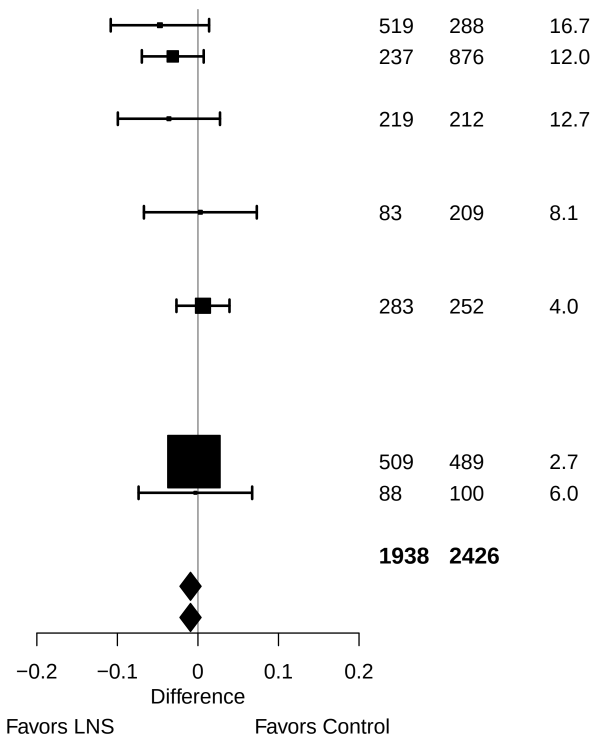

Supplemental figure 8F: Wasting prevalence difference

8F6: Stratified by Child sex

P−for−interaction = 0.876
Difference in PDs = 0.00 (−0.01, 0.01)

Country	Trial	Male LNS N	Male Control N	Male Control Prevalence	Male PD (95% CI)	Male Fixed W	Male Random W	Female LNS N	Female Control N	Female Control Prevalence	Female PD (95% CI)	Female Fixed W	Female Random W
Bangladesh	JiVitA−4 (34)	1420	586	15.6	0.01 (−0.03, 0.04)	0.06	0.06	1363	631	17.1	−0.04 (−0.07, −0.01)	0.03	0.09
Bangladesh	RDNS (35)	832	411	15.6	−0.02 (−0.06, 0.02)	0.04	0.04	829	404	14.6	−0.02 (−0.07, 0.02)	0.02	0.05
Bangladesh	WASH−B (36)	564	1711	12.2	−0.01 (−0.04, 0.02)	0.08	0.08	592	1713	10.3	−0.04 (−0.06, −0.01)	0.05	0.10
Burkina Faso	iLiNS−Zinc (37)	990	336	14.9	−0.03 (−0.06, 0.00)	0.06	0.06	962	328	12.2	−0.05 (−0.09, −0.02)	0.02	0.07
Burkina Faso	PROMIS (38)	434	480	12.5	−0.04 (−0.09, 0.02)	0.02	0.02	422	427	4.9	0.01 (−0.01, 0.04)	0.04	0.09
Burkina Faso	PROMIS CS (38)	234	221	13.6	0.02 (−0.05, 0.10)	0.01	0.01	196	215	11.2	−0.02 (−0.08, 0.04)	0.01	0.03
Ghana	GHANA (39)												
Ghana	iLiNS−DYADG (40)	175	323	9.9	−0.04 (−0.09, 0.02)	0.02	0.02	172	369	5.4	0.01 (−0.03, 0.05)	0.02	0.06
Haiti	HAITI (41)												
Kenya	WASH−B (42)	732	2431	2.0	0.00 (−0.01, 0.01)	0.37	0.37	723	2687	1.0	0.00 (−0.01, 0.00)	0.53	0.17
Madagascar	MAHAY (43)	824	832	8.1	−0.01 (−0.04, 0.03)	0.06	0.06	876	850	3.6	0.00 (−0.02, 0.02)	0.08	0.12
Malawi	iLiNS−DYADM (44)												
Malawi	iLiNS−DOSE (45)												
Mali	PROMIS (46)												
Mali	PROMIS CS (46)	472	520	11.5	−0.01 (−0.05, 0.04)	0.03	0.03	472	439	8.9	−0.03 (−0.06, 0.01)	0.02	0.07
Zimbabwe	SHINE (HIV−) (47)	924	905	3.3	−0.01 (−0.02, 0.01)	0.26	0.26	945	879	1.8	0.00 (−0.01, 0.01)	0.18	0.15
Zimbabwe	SHINE (HIV+) (48)												
		**7601**	**8756**		**I² = 0.00, Tau² = 0.00**			**7552**	**8942**		**I² = 0.57, Tau² = 0.00**		
**Fixed**					**−0.01 (−0.01, 0.00)**						**−0.01 (−0.01, 0.00)**		
**Random**					**−0.01 (−0.01, 0.00)**						**−0.01 (−0.02, 0.00)**		

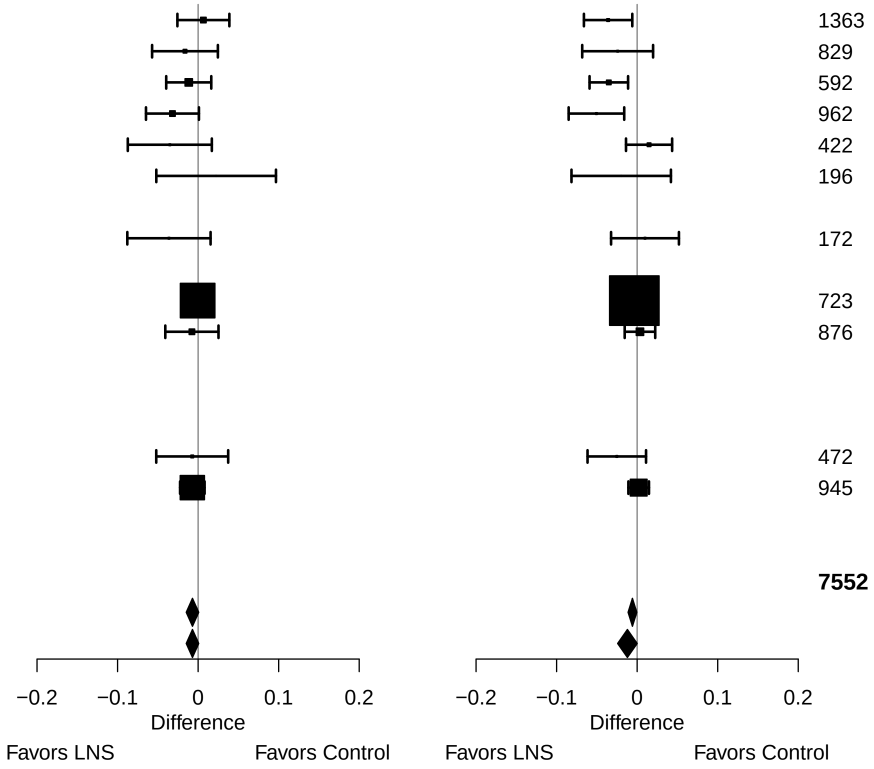

Supplemental figure 8F: Wasting prevalence difference

8F7: Stratified by Child birth order

P−for−interaction = 0.549
Difference in PDs = 0.00 (−0.01, 0.01)

Country	Trial	Later born LNS N	Later born Control N	Later born Control Prevalence	Later born PD (95% CI)	Later born Fixed W	Later born Random W	Firstborn LNS N	Firstborn Control N	Firstborn Control Prevalence	Firstborn PD (95% CI)	Firstborn Fixed W	Firstborn Random W
Bangladesh	JiVitA−4 (34)	598	247	17.1	−0.02 (−0.06, 0.03)	0.01	0.03	2181	964	16.2	−0.01 (−0.04, 0.01)	0.12	0.12
Bangladesh	RDNS (35)	972	508	15.7	−0.02 (−0.06, 0.02)	0.02	0.05	688	307	14.0	−0.02 (−0.06, 0.03)	0.03	0.03
Bangladesh	WASH−B (36)	735	2285	11.3	−0.02 (−0.05, 0.00)	0.05	0.09	421	1139	11.0	−0.02 (−0.05, 0.01)	0.08	0.08
Burkina Faso	iLiNS−Zinc (37)	1520	519	13.7	−0.05 (−0.07, −0.02)	0.05	0.09	432	145	13.1	−0.02 (−0.07, 0.03)	0.03	0.03
Burkina Faso	PROMIS (38)	708	747	9.4	−0.01 (−0.04, 0.02)	0.03	0.07	148	158	7.0	−0.02 (−0.06, 0.03)	0.03	0.03
Burkina Faso	PROMIS CS (38)	366	357	11.5	0.02 (−0.04, 0.07)	0.01	0.03	63	79	16.5	−0.05 (−0.15, 0.04)	0.01	0.01
Ghana	GHANA (39)												
Ghana	iLiNS−DYADG (40)												
Haiti	HAITI (41)												
Kenya	WASH−B (42)	1132	4027	1.5	0.00 (−0.01, 0.01)	0.52	0.27	321	1086	1.5	0.00 (−0.01, 0.02)	0.35	0.35
Madagascar	MAHAY (43)	1270	1217	6.4	−0.01 (−0.03, 0.02)	0.05	0.10	430	465	4.3	0.01 (−0.02, 0.03)	0.13	0.13
Malawi	iLiNS−DYADM (44)												
Malawi	iLiNS−DOSE (45)												
Mali	PROMIS (46)												
Mali	PROMIS CS (46)	783	821	10.5	−0.02 (−0.04, 0.01)	0.04	0.08	144	126	9.5	−0.05 (−0.11, 0.01)	0.02	0.02
Zimbabwe	SHINE (HIV−) (47)	1295	1218	2.5	0.00 (−0.02, 0.01)	0.21	0.21	487	483	2.9	0.00 (−0.02, 0.02)	0.19	0.19
Zimbabwe	SHINE (HIV+) (48)												
		**9379**	**11946**		**I² = 0.38, Tau² = 0.00**			**5315**	**4952**		**I² = 0.00, Tau² = 0.00**		
**Fixed**					**−0.01 (−0.01, 0.00)**						**−0.01 (−0.01, 0.00)**		
**Random**					**−0.01 (−0.02, 0.00)**						**−0.01 (−0.01, 0.00)**		

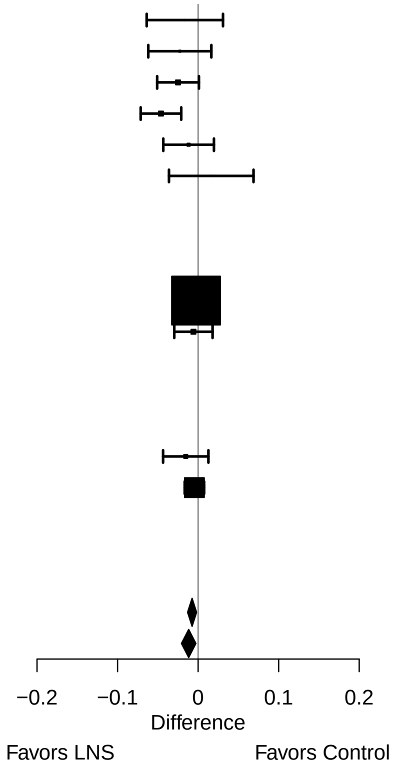

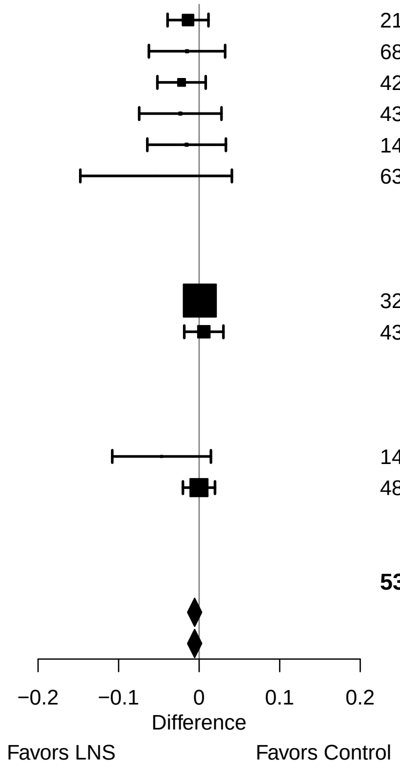

Supplemental figure 8F: Wasting prevalence difference

8F8: Stratified by Child baseline anthropometric status

P−for−interaction = 0.062
Difference in PDs = −0.02 (−0.04, 0.00)

		WLZ at least 0 Z						WLZ less than 0 Z					
**Country**	**Trial**	**LNS N**	**Control N**	**Control Prevalence**	**PD (95% CI)**	**Fixed W**	**Random W**	**LNS N**	**Control N**	**Control Prevalence**	**PD (95% CI)**	**Fixed W**	**Random W**
Bangladesh	JiVitA−4 (34)	961	423	2.4	0.00 (−0.02, 0.01)	0.21	0.21	1822	794	23.9	−0.02 (−0.06, 0.01)	0.32	0.32
Bangladesh	RDNS (35)												
Bangladesh	WASH−B (36)												
Burkina Faso	iLiNS−Zinc (37)												
Burkina Faso	PROMIS (38)	279	350	2.0	0.00 (−0.03, 0.02)	0.12	0.12	413	393	14.2	−0.03 (−0.08, 0.02)	0.16	0.16
Burkina Faso	PROMIS CS (38)												
Ghana	GHANA (39)												
Ghana	iLiNS−DYADG (40)												
Haiti	HAITI (41)												
Kenya	WASH−B (42)												
Madagascar	MAHAY (43)												
Malawi	iLiNS−DYADM (44)												
Malawi	iLiNS−DOSE (45)												
Mali	PROMIS (46)												
Mali	PROMIS CS (46)												
Zimbabwe	SHINE (HIV−) (47)	743	702	1.0	0.00 (−0.01, 0.01)	0.68	0.68	555	479	4.8	−0.02 (−0.04, 0.01)	0.52	0.52
Zimbabwe	SHINE (HIV+) (48)												
		**1983**	**1475**		**I² = 0.00, Tau² = 0.00**			**2790**	**1666**		**I² = 0.00, Tau² = 0.00**		
**Fixed**					**0.00 (−0.01, 0.01)**						**−0.02 (−0.04, 0.00)**		
**Random**					**0.00 (−0.01, 0.01)**						**−0.02 (−0.04, 0.00)**		

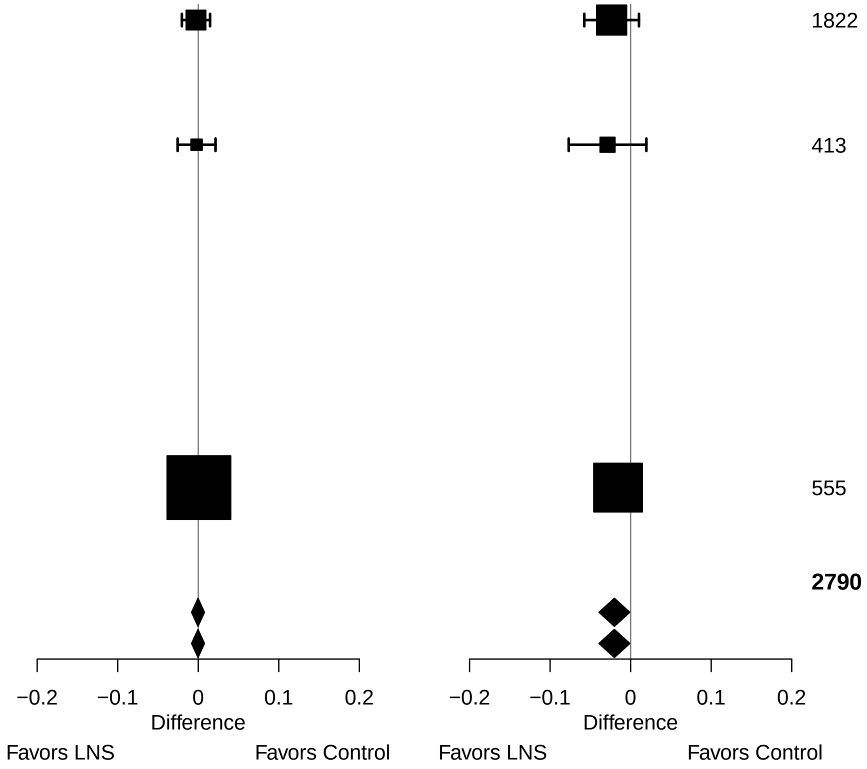

Supplemental figure 8G: Mean difference in MUACZ

8G1: Stratified by Maternal height

P−for−interaction = 0.016
Difference in MDs = −0.09 (−0.17, −0.02)

					At least 150.1 cm						Less than 150.1 cm		
		LNS	Control	Control	MD	Fixed	Random	LNS	Control	Control	MD	Fixed	Random
Country	Trial	N	N	Mean	(95% CI)	W	W	N	N	Mean	(95% CI)	W	W
Bangladesh	JiVitA−4 (34)												
Bangladesh	RDNS (35)	868	432	−0.65	0.06 (−0.02, 0.14)	0.12	0.10	735	358	−0.79	0.05 (−0.08, 0.17)	0.38	0.28
Bangladesh	WASH−B (36)												
Burkina Faso	iLiNS−Zinc (37)	1923	651	−1.13	0.30 (0.21, 0.39)	0.10	0.10	34	15	−1.22	0.04 (−0.35, 0.44)	0.04	0.05
Burkina Faso	PROMIS (38)	826	883	−0.73	0.10 (−0.03, 0.23)	0.05	0.07	25	24	−0.98	−0.13 (−0.56, 0.29)	0.03	0.04
Burkina Faso	PROMIS CS (38)												
Ghana	GHANA (39)												
Ghana	iLiNS−DYADG (40)	326	646	−0.46	0.09 (−0.03, 0.22)	0.06	0.08	16	36	−0.58	−0.12 (−0.61, 0.36)	0.02	0.03
Haiti	HAITI (41)												
Kenya	WASH−B (42)	1316	4728	−0.45	0.12 (0.05, 0.19)	0.17	0.11	57	195	−0.56	−0.04 (−0.35, 0.27)	0.06	0.08
Madagascar	MAHAY (43)	987	1021	−0.71	0.00 (−0.13, 0.13)	0.05	0.07	594	557	−0.78	−0.07 (−0.23, 0.09)	0.22	0.21
Malawi	iLiNS−DYADM (44)	192	381	0.09	0.01 (−0.16, 0.18)	0.03	0.05	28	60	−0.02	−0.39 (−0.88, 0.09)	0.02	0.03
Malawi	iLiNS−DOSE (45)	583	197	0.06	0.06 (−0.07, 0.18)	0.06	0.08	112	42	−0.15	0.13 (−0.15, 0.41)	0.07	0.09
Mali	PROMIS (46)	482	495	−0.78	0.20 (0.07, 0.34)	0.05	0.07	19	9	−0.79	−0.22 (−0.65, 0.21)	0.03	0.04
Mali	PROMIS CS (46)	916	937	−0.83	0.15 (0.04, 0.27)	0.06	0.08	33	27	−1.05	−0.30 (−0.75, 0.14)	0.03	0.04
Zimbabwe	SHINE (HIV−) (47)	1747	1684	0.00	0.07 (0.01, 0.13)	0.21	0.12	76	62	0.13	−0.16 (−0.43, 0.11)	0.08	0.09
Zimbabwe	SHINE (HIV+) (48)	314	311	−0.16	0.00 (−0.14, 0.14)	0.04	0.07	18	12	−0.85	0.68 (−0.02, 1.37)	0.01	0.02
		**10480**	**12366**		**I² = 0.63, Tau² = 0.00**			**1747**	**1397**		**I² = 0.13, Tau² = 0.00**		
**Fixed**					**0.10 (0.08, 0.13)**						**−0.03 (−0.10, 0.05)**		
**Random**					**0.10 (0.05, 0.15)**						**−0.04 (−0.13, 0.05)**		

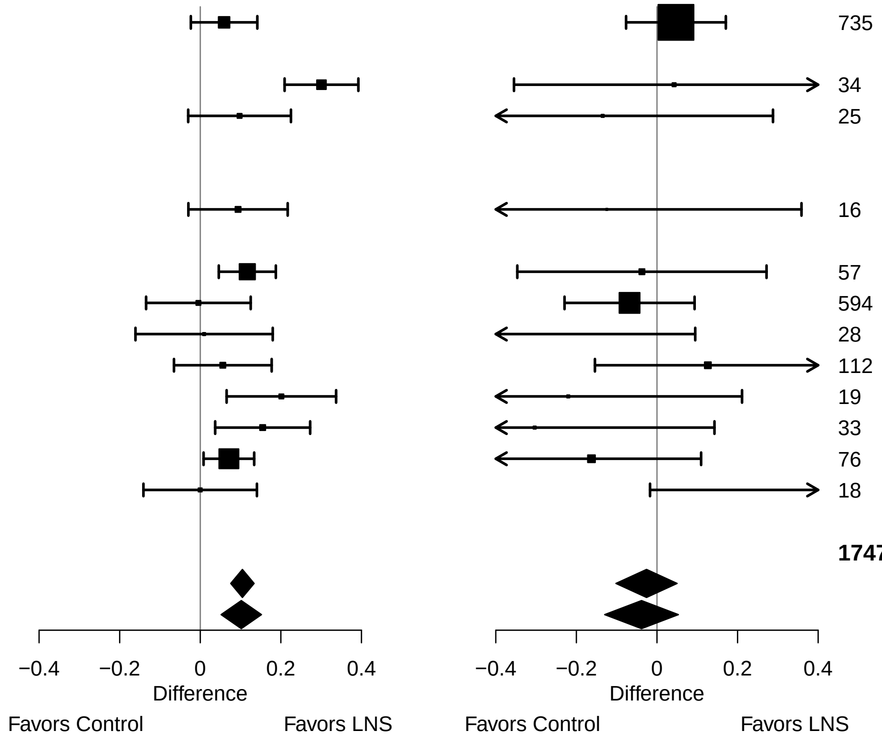

Supplemental figure 8G: Mean difference in MUACZ

8G2: Stratified by Maternal BMI

P−for−interaction = 0.580
Difference in MDs = −0.01 (−0.07, 0.04)

Country	Trial	At least 20 kg/m² LNS N	Control N	Control Mean	MD (95% CI)	Fixed W	Random W	Less than 20 kg/m² LNS N	Control N	Control Mean	MD (95% CI)	Fixed W	Random W
Bangladesh	JiVitA−4 (34)												
Bangladesh	RDNS (35)	730	335	−0.57	0.07 (−0.03, 0.18)	0.11	0.10	873	455	−0.82	0.02 (−0.08, 0.12)	0.19	0.15
Bangladesh	WASH−B (36)												
Burkina Faso	iLiNS−Zinc (37)	1218	391	−1.01	0.31 (0.19, 0.42)	0.08	0.09	739	275	−1.31	0.27 (0.17, 0.36)	0.23	0.16
Burkina Faso	PROMIS (38)	641	673	−0.66	0.11 (−0.02, 0.24)	0.06	0.08	210	233	−0.93	0.01 (−0.16, 0.18)	0.07	0.09
Burkina Faso	PROMIS CS (38)	241	266	−0.69	−0.12 (−0.30, 0.06)	0.03	0.06	183	166	−1.00	0.05 (−0.15, 0.24)	0.05	0.07
Ghana	GHANA (39)												
Ghana	iLiNS−DYADG (40)	305	563	−0.44	0.07 (−0.06, 0.20)	0.06	0.08	37	119	−0.61	0.12 (−0.21, 0.45)	0.02	0.03
Haiti	HAITI (41)												
Kenya	WASH−B (42)	1101	3837	−0.38	0.07 (0.00, 0.15)	0.19	0.11	272	1086	−0.73	0.22 (0.09, 0.35)	0.12	0.11
Madagascar	MAHAY (43)												
Malawi	iLiNS−DYADM (44)	131	263	0.21	−0.06 (−0.28, 0.15)	0.02	0.05	88	178	−0.12	0.00 (−0.24, 0.24)	0.03	0.05
Malawi	iLiNS−DOSE (45)	515	174	0.17	0.05 (−0.08, 0.17)	0.07	0.08	178	64	−0.38	0.11 (−0.12, 0.33)	0.04	0.06
Mali	PROMIS (46)	381	369	−0.71	0.20 (0.06, 0.35)	0.05	0.08	120	135	−0.96	0.14 (−0.07, 0.34)	0.05	0.06
Mali	PROMIS CS (46)	703	685	−0.78	0.16 (0.04, 0.29)	0.07	0.09	246	279	−0.97	0.05 (−0.10, 0.20)	0.09	0.10
Zimbabwe	SHINE (HIV−) (47)	1357	1294	0.03	0.09 (0.02, 0.16)	0.21	0.12	240	249	−0.19	0.06 (−0.10, 0.22)	0.08	0.09
Zimbabwe	SHINE (HIV+) (48)	247	253	−0.16	0.13 (−0.04, 0.30)	0.04	0.07	62	52	−0.38	−0.06 (−0.32, 0.21)	0.03	0.04
		**7570**	**9103**		**I² = 0.56, Tau² = 0.01**			**3248**	**3291**		**I² = 0.49, Tau² = 0.00**		
**Fixed**					**0.10 (0.07, 0.13)**						**0.12 (0.07, 0.16)**		
**Random**					**0.10 (0.04, 0.16)**						**0.10 (0.04, 0.16)**		

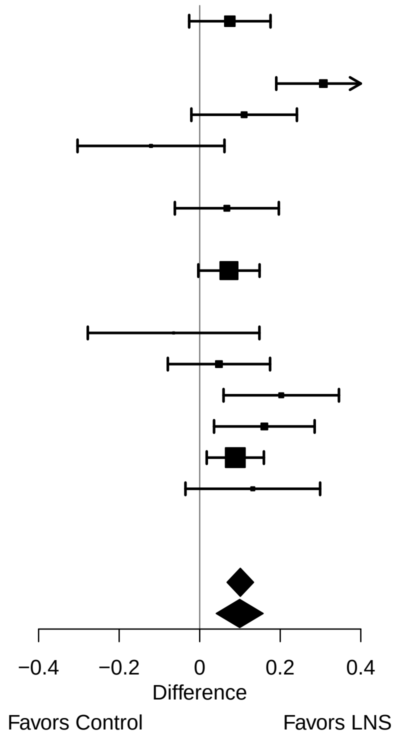

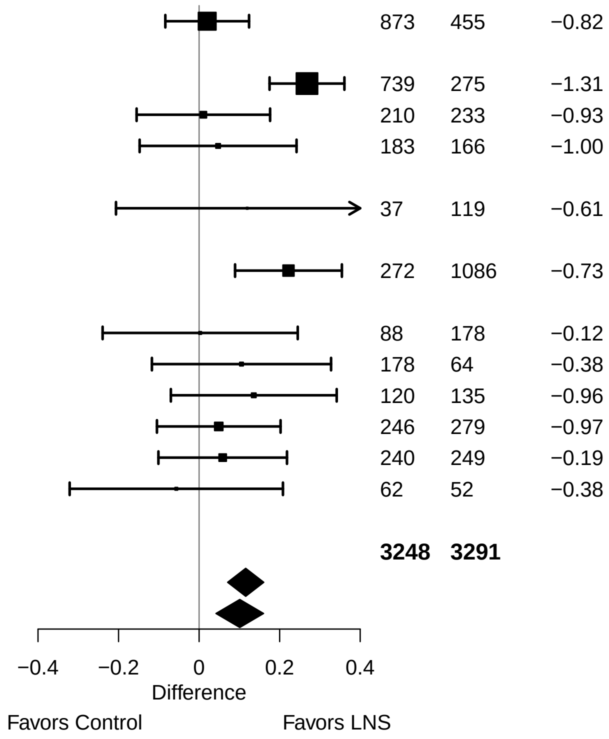

Supplemental figure 8G: Mean difference in MUACZ

8G3: Stratified by Maternal age

P−for−interaction = 0.395
Difference in MDs = −0.02 (−0.06, 0.02)

Country	Trial	At least 25 y LNS N	Control N	Control Mean	MD (95% CI)	Fixed W	Random W	Less than 25 y LNS N	Control N	Control Mean	MD (95% CI)	Fixed W	Random W
Bangladesh	JiVitA−4 (34)	1244	555	−1.07	0.09 (0.02, 0.15)	0.25	0.12	1640	711	−1.06	0.07 (0.01, 0.13)	0.26	0.14
Bangladesh	RDNS (35)	453	221	−0.70	0.02 (−0.13, 0.16)	0.05	0.07	1210	594	−0.70	0.05 (−0.03, 0.12)	0.16	0.12
Bangladesh	WASH−B (36)												
Burkina Faso	iLiNS−Zinc (37)	1134	401	−1.13	0.31 (0.20, 0.42)	0.08	0.08	812	262	−1.12	0.26 (0.13, 0.40)	0.05	0.07
Burkina Faso	PROMIS (38)	481	525	−0.77	0.12 (−0.02, 0.26)	0.05	0.07	373	387	−0.69	0.05 (−0.13, 0.23)	0.03	0.05
Burkina Faso	PROMIS CS (38)	200	190	−0.89	−0.03 (−0.20, 0.15)	0.03	0.05	224	246	−0.76	−0.08 (−0.28, 0.12)	0.02	0.04
Ghana	GHANA (39)												
Ghana	iLiNS−DYADG (40)	220	417	−0.43	0.03 (−0.13, 0.18)	0.04	0.06	127	276	−0.54	0.17 (0.00, 0.35)	0.03	0.05
Haiti	HAITI (41)												
Kenya	WASH−B (42)	845	2838	−0.44	0.13 (0.05, 0.22)	0.15	0.10	601	2315	−0.48	0.09 (0.00, 0.18)	0.12	0.11
Madagascar	MAHAY (43)	887	933	−0.77	−0.02 (−0.15, 0.11)	0.06	0.07	792	725	−0.69	−0.05 (−0.20, 0.10)	0.04	0.06
Malawi	iLiNS−DYADM (44)	106	226	0.10	−0.04 (−0.28, 0.19)	0.02	0.03	114	218	0.05	−0.03 (−0.25, 0.19)	0.02	0.04
Malawi	iLiNS−DOSE (45)	369	134	0.00	0.07 (−0.07, 0.21)	0.05	0.07	313	99	0.08	0.01 (−0.17, 0.18)	0.03	0.05
Mali	PROMIS (46)	347	358	−0.74	0.17 (0.02, 0.33)	0.04	0.06	160	147	−0.86	0.22 (0.05, 0.40)	0.03	0.05
Mali	PROMIS CS (46)	503	508	−0.84	0.13 (−0.02, 0.28)	0.04	0.06	451	462	−0.81	0.13 (−0.01, 0.26)	0.05	0.07
Zimbabwe	SHINE (HIV−) (47)	870	794	0.01	0.09 (0.00, 0.18)	0.12	0.10	805	790	0.01	0.04 (−0.05, 0.13)	0.13	0.11
Zimbabwe	SHINE (HIV+) (48)	244	230	−0.17	0.00 (−0.17, 0.16)	0.04	0.06	73	78	−0.22	0.12 (−0.16, 0.40)	0.01	0.02
		**7903**	**8330**		**I^2 = 0.52, Tau^2 = 0.00**			**7695**	**7310**		**I^2 = 0.35, Tau^2 = 0.00**		
**Fixed**					**0.10 (0.07, 0.13)**						**0.07 (0.04, 0.10)**		
**Random**					**0.09 (0.04, 0.14)**						**0.08 (0.03, 0.12)**		

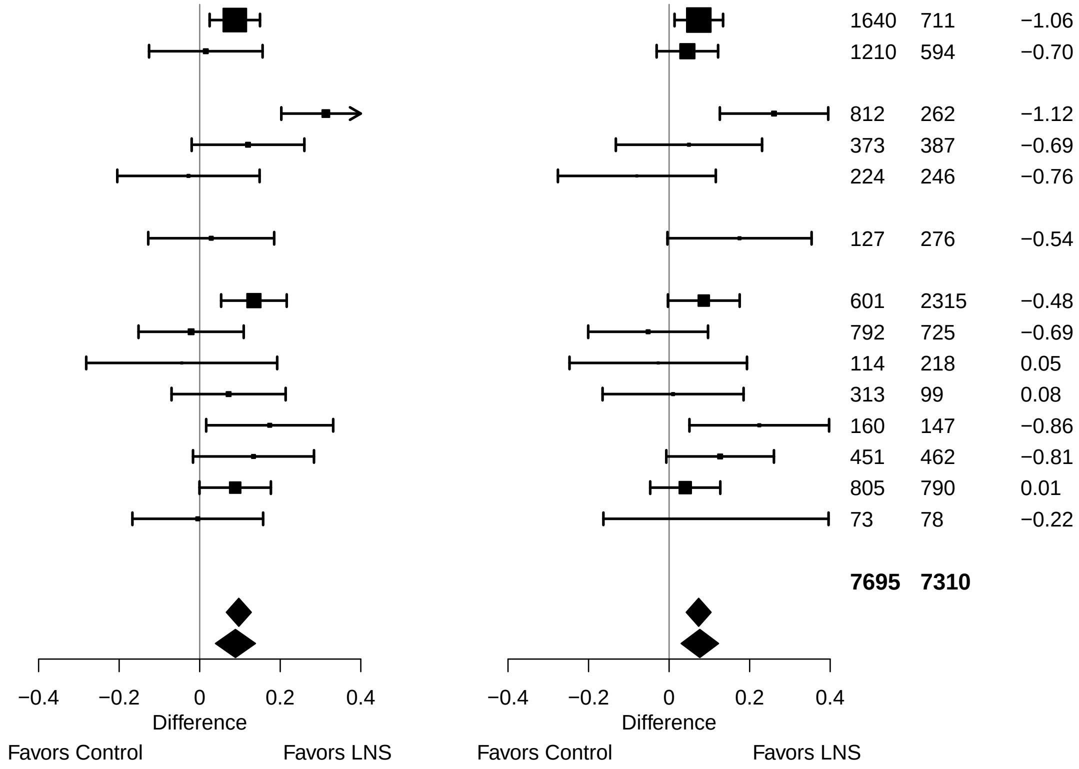

Supplemental figure 8G: Mean difference in MUACZ

8G4: Stratified by Maternal education

P−for−interaction = 0.688
Difference in MDs = 0.01 (−0.04, 0.06)

		Primary or greater						Incomplete or no formal					
Country	Trial	LNS N	Control N	Control Mean	MD (95% CI)	Fixed W	Random W	LNS N	Control N	Control Mean	MD (95% CI)	Fixed W	Random W
Bangladesh	JiVitA−4 (34)	1865	777	−0.98	0.08 (0.02, 0.13)	0.30	0.21	1030	491	−1.20	0.08 (0.01, 0.15)	0.20	0.11
Bangladesh	RDNS (35)	1245	589	−0.68	0.05 (−0.03, 0.14)	0.13	0.14	418	226	−0.76	−0.02 (−0.16, 0.12)	0.05	0.07
Bangladesh	WASH−B (36)												
Burkina Faso	iLiNS−Zinc (37)	82	16	−0.76	0.23 (−0.19, 0.66)	0.01	0.01	1864	647	−1.14	0.30 (0.21, 0.38)	0.14	0.10
Burkina Faso	PROMIS (38)	69	54	−0.78	0.09 (−0.19, 0.36)	0.01	0.02	785	858	−0.73	0.09 (−0.04, 0.22)	0.06	0.08
Burkina Faso	PROMIS CS (38)	34	39	−0.83	−0.03 (−0.53, 0.48)	0.00	0.01	388	396	−0.82	−0.06 (−0.22, 0.09)	0.04	0.07
Ghana	GHANA (39)												
Ghana	iLiNS−DYADG (40)	267	549	−0.44	0.04 (−0.09, 0.18)	0.05	0.08	80	144	−0.60	0.24 (0.01, 0.47)	0.02	0.04
Haiti	HAITI (41)												
Kenya	WASH−B (42)	704	2460	−0.36	0.10 (0.00, 0.19)	0.11	0.13	755	2721	−0.54	0.13 (0.06, 0.21)	0.20	0.11
Madagascar	MAHAY (43)	344	420	−0.48	−0.08 (−0.28, 0.13)	0.02	0.04	1336	1238	−0.82	0.00 (−0.12, 0.12)	0.07	0.08
Malawi	iLiNS−DYADM (44)	35	69	0.39	−0.21 (−0.61, 0.19)	0.01	0.01	184	372	0.01	−0.01 (−0.18, 0.17)	0.03	0.06
Malawi	iLiNS−DOSE (45)	165	56	0.34	0.13 (−0.09, 0.35)	0.02	0.04	519	177	−0.07	0.02 (−0.11, 0.15)	0.06	0.08
Mali	PROMIS (46)	41	37	−0.59	−0.02 (−0.33, 0.30)	0.01	0.02	465	468	−0.79	0.21 (0.07, 0.34)	0.05	0.07
Mali	PROMIS CS (46)	107	96	−0.75	0.41 (0.17, 0.65)	0.02	0.03	846	874	−0.83	0.09 (−0.02, 0.21)	0.07	0.09
Zimbabwe	SHINE (HIV−) (47)	1709	1630	0.02	0.05 (−0.01, 0.11)	0.25	0.20	69	61	−0.05	0.15 (−0.19, 0.49)	0.01	0.02
Zimbabwe	SHINE (HIV+) (48)	301	292	−0.16	0.01 (−0.13, 0.15)	0.05	0.07	21	18	−0.38	0.27 (−0.35, 0.88)	0.00	0.01
		**6968**	**7084**		**I² = 0.12, Tau² = 0.00**			**8760**	**8691**		**I² = 0.66, Tau² = 0.01**		
**Fixed**					**0.07 (0.03, 0.10)**						**0.11 (0.08, 0.14)**		
**Random**					**0.07 (0.02, 0.11)**						**0.10 (0.04, 0.15)**		

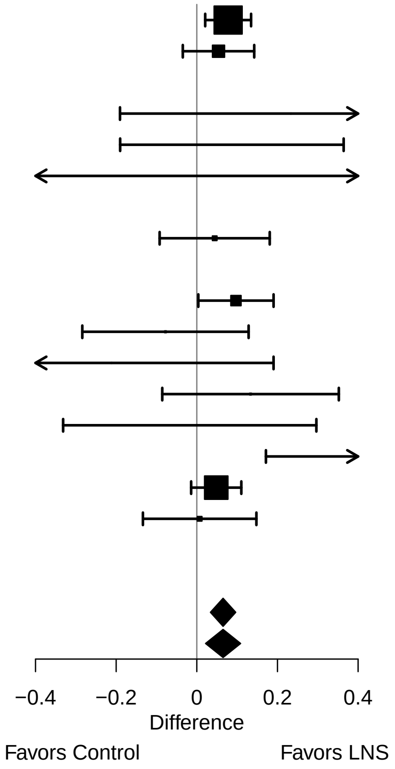

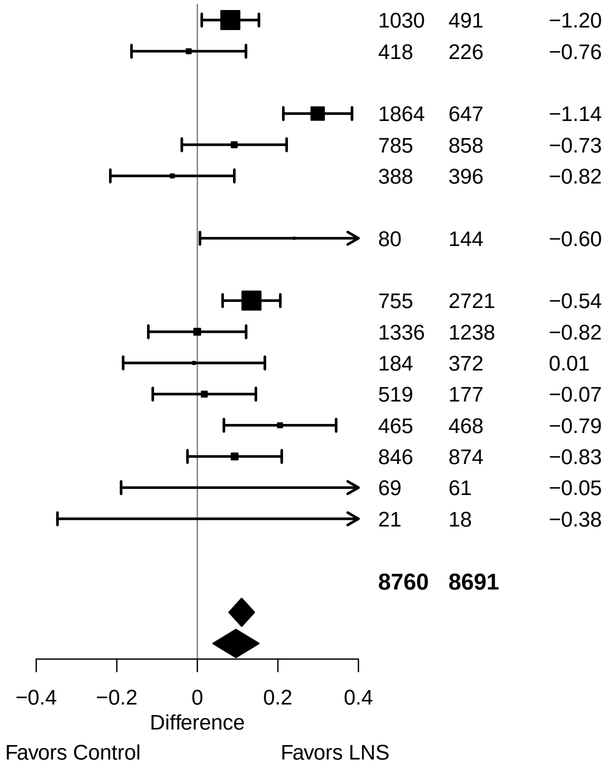

Supplemental figure 8G: Mean difference in MUACZ

8G5: Stratified by Maternal depressive symptoms

P−for−interaction = **0.348**
Difference in MDs = **−0.03 (−0.09, 0.03)**

		Less than 75th percentile						At least 75th percentile					
**Country**	**Trial**	**LNS N**	**Control N**	**Control Mean**	**MD (95% CI)**	**Fixed W**	**Random W**	**LNS N**	**Control N**	**Control Mean**	**MD (95% CI)**	**Fixed W**	**Random W**
Bangladesh	JiVitA−4 (34)												
Bangladesh	RDNS (35)	1081	472	−0.68	0.03 (−0.07, 0.14)	0.13	0.13	520	288	−0.72	0.01 (−0.10, 0.13)	0.21	0.14
Bangladesh	WASH−B (36)												
Burkina Faso	iLiNS−Zinc (37)												
Burkina Faso	PROMIS (38)	500	573	−0.76	0.11 (−0.01, 0.24)	0.09	0.09	224	215	−0.77	0.07 (−0.10, 0.23)	0.11	0.12
Burkina Faso	PROMIS CS (38)												
Ghana	GHANA (39)												
Ghana	iLiNS−DYADG (40)	252	460	−0.43	0.08 (−0.06, 0.22)	0.07	0.07	83	209	−0.56	0.12 (−0.11, 0.36)	0.05	0.09
Haiti	HAITI (41)												
Kenya	WASH−B (42)	999	3523	−0.43	0.10 (0.03, 0.18)	0.27	0.27	340	1249	−0.49	0.11 (−0.02, 0.23)	0.20	0.14
Madagascar	MAHAY (43)	656	707	−0.74	0.00 (−0.16, 0.17)	0.05	0.05	282	245	−0.63	−0.17 (−0.35, 0.01)	0.09	0.12
Malawi	iLiNS−DYADM (44)	157	303	0.13	−0.08 (−0.27, 0.11)	0.04	0.04	47	113	−0.05	0.17 (−0.18, 0.51)	0.02	0.06
Malawi	iLiNS−DOSE (45)												
Mali	PROMIS (46)	379	373	−0.72	0.11 (−0.04, 0.26)	0.07	0.07	124	131	−0.96	0.42 (0.20, 0.64)	0.06	0.10
Mali	PROMIS CS (46)												
Zimbabwe	SHINE (HIV−) (47)	1194	1135	0.03	0.03 (−0.05, 0.11)	0.24	0.24	509	487	−0.07	0.19 (0.07, 0.30)	0.22	0.15
Zimbabwe	SHINE (HIV+) (48)	234	215	−0.20	0.04 (−0.14, 0.21)	0.05	0.05	89	99	−0.17	0.02 (−0.26, 0.30)	0.04	0.08
		**5452**	**7761**		**I^2 = 0.00, Tau^2 = 0.00**			**2218**	**3036**		**I^2 = 0.63, Tau^2 = 0.02**		
**Fixed**					**0.06 (0.02, 0.10)**						**0.09 (0.04, 0.15)**		
**Random**					**0.06 (0.02, 0.10)**						**0.10 (−0.01, 0.20)**		

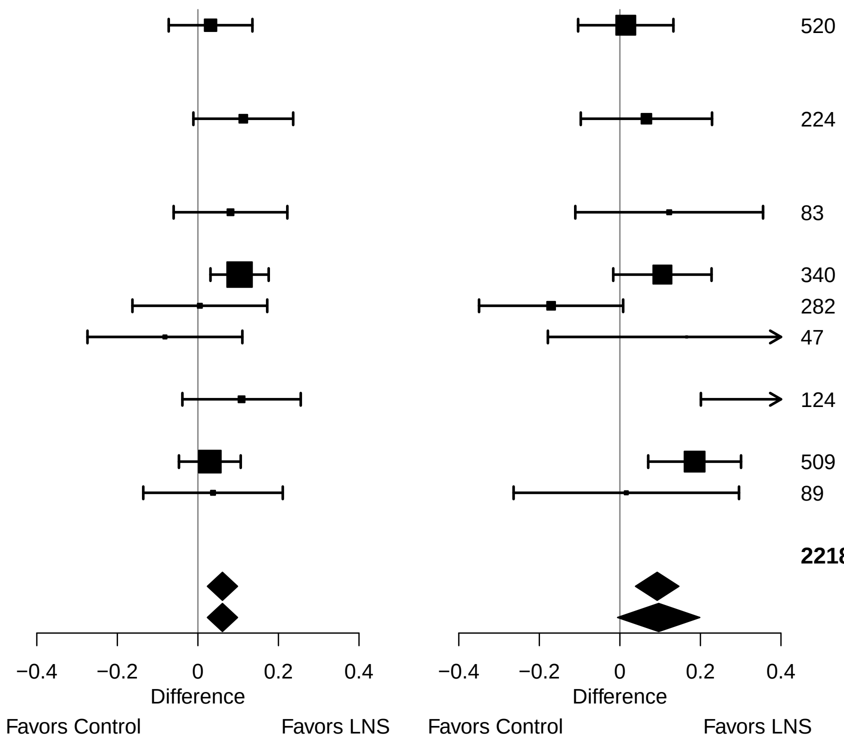

Supplemental figure 8G: Mean difference in MUACZ

8G6: Stratified by Child sex

P−for−interaction = 0.131
Difference in MDs = 0.03 (−0.01, 0.07)

		Male						Female					
Country	Trial	LNS N	Control N	Control Mean	MD (95% CI)	Fixed W	Random W	LNS N	Control N	Control Mean	MD (95% CI)	Fixed W	Random W
Bangladesh	JiVitA−4 (34)	1477	612	−1.02	0.05 (−0.01, 0.12)	0.25	0.11	1420	659	−1.10	0.11 (0.05, 0.17)	0.25	0.13
Bangladesh	RDNS (35)	833	411	−0.66	−0.03 (−0.13, 0.07)	0.10	0.09	830	404	−0.75	0.10 (−0.01, 0.21)	0.08	0.09
Bangladesh	WASH−B (36)												
Burkina Faso	iLiNS−Zinc (37)	995	336	−1.24	0.33 (0.22, 0.45)	0.08	0.08	964	330	−1.02	0.26 (0.14, 0.37)	0.07	0.08
Burkina Faso	PROMIS (38)	432	482	−0.80	0.11 (−0.03, 0.25)	0.05	0.07	422	430	−0.66	0.06 (−0.09, 0.22)	0.04	0.06
Burkina Faso	PROMIS CS (38)	229	219	−0.84	−0.08 (−0.25, 0.09)	0.03	0.06	195	217	−0.79	−0.03 (−0.25, 0.19)	0.02	0.03
Ghana	GHANA (39)												
Ghana	iLiNS−DYADG (40)	175	324	−0.55	0.09 (−0.08, 0.26)	0.03	0.06	172	369	−0.40	0.09 (−0.07, 0.25)	0.03	0.05
Haiti	HAITI (41)												
Kenya	WASH−B (42)	736	2467	−0.47	0.08 (−0.02, 0.18)	0.11	0.09	725	2718	−0.44	0.16 (0.08, 0.23)	0.16	0.12
Madagascar	MAHAY (43)	817	817	−0.80	−0.07 (−0.21, 0.07)	0.05	0.07	863	841	−0.67	0.00 (−0.13, 0.14)	0.05	0.07
Malawi	iLiNS−DYADM (44)	107	207	0.00	0.07 (−0.16, 0.30)	0.02	0.04	113	237	0.14	−0.13 (−0.36, 0.10)	0.02	0.03
Malawi	iLiNS−DOSE (45)	351	123	−0.12	0.02 (−0.14, 0.18)	0.04	0.06	345	117	0.16	0.11 (−0.05, 0.26)	0.04	0.06
Mali	PROMIS (46)	267	263	−0.80	0.16 (0.00, 0.33)	0.04	0.06	240	242	−0.75	0.21 (0.06, 0.37)	0.04	0.06
Mali	PROMIS CS (46)	479	526	−0.90	0.11 (−0.06, 0.29)	0.03	0.06	475	444	−0.74	0.13 (0.01, 0.26)	0.06	0.07
Zimbabwe	SHINE (HIV−) (47)	928	900	−0.05	0.09 (0.01, 0.18)	0.13	0.10	944	880	0.07	0.03 (−0.06, 0.11)	0.12	0.10
Zimbabwe	SHINE (HIV+) (48)	162	167	−0.25	−0.01 (−0.19, 0.17)	0.03	0.06	175	161	−0.13	0.07 (−0.11, 0.25)	0.03	0.05
		**7988**	**7854**		**I² = 0.62, Tau² = 0.01**			**7883**	**8049**		**I² = 0.41, Tau² = 0.00**		
**Fixed**					**0.07 (0.04, 0.10)**						**0.11 (0.08, 0.14)**		
**Random**					**0.07 (0.01, 0.13)**						**0.10 (0.05, 0.14)**		

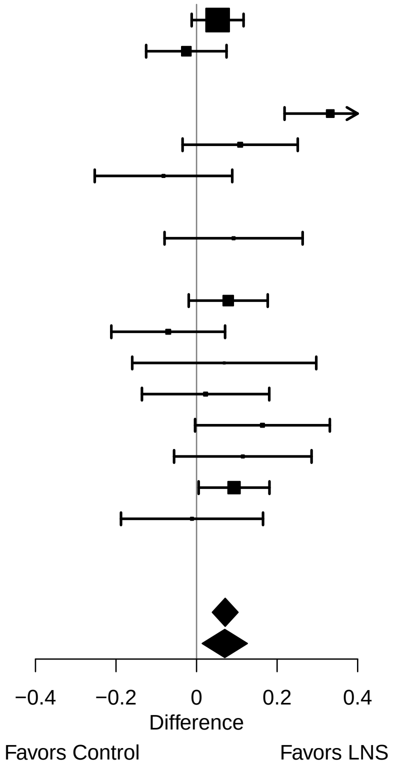

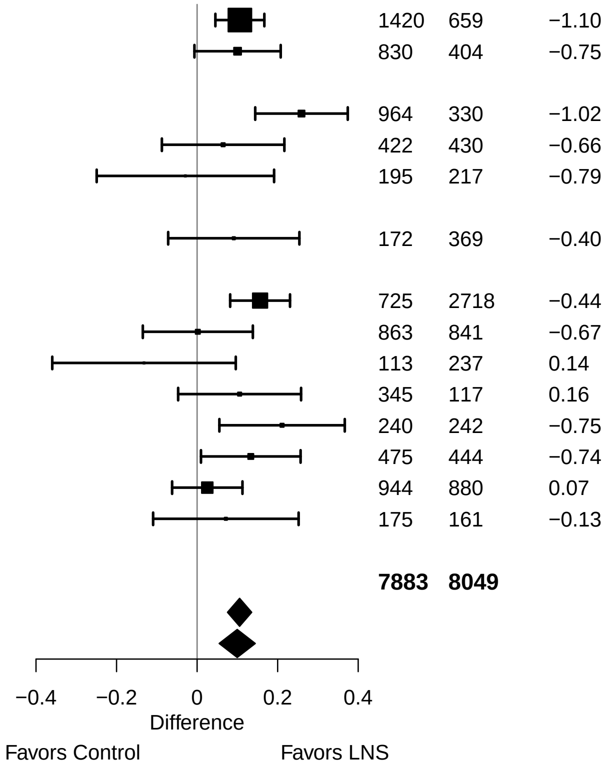

Supplemental figure 8G: Mean difference in MUACZ

8G7: Stratified by Child birth order

P−for−interaction = 0.004
Difference in MDs = −0.07 (−0.11, −0.02)

		Later born						Firstborn					
Country	Trial	LNS N	Control N	Control Mean	MD (95% CI)	Fixed W	Random W	LNS N	Control N	Control Mean	MD (95% CI)	Fixed W	Random W
Bangladesh	JiVitA−4 (34)	617	260	−1.16	0.18 (0.09, 0.27)	0.11	0.09	2276	1004	−1.04	0.05 (0.00, 0.10)	0.46	0.13
Bangladesh	RDNS (35)	974	508	−0.73	0.04 (−0.05, 0.14)	0.09	0.08	688	307	−0.66	0.02 (−0.08, 0.12)	0.11	0.11
Bangladesh	WASH−B (36)												
Burkina Faso	iLiNS−Zinc (37)	1524	521	−1.13	0.31 (0.21, 0.40)	0.10	0.08	435	145	−1.16	0.27 (0.10, 0.43)	0.04	0.08
Burkina Faso	PROMIS (38)	709	753	−0.74	0.11 (−0.03, 0.25)	0.04	0.06	145	158	−0.69	0.00 (−0.20, 0.20)	0.03	0.06
Burkina Faso	PROMIS CS (38)	362	356	−0.84	−0.03 (−0.20, 0.14)	0.03	0.05	61	80	−0.70	−0.20 (−0.48, 0.07)	0.02	0.04
Ghana	GHANA (39)												
Ghana	iLiNS−DYADG (40)	237	459	−0.45	0.03 (−0.11, 0.18)	0.04	0.06	110	234	−0.51	0.20 (0.00, 0.40)	0.03	0.06
Haiti	HAITI (41)												
Kenya	WASH−B (42)	1137	4083	−0.47	0.12 (0.04, 0.20)	0.15	0.09	322	1097	−0.39	0.10 (−0.02, 0.22)	0.08	0.10
Madagascar	MAHAY (43)	1263	1199	−0.78	−0.01 (−0.13, 0.11)	0.06	0.07	417	459	−0.63	−0.09 (−0.28, 0.10)	0.03	0.07
Malawi	iLiNS−DYADM (44)	173	353	0.10	−0.03 (−0.21, 0.15)	0.03	0.05	47	89	−0.06	−0.06 (−0.43, 0.31)	0.01	0.03
Malawi	iLiNS−DOSE (45)	459	164	0.00	0.06 (−0.07, 0.19)	0.05	0.07	148	45	0.24	−0.07 (−0.33, 0.19)	0.02	0.05
Mali	PROMIS (46)	443	444	−0.77	0.20 (0.06, 0.34)	0.04	0.06	63	59	−0.84	0.12 (−0.09, 0.32)	0.03	0.06
Mali	PROMIS CS (46)	791	832	−0.82	0.12 (0.01, 0.24)	0.06	0.07	146	126	−0.86	0.23 (0.03, 0.42)	0.03	0.07
Zimbabwe	SHINE (HIV−) (47)	1296	1211	−0.02	0.11 (0.04, 0.18)	0.16	0.10	489	486	0.07	−0.06 (−0.17, 0.05)	0.10	0.10
Zimbabwe	SHINE (HIV+) (48)	280	245	−0.15	−0.03 (−0.19, 0.13)	0.03	0.06	54	78	−0.29	0.27 (−0.03, 0.56)	0.01	0.04
		**10265**	**11388**		**I² = 0.63, Tau² = 0.01**			**5401**	**4367**		**I² = 0.50, Tau² = 0.01**		
**Fixed**					**0.11 (0.08, 0.14)**						**0.05 (0.02, 0.09)**		
**Random**					**0.10 (0.05, 0.15)**						**0.06 (−0.01, 0.13)**		

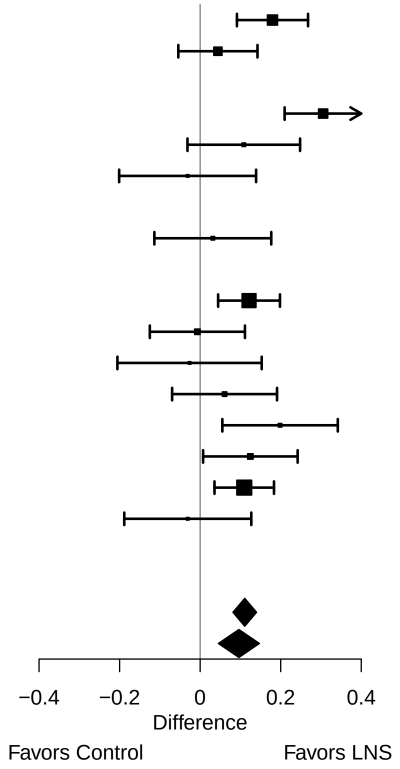

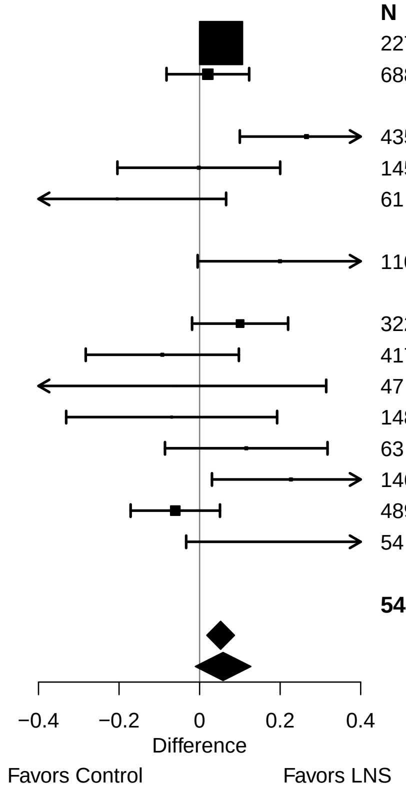

Supplemental figure 8G: Mean difference in MUACZ

8G8: Stratified by Child baseline anthropometric status

P−for−interaction = 0.177
Difference in MDs = 0.04 (−0.02, 0.10)

		MUACZ at least 0 Z							MUACZ less than 0 Z					
Country	Trial	LNS N	Control N	Control Mean	MD (95% CI)	Fixed W	Random W	LNS N	Control N	Control Mean	MD (95% CI)	Fixed W	Random W
Bangladesh	JiVitA−4 (34)	696	295	−0.26	0.08 (−0.02, 0.19)	0.17	0.17	2201	976	−1.31	0.08 (0.02, 0.14)	0.34	0.14
Bangladesh	RDNS (35)	702	321	−0.14	−0.02 (−0.10, 0.06)	0.28	0.28	899	439	−1.10	0.04 (−0.05, 0.12)	0.18	0.13
Bangladesh	WASH−B (36)												
Burkina Faso	iLiNS−Zinc (37)	349	128	−0.05	0.24 (0.04, 0.44)	0.05	0.05	1610	538	−1.39	0.36 (0.24, 0.48)	0.10	0.12
Burkina Faso	PROMIS (38)												
Burkina Faso	PROMIS CS (38)												
Ghana	GHANA (39)												
Ghana	iLiNS−DYADG (40)	149	290	0.10	0.08 (−0.08, 0.24)	0.07	0.07	174	352	−0.92	0.06 (−0.07, 0.20)	0.07	0.11
Haiti	HAITI (41)												
Kenya	WASH−B (42)												
Madagascar	MAHAY (43)												
Malawi	iLiNS−DYADM (44)	111	233	0.54	−0.01 (−0.19, 0.17)	0.06	0.06	93	193	−0.50	−0.07 (−0.29, 0.16)	0.03	0.08
Malawi	iLiNS−DOSE (45)	397	141	0.46	0.01 (−0.16, 0.17)	0.07	0.07	299	99	−0.61	0.04 (−0.15, 0.23)	0.04	0.09
Mali	PROMIS (46)	185	156	−0.18	0.08 (−0.10, 0.25)	0.06	0.06	322	349	−1.05	0.20 (0.04, 0.37)	0.05	0.10
Mali	PROMIS CS (46)												
Zimbabwe	SHINE (HIV−) (47)	678	616	0.40	0.05 (−0.05, 0.14)	0.21	0.21	640	578	−0.46	0.14 (0.05, 0.23)	0.17	0.13
Zimbabwe	SHINE (HIV+) (48)	123	96	0.29	0.10 (−0.12, 0.31)	0.04	0.04	144	139	−0.45	−0.08 (−0.26, 0.11)	0.04	0.09
		**3390**	**2276**		**I² = 0.00, Tau² = 0.00**			**6382**	**3663**		**I² = 0.73, Tau² = 0.01**		
**Fixed**					**0.04 (0.00, 0.09)**						**0.10 (0.07, 0.14)**		
**Random**					**0.04 (0.00, 0.09)**						**0.10 (0.01, 0.18)**		

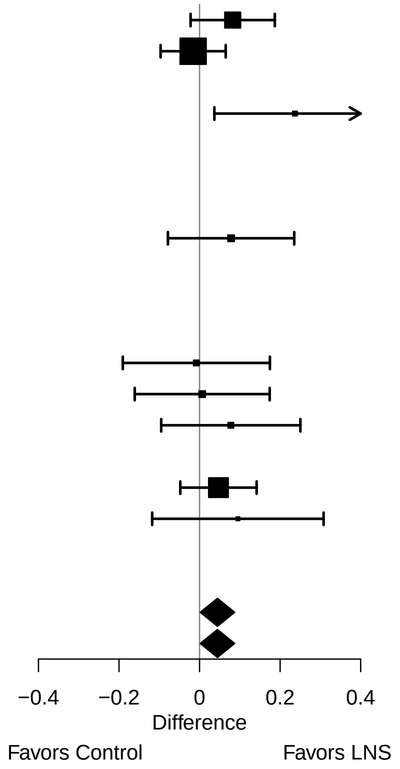

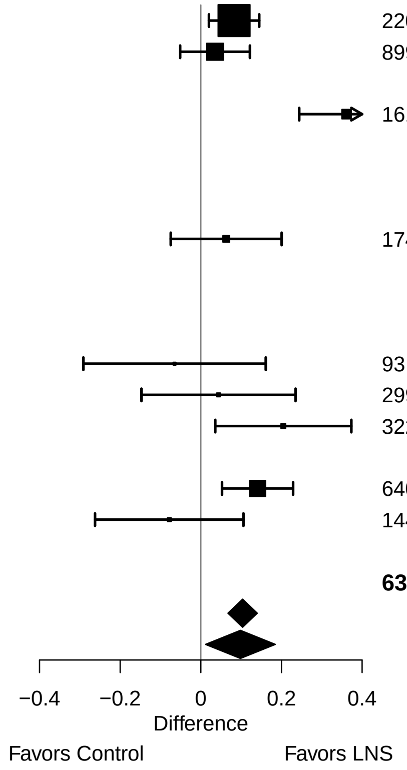

Supplemental figure 8H: Low MUAC prevalence ratio

8H1: Stratified by Maternal height (insufficient comparisons)

Supplemental figure 8H: Low MUAC prevalence ratio

8H2: Stratified by Maternal BMI

P−for−interaction = 0.337
Ratio of PRs = 1.12 (0.89, 1.40)

Country	Trial	At least 20 kg/m²: LNS N	Control N	Control Prevalence	PR (95% CI)	Fixed W	Random W	Less than 20 kg/m²: LNS N	Control N	Control Prevalence	PR (95% CI)	Fixed W	Random W
Bangladesh	JiVitA−4 (34)												
Bangladesh	RDNS (35)	730	335	3.0	0.78 (0.39, 1.54)	0.05	0.07	873	455	7.0	1.11 (0.72, 1.71)	0.14	0.14
Bangladesh	WASH−B (36)												
Burkina Faso	iLiNS−Zinc (37)	1218	391	14.6	0.55 (0.42, 0.74)	0.30	0.24	739	275	23.6	0.75 (0.57, 0.99)	0.36	0.36
Burkina Faso	PROMIS (38)	641	673	7.1	0.92 (0.56, 1.50)	0.10	0.12	210	233	10.3	1.02 (0.62, 1.67)	0.11	0.11
Burkina Faso	PROMIS CS (38)	241	266	12.0	0.93 (0.54, 1.60)	0.08	0.10	183	166	19.3	0.88 (0.58, 1.32)	0.16	0.16
Ghana	GHANA (39)												
Ghana	iLiNS−DYADG (40)												
Haiti	HAITI (41)												
Kenya	WASH−B (42)	1101	3837	4.4	0.89 (0.62, 1.29)	0.18	0.18	272	1086	8.5	0.48 (0.25, 0.92)	0.06	0.06
Madagascar	MAHAY (43)												
Malawi	iLiNS−DYADM (44)												
Malawi	iLiNS−DOSE (45)												
Mali	PROMIS (46)	381	369	5.7	0.47 (0.23, 0.97)	0.05	0.07	120	135	5.9	0.95 (0.39, 2.35)	0.03	0.03
Mali	PROMIS CS (46)	703	685	10.1	0.95 (0.69, 1.30)	0.24	0.21	246	279	13.6	0.84 (0.53, 1.32)	0.13	0.13
Zimbabwe	SHINE (HIV−) (47)												
Zimbabwe	SHINE (HIV+) (48)												
		**5015**	**6556**		**I² = 0.40, Tau² = 0.02**			**2643**	**2629**		**I² = 0.00, Tau² = 0.00**		
**Fixed**					**0.76 (0.65, 0.89)**						**0.84 (0.71, 0.99)**		
**Random**					**0.77 (0.63, 0.94)**						**0.84 (0.71, 0.99)**		

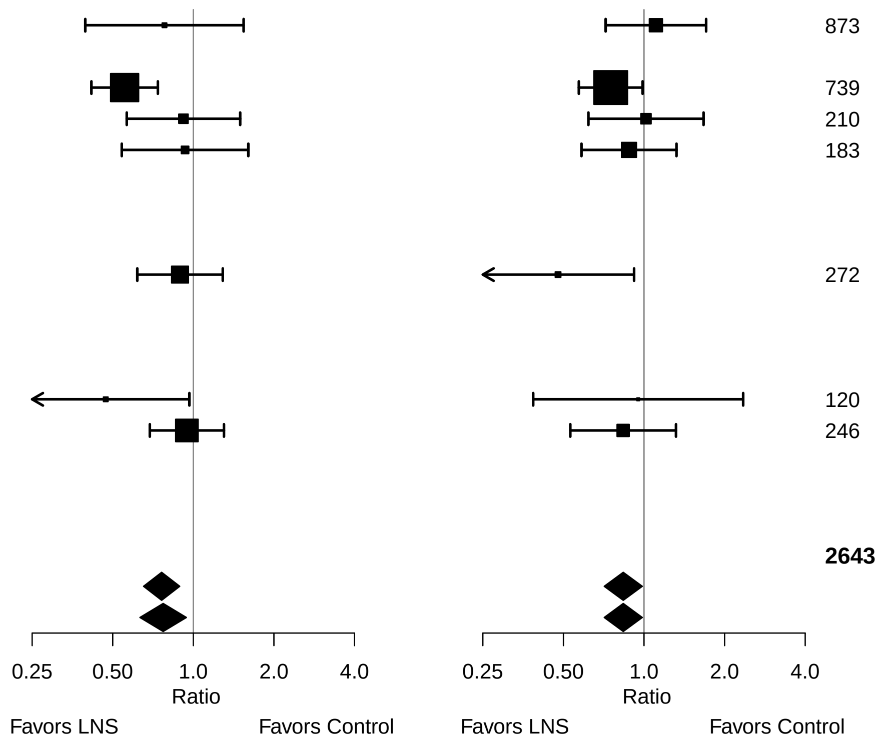

Supplemental figure 8H: Low MUAC prevalence ratio

8H3: Stratified by Maternal age

P−for−interaction = 0.741
Ratio of PRs = 1.03 (0.87, 1.22)

		At least 25 y						Less than 25 y					
**Country**	**Trial**	**LNS N**	**Control N**	**Control Prevalence**	**PR (95% CI)**	**Fixed W**	**Random W**	**LNS N**	**Control N**	**Control Prevalence**	**PR (95% CI)**	**Fixed W**	**Random W**
Bangladesh	JiVitA−4 (34)	1244	555	15.7	0.82 (0.63, 1.07)	0.23	0.15	1640	711	14.7	0.84 (0.67, 1.05)	0.30	0.30
Bangladesh	RDNS (35)	453	221	5.0	1.24 (0.66, 2.34)	0.04	0.07	1210	594	5.2	0.93 (0.61, 1.44)	0.08	0.08
Bangladesh	WASH−B (36)												
Burkina Faso	iLiNS−Zinc (37)	1134	401	18.5	0.59 (0.44, 0.81)	0.17	0.14	812	262	17.9	0.69 (0.51, 0.94)	0.15	0.15
Burkina Faso	PROMIS (38)	481	525	8.8	0.78 (0.51, 1.21)	0.08	0.11	373	387	7.0	1.19 (0.65, 2.20)	0.04	0.04
Burkina Faso	PROMIS CS (38)	200	190	15.8	0.95 (0.59, 1.54)	0.07	0.10	224	246	14.2	0.88 (0.55, 1.41)	0.06	0.06
Ghana	GHANA (39)												
Ghana	iLiNS−DYADG (40)												
Haiti	HAITI (41)												
Kenya	WASH−B (42)	845	2838	5.0	0.69 (0.46, 1.02)	0.10	0.12	601	2315	6.1	0.71 (0.47, 1.08)	0.08	0.08
Madagascar	MAHAY (43)	887	933	10.1	1.07 (0.80, 1.45)	0.18	0.14	792	725	9.1	0.86 (0.62, 1.19)	0.14	0.14
Malawi	iLiNS−DYADM (44)												
Malawi	iLiNS−DOSE (45)												
Mali	PROMIS (46)	347	358	5.8	0.41 (0.17, 0.99)	0.02	0.04	160	147	5.4	0.89 (0.38, 2.09)	0.02	0.02
Mali	PROMIS CS (46)	503	508	10.6	0.86 (0.56, 1.32)	0.09	0.11	451	462	11.5	0.95 (0.65, 1.38)	0.10	0.10
Zimbabwe	SHINE (HIV−) (47)	870	794	2.0	0.29 (0.11, 0.77)	0.02	0.04	805	790	1.0	0.86 (0.33, 2.24)	0.02	0.02
Zimbabwe	SHINE (HIV+) (48)												
		**6964**	**7323**		**I^2 = 0.46, Tau^2 = 0.06**			**7068**	**6639**		**I^2 = 0.00, Tau^2 = 0.00**		
**Fixed**					**0.80 (0.70, 0.91)**						**0.84 (0.74, 0.95)**		
**Random**					**0.78 (0.63, 0.96)**						**0.84 (0.74, 0.95)**		

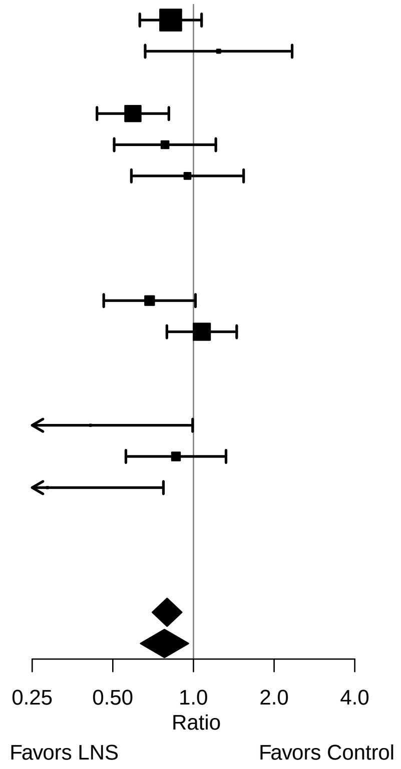

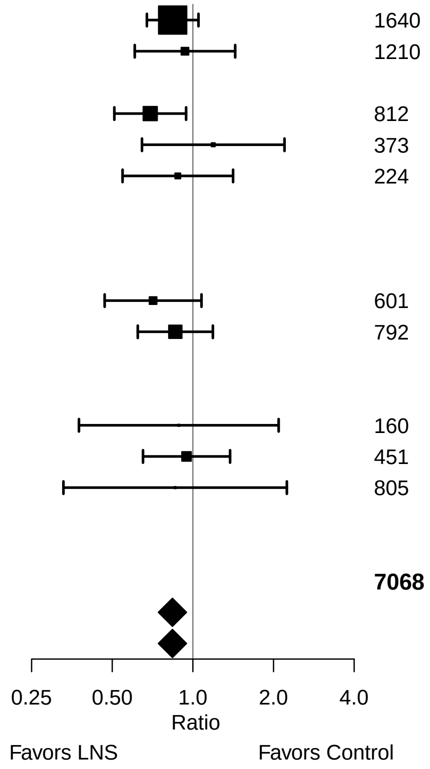

Supplemental figure 8H: Low MUAC prevalence ratio

8H4: Stratified by Maternal education

P−for−interaction = 0.240
Ratio of PRs = 1.16 (0.91, 1.48)

		Primary or greater						Incomplete or no formal					
**Country**	**Trial**	**LNS N**	**Control N**	**Control Prevalence**	**PR (95% CI)**	**Fixed W**	**Random W**	**LNS N**	**Control N**	**Control Prevalence**	**PR (95% CI)**	**Fixed W**	**Random W**
Bangladesh	JiVitA−4 (34)	1865	777	14.5	0.72 (0.59, 0.88)	0.64	0.42	1030	491	16.2	0.98 (0.75, 1.27)	0.23	0.23
Bangladesh	RDNS (35)	1245	589	4.1	1.18 (0.75, 1.86)	0.13	0.19	418	226	8.0	0.81 (0.46, 1.44)	0.05	0.05
Bangladesh	WASH−B (36)												
Burkina Faso	iLiNS−Zinc (37)												
Burkina Faso	PROMIS (38)												
Burkina Faso	PROMIS CS (38)	34	39	15.4	0.96 (0.31, 2.92)	0.02	0.04	388	396	14.9	0.92 (0.65, 1.29)	0.14	0.14
Ghana	GHANA (39)												
Ghana	iLiNS−DYADG (40)												
Haiti	HAITI (41)												
Kenya	WASH−B (42)	704	2460	4.2	0.57 (0.32, 1.01)	0.08	0.13	755	2721	6.6	0.80 (0.57, 1.12)	0.15	0.15
Madagascar	MAHAY (43)	344	420	5.7	1.07 (0.63, 1.81)	0.10	0.15	1336	1238	11.0	0.93 (0.71, 1.22)	0.22	0.22
Malawi	iLiNS−DYADM (44)												
Malawi	iLiNS−DOSE (45)												
Mali	PROMIS (46)												
Mali	PROMIS CS (46)	107	96	10.4	0.54 (0.22, 1.33)	0.03	0.06	846	874	11.1	0.95 (0.72, 1.25)	0.21	0.21
Zimbabwe	SHINE (HIV−) (47)												
Zimbabwe	SHINE (HIV+) (48)												
		**4299**	**4381**		**I² = 0.30, Tau² = 0.02**			**4773**	**5946**		**I² = 0.00, Tau² = 0.00**		
**Fixed**					**0.78 (0.66, 0.92)**						**0.92 (0.81, 1.04)**		
**Random**					**0.81 (0.64, 1.02)**						**0.92 (0.81, 1.04)**		

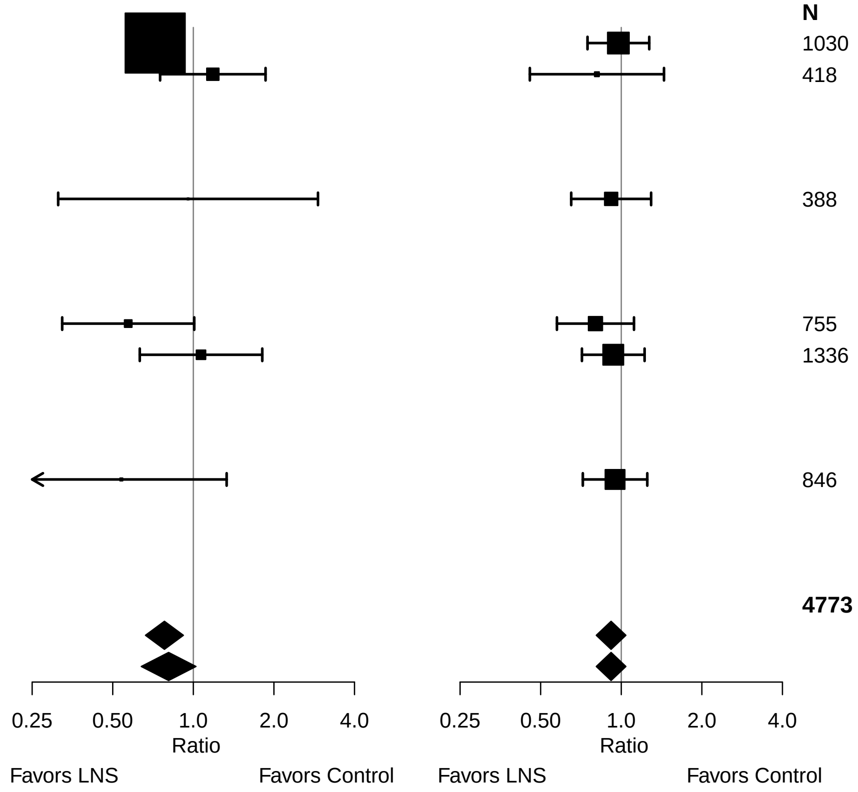

Supplemental figure 8H: Low MUAC prevalence ratio

8H5: Stratified by Maternal depressive symptoms

P−for−interaction = 0.798
Ratio of PRs = 1.04 (0.77, 1.40)

		Less than 75th percentile						At least 75th percentile					
**Country**	**Trial**	**LNS N**	**Control N**	**Control Prevalence**	**PR (95% CI)**	**Fixed W**	**Random W**	**LNS N**	**Control N**	**Control Prevalence**	**PR (95% CI)**	**Fixed W**	**Random W**
Bangladesh	JiVitA−4 (34)												
Bangladesh	RDNS (35)	1081	472	5.3	0.94 (0.61, 1.45)	0.18	0.19	520	288	4.5	1.28 (0.67, 2.44)	0.20	0.20
Bangladesh	WASH−B (36)												
Burkina Faso	iLiNS−Zinc (37)												
Burkina Faso	PROMIS (38)	500	573	7.9	0.99 (0.64, 1.54)	0.17	0.19	224	215	9.3	0.72 (0.37, 1.42)	0.18	0.18
Burkina Faso	PROMIS CS (38)												
Ghana	GHANA (39)												
Ghana	iLiNS−DYADG (40)												
Haiti	HAITI (41)												
Kenya	WASH−B (42)	999	3523	5.4	0.76 (0.57, 1.01)	0.40	0.36	340	1249	4.9	0.72 (0.37, 1.40)	0.19	0.19
Madagascar	MAHAY (43)	656	707	8.5	0.99 (0.66, 1.49)	0.20	0.21	282	245	10.6	0.87 (0.54, 1.40)	0.36	0.36
Malawi	iLiNS−DYADM (44)												
Malawi	iLiNS−DOSE (45)												
Mali	PROMIS (46)												
Mali	PROMIS CS (46)												
Zimbabwe	SHINE (HIV−) (47)	1194	1135	1.4	0.42 (0.18, 0.98)	0.05	0.06	509	487	1.8	0.53 (0.17, 1.62)	0.07	0.07
Zimbabwe	SHINE (HIV+) (48)												
		**4430**	**6410**		**I² = 0.12, Tau² = 0.01**			**1875**	**2484**		**I² = 0.00, Tau² = 0.00**		
**Fixed**					**0.85 (0.71, 1.02)**						**0.85 (0.64, 1.13)**		
**Random**					**0.85 (0.69, 1.04)**						**0.85 (0.64, 1.13)**		

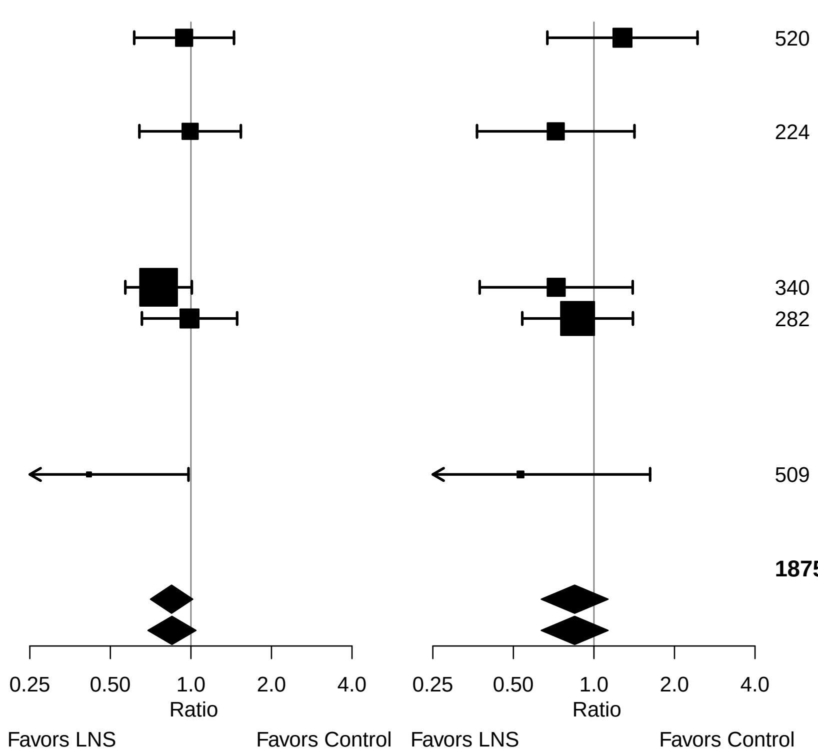

Supplemental figure 8H: Low MUAC prevalence ratio

8H6: Stratified by Child sex

P−for−interaction = 0.002
Ratio of PRs = 0.77 (0.66, 0.91)

		Male						Female					
**Country**	**Trial**	**LNS N**	**Control N**	**Control Prevalence**	**PR (95% CI)**	**Fixed W**	**Random W**	**LNS N**	**Control N**	**Control Prevalence**	**PR (95% CI)**	**Fixed W**	**Random W**
Bangladesh	JiVitA−4 (34)	1477	612	12.1	1.00 (0.73, 1.36)	0.16	0.16	1420	659	18.1	0.76 (0.62, 0.94)	0.32	0.22
Bangladesh	RDNS (35)	833	411	4.1	1.45 (0.87, 2.42)	0.06	0.06	830	404	6.2	0.72 (0.43, 1.20)	0.05	0.07
Bangladesh	WASH−B (36)												
Burkina Faso	iLiNS−Zinc (37)	995	336	17.9	0.70 (0.52, 0.95)	0.17	0.17	964	330	18.8	0.57 (0.43, 0.76)	0.17	0.16
Burkina Faso	PROMIS (38)	432	482	8.1	1.00 (0.58, 1.73)	0.05	0.05	422	430	7.9	0.87 (0.52, 1.44)	0.05	0.07
Burkina Faso	PROMIS CS (38)	229	219	10.5	1.12 (0.71, 1.77)	0.07	0.07	195	217	19.4	0.82 (0.52, 1.30)	0.07	0.08
Ghana	GHANA (39)												
Ghana	iLiNS−DYADG (40)	175	324	4.3	0.66 (0.24, 1.81)	0.02	0.02	172	369	3.3	0.89 (0.32, 2.50)	0.01	0.02
Haiti	HAITI (41)												
Kenya	WASH−B (42)	736	2467	5.9	0.92 (0.66, 1.28)	0.14	0.14	725	2718	5.1	0.46 (0.29, 0.72)	0.07	0.09
Madagascar	MAHAY (43)	817	817	10.5	0.99 (0.73, 1.34)	0.17	0.17	863	841	8.8	0.96 (0.70, 1.31)	0.14	0.14
Malawi	iLiNS−DYADM (44)												
Malawi	iLiNS−DOSE (45)												
Mali	PROMIS (46)	267	263	5.3	0.79 (0.40, 1.59)	0.03	0.03	240	242	6.2	0.37 (0.12, 1.14)	0.01	0.02
Mali	PROMIS CS (46)	479	526	11.0	0.95 (0.63, 1.42)	0.10	0.10	475	444	11.0	0.86 (0.58, 1.26)	0.09	0.11
Zimbabwe	SHINE (HIV−) (47)	928	900	1.6	0.55 (0.24, 1.30)	0.02	0.02	944	880	1.4	0.47 (0.18, 1.21)	0.02	0.02
Zimbabwe	SHINE (HIV+) (48)												
		**7368**	**7357**		**I² = 0.00, Tau² = 0.00**			**7250**	**7534**		**I² = 0.28, Tau² = 0.02**		
**Fixed**					**0.93 (0.82, 1.05)**						**0.73 (0.65, 0.82)**		
**Random**					**0.93 (0.82, 1.05)**						**0.72 (0.62, 0.84)**		

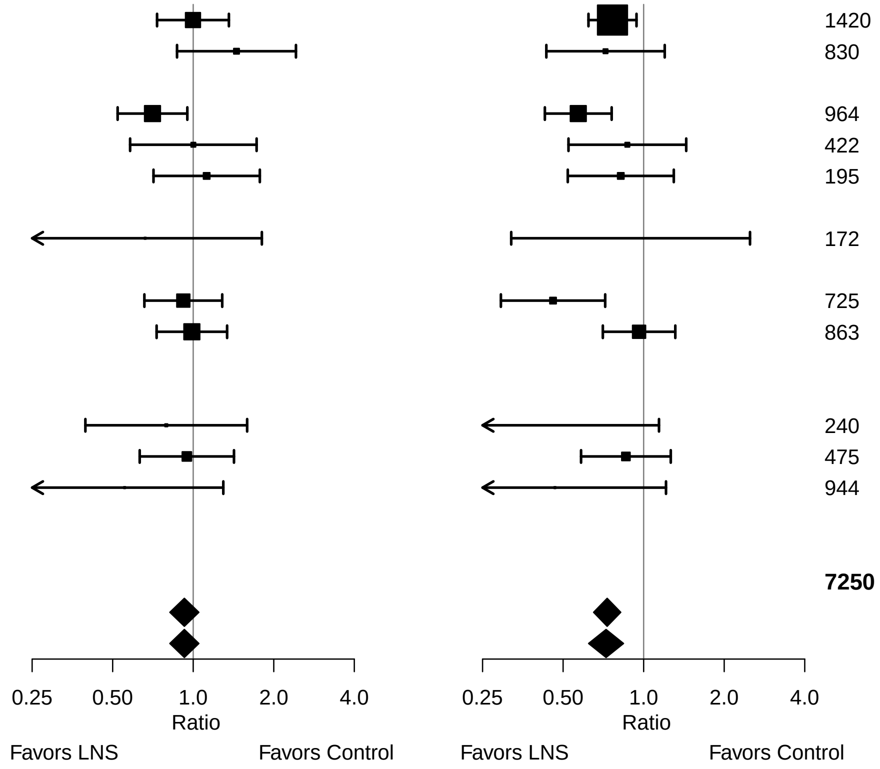

Supplemental figure 8H: Low MUAC prevalence ratio

8H7: Stratified by Child birth order

P−for−interaction = 0.002
Ratio of PRs = 1.37 (1.12, 1.67)

		Later born						Firstborn					
**Country**	**Trial**	**LNS N**	**Control N**	**Control Prevalence**	**PR (95% CI)**	**Fixed W**	**Random W**	**LNS N**	**Control N**	**Control Prevalence**	**PR (95% CI)**	**Fixed W**	**Random W**
Bangladesh	JiVitA−4 (34)	617	260	17.0	0.58 (0.42, 0.80)	0.11	0.12	2276	1004	14.6	0.94 (0.76, 1.17)	0.53	0.32
Bangladesh	RDNS (35)	974	508	6.9	0.82 (0.54, 1.25)	0.07	0.08	688	307	2.3	2.04 (0.83, 5.04)	0.03	0.05
Bangladesh	WASH−B (36)												
Burkina Faso	iLiNS−Zinc (37)	1524	521	18.2	0.60 (0.46, 0.76)	0.19	0.17	435	145	18.6	0.78 (0.53, 1.13)	0.17	0.20
Burkina Faso	PROMIS (38)	709	753	8.6	0.87 (0.58, 1.29)	0.07	0.09	145	158	5.1	1.50 (0.62, 3.61)	0.03	0.06
Burkina Faso	PROMIS CS (38)	362	356	16.0	0.81 (0.57, 1.16)	0.09	0.11	61	80	10.0	1.80 (0.82, 3.98)	0.04	0.07
Ghana	GHANA (39)												
Ghana	iLiNS−DYADG (40)												
Haiti	HAITI (41)												
Kenya	WASH−B (42)	1137	4083	5.5	0.72 (0.52, 0.99)	0.12	0.12	322	1097	5.4	0.69 (0.37, 1.30)	0.06	0.10
Madagascar	MAHAY (43)	1263	1199	10.4	0.96 (0.75, 1.22)	0.20	0.17	417	459	7.6	1.01 (0.60, 1.68)	0.09	0.13
Malawi	iLiNS−DYADM (44)												
Malawi	iLiNS−DOSE (45)												
Mali	PROMIS (46)												
Mali	PROMIS CS (46)	791	832	10.8	0.91 (0.68, 1.22)	0.14	0.14	146	126	10.3	0.80 (0.37, 1.72)	0.04	0.07
Zimbabwe	SHINE (HIV−) (47)												
Zimbabwe	SHINE (HIV+) (48)												
		**7377**	**8512**		**I² = 0.42, Tau² = 0.01**			**4490**	**3376**		**I² = 0.19, Tau² = 0.03**		
**Fixed**					**0.77 (0.69, 0.86)**						**0.95 (0.81, 1.11)**		
**Random**					**0.77 (0.67, 0.88)**						**0.98 (0.79, 1.22)**		

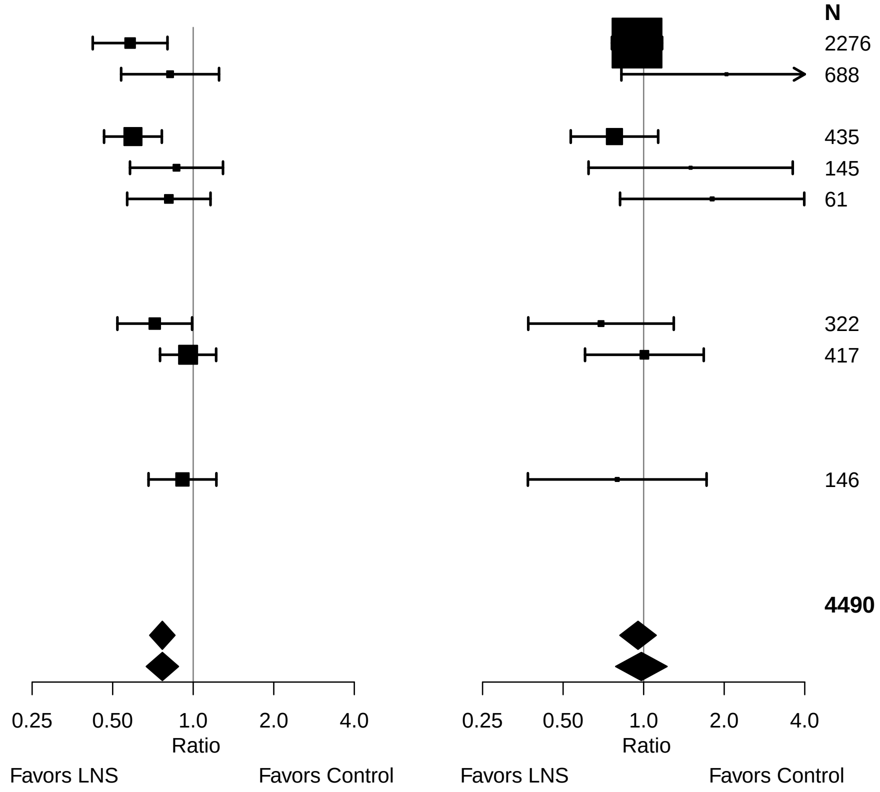

8H8: Stratified by Child baseline anthropometric status (insufficient comparisons)

Supplemental figure 8I: Low MUAC prevalence difference

8I1: Stratified by Maternal height (insufficient comparisons)

Supplemental figure 8I: Low MUAC prevalence difference

8I2: Stratified by Maternal BMI

P−for−interaction = 0.260
Difference in PDs = −0.01 (−0.03, 0.01)

Country	Trial	At least 20 kg/m² LNS N	Control N	Control Prevalence	PD (95% CI)	Fixed W	Random W	Less than 20 kg/m² LNS N	Control N	Control Prevalence	PD (95% CI)	Fixed W	Random W
Bangladesh	JiVitA−4 (34)												
Bangladesh	RDNS (35)	730	335	3.0	−0.01 (−0.03, 0.01)	0.24	0.20	873	455	7.0	0.01 (−0.02, 0.04)	0.26	0.21
Bangladesh	WASH−B (36)												
Burkina Faso	iLiNS−Zinc (37)	1218	391	14.6	−0.06 (−0.09, −0.03)	0.08	0.13	739	275	23.6	−0.08 (−0.12, −0.03)	0.12	0.14
Burkina Faso	PROMIS (38)	641	673	7.1	−0.01 (−0.04, 0.03)	0.08	0.12	210	233	10.3	0.00 (−0.05, 0.05)	0.10	0.12
Burkina Faso	PROMIS CS (38)	241	266	12.0	−0.01 (−0.07, 0.05)	0.02	0.05	183	166	19.3	−0.02 (−0.10, 0.05)	0.05	0.07
Ghana	GHANA (39)												
Ghana	iLiNS−DYADG (40)												
Haiti	HAITI (41)												
Kenya	WASH−B (42)	1101	3837	4.4	0.00 (−0.02, 0.01)	0.40	0.23	272	1086	8.5	−0.04 (−0.07, −0.02)	0.31	0.22
Madagascar	MAHAY (43)												
Malawi	iLiNS−DYADM (44)												
Malawi	iLiNS−DOSE (45)												
Mali	PROMIS (46)	381	369	5.7	−0.03 (−0.06, 0.00)	0.09	0.13	120	135	5.9	0.00 (−0.05, 0.05)	0.10	0.13
Mali	PROMIS CS (46)	703	685	10.1	−0.01 (−0.04, 0.03)	0.09	0.13	246	279	13.6	−0.02 (−0.08, 0.03)	0.08	0.11
Zimbabwe	SHINE (HIV−) (47)												
Zimbabwe	SHINE (HIV+) (48)												
		**5015**	**6556**		**I² = 0.49, Tau² = 0.00**			**2643**	**2629**		**I² = 0.51, Tau² = 0.00**		
**Fixed**					**−0.01 (−0.02, 0.00)**						**−0.02 (−0.04, −0.01)**		
**Random**					**−0.02 (−0.03, 0.00)**						**−0.02 (−0.05, 0.00)**		

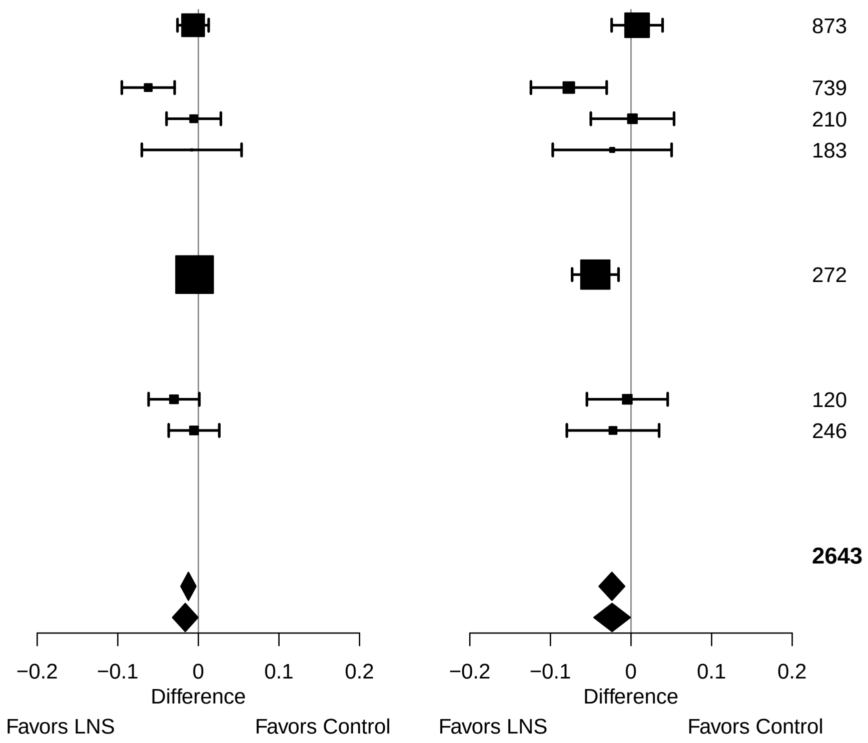

Supplemental figure 8I: Low MUAC prevalence difference

8I3: Stratified by Maternal age

P−for−interaction = 0.160
Difference in PDs = 0.01 (0.00, 0.02)

Country	Trial	At least 25 y: LNS N	Control N	Control Prevalence	PD (95% CI)	Fixed W	Random W	Less than 25 y: LNS N	Control N	Control Prevalence	PD (95% CI)	Fixed W	Random W
Bangladesh	JiVitA−4 (34)	1244	555	15.7	−0.03 (−0.06, 0.01)	0.05	0.09	1640	711	14.7	−0.02 (−0.04, 0.01)	0.06	0.06
Bangladesh	RDNS (35)	453	221	5.0	0.01 (−0.02, 0.05)	0.05	0.09	1210	594	5.2	0.00 (−0.03, 0.02)	0.10	0.10
Bangladesh	WASH−B (36)												
Burkina Faso	iLiNS−Zinc (37)	1134	401	18.5	−0.08 (−0.13, −0.03)	0.02	0.05	812	262	17.9	−0.06 (−0.11, −0.01)	0.02	0.02
Burkina Faso	PROMIS (38)	481	525	8.8	−0.02 (−0.05, 0.02)	0.04	0.08	373	387	7.0	0.01 (−0.03, 0.06)	0.02	0.02
Burkina Faso	PROMIS CS (38)	200	190	15.8	−0.01 (−0.08, 0.07)	0.01	0.02	224	246	14.2	−0.02 (−0.08, 0.05)	0.01	0.01
Ghana	GHANA (39)												
Ghana	iLiNS−DYADG (40)												
Haiti	HAITI (41)												
Kenya	WASH−B (42)	845	2838	5.0	−0.02 (−0.03, 0.00)	0.26	0.20	601	2315	6.1	−0.02 (−0.04, 0.00)	0.13	0.13
Madagascar	MAHAY (43)	887	933	10.1	0.01 (−0.02, 0.04)	0.06	0.10	792	725	9.1	−0.01 (−0.04, 0.01)	0.06	0.06
Malawi	iLiNS−DYADM (44)												
Malawi	iLiNS−DOSE (45)												
Mali	PROMIS (46)	347	358	5.8	−0.03 (−0.07, 0.00)	0.06	0.10	160	147	5.4	0.00 (−0.05, 0.04)	0.02	0.02
Mali	PROMIS CS (46)	503	508	10.6	−0.01 (−0.06, 0.03)	0.03	0.06	451	462	11.5	−0.01 (−0.05, 0.04)	0.03	0.03
Zimbabwe	SHINE (HIV−) (47)	870	794	2.0	−0.01 (−0.03, 0.00)	0.41	0.22	805	790	1.0	0.00 (−0.01, 0.01)	0.56	0.56
Zimbabwe	SHINE (HIV+) (48)												
		**6964**	**7323**		**I^2 = 0.28, Tau^2 = 0.00**			**7068**	**6639**		**I^2 = 0.00, Tau^2 = 0.00**		
**Fixed**					**−0.02 (−0.02, −0.01)**						**−0.01 (−0.01, 0.00)**		
**Random**					**−0.02 (−0.03, 0.00)**						**−0.01 (−0.01, 0.00)**		

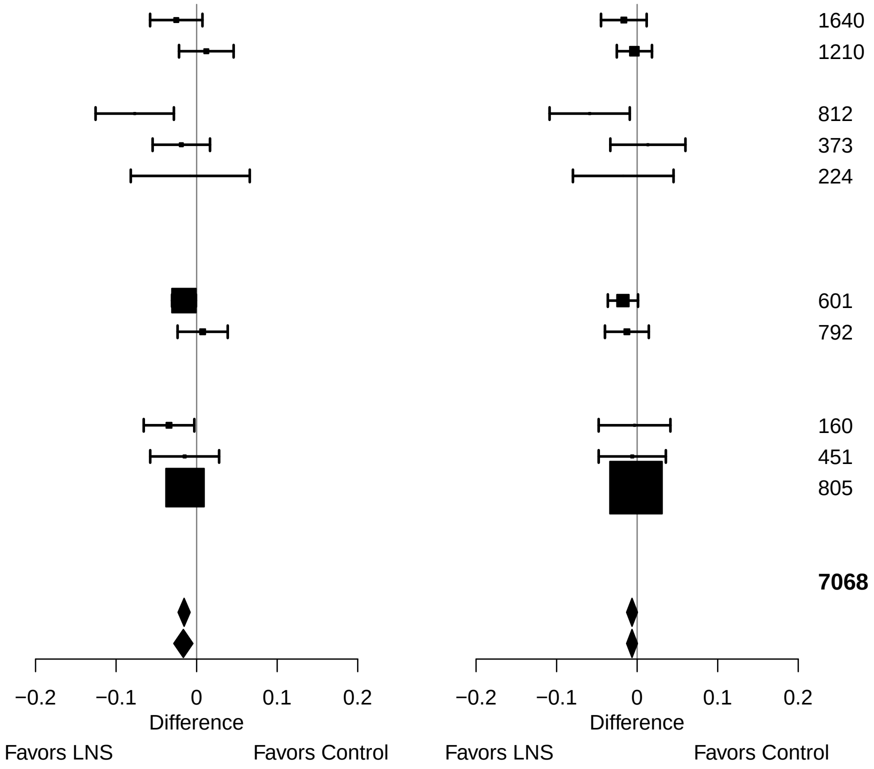

Supplemental figure 8I: Low MUAC prevalence difference

8I4: Stratified by Maternal education

P−for−interaction = 0.676
Difference in PDs = 0.00 (−0.01, 0.02)

Country	Trial	Primary or greater: LNS N	Control N	Control Prevalence	PD (95% CI)	Fixed W	Random W	Incomplete or no formal: LNS N	Control N	Control Prevalence	PD (95% CI)	Fixed W	Random W
Bangladesh	JiVitA−4 (34)	1865	777	14.5	−0.03 (−0.06, −0.01)	0.15	0.20	1030	491	16.2	−0.01 (−0.04, 0.03)	0.11	0.11
Bangladesh	RDNS (35)	1245	589	4.1	0.01 (−0.01, 0.03)	0.25	0.26	418	226	8.0	−0.02 (−0.06, 0.03)	0.08	0.08
Bangladesh	WASH−B (36)												
Burkina Faso	iLiNS−Zinc (37)												
Burkina Faso	PROMIS (38)												
Burkina Faso	PROMIS CS (38)	34	39	15.4	−0.01 (−0.17, 0.16)	0.00	0.01	388	396	14.9	−0.01 (−0.06, 0.04)	0.06	0.06
Ghana	GHANA (39)												
Ghana	iLiNS−DYADG (40)												
Haiti	HAITI (41)												
Kenya	WASH−B (42)	704	2460	4.2	−0.02 (−0.03, 0.00)	0.46	0.34	755	2721	6.6	−0.01 (−0.03, 0.00)	0.43	0.43
Madagascar	MAHAY (43)	344	420	5.7	0.00 (−0.03, 0.04)	0.10	0.15	1336	1238	11.0	−0.01 (−0.04, 0.02)	0.17	0.17
Malawi	iLiNS−DYADM (44)												
Malawi	iLiNS−DOSE (45)												
Mali	PROMIS (46)												
Mali	PROMIS CS (46)	107	96	10.4	−0.05 (−0.12, 0.02)	0.02	0.04	846	874	11.1	−0.01 (−0.04, 0.02)	0.16	0.16
Zimbabwe	SHINE (HIV−) (47)												
Zimbabwe	SHINE (HIV+) (48)												
		**4299**	**4381**		**I² = 0.43, Tau² = 0.00**			**4773**	**5946**		**I² = 0.00, Tau² = 0.00**		
**Fixed**					**−0.01 (−0.02, 0.00)**						**−0.01 (−0.02, 0.00)**		
**Random**					**−0.01 (−0.03, 0.00)**						**−0.01 (−0.02, 0.00)**		

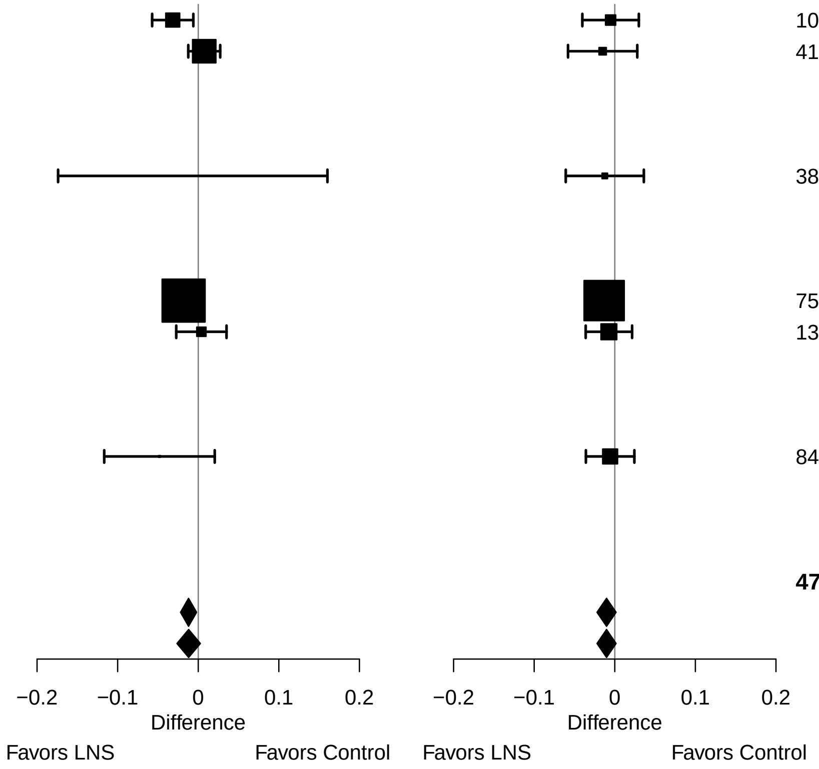

Supplemental figure 8I: Low MUAC prevalence difference

8I5: Stratified by Maternal depressive symptoms

P−for−interaction = 0.946
Difference in PDs = 0.00 (−0.01, 0.01)

		Less than 75th percentile						At least 75th percentile					
**Country**	**Trial**	**LNS N**	**Control N**	**Control Prevalence**	**PD (95% CI)**	**Fixed W**	**Random W**	**LNS N**	**Control N**	**Control Prevalence**	**PD (95% CI)**	**Fixed W**	**Random W**
Bangladesh	JiVitA−4 (34)												
Bangladesh	RDNS (35)	1081	472	5.3	0.00 (−0.03, 0.02)	0.08	0.08	520	288	4.5	0.01 (−0.02, 0.04)	0.13	0.13
Bangladesh	WASH−B (36)												
Burkina Faso	iLiNS−Zinc (37)												
Burkina Faso	PROMIS (38)	500	573	7.9	0.00 (−0.03, 0.03)	0.03	0.03	224	215	9.3	−0.03 (−0.08, 0.03)	0.05	0.05
Burkina Faso	PROMIS CS (38)												
Ghana	GHANA (39)												
Ghana	iLiNS−DYADG (40)												
Haiti	HAITI (41)												
Kenya	WASH−B (42)	999	3523	5.4	−0.01 (−0.03, 0.00)	0.25	0.25	340	1249	4.9	−0.01 (−0.04, 0.01)	0.22	0.22
Madagascar	MAHAY (43)	656	707	8.5	0.00 (−0.04, 0.03)	0.03	0.03	282	245	10.6	−0.01 (−0.06, 0.03)	0.06	0.06
Malawi	iLiNS−DYADM (44)												
Malawi	iLiNS−DOSE (45)												
Mali	PROMIS (46)												
Mali	PROMIS CS (46)												
Zimbabwe	SHINE (HIV−) (47)	1194	1135	1.4	−0.01 (−0.02, 0.00)	0.60	0.60	509	487	1.8	−0.01 (−0.02, 0.01)	0.55	0.55
Zimbabwe	SHINE (HIV+) (48)												
		**4430**	**6410**		**I² = 0.00, Tau² = 0.00**			**1875**	**2484**		**I² = 0.00, Tau² = 0.00**		
**Fixed**					**−0.01 (−0.01, 0.00)**						**−0.01 (−0.02, 0.00)**		
**Random**					**−0.01 (−0.01, 0.00)**						**−0.01 (−0.02, 0.00)**		

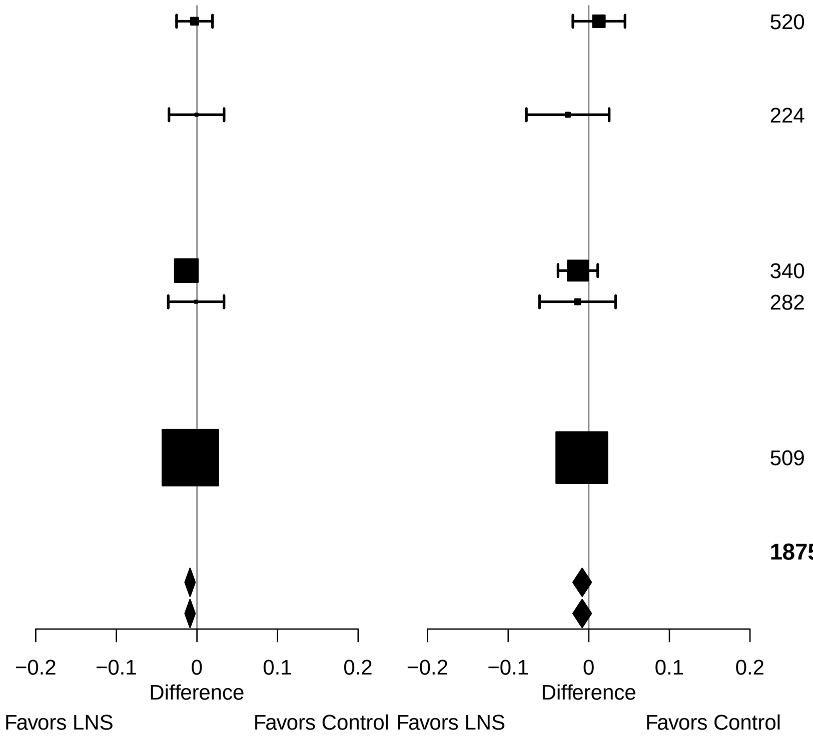

Supplemental figure 8I: Low MUAC prevalence difference

8I6: Stratified by Child sex

P−for−interaction = 0.032
Difference in PDs = −0.01 (−0.02, 0.00)

Country	Trial	Male LNS N	Male Control N	Male Control Prevalence	Male PD (95% CI)	Male Fixed W	Male Random W	Female LNS N	Female Control N	Female Control Prevalence	Female PD (95% CI)	Female Fixed W	Female Random W
Bangladesh	JiVitA−4 (34)	1477	612	12.1	0.00 (−0.03, 0.03)	0.06	0.06	1420	659	18.1	−0.04 (−0.06, −0.01)	0.05	0.09
Bangladesh	RDNS (35)	833	411	4.1	0.02 (−0.01, 0.04)	0.08	0.08	830	404	6.2	−0.02 (−0.04, 0.01)	0.05	0.09
Bangladesh	WASH−B (36)												
Burkina Faso	iLiNS−Zinc (37)	995	336	17.9	−0.06 (−0.10, −0.02)	0.03	0.03	964	330	18.8	−0.08 (−0.13, −0.03)	0.01	0.03
Burkina Faso	PROMIS (38)	432	482	8.1	0.00 (−0.04, 0.04)	0.03	0.03	422	430	7.9	−0.01 (−0.05, 0.03)	0.03	0.06
Burkina Faso	PROMIS CS (38)	229	219	10.5	0.01 (−0.04, 0.06)	0.02	0.02	195	217	19.4	−0.03 (−0.11, 0.04)	0.01	0.02
Ghana	GHANA (39)												
Ghana	iLiNS−DYADG (40)	175	324	4.3	−0.01 (−0.05, 0.02)	0.04	0.04	172	369	3.3	0.00 (−0.04, 0.03)	0.04	0.07
Haiti	HAITI (41)												
Kenya	WASH−B (42)	736	2467	5.9	0.00 (−0.02, 0.01)	0.14	0.14	725	2718	5.1	−0.03 (−0.04, −0.02)	0.25	0.20
Madagascar	MAHAY (43)	817	817	10.5	0.00 (−0.03, 0.03)	0.05	0.05	863	841	8.8	0.00 (−0.03, 0.02)	0.05	0.09
Malawi	iLiNS−DYADM (44)												
Malawi	iLiNS−DOSE (45)												
Mali	PROMIS (46)	267	263	5.3	−0.01 (−0.04, 0.02)	0.05	0.05	240	242	6.2	−0.04 (−0.08, 0.00)	0.02	0.05
Mali	PROMIS CS (46)	479	526	11.0	−0.01 (−0.05, 0.04)	0.03	0.03	475	444	11.0	−0.02 (−0.06, 0.02)	0.03	0.05
Zimbabwe	SHINE (HIV−) (47)	928	900	1.6	−0.01 (−0.02, 0.00)	0.49	0.49	944	880	1.4	−0.01 (−0.02, 0.00)	0.46	0.24
Zimbabwe	SHINE (HIV+) (48)												
		**7368**	**7357**		**I² = 0.06, Tau² = 0.00**			**7250**	**7534**		**I² = 0.44, Tau² = 0.00**		
**Fixed**					**−0.01 (−0.01, 0.00)**						**−0.02 (−0.02, −0.01)**		
**Random**					**−0.01 (−0.01, 0.00)**						**−0.02 (−0.03, −0.01)**		

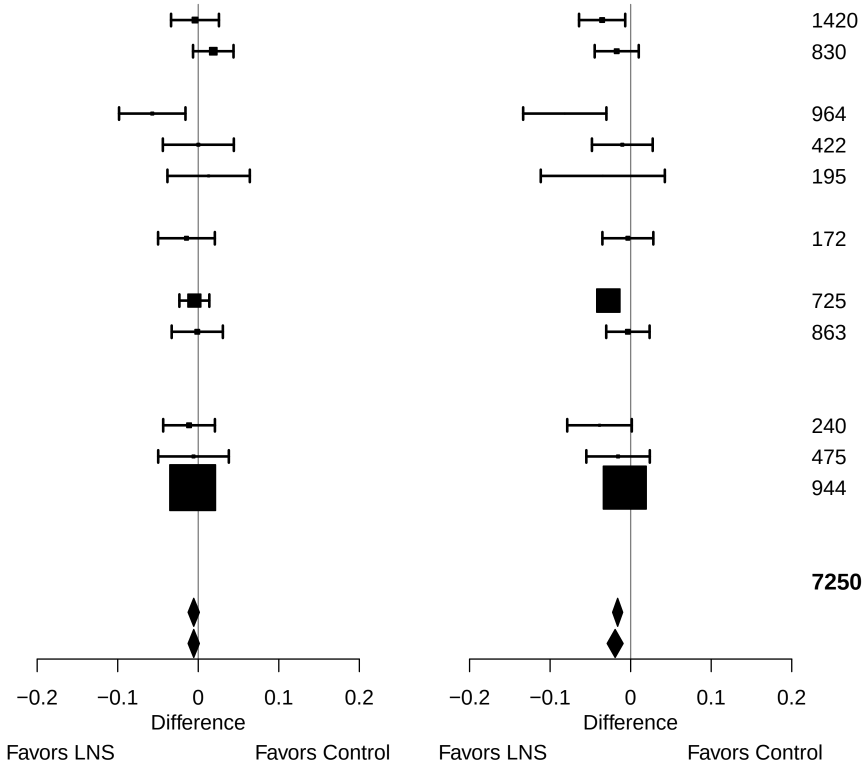

Supplemental figure 8I: Low MUAC prevalence difference

8I7: Stratified by Child birth order

P−for−interaction = 0.032
Difference in PDs = 0.02 (0.00, 0.03)

		Later born						Firstborn					
**Country**	**Trial**	**LNS N**	**Control N**	**Control Prevalence**	**PD (95% CI)**	**Fixed W**	**Random W**	**LNS N**	**Control N**	**Control Prevalence**	**PD (95% CI)**	**Fixed W**	**Random W**
Bangladesh	JiVitA−4 (34)	617	260	17.0	−0.06 (−0.10, −0.01)	0.04	0.09	2276	1004	14.6	−0.01 (−0.04, 0.01)	0.28	0.21
Bangladesh	RDNS (35)	974	508	6.9	−0.01 (−0.04, 0.01)	0.12	0.14	688	307	2.3	0.02 (0.00, 0.05)	0.21	0.19
Bangladesh	WASH−B (36)												
Burkina Faso	iLiNS−Zinc (37)	1524	521	18.2	−0.07 (−0.11, −0.03)	0.06	0.10	435	145	18.6	−0.05 (−0.11, 0.00)	0.06	0.09
Burkina Faso	PROMIS (38)	709	753	8.6	−0.01 (−0.04, 0.02)	0.08	0.12	145	158	5.1	0.03 (−0.03, 0.08)	0.05	0.08
Burkina Faso	PROMIS CS (38)	362	356	16.0	−0.03 (−0.08, 0.02)	0.03	0.07	61	80	10.0	0.08 (−0.04, 0.20)	0.01	0.02
Ghana	GHANA (39)												
Ghana	iLiNS−DYADG (40)												
Haiti	HAITI (41)												
Kenya	WASH−B (42)	1137	4083	5.5	−0.02 (−0.03, 0.00)	0.45	0.20	322	1097	5.4	−0.02 (−0.04, 0.01)	0.26	0.21
Madagascar	MAHAY (43)	1263	1199	10.4	0.00 (−0.03, 0.02)	0.14	0.15	417	459	7.6	0.00 (−0.04, 0.04)	0.10	0.14
Malawi	iLiNS−DYADM (44)												
Malawi	iLiNS−DOSE (45)												
Mali	PROMIS (46)												
Mali	PROMIS CS (46)	791	832	10.8	−0.01 (−0.04, 0.02)	0.09	0.13	146	126	10.3	−0.02 (−0.09, 0.05)	0.03	0.06
Zimbabwe	SHINE (HIV−) (47)												
Zimbabwe	SHINE (HIV+) (48)												
		**7377**	**8512**		**I^2 = 0.46, Tau^2 = 0.00**			**4490**	**3376**		**I^2 = 0.41, Tau^2 = 0.00**		
**Fixed**					**−0.02 (−0.03, −0.01)**						**0.00 (−0.02, 0.01)**		
**Random**					**−0.02 (−0.04, −0.01)**						**0.00 (−0.02, 0.02)**		

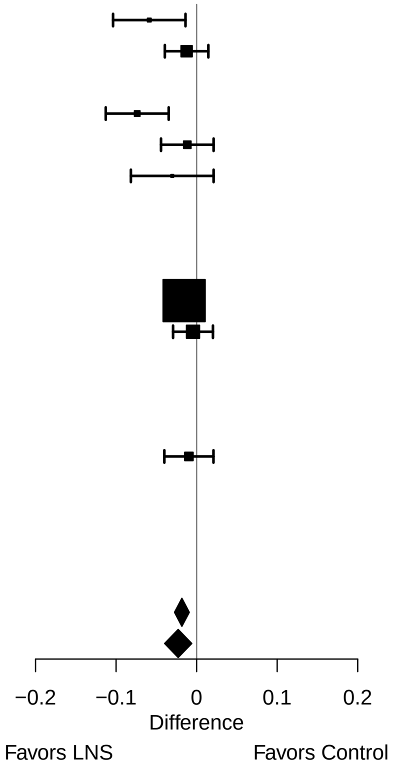

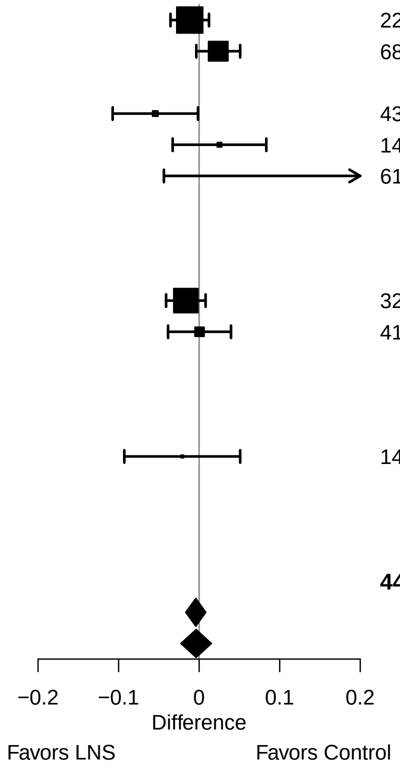

8I8: Stratified by Child baseline anthropometric status (insufficient comparisons)

Supplemental figure 8J: Acute malnutrition prevalence ratio

8J1: Stratified by Maternal height

P−for−interaction = 0.435
Ratio of PRs = 0.90 (0.68, 1.18)

		At least 150.1 cm						Less than 150.1 cm					
**Country**	**Trial**	**LNS N**	**Control N**	**Control Prevalence**	**PR (95% CI)**	**Fixed W**	**Random W**	**LNS N**	**Control N**	**Control Prevalence**	**PR (95% CI)**	**Fixed W**	**Random W**
Bangladesh	JiVitA−4 (34)												
Bangladesh	RDNS (35)	868	432	14.1	0.86 (0.66, 1.11)	0.36	0.32	733	358	17.0	0.83 (0.60, 1.15)	0.47	0.47
Bangladesh	WASH−B (36)												
Burkina Faso	iLiNS−Zinc (37)												
Burkina Faso	PROMIS (38)												
Burkina Faso	PROMIS CS (38)												
Ghana	GHANA (39)												
Ghana	iLiNS−DYADG (40)												
Haiti	HAITI (41)												
Kenya	WASH−B (42)												
Madagascar	MAHAY (43)	983	1018	7.6	1.10 (0.77, 1.57)	0.19	0.24	594	557	12.0	0.88 (0.62, 1.25)	0.39	0.39
Malawi	iLiNS−DYADM (44)												
Malawi	iLiNS−DOSE (45)	577	197	4.6	1.64 (0.85, 3.19)	0.06	0.10	112	42	11.9	0.90 (0.36, 2.28)	0.06	0.06
Mali	PROMIS (46)												
Mali	PROMIS CS (46)	907	927	12.8	0.86 (0.67, 1.10)	0.39	0.34	32	26	23.1	1.08 (0.52, 2.26)	0.09	0.09
Zimbabwe	SHINE (HIV−) (47)												
Zimbabwe	SHINE (HIV+) (48)												
		**3335**	**2574**		**I² = 0.33, Tau² = 0.02**			**1471**	**983**		**I² = 0.00, Tau² = 0.00**		
**Fixed**					**0.93 (0.80, 1.09)**						**0.88 (0.70, 1.09)**		
**Random**					**0.97 (0.77, 1.22)**						**0.88 (0.70, 1.09)**		

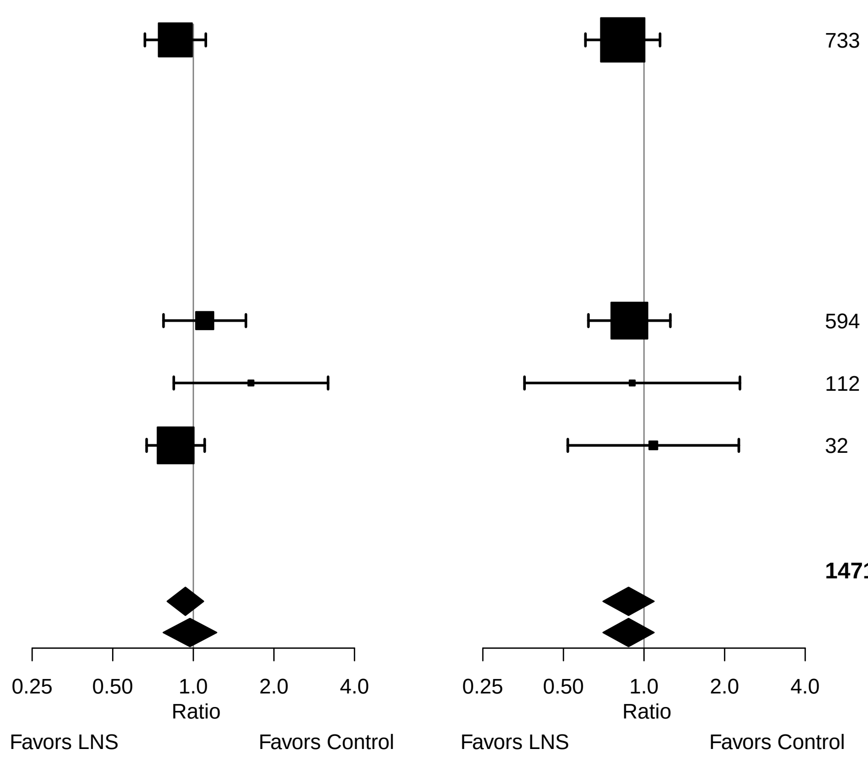

Supplemental figure 8J: Acute malnutrition prevalence ratio

8J2: Stratified by Maternal BMI

P−for−interaction = 0.490
Ratio of PRs = 1.07 (0.88, 1.30)

Country	Trial	LNS N	Control N	Control Prevalence	At least 20 kg/m² PR (95% CI)	Fixed W	Random W	LNS N	Control N	Control Prevalence	Less than 20 kg/m² PR (95% CI)	Fixed W	Random W
Bangladesh	JiVitA−4 (34)												
Bangladesh	RDNS (35)	730	335	9.6	0.99 (0.68, 1.44)	0.16	0.16	871	455	19.8	0.81 (0.65, 1.02)	0.27	0.27
Bangladesh	WASH−B (36)												
Burkina Faso	iLiNS−Zinc (37)	1214	390	15.3	0.59 (0.40, 0.87)	0.15	0.15	735	274	24.8	0.77 (0.63, 0.93)	0.36	0.34
Burkina Faso	PROMIS (38)	637	663	9.5	0.89 (0.58, 1.36)	0.13	0.13	206	232	17.7	0.74 (0.49, 1.11)	0.08	0.09
Burkina Faso	PROMIS CS (38)	241	265	15.5	0.89 (0.58, 1.34)	0.13	0.13	183	165	25.5	0.97 (0.71, 1.32)	0.14	0.15
Ghana	GHANA (39)												
Ghana	iLiNS−DYADG (40)												
Haiti	HAITI (41)												
Kenya	WASH−B (42)												
Madagascar	MAHAY (43)												
Malawi	iLiNS−DYADM (44)												
Malawi	iLiNS−DOSE (45)	509	174	5.2	1.00 (0.49, 2.02)	0.05	0.05	178	64	7.8	1.77 (0.72, 4.31)	0.02	0.02
Mali	PROMIS (46)												
Mali	PROMIS CS (46)	696	675	12.3	0.82 (0.61, 1.09)	0.27	0.27	243	278	15.1	1.04 (0.72, 1.49)	0.10	0.11
Zimbabwe	SHINE (HIV−) (47)	1353	1293	3.4	0.74 (0.47, 1.16)	0.11	0.11	239	247	5.7	0.66 (0.32, 1.40)	0.02	0.03
Zimbabwe	SHINE (HIV+) (48)												
		**5380**	**3795**		**I² = 0.00, Tau² = 0.00**			**2655**	**1715**		**I² = 0.06, Tau² = 0.00**		
**Fixed**					**0.82 (0.70, 0.95)**						**0.84 (0.74, 0.94)**		
**Random**					**0.82 (0.70, 0.95)**						**0.84 (0.74, 0.95)**		

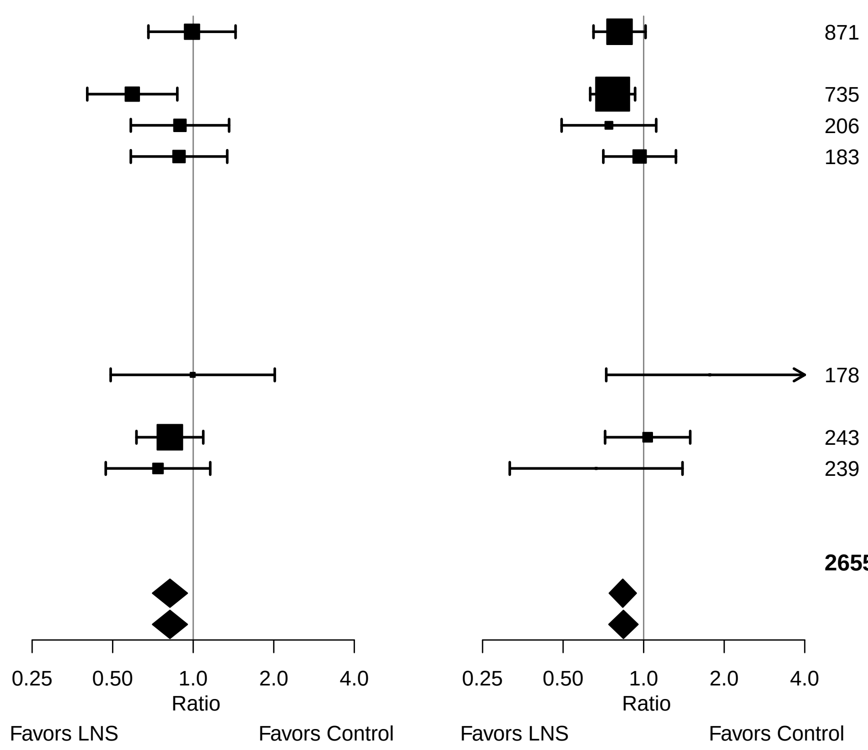

Supplemental figure 8J: Acute malnutrition prevalence ratio

8J3: Stratified by Maternal age

P−for−interaction = 0.429
Ratio of PRs = 1.06 (0.91, 1.23)

		At least 25 y						Less than 25 y					
Country	Trial	LNS N	Control N	Control Prevalence	PR (95% CI)	Fixed W	Random W	LNS N	Control N	Control Prevalence	PR (95% CI)	Fixed W	Random W
Bangladesh	JiVitA−4 (34)	1191	534	21.5	0.88 (0.72, 1.07)	0.30	0.19	1580	677	20.5	0.91 (0.76, 1.08)	0.35	0.35
Bangladesh	RDNS (35)	453	221	14.0	1.18 (0.83, 1.67)	0.10	0.11	1208	594	15.8	0.75 (0.58, 0.98)	0.16	0.16
Bangladesh	WASH−B (36)												
Burkina Faso	iLiNS−Zinc (37)	1131	399	19.5	0.62 (0.47, 0.81)	0.16	0.15	807	262	18.7	0.75 (0.53, 1.07)	0.09	0.09
Burkina Faso	PROMIS (38)	476	519	11.4	0.81 (0.55, 1.20)	0.08	0.10	370	382	12.0	0.85 (0.52, 1.40)	0.04	0.04
Burkina Faso	PROMIS CS (38)	200	189	20.6	0.90 (0.58, 1.38)	0.06	0.08	224	245	18.4	1.00 (0.73, 1.37)	0.11	0.11
Ghana	GHANA (39)												
Ghana	iLiNS−DYADG (40)												
Haiti	HAITI (41)												
Kenya	WASH−B (42)	841	2801	3.2	0.62 (0.38, 1.03)	0.05	0.07	598	2284	3.2	0.80 (0.46, 1.39)	0.04	0.04
Madagascar	MAHAY (43)	883	930	10.5	0.96 (0.71, 1.28)	0.14	0.14	791	725	8.0	1.04 (0.72, 1.50)	0.08	0.08
Malawi	iLiNS−DYADM (44)												
Malawi	iLiNS−DOSE (45)												
Mali	PROMIS (46)												
Mali	PROMIS CS (46)	498	501	13.8	0.82 (0.57, 1.18)	0.09	0.11	446	458	12.2	0.97 (0.70, 1.36)	0.10	0.10
Zimbabwe	SHINE (HIV−) (47)	870	794	4.5	0.53 (0.29, 0.97)	0.03	0.05	800	787	2.4	1.09 (0.62, 1.89)	0.04	0.04
Zimbabwe	SHINE (HIV+) (48)												
		**6543**	**6888**		**I² = 0.39, Tau² = 0.02**			**6824**	**6414**		**I² = 0.00, Tau² = 0.00**		
**Fixed**					**0.83 (0.74, 0.92)**						**0.89 (0.80, 0.99)**		
**Random**					**0.82 (0.71, 0.95)**						**0.89 (0.80, 0.99)**		

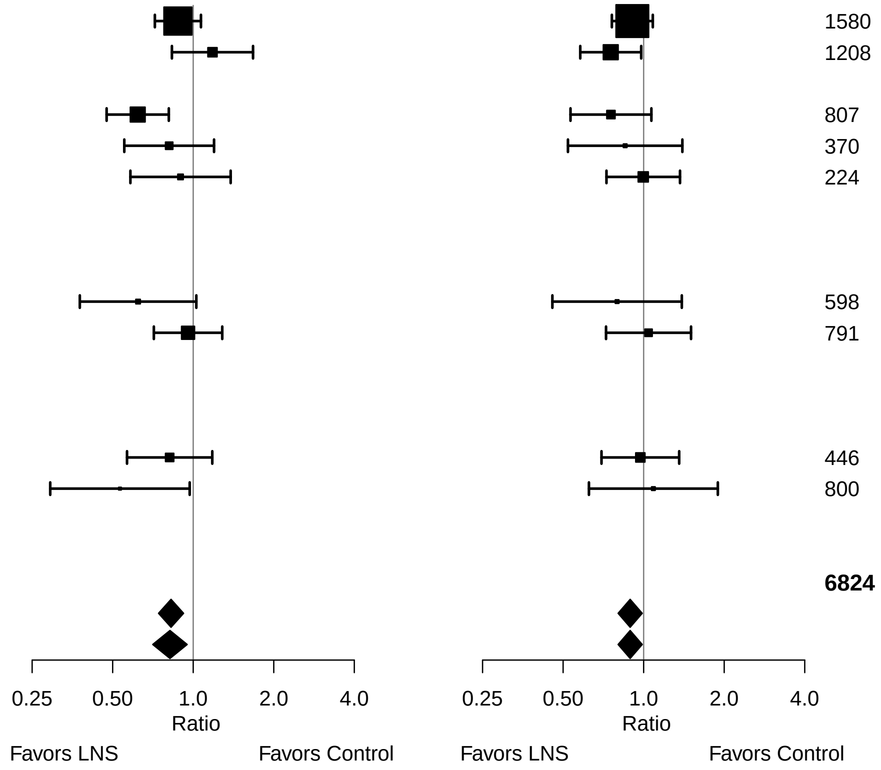

Supplemental figure 8J: Acute malnutrition prevalence ratio

8J4: Stratified by Maternal education

P−for−interaction = 0.659
Ratio of PRs = 1.04 (0.86, 1.26)

		Primary or greater						Incomplete or no formal					
**Country**	**Trial**	**LNS N**	**Control N**	**Control Prevalence**	**PR (95% CI)**	**Fixed W**	**Random W**	**LNS N**	**Control N**	**Control Prevalence**	**PR (95% CI)**	**Fixed W**	**Random W**
Bangladesh	JiVitA−4 (34)	1797	739	20.2	0.89 (0.75, 1.05)	0.57	0.57	984	474	22.3	0.91 (0.74, 1.11)	0.30	0.30
Bangladesh	RDNS (35)	1245	589	15.1	0.81 (0.63, 1.04)	0.25	0.25	416	226	15.9	1.01 (0.73, 1.40)	0.11	0.11
Bangladesh	WASH−B (36)												
Burkina Faso	iLiNS−Zinc (37)												
Burkina Faso	PROMIS (38)												
Burkina Faso	PROMIS CS (38)	34	39	20.5	1.15 (0.41, 3.24)	0.01	0.01	388	394	19.3	0.94 (0.71, 1.23)	0.16	0.16
Ghana	GHANA (39)												
Ghana	iLiNS−DYADG (40)	267	548	7.3	0.92 (0.54, 1.58)	0.05	0.05	80	144	11.1	0.68 (0.28, 1.66)	0.01	0.01
Haiti	HAITI (41)												
Kenya	WASH−B (42)	700	2426	2.5	0.75 (0.41, 1.39)	0.04	0.04	752	2687	3.8	0.69 (0.44, 1.11)	0.06	0.06
Madagascar	MAHAY (43)	342	420	5.5	1.23 (0.72, 2.08)	0.06	0.06	1333	1235	10.8	0.92 (0.68, 1.24)	0.14	0.14
Malawi	iLiNS−DYADM (44)												
Malawi	iLiNS−DOSE (45)												
Mali	PROMIS (46)												
Mali	PROMIS CS (46)	107	96	13.5	0.41 (0.17, 1.02)	0.02	0.02	836	863	13.0	0.95 (0.75, 1.20)	0.22	0.22
Zimbabwe	SHINE (HIV−) (47)												
Zimbabwe	SHINE (HIV+) (48)												
		**4492**	**4857**		**I² = 0.00, Tau² = 0.00**			**4789**	**6023**		**I² = 0.00, Tau² = 0.00**		
**Fixed**					**0.87 (0.77, 0.98)**						**0.92 (0.82, 1.02)**		
**Random**					**0.87 (0.77, 0.98)**						**0.92 (0.82, 1.02)**		

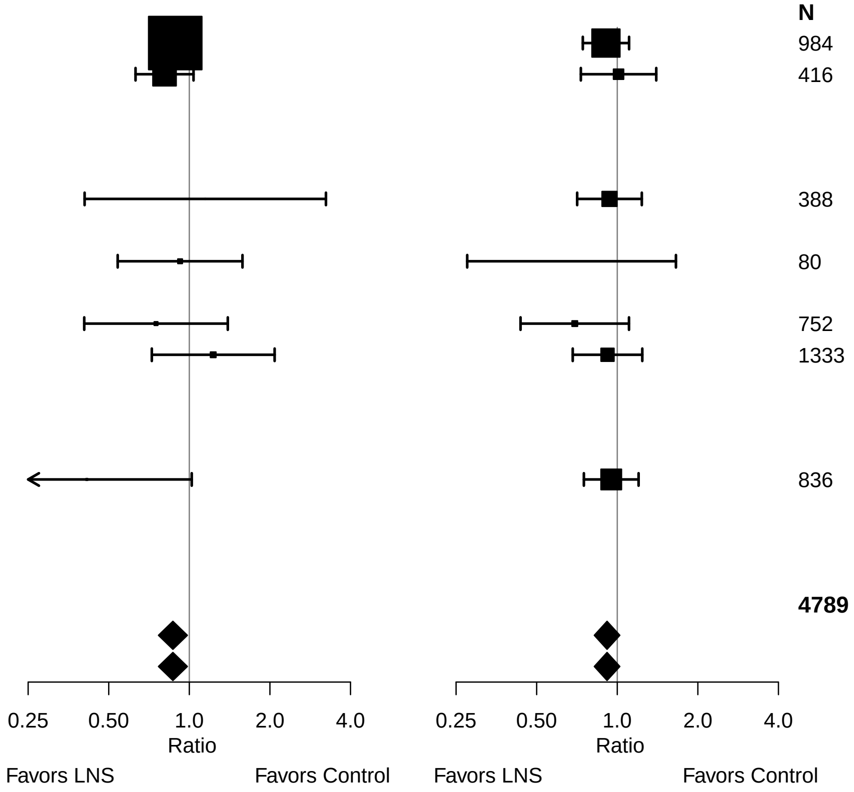

Supplemental figure 8J: Acute malnutrition prevalence ratio

8J5: Stratified by Maternal depressive symptoms

P−for−interaction = 0.532
Ratio of PRs = 1.08 (0.84, 1.39)

		Less than 75th percentile						At least 75th percentile					
**Country**	**Trial**	**LNS N**	**Control N**	**Control Prevalence**	**PR (95% CI)**	**Fixed W**	**Random W**	**LNS N**	**Control N**	**Control Prevalence**	**PR (95% CI)**	**Fixed W**	**Random W**
Bangladesh	JiVitA−4 (34)												
Bangladesh	RDNS (35)	1081	472	13.8	0.99 (0.77, 1.29)	0.36	0.36	519	288	17.0	0.71 (0.49, 1.04)	0.31	0.31
Bangladesh	WASH−B (36)												
Burkina Faso	iLiNS−Zinc (37)												
Burkina Faso	PROMIS (38)	497	567	11.6	0.78 (0.54, 1.12)	0.18	0.18	219	211	14.7	0.75 (0.47, 1.19)	0.20	0.20
Burkina Faso	PROMIS CS (38)												
Ghana	GHANA (39)												
Ghana	iLiNS−DYADG (40)	252	459	7.4	0.86 (0.48, 1.52)	0.07	0.07	83	209	8.6	1.12 (0.51, 2.47)	0.07	0.07
Haiti	HAITI (41)												
Kenya	WASH−B (42)	994	3480	3.0	0.64 (0.39, 1.04)	0.10	0.10	338	1232	3.5	0.85 (0.44, 1.62)	0.11	0.11
Madagascar	MAHAY (43)	654	707	9.1	0.96 (0.65, 1.42)	0.16	0.16	280	245	9.0	0.99 (0.60, 1.65)	0.17	0.17
Malawi	iLiNS−DYADM (44)												
Malawi	iLiNS−DOSE (45)												
Mali	PROMIS (46)												
Mali	PROMIS CS (46)												
Zimbabwe	SHINE (HIV−) (47)	1191	1133	3.4	0.78 (0.48, 1.24)	0.11	0.11	508	486	3.9	0.70 (0.36, 1.37)	0.10	0.10
Zimbabwe	SHINE (HIV+) (48)	234	214	3.7	1.49 (0.62, 3.54)	0.03	0.03	88	99	6.1	0.94 (0.28, 3.12)	0.03	0.03
		**4903**	**7032**		**I² = 0.00, Tau² = 0.00**			**2035**	**2770**		**I² = 0.00, Tau² = 0.00**		
**Fixed**					**0.88 (0.76, 1.03)**						**0.81 (0.65, 1.00)**		
**Random**					**0.88 (0.76, 1.03)**						**0.81 (0.65, 1.00)**		

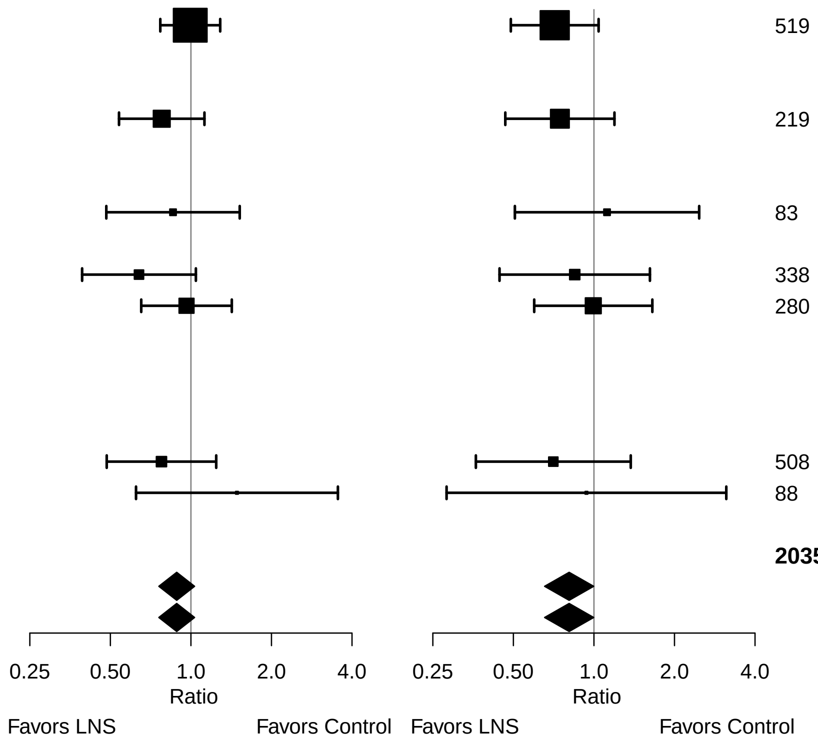

Supplemental figure 8J: Acute malnutrition prevalence ratio

8J6: Stratified by Child sex

P−for−interaction = 0.307
Ratio of PRs = 0.93 (0.81, 1.07)

Country	Trial	Male LNS N	Male Control N	Male Control Prevalence	Male PR (95% CI)	Male Fixed W	Male Random W	Female LNS N	Female Control N	Female Control Prevalence	Female PR (95% CI)	Female Fixed W	Female Random W
Bangladesh	JiVitA−4 (34)	1420	586	17.8	1.01 (0.83, 1.22)	0.30	0.30	1363	630	24.1	0.81 (0.69, 0.96)	0.37	0.30
Bangladesh	RDNS (35)	832	411	15.6	0.90 (0.68, 1.18)	0.15	0.15	829	404	15.1	0.82 (0.61, 1.11)	0.12	0.12
Bangladesh	WASH−B (36)												
Burkina Faso	iLiNS−Zinc (37)	990	336	17.3	0.75 (0.59, 0.96)	0.18	0.18	961	328	21.3	0.62 (0.45, 0.86)	0.10	0.11
Burkina Faso	PROMIS (38)	427	477	13.0	0.74 (0.46, 1.19)	0.05	0.05	419	424	10.1	0.96 (0.62, 1.51)	0.05	0.06
Burkina Faso	PROMIS CS (38)	229	219	17.8	1.01 (0.66, 1.52)	0.06	0.06	195	215	20.9	0.91 (0.61, 1.34)	0.07	0.08
Ghana	GHANA (39)												
Ghana	iLiNS−DYADG (40)	175	323	10.5	0.60 (0.31, 1.15)	0.03	0.03	172	369	6.0	1.27 (0.65, 2.46)	0.02	0.03
Haiti	HAITI (41)												
Kenya	WASH−B (42)	732	2430	3.1	0.83 (0.49, 1.40)	0.04	0.04	722	2687	3.3	0.59 (0.35, 1.01)	0.04	0.04
Madagascar	MAHAY (43)	812	814	10.3	0.88 (0.61, 1.28)	0.08	0.08	863	841	8.6	1.10 (0.80, 1.50)	0.11	0.11
Malawi	iLiNS−DYADM (44)												
Malawi	iLiNS−DOSE (45)												
Mali	PROMIS (46)												
Mali	PROMIS CS (46)	472	520	13.3	0.91 (0.63, 1.31)	0.08	0.08	472	439	12.8	0.86 (0.61, 1.22)	0.09	0.10
Zimbabwe	SHINE (HIV−) (47)	924	900	3.9	0.75 (0.47, 1.21)	0.05	0.05	943	877	2.9	0.86 (0.49, 1.48)	0.03	0.04
Zimbabwe	SHINE (HIV+) (48)												
		**7013**	**7016**		**I² = 0.00, Tau² = 0.00**			**6939**	**7214**		**I² = 0.10, Tau² = 0.00**		
**Fixed**					**0.88 (0.79, 0.97)**						**0.84 (0.75, 0.92)**		
**Random**					**0.88 (0.79, 0.97)**						**0.84 (0.75, 0.94)**		

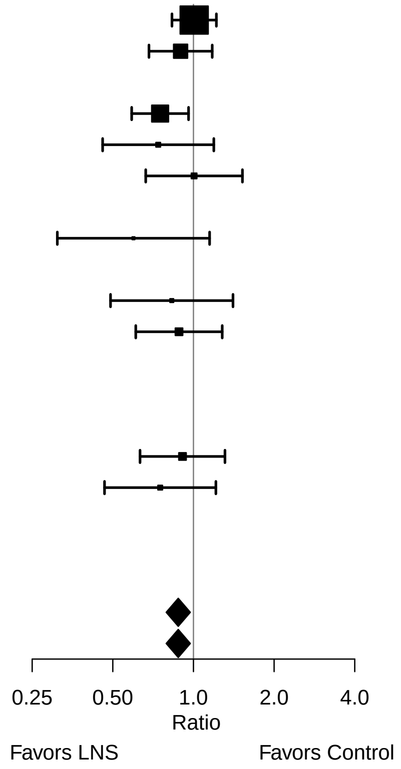

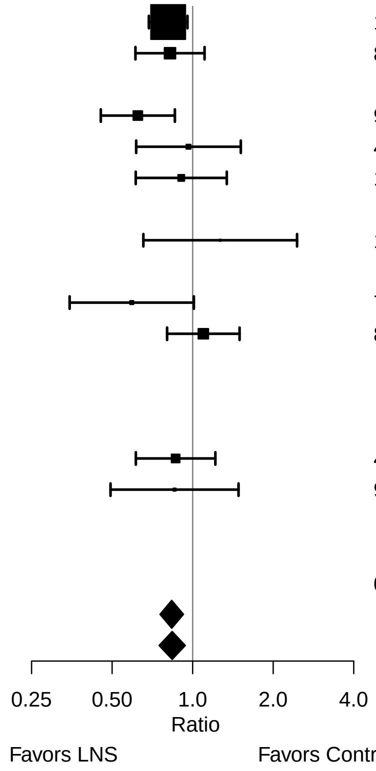

Supplemental figure 8J: Acute malnutrition prevalence ratio

8J7: Stratified by Child birth order

P−for−interaction = 0.124
Ratio of PRs = 1.14 (0.96, 1.35)

Country	Trial	Later born LNS N	Later born Control N	Later born Control Prevalence	Later born PR (95% CI)	Later born Fixed W	Later born Random W	Firstborn LNS N	Firstborn Control N	Firstborn Control Prevalence	Firstborn PR (95% CI)	Firstborn Fixed W	Firstborn Random W
Bangladesh	JiVitA−4 (34)	598	247	23.1	0.79 (0.61, 1.03)	0.12	0.12	2181	963	20.4	0.93 (0.81, 1.08)	0.62	0.62
Bangladesh	RDNS (35)	972	508	16.1	0.85 (0.66, 1.08)	0.15	0.15	688	307	14.0	0.89 (0.63, 1.27)	0.11	0.11
Bangladesh	WASH−B (36)												
Burkina Faso	iLiNS−Zinc (37)	1520	519	18.5	0.66 (0.53, 0.81)	0.20	0.20	431	145	22.1	0.72 (0.46, 1.13)	0.06	0.06
Burkina Faso	PROMIS (38)	701	743	12.0	0.83 (0.61, 1.15)	0.09	0.09	145	157	10.2	0.81 (0.42, 1.58)	0.03	0.03
Burkina Faso	PROMIS CS (38)	362	355	19.7	0.90 (0.67, 1.20)	0.11	0.11	61	79	17.7	1.30 (0.70, 2.41)	0.03	0.03
Ghana	GHANA (39)												
Ghana	iLiNS−DYADG (40)												
Haiti	HAITI (41)												
Kenya	WASH−B (42)	1131	4026	3.4	0.69 (0.45, 1.05)	0.05	0.05	321	1086	2.6	0.85 (0.39, 1.85)	0.02	0.02
Madagascar	MAHAY (43)	1259	1196	10.4	0.93 (0.70, 1.25)	0.11	0.11	416	459	7.0	1.14 (0.74, 1.75)	0.07	0.07
Malawi	iLiNS−DYADM (44)												
Malawi	iLiNS−DOSE (45)												
Mali	PROMIS (46)												
Mali	PROMIS CS (46)	783	821	13.0	0.90 (0.70, 1.16)	0.14	0.14	144	126	11.1	0.75 (0.36, 1.55)	0.02	0.02
Zimbabwe	SHINE (HIV−) (47)	1294	1211	3.5	0.71 (0.45, 1.14)	0.04	0.04	486	483	3.5	0.94 (0.51, 1.73)	0.03	0.03
Zimbabwe	SHINE (HIV+) (48)												
		**8620**	**9626**		**I² = 0.00, Tau² = 0.00**			**4873**	**3805**		**I² = 0.00, Tau² = 0.00**		
**Fixed**					**0.80 (0.73, 0.88)**						**0.93 (0.83, 1.04)**		
**Random**					**0.80 (0.73, 0.88)**						**0.93 (0.83, 1.04)**		

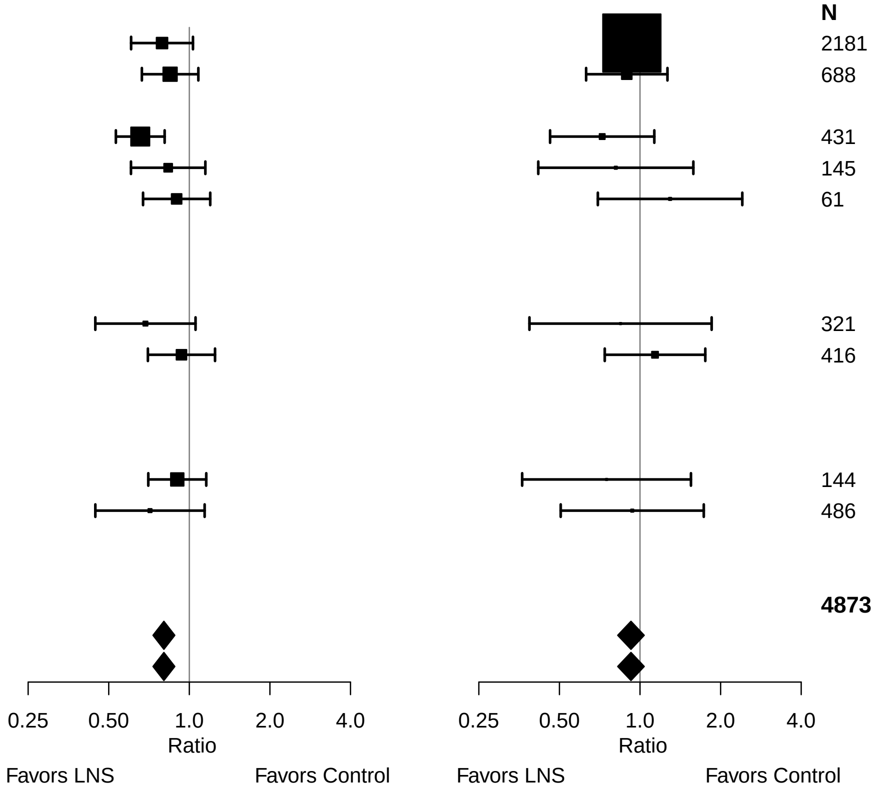

Supplemental figure 8J: Acute malnutrition prevalence ratio

8J8: Stratified by Child baseline anthropometric status

P−for−interaction = 0.490
Ratio of PRs = 0.85 (0.53, 1.36)

Country	Trial	WLZ at least 0 Z: LNS N	Control N	Control Prevalence	PR (95% CI)	Fixed W	Random W	WLZ less than 0 Z: LNS N	Control N	Control Prevalence	PR (95% CI)	Fixed W	Random W
Bangladesh	JiVitA−4 (34)	961	423	4.0	0.93 (0.51, 1.71)	0.55	0.55	1822	793	30.0	0.88 (0.78, 1.00)	0.84	0.84
Bangladesh	RDNS (35)												
Bangladesh	WASH−B (36)												
Burkina Faso	iLiNS−Zinc (37)												
Burkina Faso	PROMIS (38)	275	346	3.5	0.73 (0.25, 2.19)	0.17	0.17	408	392	18.6	0.78 (0.56, 1.08)	0.12	0.12
Burkina Faso	PROMIS CS (38)												
Ghana	GHANA (39)												
Ghana	iLiNS−DYADG (40)												
Haiti	HAITI (41)												
Kenya	WASH−B (42)												
Madagascar	MAHAY (43)												
Malawi	iLiNS−DYADM (44)												
Malawi	iLiNS−DOSE (45)												
Mali	PROMIS (46)												
Mali	PROMIS CS (46)												
Zimbabwe	SHINE (HIV−) (47)	742	699	1.1	1.18 (0.50, 2.78)	0.28	0.28	555	476	6.5	0.61 (0.34, 1.09)	0.04	0.04
Zimbabwe	SHINE (HIV+) (48)												
		**1978**	**1468**		**I² = 0.00, Tau² = 0.00**			**2785**	**1661**		**I² = 0.00, Tau² = 0.00**		
**Fixed**					**0.95 (0.61, 1.50)**						**0.85 (0.76, 0.96)**		
**Random**					**0.95 (0.61, 1.50)**						**0.85 (0.76, 0.96)**		

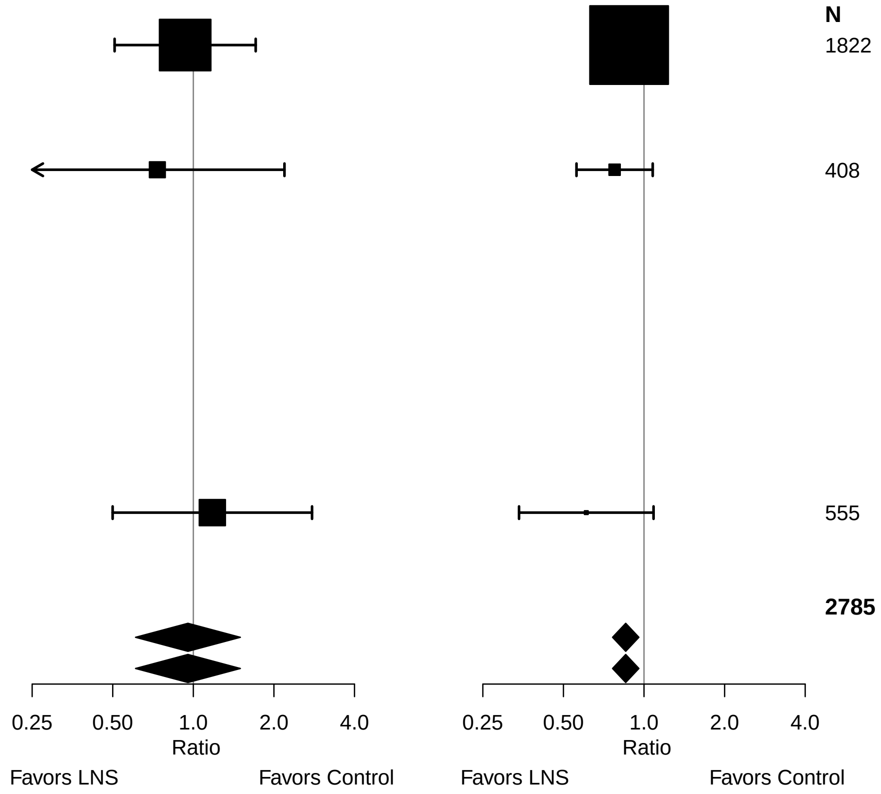

Supplemental figure 8K: Acute malnutrition prevalence difference

8K1: Stratified by Maternal height

P−for−interaction = 0.297
Difference in PDs = −0.02 (−0.05, 0.02)

		At least 150.1 cm						Less than 150.1 cm					
**Country**	**Trial**	**LNS N**	**Control N**	**Control Prevalence**	**PD (95% CI)**	**Fixed W**	**Random W**	**LNS N**	**Control N**	**Control Prevalence**	**PD (95% CI)**	**Fixed W**	**Random W**
Bangladesh	JiVitA−4 (34)												
Bangladesh	RDNS (35)	868	432	14.1	−0.02 (−0.05, 0.01)	0.22	0.22	733	358	17.0	−0.03 (−0.08, 0.02)	0.33	0.33
Bangladesh	WASH−B (36)												
Burkina Faso	iLiNS−Zinc (37)												
Burkina Faso	PROMIS (38)												
Burkina Faso	PROMIS CS (38)												
Ghana	GHANA (39)												
Ghana	iLiNS−DYADG (40)												
Haiti	HAITI (41)												
Kenya	WASH−B (42)												
Madagascar	MAHAY (43)	983	1018	7.6	0.01 (−0.02, 0.04)	0.32	0.32	594	557	12.0	−0.01 (−0.05, 0.03)	0.56	0.56
Malawi	iLiNS−DYADM (44)												
Malawi	iLiNS−DOSE (45)	577	197	4.6	0.01 (−0.02, 0.05)	0.18	0.18	112	42	11.9	−0.02 (−0.13, 0.08)	0.08	0.08
Mali	PROMIS (46)												
Mali	PROMIS CS (46)	907	927	12.8	−0.02 (−0.05, 0.01)	0.28	0.28	32	26	23.1	0.02 (−0.16, 0.20)	0.03	0.03
Zimbabwe	SHINE (HIV−) (47)												
Zimbabwe	SHINE (HIV+) (48)												
		**3335**	**2574**		**I² = 0.08, Tau² = 0.00**			**1471**	**983**		**I² = 0.00, Tau² = 0.00**		
**Fixed**					**0.00 (−0.02, 0.01)**						**−0.02 (−0.05, 0.01)**		
**Random**					**0.00 (−0.02, 0.01)**						**−0.02 (−0.05, 0.01)**		

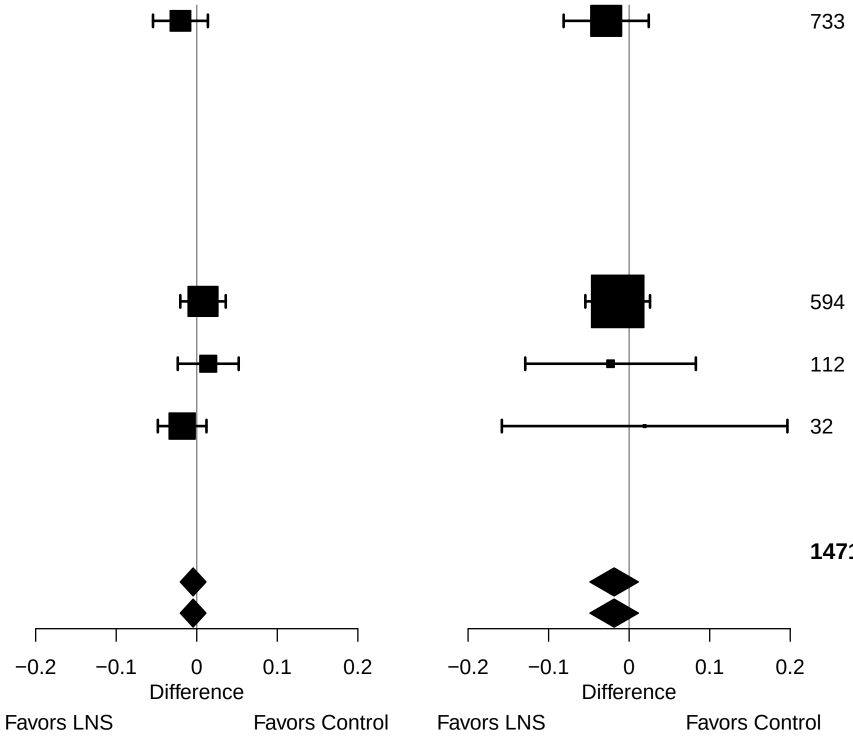

Supplemental figure 8K: Acute malnutrition prevalence difference

8K2: Stratified by Maternal BMI

P−for−interaction = 0.580
Difference in PDs = −0.01 (−0.03, 0.02)

Country	Trial	At least 20 kg/m² LNS N	Control N	Control Prevalence	PD (95% CI)	Fixed W	Random W	Less than 20 kg/m² LNS N	Control N	Control Prevalence	PD (95% CI)	Fixed W	Random W
Bangladesh	JiVitA−4 (34)												
Bangladesh	RDNS (35)	730	335	9.6	0.00 (−0.04, 0.03)	0.08	0.08	871	455	19.8	−0.04 (−0.08, 0.00)	0.21	0.20
Bangladesh	WASH−B (36)												
Burkina Faso	iLiNS−Zinc (37)	1214	390	15.3	−0.06 (−0.10, −0.02)	0.06	0.06	735	274	24.8	−0.06 (−0.11, −0.02)	0.18	0.18
Burkina Faso	PROMIS (38)	637	663	9.5	−0.01 (−0.05, 0.03)	0.07	0.07	206	232	17.7	−0.05 (−0.11, 0.02)	0.09	0.10
Burkina Faso	PROMIS CS (38)	241	265	15.5	−0.02 (−0.08, 0.04)	0.03	0.03	183	165	25.5	−0.01 (−0.09, 0.07)	0.06	0.07
Ghana	GHANA (39)												
Ghana	iLiNS−DYADG (40)												
Haiti	HAITI (41)												
Kenya	WASH−B (42)												
Madagascar	MAHAY (43)												
Malawi	iLiNS−DYADM (44)												
Malawi	iLiNS−DOSE (45)	509	174	5.2	−0.01 (−0.04, 0.03)	0.07	0.07	178	64	7.8	0.04 (−0.05, 0.13)	0.05	0.06
Mali	PROMIS (46)												
Mali	PROMIS CS (46)	696	675	12.3	−0.02 (−0.06, 0.01)	0.09	0.09	243	278	15.1	0.01 (−0.05, 0.06)	0.11	0.12
Zimbabwe	SHINE (HIV−) (47)	1353	1293	3.4	−0.01 (−0.02, 0.00)	0.59	0.59	239	247	5.7	−0.02 (−0.05, 0.02)	0.31	0.27
Zimbabwe	SHINE (HIV+) (48)												
		**5380**	**3795**		**I² = 0.00, Tau² = 0.00**			**2655**	**1715**		**I² = 0.16, Tau² = 0.00**		
**Fixed**					**−0.01 (−0.02, 0.00)**						**−0.03 (−0.05, −0.01)**		
**Random**					**−0.01 (−0.02, 0.00)**						**−0.03 (−0.05, 0.00)**		

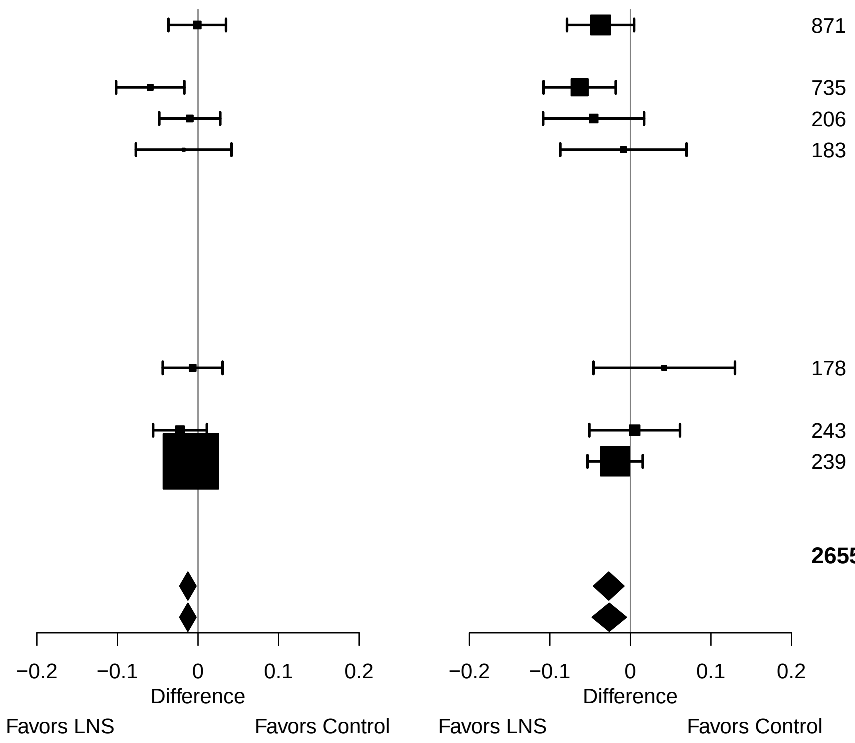

Supplemental figure 8K: Acute malnutrition prevalence difference

8K3: Stratified by Maternal age

P−for−interaction = 0.115
Difference in PDs = 0.01 (0.00, 0.02)

Country	Trial	At least 25 y: LNS N	Control N	Control Prevalence	PD (95% CI)	Fixed W	Random W	Less than 25 y: LNS N	Control N	Control Prevalence	PD (95% CI)	Fixed W	Random W
Bangladesh	JiVitA−4 (34)	1191	534	21.5	−0.02 (−0.06, 0.01)	0.05	0.09	1580	677	20.5	−0.02 (−0.05, 0.01)	0.07	0.07
Bangladesh	RDNS (35)	453	221	14.0	0.03 (−0.03, 0.08)	0.03	0.05	1208	594	15.8	−0.04 (−0.08, 0.00)	0.05	0.05
Bangladesh	WASH−B (36)												
Burkina Faso	iLiNS−Zinc (37)	1131	399	19.5	−0.07 (−0.12, −0.03)	0.04	0.07	807	262	18.7	−0.05 (−0.10, 0.01)	0.02	0.02
Burkina Faso	PROMIS (38)	476	519	11.4	−0.02 (−0.06, 0.02)	0.04	0.09	370	382	12.0	−0.02 (−0.07, 0.04)	0.02	0.02
Burkina Faso	PROMIS CS (38)	200	189	20.6	−0.02 (−0.11, 0.06)	0.01	0.02	224	245	18.4	0.00 (−0.06, 0.06)	0.02	0.02
Ghana	GHANA (39)												
Ghana	iLiNS−DYADG (40)												
Haiti	HAITI (41)												
Kenya	WASH−B (42)	841	2801	3.2	−0.01 (−0.02, 0.00)	0.55	0.28	598	2284	3.2	−0.01 (−0.02, 0.01)	0.34	0.34
Madagascar	MAHAY (43)	883	930	10.5	0.00 (−0.03, 0.03)	0.07	0.12	791	725	8.0	0.00 (−0.03, 0.03)	0.08	0.08
Malawi	iLiNS−DYADM (44)												
Malawi	iLiNS−DOSE (45)												
Mali	PROMIS (46)												
Mali	PROMIS CS (46)	498	501	13.8	−0.03 (−0.07, 0.02)	0.03	0.07	446	458	12.2	0.00 (−0.04, 0.04)	0.04	0.04
Zimbabwe	SHINE (HIV−) (47)	870	794	4.5	−0.02 (−0.04, 0.00)	0.18	0.20	800	787	2.4	0.00 (−0.01, 0.02)	0.36	0.36
Zimbabwe	SHINE (HIV+) (48)												
		**6543**	**6888**		**I² = 0.21, Tau² = 0.00**			**6824**	**6414**		**I² = 0.00, Tau² = 0.00**		
**Fixed**					**−0.02 (−0.02, −0.01)**						**−0.01 (−0.01, 0.00)**		
**Random**					**−0.02 (−0.03, 0.00)**						**−0.01 (−0.01, 0.00)**		

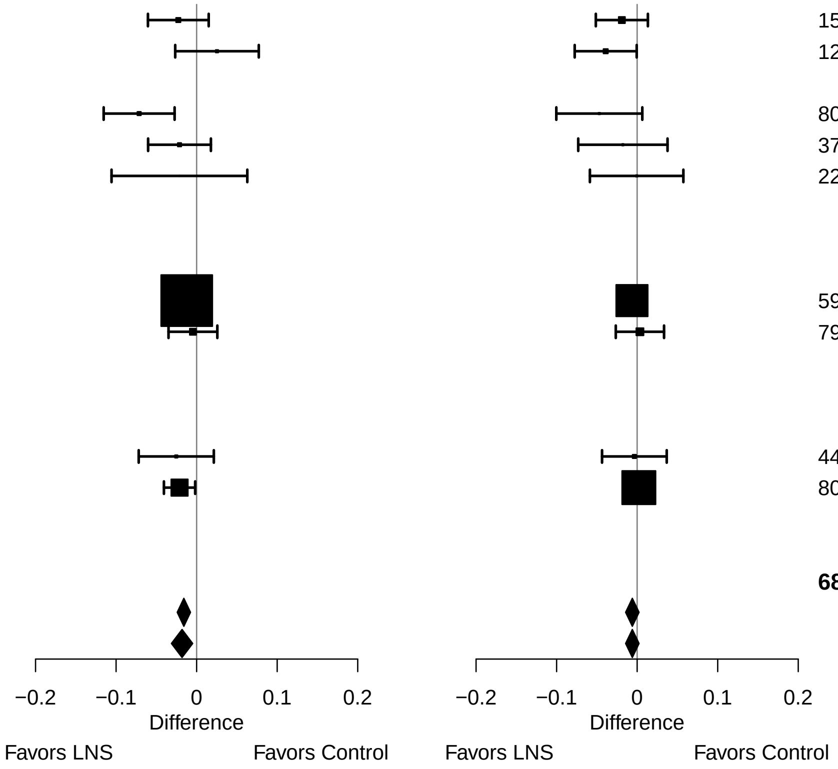

Supplemental figure 8K: Acute malnutrition prevalence difference

8K4: Stratified by Maternal education

P−for−interaction = 0.659
Difference in PDs = 0.00 (−0.02, 0.01)

Country	Trial	Primary or greater: LNS N	Control N	Control Prevalence	PD (95% CI)	Fixed W	Random W	Incomplete or no formal: LNS N	Control N	Control Prevalence	PD (95% CI)	Fixed W	Random W
Bangladesh	JiVitA−4 (34)	1797	739	20.2	−0.03 (−0.05, 0.00)	0.11	0.16	984	474	22.3	−0.02 (−0.06, 0.03)	0.06	0.06
Bangladesh	RDNS (35)	1245	589	15.1	−0.03 (−0.06, 0.01)	0.07	0.12	416	226	15.9	0.00 (−0.05, 0.05)	0.04	0.04
Bangladesh	WASH−B (36)												
Burkina Faso	iLiNS−Zinc (37)												
Burkina Faso	PROMIS (38)												
Burkina Faso	PROMIS CS (38)	34	39	20.5	0.03 (−0.20, 0.26)	0.00	0.00	388	394	19.3	−0.01 (−0.06, 0.04)	0.04	0.04
Ghana	GHANA (39)												
Ghana	iLiNS−DYADG (40)	267	548	7.3	−0.01 (−0.04, 0.03)	0.06	0.10	80	144	11.1	−0.04 (−0.12, 0.05)	0.02	0.02
Haiti	HAITI (41)												
Kenya	WASH−B (42)	700	2426	2.5	−0.01 (−0.02, 0.01)	0.65	0.46	752	2687	3.8	−0.01 (−0.02, 0.00)	0.62	0.62
Madagascar	MAHAY (43)	342	420	5.5	0.01 (−0.02, 0.05)	0.08	0.13	1333	1235	10.8	−0.01 (−0.04, 0.02)	0.11	0.11
Malawi	iLiNS−DYADM (44)												
Malawi	iLiNS−DOSE (45)												
Mali	PROMIS (46)												
Mali	PROMIS CS (46)	107	96	13.5	−0.08 (−0.16, 0.00)	0.01	0.03	836	863	13.0	−0.01 (−0.04, 0.02)	0.12	0.12
Zimbabwe	SHINE (HIV−) (47)												
Zimbabwe	SHINE (HIV+) (48)												
		**4492**	**4857**		**I² = 0.18, Tau² = 0.00**			**4789**	**6023**		**I² = 0.00, Tau² = 0.00**		
**Fixed**					**−0.01 (−0.02, 0.00)**						**−0.01 (−0.02, 0.00)**		
**Random**					**−0.01 (−0.02, 0.00)**						**−0.01 (−0.02, 0.00)**		

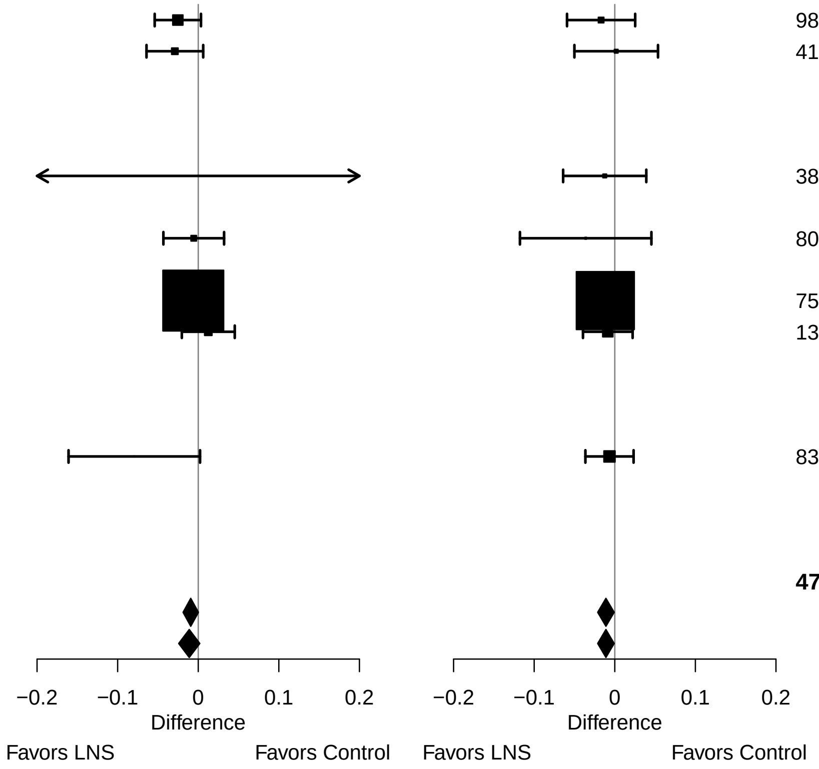

Supplemental figure 8K: Acute malnutrition prevalence difference

8K5: Stratified by Maternal depressive symptoms

P−for−interaction = 0.781
Difference in PDs = 0.00 (−0.01, 0.02)

		Less than 75th percentile						At least 75th percentile					
**Country**	**Trial**	**LNS N**	**Control N**	**Control Prevalence**	**PD (95% CI)**	**Fixed W**	**Random W**	**LNS N**	**Control N**	**Control Prevalence**	**PD (95% CI)**	**Fixed W**	**Random W**
Bangladesh	JiVitA−4 (34)												
Bangladesh	RDNS (35)	1081	472	13.8	0.00 (−0.04, 0.03)	0.04	0.04	519	288	17.0	−0.05 (−0.11, 0.01)	0.05	0.05
Bangladesh	WASH−B (36)												
Burkina Faso	iLiNS−Zinc (37)												
Burkina Faso	PROMIS (38)	497	567	11.6	−0.03 (−0.06, 0.01)	0.04	0.04	219	211	14.7	−0.04 (−0.10, 0.02)	0.04	0.04
Burkina Faso	PROMIS CS (38)												
Ghana	GHANA (39)												
Ghana	iLiNS−DYADG (40)	252	459	7.4	−0.01 (−0.05, 0.03)	0.03	0.03	83	209	8.6	0.01 (−0.06, 0.08)	0.03	0.03
Haiti	HAITI (41)												
Kenya	WASH−B (42)	994	3480	3.0	−0.01 (−0.02, 0.00)	0.52	0.52	338	1232	3.5	−0.01 (−0.02, 0.01)	0.42	0.42
Madagascar	MAHAY (43)	654	707	9.1	0.00 (−0.04, 0.03)	0.04	0.04	280	245	9.0	0.00 (−0.05, 0.05)	0.08	0.08
Malawi	iLiNS−DYADM (44)												
Malawi	iLiNS−DOSE (45)												
Mali	PROMIS (46)												
Mali	PROMIS CS (46)												
Zimbabwe	SHINE (HIV−) (47)	1191	1133	3.4	−0.01 (−0.02, 0.01)	0.28	0.28	508	486	3.9	−0.01 (−0.03, 0.01)	0.35	0.35
Zimbabwe	SHINE (HIV+) (48)	234	214	3.7	0.02 (−0.02, 0.06)	0.03	0.03	88	99	6.1	0.00 (−0.07, 0.07)	0.03	0.03
		**4903**	**7032**		**I² = 0.00, Tau² = 0.00**			**2035**	**2770**		**I² = 0.00, Tau² = 0.00**		
**Fixed**					**−0.01 (−0.02, 0.00)**						**−0.01 (−0.02, 0.00)**		
**Random**					**−0.01 (−0.02, 0.00)**						**−0.01 (−0.02, 0.00)**		

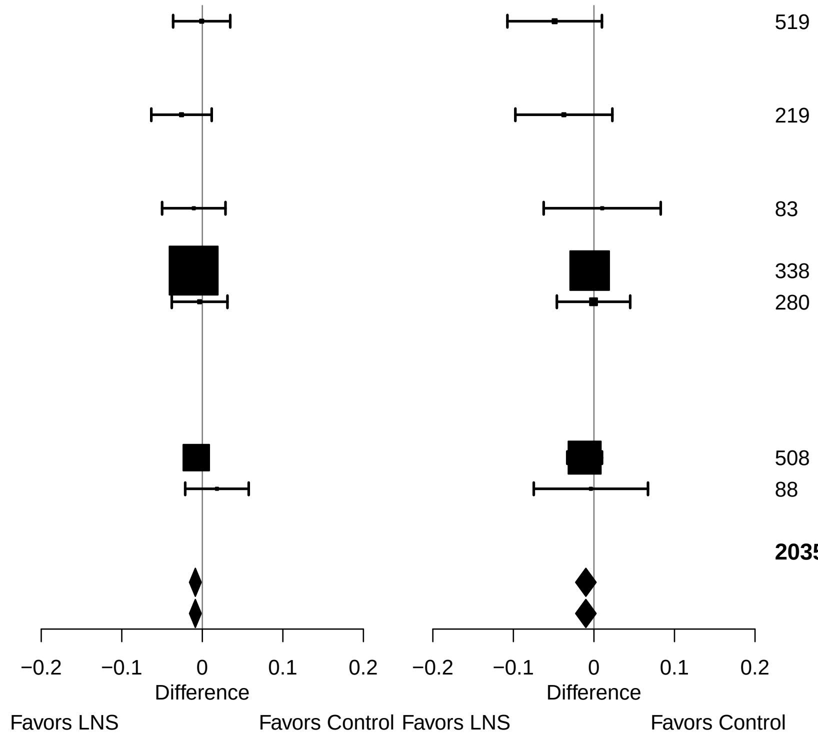

Supplemental figure 8K: Acute malnutrition prevalence difference

8K6: Stratified by Child sex

P−for−interaction = 0.708
Difference in PDs = 0.00 (−0.01, 0.01)

Country	Trial	Male LNS N	Male Control N	Male Control Prevalence	Male PD (95% CI)	Male Fixed W	Male Random W	Female LNS N	Female Control N	Female Control Prevalence	Female PD (95% CI)	Female Fixed W	Female Random W
Bangladesh	JiVitA−4 (34)	1420	586	17.8	0.00 (−0.03, 0.03)	0.07	0.07	1363	630	24.1	−0.04 (−0.08, −0.01)	0.05	0.10
Bangladesh	RDNS (35)	832	411	15.6	−0.02 (−0.06, 0.02)	0.04	0.04	829	404	15.1	−0.03 (−0.07, 0.01)	0.03	0.08
Bangladesh	WASH−B (36)												
Burkina Faso	iLiNS−Zinc (37)	990	336	17.3	−0.04 (−0.07, −0.01)	0.07	0.07	961	328	21.3	−0.08 (−0.14, −0.03)	0.02	0.05
Burkina Faso	PROMIS (38)	427	477	13.0	−0.03 (−0.09, 0.02)	0.03	0.03	419	424	10.1	0.00 (−0.05, 0.04)	0.03	0.07
Burkina Faso	PROMIS CS (38)	229	219	17.8	0.00 (−0.07, 0.08)	0.01	0.01	195	215	20.9	−0.02 (−0.10, 0.06)	0.01	0.03
Ghana	GHANA (39)												
Ghana	iLiNS−DYADG (40)	175	323	10.5	−0.04 (−0.10, 0.01)	0.03	0.03	172	369	6.0	0.02 (−0.03, 0.06)	0.03	0.07
Haiti	HAITI (41)												
Kenya	WASH−B (42)	732	2430	3.1	−0.01 (−0.02, 0.01)	0.37	0.37	722	2687	3.3	−0.01 (−0.03, 0.00)	0.43	0.20
Madagascar	MAHAY (43)	812	814	10.3	−0.01 (−0.05, 0.02)	0.06	0.06	863	841	8.6	0.01 (−0.02, 0.04)	0.08	0.12
Malawi	iLiNS−DYADM (44)												
Malawi	iLiNS−DOSE (45)												
Mali	PROMIS (46)												
Mali	PROMIS CS (46)	472	520	13.3	−0.01 (−0.06, 0.03)	0.03	0.03	472	439	12.8	−0.02 (−0.06, 0.02)	0.04	0.08
Zimbabwe	SHINE (HIV−) (47)	924	900	3.9	−0.01 (−0.03, 0.01)	0.28	0.28	943	877	2.9	0.00 (−0.02, 0.01)	0.28	0.18
Zimbabwe	SHINE (HIV+) (48)												
		**7013**	**7016**		**I² = 0.00, Tau² = 0.00**			**6939**	**7214**		**I² = 0.41, Tau² = 0.00**		
**Fixed**					**−0.01 (−0.02, 0.00)**						**−0.01 (−0.02, 0.00)**		
**Random**					**−0.01 (−0.02, 0.00)**						**−0.01 (−0.03, 0.00)**		

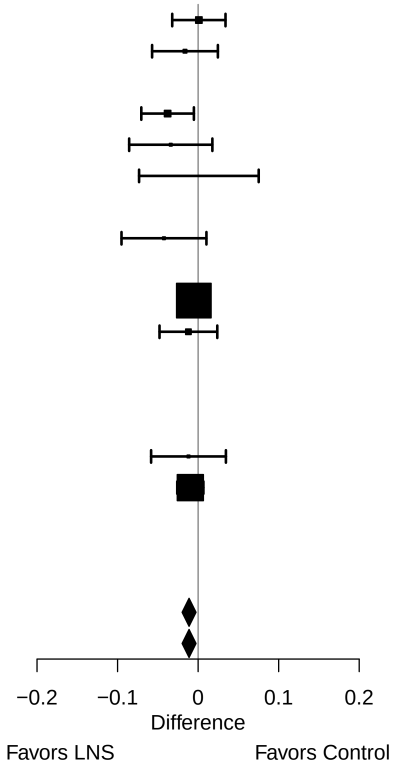

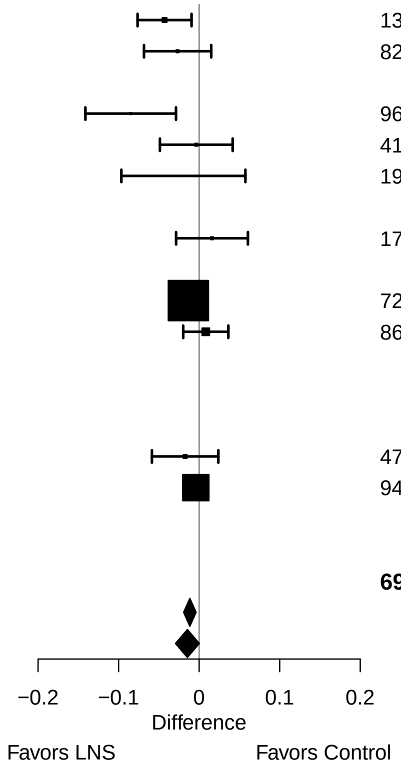

Supplemental figure 8K: Acute malnutrition prevalence difference

8K7: Stratified by Child birth order

P−for−interaction = 0.221
Difference in PDs = 0.01 (−0.01, 0.02)

Country	Trial	Later born LNS N	Later born Control N	Later born Control Prevalence	Later born PD (95% CI)	Later born Fixed W	Later born Random W	Firstborn LNS N	Firstborn Control N	Firstborn Control Prevalence	Firstborn PD (95% CI)	Firstborn Fixed W	Firstborn Random W
Bangladesh	JiVitA−4 (34)	598	247	23.1	−0.04 (−0.10, 0.01)	0.02	0.04	2181	963	20.4	−0.02 (−0.04, 0.01)	0.15	0.15
Bangladesh	RDNS (35)	972	508	16.1	−0.02 (−0.06, 0.01)	0.04	0.07	688	307	14.0	−0.02 (−0.06, 0.03)	0.05	0.05
Bangladesh	WASH−B (36)												
Burkina Faso	iLiNS−Zinc (37)	1520	519	18.5	−0.06 (−0.09, −0.03)	0.05	0.08	431	145	22.1	−0.06 (−0.14, 0.01)	0.02	0.02
Burkina Faso	PROMIS (38)	701	743	12.0	−0.02 (−0.06, 0.02)	0.04	0.08	145	157	10.2	−0.02 (−0.08, 0.04)	0.03	0.03
Burkina Faso	PROMIS CS (38)	362	355	19.7	−0.02 (−0.07, 0.03)	0.02	0.04	61	79	17.7	0.05 (−0.08, 0.19)	0.01	0.01
Ghana	GHANA (39)												
Ghana	iLiNS−DYADG (40)												
Haiti	HAITI (41)												
Kenya	WASH−B (42)	1131	4026	3.4	−0.01 (−0.02, 0.00)	0.47	0.28	321	1086	2.6	0.00 (−0.02, 0.01)	0.36	0.36
Madagascar	MAHAY (43)	1259	1196	10.4	−0.01 (−0.04, 0.02)	0.06	0.10	416	459	7.0	0.01 (−0.02, 0.04)	0.11	0.11
Malawi	iLiNS−DYADM (44)												
Malawi	iLiNS−DOSE (45)												
Mali	PROMIS (46)												
Mali	PROMIS CS (46)	783	821	13.0	−0.01 (−0.04, 0.02)	0.05	0.09	144	126	11.1	−0.03 (−0.10, 0.05)	0.02	0.02
Zimbabwe	SHINE (HIV−) (47)	1294	1211	3.5	−0.01 (−0.02, 0.00)	0.26	0.23	486	483	3.5	0.00 (−0.02, 0.02)	0.25	0.25
Zimbabwe	SHINE (HIV+) (48)												
		**8620**	**9626**		**I² = 0.21, Tau² = 0.00**			**4873**	**3805**		**I² = 0.00, Tau² = 0.00**		
**Fixed**					**−0.01 (−0.02, −0.01)**						**−0.01 (−0.02, 0.00)**		
**Random**					**−0.02 (−0.03, −0.01)**						**−0.01 (−0.02, 0.00)**		

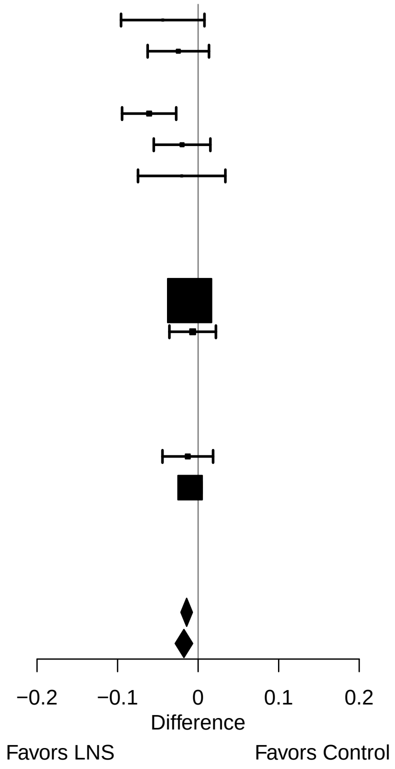

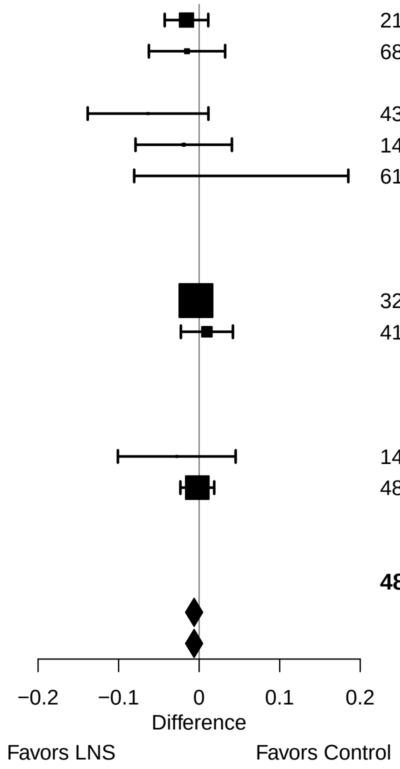

Supplemental figure 8K: Acute malnutrition prevalence difference

8K8: Stratified by Child baseline anthropometric status

P−for−interaction = 0.010
Difference in PDs = −0.03 (−0.05, −0.01)

		WLZ at least 0 Z						WLZ less than 0 Z					
**Country**	**Trial**	**LNS N**	**Control N**	**Control Prevalence**	**PD (95% CI)**	**Fixed W**	**Random W**	**LNS N**	**Control N**	**Control Prevalence**	**PD (95% CI)**	**Fixed W**	**Random W**
Bangladesh	JiVitA−4 (34)	961	423	4.0	0.00 (−0.03, 0.02)	0.15	0.15	1822	793	30.0	−0.04 (−0.07, 0.00)	0.34	0.34
Bangladesh	RDNS (35)												
Bangladesh	WASH−B (36)												
Burkina Faso	iLiNS−Zinc (37)												
Burkina Faso	PROMIS (38)	275	346	3.5	−0.01 (−0.04, 0.02)	0.08	0.08	408	392	18.6	−0.04 (−0.09, 0.01)	0.16	0.16
Burkina Faso	PROMIS CS (38)												
Ghana	GHANA (39)												
Ghana	iLiNS−DYADG (40)												
Haiti	HAITI (41)												
Kenya	WASH−B (42)												
Madagascar	MAHAY (43)												
Malawi	iLiNS−DYADM (44)												
Malawi	iLiNS−DOSE (45)												
Mali	PROMIS (46)												
Mali	PROMIS CS (46)												
Zimbabwe	SHINE (HIV−) (47)	742	699	1.1	0.00 (−0.01, 0.01)	0.77	0.77	555	476	6.5	−0.03 (−0.05, 0.00)	0.50	0.50
Zimbabwe	SHINE (HIV+) (48)												
		**1978**	**1468**		**I² = 0.00, Tau² = 0.00**			**2785**	**1661**		**I² = 0.00, Tau² = 0.00**		
**Fixed**					**0.00 (−0.01, 0.01)**						**−0.03 (−0.05, −0.01)**		
**Random**					**0.00 (−0.01, 0.01)**						**−0.03 (−0.05, −0.01)**		

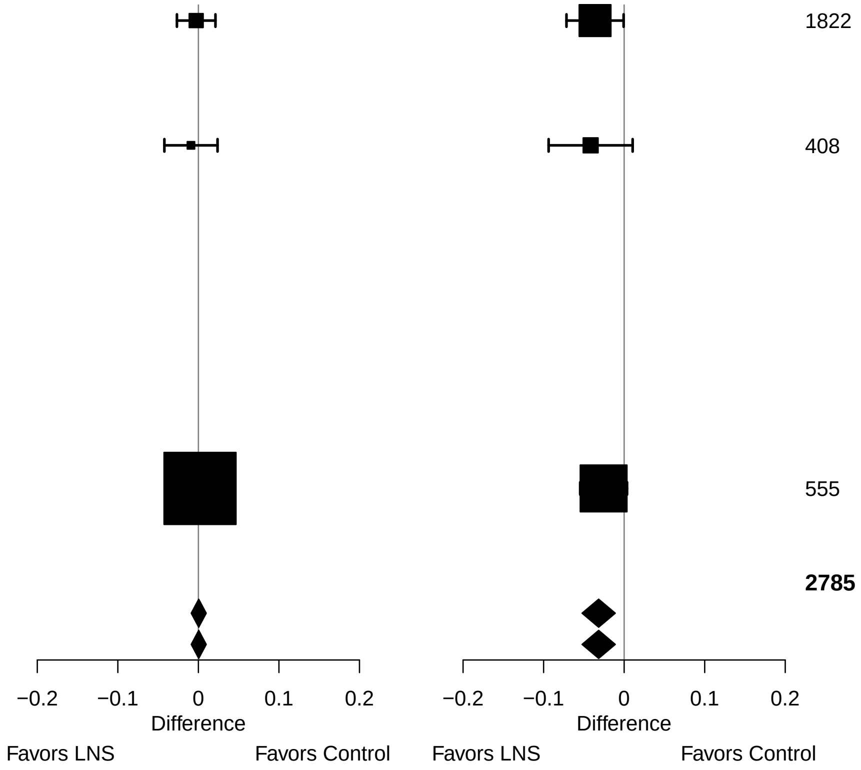

Supplemental figure 8L: Mean difference in WAZ

8L1: Stratified by Maternal height

P−for−interaction = 0.234
Difference in MDs = −0.04 (−0.11, 0.03)

		At least 150.1 cm						Less than 150.1 cm					
**Country**	**Trial**	**LNS N**	**Control N**	**Control Mean**	**MD (95% CI)**	**Fixed W**	**Random W**	**LNS N**	**Control N**	**Control Mean**	**MD (95% CI)**	**Fixed W**	**Random W**
Bangladesh	JiVitA−4 (34)												
Bangladesh	RDNS (35)	868	432	−1.58	0.13 (0.02, 0.24)	0.06	0.07	735	358	−1.97	0.09 (−0.02, 0.21)	0.26	0.26
Bangladesh	WASH−B (36)	620	1898	−1.37	0.21 (0.12, 0.30)	0.09	0.08	543	1557	−1.78	0.19 (0.10, 0.29)	0.39	0.39
Burkina Faso	iLiNS−Zinc (37)	1924	651	−1.53	0.28 (0.21, 0.35)	0.16	0.09	34	15	−1.84	0.00 (−0.40, 0.40)	0.02	0.02
Burkina Faso	PROMIS (38)	829	881	−1.20	0.15 (0.03, 0.28)	0.05	0.07	25	25	−1.86	0.04 (−0.43, 0.51)	0.02	0.02
Burkina Faso	PROMIS CS (38)	419	423	−1.24	−0.05 (−0.19, 0.10)	0.03	0.06	11	9	−2.14	0.05 (−0.92, 1.02)	0.00	0.00
Ghana	GHANA (39)												
Ghana	iLiNS−DYADG (40)	326	646	−0.83	0.16 (0.02, 0.30)	0.04	0.06	16	36	−1.26	0.10 (−0.46, 0.66)	0.01	0.01
Haiti	HAITI (41)												
Kenya	WASH−B (42)	1316	4709	−0.72	0.13 (0.06, 0.20)	0.15	0.09	57	195	−1.17	0.09 (−0.23, 0.41)	0.04	0.04
Madagascar	MAHAY (43)	1003	1040	−1.33	0.02 (−0.10, 0.14)	0.05	0.07	598	565	−1.67	0.04 (−0.13, 0.20)	0.13	0.13
Malawi	iLiNS−DYADM (44)	192	381	−0.82	−0.05 (−0.23, 0.12)	0.02	0.05	28	60	−1.21	−0.24 (−0.72, 0.23)	0.02	0.02
Malawi	iLiNS−DOSE (45)	586	198	−0.99	0.03 (−0.09, 0.14)	0.06	0.07	115	42	−1.51	0.20 (−0.07, 0.47)	0.05	0.05
Mali	PROMIS (46)	475	492	−1.14	0.18 (0.06, 0.31)	0.05	0.07	19	9	−1.32	−0.29 (−0.83, 0.25)	0.01	0.01
Mali	PROMIS CS (46)	909	928	−1.33	0.25 (0.13, 0.38)	0.05	0.07	32	26	−1.75	−0.17 (−0.69, 0.35)	0.01	0.01
Zimbabwe	SHINE (HIV−) (47)	1745	1690	−0.75	0.15 (0.08, 0.22)	0.16	0.09	75	62	−1.04	−0.04 (−0.33, 0.25)	0.04	0.04
Zimbabwe	SHINE (HIV+) (48)	313	311	−0.92	0.00 (−0.18, 0.17)	0.02	0.05	18	12	−1.60	0.33 (−0.49, 1.15)	0.01	0.01
		**11525**	**14680**		**I^2 = 0.70, Tau^2 = 0.01**			**2306**	**2971**		**I^2 = 0.00, Tau^2 = 0.00**		
**Fixed**					**0.15 (0.12, 0.18)**						**0.11 (0.05, 0.17)**		
**Random**					**0.13 (0.07, 0.18)**						**0.11 (0.05, 0.17)**		

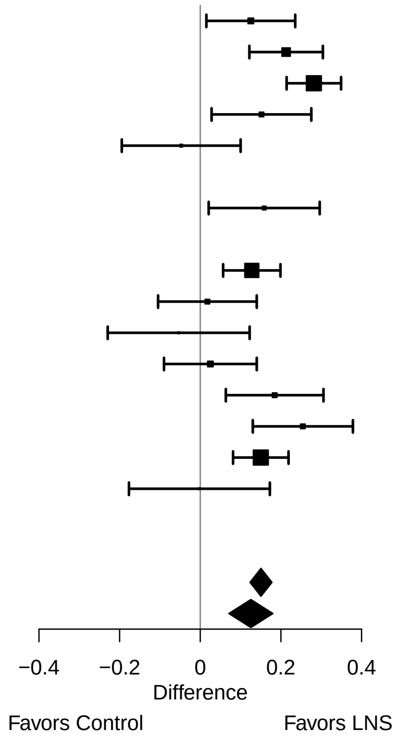

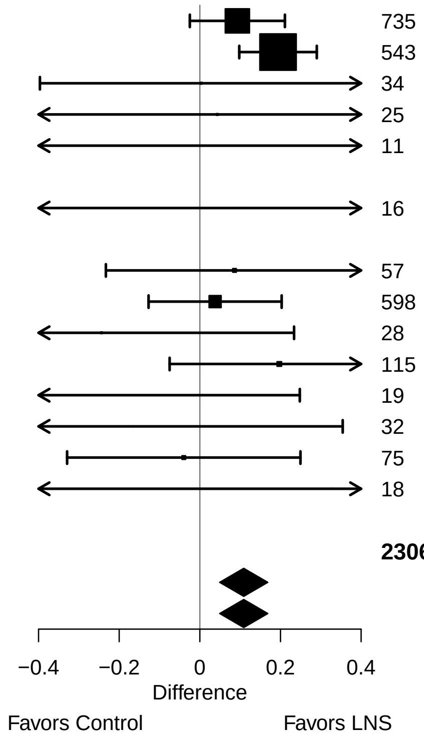

Supplemental figure 8L: Mean difference in WAZ

8L2: Stratified by Maternal BMI

P−for−interaction = 0.999
Difference in MDs = 0.00 (−0.05, 0.05)

Country	Trial	At least 20 kg/m² LNS N	Control N	Control Mean	MD (95% CI)	Fixed W	Random W	Less than 20 kg/m² LNS N	Control N	Control Mean	MD (95% CI)	Fixed W	Random W
Bangladesh	JiVitA−4 (34)												
Bangladesh	RDNS (35)	730	335	−1.53	0.08 (−0.04, 0.19)	0.08	0.08	873	455	−1.92	0.11 (0.01, 0.22)	0.13	0.12
Bangladesh	WASH−B (36)	518	1595	−1.34	0.22 (0.10, 0.34)	0.07	0.08	645	1860	−1.74	0.19 (0.12, 0.27)	0.26	0.15
Burkina Faso	iLiNS−Zinc (37)	1218	391	−1.39	0.28 (0.20, 0.37)	0.13	0.10	740	275	−1.74	0.26 (0.18, 0.34)	0.20	0.14
Burkina Faso	PROMIS (38)	645	673	−1.15	0.19 (0.06, 0.32)	0.06	0.08	209	232	−1.42	−0.01 (−0.19, 0.17)	0.04	0.07
Burkina Faso	PROMIS CS (38)	246	267	−1.09	−0.08 (−0.24, 0.07)	0.04	0.07	184	165	−1.54	0.05 (−0.12, 0.21)	0.05	0.07
Ghana	GHANA (39)												
Ghana	iLiNS−DYADG (40)	305	563	−0.80	0.13 (−0.02, 0.27)	0.05	0.07	37	119	−1.06	0.24 (−0.12, 0.60)	0.01	0.02
Haiti	HAITI (41)												
Kenya	WASH−B (42)	1100	3822	−0.66	0.08 (0.01, 0.16)	0.18	0.10	273	1082	−1.02	0.26 (0.12, 0.39)	0.08	0.09
Madagascar	MAHAY (43)												
Malawi	iLiNS−DYADM (44)	131	263	−0.75	−0.06 (−0.28, 0.16)	0.02	0.04	88	178	−1.05	−0.08 (−0.33, 0.17)	0.02	0.04
Malawi	iLiNS−DOSE (45)	519	174	−0.96	0.05 (−0.07, 0.18)	0.06	0.08	180	65	−1.43	0.05 (−0.15, 0.25)	0.03	0.06
Mali	PROMIS (46)	377	368	−1.05	0.17 (0.04, 0.30)	0.06	0.07	117	133	−1.41	0.14 (−0.02, 0.30)	0.06	0.08
Mali	PROMIS CS (46)	697	676	−1.25	0.23 (0.09, 0.37)	0.05	0.07	244	278	−1.57	0.22 (0.05, 0.39)	0.05	0.07
Zimbabwe	SHINE (HIV−) (47)	1355	1300	−0.73	0.17 (0.09, 0.24)	0.18	0.10	239	249	−1.02	0.12 (−0.05, 0.30)	0.04	0.07
Zimbabwe	SHINE (HIV+) (48)	246	253	−0.87	0.08 (−0.11, 0.27)	0.03	0.05	62	52	−1.34	−0.05 (−0.40, 0.31)	0.01	0.02
		**8087**	**10680**		**I² = 0.62, Tau² = 0.01**			**3891**	**5143**		**I² = 0.44, Tau² = 0.00**		
**Fixed**					**0.14 (0.11, 0.17)**						**0.17 (0.13, 0.20)**		
**Random**					**0.13 (0.08, 0.19)**						**0.14 (0.09, 0.20)**		

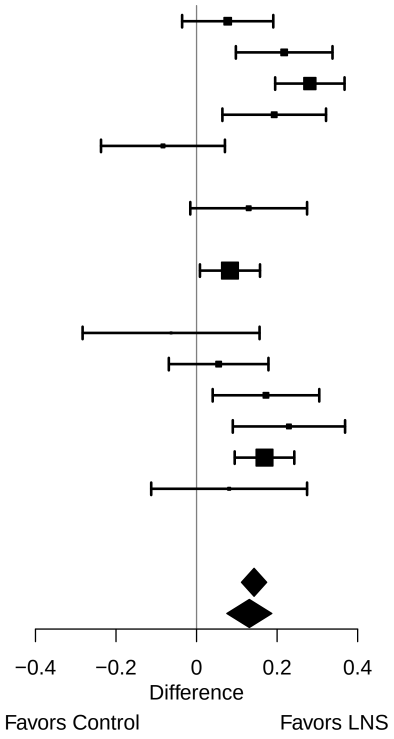

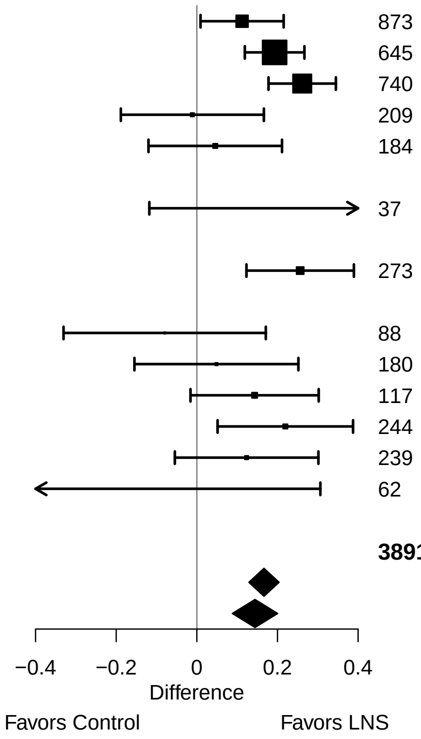

Supplemental figure 8L: Mean difference in WAZ

8L3: Stratified by Maternal age

P−for−interaction = 0.880
Difference in MDs = 0.00 (−0.04, 0.04)

		At least 25 y							Less than 25 y					
**Country**	**Trial**	**LNS N**	**Control N**	**Control Mean**	**MD (95% CI)**	**Fixed W**	**Random W**	**LNS N**	**Control N**	**Control Mean**	**MD (95% CI)**	**Fixed W**	**Random W**
Bangladesh	JiVitA−4 (34)	1204	540	−1.79	0.10 (0.05, 0.16)	0.25	0.12	1597	687	−1.75	0.11 (0.06, 0.15)	0.33	0.10
Bangladesh	RDNS (35)	453	221	−1.74	0.11 (−0.04, 0.27)	0.03	0.05	1210	594	−1.75	0.09 (0.01, 0.17)	0.11	0.09
Bangladesh	WASH−B (36)	493	1521	−1.60	0.13 (0.03, 0.23)	0.08	0.08	665	1924	−1.52	0.24 (0.15, 0.34)	0.09	0.08
Burkina Faso	iLiNS−Zinc (37)	1134	401	−1.51	0.28 (0.19, 0.37)	0.10	0.09	813	262	−1.55	0.26 (0.14, 0.38)	0.05	0.07
Burkina Faso	PROMIS (38)	480	524	−1.24	0.18 (0.06, 0.30)	0.05	0.07	377	387	−1.20	0.11 (−0.08, 0.29)	0.02	0.05
Burkina Faso	PROMIS CS (38)	202	190	−1.28	−0.02 (−0.21, 0.17)	0.02	0.04	228	246	−1.26	−0.07 (−0.24, 0.11)	0.02	0.05
Ghana	GHANA (39)	69	63	−0.68	0.40 (0.00, 0.81)	0.00	0.01	29	25	−0.59	−0.09 (−0.70, 0.52)	0.00	0.01
Ghana	iLiNS−DYADG (40)	220	417	−0.79	0.06 (−0.11, 0.24)	0.03	0.04	127	276	−0.94	0.31 (0.11, 0.52)	0.02	0.04
Haiti	HAITI (41)	100	104	−0.37	0.08 (−0.07, 0.22)	0.04	0.05	47	45	−0.41	0.04 (−0.20, 0.28)	0.01	0.04
Kenya	WASH−B (42)	845	2827	−0.71	0.14 (0.06, 0.22)	0.12	0.10	601	2306	−0.80	0.13 (0.04, 0.21)	0.11	0.09
Madagascar	MAHAY (43)	895	952	−1.50	0.04 (−0.09, 0.18)	0.04	0.06	806	733	−1.37	−0.03 (−0.17, 0.10)	0.04	0.07
Malawi	iLiNS−DYADM (44)	106	226	−0.87	−0.04 (−0.28, 0.20)	0.01	0.02	114	218	−0.89	−0.09 (−0.33, 0.14)	0.01	0.04
Malawi	iLiNS−DOSE (45)	372	134	−1.10	0.01 (−0.13, 0.14)	0.04	0.06	316	100	−1.07	0.09 (−0.08, 0.25)	0.03	0.06
Mali	PROMIS (46)	342	355	−1.11	0.15 (0.01, 0.29)	0.04	0.06	158	147	−1.23	0.21 (0.03, 0.39)	0.02	0.05
Mali	PROMIS CS (46)	498	502	−1.30	0.20 (0.05, 0.35)	0.03	0.05	448	458	−1.38	0.26 (0.11, 0.42)	0.03	0.06
Zimbabwe	SHINE (HIV−) (47)	870	797	−0.75	0.19 (0.09, 0.28)	0.08	0.08	802	793	−0.76	0.09 (0.00, 0.19)	0.08	0.08
Zimbabwe	SHINE (HIV+) (48)	243	231	−0.97	0.04 (−0.17, 0.25)	0.02	0.03	73	77	−0.82	−0.11 (−0.46, 0.25)	0.01	0.02
		**8526**	**10005**		**I² = 0.41, Tau² = 0.00**			**8411**	**9278**		**I² = 0.55, Tau² = 0.01**		
**Fixed**					**0.13 (0.10, 0.16)**						**0.12 (0.09, 0.15)**		
**Random**					**0.12 (0.08, 0.17)**						**0.12 (0.06, 0.17)**		

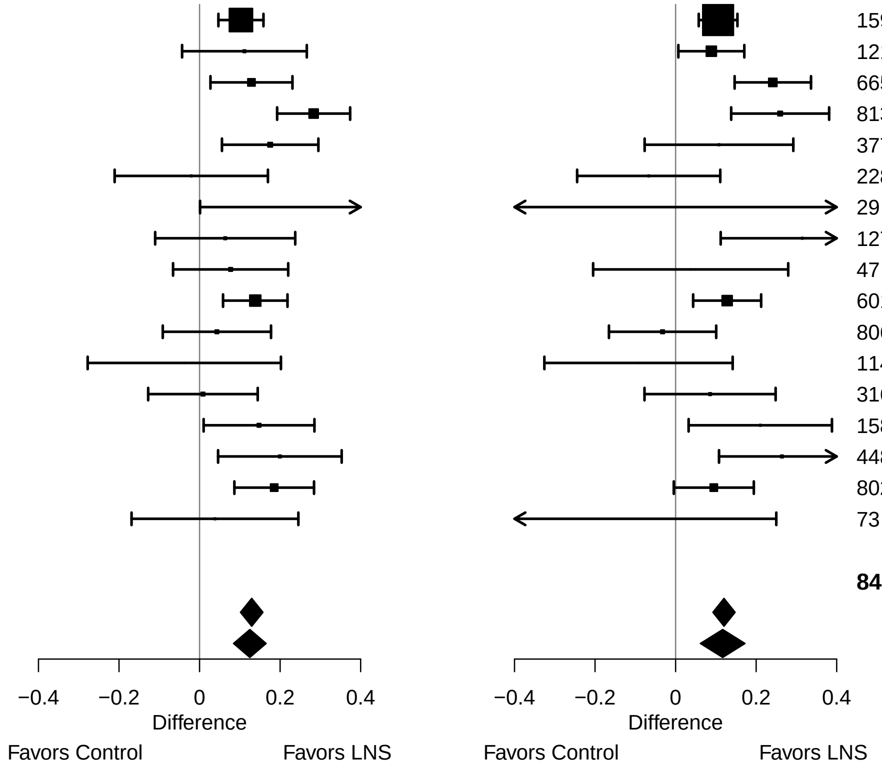

Supplemental figure 8L: Mean difference in WAZ

8L4: Stratified by Maternal education

P−for−interaction = 0.600
Difference in MDs = 0.01 (−0.03, 0.06)

		Primary or greater							Incomplete or no formal					
**Country**	**Trial**	**LNS N**	**Control N**	**Control Mean**	**MD (95% CI)**	**Fixed W**	**Random W**	**LNS N**	**Control N**	**Control Mean**	**MD (95% CI)**	**Fixed W**	**Random W**
Bangladesh	JiVitA−4 (34)	1816	749	−1.67	0.10 (0.06, 0.15)	0.36	0.22	996	480	−1.93	0.10 (0.04, 0.16)	0.24	0.11
Bangladesh	RDNS (35)	1245	589	−1.72	0.12 (0.03, 0.22)	0.08	0.10	418	226	−1.81	0.00 (−0.16, 0.15)	0.03	0.06
Bangladesh	WASH−B (36)	818	2476	−1.45	0.21 (0.14, 0.29)	0.12	0.13	345	985	−1.83	0.18 (0.06, 0.29)	0.06	0.08
Burkina Faso	iLiNS−Zinc (37)	82	16	−1.16	0.17 (−0.20, 0.54)	0.01	0.01	1865	647	−1.54	0.28 (0.21, 0.35)	0.18	0.10
Burkina Faso	PROMIS (38)	70	54	−1.23	0.03 (−0.22, 0.28)	0.01	0.02	787	857	−1.22	0.16 (0.03, 0.28)	0.05	0.07
Burkina Faso	PROMIS CS (38)	34	39	−1.24	−0.10 (−0.63, 0.42)	0.00	0.00	393	396	−1.27	−0.04 (−0.18, 0.09)	0.04	0.07
Ghana	GHANA (39)	91	75	−0.69	0.31 (−0.05, 0.68)	0.01	0.01	7	15	−0.47	−0.23 (−1.22, 0.76)	0.00	0.00
Ghana	iLiNS−DYADG (40)	267	549	−0.83	0.13 (−0.03, 0.28)	0.03	0.05	80	144	−0.94	0.28 (0.01, 0.55)	0.01	0.03
Haiti	HAITI (41)	129	126	−0.29	0.08 (−0.05, 0.21)	0.04	0.06	19	24	−0.82	−0.08 (−0.44, 0.27)	0.01	0.02
Kenya	WASH−B (42)	705	2451	−0.63	0.07 (−0.01, 0.16)	0.10	0.11	754	2710	−0.86	0.19 (0.11, 0.26)	0.13	0.10
Madagascar	MAHAY (43)	352	431	−1.25	0.01 (−0.16, 0.18)	0.03	0.04	1350	1254	−1.51	0.03 (−0.09, 0.15)	0.05	0.07
Malawi	iLiNS−DYADM (44)	35	69	−0.66	−0.20 (−0.60, 0.21)	0.00	0.01	184	372	−0.92	−0.05 (−0.23, 0.14)	0.02	0.05
Malawi	iLiNS−DOSE (45)	165	56	−0.81	0.09 (−0.12, 0.29)	0.02	0.03	525	178	−1.17	0.04 (−0.08, 0.16)	0.06	0.07
Mali	PROMIS (46)	39	37	−0.91	−0.03 (−0.34, 0.28)	0.01	0.01	460	465	−1.16	0.18 (0.05, 0.31)	0.05	0.07
Mali	PROMIS CS (46)	107	96	−1.21	0.38 (0.10, 0.66)	0.01	0.02	838	864	−1.35	0.21 (0.08, 0.33)	0.05	0.07
Zimbabwe	SHINE (HIV−) (47)	1707	1636	−0.74	0.12 (0.05, 0.19)	0.14	0.14	68	61	−0.80	0.16 (−0.26, 0.59)	0.00	0.01
Zimbabwe	SHINE (HIV+) (48)	300	292	−0.91	−0.01 (−0.19, 0.17)	0.02	0.04	21	18	−1.20	0.05 (−0.61, 0.70)	0.00	0.01
		**7962**	**9741**		**I² = 0.18, Tau² = 0.00**			**9110**	**9696**		**I² = 0.64, Tau² = 0.01**		
**Fixed**					**0.11 (0.09, 0.14)**						**0.14 (0.11, 0.17)**		
**Random**					**0.11 (0.08, 0.15)**						**0.12 (0.07, 0.17)**		

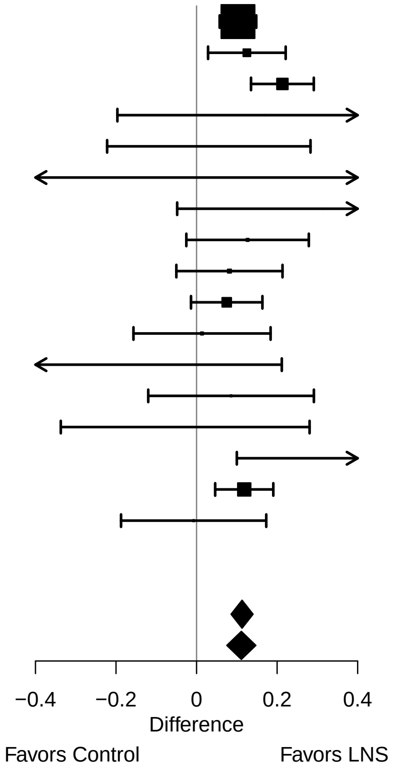

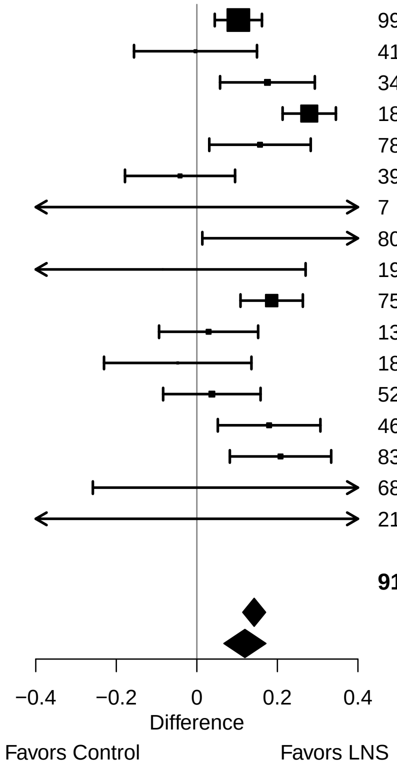

Supplemental figure 8L: Mean difference in WAZ

8L5: Stratified by Maternal depressive symptoms

P−for−interaction = 0.345
Difference in MDs = 0.03 (−0.03, 0.09)

		Less than 75th percentile						At least 75th percentile					
**Country**	**Trial**	**LNS N**	**Control N**	**Control Mean**	**MD (95% CI)**	**Fixed W**	**Random W**	**LNS N**	**Control N**	**Control Mean**	**MD (95% CI)**	**Fixed W**	**Random W**
Bangladesh	JiVitA−4 (34)												
Bangladesh	RDNS (35)	1081	472	−1.73	0.10 (0.00, 0.20)	0.13	0.13	520	288	−1.77	0.07 (−0.07, 0.21)	0.13	0.13
Bangladesh	WASH−B (36)	899	2487	−1.53	0.21 (0.13, 0.29)	0.18	0.15	240	883	−1.61	0.16 (0.03, 0.29)	0.16	0.14
Burkina Faso	iLiNS−Zinc (37)												
Burkina Faso	PROMIS (38)	505	571	−1.27	0.22 (0.08, 0.37)	0.06	0.07	220	213	−1.20	0.01 (−0.15, 0.16)	0.11	0.12
Burkina Faso	PROMIS CS (38)												
Ghana	GHANA (39)												
Ghana	iLiNS−DYADG (40)	252	460	−0.83	0.17 (0.01, 0.32)	0.05	0.07	83	209	−0.89	0.17 (−0.10, 0.44)	0.04	0.06
Haiti	HAITI (41)												
Kenya	WASH−B (42)	997	3506	−0.72	0.13 (0.06, 0.20)	0.23	0.17	342	1248	−0.79	0.09 (−0.04, 0.22)	0.16	0.14
Madagascar	MAHAY (43)	673	721	−1.47	0.08 (−0.05, 0.21)	0.07	0.09	283	252	−1.42	−0.15 (−0.34, 0.04)	0.07	0.09
Malawi	iLiNS−DYADM (44)	157	303	−0.80	−0.09 (−0.29, 0.11)	0.03	0.05	47	113	−1.05	0.05 (−0.30, 0.40)	0.02	0.04
Malawi	iLiNS−DOSE (45)												
Mali	PROMIS (46)	374	372	−1.10	0.14 (0.01, 0.27)	0.07	0.09	122	129	−1.28	0.25 (0.09, 0.42)	0.09	0.11
Mali	PROMIS CS (46)												
Zimbabwe	SHINE (HIV−) (47)	1192	1137	−0.73	0.13 (0.04, 0.21)	0.16	0.14	509	490	−0.85	0.22 (0.10, 0.34)	0.18	0.15
Zimbabwe	SHINE (HIV+) (48)	234	214	−0.90	−0.02 (−0.24, 0.20)	0.03	0.04	88	100	−1.08	0.05 (−0.26, 0.36)	0.03	0.05
		**6364**	**10243**		**I² = 0.29, Tau² = 0.00**			**2454**	**3925**		**I² = 0.46, Tau² = 0.01**		
**Fixed**					**0.13 (0.10, 0.17)**						**0.11 (0.06, 0.16)**		
**Random**					**0.13 (0.08, 0.17)**						**0.10 (0.03, 0.18)**		

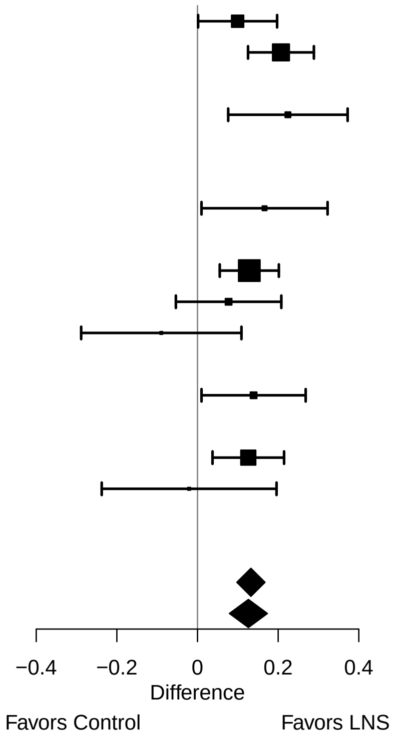

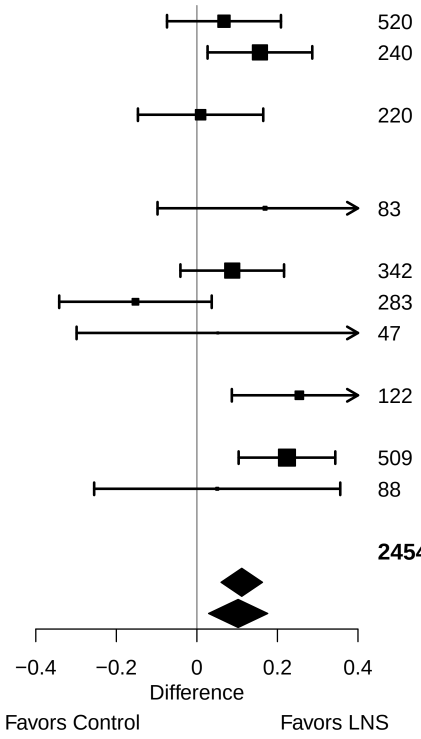

Supplemental figure 8L: Mean difference in WAZ

8L6: Stratified by Child sex

P−for−interaction = 0.292
Difference in MDs = 0.02 (−0.02, 0.06)

Male

Country	Trial	LNS N	Control N	Control Mean	MD (95% CI)	Fixed W	Random W
Bangladesh	JiVitA−4 (34)	1440	594	−1.74	0.09 (0.04, 0.15)	0.27	0.10
Bangladesh	RDNS (35)	833	411	−1.70	0.01 (−0.12, 0.13)	0.05	0.07
Bangladesh	WASH−B (36)	567	1730	−1.55	0.20 (0.11, 0.29)	0.10	0.08
Burkina Faso	iLiNS−Zinc (37)	995	336	−1.65	0.29 (0.18, 0.39)	0.08	0.08
Burkina Faso	PROMIS (38)	435	482	−1.35	0.19 (0.04, 0.33)	0.04	0.06
Burkina Faso	PROMIS CS (38)	234	221	−1.35	−0.09 (−0.28, 0.11)	0.02	0.04
Ghana	GHANA (39)	60	41	−0.72	0.22 (−0.21, 0.65)	0.00	0.01
Ghana	iLiNS−DYADG (40)	175	324	−0.94	0.15 (−0.05, 0.35)	0.02	0.04
Haiti	HAITI (41)	70	60	−0.39	0.02 (−0.17, 0.21)	0.02	0.04
Kenya	WASH−B (42)	734	2457	−0.83	0.11 (0.02, 0.20)	0.10	0.08
Madagascar	MAHAY (43)	826	835	−1.59	0.01 (−0.12, 0.14)	0.05	0.07
Malawi	iLiNS−DYADM (44)	107	207	−0.93	−0.09 (−0.34, 0.15)	0.01	0.03
Malawi	iLiNS−DOSE (45)	353	124	−1.29	−0.02 (−0.17, 0.13)	0.04	0.06
Mali	PROMIS (46)	265	262	−1.19	0.13 (0.00, 0.25)	0.05	0.07
Mali	PROMIS CS (46)	474	521	−1.40	0.20 (0.04, 0.37)	0.03	0.05
Zimbabwe	SHINE (HIV−) (47)	924	906	−0.88	0.19 (0.10, 0.27)	0.10	0.08
Zimbabwe	SHINE (HIV+) (48)	161	167	−1.09	0.02 (−0.21, 0.24)	0.02	0.03
		**8653**	**9678**		**I² = 0.56, Tau² = 0.01**		
**Fixed**					**0.12 (0.09, 0.15)**		
**Random**					**0.11 (0.06, 0.16)**		

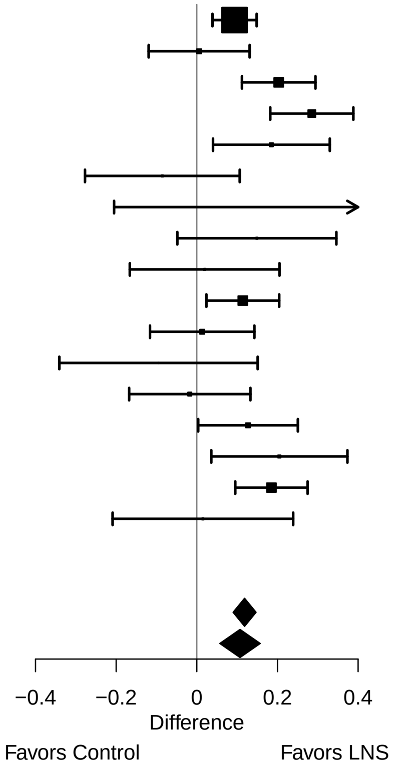

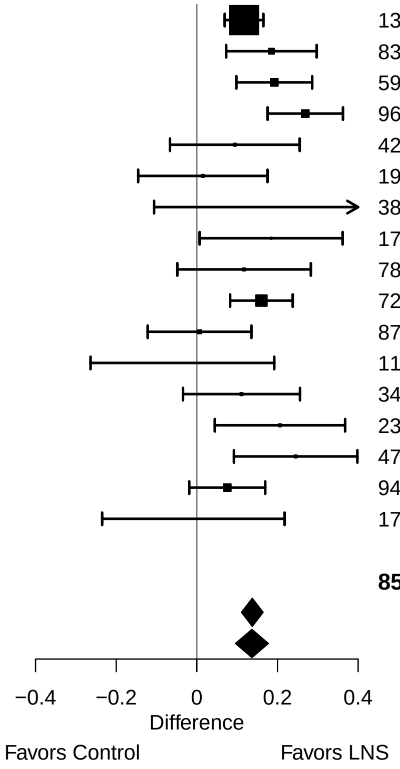

Female

LNS N	Control N	Control Mean	MD (95% CI)	Fixed W	Random W
1374	638	−1.80	0.12 (0.07, 0.17)	0.31	0.13
830	404	−1.79	0.18 (0.07, 0.30)	0.06	0.07
596	1731	−1.56	0.19 (0.10, 0.29)	0.08	0.09
965	330	−1.42	0.27 (0.18, 0.36)	0.08	0.09
422	429	−1.08	0.09 (−0.07, 0.26)	0.03	0.05
196	215	−1.18	0.01 (−0.15, 0.18)	0.03	0.05
38	55	−0.64	0.42 (−0.11, 0.94)	0.00	0.01
172	369	−0.78	0.18 (0.01, 0.36)	0.02	0.04
78	90	−0.37	0.12 (−0.05, 0.28)	0.03	0.04
727	2708	−0.68	0.16 (0.08, 0.24)	0.12	0.10
876	850	−1.30	0.01 (−0.12, 0.14)	0.04	0.06
113	237	−0.84	−0.04 (−0.26, 0.19)	0.01	0.03
349	117	−0.88	0.11 (−0.03, 0.26)	0.03	0.05
235	240	−1.09	0.21 (0.04, 0.37)	0.03	0.05
472	439	−1.27	0.25 (0.09, 0.40)	0.03	0.05
945	880	−0.62	0.08 (−0.02, 0.17)	0.08	0.09
175	161	−0.80	−0.01 (−0.23, 0.22)	0.01	0.03
**8563**	**9893**		**I² = 0.40, Tau² = 0.00**		
			**0.14 (0.11, 0.16)**		
			**0.14 (0.10, 0.18)**		

Supplemental figure 8L: Mean difference in WAZ

8L7: Stratified by Child birth order

P−for−interaction = 0.021
Difference in MDs = −0.05 (−0.09, −0.01)

Country	Trial	Later born LNS N	Later born Control N	Later born Control Mean	Later born MD (95% CI)	Later born Fixed W	Later born Random W	Firstborn LNS N	Firstborn Control N	Firstborn Control Mean	Firstborn MD (95% CI)	Firstborn Fixed W	Firstborn Random W
Bangladesh	JiVitA−4 (34)	600	250	−1.89	0.16 (0.09, 0.24)	0.12	0.08	2210	976	−1.74	0.09 (0.05, 0.13)	0.55	0.10
Bangladesh	RDNS (35)	974	508	−1.77	0.11 (0.01, 0.22)	0.06	0.07	688	307	−1.71	0.06 (−0.06, 0.18)	0.06	0.08
Bangladesh	WASH−B (36)	737	2310	−1.61	0.19 (0.11, 0.26)	0.11	0.08	426	1151	−1.44	0.20 (0.08, 0.32)	0.06	0.08
Burkina Faso	iLiNS−Zinc (37)	1524	521	−1.52	0.28 (0.21, 0.35)	0.14	0.08	436	145	−1.60	0.27 (0.14, 0.40)	0.05	0.08
Burkina Faso	PROMIS (38)	709	750	−1.22	0.17 (0.05, 0.30)	0.05	0.06	148	159	−1.22	0.01 (−0.21, 0.24)	0.02	0.05
Burkina Faso	PROMIS CS (38)	366	357	−1.25	−0.07 (−0.21, 0.07)	0.03	0.05	63	79	−1.36	0.04 (−0.28, 0.37)	0.01	0.03
Ghana	GHANA (39)	60	54	−0.67	0.39 (−0.07, 0.85)	0.00	0.01	38	38	−0.62	0.04 (−0.44, 0.51)	0.00	0.02
Ghana	iLiNS−DYADG (40)	237	459	−0.83	0.07 (−0.09, 0.23)	0.03	0.05	110	234	−0.89	0.35 (0.11, 0.58)	0.02	0.05
Haiti	HAITI (41)	88	100	−0.47	0.14 (−0.02, 0.30)	0.03	0.05	60	50	−0.19	−0.09 (−0.29, 0.10)	0.02	0.06
Kenya	WASH−B (42)	1137	4065	−0.75	0.12 (0.05, 0.20)	0.11	0.08	322	1095	−0.73	0.17 (0.05, 0.29)	0.06	0.08
Madagascar	MAHAY (43)	1271	1220	−1.49	0.06 (−0.06, 0.18)	0.05	0.06	431	465	−1.33	−0.10 (−0.27, 0.07)	0.03	0.07
Malawi	iLiNS−DYADM (44)	173	353	−0.87	−0.07 (−0.26, 0.12)	0.02	0.04	47	89	−0.89	−0.06 (−0.44, 0.31)	0.01	0.03
Malawi	iLiNS−DOSE (45)	463	165	−1.13	0.06 (−0.06, 0.18)	0.04	0.06	150	45	−1.01	0.04 (−0.21, 0.28)	0.01	0.05
Mali	PROMIS (46)	437	441	−1.14	0.19 (0.07, 0.32)	0.04	0.06	62	59	−1.15	−0.07 (−0.26, 0.13)	0.02	0.06
Mali	PROMIS CS (46)	784	822	−1.31	0.22 (0.10, 0.35)	0.04	0.06	145	126	−1.46	0.35 (0.14, 0.56)	0.02	0.06
Zimbabwe	SHINE (HIV−) (47)	1295	1220	−0.77	0.21 (0.13, 0.28)	0.11	0.08	487	483	−0.71	−0.02 (−0.14, 0.11)	0.05	0.08
Zimbabwe	SHINE (HIV+) (48)	279	245	−0.84	−0.11 (−0.31, 0.09)	0.02	0.04	54	78	−1.25	0.40 (0.11, 0.69)	0.01	0.04
		**11134**	**13840**		**I² = 0.66, Tau² = 0.01**			**5877**	**5579**		**I² = 0.62, Tau² = 0.01**		
**Fixed**					**0.16 (0.13, 0.18)**						**0.10 (0.07, 0.13)**		
**Random**					**0.13 (0.08, 0.18)**						**0.10 (0.03, 0.18)**		

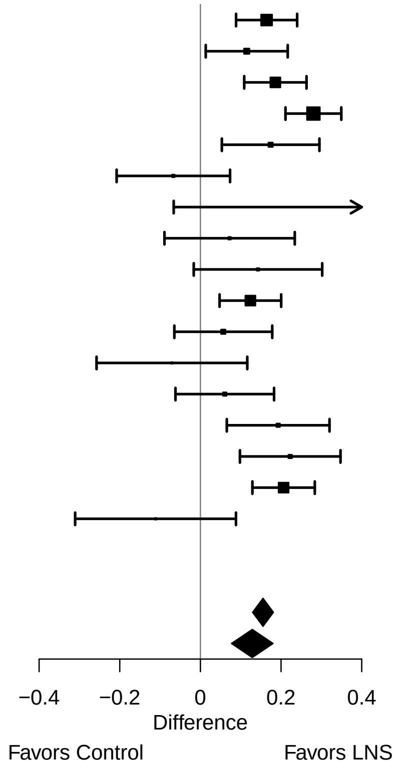

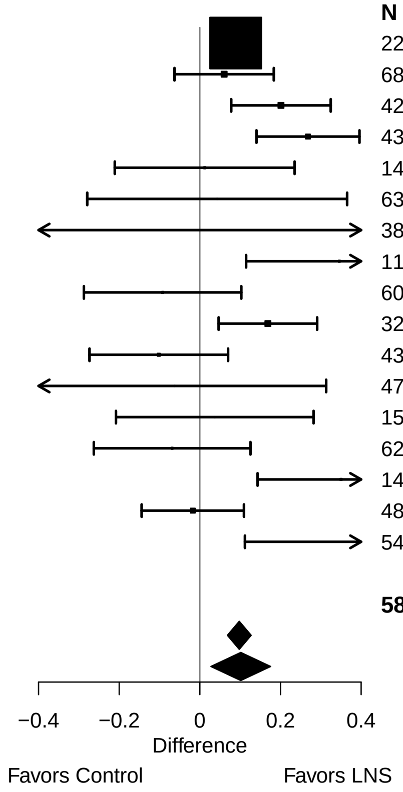

Supplemental figure 8L: Mean difference in WAZ

8L8: Stratified by Child baseline anthropometric status

P−for−interaction = 0.078
Difference in MDs = 0.05 (−0.01, 0.10)

WAZ at least −1 Z

Country	Trial	LNS N	Control N	Control Mean	MD (95% CI)	Fixed W	Random W
Bangladesh	JiVitA−4 (34)	1198	531	−1.05	0.14 (0.06, 0.21)	0.24	0.18
Bangladesh	RDNS (35)	780	338	−1.05	0.01 (−0.08, 0.10)	0.17	0.15
Bangladesh	WASH−B (36)						
Burkina Faso	iLiNS−Zinc (37)	718	235	−0.57	0.14 (−0.08, 0.37)	0.03	0.04
Burkina Faso	PROMIS (38)	427	482	−0.82	0.15 (0.03, 0.28)	0.09	0.10
Burkina Faso	PROMIS CS (38)						
Ghana	GHANA (39)						
Ghana	iLiNS−DYADG (40)	222	424	−0.40	0.13 (−0.01, 0.27)	0.07	0.08
Haiti	HAITI (41)	113	110	0.02	0.00 (−0.23, 0.23)	0.03	0.04
Kenya	WASH−B (42)						
Madagascar	MAHAY (43)						
Malawi	iLiNS−DYADM (44)	129	292	−0.44	−0.10 (−0.27, 0.07)	0.05	0.06
Malawi	iLiNS−DOSE (45)	419	143	−0.50	−0.03 (−0.19, 0.14)	0.05	0.06
Mali	PROMIS (46)	279	243	−0.65	0.12 (−0.01, 0.26)	0.07	0.09
Mali	PROMIS CS (46)						
Zimbabwe	SHINE (HIV−) (47)	917	836	−0.37	0.09 (0.01, 0.18)	0.19	0.16
Zimbabwe	SHINE (HIV+) (48)	151	144	−0.42	0.06 (−0.16, 0.28)	0.03	0.04
		**5353**	**3778**		**I^2 = 0.27, Tau^2 = 0.00**		
**Fixed**					**0.08 (0.05, 0.12)**		
**Random**					**0.08 (0.03, 0.12)**		

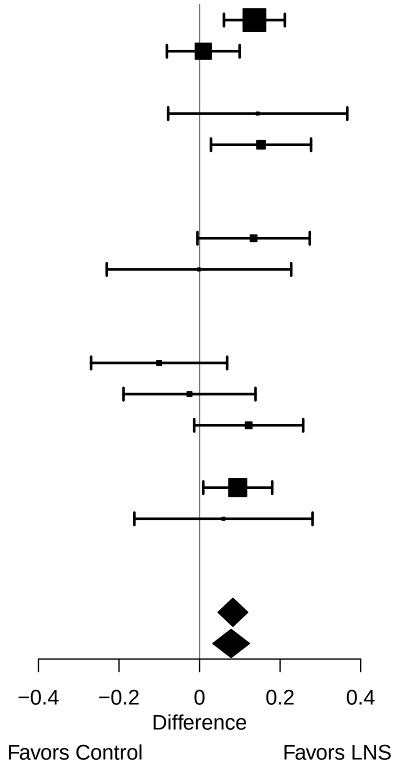

WAZ less than −1 Z

Country	Trial	LNS N	Control N	Control Mean	MD (95% CI)	Fixed W	Random W
Bangladesh	JiVitA−4 (34)	1616	701	−2.31	0.11 (0.05, 0.18)	0.33	0.18
Bangladesh	RDNS (35)	821	422	−2.29	0.07 (−0.01, 0.14)	0.22	0.16
Bangladesh	WASH−B (36)						
Burkina Faso	iLiNS−Zinc (37)	1242	431	−2.06	0.37 (0.25, 0.48)	0.11	0.12
Burkina Faso	PROMIS (38)	268	267	−1.92	0.14 (0.01, 0.27)	0.08	0.11
Burkina Faso	PROMIS CS (38)						
Ghana	GHANA (39)						
Ghana	iLiNS−DYADG (40)	101	215	−1.71	0.08 (−0.10, 0.25)	0.04	0.07
Haiti	HAITI (41)	35	40	−1.48	0.20 (−0.09, 0.48)	0.02	0.04
Kenya	WASH−B (42)						
Madagascar	MAHAY (43)						
Malawi	iLiNS−DYADM (44)	75	133	−1.86	0.22 (−0.03, 0.47)	0.02	0.04
Malawi	iLiNS−DOSE (45)	283	98	−1.95	0.08 (−0.13, 0.28)	0.03	0.06
Mali	PROMIS (46)	221	259	−1.61	0.21 (0.02, 0.39)	0.04	0.07
Mali	PROMIS CS (46)						
Zimbabwe	SHINE (HIV−) (47)	383	349	−1.57	0.18 (0.05, 0.31)	0.08	0.11
Zimbabwe	SHINE (HIV+) (48)	113	92	−1.68	0.07 (−0.17, 0.32)	0.02	0.04
		**5158**	**3007**		**I^2 = 0.55, Tau^2 = 0.00**		
**Fixed**					**0.14 (0.10, 0.18)**		
**Random**					**0.15 (0.10, 0.21)**		

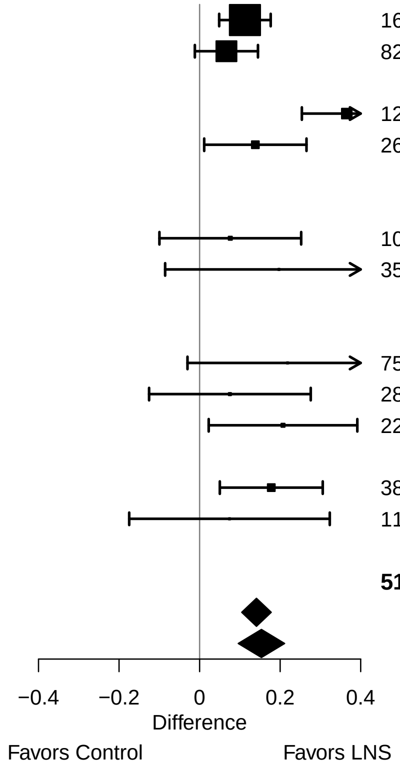

Supplemental figure 8M: Underweight prevalence ratio

8M1: Stratified by Maternal height

P−for−interaction = 0.546
Ratio of PRs = 1.04 (0.92, 1.16)

		At least 150.1 cm						Less than 150.1 cm					
**Country**	**Trial**	**LNS N**	**Control N**	**Control Prevalence**	**PR (95% CI)**	**Fixed W**	**Random W**	**LNS N**	**Control N**	**Control Prevalence**	**PR (95% CI)**	**Fixed W**	**Random W**
Bangladesh	JiVitA−4 (34)												
Bangladesh	RDNS (35)	868	432	31.7	0.81 (0.67, 0.96)	0.14	0.12	735	358	45.8	0.99 (0.87, 1.12)	0.38	0.27
Bangladesh	WASH−B (36)	620	1898	25.7	0.80 (0.67, 0.96)	0.14	0.12	543	1557	40.7	0.76 (0.67, 0.87)	0.36	0.26
Burkina Faso	iLiNS−Zinc (37)	1924	651	30.9	0.68 (0.58, 0.81)	0.17	0.13	34	15	33.3	1.25 (0.76, 2.07)	0.02	0.06
Burkina Faso	PROMIS (38)	829	881	19.3	0.84 (0.67, 1.04)	0.10	0.10	25	25	36.0	1.00 (0.49, 2.04)	0.01	0.03
Burkina Faso	PROMIS CS (38)												
Ghana	GHANA (39)												
Ghana	iLiNS−DYADG (40)												
Haiti	HAITI (41)												
Kenya	WASH−B (42)	1316	4709	9.0	0.81 (0.65, 1.02)	0.09	0.10	57	195	20.0	0.53 (0.25, 1.10)	0.01	0.03
Madagascar	MAHAY (43)	1003	1040	22.2	1.07 (0.88, 1.30)	0.11	0.11	598	565	33.8	0.93 (0.76, 1.15)	0.14	0.18
Malawi	iLiNS−DYADM (44)	192	381	9.7	1.07 (0.64, 1.80)	0.02	0.03	28	60	21.7	1.48 (0.72, 3.05)	0.01	0.03
Malawi	iLiNS−DOSE (45)	586	198	16.7	1.09 (0.79, 1.50)	0.04	0.07	115	42	33.3	0.82 (0.53, 1.27)	0.03	0.07
Mali	PROMIS (46)												
Mali	PROMIS CS (46)	909	928	22.3	0.86 (0.69, 1.06)	0.10	0.10	32	26	34.6	1.17 (0.67, 2.07)	0.02	0.05
Zimbabwe	SHINE (HIV−) (47)	1745	1690	10.4	0.69 (0.55, 0.86)	0.09	0.10	75	62	19.4	0.62 (0.29, 1.30)	0.01	0.03
Zimbabwe	SHINE (HIV+) (48)												
		**9992**	**12808**		**I² = 0.51, Tau² = 0.01**			**2242**	**2905**		**I² = 0.41, Tau² = 0.01**		
**Fixed**					**0.82 (0.77, 0.88)**						**0.89 (0.82, 0.96)**		
**Random**					**0.83 (0.76, 0.92)**						**0.90 (0.79, 1.03)**		

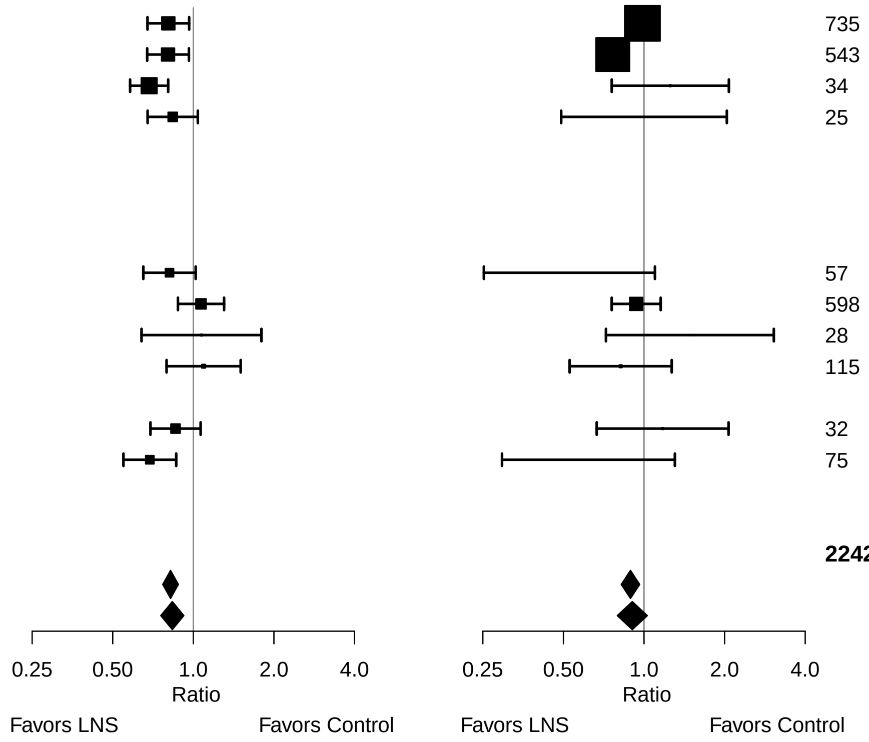

Supplemental figure 8M: Underweight prevalence ratio

8M2: Stratified by Maternal BMI

P−for−interaction = 0.545
Ratio of PRs = 1.03 (0.93, 1.15)

		At least 20 kg/m²						Less than 20 kg/m²					
**Country**	**Trial**	**LNS N**	**Control N**	**Control Prevalence**	**PR (95% CI)**	**Fixed W**	**Random W**	**LNS N**	**Control N**	**Control Prevalence**	**PR (95% CI)**	**Fixed W**	**Random W**
Bangladesh	JiVitA−4 (34)												
Bangladesh	RDNS (35)	730	335	29.3	0.92 (0.76, 1.10)	0.19	0.19	873	455	44.6	0.92 (0.82, 1.04)	0.31	0.21
Bangladesh	WASH−B (36)	518	1595	24.6	0.82 (0.66, 1.02)	0.13	0.13	645	1860	39.2	0.76 (0.67, 0.87)	0.26	0.20
Burkina Faso	iLiNS−Zinc (37)	1218	391	24.8	0.68 (0.53, 0.88)	0.10	0.10	740	275	39.6	0.72 (0.60, 0.86)	0.13	0.14
Burkina Faso	PROMIS (38)	645	673	17.8	0.73 (0.55, 0.97)	0.08	0.08	209	232	25.0	1.13 (0.85, 1.51)	0.05	0.08
Burkina Faso	PROMIS CS (38)	246	267	18.4	1.02 (0.72, 1.44)	0.05	0.05	184	165	33.9	0.91 (0.70, 1.18)	0.07	0.09
Ghana	GHANA (39)												
Ghana	iLiNS−DYADG (40)												
Haiti	HAITI (41)												
Kenya	WASH−B (42)	1100	3822	8.4	0.81 (0.63, 1.06)	0.09	0.09	273	1082	13.4	0.77 (0.54, 1.09)	0.03	0.05
Madagascar	MAHAY (43)												
Malawi	iLiNS−DYADM (44)	131	263	11.4	0.87 (0.47, 1.61)	0.02	0.02	88	178	11.2	1.52 (0.82, 2.82)	0.01	0.02
Malawi	iLiNS−DOSE (45)	519	174	15.5	0.99 (0.70, 1.40)	0.05	0.05	180	65	30.8	0.99 (0.67, 1.47)	0.03	0.05
Mali	PROMIS (46)	377	368	14.3	0.86 (0.57, 1.29)	0.04	0.04	117	133	22.2	0.85 (0.50, 1.44)	0.02	0.03
Mali	PROMIS CS (46)	697	676	20.4	0.91 (0.70, 1.17)	0.10	0.10	244	278	28.1	0.85 (0.63, 1.15)	0.05	0.07
Zimbabwe	SHINE (HIV−) (47)	1355	1300	10.2	0.64 (0.49, 0.83)	0.10	0.10	239	249	14.9	0.84 (0.52, 1.37)	0.02	0.03
Zimbabwe	SHINE (HIV+) (48)	246	253	15.4	0.90 (0.60, 1.34)	0.04	0.04	62	52	26.9	1.08 (0.62, 1.87)	0.01	0.03
		**7782**	**10117**		**I² = 0.00, Tau² = 0.00**			**3854**	**5024**		**I² = 0.33, Tau² = 0.01**		
**Fixed**					**0.83 (0.76, 0.90)**						**0.85 (0.80, 0.91)**		
**Random**					**0.83 (0.76, 0.90)**						**0.87 (0.79, 0.95)**		

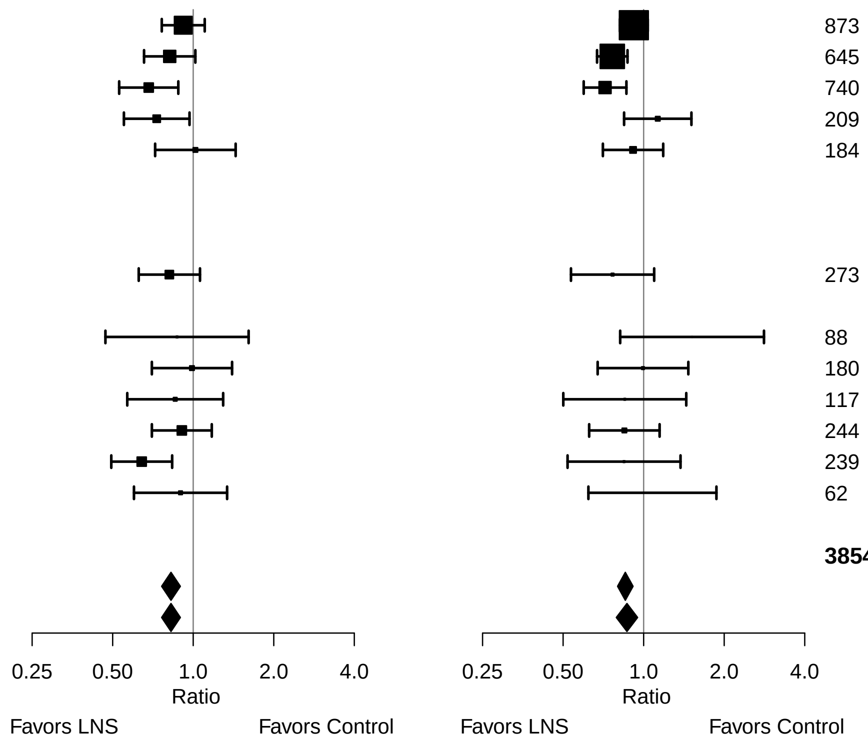

Supplemental figure 8M: Underweight prevalence ratio

8M3: Stratified by Maternal age

P−for−interaction = 0.245
Ratio of PRs = 1.05 (0.97, 1.15)

					At least 25 y						Less than 25 y		
**Country**	**Trial**	**LNS N**	**Control N**	**Control Prevalence**	**PR (95% CI)**	**Fixed W**	**Random W**	**LNS N**	**Control N**	**Control Prevalence**	**PR (95% CI)**	**Fixed W**	**Random W**
Bangladesh	JiVitA−4 (34)	1204	540	40.6	0.88 (0.76, 1.01)	0.20	0.13	1597	687	38.1	0.92 (0.82, 1.03)	0.24	0.21
Bangladesh	RDNS (35)	453	221	36.7	0.92 (0.75, 1.14)	0.10	0.09	1210	594	38.2	0.92 (0.83, 1.02)	0.27	0.22
Bangladesh	WASH−B (36)	493	1521	34.8	0.83 (0.69, 0.99)	0.13	0.11	665	1924	30.6	0.76 (0.64, 0.90)	0.11	0.11
Burkina Faso	iLiNS−Zinc (37)	1134	401	31.7	0.61 (0.49, 0.77)	0.09	0.09	813	262	29.8	0.85 (0.72, 1.01)	0.10	0.11
Burkina Faso	PROMIS (38)	480	524	20.2	0.75 (0.59, 0.96)	0.07	0.08	377	387	18.9	1.01 (0.73, 1.40)	0.03	0.04
Burkina Faso	PROMIS CS (38)	202	190	24.2	0.94 (0.62, 1.43)	0.02	0.04	228	246	24.4	1.02 (0.74, 1.42)	0.03	0.04
Ghana	GHANA (39)												
Ghana	iLiNS−DYADG (40)	220	417	12.0	0.99 (0.63, 1.54)	0.02	0.03	127	276	14.5	0.60 (0.32, 1.13)	0.01	0.01
Haiti	HAITI (41)												
Kenya	WASH−B (42)	845	2827	9.3	0.79 (0.60, 1.04)	0.06	0.07	601	2306	10.8	0.72 (0.53, 0.98)	0.03	0.04
Madagascar	MAHAY (43)	895	952	27.4	1.01 (0.84, 1.22)	0.12	0.10	806	733	24.1	1.08 (0.87, 1.34)	0.06	0.07
Malawi	iLiNS−DYADM (44)	106	226	11.1	1.11 (0.59, 2.08)	0.01	0.02	114	218	12.4	1.13 (0.64, 2.01)	0.01	0.01
Malawi	iLiNS−DOSE (45)	372	134	20.1	1.13 (0.81, 1.57)	0.04	0.05	316	100	18.0	0.97 (0.63, 1.51)	0.02	0.02
Mali	PROMIS (46)	342	355	15.6	0.95 (0.63, 1.43)	0.03	0.04	158	147	18.4	0.67 (0.37, 1.20)	0.01	0.01
Mali	PROMIS CS (46)	498	502	21.5	0.91 (0.68, 1.21)	0.05	0.06	448	458	23.6	0.86 (0.66, 1.12)	0.04	0.05
Zimbabwe	SHINE (HIV−) (47)	870	797	10.9	0.59 (0.42, 0.83)	0.04	0.05	802	793	10.3	0.80 (0.59, 1.07)	0.03	0.04
Zimbabwe	SHINE (HIV+) (48)	243	231	17.7	0.95 (0.63, 1.44)	0.02	0.04	73	77	14.3	1.25 (0.60, 2.60)	0.01	0.01
		**8357**	**9838**		**I² = 0.38, Tau² = 0.01**			**8335**	**9208**		**I² = 0.11, Tau² = 0.00**		
**Fixed**					**0.85 (0.80, 0.91)**						**0.89 (0.85, 0.94)**		
**Random**					**0.85 (0.78, 0.93)**						**0.89 (0.84, 0.95)**		

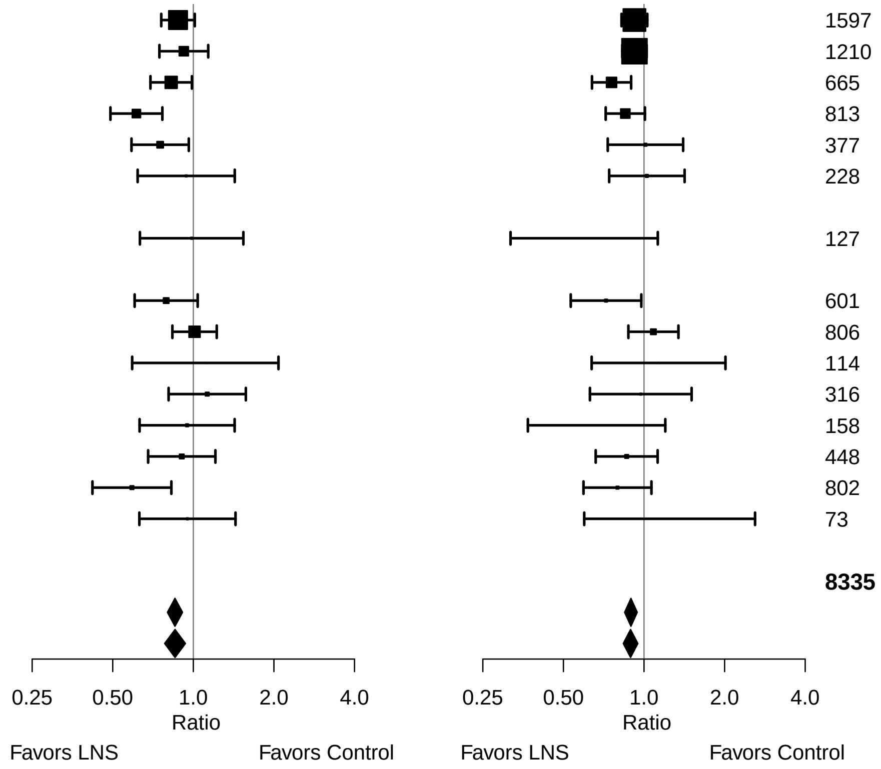

Supplemental figure 8M: Underweight prevalence ratio

8M4: Stratified by Maternal education

P−for−interaction = 0.399
Ratio of PRs = 1.05 (0.94, 1.17)

		Primary or greater						Incomplete or no formal					
**Country**	**Trial**	**LNS N**	**Control N**	**Control Prevalence**	**PR (95% CI)**	**Fixed W**	**Random W**	**LNS N**	**Control N**	**Control Prevalence**	**PR (95% CI)**	**Fixed W**	**Random W**
Bangladesh	JiVitA−4 (34)	1816	749	36.6	0.84 (0.74, 0.95)	0.29	0.22	996	480	43.3	0.98 (0.86, 1.12)	0.26	0.19
Bangladesh	RDNS (35)	1245	589	35.8	0.93 (0.82, 1.06)	0.26	0.21	418	226	42.9	0.92 (0.74, 1.14)	0.10	0.11
Bangladesh	WASH−B (36)	818	2476	28.7	0.75 (0.66, 0.87)	0.22	0.20	345	985	41.8	0.83 (0.69, 1.00)	0.13	0.13
Burkina Faso	iLiNS−Zinc (37)												
Burkina Faso	PROMIS (38)	70	54	13.0	1.65 (0.69, 3.98)	0.01	0.01	787	857	20.1	0.82 (0.67, 1.01)	0.10	0.12
Burkina Faso	PROMIS CS (38)	34	39	28.2	0.94 (0.41, 2.13)	0.01	0.01	393	396	24.0	1.00 (0.77, 1.28)	0.07	0.09
Ghana	GHANA (39)												
Ghana	iLiNS−DYADG (40)	267	549	12.4	0.94 (0.63, 1.40)	0.03	0.05	80	144	15.3	0.49 (0.21, 1.16)	0.01	0.01
Haiti	HAITI (41)												
Kenya	WASH−B (42)	705	2451	6.7	0.96 (0.68, 1.36)	0.04	0.07	754	2710	13.1	0.67 (0.52, 0.87)	0.06	0.08
Madagascar	MAHAY (43)	352	431	21.1	1.05 (0.79, 1.39)	0.05	0.09	1350	1254	27.7	1.02 (0.86, 1.21)	0.15	0.15
Malawi	iLiNS−DYADM (44)												
Malawi	iLiNS−DOSE (45)												
Mali	PROMIS (46)												
Mali	PROMIS CS (46)	107	96	19.8	0.57 (0.28, 1.14)	0.01	0.02	838	864	22.8	0.92 (0.75, 1.13)	0.10	0.11
Zimbabwe	SHINE (HIV−) (47)	1707	1636	10.5	0.70 (0.56, 0.88)	0.09	0.12	68	61	13.1	0.90 (0.37, 2.20)	0.01	0.01
Zimbabwe	SHINE (HIV+) (48)												
		**7121**	**9070**		**I² = 0.34, Tau² = 0.01**			**6029**	**7977**		**I² = 0.29, Tau² = 0.01**		
**Fixed**					**0.85 (0.79, 0.90)**						**0.91 (0.85, 0.97)**		
**Random**					**0.85 (0.77, 0.94)**						**0.90 (0.82, 0.98)**		

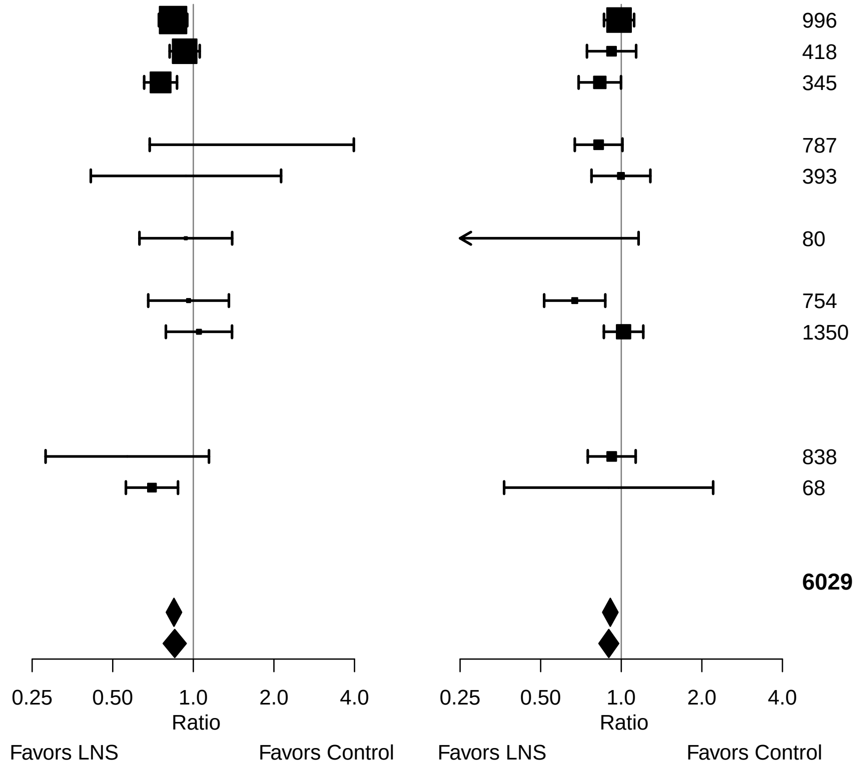

Supplemental figure 8M: Underweight prevalence ratio

8M5: Stratified by Maternal depressive symptoms

P−for−interaction = 0.490
Ratio of PRs = 0.96 (0.84, 1.09)

		Less than 75th percentile						At least 75th percentile					
Country	Trial	LNS N	Control N	Control Prevalence	PR (95% CI)	Fixed W	Random W	LNS N	Control N	Control Prevalence	PR (95% CI)	Fixed W	Random W
Bangladesh	JiVitA−4 (34)												
Bangladesh	RDNS (35)	1081	472	36.9	0.93 (0.83, 1.04)	0.35	0.34	520	288	38.2	0.93 (0.75, 1.16)	0.26	0.18
Bangladesh	WASH−B (36)	899	2487	31.6	0.79 (0.69, 0.90)	0.26	0.25	240	883	34.7	0.78 (0.61, 1.01)	0.19	0.16
Burkina Faso	iLiNS−Zinc (37)												
Burkina Faso	PROMIS (38)	505	571	19.4	0.79 (0.59, 1.08)	0.05	0.05	220	213	21.1	0.95 (0.71, 1.26)	0.15	0.14
Burkina Faso	PROMIS CS (38)												
Ghana	GHANA (39)												
Ghana	iLiNS−DYADG (40)	252	460	12.4	0.70 (0.44, 1.12)	0.02	0.02	83	209	12.9	1.31 (0.72, 2.36)	0.03	0.06
Haiti	HAITI (41)												
Kenya	WASH−B (42)	997	3506	9.6	0.82 (0.66, 1.02)	0.09	0.09	342	1248	10.5	0.72 (0.46, 1.15)	0.06	0.08
Madagascar	MAHAY (43)	673	721	27.2	0.99 (0.81, 1.21)	0.11	0.11	283	252	24.2	1.23 (0.90, 1.68)	0.12	0.13
Malawi	iLiNS−DYADM (44)	157	303	11.2	1.02 (0.60, 1.75)	0.02	0.02	47	113	12.4	1.20 (0.52, 2.79)	0.02	0.03
Malawi	iLiNS−DOSE (45)												
Mali	PROMIS (46)	374	372	15.8	0.79 (0.52, 1.18)	0.03	0.03	122	129	18.3	1.06 (0.66, 1.70)	0.05	0.08
Mali	PROMIS CS (46)												
Zimbabwe	SHINE (HIV−) (47)	1192	1137	9.9	0.76 (0.57, 1.02)	0.05	0.05	509	490	12.7	0.54 (0.37, 0.81)	0.08	0.10
Zimbabwe	SHINE (HIV+) (48)	234	214	16.4	1.10 (0.74, 1.63)	0.03	0.03	88	100	20.0	0.85 (0.46, 1.59)	0.03	0.05
		**6364**	**10243**		**I² = 0.03, Tau² = 0.00**			**2454**	**3925**		**I² = 0.38, Tau² = 0.02**		
**Fixed**					**0.87 (0.81, 0.93)**						**0.90 (0.81, 1.01)**		
**Random**					**0.87 (0.81, 0.93)**						**0.90 (0.77, 1.06)**		

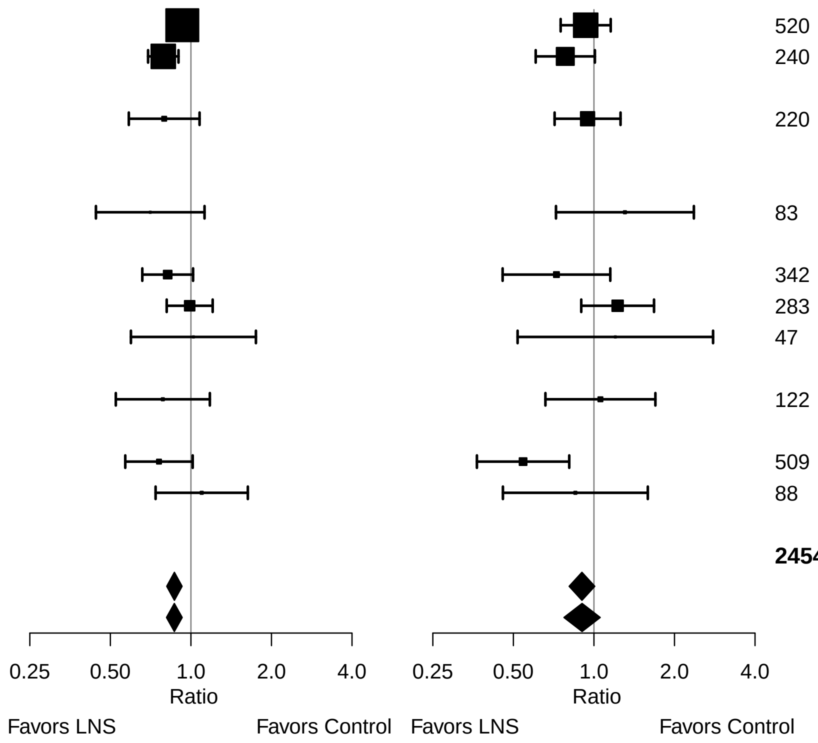

Supplemental figure 8M: Underweight prevalence ratio

8M6: Stratified by Child sex

P−for−interaction = 0.617
Ratio of PRs = 0.98 (0.91, 1.06)

Country	Trial	Male LNS N	Male Control N	Male Control Prevalence	Male PR (95% CI)	Male Fixed W	Male Random W	Female LNS N	Female Control N	Female Control Prevalence	Female PR (95% CI)	Female Fixed W	Female Random W
Bangladesh	JiVitA−4 (34)	1440	594	38.2	0.95 (0.83, 1.09)	0.19	0.13	1374	638	40.1	0.87 (0.77, 0.98)	0.25	0.14
Bangladesh	RDNS (35)	833	411	38.0	0.95 (0.82, 1.10)	0.14	0.12	830	404	37.6	0.89 (0.78, 1.02)	0.21	0.13
Bangladesh	WASH−B (36)	567	1730	33.2	0.78 (0.67, 0.91)	0.14	0.12	596	1731	31.7	0.79 (0.67, 0.94)	0.13	0.12
Burkina Faso	iLiNS−Zinc (37)	995	336	32.4	0.78 (0.64, 0.95)	0.08	0.09	965	330	29.4	0.60 (0.48, 0.75)	0.08	0.09
Burkina Faso	PROMIS (38)	435	482	24.9	0.81 (0.64, 1.04)	0.05	0.07	422	429	13.8	0.98 (0.67, 1.43)	0.03	0.05
Burkina Faso	PROMIS CS (38)	234	221	27.6	1.04 (0.75, 1.43)	0.03	0.04	196	215	20.9	0.88 (0.59, 1.30)	0.02	0.04
Ghana	GHANA (39)												
Ghana	iLiNS−DYADG (40)	175	324	16.4	0.73 (0.46, 1.17)	0.01	0.02	172	369	10.0	0.93 (0.53, 1.62)	0.01	0.03
Haiti	HAITI (41)												
Kenya	WASH−B (42)	734	2457	11.8	0.74 (0.57, 0.96)	0.05	0.06	727	2708	8.4	0.77 (0.59, 1.00)	0.05	0.08
Madagascar	MAHAY (43)	826	835	31.4	1.00 (0.84, 1.19)	0.11	0.10	876	850	20.7	1.10 (0.90, 1.35)	0.09	0.10
Malawi	iLiNS−DYADM (44)	107	207	12.6	1.41 (0.82, 2.43)	0.01	0.02	113	237	11.0	0.81 (0.40, 1.61)	0.01	0.02
Malawi	iLiNS−DOSE (45)	353	124	29.0	0.97 (0.71, 1.31)	0.04	0.05	349	117	10.3	1.19 (0.73, 1.93)	0.02	0.03
Mali	PROMIS (46)	265	262	18.6	0.83 (0.60, 1.16)	0.03	0.04	235	240	14.0	0.87 (0.50, 1.50)	0.01	0.03
Mali	PROMIS CS (46)	474	521	25.1	0.88 (0.66, 1.17)	0.04	0.05	472	439	19.4	0.91 (0.68, 1.21)	0.05	0.07
Zimbabwe	SHINE (HIV−) (47)	924	906	13.4	0.66 (0.51, 0.84)	0.05	0.06	945	880	7.7	0.81 (0.57, 1.14)	0.03	0.05
Zimbabwe	SHINE (HIV+) (48)	161	167	22.8	0.79 (0.53, 1.19)	0.02	0.03	175	161	11.2	1.48 (0.88, 2.49)	0.01	0.03
		**8523**	**9577**		**I² = 0.32, Tau² = 0.01**			**8447**	**9748**		**I² = 0.44, Tau² = 0.01**		
**Fixed**					**0.87 (0.82, 0.92)**						**0.86 (0.81, 0.92)**		
**Random**					**0.87 (0.80, 0.93)**						**0.87 (0.79, 0.96)**		

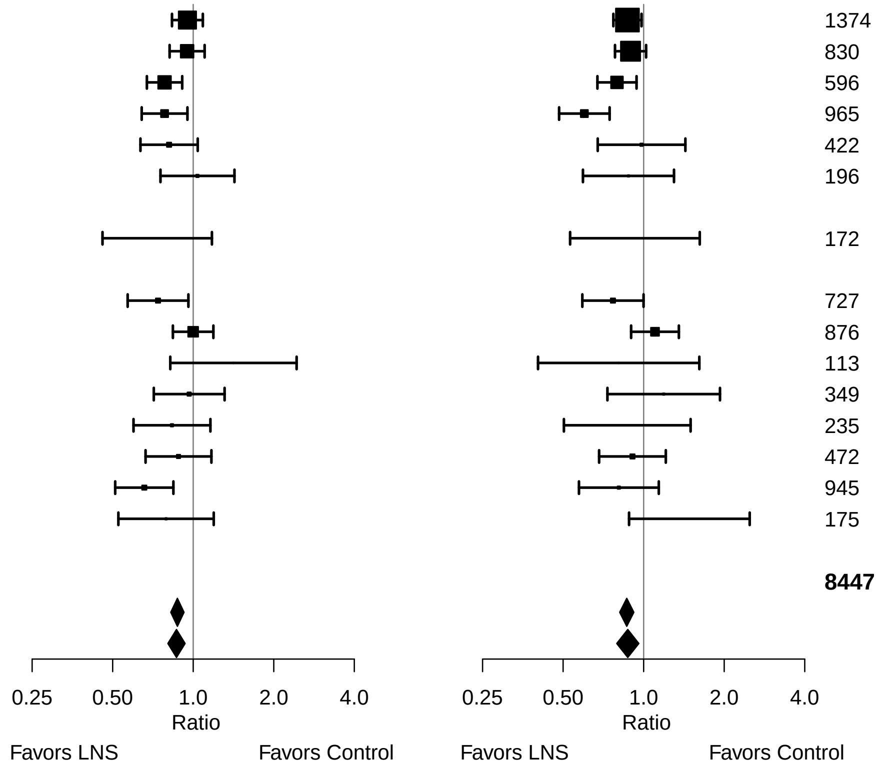

Supplemental figure 8M: Underweight prevalence ratio

8M7: Stratified by Child birth order

P−for−interaction = 0.001
Ratio of PRs = 1.16 (1.06, 1.27)

		Later born						Firstborn					
Country	Trial	LNS N	Control N	Control Prevalence	PR (95% CI)	Fixed W	Random W	LNS N	Control N	Control Prevalence	PR (95% CI)	Fixed W	Random W
Bangladesh	JiVitA−4 (34)	600	250	43.3	0.80 (0.69, 0.92)	0.13	0.09	2210	976	38.0	0.95 (0.85, 1.06)	0.35	0.31
Bangladesh	RDNS (35)	974	508	38.0	0.91 (0.79, 1.05)	0.15	0.09	688	307	37.5	0.94 (0.82, 1.08)	0.25	0.24
Bangladesh	WASH−B (36)	737	2310	34.3	0.78 (0.67, 0.90)	0.14	0.09	426	1151	28.6	0.82 (0.67, 1.01)	0.11	0.12
Burkina Faso	iLiNS−Zinc (37)	1524	521	30.9	0.63 (0.53, 0.75)	0.10	0.09	436	145	31.0	0.93 (0.71, 1.21)	0.06	0.07
Burkina Faso	PROMIS (38)	709	750	19.9	0.80 (0.66, 0.98)	0.07	0.08	148	159	18.9	1.15 (0.72, 1.83)	0.02	0.02
Burkina Faso	PROMIS CS (38)	366	357	23.8	0.99 (0.76, 1.29)	0.04	0.06	63	79	26.6	1.02 (0.62, 1.66)	0.02	0.02
Ghana	GHANA (39)												
Ghana	iLiNS−DYADG (40)	237	459	12.2	0.97 (0.63, 1.48)	0.02	0.03	110	234	14.5	0.56 (0.28, 1.13)	0.01	0.01
Haiti	HAITI (41)												
Kenya	WASH−B (42)	1137	4065	10.2	0.76 (0.60, 0.95)	0.06	0.07	322	1095	9.2	0.77 (0.50, 1.19)	0.03	0.03
Madagascar	MAHAY (43)	1271	1220	27.2	0.98 (0.83, 1.16)	0.10	0.09	431	465	22.8	1.23 (0.94, 1.62)	0.06	0.07
Malawi	iLiNS−DYADM (44)	173	353	11.3	1.17 (0.73, 1.90)	0.01	0.03	47	89	12.4	1.03 (0.41, 2.62)	0.01	0.01
Malawi	iLiNS−DOSE (45)	463	165	19.4	1.06 (0.79, 1.42)	0.03	0.05	150	45	22.2	0.81 (0.44, 1.50)	0.01	0.01
Mali	PROMIS (46)	437	441	16.9	0.79 (0.57, 1.09)	0.03	0.05	62	59	11.9	1.57 (0.76, 3.25)	0.01	0.01
Mali	PROMIS CS (46)	784	822	22.5	0.86 (0.69, 1.06)	0.06	0.07	145	126	20.6	1.04 (0.67, 1.61)	0.02	0.03
Zimbabwe	SHINE (HIV−) (47)	1295	1220	10.4	0.61 (0.45, 0.82)	0.03	0.05	487	483	11.2	0.92 (0.64, 1.31)	0.04	0.04
Zimbabwe	SHINE (HIV+) (48)	279	245	14.3	1.25 (0.84, 1.88)	0.02	0.04	54	78	25.6	0.51 (0.25, 1.02)	0.01	0.01
		**10986**	**13686**		**I² = 0.56, Tau² = 0.02**			**5779**	**5491**		**I² = 0.03, Tau² = 0.00**		
**Fixed**					**0.83 (0.79, 0.88)**						**0.94 (0.88, 1.00)**		
**Random**					**0.84 (0.77, 0.93)**						**0.94 (0.87, 1.01)**		

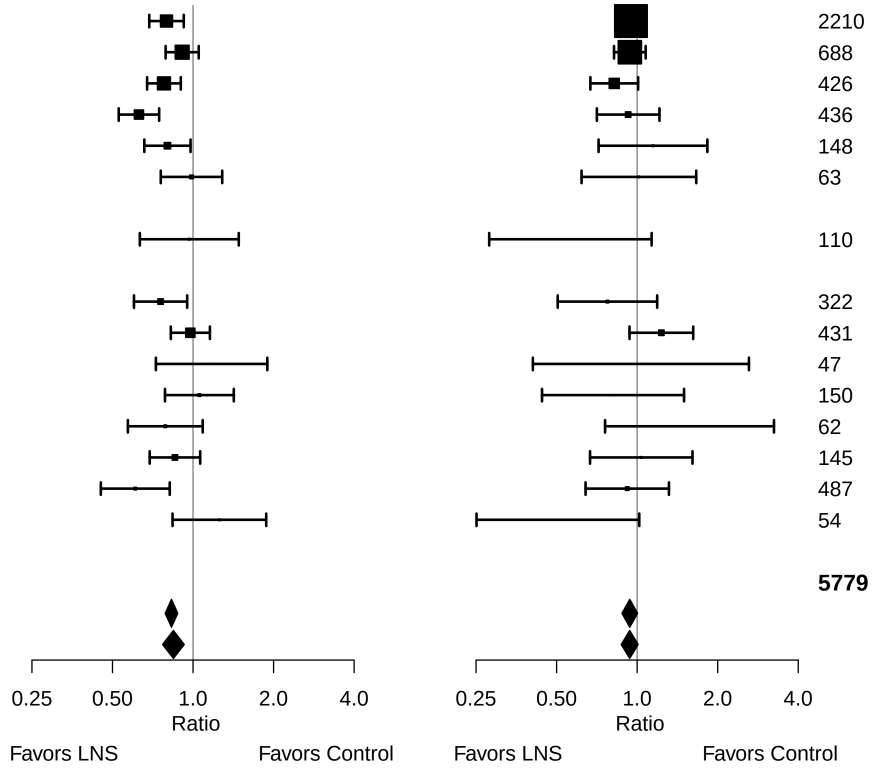

Supplemental figure 8M: Underweight prevalence ratio

8M8: Stratified by Child baseline anthropometric status

P−for−interaction = 0.493
Ratio of PRs = 1.09 (0.85, 1.39)

Country	Trial	WAZ at least −1 Z: LNS N	Control N	Control Prevalence	PR (95% CI)	Fixed W	Random W	WAZ less than −1 Z: LNS N	Control N	Control Prevalence	PR (95% CI)	Fixed W	Random W
Bangladesh	JiVitA−4 (34)	1198	531	6.4	0.61 (0.40, 0.95)	0.30	0.30	1616	701	64.3	0.89 (0.84, 0.96)	0.50	0.36
Bangladesh	RDNS (35)	780	338	5.6	1.12 (0.69, 1.81)	0.24	0.24	821	422	62.8	0.98 (0.90, 1.07)	0.33	0.31
Bangladesh	WASH−B (36)												
Burkina Faso	iLiNS−Zinc (37)												
Burkina Faso	PROMIS (38)	427	482	5.8	0.77 (0.44, 1.32)	0.19	0.19	268	267	42.7	0.87 (0.72, 1.06)	0.06	0.11
Burkina Faso	PROMIS CS (38)												
Ghana	GHANA (39)												
Ghana	iLiNS−DYADG (40)	222	424	1.7	1.36 (0.44, 4.25)	0.04	0.04	101	215	34.0	0.93 (0.66, 1.31)	0.02	0.04
Haiti	HAITI (41)												
Kenya	WASH−B (42)												
Madagascar	MAHAY (43)												
Malawi	iLiNS−DYADM (44)												
Malawi	iLiNS−DOSE (45)	419	143	3.5	0.89 (0.32, 2.45)	0.06	0.06	283	98	43.9	0.96 (0.74, 1.25)	0.03	0.07
Mali	PROMIS (46)												
Mali	PROMIS CS (46)												
Zimbabwe	SHINE (HIV−) (47)	917	836	1.9	0.85 (0.41, 1.77)	0.11	0.11	383	349	30.1	0.70 (0.56, 0.89)	0.04	0.08
Zimbabwe	SHINE (HIV+) (48)	151	144	5.6	0.83 (0.29, 2.36)	0.05	0.05	113	92	32.6	1.03 (0.70, 1.51)	0.02	0.03
		**4114**	**2898**		**I² = 0.00, Tau² = 0.00**			**3585**	**2144**		**I² = 0.31, Tau² = 0.00**		
**Fixed**					**0.83 (0.65, 1.05)**						**0.92 (0.87, 0.96)**		
**Random**					**0.83 (0.65, 1.05)**						**0.91 (0.85, 0.98)**		

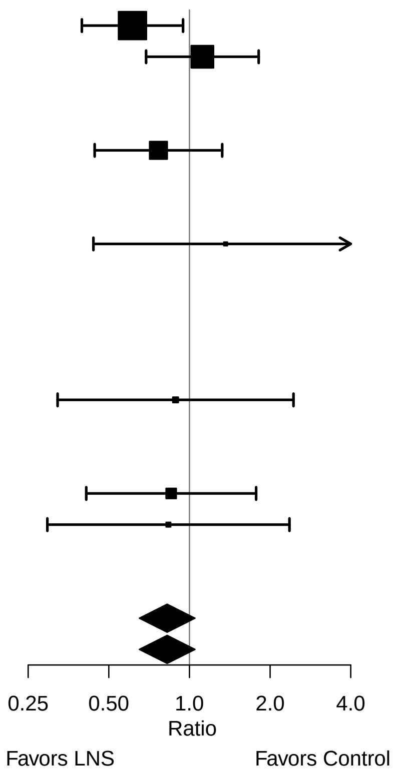

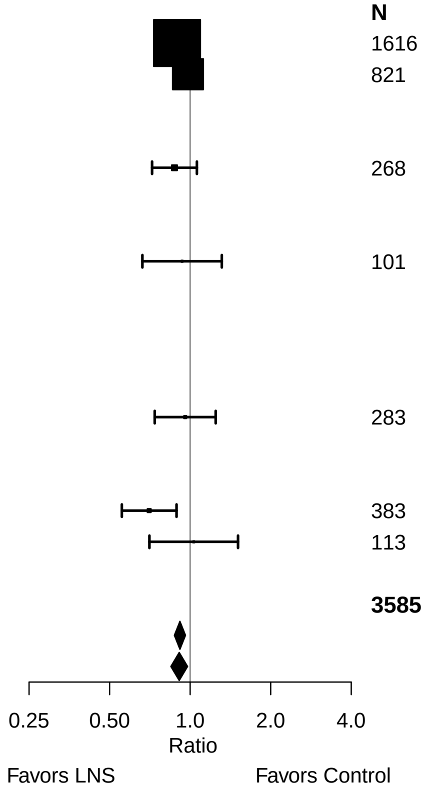

Supplemental figure 8N: Underweight prevalence difference

8N1: Stratified by Maternal height

P−for−interaction **= 0.211**
Difference in PDs = **−0.02 (−0.05, 0.01)**

					**At least 150.1 cm**						**Less than 150.1 cm**		
**Country**	**Trial**	**LNS N**	**Control N**	**Control Prevalence**	**PD (95% CI)**	**Fixed W**	**Random W**	**LNS N**	**Control N**	**Control Prevalence**	**PD (95% CI)**	**Fixed W**	**Random W**
Bangladesh	JiVitA−4 (34)												
Bangladesh	RDNS (35)	868	432	31.7	−0.06 (−0.11, −0.01)	0.04	0.08	735	358	45.8	−0.01 (−0.07, 0.05)	0.22	0.20
Bangladesh	WASH−B (36)	620	1898	25.7	−0.05 (−0.09, −0.01)	0.07	0.10	543	1557	40.7	−0.10 (−0.14, −0.05)	0.37	0.25
Burkina Faso	iLiNS−Zinc (37)	1924	651	30.9	−0.10 (−0.14, −0.07)	0.08	0.11	34	15	33.3	0.11 (−0.09, 0.30)	0.02	0.03
Burkina Faso	PROMIS (38)	829	881	19.3	−0.03 (−0.07, 0.01)	0.06	0.10	25	25	36.0	0.00 (−0.26, 0.26)	0.01	0.02
Burkina Faso	PROMIS CS (38)												
Ghana	GHANA (39)												
Ghana	iLiNS−DYADG (40)												
Haiti	HAITI (41)												
Kenya	WASH−B (42)	1316	4709	9.0	−0.02 (−0.03, 0.00)	0.33	0.14	57	195	20.0	−0.09 (−0.19, 0.00)	0.09	0.12
Madagascar	MAHAY (43)	1003	1040	22.2	0.02 (−0.03, 0.06)	0.05	0.09	598	565	33.8	−0.02 (−0.09, 0.05)	0.16	0.17
Malawi	iLiNS−DYADM (44)	192	381	9.7	0.01 (−0.04, 0.06)	0.04	0.08	28	60	21.7	0.10 (−0.09, 0.30)	0.02	0.03
Malawi	iLiNS−DOSE (45)	586	198	16.7	−0.01 (−0.06, 0.05)	0.04	0.08	115	42	33.3	−0.06 (−0.19, 0.07)	0.04	0.07
Mali	PROMIS (46)												
Mali	PROMIS CS (46)	909	928	22.3	−0.03 (−0.08, 0.01)	0.05	0.09	32	26	34.6	0.06 (−0.15, 0.27)	0.02	0.03
Zimbabwe	SHINE (HIV−) (47)	1745	1690	10.4	−0.03 (−0.05, −0.01)	0.26	0.14	75	62	19.4	−0.07 (−0.19, 0.04)	0.06	0.08
Zimbabwe	SHINE (HIV+) (48)												
		**9992**	**12808**		**I² = 0.68, Tau² = 0.00**			**2242**	**2905**		**I² = 0.35, Tau² = 0.00**		
**Fixed**					**−0.03 (−0.04, −0.02)**						**−0.05 (−0.08, −0.02)**		
**Random**					**−0.03 (−0.05, −0.01)**						**−0.04 (−0.08, 0.00)**		

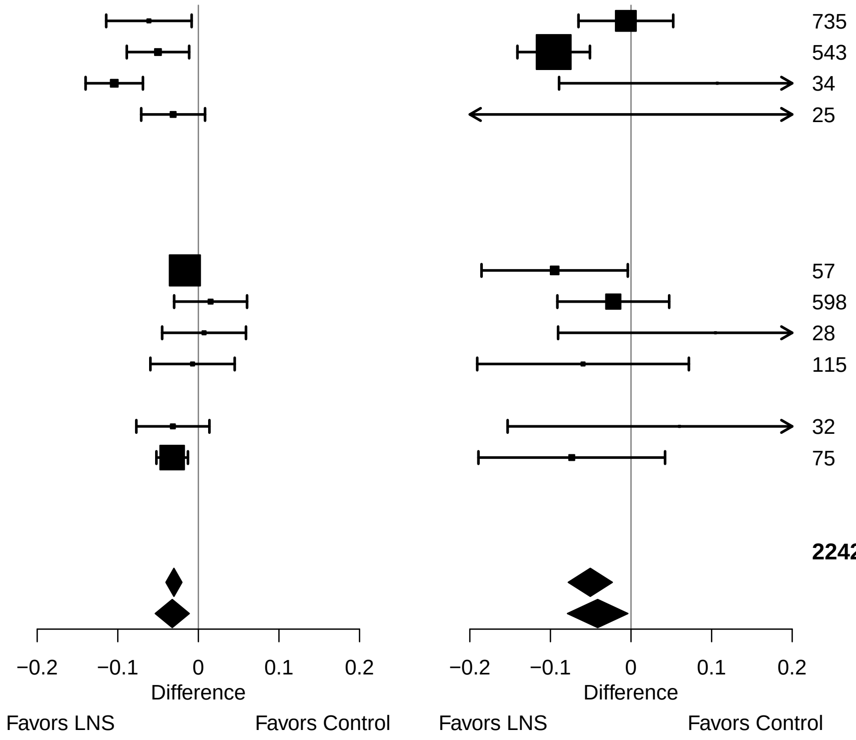

Supplemental figure 8N: Underweight prevalence difference

8N2: Stratified by Maternal BMI

P−for−interaction = 0.263
Difference in PDs = −0.01 (−0.03, 0.01)

Country	Trial	At least 20 kg/m² LNS N	Control N	Control Prevalence	PD (95% CI)	Fixed W	Random W	Less than 20 kg/m² LNS N	Control N	Control Prevalence	PD (95% CI)	Fixed W	Random W
Bangladesh	JiVitA−4 (34)												
Bangladesh	RDNS (35)	730	335	29.3	−0.02 (−0.08, 0.03)	0.04	0.04	873	455	44.6	−0.04 (−0.09, 0.02)	0.12	0.11
Bangladesh	WASH−B (36)	518	1595	24.6	−0.04 (−0.09, 0.00)	0.05	0.05	645	1860	39.2	−0.09 (−0.14, −0.05)	0.18	0.12
Burkina Faso	iLiNS−Zinc (37)	1218	391	24.8	−0.08 (−0.13, −0.03)	0.05	0.05	740	275	39.6	−0.13 (−0.18, −0.08)	0.14	0.11
Burkina Faso	PROMIS (38)	645	673	17.8	−0.05 (−0.09, 0.00)	0.06	0.06	209	232	25.0	0.03 (−0.04, 0.11)	0.05	0.08
Burkina Faso	PROMIS CS (38)	246	267	18.4	0.00 (−0.06, 0.07)	0.03	0.03	184	165	33.9	−0.03 (−0.11, 0.05)	0.05	0.07
Ghana	GHANA (39)												
Ghana	iLiNS−DYADG (40)												
Haiti	HAITI (41)												
Kenya	WASH−B (42)	1100	3822	8.4	−0.02 (−0.03, 0.00)	0.32	0.32	273	1082	13.4	−0.03 (−0.07, 0.01)	0.21	0.13
Madagascar	MAHAY (43)												
Malawi	iLiNS−DYADM (44)	131	263	11.4	−0.01 (−0.08, 0.05)	0.03	0.03	88	178	11.2	0.06 (−0.03, 0.14)	0.04	0.07
Malawi	iLiNS−DOSE (45)	519	174	15.5	−0.01 (−0.06, 0.05)	0.04	0.04	180	65	30.8	−0.05 (−0.15, 0.06)	0.03	0.05
Mali	PROMIS (46)	377	368	14.3	−0.04 (−0.09, 0.02)	0.04	0.04	117	133	22.2	−0.04 (−0.13, 0.05)	0.04	0.06
Mali	PROMIS CS (46)	697	676	20.4	−0.02 (−0.07, 0.03)	0.05	0.05	244	278	28.1	−0.04 (−0.12, 0.04)	0.05	0.08
Zimbabwe	SHINE (HIV−) (47)	1355	1300	10.2	−0.04 (−0.06, −0.02)	0.25	0.25	239	249	14.9	−0.02 (−0.09, 0.04)	0.07	0.09
Zimbabwe	SHINE (HIV+) (48)	246	253	15.4	−0.02 (−0.07, 0.04)	0.03	0.03	62	52	26.9	0.02 (−0.13, 0.18)	0.01	0.03
		**7782**	**10117**		**I² = 0.00, Tau² = 0.00**			**3854**	**5024**		**I² = 0.60, Tau² = 0.00**		
**Fixed**					**−0.03 (−0.04, −0.02)**						**−0.05 (−0.07, −0.03)**		
**Random**					**−0.03 (−0.04, −0.02)**						**−0.04 (−0.07, −0.01)**		

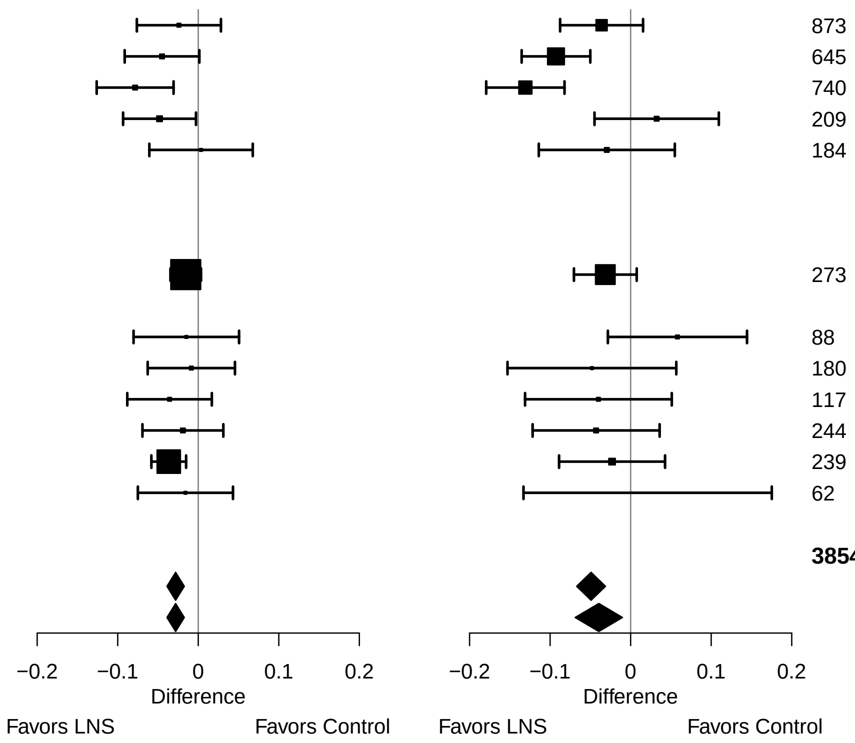

Supplemental figure 8N: Underweight prevalence difference

8N3: Stratified by Maternal age

P−for−interaction = 0.628
Difference in PDs = 0.00 (−0.01, 0.02)

		At least 25 y						Less than 25 y					
**Country**	**Trial**	**LNS N**	**Control N**	**Control Prevalence**	**PD (95% CI)**	**Fixed W**	**Random W**	**LNS N**	**Control N**	**Control Prevalence**	**PD (95% CI)**	**Fixed W**	**Random W**
Bangladesh	JiVitA−4 (34)	1204	540	40.6	−0.05 (−0.09, −0.01)	0.08	0.09	1597	687	38.1	−0.04 (−0.07, 0.00)	0.12	0.11
Bangladesh	RDNS (35)	453	221	36.7	−0.03 (−0.10, 0.05)	0.02	0.04	1210	594	38.2	−0.03 (−0.07, 0.01)	0.09	0.09
Bangladesh	WASH−B (36)	493	1521	34.8	−0.06 (−0.11, −0.01)	0.04	0.06	665	1924	30.6	−0.07 (−0.12, −0.03)	0.07	0.08
Burkina Faso	iLiNS−Zinc (37)	1134	401	31.7	−0.12 (−0.17, −0.07)	0.05	0.07	813	262	29.8	−0.07 (−0.12, −0.03)	0.07	0.08
Burkina Faso	PROMIS (38)	480	524	20.2	−0.05 (−0.09, −0.01)	0.07	0.08	377	387	18.9	0.00 (−0.06, 0.06)	0.04	0.05
Burkina Faso	PROMIS CS (38)	202	190	24.2	−0.01 (−0.11, 0.08)	0.01	0.02	228	246	24.4	0.01 (−0.07, 0.09)	0.02	0.03
Ghana	GHANA (39)												
Ghana	iLiNS−DYADG (40)	220	417	12.0	0.00 (−0.05, 0.05)	0.05	0.06	127	276	14.5	−0.06 (−0.13, 0.01)	0.03	0.04
Haiti	HAITI (41)												
Kenya	WASH−B (42)	845	2827	9.3	−0.02 (−0.04, 0.00)	0.30	0.15	601	2306	10.8	−0.03 (−0.06, 0.00)	0.21	0.15
Madagascar	MAHAY (43)	895	952	27.4	0.00 (−0.05, 0.06)	0.05	0.06	806	733	24.1	0.02 (−0.03, 0.07)	0.05	0.06
Malawi	iLiNS−DYADM (44)	106	226	11.1	0.01 (−0.06, 0.09)	0.02	0.04	114	218	12.4	0.02 (−0.06, 0.09)	0.02	0.03
Malawi	iLiNS−DOSE (45)	372	134	20.1	0.00 (−0.06, 0.07)	0.03	0.05	316	100	18.0	−0.03 (−0.10, 0.05)	0.02	0.03
Mali	PROMIS (46)	342	355	15.6	−0.02 (−0.08, 0.03)	0.05	0.06	158	147	18.4	−0.07 (−0.15, 0.00)	0.02	0.03
Mali	PROMIS CS (46)	498	502	21.5	−0.02 (−0.08, 0.04)	0.04	0.05	448	458	23.6	−0.03 (−0.09, 0.02)	0.04	0.05
Zimbabwe	SHINE (HIV−) (47)	870	797	10.9	−0.04 (−0.07, −0.02)	0.16	0.12	802	793	10.3	−0.02 (−0.05, 0.01)	0.19	0.14
Zimbabwe	SHINE (HIV+) (48)	243	231	17.7	−0.01 (−0.08, 0.06)	0.03	0.04	73	77	14.3	0.04 (−0.08, 0.15)	0.01	0.01
		**8357**	**9838**		**I² = 0.39, Tau² = 0.00**			**8335**	**9208**		**I² = 0.23, Tau² = 0.00**		
**Fixed**					**−0.03 (−0.04, −0.02)**						**−0.03 (−0.04, −0.02)**		
**Random**					**−0.03 (−0.05, −0.02)**						**−0.03 (−0.05, −0.02)**		

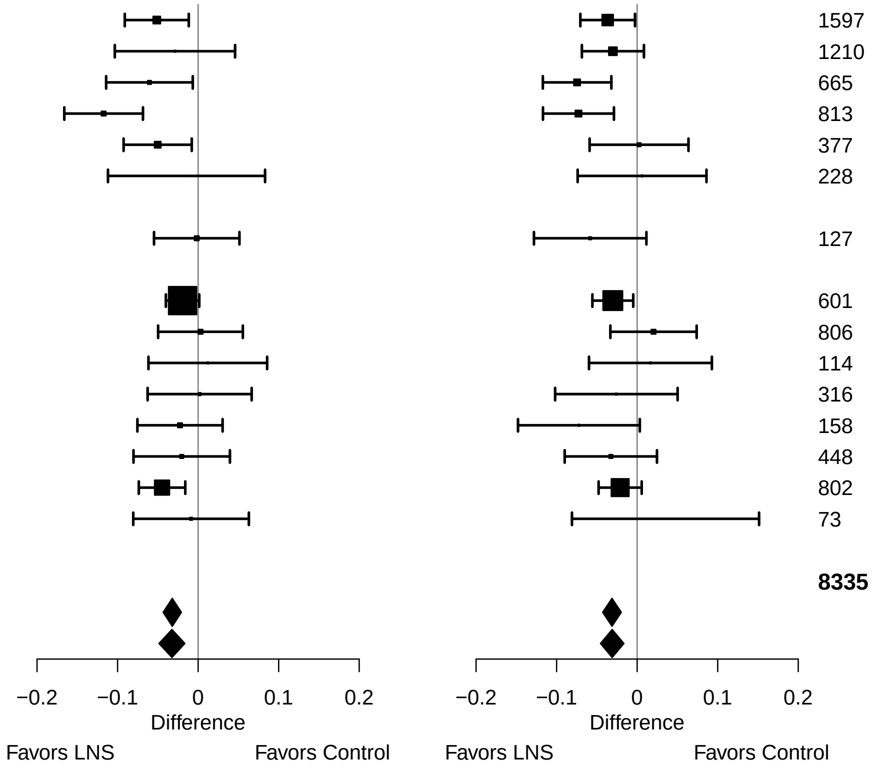

Supplemental figure 8N: Underweight prevalence difference

8N4: Stratified by Maternal education

P−for−interaction = 0.136
Difference in PDs = −0.02 (−0.04, 0.01)

Country	Trial	Primary or greater: LNS N	Control N	Control Prevalence	PD (95% CI)	Fixed W	Random W	Incomplete or no formal: LNS N	Control N	Control Prevalence	PD (95% CI)	Fixed W	Random W
Bangladesh	JiVitA−4 (34)	1816	749	36.6	−0.06 (−0.09, −0.02)	0.11	0.14	996	480	43.3	−0.02 (−0.06, 0.02)	0.13	0.13
Bangladesh	RDNS (35)	1245	589	35.8	−0.03 (−0.07, 0.02)	0.06	0.11	418	226	42.9	−0.03 (−0.12, 0.05)	0.03	0.03
Bangladesh	WASH−B (36)	818	2476	28.7	−0.07 (−0.10, −0.04)	0.12	0.14	345	985	41.8	−0.07 (−0.14, 0.00)	0.05	0.05
Burkina Faso	iLiNS−Zinc (37)												
Burkina Faso	PROMIS (38)	70	54	13.0	0.08 (−0.04, 0.21)	0.01	0.02	787	857	20.1	−0.04 (−0.07, 0.00)	0.15	0.15
Burkina Faso	PROMIS CS (38)	34	39	28.2	−0.02 (−0.24, 0.20)	0.00	0.01	393	396	24.0	0.00 (−0.06, 0.06)	0.06	0.06
Ghana	GHANA (39)												
Ghana	iLiNS−DYADG (40)	267	549	12.4	−0.01 (−0.06, 0.04)	0.06	0.10	80	144	15.3	−0.08 (−0.17, 0.01)	0.03	0.03
Haiti	HAITI (41)												
Kenya	WASH−B (42)	705	2451	6.7	0.00 (−0.03, 0.02)	0.26	0.18	754	2710	13.1	−0.04 (−0.07, −0.02)	0.35	0.35
Madagascar	MAHAY (43)	352	431	21.1	0.01 (−0.05, 0.07)	0.03	0.08	1350	1254	27.7	0.01 (−0.04, 0.05)	0.10	0.10
Malawi	iLiNS−DYADM (44)												
Malawi	iLiNS−DOSE (45)												
Mali	PROMIS (46)												
Mali	PROMIS CS (46)	107	96	19.8	−0.09 (−0.20, 0.03)	0.01	0.03	838	864	22.8	−0.02 (−0.06, 0.03)	0.10	0.10
Zimbabwe	SHINE (HIV−) (47)	1707	1636	10.5	−0.03 (−0.05, −0.01)	0.34	0.19	68	61	13.1	−0.01 (−0.12, 0.10)	0.02	0.02
Zimbabwe	SHINE (HIV+) (48)												
		**7121**	**9070**		**I² = 0.56, Tau² = 0.00**			**6029**	**7977**		**I² = 0.00, Tau² = 0.00**		
**Fixed**					**−0.03 (−0.04, −0.02)**						**−0.03 (−0.05, −0.02)**		
**Random**					**−0.03 (−0.05, −0.01)**						**−0.03 (−0.05, −0.02)**		

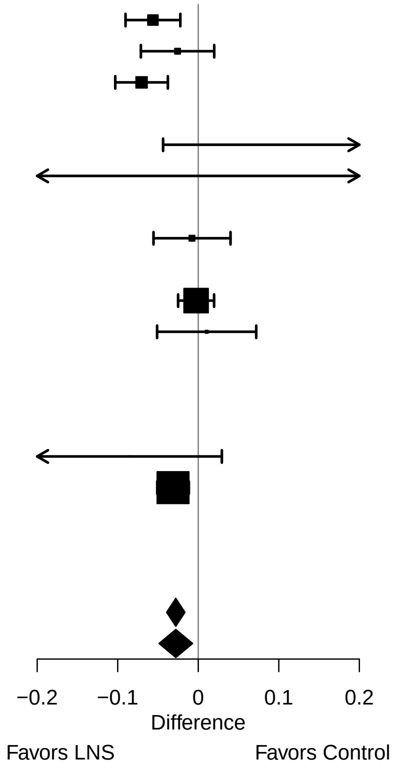

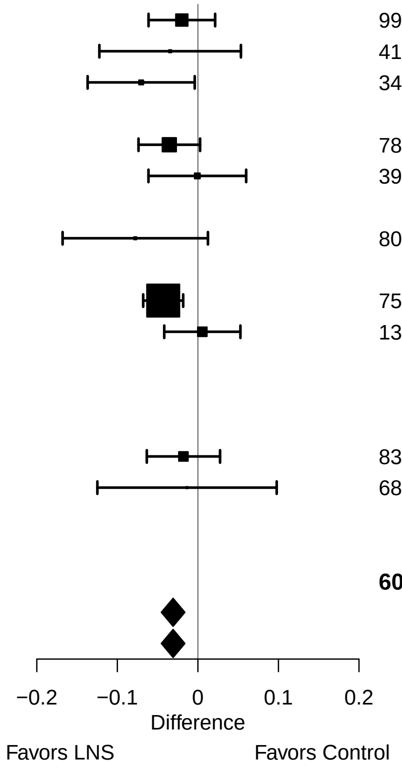

Supplemental figure 8N: Underweight prevalence difference

8N5: Stratified by Maternal depressive symptoms

P−for−interaction = 0.717
Difference in PDs = 0.00 (−0.02, 0.03)

		Less than 75th percentile						At least 75th percentile					
**Country**	**Trial**	**LNS N**	**Control N**	**Control Prevalence**	**PD (95% CI)**	**Fixed W**	**Random W**	**LNS N**	**Control N**	**Control Prevalence**	**PD (95% CI)**	**Fixed W**	**Random W**
Bangladesh	JiVitA−4 (34)												
Bangladesh	RDNS (35)	1081	472	36.9	−0.03 (−0.07, 0.01)	0.08	0.09	520	288	38.2	−0.03 (−0.11, 0.05)	0.05	0.07
Bangladesh	WASH−B (36)	899	2487	31.6	−0.07 (−0.10, −0.03)	0.10	0.11	240	883	34.7	−0.08 (−0.15, 0.00)	0.07	0.08
Burkina Faso	iLiNS−Zinc (37)												
Burkina Faso	PROMIS (38)	505	571	19.4	−0.04 (−0.09, 0.01)	0.04	0.06	220	213	21.1	−0.01 (−0.07, 0.05)	0.11	0.12
Burkina Faso	PROMIS CS (38)												
Ghana	GHANA (39)												
Ghana	iLiNS−DYADG (40)	252	460	12.4	−0.04 (−0.08, 0.01)	0.05	0.07	83	209	12.9	0.04 (−0.05, 0.13)	0.05	0.06
Haiti	HAITI (41)												
Kenya	WASH−B (42)	997	3506	9.6	−0.02 (−0.03, 0.00)	0.39	0.29	342	1248	10.5	−0.03 (−0.07, 0.01)	0.25	0.21
Madagascar	MAHAY (43)	673	721	27.2	0.00 (−0.06, 0.05)	0.04	0.05	283	252	24.2	0.05 (−0.03, 0.14)	0.05	0.07
Malawi	iLiNS−DYADM (44)	157	303	11.2	0.00 (−0.06, 0.06)	0.03	0.04	47	113	12.4	0.03 (−0.09, 0.14)	0.03	0.04
Malawi	iLiNS−DOSE (45)												
Mali	PROMIS (46)	374	372	15.8	−0.04 (−0.10, 0.01)	0.04	0.06	122	129	18.3	−0.01 (−0.09, 0.06)	0.06	0.08
Mali	PROMIS CS (46)												
Zimbabwe	SHINE (HIV−) (47)	1192	1137	9.9	−0.02 (−0.05, 0.00)	0.19	0.19	509	490	12.7	−0.06 (−0.09, −0.02)	0.30	0.23
Zimbabwe	SHINE (HIV+) (48)	234	214	16.4	0.02 (−0.05, 0.08)	0.03	0.04	88	100	20.0	−0.03 (−0.14, 0.08)	0.03	0.04
		**6364**	**10243**		**I² = 0.11, Tau² = 0.00**			**2454**	**3925**		**I² = 0.25, Tau² = 0.00**		
**Fixed**					**−0.03 (−0.04, −0.01)**						**−0.03 (−0.05, −0.01)**		
**Random**					**−0.03 (−0.04, −0.01)**						**−0.02 (−0.05, 0.00)**		

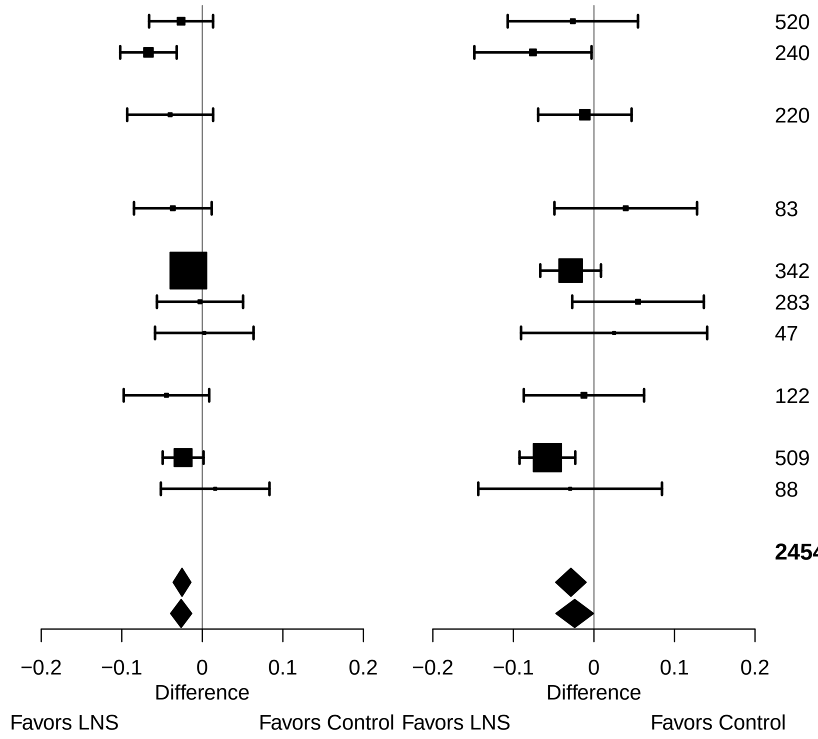

Supplemental figure 8N: Underweight prevalence difference

8N6: Stratified by Child sex

P−for−interaction = 0.231
Difference in PDs = 0.01 (−0.01, 0.03)

Country	Trial	LNS N	Control N	Control Prevalence	Male PD (95% CI)	Fixed W	Random W	LNS N	Control N	Control Prevalence	Female PD (95% CI)	Fixed W	Random W
Bangladesh	JiVitA−4 (34)	1440	594	38.2	−0.03 (−0.06, 0.01)	0.11	0.11	1374	638	40.1	−0.06 (−0.09, −0.02)	0.09	0.08
Bangladesh	RDNS (35)	833	411	38.0	−0.02 (−0.08, 0.04)	0.04	0.04	830	404	37.6	−0.04 (−0.09, 0.01)	0.05	0.07
Bangladesh	WASH−B (36)	567	1730	33.2	−0.07 (−0.11, −0.03)	0.08	0.08	596	1731	31.7	−0.06 (−0.11, −0.02)	0.05	0.07
Burkina Faso	iLiNS−Zinc (37)	995	336	32.4	−0.08 (−0.12, −0.03)	0.06	0.06	965	330	29.4	−0.12 (−0.17, −0.08)	0.05	0.07
Burkina Faso	PROMIS (38)	435	482	24.9	−0.05 (−0.10, 0.01)	0.05	0.05	422	429	13.8	0.00 (−0.05, 0.05)	0.04	0.06
Burkina Faso	PROMIS CS (38)	234	221	27.6	0.01 (−0.08, 0.10)	0.02	0.02	196	215	20.9	−0.03 (−0.10, 0.05)	0.02	0.04
Ghana	GHANA (39)												
Ghana	iLiNS−DYADG (40)	175	324	16.4	−0.04 (−0.11, 0.02)	0.03	0.03	172	369	10.0	−0.01 (−0.06, 0.05)	0.04	0.06
Haiti	HAITI (41)												
Kenya	WASH−B (42)	734	2457	11.8	−0.03 (−0.05, −0.01)	0.24	0.24	727	2708	8.4	−0.02 (−0.04, 0.00)	0.31	0.10
Madagascar	MAHAY (43)	826	835	31.4	0.00 (−0.05, 0.05)	0.05	0.05	876	850	20.7	0.02 (−0.02, 0.07)	0.05	0.07
Malawi	iLiNS−DYADM (44)	107	207	12.6	0.05 (−0.03, 0.13)	0.02	0.02	113	237	11.0	−0.02 (−0.09, 0.05)	0.02	0.05
Malawi	iLiNS−DOSE (45)	353	124	29.0	−0.05 (−0.12, 0.03)	0.03	0.03	349	117	10.3	0.02 (−0.05, 0.08)	0.03	0.05
Mali	PROMIS (46)	265	262	18.6	−0.04 (−0.10, 0.01)	0.05	0.05	235	240	14.0	−0.03 (−0.09, 0.03)	0.03	0.06
Mali	PROMIS CS (46)	474	521	25.1	−0.03 (−0.10, 0.04)	0.03	0.03	472	439	19.4	−0.02 (−0.07, 0.04)	0.04	0.06
Zimbabwe	SHINE (HIV−) (47)	924	906	13.4	−0.05 (−0.07, −0.02)	0.18	0.18	945	880	7.7	−0.01 (−0.04, 0.01)	0.18	0.09
Zimbabwe	SHINE (HIV+) (48)	161	167	22.8	−0.05 (−0.13, 0.03)	0.02	0.02	175	161	11.2	0.05 (−0.02, 0.13)	0.02	0.05
		**8523**	**9577**		**I² = 0.01, Tau² = 0.00**			**8447**	**9748**		**I² = 0.63, Tau² = 0.00**		
**Fixed**					**−0.04 (−0.05, −0.03)**						**−0.03 (−0.04, −0.01)**		
**Random**					**−0.04 (−0.05, −0.03)**						**−0.02 (−0.05, 0.00)**		

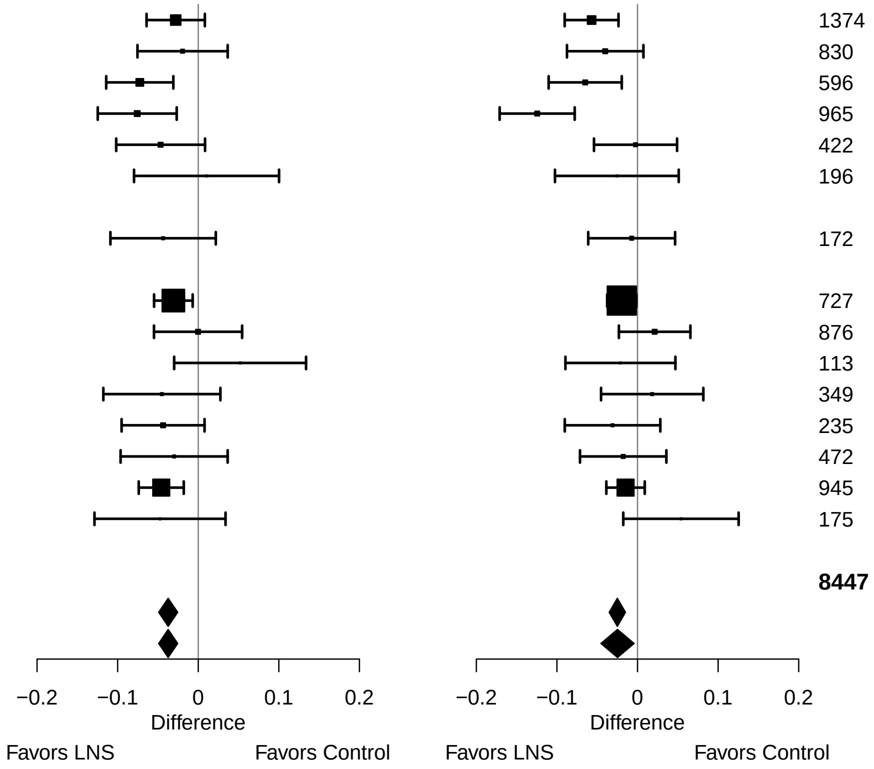

Supplemental figure 8N: Underweight prevalence difference

8N7: Stratified by Child birth order

P−for−interaction = 0.043
Difference in PDs = 0.02 (0.00, 0.04)

Country	Trial	Later born LNS N	Later born Control N	Later born Control Prevalence	Later born PD (95% CI)	Later born Fixed W	Later born Random W	Firstborn LNS N	Firstborn Control N	Firstborn Control Prevalence	Firstborn PD (95% CI)	Firstborn Fixed W	Firstborn Random W
Bangladesh	JiVitA−4 (34)	600	250	43.3	−0.07 (−0.12, −0.02)	0.04	0.06	2210	976	38.0	−0.04 (−0.06, −0.01)	0.26	0.18
Bangladesh	RDNS (35)	974	508	38.0	−0.03 (−0.09, 0.02)	0.04	0.06	688	307	37.5	−0.02 (−0.07, 0.03)	0.08	0.10
Bangladesh	WASH−B (36)	737	2310	34.3	−0.08 (−0.12, −0.04)	0.06	0.07	426	1151	28.6	−0.05 (−0.10, 0.00)	0.08	0.09
Burkina Faso	iLiNS−Zinc (37)	1524	521	30.9	−0.11 (−0.15, −0.08)	0.07	0.08	436	145	31.0	−0.06 (−0.13, 0.02)	0.04	0.05
Burkina Faso	PROMIS (38)	709	750	19.9	−0.04 (−0.08, 0.00)	0.07	0.08	148	159	18.9	0.03 (−0.07, 0.12)	0.02	0.03
Burkina Faso	PROMIS CS (38)	366	357	23.8	0.00 (−0.07, 0.06)	0.02	0.05	63	79	26.6	0.00 (−0.13, 0.14)	0.01	0.02
Ghana	GHANA (39)												
Ghana	iLiNS−DYADG (40)	237	459	12.2	0.00 (−0.06, 0.05)	0.04	0.06	110	234	14.5	−0.06 (−0.14, 0.01)	0.04	0.05
Haiti	HAITI (41)												
Kenya	WASH−B (42)	1137	4065	10.2	−0.02 (−0.04, −0.01)	0.28	0.10	322	1095	9.2	−0.02 (−0.05, 0.01)	0.20	0.16
Madagascar	MAHAY (43)	1271	1220	27.2	−0.01 (−0.05, 0.04)	0.05	0.07	431	465	22.8	0.05 (−0.02, 0.12)	0.04	0.06
Malawi	iLiNS−DYADM (44)	173	353	11.3	0.02 (−0.04, 0.08)	0.03	0.05	47	89	12.4	0.00 (−0.11, 0.12)	0.01	0.02
Malawi	iLiNS−DOSE (45)	463	165	19.4	−0.01 (−0.07, 0.05)	0.03	0.05	150	45	22.2	−0.07 (−0.19, 0.04)	0.02	0.02
Mali	PROMIS (46)	437	441	16.9	−0.05 (−0.09, 0.00)	0.05	0.07	62	59	11.9	0.06 (−0.05, 0.16)	0.02	0.03
Mali	PROMIS CS (46)	784	822	22.5	−0.03 (−0.08, 0.01)	0.05	0.07	145	126	20.6	0.01 (−0.08, 0.10)	0.02	0.04
Zimbabwe	SHINE (HIV−) (47)	1295	1220	10.4	−0.04 (−0.07, −0.02)	0.16	0.09	487	483	11.2	−0.01 (−0.05, 0.03)	0.14	0.13
Zimbabwe	SHINE (HIV+) (48)	279	245	14.3	0.04 (−0.03, 0.10)	0.02	0.05	54	78	25.6	−0.13 (−0.24, −0.01)	0.02	0.02
		**10986**	**13686**		**I² = 0.62, Tau² = 0.00**			**5779**	**5491**		**I² = 0.19, Tau² = 0.00**		
**Fixed**					**−0.04 (−0.05, −0.03)**						**−0.02 (−0.04, −0.01)**		
**Random**					**−0.03 (−0.05, −0.01)**						**−0.02 (−0.04, 0.00)**		

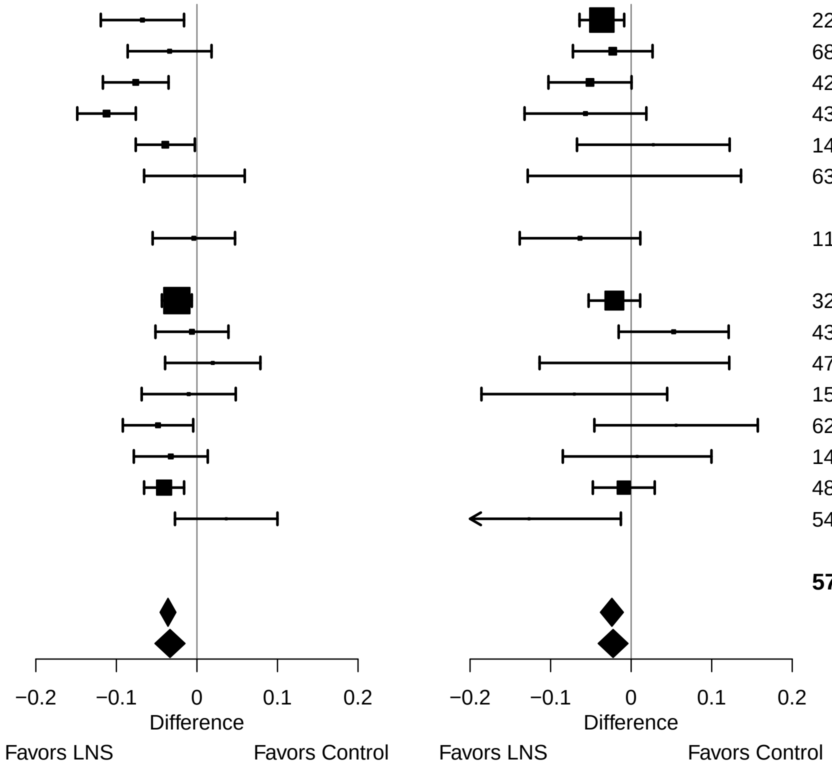

Supplemental figure 8N: Underweight prevalence difference

8N8: Stratified by Child baseline anthropometric status

P−for−interaction = 0.004
Difference in PDs = −0.04 (−0.07, −0.01)

		WAZ at least −1 Z						WAZ less than −1 Z					
Country	Trial	LNS N	Control N	Control Prevalence	PD (95% CI)	Fixed W	Random W	LNS N	Control N	Control Prevalence	PD (95% CI)	Fixed W	Random W
Bangladesh	JiVitA−4 (34)	1198	531	6.4	−0.02 (−0.05, 0.00)	0.13	0.13	1616	701	64.3	−0.07 (−0.11, −0.03)	0.35	0.35
Bangladesh	RDNS (35)	780	338	5.6	0.01 (−0.02, 0.03)	0.09	0.09	821	422	62.8	−0.01 (−0.06, 0.04)	0.23	0.23
Bangladesh	WASH−B (36)												
Burkina Faso	iLiNS−Zinc (37)												
Burkina Faso	PROMIS (38)	427	482	5.8	−0.01 (−0.04, 0.01)	0.10	0.10	268	267	42.7	−0.05 (−0.13, 0.02)	0.11	0.11
Burkina Faso	PROMIS CS (38)												
Ghana	GHANA (39)												
Ghana	iLiNS−DYADG (40)	222	424	1.7	0.01 (−0.02, 0.03)	0.15	0.15	101	215	34.0	−0.02 (−0.13, 0.09)	0.05	0.05
Haiti	HAITI (41)												
Kenya	WASH−B (42)												
Madagascar	MAHAY (43)												
Malawi	iLiNS−DYADM (44)												
Malawi	iLiNS−DOSE (45)	419	143	3.5	0.00 (−0.04, 0.03)	0.06	0.06	283	98	43.9	−0.02 (−0.13, 0.10)	0.05	0.05
Mali	PROMIS (46)												
Mali	PROMIS CS (46)												
Zimbabwe	SHINE (HIV−) (47)	917	836	1.9	0.00 (−0.02, 0.01)	0.45	0.45	383	349	30.1	−0.09 (−0.15, −0.03)	0.18	0.18
Zimbabwe	SHINE (HIV+) (48)	151	144	5.6	−0.01 (−0.06, 0.04)	0.03	0.03	113	92	32.6	0.01 (−0.12, 0.14)	0.04	0.04
		**4114**	**2898**		**I^2 = 0.00, Tau^2 = 0.00**			**3585**	**2144**		**I^2 = 0.00, Tau^2 = 0.00**		
**Fixed**					**0.00 (−0.01, 0.00)**						**−0.05 (−0.08, −0.03)**		
**Random**					**0.00 (−0.01, 0.00)**						**−0.05 (−0.08, −0.03)**		

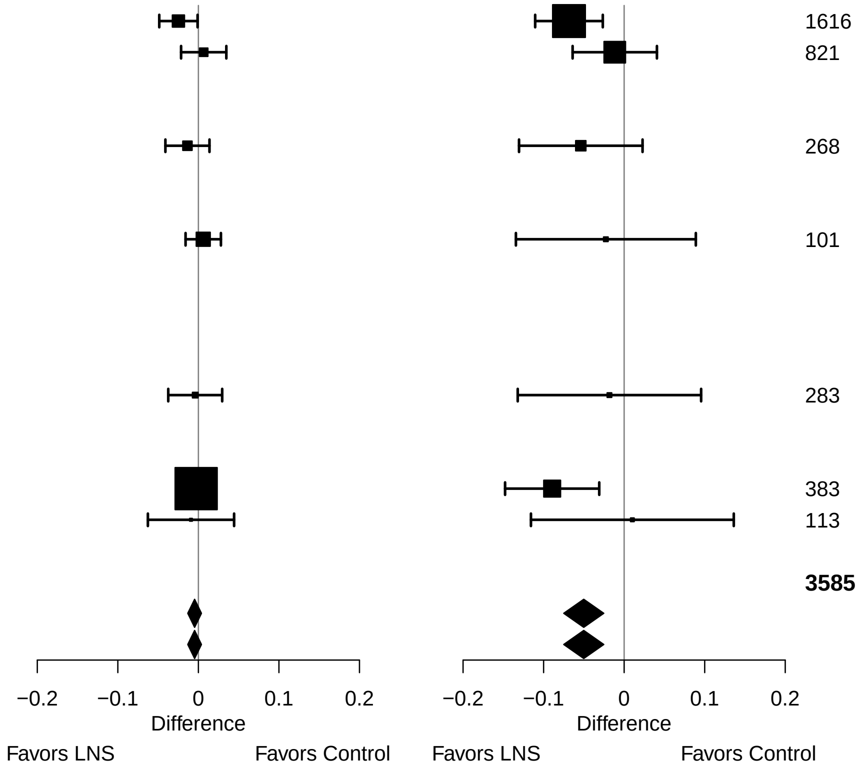

Supplemental figure 8O: Mean difference in HCZ

8O1: Stratified by Maternal height

P−for−interaction = 0.185
Difference in MDs = 0.05 (−0.03, 0.13)

		At least 150.1 cm							Less than 150.1 cm					
Country	Trial	LNS N	Control N	Control Mean	MD (95% CI)	Fixed W	Random W		LNS N	Control N	Control Mean	MD (95% CI)	Fixed W	Random W
Bangladesh	JiVitA−4 (34)													
Bangladesh	RDNS (35)	868	432	−1.74	0.09 (−0.01, 0.18)	0.09	0.11		735	358	−2.01	0.13 (0.03, 0.23)	0.37	0.20
Bangladesh	WASH−B (36)	619	1899	−1.48	0.09 (0.00, 0.17)	0.12	0.13		544	1559	−1.78	0.17 (0.09, 0.26)	0.45	0.20
Burkina Faso	iLiNS−Zinc (37)	1911	649	−1.83	0.16 (0.10, 0.23)	0.22	0.18		33	15	−2.10	0.23 (−0.07, 0.53)	0.04	0.12
Burkina Faso	PROMIS (38)													
Burkina Faso	PROMIS CS (38)													
Ghana	GHANA (39)													
Ghana	iLiNS−DYADG (40)	326	645	−1.13	0.07 (−0.05, 0.18)	0.06	0.08		16	36	−1.44	0.22 (−0.35, 0.78)	0.01	0.05
Haiti	HAITI (41)													
Kenya	WASH−B (42)	1316	4720	−0.26	0.04 (−0.02, 0.10)	0.24	0.19		57	194	−0.52	−0.02 (−0.36, 0.32)	0.03	0.10
Madagascar	MAHAY (43)													
Malawi	iLiNS−DYADM (44)	192	381	−0.75	−0.09 (−0.27, 0.08)	0.03	0.04		28	60	−0.91	−0.41 (−0.78, −0.04)	0.03	0.09
Malawi	iLiNS−DOSE (45)	581	195	−1.03	0.07 (−0.07, 0.20)	0.05	0.07		111	42	−1.60	0.29 (−0.03, 0.60)	0.04	0.11
Mali	PROMIS (46)													
Mali	PROMIS CS (46)													
Zimbabwe	SHINE (HIV−) (47)	1746	1687	−0.26	0.12 (0.04, 0.19)	0.16	0.15		76	62	−0.65	−0.20 (−0.59, 0.20)	0.02	0.09
Zimbabwe	SHINE (HIV+) (48)	314	312	−0.52	0.10 (−0.09, 0.28)	0.03	0.04		18	12	−1.28	0.53 (−0.27, 1.33)	0.01	0.03
		**7873**	**10920**		**I² = 0.39, Tau² = 0.00**				**1618**	**2338**		**I² = 0.46, Tau² = 0.03**		
**Fixed**					**0.09 (0.06, 0.12)**							**0.14 (0.08, 0.20)**		
**Random**					**0.08 (0.05, 0.12)**							**0.09 (−0.06, 0.24)**		

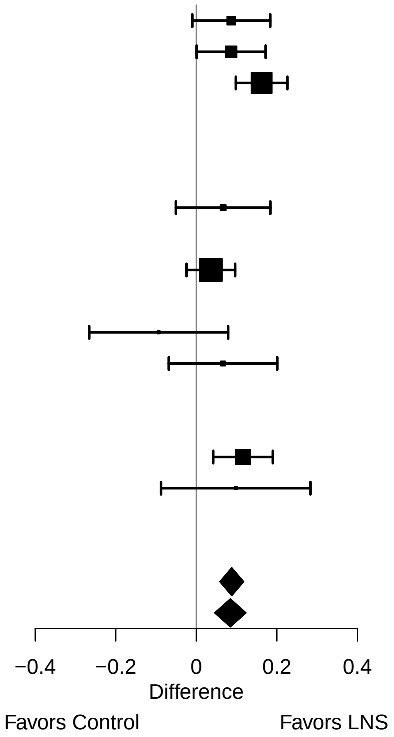

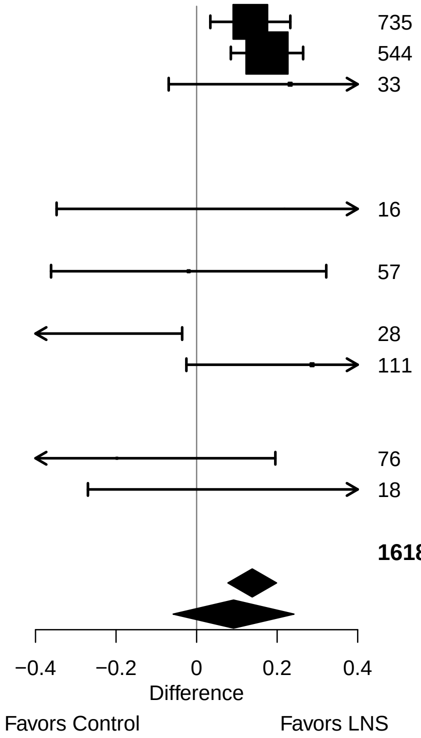

Supplemental figure 8O: Mean difference in HCZ

8O2: Stratified by Maternal BMI

P−for−interaction = 0.407
Difference in MDs = 0.02 (−0.03, 0.07)

		At least 20 kg/m²						Less than 20 kg/m²					
**Country**	**Trial**	**LNS N**	**Control N**	**Control Mean**	**MD (95% CI)**	**Fixed W**	**Random W**	**LNS N**	**Control N**	**Control Mean**	**MD (95% CI)**	**Fixed W**	**Random W**
Bangladesh	JiVitA−4 (34)												
Bangladesh	RDNS (35)	730	335	−1.76	0.09 (−0.02, 0.20)	0.09	0.12	873	455	−1.94	0.11 (0.03, 0.19)	0.24	0.24
Bangladesh	WASH−B (36)	519	1595	−1.50	0.09 (−0.01, 0.19)	0.10	0.12	644	1863	−1.70	0.15 (0.08, 0.22)	0.31	0.31
Burkina Faso	iLiNS−Zinc (37)	1210	389	−1.81	0.17 (0.11, 0.23)	0.29	0.16	734	275	−1.88	0.16 (0.07, 0.24)	0.24	0.24
Burkina Faso	PROMIS (38)												
Burkina Faso	PROMIS CS (38)												
Ghana	GHANA (39)												
Ghana	iLiNS−DYADG (40)	305	562	−1.12	0.03 (−0.09, 0.16)	0.07	0.11	37	119	−1.26	0.34 (0.01, 0.67)	0.02	0.02
Haiti	HAITI (41)												
Kenya	WASH−B (42)	1100	3831	−0.22	0.02 (−0.05, 0.09)	0.21	0.15	273	1083	−0.46	0.07 (−0.08, 0.21)	0.08	0.08
Madagascar	MAHAY (43)												
Malawi	iLiNS−DYADM (44)	131	263	−0.67	−0.21 (−0.41, −0.01)	0.03	0.06	88	178	−0.92	0.01 (−0.26, 0.27)	0.02	0.02
Malawi	iLiNS−DOSE (45)	512	172	−1.03	0.10 (−0.04, 0.24)	0.05	0.09	178	64	−1.41	0.14 (−0.10, 0.39)	0.03	0.03
Mali	PROMIS (46)												
Mali	PROMIS CS (46)												
Zimbabwe	SHINE (HIV−) (47)	1358	1298	−0.25	0.09 (0.01, 0.18)	0.15	0.14	239	248	−0.44	0.08 (−0.11, 0.26)	0.05	0.05
Zimbabwe	SHINE (HIV+) (48)	247	254	−0.53	0.16 (−0.06, 0.38)	0.02	0.05	62	52	−0.60	−0.05 (−0.38, 0.28)	0.02	0.02
		**6112**	**8699**		**I² = 0.62, Tau² = 0.01**			**3128**	**4337**		**I² = 0.00, Tau² = 0.00**		
**Fixed**					**0.09 (0.06, 0.12)**						**0.13 (0.09, 0.17)**		
**Random**					**0.07 (0.01, 0.13)**						**0.13 (0.09, 0.17)**		

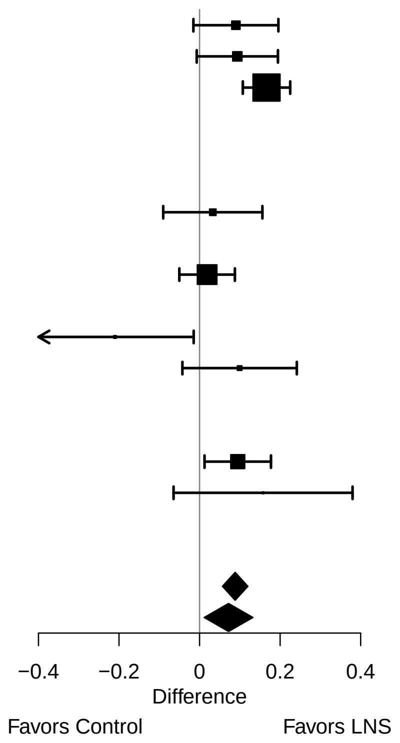

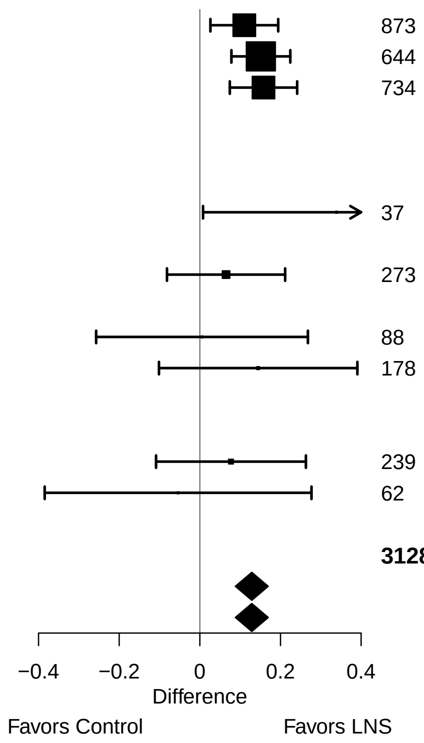

Supplemental figure 8O: Mean difference in HCZ

8O3: Stratified by Maternal age

P−for−interaction = 0.901
Difference in MDs = 0.00 (−0.04, 0.04)

Country	Trial	At least 25 y LNS N	Control N	Control Mean	MD (95% CI)	Fixed W	Random W	Less than 25 y LNS N	Control N	Control Mean	MD (95% CI)	Fixed W	Random W
Bangladesh	JiVitA−4 (34)	1238	554	−1.43	0.06 (−0.01, 0.12)	0.27	0.27	1634	708	−1.45	0.06 (0.00, 0.12)	0.25	0.15
Bangladesh	RDNS (35)	453	221	−1.83	0.13 (0.00, 0.26)	0.06	0.06	1210	594	−1.88	0.09 (0.00, 0.18)	0.11	0.13
Bangladesh	WASH−B (36)	492	1523	−1.60	0.09 (−0.01, 0.19)	0.10	0.10	666	1924	−1.63	0.15 (0.06, 0.23)	0.10	0.12
Burkina Faso	iLiNS−Zinc (37)	1128	399	−1.78	0.16 (0.07, 0.24)	0.15	0.15	805	262	−1.92	0.17 (0.12, 0.23)	0.28	0.15
Burkina Faso	PROMIS (38)												
Burkina Faso	PROMIS CS (38)												
Ghana	GHANA (39)	69	63	−0.67	0.29 (−0.03, 0.61)	0.01	0.01	29	25	−0.38	−0.09 (−0.58, 0.41)	0.00	0.01
Ghana	iLiNS−DYADG (40)	220	417	−1.12	0.02 (−0.12, 0.16)	0.05	0.05	127	275	−1.18	0.15 (−0.05, 0.34)	0.02	0.06
Haiti	HAITI (41)												
Kenya	WASH−B (42)	845	2831	−0.23	0.06 (−0.02, 0.13)	0.18	0.18	601	2313	−0.35	0.02 (−0.06, 0.11)	0.11	0.12
Madagascar	MAHAY (43)												
Malawi	iLiNS−DYADM (44)	106	226	−0.72	0.03 (−0.20, 0.25)	0.02	0.02	114	218	−0.83	−0.26 (−0.49, −0.04)	0.02	0.05
Malawi	iLiNS−DOSE (45)	368	134	−1.11	0.05 (−0.12, 0.22)	0.04	0.04	310	97	−1.16	0.19 (0.01, 0.38)	0.02	0.07
Mali	PROMIS (46)												
Mali	PROMIS CS (46)												
Zimbabwe	SHINE (HIV−) (47)	869	795	−0.19	0.10 (−0.01, 0.21)	0.09	0.09	805	792	−0.36	0.09 (−0.02, 0.19)	0.08	0.11
Zimbabwe	SHINE (HIV+) (48)	244	231	−0.49	0.05 (−0.16, 0.26)	0.02	0.02	73	78	−0.69	0.10 (−0.23, 0.44)	0.01	0.03
		**6032**	**7394**		**I² = 0.00, Tau² = 0.00**			**6374**	**7286**		**I² = 0.59, Tau² = 0.01**		
**Fixed**					**0.08 (0.05, 0.12)**						**0.10 (0.07, 0.13)**		
**Random**					**0.08 (0.05, 0.12)**						**0.09 (0.02, 0.15)**		

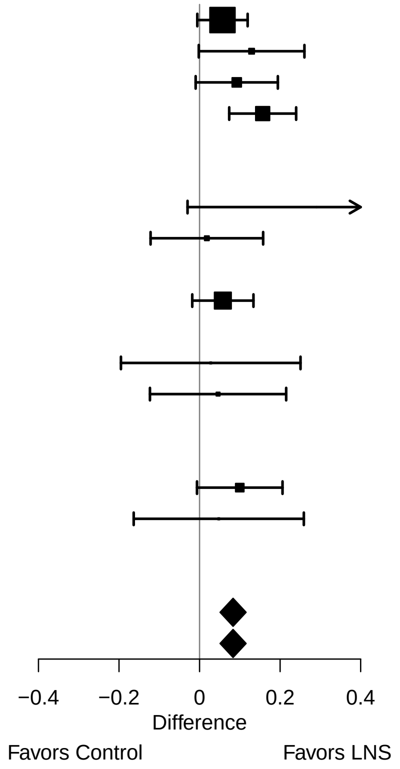

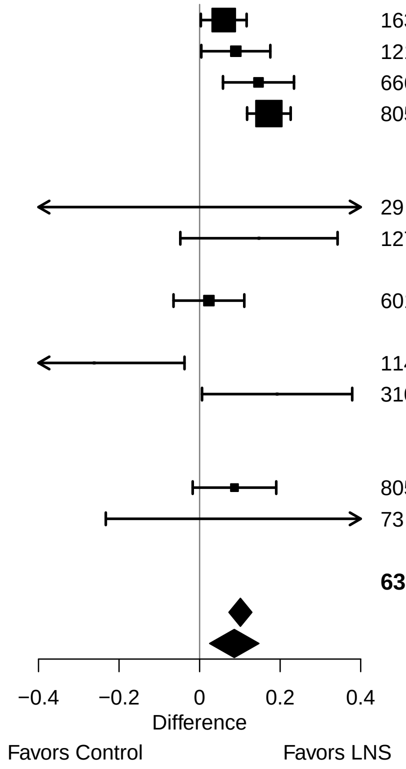

Supplemental figure 8O: Mean difference in HCZ

8O4: Stratified by Maternal education

P−for−interaction = 0.711
Difference in MDs = 0.01 (−0.04, 0.06)

Primary or greater

Country	Trial	LNS N	Control N	Control Mean	MD (95% CI)	Fixed W	Random W
Bangladesh	JiVitA−4 (34)	1855	775	−1.38	0.08 (0.02, 0.13)	0.29	0.12
Bangladesh	RDNS (35)	1245	589	−1.85	0.12 (0.04, 0.20)	0.14	0.11
Bangladesh	WASH−B (36)	817	2477	−1.55	0.13 (0.05, 0.20)	0.16	0.11
Burkina Faso	iLiNS−Zinc (37)	82	16	−1.65	0.03 (−0.16, 0.22)	0.03	0.08
Burkina Faso	PROMIS (38)						
Burkina Faso	PROMIS CS (38)						
Ghana	GHANA (39)	91	75	−0.64	0.29 (0.01, 0.57)	0.01	0.06
Ghana	iLiNS−DYADG (40)	267	548	−1.12	0.05 (−0.08, 0.18)	0.06	0.10
Haiti	HAITI (41)						
Kenya	WASH−B (42)	703	2456	−0.15	−0.05 (−0.14, 0.04)	0.12	0.11
Madagascar	MAHAY (43)						
Malawi	iLiNS−DYADM (44)	35	69	−0.56	−0.55 (−0.91, −0.18)	0.01	0.04
Malawi	iLiNS−DOSE (45)	163	55	−1.02	0.25 (−0.01, 0.50)	0.01	0.07
Mali	PROMIS (46)						
Mali	PROMIS CS (46)						
Zimbabwe	SHINE (HIV−) (47)	1708	1633	−0.28	0.11 (0.03, 0.18)	0.16	0.11
Zimbabwe	SHINE (HIV+) (48)	301	293	−0.51	0.06 (−0.13, 0.25)	0.03	0.08
		**7267**	**8986**		**I² = 0.62, Tau² = 0.02**		
**Fixed**					**0.08 (0.05, 0.11)**		
**Random**					**0.07 (−0.03, 0.16)**		

Incomplete or no formal

Country	Trial	LNS N	Control N	Control Mean	MD (95% CI)	Fixed W	Random W
Bangladesh	JiVitA−4 (34)	1028	489	−1.54	0.03 (−0.04, 0.09)	0.25	0.17
Bangladesh	RDNS (35)	418	226	−1.89	0.04 (−0.10, 0.18)	0.06	0.10
Bangladesh	WASH−B (36)	346	986	−1.76	0.12 (0.00, 0.24)	0.08	0.12
Burkina Faso	iLiNS−Zinc (37)	1851	645	−1.84	0.17 (0.11, 0.23)	0.31	0.18
Burkina Faso	PROMIS (38)						
Burkina Faso	PROMIS CS (38)						
Ghana	GHANA (39)	7	15	−0.31	−0.81 (−1.71, 0.10)	0.00	0.00
Ghana	iLiNS−DYADG (40)	80	144	−1.22	0.13 (−0.11, 0.37)	0.02	0.05
Haiti	HAITI (41)						
Kenya	WASH−B (42)	756	2716	−0.40	0.12 (0.05, 0.20)	0.18	0.16
Madagascar	MAHAY (43)						
Malawi	iLiNS−DYADM (44)	184	372	−0.81	−0.04 (−0.22, 0.13)	0.04	0.08
Malawi	iLiNS−DOSE (45)	517	176	−1.16	0.07 (−0.07, 0.21)	0.06	0.10
Mali	PROMIS (46)						
Mali	PROMIS CS (46)						
Zimbabwe	SHINE (HIV−) (47)	69	61	−0.32	−0.15 (−0.52, 0.23)	0.01	0.02
Zimbabwe	SHINE (HIV+) (48)	21	18	−1.17	0.47 (−0.20, 1.14)	0.00	0.01
		**5277**	**5848**		**I² = 0.51, Tau² = 0.00**		
**Fixed**					**0.10 (0.06, 0.13)**		
**Random**					**0.08 (0.02, 0.14)**		

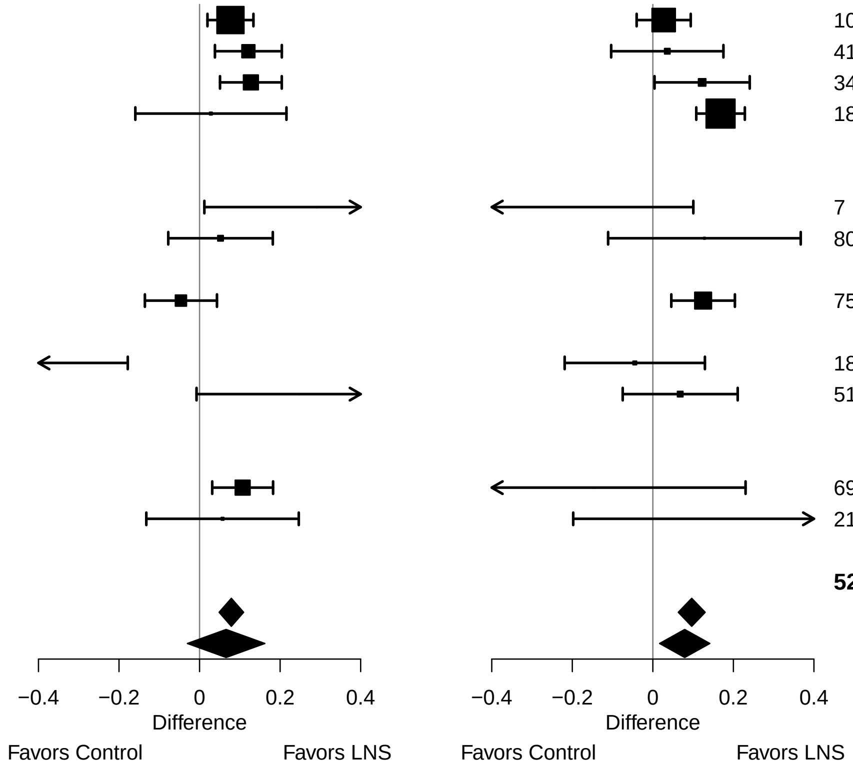

Supplemental figure 8O: Mean difference in HCZ

8O5: Stratified by Maternal depressive symptoms

P−for−interaction = **0.386**
Difference in MDs = **−0.03 (−0.10, 0.04)**

Less than 75th percentile

Country	Trial	LNS N	Control N	Control Mean	MD (95% CI)	Fixed W	Random W
Bangladesh	JiVitA−4 (34)						
Bangladesh	RDNS (35)	1081	472	−1.81	0.04 (−0.03, 0.12)	0.22	0.20
Bangladesh	WASH−B (36)	901	2487	−1.60	0.13 (0.06, 0.20)	0.27	0.22
Burkina Faso	iLiNS−Zinc (37)						
Burkina Faso	PROMIS (38)						
Burkina Faso	PROMIS CS (38)						
Ghana	GHANA (39)						
Ghana	iLiNS−DYADG (40)	252	459	−1.13	0.07 (−0.07, 0.20)	0.07	0.10
Haiti	HAITI (41)						
Kenya	WASH−B (42)	998	3517	−0.27	0.05 (−0.02, 0.13)	0.23	0.21
Madagascar	MAHAY (43)						
Malawi	iLiNS−DYADM (44)	157	303	−0.74	−0.15 (−0.33, 0.04)	0.04	0.06
Malawi	iLiNS−DOSE (45)						
Mali	PROMIS (46)						
Mali	PROMIS CS (46)						
Zimbabwe	SHINE (HIV−) (47)	1193	1135	−0.24	0.06 (−0.03, 0.15)	0.15	0.16
Zimbabwe	SHINE (HIV+) (48)	234	215	−0.52	0.01 (−0.20, 0.23)	0.03	0.05
		**4816**	**8588**		**I² = 0.36, Tau² = 0.00**		
**Fixed**					**0.07 (0.03, 0.10)**		
**Random**					**0.06 (0.01, 0.11)**		

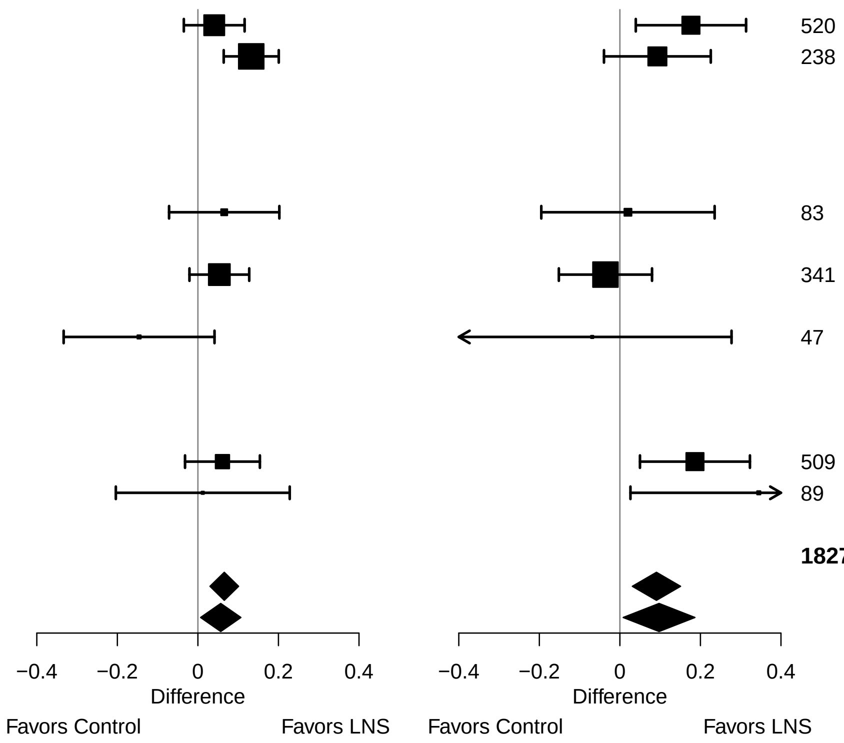

At least 75th percentile

Country	Trial	LNS N	Control N	Control Mean	MD (95% CI)	Fixed W	Random W
Bangladesh	JiVitA−4 (34)						
Bangladesh	RDNS (35)	520	288	−1.95	0.18 (0.04, 0.31)	0.19	0.18
Bangladesh	WASH−B (36)	238	885	−1.61	0.09 (−0.04, 0.23)	0.20	0.19
Burkina Faso	iLiNS−Zinc (37)						
Burkina Faso	PROMIS (38)						
Burkina Faso	PROMIS CS (38)						
Ghana	GHANA (39)						
Ghana	iLiNS−DYADG (40)	83	209	−1.15	0.02 (−0.20, 0.24)	0.08	0.11
Haiti	HAITI (41)						
Kenya	WASH−B (42)	341	1248	−0.29	−0.04 (−0.15, 0.08)	0.27	0.21
Madagascar	MAHAY (43)						
Malawi	iLiNS−DYADM (44)	47	113	−0.84	−0.07 (−0.41, 0.28)	0.03	0.06
Malawi	iLiNS−DOSE (45)						
Mali	PROMIS (46)						
Mali	PROMIS CS (46)						
Zimbabwe	SHINE (HIV−) (47)	509	490	−0.35	0.19 (0.05, 0.32)	0.19	0.18
Zimbabwe	SHINE (HIV+) (48)	89	100	−0.64	0.34 (0.03, 0.66)	0.04	0.06
		**1827**	**3333**		**I² = 0.49, Tau² = 0.01**		
**Fixed**					**0.09 (0.03, 0.15)**		
**Random**					**0.10 (0.01, 0.19)**		

Supplemental figure 8O: Mean difference in HCZ

8O6: Stratified by Child sex

P−for−interaction = 0.307
Difference in MDs = 0.02 (−0.02, 0.06)

Country	Trial	Male LNS N	Male Control N	Male Control Mean	Male MD (95% CI)	Male Fixed W	Male Random W	Female LNS N	Female Control N	Female Control Mean	Female MD (95% CI)	Female Fixed W	Female Random W
Bangladesh	JiVitA−4 (34)	1475	612	−1.45	0.07 (0.02, 0.13)	0.30	0.24	1410	655	−1.44	0.04 (−0.02, 0.10)	0.25	0.14
Bangladesh	RDNS (35)	833	411	−1.78	0.00 (−0.11, 0.11)	0.09	0.10	830	404	−1.96	0.20 (0.10, 0.30)	0.08	0.11
Bangladesh	WASH−B (36)	566	1730	−1.60	0.09 (0.00, 0.19)	0.11	0.12	597	1733	−1.63	0.15 (0.07, 0.24)	0.12	0.12
Burkina Faso	iLiNS−Zinc (37)	991	335	−1.89	0.14 (0.05, 0.23)	0.12	0.13	955	329	−1.79	0.19 (0.13, 0.25)	0.24	0.14
Burkina Faso	PROMIS (38)												
Burkina Faso	PROMIS CS (38)												
Ghana	GHANA (39)	60	41	−0.70	0.24 (−0.13, 0.61)	0.01	0.01	38	55	−0.53	0.21 (−0.17, 0.59)	0.01	0.02
Ghana	iLiNS−DYADG (40)	175	323	−1.24	0.18 (0.01, 0.35)	0.04	0.04	172	369	−1.06	−0.03 (−0.18, 0.12)	0.04	0.08
Haiti	HAITI (41)												
Kenya	WASH−B (42)	736	2466	−0.29	0.02 (−0.06, 0.10)	0.15	0.15	725	2710	−0.27	0.07 (−0.02, 0.15)	0.12	0.12
Madagascar	MAHAY (43)												
Malawi	iLiNS−DYADM (44)	107	207	−0.82	−0.13 (−0.37, 0.11)	0.02	0.02	113	237	−0.73	−0.12 (−0.33, 0.09)	0.02	0.05
Malawi	iLiNS−DOSE (45)	349	123	−1.25	0.00 (−0.16, 0.17)	0.04	0.04	343	115	−1.00	0.22 (0.04, 0.40)	0.03	0.06
Mali	PROMIS (46)												
Mali	PROMIS CS (46)												
Zimbabwe	SHINE (HIV−) (47)	927	903	−0.32	0.08 (−0.02, 0.18)	0.11	0.12	944	880	−0.23	0.09 (−0.01, 0.19)	0.09	0.11
Zimbabwe	SHINE (HIV+) (48)	162	168	−0.63	0.15 (−0.12, 0.42)	0.01	0.02	175	161	−0.48	0.05 (−0.16, 0.26)	0.02	0.05
		**6381**	**7319**		**I² = 0.12, Tau² = 0.00**			**6302**	**7648**		**I² = 0.65, Tau² = 0.00**		
**Fixed**					**0.07 (0.04, 0.10)**						**0.11 (0.08, 0.14)**		
**Random**					**0.07 (0.03, 0.11)**						**0.10 (0.05, 0.16)**		

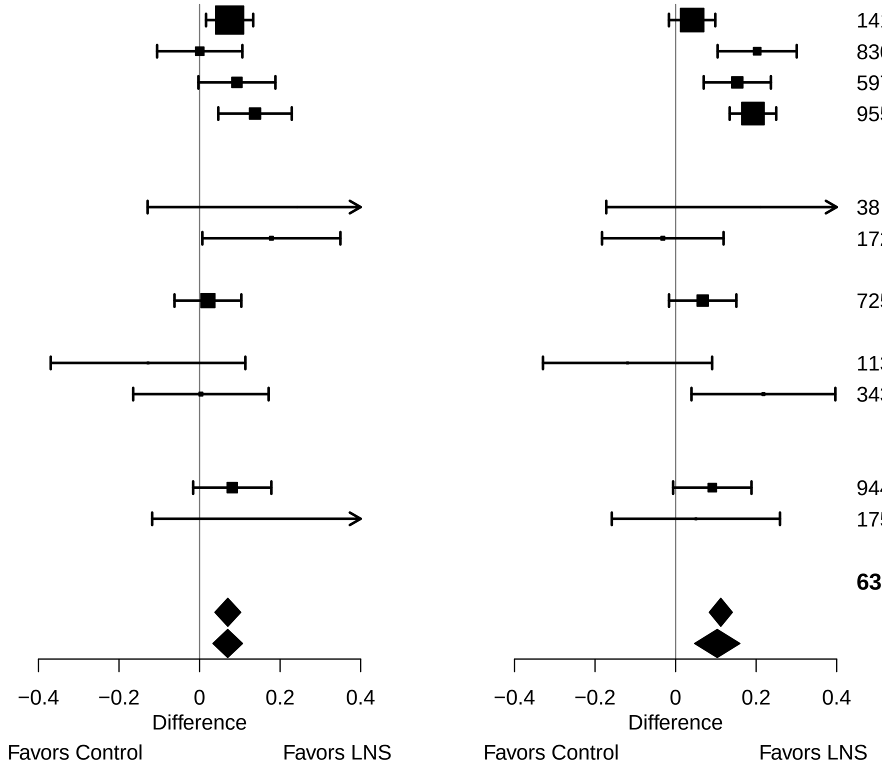

Supplemental figure 8O: Mean difference in HCZ

8O7: Stratified by Child birth order

P−for−interaction = 0.966
Difference in MDs = 0.00 (−0.04, 0.04)

Country	Trial	Later born LNS N	Later born Control N	Later born Control Mean	Later born MD (95% CI)	Later born Fixed W	Later born Random W	Firstborn LNS N	Firstborn Control N	Firstborn Control Mean	Firstborn MD (95% CI)	Firstborn Fixed W	Firstborn Random W
Bangladesh	JiVitA−4 (34)	616	260	−1.50	0.10 (0.02, 0.17)	0.15	0.14	2265	1000	−1.43	0.05 (0.00, 0.10)	0.41	0.14
Bangladesh	RDNS (35)	974	508	−1.85	0.12 (0.03, 0.22)	0.09	0.10	688	307	−1.89	0.07 (−0.05, 0.19)	0.08	0.12
Bangladesh	WASH−B (36)	736	2313	−1.60	0.14 (0.06, 0.21)	0.15	0.14	427	1150	−1.63	0.11 (−0.01, 0.22)	0.09	0.12
Burkina Faso	iLiNS−Zinc (37)	1515	519	−1.82	0.16 (0.09, 0.22)	0.20	0.18	431	145	−1.92	0.20 (0.12, 0.27)	0.21	0.14
Burkina Faso	PROMIS (38)												
Burkina Faso	PROMIS CS (38)												
Ghana	GHANA (39)	60	54	−0.60	0.25 (−0.11, 0.61)	0.01	0.01	38	38	−0.57	0.08 (−0.32, 0.47)	0.01	0.04
Ghana	iLiNS−DYADG (40)	237	458	−1.11	0.03 (−0.11, 0.17)	0.04	0.05	110	234	−1.20	0.14 (−0.06, 0.35)	0.03	0.08
Haiti	HAITI (41)												
Kenya	WASH−B (42)	1137	4074	−0.26	0.05 (−0.02, 0.12)	0.17	0.16	322	1097	−0.35	0.02 (−0.11, 0.14)	0.08	0.12
Madagascar	MAHAY (43)												
Malawi	iLiNS−DYADM (44)	173	353	−0.77	−0.06 (−0.24, 0.11)	0.03	0.03	47	89	−0.76	−0.38 (−0.74, −0.03)	0.01	0.04
Malawi	iLiNS−DOSE (45)	458	162	−1.13	0.03 (−0.13, 0.18)	0.04	0.04	146	45	−1.19	0.38 (0.11, 0.64)	0.02	0.06
Mali	PROMIS (46)												
Mali	PROMIS CS (46)												
Zimbabwe	SHINE (HIV−) (47)	1295	1214	−0.24	0.11 (0.02, 0.19)	0.11	0.12	489	486	−0.37	0.07 (−0.06, 0.21)	0.06	0.11
Zimbabwe	SHINE (HIV+) (48)	280	246	−0.49	0.03 (−0.18, 0.24)	0.02	0.02	54	78	−0.69	0.30 (−0.05, 0.65)	0.01	0.04
		**7481**	**10161**		**I² = 0.18, Tau² = 0.00**			**5017**	**4669**		**I² = 0.60, Tau² = 0.01**		
**Fixed**					**0.10 (0.07, 0.13)**						**0.09 (0.06, 0.13)**		
**Random**					**0.10 (0.06, 0.13)**						**0.10 (0.01, 0.18)**		

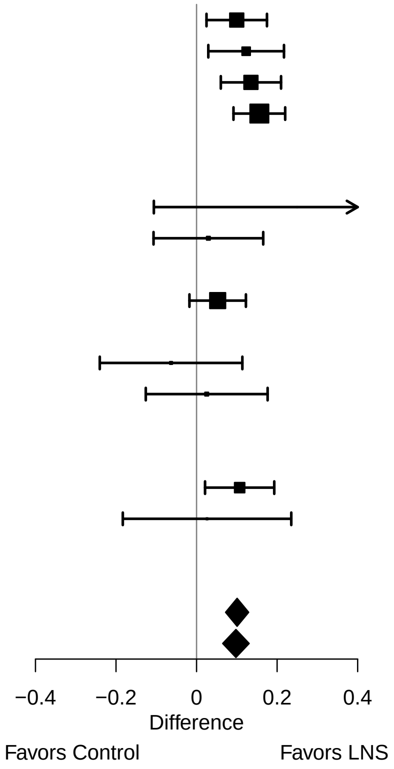

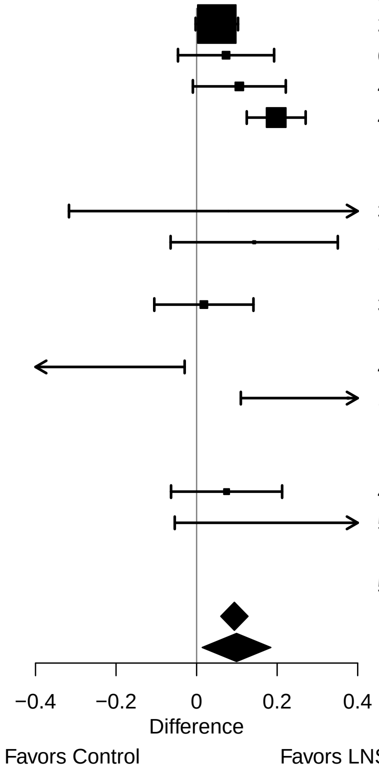

Supplemental figure 8O: Mean difference in HCZ

8O8: Stratified by Child baseline anthropometric status

P−for−interaction = 0.980
Difference in MDs = 0.00 (−0.05, 0.05)

		HCZ at least −1 Z						HCZ less than −1 Z					
**Country**	**Trial**	**LNS N**	**Control N**	**Control Mean**	**MD (95% CI)**	**Fixed W**	**Random W**	**LNS N**	**Control N**	**Control Mean**	**MD (95% CI)**	**Fixed W**	**Random W**
Bangladesh	JiVitA−4 (34)	1411	630	−0.88	0.09 (0.02, 0.16)	0.26	0.16	1474	637	−2.01	0.07 (0.00, 0.15)	0.30	0.30
Bangladesh	RDNS (35)	521	239	−1.06	0.02 (−0.06, 0.11)	0.18	0.15	1080	521	−2.23	0.11 (0.04, 0.17)	0.35	0.35
Bangladesh	WASH−B (36)												
Burkina Faso	iLiNS−Zinc (37)	550	176	−0.96	0.26 (0.16, 0.36)	0.13	0.14	1396	488	−2.16	0.16 (0.06, 0.26)	0.16	0.16
Burkina Faso	PROMIS (38)												
Burkina Faso	PROMIS CS (38)												
Ghana	GHANA (39)												
Ghana	iLiNS−DYADG (40)	189	384	−0.70	0.05 (−0.08, 0.17)	0.09	0.12	134	257	−1.81	0.12 (−0.01, 0.26)	0.08	0.08
Haiti	HAITI (41)												
Kenya	WASH−B (42)												
Madagascar	MAHAY (43)												
Malawi	iLiNS−DYADM (44)	155	323	−0.43	−0.15 (−0.30, 0.00)	0.06	0.11	49	101	−1.90	0.01 (−0.26, 0.28)	0.02	0.02
Malawi	iLiNS−DOSE (45)	381	132	−0.65	0.08 (−0.10, 0.27)	0.04	0.09	311	106	−1.73	0.16 (−0.05, 0.36)	0.04	0.04
Mali	PROMIS (46)												
Mali	PROMIS CS (46)												
Zimbabwe	SHINE (HIV−) (47)	1072	967	−0.02	0.06 (−0.02, 0.14)	0.21	0.15	254	239	−1.07	−0.03 (−0.21, 0.16)	0.04	0.04
Zimbabwe	SHINE (HIV+) (48)	200	168	−0.23	0.11 (−0.11, 0.32)	0.03	0.08	71	67	−1.30	0.16 (−0.25, 0.57)	0.01	0.01
		**4479**	**3019**		**I² = 0.69, Tau² = 0.01**			**4769**	**2416**		**I² = 0.00, Tau² = 0.00**		
**Fixed**					**0.08 (0.04, 0.11)**						**0.10 (0.06, 0.14)**		
**Random**					**0.07 (−0.01, 0.15)**						**0.10 (0.06, 0.14)**		

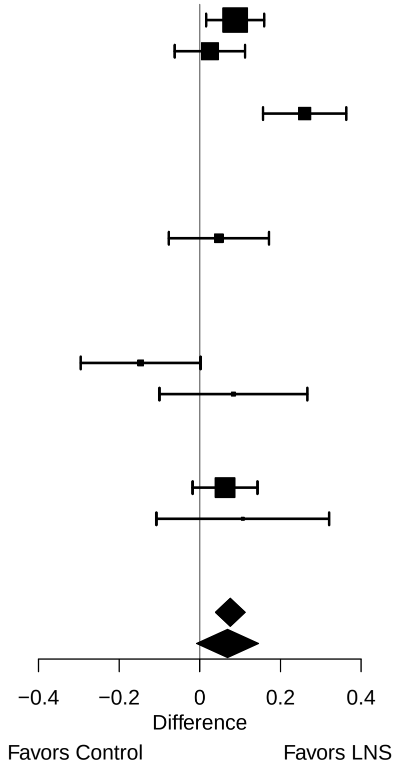

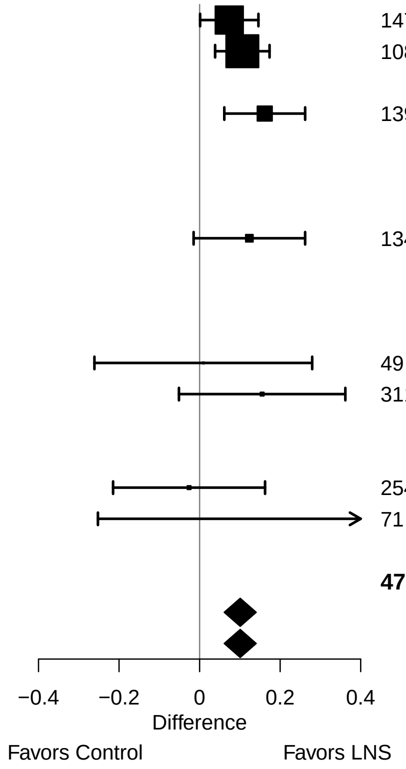

Supplemental figure 8P: Small head size prevalence ratio

8P1: Stratified by Maternal height

P−for−interaction = 0.420
Ratio of PRs = 0.95 (0.85, 1.07)

		At least 150.1 cm						Less than 150.1 cm					
**Country**	**Trial**	**LNS N**	**Control N**	**Control Prevalence**	**PR (95% CI)**	**Fixed W**	**Random W**	**LNS N**	**Control N**	**Control Prevalence**	**PR (95% CI)**	**Fixed W**	**Random W**
Bangladesh	JiVitA−4 (34)												
Bangladesh	RDNS (35)	868	432	37.7	0.88 (0.77, 1.01)	0.30	0.30	735	358	50.0	0.88 (0.79, 0.97)	0.56	0.56
Bangladesh	WASH−B (36)	619	1899	27.3	0.96 (0.83, 1.12)	0.24	0.24	544	1559	39.4	0.85 (0.75, 0.97)	0.38	0.38
Burkina Faso	iLiNS−Zinc (37)	1911	649	40.9	0.89 (0.78, 1.02)	0.28	0.28	33	15	46.7	0.92 (0.53, 1.60)	0.02	0.02
Burkina Faso	PROMIS (38)												
Burkina Faso	PROMIS CS (38)												
Ghana	GHANA (39)												
Ghana	iLiNS−DYADG (40)												
Haiti	HAITI (41)												
Kenya	WASH−B (42)	1316	4720	4.2	0.93 (0.68, 1.28)	0.05	0.05	57	194	7.7	1.13 (0.42, 3.05)	0.01	0.01
Madagascar	MAHAY (43)												
Malawi	iLiNS−DYADM (44)												
Malawi	iLiNS−DOSE (45)	581	195	18.9	0.82 (0.60, 1.13)	0.05	0.05	111	42	35.7	0.72 (0.44, 1.17)	0.03	0.03
Mali	PROMIS (46)												
Mali	PROMIS CS (46)												
Zimbabwe	SHINE (HIV−) (47)	1746	1687	5.6	0.78 (0.60, 1.02)	0.08	0.08	76	62	12.9	0.92 (0.38, 2.22)	0.01	0.01
Zimbabwe	SHINE (HIV+) (48)												
		**7041**	**9582**		**I² = 0.00, Tau² = 0.00**			**1556**	**2230**		**I² = 0.00, Tau² = 0.00**		
**Fixed**					**0.89 (0.83, 0.96)**						**0.87 (0.80, 0.94)**		
**Random**					**0.89 (0.83, 0.96)**						**0.87 (0.80, 0.94)**		

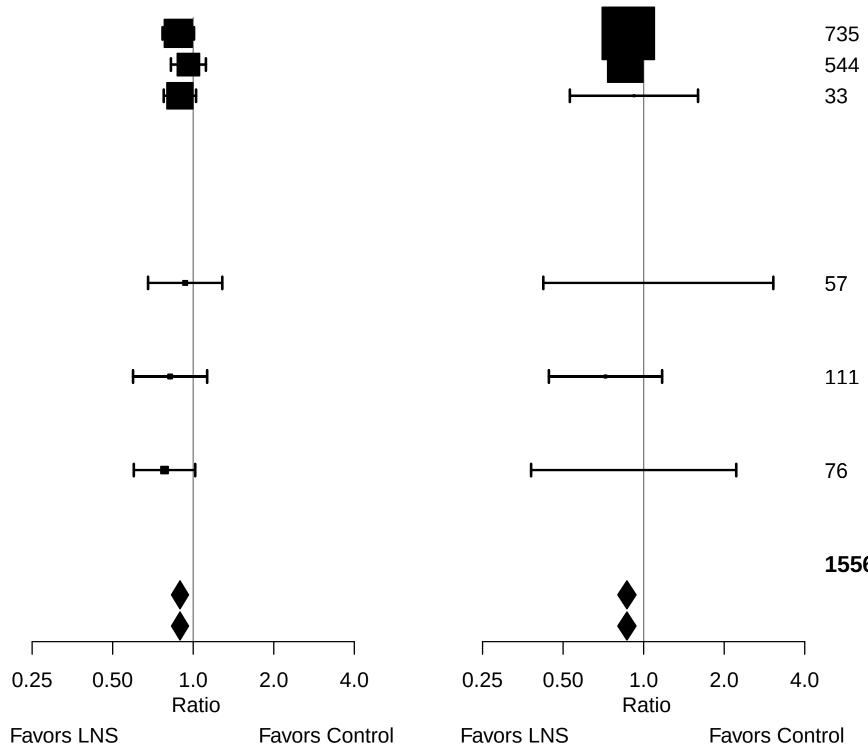

Supplemental figure 8P: Small head size prevalence ratio

8P2: Stratified by Maternal BMI

P−for−interaction = 0.607
Ratio of PRs = 0.97 (0.88, 1.08)

		At least 20 kg/m²						Less than 20 kg/m²					
**Country**	**Trial**	**LNS N**	**Control N**	**Control Prevalence**	**PR (95% CI)**	**Fixed W**	**Random W**	**LNS N**	**Control N**	**Control Prevalence**	**PR (95% CI)**	**Fixed W**	**Random W**
Bangladesh	JiVitA−4 (34)												
Bangladesh	RDNS (35)	730	335	39.7	0.85 (0.72, 1.00)	0.22	0.22	873	455	45.9	0.91 (0.80, 1.03)	0.37	0.37
Bangladesh	WASH−B (36)	519	1595	28.2	0.99 (0.84, 1.17)	0.20	0.20	644	1863	36.6	0.85 (0.75, 0.96)	0.36	0.36
Burkina Faso	iLiNS−Zinc (37)	1210	389	38.9	0.90 (0.79, 1.01)	0.38	0.38	734	275	44.0	0.89 (0.74, 1.06)	0.18	0.18
Burkina Faso	PROMIS (38)												
Burkina Faso	PROMIS CS (38)												
Ghana	GHANA (39)												
Ghana	iLiNS−DYADG (40)	305	562	15.3	0.90 (0.64, 1.27)	0.05	0.05	37	119	20.2	0.80 (0.36, 1.82)	0.01	0.01
Haiti	HAITI (41)												
Kenya	WASH−B (42)	1100	3831	4.0	0.90 (0.64, 1.28)	0.05	0.05	273	1083	5.3	1.11 (0.65, 1.91)	0.02	0.02
Madagascar	MAHAY (43)												
Malawi	iLiNS−DYADM (44)	131	263	7.6	1.00 (0.48, 2.08)	0.01	0.01	88	178	14.6	0.78 (0.39, 1.54)	0.01	0.01
Malawi	iLiNS−DOSE (45)	512	172	17.3	0.87 (0.61, 1.25)	0.04	0.04	178	64	34.4	0.66 (0.45, 0.95)	0.04	0.04
Mali	PROMIS (46)												
Mali	PROMIS CS (46)												
Zimbabwe	SHINE (HIV−) (47)	1358	1298	5.9	0.76 (0.56, 1.03)	0.06	0.06	239	248	6.0	1.18 (0.61, 2.26)	0.01	0.01
Zimbabwe	SHINE (HIV+) (48)												
		**5865**	**8445**		**I² = 0.00, Tau² = 0.00**			**3066**	**4285**		**I² = 0.00, Tau² = 0.00**		
**Fixed**					**0.89 (0.83, 0.96)**						**0.87 (0.81, 0.94)**		
**Random**					**0.89 (0.83, 0.96)**						**0.87 (0.81, 0.94)**		

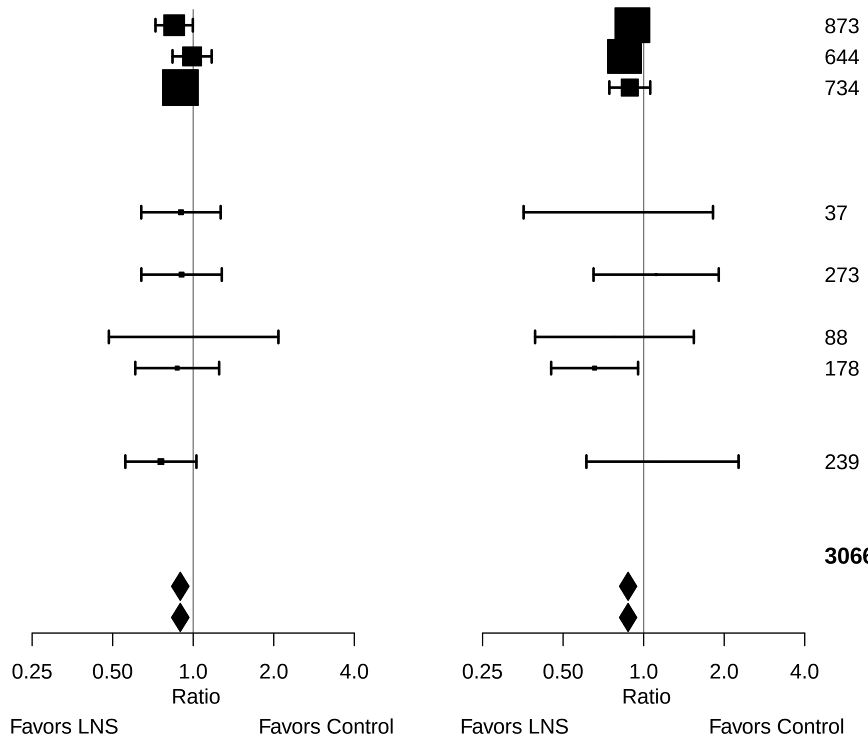

Supplemental figure 8P: Small head size prevalence ratio

8P3: Stratified by Maternal age

P−for−interaction = 0.907
Ratio of PRs = 0.99 (0.89, 1.11)

Country	Trial	At least 25 y: LNS N	At least 25 y: Control N	At least 25 y: Control Prevalence	At least 25 y: PR (95% CI)	At least 25 y: Fixed W	At least 25 y: Random W	Less than 25 y: LNS N	Less than 25 y: Control N	Less than 25 y: Control Prevalence	Less than 25 y: PR (95% CI)	Less than 25 y: Fixed W	Less than 25 y: Random W
Bangladesh	JiVitA−4 (34)	1238	554	22.1	1.12 (0.91, 1.39)	0.16	0.16	1634	708	25.6	0.96 (0.82, 1.13)	0.14	0.16
Bangladesh	RDNS (35)	453	221	42.5	0.82 (0.69, 0.98)	0.23	0.23	1210	594	43.1	0.92 (0.82, 1.04)	0.29	0.22
Bangladesh	WASH−B (36)	492	1523	32.2	0.91 (0.77, 1.07)	0.26	0.26	666	1924	33.4	0.90 (0.79, 1.03)	0.22	0.20
Burkina Faso	iLiNS−Zinc (37)	1128	399	39.4	0.84 (0.67, 1.04)	0.14	0.14	805	262	43.1	0.98 (0.86, 1.11)	0.24	0.21
Burkina Faso	PROMIS (38)												
Burkina Faso	PROMIS CS (38)												
Ghana	GHANA (39)												
Ghana	iLiNS−DYADG (40)	220	417	14.6	0.87 (0.57, 1.32)	0.04	0.04	127	275	18.2	0.91 (0.57, 1.45)	0.02	0.03
Haiti	HAITI (41)												
Kenya	WASH−B (42)	845	2831	4.0	0.94 (0.64, 1.38)	0.05	0.05	601	2313	4.7	0.92 (0.59, 1.42)	0.02	0.04
Madagascar	MAHAY (43)												
Malawi	iLiNS−DYADM (44)	106	226	9.3	0.71 (0.31, 1.62)	0.01	0.01	114	218	12.4	0.99 (0.54, 1.81)	0.01	0.02
Malawi	iLiNS−DOSE (45)	368	134	16.4	1.09 (0.73, 1.63)	0.04	0.04	310	97	29.6	0.53 (0.37, 0.75)	0.03	0.05
Mali	PROMIS (46)												
Mali	PROMIS CS (46)												
Zimbabwe	SHINE (HIV−) (47)	869	795	5.4	0.79 (0.52, 1.18)	0.04	0.04	805	792	6.3	0.81 (0.54, 1.21)	0.02	0.04
Zimbabwe	SHINE (HIV+) (48)	244	231	8.2	1.20 (0.64, 2.22)	0.02	0.02	73	78	14.1	0.78 (0.37, 1.65)	0.01	0.01
		**5963**	**7331**		**I² = 0.00, Tau² = 0.00**			**6345**	**7261**		**I² = 0.21, Tau² = 0.01**		
**Fixed**					**0.91 (0.84, 0.99)**						**0.92 (0.86, 0.98)**		
**Random**					**0.91 (0.84, 0.99)**						**0.90 (0.82, 0.99)**		

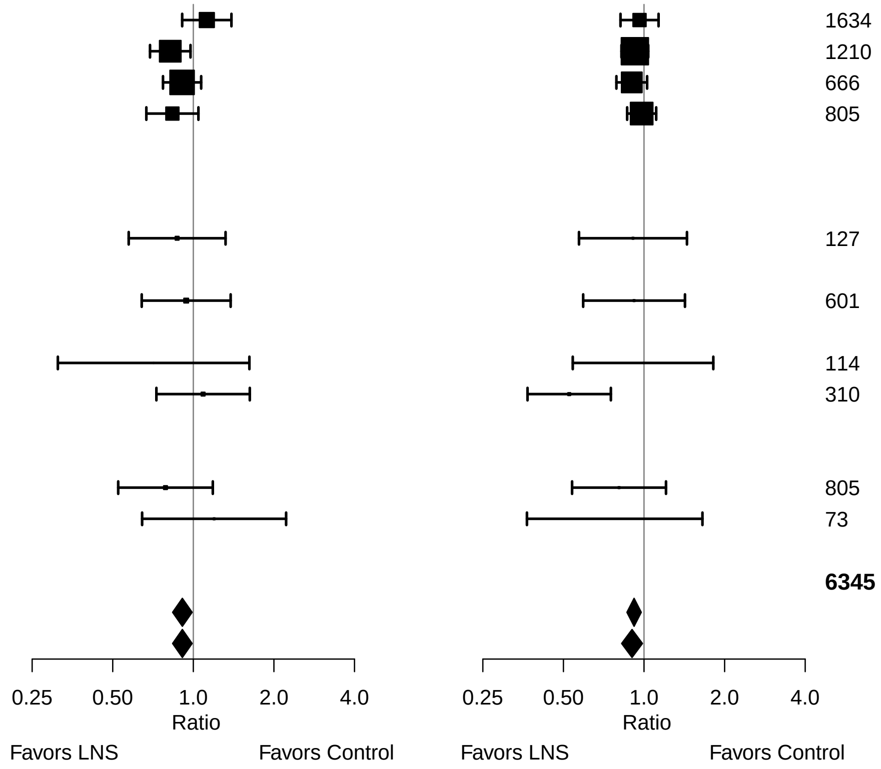

Supplemental figure 8P: Small head size prevalence ratio

8P4: Stratified by Maternal education

P−for−interaction = 0.091
Ratio of PRs = 1.11 (0.98, 1.26)

Country	Trial	Primary or greater: LNS N	Control N	Control Prevalence	PR (95% CI)	Fixed W	Random W	Incomplete or no formal: LNS N	Control N	Control Prevalence	PR (95% CI)	Fixed W	Random W
Bangladesh	JiVitA−4 (34)	1855	775	23.3	0.91 (0.77, 1.08)	0.18	0.18	1028	489	25.5	1.21 (0.98, 1.48)	0.14	0.16
Bangladesh	RDNS (35)	1245	589	41.6	0.88 (0.78, 0.98)	0.41	0.41	418	226	46.5	0.96 (0.80, 1.14)	0.18	0.19
Bangladesh	WASH−B (36)	817	2477	31.2	0.85 (0.75, 0.97)	0.32	0.32	346	986	36.7	1.00 (0.84, 1.19)	0.20	0.19
Burkina Faso	iLiNS−Zinc (37)	82	16	31.2	1.20 (0.67, 2.14)	0.02	0.02	1851	645	41.1	0.88 (0.78, 1.01)	0.34	0.24
Burkina Faso	PROMIS (38)												
Burkina Faso	PROMIS CS (38)												
Ghana	GHANA (39)												
Ghana	iLiNS−DYADG (40)	267	548	15.3	0.86 (0.59, 1.23)	0.04	0.04	80	144	18.8	0.93 (0.52, 1.67)	0.02	0.03
Haiti	HAITI (41)												
Kenya	WASH−B (42)	703	2456	2.8	1.16 (0.68, 1.99)	0.02	0.02	756	2716	5.7	0.81 (0.59, 1.12)	0.06	0.09
Madagascar	MAHAY (43)												
Malawi	iLiNS−DYADM (44)												
Malawi	iLiNS−DOSE (45)	163	55	16.1	0.86 (0.45, 1.65)	0.01	0.01	517	176	23.9	0.74 (0.55, 0.99)	0.07	0.10
Mali	PROMIS (46)												
Mali	PROMIS CS (46)												
Zimbabwe	SHINE (HIV−) (47)												
Zimbabwe	SHINE (HIV+) (48)												
		**5132**	**6916**		**I² = 0.00, Tau² = 0.00**			**4996**	**5382**		**I² = 0.43, Tau² = 0.01**		
**Fixed**					**0.88 (0.82, 0.95)**						**0.94 (0.87, 1.02)**		
**Random**					**0.88 (0.82, 0.95)**						**0.94 (0.84, 1.05)**		

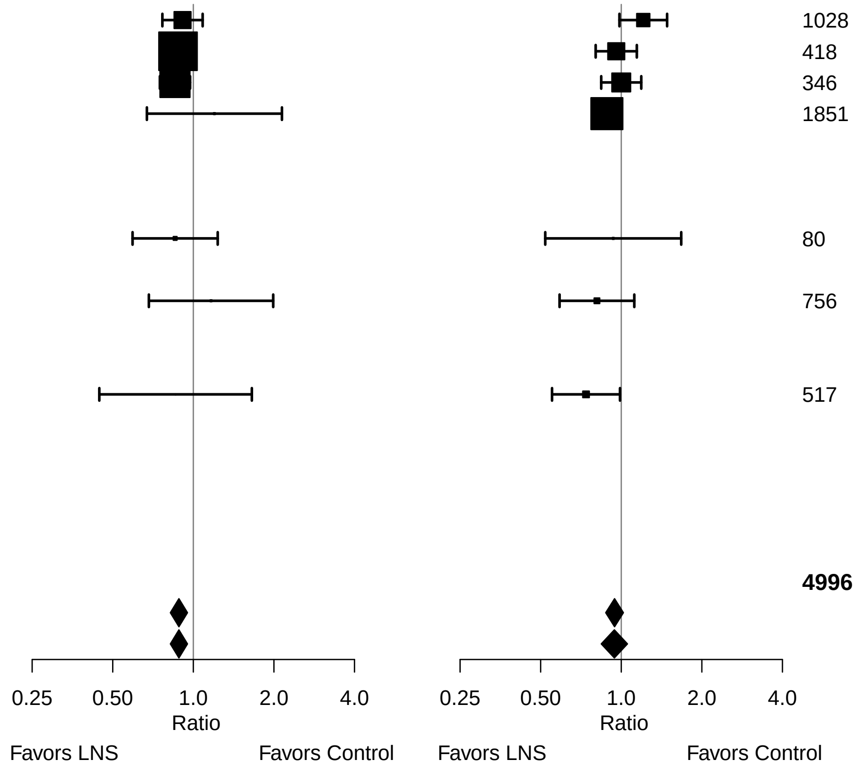

Supplemental figure 8P: Small head size prevalence ratio

8P5: Stratified by Maternal depressive symptoms

P−for−interaction = 0.095
Ratio of PRs = 1.12 (0.98, 1.27)

		Less than 75th percentile						At least 75th percentile					
**Country**	**Trial**	**LNS N**	**Control N**	**Control Prevalence**	**PR (95% CI)**	**Fixed W**	**Random W**	**LNS N**	**Control N**	**Control Prevalence**	**PR (95% CI)**	**Fixed W**	**Random W**
Bangladesh	JiVitA−4 (34)												
Bangladesh	RDNS (35)	1081	472	39.0	1.01 (0.91, 1.11)	0.48	0.48	520	288	49.3	0.74 (0.63, 0.88)	0.50	0.42
Bangladesh	WASH−B (36)	901	2487	33.0	0.85 (0.76, 0.96)	0.38	0.38	238	885	31.9	1.05 (0.87, 1.28)	0.35	0.34
Burkina Faso	iLiNS−Zinc (37)												
Burkina Faso	PROMIS (38)												
Burkina Faso	PROMIS CS (38)												
Ghana	GHANA (39)												
Ghana	iLiNS−DYADG (40)	252	459	15.0	1.00 (0.70, 1.44)	0.04	0.04	83	209	17.7	0.68 (0.36, 1.30)	0.03	0.05
Haiti	HAITI (41)												
Kenya	WASH−B (42)	998	3517	4.0	0.92 (0.64, 1.31)	0.04	0.04	341	1248	5.4	0.71 (0.32, 1.56)	0.02	0.04
Madagascar	MAHAY (43)												
Malawi	iLiNS−DYADM (44)	157	303	10.2	0.81 (0.44, 1.50)	0.01	0.01	47	113	11.5	1.29 (0.55, 3.04)	0.02	0.03
Malawi	iLiNS−DOSE (45)												
Mali	PROMIS (46)												
Mali	PROMIS CS (46)												
Zimbabwe	SHINE (HIV−) (47)	1193	1135	5.0	0.88 (0.60, 1.30)	0.03	0.03	509	490	7.1	0.72 (0.45, 1.14)	0.06	0.09
Zimbabwe	SHINE (HIV+) (48)	234	215	8.8	1.16 (0.68, 1.99)	0.02	0.02	89	100	12.0	0.75 (0.32, 1.75)	0.02	0.03
		**4816**	**8588**		**I² = 0.00, Tau² = 0.00**			**1827**	**3333**		**I² = 0.37, Tau² = 0.01**		
**Fixed**					**0.94 (0.87, 1.01)**						**0.84 (0.75, 0.95)**		
**Random**					**0.94 (0.87, 1.01)**						**0.85 (0.73, 0.98)**		

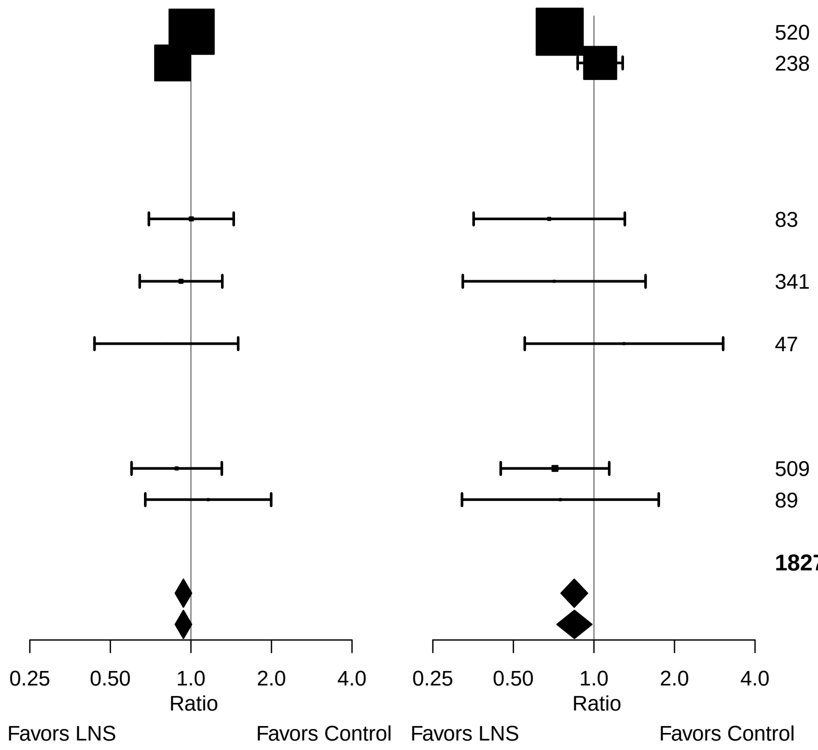

Supplemental figure 8P: Small head size prevalence ratio

8P6: Stratified by Child sex

P−for−interaction = 0.020
Ratio of PRs = 0.89 (0.81, 0.98)

Country	Trial	Male LNS N	Male Control N	Male Control Prevalence	Male PR (95% CI)	Male Fixed W	Male Random W	Female LNS N	Female Control N	Female Control Prevalence	Female PR (95% CI)	Female Fixed W	Female Random W
Bangladesh	JiVitA−4 (34)	1475	612	25.8	0.92 (0.79, 1.08)	0.18	0.18	1410	655	22.4	1.22 (0.99, 1.49)	0.13	0.15
Bangladesh	RDNS (35)	833	411	37.7	1.07 (0.94, 1.21)	0.28	0.28	830	404	48.3	0.76 (0.65, 0.89)	0.24	0.17
Bangladesh	WASH−B (36)	566	1730	32.4	0.93 (0.81, 1.07)	0.22	0.22	597	1733	33.2	0.88 (0.76, 1.02)	0.26	0.17
Burkina Faso	iLiNS−Zinc (37)	991	335	42.3	0.97 (0.82, 1.14)	0.16	0.16	955	329	39.7	0.78 (0.67, 0.92)	0.23	0.17
Burkina Faso	PROMIS (38)												
Burkina Faso	PROMIS CS (38)												
Ghana	GHANA (39)												
Ghana	iLiNS−DYADG (40)	175	323	19.2	0.86 (0.58, 1.29)	0.03	0.03	172	369	13.3	0.88 (0.54, 1.43)	0.02	0.06
Haiti	HAITI (41)												
Kenya	WASH−B (42)	736	2466	4.9	0.87 (0.58, 1.28)	0.03	0.03	725	2710	3.8	0.97 (0.62, 1.51)	0.03	0.07
Madagascar	MAHAY (43)												
Malawi	iLiNS−DYADM (44)	107	207	13.0	0.86 (0.45, 1.63)	0.01	0.01	113	237	8.9	0.90 (0.43, 1.90)	0.01	0.03
Malawi	iLiNS−DOSE (45)	349	123	23.6	0.97 (0.69, 1.36)	0.04	0.04	343	115	19.8	0.57 (0.37, 0.87)	0.03	0.07
Mali	PROMIS (46)												
Mali	PROMIS CS (46)												
Zimbabwe	SHINE (HIV−) (47)	927	903	6.4	0.87 (0.63, 1.21)	0.04	0.04	944	880	5.1	0.75 (0.50, 1.11)	0.04	0.08
Zimbabwe	SHINE (HIV+) (48)	162	168	14.3	0.73 (0.40, 1.33)	0.01	0.01	175	161	5.6	1.64 (0.68, 3.93)	0.01	0.02
		**6321**	**7278**		**I² = 0.00, Tau² = 0.00**			**6264**	**7593**		**I² = 0.58, Tau² = 0.02**		
**Fixed**					**0.96 (0.90, 1.03)**						**0.85 (0.79, 0.92)**		
**Random**					**0.96 (0.90, 1.03)**						**0.86 (0.75, 0.99)**		

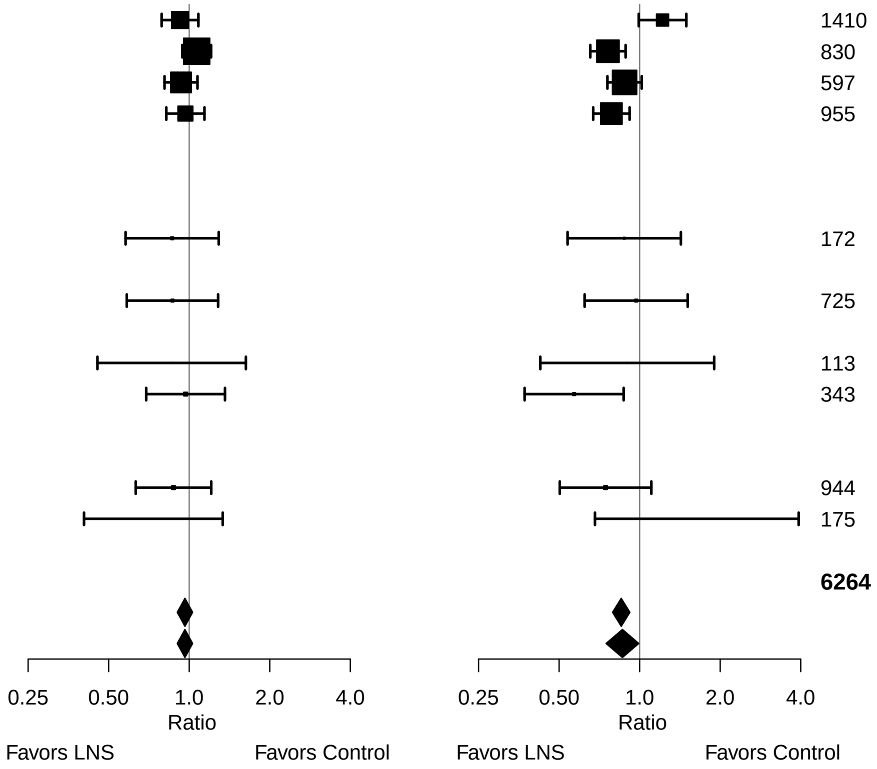

Supplemental figure 8P: Small head size prevalence ratio

8P7: Stratified by Child birth order

P−for−interaction = 0.696
Ratio of PRs = 1.02 (0.92, 1.14)

Country	Trial	Later born LNS N	Later born Control N	Later born Control Prevalence	Later born PR (95% CI)	Later born Fixed W	Later born Random W	Firstborn LNS N	Firstborn Control N	Firstborn Control Prevalence	Firstborn PR (95% CI)	Firstborn Fixed W	Firstborn Random W
Bangladesh	JiVitA−4 (34)	616	260	25.0	0.99 (0.78, 1.26)	0.08	0.08	2265	1000	23.9	1.03 (0.88, 1.21)	0.25	0.18
Bangladesh	RDNS (35)	974	508	43.1	0.87 (0.77, 0.97)	0.34	0.34	688	307	42.7	0.94 (0.80, 1.09)	0.27	0.18
Bangladesh	WASH−B (36)	736	2313	31.9	0.90 (0.79, 1.02)	0.27	0.27	427	1150	34.6	0.91 (0.77, 1.07)	0.22	0.18
Burkina Faso	iLiNS−Zinc (37)	1515	519	40.3	0.86 (0.72, 1.02)	0.14	0.14	431	145	43.4	0.99 (0.81, 1.19)	0.17	0.17
Burkina Faso	PROMIS (38)												
Burkina Faso	PROMIS CS (38)												
Ghana	GHANA (39)												
Ghana	iLiNS−DYADG (40)	237	458	14.4	0.97 (0.66, 1.42)	0.03	0.03	110	234	19.2	0.76 (0.45, 1.28)	0.02	0.07
Haiti	HAITI (41)												
Kenya	WASH−B (42)	1137	4074	4.3	0.97 (0.70, 1.35)	0.04	0.04	322	1097	4.3	0.72 (0.40, 1.30)	0.02	0.06
Madagascar	MAHAY (43)												
Malawi	iLiNS−DYADM (44)	173	353	10.8	0.75 (0.42, 1.35)	0.01	0.01	47	89	10.1	1.47 (0.59, 3.70)	0.01	0.03
Malawi	iLiNS−DOSE (45)	458	162	20.2	0.91 (0.65, 1.27)	0.04	0.04	146	45	26.7	0.42 (0.25, 0.71)	0.02	0.07
Mali	PROMIS (46)												
Mali	PROMIS CS (46)												
Zimbabwe	SHINE (HIV−) (47)	1295	1214	5.0	0.80 (0.58, 1.10)	0.04	0.04	489	486	7.8	0.76 (0.46, 1.25)	0.03	0.07
Zimbabwe	SHINE (HIV+) (48)												
		**7141**	**9861**		**I² = 0.00, Tau² = 0.00**			**4925**	**4553**		**I² = 0.42, Tau² = 0.03**		
**Fixed**					**0.89 (0.83, 0.95)**						**0.93 (0.86, 1.01)**		
**Random**					**0.89 (0.83, 0.95)**						**0.88 (0.74, 1.03)**		

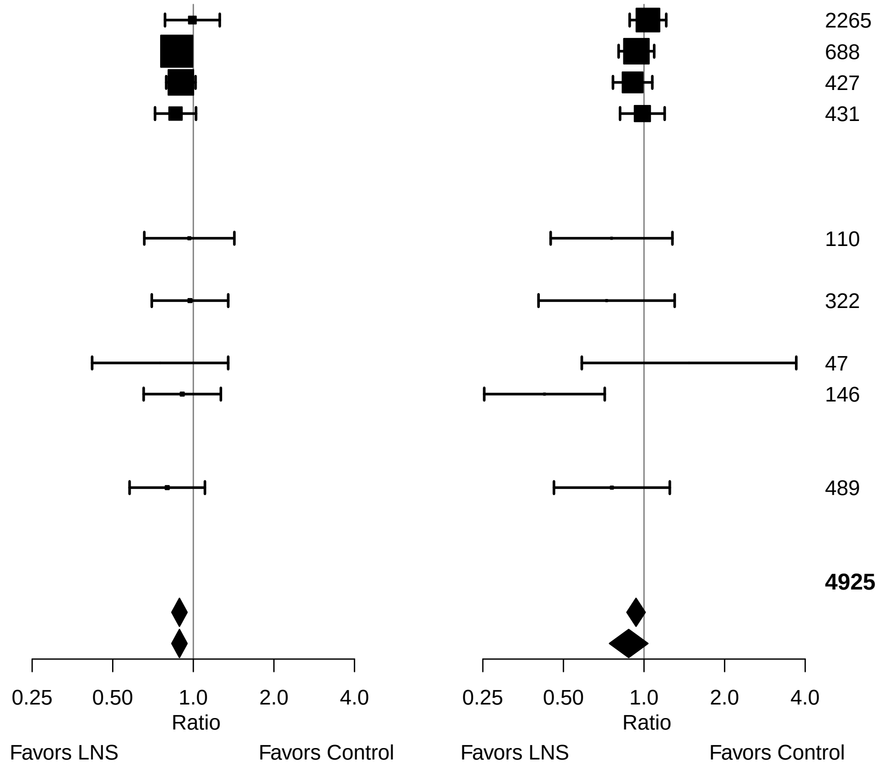

Supplemental figure 8P: Small head size prevalence ratio

8P8: Stratified by Child baseline anthropometric status

P−for−interaction = 0.152
Ratio of PRs = 1.25 (0.92, 1.70)

		HCZ at least −1 Z						HCZ less than −1 Z					
**Country**	**Trial**	**LNS N**	**Control N**	**Control Prevalence**	**PR (95% CI)**	**Fixed W**	**Random W**	**LNS N**	**Control N**	**Control Prevalence**	**PR (95% CI)**	**Fixed W**	**Random W**
Bangladesh	JiVitA−4 (34)	1411	630	2.7	1.05 (0.54, 2.04)	0.18	0.16	1474	637	45.4	0.95 (0.85, 1.06)	0.26	0.26
Bangladesh	RDNS (35)	521	239	5.0	0.50 (0.23, 1.07)	0.13	0.14	1080	521	60.3	0.92 (0.85, 1.00)	0.48	0.48
Bangladesh	WASH−B (36)												
Burkina Faso	iLiNS−Zinc (37)	550	176	5.1	0.21 (0.09, 0.53)	0.10	0.12	1396	488	54.1	0.89 (0.77, 1.03)	0.16	0.16
Burkina Faso	PROMIS (38)												
Burkina Faso	PROMIS CS (38)												
Ghana	GHANA (39)												
Ghana	iLiNS−DYADG (40)	189	384	2.3	1.35 (0.49, 3.75)	0.08	0.11	134	257	35.4	0.89 (0.66, 1.20)	0.04	0.04
Haiti	HAITI (41)												
Kenya	WASH−B (42)												
Madagascar	MAHAY (43)												
Malawi	iLiNS−DYADM (44)												
Malawi	iLiNS−DOSE (45)	381	132	7.6	0.90 (0.45, 1.82)	0.16	0.16	311	106	39.6	0.76 (0.57, 1.02)	0.04	0.04
Mali	PROMIS (46)												
Mali	PROMIS CS (46)												
Zimbabwe	SHINE (HIV−) (47)	1072	967	2.8	0.77 (0.45, 1.31)	0.27	0.19	254	239	15.5	0.94 (0.62, 1.44)	0.02	0.02
Zimbabwe	SHINE (HIV+) (48)	200	168	4.2	0.84 (0.33, 2.17)	0.09	0.12	71	67	28.4	0.74 (0.37, 1.48)	0.01	0.01
		**4324**	**2696**		**I² = 0.46, Tau² = 0.17**			**4720**	**2315**		**I² = 0.00, Tau² = 0.00**		
**Fixed**					**0.73 (0.55, 0.97)**						**0.92 (0.87, 0.97)**		
**Random**					**0.71 (0.47, 1.09)**						**0.92 (0.87, 0.97)**		

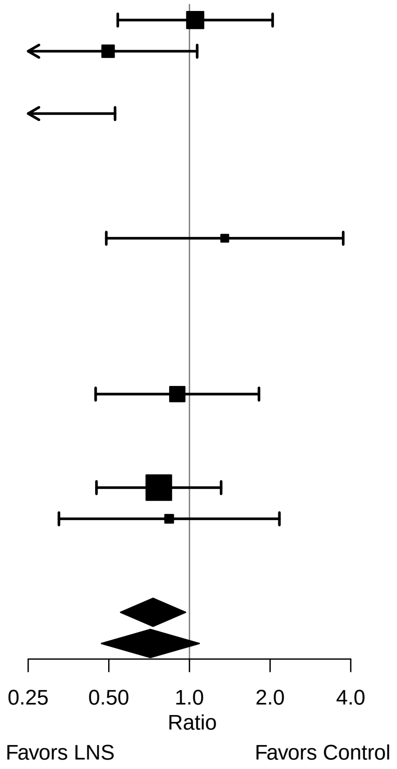

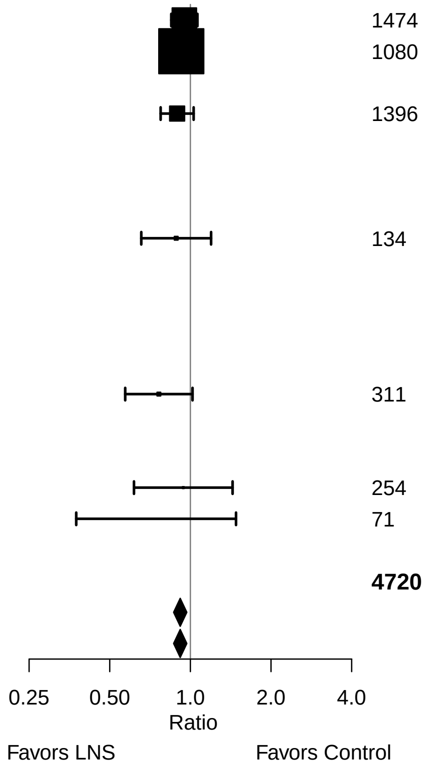

Supplemental figure 8Q: Small head size prevalence difference

8Q1: Stratified by Maternal height

P−for−interaction = 0.301
Difference in PDs = −0.02 (−0.06, 0.02)

		At least 150.1 cm						Less than 150.1 cm					
**Country**	**Trial**	**LNS N**	**Control N**	**Control Prevalence**	**PD (95% CI)**	**Fixed W**	**Random W**	**LNS N**	**Control N**	**Control Prevalence**	**PD (95% CI)**	**Fixed W**	**Random W**
Bangladesh	JiVitA−4 (34)												
Bangladesh	RDNS (35)	868	432	37.7	−0.05 (−0.09, 0.00)	0.03	0.03	735	358	50.0	−0.06 (−0.11, −0.01)	0.34	0.34
Bangladesh	WASH−B (36)	619	1899	27.3	−0.01 (−0.05, 0.03)	0.05	0.05	544	1559	39.4	−0.06 (−0.10, −0.01)	0.42	0.42
Burkina Faso	iLiNS−Zinc (37)	1911	649	40.9	−0.04 (−0.10, 0.01)	0.02	0.02	33	15	46.7	−0.01 (−0.26, 0.23)	0.01	0.01
Burkina Faso	PROMIS (38)												
Burkina Faso	PROMIS CS (38)												
Ghana	GHANA (39)												
Ghana	iLiNS−DYADG (40)												
Haiti	HAITI (41)												
Kenya	WASH−B (42)	1316	4720	4.2	0.00 (−0.02, 0.01)	0.47	0.47	57	194	7.7	0.01 (−0.07, 0.09)	0.12	0.12
Madagascar	MAHAY (43)												
Malawi	iLiNS−DYADM (44)												
Malawi	iLiNS−DOSE (45)	581	195	18.9	−0.04 (−0.09, 0.02)	0.02	0.02	111	42	35.7	−0.07 (−0.22, 0.07)	0.04	0.04
Mali	PROMIS (46)												
Mali	PROMIS CS (46)												
Zimbabwe	SHINE (HIV−) (47)	1746	1687	5.6	−0.01 (−0.03, 0.00)	0.41	0.41	76	62	12.9	−0.01 (−0.12, 0.10)	0.07	0.07
Zimbabwe	SHINE (HIV+) (48)												
		**7041**	**9582**		**I² = 0.12, Tau² = 0.00**			**1556**	**2230**		**I² = 0.00, Tau² = 0.00**		
**Fixed**					**−0.01 (−0.02, 0.00)**						**−0.05 (−0.08, −0.02)**		
**Random**					**−0.01 (−0.02, 0.00)**						**−0.05 (−0.08, −0.02)**		

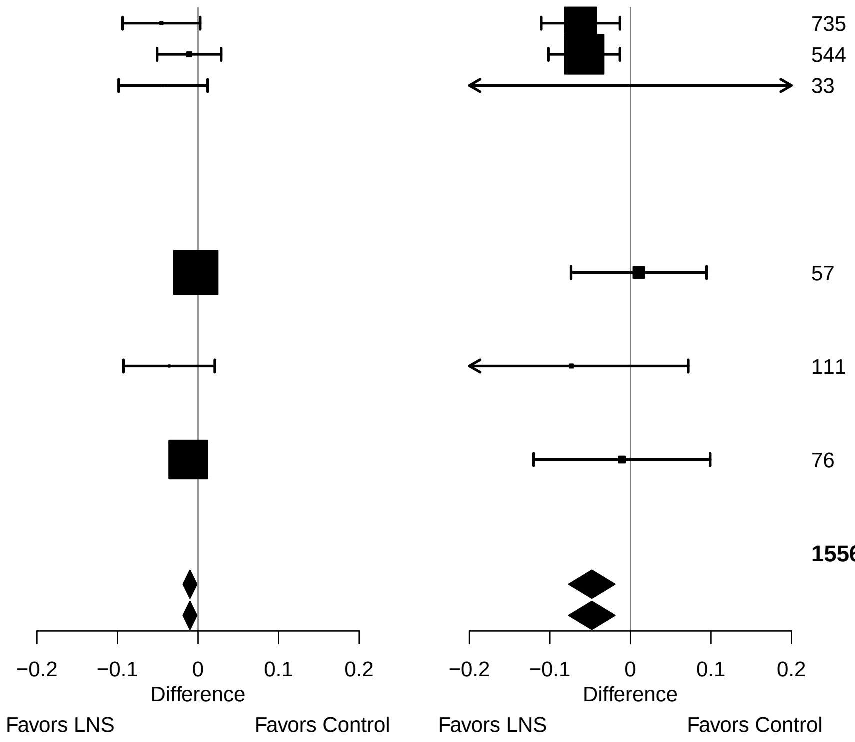

Supplemental figure 8Q: Small head size prevalence difference

8Q2: Stratified by Maternal BMI

P−for−interaction = 0.881
Difference in PDs = 0.00 (−0.02, 0.02)

		At least 20 kg/m²						Less than 20 kg/m²					
**Country**	**Trial**	**LNS N**	**Control N**	**Control Prevalence**	**PD (95% CI)**	**Fixed W**	**Random W**	**LNS N**	**Control N**	**Control Prevalence**	**PD (95% CI)**	**Fixed W**	**Random W**
Bangladesh	JiVitA−4 (34)												
Bangladesh	RDNS (35)	730	335	39.7	−0.06 (−0.12, 0.00)	0.02	0.02	873	455	45.9	−0.04 (−0.10, 0.01)	0.11	0.14
Bangladesh	WASH−B (36)	519	1595	28.2	0.00 (−0.05, 0.04)	0.04	0.04	644	1863	36.6	−0.06 (−0.09, −0.02)	0.21	0.20
Burkina Faso	iLiNS−Zinc (37)	1210	389	38.9	−0.04 (−0.08, 0.00)	0.05	0.05	734	275	44.0	−0.05 (−0.12, 0.02)	0.06	0.09
Burkina Faso	PROMIS (38)												
Burkina Faso	PROMIS CS (38)												
Ghana	GHANA (39)												
Ghana	iLiNS−DYADG (40)	305	562	15.3	−0.02 (−0.06, 0.03)	0.03	0.03	37	119	20.2	−0.04 (−0.19, 0.11)	0.02	0.03
Haiti	HAITI (41)												
Kenya	WASH−B (42)	1100	3831	4.0	0.00 (−0.02, 0.01)	0.50	0.50	273	1083	5.3	0.01 (−0.02, 0.04)	0.34	0.25
Madagascar	MAHAY (43)												
Malawi	iLiNS−DYADM (44)	131	263	7.6	0.00 (−0.06, 0.06)	0.03	0.03	88	178	14.6	−0.03 (−0.12, 0.06)	0.04	0.07
Malawi	iLiNS−DOSE (45)	512	172	17.3	−0.02 (−0.08, 0.04)	0.02	0.02	178	64	34.4	−0.11 (−0.22, 0.00)	0.03	0.04
Mali	PROMIS (46)												
Mali	PROMIS CS (46)												
Zimbabwe	SHINE (HIV−) (47)	1358	1298	5.9	−0.01 (−0.03, 0.00)	0.31	0.31	239	248	6.0	0.01 (−0.03, 0.05)	0.18	0.18
Zimbabwe	SHINE (HIV+) (48)												
		**5865**	**8445**		**I² = 0.00, Tau² = 0.00**			**3066**	**4285**		**I² = 0.41, Tau² = 0.00**		
**Fixed**					**−0.01 (−0.02, 0.00)**						**−0.02 (−0.04, 0.00)**		
**Random**					**−0.01 (−0.02, 0.00)**						**−0.03 (−0.05, 0.00)**		

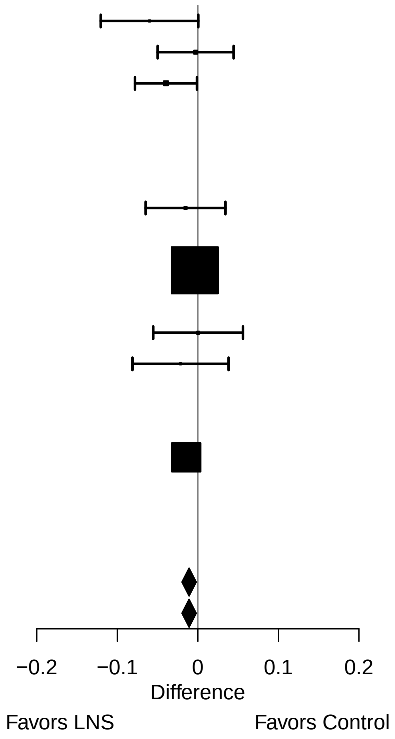

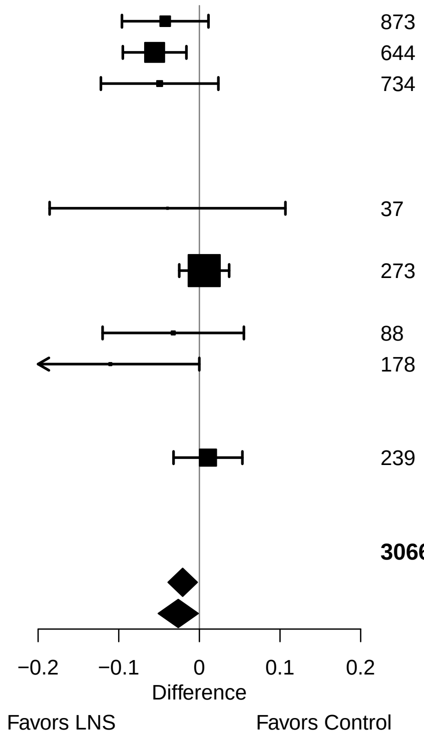

Supplemental figure 8Q: Small head size prevalence difference

8Q3: Stratified by Maternal age

P−for−interaction = 0.536
Difference in PDs = 0.00 (−0.02, 0.01)

		At least 25 y						Less than 25 y					
**Country**	**Trial**	**LNS N**	**Control N**	**Control Prevalence**	**PD (95% CI)**	**Fixed W**	**Random W**	**LNS N**	**Control N**	**Control Prevalence**	**PD (95% CI)**	**Fixed W**	**Random W**
Bangladesh	JiVitA−4 (34)	1238	554	22.1	0.02 (−0.02, 0.05)	0.07	0.11	1634	708	25.6	−0.02 (−0.05, 0.02)	0.12	0.14
Bangladesh	RDNS (35)	453	221	42.5	−0.08 (−0.14, −0.01)	0.02	0.05	1210	594	43.1	−0.03 (−0.08, 0.02)	0.06	0.10
Bangladesh	WASH−B (36)	492	1523	32.2	−0.03 (−0.08, 0.02)	0.04	0.08	666	1924	33.4	−0.03 (−0.07, 0.01)	0.08	0.12
Burkina Faso	iLiNS−Zinc (37)	1128	399	39.4	−0.06 (−0.13, 0.01)	0.02	0.05	805	262	43.1	−0.01 (−0.06, 0.04)	0.05	0.09
Burkina Faso	PROMIS (38)												
Burkina Faso	PROMIS CS (38)												
Ghana	GHANA (39)												
Ghana	iLiNS−DYADG (40)	220	417	14.6	−0.02 (−0.08, 0.04)	0.03	0.06	127	275	18.2	−0.02 (−0.10, 0.06)	0.02	0.05
Haiti	HAITI (41)												
Kenya	WASH−B (42)	845	2831	4.0	0.00 (−0.02, 0.01)	0.48	0.26	601	2313	4.7	0.00 (−0.02, 0.02)	0.37	0.19
Madagascar	MAHAY (43)												
Malawi	iLiNS−DYADM (44)	106	226	9.3	−0.03 (−0.09, 0.04)	0.02	0.05	114	218	12.4	0.00 (−0.08, 0.07)	0.02	0.05
Malawi	iLiNS−DOSE (45)	368	134	16.4	0.03 (−0.04, 0.10)	0.02	0.05	310	97	29.6	−0.16 (−0.24, −0.07)	0.02	0.05
Mali	PROMIS (46)												
Mali	PROMIS CS (46)												
Zimbabwe	SHINE (HIV−) (47)	869	795	5.4	−0.01 (−0.03, 0.01)	0.26	0.22	805	792	6.3	−0.01 (−0.04, 0.01)	0.24	0.17
Zimbabwe	SHINE (HIV+) (48)	244	231	8.2	0.02 (−0.04, 0.07)	0.03	0.07	73	78	14.1	−0.03 (−0.12, 0.06)	0.02	0.04
		**5963**	**7331**		**I² = 0.21, Tau² = 0.00**			**6345**	**7261**		**I² = 0.35, Tau² = 0.00**		
**Fixed**					**−0.01 (−0.02, 0.00)**						**−0.02 (−0.03, 0.00)**		
**Random**					**−0.01 (−0.03, 0.01)**						**−0.02 (−0.04, 0.00)**		

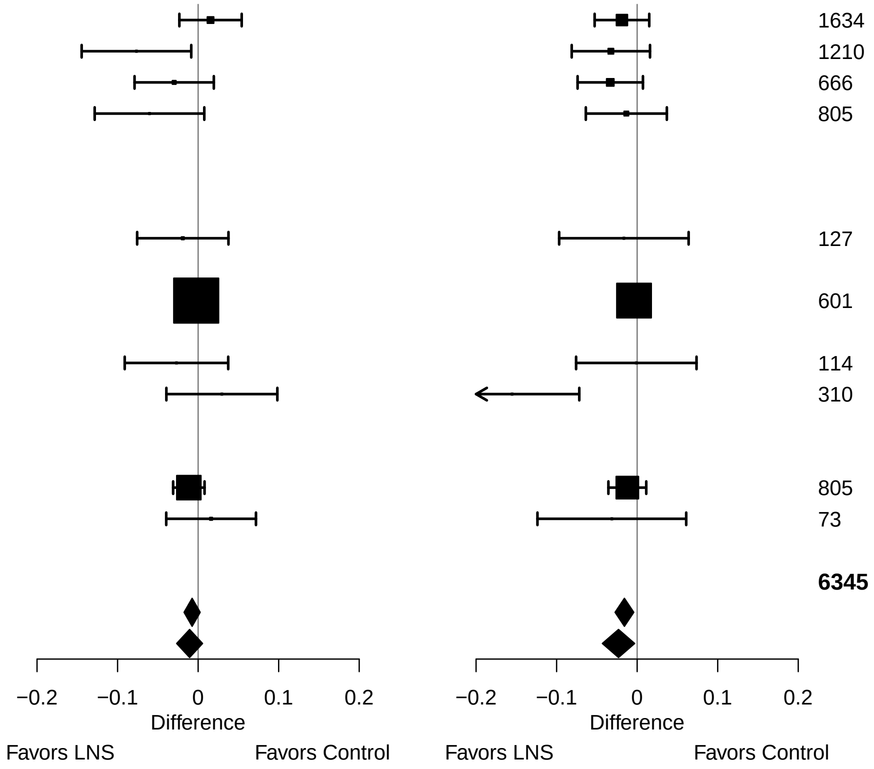

Supplemental figure 8Q: Small head size prevalence difference

8Q4: Stratified by Maternal education

P−for−interaction = 0.801
Difference in PDs = 0.00 (−0.02, 0.02)

		Primary or greater						Incomplete or no formal					
**Country**	**Trial**	**LNS N**	**Control N**	**Control Prevalence**	**PD (95% CI)**	**Fixed W**	**Random W**	**LNS N**	**Control N**	**Control Prevalence**	**PD (95% CI)**	**Fixed W**	**Random W**
Bangladesh	JiVitA−4 (34)	1855	775	23.3	−0.03 (−0.06, 0.00)	0.15	0.20	1028	489	25.5	0.04 (−0.01, 0.08)	0.08	0.17
Bangladesh	RDNS (35)	1245	589	41.6	−0.05 (−0.10, −0.01)	0.08	0.12	418	226	46.5	−0.02 (−0.10, 0.06)	0.03	0.07
Bangladesh	WASH−B (36)	817	2477	31.2	−0.05 (−0.08, −0.01)	0.13	0.17	346	986	36.7	0.00 (−0.06, 0.06)	0.04	0.10
Burkina Faso	iLiNS−Zinc (37)	82	16	31.2	0.07 (−0.11, 0.24)	0.01	0.01	1851	645	41.1	−0.05 (−0.10, 0.00)	0.07	0.15
Burkina Faso	PROMIS (38)												
Burkina Faso	PROMIS CS (38)												
Ghana	GHANA (39)												
Ghana	iLiNS−DYADG (40)	267	548	15.3	−0.02 (−0.07, 0.03)	0.06	0.10	80	144	18.8	−0.01 (−0.12, 0.09)	0.01	0.04
Haiti	HAITI (41)												
Kenya	WASH−B (42)	703	2456	2.8	0.00 (−0.01, 0.02)	0.56	0.36	756	2716	5.7	−0.01 (−0.03, 0.00)	0.73	0.37
Madagascar	MAHAY (43)												
Malawi	iLiNS−DYADM (44)												
Malawi	iLiNS−DOSE (45)	163	55	16.1	−0.02 (−0.12, 0.09)	0.01	0.03	517	176	23.9	−0.06 (−0.12, 0.00)	0.04	0.11
Mali	PROMIS (46)												
Mali	PROMIS CS (46)												
Zimbabwe	SHINE (HIV−) (47)												
Zimbabwe	SHINE (HIV+) (48)												
		**5132**	**6916**		**I² = 0.49, Tau² = 0.00**			**4996**	**5382**		**I² = 0.33, Tau² = 0.00**		
**Fixed**					**−0.01 (−0.03, 0.00)**						**−0.01 (−0.02, 0.00)**		
**Random**					**−0.02 (−0.04, 0.00)**						**−0.01 (−0.04, 0.01)**		

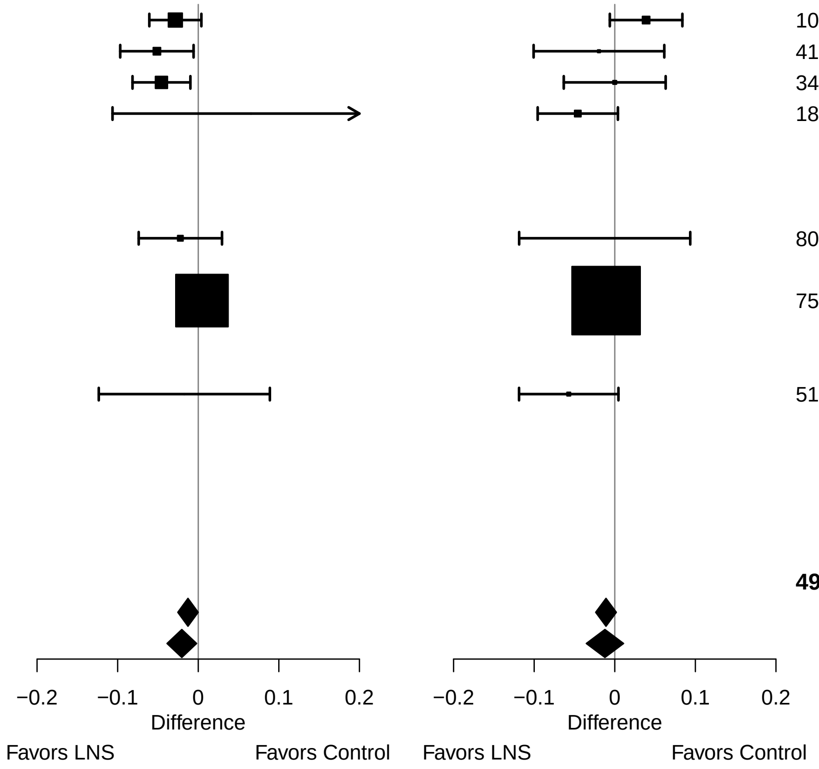

Supplemental figure 8Q: Small head size prevalence difference

8Q5: Stratified by Maternal depressive symptoms

P−for−interaction = 0.134
Difference in PDs = 0.01 (0.00, 0.03)

Country	Trial	LNS N	Control N	Control Prevalence	Less than 75th percentile PD (95% CI)	Fixed W	Random W	LNS N	Control N	Control Prevalence	At least 75th percentile PD (95% CI)	Fixed W	Random W
Bangladesh	JiVitA−4 (34)												
Bangladesh	RDNS (35)	1081	472	39.0	0.00 (−0.04, 0.04)	0.06	0.09	520	288	49.3	−0.13 (−0.20, −0.06)	0.07	0.13
Bangladesh	WASH−B (36)	901	2487	33.0	−0.05 (−0.08, −0.01)	0.08	0.12	238	885	31.9	0.02 (−0.05, 0.08)	0.08	0.14
Burkina Faso	iLiNS−Zinc (37)												
Burkina Faso	PROMIS (38)												
Burkina Faso	PROMIS CS (38)												
Ghana	GHANA (39)												
Ghana	iLiNS−DYADG (40)	252	459	15.0	0.00 (−0.05, 0.06)	0.03	0.05	83	209	17.7	−0.06 (−0.15, 0.04)	0.04	0.09
Haiti	HAITI (41)												
Kenya	WASH−B (42)	998	3517	4.0	0.00 (−0.02, 0.01)	0.51	0.37	341	1248	5.4	−0.02 (−0.05, 0.01)	0.36	0.23
Madagascar	MAHAY (43)												
Malawi	iLiNS−DYADM (44)	157	303	10.2	−0.02 (−0.08, 0.04)	0.03	0.05	47	113	11.5	0.03 (−0.08, 0.15)	0.03	0.07
Malawi	iLiNS−DOSE (45)												
Mali	PROMIS (46)												
Mali	PROMIS CS (46)												
Zimbabwe	SHINE (HIV−) (47)	1193	1135	5.0	−0.01 (−0.02, 0.01)	0.26	0.27	509	490	7.1	−0.02 (−0.05, 0.01)	0.40	0.23
Zimbabwe	SHINE (HIV+) (48)	234	215	8.8	0.01 (−0.04, 0.07)	0.03	0.06	89	100	12.0	−0.03 (−0.12, 0.06)	0.04	0.10
		**4816**	**8588**		**I² = 0.17, Tau² = 0.00**			**1827**	**3333**		**I² = 0.48, Tau² = 0.00**		
**Fixed**					**−0.01 (−0.02, 0.00)**						**−0.02 (−0.04, −0.01)**		
**Random**					**−0.01 (−0.02, 0.00)**						**−0.03 (−0.06, 0.01)**		

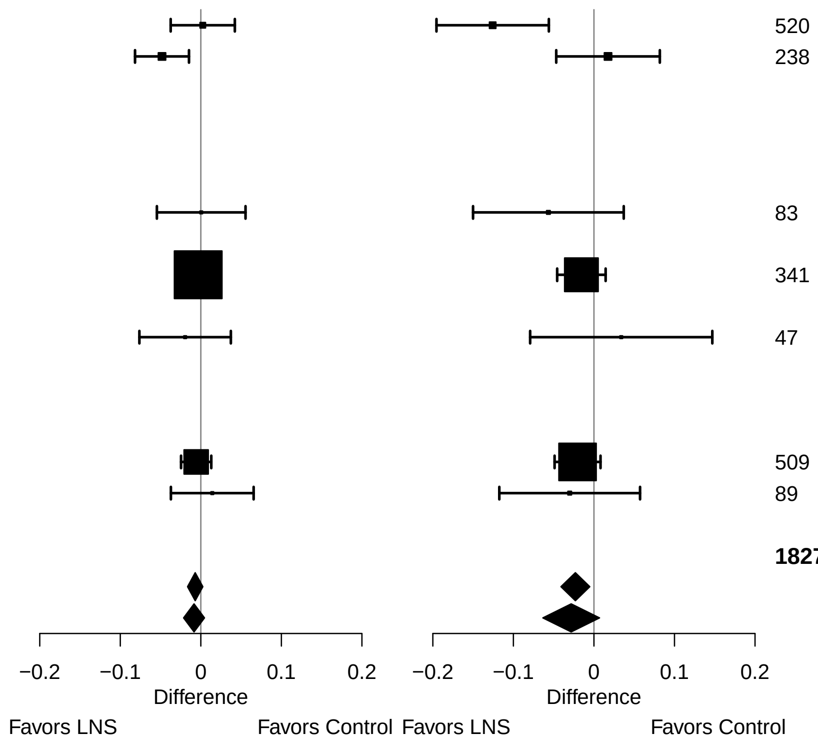

Supplemental figure 8Q: Small head size prevalence difference

8Q6: Stratified by Child sex

P−for−interaction = 0.624
Difference in PDs = 0.00 (−0.02, 0.01)

Country	Trial	Male LNS N	Male Control N	Male Control Prevalence	Male PD (95% CI)	Male Fixed W	Male Random W	Female LNS N	Female Control N	Female Control Prevalence	Female PD (95% CI)	Female Fixed W	Female Random W
Bangladesh	JiVitA−4 (34)	1475	612	25.8	−0.02 (−0.05, 0.02)	0.09	0.09	1410	655	22.4	0.01 (−0.03, 0.04)	0.08	0.12
Bangladesh	RDNS (35)	833	411	37.7	0.03 (−0.02, 0.07)	0.05	0.05	830	404	48.3	−0.12 (−0.18, −0.05)	0.02	0.08
Bangladesh	WASH−B (36)	566	1730	32.4	−0.02 (−0.07, 0.02)	0.06	0.06	597	1733	33.2	−0.04 (−0.08, 0.00)	0.05	0.11
Burkina Faso	iLiNS−Zinc (37)	991	335	42.3	−0.02 (−0.09, 0.05)	0.02	0.02	955	329	39.7	−0.07 (−0.12, −0.02)	0.04	0.10
Burkina Faso	PROMIS (38)												
Burkina Faso	PROMIS CS (38)												
Ghana	GHANA (39)												
Ghana	iLiNS−DYADG (40)	175	323	19.2	−0.03 (−0.10, 0.04)	0.02	0.02	172	369	13.3	−0.02 (−0.08, 0.04)	0.03	0.08
Haiti	HAITI (41)												
Kenya	WASH−B (42)	736	2466	4.9	−0.01 (−0.02, 0.01)	0.40	0.40	725	2710	3.8	0.00 (−0.02, 0.02)	0.37	0.14
Madagascar	MAHAY (43)												
Malawi	iLiNS−DYADM (44)	107	207	13.0	−0.02 (−0.10, 0.06)	0.02	0.02	113	237	8.9	−0.01 (−0.07, 0.05)	0.03	0.08
Malawi	iLiNS−DOSE (45)	349	123	23.6	−0.01 (−0.09, 0.07)	0.02	0.02	343	115	19.8	−0.08 (−0.15, −0.01)	0.02	0.07
Mali	PROMIS (46)												
Mali	PROMIS CS (46)												
Zimbabwe	SHINE (HIV−) (47)	927	903	6.4	−0.01 (−0.03, 0.01)	0.30	0.30	944	880	5.1	−0.01 (−0.03, 0.00)	0.34	0.14
Zimbabwe	SHINE (HIV+) (48)	162	168	14.3	−0.04 (−0.11, 0.03)	0.02	0.02	175	161	5.6	0.04 (−0.02, 0.09)	0.03	0.09
		**6321**	**7278**		**I² = 0.00, Tau² = 0.00**			**6264**	**7593**		**I² = 0.64, Tau² = 0.00**		
**Fixed**					**−0.01 (−0.02, 0.00)**						**−0.01 (−0.02, 0.00)**		
**Random**					**−0.01 (−0.02, 0.00)**						**−0.03 (−0.05, 0.00)**		

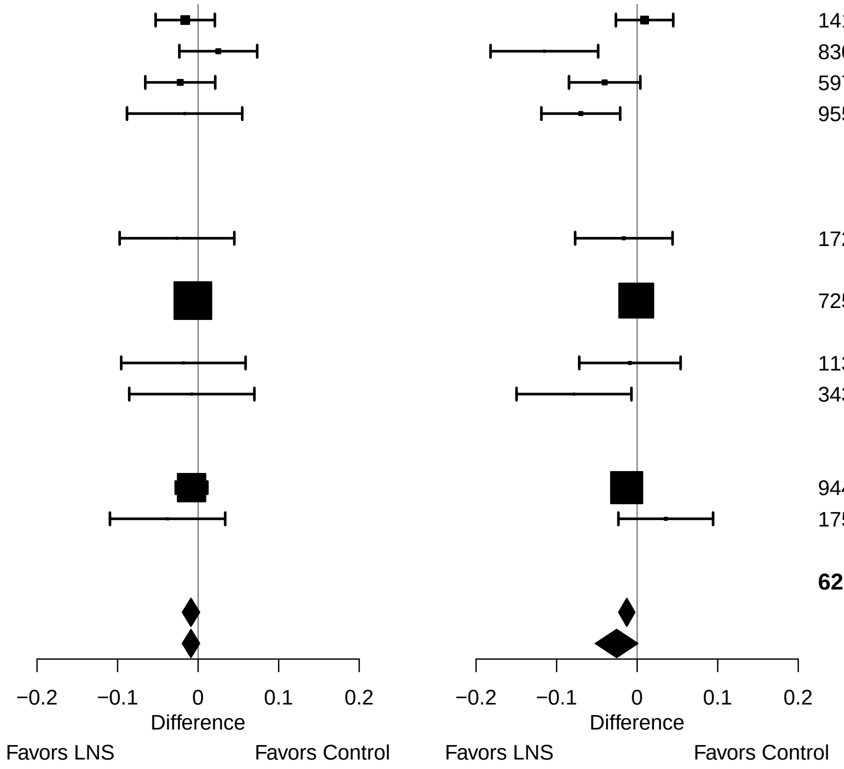

Supplemental figure 8Q: Small head size prevalence difference

8Q7: Stratified by Child birth order

P−for−interaction = 0.402
Difference in PDs = −0.01 (−0.02, 0.01)

Country	Trial	Later born LNS N	Later born Control N	Later born Control Prevalence	Later born PD (95% CI)	Later born Fixed W	Later born Random W	Firstborn LNS N	Firstborn Control N	Firstborn Control Prevalence	Firstborn PD (95% CI)	Firstborn Fixed W	Firstborn Random W
Bangladesh	JiVitA−4 (34)	616	260	25.0	0.00 (−0.05, 0.05)	0.03	0.05	2265	1000	23.9	0.00 (−0.04, 0.03)	0.18	0.18
Bangladesh	RDNS (35)	974	508	43.1	−0.06 (−0.10, −0.01)	0.04	0.06	688	307	42.7	−0.03 (−0.09, 0.04)	0.04	0.04
Bangladesh	WASH−B (36)	736	2313	31.9	−0.03 (−0.07, 0.00)	0.06	0.09	427	1150	34.6	−0.03 (−0.09, 0.02)	0.06	0.06
Burkina Faso	iLiNS−Zinc (37)	1515	519	40.3	−0.05 (−0.10, 0.01)	0.02	0.04	431	145	43.4	−0.04 (−0.11, 0.04)	0.03	0.03
Burkina Faso	PROMIS (38)												
Burkina Faso	PROMIS CS (38)												
Ghana	GHANA (39)												
Ghana	iLiNS−DYADG (40)	237	458	14.4	0.00 (−0.06, 0.05)	0.03	0.04	110	234	19.2	−0.05 (−0.13, 0.04)	0.02	0.02
Haiti	HAITI (41)												
Kenya	WASH−B (42)	1137	4074	4.3	0.00 (−0.02, 0.01)	0.41	0.33	322	1097	4.3	−0.01 (−0.03, 0.01)	0.48	0.48
Madagascar	MAHAY (43)												
Malawi	iLiNS−DYADM (44)	173	353	10.8	−0.03 (−0.08, 0.03)	0.03	0.04	47	89	10.1	0.05 (−0.07, 0.16)	0.01	0.01
Malawi	iLiNS−DOSE (45)	458	162	20.2	−0.01 (−0.08, 0.05)	0.02	0.03	146	45	26.7	−0.13 (−0.25, −0.01)	0.01	0.01
Mali	PROMIS (46)												
Mali	PROMIS CS (46)												
Zimbabwe	SHINE (HIV−) (47)	1295	1214	5.0	−0.01 (−0.02, 0.00)	0.37	0.31	489	486	7.8	−0.02 (−0.05, 0.02)	0.15	0.15
Zimbabwe	SHINE (HIV+) (48)												
		**7141**	**9861**		**I^2 = 0.14, Tau^2 = 0.00**			**4925**	**4553**		**I^2 = 0.00, Tau^2 = 0.00**		
**Fixed**					**−0.01 (−0.02, 0.00)**						**−0.02 (−0.03, 0.00)**		
**Random**					**−0.01 (−0.03, 0.00)**						**−0.02 (−0.03, 0.00)**		

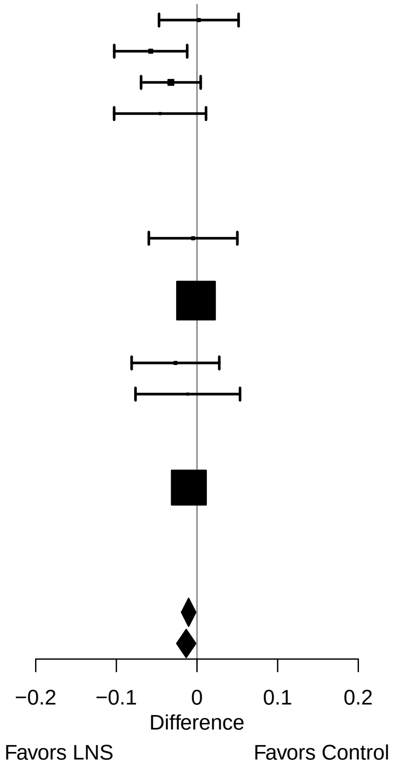

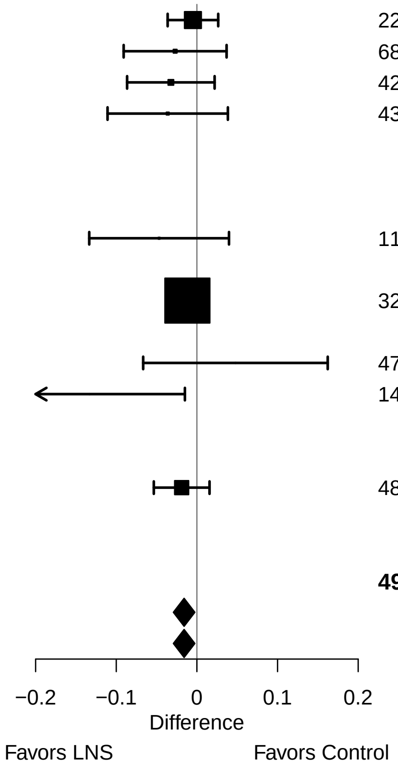

Supplemental figure 8Q: Small head size prevalence difference

8Q8: Stratified by Child baseline anthropometric status

P−for−interaction = 0.069

Difference in PDs = −0.02 (−0.05, 0.00)

		HCZ at least −1 Z						HCZ less than −1 Z					
**Country**	**Trial**	**LNS N**	**Control N**	**Control Prevalence**	**PD (95% CI)**	**Fixed W**	**Random W**	**LNS N**	**Control N**	**Control Prevalence**	**PD (95% CI)**	**Fixed W**	**Random W**
Bangladesh	JiVitA−4 (34)	1411	630	2.7	0.00 (−0.02, 0.02)	0.21	0.21	1474	637	45.4	−0.02 (−0.07, 0.03)	0.27	0.27
Bangladesh	RDNS (35)	521	239	5.0	−0.03 (−0.05, 0.00)	0.10	0.14	1080	521	60.3	−0.05 (−0.09, 0.00)	0.29	0.29
Bangladesh	WASH−B (36)												
Burkina Faso	iLiNS−Zinc (37)	550	176	5.1	−0.04 (−0.07, −0.01)	0.08	0.12	1396	488	54.1	−0.06 (−0.13, 0.02)	0.12	0.12
Burkina Faso	PROMIS (38)												
Burkina Faso	PROMIS CS (38)												
Ghana	GHANA (39)												
Ghana	iLiNS−DYADG (40)	189	384	2.3	0.01 (−0.02, 0.04)	0.09	0.13	134	257	35.4	−0.04 (−0.14, 0.06)	0.07	0.07
Haiti	HAITI (41)												
Kenya	WASH−B (42)												
Madagascar	MAHAY (43)												
Malawi	iLiNS−DYADM (44)												
Malawi	iLiNS−DOSE (45)	381	132	7.6	−0.01 (−0.06, 0.04)	0.03	0.05	311	106	39.6	−0.09 (−0.20, 0.01)	0.06	0.06
Mali	PROMIS (46)												
Mali	PROMIS CS (46)												
Zimbabwe	SHINE (HIV−) (47)	1072	967	2.8	−0.01 (−0.02, 0.01)	0.43	0.26	254	239	15.5	−0.01 (−0.07, 0.05)	0.17	0.17
Zimbabwe	SHINE (HIV+) (48)	200	168	4.2	−0.01 (−0.04, 0.03)	0.05	0.09	71	67	28.4	−0.07 (−0.24, 0.09)	0.02	0.02
		**4324**	**2696**		**I² = 0.31, Tau² = 0.00**			**4720**	**2315**		**I² = 0.00, Tau² = 0.00**		
**Fixed**					**−0.01 (−0.02, 0.00)**						**−0.04 (−0.06, −0.01)**		
**Random**					**−0.01 (−0.02, 0.00)**						**−0.04 (−0.06, −0.01)**		

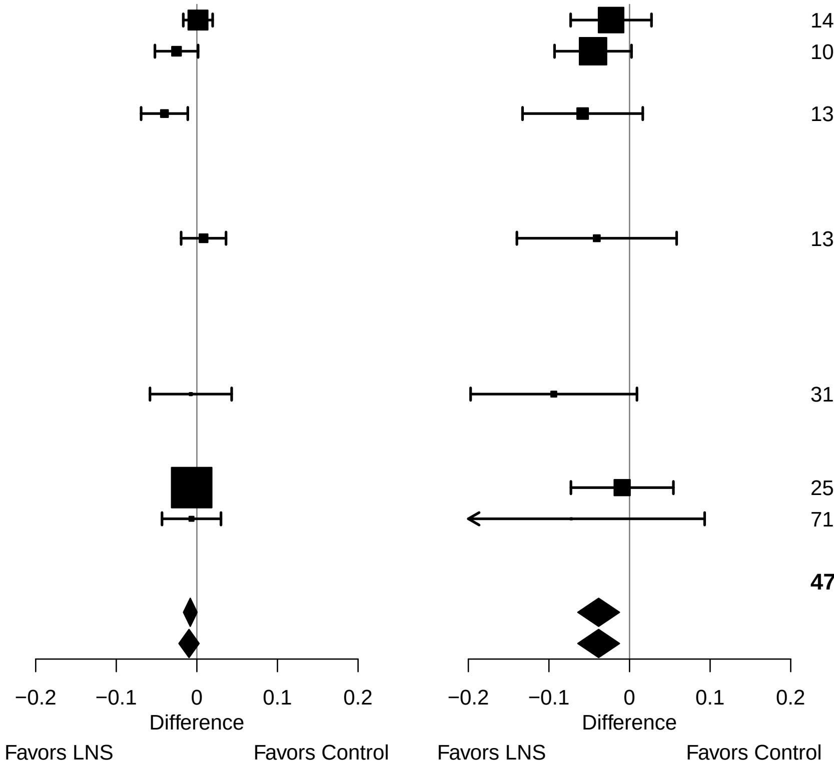

Supplement: nqab278_Supplemental_Files [file nqab278_supplemental_files.zip › 12_SQ-LNS_IPD_growth_Supplemental_Figure_8.pdf]
